# A simple feature construction method for predicting upstream/downstream signal flow in human protein-protein interaction networks

**DOI:** 10.1038/srep17983

**Published:** 2015-12-09

**Authors:** Suyu Mei, Hao Zhu

**Affiliations:** 1 Software College, Shenyang Normal University, Shenyang, China; 2 Bioinformatics Section, School of Basic Medical Sciences, Southern Medical University, Guangzhou, China

## Abstract

Signaling pathways play important roles in understanding the underlying mechanism of cell growth, cell apoptosis, organismal development and pathways-aberrant diseases. Protein-protein interaction (PPI) networks are commonly-used infrastructure to infer signaling pathways. However, PPI networks generally carry no information of upstream/downstream relationship between interacting proteins, which retards our inferring the signal flow of signaling pathways. In this work, we propose a simple feature construction method to train a SVM (support vector machine) classifier to predict PPI upstream/downstream relations. The domain based asymmetric feature representation naturally embodies domain-domain upstream/downstream relations, providing an unconventional avenue to predict the directionality between two objects. Moreover, we propose a semantically interpretable decision function and a macro bag-level performance metric to satisfy the need of two-instance depiction of an interacting protein pair. Experimental results show that the proposed method achieves satisfactory cross validation performance and independent test performance. Lastly, we use the trained model to predict the PPIs in HPRD, Reactome and IntAct. Some predictions have been validated against recent literature.

Signal transduction plays important roles in the life processes of cell, e.g., growth, differentiation, metabolism and apoptosis. Malfunction of signaling pathways would lead to a variety of pathologies^1^. Along signaling pathways signals are sensed, amplified and transducted from extracellular microenvironment, cellular matrix or cell membrane to the cell nucleus to yield various complex biological responses, e.g. enzyme activity, transcription factors activation/deactivation, gene expression, ion-channel activity, etc.^2^. Since signals are transmitted via a series of molecular interactions especially protein-protein interactions (PPI), reconstruction of PPI networks has gained much attention from experimental biologists^2^,^3^,^4^ and computational biologists^5^,^6^,^7^,^8^,^9^,^10^,^11^ in recent years. However, PPI networks generally do not carry upstream/downstream relationship between two interacting proteins, which retards our understanding of the stimuli-response pathways that signals traverse. Thus we need to further annotate the current PPI networks with the directions of signal flow.

At present the computational methods that infer signaling pathways focus on predicting novel signaling components or pathway targeted proteins^12^, expanding the current signaling pathways with the knowledge of orthologs^13^,^14^ or modeling the cross-talks between signaling pathways^15^,^16^. A few methods attempt to infer the directions of signal flow directions from PPI networks^17^,^18^,^19^,^20^. Vinayagam *et al.*^17^ developed a computational method to predict the activation/inhibition relationship within protein complex. The method does not predict the upstream/downstream relationship in a strict sense, but predicts two major PPI types (activation/inhibition) within protein complex. Gitter *et al.*^18^ proposed an optimization method to solve the NP-hard problem of maximum edge orientation. The method needs no other information but the information of PPI network topology alone with the demerit of computational intensity. Tuncbag *et al.*^19^ used message-passing algorithm (PCST, Prize-collecting Steiner Tree) to infer directed forest from PPI networks. The method has the merit of simultaneously deriving multiple signaling pathways and modeling their cross-talks. Vinayagam *et al.*^20^ assumed that signaling pathway starts from plasma membrane-associated receptors to transcription factors and then derived SPC (shortest path connection) features from PPI networks to train a naïve Bayesian classifier for PPI directionality prediction. These PPI network topologies based methods, simple and intuitive as they are, generally do not exploit the experimentally verified upstream/downstream information that is provided in KEGG^21^ and NetPath^22^, thus are prone to yield false signaling proteins, false pathways and incorrect directions of signal flow. Moreover, the current PPI networks are incomplete and noisy, thus the topologies based methods are likely to yield false results.

Liu *et al.*^23^ first derived from the directional information between two interacting proteins the probability of the direction between two domains of the interacting proteins. Based on the probabilistic statistics, the authors proposed a function *F* to predict the direction of any domain pair, and the authors further proposed a parameter *PIDS* to predict the direction of any protein pair. As compared to those PPI network topologies based methods, this method effectively exploit the experimentally verified upstream/downstream information between interacting proteins. But the upstream/downstream information of two domains may not be sufficient to determine the upstream/downstream relationship between two proteins. The rules may not be so simple. The directional determinant may be highly non-linear domain combinations. For instance, upstream/downstream relationship between two proteins is potentially determined by two up-regulating domains from one protein and one down-regulated domain from the other protein. Therefore, nonlinear combination of up-regulating domains and down-regulated domains should be taken into account for prediction of upstream/downstream relations between interacting proteins.

In this work we propose an asymmetric domain feature representation method to train a nonlinear SVM (support vector machine) to annotate the upstream/downstream relations of signal flow in human protein-protein interaction networks. Using this feature representation, the relations between the up-regulating domains and the down-regulated domains can be easily incorporated into the feature vectors, and the trained SVM easily maps the nonlinear domain regulatory combinations to regulatory direction between interacting proteins. However, the intuitive feature representation makes the final decision complicated, i.e. each protein pair (*A*, *B*) is represented with two instances 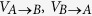, thus we have to make a rational decision between the outputs 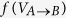 and 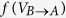. For this reason, we propose a macro performance metric to combine 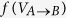 and 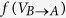 for final decision. To verify the effectiveness of the proposed method, we conduct 20-fold cross validation experiment on NetPath training data and further conduct independent test on KEGG data. Lastly, we predict the upstream/downstream relations for all the PPIs in human PPI networks and validate the predictions against experimental data and recent literature.

## Data and Methods

### Data and materials

We have collected 1,148 annotated PPIs from 22 human signaling pathways in KEGG^21^. The PPIs are classified into 13 types, e.g., activation, inhibition, phosphorylation, ubiquitination, methylation, dephosphorylation, indirect, binding, compound, etc., most of which are directed. Since we focus on the direction between two physically interacting proteins, the PPIs of *indirect* type are removed. Some PPI types like binding/association, compound, interaction, complex, etc. have no clear implication of directions. To avoid ambiguity, these PPI types are also excluded out of training data, thus we obtain 893 PPIs that are unambiguously directed (called KEGG).

NetPath^22^ collects 36 human cancer signaling pathways. As compared with KEGG, NetPath provides more abundant knowledge about signaling components, signaling pathways, enzyme catalysis and pathway targeted genes. Unfortunately, the PPIs in NetPath are generally not annotated and only *enzyme catalysis* explicitly provides the information of PPI upstream/downstream relations. The PPIs of enzyme catalysis are directional and can be used as independent test set. We collect 730 PPIs of enzyme catalysis (called NetPath-EC) and 3,216 physical PPIs (called NetPath-PI) with no directional information from 18 human cancer signaling pathways (TGFBeta, TNF, TCR, Notch, Leptin, Kit, RANKL, Prolactin, Wnt, ID, Gastrin, Ghrelin, Hedgehog, RAGE, AR, BCR, EGFR, IL). NetPath-PI will be annotated using the proposed predictive model.

HPRD^4^ is a well-established repository of physical protein-protein interactions. Since signals are mostly transmitted via neighbour proteins that physically interact, HPRD is fairly suited to be used as PPI infrastructure for reconstruction of signal flows. By removing those obsolete proteins, uncurated proteins and those proteins that have been included in KEGG + NetPath (the union of KEGG and NetPath-EC, we obtain 36,416 PPIs to have their directions predicted (called HPRD prediction set). Actually HPRD database has also curated some directional information of physically interacting proteins that fulfil the function of protein post-translational modifications (PTM) such as phosphorylation, proteolytic cleavage, acetylation, methylation, etc. Totally we obtain 2547 PTM PPIs (called HPRD-PTM) that are disjoint with KEGG + NetPath. HPRD-PTM can be used as validation set.

HPRD is specialized to collect physical protein-protein interactions of Homo sapiens. Comparatively, Reactome^23^ and IntAct^24^ are recently updated comprehensive repositories of protein-protein interactions. In this work, we also use the trained SVM classifier to annotate the PPIs in Reactome and IntAct. Especially, Wu *et al.*^25^ has collected 70,557 PPIs that are annotated with upstream/downstream relations, which has been submitted to Reactome website (http://reactomews.oicr.on.ca:8080/caBigR3WebApp2014/FIsInGene_121514_with_annotations.txt.zip) and is suited to be used to validate our proposed model (called Reactome). Nevertheless, the entries in this dataset are provided without reliable sources, so we do not incorporate this dataset into the training data. Actually, Wu *et al.*^25^ borrowed the information of signaling pathways and PPI directions to predict novel functional interactions, similar to^26^. His work does not aim to predict novel PPI upstream/downstream relations, which is quite different to our proposed method. The details of above-mentioned datasets are described in Table 1. It is noted that those proteins from Reactome and IntAct have no corresponding entries in Uniprot (http://www.uniprot.org/) are removed.

**Figure 1:**
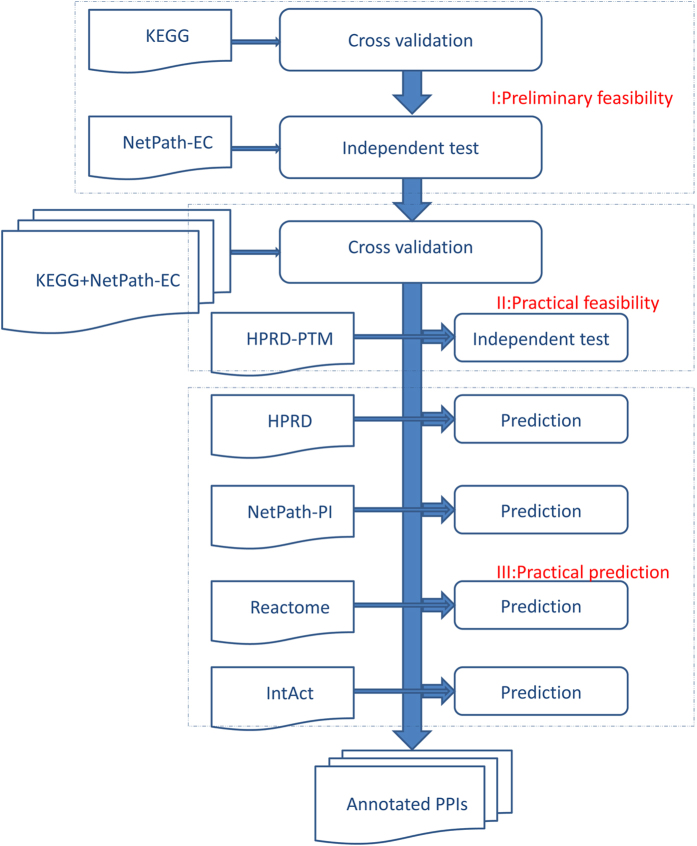
Data flow chart for model training, estimation and prediction. The experimental setting includes three major phases that are organized in three dotted boxes. Phase I uses KEGG as training data and uses NetPath-EC as validation set. Phase II uses the augmented training data KEGG+ NetPath-EC and uses HPRD-PTM as validation set. Phase III conducts practical predictions.

For clarity, the data flow chart for model training, evaluation and prediction is illustrated in Fig. 1. As shown in Fig. 1, the experimental setting includes three major phases organized in three dotted boxes (I) preliminary feasibility study (II) practical feasibility study and (III) practical prediction. KEGG is used to train and evaluate a SVM classifier and NetPath-EC is used to validate the trained classifier. If the results are acceptable, we further conduct the experiments of phase II. In phase II, the training data is augmented using NetPath-EC and the model is validated using HPRD-PTM. If the results are also acceptable, we then enter into the final phase to conduct practical predictions on HPRD, Reactome and IntAct. It is noted that all the independent test set and prediction set have no overlap with the training data.

### Feature construction

Protein domain or domain-domain interaction has been used to predict the directionality between two interacting proteins^27^, where the probabilities of all upstream/downstream domain pairs are derived from the known directional PPIs and then a predictive function is accordingly defined. In the method, no feature construction is needed for model training in that the final decision function is simply based on the pair-wise probabilities of upstream/downstream domain pairs. PPI directionality is predicted by domain-domain directionality. Vinayagam *et al.*^20^ derived eight SPC (shortest path connections) topologies based probabilistic features to train a naïve Bayesian classifier. In the method, the directionality between two interacting proteins is incorporated into the feature vector by the directionality along SPC pathways.

Here we also use protein domains to predict the directionality between two interacting proteins since domain-domain interaction can be inferred from protein-protein interaction^28^ and protein-protein interaction can also be recovered from domain-domain interaction^29^. But different to^27^, we construct a feature vector for each protein pair to incorporate the upstream/downstream relations of domains, based on which to train a SVM classifier for PPI directionality prediction. Pfam domains or profile HMMs (hidden Markov models) are probabilistic models used for the statistical inference of homology built from an aligned set of curator-defined family representative sequences^30^. Assume that the pfam domains of all the proteins from KEGG and Netpath are collected into the domain set 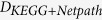, and all the domains are orderly arranged in one dimension. Each domain *g* (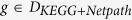) is assigned a unique integer index *g* (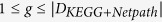), where the domain name and its index are both denoted as *g* for convenient reference. It is noted that the domain set is denoted as 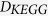 if the training data is from KEGG only. The one-dimensional domain vector has actually reflected the order or upstream/downstream relations of domains. For an interacting protein pair (A, B), the interacting direction could be 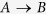 or 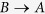. Assume 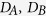 as the pfam domain set of protein A and protein B, respectively, and use 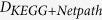 to denote the pfam domain set of the whole training data. Then the component of feature vector of 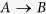 is defined as follows:


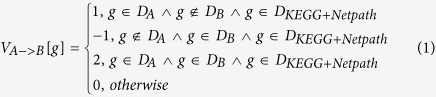


Accordingly, the component of feature vector of 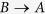 is defined as follows:


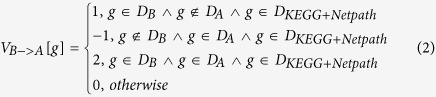



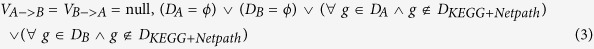


Here the domain name g also denotes its index for the convenience of description. Actually some modern programming language also supports retrieving array element by its attribute name. 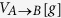 denotes the value of the component g of feature vector 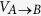. Formula (1) means that if the upstream protein A possesses the pfam domain g but the downstream protein B does not, then the corresponding component g in the feature vector 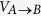 is set 1; if the upstream protein A does not possess the pfam domain g but the downstream protein B does, then the corresponding component g in the feature vector 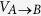 is set −1; if both the upstream protein A and the downstream protein B possess the pfam domain g, then then the corresponding component g in the feature vector 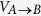 is set 2; otherwise, the component g is set 0. It is noted that formula (1) and formula (2) define the numeric values that the components of feature vector assume. As for the class label of protein pair (A, B), the feature vector is labelled + 1 if A- > B is experimentally confirmed, otherwise it is labelled −1.We can see that the definitions formulated as formula (1) and formula (2) are asymmetrical. Such a representation method is convenient to reflect the order of domains and the directionality of protein pairs. Formula (3) means that the feature vector would be treated as null vector and are removed if either protein of protein pair (A, B) contains zero pfam domain or does not contain any domain of the training data.

Similar to^20^, each protein pair is represented with two instances 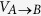 (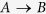) and 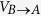 (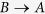), which poses a new challenge to model training, model evaluation and network-wide prediction (see next section for solutions). The proposed feature representation as formula (1) and formula (2) differs from^20^ from the aspects^1^ the proposed method incorporates the directionality of protein pair into the component value of one-dimensional domain feature vector, while^20^ used the topological SPC to derive 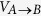 and 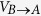
2 the proposed method uses the experimentally verified upstream/downstream relations in KEGG^21^ and Netpath^22^ to derive feature vectors, while^20^ derived feature vectors from the shortest path from plasma membrane receptors to transcription factors in PPI networks. The shortest paths are not necessarily verified signaling pathways.

### Model training and evaluation metrics

After constructing the feature vectors from the experimentally verified upstream/downstream relations in KEGG and Netpath, we further construct vector-represented two-class training data. Since we have the experimentally verified PPIs at hand, for instance (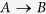), how to derive the negative data is a critical issue to two-class classification. But here negative does not mean the set of interactions without any directions. For those seemingly unidirectional protein complexes, directions still exist among the complex components. For instance, Vinayagam *et al.*^17^ developed a computational method to explicitly predict the activation/inhibition relations between complex components. Nevertheless, Liu *et al.*^23^ chose protein complex as negative data, seemingly in that they semantically interpret negative in their method as those protein pairs that have not been experimentally annotated with directionalities. Actually, predicting the directionality between two objects A and B is an unconventional classification problem. Here 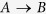 and 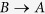 are two basic problems. Now that we have only 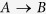 available, which are experimentally verified, so we naturally treat 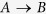 as positive (experimentally verified) and treat its corresponding 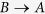 as negative (NOT experimentally verified). Negative does not traditionally mean experimentally not to happen or no directional information. Negative 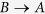 should be semantically interpreted as opposite to the positive 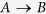 or semantically “NOT experimentally verified”. Negative 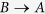 may not exist with high probability, but it does not mean impossible, it could happen with relatively low probability. Actually, Vinayagam *et al.*^20^ also used the reversed directions as negative data. Using the two-class data to train a decision function 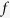, we can judge the directionality between A and B by combining 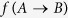 and 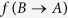.

For the aforesaid reasons, we, similar to^20^, treat the feature vectors 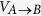 (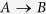) of the known PPI directions (denoted as T) as positive with class label + 1, and conversely treat the corresponding feature vectors 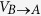 (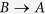) as negative with class label −1. The reason why we construct two-class training data this way is that we need the predictive model to yield the decision scores for both 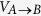 and 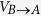. The simplest way is treat 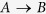 as one class and treat 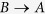 as the other class. Here *positive* actually means experimentally verified relations, while *negative* means NOT experimentally verified relations. *Negative* does not mean that the relations are experimentally verified not to exist. Moreover, neither does *negative* mean bi-direction or no directional information (undirected). Here we only focus on 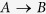, 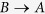 and 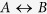 for a protein pair 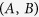. For the reason, we formulate the problem of PPI directionality prediction as two-class classification, and the data of *undirected* is not needed. 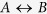 is unnecessarily treated as a third class, because it can be inferred from two-class classifier (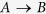, 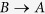).

Now we have constructed the positive data (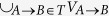) and the negative data (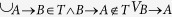) to train a SVM (support vector machine) classifier. Because each protein pair (A, B) is represented with two instances (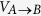, 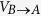), we need to consider two-level model estimation, i.e. micro instance-level performance and macro bag-level performance. The micro instance-level performance is used to estimate how well the classifier discriminates 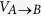 and 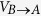. We can use the traditional performance metrics such as ROC-AUC (Area under curve of Receiver Operating Characteristic), SE (Sensitivity), SP (Specificity), MCC (Matthews correlation coefficient) and Accuracy to measure the instance-level performance. We first derive a confusion matrix *M* by 20-fold cross validation on the training data, based on which to further calculate several intermediate variables as formula (4), SP_l_, SE_l_ and MCC_l_ for each label as formula (5), and overall Accuracy & MCC as formula (6).


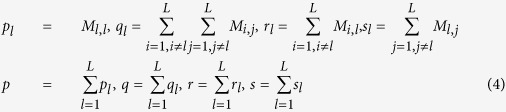



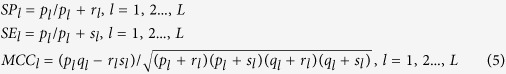



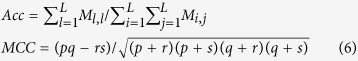


The confusion matrix 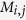 records the counts that class 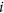 are classified to class 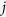, and *L* denotes the number of labels. AUC is calculated based on the decision values of two-class SVM. F1 score can be derived from formula (7)


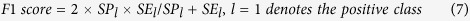


The micro instance-level performance metrics are used to estimate the basic performance that discriminates the positive instances 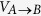 and the negative instances 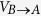, but the metrics cannot interpret how well the upstream/downstream relations of protein pairs are correctly predicted. For the reason, we need to combine the outputs 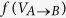 and 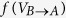 to define a more explicable performance metric, i.e. macro bag-level performance metric. For an interacting protein pair (A, B), the combined output is defined as follows:


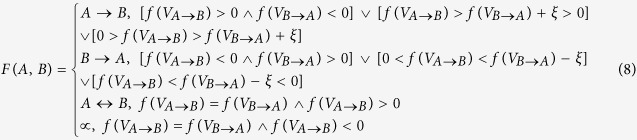


Where 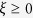 denotes threshold of the difference between 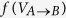 and 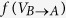. The function 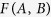 determines the directionality of the protein pair (A, B) according to the decision scores (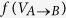,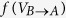) of the two instances (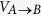,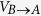). If 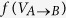 and 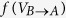 are oppositely signed, the instance that is predicted as positive determines the directionality. If 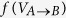 and 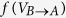 are identically signed, the final decision would be a little more complicated. If 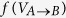 and 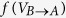 are both positive and unequal, the instance that achieves greater function score or function score greater than a threshold 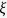 determines the directionality. If 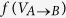 and 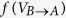 are equal or 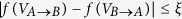 is satisfied, the directionality can be deemed as bi-directional. The case that 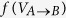 and 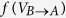 are both negative is hard to interpret because the *negative* class is semantically defined as NOT experimentally verified or unlikely to exist. Nevertheless, we can still semantically interpret the case 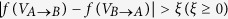 for 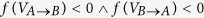. The instance that achieves a lower amplitude of decision score would be deemed to be probabilistically less incredible, for instance, 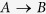 would be preferred over 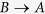 to be assigned to protein pair (A, B) if 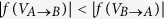 or 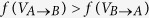 is satisfied. If 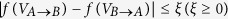 especially 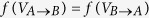 is satisfied, there exists semantic ambiguity, indicating that the trained predictive model cannot yield a rational decision. Here we use 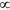 to denote that the directionality of protein pair (A, B) is undetermined.

Based on the combined output 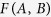 as defined in formula (7–8), we further define an indicator function as follows:


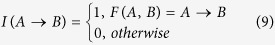


Based on formula (9), the macro bag-level performance metric is defined as follows:


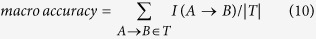


where T denotes the set of experimentally verified PPI upstream/downstream relations. *Macro_accuracy* denotes the coincident rate between the predicted directions and the true directions.

Comparatively, Liu *et al.*^27^ did not discuss the potentially predicted bi-directions, i.e. 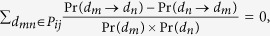 where 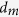 denotes the domain of protein 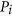 and 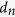 denotes the domain of protein 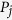. Vinayagam *et al.*^20^ also represent protein pair (A, B) with two instances (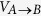, 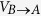), but how to combine the final decision is not clarified.

Here the classifier SVM assumes RBF (Radial Basis Function) kernel as defined below:


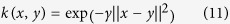


where 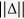 denotes 2-norm of vector 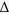 and the hyperparameter 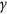 controls the flexibility of kernel. LIBSVM (https://www.csie.ntu.edu.tw/~cjlin/libsvmtools/) is adopted and the hyperparameter pair 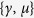 is tuned within 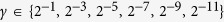 and 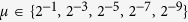 where 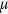 denotes SVM regularizer. Here we choose large intervals to reduce the complexity of model selection.

## Results

### Model performance estimation

#### Cross validation

First, we conduct 20-fold cross validation on KEGG that contains 893 positive instances and 893 negative instances (see the previous section feature construction), i.e. Phase I as illustrated in Fig. 1. The micro instance-level performance and the macro bag-level performance are provided in Table 2. As shown in Table 2, the proposed method achieves 0.8399 ROC-AUC score,, 0.5920 MCC, 0.7560 F1 score and 75.60% accuracy. From the micro instance-level performance on the positive class (SP = 0.7560; SE = 0.7560; MCC = 0.5920) and the negative class (SP = 0.7560; SE = 0.7560; MCC = 0.5920), we can see that the proposed method shows little tendency of predictive bias. From the micro instance-level performance, we can see that the proposed method satisfactorily discriminates the positive instances from the negative instances. Besides, the macro bag-level performance metric *macro_accuracy* is also promising, indicating that the proposed method correctly recognizes 74.12% PPI upstream/downstream relations.

**Figure 2:**
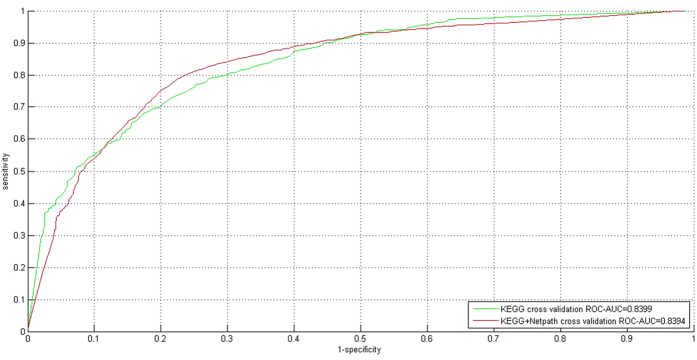
ROC curves for 20-fold cross validation on KEGG and KEGG+NetPath. The ROC-AUC scores are used to estimate the micro instance-level performance.

To take full advantage of the currently available data, we further merge KEGG with NetPath-EC into a larger training data KEGG + NetPath-EC to train the final SVM for network-wide prediction, i.e. Phase II as illustrated in Fig. 1. Before prediction we also need to conduct cross validation on the data to estimate the model performance. The results are provided in Table 2. As compared with KEGG, the performance on KEGG + NetPath-EC shows a little increase (Accuracy = 78.01%; MCC = 0.7162; F1 score = 0.7808) versus (Accuracy = 75.60%; MCC = 0.5920; F1 score = 0.7560). Comparing the ROC-AUC score (see Fig. 2), the ROC-AUC score shows a slight decrease (0.8394 versus 0.8399). The micro instance level performance on the positive class (SP = 0.7782; SE = 0.7835; MCC = 0.6277) and the negative class (SP = 0.7820; SE = 0.7767; MCC = 0.6270) also shows no tendency of predictive bias on KEGG+NetPath-EC. The macro bag-level performance *macro_accuracy* indicates that 75.87% PPI upstream/downstream relations are correctly recognized. Liu *et al.*^23^ adopts 2,803 PPIs with known domain-domain directions that are quite different to the 1,623 experimentally verified PPI upstream/downstream relations in KEGG and NetPath, thus it makes little sense to compare the two methods in terms of cross validation performance. The optimal hyperparameter pair is 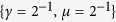 on KEGG and 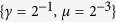 on KEGG+NetPath-EC.

#### Independent test & validation

**Figure 3:**
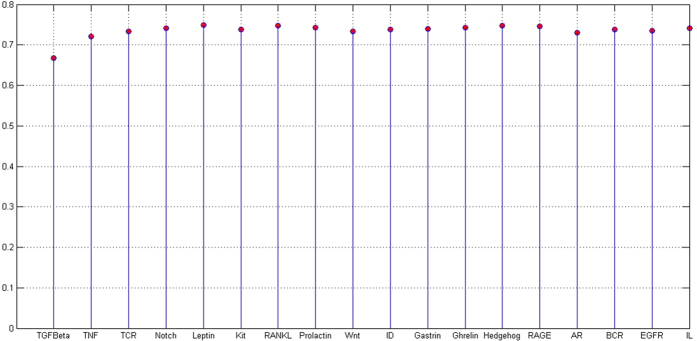
*Macro_accuracy* scores on the enzyme catalysis data of human signaling pathways in NetPath database. The scores are predicted by the SVM model that is trained on KEGG training data. The scores suggest that the proposed method generalizes well to the unseen data.

Only cross validation on the training data is not sufficient to demonstrate the generalization ability of the trained model. We further use NetPath (enzyme catalysis) to evaluate the model that is trained on KEGG (Phase I as illustrated in Fig. 1), and use HPRD-PTM to evaluate the model that is trained on KEGG+NetPath-EC (Phase II as illustrated in Fig. 1). Remarkably, many proteins in the independent test and the prediction set are likely not to contain any signature domains of the proteins in the training data, thus the PPIs to be predicted are degenerated to null vectors as depicted in formula (3). Null vectors should be removed for credible predictions. For the reason, NetPath-EC is reduced to 648 PPIs and HPRD-PTM is reduced to 1,853 PPIs. The macro bag-level performance *macro_accuracy* shows that 74.54% NetPath-EC PPI upstream/downstream relations (independent test in Phase I as illustrated in Fig. 1) and 81.92% HPRD-PTM upstream/downstream modifications are correctly recognized (independent test in Phase I as illustrated in Fig. 1). The predictions of NetPath-EC enzyme catalysis are provided in Section 1 of the supplementary file and the predictions of HPRD-PTM are provided in Section 2 of the supplementary file. In the supplementary file, each protein pair (A, B) is followed by the predicted directionality (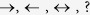), the function score 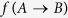, and the function score 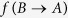. Question mark ? means the directionality is undetermined. The *macro_accuracy* scores of the 18 human signaling pathways in NetPath-EC are shown in Fig. 3. From Fig. 3, we can see that most of the *macro_accuracy* scores are over 70%.

Liu *et al.*^23^ also predicted the PPIs in HPRD. We extract their experimental results and found 502 PPIs that overlap with HPRD-PTM. Among the 502 post-translational modifications, Liu *et al.*^23^ correctly recognized 370 PPIs, accounting for 73.71%. Among the 502 PPIs, our proposed method correctly recognizes 428 PPIs, accounting for 85.26%. The performance increase is partly attributed to the merit that our proposed machine learning method can easily incorporate into the feature vectors the information of up-regulating domains and the down-regulated domains, and adopts SVM to exploit the nonlinearity of domain combination.

### Prediction of upstream/downstream signal flow in human PPI networks and literature validation

#### Network-wide prediction

**Figure 4:**
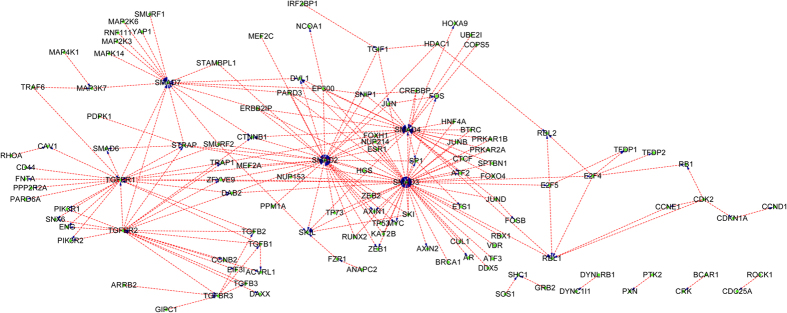
The predicted PPI upstream/downstream relations in TGF-β signaling pathway taken from NetPath database. The protein at the arrow start denotes the upstream protein and the protein at the arrow end denotes the downstream protein. For clarity, the predicted bi-directions and the undetermined directions are not drawn.

**Figure 5:**
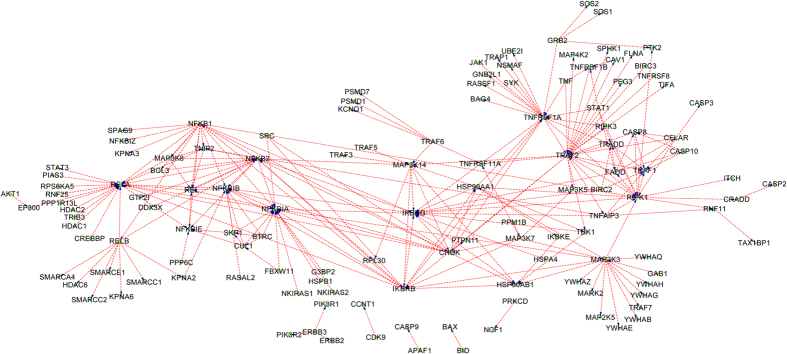
The predicted PPI upstream/downstream relations in TNF-αsignaling pathway taken from NetPath database. The protein at the arrow start denotes the upstream protein and the protein at the arrow end denotes the downstream protein. For clarity, the predicted bi-directions and the undetermined directions are not drawn.

**Figure 6:**
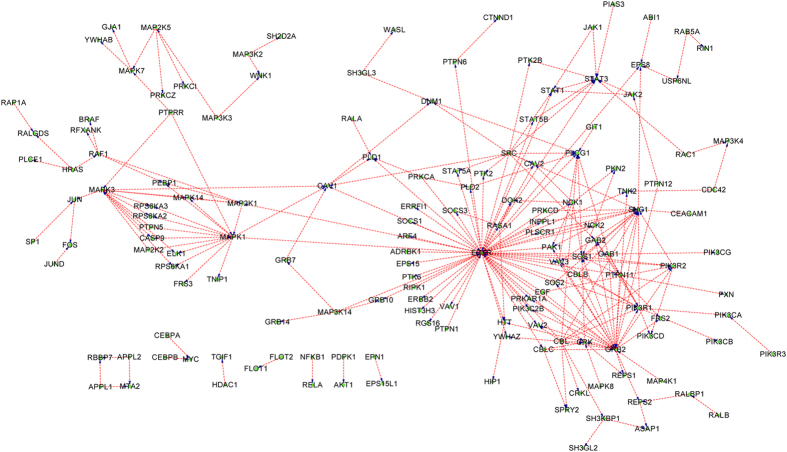
The predicted PPI upstream/downstream relations in EGFR signaling pathway taken from NetPath database. The protein at the arrow start denotes the upstream protein and the protein at the arrow end denotes the downstream protein. For clarity, the predicted bi-directions and the undetermined directions are not drawn.

**Figure 7:**
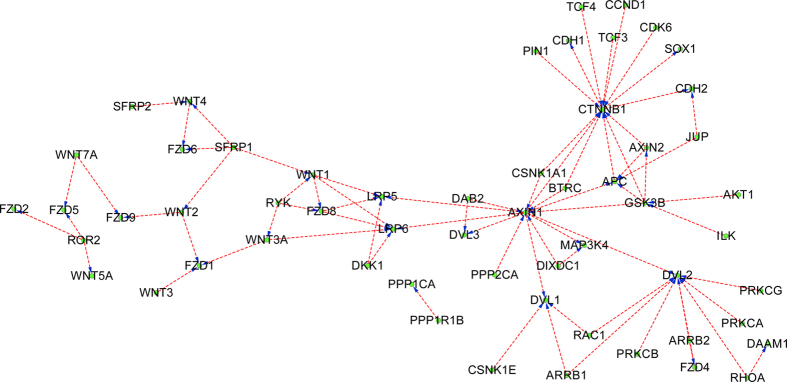
The predicted PPI upstream/downstream relations in WNT signaling pathway taken from NetPath database. The protein at the arrow start denotes the upstream protein and the protein at the arrow end denotes the downstream protein. For clarity, the predicted bi-directions and the undetermined directions are not drawn.

The final SVM classifier is trained with KEGG+NetPath-EC and then is used to predict HPRD prediction set (Phase III as illustrated in Fig. 1). Similarly, the proteins in the prediction set that do not contain any signature domains of the proteins in the training data are removed, thus HPRD is reduced to 19,736 PPIs. The results are provided in Section 3 of the supplementary file. Combining the results provided in Section 3 of in the supplementary file with the experimental KEGG+NetPath-EC, the network-wide PPI upstream/downstream relations can be easily reconstructed. Most physical PPIs in NetPath^22^ (called NetPath-PI) signaling pathways are not provided with directional annotations, so we also annotate NetPath-PI using the trained model as Phase III in Fig. 1. The predicted NetPath-PI upstream/downstream relations are provided in Section 4 of the supplementary file. Take TGF-β, TNF-α,EGFR and WNT signaling pathways for examples, the predicted upstream/downstream signal flow networks exclusive of the predicted bi-directional and undetermined are illustrated with Cytoscape^31^ as Figs 4, 5, 6, 7. Some predictions in Figs 4, 5, 6, 7 have been validated against recent literature as provided in Table 3 and will be discussed as follows. Since the latest experimental evidences are scarce and scattered among thousands of literature, literature validation is very hard, so we only provide several examples in Table 3.

#### Predicted PPI upstream/downstream relations in TGF-β signaling pathway

We use the trained SVM classifier to predict the upstream/downstream relations of physical protein-protein interactions in TGF-βsignaling pathway of NetPath database. As can be easily seen in Fig. 4, the hub proteins {SMAD2, SMAD3, SMAD4, SMAD7} are predicted to be heavily targeted. For instances, SMAD3 is predicted to be targeted by SKI (SKI--->SMAD3), TP53 (TP53--->SMAD3), ATF3 (ATF3--->SMAD3), etc., most of which have been experimentally validated. In^32^, SKI proteins have been experimentally verified to repress SMAD ability to transactivate TGFBeta target genes by disrupting the active heteromeric complexes of SMAD2 or SMAD3 with SMAD4. The relation between upstream SKI and downstream SMAD3 (SKI--->SMAD3) is validated. In^33^, experimental results have shown that the induction of cellular p53 results in the repression of TGF-induced Smad3 interaction with the Smad-binding element and with p300, and thus acts as a potent negative modulator of TGFBeta signaling. The relation between upstream TP53 and downstream SMAD3 (TP53--->SMAD3) is validated. In^34^, ATF3 knockdown has been experimentally verified to dampen the effect of SMAD3. The relation between upstream ATF3 and downstream SMAD3 (ATF3--->SMAD3) is validated. Besides being heavily targeted, we can see from Fig. 4 that SMAD3 also targets other proteins such as SP1 (SMAD3--->SP1), SKIL (SMAD4--->SKIL), AR (SMAD3---> AR), etc. These predictions are also validated by recent literature. In^35^, SMAD3 has been experimentally verified to enhance the transcriptional activity of SP1 that is fused to a Gal4 DNA-binding domain (SMAD3--->SP1). In^36^, experimental results show that the SNON-SMAD4 complex negatively regulates the basal SKIL gene expression through binding the promoter and recruiting histone deacetylases (SMAD4--->SKIL). In^37^, SMAD3 is experimentally demonstrated to repress the androgen receptor (AR) through MH2 domain to regulate the androgen-signaling pathway in prostate cancer cells. The other hub or sub-hub proteins, e.g. TGFBR1, TGFBR2, STRAP, etc., are predicted to target and to be targeted by other proteins. It is noted that Fig. 4 is only a part of TGF-βsignaling pathway that ignores those experimental PPI directions and those predicted bi-directional PPIs. Therefore, there are some orphan PPIs in Fig. 4, e.g. ROCK1--->CDC25A, BCAR1--->CRK, etc.

#### Predicted PPI upstream/downstream relations in TNF-α signaling pathway

Similar to TGF--βsignaling pathway, the hub proteins {TRAF2, RELA, NFKB1A, NFKB1B, NFRSF1A} are predicted to be heavily targeted and also target other proteins as illustrated in Fig. 5. For instances, TRAF2 is predicted to be targeted by TNFRSF1A (TNFRSF1A--->TRAF2) and to target RIPK1 (TRAF2--->RIPK1), FLNA (TRAF2--->FLNA), etc. In^38^, experimental results have shown that TNF increases TNF receptor-associated factor 1(TRAF1) and decreases TRAF2 protein expression through TNFRSF1A, indicating that TNFRSF1A--->TRAF2 is validated. In^39^, the pro-survival effect of RIPK1 is experimentally verified to be mediated by the stabilization of TRAF2 and cIAP1 (TRAF2--->RIPK1). In^40^, the directional arc TRAF2--->FLNA is experimentally claimed to have a strong positive weight. In Fig. 5, RELA is predicted to be targeted by PIAS3 (PIAS3--->RELA) and to target NFKB2 (RELA--->NFKB2). The predictions are also validated by recent literature. In^41^, it is claimed that the RELA subunit of NF-kB is sumoylated by PIAS3 (PIAS3--->RELA). In^42^, experimental results have shown that RELA transactivates the NFKB2 promoter in a dose-dependent manner (RELA--->NFKB2).

#### Predicted PPI upstream/downstream relations in EGFR signaling pathway

As illustrated in Fig. 6, the hub protein EGFR is predicted to be heavily targeted and also to target many peripheral proteins. For instance, EGFR is predicted to target GAB1 (EGFR---> GAB1), GRB2 (EGFR--->GRB2), etc. In^43^, it has experimentally verified that the phosphorylation of GAB1 by the EGFR and possibly other tyrosine kinases leads to recruitment and activation of multiple signal relay molecules, including PI3K (EGFR---> GAB1). In^44^, Epidermal Growth Factor Receptor (EGFR) is experimentally verified to recruit GRB2 to the plasma membrane upon cDNA expression (EGFR--->GRB2). In^45^, experimental results have shown that EGFR activates SRC and is phosphorylated by SRC on Tyr-845, indicating bi-directional relation between EGFR and SRC. In this work, only SRC--->EGFR is recognized (see Fig. 6). In^46^, experimental results show that GRB2 recruits SOS1 to the membrane to form GRB2-SOS1 complex upon the activation of EGFR or other RTKs. Here GRB2--->SOS1 is correctly recognized by the proposed method (see Fig. 6).

#### Predicted PPI upstream/downstream relations in WNT signaling pathway

Similarly, the hub proteins, e.g. {CTNNB1, GSK3B, DVL1, AXIN1}, are predicted to be heavily targeted or to target many other peripheral proteins as illustrated in Fig. 7. For instance, the hub protein CTNNB1 is predicted to be targeted by BTRC (BTRC---> CTNNB1), GSK3B (GSK3B--->CTNNB1), etc. In^47^, BTRC has been experimentally verified to inhibit the beta-catenin (CTNNB1) pathway that is upregulated after insults such as seizures and promotes adult neurogenesis (BTRC--->CTNNB1). In^48^, experimental results have demonstrated that *in vitro* phosphorylation of beta-catenin (CTNNB1) by GSK3 is inhibited by PPPSPxS motif peptides or by phosphorylated LRP6 cytoplasmic domain (GSK3B--->CTNNB1). In^49^, AKT has been reported to promote protein synthesis by phosphorylating and inactivating GSK3B (AKT1--->GSK3B).

## Discussion

Protein-protein interaction (PPI) networks are important infrastructures to infer signaling pathways, based on which to further understand the underlying mechanism of cell growth, cell apoptosis, organismal development and pathways-aberrant diseases. However, the current PPI networks do not carry the upstream/downstream information of two interacting proteins, preventing us to gain knowledge about signal flows in PPI networks. At present the experimentally derived PPI upstream/downstream relations are scarcely collected in such the repositories as KEGG, NetPath etc. Computational modeling is a cheap and efficient approach to the reconstruction of signal flows in PPI networks. To date there are a very few computational methods to annotate the current PPI networks with upstream/downstream information. Most of the existing computational methods heavily depend on PPI network topologies to predict the signal flows from membrane receptors to nucleus transcription factors. However, overdependence on PPI network topologies has the drawbacks (1) the network is incomplete and contains a certain level of noise so that the shortest paths are inaccurate; (2) the shortest path algorithm cannot tackle the signaling feedback loops and the cross-talks between signaling pathways; (3) the experimentally verified PPI upstream/downstream relations are not exploited The domain based method derives statistics of domain-domain upstream/downstream relations to predict PPI upstream/downstream relations. Simple as it is and it does not depend on PPI network topologies, the method cannot tackle non-linear domain combination. Similarly, the method neither exploits the experimentally verified PPI upstream/downstream relations for model training.

In this work, we propose a simple feature construction method to incorporate the domain-domain upstream/downstream relations, based on which to train a nonlinear SVM classifier to predict PPI upstream/downstream relations. Differently the proposed method does not depend on PPI network topologies but directly exploit the experimentally verified PPI upstream/downstream relations in KEGG and NetPath. For each protein pair (A, B), there are two basic directions i.e. 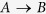, 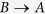 (or 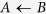), thus protein pair (A, B) can be naturally depicted with two instances 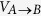 and 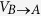. Two instances (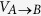, 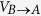) collaboratively depict the same protein pair, inherently demanding that the feature representation is asymmetrical to embody the directionality. In a sense, asymmetrical feature representation is suited to the problem of predicting the directionality between two objects. If a decision function 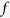 can be derived from empirical data, then a rational decision can be made according to the function scores 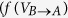, 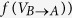. Then we encounter two problems (1) how to derive the function 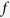; (2) how to combine 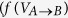 with 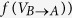 to make a decision. The first problem is addressed by defining 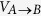 and 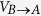 according to formula (1), treating the experimentally verified PPI directions 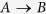 as positive class and the reverse 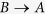 as negative class (experimentally NOT verified), and then using the feature vectors to train a nonlinear SVM classifier. The other problem is addressed by defining a semantically interpretable decision function as formula (8), based on which we further define a macro bag-level performance metric as formula (10). Two-phase cross validation and independent test as illustrated in Fig. 1 show that the proposed method achieves satisfactory micro instance-level performance and macro bag-level performance.

Lastly, we use the trained model to annotate the PPIs in HPRD, Reactome and IntAct. Nevertheless, we cannot annotate the whole PPIs in the three databases for two reasons (1) not all proteins contain already known pfam domains; (2) the training data is relatively small, so that the coverage of pfam domains is rather limited, thus the proteins that do not contain the signature domains of the training data should be removed. For the reasons, HPRD is reduced to 19,736 PPIs, Reactome is reduced to 36,242 PPIs and IntAct is reduced to 17,351. The predictions of Reactome and IntAct are provided in Section 5 and Section 6 of the supplementary file accordingly. Computational results show that the proposed method confirms 54.62% PPI upstream/downstream relations that are collected by Wu *et al.*^25^.

To enlarge the coverage of domains is the key factor for our proposed method to gain wide applicability. In our future work, we will solve the problem from the two aspects (1) augment the training data; (2) transfer the domains of the homologs to the target proteins. The training data can be augmented by borrowing some data with reliable sources that are collected by Wu *et al.*^25^. The homologous domains can be properly transferred by developing rational transfer learning methods like^8^,^9^,^10^.

## Additional Information

**How to cite this article**: Mei, S. and Zhu, H. A simple feature construction method for predicting upstream/downstream signal flow in human protein-protein interaction networks. *Sci. Rep.* **5**, 17983; doi: 10.1038/srep17983 (2015).

## Supplementary Material

Supplementary Information

## Figures and Tables

**Table 1:** The summary of data that are used as training set, validation set and network-wide prediction set.

Dataset	KEGG	NetPath-EC	NetPath-PI	HPRD	HPRD-PTM	Reactome	IntAct
Size	893	730	3216	36,416	2,547	70,557	61,462

**Table 2:** 20-fold cross validation performance estimation on KEGG and KEGG+NetPath.

	KEGG			KEGG+NetPath-EC		
	SP	SE	MCC	SP	SE	MCC
Positive	0.7560	0.7560	0.5920	0.7782	0.7835	0.6277
Negative	0.7560	0.7560	0.5920	0.7820	0.7767	0.6270
[Acc; MCC]	[75.60%; 0.5920]			[78.01%; 0.7162]		
[ROC-AUC]	[0.8399]			[0.8394]		
F1 Score	0.7560			0.7808		
Macro_accuracy	74.12%			75.87%		

*Macro_accuracy* is used to measure the reliability that the proposed method predicts upstream/downstream relations, and the other metrics are used to estimate the instance-level performance.

**Table 3:** Predicted PPI upstream/downstream relations that are validate by recent literature.

Signaling pathway	PPI upstream/downstream relations validated by recent literature
TGF-β	SKI--->SMAD3^32^; TP53--->SMAD3^33^; ATF3--->SMAD3^34^; SMAD3--->SP1^35^; SMAD3--->SKIL^36^; SMAD3--->AR^37^
TNF-α	TNFRSF1A--->TRAF^38^; TRAF2--->RIPK1^39^; TRAF2--->FLNA^40^; PIAS3--->RELA^41^; RELA--->NFKB2^42^
EGFR	EGFR--->GAB1^43^; EGFR--->GRB2^44^; SRC--->EGFR^45^; GRB2--->SOS1^46^
WNT	BTRC--->CTNNB1^47^; GSK3B--->CTNNB1^48^; AKT1--->GSK3B^49^

We take only four signaling pathways from NetPath database as examples. For each signaling pathway, only several examples are provided. The square bracketed number that follows the predictions denotes the reference number of literature.
