# Supplementary material for: A simple feature construction method for predicting upstream/downstream signal flow in human protein-protein interaction networks
Source: Sci Rep. 2015 Dec 9;5:17983. doi: 10.1038/srep17983 (PMC4673612; doi:10.1038/srep17983)
Supplement: Supplementary Information [file srep17983-s1.pdf]

A simple feature construction method for predicting upstream/downstream signal flow in human protein-protein interaction networks

Suyu Mei[①②] , Hao Zhu[②§]

[①]Software College, Shenyang Normal University, Shenyang, 110034, China

[②] Bioinformatics Section, School of Biomedical Sciences, Southern Medical University, Guangzhou, 510515, China

Email addresses: meisygle@gmail.com, [§]zhuhao@smu.edu.cn

```
[Section 1] Independent test on NetPath-EC (enzyme catalysis)

[1]     MAP3K7  <--->   MAP3K7  0.33559 0.33559 [Recognized]
[2]     TGFBR1  --->    SMAD3   0.79167 -0.85204        [Recognized]
[3]     TGFBR2  --->    PARD6A  0.23971 -0.38247        [Recognized]
[4]     EP300   <---    SMAD2   -0.011179       -0.010701       [NOT recognized]
[5]     NEDD4L  --->    SMAD2   0.33262 -0.35584        [Recognized]
[6]     TGFBR1  --->    SMAD2   0.79167 -0.85204        [Recognized]
[7]     PIAS2   <---    SMAD4   -0.02151        0.012379        [NOT recognized]
[8]     MAPK14  --->    ATF2    1.1569  -1.1226 [Recognized]
[9]     RNF111  <---    SMAD7   -0.043036       0.046892        [NOT recognized]
[10]    TGFBR1  --->    SHC1    0.15267 -0.26419        [Recognized]
[11]    SRC     --->    TGFBR2  0.36131 -0.4709 [Recognized]
[12]    PPP2CA  --->    RPS6KB2 0.5602  -0.71471        [Recognized]
[13]    PPP2R1B <---    RPS6KB2 -0.34192        0.25394 [NOT recognized]
[14]    TGFBR2  --->    CDK1    0.40075 0.18955 [Recognized]
[15]    PIAS1   <---    SMAD4   -0.02151        0.012379        [NOT recognized]
[16]    CAMK2A  --->    SMAD2   0.60922 -0.62073        [Recognized]
[17]    MAPK8   --->    SMAD3   1.0004  -0.99984        [Recognized]
[18]    SMURF2  <--->   SMURF2  0.0023737       0.0023737       [Recognized]
[19]    MAPK14  --->    SMAD3   1.0004  -0.99984        [Recognized]
[20]    PJA1    <--->   PJA1    0.015124        0.015124        [Recognized]
[21]    MAPK8   --->    JUN     1.0007  -0.99982        [Recognized]
[22]    CREBBP  <---    SMAD3   -0.011179       -0.010701       [NOT recognized]
[23]    TGFBR2  --->    TGFBR1  0.12499 0.080077        [Recognized]
[24]    ROCK1   <---    CDC25A  -0.096351       0.062899        [NOT recognized]
[25]    TGFBR2  <--->   TGFBR2  0.16963 0.16963 [Recognized]
[26]    PJA1    --->    SPTBN1  0.02195 -0.021161       [Recognized]
[27]    MAP3K7  --->    MAPK14  1.0003  -1.0004 [Recognized]
[28]    CCND1   <---    RB1     -0.081997       0.067076        [NOT recognized]
[29]    PJA1    <---    SMAD3   -0.043036       0.046892        [NOT recognized]
[30]    SMURF1  --->    RHOA    0.15868 -0.17008        [Recognized]
[31]    CSNK1G2 --->    SMAD3   1.0004  -0.99984        [Recognized]
[32]    KAT2B   <---    SMAD2   -0.054041       0.057881        [NOT recognized]
[33]    PDK1    --->    SMAD4   0.18144 -0.19122        [Recognized]
[34]    CREBBP  <---    SMAD2   -0.011179       -0.010701       [NOT recognized]
[35]    PPP2CA  --->    RPS6KB1 0.5602  -0.71471        [Recognized]
[36]    ITCH    --->    KLF10   0.10847 -0.12783        [Recognized]
[37]    SMURF1  <---    TGFBR1  -0.30105        0.22984 [NOT recognized]
[38]    PDK1    --->    SMAD3   0.18144 -0.19122        [Recognized]
[39]    EP300   <---    SMAD3   -0.011179       -0.010701       [NOT recognized]
[40]    PPM1A   --->    SMAD2   0.46651 -0.47464        [Recognized]
[41]    ITCH    --->    SMAD7   0.33262 -0.35584        [Recognized]
[42]    NEDD4L  <---    TGFBR1  -0.30105        0.22984 [NOT recognized]
[43]    SMURF1  --->    SMAD7   0.33262 -0.35584        [Recognized]
[44]    BTRC    --->    SMAD4   0.33894 -0.35179        [Recognized]
[45]    MAP2K3  <--->   MAPK14  0.4189  0.4189  [Recognized]
[46]    MAP2K6  <--->   MAPK14  0.4189  0.4189  [Recognized]
[47]    MAPK14  --->    RPS6KA5 0.59077 -0.095222       [Recognized]
[48]    MAPK14  --->    MEF2C   0.99944 -1.0001 [Recognized]
[49]    MAP3K14 <--->   MAP3K14 0.4189  0.4189  [Recognized]
[50]    IKBKB   --->    NFKBIA  0.99951 -1.0002 [Recognized]
[51]    BTRC    <---    NFKB1   -0.18432        0.16691 [NOT recognized]
[52]    CASP7   --->    IKBKB   0.15056 -0.48114        [Recognized]
[53]    PRKCZ   <---    TRAF2   -0.16536        0.15613 [NOT recognized]
[54]    ITCH    --->    CFLAR   1       -1.0001 [Recognized]
[55]    CASP3   --->    IKBKB   0.15056 -0.48114        [Recognized]
[56]    PPP2CA  --->    RELA    0.9999  -0.99984        [Recognized]
[57]    PRKCE   <---    TRAF2   -0.033537       -0.012152       [NOT recognized]
[58]    FBXW11  --->    NFKBIA  0.61683 -0.61306        [Recognized]
[59]    BIRC2   --->    RIPK1   0.99968 -1.0004 [Recognized]
[60]    PPP4C   --->    IRS4    0.75301 -0.74349        [Recognized]
[61]    BIRC2   <---    IKBKG   -0.080125       0.074051        [NOT recognized]
[62]    TRIM32  --->    XIAP    0.16821 -0.16761        [Recognized]
[63]    CASP7   <---    TNFRSF1A        -1.0005 0.99992 [NOT recognized]
[64]    TRAF2   --->    RIPK1   0.13467 -0.13265        [Recognized]
[65]    MAPKAPK2        <---    EGFR    -0.99996        1.0005  [NOT recognized]
[66]    BIRC2   --->    MAP3K5  0.18725 -0.043132       [Recognized]
[67]    CHUK    --->    NFKBIA  0.99951 -1.0002 [Recognized]
[68]    RIPK3   <---    RIPK1   0.37593 0.50335 [NOT recognized]
[69]    SRC     --->    IKBKB   0.56498 -0.67212        [Recognized]
[70]    MAP3K2  --->    NFKBIA  0.79211 -0.57759        [Recognized]
[71]    AKT1    --->    CFLAR   0.0017146       -0.0087802      [Recognized]
[72]    IKBKB   --->    NFKBIB  0.99951 -1.0002 [Recognized]
[73]    CASP8   --->    TRAF1   0.18484 -0.0074858      [Recognized]
[74]    CHUK    --->    PIAS1   0.17021 -0.3226 [Recognized]
```

```
[75]    PPP2R1B <---    RELA    -0.22861        0.23047 [NOT recognized]
[76]    MAP2K1  <---    RAF1    -0.99998        0.99952 [NOT recognized]
[77]    RPS6KB1 --->    IRS1    0.9997  -1.0007 [Recognized]
[78]    IKBKB   --->    TSC1    0.99942 -0.99994        [Recognized]
[79]    SRC     <---    ERBB2   -1.0005 0.99949 [NOT recognized]
[80]    IKBKB   --->    RELA    0.61419 -0.83604        [Recognized]
[81]    RPS6KA5 --->    RELA    0.62613 -0.74502        [Recognized]
[82]    MAPK8   --->    SENP1   0.68271 -0.69465        [Recognized]
[83]    PRKCA   --->    ARHGEF1 0.73698 -0.82216        [Recognized]
[84]    AKT1    --->    CHUK    0.40447 -0.41722        [Recognized]
[85]    PRKCZ   --->    RELA    0.40051 -0.38518        [Recognized]
[86]    MAP2K4  <--->   MAPK8   0.4189  0.4189  [Recognized]
[87]    BIRC2   <---    TRAF2   -0.84607        0.82841 [NOT recognized]
[88]    BTRC    --->    NFKBIB  0.61683 -0.61306        [Recognized]
[89]    SRC     --->    NFKBIA  0.50904 -0.58207        [Recognized]
[90]    BTRC    --->    NFKBIA  0.61683 -0.61306        [Recognized]
[91]    IKBKB   <---    IKBKB   -0.92648        -0.92648        [NOT recognized]
[92]    CASP7   <---    PRKCE   -0.56943        0.44413 [NOT recognized]
[93]    PRKCD   --->    TNFRSF1A        0.15901 -0.21253        [Recognized]
[94]    MAP2K4  <--->   MAPK9   0.4189  0.4189  [Recognized]
[95]    RIPK1   <--->   RIPK1   0.15068 0.15068 [Recognized]
[96]    MAP3K3  --->    NFKBIA  0.79211 -0.57759        [Recognized]
[97]    PRKCD   <---    TRAF2   -0.24631        0.20702 [NOT recognized]
[98]    CHUK    --->    NFKBIB  0.99951 -1.0002 [Recognized]
[99]    MYLK    --->    MYL9    0.21664 -0.2004 [Recognized]
[100]   MAP3K3  --->    IKBKB   0.90816 -0.934  [Recognized]
[101]   CHUK    --->    NFKB1   1.0002  -0.99999        [Recognized]
[102]   CASP8   <---    RIPK1   -0.34669        0.33205 [NOT recognized]
[103]   CHUK    --->    RELA    0.61419 -0.83604        [Recognized]
[104]   MAP3K1  --->    NFKBIA  1.0497  -1.0004 [Recognized]
[105]   RFFL    <---    RIPK1   -0.33836        0.3795  [NOT recognized]
[106]   IKBKB   --->    DOK1    0.41633 -0.63246        [Recognized]
[107]   TBK1    --->    NFKBIA  1.0497  -1.0004 [Recognized]
[108]   TNFAIP3 <---    RIPK1   -0.45843        0.47225 [NOT recognized]
[109]   SRC     --->    CHUK    0.56498 -0.67212        [Recognized]
[110]   PRKCA   <--->   PRKCA   0.052918        0.052918        [Recognized]
[111]   RIPK1   --->    MAP3K1  0.99976 -1.0003 [Recognized]
[112]   MAP3K1  <--->   MAP3K1  0.4189  0.4189  [Recognized]
[113]   PRKCD   --->    NCF1    0.30177 -0.37686        [Recognized]
[114]   MAP3K14 --->    CHUK    1       -0.99981        [Recognized]
[115]   MAP3K8  <--->   MAP2K1  0.4189  0.4189  [Recognized]
[116]   AKT2    --->    CHUK    0.40447 -0.41722        [Recognized]
[117]   CSNK2A1 --->    RELA    0.99973 -0.99955        [Recognized]
[118]   IKBKB   --->    NFKB1   1.0002  -0.99999        [Recognized]
[119]   HSPB1   <---    NFKBIA  -0.024824       0.094274        [NOT recognized]
[120]   MAP2K7  <--->   MAPK8   0.4189  0.4189  [Recognized]
[121]   CASP8   --->    MAP3K14 0.32725 -0.22955        [Recognized]
[122]   PTPRC   <---    LCK     -0.37285        0.31189 [NOT recognized]
[123]   ITK     --->    PLCG1   1.0002  -1      [Recognized]
[124]   ZAP70   --->    VAV1    0.099509        0.057504        [Recognized]
[125]   ITK     --->    LAT     0.28825 -0.47405        [Recognized]
[126]   PTPN7   <---    MAPK3   -0.4667 0.45882 [NOT recognized]
[127]   LCK     <---    VAV1    -0.026855       0.038756        [NOT recognized]
[128]   SYK     --->    DUSP3   0.16349 -0.12488        [Recognized]
[129]   ZAP70   --->    DUSP3   0.16349 -0.12488        [Recognized]
[130]   IKBKB   --->    NFKBIA  0.99951 -1.0002 [Recognized]
[131]   FYN     --->    CTLA4   0.46959 -0.56208        [Recognized]
[132]   FYN     <---    TXK     -0.32358        -0.32358        [NOT recognized]
[133]   ITK     <---    VAV1    -0.038353       -0.0047825      [NOT recognized]
[134]   PTPN6   <---    ZAP70   0.092992        0.33247 [NOT recognized]
[135]   ZAP70   --->    LAT     1.0007  -0.99996        [Recognized]
[136]   PTPRJ   --->    PLCG1   0.23651 -0.27768        [Recognized]
[137]   CHUK    --->    NFKBIA  0.99951 -1.0002 [Recognized]
[138]   LCK     <---    ZAP70   -0.138  0.47881 [NOT recognized]
[139]   LCK     --->    LCP2    0.12849 -0.10975        [Recognized]
[140]   FYN     --->    WAS     0.31751 -0.38602        [Recognized]
[141]   LCK     --->    GAB2    0.49307 -0.5279 [Recognized]
[142]   MAPK14  --->    ATF2    1.1569  -1.1226 [Recognized]
[143]   LCK     --->    CTLA4   0.46959 -0.56208        [Recognized]
[144]   FYN     <---    VAV1    -0.026855       0.038756        [NOT recognized]
[145]   ZAP70   --->    SHC1    0.38024 -0.055938       [Recognized]
[146]   LCK     --->    ITK     -0.048827       -0.64383        [Recognized]
[147]   ZAP70   --->    GAB2    0.68332 -0.54983        [Recognized]
[148]   MAPK11  <--->   MAPK11  0.4189  0.4189  [Recognized]
[149]   MAPK14  <--->   MAPK14  0.4189  0.4189  [Recognized]
[150]   PRKCQ   <---    STK39   0.0098884       0.47787 [NOT recognized]
```

[151]	DTX1	<---	MAP3K1	-0.43693	0.41786	[NOT recognized]
[152]	FCGR3A	<---	ZAP70	-0.99982	1.0001	[NOT recognized]
[153]	PTK2B	<---	PTK2B	-0.1406	-0.1406	[NOT recognized]
[154]	FYN	--->	ITPR1	0.20652	-0.24022	[Recognized]
[155]	CBL	--->	ZAP70	0.22725	-0.23567	[Recognized]
[156]	PTPN12	--->	WAS	0.14105	-0.15956	[Recognized]
[157]	PTPRJ	<---	LAT	-0.11921	0.11583	[NOT recognized]
[158]	FYN	--->	FYB	0.33631	-0.51542	[Recognized]
[159]	FYN	--->	LCP2	0.12849	-0.10975	[Recognized]
[160]	CSK	<---	LCK	-0.32358	-0.32358	[NOT recognized]
[161]	MAPK14	--->	STAT4	0.32122	-0.3529	[Recognized]
[162]	ZAP70	--->	SH2B3	0.53451	0.060859	[Recognized]
[163]	FCGR3A	<---	SYK	-0.99982	1.0001	[NOT recognized]
[164]	MAPK9	--->	JUN	1.0007	-0.99982	[Recognized]
[165]	MAP4K1	--->	CARD11	0.63309	-0.48191	[Recognized]
[166]	ZAP70	<--->	ZAP70	0.35049	0.35049	[Recognized]
[167]	FYN	--->	SKAP1	0.28601	-0.3192	[Recognized]
[168]	PTPRH	<---	LCK	-0.37285	0.31189	[NOT recognized]
[169]	ZAP70	--->	DBNL	0.65394	-0.61718	[Recognized]
[170]	PTPRC	<---	FYN	-0.37285	0.31189	[NOT recognized]
[171]	MAP2K1	<--->	MAPK1	0.4189	0.4189	[Recognized]
[172]	MAP2K7	<--->	MAPK8	0.4189	0.4189	[Recognized]
[173]	PPP3CB	--->	NFATC2	0.99972	-0.99927	[Recognized]
[174]	LCK	--->	SH2B3	0.20536	0.0037779	[Recognized]
[175]	ZAP70	--->	LCP2	0.4075	-0.1337	[Recognized]
[176]	CBL	--->	LCK	0.091374	-0.12869	[Recognized]
[177]	PSEN1	--->	NOTCH4	0.064129	-0.096246	[Recognized]
[178]	GSK3B	--->	NOTCH2	0.16574	-0.18729	[Recognized]
[179]	MFNG	<---	DLL1	-0.080021	0.072736	[NOT recognized]
[180]	ADAM10	<---	DLL1	-0.038403	0.016314	[NOT recognized]
[181]	MAPK3	--->	TCF3	1.0104	-1.02	[Recognized]
[182]	PSEN1	<---	JAG2	-0.012	-0.0065656	[NOT recognized]
[183]	PSEN1	--->	NOTCH2	0.13646	-0.15856	[Recognized]
[184]	NUMB	--->	NOTCH1	1	-0.99985	[Recognized]
[185]	MFNG	--->	NOTCH2	0.34747	-0.34935	[Recognized]
[186]	LFNG	--->	NOTCH1	0.99975	-0.99968	[Recognized]
[187]	MFNG	<---	JAG1	-0.049898	0.044126	[NOT recognized]
[188]	ADAM17	--->	NOTCH1	1	-1.0002	[Recognized]
[189]	CBL	--->	NOTCH3	0.08658	-0.12235	[Recognized]
[190]	PSEN1	--->	NOTCH1	0.41036	-0.4651	[Recognized]
[191]	LFNG	--->	NOTCH2	0.34747	-0.34935	[Recognized]
[192]	ARRDC1	--->	NOTCH1	0.23729	-0.28248	[Recognized]
[193]	PSEN1	--->	NOTCH3	0.41036	-0.4651	[Recognized]
[194]	PRKCQ	--->	CBL	0.015972	-0.05525	[Recognized]
[195]	MAPK1	--->	TCF3	1.0104	-1.02	[Recognized]
[196]	PSEN1	--->	DLL1	0.025183	-0.034141	[Recognized]
[197]	MIB1	--->	DLL1	-0.0038638	-0.017216	[Recognized]
[198]	CSNK2A1	--->	PTEN	0.0050355	-0.016846	[Recognized]
[199]	JAK2	--->	STAT3	0.27681	-0.56742	[Recognized]
[200]	PTPN1	--->	STAT3	0.30458	-0.33308	[Recognized]
[201]	MAP2K1	<--->	MAPK3	0.4189	0.4189	[Recognized]
[202]	PTPN11	<---	JAK2	0.092992	0.33247	[NOT recognized]
[203]	PRKCD	--->	IRS1	0.83388	-0.82324	[Recognized]
[204]	SRC	--->	STAT3	0.12044	-0.35851	[Recognized]
[205]	MAPK14	--->	ATF1	1.1569	-1.1226	[Recognized]
[206]	JAK2	--->	IRS1	0.44988	-0.37277	[Recognized]
[207]	JAK2	--->	STAT5B	0.27681	-0.56742	[Recognized]
[208]	FYN	--->	STAT3	0.12044	-0.35851	[Recognized]
[209]	JAK2	--->	IRS2	0.44988	-0.37277	[Recognized]
[210]	JAK2	--->	STAT1	0.31398	-0.74719	[Recognized]
[211]	JAK2	<--->	JAK2	0.35049	0.35049	[Recognized]
[212]	JAK2	--->	LEPR	0.90718	-0.87271	[Recognized]
[213]	PTPN1	<---	JAK2	-0.52985	0.56715	[NOT recognized]
[214]	AKT1	<---	GSK3B	0.064251	0.16903	[NOT recognized]
[215]	CDK5	--->	STAT3	0.32122	-0.3529	[Recognized]
[216]	GSK3B	--->	PTEN	0.0050355	-0.016846	[Recognized]
[217]	JAK3	<--->	JAK3	0.33559	0.33559	[Recognized]
[218]	MTOR	--->	AKT1	0.235	-0.25223	[Recognized]
[219]	MAPK1	--->	ELK1	1.0002	-1.0006	[Recognized]
[220]	AKT1	<---	GSK3A	0.064251	0.16903	[NOT recognized]
[221]	KIT	<---	FES	0.065364	0.13793	[NOT recognized]
[222]	SRC	<---	KIT	0.026338	0.072439	[NOT recognized]
[223]	AKT1	--->	BAD	0.56932	-0.67709	[Recognized]
[224]	LYN	<---	LYN	-0.32358	-0.32358	[NOT recognized]
[225]	MAPK1	--->	MITF	1.0104	-1.02	[Recognized]
[226]	KIT	--->	STAT1	0.27735	-0.29878	[Recognized]

[227]	KIT	--->	STAP1	0.086866	-0.10902	[Recognized]
[228]	KIT	--->	SPRED2	0.0010633	-0.014605	[Recognized]
[229]	KIT	--->	VAV1	-0.0086242	-0.019088	[Recognized]
[230]	MAPK3	--->	ELK1	1.0002	-1.0006	[Recognized]
[231]	KIT	--->	STAT5A	0.28105	-0.29809	[Recognized]
[232]	TEC	--->	DOK1	0.22383	-0.40316	[Recognized]
[233]	FYN	<---	KIT	0.026338	0.072439	[NOT recognized]
[234]	TEC	<---	TEC	-0.23569	-0.23569	[NOT recognized]
[235]	KIT	--->	SPRED1	0.0010633	-0.014605	[Recognized]
[236]	PRKCA	--->	KIT	0.055754	-0.089549	[Recognized]
[237]	RPS6KA1	--->	MITF	0.63259	-0.75742	[Recognized]
[238]	LYN	--->	DOK1	0.50189	-0.57993	[Recognized]
[239]	PTPN6	<---	KIT	-0.045455	0.023689	[NOT recognized]
[240]	PRKCB	--->	KIT	0.055754	-0.089549	[Recognized]
[241]	KIT	--->	PIK3R1	0.035237	-0.056032	[Recognized]
[242]	PDPK1	--->	AKT1	0.72803	-0.76568	[Recognized]
[243]	KIT	<---	KIT	-0.010204	-0.010204	[NOT recognized]
[244]	KIT	--->	STAT5B	0.28105	-0.29809	[Recognized]
[245]	IKBKB	--->	NFKBIA	0.99951	-1.0002	[Recognized]
[246]	MAP3K7	--->	MAP2K6	1.0003	-1.0004	[Recognized]
[247]	MAPK14	--->	NFATC1	1	-1.0006	[Recognized]
[248]	MAPK14	--->	ATF2	1.1569	-1.1226	[Recognized]
[249]	MTOR	<---	MTOR	-0.01062	-0.01062	[NOT recognized]
[250]	MAPK8	--->	JUN	1.0007	-0.99982	[Recognized]
[251]	MAPK14	--->	MITF	1.0104	-1.02	[Recognized]
[252]	MAP2K7	<--->	MAPK9	0.4189	0.4189	[Recognized]
[253]	CAPN1	--->	NFKBIA	0.13018	-0.11511	[Recognized]
[254]	PTPN6	--->	GAB2	0.24323	-0.2299	[Recognized]
[255]	MAP3K8	<--->	MAP2K1	0.4189	0.4189	[Recognized]
[256]	SRC	--->	ABL1	-0.094124	-0.48527	[Recognized]
[257]	TRAF6	--->	FHL2	0.29122	-0.29634	[Recognized]
[258]	MAP2K7	<--->	MAPK8	0.4189	0.4189	[Recognized]
[259]	FYN	<---	CBL	-0.12869	0.091374	[NOT recognized]
[260]	JAK2	--->	PTPN11	0.33247	0.092992	[Recognized]
[261]	PTPN11	<---	JAK2	0.092992	0.33247	[NOT recognized]
[262]	JAK2	--->	HLTF	0.20452	-0.2098	[Recognized]
[263]	LYN	<---	LYN	-0.32358	-0.32358	[NOT recognized]
[264]	PTPN1	--->	STAT5A	0.30458	-0.33308	[Recognized]
[265]	JAK2	<---	ERBB4	-0.51417	0.77182	[NOT recognized]
[266]	RAF1	--->	MAP2K1	0.99952	-0.99998	[Recognized]
[267]	PTPN2	--->	STAT5B	0.30458	-0.33308	[Recognized]
[268]	FYN	<---	FYN	-0.32358	-0.32358	[NOT recognized]
[269]	JAK2	--->	PAK1	0.80776	-0.67827	[Recognized]
[270]	JAK2	<--->	JAK2	0.35049	0.35049	[Recognized]
[271]	JAK2	--->	GRB2	0.47299	0.18843	[Recognized]
[272]	PTPN2	--->	STAT5A	0.30458	-0.33308	[Recognized]
[273]	PTPN1	--->	STAT5B	0.30458	-0.33308	[Recognized]
[274]	PPP2CA	--->	AXIN1	0.43162	-0.44814	[Recognized]
[275]	GSK3B	<---	ROR2	-0.25032	0.22899	[NOT recognized]
[276]	CSNK1E	<---	DVL3	-0.57024	0.55514	[NOT recognized]
[277]	ROR2	<---	YWHAB	-0.010369	-0.0015893	[NOT recognized]
[278]	ILK	--->	GSK3B	0.29252	-0.2795	[Recognized]
[279]	MAP3K7	<--->	MAP3K7	0.33559	0.33559	[Recognized]
[280]	MARK2	<---	DVL3	-0.57024	0.55514	[NOT recognized]
[281]	NLK	--->	TCF4	1.0104	-1.02	[Recognized]
[282]	GSK3B	--->	CTNNB1	1.0829	-1.1043	[Recognized]
[283]	CSNK1E	--->	APC	0.13332	-0.16268	[Recognized]
[284]	NLK	--->	SETDB1	0.42638	-0.43821	[Recognized]
[285]	CSNK1E	<---	DVL1	-0.57024	0.55514	[NOT recognized]
[286]	CSNK1D	<---	DVL2	-0.57024	0.55514	[NOT recognized]
[287]	PRKCG	<---	DVL2	-0.064155	0.028923	[NOT recognized]
[288]	NLK	--->	LEF1	1	-1	[Recognized]
[289]	PRKCB	<---	DVL2	-0.064155	0.028923	[NOT recognized]
[290]	CSNK1D	<---	DVL3	-0.57024	0.55514	[NOT recognized]
[291]	GSK3A	--->	LRP6	0.91683	-0.93253	[Recognized]
[292]	NLK	<--->	NLK	0.4189	0.4189	[Recognized]
[293]	CSNK1A1	--->	CTNNB1	1.0829	-1.1043	[Recognized]
[294]	PRKACA	--->	PPP1R1B	1.0001	-1.0001	[Recognized]
[295]	PRKCA	<---	DVL2	-0.064155	0.028923	[NOT recognized]
[296]	GSK3A	<---	ROR2	-0.25032	0.22899	[NOT recognized]
[297]	CSNK1E	<---	DVL2	-0.57024	0.55514	[NOT recognized]
[298]	SMURF2	--->	ID3	0.17716	-0.19773	[Recognized]
[299]	PRKCA	--->	ID3	0.76122	-0.84719	[Recognized]
[300]	PRKCA	--->	ID1	0.76122	-0.84719	[Recognized]
[301]	CDK2	--->	ID2	1.0104	-1.02	[Recognized]
[302]	PRKCA	--->	ID2	0.76122	-0.84719	[Recognized]

[303]	CDK2	--->	ID3	1.0104	-1.02	[Recognized]
[304]	SMURF2	--->	ID1	0.17716	-0.19773	[Recognized]
[305]	MAPK14	--->	ATF2	1.1569	-1.1226	[Recognized]
[306]	JAK2	<--->	JAK2	0.35049	0.35049	[Recognized]
[307]	SRC	--->	IRS1	0.31818	-0.34638	[Recognized]
[308]	RPS6KB1	--->	RPS6	0.99939	-0.99992	[Recognized]
[309]	AKT1	<---	GSK3A	0.064251	0.16903	[NOT recognized]
[310]	AKT1	--->	BAD	0.56932	-0.67709	[Recognized]
[311]	PRKCD	--->	CREB1	0.80849	-0.86589	[Recognized]
[312]	SRC	--->	AKT1	0.21033	-0.33587	[Recognized]
[313]	CAMKK2	--->	AKT1	0.16903	0.064251	[Recognized]
[314]	CAMKK2	<--->	PRKAA1	0.4189	0.4189	[Recognized]
[315]	SRC	--->	PTPN6	0.082985	0.027744	[Recognized]
[316]	PRKAA2	--->	NOS3	0.41751	-0.44164	[Recognized]
[317]	MTOR	--->	RPS6KB1	0.3958	-0.4123	[Recognized]
[318]	PTPN6	<---	SRC	0.027744	0.082985	[NOT recognized]
[319]	AKT1	<---	GSK3B	0.064251	0.16903	[NOT recognized]
[320]	AKT1	--->	FOXO1	0.53688	-0.61874	[Recognized]
[321]	AKT1	--->	NOS3	0.43773	-0.47547	[Recognized]
[322]	CAMKK2	<--->	PRKAA2	0.4189	0.4189	[Recognized]
[323]	HHAT	--->	SHH	0.52943	-0.52999	[Recognized]
[324]	DYRK1A	--->	GLI1	1.0004	-1.0001	[Recognized]
[325]	PRKACA	--->	GLI3	1.0004	-1.0001	[Recognized]
[326]	ADRBK1	--->	SMO	0.31295	-0.35567	[Recognized]
[327]	PRKACA	--->	GLI1	1.0004	-1.0001	[Recognized]
[328]	SRC	<---	SRC	-0.32358	-0.32358	[NOT recognized]
[329]	PRKCZ	--->	AGER	0.23617	-0.24027	[Recognized]
[330]	CDK9	--->	AR	1.0001	-1.0004	[Recognized]
[331]	EP300	<---	AR	-0.018429	-0.0036155	[NOT recognized]
[332]	CDK5	--->	AR	1.0001	-1.0004	[Recognized]
[333]	CCND3	<---	AR	-0.54646	0.53635	[NOT recognized]
[334]	RNF6	<---	AR	-0.18864	0.18922	[NOT recognized]
[335]	KAT2B	<---	AR	-0.19965	0.2002	[NOT recognized]
[336]	CASP3	--->	AR	-0.0026381	-0.036683	[Recognized]
[337]	CASP7	--->	AR	-0.0026381	-0.036683	[Recognized]
[338]	CDK1	--->	AR	1.0001	-1.0004	[Recognized]
[339]	CASP8	<---	AR	0.041035	0.053651	[NOT recognized]
[340]	MDM2	--->	AR	0.033776	-0.052501	[Recognized]
[341]	PAK6	--->	AR	0.54562	-0.41761	[Recognized]
[342]	HDAC1	<---	AR	-0.020129	0.020201	[NOT recognized]
[343]	SENP1	<---	HDAC1	-0.24239	0.26263	[NOT recognized]
[344]	AKT1	--->	AR	0.5984	-0.66142	[Recognized]
[345]	GSK3B	--->	AR	1.0001	-1.0004	[Recognized]
[346]	CASP1	<---	AR	-0.04587	0.011431	[NOT recognized]
[347]	ITK	--->	PLCG1	1.0002	-1	[Recognized]
[348]	SYK	--->	BLNK	0.61082	0.060224	[Recognized]
[349]	SYK	--->	GAB2	0.68332	-0.54983	[Recognized]
[350]	PRKCB	--->	CHUK	0.14107	0.14095	[Recognized]
[351]	IKBKB	--->	NFKBIA	0.99951	-1.0002	[Recognized]
[352]	CDK7	<--->	CDK2	0.4189	0.4189	[Recognized]
[353]	RPS6KB1	--->	RPS6	0.99939	-0.99992	[Recognized]
[354]	SYK	--->	SH3BP2	0.53451	0.060859	[Recognized]
[355]	MAPK1	--->	ELK1	1.0002	-1.0006	[Recognized]
[356]	SYK	--->	CD79A	0.65039	-0.6231	[Recognized]
[357]	PTPN6	--->	LCP2	0.21571	-0.086008	[Recognized]
[358]	AKT1	<---	GSK3A	0.064251	0.16903	[NOT recognized]
[359]	TEC	--->	PLCG2	1.0002	-1	[Recognized]
[360]	BTK	--->	PIK3AP1	0.22036	-0.39907	[Recognized]
[361]	SYK	--->	HCLS1	0.4916	-0.31761	[Recognized]
[362]	CHUK	--->	NFKBIA	0.99951	-1.0002	[Recognized]
[363]	PDPK2	--->	AKT1	0.16903	0.064251	[Recognized]
[364]	SYK	<---	CBL	-0.23567	0.22725	[NOT recognized]
[365]	PTK2	<---	PTK2	-0.1406	-0.1406	[NOT recognized]
[366]	LYN	<---	LYN	-0.32358	-0.32358	[NOT recognized]
[367]	CBL	--->	LYN	0.091374	-0.12869	[Recognized]
[368]	MAPK1	--->	BCL6	0.80211	-0.81576	[Recognized]
[369]	PTPN18	<---	TEC	-0.38005	0.20142	[NOT recognized]
[370]	PTPN6	--->	CD79A	0.30408	-0.30043	[Recognized]
[371]	MAPK14	--->	ATF2	1.1569	-1.1226	[Recognized]
[372]	SYK	--->	PLCG2	0.08731	-0.50779	[Recognized]
[373]	ITK	--->	PLCG2	1.0002	-1	[Recognized]
[374]	SYK	--->	PLCG1	0.08731	-0.50779	[Recognized]
[375]	PRKCB	--->	BTK	-0.010775	-0.032298	[Recognized]
[376]	CBLB	--->	SYK	0.22725	-0.23567	[Recognized]
[377]	TEC	--->	PLCG1	1.0002	-1	[Recognized]
[378]	SYK	--->	MAPK3	1.0339	-1.0323	[Recognized]

[379]	SYK	--->	SHC1	0.38024	-0.055938	[Recognized]
[380]	CDK2	--->	RB1	0.5575	-0.57598	[Recognized]
[381]	PRKCQ	--->	CARD11	1.0005	-0.99993	[Recognized]
[382]	CDK4	--->	RB1	0.5575	-0.57598	[Recognized]
[383]	LYN	--->	CD79A	0.46959	-0.56208	[Recognized]
[384]	PRKCB	--->	RASGRP3	0.11253	-0.049894	[Recognized]
[385]	PRKCQ	--->	RASGRP3	0.078761	-0.011683	[Recognized]
[386]	MAP3K7	--->	IKBKB	0.99965	-1.0008	[Recognized]
[387]	LYN	--->	ITPR1	0.20652	-0.24022	[Recognized]
[388]	RPS6KA1	--->	CREB1	0.78051	-0.87159	[Recognized]
[389]	LYN	<---	BCAR1	-0.36108	0.36268	[NOT recognized]
[390]	MAPK8	--->	JUN	1.0007	-0.99982	[Recognized]
[391]	LYN	--->	CD22	0.19898	-0.30084	[Recognized]
[392]	SYK	--->	RPS6KA1	0.51314	-0.6331	[Recognized]
[393]	MAPK1	<---	BRAF	-0.99998	0.99952	[NOT recognized]
[394]	PRKCB	--->	CARD11	1.0001	-0.99941	[Recognized]
[395]	PRKD1	<---	PRKD1	-0.040523	-0.040523	[NOT recognized]
[396]	LYN	--->	GAB2	0.49307	-0.5279	[Recognized]
[397]	LCK	<---	SYK	-0.138	0.47881	[NOT recognized]
[398]	PRKD1	<---	SYK	-0.2371	0.27016	[NOT recognized]
[399]	PRKD1	--->	PLCG1	0.19147	-0.26442	[Recognized]
[400]	PPP3CA	--->	NFATC3	0.9999	-0.99984	[Recognized]
[401]	CASP7	<---	LYN	-0.42898	0.3178	[NOT recognized]
[402]	TEC	--->	DOK1	0.22383	-0.40316	[Recognized]
[403]	CASP9	<---	LYN	-0.31049	0.23033	[NOT recognized]
[404]	TEC	--->	PTPN18	0.20142	-0.38005	[Recognized]
[405]	LYN	--->	CD19	0.68171	-0.79057	[Recognized]
[406]	TEC	<---	TEC	-0.23569	-0.23569	[NOT recognized]
[407]	SYK	--->	RASA1	0.18118	0.050579	[Recognized]
[408]	SYK	<--->	SYK	0.35049	0.35049	[Recognized]
[409]	LCK	--->	CD79B	0.46959	-0.56208	[Recognized]
[410]	LYN	<---	SYK	-0.138	0.47881	[NOT recognized]
[411]	LYN	--->	BTK	-0.048827	-0.64383	[Recognized]
[412]	PPP3CA	--->	NFATC2	0.99972	-0.99927	[Recognized]
[413]	BTK	--->	GTF2I	0.22475	-0.40664	[Recognized]
[414]	PTPN6	--->	CD79B	0.30408	-0.30043	[Recognized]
[415]	LYN	<---	HCLS1	0.080822	0.12656	[NOT recognized]
[416]	BTK	--->	PLCG1	1.0002	-1	[Recognized]
[417]	LYN	--->	CD79B	0.46959	-0.56208	[Recognized]
[418]	LYN	--->	BANK1	0.48029	-0.56036	[Recognized]
[419]	PRKCB	--->	IKBKB	0.14107	0.14095	[Recognized]
[420]	MAPK8	--->	ATF2	1.1569	-1.1226	[Recognized]
[421]	BTK	--->	PLCG2	1.0002	-1	[Recognized]
[422]	LCK	--->	CD79A	0.46959	-0.56208	[Recognized]
[423]	AKT1	<---	GSK3B	0.064251	0.16903	[NOT recognized]
[424]	SYK	--->	CD79B	0.65039	-0.6231	[Recognized]
[425]	PDPK1	--->	AKT1	0.72803	-0.76568	[Recognized]
[426]	AKT1	--->	FOXO1	0.53688	-0.61874	[Recognized]
[427]	BTK	<---	BTK	-0.23569	-0.23569	[NOT recognized]
[428]	TEC	<---	STAP1	-0.22191	-0.0078052	[NOT recognized]
[429]	MAPKAPK2	--->	CREB1	1.1569	-1.1226	[Recognized]
[430]	SYK	--->	PIK3AP1	0.65204	-0.61511	[Recognized]
[431]	FYN	<---	SYK	-0.138	0.47881	[NOT recognized]
[432]	CDK6	--->	RB1	0.5575	-0.57598	[Recognized]
[433]	PTPN6	--->	BLNK	0.29455	0.13878	[Recognized]
[434]	LYN	--->	TEC	-0.048827	-0.64383	[Recognized]
[435]	EGFR	--->	PLCG1	1.0007	-0.99988	[Recognized]
[436]	STAM2	--->	HGS	0.24756	-0.015597	[Recognized]
[437]	MAPK1	--->	PTPN5	0.45882	-0.4667	[Recognized]
[438]	EGFR	--->	PLD2	0.11138	-0.12819	[Recognized]
[439]	MAP2K2	<--->	MAP2K2	0.4189	0.4189	[Recognized]
[440]	MAPK8	--->	ELK1	1.0002	-1.0006	[Recognized]
[441]	ITCH	--->	SH3GL2	0.079613	-0.10005	[Recognized]
[442]	MTOR	--->	AKT1	0.235	-0.25223	[Recognized]
[443]	MAPK1	--->	ELK1	1.0002	-1.0006	[Recognized]
[444]	PTK6	--->	ANKS1A	0.056671	-0.076519	[Recognized]
[445]	MAPK1	--->	SH2D3C	0.31325	-0.32527	[Recognized]
[446]	PAK1	--->	NCOA3	0.15352	-0.035919	[Recognized]
[447]	KSR1	--->	RAF1	0.24203	-0.072696	[Recognized]
[448]	EGFR	--->	GRB2	1.0213	-0.99964	[Recognized]
[449]	EGFR	--->	JAK1	0.45132	-0.34567	[Recognized]
[450]	PRKCA	--->	PLD1	0.19309	-0.22364	[Recognized]
[451]	MAPK1	--->	SMAD2	1.0004	-0.99984	[Recognized]
[452]	PTPRR	<---	MAPK1	-0.4667	0.45882	[NOT recognized]
[453]	MAP2K1	<--->	MAPK3	0.4189	0.4189	[Recognized]
[454]	MAPK7	--->	ELK4	1.0002	-1.0006	[Recognized]

[455]	MAPK1	--->	FRS2	0.88845	-0.87379	[Recognized]
[456]	EGFR	<---	CEACAM1	-0.12956	0.10084	[NOT recognized]
[457]	RPS6KA3	--->	HIST3H3	0.41116	-0.53711	[Recognized]
[458]	PTPRR	<---	MAPK7	-0.4667	0.45882	[NOT recognized]
[459]	MAPK3	--->	PTPN11	0.052269	-0.065336	[Recognized]
[460]	PRKCI	<---	EGFR	-0.18501	0.17566	[NOT recognized]
[461]	SRC	<---	EGFR	-1.0005	0.99949	[NOT recognized]
[462]	PTK6	--->	PXN	0.048406	-0.055088	[Recognized]
[463]	MAP3K3	<--->	MAP3K3	0.3876	0.3876	[Recognized]
[464]	MAPK3	--->	PTPRR	0.45882	-0.4667	[Recognized]
[465]	EGFR	--->	PTPN6	0.50924	-0.50828	[Recognized]
[466]	PAK1	--->	MAP2K1	0.70126	-0.44433	[Recognized]
[467]	MAP3K3	<--->	MAP2K5	0.3876	0.3876	[Recognized]
[468]	EGFR	--->	SHC1	1.0008	-0.99991	[Recognized]
[469]	SRC	--->	SH2D3C	0.21045	-0.018697	[Recognized]
[470]	RAF1	--->	MAP2K1	0.99952	-0.99998	[Recognized]
[471]	MAPK3	--->	STAT3	0.32122	-0.3529	[Recognized]
[472]	RPS6KA5	--->	CREB1	0.78051	-0.87159	[Recognized]
[473]	PTPN11	<---	PXN	-0.061679	0.051856	[NOT recognized]
[474]	MAP2K1	<--->	MAP2K1	0.4189	0.4189	[Recognized]
[475]	ITCH	<--->	ITCH	0.0023737	0.0023737	[Recognized]
[476]	PTPN6	--->	CTNND1	0.53782	-0.53747	[Recognized]
[477]	PTPN5	<---	MAPK3	-0.4667	0.45882	[NOT recognized]
[478]	EGFR	--->	JAK2	0.77182	-0.51417	[Recognized]
[479]	MAPK3	--->	ELK1	1.0002	-1.0006	[Recognized]
[480]	MAPK1	--->	RPS6KA5	0.59077	-0.095222	[Recognized]
[481]	MAPK1	--->	KLF11	1.0402	-1.0441	[Recognized]
[482]	EGFR	--->	STAT3	0.99977	-0.9992	[Recognized]
[483]	AKT1	--->	FOXO4	0.53688	-0.61874	[Recognized]
[484]	EGFR	--->	VAV2	0.06654	-0.090029	[Recognized]
[485]	PRKCZ	<---	EGFR	-0.18501	0.17566	[NOT recognized]
[486]	SRC	<---	EPS8	0.043002	0.070743	[NOT recognized]
[487]	PTPN11	<---	EGFR	-0.50828	0.50924	[NOT recognized]
[488]	MAPK8	--->	JUN	1.0007	-0.99982	[Recognized]
[489]	MAP2K2	<--->	MAPK1	0.4189	0.4189	[Recognized]
[490]	MAPK3	--->	JUND	1.0007	-0.99982	[Recognized]
[491]	JUND	<---	MAPK3	-0.99982	1.0007	[NOT recognized]
[492]	PRKCA	<---	EGFR	-0.11277	0.082104	[NOT recognized]
[493]	PTK2B	<---	PXN	-0.18164	0.084505	[NOT recognized]
[494]	CDK5	--->	PLD2	0.3012	-0.32468	[Recognized]
[495]	AKT1	--->	STAT1	0.047669	-0.095755	[Recognized]
[496]	EGFR	--->	GAB1	0.36019	-0.35169	[Recognized]
[497]	PRKD1	<---	EGFR	-0.33112	0.31857	[NOT recognized]
[498]	AKT1	<---	RAF1	-0.39223	-0.11684	[NOT recognized]
[499]	EGFR	--->	STAT1	0.71328	-0.72403	[Recognized]
[500]	PTPRR	<---	MAPK3	-0.4667	0.45882	[NOT recognized]
[501]	PRKCB	<---	EGFR	-0.11277	0.082104	[NOT recognized]
[502]	MAPK1	--->	JUND	1.0007	-0.99982	[Recognized]
[503]	MAPK3	--->	PTPN5	0.45882	-0.4667	[Recognized]
[504]	RPS6KA5	--->	ATF1	0.78051	-0.87159	[Recognized]
[505]	MAP3K2	<--->	MAP2K5	0.3876	0.3876	[Recognized]
[506]	EGFR	--->	EPS8	0.32237	-0.32415	[Recognized]
[507]	MAP2K2	<--->	MAPK3	0.4189	0.4189	[Recognized]
[508]	SRC	--->	GIT1	0.29392	-0.3501	[Recognized]
[509]	EGFR	<--->	EGFR	0.079135	0.079135	[Recognized]
[510]	PAK1	<---	CTBP1	0.048583	0.068161	[NOT recognized]
[511]	EGFR	--->	CRK	0.99944	-1.0004	[Recognized]
[512]	NEDD4	<---	TNK2	-0.047445	0.046627	[NOT recognized]
[513]	MAPK1	--->	PTPRR	0.45882	-0.4667	[Recognized]
[514]	EGFR	<--->	ERBB3	0.079135	0.079135	[Recognized]
[515]	TNK2	--->	MCF2	0.14468	-0.14465	[Recognized]
[516]	MAPK1	--->	SMAD3	1.0004	-0.99984	[Recognized]
[517]	MAPK1	<---	CASP9	-0.24287	0.1367	[NOT recognized]
[518]	CBLB	<---	EGFR	-0.64709	0.64039	[NOT recognized]
[519]	EGFR	--->	STAT5B	0.99977	-0.9992	[Recognized]
[520]	CBL	<---	EGFR	-0.64709	0.64039	[NOT recognized]
[521]	EGFR	--->	RGS16	0.26243	-0.26084	[Recognized]
[522]	PTPN6	<--->	PTPN6	0.11301	0.11301	[Recognized]
[523]	MAPK8	--->	JUND	1.0007	-0.99982	[Recognized]
[524]	AKT1	--->	FOXO1	0.53688	-0.61874	[Recognized]
[525]	EGFR	--->	RASA1	0.26129	-0.27694	[Recognized]
[526]	PTPN5	<---	MAPK1	-0.4667	0.45882	[NOT recognized]
[527]	RAF1	--->	RFXANK	0.39234	-0.40533	[Recognized]
[528]	MAPK1	--->	STAT3	0.32122	-0.3529	[Recognized]
[529]	PTPN12	<---	JAK2	-0.52985	0.56715	[NOT recognized]
[530]	ARAF	--->	MAP2K1	0.99952	-0.99998	[Recognized]

```
[531]   CAMK2A  <---    EGFR    -0.60942        0.59776 [NOT recognized]
[532]   MAP2K1  <--->   MAPK1   0.4189  0.4189  [Recognized]
[533]   SRC     <---    BCAR1   -0.36108        0.36268 [NOT recognized]
[534]   ADAM17  --->    PTPRF   0.0044982       -0.028263       [Recognized]
[535]   SRC     --->    PLCG1   0.34383 -0.49927        [Recognized]
[536]   MAPK7   --->    PTPRR   0.45882 -0.4667 [Recognized]
[537]   JAK1    <---    SYK     -0.060038       0.67064 [NOT recognized]
[538]   MAPK3   --->    GAB2    0.70629 -0.78567        [Recognized]
[539]   LYN     --->    CSF2RB  0.37119 -0.43179        [Recognized]
[540]   MAP3K14 <--->   MAP3K14 0.4189  0.4189  [Recognized]
[541]   JAK2    <---    STAM    -0.42564        0.48175 [NOT recognized]
[542]   MAP2K2  <--->   MAP2K2  0.4189  0.4189  [Recognized]
[543]   FYN     <---    PIK3R1  -0.10258        0.0079886       [NOT recognized]
[544]   RPS6KB1 --->    RPS6    0.99939 -0.99992        [Recognized]
[545]   IRAK1   --->    TOLLIP  0.56114 -0.58842        [Recognized]
[546]   PRKACA  --->    BCL2    1.1261  -1.1437 [Recognized]
[547]   JAK3    --->    STAT3   0.82423 -0.81159        [Recognized]
[548]   MAP3K7  --->    MAP2K6  1.0003  -1.0004 [Recognized]
[549]   PIK3CG  <---    PTK2B   -0.63083        0.60327 [NOT recognized]
[550]   PRKACA  --->    BAD     1.0006  -1.0345 [Recognized]
[551]   JAK3    --->    STAT5A  0.82423 -0.81159        [Recognized]
[552]   AKT1    <---    GSK3A   0.064251        0.16903 [NOT recognized]
[553]   JAK2    --->    IL6ST   0.4316  -0.43801        [Recognized]
[554]   JAK1    --->    IRS2    0.21545 -0.17861        [Recognized]
[555]   AKT1    --->    BAD     0.56932 -0.67709        [Recognized]
[556]   MAPK3   --->    GATA1   0.72658 -0.73887        [Recognized]
[557]   FES     --->    IRS1    0.44988 -0.37277        [Recognized]
[558]   PTPN6   --->    CSF2RB  0.32189 -0.33046        [Recognized]
[559]   LYN     <---    LYN     -0.32358        -0.32358        [NOT recognized]
[560]   PTPN11  --->    PTK2B   0.014562        -0.23705        [Recognized]
[561]   EP300   --->    STAT3   0.0010271       -0.028951       [Recognized]
[562]   MAPK1   --->    GATA1   0.72658 -0.73887        [Recognized]
[563]   MAPK14  --->    ATF2    1.1569  -1.1226 [Recognized]
[564]   MAPK1   --->    GATA2   0.72658 -0.73887        [Recognized]
[565]   MAP3K7  --->    MAP3K14 1.0003  -1.0004 [Recognized]
[566]   MAPK3   --->    PLCB1   0.72122 -0.72473        [Recognized]
[567]   IRAK1   <--->   IRAK1   0.30117 0.30117 [Recognized]
[568]   IKBKB   --->    RELA    0.61419 -0.83604        [Recognized]
[569]   JAK2    --->    STAT5A  0.27681 -0.56742        [Recognized]
[570]   SRC     --->    STAT3   0.12044 -0.35851        [Recognized]
[571]   PRKCZ   <---    JAK1    -0.011033       0.051345        [NOT recognized]
[572]   MAPK8   --->    BAD     1.0006  -1.0345 [Recognized]
[573]   JAK2    --->    PPP2CA  0.11902 -0.11035        [Recognized]
[574]   JAK3    <---    FES     -0.060038       0.67064 [NOT recognized]
[575]   MAPK3   --->    STAT3   0.32122 -0.3529 [Recognized]
[576]   ERBB2   <--->   ERBB2   0.079135        0.079135        [Recognized]
[577]   PRKCD   --->    STAT3   0.1881  -0.23137        [Recognized]
[578]   MAPK1   --->    PLCB1   0.72122 -0.72473        [Recognized]
[579]   IRAK2   <--->   IRAK2   0.30117 0.30117 [Recognized]
[580]   CDK5    --->    MAPT    1.0005  -1.0004 [Recognized]
[581]   MAPK3   --->    ELK1    1.0002  -1.0006 [Recognized]
[582]   JAK3    --->    IRS2    0.21545 -0.17861        [Recognized]
[583]   PIK3CD  <---    PIK3CD  -0.10115        -0.10115        [NOT recognized]
[584]   MAP3K2  --->    MAP2K4  0.75525 -0.28212        [Recognized]
[585]   MAPK3   --->    BCL2    1.1261  -1.1437 [Recognized]
[586]   JAK1    --->    IRS1    0.21545 -0.17861        [Recognized]
[587]   MAPK1   --->    GAB2    0.70629 -0.78567        [Recognized]
[588]   LYN     --->    MAPK3   0.69943 -0.86291        [Recognized]
[589]   JAK2    --->    STAT5B  0.27681 -0.56742        [Recognized]
[590]   MAPK8   --->    JUN     1.0007  -0.99982        [Recognized]
[591]   AKT1    --->    FOXO3   0.53688 -0.61874        [Recognized]
[592]   PAK1    --->    BAD     0.51029 -0.31169        [Recognized]
[593]   LCK     <---    PIK3R1  -0.10258        0.0079886       [NOT recognized]
[594]   TYK2    <--->   TYK2    0.33559 0.33559 [Recognized]
[595]   LYN     --->    IL5RA   0.52658 -0.60583        [Recognized]
[596]   IRAK4   <--->   IRAK4   0.4189  0.4189  [Recognized]
[597]   PTPN11  --->    GAB2    0.24323 -0.2299 [Recognized]
[598]   PTPN11  --->    STAT5B  0.20575 -0.28198        [Recognized]
[599]   PTK2B   <---    PTK2B   -0.1406 -0.1406 [NOT recognized]
[600]   MAPK11  --->    ATF2    1.1569  -1.1226 [Recognized]
[601]   TRAF6   --->    TAB3    0.44603 -0.45053        [Recognized]
[602]   PIK3CA  <---    STAT3   -0.12023        0.092974        [NOT recognized]
[603]   JAK3    <---    STAM    -0.99998        1       [NOT recognized]
[604]   SRC     <---    SRC     -0.32358        -0.32358        [NOT recognized]
[605]   IL2RG   <---    PIK3R1  -0.22861        0.21478 [NOT recognized]
[606]   JAK1    <---    SOCS3   -0.42994        0.47443 [NOT recognized]
```

[607]	MAPK1	--->	BCL2	1.1261	-1.1437	[Recognized]
[608]	JAK1	--->	BTK	-0.11155	-0.26517	[Recognized]
[609]	LCK	--->	CSF2RB	0.37119	-0.43179	[Recognized]
[610]	PRKACA	<---	RAF1	-0.99998	0.99952	[NOT recognized]
[611]	JAK3	--->	IRS1	0.21545	-0.17861	[Recognized]
[612]	IKBKB	--->	DOK1	0.41633	-0.63246	[Recognized]
[613]	IRAK3	<--->	IRAK3	0.30117	0.30117	[Recognized]
[614]	IRAK1	<--->	IRAK3	0.30117	0.30117	[Recognized]
[615]	MAPK14	--->	ELK1	1.0002	-1.0006	[Recognized]
[616]	PTPN6	--->	JAK1	0.11961	-0.123	[Recognized]
[617]	JAK2	<--->	JAK2	0.35049	0.35049	[Recognized]
[618]	JAK1	--->	SHC1	0.19464	-0.19725	[Recognized]
[619]	MAPK8	--->	ATF2	1.1569	-1.1226	[Recognized]
[620]	JAK1	--->	STAT5B	0.82423	-0.81159	[Recognized]
[621]	HCK	<---	HCK	-0.32358	-0.32358	[NOT recognized]
[622]	PTPN11	--->	STAT5A	0.20575	-0.28198	[Recognized]
[623]	JAK1	--->	INPP5D	0.020083	-0.024408	[Recognized]
[624]	AKT1	<---	GSK3B	0.064251	0.16903	[NOT recognized]
[625]	RAF1	--->	BAD	0.47315	-0.50123	[Recognized]
[626]	JAK1	<---	STAM2	-0.99998	1	[NOT recognized]
[627]	LCK	--->	MAPK1	0.69943	-0.86291	[Recognized]
[628]	TEC	<---	VAV1	-0.038353	-0.0047825	[NOT recognized]
[629]	IRAK1	<--->	IRAK2	0.30117	0.30117	[Recognized]
[630]	NLK	<--->	NLK	0.4189	0.4189	[Recognized]
[631]	JAK1	--->	STAT6	0.82423	-0.81159	[Recognized]
[632]	FES	--->	IRS2	0.44988	-0.37277	[Recognized]
[633]	PTPN6	--->	JAK3	0.11961	-0.123	[Recognized]
[634]	TRAF6	--->	TAB2	0.13453	-0.15101	[Recognized]
[635]	JAK1	--->	STAT5A	0.82423	-0.81159	[Recognized]
[636]	PTPN11	--->	CSF2RB	0.32189	-0.33046	[Recognized]
[637]	BTK	<---	BTK	-0.23569	-0.23569	[NOT recognized]
[638]	MAPK1	--->	STAT3	0.32122	-0.3529	[Recognized]
[639]	JAK1	<---	FES	-0.060038	0.67064	[NOT recognized]
[640]	PIK3CA	<---	RPS6KB1	-0.47591	0.44094	[NOT recognized]
[641]	JAK1	<--->	JAK1	0.33559	0.33559	[Recognized]
[642]	MAP3K2	--->	MAP2K7	0.75525	-0.28212	[Recognized]
[643]	CREBBP	--->	STAT6	0.0010271	-0.028951	[Recognized]
[644]	JAK3	<--->	JAK1	0.33559	0.33559	[Recognized]
[645]	IRAK4	<---	IRAK1	-0.12437	0.64012	[NOT recognized]
[646]	JAK3	<---	SOCS3	-0.42994	0.47443	[NOT recognized]
[647]	JAK3	<--->	JAK3	0.33559	0.33559	[Recognized]
[648]	TRAF6	--->	MAP3K7	1.0005	-1.0007	[Recognized]

[Section 2] Independent test on HPRD-PTM(post-translational modification)

[1]	MAPK1	--->	PPARA	0.99834	-1.0079	[Recognized]
[2]	CAMKK2	--->	CAMK4	0.83082	-0.43719	[Recognized]
[3]	PRKCB	--->	GSK3A	0.22826	0.12473	[Recognized]
[4]	JAK2	--->	STAT3	0.99602	-0.98887	[Recognized]
[5]	GZMB	--->	BID	0.087842	-0.041342	[Recognized]
[6]	MAPK3	<---	MKNK1	0.13867	0.16997	[Unrecognized]
[7]	PIAS1	--->	ZNF76	0.029951	-0.036856	[Recognized]
[8]	CDK1	--->	KHDRBS1	0.35791	-0.37587	[Recognized]
[9]	PRKCA	--->	PTPN11	0.6319	-0.62682	[Recognized]
[10]	ATM	<---	CREB1	-0.054665	0.041638	[Unrecognized]
[11]	CAMK2G	--->	CREM	0.61276	-0.61036	[Recognized]
[12]	PAK1	--->	CASP1	0.58095	-0.43163	[Recognized]
[13]	CREBBP	--->	KLF13	0.071128	-0.081623	[Recognized]
[14]	PRTN3	<---	F2R	-0.42368	0.45462	[Unrecognized]
[15]	CSNK2A2	--->	HNRNPC	0.72994	-0.75755	[Recognized]
[16]	JAK2	--->	CCR2	0.2955	-0.17451	[Recognized]
[17]	ACP1	--->	ZAP70	1.0132	-0.99905	[Recognized]
[18]	ACAN	--->	MMP14	0.022421	-0.030194	[Unrecognized]
[19]	GSK3B	--->	CREB1	0.96654	-0.95401	[Recognized]
[20]	CAMK2G	<---	STAT1	-0.60307	0.59503	[Unrecognized]
[21]	AKT1	--->	CHUK	1.0001	-0.99768	[Recognized]
[22]	ELK1	<---	MAPK1	-0.98814	1.0428	[Recognized]
[23]	EIF2AK2	<---	JAK1	-0.5173	0.50431	[Recognized]
[24]	PRKCD	--->	PTPRA	0.42074	-0.41964	[Recognized]
[25]	MAPK1	<---	SOS1	-0.40733	0.394	[Unrecognized]
[26]	PTPRZ1	--->	GRLF1	0.21289	-0.22013	[Recognized]
[27]	MAPK9	--->	ATF2	1.0089	-1.0006	[Recognized]
[28]	TESK2	--->	DSTN	0.43358	-0.44548	[Recognized]
[29]	IGF1R	--->	CRK	0.098003	-0.11926	[Recognized]
[30]	PRKCA	--->	APLP2	0.12402	-0.13582	[Recognized]
[31]	CHEK2	--->	MDM4	0.052648	-0.070925	[Recognized]
[32]	MMP3	<---	CCL8	-0.16713	0.18806	[Unrecognized]
[33]	MMP2	--->	FGFR1	0.10201	-0.10663	[Recognized]
[34]	PRKACA	<---	RAP1A	-1.0074	1.0132	[Unrecognized]
[35]	MAPK14	--->	PPARGC1A	0.058876	-0.10921	[Recognized]
[36]	CMA1	--->	PFN1	0.095133	-0.062435	[Recognized]
[37]	NRK	--->	CFL1	0.34542	-0.29737	[Recognized]
[38]	MMP7	<---	BCAN	-0.030194	0.022421	[Unrecognized]
[39]	SRC	--->	GTF2I	1.0671	-0.94248	[Recognized]
[40]	MMP14	<---	F12	-0.073981	0.072802	[Unrecognized]
[41]	NTRK2	--->	FRS3	0.20066	-0.2014	[Recognized]
[42]	CSNK2A2	--->	PPP1R8	0.7025	-0.72853	[Recognized]
[43]	MMP2	<---	DCN	-0.18966	0.20515	[Unrecognized]
[44]	MAPK3	--->	STMN2	1.0058	-0.99021	[Recognized]
[45]	ACLY	<---	GSK3B	-0.72114	0.69525	[Recognized]
[46]	MAPK1	--->	ERF	1.1285	-1.1212	[Recognized]
[47]	PPP1CA	<---	PRKCD	-0.16217	0.15344	[Unrecognized]
[48]	SHC1	<---	ZAP70	-0.99981	1.0049	[Unrecognized]
[49]	CDK8	--->	CCNH	1.083	-1.1093	[Recognized]
[50]	KAT2B	--->	KLF13	0.14975	-0.15936	[Recognized]
[51]	SRC	--->	ABL1	0.99536	-1.0073	[Recognized]
[52]	PIK3CA	--->	RPS6KB1	1.0159	-0.98663	[Recognized]
[53]	INSR	<---	PTK2	-0.041494	-0.016988	[Unrecognized]
[54]	AKT1	--->	MAP3K8	1.0168	-1.0071	[Recognized]
[55]	IKBKB	--->	NFKB1	1.001	-1.0129	[Recognized]
[56]	CREBBP	--->	MYOD1	0.19969	-0.20391	[Recognized]
[57]	CSK	--->	RGS16	0.94045	-0.7821	[Recognized]
[58]	NCOA3	<---	CHUK	-0.11492	0.091219	[Recognized]
[59]	PRKCB	--->	GRM5	0.91747	-0.9157	[Recognized]
[60]	CAMK2G	--->	ATF1	0.80987	-0.78916	[Recognized]
[61]	MAPK14	--->	SLC9A1	0.37568	-0.41499	[Recognized]
[62]	MAPK1	--->	FOS	0.53871	-0.68783	[Recognized]
[63]	PRKCA	--->	GRIA1	0.19252	-0.20227	[Recognized]
[64]	CASP3	--->	ATN1	0.053098	-0.077022	[Recognized]
[65]	PTPN1	--->	PDGFRB	0.11574	-0.12276	[Recognized]
[66]	CDK5	--->	ERBB3	0.29741	-0.32251	[Recognized]
[67]	PRKACA	<---	CSK	-1.2776	1.3862	[Unrecognized]
[68]	ABL1	<---	CASP9	-0.41968	0.30417	[Unrecognized]
[69]	SRC	--->	DAPP1	1.2396	-0.79845	[Recognized]
[70]	CSNK1E	--->	APC	0.98885	-0.99835	[Recognized]
[71]	PRKCA	--->	ADRBK1	0.23691	0.014495	[Recognized]
[72]	CDK5	--->	TP53	1.0002	-0.99962	[Recognized]
[73]	CAPN1	--->	CASP14	0.20931	-0.21258	[Recognized]
[74]	CDK2	<--->	CDC7	0.23429	0.23429	[Recognized]

[75]	JAK2	<---	EGFR	-1.0154	1.0001	[Unrecognized]
[76]	MAPK3	<---	ADRBK1	-0.049315	0.28927	[Unrecognized]
[77]	PTPRC	--->	EZR	0.16704	-0.17508	[Recognized]
[78]	SRC	--->	RAF1	0.45157	-0.19021	[Recognized]
[79]	GGCX	--->	PROC	0.1313	-0.11522	[Recognized]
[80]	MMP25	?	MMP2	-0.12951	-0.12951	[Unrecognized]
[81]	SGK1	--->	FOXO3	0.46293	-0.56866	[Recognized]
[82]	MAPK3	--->	STAT3	1.0077	-1.0166	[Recognized]
[83]	FER	--->	JUP	0.048291	-0.071127	[Recognized]
[84]	PRKCD	--->	MUC1	0.29152	-0.29695	[Recognized]
[85]	CDK1	<--->	CSNK2A1	0.23429	0.23429	[Recognized]
[86]	CSNK2A1	--->	MAX	1.5563	-1.5271	[Recognized]
[87]	CTSG	<---	F2R	-0.42368	0.45462	[Unrecognized]
[88]	PRKCA	--->	CFTR	0.19214	-0.20268	[Recognized]
[89]	ROCK1	--->	ARHGAP24	-0.0070945	-0.02987	[Recognized]
[90]	CSNK2A1	<---	PTPN1	-1.009	1.0061	[Unrecognized]
[91]	MAPK12	--->	MAPT	0.60023	-0.67052	[Recognized]
[92]	CRK	--->	PTK2	-0.04842	-0.21492	[Recognized]
[93]	PDGFRB	<---	FYN	-0.058137	0.049038	[Unrecognized]
[94]	CSNK2A1	--->	VDR	0.99834	-1.0079	[Recognized]
[95]	KLK2	--->	SERPINA5	0.11833	-0.082185	[Recognized]
[96]	AKT1	<---	MTOR	-0.99349	0.99887	[Unrecognized]
[97]	FNTA	<---	HRAS	-0.44041	0.47292	[Unrecognized]
[98]	PRKACA	--->	PPP1R9B	0.62013	-0.64226	[Recognized]
[99]	CDK1	--->	ITPR1	0.3288	-0.33886	[Recognized]
[100]	ROCK1	--->	RDX	-0.0022587	-0.012307	[Recognized]
[101]	PTPN6	--->	HOXA10	0.54979	-0.43989	[Recognized]
[102]	MAP2K4	--->	MAPK12	0.64901	-0.41806	[Recognized]
[103]	UBE2I	--->	ARNTL	0.29209	-0.28719	[Recognized]
[104]	PRKCA	--->	HAND1	1.0168	-1.0076	[Recognized]
[105]	LCK	--->	DOK1	0.95892	-0.88457	[Recognized]
[106]	MAPK1	--->	SLC9A1	1.0078	-0.99872	[Recognized]
[107]	LCK	<---	PRKCD	-0.09907	0.095887	[Unrecognized]
[108]	MATK	<--->	LYN	0.37153	0.37153	[Recognized]
[109]	PRKACA	--->	CIITA	0.6377	-0.65892	[Recognized]
[110]	AKT1	--->	AR	0.99897	-1.012	[Recognized]
[111]	CASP6	--->	APP	0.23458	-0.24991	[Recognized]
[112]	MAPK3	--->	SP1	0.12886	-0.14656	[Recognized]
[113]	ZAP70	--->	SH2B3	1.0101	-0.99632	[Recognized]
[114]	GSK3B	--->	PSEN1	0.67463	-0.70045	[Recognized]
[115]	ABL1	<---	PLSCR1	-0.085324	-0.080616	[Unrecognized]
[116]	TLL1	<---	CHRD	-0.16477	0.17437	[Unrecognized]
[117]	RPS6KA1	--->	SRF	0.12564	-0.26444	[Recognized]
[118]	CDK1	--->	ECT2	0.67984	-0.70437	[Recognized]
[119]	CDK5	<---	PTPN2	-1.009	1.0061	[Unrecognized]
[120]	ZAP70	--->	LCP2	1.0092	-1.0144	[Recognized]
[121]	CREBBP	--->	STAT3	0.98672	-1.0013	[Recognized]
[122]	CDK1	--->	STMN2	1.0058	-0.99021	[Recognized]
[123]	CDK2	--->	FZR1	0.83716	-0.86422	[Recognized]
[124]	CASP8	--->	CASP3	1.0138	-1.0003	[Recognized]
[125]	PRKD1	--->	HDAC5	0.43108	-0.46538	[Recognized]
[126]	CSNK2A1	--->	MEF2C	0.43947	-0.44112	[Recognized]
[127]	RET	--->	MAPK1	0.20607	-0.24517	[Recognized]
[128]	CASP3	--->	FYN	1.0113	-1.0017	[Recognized]
[129]	SRC	--->	CTTN	0.75064	-0.61051	[Recognized]
[130]	GSK3B	--->	MCL1	1.2421	-1.2851	[Recognized]
[131]	PRKACA	--->	CACNA1C	0.99121	-1.0053	[Recognized]
[132]	AKT1	--->	BAD	0.99987	-0.9934	[Recognized]
[133]	CSNK2A2	--->	CAV1	0.70376	-0.73589	[Recognized]
[134]	PPM1A	--->	MSN	0.10633	-0.11549	[Recognized]
[135]	CDK1	--->	MAP4	0.9565	-0.97405	[Recognized]
[136]	FYN	--->	SHC1	0.73568	-0.65926	[Recognized]
[137]	HCK	--->	RAPGEF1	0.34923	-0.34388	[Recognized]
[138]	SRC	--->	LRP1	0.077907	-0.08422	[Recognized]
[139]	MAPK10	--->	MAPK8IP3	0.086975	-0.12532	[Recognized]
[140]	CSNK1G1	--->	FOXO1	0.12051	-0.13229	[Recognized]
[141]	MAPK14	--->	ESR1	0.38823	-0.49258	[Recognized]
[142]	GSK3B	--->	AXIN1	0.26825	-0.28011	[Recognized]
[143]	CSNK2A2	--->	CABP1	1.007	-1.0109	[Recognized]
[144]	CSNK2A2	--->	RGS19	0.28697	-0.32153	[Recognized]
[145]	PRKACA	--->	WT1	0.81966	-0.82415	[Recognized]
[146]	CAMK2B	--->	COL4A3	0.045455	-0.059968	[Recognized]
[147]	GRK4	--->	BDKRB2	0.17121	-0.19042	[Recognized]
[148]	CSNK2A2	--->	ACACA	0.41949	-0.42896	[Recognized]
[149]	ACAN	--->	MMP1	0.022421	-0.030194	[Unrecognized]
[150]	PRDM2	--->	HIST3H3	0.1669	-0.15529	[Recognized]

[151]	ADAMTS4	--->	ACAN	0.0043435	-0.015468	[Recognized]
[152]	CASP1	<---	PSEN1	-0.048154	0.056436	[Unrecognized]
[153]	PTK2	--->	BCAR1	-0.01537	-0.15041	[Recognized]
[154]	PRKACA	--->	SPTBN1	0.28219	-0.29697	[Recognized]
[155]	CSNK2A1	--->	RANGAP1	0.22517	-0.25741	[Recognized]
[156]	ADRBK1	--->	FPR1	0.093699	-0.11125	[Recognized]
[157]	PRKCZ	--->	IKBKB	0.25124	-0.25473	[Recognized]
[158]	PRKACA	<---	PTPN7	-1.009	1.0061	[Unrecognized]
[159]	MAPK14	<---	MAPKAPK2	-0.62285	0.64491	[Unrecognized]
[160]	PRKCG	--->	GRIN2B	0.22109	-0.2311	[Recognized]
[161]	CDK2	--->	MYOD1	1.5563	-1.5271	[Recognized]
[162]	PRKCA	--->	EIF4E	0.94696	-0.94609	[Recognized]
[163]	AMFR	<---	UBE2G2	-0.0077172	0.021366	[Unrecognized]
[164]	MAPK3	--->	TAL1	0.95173	-0.93955	[Recognized]
[165]	GSK3B	--->	GYS1	1.0067	-1.0067	[Recognized]
[166]	PRKCD	--->	GNAZ	0.77273	-0.76398	[Recognized]
[167]	PRKCD	--->	PEBP1	0.84277	-0.83157	[Recognized]
[168]	SRC	--->	DAB1	1.0524	-0.90972	[Recognized]
[169]	MAP2K1	--->	CASP9	0.66416	-0.42184	[Recognized]
[170]	GGCX	--->	F10	0.040593	-0.04351	[Recognized]
[171]	PRKAA2	--->	ACACB	0.13992	-0.15543	[Recognized]
[172]	SRC	--->	JUP	0.22916	-0.21486	[Recognized]
[173]	PAK2	--->	RPS6	0.78136	-0.45611	[Recognized]
[174]	GRK6	--->	SLC9A3R1	0.45593	-0.54121	[Recognized]
[175]	CTSD	--->	IGFBP3	0.41796	-0.40802	[Recognized]
[176]	RPS6KB1	<---	STK11	-0.74727	0.78966	[Unrecognized]
[177]	PRKCA	--->	RGS7	0.57282	-0.57611	[Recognized]
[178]	FURIN	<---	EDA	-0.038723	0.029066	[Unrecognized]
[179]	PLK1	<---	CHEK2	-0.00064284	0.1132	[Unrecognized]
[180]	PRKCG	--->	EIF4E	0.94696	-0.94609	[Recognized]
[181]	TXK	--->	CTLA4	0.99605	-0.98986	[Recognized]
[182]	CAMK2G	--->	GRIA4	0.47913	-0.49361	[Recognized]
[183]	PRKCA	--->	RRAD	1.0123	-0.99154	[Recognized]
[184]	CSNK2A2	--->	WAS	0.18552	-0.50928	[Recognized]
[185]	PRKCG	--->	IRS1	0.9708	-0.84897	[Recognized]
[186]	MMP9	<---	PLG	-0.11212	0.11927	[Unrecognized]
[187]	JAK1	--->	STAT3	0.99633	-1.0023	[Recognized]
[188]	LYN	--->	DOK2	1.3209	-1.1853	[Recognized]
[189]	ADAMTS1	--->	ACAN	0.0043435	-0.015468	[Recognized]
[190]	PTPN11	--->	JAK2	0.99157	-1.0118	[Recognized]
[191]	NEK6	--->	SGK1	1.002	-1.0109	[Recognized]
[192]	PTPRJ	--->	PDGFRB	0.076677	-0.086055	[Recognized]
[193]	PRKCA	--->	GRM1	0.91747	-0.9157	[Recognized]
[194]	CSNK1A1	--->	TP53	0.3735	-0.34877	[Recognized]
[195]	RPS6KA5	--->	HIST3H2A	1.009	-0.99586	[Recognized]
[196]	PDPK1	--->	AKT3	0.99822	-0.99934	[Recognized]
[197]	PTPN2	--->	JAK1	0.38802	-0.46748	[Recognized]
[198]	PTPN1	--->	ACTN1	0.46815	-0.47383	[Recognized]
[199]	CASP7	--->	SRF	0.32345	-0.39257	[Recognized]
[200]	PRKACB	--->	NGFR	0.23445	-0.27811	[Recognized]
[201]	LCK	--->	SHC1	0.36949	-0.31772	[Recognized]
[202]	CASP3	--->	PRKDC	0.21872	-0.26808	[Recognized]
[203]	CDK1	--->	EPN1	0.70002	-0.72593	[Recognized]
[204]	CDK1	--->	FOXM1	0.85888	-0.87871	[Recognized]
[205]	SGK3	<---	GSK3A	-0.097142	0.096555	[Unrecognized]
[206]	PRKG1	--->	VASP	0.99265	-0.99079	[Recognized]
[207]	ATM	<---	TP53BP1	-0.22691	0.2168	[Unrecognized]
[208]	LCK	--->	ITK	0.98538	-0.99553	[Recognized]
[209]	CSNK2A2	--->	PPP1R1B	1.0061	-1.0155	[Recognized]
[210]	PTPN6	--->	LYN	0.3856	-0.16887	[Recognized]
[211]	PRKCQ	--->	STK39	0.9999	-0.99823	[Recognized]
[212]	PDPK1	--->	PRKCD	0.24482	-0.071276	[Recognized]
[213]	HPN	<---	F7	-0.0064733	-0.0028364	[Unrecognized]
[214]	AKT1	--->	CHEK1	1.0168	-1.0071	[Recognized]
[215]	SRC	--->	PTPRA	0.16479	-0.2369	[Recognized]
[216]	PPP1CA	--->	H2AFX	0.25877	-0.26197	[Recognized]
[217]	CASP7	--->	RB1	0.41973	-0.45426	[Recognized]
[218]	CHEK1	--->	TP53	1.0002	-0.99962	[Recognized]
[219]	PRKG1	--->	SF1	0.65757	-0.65853	[Recognized]
[220]	CSNK2A1	--->	HDAC1	0.4	-0.42066	[Recognized]
[221]	GZMA	<---	HMGB2	-0.17017	0.20338	[Unrecognized]
[222]	RPS6KA4	--->	CREB1	1.0082	-0.98976	[Recognized]
[223]	PRKG1	--->	ITPR1	0.11859	-0.13105	[Recognized]
[224]	PDPK1	<---	PAK1	-0.20725	0.22288	[Unrecognized]
[225]	SERPINI1	<---	PLG	-0.040728	0.057207	[Recognized]
[226]	PRKACA	<---	RAP1GAP	-0.21348	0.19914	[Unrecognized]

[227]	AKT1	--->	ITGB3	0.10202	-0.079994	[Recognized]
[228]	CDK5	<---	MAP2K1	-0.99672	1.0159	[Unrecognized]
[229]	ATM	<---	WRN	-0.14022	0.12861	[Unrecognized]
[230]	MAPK9	--->	ELK1	1.0428	-0.98814	[Recognized]
[231]	PRKCE	--->	SLC4A3	0.61534	-0.68295	[Recognized]
[232]	PRKAA1	--->	PFKFB2	0.086531	-0.28854	[Recognized]
[233]	CAMK4	--->	NOS1	0.48839	-0.49502	[Recognized]
[234]	ATM	<---	ABL1	-0.025238	0.011033	[Unrecognized]
[235]	STK11	--->	MARK1	0.045757	-0.0047098	[Recognized]
[236]	PPP2CB	--->	MAPT	0.29647	-0.30306	[Recognized]
[237]	CDK1	<--->	BUB1	0.23429	0.23429	[Recognized]
[238]	CDK2	--->	TP53	1.0002	-0.99962	[Recognized]
[239]	PRKDC	--->	JUN	0.16277	-0.18797	[Recognized]
[240]	MMP3	<---	HAPLN1	-0.093464	0.097177	[Unrecognized]
[241]	FYN	--->	CDK5	1.3862	-1.2776	[Recognized]
[242]	MAPK1	--->	UBTF	0.92033	-0.89929	[Recognized]
[243]	FURIN	--->	NOTCH1	0.99505	-1.0035	[Recognized]
[244]	ADRBK2	--->	OPRM1	0.25048	-0.27132	[Recognized]
[245]	PRKCB	--->	EPB41	0.33349	-0.34166	[Recognized]
[246]	MTOR	--->	RPS6KB2	1.0159	-1.0002	[Recognized]
[247]	CSNK2A2	--->	HDAC1	0.4	-0.42066	[Recognized]
[248]	MMP1	<---	CCL2	-0.16713	0.18806	[Unrecognized]
[249]	PRKG1	--->	CFTR	0.47148	-0.4767	[Recognized]
[250]	INSR	--->	SHC1	0.10914	-0.14379	[Recognized]
[251]	MAP2K3	--->	MAPK12	0.64901	-0.41806	[Recognized]
[252]	ABL1	<---	JAK2	-0.028405	0.1162	[Unrecognized]
[253]	MAPK1	--->	NR5A1	0.99834	-1.0079	[Recognized]
[254]	MMP14	<---	CCL7	-0.16713	0.18806	[Unrecognized]
[255]	PTPN2	--->	STAT1	-0.010277	-0.080675	[Recognized]
[256]	CASP3	--->	KCNIP3	0.20167	-0.27393	[Recognized]
[257]	CAPN1	--->	TP53	0.17223	-0.17927	[Recognized]
[258]	PRKACA	<---	RRAD	-1.0074	1.0132	[Unrecognized]
[259]	AKT1	--->	S1PR1	0.25532	-0.17208	[Recognized]
[260]	TGFBR1	--->	SMAD2	1.0115	-1.0087	[Recognized]
[261]	CDK1	--->	E2F1	0.44723	-0.46975	[Recognized]
[262]	AKT1	--->	HTT	0.45296	-0.3061	[Recognized]
[263]	CSNK2A1	--->	NOL3	1.0341	-1.0599	[Recognized]
[264]	PDPK1	--->	PRKCE	0.86338	-0.85829	[Recognized]
[265]	MMP13	<---	BCAN	-0.030194	0.022421	[Unrecognized]
[266]	CSNK2A1	--->	GTF2A1	0.69606	-0.7619	[Recognized]
[267]	IGFBP3	<---	F2	-0.013006	0.028162	[Recognized]
[268]	PAK1	--->	MAP2K1	1.0033	-1.0022	[Recognized]
[269]	CSNK2A1	<---	HSP90AA1	-0.056762	0.040193	[Unrecognized]
[270]	FES	--->	BCR	0.16974	-0.14841	[Recognized]
[271]	PRKDC	<---	SRF	-0.026612	0.025906	[Unrecognized]
[272]	PRKCB	--->	ITGB2	0.054379	-0.07038	[Recognized]
[273]	EGFR	--->	STAM2	0.095034	-0.090392	[Recognized]
[274]	PRKCQ	--->	ICAM3	0.15325	-0.15312	[Recognized]
[275]	CDK2	--->	BRCA1	0.32242	-0.34057	[Recognized]
[276]	PRKCZ	<---	WWC1	-0.03202	0.018488	[Unrecognized]
[277]	MAPK8	--->	CDKN1A	0.51838	-0.55156	[Recognized]
[278]	SMYD2	--->	TP53	0.39751	-0.38538	[Recognized]
[279]	FURIN	--->	SORL1	0.021414	-0.032828	[Recognized]
[280]	MTOR	--->	EIF4EBP1	1.0129	-0.9999	[Recognized]
[281]	CSNK2A1	--->	MAZ	0.70307	-0.63523	[Recognized]
[282]	GZMB	<---	PTK2	-0.12359	0.01814	[Unrecognized]
[283]	PRKAA1	--->	MTOR	0.99912	-0.99491	[Recognized]
[284]	PPM1A	--->	PIK3R1	0.20182	-0.27016	[Recognized]
[285]	MAPK3	--->	SPIB	1.1285	-1.1212	[Recognized]
[286]	PDP1	--->	PDHA1	0.52403	-0.50713	[Recognized]
[287]	PCSK4	<---	IGF2	-0.019533	0.034667	[Unrecognized]
[288]	BTK	--->	PLCG2	1.0063	-1.0027	[Recognized]
[289]	PRKACA	--->	HNRNPD	0.72994	-0.75755	[Recognized]
[290]	CTSD	--->	CCL21	0.4106	-0.40051	[Recognized]
[291]	ACAN	<---	ADAMTS5	-0.015468	0.0043435	[Recognized]
[292]	PTPRE	<---	SRC	-0.37818	0.26821	[Unrecognized]
[293]	PRKCD	--->	EGFR	0.40309	-0.40693	[Recognized]
[294]	FYN	--->	JUP	0.251	-0.22621	[Recognized]
[295]	PTPN1	--->	ESR1	0.83604	-0.82697	[Recognized]
[296]	MAPK1	<---	GAB1	-0.19052	0.17127	[Unrecognized]
[297]	SRC	--->	CTNND1	0.44243	-0.4172	[Recognized]
[298]	PLG	--->	MMP3	0.11927	-0.11212	[Unrecognized]
[299]	GGCX	--->	F7	0.054476	-0.050377	[Recognized]
[300]	PLG	<---	F2RL1	-0.27153	0.28486	[Unrecognized]
[301]	CAMK2A	--->	EGFR	1.0058	-1.0028	[Recognized]
[302]	STK25	--->	YWHAZ	0.62776	-0.66368	[Recognized]

[303]	RPS6KA1	--->	ETV1	0.39908	-0.61069	[Recognized]
[304]	NR4A1	<---	RPS6KA5	-0.73191	0.56871	[Recognized]
[305]	EGFR	--->	HGS	0.055598	-0.077423	[Recognized]
[306]	AKT1	--->	EP300	0.048622	-0.055988	[Recognized]
[307]	PRKACA	<---	RHOA	-1.0074	1.0132	[Unrecognized]
[308]	CSNK2A2	--->	MYC	1.0154	-1.0065	[Recognized]
[309]	MAPK14	--->	ZFYVE20	0.29074	-0.46077	[Recognized]
[310]	MMP2	<---	BCAN	-0.030194	0.022421	[Unrecognized]
[311]	CDK5	--->	MEF2A	0.35609	-0.36411	[Recognized]
[312]	MAPK11	--->	MAPKAPK5	0.73174	-0.53091	[Recognized]
[313]	MAPK1	--->	EIF4EBP1	1.0012	-1.0031	[Recognized]
[314]	PRKCG	--->	GSK3A	0.22826	0.12473	[Recognized]
[315]	PRKCD	--->	EP300	0.032186	-0.044787	[Recognized]
[316]	GZMB	--->	CASP9	0.039556	-0.031345	[Recognized]
[317]	PTEN	<---	STK11	-0.79908	0.84207	[Recognized]
[318]	STK11	--->	SNRK	0.6413	-0.21779	[Recognized]
[319]	MMP2	<---	PZP	-0.11161	0.132	[Unrecognized]
[320]	BTK	--->	DAPP1	1.0024	-1.001	[Recognized]
[321]	RHEB	--->	BRAF	1.0064	-0.99496	[Recognized]
[322]	CSNK2A1	--->	F11R	0.090263	-0.12115	[Recognized]
[323]	PRMT1	--->	FGF2	0.29521	-0.29296	[Recognized]
[324]	CSK	<--->	HCK	0.37153	0.37153	[Recognized]
[325]	RET	--->	MAPK14	0.058583	-0.16526	[Recognized]
[326]	CTSE	--->	A2M	0.46612	-0.45657	[Recognized]
[327]	CAMK2A	--->	SMAD2	1.0038	-1.0107	[Recognized]
[328]	PRKACA	<--->	AURKA	0.23429	0.23429	[Recognized]
[329]	PRMT1	--->	HNRNPA1	0.28801	-0.28644	[Recognized]
[330]	MKNK2	--->	EIF4E	1.1369	-1.1717	[Recognized]
[331]	AKT1	--->	FOXO3	0.99774	-1.0046	[Recognized]
[332]	PRKACA	--->	RGS10	0.28697	-0.32153	[Recognized]
[333]	AKT1	--->	GAB2	0.062026	-0.15811	[Recognized]
[334]	ATR	--->	TP53	0.074664	-0.082792	[Recognized]
[335]	RPS6KA5	--->	PLA2G4A	0.24128	-0.35828	[Recognized]
[336]	PRKCG	--->	OCLN	0.94019	-0.93859	[Recognized]
[337]	CDK2	--->	ID3	1.5563	-1.5271	[Recognized]
[338]	PTPN11	--->	IL6ST	0.41197	-0.42337	[Recognized]
[339]	PRKACA	<--->	GSK3B	0.23429	0.23429	[Recognized]
[340]	ACAN	--->	MMP2	0.022421	-0.030194	[Unrecognized]
[341]	PRKCQ	--->	GRM5	0.68403	-0.63679	[Recognized]
[342]	NR4A1	<---	RPS6KA1	-0.73191	0.56871	[Recognized]
[343]	CSK	--->	LCK	0.99631	-0.99108	[Recognized]
[344]	PRKACA	--->	CCND1	1.0544	-1.0774	[Recognized]
[345]	ADAM17	<---	TNF	-0.11106	0.11277	[Unrecognized]
[346]	MAPK1	--->	NCOA1	0.30243	-0.31801	[Recognized]
[347]	MTOR	--->	RPS6KB1	1.0159	-1.0002	[Recognized]
[348]	FYN	--->	GRIN2B	0.27342	-0.24765	[Recognized]
[349]	CASP3	--->	BRCA1	0.34307	-0.41552	[Recognized]
[350]	CASP6	<--->	CASP3	0.35273	0.35273	[Recognized]
[351]	LCK	--->	DEF6	0.71858	-0.52901	[Recognized]
[352]	EGFR	--->	RGS16	0.99759	-1.0133	[Recognized]
[353]	EGFR	--->	CALM2	0.34959	-0.34973	[Recognized]
[354]	CCNB1	<---	PBK	-1.0774	1.0544	[Unrecognized]
[355]	TESK2	--->	CFL1	0.43358	-0.44548	[Recognized]
[356]	SRC	--->	PRKD1	0.11747	-0.11367	[Recognized]
[357]	ATM	<---	EIF4EBP1	-0.22623	0.21616	[Unrecognized]
[358]	ROCK1	<---	LIMK1	-0.053928	0.039587	[Unrecognized]
[359]	AKT2	--->	CHUK	1.0001	-0.99768	[Recognized]
[360]	ELANE	<---	F2RL1	-0.42368	0.45462	[Unrecognized]
[361]	ATR	--->	E2F1	-0.0011743	-0.0063021	[Recognized]
[362]	PRKCB	--->	OCLN	0.94019	-0.93859	[Recognized]
[363]	MAPK14	--->	DDIT3	1.0113	-0.99712	[Recognized]
[364]	SETD7	--->	HIST2H3C	0.43678	-0.40934	[Recognized]
[365]	ITK	<---	BMX	-0.20499	-0.055822	[Unrecognized]
[366]	EP300	--->	KPNA2	0.18327	-0.18765	[Recognized]
[367]	DOK1	<---	RET	-0.50436	0.48844	[Recognized]
[368]	CASP7	<---	NEDD4	-0.072983	0.051513	[Unrecognized]
[369]	VRK1	--->	TP53	0.64996	-0.64026	[Recognized]
[370]	CDK2	--->	CCNE1	1.0544	-1.0774	[Recognized]
[371]	MAPK7	--->	MEF2D	0.08921	-0.12769	[Recognized]
[372]	MAPK9	--->	BCL2L11	0.43567	-0.45207	[Recognized]
[373]	CDK5	--->	WASF1	0.5158	-0.53847	[Recognized]
[374]	GSK3B	--->	FOXO1	0.32546	-0.33505	[Recognized]
[375]	CASP3	--->	APP	0.23458	-0.24991	[Recognized]
[376]	CASP1	--->	BCL2L1	0.43666	-0.44173	[Recognized]
[377]	PRKCA	--->	PEA15	0.92276	-0.91829	[Recognized]
[378]	CDK2	--->	RB1	0.98937	-1.0015	[Recognized]

[379]	CDK1	<---	RAP1GAP	-0.21348	0.19914	[Unrecognized]
[380]	PRKAA1	--->	GRIK2	0.082786	-0.28167	[Recognized]
[381]	CASP3	<---	AKT1	-0.21376	0.25651	[Unrecognized]
[382]	PIM1	--->	BAD	1.2302	-1.4217	[Recognized]
[383]	PRKCA	--->	RAF1	0.99386	-0.99255	[Recognized]
[384]	PRKCD	--->	ADRA2A	0.46078	-0.46051	[Recognized]
[385]	CASP3	--->	DSG3	0.41171	-0.48435	[Recognized]
[386]	PPP2CA	--->	PRKCD	0.070905	-0.079371	[Recognized]
[387]	EIF2AK2	--->	NFKBIA	1.454	-1.5082	[Recognized]
[388]	AKT1	--->	PTEN	0.35215	-0.26364	[Recognized]
[389]	LYN	--->	BTK	1.0008	-0.98497	[Recognized]
[390]	TP53	<---	CDC25C	-0.18195	0.18798	[Recognized]
[391]	MAPK14	<---	PTPN7	-0.33377	0.17221	[Unrecognized]
[392]	BMP1	<---	CHRD	-0.12129	0.10766	[Unrecognized]
[393]	BLK	--->	FCGR2B	0.11748	0.015127	[Recognized]
[394]	F2	<---	F2RL1	-0.27153	0.28486	[Unrecognized]
[395]	IGF1R	--->	IRS1	0.98642	-1.0008	[Recognized]
[396]	MMP3	<---	SERPINE1	-0.13073	0.15283	[Unrecognized]
[397]	PTPN6	--->	GAB2	0.99997	-1.0165	[Recognized]
[398]	FURIN	--->	ZP3	0.041432	-0.037698	[Recognized]
[399]	SRC	--->	CAV1	0.99937	-1.0053	[Recognized]
[400]	AKT1	--->	FOXO1	1.0157	-0.99901	[Recognized]
[401]	AKT1	--->	NOS3	0.99914	-1.0011	[Recognized]
[402]	MAPK14	--->	RPS6KA4	1.0109	-0.99775	[Recognized]
[403]	BCR	--->	YWHAQ	0.078735	-0.083313	[Recognized]
[404]	CASP4	<---	CASP3	0.10027	0.17851	[Unrecognized]
[405]	TFPI	--->	MMP8	0.20851	-0.1877	[Unrecognized]
[406]	INSR	--->	DOK1	0.35356	-0.35555	[Recognized]
[407]	RPS6KA5	--->	EIF4EBP1	0.35226	-0.56724	[Recognized]
[408]	CDK7	--->	LASP1	0.16334	-0.17365	[Recognized]
[409]	CCNB1	<---	MEF2C	-0.01407	0.0070079	[Unrecognized]
[410]	MAPK14	--->	MEF2C	0.99836	-0.99617	[Recognized]
[411]	APC	<---	GSK3B	-0.15381	0.13984	[Recognized]
[412]	CCNE1	<---	CABLES1	-0.65097	-0.11778	[Unrecognized]
[413]	NFKBIB	<---	CHUK	-1.0011	1.0006	[Recognized]
[414]	ATM	<---	CHEK2	-0.1418	0.13005	[Unrecognized]
[415]	PYY	--->	MEP1B	0.31554	-0.27935	[Unrecognized]
[416]	MAPKAPK3	--->	HSPB1	1.0388	-0.99926	[Recognized]
[417]	ABL2	--->	CAT	-0.040953	-0.055538	[Recognized]
[418]	CASP7	--->	MAPT	0.50832	-0.57833	[Recognized]
[419]	PRKACA	--->	VASP	0.5801	-0.59926	[Recognized]
[420]	CDK1	<---	ABL1	-0.14811	-0.013323	[Unrecognized]
[421]	PLK1	--->	NUDC	0.29017	-0.31041	[Recognized]
[422]	CASP3	--->	HNRNPU	0.59134	-0.70233	[Recognized]
[423]	MAPKAPK2	--->	HSPB1	0.73124	-0.7127	[Recognized]
[424]	LIMK1	--->	CFL1	0.16176	-0.17545	[Recognized]
[425]	PPP2CA	--->	RHO	0.25904	-0.26619	[Recognized]
[426]	PRKACA	--->	STMN2	1.0058	-0.99021	[Recognized]
[427]	PRKCA	--->	VCL	0.86863	-0.86787	[Recognized]
[428]	PPP2CA	--->	RACGAP1	0.15866	-0.16819	[Recognized]
[429]	CAPN1	--->	IL1A	0.13209	-0.13754	[Recognized]
[430]	MELK	--->	CDC25B	0.41934	-0.41468	[Recognized]
[431]	CDK1	--->	TP73	0.45486	-0.4556	[Recognized]
[432]	FYN	--->	CD79B	1.0799	-1.0399	[Recognized]
[433]	JAK2	--->	STAT5A	0.99488	-1.0064	[Recognized]
[434]	PIM1	--->	CDC25A	0.69298	-0.67632	[Recognized]
[435]	CSNK2A2	--->	KIF1C	0.42625	-0.44778	[Recognized]
[436]	CDK2	--->	HIRA	0.83716	-0.86422	[Recognized]
[437]	HCK	--->	CSF3R	0.64184	-0.56719	[Recognized]
[438]	AKT1	--->	DLC1	0.25476	-0.16872	[Recognized]
[439]	MAPK1	--->	MYB	0.092606	-0.10814	[Recognized]
[440]	SENP3	<---	EP300	-0.11677	0.10885	[Unrecognized]
[441]	PPP1CA	--->	CAD	0.12592	-0.13568	[Recognized]
[442]	MMP3	<---	IGFBP3	-0.15977	0.18055	[Unrecognized]
[443]	EGFR	--->	EPS15	0.99691	-0.9942	[Recognized]
[444]	ITK	?	BTK	-0.2242	-0.2242	[Unrecognized]
[445]	CSNK1A1	--->	HNRNPC	0.58079	-0.50147	[Recognized]
[446]	MMP7	<---	DCN	-0.18966	0.20515	[Unrecognized]
[447]	EGFR	--->	GAB1	0.9971	-0.98971	[Recognized]
[448]	CSNK2A2	--->	CREM	0.96654	-0.95401	[Recognized]
[449]	FGFR4	--->	STAT3	0.13994	-0.19056	[Recognized]
[450]	HIPK2	--->	TP53	1.0002	-0.99962	[Recognized]
[451]	EIF2AK2	--->	TP53	1.0002	-0.99962	[Recognized]
[452]	AKT1	--->	SH2B2	0.092343	-0.068454	[Recognized]
[453]	PIM3	--->	BAD	0.75379	-0.87574	[Recognized]
[454]	MAPK14	--->	ELK3	1.0099	-1.0011	[Recognized]

[455]	PRKCA	--->	ITGB2	0.054379	-0.07038	[Recognized]
[456]	HCK	--->	STAT3	0.99917	-0.99055	[Recognized]
[457]	CMA1	--->	MMP1	0.21298	-0.1922	[Recognized]
[458]	MAP2K7	<--->	MAPK9	0.23429	0.23429	[Recognized]
[459]	AKT1	--->	PEA15	0.73681	-0.61247	[Recognized]
[460]	PRKCA	--->	GMFB	0.91579	-0.9142	[Recognized]
[461]	CSNK2A1	--->	IRS1	0.5492	-0.60947	[Recognized]
[462]	CAPN2	--->	F2RL1	0.075727	-0.071443	[Recognized]
[463]	CSNK2A1	--->	HES6	0.97225	-0.95979	[Recognized]
[464]	F10	--->	PROS1	-0.007533	-0.0076005	[Recognized]
[465]	ADAM9	<---	TNF	-0.13274	0.1341	[Unrecognized]
[466]	MAPK8	--->	PXN	0.57323	-0.59278	[Recognized]
[467]	CASP3	--->	HTT	0.60022	-0.71121	[Recognized]
[468]	BACE2	--->	APP	0.36802	-0.37802	[Recognized]
[469]	PRKACA	--->	PLN	1.0045	-0.98691	[Recognized]
[470]	PTPN11	--->	FRS2	0.90858	-0.72183	[Recognized]
[471]	PLAU	?	PLG	-0.010603	-0.010603	[Unrecognized]
[472]	PRKCZ	<---	PRKCA	0.00070791	0.10014	[Unrecognized]
[473]	PTK2	<---	BMX	-0.16631	0.071074	[Unrecognized]
[474]	CASP7	--->	PSEN2	0.58173	-0.69269	[Recognized]
[475]	AKT1	--->	BRCA1	0.2728	-0.18845	[Recognized]
[476]	CTSE	--->	INS	0.35706	-0.34709	[Recognized]
[477]	UBE2I	--->	SP3	0.32873	-0.31052	[Recognized]
[478]	TAOK2	--->	ELK1	1.0428	-0.98814	[Recognized]
[479]	BMP1	<---	HSPG2	-0.03602	0.023365	[Unrecognized]
[480]	CSNK2A1	<---	RRAD	-1.0074	1.0132	[Unrecognized]
[481]	CAPN1	--->	F2R	0.16203	-0.16251	[Recognized]
[482]	RPS6KA3	--->	ESR1	0.56871	-0.73191	[Recognized]
[483]	PRMT3	--->	PABPN1	0.18339	-0.17035	[Recognized]
[484]	CSNK2A1	--->	HMOX2	0.70065	-0.72661	[Recognized]
[485]	MARK1	--->	MAP2	0.059939	-0.0736	[Recognized]
[486]	ATM	<---	MDM4	-0.056244	0.043154	[Unrecognized]
[487]	DMPK	--->	PPP1R12A	0.35178	-0.366	[Recognized]
[488]	IKBKB	<---	PRKCQ	-0.40526	0.42813	[Recognized]
[489]	PRKCB	--->	CYTH2	1.2556	-1.0819	[Recognized]
[490]	JAK2	--->	EPOR	0.27406	-0.20849	[Recognized]
[491]	PRKACA	--->	GP1BB	0.63909	-0.66131	[Recognized]
[492]	CSNK2A1	--->	EGR1	0.91941	-0.80386	[Recognized]
[493]	CASP3	--->	SRF	0.32345	-0.39257	[Recognized]
[494]	CSNK2A1	--->	PPP1R1B	1.0061	-1.0155	[Recognized]
[495]	GALNT12	--->	MUC1	0.17779	-0.17757	[Recognized]
[496]	BRSK2	<---	WEE1	0.13714	0.14515	[Unrecognized]
[497]	PTPN5	--->	FYN	-0.065476	-0.14195	[Recognized]
[498]	MAPKAPK5	--->	PLA2G4A	0.45873	-0.58026	[Recognized]
[499]	PRKCZ	<---	PRKCD	-0.0071611	0.068471	[Unrecognized]
[500]	CSNK2A1	--->	CREM	0.96654	-0.95401	[Recognized]
[501]	CSNK2A1	--->	ARNT	0.58841	-0.58643	[Recognized]
[502]	CTSD	--->	INS	0.35706	-0.34709	[Recognized]
[503]	LRRC7	<---	CAMK2A	-0.05314	0.04106	[Recognized]
[504]	BTK	--->	GTF2I	1.0094	-0.99988	[Recognized]
[505]	AKT1	--->	YAP1	0.27513	-0.18807	[Recognized]
[506]	CDK7	--->	E2F1	0.44723	-0.46975	[Recognized]
[507]	MAPK1	--->	TNFRSF1A	0.23445	-0.27811	[Recognized]
[508]	EGFR	--->	RASA1	1.0038	-0.99351	[Recognized]
[509]	PTPN6	--->	ESR1	0.52288	-0.40649	[Recognized]
[510]	CASP4	<---	IL1B	-0.084506	0.083786	[Unrecognized]
[511]	GRK6	--->	BDKRB2	0.13522	-0.19362	[Recognized]
[512]	PRKCA	--->	RHO	0.53258	-0.53729	[Recognized]
[513]	JAK3	--->	JAK1	0.99665	-1.0003	[Recognized]
[514]	MAPK3	<---	PTPN7	-1.009	1.0061	[Unrecognized]
[515]	FER	--->	CTTN	0.19937	-0.20369	[Recognized]
[516]	MMP9	<---	PZP	-0.11161	0.132	[Unrecognized]
[517]	UBE2E3	--->	NFKBIA	0.99947	-0.99915	[Recognized]
[518]	LYN	<---	MAP4K1	0.0088646	0.018188	[Unrecognized]
[519]	SFRS2IP	--->	PSEN1	0.10047	-0.086186	[Recognized]
[520]	MMP11	<---	SERPINA1	0.012498	0.025482	[Unrecognized]
[521]	PRMT1	--->	KHDRBS1	0.13822	-0.146	[Recognized]
[522]	PRKACA	<--->	CDK16	0.23429	0.23429	[Recognized]
[523]	RPS6KA3	--->	HIST3H3	1.009	-0.99586	[Recognized]
[524]	CSK	--->	MAPK15	1.3862	-1.2776	[Recognized]
[525]	PTPRR	--->	MAPK7	0.020191	-0.10294	[Recognized]
[526]	EP300	--->	TP53	0.073526	-0.083705	[Recognized]
[527]	GSK3A	--->	SGK1	0.15763	-0.14926	[Unrecognized]
[528]	F10	<---	GAD2	-0.015975	0.012987	[Unrecognized]
[529]	PRKACA	--->	RYR1	0.21832	-0.22731	[Recognized]
[530]	CDK1	--->	CREM	0.96654	-0.95401	[Recognized]

[531]	CDK1	--->	STMN1	1.0058	-0.99021	[Recognized]
[532]	EP300	--->	ETV1	0.18812	-0.1922	[Recognized]
[533]	AKT1	<---	RAC1	0.060359	0.09142	[Unrecognized]
[534]	MAPK1	--->	PPARG	0.60373	-0.61504	[Recognized]
[535]	CASP3	--->	BCL2	0.81871	-0.89609	[Recognized]
[536]	PCSK1	<---	REN	-0.22971	0.23025	[Unrecognized]
[537]	KLKB1	--->	HGF	0.0012258	-0.0091243	[Recognized]
[538]	AKT1	--->	CASP9	1.0121	-1.0107	[Recognized]
[539]	CASP8	--->	BID	1.0049	-1	[Recognized]
[540]	PAK3	--->	MYO6	0.36482	-0.20271	[Recognized]
[541]	SRC	--->	NFKBIA	1.0084	-1.0021	[Recognized]
[542]	PRKCA	--->	TRPC3	0.044618	-0.057753	[Recognized]
[543]	NFKB1	<---	CHUK	-1.0129	1.001	[Recognized]
[544]	RPS6KB1	--->	EIF4B	1.0004	-1.0123	[Recognized]
[545]	MAPK8	--->	BCL2L11	0.43567	-0.45207	[Recognized]
[546]	TGFBR2	--->	TGFBR1	0.99398	-1.0032	[Recognized]
[547]	AKT1	--->	GATA1	0.49316	-0.34165	[Recognized]
[548]	MAPK8	--->	NR3C1	0.99834	-1.0079	[Recognized]
[549]	FURIN	<---	PDGFA	-0.012903	0.0097438	[Recognized]
[550]	MAPK1	<---	DUSP1	-1.0004	1.0016	[Unrecognized]
[551]	GSK3B	--->	CCNE1	1.0544	-1.0774	[Recognized]
[552]	PLK3	--->	TP53	0.55158	-0.55633	[Recognized]
[553]	AURKA	--->	MBD3	0.91183	-0.93004	[Recognized]
[554]	GZMB	--->	PRKDC	0.0066816	-0.0025076	[Recognized]
[555]	MAPK3	--->	ETS1	1.0002	-0.99981	[Recognized]
[556]	CASP8	--->	MAPT	0.43362	-0.52325	[Recognized]
[557]	CAMK4	--->	CREBBP	0.32272	-0.32425	[Recognized]
[558]	ADRBK2	--->	BDKRB2	0.20207	-0.22265	[Recognized]
[559]	CASP7	--->	EIF2AK2	0.87492	-0.98085	[Recognized]
[560]	SRC	--->	DNM1	0.3136	-0.28153	[Recognized]
[561]	FURIN	--->	DSG3	0.030103	-0.033247	[Recognized]
[562]	PPP3CA	--->	BAD	0.22491	-0.23594	[Recognized]
[563]	CSNK2A1	--->	SPTBN1	0.28219	-0.29697	[Recognized]
[564]	PRKCD	--->	MAPT	0.50001	-0.49874	[Recognized]
[565]	CSNK2A1	--->	ATF1	1.2086	-1.1667	[Recognized]
[566]	MMP14	?	MMP2	-0.12951	-0.12951	[Unrecognized]
[567]	PAK1	--->	ESR1	0.73388	-0.56277	[Recognized]
[568]	PDK1	<---	RPS6KB1	-0.15143	0.0090483	[Unrecognized]
[569]	PRKCA	--->	HES1	0.14857	-0.15922	[Recognized]
[570]	PRKCD	--->	STAT1	0.55428	-0.55913	[Recognized]
[571]	PRKCB	--->	TOP2A	0.89006	-0.88857	[Recognized]
[572]	MMP14	?	MMP13	-0.12951	-0.12951	[Unrecognized]
[573]	PTPRC	--->	FYN	1.007	-0.99313	[Recognized]
[574]	CASP9	<---	BIRC8	-0.036843	0.035713	[Recognized]
[575]	UBE2I	--->	ARNT	0.29209	-0.28719	[Recognized]
[576]	SRC	--->	PDPK1	0.50791	-0.45358	[Recognized]
[577]	PLG	<---	MEP1A	-0.036571	0.041491	[Unrecognized]
[578]	MAPK14	--->	PLA2G4A	0.31934	-0.41482	[Recognized]
[579]	LYN	--->	RGS16	0.94045	-0.7821	[Recognized]
[580]	CSNK2A1	--->	HOXB7	0.79979	-0.82765	[Recognized]
[581]	SRC	--->	AFAP1	1.2771	-1.0835	[Recognized]
[582]	MAPK3	--->	GAB2	0.99738	-0.99704	[Recognized]
[583]	FYN	<---	CBL	-0.7389	0.74409	[Unrecognized]
[584]	ADAM17	--->	TGFA	0.024962	-0.020542	[Recognized]
[585]	CASP3	--->	CDC42	0.36319	-0.47633	[Recognized]
[586]	CDC42BPA	--->	PPP1R12C	0.30518	-0.31765	[Recognized]
[587]	SRC	--->	IGF1R	0.12772	-0.061466	[Recognized]
[588]	PRKG1	--->	CRIP2	1.0027	-0.98912	[Recognized]
[589]	PRKACA	--->	RASGRF1	0.99772	-0.99833	[Recognized]
[590]	EGFR	--->	EZR	0.10938	-0.11225	[Recognized]
[591]	PRKCA	--->	DLX3	0.91929	-0.91785	[Recognized]
[592]	PLK1	--->	CCNB1	0.55049	-0.56928	[Recognized]
[593]	MAPK3	--->	PPARA	0.99834	-1.0079	[Recognized]
[594]	CASP3	--->	BLM	0.35933	-0.43201	[Recognized]
[595]	CSNK1D	--->	PER2	0.72042	-0.58384	[Recognized]
[596]	SRC	--->	CAV2	0.99937	-1.0053	[Recognized]
[597]	SRC	--->	ESR1	0.61826	-0.55261	[Recognized]
[598]	PKN1	--->	CDC25C	0.0050881	-0.076912	[Recognized]
[599]	AKT1	--->	MAP3K5	0.98638	-0.9981	[Recognized]
[600]	FYN	<---	PTPRF	-0.099692	0.053276	[Unrecognized]
[601]	ACLY	<---	PRKACA	-0.72114	0.69525	[Recognized]
[602]	CAMKK1	--->	AKT1	1.007	-1.0026	[Recognized]
[603]	BAD	<---	PIM2	-1.4217	1.2302	[Recognized]
[604]	MAPK3	--->	SMAD2	1.012	-1.0074	[Recognized]
[605]	PRKCB	--->	ITGB7	0.33316	-0.34136	[Recognized]
[606]	MAPK8	--->	TP53	1.0002	-0.99962	[Recognized]

[607]	CTNNB1	<---	CDK6	-0.99713	0.99917	[Recognized]
[608]	TESK1	--->	CFL1	0.45348	-0.47476	[Recognized]
[609]	KLK2	--->	SERPINE1	0.11833	-0.082185	[Recognized]
[610]	CAMK2G	--->	RYR1	0.12968	-0.14062	[Recognized]
[611]	CASP1	--->	MAPT	0.12628	-0.12396	[Recognized]
[612]	CREBBP	--->	KPNA2	0.18327	-0.18765	[Recognized]
[613]	CAPN1	--->	BID	0.27617	-0.27318	[Recognized]
[614]	CAMK2A	--->	ATP2A2	0.41881	-0.42746	[Recognized]
[615]	GRK1	--->	RHO	0.29762	-0.3178	[Recognized]
[616]	SRC	--->	SHC1	0.5117	-0.43547	[Recognized]
[617]	KLK2	--->	SERPINA3	0.11833	-0.082185	[Recognized]
[618]	BTK	--->	CD19	0.5753	-0.62253	[Recognized]
[619]	MAPK9	--->	JDP2	0.97462	-1.029	[Recognized]
[620]	ELANE	--->	LPA	0.0012258	-0.0091243	[Recognized]
[621]	CASP1	<---	NEDD4	-0.12469	0.113	[Unrecognized]
[622]	CAMK2G	--->	EGFR	1.0058	-1.0028	[Recognized]
[623]	MAPK1	--->	RAF1	0.98888	-1.0049	[Recognized]
[624]	EGFR	<---	CBL	-1.0135	1.0132	[Unrecognized]
[625]	NUAK1	<---	CASP6	-0.59442	0.52477	[Unrecognized]
[626]	PRKACA	--->	ITPKA	0.3157	-0.34471	[Recognized]
[627]	PTPN1	--->	MAPK1	1.0061	-1.009	[Recognized]
[628]	JAK1	--->	TYK2	0.012969	-0.0068414	[Recognized]
[629]	HCK	--->	DOK2	1.3209	-1.1853	[Recognized]
[630]	PLAT	--->	SERPINI1	0.037242	-0.032776	[Recognized]
[631]	JAK2	<---	RAF1	0.049279	0.06529	[Unrecognized]
[632]	PRKG1	--->	PDE5A	0.66782	-0.68921	[Recognized]
[633]	PRKCA	--->	PLCB1	0.26658	-0.27591	[Recognized]
[634]	RPS6KA1	--->	FOS	0.29655	-0.38418	[Recognized]
[635]	PAK1	--->	FOXL2	0.70562	-0.42214	[Recognized]
[636]	PTPN22	--->	LCK	0.034522	-0.05555	[Recognized]
[637]	DDR2	<---	SRC	-0.17141	0.74642	[Recognized]
[638]	MAPK1	--->	SP1	0.12886	-0.14656	[Recognized]
[639]	CASP5	--->	MAX	0.32179	-0.31143	[Recognized]
[640]	CSNK2A1	--->	MYF5	1.5563	-1.5271	[Recognized]
[641]	CAMK1	--->	EIF4G3	0.33156	-0.35333	[Recognized]
[642]	PRKCA	--->	F11R	0.35191	-0.36094	[Recognized]
[643]	ABL1	--->	MAPT	-0.01756	-0.082623	[Recognized]
[644]	JAK2	--->	CTLA4	0.17943	-0.11187	[Recognized]
[645]	CSNK1A1	--->	CTNNB1	0.99847	-0.98639	[Recognized]
[646]	RPS6KA5	--->	STAT1	0.020187	-0.03925	[Recognized]
[647]	CSNK2A2	--->	HDAC2	0.4	-0.42066	[Recognized]
[648]	PHKG1	--->	MAPT	0.99929	-1.0027	[Recognized]
[649]	RPS6KA1	--->	CEBPB	0.34196	-0.54207	[Recognized]
[650]	IRAK4	--->	IRAK1	1.0149	-1.0046	[Recognized]
[651]	MAPK8	--->	ELK3	1.0428	-0.98814	[Recognized]
[652]	GRIN2B	<---	CAMK2A	-0.30134	0.28954	[Recognized]
[653]	PRKCA	--->	SRC	0.25654	-0.23268	[Recognized]
[654]	PTPRC	--->	LCK	0.99837	-0.99758	[Recognized]
[655]	PPM1B	<---	IKBKB	-0.15493	0.026748	[Unrecognized]
[656]	CAMK2G	--->	FLNA	0.5684	-0.58821	[Recognized]
[657]	CDK1	--->	TSC1	0.79117	-0.7903	[Recognized]
[658]	BMP1	<---	COL1A1	-0.025745	0.012412	[Unrecognized]
[659]	KSR1	--->	MAPK1	0.19786	-0.090179	[Unrecognized]
[660]	SYK	--->	SH3BP2	1.004	-0.99817	[Recognized]
[661]	KAT5	<---	CDK1	-0.43407	0.41309	[Recognized]
[662]	EGLN2	--->	HIF1A	0.99907	-0.99517	[Recognized]
[663]	MAPK14	--->	JUNB	0.55135	-0.62628	[Recognized]
[664]	CASP1	<---	IL18	-0.065744	0.073886	[Unrecognized]
[665]	SENP6	--->	ARNT	0.22576	-0.21987	[Recognized]
[666]	GALNT1	--->	MUC1	0.17779	-0.17757	[Recognized]
[667]	ABL1	--->	PSTPIP1	-0.039964	-0.058413	[Recognized]
[668]	CDK7	<--->	CDK1	0.23429	0.23429	[Recognized]
[669]	ELANE	<---	F2R	-0.42368	0.45462	[Unrecognized]
[670]	MMP26	--->	MMP9	-0.025733	-0.20541	[Recognized]
[671]	INSR	--->	GAB1	0.13532	-0.14561	[Recognized]
[672]	CHUK	--->	PIAS1	1.0033	-1.0029	[Recognized]
[673]	PTK2B	?	PTK2	-0.13006	-0.13006	[Unrecognized]
[674]	LYN	--->	FCGR2A	0.11748	0.015127	[Recognized]
[675]	CASP6	--->	PCYT1A	0.58353	-0.69319	[Recognized]
[676]	LCK	--->	CD3E	1.1653	-1.1037	[Recognized]
[677]	CDK5	--->	PSEN1	0.67463	-0.70045	[Recognized]
[678]	VRK1	--->	PLK3	0.23606	-0.12147	[Unrecognized]
[679]	PRKCD	--->	GRM5	0.78537	-0.77559	[Recognized]
[680]	CSK	--->	PXN	0.99333	-0.91096	[Recognized]
[681]	FNTA	<---	RHOB	-0.44041	0.47292	[Unrecognized]
[682]	CDX2	<---	GSK3B	-0.82765	0.79979	[Recognized]

[683]	CASP3	--->	LYN	1.0113	-1.0017	[Recognized]
[684]	ACVR1	--->	SMAD1	1.5013	-1.5009	[Recognized]
[685]	PDPK1	--->	SGK1	1.0929	-1.1695	[Recognized]
[686]	MAPK8	--->	JUN	1.0022	-1.0148	[Recognized]
[687]	MAP4K1	--->	MAP3K11	1.0012	-1.003	[Recognized]
[688]	ATM	<---	TP53	-0.086307	0.073809	[Unrecognized]
[689]	NEK9	<--->	NEK6	0.23429	0.23429	[Recognized]
[690]	CSNK1D	--->	PSEN2	0.71499	-0.5784	[Recognized]
[691]	PRKCD	--->	MEP1B	0.89746	-0.88667	[Recognized]
[692]	CASP3	--->	ACIN1	0.54334	-0.64977	[Recognized]
[693]	PAK1	--->	RAF1	0.65894	-0.56404	[Recognized]
[694]	MAPK1	--->	NR3C1	0.99834	-1.0079	[Recognized]
[695]	CDK1	<---	RAB4A	-1.0074	1.0132	[Unrecognized]
[696]	SRC	--->	PRKCI	0.0965	-0.093142	[Recognized]
[697]	PLK1	--->	CDC25C	0.33126	-0.32604	[Recognized]
[698]	PRKCD	--->	PLCB1	0.21205	-0.21981	[Recognized]
[699]	PTPN12	--->	ABL1	0.049094	-0.14288	[Recognized]
[700]	PRKCD	--->	ITGB2	0.041771	-0.056258	[Recognized]
[701]	FGR	--->	HCLS1	0.99631	-1.0146	[Recognized]
[702]	CSNK2A1	--->	BRCA1	0.32242	-0.34057	[Recognized]
[703]	PRKCB	--->	PTPN11	0.6319	-0.62682	[Recognized]
[704]	PRKCG	--->	GJA1	0.22576	-0.23456	[Recognized]
[705]	SRC	--->	HNRNPK	1.0415	-0.92798	[Recognized]
[706]	CSNK2A1	--->	MYB	0.092606	-0.10814	[Recognized]
[707]	MAPK1	--->	SMAD4	1.012	-1.0074	[Recognized]
[708]	SYK	<---	LCK	-0.98599	1.0002	[Unrecognized]
[709]	ROCK1	<---	LIMK2	-0.053928	0.039587	[Unrecognized]
[710]	GSK3A	--->	MCL1	0.49108	-0.54794	[Recognized]
[711]	PRKCA	--->	PFKFB2	0.57052	-0.57532	[Recognized]
[712]	PRKACA	<---	PTPRR	-0.37559	0.36571	[Unrecognized]
[713]	CDK2	--->	MYBL2	0.24948	-0.26781	[Recognized]
[714]	AKT1	--->	GSK3A	0.99492	-0.99817	[Recognized]
[715]	PTPN12	--->	PTK2B	0.29598	-0.3482	[Recognized]
[716]	PTPRR	--->	MAPK1	0.36571	-0.37559	[Unrecognized]
[717]	SRC	--->	PTK2	0.9912	-1.0004	[Recognized]
[718]	CASP7	--->	MEF2A	0.17117	-0.20473	[Recognized]
[719]	PRKCA	--->	SHC1	0.15385	-0.17634	[Recognized]
[720]	PRKCD	--->	KCNJ1	0.81962	-0.8066	[Recognized]
[721]	ABL1	--->	JUN	-0.017125	-0.08419	[Recognized]
[722]	ADRBK1	--->	BDKRB2	0.20207	-0.22265	[Recognized]
[723]	CASP3	--->	MDM4	0.092589	-0.12838	[Recognized]
[724]	SRC	--->	CDH5	0.62261	-0.55927	[Recognized]
[725]	KIT	--->	STAT1	1.0099	-1.0004	[Recognized]
[726]	MAPK1	--->	CD19	0.55021	-0.57067	[Recognized]
[727]	CAMK2G	--->	GRIA1	0.62485	-0.62407	[Recognized]
[728]	PRKCA	--->	CYTH2	1.2556	-1.0819	[Recognized]
[729]	FYN	--->	FCGR2A	0.11748	0.015127	[Recognized]
[730]	CSNK2A1	--->	NFKBIA	1.454	-1.5082	[Recognized]
[731]	SYK	<---	PRKCA	-0.60642	0.70021	[Unrecognized]
[732]	MAPK8	--->	YWHAZ	0.62776	-0.66368	[Recognized]
[733]	MMP12	<---	F12	-0.073981	0.072802	[Unrecognized]
[734]	CDK5	<---	SRC	-1.3225	1.3946	[Unrecognized]
[735]	GALNT2	--->	MUC1	0.17779	-0.17757	[Recognized]
[736]	CDK1	<--->	PBK	0.23429	0.23429	[Recognized]
[737]	RAF1	--->	MAP2K1	0.9856	-1.0047	[Recognized]
[738]	CDK2	--->	CDX2	0.79979	-0.82765	[Recognized]
[739]	PDPK1	--->	ITGB3	0.10613	-0.084177	[Recognized]
[740]	CASP3	--->	STK3	1.0019	-1.0107	[Recognized]
[741]	MAP2K4	<--->	MAPK8	0.23429	0.23429	[Recognized]
[742]	CSNK2A1	--->	WAS	0.18552	-0.50928	[Recognized]
[743]	PRKACA	--->	GRIA1	1.0055	-0.99542	[Recognized]
[744]	GZMB	<---	CASP3	-0.70957	0.59854	[Unrecognized]
[745]	SGK1	--->	BRAF	0.42615	-0.56641	[Recognized]
[746]	PRKCA	--->	NFATC1	0.10256	-0.11537	[Recognized]
[747]	KLKB1	--->	F12	-0.0039613	-0.0063334	[Unrecognized]
[748]	PRKACA	<---	SRC	-1.3225	1.3946	[Unrecognized]
[749]	CASP3	--->	CAD	0.36691	-0.43968	[Recognized]
[750]	MAPK9	--->	NFATC3	1.0021	-0.99987	[Recognized]
[751]	CSNK2A2	--->	MYF5	1.5563	-1.5271	[Recognized]
[752]	SRC	--->	GRB10	0.7343	-0.48402	[Recognized]
[753]	SRC	--->	GRLF1	0.37553	-0.33012	[Recognized]
[754]	MAPK1	--->	GRB10	0.17357	-0.34335	[Recognized]
[755]	MMP9	<---	IL8	-0.16713	0.18806	[Unrecognized]
[756]	RPS6KA5	--->	ATF1	0.98749	-1.0017	[Recognized]
[757]	PRKCD	--->	HNRNPK	0.78324	-0.77356	[Recognized]
[758]	PRKACA	--->	RELA	1.0015	-1.0004	[Recognized]

[759]	PIAS1	--->	NR5A1	0.095399	-0.090133	[Recognized]
[760]	PTPN11	--->	STAT1	0.63902	-0.4446	[Recognized]
[761]	CMA1	<---	EDN1	0.015296	0.018665	[Unrecognized]
[762]	CSNK2A1	--->	ACACA	0.41949	-0.42896	[Recognized]
[763]	CAMK2A	--->	CEBPB	0.7187	-0.69952	[Recognized]
[764]	CDK1	<---	RAB5B	-1.0074	1.0132	[Unrecognized]
[765]	CDK3	--->	CABLES1	1.083	-1.1093	[Recognized]
[766]	PPP5C	--->	MAP3K5	0.092599	0.050577	[Recognized]
[767]	PTPN2	--->	INSR	0.26902	-0.28101	[Recognized]
[768]	CAMK1	--->	NOS1	0.48839	-0.49502	[Recognized]
[769]	MET	<---	GAB1	-0.038307	0.026098	[Unrecognized]
[770]	STK4	<---	CASP3	-0.59512	0.58465	[Recognized]
[771]	LYN	--->	PLCG1	0.81503	-1.0097	[Recognized]
[772]	EGFR	--->	CALM1	0.34959	-0.34973	[Recognized]
[773]	UHMK1	--->	PAM	0.65556	-0.68136	[Recognized]
[774]	CASP8	--->	EIF2AK2	0.99111	-0.99197	[Recognized]
[775]	CAPN1	--->	ITGB3	0.063315	-0.07395	[Recognized]
[776]	SRC	--->	BCAR1	0.99683	-0.98488	[Recognized]
[777]	MAPK7	--->	SGK1	0.23945	-0.1834	[Recognized]
[778]	MAP2K3	--->	MAPK14	1.0074	-0.9992	[Recognized]
[779]	CSK	<--->	FGR	0.37153	0.37153	[Recognized]
[780]	ZAP70	--->	DUSP3	1.0053	-1.0087	[Recognized]
[781]	CASP3	--->	PSEN2	0.58173	-0.69269	[Recognized]
[782]	HIPK2	--->	PAX6	0.64333	-0.66021	[Recognized]
[783]	CSNK2A2	--->	SPIB	1.1285	-1.1212	[Recognized]
[784]	PRKACA	--->	GYS1	1.0067	-1.0067	[Recognized]
[785]	CSNK2A1	--->	IL16	0.62013	-0.64226	[Recognized]
[786]	ABL1	<---	MAP4K1	-0.046372	0.017907	[Unrecognized]
[787]	C1S	--->	SERPING1	0.04046	-0.023809	[Recognized]
[788]	CAMK2G	--->	PLCB3	0.41213	-0.4132	[Recognized]
[789]	PTPN6	<---	LCK	-0.17366	0.056644	[Unrecognized]
[790]	ROCK2	--->	PPP1R12A	0.13459	-0.14024	[Recognized]
[791]	CASP1	--->	BID	0.067368	-0.028077	[Recognized]
[792]	PRKACA	--->	GMFB	0.75717	-0.78322	[Recognized]
[793]	ABL1	--->	CDK5	-0.013323	-0.14811	[Recognized]
[794]	EP300	--->	STAT3	0.98672	-1.0013	[Recognized]
[795]	PRKACA	--->	PFKFB2	0.6735	-0.69812	[Recognized]
[796]	MAPK7	--->	MEF2C	0.17185	-0.20713	[Recognized]
[797]	PTPN1	<---	SRC	-0.37818	0.26821	[Unrecognized]
[798]	JAK1	<---	IL10RA	-0.052954	0.045309	[Unrecognized]
[799]	CHEK1	--->	CDC25C	0.69298	-0.67632	[Recognized]
[800]	CSNK2A1	--->	TP53	1.0002	-0.99962	[Recognized]
[801]	CSNK2A1	--->	ARRB2	0.42184	-0.44306	[Recognized]
[802]	PAM	--->	FXYD1	0.20355	-0.1643	[Recognized]
[803]	IGFBP5	<---	PAPPA2	-0.038431	0.07239	[Recognized]
[804]	RPS6KA4	--->	ATF1	0.98749	-1.0017	[Recognized]
[805]	CDK1	--->	RB1	0.98937	-1.0015	[Recognized]
[806]	SRC	--->	STAT3	0.9865	-0.9998	[Recognized]
[807]	CDK5	--->	DAB1	0.78366	-0.80702	[Recognized]
[808]	RPS6KA3	--->	MAPT	0.35614	-0.49081	[Recognized]
[809]	FYN	--->	PLCG2	0.59106	-0.8605	[Recognized]
[810]	MAPK3	--->	TCF3	0.99488	-0.99939	[Recognized]
[811]	SGK3	<---	GSK3B	-0.6264	0.61504	[Unrecognized]
[812]	PTPN6	--->	PECAM1	0.26319	-0.077948	[Recognized]
[813]	INSR	<---	PTPN6	-0.13143	0.15547	[Unrecognized]
[814]	PPP2CA	--->	MAPT	0.29647	-0.30306	[Recognized]
[815]	CDK2	--->	UBTF	0.92033	-0.89929	[Recognized]
[816]	ADRBK1	--->	MC4R	0.1689	-0.18714	[Recognized]
[817]	LCK	--->	PIK3R1	1.0077	-0.99637	[Recognized]
[818]	PRKCD	--->	DAB2	0.78567	-0.77586	[Recognized]
[819]	PRKACA	--->	LIPE	0.53776	-0.55596	[Recognized]
[820]	PTPRE	--->	KCNB1	1.2976	-1.2911	[Recognized]
[821]	BCAN	--->	MMP10	0.022421	-0.030194	[Unrecognized]
[822]	PRKCB	--->	EIF4E	0.94696	-0.94609	[Recognized]
[823]	NR4A2	<---	PIAS4	-0.10153	0.10957	[Recognized]
[824]	MAPK1	--->	CEBPB	0.97322	-0.93471	[Recognized]
[825]	PRKG1	--->	RYR1	0.075945	-0.088067	[Recognized]
[826]	ABL1	--->	TP73	-0.012652	-0.034855	[Recognized]
[827]	PRKACA	--->	PDE4B	0.99264	-1.0111	[Recognized]
[828]	PRKCD	--->	CYTH1	1.033	-1.0023	[Recognized]
[829]	LYN	--->	HCLS1	0.99631	-1.0146	[Recognized]
[830]	FURIN	<---	NAGPA	-0.010292	-0.00123	[Unrecognized]
[831]	CDK5	<---	PAK1	-1.3014	1.4008	[Unrecognized]
[832]	MAPK1	--->	SMAD1	1.2525	-1.3294	[Recognized]
[833]	MAPK1	--->	CASP9	0.99863	-0.99771	[Recognized]
[834]	CHEK2	--->	CDC25C	0.41667	-0.41209	[Recognized]

[835]	CTSG	<---	F2RL1	-0.42368	0.45462	[Unrecognized]
[836]	CTSD	--->	APP	0.22815	-0.23983	[Recognized]
[837]	AURKA	--->	BRCA1	0.32242	-0.34057	[Recognized]
[838]	PRKCZ	--->	PSEN1	0.25781	-0.27139	[Recognized]
[839]	PRKDC	<---	HSP90AA1	-0.027061	0.01538	[Unrecognized]
[840]	ADAMTS4	--->	BCAN	0.0043435	-0.015468	[Recognized]
[841]	SRC	--->	PLCG1	1.0088	-0.9985	[Recognized]
[842]	MAPK14	--->	JDP2	0.5412	-0.63148	[Recognized]
[843]	LYN	--->	TRPV4	0.34238	-0.29213	[Recognized]
[844]	RANBP2	<---	MDM2	-0.0067123	0.033891	[Unrecognized]
[845]	BRAF	--->	MAP2K1	1.0017	-1.0053	[Recognized]
[846]	LYN	--->	FCGR2B	0.11748	0.015127	[Recognized]
[847]	CDK1	--->	PLEC1	0.72542	-0.75118	[Recognized]
[848]	EGFR	--->	PLCG1	1.0042	-1.0044	[Recognized]
[849]	MAPK3	--->	GJA1	0.49049	-0.48658	[Recognized]
[850]	MAPK8	--->	IRS1	0.5492	-0.60947	[Recognized]
[851]	MAPK1	<---	DUSP16	-1.0004	1.0016	[Unrecognized]
[852]	PRKACA	<---	NFKB1	-0.6564	0.6581	[Unrecognized]
[853]	DAPK1	--->	MAPK1	0.17015	-0.14879	[Unrecognized]
[854]	MAPK3	--->	CCDC6	0.71754	-0.7435	[Recognized]
[855]	RPS6KB1	--->	MAPT	0.35614	-0.49081	[Recognized]
[856]	WEE1	<--->	CDK2	0.23429	0.23429	[Recognized]
[857]	RHEBL1	--->	AKT1	0.09142	0.060359	[Recognized]
[858]	PRKACA	--->	RYR2	1.0028	-0.99449	[Recognized]
[859]	RPS6KA1	--->	NR4A2	0.56871	-0.73191	[Recognized]
[860]	CDC14A	--->	TP53	0.61877	-0.60562	[Recognized]
[861]	PRKCD	--->	ITGB7	0.28454	-0.28978	[Recognized]
[862]	CSNK2A1	--->	TCF7L2	1.0076	-0.99098	[Recognized]
[863]	MMP1	<---	COL2A1	-0.051184	0.041848	[Unrecognized]
[864]	SGK1	<---	NEDD4L	-0.16119	0.11149	[Unrecognized]
[865]	CASP3	--->	PKN1	0.066252	-0.14308	[Recognized]
[866]	AKT1	--->	MDM2	0.99136	-0.99587	[Recognized]
[867]	PELI3	<---	IRAK1	-0.98981	1.0139	[Unrecognized]
[868]	BRSK1	--->	CDC25C	0.41582	-0.41122	[Recognized]
[869]	CDK4	--->	RB1	0.98937	-1.0015	[Recognized]
[870]	ELANE	--->	TFPI	0.024385	0.0096062	[Recognized]
[871]	ZAP70	--->	GAB2	1.0021	-0.98622	[Recognized]
[872]	PTPN11	<---	EGFR	-0.25411	0.37037	[Unrecognized]
[873]	CTSG	<---	C3	-0.10518	0.11279	[Unrecognized]
[874]	PRKCA	--->	ADRA1B	0.3038	-0.31201	[Recognized]
[875]	PDK3	--->	PDHA1	1.0004	-0.99606	[Recognized]
[876]	CAMK2G	--->	ADCY3	0.35746	-0.37383	[Recognized]
[877]	PRKCA	--->	RALBP1	0.53812	-0.54304	[Recognized]
[878]	CASP3	<---	MAP4K1	-0.018328	0.03452	[Unrecognized]
[879]	PRKCA	--->	GNA15	0.89037	-0.89029	[Recognized]
[880]	EPHA3	--->	STAT3	0.31675	-0.38189	[Recognized]
[881]	CDC42BPA	--->	LIMK1	0.011701	-0.014076	[Recognized]
[882]	TAF1	--->	GTF2A1	0.043644	-0.045238	[Recognized]
[883]	CDK1	--->	AMPH	0.2474	-0.26764	[Recognized]
[884]	CSNK2A1	--->	SHOX	0.60726	-0.63441	[Recognized]
[885]	FYN	--->	CNN1	1.0728	-0.91195	[Recognized]
[886]	AKT1	--->	ESR1	1.0083	-1.0039	[Recognized]
[887]	RORC	<---	EIF4EBP1	-0.099837	0.12515	[Unrecognized]
[888]	FYN	--->	CD79A	1.0799	-1.0399	[Recognized]
[889]	PRKACA	--->	STMN1	1.0058	-0.99021	[Recognized]
[890]	PTPN11	<---	GAB1	-0.0502	0.060242	[Unrecognized]
[891]	RALA	<---	PPP2R1B	-0.13281	0.13801	[Recognized]
[892]	CAMK4	--->	HDAC4	0.68838	-0.71414	[Recognized]
[893]	CSNK2A2	<---	HSPH1	-0.99901	1.0063	[Unrecognized]
[894]	PTPRC	--->	JAK2	0.19532	-0.20418	[Recognized]
[895]	BMP1	<---	COL7A1	-0.013155	0.00062382	[Unrecognized]
[896]	MMP17	<---	TNF	-0.35472	0.3692	[Unrecognized]
[897]	RPS6KA2	--->	L1CAM	0.03878	-0.13396	[Recognized]
[898]	MMP2	<---	HAPLN1	-0.093464	0.097177	[Unrecognized]
[899]	PRKCA	--->	GRM5	0.91747	-0.9157	[Recognized]
[900]	GSK3B	--->	CREM	0.96654	-0.95401	[Recognized]
[901]	PRKCA	--->	PLD1	0.99655	-1.0006	[Recognized]
[902]	CDX2	<---	MAPK14	-0.50238	0.33164	[Recognized]
[903]	PLK3	<---	CHEK2	-0.00064284	0.1132	[Unrecognized]
[904]	AKT1	<---	MAP3K11	0.0045156	0.046402	[Unrecognized]
[905]	TFPI	--->	MMP12	0.20851	-0.1877	[Unrecognized]
[906]	MAPK3	--->	UBTF	0.92033	-0.89929	[Recognized]
[907]	MAPK14	--->	EIF4EBP1	0.40574	-0.56693	[Recognized]
[908]	PTPRC	--->	LYN	1.007	-0.99313	[Recognized]
[909]	PRKCA	--->	CREM	0.54759	-0.54466	[Recognized]
[910]	MYT1	<---	CDK1	-0.44897	0.42777	[Unrecognized]

[911]	PTPN22	--->	ZAP70	0.10398	-0.33186	[Recognized]
[912]	PRKCA	--->	RGS19	0.87438	-0.87353	[Recognized]
[913]	MAP3K7	--->	SMAD3	0.23582	-0.21256	[Recognized]
[914]	CSNK2A1	--->	DDIT3	0.97322	-0.93471	[Recognized]
[915]	MMP1	<---	TFPI	-0.1877	0.20851	[Unrecognized]
[916]	AKT2	--->	XIAP	0.30869	-0.23198	[Recognized]
[917]	CDK5	--->	MAPT	0.99929	-1.0027	[Recognized]
[918]	CDK20	<--->	CDK2	0.23429	0.23429	[Recognized]
[919]	PRKACA	--->	MAP2	0.56962	-0.58579	[Recognized]
[920]	MAPK1	--->	KHDRBS1	0.35791	-0.37587	[Recognized]
[921]	AKT1	--->	FOXO4	0.99774	-1.0046	[Recognized]
[922]	MAP2K4	<--->	MAPK9	0.23429	0.23429	[Recognized]
[923]	CSNK2A1	--->	APEX1	0.70304	-0.7292	[Recognized]
[924]	MAPKAPK2	--->	SRF	0.28633	-0.30066	[Recognized]
[925]	MEP1B	<---	NPY	-0.27935	0.31554	[Unrecognized]
[926]	PRKCA	--->	CASR	0.55548	-0.55976	[Recognized]
[927]	ILK	--->	AKT1	0.04085	0.0092002	[Recognized]
[928]	MAPK1	--->	BCL2	1.1791	-1.2078	[Recognized]
[929]	SRC	--->	CTNNB1	0.16647	-0.1713	[Recognized]
[930]	PAPPA	--->	IGFBP4	0.07239	-0.038431	[Recognized]
[931]	MAPK8	--->	BCL2	1.1791	-1.2078	[Recognized]
[932]	PRKACA	--->	RAF1	0.98888	-1.0049	[Recognized]
[933]	GZMB	--->	GRIA3	0.13835	-0.13152	[Recognized]
[934]	MKNK1	--->	PLA2G4A	0.50475	-0.50164	[Recognized]
[935]	CASP3	--->	RASA1	-0.026536	-0.078902	[Recognized]
[936]	PRKCA	--->	OPRD1	0.87512	-0.87377	[Recognized]
[937]	PRKACA	--->	CUL5	0.30021	-0.32466	[Recognized]
[938]	FYN	--->	TOM1L1	1.0637	-0.91225	[Recognized]
[939]	RB1	<---	EP300	-0.061481	0.049731	[Recognized]
[940]	PRKACA	--->	GJB1	0.53362	-0.54829	[Recognized]
[941]	KAT2B	--->	RELA	0.064754	-0.072138	[Recognized]
[942]	ABL1	<---	BTK	-0.098628	-0.018782	[Unrecognized]
[943]	DUSP3	--->	MAP2K2	0.9172	-0.50725	[Recognized]
[944]	PRKCA	--->	TRPV6	0.59235	-0.59608	[Recognized]
[945]	CASP3	--->	IL18	0.56414	-0.67524	[Recognized]
[946]	ATM	<---	MRE11A	-0.227	0.21692	[Unrecognized]
[947]	PRKACA	--->	ITPR1	0.3288	-0.33886	[Recognized]
[948]	CDK1	--->	CDC25A	0.69298	-0.67632	[Recognized]
[949]	CASP6	<---	NEDD4	-0.072983	0.051513	[Unrecognized]
[950]	MAP3K14	--->	IKBKB	1.0117	-1.003	[Recognized]
[951]	PRKD1	--->	HDAC7	0.44235	-0.46746	[Recognized]
[952]	HCK	<---	CBL	-0.7389	0.74409	[Unrecognized]
[953]	NR4A1	<---	RPS6KA3	-0.73191	0.56871	[Recognized]
[954]	PRKCH	--->	PRKD1	0.55695	-0.52651	[Recognized]
[955]	PRKACA	--->	ETV1	1.1285	-1.1212	[Recognized]
[956]	EGFR	<---	PTPN1	-0.079237	0.074053	[Unrecognized]
[957]	PRKCA	--->	DGKZ	0.50196	-0.17365	[Recognized]
[958]	CSNK2A1	--->	TOP2A	0.52133	-0.54823	[Recognized]
[959]	SRC	--->	BMX	0.93228	-0.45147	[Recognized]
[960]	SRC	--->	KIT	0.98487	-0.98796	[Recognized]
[961]	ABL1	<---	MDM2	-0.038456	-0.0058126	[Unrecognized]
[962]	SYK	--->	HCLS1	0.98897	-0.99533	[Recognized]
[963]	PTPN6	--->	CD22	0.31368	-0.29575	[Recognized]
[964]	PRKACA	<---	LCK	-0.99895	0.99734	[Unrecognized]
[965]	GSK3B	--->	MUC1	0.42246	-0.44429	[Recognized]
[966]	LCK	--->	CTLA4	0.99954	-1.0121	[Recognized]
[967]	CDK1	--->	UBE2A	0.50887	-0.53523	[Recognized]
[968]	SRC	--->	GRB2	0.86853	-0.088967	[Recognized]
[969]	BRSK1	<---	WEE1	0.13714	0.14515	[Unrecognized]
[970]	GRIN2B	<---	CAMK2B	-0.30134	0.28954	[Recognized]
[971]	MMP2	<---	CCL7	-0.16713	0.18806	[Unrecognized]
[972]	PRKCA	--->	EZR	0.33349	-0.34166	[Recognized]
[973]	CAMK2A	--->	SRF	0.30287	-0.31641	[Recognized]
[974]	GSK3B	--->	CEBPA	0.97322	-0.93471	[Recognized]
[975]	MAPK1	--->	IRS1	0.5492	-0.60947	[Recognized]
[976]	PIAS1	--->	TP73	0.068649	-0.068273	[Recognized]
[977]	ATM	<---	E2F1	-0.14013	0.12852	[Unrecognized]
[978]	MAPK3	--->	SREBF1	0.54159	-0.54127	[Recognized]
[979]	MAPK1	--->	GAB2	0.99738	-0.99704	[Recognized]
[980]	CSK	<--->	LYN	0.37153	0.37153	[Recognized]
[981]	PRKD1	<---	EPAS1	-0.053023	0.039807	[Unrecognized]
[982]	MAPK12	--->	SNTA1	0.36823	-0.49063	[Recognized]
[983]	MMP2	<---	IGFBP3	-0.15977	0.18055	[Unrecognized]
[984]	LYN	<---	PRKCD	-0.3255	0.34363	[Unrecognized]
[985]	PRKCA	--->	ATP2B1	0.14677	-0.15808	[Recognized]
[986]	MMP1	<---	CCL13	-0.16713	0.18806	[Unrecognized]

[987]	LYN	--->	SYK	1.0025	-1.0044	[Recognized]
[988]	CTRC	<---	PHKG1	-0.72828	0.70234	[Unrecognized]
[989]	PPP2CB	--->	PRKCB	0.15086	-0.15768	[Recognized]
[990]	MMP7	<---	SERPINA1	-0.13073	0.15283	[Unrecognized]
[991]	MAPK1	--->	SMAD3	1.012	-1.0074	[Recognized]
[992]	MAPK1	<---	MAP2K1	-0.99672	1.0159	[Unrecognized]
[993]	ABL1	<---	CDKN1B	-0.033446	-0.030457	[Unrecognized]
[994]	CSNK2A1	--->	CDC25B	0.69298	-0.67632	[Recognized]
[995]	PLCG1	<---	RET	-0.66968	0.49605	[Recognized]
[996]	ITK	--->	CD28	0.78153	-0.85158	[Recognized]
[997]	CDK2	--->	TP73	0.45486	-0.4556	[Recognized]
[998]	KAT5	--->	H3F3B	0.15564	-0.12976	[Recognized]
[999]	MMP1	--->	CD44	-0.0016332	-0.0037852	[Recognized]
[1000]	IGFBP5	<---	F2	-0.013006	0.028162	[Recognized]
[1001]	SRC	--->	KCNB1	1.0056	-0.89226	[Recognized]
[1002]	FYN	<---	ACP1	0.010724	0.13856	[Unrecognized]
[1003]	RPS6KA1	--->	GMFB	0.26248	-0.48635	[Recognized]
[1004]	MAPK8IP3	<---	MAPK8	-0.16592	0.14982	[Recognized]
[1005]	PTPN6	--->	CD33	0.65471	-0.5666	[Recognized]
[1006]	INSR	<---	CALM2	-0.1135	0.10567	[Unrecognized]
[1007]	PRKCA	--->	ADAP1	0.91081	-0.90921	[Recognized]
[1008]	CASP3	--->	PRKCQ	0.20957	-0.1791	[Recognized]
[1009]	CASP7	--->	TNFRSF1A	0.35625	-0.32424	[Recognized]
[1010]	FYN	--->	GRB10	0.85304	-0.6448	[Recognized]
[1011]	RPS6KA1	--->	NR4A3	0.56871	-0.73191	[Recognized]
[1012]	EGFR	--->	CTNND1	0.10935	-0.12117	[Recognized]
[1013]	CASP8	--->	TRAF1	0.99771	-1.011	[Recognized]
[1014]	STK11	--->	STRADA	0.6413	-0.21779	[Recognized]
[1015]	PRKCA	--->	OCLN	0.94019	-0.93859	[Recognized]
[1016]	PRKCA	--->	CORO1B	0.92236	-0.92085	[Recognized]
[1017]	CASP9	<---	CASP3	0.10027	0.17851	[Recognized]
[1018]	SYK	--->	PLCG1	1.0054	-1.0267	[Recognized]
[1019]	PRKDC	--->	NR3C1	0.025211	-0.024204	[Recognized]
[1020]	SRC	--->	AKT1	0.99355	-0.99803	[Recognized]
[1021]	AKT1	--->	EZH2	0.45566	-0.30774	[Recognized]
[1022]	SRC	--->	ITGB3	0.22832	-0.2137	[Recognized]
[1023]	PRKACA	--->	RGS14	0.55444	-0.55865	[Recognized]
[1024]	TYK2	--->	IFNAR1	0.17162	-0.18353	[Recognized]
[1025]	RPS6KA5	--->	CREB1	1.0082	-0.98976	[Recognized]
[1026]	PRKDC	--->	TP53	0.12799	-0.13244	[Recognized]
[1027]	PIAS1	--->	SMAD4	1.0161	-0.98478	[Recognized]
[1028]	RPS6KA1	--->	CREB1	1.0082	-0.98976	[Recognized]
[1029]	ZAP70	--->	MUC1	0.98926	-1.0023	[Recognized]
[1030]	KLK3	--->	IGFBP3	0.070438	-0.036479	[Recognized]
[1031]	LCK	--->	IL2RB	0.99939	-1.0044	[Recognized]
[1032]	ATM	<---	STK11	-0.14308	0.13159	[Unrecognized]
[1033]	MARK1	--->	MAP4	0.1044	-0.11787	[Recognized]
[1034]	MAPKAPK2	--->	TSC2	0.63455	-0.63713	[Recognized]
[1035]	MMP13	<---	F12	-0.073981	0.072802	[Unrecognized]
[1036]	MAPK14	--->	MAX	0.99106	-0.98599	[Recognized]
[1037]	SRC	--->	CD33	0.74656	-0.70289	[Recognized]
[1038]	CSNK1E	--->	PER3	0.72042	-0.58384	[Recognized]
[1039]	EPHB2	--->	RRAS	0.17617	-0.18451	[Recognized]
[1040]	CASP6	--->	MAPT	0.50832	-0.57833	[Recognized]
[1041]	LYN	--->	CDK1	1.3862	-1.2776	[Recognized]
[1042]	AKT1	--->	RAF1	1.0008	-1.0133	[Recognized]
[1043]	TERF1	--->	ATM	0.21663	-0.22672	[Unrecognized]
[1044]	PRKCZ	--->	CASP9	0.2012	-0.21571	[Recognized]
[1045]	CDK1	--->	TOP2A	0.52133	-0.54823	[Recognized]
[1046]	MAPK7	--->	ETS1	0.57541	-0.6753	[Recognized]
[1047]	CDK2	--->	PRKAR1A	0.27068	-0.33631	[Recognized]
[1048]	EP300	--->	MYB	0.018722	-0.03137	[Recognized]
[1049]	MAPK14	--->	ELK1	1.0099	-1.0011	[Recognized]
[1050]	FCGR3A	<---	LCK	-0.44297	0.51622	[Unrecognized]
[1051]	PTPN6	<---	SRC	0.083854	0.2006	[Unrecognized]
[1052]	PRKCD	--->	CREBBP	0.032186	-0.044787	[Recognized]
[1053]	AKT1	--->	YWHAZ	0.44249	-0.29699	[Recognized]
[1054]	EGFR	--->	STAT5B	0.99454	-0.99025	[Recognized]
[1055]	PRKCD	--->	OPRD1	0.75968	-0.75016	[Recognized]
[1056]	CSNK2A2	--->	BID	0.77321	-0.7866	[Recognized]
[1057]	MAP3K14	--->	CHUK	1.0117	-1.003	[Recognized]
[1058]	MAPK1	--->	STAT3	1.0077	-1.0166	[Recognized]
[1059]	PAK1	--->	MYLK	0.53897	-0.35021	[Recognized]
[1060]	CSNK2A1	--->	RELA	1.0015	-1.0004	[Recognized]
[1061]	TFPI	--->	MMP7	0.20851	-0.1877	[Unrecognized]
[1062]	PRKAA2	--->	HNF4A	-0.009204	-0.11591	[Recognized]

[1063]	GZMA	--->	APEX1	0.017674	0.016057	[Recognized]
[1064]	MAP2K1	--->	RPS6KA4	0.92747	-1.0338	[Recognized]
[1065]	SRC	--->	STAT5B	0.57328	-0.56898	[Recognized]
[1066]	CDK7	<--->	CDK2	0.23429	0.23429	[Recognized]
[1067]	CSNK2A2	--->	EIF4EBP1	1.0012	-1.0031	[Recognized]
[1068]	ABL1	--->	CD19	0.037898	-0.14493	[Recognized]
[1069]	GSK3A	--->	MAPT	0.38658	-0.41618	[Recognized]
[1070]	CSNK2A1	--->	MYC	1.0154	-1.0065	[Recognized]
[1071]	CASP1	<---	IL1B	-0.084506	0.083786	[Unrecognized]
[1072]	SYK	<---	MAP4K1	-0.15553	0.18246	[Unrecognized]
[1073]	PTPN1	--->	INSR	0.26902	-0.28101	[Recognized]
[1074]	CAMK2G	--->	PEA15	0.47512	-0.45726	[Recognized]
[1075]	PRKCQ	--->	MSN	0.089314	-0.09498	[Recognized]
[1076]	ACAN	--->	MMP8	0.022421	-0.030194	[Unrecognized]
[1077]	MAPK3	--->	PLCB1	1.0018	-1.001	[Recognized]
[1078]	RHEB	--->	RAF1	1.0008	-0.9994	[Recognized]
[1079]	MAPK1	--->	NR4A1	0.99834	-1.0079	[Recognized]
[1080]	CDC42BPA	--->	MYL2	0.11462	-0.12425	[Recognized]
[1081]	MAPK1	--->	RXRA	0.60286	-0.8344	[Recognized]
[1082]	CDK1	--->	LATS1	0.18008	-0.12423	[Recognized]
[1083]	BMP1	<---	COL5A2	-0.037281	0.024115	[Unrecognized]
[1084]	CSNK2A1	--->	C1R	0.45691	-0.47778	[Recognized]
[1085]	PRKACA	--->	GJA5	0.53362	-0.54829	[Recognized]
[1086]	PRKACA	<---	STK11	-0.21779	0.6413	[Unrecognized]
[1087]	TP53	<---	HDAC1	-0.22533	0.22693	[Recognized]
[1088]	SRC	--->	RGS16	0.92473	-0.81283	[Recognized]
[1089]	PRKD3	--->	GSK3A	0.28762	-0.077068	[Recognized]
[1090]	MAP3K7	--->	IKBKB	1.0099	-0.99837	[Recognized]
[1091]	MAPK3	<---	GAB1	-0.19052	0.17127	[Unrecognized]
[1092]	CSNK2A2	--->	UBE2R2	0.2372	-0.25823	[Recognized]
[1093]	PRKCG	--->	GRM5	0.91747	-0.9157	[Recognized]
[1094]	PRKCE	--->	ADAP1	0.61485	-0.68245	[Recognized]
[1095]	CDK2	--->	ID2	1.5563	-1.5271	[Recognized]
[1096]	ABL1	<---	PTPN6	-0.42833	0.086412	[Unrecognized]
[1097]	PTPN18	<---	SRC	-0.37818	0.26821	[Unrecognized]
[1098]	PRKCZ	--->	GRM5	0.26626	-0.27969	[Recognized]
[1099]	CLK1	<---	PTPN1	-1.009	1.0061	[Unrecognized]
[1100]	MAPKAPK5	--->	TP53	0.58544	-0.66628	[Recognized]
[1101]	GZMB	<---	CASP7	-0.70957	0.59854	[Unrecognized]
[1102]	CDC14B	--->	TP53	0.61877	-0.60562	[Recognized]
[1103]	PRKACA	--->	ATP2B1	1.006	-1.0042	[Recognized]
[1104]	CSNK2A1	--->	HCLS1	0.42702	-0.44058	[Recognized]
[1105]	IKBKB	--->	NCOA3	0.091219	-0.11492	[Recognized]
[1106]	MMP13	<---	CCL7	-0.16713	0.18806	[Unrecognized]
[1107]	AKT1	--->	GATA2	0.49316	-0.34165	[Recognized]
[1108]	PRKACA	--->	PSEN1	0.67463	-0.70045	[Recognized]
[1109]	AKT1	--->	GSK3B	1.0168	-1.0071	[Recognized]
[1110]	PRKCB	--->	RRAD	1.0123	-0.99154	[Recognized]
[1111]	CDK1	--->	MEF2C	0.43947	-0.44112	[Recognized]
[1112]	HCK	--->	PLCG2	0.59106	-0.8605	[Recognized]
[1113]	MAPK9	--->	H3F3A	1.0832	-1.0289	[Recognized]
[1114]	SSH1	--->	CFL2	0.3315	-0.32562	[Recognized]
[1115]	CSNK2A1	--->	SPIB	1.1285	-1.1212	[Recognized]
[1116]	PTPRJ	--->	MET	0.44701	-0.46104	[Recognized]
[1117]	RPS6KA1	--->	BAD	0.58958	-0.91274	[Recognized]
[1118]	DYRK1A	--->	CCNL2	0.64897	-0.66864	[Recognized]
[1119]	CAMK2D	--->	CREM	0.61276	-0.61036	[Recognized]
[1120]	LCK	--->	RASA1	0.096327	-0.22768	[Recognized]
[1121]	ABL2	<---	CRK	-0.99908	0.99616	[Unrecognized]
[1122]	LCK	--->	VAV1	1.0032	-1.0051	[Recognized]
[1123]	LIMK2	--->	CFL1	0.16176	-0.17545	[Recognized]
[1124]	BACE1	--->	APP	0.36802	-0.37802	[Recognized]
[1125]	PRKCD	--->	IL6ST	0.176	-0.18517	[Recognized]
[1126]	PRKACA	--->	BAD	1.2302	-1.4217	[Recognized]
[1127]	CHEK2	--->	E2F1	0.26761	-0.2868	[Recognized]
[1128]	MMP10	?	MMP9	-0.12951	-0.12951	[Unrecognized]
[1129]	SRC	--->	DOK2	1.2026	-1.146	[Recognized]
[1130]	CAPN2	<---	ADRBK1	-0.091658	0.074704	[Unrecognized]
[1131]	CSNK1A1	--->	CREM	0.30471	-0.29126	[Recognized]
[1132]	PRKACA	--->	CAD	0.43416	-0.45528	[Recognized]
[1133]	PRKCZ	--->	PEBP1	1.0061	-1.0011	[Recognized]
[1134]	MBTPS1	--->	BDNF	0.083504	-0.088859	[Recognized]
[1135]	GSK3B	--->	CTNNB1	0.99917	-0.99713	[Recognized]
[1136]	AKT1	--->	CCDC88A	0.45026	-0.3034	[Recognized]
[1137]	FURIN	<---	ADAM19	-0.013374	0.01027	[Unrecognized]
[1138]	ADAM17	<---	ERBB4	-0.058927	0.048672	[Unrecognized]

[1139]	CSNK1D	--->	CTNNB1	0.62178	-0.61528	[Recognized]
[1140]	CMA1	<---	EDN3	0.015296	0.018665	[Unrecognized]
[1141]	SYK	--->	SHC1	1.0049	-0.99981	[Recognized]
[1142]	FYN	--->	CTNNB1	0.24969	-0.25034	[Recognized]
[1143]	PRKG1	--->	CREB1	0.54384	-0.54419	[Recognized]
[1144]	MMP3	<---	CCL7	-0.16713	0.18806	[Unrecognized]
[1145]	CASP3	--->	PRKCZ	0.19066	-0.19986	[Recognized]
[1146]	BTK	--->	WAS	0.4868	-0.53132	[Recognized]
[1147]	CSNK2A1	--->	MYCN	1.1385	-1.1233	[Recognized]
[1148]	CDK2	--->	FOXM1	0.85888	-0.87871	[Recognized]
[1149]	PTPN11	--->	GAB2	0.99997	-1.0165	[Recognized]
[1150]	PRKCE	--->	TRPV1	0.42132	-0.46647	[Recognized]
[1151]	MAPK1	<---	LCK	-0.99895	0.99734	[Unrecognized]
[1152]	MMP8	<---	CCL2	-0.16713	0.18806	[Unrecognized]
[1153]	PTPRR	--->	MAPK3	0.36571	-0.37559	[Unrecognized]
[1154]	PRKCA	--->	BCL2	0.61202	-0.61716	[Recognized]
[1155]	PRKACA	--->	PDE3B	0.53012	-0.53853	[Recognized]
[1156]	PRKCD	--->	ADRBK1	0.20529	0.0040446	[Recognized]
[1157]	CDK1	<---	PTPN1	-1.009	1.0061	[Unrecognized]
[1158]	CAMK2G	<---	RRAD	-0.48262	0.48194	[Unrecognized]
[1159]	KLKB1	--->	MST1	0.0012258	-0.0091243	[Recognized]
[1160]	MMP9	<---	HAPLN1	-0.093464	0.097177	[Unrecognized]
[1161]	CASP8	--->	PSEN1	0.56121	-0.70272	[Recognized]
[1162]	TGFBR1	--->	SMAD1	1.6219	-1.6278	[Recognized]
[1163]	MAPK8	--->	MAPK8IP1	0.2909	-0.30961	[Recognized]
[1164]	ABL1	<---	CRK	-0.99908	0.99616	[Unrecognized]
[1165]	GSK3B	<---	NFKB1	-0.6564	0.6581	[Unrecognized]
[1166]	PRKG1	--->	MRVI1	0.99161	-1.0025	[Recognized]
[1167]	CSNK2A1	--->	NKX2-5	0.79979	-0.82765	[Recognized]
[1168]	PAK2	--->	MKNK1	0.84646	-0.77353	[Recognized]
[1169]	AKT1	--->	TSC2	1.0085	-0.99774	[Recognized]
[1170]	CHEK2	--->	CDC25A	0.41667	-0.41209	[Recognized]
[1171]	MAPK9	--->	ELK3	1.0428	-0.98814	[Recognized]
[1172]	TAOK1	<---	MARK1	0.012035	0.045095	[Unrecognized]
[1173]	PRKG1	--->	PLCB3	0.62876	-0.62602	[Recognized]
[1174]	JAK1	--->	STAT1	1.0095	-1.0128	[Recognized]
[1175]	MAPK1	--->	LIFR	0.74193	-0.77508	[Recognized]
[1176]	MMP12	<---	LPA	-0.11212	0.11927	[Unrecognized]
[1177]	FYN	--->	CTLA4	0.99605	-0.98986	[Recognized]
[1178]	FYN	--->	MAG	0.088878	-0.12752	[Recognized]
[1179]	PPP5C	--->	MAPT	0.12638	-0.12832	[Recognized]
[1180]	CHEK1	<--->	TLK1	0.23429	0.23429	[Recognized]
[1181]	PRKCG	--->	CYTH2	1.2556	-1.0819	[Recognized]
[1182]	PRKAA2	--->	PFKFB2	0.086531	-0.28854	[Recognized]
[1183]	MAPK3	<---	LCK	-0.99895	0.99734	[Unrecognized]
[1184]	PTPRC	--->	TYK2	0.14022	-0.1486	[Recognized]
[1185]	CTSG	--->	CXCL12	0.058311	-0.024098	[Recognized]
[1186]	PTPN1	--->	STAT5A	1.0055	-1.005	[Recognized]
[1187]	INSR	--->	PIK3R1	0.031725	-0.06783	[Recognized]
[1188]	SETD7	--->	TP53	0.32543	-0.32337	[Recognized]
[1189]	PRKCA	--->	DDX5	0.33929	-0.34734	[Recognized]
[1190]	CASP7	--->	PRKCZ	0.19066	-0.19986	[Recognized]
[1191]	PRKACA	--->	KLF1	0.63385	-0.64027	[Recognized]
[1192]	PRKCA	--->	PTGIR	0.87512	-0.87377	[Recognized]
[1193]	CAMKK1	--->	CAMK1D	0.83082	-0.43719	[Recognized]
[1194]	SRC	--->	MPZL1	1.0651	-1.0576	[Recognized]
[1195]	MAPK14	--->	MEF2A	0.63663	-0.66297	[Recognized]
[1196]	ATR	<---	CHEK2	-0.099248	0.084255	[Unrecognized]
[1197]	PDPK1	--->	SGK2	1.0929	-1.1695	[Recognized]
[1198]	CDK2	--->	SMAD3	1.012	-1.0074	[Recognized]
[1199]	PTPN1	--->	BCR	0.18717	-0.19601	[Recognized]
[1200]	GSK3B	--->	APP	0.38256	-0.39431	[Recognized]
[1201]	PRMT1	<---	STAT1	-0.082675	0.028398	[Unrecognized]
[1202]	PTPN6	--->	SIRPA	0.97001	-0.82769	[Recognized]
[1203]	SIRT1	--->	RELA	0.037635	-0.048408	[Recognized]
[1204]	EGFR	--->	STAT1	0.99452	-0.99826	[Recognized]
[1205]	CDK1	--->	APLP2	0.17087	-0.18588	[Recognized]
[1206]	PRKCD	--->	YWHAZ	0.77791	-0.76873	[Recognized]
[1207]	CSNK2A1	<---	HSPH1	-0.99901	1.0063	[Unrecognized]
[1208]	PAK1	--->	ARHGEF2	0.25671	-0.13368	[Recognized]
[1209]	CSNK2A2	--->	ARRB2	0.42184	-0.44306	[Recognized]
[1210]	PRKCA	--->	CBL	0.29229	-0.30458	[Recognized]
[1211]	PRKACA	--->	KCNH2	0.18176	-0.22705	[Recognized]
[1212]	TBK1	--->	NFKBIA	1.0116	-0.99754	[Recognized]
[1213]	MAPK14	--->	STAT4	0.98606	-1.0018	[Recognized]
[1214]	CASP3	--->	EIF4B	0.15504	-0.17985	[Recognized]

[1215]	MAP2K6	--->	MAPK12	0.64901	-0.41806	[Recognized]
[1216]	PTPN6	--->	KIT	0.98781	-1.0015	[Recognized]
[1217]	SIK3	<---	CRTC2	-0.075794	0.069802	[Unrecognized]
[1218]	MMP7	<---	PLG	-0.11212	0.11927	[Unrecognized]
[1219]	PTPRC	--->	JAK1	0.085657	-0.11378	[Recognized]
[1220]	PTPN11	--->	PDGFRB	0.13294	-0.12047	[Recognized]
[1221]	CASP3	<---	NEDD4	-0.072983	0.051513	[Unrecognized]
[1222]	LCK	--->	ACP1	0.47695	-0.39756	[Recognized]
[1223]	FURIN	--->	ADAMTS1	-0.00093443	-0.0067844	[Recognized]
[1224]	GSK3B	--->	CCND1	1.0544	-1.0774	[Recognized]
[1225]	MAPK8	--->	PPARG	0.60373	-0.61504	[Recognized]
[1226]	RPS6KA2	--->	CREB1	1.0082	-0.98976	[Recognized]
[1227]	LCK	--->	SH2B3	0.99538	-1.0003	[Recognized]
[1228]	MAP2K6	--->	MAPK14	1.0074	-0.9992	[Recognized]
[1229]	RPS6KA5	--->	HIST3H3	1.009	-0.99586	[Recognized]
[1230]	ELANE	--->	SERPIND1	0.11833	-0.082185	[Recognized]
[1231]	CSNK1D	--->	GJA1	0.20086	-0.17844	[Recognized]
[1232]	BTK	--->	STAT5A	0.22463	-0.23196	[Recognized]
[1233]	ACP1	--->	SPTAN1	0.1578	-0.16593	[Recognized]
[1234]	PRTN3	<---	F2RL1	-0.42368	0.45462	[Unrecognized]
[1235]	GSK3A	--->	CREM	0.35976	-0.37116	[Recognized]
[1236]	KAT5	--->	CCNB1	0.39097	-0.38819	[Unrecognized]
[1237]	CASP3	--->	EIF2AK2	0.87492	-0.98085	[Recognized]
[1238]	AKT1	--->	PFKFB2	0.30563	-0.22251	[Recognized]
[1239]	PRKCD	--->	ADRB2	0.46078	-0.46051	[Recognized]
[1240]	SRC	--->	EGFR	0.58287	-0.14267	[Recognized]
[1241]	OXSR1	<---	PAK1	-0.84122	0.89243	[Unrecognized]
[1242]	LCK	--->	LCP2	1.0058	-0.99925	[Recognized]
[1243]	FURIN	<---	TNFSF13	-0.13923	0.14441	[Unrecognized]
[1244]	CASP6	--->	TRAF1	0.72974	-0.75525	[Recognized]
[1245]	MMP7	<---	HAPLN1	-0.093464	0.097177	[Unrecognized]
[1246]	PRKCA	--->	GRIA4	0.56969	-0.57379	[Recognized]
[1247]	HCK	--->	BCR	0.36363	-0.25336	[Recognized]
[1248]	PRKCB	--->	BTK	0.99524	-0.99198	[Recognized]
[1249]	LCK	--->	NFKBIA	0.68922	-0.65792	[Recognized]
[1250]	CDK2	--->	CDKN1B	0.34497	-0.36687	[Recognized]
[1251]	PRKCA	--->	PEBP1	1.0121	-1.008	[Recognized]
[1252]	CHEK2	--->	AATF	0.3529	-0.3694	[Recognized]
[1253]	MAPK1	--->	RGS19	0.28697	-0.32153	[Recognized]
[1254]	PRSS2	--->	MMP9	0.21298	-0.1922	[Recognized]
[1255]	CASP3	--->	RB1	0.41973	-0.45426	[Recognized]
[1256]	DYRK1A	--->	FOXO1	0.19737	-0.20874	[Recognized]
[1257]	CASP8	--->	PRKCZ	0.12685	-0.1345	[Recognized]
[1258]	MAPK8	--->	MYC	1.0154	-1.0065	[Recognized]
[1259]	SRC	--->	CEACAM1	0.32027	-0.20338	[Recognized]
[1260]	MAPK7	--->	RXRA	0.61286	-0.69248	[Recognized]
[1261]	PRKD1	--->	PLCG1	1.0014	-1.0056	[Recognized]
[1262]	CDK5	--->	APP	0.38256	-0.39431	[Recognized]
[1263]	CASP3	--->	SP1	0.032319	-0.072072	[Recognized]
[1264]	FGR	<---	SRC	-0.016792	0.60459	[Unrecognized]
[1265]	MAPK1	--->	ESR1	0.99834	-1.0079	[Recognized]
[1266]	AKT1	--->	EIF4EBP1	0.49303	-0.34292	[Recognized]
[1267]	CSNK1A1	--->	YWHAQ	0.55799	-0.48053	[Recognized]
[1268]	PRKACA	--->	HAND1	1.5563	-1.5271	[Recognized]
[1269]	CDK5	--->	MAPK10	0.64901	-0.41806	[Recognized]
[1270]	FYN	--->	FYB	0.99663	-1.0021	[Recognized]
[1271]	MAPK1	--->	SREBF1	0.54159	-0.54127	[Recognized]
[1272]	EP300	--->	MYOD1	0.19969	-0.20391	[Recognized]
[1273]	MAP2K2	--->	MAPK3	1.0159	-0.99672	[Recognized]
[1274]	C1RL	?	HP	-0.00064986	-0.00064986	[Unrecognized]
[1275]	RHEBL1	--->	EIF4EBP1	0.31457	-0.28483	[Recognized]
[1276]	LYN	--->	CD79B	1.0799	-1.0399	[Recognized]
[1277]	PRKCA	--->	GJA1	0.22576	-0.23456	[Recognized]
[1278]	CASP6	--->	HTT	0.60022	-0.71121	[Recognized]
[1279]	ST14	<---	HGF	-0.012425	0.00068251	[Unrecognized]
[1280]	CDK5	<--->	CDK16	0.23429	0.23429	[Recognized]
[1281]	GGCX	--->	F9	0.071085	-0.066849	[Recognized]
[1282]	SYK	<---	FCGR2A	-1.0112	1.0048	[Unrecognized]
[1283]	CTSG	--->	SERPIND1	0.11833	-0.082185	[Recognized]
[1284]	PTPN2	--->	PDGFRB	0.11574	-0.12276	[Recognized]
[1285]	CASP3	--->	PKN2	0.066252	-0.14308	[Recognized]
[1286]	PDPK1	--->	RPS6KB2	1.0929	-1.1695	[Recognized]
[1287]	KAT5	--->	AR	1.0146	-1.0053	[Recognized]
[1288]	MAPK1	--->	PPP1R9B	0.62013	-0.64226	[Recognized]
[1289]	F2	<---	F2R	-0.27153	0.28486	[Unrecognized]
[1290]	CSNK2A1	--->	SSRP1	0.56249	-0.55527	[Recognized]

[1291]	PLK1	<---	NINL	-0.32727	0.31052	[Unrecognized]
[1292]	TP53	<---	MDM2	-0.9968	0.9983	[Recognized]
[1293]	EGFR	--->	PLD2	0.99738	-1.0064	[Recognized]
[1294]	ADRBK2	--->	CCR5	0.39714	-0.42229	[Recognized]
[1295]	UBE2I	--->	DAXX	0.24731	-0.2755	[Recognized]
[1296]	IKBKB	--->	IRS1	0.56786	-0.6179	[Recognized]
[1297]	AKT1	--->	HMOX1	0.45236	-0.30551	[Recognized]
[1298]	MAPK10	--->	BCL2L11	0.25961	-0.31312	[Recognized]
[1299]	CSNK2A1	--->	JUN	1.0022	-1.0148	[Recognized]
[1300]	PRKCA	--->	PAM	0.90745	-0.90584	[Recognized]
[1301]	PRKCG	--->	DAB2	0.91797	-0.91615	[Recognized]
[1302]	TGM2	--->	SERPINF2	0.12551	-0.089358	[Recognized]
[1303]	EP300	--->	JUN	0.12142	-0.13136	[Recognized]
[1304]	MARK3	--->	CDC25C	0.070066	-0.081031	[Recognized]
[1305]	MMP3	<---	CCL2	-0.16713	0.18806	[Unrecognized]
[1306]	KAT5	<---	EP300	-0.11551	0.1074	[Recognized]
[1307]	PTPN18	<---	TEC	-0.43908	0.26244	[Recognized]
[1308]	PRKCA	--->	HSPB8	0.93891	-0.93193	[Recognized]
[1309]	MMP1	<---	CCL7	-0.16713	0.18806	[Unrecognized]
[1310]	IKBKB	--->	NFKBIB	1.0006	-1.0011	[Recognized]
[1311]	PRKCE	--->	OPRD1	0.556	-0.62401	[Recognized]
[1312]	MAPK14	--->	ATF2	1.0024	-0.99433	[Recognized]
[1313]	ATR	<---	BRCA1	-0.012028	0.0049915	[Unrecognized]
[1314]	TEC	?	BTK	-0.2242	-0.2242	[Unrecognized]
[1315]	ATM	<---	MDM2	-0.056522	0.043434	[Unrecognized]
[1316]	AKT1	--->	ATXN1	0.12082	-0.098954	[Recognized]
[1317]	MAPKAPK2	--->	YWHAZ	0.4819	-0.50916	[Recognized]
[1318]	PRKG1	--->	GTF2I	1.0565	-1.0486	[Recognized]
[1319]	PRKCA	--->	DNM1	0.28727	-0.25947	[Recognized]
[1320]	KAT5	<---	CREBBP	-0.11551	0.1074	[Recognized]
[1321]	TEC	<---	BMX	-0.20499	-0.055822	[Unrecognized]
[1322]	LYN	--->	DAPP1	1.4511	-1.0668	[Recognized]
[1323]	KAT2B	--->	TAL1	0.21156	-0.21587	[Recognized]
[1324]	PRKACA	--->	CFTR	1.0007	-1.0004	[Recognized]
[1325]	PRKCA	--->	PTPN12	0.27145	-0.27861	[Recognized]
[1326]	F2	--->	SERPINC1	0.057207	-0.040728	[Recognized]
[1327]	NEK9	<--->	NEK7	0.23429	0.23429	[Recognized]
[1328]	ADRBK1	--->	ADRA2A	0.25048	-0.27132	[Recognized]
[1329]	LCK	--->	CD79B	0.99528	-0.99051	[Recognized]
[1330]	CASP7	--->	MAX	0.95167	-1.0606	[Recognized]
[1331]	CSNK2A1	--->	BID	0.77321	-0.7866	[Recognized]
[1332]	KAT5	--->	HIST2H4A	0.15564	-0.12976	[Recognized]
[1333]	PRKCA	--->	LCK	0.16189	-0.15354	[Recognized]
[1334]	PTPN11	--->	TEK	0.014995	-0.021985	[Recognized]
[1335]	PRKCD	--->	KLF5	0.49984	-0.49851	[Recognized]
[1336]	PTK2	<---	TRIO	-0.0222	-0.008627	[Unrecognized]
[1337]	ABL1	<---	PRKD1	-0.17642	0.11013	[Unrecognized]
[1338]	GSK3B	--->	MITF	1.0118	-0.98821	[Recognized]
[1339]	LCK	--->	DAPP1	1.2932	-0.84987	[Recognized]
[1340]	PRKACA	--->	PDE4D	0.99264	-1.0111	[Recognized]
[1341]	JAK2	--->	GTF2I	0.29776	-0.17667	[Recognized]
[1342]	PTPN11	--->	CSF2RB	0.99989	-1.0072	[Recognized]
[1343]	CSNK2A1	--->	HNRNPC	0.72994	-0.75755	[Recognized]
[1344]	CAMK2A	--->	MAPT	0.68291	-0.68964	[Recognized]
[1345]	PDPK1	--->	PKN2	0.68989	-0.69757	[Recognized]
[1346]	CSNK2A1	--->	HDAC3	0.68838	-0.71414	[Recognized]
[1347]	AKT1	--->	PPARGC1B	0.1037	-0.081786	[Recognized]
[1348]	CDK4	--->	RBL1	0.46185	-0.47579	[Recognized]
[1349]	CHUK	<---	PRKCQ	-0.40526	0.42813	[Recognized]
[1350]	MAPK1	--->	RPS6KA1	1.002	-1.0109	[Recognized]
[1351]	CASP3	--->	MEF2A	0.17117	-0.20473	[Recognized]
[1352]	ATF2	<---	HIST2H2BE	-0.33758	0.33912	[Unrecognized]
[1353]	BMP1	<---	COL5A1	-0.041937	0.035051	[Unrecognized]
[1354]	GSK3B	--->	NOTCH2	0.99811	-1.0133	[Recognized]
[1355]	PTK2	--->	PXN	1.009	-0.99895	[Recognized]
[1356]	SIK1	<---	CRTC2	-0.075794	0.069802	[Unrecognized]
[1357]	CDK4	--->	SMAD3	1.012	-1.0074	[Recognized]
[1358]	ZAP70	--->	LAT	0.99842	-1.0126	[Recognized]
[1359]	PRKCD	--->	PTPN22	0.25179	-0.25663	[Recognized]
[1360]	KLK3	--->	PLG	0.0012258	-0.0091243	[Recognized]
[1361]	PLG	<---	PCOLCE	-0.13445	0.14183	[Unrecognized]
[1362]	MMP2	<---	A2M	-0.11161	0.132	[Unrecognized]
[1363]	SRC	--->	CDKN1B	0.36964	-0.33668	[Recognized]
[1364]	MAPK14	--->	EEA1	0.29074	-0.46077	[Recognized]
[1365]	PRKCB	--->	DAB2	0.91797	-0.91615	[Recognized]
[1366]	CSNK2A1	--->	L1CAM	0.088336	-0.11794	[Recognized]

[1367]	PRKACA	--->	ABCA1	0.16549	-0.18176	[Recognized]
[1368]	PRKACA	--->	MC4R	0.21381	-0.23401	[Recognized]
[1369]	PRKCQ	--->	WIPF1	0.9992	-1.0006	[Recognized]
[1370]	AKT1	--->	NR4A1	1.0083	-1.0039	[Recognized]
[1371]	PTPRA	--->	FYN	-0.050335	-0.080843	[Recognized]
[1372]	FGFR3	--->	STAT3	0.10807	-0.14294	[Recognized]
[1373]	PRKCZ	--->	RELA	1.0022	-0.99892	[Recognized]
[1374]	MAP2K4	--->	MAPK10	0.64901	-0.41806	[Recognized]
[1375]	FURIN	--->	LRP1	0.049899	-0.062083	[Recognized]
[1376]	TSC2	<---	RPS6KA1	-1.0083	1.002	[Recognized]
[1377]	PDCD6IP	<---	SRC	-0.89191	1.0053	[Recognized]
[1378]	PAK2	--->	MYLK	0.53897	-0.35021	[Recognized]
[1379]	PRKCA	--->	GRIN2B	0.22109	-0.2311	[Recognized]
[1380]	JAK1	--->	IRS1	1.0117	-1.0143	[Recognized]
[1381]	KHDRBS1	<---	PTK6	-0.99763	0.99942	[Recognized]
[1382]	PTPN12	--->	PTK2	0.29598	-0.3482	[Recognized]
[1383]	ADAMTS1	--->	VCAN	5.5806e-005	-0.012914	[Recognized]
[1384]	INSR	<---	CALM3	-0.1135	0.10567	[Unrecognized]
[1385]	PRKCA	--->	TOP2A	0.89006	-0.88857	[Recognized]
[1386]	BCAN	--->	MMP3	0.022421	-0.030194	[Unrecognized]
[1387]	CDC25C	<---	CDK1	-0.67632	0.69298	[Unrecognized]
[1388]	PRKCA	--->	MYOD1	1.0168	-1.0076	[Recognized]
[1389]	HAT1	--->	SMAD7	0.37619	-0.33502	[Recognized]
[1390]	PRKACA	<---	PTPN12	-0.46863	0.46317	[Unrecognized]
[1391]	PRKCA	--->	KIT	1.0004	-0.99338	[Recognized]
[1392]	RPS6KA5	--->	ETV1	0.39908	-0.61069	[Recognized]
[1393]	CASP8	--->	RIPK1	1.006	-0.99988	[Recognized]
[1394]	CSNK2A2	--->	IL16	0.62013	-0.64226	[Recognized]
[1395]	GSK3B	--->	LRP6	0.91677	-0.91735	[Recognized]
[1396]	PRKCB	--->	GRIN2B	0.22109	-0.2311	[Recognized]
[1397]	PDPK1	--->	AKT2	0.99822	-0.99934	[Recognized]
[1398]	MAP2K6	--->	STAT4	1.0227	-1.0418	[Recognized]
[1399]	INSR	--->	IRS2	0.2388	-0.24842	[Recognized]
[1400]	PLK1	<---	WEE1	0.0029423	0.19171	[Unrecognized]
[1401]	MAPK8	--->	JUNB	1.0022	-1.0148	[Recognized]
[1402]	GGCX	--->	F2	0.11438	-0.09847	[Recognized]
[1403]	PDK2	--->	PDHA1	1.0004	-0.99606	[Recognized]
[1404]	CSNK2A1	--->	SRF	0.37272	-0.3873	[Recognized]
[1405]	TYK2	--->	STAT1	0.12795	-0.18775	[Recognized]
[1406]	CSNK2A2	--->	TCF7L2	1.0076	-0.99098	[Recognized]
[1407]	CASP3	--->	TRAF1	0.72974	-0.75525	[Recognized]
[1408]	PPM1B	--->	MSN	0.10633	-0.11549	[Recognized]
[1409]	PRKCD	--->	AKT3	0.11908	0.046737	[Recognized]
[1410]	MAP2K2	--->	CASP9	0.66416	-0.42184	[Recognized]
[1411]	MAPKAPK2	--->	CREB1	0.99947	-1.0056	[Recognized]
[1412]	PRKACA	<---	CETN1	-1.0113	1.0111	[Unrecognized]
[1413]	PRKACA	--->	GRIA4	0.66332	-0.67945	[Recognized]
[1414]	PRKCA	--->	GJB1	0.55904	-0.56302	[Recognized]
[1415]	MMP9	<---	CXCL1	-0.16713	0.18806	[Unrecognized]
[1416]	PRKACA	--->	NOS1	0.48839	-0.49502	[Recognized]
[1417]	CCND2	--->	RB1	0.99958	-1.0044	[Recognized]
[1418]	PDK1	--->	PDHA1	1.0004	-0.99606	[Recognized]
[1419]	CHEK1	--->	MDM4	0.091873	-0.11293	[Recognized]
[1420]	SSH1	--->	CFL1	0.3315	-0.32562	[Recognized]
[1421]	YES1	--->	CDKN1B	0.3974	-0.34695	[Recognized]
[1422]	EGFR	--->	GRB2	1.1215	-1.1271	[Recognized]
[1423]	PRKG2	--->	PLCB3	0.62876	-0.62602	[Recognized]
[1424]	F11	--->	HGF	0.0012258	-0.0091243	[Recognized]
[1425]	MDM2	<---	ADRBK1	-0.28847	0.2659	[Unrecognized]
[1426]	F2	<---	HGFAC	-0.0096131	-0.0087417	[Unrecognized]
[1427]	CAMK1	--->	ATF1	1.2086	-1.1667	[Recognized]
[1428]	PIAS1	--->	SATB2	0.067311	-0.065506	[Recognized]
[1429]	AURKA	--->	CDC25B	0.69298	-0.67632	[Recognized]
[1430]	EGFR	--->	MUC1	0.1949	-0.20625	[Recognized]
[1431]	PRKACA	--->	KCNJ12	0.73999	-0.76275	[Recognized]
[1432]	LYN	--->	CD79A	1.0799	-1.0399	[Recognized]
[1433]	PRKCD	--->	STAT3	0.99971	-0.99944	[Recognized]
[1434]	KLK2	--->	SERPINF2	0.11833	-0.082185	[Recognized]
[1435]	CSNK2A1	--->	GYS1	1.0067	-1.0067	[Recognized]
[1436]	CDK5	--->	AMPH	0.2474	-0.26764	[Recognized]
[1437]	FLT1	--->	PTPN11	0.085004	-0.0095962	[Unrecognized]
[1438]	CASP3	--->	CDKN1A	0.38241	-0.45789	[Recognized]
[1439]	PTPN2	--->	SHC1	0.34818	-0.56392	[Recognized]
[1440]	CASP9	<---	DCC	-0.20326	0.19438	[Unrecognized]
[1441]	PPP2CB	--->	MID1	0.094726	-0.10622	[Recognized]
[1442]	PRKCD	--->	GSK3A	0.26908	-0.016385	[Recognized]

[1443]	MAPK8	--->	STAT3	1.0077	-1.0166	[Recognized]
[1444]	CSNK1D	--->	CDK5	0.9966	-0.99147	[Recognized]
[1445]	PAK1	<---	LIMK1	-0.08975	0.17985	[Unrecognized]
[1446]	AKT1	--->	PDE3B	0.19946	-0.15291	[Recognized]
[1447]	CSNK1A1	--->	BID	0.59045	-0.50796	[Recognized]
[1448]	CAMK2A	--->	NOS1	0.30424	-0.31365	[Recognized]
[1449]	CSNK1A1	--->	PSEN2	0.56845	-0.48873	[Recognized]
[1450]	PRKD1	--->	KIDINS220	0.41912	-0.43688	[Recognized]
[1451]	RET	--->	MAPK8	0.20607	-0.24517	[Recognized]
[1452]	PTPRC	--->	INSR	0.032808	-0.062922	[Recognized]
[1453]	AKT1	--->	CDKN1A	0.99717	-0.99814	[Recognized]
[1454]	MAPK14	--->	HNF4A	0.1013	-0.17053	[Recognized]
[1455]	MAPK10	--->	HNRNPK	0.43781	-0.56248	[Recognized]
[1456]	AKT1	--->	MDM4	0.41208	-0.39989	[Recognized]
[1457]	SRC	--->	VCL	0.88389	-0.77281	[Recognized]
[1458]	CDK7	--->	PCGF6	0.5807	-0.60171	[Recognized]
[1459]	SRC	--->	WWOX	0.55602	-0.49297	[Recognized]
[1460]	PRKCA	--->	NOS1	0.1484	-0.16067	[Recognized]
[1461]	LYN	--->	PLCG2	0.59106	-0.8605	[Recognized]
[1462]	PTPN11	--->	STAT5A	1.0012	-1.0146	[Recognized]
[1463]	PIM1	--->	CDKN1A	0.51838	-0.55156	[Recognized]
[1464]	FGFR4	--->	STAT1	-0.026735	-0.027648	[Recognized]
[1465]	CDK1	--->	RPS6KB1	1.002	-1.0109	[Recognized]
[1466]	CASP3	--->	PAK2	1.002	-0.99322	[Recognized]
[1467]	CSNK2A1	--->	RGS19	0.28697	-0.32153	[Recognized]
[1468]	PRKCA	--->	CD163	0.92534	-0.92282	[Recognized]
[1469]	INSR	<---	CALM1	-0.1135	0.10567	[Unrecognized]
[1470]	PRKCG	--->	ADRBK1	0.23691	0.014495	[Recognized]
[1471]	PDPK1	--->	SGK3	0.66384	-0.71595	[Recognized]
[1472]	EP300	--->	ABL1	0.0167	-0.034052	[Recognized]
[1473]	MAPK1	--->	SMAD2	1.012	-1.0074	[Recognized]
[1474]	MAPK3	--->	LIPE	0.53776	-0.55596	[Recognized]
[1475]	SGK1	--->	CREB1	1.0082	-0.98976	[Recognized]
[1476]	JAK1	--->	IL2RB	1.0065	-1.0011	[Recognized]
[1477]	SIK2	<---	CRTC2	-0.075794	0.069802	[Unrecognized]
[1478]	RPS6KA3	--->	CREB1	1.0082	-0.98976	[Recognized]
[1479]	PTPN11	--->	PTK2B	0.99625	-0.99764	[Recognized]
[1480]	CDC25A	<---	RAF1	-0.05239	0.036016	[Unrecognized]
[1481]	HCK	--->	WAS	0.99511	-1.0011	[Recognized]
[1482]	CAMKK2	--->	CAMK1	0.83082	-0.43719	[Recognized]
[1483]	CASP6	--->	PSEN1	0.58173	-0.69269	[Recognized]
[1484]	FYN	--->	SNX26	0.4469	-0.38316	[Recognized]
[1485]	CASP10	--->	CASP3	0.99569	-0.9907	[Recognized]
[1486]	MYC	<---	GSK3B	-1.0065	1.0154	[Recognized]
[1487]	MAP3K10	--->	CEACAM1	0.13136	-0.14266	[Recognized]
[1488]	KAT2B	--->	ETV1	0.20937	-0.20573	[Recognized]
[1489]	LCK	--->	STAT1	0.324	-0.25356	[Recognized]
[1490]	EGFR	--->	EPB41	0.10938	-0.11225	[Recognized]
[1491]	PRKCE	--->	GRM5	0.62582	-0.69315	[Recognized]
[1492]	ILK	--->	PPP1R12A	0.081762	-0.072065	[Recognized]
[1493]	CSNK2A2	--->	GTF2A1	0.69606	-0.7619	[Recognized]
[1494]	PRKCA	--->	TP53	0.39209	-0.39877	[Recognized]
[1495]	CASP1	<---	PSEN2	-0.048154	0.056436	[Unrecognized]
[1496]	MAP3K8	<--->	MAP3K14	0.23429	0.23429	[Recognized]
[1497]	DYRK1A	--->	SF3B1	0.39838	-0.41811	[Recognized]
[1498]	CSNK1A1	--->	YWHAZ	0.55799	-0.48053	[Recognized]
[1499]	CASP3	--->	PSEN1	0.58173	-0.69269	[Recognized]
[1500]	CDK3	--->	KIR3DL1	0.11063	-0.11082	[Recognized]
[1501]	MMP26	<---	IGFBP1	-0.03539	0.071188	[Unrecognized]
[1502]	PRKACA	<---	ADRBK1	-0.049315	0.28927	[Unrecognized]
[1503]	MAPK1	--->	LIPE	0.53776	-0.55596	[Recognized]
[1504]	AKT1	--->	CREB1	0.4893	-0.45124	[Recognized]
[1505]	MAPK3	--->	ARRB1	0.42184	-0.44306	[Recognized]
[1506]	CAMK2B	--->	PLCB3	0.41213	-0.4132	[Recognized]
[1507]	HCK	--->	PLCG1	0.81503	-1.0097	[Recognized]
[1508]	CTSD	--->	IGFBP4	0.41796	-0.40802	[Recognized]
[1509]	CAPN1	--->	BCL2L1	0.32381	-0.33086	[Recognized]
[1510]	EGFR	--->	ERBB2	0.0023711	-9.0961e-005	[Recognized]
[1511]	MAPK8	--->	JDP2	0.97462	-1.029	[Recognized]
[1512]	PRKCZ	<---	AKT3	-0.0047278	0.082925	[Unrecognized]
[1513]	PRKACA	--->	FOS	0.53871	-0.68783	[Recognized]
[1514]	UBE2I	--->	HDAC1	0.10139	-0.085985	[Recognized]
[1515]	ST8SIA4	<---	NCAM1	-0.085249	0.094891	[Unrecognized]
[1516]	PRKG1	--->	TRPC3	0.62998	-0.63462	[Recognized]
[1517]	MAPK1	--->	TP53	1.0002	-0.99962	[Recognized]
[1518]	PTPN11	--->	SELE	0.58187	-0.48449	[Recognized]

[1519]	SRC	--->	GJA1	0.24586	-0.22975	[Recognized]
[1520]	CSNK2A1	--->	STMN1	1.0058	-0.99021	[Recognized]
[1521]	CDK9	--->	TP53	1.0002	-0.99962	[Recognized]
[1522]	CSK	<--->	FYN	0.37153	0.37153	[Recognized]
[1523]	ELANE	--->	SERPINF2	0.11833	-0.082185	[Recognized]
[1524]	GSK3B	--->	CTNND1	0.31524	-0.34547	[Recognized]
[1525]	BCR	--->	MLLT4	0.053585	-0.053555	[Recognized]
[1526]	PRKCA	--->	PTPN6	0.6319	-0.62682	[Recognized]
[1527]	IGFBP3	<---	PLG	-0.013006	0.028162	[Recognized]
[1528]	ATM	<---	H2AFX	-0.22572	0.2157	[Unrecognized]
[1529]	PTPN6	--->	CSF2RB	0.99989	-1.0072	[Recognized]
[1530]	CDC42BPA	--->	LIMK2	0.011701	-0.014076	[Recognized]
[1531]	CAMK2A	--->	ETS1	0.68344	-0.68792	[Recognized]
[1532]	BMPR1B	--->	STAMBP	0.0012705	-0.029928	[Recognized]
[1533]	CASP8	<---	CFLAR	-0.99651	1.0068	[Unrecognized]
[1534]	CASP8	--->	PSEN2	0.56121	-0.70272	[Recognized]
[1535]	CAMK1	--->	HDAC5	0.4123	-0.45761	[Recognized]
[1536]	AKT1	--->	CDKN1B	1.0151	-1.0131	[Recognized]
[1537]	CHUK	?	IKBKB	-0.14626	-0.14626	[Unrecognized]
[1538]	CSNK1E	--->	BID	0.72833	-0.59006	[Recognized]
[1539]	ADRBK1	--->	OPRD1	0.39714	-0.42229	[Recognized]
[1540]	AKT2	--->	FOXO4	0.99774	-1.0046	[Recognized]
[1541]	WEE1	<--->	CDK1	0.23429	0.23429	[Recognized]
[1542]	HIPK3	--->	FADD	0.18204	-0.46216	[Recognized]
[1543]	PTPN2	<---	SRC	-0.37818	0.26821	[Unrecognized]
[1544]	GRK5	--->	ADRB2	0.21152	-0.27051	[Recognized]
[1545]	ABL1	--->	WASL	0.057901	-0.10978	[Recognized]
[1546]	CASP7	--->	MEF2D	0.10147	-0.12731	[Recognized]
[1547]	PTPN11	--->	SIRPA	0.97001	-0.82769	[Recognized]
[1548]	PRKCA	<---	PRKG1	-0.4183	0.6366	[Unrecognized]
[1549]	MAPK1	--->	ARRB1	0.42184	-0.44306	[Recognized]
[1550]	LCK	--->	PRKCQ	0.073791	-0.063362	[Recognized]
[1551]	CAPN1	--->	F2RL1	0.16203	-0.16251	[Recognized]
[1552]	AURKB	--->	CENPA	1.0832	-1.0289	[Recognized]
[1553]	CASP3	--->	MDM2	0.049195	-0.084734	[Recognized]
[1554]	PRKACA	--->	PPP1R8	0.7025	-0.72853	[Recognized]
[1555]	PPM1A	--->	GRM3	0.76481	-0.74426	[Recognized]
[1556]	SRC	--->	INPPL1	0.49697	-0.27975	[Recognized]
[1557]	PLK1	<---	PKMYT1	0.0029423	0.19171	[Unrecognized]
[1558]	MMP3	<---	DCN	-0.18966	0.20515	[Unrecognized]
[1559]	CREBBP	--->	CTNNB1	0.019033	-0.032118	[Recognized]
[1560]	CSNK2A1	--->	IGFBP3	0.7558	-0.78174	[Recognized]
[1561]	PTPN12	--->	PSTPIP1	0.30035	-0.30748	[Recognized]
[1562]	PRKCA	--->	GRIA2	0.56969	-0.57379	[Recognized]
[1563]	CHUK	--->	RELA	1.0019	-0.99319	[Recognized]
[1564]	SRC	--->	SPTAN1	0.25187	-0.12836	[Recognized]
[1565]	SIK2	--->	IRS1	0.5492	-0.60947	[Recognized]
[1566]	CDK1	<---	PTPN2	-1.009	1.0061	[Unrecognized]
[1567]	TNK2	<---	FYN	-0.013149	0.0028058	[Recognized]
[1568]	CASP3	--->	MAPK8IP3	0.12331	-0.15875	[Recognized]
[1569]	ADAM17	--->	NOTCH1	0.99876	-1.0036	[Recognized]
[1570]	EGFR	--->	CRK	0.99929	-0.99608	[Recognized]
[1571]	CAMK2B	--->	ETS1	0.68344	-0.68792	[Recognized]
[1572]	ROCK1	--->	PPP1R12A	0.99868	-0.99668	[Recognized]
[1573]	AR	<---	KAT2B	-0.99994	1.0101	[Recognized]
[1574]	MAPK3	--->	CASP9	0.99863	-0.99771	[Recognized]
[1575]	MAPK1	--->	TSC2	1.0125	-1.0077	[Recognized]
[1576]	SRC	--->	TERT	0.13979	-0.1366	[Recognized]
[1577]	CSNK2A1	--->	GMFB	0.75717	-0.78322	[Recognized]
[1578]	PAK1	--->	NF2	0.23985	-0.14532	[Recognized]
[1579]	UBE2I	--->	SMAD4	0.76473	-0.70088	[Recognized]
[1580]	CELA1	--->	LPA	0.0012258	-0.0091243	[Recognized]
[1581]	PPP3CB	--->	NFATC2	0.99656	-0.99518	[Recognized]
[1582]	BRSK2	--->	CDC25C	0.41582	-0.41122	[Recognized]
[1583]	CASP7	--->	CDC42	0.36319	-0.47633	[Recognized]
[1584]	MAPK14	--->	NCOA3	0.22463	-0.24638	[Recognized]
[1585]	CASP2	--->	BID	0.067368	-0.028077	[Recognized]
[1586]	CASP3	--->	BCAR1	0.10829	-0.13063	[Recognized]
[1587]	PRKAA1	--->	ACACA	0.14518	-0.16304	[Recognized]
[1588]	PRKACA	--->	LRP1	0.22111	-0.23243	[Recognized]
[1589]	MMP3	<---	CCL13	-0.16713	0.18806	[Unrecognized]
[1590]	PRKACA	--->	SLC2A2	1.0445	-1.078	[Recognized]
[1591]	AKT1	--->	BRAF	0.65966	-0.67061	[Recognized]
[1592]	PAK1	--->	ARPC1B	0.69245	-0.41336	[Recognized]
[1593]	AR	<---	EP300	-0.99484	0.99704	[Recognized]
[1594]	KLKB1	--->	HGFAC	-0.0039613	-0.0063334	[Recognized]

```
[1595]  SRC      --->    GRIN2B  0.24276 -0.22786        [Recognized]
[1596]  CSNK2A1  --->    SP1     0.12886 -0.14656        [Recognized]
[1597]  PRKAA1   --->    RAF1    0.26426 -0.39064        [Recognized]
[1598]  CSNK1D   --->    TP53    0.361   -0.31701        [Recognized]
[1599]  SRC      --->    TXK     0.60459 -0.016792       [Recognized]
[1600]  PTPN6    <---    EGFR    -0.25411        0.37037 [Unrecognized]
[1601]  CAMKK1   --->    CAMK4   0.83082 -0.43719        [Recognized]
[1602]  MAPK14   --->    NFATC4  0.2651  -0.3326 [Recognized]
[1603]  RPS6KA1  --->    L1CAM   0.03878 -0.13396        [Recognized]
[1604]  CDK1     --->    MAPT    0.99929 -1.0027 [Recognized]
[1605]  ACAN     --->    MMP19   0.022421        -0.030194       [Unrecognized]
[1606]  CDC25A   <---    CDK2    -0.67632        0.69298 [Unrecognized]
[1607]  MAPK14   --->    SMAD3   1.0053  -0.99469        [Recognized]
[1608]  PRKG2    --->    LASP1   0.53854 -0.53556        [Recognized]
[1609]  MAPK1    <---    PAK1    -1.3014 1.4008  [Unrecognized]
[1610]  FYN      --->    ITPR1   0.99821 -1.0037 [Recognized]
[1611]  CREBBP   --->    RELA    0.038481        -0.050014       [Recognized]
[1612]  CDK1     --->    IL16    0.62013 -0.64226        [Recognized]
[1613]  PTPN12   --->    WAS     0.99884 -0.98948        [Recognized]
[1614]  EP300    --->    KLF5    0.11387 -0.12188        [Recognized]
[1615]  TXK      --->    LCP2    1.2119  -0.99119        [Recognized]
[1616]  CSNK2A1  --->    EIF4EBP1        1.0012  -1.0031 [Recognized]
[1617]  PRKACA   --->    HMGCR   0.54046 -0.56003        [Recognized]
[1618]  CSNK2A1  --->    CDH1    0.39746 -0.41519        [Recognized]
[1619]  BTK      <---    BMX     -0.20499        -0.055822       [Unrecognized]
[1620]  CAPN1    --->    SPTAN1  0.062177        -0.07387        [Recognized]
[1621]  CLK2     <---    PTPN1   -1.009  1.0061  [Unrecognized]
[1622]  SRC      --->    PAK2    0.98977 -0.89147        [Recognized]
[1623]  MAPK3    --->    STMN1   1.0058  -0.99021        [Recognized]
[1624]  CSNK2A2  <---    PTPRC   -0.35814        0.35828 [Unrecognized]
[1625]  PRKACA   --->    CREM    0.96654 -0.95401        [Recognized]
[1626]  CDC25B   <---    CDK1    -0.67632        0.69298 [Recognized]
[1627]  CSNK2A2  <---    PTPN1   -1.009  1.0061  [Unrecognized]
[1628]  RET      --->    PTK2    0.27422 -0.092834       [Recognized]
[1629]  PAK3     --->    RAF1    0.65894 -0.56404        [Recognized]
[1630]  PAK7     --->    BAD     0.40593 -0.40769        [Recognized]
[1631]  ARAF     --->    MAP2K1  0.9856  -1.0047 [Recognized]
[1632]  CASP3    --->    MAPT    0.50832 -0.57833        [Recognized]
[1633]  GRK6     --->    LTB4R   0.31971 -0.40764        [Recognized]
[1634]  UBE2I    --->    SALL1   0.35582 -0.35511        [Recognized]
[1635]  CHEK1    --->    CDC25A  0.69298 -0.67632        [Recognized]
[1636]  CAMK2A   --->    SYNGAP1 0.21355 -0.21755        [Recognized]
[1637]  PRKCD    --->    BDKRB2  0.45098 -0.45062        [Recognized]
[1638]  MAP2K6   --->    MAPK13  0.64901 -0.41806        [Recognized]
[1639]  PRKCD    --->    TAGLN   0.78277 -0.77308        [Recognized]
[1640]  KAT2B    --->    CIITA   0.1123  -0.10992        [Recognized]
[1641]  MMP9     <---    CXCL5   -0.16713        0.18806 [Unrecognized]
[1642]  PRKACA   --->    APC     0.13984 -0.15381        [Recognized]
[1643]  CSNK2A1  --->    PTEN    1.0172  -1.002  [Recognized]
[1644]  PPM1A    <---    PRKCD   -0.39783        0.39982 [Unrecognized]
[1645]  FYN      <--->   TXK     0.37153 0.37153 [Recognized]
[1646]  PRKAA2   --->    ACACA   0.14518 -0.16304        [Recognized]
[1647]  MMP9     <---    TFPI    -0.1877 0.20851 [Unrecognized]
[1648]  TSSK4    --->    CREB1   0.96654 -0.95401        [Recognized]
[1649]  RPS6KA5  <---    STK11   -0.74727        0.78966 [Unrecognized]
[1650]  CSNK2A1  <---    HIF1A   -0.84006        0.84197 [Unrecognized]
[1651]  KAT2B    --->    TP53    0.17897 -0.18472        [Recognized]
[1652]  GALNT1   <---    PTPRF   -0.075229       0.068107        [Unrecognized]
[1653]  KDM2A    --->    HIST2H3C        0.28796 -0.23643        [Recognized]
[1654]  F2       --->    PROS1   0.005203        -0.0080779      [Recognized]
[1655]  CDK1     --->    NUP210  0.64216 -0.66848        [Recognized]
[1656]  MAP3K14  --->    NFKB2   0.07198 -0.092566       [Recognized]
[1657]  LCK      --->    ESR1    0.53046 -0.4838 [Recognized]
[1658]  LYN      --->    CDKN1B  0.3974  -0.34695        [Recognized]
[1659]  RPS6KA1  --->    NFKBIA  0.53101 -0.68424        [Recognized]
[1660]  PRKCA    --->    GSK3A   0.22826 0.12473 [Recognized]
[1661]  CSNK1E   --->    PER2    0.72042 -0.58384        [Recognized]
[1662]  LCK      --->    PLCG2   0.1769  -0.32378        [Recognized]
[1663]  PRKACA   --->    ATF1    1.2086  -1.1667 [Recognized]
[1664]  PRKACA   --->    LCP1    0.62336 -0.63109        [Recognized]
[1665]  CSK      <---    SRC     -0.016792       0.60459 [Unrecognized]
[1666]  PTPN11   --->    IRS1    0.58189 -0.41492        [Recognized]
[1667]  ACAN     --->    MMP3    0.022421        -0.030194       [Unrecognized]
[1668]  PRKCZ    --->    NUMB    0.15905 -0.17265        [Recognized]
[1669]  FYN      --->    CNN3    1.0728  -0.91195        [Recognized]
[1670]  RPS6KA2  --->    BAD     0.58958 -0.91274        [Recognized]
```

[1671]	RPS6KA5	--->	BAD	0.58958	-0.91274	[Recognized]
[1672]	MAPK7	--->	GJA1	0.99636	-0.99797	[Recognized]
[1673]	LYN	--->	CD19	1.0564	-1.0123	[Recognized]
[1674]	LCK	--->	EZR	0.33022	-0.2959	[Recognized]
[1675]	PRKACA	--->	GSK3A	0.16704	0.057781	[Recognized]
[1676]	PAK1	--->	FOXO1	0.22002	-0.19059	[Recognized]
[1677]	CSNK1A1	--->	NFATC3	0.37393	-0.34882	[Recognized]
[1678]	CASP7	--->	RASA1	-0.026536	-0.078902	[Recognized]
[1679]	SRC	--->	ATP2B4	0.15418	-0.15631	[Recognized]
[1680]	PRKCH	--->	ITGB2	0.049804	-0.071081	[Recognized]
[1681]	JAK1	--->	STAT2	1.0942	-1.0882	[Recognized]
[1682]	CAMK2A	--->	DLG1	0.13725	-0.15924	[Recognized]
[1683]	CSNK1A1	--->	FADD	0.29197	-0.30689	[Recognized]
[1684]	PTPRC	--->	SKAP1	0.14419	-0.15643	[Recognized]
[1685]	MAPKAPK2	--->	BAG2	0.59959	-0.62486	[Recognized]
[1686]	CSNK2A1	<---	PTPRC	-0.35814	0.35828	[Unrecognized]
[1687]	SIRT1	<---	EP300	-0.027305	0.014726	[Unrecognized]
[1688]	CSNK2A1	--->	CTNNB1	0.99917	-0.99713	[Recognized]
[1689]	MAPK1	--->	TCF3	0.99488	-0.99939	[Recognized]
[1690]	PKMYT1	<--->	CDK1	0.23429	0.23429	[Recognized]
[1691]	MAP2K7	<--->	MAPK8	0.23429	0.23429	[Recognized]
[1692]	NFKBIA	<---	PTPN13	-0.17394	0.16053	[Recognized]
[1693]	AKT1	--->	MAP2K4	1.0168	-1.0071	[Recognized]
[1694]	PTPN1	--->	STAT5B	1.0055	-1.005	[Recognized]
[1695]	CAMK2G	<---	MYLK	0.12121	0.16206	[Unrecognized]
[1696]	LYN	--->	PPP1R8	1.0644	-0.90362	[Recognized]
[1697]	ATR	<---	CHEK1	-0.15963	0.14398	[Unrecognized]
[1698]	CDK5	--->	PPP1R1B	1.0061	-1.0155	[Recognized]
[1699]	PDPK1	--->	RPS6KB1	1.0929	-1.1695	[Recognized]
[1700]	MKNK1	--->	EIF4E	0.72573	-0.75265	[Recognized]
[1701]	ACVR1B	--->	SMAD2	1.0115	-1.0087	[Recognized]
[1702]	MAPKAPK2	--->	ETV1	0.7856	-0.78667	[Recognized]
[1703]	FER	--->	CTNNB1	0.078601	-0.092823	[Recognized]
[1704]	AKT1	--->	XIAP	0.30869	-0.23198	[Recognized]
[1705]	PRKCB	--->	PEBP1	1.0121	-1.008	[Recognized]
[1706]	LCK	--->	ZAP70	1.0002	-0.98599	[Recognized]
[1707]	MAPK1	--->	AR	1.2465	-1.2513	[Recognized]
[1708]	CDK1	--->	BIRC5	0.65723	-0.69965	[Recognized]
[1709]	ATM	<---	NBN	-0.22664	0.21656	[Unrecognized]
[1710]	PLAT	--->	PLG	-0.0087417	-0.0096131	[Recognized]
[1711]	PRKCB	--->	GJA1	0.22576	-0.23456	[Recognized]
[1712]	PLK1	--->	MYT1	0.1704	-0.18815	[Recognized]
[1713]	PRKACA	--->	CACNB2	0.72893	-0.75441	[Recognized]
[1714]	PRKCA	--->	DGKD	0.43581	-0.10826	[Recognized]
[1715]	PRKACA	<---	MAP3K3	-0.99622	0.99233	[Unrecognized]
[1716]	GSK3B	--->	CDH1	0.39746	-0.41519	[Recognized]
[1717]	TP53	<---	FBXO11	-0.39539	0.40666	[Recognized]
[1718]	CSNK2A1	--->	CAV1	0.70376	-0.73589	[Recognized]
[1719]	AKT1	--->	PTPN1	0.21859	-0.068869	[Recognized]
[1720]	PAK1	--->	BAD	1.001	-0.99724	[Recognized]
[1721]	AKT1	--->	IRAK1	0.19598	-0.16806	[Recognized]
[1722]	JAK2	--->	STAT1	1.0102	-0.99843	[Recognized]
[1723]	GSK3B	--->	HNRNPD	0.72994	-0.75755	[Recognized]
[1724]	IKBKG	<---	IKBKB	-0.4146	0.27526	[Recognized]
[1725]	CASP3	--->	IL16	0.59353	-0.70295	[Recognized]
[1726]	PLG	<---	F2R	-0.27153	0.28486	[Unrecognized]
[1727]	CSNK1D	--->	BACE1	0.69091	-0.55457	[Recognized]
[1728]	PRKCQ	--->	IRS1	0.86592	-0.78274	[Recognized]
[1729]	MMP1	<---	IGFBP3	-0.15977	0.18055	[Unrecognized]
[1730]	CTSG	--->	AGT	0.11833	-0.082185	[Recognized]
[1731]	CDK2	<---	PTPN2	-1.009	1.0061	[Unrecognized]
[1732]	EP300	--->	APEX1	0.18213	-0.18646	[Recognized]
[1733]	CSNK2A1	--->	PSEN2	0.67463	-0.70045	[Recognized]
[1734]	PDIK1L	<---	TNF	-0.059898	0.062342	[Unrecognized]
[1735]	GSK3B	--->	TP53	1.0002	-0.99962	[Recognized]
[1736]	NFKBIA	<---	IKBKG	-0.33406	0.3181	[Recognized]
[1737]	PLK3	--->	CDC25C	0.33126	-0.32604	[Recognized]
[1738]	EP300	--->	RELA	0.038481	-0.050014	[Recognized]
[1739]	PPM1B	--->	CDK2	1.0075	-0.9963	[Recognized]
[1740]	MAPK1	--->	GJA1	0.49049	-0.48658	[Recognized]
[1741]	BLK	--->	FCGR2A	0.11748	0.015127	[Recognized]
[1742]	INSR	<---	ADRB2	-0.11081	0.10006	[Unrecognized]
[1743]	EGFR	--->	CALM3	0.34959	-0.34973	[Recognized]
[1744]	CSNK2A2	--->	PTEN	1.0172	-1.002	[Recognized]
[1745]	CASP6	--->	SATB1	0.20813	-0.25733	[Recognized]
[1746]	CSNK2A1	--->	UBE2R2	0.2372	-0.25823	[Recognized]

[1747]	PRKACA	--->	GNA13	0.52385	-0.56835	[Recognized]
[1748]	FNTA	<---	PTP4A3	-1.3382	1.3467	[Unrecognized]
[1749]	PRKCG	--->	TOP2A	0.89006	-0.88857	[Recognized]
[1750]	CASP3	--->	PXN	0.4894	-0.56695	[Recognized]
[1751]	AURKB	--->	RACGAP1	0.23631	-0.25262	[Recognized]
[1752]	DAPK3	--->	CDKN1A	0.51838	-0.55156	[Recognized]
[1753]	CASP9	<---	CAPN2	-0.15804	0.15328	[Recognized]
[1754]	MARK1	--->	MAPT	0.095209	-0.10782	[Recognized]
[1755]	PRKCD	--->	IRS1	0.99369	-0.99616	[Recognized]
[1756]	CSNK2A1	--->	PPP1R8	0.7025	-0.72853	[Recognized]
[1757]	ABL1	<---	YAP1	-0.10901	0.012183	[Unrecognized]
[1758]	ACAN	--->	MMP20	0.022421	-0.030194	[Unrecognized]
[1759]	CTSG	--->	IGFBP3	0.070438	-0.036479	[Recognized]
[1760]	RPS6KA5	--->	STAT3	0.6387	-0.6669	[Recognized]
[1761]	PRKG1	--->	RGS2	1.0011	-1.0099	[Recognized]
[1762]	CDK5	--->	EZR	0.28628	-0.30395	[Recognized]
[1763]	MAPK3	<---	DUSP1	-1.0004	1.0016	[Unrecognized]
[1764]	INSR	<---	CBL	-0.20671	0.19508	[Unrecognized]
[1765]	MAPK1	<---	DUSP6	-1.0004	1.0016	[Unrecognized]
[1766]	PPP2CA	--->	CAD	0.27734	-0.28429	[Recognized]
[1767]	MMP13	<---	COL2A1	-0.051184	0.041848	[Unrecognized]
[1768]	FGFR3	--->	STAT1	0.014039	-0.050707	[Recognized]
[1769]	PTPRE	--->	MAPK1	1.0061	-1.009	[Recognized]
[1770]	MAPK1	<---	PTPN7	-1.009	1.0061	[Unrecognized]
[1771]	PDK4	--->	PDHA1	1.0004	-0.99606	[Recognized]
[1772]	PRKCG	--->	PEBP1	1.0121	-1.008	[Recognized]
[1773]	PTPN11	--->	STAT5B	1.0012	-1.0146	[Recognized]
[1774]	CASP6	--->	PSEN2	0.58173	-0.69269	[Recognized]
[1775]	PRKCZ	--->	YWHAB	0.25396	-0.26837	[Recognized]
[1776]	CSNK2A1	--->	KIF1C	0.42625	-0.44778	[Recognized]
[1777]	MMP10	<---	HAPLN1	-0.093464	0.097177	[Unrecognized]
[1778]	KAT2B	--->	MYOD1	0.35027	-0.34832	[Recognized]
[1779]	PTPN11	--->	PECAM1	0.26319	-0.077948	[Recognized]
[1780]	IKBKE	--->	NFKBIA	1.0116	-0.99754	[Recognized]
[1781]	SYK	--->	BTK	1.0133	-1.0127	[Recognized]
[1782]	E4F1	<---	TP53	-0.030171	0.020776	[Unrecognized]
[1783]	JAK2	--->	SIRPA	0.29881	-0.17811	[Recognized]
[1784]	PLK1	--->	ECT2	0.2673	-0.28668	[Recognized]
[1785]	PTK2	--->	ACTN1	0.0035192	-0.050849	[Recognized]
[1786]	PRKAR2B	--->	LASP1	0.13443	-0.12098	[Recognized]
[1787]	MAPK14	--->	CDC25C	1.0024	-1.001	[Recognized]
[1788]	PKN1	--->	MAPT	-0.005192	-0.067776	[Recognized]
[1789]	PRKCA	--->	ITPKA	0.87955	-0.8782	[Recognized]
[1790]	GRK5	--->	BDKRB2	0.13522	-0.19362	[Recognized]
[1791]	EHMT2	<---	HIST3H3	-0.11384	0.11647	[Unrecognized]
[1792]	CSNK2A1	--->	PGR	0.99834	-1.0079	[Recognized]
[1793]	PRKACA	--->	HDAC8	0.4	-0.42066	[Recognized]
[1794]	PDPK1	--->	AKT1	0.99822	-0.99934	[Recognized]
[1795]	AKT1	--->	TERT	1.0008	-1.0008	[Recognized]
[1796]	CASP3	--->	BMX	0.049596	-0.14137	[Recognized]
[1797]	CHEK2	--->	BRCA1	0.19191	-0.20845	[Recognized]
[1798]	FURIN	<---	BACE1	-0.072907	0.075966	[Unrecognized]
[1799]	PRKACA	--->	PPP1R1B	1.0061	-1.0155	[Recognized]
[1800]	ZAP70	--->	DBNL	0.9915	-0.99905	[Recognized]
[1801]	MAP2K1	--->	PARVA	0.53816	-0.15162	[Recognized]
[1802]	ATM	<---	BRCA1	-0.14033	0.12872	[Unrecognized]
[1803]	IKBKB	--->	NFKBIA	1.0136	-1.0087	[Recognized]
[1804]	MAPK14	--->	MAPKAPK5	1.008	-1.0015	[Recognized]
[1805]	PRKCA	--->	INSR	0.19651	-0.20082	[Recognized]
[1806]	SIRT1	--->	TP53	1.0032	-0.99659	[Recognized]
[1807]	ROCK1	--->	MSN	-0.0036985	-0.010475	[Recognized]
[1808]	MAPK1	--->	MCL1	1.2421	-1.2851	[Recognized]
[1809]	PDPK1	--->	RPS6KA1	1.0929	-1.1695	[Recognized]
[1810]	KAT5	--->	HIST2H2AC	0.15564	-0.12976	[Recognized]
[1811]	CDK2	--->	UBE2A	0.50887	-0.53523	[Recognized]
[1812]	LIMK1	--->	CFL2	0.16176	-0.17545	[Recognized]
[1813]	SETDB1	<---	HIST3H3	-0.15166	0.17341	[Unrecognized]
[1814]	CDK19	--->	EIF3F	0.46374	-0.49734	[Recognized]
[1815]	PPP2CA	--->	AKT1	0.35089	-0.342	[Recognized]
[1816]	PDPK1	--->	PRKCZ	0.27769	-0.26638	[Recognized]
[1817]	SUV39H1	--->	HIST3H3	0.043597	-0.029918	[Recognized]
[1818]	PRKCG	--->	CASR	0.55548	-0.55976	[Recognized]
[1819]	ROCK1	--->	EZR	-0.0022587	-0.012307	[Recognized]
[1820]	CDK5	--->	DNM1	0.24668	-0.29072	[Recognized]
[1821]	RPS6KA1	<---	MTOR	-1.0002	1.0159	[Unrecognized]
[1822]	MAPK1	--->	CAD	0.43416	-0.45528	[Recognized]

```
[1823]  MAPK3   --->    HSPB8   1.0388  -0.99926        [Recognized]
[1824]  PRKCH   --->    PTPN11  0.44605 -0.47505        [Recognized]
[1825]  MMP2    <---    IL1B    -0.18207        0.18897 [Unrecognized]
[1826]  PRKCA   --->    ATP2B2  0.14677 -0.15808        [Recognized]
[1827]  EGFR    --->    VAV2    0.99895 -0.99724        [Recognized]
[1828]  PRKACA  --->    BRAF    1.0065  -1.0057 [Recognized]
[1829]  CASP3   --->    CDH1    0.30281 -0.3521 [Recognized]
[1830]  PRKACA  --->    ITGA4   0.026402        -0.20724        [Recognized]
[1831]  CASP6   --->    PRKCZ   0.19066 -0.19986        [Recognized]
[1832]  CASP7   --->    PSEN1   0.58173 -0.69269        [Recognized]
[1833]  PRKCB   --->    CASR    0.55548 -0.55976        [Recognized]
[1834]  MAPK7   --->    MEF2A   0.15063 -0.20504        [Recognized]
[1835]  MMP1    <---    SERPINA3        -0.13073        0.15283 [Unrecognized]
[1836]  CASP8   --->    PCYT1A  0.5623  -0.70302        [Recognized]
[1837]  PRKACA  <---    RAP1B   -1.0074 1.0132  [Unrecognized]
[1838]  CASP8   --->    PLEC1   0.57989 -0.72138        [Recognized]
[1839]  CDK7    --->    ESR1    0.99834 -1.0079 [Recognized]
[1840]  CMA1    --->    AGT     0.11833 -0.082185       [Recognized]
[1841]  RPS6KA3 <---    STK11   -0.74727        0.78966 [Unrecognized]
[1842]  PRKCD   --->    PLSCR1  0.78169 -0.77232        [Recognized]
[1843]  CASP3   --->    STK24   0.87492 -0.98085        [Recognized]
[1844]  CSNK2A1 --->    HDAC2   0.4     -0.42066        [Recognized]
[1845]  CSNK2A2 --->    MYCN    1.1385  -1.1233 [Recognized]
[1846]  AKT2    --->    ESR1    1.0083  -1.0039 [Recognized]
[1847]  PTPN11  --->    MPZL1   0.97001 -0.82769        [Recognized]
[1848]  PRKCA   --->    KCNQ2   0.9144  -0.91279        [Recognized]
[1849]  BUB1    --->    CDC20   0.83716 -0.86422        [Recognized]
[1850]  GSK3B   --->    MAPT    0.99929 -1.0027 [Recognized]
[1851]  RPS6KA1 --->    ESR1    0.56871 -0.73191        [Recognized]
[1852]  PRKCD   --->    NCF4    0.51755 -0.53024        [Recognized]
[1853]  PDPK1   --->    PKN1    0.68989 -0.69757        [Recognized]
```

[Section 3] Predictions on HPRD

[1]	DLG1	--->	ATP2B2	0.12627	-0.13697
[2]	ACVR1	<---	RAB6B	-0.32935	0.31819
[3]	BDP1	--->	ZBTB43	0.25674	-0.25902
[4]	PTPRN	--->	SNTB2	1.2669	-1.258
[5]	GMFB	<---	MAPK1	-0.78322	0.75717
[6]	TAX1BP3	--->	GLS2	0.61152	-0.57593
[7]	DLG1	--->	KCNA3	0.047736	-0.049447
[8]	GRK4	--->	FSHR	0.11471	-0.1272
[9]	MAPK14	--->	NFATC1	0.99419	-1.0008
[10]	CTSG	--->	SERPIND1	0.11833	-0.082185
[11]	CTLA4	<---	CD80	-0.050761	-0.028954
[12]	TGIF1	<---	WWP1	-0.2468	0.23487
[13]	GPSM2	<---	GNAI3	-0.20473	0.22114
[14]	LYN	--->	DOK1	1.3209	-1.1853
[15]	SMAD5	--->	ZNF8	0.1194	-0.13537
[16]	HAND2	?	HAND1	-0.65635	-0.65635
[17]	PIK3R2	<---	KIT	-0.63726	0.60534
[18]	IL1RAP	<---	IL1B	-0.18407	0.18683
[19]	TAF1	--->	RBL1	0.097803	-0.11032
[20]	FGF1	<---	RPS2	-0.013688	0.047514
[21]	MAPK1	--->	CAV1	0.70376	-0.73589
[22]	NBEA	<---	BMPR2	-0.11896	0.098606
[23]	SEMA4D	--->	CD72	0.26693	-0.25476
[24]	BTBD2	--->	SMAD1	0.084109	-0.10002
[25]	BIRC2	--->	TRAF1	0.19555	-0.19908
[26]	NKRF	--->	REL	0.1584	-0.16792
[27]	BCL3	<---	MAPK3	-0.26299	0.25926
[28]	PRSS23	<---	MAPK8IP2	-0.032535	0.047724
[29]	PPP2R1A	--->	RAB18	0.13801	-0.13281
[30]	LCK	--->	PTK2	0.51061	-0.24948
[31]	SMAD2	<---	RUNX2	-0.31445	0.39743
[32]	CCR5	<--->	CXCR4	0.49511	0.49511
[33]	ETS2	<---	ZFYVE9	-0.11525	0.10591
[34]	ACTN4	<---	COL17A1	-0.012425	0.0026579
[35]	CASP9	<---	CASP3	0.10027	0.17851
[36]	PAK4	--->	YWHAG	0.14174	0.020227
[37]	SSH2	--->	ACTB	0.63171	-0.63329
[38]	CDK1	--->	UBE2A	0.50887	-0.53523
[39]	HIC1	<---	SIRT1	-0.06171	0.049479
[40]	BCAR1	<---	PTPN12	-0.077201	0.068808
[41]	KCNJ12	<---	DMD	-0.057401	0.054136
[42]	MAPK1	--->	SORBS3	-0.0080417	-0.034505
[43]	CORO2A	<---	TBL1X	-0.041129	-0.024823
[44]	SERPINA5	<---	PROC	-0.023809	0.04046
[45]	RGS7	--->	GNAO1	0.60077	-0.60607
[46]	CALCOCO1	<---	TBC1D17	-0.0041576	0.039974
[47]	DRD2	--->	CADPS2	0.13617	-0.13006
[48]	PRKCI	<---	IRAK1	-0.015265	0.068486
[49]	ZHX1	<---	TOLLIP	-0.012971	0.0099911
[50]	FLNA	<---	DRD3	-0.44658	0.47769
[51]	BRAF	<---	MAPK1	-1.0057	1.0065
[52]	NUP214	--->	SMAD3	0.41245	-0.32954
[53]	KDR	--->	FYN	0.84641	-0.66543
[54]	GAS7	--->	ACTA1	0.29677	-0.3054
[55]	RBL2	<---	CCNA2	-0.28031	0.26997
[56]	SIGLEC11	<---	PTPN6	-0.13709	0.14161
[57]	TRIP4	<---	EP300	-0.19086	0.18651
[58]	RASSF1	<---	TNFRSF10A	0.011695	0.095942
[59]	CXCR4	--->	VAV1	0.049713	-0.23901
[60]	KAT2A	--->	MYC	0.15101	-0.15715
[61]	SORBS2	--->	DLGAP1	0.20943	-0.2074
[62]	CTBP2	<---	EP300	-0.071355	0.060646
[63]	HBEGF	--->	CD44	0.060437	-0.06726
[64]	UBE2V2	<---	IGSF21	-0.13191	0.12779
[65]	MAGI2	<---	PLXNB3	-0.062789	0.07128
[66]	CPNE4	<---	UBE2O	-0.12637	0.16628
[67]	BTK	--->	GTF2I	1.0094	-0.99988
[68]	COPS5	--->	SMAD4	0.99487	-0.99775
[69]	TNFRSF11A	--->	TRAF3	0.088651	-0.095839
[70]	SOCS6	--->	IRS4	0.078985	-0.080805
[71]	NSD1	--->	THRA	0.12772	-0.10069
[72]	FXYD1	<---	ATP1B1	-0.089911	0.12923
[73]	ABL1	--->	PIK3R1	0.057373	-0.22433
[74]	ELF4	<---	SKP2	-0.2897	0.33154

[75]	SH2D3C	<---	EFS	-0.11314	0.092592
[76]	FGG	<--->	FGB	0.0011944	0.0011944
[77]	MAPK1	--->	AR	1.2465	-1.2513
[78]	PRKCA	--->	HSPB8	0.93891	-0.93193
[79]	FHL3	<---	CTBP2	-0.052578	0.046869
[80]	GNA13	<---	PRKCD	-0.76398	0.77273
[81]	CHRDL2	--->	BMP2	0.71403	-0.67452
[82]	CCR4	--->	CCL17	0.45214	-0.43752
[83]	HIPK3	--->	ZYX	0.5736	-0.58259
[84]	SMAD3	<---	SQSTM1	-0.22445	0.26503
[85]	PPYR1	<---	PPY	-0.19468	0.20502
[86]	SYTL3	--->	NRXN1	0.083467	-0.043035
[87]	LRP5	<---	SMAD9	-0.21453	0.20387
[88]	HGS	--->	PIK3R1	0.026935	-0.067689
[89]	SFRS7	<---	NXF1	-0.011155	0.042109
[90]	CSNK2A1	--->	DDIT3	0.97322	-0.93471
[91]	PAK1	--->	RAF1	0.65894	-0.56404
[92]	PLEKHG2	<---	RHOA	-0.29753	0.32945
[93]	RAPGEF5	<---	RAP2B	-0.99657	0.99903
[94]	CSNK2A1	--->	TCF7L2	1.0076	-0.99098
[95]	CSNK1A1	--->	TNFRSF1B	0.20779	-0.21541
[96]	C5	--->	C3	0.13939	0.12665
[97]	CTNNA2	--->	CTNNBIP1	0.51284	-0.4891
[98]	ROCK1	--->	ARHGAP24	-0.0070945	-0.02987
[99]	PAX6	<---	SOX3	-0.084966	-0.07746
[100]	RPH3A	<---	RAB15	-0.096859	0.11783
[101]	SORBS2	--->	PTK2B	-0.069674	-0.09855
[102]	CASP3	--->	PXN	0.4894	-0.56695
[103]	TNF	--->	DCN	0.17426	-0.15485
[104]	FNBP1	--->	TNKS	0.065594	-0.061122
[105]	SGK3	<---	GSK3A	-0.097142	0.096555
[106]	SCARB2	--->	THBS1	0.38122	-0.2868
[107]	FANCB	--->	F3	0.37059	-0.37629
[108]	GATAD2B	<---	KAT2B	-0.1833	0.18705
[109]	BMPR1B	--->	SASH3	0.035114	-0.049436
[110]	NLRP1	--->	CASP9	-0.038798	-0.12583
[111]	YWHAZ	--->	BRAF	0.18212	-0.19664
[112]	SMURF2	--->	SMAD1	0.84042	-0.86047
[113]	RNF11	<---	HUWE1	-0.083767	0.076564
[114]	ESR1	--->	SMAD3	0.22689	-0.17818
[115]	FGF3	--->	FGFR2	0.54173	-0.53789
[116]	RNF216	<---	TLR3	-0.27863	0.30781
[117]	SRI	--->	CACNA1S	0.13593	-0.14569
[118]	AR	<---	CCND1	-1.004	0.99358
[119]	STK4	--->	TP53	0.1946	-0.19278
[120]	RPS6KA5	--->	HIST3H2A	1.009	-0.99586
[121]	TAF1	--->	RB1	0.17555	-0.18494
[122]	ACTR3	<---	CTTN	-0.1761	0.21101
[123]	GNAO1	<---	ADORA1	-1.008	1.0129
[124]	ATM	<---	NBN	-0.22664	0.21656
[125]	NR1D2	<---	CUL1	-0.10593	0.11411
[126]	SYNJ1	<---	PACSIN3	-0.096023	0.10986
[127]	YWHAE	<---	RGS3	-0.15346	0.15514
[128]	GRID1	<---	EML1	-0.12528	0.13185
[129]	WT1	<---	U2AF2	-0.11317	0.11986
[130]	JAK1	--->	PLCG2	0.051189	-0.062261
[131]	KCNG4	?	KCNB1	-0.025901	-0.025901
[132]	ZBTB16	<---	VDR	-0.059674	0.055651
[133]	TLN1	<---	ITGB7	-0.079668	0.067751
[134]	EPHB2	--->	VAV2	0.10096	-0.16475
[135]	PTPN18	<---	TEC	-0.43908	0.26244
[136]	MITF	<---	MAPK1	-0.98821	1.0118
[137]	TNC	--->	EGFR	-0.0017137	-0.011891
[138]	CAPN2	--->	NFKBIA	0.82628	-0.82769
[139]	F11R	--->	PARD3	0.053652	-0.065238
[140]	ACVR1	<---	RAB3B	-0.32935	0.31819
[141]	GNAI1	--->	WASF1	0.19162	-0.18921
[142]	RGS4	--->	GNAQ	1.0012	-1.0015
[143]	SRY	<---	SMAD3	-0.10994	0.18625
[144]	EFS	<---	PTPN12	-0.19304	0.18318
[145]	DNMT3B	<---	DNMT1	-0.033562	0.048618
[146]	ST14	<---	F2RL1	-0.19821	0.2007
[147]	PRSS23	<---	NCAPD3	0.014901	0.019061
[148]	SH2D1B	<---	LY9	-0.051869	0.077004
[149]	OPTN	<---	GTF3A	-0.012309	0.011952
[150]	TGFBR1	--->	WDR13	0.3741	-0.40828

[151]	GNA11	<---	ADRA1B	-0.96014	0.95799
[152]	GFI1B	<---	TENC1	-0.037189	0.02534
[153]	HCRTR1	<---	CNR1	0.00080274	0.15042
[154]	KCNH5	<--->	KCNH1	0.0023909	0.0023909
[155]	BRAF	--->	MAP2K1	1.0017	-1.0053
[156]	PRKD1	--->	MAPK9	0.57016	-0.033526
[157]	SMN1	--->	BCL2	0.45866	-0.45952
[158]	HNRNPC	?	RBM41	-0.016298	-0.016298
[159]	MATN2	<---	COL4A1	-0.02592	0.013745
[160]	FBXW11	--->	CDC25A	0.33885	-0.34518
[161]	MSX2	--->	CEBPA	0.038752	0.0090572
[162]	FES	<---	JAK3	-1.0007	0.98981
[163]	VAV3	<---	PIK3R1	-0.025062	-0.0079531
[164]	TBP	<---	ZNF76	-0.0062722	-0.0049845
[165]	PPP3R1	<---	MAP3K5	0.046307	0.37697
[166]	RNF146	<---	TGFBR1	-0.20746	0.18169
[167]	ASAP3	<---	ARF6	-0.26114	0.25744
[168]	DNMT3A	--->	SUV39H1	0.051582	-0.044259
[169]	MICALL1	<---	RAB1A	-0.22699	0.24091
[170]	PPFIBP2	<---	PTPRC	-0.1264	0.12264
[171]	ATN1	--->	WDR5	0.055796	-0.058195
[172]	CRELD1	<---	COPS6	-0.23505	0.26239
[173]	AKAP9	<---	PRKAR2A	-0.35774	0.38972
[174]	CSNK2A1	--->	ARRB2	0.42184	-0.44306
[175]	UBASH3B	<---	JAK2	-0.069785	0.10543
[176]	PIK3R1	<---	HCK	-0.99387	0.9987
[177]	PXN	<---	ITGA6	-0.02819	0.016633
[178]	GNB2	<---	PCBP1	-0.086464	0.11936
[179]	AKT1	--->	NR4A1	1.0083	-1.0039
[180]	GRIN2D	<---	DNM1	-0.0091707	-0.0028528
[181]	SUV39H1	<---	DNMT1	-0.044735	0.043628
[182]	IRAK3	<--->	IRAK2	0.56624	0.56624
[183]	ABL1	<---	PTPN6	-0.42833	0.086412
[184]	ASAP1	<---	PTK2B	-0.078275	0.013981
[185]	PCYT1B	--->	ACTA1	0.54914	-0.54501
[186]	LCP2	--->	SHC1	0.28326	-0.30576
[187]	DOK1	<---	INPP5D	-0.10368	0.094457
[188]	CLEC3B	<---	PLAT	-0.025022	0.027855
[189]	CHEK1	--->	CDC25C	0.69298	-0.67632
[190]	RNF2	--->	CBX4	0.017911	0.016956
[191]	SYT1	--->	FGF1	0.097338	-0.057512
[192]	GRB10	<---	IGF1R	-0.20624	0.16991
[193]	CREBBP	<---	CHUK	-0.064116	0.045988
[194]	ARID3A	<---	BTK	-0.75101	0.71001
[195]	DLG2	<---	DLGAP1	-0.044143	0.050677
[196]	ADAM15	<---	YES1	-0.53832	0.63049
[197]	SMAD3	<---	C10orf54	-0.30846	0.38792
[198]	TBC1D10A	<---	SLC9A3R2	0.011063	0.025653
[199]	RYR3	--->	RYR2	-0.0069818	-0.0070206
[200]	CDK6	--->	CCND1	1.0544	-1.0774
[201]	PCBP1	<---	TNRC4	-0.015404	0.029134
[202]	MBIP	<---	MAP3K12	-0.46603	0.46792
[203]	DAXX	<---	MDM2	-0.11965	0.10265
[204]	SKP2	--->	MYBL2	0.039775	-0.036163
[205]	STAT1	<---	SRC	-0.58308	0.66789
[206]	AKT2	--->	FSHR	0.11894	-0.072894
[207]	SYK	<---	SRC	-0.63447	1.0269
[208]	SDCBP2	--->	NRXN1	0.044258	-0.007542
[209]	BAG4	<---	HSPA1A	-0.73313	0.73251
[210]	SETD7	--->	HIST3H3	0.43678	-0.40934
[211]	MPP3	<---	PLK2	-0.12572	0.11145
[212]	SHC1	--->	APP	0.033503	-0.051464
[213]	STK16	--->	ADAMTSL4	0.40525	-0.42664
[214]	MAP3K4	<---	MAP2K6	-0.38277	0.050282
[215]	PTPN22	--->	GRB2	0.25319	-0.34914
[216]	SHC1	<---	PDGFRB	-0.068478	0.033365
[217]	CEP152	--->	EPB41L3	0.083725	-0.07971
[218]	PTPN13	--->	NGFR	0.08997	-0.087832
[219]	MAPK14	--->	CDC25C	1.0024	-1.001
[220]	CD86	--->	CTLA4	0.059906	-0.11655
[221]	FGFR3	<---	RNF130	-0.11244	0.10281
[222]	JUP	<---	BIRC2	-0.058856	0.047238
[223]	LCK	--->	ZAP70	1.0002	-0.98599
[224]	CUL1	--->	NFKBIB	0.48134	-0.47008
[225]	AKT2	<---	RAB3D	0.060359	0.09142
[226]	SMARCA4	<---	SMARCE1	-0.014084	0.003854

[227]	RNF2	<--->	RING1	0.003228	0.003228
[228]	MMP9	<---	CXCL5	-0.16713	0.18806
[229]	SOCS3	<---	CSF1R	-0.40275	0.43856
[230]	JAK2	<---	EGFR	-1.0154	1.0001
[231]	PRMT1	--->	HNRNPU	0.27972	-0.27726
[232]	MAP2K6	<---	MAP3K5	-0.99683	0.9934
[233]	TFG	<---	ARL15	-0.4038	0.41054
[234]	NOD2	<---	RIPK2	-0.26618	0.072332
[235]	HIPK3	<---	LIMK2	-0.2455	0.2216
[236]	PXN	<---	NCK1	-0.47459	0.45488
[237]	PARD3	<---	YWHAB	-0.030232	0.031301
[238]	SLC9A3R2	<---	SLCO1A2	-0.038024	0.07475
[239]	ASAP2	<---	PTK2B	-0.088166	0.047717
[240]	SYK	<---	LCK	-0.98599	1.0002
[241]	PPM1B	--->	MSN	0.10633	-0.11549
[242]	DVL2	--->	DAAM1	0.040131	-0.053465
[243]	ENO2	--->	MAP4	0.080122	-0.063761
[244]	TPP1	--->	TERT	0.04367	-0.051524
[245]	CREBBP	--->	FOXM1	0.17271	-0.17713
[246]	CCNH	<---	ERCC3	-0.36627	0.39992
[247]	LIMS2	<---	ILK	-0.43923	0.43884
[248]	SLC9A3R2	<---	SGK1	-0.45034	0.22846
[249]	JAK2	--->	IRS2	0.98801	-1.0002
[250]	DLG2	--->	GUCY1A2	0.01917	-0.012618
[251]	PDGFA	--->	A2M	0.12055	-0.10555
[252]	DACH1	--->	SMAD3	0.21461	-0.16954
[253]	PRKACA	<---	RHOA	-1.0074	1.0132
[254]	STAT5A	<---	GHR	-0.18191	0.16532
[255]	ANGPT1	--->	ITGA5	0.0045462	-0.016667
[256]	AKT1	--->	SMAD4	0.26467	-0.21636
[257]	RFX1	<---	HMGB1	-0.076039	0.079652
[258]	PRELP	--->	FBLN2	0.063045	-0.06078
[259]	SMAD3	<--->	SMAD2	0.68552	0.68552
[260]	SOS2	--->	HRAS	0.99534	-0.99973
[261]	DAB2	<---	FGR	-0.94185	1.1509
[262]	SMN2	<---	DDX20	-0.00071951	0.0045634
[263]	DDX5	--->	NCOA2	0.015459	-0.027033
[264]	AKAP13	<---	GNAQ	-0.042898	0.040587
[265]	MARK2	--->	YWHAB	0.078063	-0.091816
[266]	PCSK5	--->	ITGA6	0.004156	-0.016141
[267]	AR	<---	BRCA1	-0.58357	0.57775
[268]	ACP1	--->	EPHB2	0.023989	-0.034598
[269]	ESR1	--->	CALM3	0.15612	-0.12827
[270]	GRIN2D	<---	SPTAN1	-0.020948	0.0088781
[271]	RAC1	<---	PRKCD	-0.66262	0.67714
[272]	PPM1F	<---	CAMK2A	-0.084071	0.073397
[273]	PTK2	--->	BCAR1	-0.01537	-0.15041
[274]	PLG	<---	ENO1	-0.0053023	0.0090109
[275]	TGFBR2	--->	CD44	0.26771	-0.26375
[276]	GADD45G	?	GADD45B	-0.10341	-0.10341
[277]	AEBP2	<---	SUZ12	-0.029661	0.034347
[278]	DIAPH1	--->	PAX6	0.0005653	-0.010435
[279]	ZNF24	<---	ZBTB16	-0.032908	-0.016881
[280]	TNK2	<---	CSPG4	-0.019225	0.0059758
[281]	TTRAP	<---	TRAF2	-0.13869	0.14432
[282]	BTRC	--->	SKP1	0.29041	-0.30059
[283]	BAG1	<---	HBEGF	-0.14801	0.15844
[284]	EP300	--->	NCOA1	0.020953	-0.033602
[285]	EGFR	--->	HIST3H3	0.32548	-0.32251
[286]	ATXN1	--->	ZHX2	0.00068007	-0.0082126
[287]	SOCS3	--->	PRLR	0.0057133	-0.017809
[288]	POU2F2	<---	HMGB2	-0.26954	0.30083
[289]	KPNA2	<---	TXNIP	-0.018242	0.032321
[290]	HERC5	--->	CCNB1	0.47579	-0.47291
[291]	MAPT	<---	CALM1	-0.028646	0.050498
[292]	CTSE	--->	A2M	0.46612	-0.45657
[293]	YWHAB	<--->	YWHAE	0.069023	0.069023
[294]	SMAD4	<---	TSSK3	-1.0074	1.012
[295]	PTPN23	--->	DDX24	0.76916	-0.77049
[296]	GRB14	<---	PRKCZ	-0.12581	0.11836
[297]	CEBPA	<---	PIAS1	-0.1497	0.16677
[298]	TRIM37	--->	RHPN1	0.052074	-0.03303
[299]	YWHAZ	--->	TSC2	0.294	-0.29138
[300]	TRIP6	<---	SRC	-0.95102	1.0627
[301]	CUL7	--->	RBX1	0.074373	-0.041419
[302]	TENC1	<---	AXL	-0.18274	0.14902

[303]	BMP1	<---	CHRD	-0.12129	0.10766
[304]	SMAD1	<---	KAT2B	-0.6563	0.6249
[305]	GNA11	<---	EDNRA	-1.008	1.0129
[306]	EPHB2	--->	SDCBP	0.18515	-0.1943
[307]	MAP3K14	--->	EDAR	0.40179	-0.79895
[308]	MAP3K7	--->	MAP3K3	0.16009	-0.071619
[309]	HGS	<---	MET	-0.1196	0.07692
[310]	ADAM15	<---	SH3PXD2A	-0.025517	0.021174
[311]	NFKB1	--->	CFLAR	1.003	-1.005
[312]	REM1	--->	CACNB4	0.42812	-0.39718
[313]	TRIM37	<---	PBK	-0.48607	0.4657
[314]	SFRS4	--->	SFRS6	-0.0092275	-0.015971
[315]	HRAS	<---	RASA1	-1.0119	1.0001
[316]	PRF1	--->	DDX24	0.036371	-0.017156
[317]	ARHGAP26	--->	PKN1	-0.029107	-0.044734
[318]	BCAR1	<---	PPP1R15A	-0.0060986	0.0023542
[319]	PIK3CD	<---	ERAS	-1.0082	0.98913
[320]	PSTPIP1	--->	CD2	0.084614	-0.085065
[321]	MET	--->	VAV1	0.12486	-0.44518
[322]	HTRA1	--->	PARD6B	-0.0044938	-0.010538
[323]	ZRANB2	--->	SMN1	0.04101	-0.02563
[324]	ATF3	--->	SMAD3	0.10785	-0.073259
[325]	SMAD2	<---	MEF2A	-0.052689	0.061596
[326]	BLNK	<---	NCK1	-0.26884	0.41671
[327]	HIP1	<---	CASP3	-0.71324	0.60225
[328]	MIB1	--->	DAB2	-0.0029193	-0.006559
[329]	KIT	--->	EPOR	0.11906	-0.15246
[330]	BCR	--->	SFN	0.078735	-0.083313
[331]	DTX1	--->	NOTCH2	0.13087	-0.14372
[332]	MYO5B	<---	RAB11A	-0.23098	0.24373
[333]	PPFIA1	<--->	PPFIA2	0.049612	0.049612
[334]	ACP1	--->	ZAP70	1.0132	-0.99905
[335]	FBXW11	--->	GLI2	0.13465	-0.14469
[336]	GAB1	--->	CRK	0.057403	-0.069249
[337]	PDCD6IP	<---	SRC	-0.89191	1.0053
[338]	CCNA1	<---	CDC25A	-0.3246	0.33489
[339]	PRSS1	--->	SERPINF2	0.11833	-0.082185
[340]	CD209	<---	CEACAM1	-0.05234	0.048302
[341]	KCNJ1	<---	SLC9A3R1	-0.0070376	0.044422
[342]	PIAS4	--->	TP53	0.2595	-0.25937
[343]	WAS	<---	DLG1	-0.15726	0.1281
[344]	NCSTN	<---	PSEN2	-0.0074558	0.011175
[345]	NCK1	<---	EGFR	-0.65916	0.64953
[346]	ACVR1	--->	VEPH1	-0.005073	-0.039192
[347]	LAT	<---	PTPRJ	-0.99057	0.99918
[348]	UBQLN1	--->	PSEN1	0.050774	-0.016623
[349]	FLII	--->	ESR1	0.077619	-0.070414
[350]	PRKAR1B	--->	SMAD3	0.54864	-0.46649
[351]	CBL	--->	OSTF1	0.38091	-0.39253
[352]	TJP2	<---	YWHAG	-0.070569	0.073422
[353]	HCK	--->	ACTB	0.83599	-0.74852
[354]	CDC42BPA	--->	MYL9	0.40362	-0.4096
[355]	RHOA	--->	PRKCZ	0.99149	-1.0135
[356]	PRKCA	--->	BCL2	0.61202	-0.61716
[357]	MAPK12	--->	RGS3	0.063422	-0.10903
[358]	OPRD1	--->	GNAI1	1.0129	-1.008
[359]	BLM	<---	TERF2	-0.0012546	0.016635
[360]	ATN1	--->	RAD54L2	0.019876	-0.026722
[361]	TRAF2	<---	BIRC3	-0.1025	0.095718
[362]	HSP90AB1	--->	WASL	0.034152	-0.051589
[363]	NFKBIA	<---	UBE2D2	-0.99915	0.99947
[364]	HNRNPR	<---	BMPR2	-0.24925	0.21769
[365]	KIF13B	<---	DLG4	-0.028391	0.020395
[366]	VAV1	<---	ACTA1	-0.12378	0.0031592
[367]	RANBP10	<---	RAN	-0.21869	0.23174
[368]	SCTR	<---	PRKCA	-0.36966	0.36143
[369]	DDR2	--->	COL1A1	0.033077	-0.046934
[370]	MDM4	--->	SFN	0.040034	-0.045818
[371]	NID1	<---	COL13A1	-0.035878	0.026339
[372]	BTRC	--->	CDC34	0.27621	-0.28531
[373]	ELANE	<---	CSF3	-0.25283	0.26986
[374]	SMAD1	<---	VEPH1	-0.46893	0.443
[375]	ABCA1	<---	RAPGEF6	-0.095934	0.079613
[376]	NCOA3	<---	CREBBP	-0.022438	0.0089653
[377]	TLE1	<---	HMGB1	-0.30612	0.3382
[378]	RALGPS2	<---	YWHAG	-0.17019	0.18217

[379]	KLHL3	<---	OPRD1	-0.48745	0.51726
[380]	SIX3	<---	NR4A3	-0.063713	0.064214
[381]	CIB1	--->	TERT	-0.0015079	-0.0058809
[382]	CCND2	<---	PCGF2	-0.62668	0.64062
[383]	E2F4	--->	RB1	0.47244	-0.47441
[384]	CSNK1E	--->	DVL3	0.99947	-0.9989
[385]	SOS1	--->	EPS8L2	-0.0065132	-0.011182
[386]	NLRP1	--->	BCL2L1	0.45391	-0.45402
[387]	COPS6	<--->	COPS5	0.25872	0.25872
[388]	APC2	<---	MAPRE1	-0.017246	0.0071203
[389]	ZAP70	<---	CBL	-1.0008	0.99213
[390]	PTPRN	--->	NOS1	0.3767	-0.3886
[391]	ARHGAP5	--->	ITGB1	0.058954	-0.071324
[392]	FHL2	<---	ZFYVE9	-0.12891	0.12153
[393]	SMURF2	--->	SMAD5	0.84042	-0.86047
[394]	BCL3	<---	GSK3A	-0.14407	0.11434
[395]	PTCH2	<---	IHH	-0.56975	0.56021
[396]	RHOC	--->	ARHGAP1	0.41486	-0.40165
[397]	PPA1	--->	TP53	0.36556	-0.3542
[398]	PDLIM7	<---	RET	-0.24879	0.24131
[399]	PRKAA2	--->	PFKFB2	0.086531	-0.28854
[400]	TANC1	<---	HOMER1	-0.14268	0.14839
[401]	THRB	--->	TRIP4	0.041496	-0.016159
[402]	CNTN1	--->	NOTCH2	0.22787	-0.24011
[403]	BCL2L1	<---	BID	-0.71278	0.72799
[404]	PPFIA3	--->	PPFIBP2	0.027796	0.0269
[405]	CASP8	--->	CASP3	1.0138	-1.0003
[406]	RYK	--->	WNT1	0.69175	-0.70876
[407]	GFRA2	--->	NRTN	0.39482	-0.35487
[408]	FES	--->	PIK3R1	0.22782	-0.27751
[409]	MARK2	--->	DVL3	1.0164	-1.0042
[410]	IKBKG	--->	NFKBIB	0.081824	-0.080483
[411]	RNF11	--->	EPN1	0.19496	-0.16235
[412]	STAMBP	--->	UACA	0.83154	-0.80626
[413]	PRMT1	--->	IGSF21	0.07518	-0.086177
[414]	ABCA1	--->	DLG2	0.003993	-0.013301
[415]	BARD1	<---	BRD7	-0.15932	0.17782
[416]	TGIF2	--->	SMAD3	0.16064	-0.11329
[417]	PRKACA	--->	MEP1B	0.4657	-0.48607
[418]	ZNF350	<---	BRCA1	-0.14399	0.13664
[419]	RASIP1	<---	HRAS	-0.64033	0.64447
[420]	CASP7	--->	PSEN1	0.58173	-0.69269
[421]	SMAD3	--->	HEY1	0.61773	-0.62633
[422]	PRKCD	--->	CD34	0.78257	-0.77289
[423]	RFX1	--->	SMAD4	0.14374	-0.12198
[424]	PAK1	<---	LIMK1	-0.08975	0.17985
[425]	CNTN1	<--->	L1CAM	0.018698	0.018698
[426]	RNF11	--->	UBQLN4	0.13586	-0.10319
[427]	PKD1	--->	TLN1	0.11837	-0.12883
[428]	TARDBP	--->	SETDB1	0.13553	-0.13214
[429]	ESRRA	<---	NR0B2	-0.028437	-0.0094276
[430]	TRAT1	<---	LYN	-0.99229	0.99353
[431]	MMP9	<---	AREG	-0.025724	0.024366
[432]	UBTF	--->	CCNA2	0.9372	-0.91387
[433]	EPB41	<---	DRD3	-0.19189	0.19515
[434]	RRAS2	<---	PTPN1	-0.88427	0.88724
[435]	PRKCA	--->	FLNC	0.91318	-0.91157
[436]	SORBS3	--->	DLG5	0.14486	-0.14617
[437]	ELANE	--->	SERPIND1	0.11833	-0.082185
[438]	ITCH	--->	JUN	0.17224	-0.18552
[439]	NRXN2	--->	MLLT4	0.25913	-0.21049
[440]	VCAN	--->	CD44	-0.0082207	-0.011626
[441]	RASSF5	<---	NRAS	-0.98684	1.0025
[442]	F2	--->	SERPIND1	0.057207	-0.040728
[443]	BTK	<---	PRKCE	-0.49716	0.49551
[444]	ZFHX3	<---	MYB	-0.01098	-0.00062643
[445]	SMAD1	<---	ERBB2IP	-0.16401	0.1549
[446]	SATB1	<---	EP300	-0.07259	0.062202
[447]	KCNJ12	<---	CASK	-0.1462	0.13185
[448]	WASL	<---	ITSN1	-0.049686	0.032502
[449]	MAP3K11	--->	CDC42	0.36613	-0.36833
[450]	PTPRS	<---	PTPRA	0.026323	0.06256
[451]	VEGFB	<---	VEGFA	0.00053249	0.062251
[452]	CDKN1A	--->	CEBPA	0.040192	-0.019889
[453]	SLC9A3R1	<---	GNAQ	-0.15061	0.16864
[454]	VASN	--->	TGFB1	0.60586	-0.61109

```
[455]   PIN1    --->    KLHL20  0.38529 -0.35422
[456]   NCS1    <---    IL1RAPL1        -0.46375        0.46616
[457]   ESRRA   <---    PRKCD   -0.53137        0.52878
[458]   CDK1    --->    STMN2   1.0058  -0.99021
[459]   TRAF2   --->    BANP    0.14352 -0.13786
[460]   AXIN1   --->    DVL2    -0.036269       -0.061523
[461]   FADD    <---    EDA2R   -0.22731        -0.10085
[462]   TOLLIP  <---    TLR2    -0.18829        0.18241
[463]   MAP4K2  <---    TRAF2   -1.0041 0.99389
[464]   SMAD3   <---    HNF4A   -0.24224        0.26585
[465]   EP300   --->    HNF1A   0.18505 -0.18943
[466]   YWHAZ   <---    MARK2   -0.091816       0.078063
[467]   GNB2L1  <---    PRKCB   -0.92085        0.92236
[468]   HIRIP3  --->    HIST3H3 0.23103 -0.17959
[469]   EGFR    --->    CALM1   0.34959 -0.34973
[470]   SRC     --->    PDPK1   0.50791 -0.45358
[471]   ASCC2   --->    TRIP4   0.15321 -0.12057
[472]   TNKS2   <---    GRB14   -0.078249       0.075732
[473]   PRMT2   --->    RB1     0.31873 -0.32535
[474]   MELK    --->    PPP1R8  0.42626 -0.44747
[475]   ITK     <---    CBL     -0.23287        0.21359
[476]   FLNA    <---    CDC42   -0.39396        0.4246
[477]   ITK     <---    FYN     -0.98497        1.0008
[478]   KDR     --->    PTPN6   0.52325 -0.39752
[479]   L1CAM   --->    ANK2    0.095121        -0.11221
[480]   SMAD2   <---    PPP2R1A -0.15926        0.17032
[481]   ABCA1   <---    TRIM27  -0.013625       0.003775
[482]   MAPKAPK5        --->    EIF4EBP1        0.55427 -0.76673
[483]   PVRL4   --->    MLLT4   0.090303        -0.081264
[484]   NEK8    --->    TGFBR1  0.36359 0.02614
[485]   NCF1    --->    WASF1   0.05779 -0.06833
[486]   APP     <---    PSEN1   -1.0006 0.99505
[487]   RAP1B   <---    SMURF2  -1.0084 1.0007
[488]   PNO1    <---    SGSM2   -0.028022       0.031993
[489]   EZR     --->    ACTB    0.1724  -0.17979
[490]   MC1R    ?       MC4R    -0.019473       -0.019473
[491]   STAT1   --->    GNB2L1  0.1635  -0.28689
[492]   RIPK1   --->    IKBKG   0.98942 -0.99021
[493]   GIT1    <---    PXN     -0.066907       0.057095
[494]   KPNA2   <---    BRCA1   -0.045225       0.059934
[495]   PAFAH1B1        <---    CLIP1   -0.038596       0.053251
[496]   LRP1    <---    CTSG    -0.15665        0.1475
[497]   RAPGEF2 --->    CTNNB1  0.08209 -0.09391
[498]   DOCK1   --->    RAC1    0.00098902      -0.014022
[499]   PRKCA   --->    SRC     0.25654 -0.23268
[500]   SLC16A2 --->    SERPINA7        0.065206        -0.048578
[501]   ESR2    <---    SP1     -0.11174        0.10251
[502]   DOK1    <---    PTPRH   -0.9138 0.92125
[503]   SMURF2  --->    SNRNP70 0.70809 -0.71228
[504]   PIK3R1  <---    TLR2    -0.16808        0.12124
[505]   SP3     <---    HDAC1   -0.13401        0.14006
[506]   FOS     <---    ETS1    -0.21883        0.13342
[507]   TLE1    --->    CDKN2C  0.50583 -0.47493
[508]   NOTCH2  <---    RBPJ    -0.23038        0.21833
[509]   LRRC1   --->    DLG4    0.0066871       -0.015514
[510]   CALCOCO2        <---    C9orf86 -0.06849        0.089298
[511]   RAP1GAP --->    BRAF    0.12317 -0.13652
[512]   PLEC1   --->    ACTG1   0.51886 -0.51601
[513]   DOCK2   --->    RAC2    0.016262        -0.029068
[514]   GUCY1A2 <---    DLG3    -0.10343        0.10173
[515]   MAPK9   --->    RPS6KB1 1.002   -1.0109
[516]   ARHGAP29        <---    RAP2A   -0.5107 0.5351
[517]   PRLR    <---    GH1     -0.015544       0.0031206
[518]   PARD6B  <---    PRKCI   -0.044435       0.043217
[519]   SKIL    <---    DHX30   -0.2481 0.24513
[520]   PRKCA   --->    PLD1    0.99655 -1.0006
[521]   BCR     <---    JAK2    -0.13206        0.14299
[522]   PRKG1   --->    LASP1   0.53854 -0.53556
[523]   CD3D    <---    CD8B    -0.11652        0.13452
[524]   ZAP70   --->    CARD11  0.15681 -0.16542
[525]   TNFRSF4 ?       TNFRSF9 -0.017717       -0.017717
[526]   EED     <---    HDAC1   -0.098152       0.11313
[527]   NLRP1   --->    BCL2    0.45391 -0.45402
[528]   PPM1D   --->    TP53    0.5543  -0.54214
[529]   PKP2    <---    KRT5    -0.055864       0.065384
[530]   RAB4A   --->    ACTB    0.99207 -0.99507
```

```
[531]   SOX18   --->    MEF2C   0.1213  -0.094467
[532]   MYST2   --->    AR      1.1491  -1.143
[533]   AGAP1   --->    GUCY1B3 0.34095 -0.34945
[534]   RARA    --->    STAT5B  0.27638 -0.29089
[535]   TCERG1  --->    SF1     0.14864 -0.14534
[536]   TP53    <---    HMGB2   -0.42172        0.43281
[537]   SKIL    <---    SPARCL1 -0.41866        0.42273
[538]   C1R     <---    C1QA    -0.052141       0.04292
[539]   TAF1    --->    CCND1   0.24062 -0.24459
[540]   BEGAIN  --->    ZFP64   0.20822 -0.20291
[541]   CDC42EP1        <---    RBPMS   -0.14135        0.057595
[542]   SMARCD3 --->    PPARG   0.029859        -0.029119
[543]   BCL6    <---    EP300   -0.058849       0.046751
[544]   IL16    --->    KCNJ15  0.044422        -0.0070376
[545]   FOXO3   <---    YWHAZ   -1.0122 1.0083
[546]   PRKACA  --->    GNA13   0.52385 -0.56835
[547]   TRPC3   --->    PLCG1   0.0086487       -0.024253
[548]   HDAC2   --->    CDH1    0.09416 -0.096836
[549]   EDN1    <---    EDNRA   -0.42199        0.45294
[550]   BCOR    <---    HDAC4   -0.35594        0.37055
[551]   BAIAP2  <---    SHANK2  -0.02811        0.018379
[552]   PPP1R9B --->    CDKN2A  0.15458 -0.13423
[553]   EIF4EBP2        --->    EIF4E   1.0033  -1.0062
[554]   CALCOCO2        <---    PIAS4   -0.11969        0.13241
[555]   CASP6   --->    MAPT    0.50832 -0.57833
[556]   KLK3    --->    PLG     0.0012258       -0.0091243
[557]   FRS2    <---    KDR     -0.67505        0.73119
[558]   FHL2    --->    AR      0.956   -0.95654
[559]   DTNB    <---    DMD     -0.022378       0.014832
[560]   POU3F1  <---    HMGB2   -0.26954        0.30083
[561]   ATM     <---    WRN     -0.14022        0.12861
[562]   SHC1    --->    STAT5B  0.0062057       -0.041328
[563]   MAPK3   --->    RPS6KA2 1.002   -1.0109
[564]   CDC40   <---    DHX38   -0.076481       0.091156
[565]   PPP1R9B <---    ADRA1B  -0.32355        0.32844
[566]   TRAF2   --->    APEX2   0.14432 -0.13869
[567]   SFN     --->    TSC2    0.294   -0.29138
[568]   TLN1    <---    ITGB1   -0.011994       -0.0013439
[569]   SPEN    --->    MTA2    0.084165        -0.065389
[570]   NR1H3   --->    RXRG    0.60453 -0.60548
[571]   RAB25   --->    TGFBR1  0.010625        -0.037483
[572]   PXN     <---    SRC     -0.7589 0.81631
[573]   IL6ST   <---    CNTFR   -0.079927       0.01759
[574]   SNTA1   <---    UTRN    -0.052248       0.048407
[575]   RAB11FIP3       <---    RAB11A  -0.43801        0.45325
[576]   DDR2    --->    COL3A1  -0.0016035      -0.01188
[577]   MDC1    <---    TP53BP1 -0.049033       0.077213
[578]   JUN     <---    ETS2    -0.16285        0.13316
[579]   DOK2    <---    LCK     -0.88457        0.95892
[580]   HIF3A   <---    EGLN1   -0.012006       0.0022157
[581]   KCNJ11  --->    ABCC8   0.064029        -0.059078
[582]   CSNK1D  --->    DVL3    0.99947 -0.9989
[583]   TRAF3   <---    TTRAP   -0.053779       0.056774
[584]   HSPB1   ?       CRYAB   -0.096038       -0.096038
[585]   ALS2CL  <---    RAB5A   -0.21671        0.23077
[586]   ATN1    --->    RCHY1   0.012962        -0.015395
[587]   INPPL1  <---    CBL     -0.29673        0.28378
[588]   LRRC1   --->    DLG1    0.0066871       -0.015514
[589]   HTR2A   --->    EIF3F   0.072501        -0.064329
[590]   SUFU    <---    RBPJ    -0.17028        0.16815
[591]   ACVR1   --->    FGD6    -0.010924       -0.013784
[592]   HIVEP1  --->    ATXN1   0.0041092       -0.0071124
[593]   SFRS1   <---    LNX1    -0.0053675      0.021194
[594]   MMP2    <---    BCAN    -0.030194       0.022421
[595]   EP300   --->    ETS2    0.11504 -0.12299
[596]   PTPN1   <---    SRC     -0.37818        0.26821
[597]   ACTG1   <---    NCF1    -0.22311        0.21028
[598]   IQGAP2  <---    CDC42   -0.13647        0.1548
[599]   SLC2A4  <---    SERPINH1        -0.23141        0.25993
[600]   CDK14   <---    SMURF1  -0.6445 0.64008
[601]   DNMT3A  <---    HDAC1   -0.0084751      0.024192
[602]   HIC1    <---    CTBP1   -0.091979       0.079104
[603]   IRX4    <---    RARA    -0.0027516      0.029881
[604]   CRK     --->    PIK3R1  0.0060627       -0.3721
[605]   HNRNPK  <---    SFRS3   -0.0048144      0.036464
[606]   EPS15   <---    STAM2   -0.017423       0.0078618
```

[607]	DCAF6	<---	NR3C1	-0.0096005	0.034681
[608]	DOK5	<---	NTRK3	-0.2014	0.20066
[609]	PTPN13	--->	FAS	0.08997	-0.087832
[610]	RNF7	<---	CASP7	-0.70443	0.59343
[611]	ID2	?	MYF5	-0.65635	-0.65635
[612]	COL4A5	--->	FBLN2	0.035051	-0.041937
[613]	CBL	--->	LYN	0.74409	-0.7389
[614]	S1PR3	--->	GNAI1	0.50718	-0.51255
[615]	CYTIP	<---	CYTH3	-0.22934	0.25364
[616]	CALCR	<---	CALM1	-0.24765	0.25258
[617]	TGFA	<---	SDCBP	-0.039126	0.05496
[618]	DOK1	<---	ITGB7	-0.009595	0.014286
[619]	MPP3	<---	RAB31	-0.13958	0.14603
[620]	GCA	<---	SRI	0.18055	0.30774
[621]	CD247	<---	ZAP70	-0.8746	0.82866
[622]	CXCR4	--->	MYH9	0.034821	-0.045017
[623]	ACAN	--->	MMP20	0.022421	-0.030194
[624]	GRK6	--->	SLC9A3R1	0.45593	-0.54121
[625]	CAMK2B	--->	PLCB3	0.41213	-0.4132
[626]	FGFR4	<---	FGF18	-0.99871	1.0053
[627]	SVIL	--->	AR	0.95865	-0.95812
[628]	ZIC2	<---	GLI1	-0.0068888	0.013977
[629]	OCLN	<---	YES1	-0.90342	1.0643
[630]	C8A	<---	CD59	-0.1146	0.12539
[631]	STIM2	<--->	STIM1	0.20989	0.20989
[632]	TICAM2	--->	IRAK1	0.75418	-0.78247
[633]	TFE3	?	MITF	-0.083565	-0.083565
[634]	HTR3A	<---	HSPA5	-0.7749	0.81635
[635]	RPS6KA2	--->	L1CAM	0.03878	-0.13396
[636]	PDZK1	--->	SLC22A5	0.37001	-0.34102
[637]	ESRRA	?	ESRRG	-0.0089162	-0.0089162
[638]	AR	<---	KIF1A	-0.38681	0.37726
[639]	CCNA2	<---	PGR	-0.31113	0.3199
[640]	NUP37	?	NUP43	-0.1554	-0.1554
[641]	VAV2	<---	PRLR	-0.013009	-0.0032268
[642]	PRKCA	--->	CORO1B	0.92236	-0.92085
[643]	PTPN21	--->	AKAP1	0.19433	-0.2045
[644]	ERBB2	--->	PIK3C2B	0.013224	-0.025394
[645]	NOS1	--->	ATP2B4	-0.00075504	-0.012338
[646]	ITGA2B	<---	F2	-0.024537	0.012664
[647]	FBLN2	<---	COL4A3	-0.033412	0.023756
[648]	ANK3	<---	FAS	-0.19426	0.17363
[649]	TBC1D7	--->	TSC1	0.057618	-0.016988
[650]	PRKG2	--->	LASP1	0.53854	-0.53556
[651]	GRM2	--->	RGS12	0.1187	-0.11565
[652]	INSR	<---	SOCS2	-0.046398	0.025183
[653]	PLSCR3	<---	PRKCD	-0.77232	0.78169
[654]	RGL2	<---	HRAS	-0.61434	0.59497
[655]	RIPK2	<---	SMURF1	-0.40135	0.39316
[656]	TERT	<---	PRKCA	-0.20738	0.1949
[657]	CTNND1	<---	PTPRJ	-0.54525	0.51795
[658]	CXCL11	<---	CXCR3	-0.46475	0.49595
[659]	GNB2L1	--->	PLCG1	0.60012	-0.66965
[660]	ZBED1	<---	SORBS3	-0.2078	0.20981
[661]	YWHAE	--->	IRS2	0.072749	-0.080235
[662]	USP7	--->	MAGEE1	0.10389	-0.066242
[663]	ESR1	--->	SMAD4	0.22689	-0.17818
[664]	USP6NL	--->	ACTN4	0.0061127	-0.001634
[665]	COL4A5	--->	CD93	0.032217	-0.042153
[666]	MAPK11	--->	BCL2	0.70926	-0.79494
[667]	SHC1	<---	PTPN12	-0.22467	0.13709
[668]	ADAMTS1	<---	VEGFA	-0.031079	0.023074
[669]	STAM2	<---	TYK2	-0.15813	0.15857
[670]	UBE2K	--->	HTT	0.16231	-0.14736
[671]	APC	--->	CTNNB1	-0.0092361	-0.016762
[672]	CSNK2A1	--->	GMFB	0.75717	-0.78322
[673]	ACTN1	<---	ADORA2A	-0.08121	0.077572
[674]	USP6NL	<---	GRB2	-0.29181	0.30475
[675]	SLIT2	<---	ROBO1	-0.010875	0.00065307
[676]	H3F3A	<---	BPTF	-0.031803	0.026668
[677]	FGF6	--->	FGFR2	0.54173	-0.53789
[678]	MAPK1	--->	NR4A1	0.99834	-1.0079
[679]	TRAF6	--->	KCNQ1	0.68431	-0.69133
[680]	MYST1	--->	RNF2	0.016613	-0.00071569
[681]	PSTPIP1	--->	WASF2	0.11644	-0.11432
[682]	EP300	--->	CDX2	0.18505	-0.18943

[683]	ACVRL1	<---	TGFB3	-0.60916	0.60319
[684]	IKZF3	<---	UBE2I	-0.19958	0.20197
[685]	ADRBK1	<---	EGF	-0.013188	-0.00066098
[686]	CASP8	--->	RB1	0.29894	-0.34071
[687]	CGN	--->	TJP1	0.050357	-0.043829
[688]	TRIP12	--->	SMAD9	0.40535	-0.42403
[689]	GADD45A	<---	CDK1	-0.76568	0.74161
[690]	ZHX1	--->	BARD1	0.020448	-0.014607
[691]	GIT1	<---	ARHGEF6	-0.069063	0.056398
[692]	COL4A5	--->	FN1	0.057845	-0.061633
[693]	PKP2	--->	DSC1	0.61098	-0.61498
[694]	PPP1R15A	--->	PPP1R1A	0.17814	-0.1605
[695]	DHX30	<---	HDAC1	-0.011157	0.015318
[696]	GRIN1	--->	GRIN2D	-0.037501	-0.03762
[697]	MDM2	<---	CREBBP	-0.043084	0.031284
[698]	ID1	<---	PSMD4	-0.56402	0.60154
[699]	PLK1	--->	ECT2	0.2673	-0.28668
[700]	SRF	<---	SRC	-0.5255	0.59014
[701]	SMARCA4	--->	SMARCC1	0.00052878	-0.0080339
[702]	MAP3K7	<---	MAP4K1	-0.044253	0.057445
[703]	CASP8	--->	RIPK1	1.006	-0.99988
[704]	PTPN6	--->	LAIR1	0.26319	-0.077948
[705]	GATA4	<---	CRIP2	-0.13822	0.18194
[706]	PPP1CC	--->	PPP1R8	0.14726	-0.15665
[707]	RAC1	--->	ACTA1	0.99207	-0.99507
[708]	CTSG	--->	CXCL12	0.058311	-0.024098
[709]	SRF	--->	TCF3	0.23654	-0.24221
[710]	SH3BP1	<---	RAC1	-0.41737	0.43038
[711]	YWHAG	<---	PPP1R12A	-0.071198	0.070521
[712]	MAPK1	--->	RAF1	0.98888	-1.0049
[713]	CNOT4	<---	UBE2D2	-0.28011	0.29304
[714]	POU2F1	<---	LIFR	-0.041811	0.068617
[715]	HIST2H2BE	<---	TGM2	-0.19982	0.25116
[716]	KRT14	<---	KRT5	-0.0067464	0.041547
[717]	NHLH2	<---	LMO3	-0.58677	0.62562
[718]	DAB2IP	--->	DAB1	0.12686	-0.1016
[719]	MAPRE1	<---	TERF1	-0.023461	0.057633
[720]	MYST2	--->	$ZBTB8$	0.16813	-0.15484
[721]	COL4A6	--->	CD93	0.032217	-0.042153
[722]	NFKBIA	<---	PTPN13	-0.17394	0.16053
[723]	CDH1	<---	EZR	-0.044402	0.037422
[724]	GFI1B	<---	EFEMP2	-0.1532	0.14936
[725]	SNX4	--->	LEPR	0.3803	-0.38118
[726]	NR3C1	--->	CEBPA	0.026385	0.008943
[727]	IGFBP3	<---	F2	-0.013006	0.028162
[728]	BCAR1	--->	EFS	-0.0025365	-0.0067731
[729]	STAM	<---	JAK2	-0.99545	0.98976
[730]	TINAG	--->	TERF1	0.033756	0.0005004
[731]	BCOR	<---	CPNE1	-0.37401	0.39237
[732]	SMAD3	--->	ZEB1	0.10343	-0.10164
[733]	CBLB	--->	SHC1	0.12586	-0.15929
[734]	SFRS2IP	--->	SNRNP70	0.12373	-0.11096
[735]	SGK3	<---	GSK3B	-0.6264	0.61504
[736]	BLK	<---	CBL	-0.7389	0.74409
[737]	STK35	--->	PDLIM1	0.29663	-0.30526
[738]	YWHAG	<---	CRTC1	-0.0047587	0.0013398
[739]	TRAF2	--->	ZNF205	0.11176	-0.11912
[740]	CSNK1D	--->	PER2	0.72042	-0.58384
[741]	SOCS7	--->	PLCG1	0.36928	-0.53614
[742]	GSK3B	--->	MCL1	1.2421	-1.2851
[743]	CCDC33	<---	CCDC85B	0.012422	0.022981
[744]	TP53	--->	NFKBIA	0.077947	-0.083618
[745]	ABL1	--->	DDB2	-0.074092	-0.075808
[746]	CASP8	--->	TRAF2	0.13467	-0.16633
[747]	CXCR1	--->	GNA15	1.0129	-1.008
[748]	NR1D2	?	NR1D1	-0.0089162	-0.0089162
[749]	GGA2	<---	SORT1	0.0083229	0.025913
[750]	HSPA4	--->	PRSS23	0.8457	-0.80361
[751]	BANK1	<---	LYN	-1.0008	0.98848
[752]	PBX2	--->	HOXB8	-0.03858	-0.054201
[753]	CDK4	--->	SMAD3	1.012	-1.0074
[754]	CDC42EP4	<---	TGFBR1	-0.41506	0.19605
[755]	EPHB6	--->	CRK	0.13036	-0.17576
[756]	PLEKHA1	--->	MPDZ	0.25364	-0.22934
[757]	HSPB8	?	HSPB7	-0.096038	-0.096038
[758]	CREBBP	--->	TP53	0.073526	-0.083705

[759]	SLC2A4	<---	HSP90AA1	-0.10731	0.098313
[760]	YWHAG	<---	ACIN1	-0.0094293	0.040981
[761]	PPP1R15A	--->	MLL	0.024287	-0.01873
[762]	SMAD3	--->	RBL1	0.069298	-0.075304
[763]	RHPN2	<---	RHOB	-0.41099	0.4427
[764]	VDR	?	RXRB	-0.0089162	-0.0089162
[765]	GGA1	<---	SYNRG	0.011562	0.022527
[766]	PRKG1	--->	TNNT1	0.99798	-1.0269
[767]	KHDRBS1	<---	ITK	-0.38206	0.35852
[768]	MAPK9	--->	H3F3A	1.0832	-1.0289
[769]	RB1	<---	CEBPA	-0.45357	0.45562
[770]	CSH1	<---	PTPN12	-0.33147	0.32487
[771]	LAMA4	<---	UBR1	-0.078148	0.11243
[772]	FCGR2B	--->	INPPL1	0.29935	-0.30982
[773]	TRADD	<---	RIPK1	-0.10444	-0.0050008
[774]	MARK3	--->	ASB13	0.016841	-0.029989
[775]	CCR5	--->	GNAI3	1.0129	-1.008
[776]	DDX17	<---	LNX1	-0.026125	0.023619
[777]	RAPGEF1	--->	HRAS	1.0045	-0.99301
[778]	FGFR2	<---	PAK4	-0.025242	0.038545
[779]	PIAS1	--->	CSRP2	0.11298	-0.10121
[780]	LRP2	<---	ALB	-0.39974	0.39857
[781]	PSD	--->	GNB5	0.1289	-0.09589
[782]	NRXN1	<---	APBA1	-0.037128	0.041981
[783]	RAB3B	--->	CALM2	0.73657	-0.70265
[784]	MAPK9	--->	ELK3	1.0428	-0.98814
[785]	HIPK3	--->	LIMD1	0.5736	-0.58259
[786]	AP3B1	<---	ARF1	-0.12845	0.11437
[787]	KLK4	--->	SERPINA3	0.11833	-0.082185
[788]	REG3A	<---	FN1	-0.020529	0.047
[789]	IL1RAP	<---	IL1R1	0.025339	0.070661
[790]	HIF1A	--->	TP53	0.30973	-0.31764
[791]	PDGFRB	--->	SYNGAP1	0.0070277	-0.01774
[792]	ABCC2	<---	RDX	-0.040079	0.030984
[793]	TRAF2	<---	TRAF6	0.0098547	0.14448
[794]	MAP3K14	--->	IKBKB	1.0117	-1.003
[795]	SLC3A2	--->	ITGB1	0.048164	-0.060441
[796]	RHO	<---	ADRBK1	-0.27132	0.25048
[797]	PAX3	<---	MSX1	-0.099649	-0.043846
[798]	NEDD9	--->	MICAL1	0.020561	-0.028196
[799]	DHX15	<---	YWHAB	-0.035857	0.045156
[800]	ACAN	--->	MMP19	0.022421	-0.030194
[801]	BDNF	<---	CADPS2	-0.10648	0.095107
[802]	CREBBP	--->	ELF3	0.11504	-0.12299
[803]	CRYAB	?	CRYAA	-0.096038	-0.096038
[804]	ATF6	--->	ATF6B	-0.16104	-0.19906
[805]	PRG2	--->	AGT	0.040239	-0.0063551
[806]	ZNF467	<---	STAT1	-0.12164	0.067618
[807]	GRIN3A	<---	DLG4	-0.13128	0.12067
[808]	BCL2L1	<---	PSEN1	-0.77257	0.78024
[809]	PFN2	<---	RNF146	-0.058416	0.072914
[810]	CAPN1	--->	ITGB3	0.063315	-0.07395
[811]	CDKN1A	<---	CCDC85B	-0.066447	0.074201
[812]	SYNJ2BP	--->	LRP1	0.14805	-0.15706
[813]	PRKCA	--->	DDX5	0.33929	-0.34734
[814]	CTNNA3	--->	CTNNB1	0.56912	-0.56839
[815]	SERPINB9	<---	IGSF21	-0.30915	0.3066
[816]	EPHA3	--->	STAT3	0.31675	-0.38189
[817]	ZNF24	<---	ZNF446	-0.16454	-0.011024
[818]	TRIP10	--->	WAS	0.23292	-0.27382
[819]	ITK	<---	BMX	-0.20499	-0.055822
[820]	LAT	--->	SHB	0.20087	-0.19114
[821]	APLP1	--->	TCF21	0.079297	-0.081435
[822]	MMP9	<---	TFPI	-0.1877	0.20851
[823]	RHOXF2	<---	DTX2	-0.076475	0.070564
[824]	SOS1	--->	EGFR	0.070156	-0.07959
[825]	ESR1	<---	RLIM	-0.081926	0.082128
[826]	PAK1	--->	ARHGEF2	0.25671	-0.13368
[827]	HSP90AA1	--->	CSNK2A2	0.040193	-0.056762
[828]	ETS1	<---	SP1	-0.15255	0.14216
[829]	SIRT1	--->	NR1H3	0.050679	-0.060507
[830]	THBS1	<---	TFPI	-0.30223	0.39705
[831]	YWHAB	--->	CDC25C	0.14141	-0.11975
[832]	KAT5	--->	ZNF24	0.17382	-0.1787
[833]	BANK1	--->	ITPR1	0.11559	-0.12807
[834]	IRS2	--->	SHC1	-0.012223	-0.017632

[835]	RAB27A	--->	GZMB	0.40153	-0.37105
[836]	RAC1	<---	STAT1	-0.28331	0.16384
[837]	CTNNBIP1	<---	TCF7L2	-0.33099	0.28613
[838]	SRC	--->	BMX	0.93228	-0.45147
[839]	RAG1	<---	HMGB1	-0.22024	0.2138
[840]	ARHGAP17	<---	CRK	-0.062337	0.045225
[841]	HIST2H4A	<---	TBL1X	-0.033407	0.034046
[842]	FADD	--->	CASP8	0.98719	-0.98831
[843]	CCR1	--->	CCL26	0.49595	-0.46475
[844]	HIVEP3	<---	TRAF2	-0.13336	0.13089
[845]	PLCG1	<---	GRIN1	-0.24837	0.21404
[846]	PTPN6	--->	HOXA10	0.54979	-0.43989
[847]	MLL	<---	FASLG	-0.14628	0.16527
[848]	RAVER1	<---	ACTN2	-0.11643	0.11947
[849]	CASP3	--->	PAK2	1.002	-0.99322
[850]	DVL3	<---	SNX22	-0.38	0.38014
[851]	ZMYND11	--->	E2F6	0.093965	-0.062638
[852]	PRKACA	--->	PSEN1	0.67463	-0.70045
[853]	CCL19	<---	CCR7	-0.43752	0.45214
[854]	TSC1	<---	YWHAB	-0.08934	0.11987
[855]	SYNE1	<---	MUSK	-0.1581	0.15705
[856]	TBL1XR1	--->	HIST1H4F	0.043907	-0.034106
[857]	DOK1	<---	KIT	-0.40752	0.35784
[858]	CSNK2A1	--->	ARNT	0.58841	-0.58643
[859]	PIK3R2	<---	SOCS7	-0.18873	0.12705
[860]	F9	<---	F7	-0.0065949	-0.0050527
[861]	EPB41	<---	YWHAQ	-0.057222	0.05792
[862]	PRKACA	--->	CAD	0.43416	-0.45528
[863]	KPNA2	--->	RAG1	0.1952	-0.20171
[864]	SOCS3	<---	CXCR4	-0.20208	0.22206
[865]	MYBL2	<---	CREBBP	-0.072857	0.062447
[866]	DUSP13	--->	CCDC85B	0.6542	-0.62031
[867]	PIK3R1	<---	PDGFRA	-0.064345	0.040644
[868]	SPI1	--->	CREM	0.28008	-0.26629
[869]	RGL2	<---	EEF1G	-0.15218	0.14519
[870]	EVL	--->	SPTAN1	0.00064306	-0.010733
[871]	MMP7	<---	BCAN	-0.030194	0.022421
[872]	LCK	--->	ACP1	0.47695	-0.39756
[873]	JAK2	--->	IRS1	1.0003	-0.99662
[874]	STAT5A	<---	CEBPB	-0.47325	0.45813
[875]	YWHAZ	--->	TRA2B	0.092231	-0.070624
[876]	TLE2	<---	HMGB1	-0.30612	0.3382
[877]	BTK	<---	BMX	-0.20499	-0.055822
[878]	RAB6B	--->	SMAD1	0.52759	-0.54836
[879]	BRSK1	--->	CDC25C	0.41582	-0.41122
[880]	ADCY5	<---	PRKACA	-0.87307	0.85122
[881]	KCNH2	--->	YWHAH	0.17316	-0.16536
[882]	FADD	--->	NOL3	0.029619	-0.11742
[883]	DNM1	<---	AMPH	-0.080916	0.071535
[884]	NIN	<---	PIAS1	-0.085157	0.096873
[885]	PRKCD	--->	TAGLN	0.78277	-0.77308
[886]	MARK4	<---	PRKCG	0.018773	0.018811
[887]	CSF2RA	<---	CSF2	-0.2484	0.23998
[888]	NCOA1	<---	THRB	-0.043163	0.034596
[889]	CSNK2A1	--->	HDAC1	0.4	-0.42066
[890]	THBS1	<---	FN1	-0.29774	0.39185
[891]	MMP9	<---	COL4A3	-0.055587	0.046581
[892]	PARD6A	<---	PRKCG	-0.38466	0.36737
[893]	HNRNPK	<---	HMGB1	-0.20261	0.23588
[894]	RALYL	--->	ZNF408	0.12758	-0.09444
[895]	AURKB	--->	BIRC5	0.65723	-0.69965
[896]	KCNJ12	<---	DLG3	-0.04695	0.04551
[897]	GRK1	--->	CALM1	0.89196	-0.90051
[898]	EPB41L2	<---	ADORA1	-0.19189	0.19515
[899]	SERPINE1	<---	ELANE	-0.082185	0.11833
[900]	PCSK5	--->	AMH	0.070128	-0.071189
[901]	RAF1	<---	HSPA8	-0.29539	0.27279
[902]	ABL2	--->	CAT	-0.040953	-0.055538
[903]	PPID	<---	HSP90AB1	-0.075692	0.072506
[904]	ATF7	--->	FOS	0.020012	-0.10183
[905]	NTRK1	<---	RAP1A	-0.075466	0.066081
[906]	FHOD1	--->	PFN2	0.058717	-0.044377
[907]	SCYL2	<---	ZFYVE9	-0.039735	0.024063
[908]	DLG3	--->	GRIN2B	0.044742	-0.055228
[909]	HDAC2	--->	ARID4A	0.036281	-0.031925
[910]	YWHAG	--->	BRAF	0.18212	-0.19664

[911]	TP53BP2	<---	WWP2	-0.21346	0.20217
[912]	MMP9	<---	EPHB2	-0.30455	0.29717
[913]	MYB	<---	BTRC	-0.079146	0.066274
[914]	HDAC1	<---	MDM2	-0.07736	0.07009
[915]	MAPKAPK5	--->	MYH11	0.026584	-0.057984
[916]	PTPN11	--->	NTRK1	0.5252	-0.37904
[917]	GNAI3	--->	CD59	0.12223	-0.11176
[918]	ATXN1	<---	RBM9	-0.032318	0.028442
[919]	CASP3	--->	PRKCQ	0.20957	-0.1791
[920]	SNX2	--->	PDGFRA	0.11348	-0.11276
[921]	NPY	--->	MC4R	0.69497	-0.69821
[922]	IRS2	<---	SOCS7	-0.13554	0.14262
[923]	CCBP2	--->	CCL13	0.49595	-0.46475
[924]	PPP1R12C	<---	IL16	-0.34616	0.3596
[925]	EFEMP2	--->	FAM115A	0.026076	-0.011549
[926]	BCAR1	<---	PTPRH	-0.12508	0.12059
[927]	YWHAG	--->	TP53BP2	0.29837	-0.30215
[928]	ZNF202	--->	ZNF473	-0.034638	-0.036331
[929]	YWHAB	--->	CDC25B	0.14141	-0.11975
[930]	CRIP2	<---	PTPN13	-0.18144	0.17025
[931]	NR0B2	<---	DDX20	-0.013794	0.018482
[932]	ARF6	--->	ZNF709	0.44745	-0.44939
[933]	LPAR1	--->	ARHGEF12	0.10316	-0.106
[934]	MAGI2	<---	ACVR2A	-0.084547	0.074641
[935]	TJP2	--->	EPB41	0.00019781	-0.0065433
[936]	SFRS9	--->	NOL3	0.095369	-0.06384
[937]	NCOR2	--->	RXRG	0.080576	-0.1102
[938]	MXD1	<---	CD2	-0.30885	0.32042
[939]	DNMBP	--->	DBN1	0.032317	-0.049767
[940]	BRSK1	<---	HSPA1A	-0.61156	0.61048
[941]	PIK3R1	<---	CDC42	-0.30832	0.21
[942]	ACVR1	--->	THRB	0.15721	-0.17116
[943]	C2	<---	C3	-0.067025	0.066153
[944]	VAV3	<---	GRB2	-0.11041	0.2794
[945]	FZD5	<---	ROR2	-0.13628	0.064759
[946]	ZNF408	<---	DDX56	-0.068065	0.084358
[947]	CAV1	--->	AR	0.95431	-0.95522
[948]	SLA	<---	CBL	-0.58879	0.57629
[949]	GHR	<---	PTPN11	-0.32057	0.36347
[950]	PPIA	<---	PRSS23	-0.0038535	0.039822
[951]	CDY1	--->	HIST2H2AC	0.24617	-0.19484
[952]	PLAT	--->	CALR	0.025185	-0.026653
[953]	LCK	--->	CD3E	1.1653	-1.1037
[954]	GZMB	<---	CASP3	-0.70957	0.59854
[955]	CCR5	<---	JAK1	-0.53456	0.52384
[956]	YES1	--->	DOK1	1.3209	-1.1853
[957]	ISL1	<---	RLIM	-0.061039	0.057332
[958]	PTPN6	--->	FLT3	0.6638	-0.42068
[959]	INSR	<---	SOCS6	-0.046398	0.025183
[960]	CD3E	--->	CD79B	-0.1007	-0.24337
[961]	GRIN2B	<---	EPHB2	-0.13004	0.11848
[962]	SHC1	--->	VAV1	0.055435	-0.064453
[963]	CBX4	<---	CTBP1	-0.061494	0.055286
[964]	MDM2	--->	COPS5	0.075398	-0.080248
[965]	CRK	--->	STAT5B	0.32804	-0.36563
[966]	PORCN	--->	WNT6	1.0005	-0.9854
[967]	CASP3	--->	ATN1	0.053098	-0.077022
[968]	CAMK2B	--->	ETS1	0.68344	-0.68792
[969]	RAN	--->	SMAD1	0.52759	-0.54836
[970]	NFKB1	<---	BTRC	-0.99914	1.0055
[971]	SHANK1	<---	BAI2	-0.011039	0.0011605
[972]	DTNB	<---	HMG20A	-0.16299	0.1963
[973]	PARD6A	--->	PLCB3	0.11106	-0.11924
[974]	ELK1	--->	ID2	0.12676	-0.084944
[975]	CHUK	--->	RELA	1.0019	-0.99319
[976]	TRIP10	--->	RXRA	0.062438	-0.20049
[977]	RNPS1	?	TRA2B	-0.016298	-0.016298
[978]	PLK3	--->	CIB1	0.46574	-0.47258
[979]	GZMB	<---	CASP7	-0.70957	0.59854
[980]	PLSCR1	--->	ZNF764	0.282	-0.28342
[981]	PELI2	<---	IRAK4	-0.44131	0.48758
[982]	MAPK14	--->	PLA2G4A	0.31934	-0.41482
[983]	MTNR1B	<---	PPM1G	-0.059863	0.092991
[984]	MAP2K4	<--->	MAPK8	0.23429	0.23429
[985]	RHOJ	--->	SMAD2	0.55322	-0.47159
[986]	SNX17	--->	LRP8	0.14342	-0.14834

[987]	ARHGAP8	--->	BNIP2	0.017753	-0.0078982
[988]	SKIV2L2	<---	SP1	-0.09914	0.087588
[989]	GRB2	--->	APP	0.22575	-0.23734
[990]	TBL1XR1	--->	HIST2H2BE	0.043907	-0.034106
[991]	SYTL5	<---	RAB27A	-0.15731	0.20092
[992]	SPINT1	<---	FAM46A	-0.022494	0.037833
[993]	GNAQ	<---	ADRBK1	-0.63219	0.60102
[994]	ARF6	<---	CAPN1	-0.16151	0.16
[995]	PLEKHA8	<---	ARF1	-0.18769	0.20203
[996]	HSPA5	--->	BCAR1	0.094716	-0.091118
[997]	SLC2A4	<---	RAB7A	-0.9951	0.99602
[998]	SLC9A3R1	<---	PDGFRB	-0.04425	0.038282
[999]	GNAI3	<---	DRD2	-1.008	1.0129
[1000]	BTC	--->	EGFR	1.005	-1.0102
[1001]	ABL1	<---	ROS1	-0.11483	-0.0085826
[1002]	MEF2A	--->	MYOG	0.060707	-0.059789
[1003]	YWHAG	--->	PABPC1	0.092231	-0.070624
[1004]	CCL14	<---	CCR3	-0.46475	0.49595
[1005]	SH3KBP1	--->	PIK3CA	0.49295	-0.49331
[1006]	JAK2	--->	IFNGR2	0.071257	-0.0029847
[1007]	ATG12	<---	PLSCR1	-0.11032	0.13373
[1008]	BCL2	<---	HSPA1A	-1.2535	1.266
[1009]	CTSD	--->	APP	0.22815	-0.23983
[1010]	HDAC2	--->	YY1	0.062791	-0.064081
[1011]	CCR8	--->	CCL4	0.49595	-0.46475
[1012]	ACTL6A	<---	TRRAP	-0.02975	0.019618
[1013]	MAP4K1	--->	LAT	0.35716	-0.30176
[1014]	ARHGEF11	<---	GNA12	-0.047502	0.041194
[1015]	ASH2L	<---	TGFBR1	-0.37529	0.34137
[1016]	DLGAP3	<---	PTK2B	-0.12257	0.017126
[1017]	MAPK1	--->	BCL2	1.1791	-1.2078
[1018]	NCK2	--->	CPSF6	0.086771	-0.089753
[1019]	MKI67	<---	PRKCB	-0.90971	0.9113
[1020]	NXF1	--->	SFRS1	0.042109	-0.011155
[1021]	POU2F1	<---	HMGB2	-0.26954	0.30083
[1022]	PTPN21	<---	TEC	-0.10123	0.058658
[1023]	TLR4	<---	TLR5	0.022065	0.026872
[1024]	ATN1	--->	ATRX	0.0084235	-0.020629
[1025]	RPTOR	<---	RPS6KB1	-0.088674	0.072711
[1026]	CNTN1	--->	TNR	-0.0001877	-0.013976
[1027]	ARRB2	--->	DVL2	0.23088	-0.2363
[1028]	EP300	--->	PPARA	0.17826	-0.18496
[1029]	NCOR1	--->	NR1H2	0.063164	-0.065644
[1030]	BMPR1B	<---	RAP2A	-0.53994	0.52577
[1031]	RB1	<---	HMGB1	-0.52236	0.52072
[1032]	RACGAP1	--->	VAV1	-0.0066478	-0.09519
[1033]	FGF7	<---	COL3A1	-0.033306	0.021987
[1034]	ZBTB32	<---	ZBTB16	-0.05489	-0.016354
[1035]	YWHAG	--->	RAB11FIP5	0.029447	-0.01637
[1036]	PIK3R1	<---	CD22	-0.11528	0.082172
[1037]	PRKCA	--->	F11R	0.35191	-0.36094
[1038]	NF2	--->	SPTBN1	0.036087	-0.045272
[1039]	TNR	<---	SIGLEC1	-0.018925	0.0039575
[1040]	NCOR2	--->	NR2F1	0.13736	-0.14734
[1041]	CAT	<---	TLR10	-0.17733	0.17916
[1042]	HDAC5	<---	NCOR1	-0.048781	0.038408
[1043]	TYK2	--->	IRS1	0.28435	-0.30267
[1044]	PRKCD	--->	GRM5	0.78537	-0.77559
[1045]	NCS1	<---	DTX2	-0.12829	0.12358
[1046]	CCNH	--->	TP53	0.22407	-0.21285
[1047]	CREBBP	--->	HNF1B	0.18505	-0.18943
[1048]	SREBF2	<---	MAPK1	-0.54127	0.54159
[1049]	CATSPER1	--->	ADAMTSL4	0.015537	-0.0016176
[1050]	NR2C2	<---	MAP3K7IP2	-0.076756	0.078604
[1051]	MAPK1	--->	RGS19	0.28697	-0.32153
[1052]	NPHS1	--->	IQGAP1	0.053754	-0.07178
[1053]	PACSIN3	--->	DNM1	0.082421	-0.091965
[1054]	SRC	--->	RAF1	0.45157	-0.19021
[1055]	MMP14	<---	LUM	-0.18966	0.20515
[1056]	PRKD1	--->	JUN	0.66069	-0.68658
[1057]	NPNT	--->	ITGA8	0.017926	-0.029633
[1058]	TYK2	<---	LYN	0.081386	0.19726
[1059]	FYB	--->	WAS	0.022086	-0.048524
[1060]	TLR6	--->	TRAP1	0.31346	-0.28434
[1061]	KCNG3	?	KCNC1	-0.025901	-0.025901
[1062]	SKIL	<---	SVEP1	-0.014381	0.0014223

[1063]	ASAP1	<---	PTK2	-0.078275	0.013981
[1064]	MMP13	<---	BCAN	-0.030194	0.022421
[1065]	CXCR2	--->	ARRB1	0.27159	-0.25831
[1066]	HMGB1	<---	TLR4	-0.38564	0.28647
[1067]	NR0B2	--->	ESRRG	-0.0094276	-0.028437
[1068]	IRAK1	--->	NLRP12	0.23255	-0.14777
[1069]	CSF2RA	--->	PIK3R1	0.59477	-0.64077
[1070]	LINGO1	<---	NGFR	0.025472	0.034571
[1071]	ABL1	<---	ROBO1	-0.091115	-0.018117
[1072]	DAXX	<---	HDAC2	-0.11277	0.090678
[1073]	DCN	<---	COL1A2	-0.030712	0.023329
[1074]	ESPN	<---	BAIAP2	-0.019086	0.0074466
[1075]	CABLES1	--->	TP53	0.22407	-0.21285
[1076]	KRT15	<---	LNX1	-0.0075088	0.012616
[1077]	NUMB	<---	PRKCE	-0.42361	0.37732
[1078]	LAMA5	--->	FBLN2	0.0053173	-0.012578
[1079]	CBX1	<---	PIM1	-0.72609	0.70015
[1080]	PKP2	--->	DSG1	0.31989	-0.319
[1081]	SH3BP1	<---	SRC	-0.53307	0.62164
[1082]	ABCD1	?	ABCD3	-0.062455	-0.062455
[1083]	CXCR2	--->	GNAI3	1.0129	-1.008
[1084]	HOMER1	<---	AGAP2	-0.20917	0.19857
[1085]	CXCR2	<---	PPP2CA	-0.42681	0.42413
[1086]	CDC25C	--->	CCNB1	0.33489	-0.3246
[1087]	RHEBL1	--->	TGFBR1	0.010625	-0.037483
[1088]	ADCY5	<---	RGS2	-0.57834	0.59103
[1089]	NFATC4	<---	YWHAZ	-0.073365	0.077401
[1090]	WWP2	--->	CLCN5	0.17906	-0.18874
[1091]	FBLN1	--->	ITGB1	0.13683	-0.146
[1092]	TLX1	<---	NFIC	-0.034621	0.041669
[1093]	TNKS	<---	TERF1	-0.024514	0.041759
[1094]	MTNR1A	--->	IRS4	0.22622	-0.22287
[1095]	KIDINS220	<---	NTRK1	-0.15616	0.14962
[1096]	GSK3B	--->	TP53	1.0002	-0.99962
[1097]	SKAP2	<---	TGFBR1	-0.14413	0.11074
[1098]	PDGFRB	--->	PIK3R1	0.14272	-0.16309
[1099]	PRKCD	--->	ITGB4	0.10097	-0.11176
[1100]	TBC1D4	<---	EP400	-0.0072929	-0.0039455
[1101]	HTR2A	<---	RPS6KA3	-0.20834	0.066253
[1102]	POU5F1	--->	ETS2	0.19026	-0.17089
[1103]	CHRNA7	--->	APP	0.36273	-0.37269
[1104]	PARK2	<---	DLG1	-0.0074553	0.0058561
[1105]	TYRO3	--->	SRC	0.065624	-0.057684
[1106]	PRKCA	--->	SHC1	0.15385	-0.17634
[1107]	SRC	--->	DAPP1	1.2396	-0.79845
[1108]	CTNNB1	<---	FOXO1	-0.083934	0.072
[1109]	ARHGEF6	<---	PAK3	-0.0010098	0.035613
[1110]	HABP2	<---	COL4A5	-0.012629	-0.00035712
[1111]	PTK2	<---	VCL	-0.17303	0.063816
[1112]	TRAF6	--->	ZFAND5	0.65723	-0.66545
[1113]	NCK1	--->	CD3E	0.47291	-0.49149
[1114]	GUCA1B	<---	GUCY2D	-0.25699	0.2459
[1115]	LIN7B	<---	CASK	-0.029385	0.030418
[1116]	RPS6KA5	--->	CREBBP	0.11396	-0.12955
[1117]	WDR1	<---	CFL1	-0.044668	0.059031
[1118]	RPS6KB2	--->	RPS6	0.98649	-0.99602
[1119]	ARHGEF7	--->	YWHAG	0.028761	-0.035837
[1120]	GFRA2	--->	GDNF	0.39482	-0.35487
[1121]	GFI1B	<---	BEGAIN	-0.15507	0.1511
[1122]	HDAC5	<---	CBX5	-0.036579	0.043335
[1123]	PPIL2	<---	CRNKL1	0.015514	0.021213
[1124]	POU3F4	--->	POU3F2	-0.034777	-0.056938
[1125]	AKT2	--->	FOXO4	0.99774	-1.0046
[1126]	USP7	--->	TRAF3	0.00096766	-0.051516
[1127]	CSNK2A1	--->	NOL3	1.0341	-1.0599
[1128]	RGS4	--->	CALM1	0.78674	-0.75366
[1129]	DNM2	<---	PFN1	-0.016995	0.010117
[1130]	RGS20	--->	GNAZ	1.0012	-1.0015
[1131]	TRAF2	--->	CAV1	0.14448	-0.14018
[1132]	ABCG4	--->	ABCG1	-0.030012	-0.052336
[1133]	CHD9	--->	PPARA	-0.0054507	-0.0084696
[1134]	COL17A1	--->	PLEC1	0.02229	-0.025292
[1135]	CTNNB1	<---	CDK6	-0.99713	0.99917
[1136]	PLD2	<---	ALDOC	-0.46543	0.46244
[1137]	MAPK14	--->	NUP153	0.28084	-0.45087
[1138]	RAPGEF1	<---	CRK	-0.99825	0.99873

[1139]	DNM1	<---	PFN2	-0.016995	0.010117
[1140]	CXCL1	<---	CXCR1	-0.46475	0.49595
[1141]	ATP2B1	<---	CALM1	-0.32324	0.31963
[1142]	SV2A	<---	LAMA5	-0.081043	0.076104
[1143]	KCNJ6	<---	ADRB2	-0.21396	0.22725
[1144]	PRKCD	--->	INSR	0.097293	-0.10679
[1145]	SHC1	<---	SOS1	-0.04276	0.014124
[1146]	RNF11	--->	EPN3	0.19496	-0.16235
[1147]	IKBKE	--->	TICAM1	0.025166	-0.17891
[1148]	RPS6KB1	<---	STK11	-0.74727	0.78966
[1149]	ZFPM1	<---	CTBP2	-0.086004	0.077682
[1150]	HSPH1	--->	CFL1	0.54043	-0.52045
[1151]	TIAM1	<---	SRC	-0.20986	0.21804
[1152]	ARF6	--->	ARRB2	0.27431	-0.26383
[1153]	AKT1	--->	SMAD2	0.26467	-0.21636
[1154]	SH3BP2	--->	TF	-0.041022	-0.11123
[1155]	PRPF40A	--->	SMAD4	0.82515	-0.75325
[1156]	IRAK1	--->	RIPK2	0.33548	-0.11815
[1157]	CDYL	<---	HDAC1	-0.014369	0.030032
[1158]	WDR16	<---	HSPA1A	-0.93955	0.98052
[1159]	PTPRH	--->	PXN	0.72139	-0.75353
[1160]	PEX2	--->	TLE1	0.15492	-0.12118
[1161]	DIXDC1	--->	MAP3K4	0.18526	-0.19582
[1162]	ASAP1	<---	HCK	-0.17411	0.43807
[1163]	MYT1	<---	CDK1	-0.44897	0.42777
[1164]	FYN	--->	FYB	0.99663	-1.0021
[1165]	COL17A1	--->	ACTN1	0.0026579	-0.012425
[1166]	CDC42EP5	<---	CDC42	-0.87115	0.57849
[1167]	TEX11	--->	MEF2B	0.064957	-0.049439
[1168]	TRAF2	--->	ZNF646	0.081031	-0.086733
[1169]	GNB2L1	--->	DNM1	0.064919	-0.072447
[1170]	THBD	<---	PROC	-0.024434	-0.021578
[1171]	BRD7	<---	PTPN13	-0.16718	0.15669
[1172]	PDZK1	--->	CFTR	0.48971	-0.48738
[1173]	CXCL13	<---	CXCR3	-0.46475	0.49595
[1174]	NCK2	--->	PKN2	0.0066868	-0.042577
[1175]	NCK1	--->	SOS1	0.62201	-0.62279
[1176]	UBQLN4	--->	ATXN1	0.04507	-0.048402
[1177]	AR	<---	FOXO1	-0.1398	0.12761
[1178]	SMARCD3	--->	ESR1	0.052806	-0.042542
[1179]	ERBB4	--->	GRB2	1.0161	-1.0143
[1180]	IGFBP5	<---	F2	-0.013006	0.028162
[1181]	NOXA1	<---	RAC2	-0.13569	0.12502
[1182]	PXN	--->	ITGB1	0.062802	-0.074544
[1183]	SH3BP2	--->	PLCG2	0.18655	-0.3092
[1184]	MAP3K7IP1	<---	MAPK14	-0.16848	0.0040406
[1185]	MAPK1	<---	DUSP5	-1.0004	1.0016
[1186]	RFX1	--->	NFIX	0.051672	-0.054792
[1187]	CHRM3	<---	ARF1	-0.055567	0.063764
[1188]	GGA1	--->	SMAD3	0.41137	-0.32841
[1189]	HSPA9	--->	FGF1	0.87594	-0.83434
[1190]	RASGRF1	<---	USP8	-0.27948	0.27678
[1191]	MSC	--->	TCF3	-0.13552	-0.39218
[1192]	NR1H2	?	RXRB	-0.0089162	-0.0089162
[1193]	CHUK	--->	PIAS1	1.0033	-1.0029
[1194]	BIRC2	--->	LTBR	0.17585	-0.18475
[1195]	SYNGAP1	<---	DLG4	-0.005916	-0.0033168
[1196]	BIN1	<---	ABL1	-0.062745	-0.03798
[1197]	WWP1	--->	IL6R	0.10216	-0.11484
[1198]	IRAK2	<---	IL1R1	-0.31799	0.36073
[1199]	MINK1	--->	YWHAB	0.066249	-0.065987
[1200]	CDKN1B	<---	CDK5	-0.36687	0.34497
[1201]	PDGFRB	--->	PLCG1	0.15011	-0.17301
[1202]	IL10RA	--->	IL10RB	-0.18165	-0.76164
[1203]	BCAR1	<---	DOCK1	-0.0073743	-0.0062901
[1204]	BEGAIN	--->	ZNF250	0.28459	-0.2837
[1205]	GNB2L1	<---	IL4R	-0.071034	0.085537
[1206]	INSRR	--->	INSR	-0.011388	-0.023675
[1207]	HNRNPC	<---	PHKB	0.0038767	0.027727
[1208]	SSTR3	--->	GNAI1	1.0129	-1.008
[1209]	NCOA3	<---	RARG	-0.13412	0.12213
[1210]	KRT15	<---	SMARCD1	-0.012236	0.016085
[1211]	ZDHHC17	<---	DLG4	-0.083347	0.077256
[1212]	STAMBP	--->	SMAD7	0.99487	-0.99775
[1213]	IL1RL2	--->	IL18	0.20447	-0.18852
[1214]	CREBBP	--->	NKX2-1	0.18505	-0.18943

[1215]	PRKCD	--->	KCNJ1	0.81962	-0.8066
[1216]	SMURF2	--->	TFPI2	0.70765	-0.71152
[1217]	PLK1	--->	NUDC	0.29017	-0.31041
[1218]	CTNNA1	--->	ACTN1	0.19265	-0.19198
[1219]	CEACAM1	<---	EGFR	-1.0042	0.99948
[1220]	FBLN1	--->	GFI1B	0.14936	-0.1532
[1221]	CSF2RB	<---	CISH	-0.28704	0.24417
[1222]	BCL2	--->	ATP2A2	0.020999	-0.0298
[1223]	TEAD4	<---	CCDC85B	0.0027999	0.026313
[1224]	TRIM55	<--->	TRIM63	0.15517	0.15517
[1225]	FOS	<---	CSNK2A2	-0.68783	0.53871
[1226]	ARNTL	<---	HLF	-0.17145	0.18231
[1227]	CALCOCO2	--->	GABARAPL2	0.12444	-0.09126
[1228]	GOPC	--->	CFTR	0.48971	-0.48738
[1229]	HMOX2	<---	COPS6	-0.21996	0.24623
[1230]	RBBP7	<---	BRCA1	-0.10329	0.11835
[1231]	PICK1	<---	PRLHR	-0.2531	0.26799
[1232]	UBQLN4	--->	NOTCH2NL	0.005392	-0.018601
[1233]	MST1R	--->	YWHAZ	0.39852	-0.41561
[1234]	KRT15	<---	KRT6A	-0.017396	0.033003
[1235]	TGFB1I1	--->	GIT2	0.03579	-0.047634
[1236]	MAPK11	--->	ATF2	1.0147	-1.0156
[1237]	LGALS3BP	<---	COL6A1	-0.055405	0.04984
[1238]	SRC	--->	GJA1	0.24586	-0.22975
[1239]	GNA14	<---	CCR1	-1.008	1.0129
[1240]	TEX11	--->	NECAB2	0.28288	-0.26661
[1241]	EIF2C1	<--->	EIF2C2	0.067101	0.067101
[1242]	CBX5	<---	ARL5A	-0.43058	0.45692
[1243]	SOS1	<---	HCK	-0.71705	0.76941
[1244]	GTF2I	--->	PRRX1	0.18189	-0.17174
[1245]	PRKCD	--->	MEP1B	0.89746	-0.88667
[1246]	GRASP	<---	DLG4	-0.018249	0.0028049
[1247]	MAPK8IP2	--->	LRP2	0.25269	-0.25877
[1248]	CCNA1	<---	CDKN1A	-0.99961	0.98767
[1249]	PIN1	--->	CTNNB1	0.50504	-0.50385
[1250]	TNFSF15	--->	TNFRSF6B	0.45861	-0.52454
[1251]	SETDB1	<---	TRIM16	-0.15253	0.15644
[1252]	ESRRA	--->	LYST	0.034681	-0.0096005
[1253]	CDH1	<---	IRS1	-0.12369	0.11954
[1254]	CXCL9	<---	CCR3	-0.46475	0.49595
[1255]	SPTB	<---	SPTA1	-0.036462	0.0050181
[1256]	EGFR	<---	LYN	0.43592	0.45517
[1257]	RAC1	--->	MAP3K4	0.54389	-0.54804
[1258]	MSX1	--->	TP53	0.32768	-0.31703
[1259]	SFRS7	<---	DUSP11	-0.68308	0.71953
[1260]	MCF2	<---	RHOG	-0.017594	0.0050659
[1261]	ARNTL	<---	HSP90AA1	-0.055793	0.044236
[1262]	EMILIN2	<---	EMILIN1	-0.0038098	0.022142
[1263]	TBP	<---	EMID2	-0.0087759	-0.0045239
[1264]	ABI1	<---	NCF1	0.01418	0.027374
[1265]	SIT1	<---	CSK	-1.0988	1.2193
[1266]	TGIF2	<---	HDAC1	-0.15464	0.16287
[1267]	GIT1	<---	PAK3	-0.19118	0.24364
[1268]	RBX1	<---	CUL1	-0.11034	0.12225
[1269]	CNR2	--->	GNA15	1.0129	-1.008
[1270]	CBX1	<---	DNMT1	-0.039457	0.054459
[1271]	CSK	<--->	FGR	0.37153	0.37153
[1272]	HDAC5	--->	GATA1	0.048777	-0.036921
[1273]	GRIN2D	--->	CTNNB1	0.11041	-0.12087
[1274]	H3F3B	<---	HIRA	-0.057829	0.10803
[1275]	TSHR	<---	JAK1	-0.32617	0.31425
[1276]	NTRK1	--->	PLCG1	0.36984	-0.47812
[1277]	PLCG1	<---	NTRK2	-0.29514	0.22525
[1278]	NCOA1	<---	CREBBP	-0.033602	0.020953
[1279]	MAP2K1	--->	PRKCI	0.13614	-0.092025
[1280]	GPR77	--->	C4A	0.23997	-0.22186
[1281]	PTK2B	--->	ITGB3	0.016172	-0.050274
[1282]	ARHGEF11	<---	GNA13	-0.047502	0.041194
[1283]	HIST1H4F	?	CENPA	-0.23226	-0.23226
[1284]	FZD7	<---	UBQLN4	-0.33534	0.3099
[1285]	CREBBP	--->	STAT4	0.89453	-0.90452
[1286]	UBE2N	<--->	UBE2V2	0.21837	0.21837
[1287]	CDH5	--->	SHC1	0.081713	-0.18309
[1288]	SATB1	<---	UBE2I	-0.055958	0.060247
[1289]	SOX9	<---	CREB1	-0.011624	-0.00012846
[1290]	GRK5	--->	TBXA2R	0.31971	-0.40764

[1291]	CSF2RB	<---	LCK	-1.0066	1.0034
[1292]	BACE1	--->	RANBP9	0.1222	-0.10805
[1293]	HSPA8	--->	STMN1	0.98516	-0.93289
[1294]	ISL1	<---	LMO2	-0.0072988	0.037594
[1295]	ADAM17	--->	FHL2	0.047604	-0.042593
[1296]	PIN1	--->	ZMIZ2	0.25968	-0.2286
[1297]	FGF2	<---	CSNK2A2	-0.75902	0.73258
[1298]	GGA1	--->	SORL1	0.069724	-0.076758
[1299]	EGFR	--->	MUC1	0.1949	-0.20625
[1300]	JAK1	--->	INPP5D	0.99645	-0.99913
[1301]	SMAD3	<---	FOXO4	-0.27389	0.2939
[1302]	TMPRSS6	<---	COL1A1	-0.018741	0.0070247
[1303]	EP300	--->	SUV39H1	0.063953	-0.074254
[1304]	ABCA1	<---	PDZK1	-0.0080068	0.005539
[1305]	AKAP10	--->	PDZK1	0.44344	-0.41116
[1306]	TGFB2	--->	CTGF	0.18381	-0.19139
[1307]	TGM2	--->	RELA	0.10819	-0.10245
[1308]	PKN2	<---	RHOA	-0.17601	0.06529
[1309]	SFRS11	?	RNPS1	-0.016298	-0.016298
[1310]	BATF	--->	FOS	0.50561	-0.71943
[1311]	ESR2	<---	PIAS3	-0.090133	0.095399
[1312]	ACVR2B	<---	ACVR1B	-0.062144	-0.022886
[1313]	GRB2	--->	CD72	0.61127	-0.65703
[1314]	RAB25	--->	SMAD4	0.55322	-0.47159
[1315]	TRAF2	<---	LTBR	-0.18595	0.18142
[1316]	PSMD4	--->	TCF3	0.37045	-0.37298
[1317]	PTPN6	<---	PTPRC	0.21056	0.4777
[1318]	PLCG1	<---	DNM1	-0.20353	0.16769
[1319]	PPARD	<---	NCOR1	-0.065644	0.063164
[1320]	RASSF5	<---	RALA	-0.98684	1.0025
[1321]	ZXDC	<---	RORA	-0.049739	0.052753
[1322]	GAB1	--->	MAP3K3	0.020577	-0.01387
[1323]	RANBP1	<---	RAN	-0.42541	0.45599
[1324]	COL23A1	--->	FURIN	0.0085239	-0.019941
[1325]	GRM8	<---	FLNA	0.0058519	0.028788
[1326]	GSK3B	--->	PXN	0.57323	-0.59278
[1327]	CFL1	<---	CLCN5	-0.47989	0.48158
[1328]	CSNK2A1	--->	EGR1	0.91941	-0.80386
[1329]	SERPINE1	--->	LRP1	0.14257	-0.15161
[1330]	PI4K2B	--->	CASR	0.037583	-0.020609
[1331]	MXI1	--->	MYC	-0.23569	-0.31161
[1332]	CAMK2A	--->	ATP2A2	0.41881	-0.42746
[1333]	SP1	--->	TBP	-0.0011161	-0.012669
[1334]	DOCK7	<---	YWHAG	-0.020276	0.0094759
[1335]	AR	<---	KAT2B	-0.99994	1.0101
[1336]	RAPGEF1	--->	NEDD9	0.033557	-0.044586
[1337]	ITGA6	<---	COL17A1	-0.011399	-0.0012541
[1338]	FOXD3	--->	POU5F1	0.25332	-0.24129
[1339]	SIT1	<---	PTPN11	-0.65515	0.8369
[1340]	CREBBP	--->	SOX9	0.013199	-0.026396
[1341]	HIPK1	--->	DAXX	0.3655	-0.48387
[1342]	HDAC3	<---	MAPK11	-0.53422	0.40963
[1343]	GIT1	<---	TRIB3	-0.35245	0.33727
[1344]	SPI1	--->	JUN	0.3457	-0.38611
[1345]	IL6R	<---	CNTF	-0.24391	0.24598
[1346]	PRKCZ	--->	IKBKB	0.25124	-0.25473
[1347]	LASP1	--->	ACTC1	0.38007	-0.38414
[1348]	CAPNS1	--->	ARHGEF6	-0.0023301	-0.0099893
[1349]	KIF13B	<---	DLG1	-0.028391	0.020395
[1350]	NRAP	--->	ACTN3	0.069648	-0.053191
[1351]	USP7	<---	HECW1	-0.19559	0.18745
[1352]	BCL10	<---	SOCS3	-0.025048	0.040924
[1353]	AKT1	--->	HIST2H2BE	0.73459	-0.50063
[1354]	WIPF2	--->	WAS	-0.010099	-0.20693
[1355]	CHD3	<---	SGSM2	-0.0079511	-0.0045276
[1356]	SOCS1	<---	ITK	-0.12165	0.27706
[1357]	HIST3H3	<---	CBX5	-0.19484	0.24617
[1358]	DMBX1	<---	OTX2	-0.19656	-0.024292
[1359]	MAPK8IP1	--->	APP	0.21795	-0.22947
[1360]	CALM1	--->	SCTR	0.25258	-0.24765
[1361]	CSNK2A1	--->	F11R	0.090263	-0.12115
[1362]	MAPK3	<---	MAP2K1	-0.99672	1.0159
[1363]	GPR50	<--->	MTNR1B	0.49511	0.49511
[1364]	RNF141	<---	SMURF1	-0.68612	0.68206
[1365]	CFLAR	--->	RIPK2	0.099928	-0.099851
[1366]	KCNJ2	--->	FLNA	0.024298	0.01061

[1367]	TSC2	<---	YWHAH	-0.29138	0.294
[1368]	HIPK3	--->	ARRB2	0.42184	-0.44306
[1369]	BCL2L10	<---	BAD	-0.28245	0.2727
[1370]	CASP8	--->	PRKCZ	0.12685	-0.1345
[1371]	KAT5	<---	NFKB1	-0.47035	0.46975
[1372]	SRC	--->	CAV1	0.99937	-1.0053
[1373]	SPOCK2	--->	TES	0.05794	-0.022505
[1374]	DLX5	?	MSX2	-0.11417	-0.11417
[1375]	DOK1	<---	CRKL	-0.2152	0.17696
[1376]	PKN3	<---	ARHGAP26	-0.077099	-0.019941
[1377]	FBXL18	--->	PLSCR1	0.13373	-0.10619
[1378]	PPM1L	--->	MAP3K7	0.99989	-0.99428
[1379]	MYLK	<---	SRC	-0.42782	0.40383
[1380]	TRAF5	<---	TNFRSF8	-0.1819	0.17914
[1381]	NFASC	--->	TNR	-0.0001877	-0.013976
[1382]	CBL	--->	STAT5A	0.16839	-0.18275
[1383]	EVL	<---	PFN2	-0.023349	0.039151
[1384]	NR3C2	<---	TRIM24	-0.088819	0.11471
[1385]	YWHAZ	<---	CDK17	-0.66368	0.62776
[1386]	DOK5	<---	IGF1R	-0.4366	0.42474
[1387]	NR4A2	<---	CDKN1C	-0.035895	0.060797
[1388]	HRAS	<---	TLR2	-1.002	0.99581
[1389]	RAGE	--->	CCNB1	1.0544	-1.0774
[1390]	TRRAP	--->	E2F4	-0.0011211	-0.0088663
[1391]	FOS	<---	MITF	-0.083597	0.02761
[1392]	TP53	<---	PCDHA4	-0.33677	0.34817
[1393]	CDK3	--->	CDKN1A	0.51838	-0.55156
[1394]	FMR1	--->	NCL	0.048616	-0.046056
[1395]	SLC9A3R2	--->	ACTN4	0.0092631	-0.0038511
[1396]	PRKCH	--->	FYN	0.15252	-0.11897
[1397]	TP53BP2	--->	APP	0.20875	-0.22066
[1398]	CAPN1	--->	SYNE1	0.16545	-0.1707
[1399]	MPRIP	--->	PPP1R12A	0.046309	-0.04691
[1400]	USP13	<---	FNBP1L	-0.071162	0.0664
[1401]	CD3G	?	CD3E	-0.22052	-0.22052
[1402]	CEBPB	?	CEBPG	-0.11448	-0.11448
[1403]	SVIL	--->	SPTBN1	0.14692	-0.14324
[1404]	PIK3R1	<---	EPOR	-0.3033	0.24162
[1405]	BCAR1	--->	FYB	0.16978	-0.048534
[1406]	KAT5	--->	LRP1	0.087238	-0.098265
[1407]	PHC1	--->	RING1	0.14598	-0.12808
[1408]	NCOA3	<---	NR1H2	-0.13412	0.12213
[1409]	FAS	--->	APAF1	0.1942	-0.089808
[1410]	FHL5	<--->	FHL2	0.029854	0.029854
[1411]	NSD1	--->	AR	0.95919	-0.95969
[1412]	ACAN	--->	MMP3	0.022421	-0.030194
[1413]	NFIC	--->	ZNF167	0.27023	-0.27734
[1414]	TNFRSF14	<---	ESR1	-0.075439	0.070919
[1415]	CBLC	<---	ITCH	-0.057845	0.044852
[1416]	XBP1	--->	FOS	0.28837	-0.41862
[1417]	IL18RAP	--->	IL18	0.20447	-0.18852
[1418]	PLCD1	<---	RALB	-0.30267	0.31625
[1419]	GNAQ	<---	ADRA1B	-0.96014	0.95799
[1420]	EP300	--->	PIAS3	0.059178	-0.069359
[1421]	CACNA1B	<---	GNAO1	-0.20148	0.19171
[1422]	CTBP1	--->	ELK3	0.12637	-0.12659
[1423]	SERPINB13	<---	PRSS1	-0.082185	0.11833
[1424]	PIN1	--->	GGA2	0.32383	-0.29148
[1425]	PPP1CA	--->	FXYD1	0.23928	-0.24521
[1426]	CHEK2	--->	MDM4	0.052648	-0.070925
[1427]	KCNF1	?	KCNV2	-0.025901	-0.025901
[1428]	FN1	<---	HGF	-0.0083667	0.02035
[1429]	INSR	--->	STAT5B	0.13049	-0.14657
[1430]	IL23R	--->	STAT3	0.56217	-0.61544
[1431]	TEX11	--->	SNX7	0.10098	-0.086661
[1432]	SHB	<---	ZAP70	-0.48173	0.48066
[1433]	CDC2L2	--->	CCNL1	0.36339	-0.38189
[1434]	YWHAG	--->	CCNY	0.4622	-0.43862
[1435]	RAB27B	--->	SYTL4	0.20092	-0.15731
[1436]	MAPK8IP2	<---	MAP3K12	-0.30003	0.29445
[1437]	SRC	--->	CAV2	0.99937	-1.0053
[1438]	WWTR1	--->	SLC9A3R2	0.47943	-0.47956
[1439]	SMAD2	<---	ANK3	-0.066496	0.070266
[1440]	PPID	--->	APP	0.36123	-0.37112
[1441]	TBP	<---	MSX1	-0.0086868	-0.0036228
[1442]	SLC8A3	<---	YWHAQ	-0.092105	0.10171

[1443]	JUN	<---	NCOA2	-0.027639	0.0094504
[1444]	RIPK1	<---	TRAF5	-0.31544	0.34056
[1445]	CHD3	--->	BHLHE40	0.02538	-0.036742
[1446]	EPB41L1	<---	DRD2	-0.19189	0.19515
[1447]	CALM1	--->	GLP2R	0.028063	-0.010874
[1448]	BRCA1	<---	CDK7	-0.34057	0.32242
[1449]	GNAO1	<--->	GNAI1	0.17409	0.17409
[1450]	NFKB2	<---	BCL3	-0.023077	-0.0063882
[1451]	LDLRAP1	--->	APP	0.35975	-0.36965
[1452]	CNKSR1	<---	MAP2K7	-0.072389	-0.009116
[1453]	IL1A	--->	IL1R1	0.99815	-1.0071
[1454]	GAK	--->	AR	0.73847	-0.74691
[1455]	SF1	<---	RBPMS	-0.049452	0.063299
[1456]	CAMK2G	--->	SMAD4	1.0038	-1.0107
[1457]	RFX1	--->	HIVEP2	0.069061	-0.072889
[1458]	PTK2	<---	BMX	-0.16631	0.071074
[1459]	WBP4	--->	KHDRBS1	0.21791	-0.21449
[1460]	GSK3B	--->	SNCAIP	1.3062	-1.3222
[1461]	DOK2	<---	NCK1	-0.42803	0.42373
[1462]	MAPK1	--->	TCF3	0.99488	-0.99939
[1463]	PAPPA2	--->	IGFBP3	0.07239	-0.038431
[1464]	HBP1	--->	HDAC1	0.097359	-0.082152
[1465]	FN1	--->	SMAD9	0.26657	-0.2884
[1466]	IL19	--->	IL20RA	0.74479	-0.74442
[1467]	CSF1R	<---	CSF1	-0.99124	0.99624
[1468]	FCGR1A	<---	HCK	0.015127	0.11748
[1469]	RIN1	<---	YWHAB	-0.24433	0.24721
[1470]	MTPN	<---	NFKB1	-0.041138	0.77463
[1471]	PLD1	<---	ACTG1	-0.26381	0.25267
[1472]	FOXP1	--->	CTBP1	0.031737	-0.041734
[1473]	BCL2	<---	NR4A1	-0.98971	0.98871
[1474]	ATR	<---	CHEK2	-0.099248	0.084255
[1475]	TLX3	--->	MEIS1	0.024726	-0.0094151
[1476]	HNRNPM	<---	CEACAM5	-0.19549	0.17582
[1477]	PARD6G	<---	RAC1	-0.18257	0.18762
[1478]	EPB41	--->	SMAD3	0.13543	-0.1135
[1479]	SQSTM1	--->	GABARAPL2	0.1048	-0.10304
[1480]	CBL	--->	PDGFRA	0.056906	-0.068774
[1481]	PECAM1	--->	ITGA5	0.079677	-0.0892
[1482]	MAPRE3	?	MAPRE2	-0.026589	-0.026589
[1483]	EPS8	<---	EGFR	-1.0004	0.99845
[1484]	ESR1	<---	YWHAH	-0.092332	0.11204
[1485]	TNC	<---	PTPRB	-0.012815	-0.0015077
[1486]	KAT2B	--->	ACTN1	0.046908	-0.055206
[1487]	SMAD4	<---	UHRF2	-0.3092	0.3922
[1488]	THRA	<---	RGN	-0.047128	0.072896
[1489]	DRD3	--->	NCS1	0.78966	-0.75527
[1490]	NOS1AP	<---	RASD1	-0.43085	0.46313
[1491]	GNA13	<--->	GNA12	0.17409	0.17409
[1492]	NFAT5	<---	FOXP3	-0.26341	0.27788
[1493]	KAT5	--->	PLA2G4A	0.066448	-0.058578
[1494]	PRKCZ	<---	FYN	-0.0074487	0.025714
[1495]	PDE1A	<---	CALM1	-0.33868	0.36302
[1496]	RGS2	--->	GNAS	1.0012	-1.0015
[1497]	SAG	<---	CHRM2	-0.12587	0.12815
[1498]	PIN1	--->	JUN	0.64702	-0.69342
[1499]	CD209	<---	ICAM3	-0.071641	0.066003
[1500]	GNAI2	<---	C5AR1	-1.008	1.0129
[1501]	HNF4A	--->	NR2F1	0.20637	-0.087134
[1502]	RGS16	--->	GNAI3	1.0012	-1.0015
[1503]	MPP6	<---	SMARCA4	-0.014326	0.0031298
[1504]	HNF4A	--->	UBE2I	0.25235	-0.24324
[1505]	IRS2	<---	NTRK1	-0.11991	0.10794
[1506]	BMPR1B	--->	STAMBP	0.0012705	-0.029928
[1507]	ADAM15	<---	SH3RF1	-0.030333	0.027108
[1508]	RFX1	--->	SMAD1	0.098402	-0.11395
[1509]	MYOT	--->	FLNC	0.12245	-0.091736
[1510]	TLE1	--->	FXYD6	0.020687	0.017299
[1511]	RCOR1	<---	SMARCE1	-0.038038	0.040956
[1512]	DZIP3	<---	NCOR1	-0.02615	0.016811
[1513]	SOCS1	--->	INSR	0.025183	-0.046398
[1514]	LAT	--->	CD4	0.10894	-0.095955
[1515]	ARR3	<---	OPN4	-0.12587	0.12815
[1516]	TMPRSS6	<---	FN1	-0.021177	0.022849
[1517]	RAC2	--->	RTKN	0.24852	-0.24337
[1518]	CIB2	--->	MYOD1	0.2676	-0.22666

[1519]	ITGA8	--->	ITGB1	-0.0090263	-0.014161
[1520]	L3MBTL	--->	HIST4H4	0.25094	-0.22253
[1521]	NCOR2	--->	RARB	0.13736	-0.14734
[1522]	CASP14	<---	CASP10	-0.47769	0.38313
[1523]	IL6ST	<---	TLE1	-0.12219	0.10921
[1524]	TRIM37	--->	FXR2	0.12333	-0.085901
[1525]	NLRP1	--->	CASP5	-0.038798	-0.12583
[1526]	ARVCF	--->	CDH2	0.98604	-0.99605
[1527]	F11R	--->	TJP1	0.069964	-0.080827
[1528]	HCK	--->	KIT	1.0007	-1.0039
[1529]	PAK7	<---	RAC1	-0.9928	0.99291
[1530]	SQSTM1	<---	LCK	-0.47296	0.5166
[1531]	RBBP6	--->	ZBTB38	0.22476	-0.22991
[1532]	DUSP22	--->	MAP2K4	1.011	-0.99819
[1533]	CDKN1B	--->	CCND3	1.0016	-1.0151
[1534]	SERPING1	<---	PLAT	-0.032776	0.037242
[1535]	E2F1	<---	CDK3	-0.46975	0.44723
[1536]	GRB2	--->	LIME1	0.33654	-0.35446
[1537]	SERPINA5	<---	PLAU	-0.040728	0.057207
[1538]	NEK3	--->	VAV2	0.11316	-0.18047
[1539]	CD2AP	<---	NPHS1	-0.041565	0.017634
[1540]	ZFPM2	<---	UBQLN4	-0.091997	0.10926
[1541]	ADAM9	--->	ITGAV	0.004233	-0.017064
[1542]	KAT5	--->	HIST2H4A	0.15564	-0.12976
[1543]	HNRNPC	--->	FXR2	0.036464	-0.0048144
[1544]	PRKACA	--->	ITPKA	0.3157	-0.34471
[1545]	ABCA1	<---	SNTA1	-0.0080068	0.005539
[1546]	KRT6A	<---	CCDC85B	-0.013197	0.042716
[1547]	GRIN2D	<---	CDH2	-0.013618	0.0044774
[1548]	SNIP1	<---	EP300	-0.11427	0.10619
[1549]	RAP1B	<---	RAPGEF1	-0.99301	1.0045
[1550]	MIB2	--->	ACTC1	0.0083908	-0.021159
[1551]	GIPC1	--->	MYO6	0.012543	0.0041759
[1552]	REM1	--->	CACNB1	0.42812	-0.39718
[1553]	PRDM2	--->	ESR1	0.071855	-0.070261
[1554]	JUP	--->	DSC2	0.15626	-0.1649
[1555]	NFKB2	--->	IKBKG	0.0085651	-0.025622
[1556]	PRKDC	--->	JUN	0.16277	-0.18797
[1557]	PRKACA	<---	RAP1B	-1.0074	1.0132
[1558]	EDNRB	--->	GNA13	1.0129	-1.008
[1559]	PECAM1	<---	FYN	0.015127	0.11748
[1560]	IRS2	<---	EPOR	-0.052138	0.042582
[1561]	SMAD1	<---	TRIP6	-0.32822	0.31038
[1562]	FOXA3	--->	HMGB1	0.13776	-0.10769
[1563]	PPP1R15A	<---	HSPA1A	-0.37432	0.39607
[1564]	PKNOX2	<---	HOXB1	-0.0094151	0.024726
[1565]	NEK6	--->	SGK1	1.002	-1.0109
[1566]	ARL5B	--->	SMAD4	0.5744	-0.4943
[1567]	TERF1	--->	ATM	0.21663	-0.22672
[1568]	SMAD4	--->	ZNF8	0.12829	-0.12271
[1569]	ZNF341	<---	PRPF40A	-0.24694	0.24235
[1570]	PPP2CA	--->	RRAS	0.42845	-0.43116
[1571]	RGS9	<---	GUCY2D	-0.024481	0.010197
[1572]	UBE2I	--->	SLC2A4	0.55215	-0.52623
[1573]	RBL1	<---	SUV420H2	-0.29143	0.28157
[1574]	TRAT1	<---	LCK	-1.0055	0.9962
[1575]	IKZF1	--->	IKZF3	-0.012038	-0.036094
[1576]	NPPC	<---	NPR2	-0.15882	0.14651
[1577]	F2	--->	SERPINB6	0.057207	-0.040728
[1578]	HMOX2	--->	GIT1	0.1802	-0.18447
[1579]	OCLN	--->	TJP1	0.049764	-0.043104
[1580]	GAS6	<---	AXL	-0.15944	0.15417
[1581]	LTBP3	--->	TGFB1	0.99619	-1.0016
[1582]	NECAB2	<---	LNX1	-0.1986	0.21795
[1583]	STAT3	<---	GATA2	-0.59647	0.54434
[1584]	SOX4	<---	UBE2I	0.0099088	0.02288
[1585]	PDZK1	<---	SSTR5	-0.19778	0.21221
[1586]	MAPK7	--->	MEF2C	0.17185	-0.20713
[1587]	YWHAE	<--->	YWHAG	0.069023	0.069023
[1588]	GABBR2	?	GABBR1	-0.039292	-0.039292
[1589]	GSK3B	<---	PRKCB	0.47633	0.49475
[1590]	XBP1	<---	ESR1	0.008943	0.026385
[1591]	SRC	--->	GRB2	0.86853	-0.088967
[1592]	CEACAM5	--->	PTCHD2	0.17998	-0.19909
[1593]	XRN2	--->	TOLLIP	0.048391	-0.044204
[1594]	ESR1	?	THRB	-0.0089162	-0.0089162

[1595]	TRAF2	--->	ZFAND6	0.12206	-0.11757
[1596]	JUN	<---	TOP2A	-0.61269	0.56679
[1597]	PPP1CA	--->	PTEN	0.16959	-0.1777
[1598]	RNF11	--->	GGA3	0.19846	-0.16573
[1599]	FCGR2B	--->	INPP5D	0.62918	-0.63997
[1600]	MEIS1	<---	HOXA5	-0.0094151	0.024726
[1601]	SETD2	--->	HTT	0.24818	-0.23282
[1602]	EPS15	<---	DNM1	-0.019797	0.0064683
[1603]	PPP2R1A	--->	PPP2R3B	0.0069256	-0.019119
[1604]	PDLIM5	--->	CACNA1B	0.10329	-0.11285
[1605]	SKIL	<---	MACF1	-0.1191	0.11212
[1606]	BCL2	<---	PRKACA	-1.2078	1.1791
[1607]	ITCH	--->	TRPV1	0.16582	-0.17728
[1608]	FYN	--->	FAS	0.59095	-0.43713
[1609]	DOK2	<---	RASA1	-0.32041	0.17841
[1610]	RET	--->	PTK2	0.27422	-0.092834
[1611]	SH2B1	<---	FGFR3	-0.064262	-0.0177
[1612]	HNRNPK	--->	KHDRBS3	0.021272	-0.0060517
[1613]	RIMS1	<---	RAB37	-0.0067445	0.016184
[1614]	BLNK	<---	CBLB	-0.52343	0.49009
[1615]	RIT1	--->	RALGDS	0.98547	-1.0084
[1616]	PAX3	--->	SOX10	0.025489	-0.029062
[1617]	IL6R	--->	STAT3	1.0066	-0.99066
[1618]	NCOA1	<---	YWHAH	-0.091303	0.082501
[1619]	EFEMP2	--->	TP53	0.35929	-0.34755
[1620]	CAMK2D	<---	STAT1	-0.60307	0.59503
[1621]	FGF4	--->	FGFR2	0.54173	-0.53789
[1622]	CASP8	--->	CDKN1B	0.21607	-0.27684
[1623]	PARD6G	<---	PRKCZ	-0.068765	0.075804
[1624]	CALCOCO2	--->	DAXX	0.11876	-0.14563
[1625]	GNA12	--->	CDH2	0.20416	-0.20669
[1626]	MAP3K2	--->	SH2D2A	0.40471	-0.27607
[1627]	HDAC7	--->	BCL6	0.22515	-0.23839
[1628]	RPS6KA3	--->	HIST3H3	1.009	-0.99586
[1629]	MYF6	<---	CALM2	-0.22666	0.2676
[1630]	CBX4	<---	RING1	0.016956	0.017911
[1631]	NR1H3	<---	PPARA	-0.0098959	-0.0063998
[1632]	SMAD3	--->	MAX	1.0041	-1.0094
[1633]	ITGB3	<---	COL1A2	-0.028526	0.016401
[1634]	PRKACA	--->	CIITA	0.6377	-0.65892
[1635]	FGFR1	<---	GRB2	-0.14334	0.086985
[1636]	CABIN1	<---	AMPH	-0.065034	0.079245
[1637]	DAXX	<---	TP73	-0.016258	0.0054877
[1638]	RIPK1	--->	TRAF1	0.15943	-0.01325
[1639]	SRC	--->	CHUK	0.99783	-0.99597
[1640]	ARHGAP26	<---	PTK2	-0.058522	-0.014276
[1641]	ACLY	<---	GSK3B	-0.72114	0.69525
[1642]	PIM1	--->	CDKN1A	0.51838	-0.55156
[1643]	POU1F1	--->	GATA2	0.056147	-0.015702
[1644]	EIF2AK2	--->	STAT3	1.0077	-1.0166
[1645]	ENOX1	<---	EEF1G	0.011855	0.020685
[1646]	EPHA3	--->	CRK	0.13036	-0.17576
[1647]	SH3GL2	--->	EGFL6	0.093076	-0.078222
[1648]	PRKCA	--->	GMFB	0.91579	-0.9142
[1649]	WWP1	--->	IL2RG	0.099376	-0.11316
[1650]	GPR125	<---	DLG1	-0.031706	0.020731
[1651]	PPP1R9B	<---	DRD2	-0.41277	0.44636
[1652]	SERPINB8	<---	F10	-0.016469	0.01376
[1653]	NR3C1	?	NR2F6	-0.0089162	-0.0089162
[1654]	GRIA2	<---	PICK1	-0.098237	0.11658
[1655]	SPAG9	<---	MAPK14	-0.10862	0.060037
[1656]	NCOR2	<---	NFKB1	-0.071829	0.059738
[1657]	EPAS1	<---	EGLN1	-0.22558	0.21644
[1658]	MPL	--->	IRS2	0.042582	-0.052138
[1659]	FXR2	<---	MCRS1	-0.015308	0.049224
[1660]	COL6A1	--->	FGF7	0.014676	-0.024499
[1661]	HSP90AB1	--->	TP53	0.10125	-0.10969
[1662]	HDAC9	<---	CTBP1	-0.057098	0.050957
[1663]	EN1	--->	JUN	0.40134	-0.44785
[1664]	NR3C2	--->	AR	-0.0031537	-0.58072
[1665]	AFAP1	<---	PRKCE	-0.82012	0.86188
[1666]	GAK	<---	CDK5	-0.089947	0.73732
[1667]	STRAP	--->	SMAD7	0.46428	-0.38187
[1668]	NPAS2	<---	HSP90AA1	-0.055793	0.044236
[1669]	ACVR1C	<---	INHBB	-0.71391	0.72054
[1670]	MAPK3	--->	PLAT	0.30477	-0.32323

[1671]	CXCR1	--->	GNA14	1.0129	-1.008
[1672]	C9	<---	CD59	-0.1146	0.12539
[1673]	KAT2B	--->	CIITA	0.1123	-0.10992
[1674]	PRKACA	<---	CSK	-1.2776	1.3862
[1675]	SYNJ1	<---	PACSIN1	-0.096023	0.10986
[1676]	IL6ST	<---	PTPN6	-0.42337	0.41197
[1677]	NR5A2	--->	ALB	0.013095	0.011907
[1678]	LASS2	--->	SLC22A1	0.25973	-0.23531
[1679]	CBL	--->	CD19	0.31821	-0.3271
[1680]	NKRF	<---	NFKB1	-0.77175	0.7798
[1681]	MRAS	--->	MLLT4	0.99712	-0.98672
[1682]	CDKN1B	--->	CCNB1	1.0016	-1.0151
[1683]	GNA13	<---	PPP5C	-0.0029914	-0.00080903
[1684]	SH2B2	<---	SYK	-0.99632	1.0101
[1685]	CDKN1A	--->	GNB2L1	0.048488	-0.038432
[1686]	NSMAF	<---	TNFRSF1A	-0.11449	0.29828
[1687]	GALNT14	--->	MUC13	0.089314	-0.055882
[1688]	MATN2	<---	COL4A6	-0.028289	0.018624
[1689]	PRKCQ	--->	VAV1	0.031692	-0.046658
[1690]	ESR2	?	ESR1	-0.0089162	-0.0089162
[1691]	SSH3	--->	CFL1	0.3315	-0.32562
[1692]	CDKN2C	<---	CDK4	-1.3222	1.3062
[1693]	ESRRA	<---	PPARGC1A	-0.021031	0.013278
[1694]	ARHGAP17	<---	PACSIN1	-0.040952	0.043569
[1695]	POU1F1	--->	NR3C1	0.012582	0.012013
[1696]	ESR1	<---	UBE2I	-0.16643	0.1925
[1697]	CAV1	--->	FLOT2	0.073391	-0.061294
[1698]	EPHB6	--->	MLLT4	0.093833	-0.08479
[1699]	ESR2	--->	STAT5A	0.27638	-0.29089
[1700]	ENAH	<---	ABI2	-0.01221	-5.6818e-005
[1701]	GRIA3	<---	GRIP2	-0.13456	0.14237
[1702]	KIDINS220	<---	CRKL	-0.14354	0.11799
[1703]	PPP3CC	<---	TGFBR1	-0.064567	0.046325
[1704]	MMP3	<---	IGFBP3	-0.15977	0.18055
[1705]	GRM8	<---	PIAS1	-0.12352	0.13497
[1706]	CAV1	--->	NOS3	0.45512	-0.46313
[1707]	GHR	<---	JAK2	-0.16016	0.19464
[1708]	SIRPA	<---	TRIM2	-0.14243	0.15163
[1709]	LIMK1	--->	SSH3	0.010617	-0.027063
[1710]	UBQLN2	<---	PRPF40A	-0.22895	0.26122
[1711]	HERC3	--->	UBQLN2	0.23253	-0.22787
[1712]	NUP37	<---	TGFBR1	-0.40828	0.3741
[1713]	CHRDL1	--->	BMP4	0.71403	-0.67452
[1714]	PLEC1	<---	SPTA1	-0.060693	0.056008
[1715]	GJA3	<---	PRKCG	-0.56302	0.55904
[1716]	DNM2	<---	CCR5	-0.19926	0.19127
[1717]	SVEP1	<---	ZFYVE9	-0.013273	-0.00021353
[1718]	CFLAR	<---	MAP2K4	-0.077064	0.090997
[1719]	AKT1	--->	IKBKB	1.0001	-0.99768
[1720]	DDR1	<---	PTPN11	-0.2959	0.53921
[1721]	LASS2	--->	ASGR1	0.090629	-0.08365
[1722]	OPTN	<---	FLII	-0.13234	0.14524
[1723]	ESRRA	--->	MACF1	0.070458	-0.068839
[1724]	INHBA	<---	FST	-0.99524	1.0085
[1725]	CD40	<---	MAP3K8	-0.64109	0.59576
[1726]	SCN5A	<---	CBL	-0.50848	0.50268
[1727]	GFI1B	<---	ANKS3	-0.052379	0.04384
[1728]	CDC42BPA	--->	PPP1R12C	0.30518	-0.31765
[1729]	NPFFR2	<---	NPY	-0.31646	0.34254
[1730]	CACNB3	--->	CACNA1C	0.46475	-0.47571
[1731]	MAPKAPK2	--->	TSC2	0.63455	-0.63713
[1732]	SYTL4	<---	RAB3A	-0.15731	0.20092
[1733]	CD40	<---	CBL	-0.33561	0.32332
[1734]	THRB	<---	TRIP6	-0.062299	0.088883
[1735]	YWHAG	<---	WNK1	-0.25592	0.21615
[1736]	CDHR2	<---	MAST2	-0.47487	0.45486
[1737]	SPTBN2	<---	SPTA1	-0.043149	0.0015482
[1738]	GATA1	--->	SPI1	0.086082	-0.035588
[1739]	EED	<---	EZH2	-0.20733	0.24227
[1740]	SNX4	?	SNX2	-0.040204	-0.040204
[1741]	RGS12	<---	ARAF	-0.11784	-0.056329
[1742]	RELA	<---	MAP2K6	-1.0004	1.0015
[1743]	ADRA1D	--->	FLNC	0.35	-0.35188
[1744]	CBX1	<--->	CBX5	0.0018385	0.0018385
[1745]	BRD7	--->	HIST2H2BE	0.29322	-0.24229
[1746]	RAPGEF4	--->	RRAS2	0.28549	-0.28977

[1747]	BMPR1B	<---	RASL12	-0.53994	0.52577
[1748]	ZFHX3	<---	NINL	-0.18854	0.19173
[1749]	CHEK1	--->	CDC25A	0.69298	-0.67632
[1750]	DDX1	--->	RELA	0.036703	-0.043401
[1751]	JAK2	--->	PPIA	0.2974	-0.17638
[1752]	PRKCQ	--->	STK39	0.9999	-0.99823
[1753]	ACTN4	--->	ITGB1	0.046115	-0.058275
[1754]	CSF2RA	--->	CSF2RB	-0.088349	-0.1316
[1755]	TGIF1	<---	CTBP1	-0.12688	0.12019
[1756]	SHC1	<---	LTK	-0.51433	0.23403
[1757]	AKT1	--->	PTEN	0.35215	-0.26364
[1758]	CDK2	--->	EP300	0.32272	-0.32425
[1759]	ARHGAP21	<---	RHOA	-0.25639	0.25597
[1760]	SYNJ1	<---	GRB2	-0.29365	0.30666
[1761]	RHEBL1	--->	SMAD2	0.55322	-0.47159
[1762]	TSC1	<---	AKT1	-0.31412	0.46461
[1763]	CHD3	<---	SATB1	-0.0075149	-0.0049233
[1764]	COL4A5	--->	DCN	0.00097089	-0.0049704
[1765]	SHC1	<---	CEACAM1	-0.10792	0.064063
[1766]	CDK9	<---	TRAF2	-0.9866	1.0023
[1767]	TRAIP	<---	TRAF2	-0.098249	0.10363
[1768]	SRC	--->	IKBKG	0.77598	-0.70907
[1769]	PTPN6	--->	TFG	0.77186	-0.59345
[1770]	MTOR	--->	RICTOR	0.015677	-0.028038
[1771]	MDM2	<---	ADRBK1	-0.28847	0.2659
[1772]	NTRK2	--->	SH2B2	0.037014	-0.16557
[1773]	SYNJ1	<---	SH3KBP1	-0.12374	0.1559
[1774]	HIF1A	--->	HDAC1	0.51099	-0.51508
[1775]	RAP1GDS1	<---	RAP1A	-0.41126	0.44004
[1776]	TCF12	<---	PRSS23	-0.31456	0.30579
[1777]	ACTN4	<---	LRRC7	-0.017903	0.0080253
[1778]	PLXNA1	<---	FES	-0.42348	0.34667
[1779]	CBX2	<---	RYBP	-0.031198	0.065553
[1780]	G6PC	<---	FOXO1	-0.20398	0.192
[1781]	INS	--->	NOV	0.46446	-0.47388
[1782]	PRELP	--->	COL1A1	-0.0021925	-0.0083512
[1783]	FXR2	?	FMR1	-0.037462	-0.037462
[1784]	PIK3R4	--->	PIK3C3	0.04886	-0.060221
[1785]	TGFB1I1	<---	PTK2B	-0.60363	0.60368
[1786]	ITGAV	--->	PDGFRA	0.00063461	-0.013559
[1787]	FHL2	--->	HAND1	0.62562	-0.58677
[1788]	PRKCE	--->	OPRD1	0.556	-0.62401
[1789]	ETS2	<---	TTRAP	-0.22957	0.24997
[1790]	HDAC4	--->	NR2C1	0.094064	-0.067996
[1791]	GGA1	--->	LRP3	0.062428	-0.027973
[1792]	RXRG	<---	MAP4	-0.20757	-0.0017638
[1793]	CASP3	<---	DCC	-0.11233	0.065757
[1794]	EPS15	<---	NUMB	-0.052561	0.040903
[1795]	NCOR2	--->	POU2F1	0.23016	-0.23916
[1796]	MAP1A	--->	ACTA1	0.30819	-0.31177
[1797]	LCK	--->	FASLG	0.81231	-0.72815
[1798]	STAT3	<---	STAT5B	-0.13786	-0.08806
[1799]	CCR5	--->	GNAI1	1.0129	-1.008
[1800]	MAPK14	--->	ELK3	1.0099	-1.0011
[1801]	TRIP4	--->	NCOA1	0.053896	-0.061442
[1802]	TP53	<---	SP1	-0.14227	0.13146
[1803]	VASP	<---	PFN1	-0.023349	0.039151
[1804]	F11R	--->	ITGAL	0.14147	-0.15195
[1805]	KPNA2	<---	RELB	-0.38418	0.38498
[1806]	ANGPT1	<---	TEK	-0.0096615	-0.0042617
[1807]	FLNA	--->	ITGB1	0.13605	-0.14529
[1808]	BRCA1	--->	SMAD2	0.25767	-0.21264
[1809]	EGFR	--->	CTNND1	0.10935	-0.12117
[1810]	IRS2	<---	JAK3	-1.0155	1.0141
[1811]	RUNX2	--->	CCNB1	0.60155	-0.58817
[1812]	BTC	--->	ERBB2	0.19086	-0.19678
[1813]	STARD13	<---	KIF1B	-0.0044908	0.0051234
[1814]	ESR2	--->	CALM2	0.15612	-0.12827
[1815]	AURKB	--->	RACGAP1	0.23631	-0.25262
[1816]	AXL	<---	PTPN11	-0.13055	0.20505
[1817]	TP53	<---	HDAC1	-0.22533	0.22693
[1818]	RPS6KA1	--->	CREB1	1.0082	-0.98976
[1819]	MATN2	--->	ATXN1	0.0021563	-0.012088
[1820]	DACH1	--->	SMAD4	0.21461	-0.16954
[1821]	CTNND1	<---	PTPN6	-0.99992	1.004
[1822]	VAV1	<---	RAC1	-0.22722	0.041359

[1823]	IKZF4	--->	IKZF3	-0.0099058	-0.021368
[1824]	RASSF8	--->	FRMD6	0.042271	-0.038573
[1825]	HNRNPK	<---	CNNM3	-0.76918	0.78118
[1826]	DAB1	--->	APLP1	0.032104	-0.034662
[1827]	NF2	--->	ITGB1	0.043917	-0.056078
[1828]	ETV4	<---	SRF	-0.1691	0.19526
[1829]	ACTB	<---	HNRNPU	-0.53721	0.54
[1830]	PRKCA	--->	NCF1	0.51538	-0.52599
[1831]	FPR1	--->	ACTG1	0.38036	-0.38812
[1832]	FGFR1	<---	CRK	-0.059022	0.019095
[1833]	RAN	--->	TGFBR1	0.010625	-0.037483
[1834]	NISCH	--->	ITGA5	0.015357	-0.025653
[1835]	CAMK2G	--->	LRRC7	0.04106	-0.05314
[1836]	ZAP70	--->	RASA1	1.0053	-0.99912
[1837]	SMAD1	--->	GLI3	0.15886	-0.15177
[1838]	RNF11	--->	EPS15	0.084412	-0.094733
[1839]	ACVR1	--->	CHN1	0.048033	-0.074183
[1840]	PLD1	<---	ACTB	-0.26381	0.25267
[1841]	PRKAA2	--->	LEP	-0.046879	-0.27535
[1842]	JUND	<---	DDIT3	-0.43348	0.3977
[1843]	KCNC1	?	KCNV2	-0.025901	-0.025901
[1844]	ZNF473	<---	TRIM41	-0.08343	0.073052
[1845]	FBXO2	<---	SKP1	-0.044878	0.055251
[1846]	YWHAG	--->	KIF1C	0.049737	-0.040995
[1847]	CMA1	<---	EDN2	0.015296	0.018665
[1848]	AXL	--->	IL2RG	0.038654	-0.081483
[1849]	KHDRBS1	<---	JAK3	-0.16169	0.15941
[1850]	PTPRR	--->	MAPK3	0.36571	-0.37559
[1851]	MEIS1	<---	HOXA11	-0.0094151	0.024726
[1852]	FXR1	?	FMR1	-0.037462	-0.037462
[1853]	DOK4	<---	CRK	-0.17891	0.16295
[1854]	MMP9	<---	COL1A1	-0.04494	0.03546
[1855]	POU2F1	--->	STAT5A	0.45894	-0.47646
[1856]	PPP1R9B	<---	CCKAR	-0.19778	0.21221
[1857]	MAPK15	--->	NR3C1	0.99834	-1.0079
[1858]	CHAF1A	--->	MBD1	0.012442	-0.015197
[1859]	WT1	<---	TP63	-0.019382	0.018391
[1860]	FRS3	<---	BMPR2	-0.40258	0.38222
[1861]	GP1BA	--->	GP5	0.0060835	-0.003674
[1862]	ACAN	--->	MMP1	0.022421	-0.030194
[1863]	PLG	--->	SERPINB6	0.057207	-0.040728
[1864]	SYTL1	--->	NRXN1	0.083467	-0.043035
[1865]	BMPR1B	<---	CDK14	-0.20564	0.30344
[1866]	MMP7	--->	CD44	-0.0016332	-0.0037852
[1867]	SCMH1	--->	UBQLN4	0.17813	-0.13989
[1868]	AMIGO3	--->	AMIGO1	0.02677	0.023408
[1869]	TGFBR3	<---	ENG	-0.1219	-0.057651
[1870]	CEBPB	<---	CCL3	-0.047059	0.097043
[1871]	FYN	--->	CNN1	1.0728	-0.91195
[1872]	DOK1	--->	ITGB3	0.021936	-0.024109
[1873]	RIPK1	<---	TRAF3	-0.029911	0.070294
[1874]	SSH1	--->	ACTB	0.63171	-0.63329
[1875]	GADD45A	<---	MAPK14	-0.47959	0.31023
[1876]	PPARGC1A	--->	NR1H4	0.013278	-0.021031
[1877]	THOC4	--->	BAT1	0.010115	-0.013668
[1878]	KCNQ5	--->	CALM2	0.32976	-0.29233
[1879]	SMAD3	<---	PRDM4	0.0043727	0.016992
[1880]	HDAC3	--->	NR2C1	0.094064	-0.067996
[1881]	CDK5	--->	ERBB3	0.29741	-0.32251
[1882]	HSP90AA1	<---	FYN	-0.1078	0.11732
[1883]	MYBL2	<---	CDC34	-0.067336	0.064161
[1884]	APEX2	--->	CALCOCO2	0.036705	-0.0012275
[1885]	ESR1	<---	COPS5	-0.19181	0.21292
[1886]	MTCP1	--->	AKT2	0.99137	-0.99692
[1887]	PTPRJ	--->	MET	0.44701	-0.46104
[1888]	SMAD4	<---	SPTBN1	-0.030643	0.038981
[1889]	ABL1	--->	TP73	-0.012652	-0.034855
[1890]	AATF	<---	PAWR	-0.034501	0.050808
[1891]	SH2B1	<---	NTRK2	-0.16557	0.037014
[1892]	ACVR1	<---	PAK1	-0.40102	0.38869
[1893]	IGFBP7	--->	CXCL10	0.050367	-0.047815
[1894]	CASP3	--->	APP	0.23458	-0.24991
[1895]	PRKCA	--->	NFATC1	0.10256	-0.11537
[1896]	CAPN1	--->	F2R	0.16203	-0.16251
[1897]	DUSP4	--->	MAPK1	1.0016	-1.0004
[1898]	BIRC3	<---	CASP3	-0.16105	0.10574

[1899]	ONECUT1	--->	NR3C1	0.012582	0.012013
[1900]	HNF4A	--->	FOXO1	0.0048153	-0.015947
[1901]	HSP90AA1	<---	PRKCA	-0.043211	0.030868
[1902]	EPHA7	--->	MLLT4	0.095031	-0.086065
[1903]	CABP4	--->	CACNA1F	0.15283	-0.164
[1904]	SMAD3	--->	AR	0.33034	-0.33543
[1905]	ADAM19	--->	A2M	0.12198	-0.10683
[1906]	RGS4	--->	GNAO1	1.0012	-1.0015
[1907]	KLHL3	<---	GNAI1	-0.22528	0.23954
[1908]	RAB11FIP2	<---	RAB25	-0.15731	0.20092
[1909]	CLSTN1	<---	TRIP12	-0.081545	0.080244
[1910]	SQSTM1	--->	MAP1LC3B	0.1048	-0.10304
[1911]	SMN1	<---	COPS6	-0.1398	0.15029
[1912]	BTRC	--->	NFKBIA	0.99943	-1.0169
[1913]	FHL2	<---	EP300	-0.18563	0.18135
[1914]	CCL8	<---	CCR5	-0.46475	0.49595
[1915]	THRB	--->	NCOA3	0.12213	-0.13412
[1916]	EP300	--->	ZBTB17	0.026511	-0.039308
[1917]	ANTXR2	<---	COL4A3	-0.042432	0.035371
[1918]	TSSK4	--->	CREB1	0.96654	-0.95401
[1919]	RELA	<---	RELB	-0.055436	0.075417
[1920]	CDK1	<--->	PBK	0.23429	0.23429
[1921]	TNFSF14	--->	LTBR	1.0116	-0.999
[1922]	PAK2	<---	MYL2	0.0024497	0.2808
[1923]	CCNE2	<---	CDK3	-1.0774	1.0544
[1924]	PAX6	<---	TRIM11	-0.070344	0.067406
[1925]	RNF41	<---	USP8	-0.038673	0.07625
[1926]	GAB1	<---	SOS1	0.033721	0.046176
[1927]	GRIK2	--->	CDH2	0.048327	-0.050607
[1928]	ZNF263	<---	TRIM41	-0.085965	0.079356
[1929]	NCAM1	--->	BDNF	0.074054	-0.083948
[1930]	MRVI1	--->	ITPR1	1.0036	-1.0037
[1931]	SYNGAP1	<---	ULK2	-0.020531	0.0073649
[1932]	PROC	--->	SERPINB6	0.04046	-0.023809
[1933]	PTPRU	--->	GNAI1	0.40483	-0.41277
[1934]	SERPINB9	--->	SETDB1	0.21405	-0.19788
[1935]	MAP4K2	<---	RAB8A	-0.15223	0.34485
[1936]	G3BP2	<---	NFKB1	-0.77151	0.77951
[1937]	PHOX2A	--->	ATF6B	0.18898	-0.15681
[1938]	RPS6KA1	<---	MTOR	-1.0002	1.0159
[1939]	NHP2L1	<---	RBM10	-0.12394	0.15841
[1940]	PRKCZ	--->	PEBP1	1.0061	-1.0011
[1941]	AR	<---	RAF1	-0.35815	0.34757
[1942]	MATN3	?	MATN1	-0.013202	-0.013202
[1943]	HPCAL1	<---	DTX2	-0.12829	0.12358
[1944]	DMD	--->	SNTA1	0.048407	-0.052248
[1945]	RGS16	--->	GNAQ	1.0012	-1.0015
[1946]	MAP3K4	<---	MAP2K4	-0.38277	0.050282
[1947]	SH3D19	--->	ADAM17	0.0027746	-0.020099
[1948]	TRAF4	<---	SMURF2	-0.25037	0.24105
[1949]	PDGFB	<---	HRAS	-0.1954	0.20867
[1950]	WIPF2	--->	PFN1	0.029503	0.0030862
[1951]	EIF2AK4	--->	SMAD4	0.61235	-0.61377
[1952]	SRGAP1	<---	CDC42	-0.093021	0.10009
[1953]	GNAT1	<---	F2R	-1.008	1.0129
[1954]	PPP2R1A	--->	SMAD3	0.17032	-0.15926
[1955]	APC	<---	PPP2CA	-0.01939	0.0056537
[1956]	RPS6KA5	<---	STK11	-0.74727	0.78966
[1957]	JAG1	<---	THBS1	-0.084363	-0.0048065
[1958]	FN1	<---	COL1A1	-0.031198	0.022644
[1959]	MNT	?	MLX	-0.65635	-0.65635
[1960]	PLCD1	<---	CALM1	-0.16024	0.17365
[1961]	ESR1	<---	MLL2	-0.10069	0.12772
[1962]	PRKACA	--->	RYR1	0.21832	-0.22731
[1963]	PRKG1	--->	VASP	0.99265	-0.99079
[1964]	SYT8	--->	SYNCRIP	0.077746	-0.039791
[1965]	EGLN2	--->	HIF1A	0.99907	-0.99517
[1966]	DLG1	<---	LCK	-0.10902	0.12199
[1967]	NOTCH1	<---	NFKB1	-0.019922	-0.0064626
[1968]	PI4KA	--->	CD4	-0.00095456	-0.007114
[1969]	CPNE4	--->	ACTB	0.56315	-0.55654
[1970]	GNB3	<---	TGFBR1	-0.40828	0.3741
[1971]	CREBBP	--->	NR3C1	0.17826	-0.18496
[1972]	HIP1R	<---	HTT	0.013708	0.020393
[1973]	BARD1	<---	MDC1	-0.12912	0.1478
[1974]	PRUNE2	<---	RCN3	-0.16469	0.16926

[1975]	BGN	--->	COL1A1	-0.00034351	-0.0080368
[1976]	CACNB3	<---	SYT1	-0.052901	0.093683
[1977]	SVIL	--->	MMP25	0.26099	-0.22635
[1978]	MEGF10	--->	KIAA1598	-0.0042695	-0.0087949
[1979]	TRAF6	--->	EDA2R	0.6024	-0.61161
[1980]	AR	<---	NONO	-0.95476	0.95404
[1981]	GIT1	<---	ARHGEF7	-0.058731	0.046581
[1982]	CHUK	--->	NR2C2	0.29669	-0.42769
[1983]	KHDRBS2	<---	GRB2	-0.28063	0.27717
[1984]	CFLAR	--->	CASP3	0.99569	-0.9907
[1985]	DUSP3	--->	FGF7	0.4364	-0.41865
[1986]	CASP4	<---	PSEN1	-0.048154	0.056436
[1987]	DNAJC2	<---	HSPA14	-0.78603	0.82816
[1988]	ARF6	--->	CHRM3	0.063764	-0.055567
[1989]	ITGB4	<---	ATXN1	-0.0064328	-0.0062027
[1990]	SORBS1	<---	PTK2	-0.10077	0.027362
[1991]	NFKB2	<---	IKBKB	-0.99594	0.99702
[1992]	KCNA3	?	KCNA2	-0.025901	-0.025901
[1993]	KDM2A	--->	SMAD7	0.42793	-0.34495
[1994]	PLEKHG2	<---	CDC42	-0.29753	0.32945
[1995]	LYN	--->	CSF1R	0.51153	0.27207
[1996]	GGA3	<---	ARF1	-0.4338	0.46028
[1997]	PXN	<---	LCK	-0.60891	0.65058
[1998]	KPNA2	--->	PAX5	0.21934	-0.1984
[1999]	CASP3	--->	BRCA1	0.34307	-0.41552
[2000]	PDGFRB	--->	TYK2	0.00056911	-0.01591
[2001]	CPNE4	<---	PPP5C	-0.043387	0.048759
[2002]	TBL1X	--->	CTNNB1	1.0016	-0.99627
[2003]	CDK14	--->	CDKN1A	0.51838	-0.55156
[2004]	COPB2	<---	RGS2	-0.55801	0.58652
[2005]	GDF7	?	BMP7	-0.061425	-0.061425
[2006]	PRPF40A	--->	CUL1	0.069919	-0.059107
[2007]	TRERF1	--->	NR5A1	0.013367	-0.012578
[2008]	NUMB	<---	PRKCA	-0.55841	0.55403
[2009]	AR	<---	CDK6	-1.2513	1.2465
[2010]	RXRB	--->	PPARG	-0.0079439	-0.0087197
[2011]	BRCA1	--->	YY1	0.064709	-0.065971
[2012]	ARRB2	<---	ADRB2	-0.12587	0.12815
[2013]	CDK6	--->	CDKN2A	0.25926	-0.26299
[2014]	TRIP6	--->	ODF1	0.14572	-0.096589
[2015]	HIPK1	--->	TP53	1.0002	-0.99962
[2016]	TNFSF14	<--->	LTB	0.44363	0.44363
[2017]	SV2B	<---	SYT1	-0.37651	0.40922
[2018]	MCL1	?	BAK1	-0.63915	-0.63915
[2019]	SKIL	<---	COL4A2	-0.069362	0.057937
[2020]	PTPN12	--->	LPXN	0.5739	-0.58292
[2021]	KCNJ6	<---	DRD2	-0.43943	0.47115
[2022]	ERBB3	--->	PIK3R2	0.23248	-0.28371
[2023]	HIST2H2BE	<---	GADD45A	-0.10198	0.15343
[2024]	LTBP4	--->	TGFB1	0.99619	-1.0016
[2025]	FGFR1	<---	FGF2	-0.99871	1.0053
[2026]	TSC2	<---	SMAD2	-0.014015	0.024661
[2027]	BAX	<---	BID	-0.46902	0.49845
[2028]	NR2C2	<---	PEBP1	-0.029851	0.055828
[2029]	FOXG1	<---	FOXO4	0.034125	0.39933
[2030]	KHDRBS3	<---	RBM7	-0.042052	0.055977
[2031]	SIRT2	<---	CDC14B	-0.65264	0.69158
[2032]	TRAF2	<---	CD40	-0.64504	0.64735
[2033]	MCRS1	--->	PBX2	0.066612	-0.05273
[2034]	ADAP1	<---	PRKD1	-0.75267	0.71973
[2035]	GNAQ	<---	HTR2C	-0.98627	0.9872
[2036]	ATXN1	<---	SGMS1	-0.083421	0.081012
[2037]	PLCG1	<---	GRAP	-0.46724	0.29528
[2038]	STAT3	<---	PDGFRB	-0.16183	0.14585
[2039]	RGS18	--->	GNAI3	1.0012	-1.0015
[2040]	PRKCI	--->	YWHAH	0.25396	-0.26837
[2041]	C1R	<---	C1QB	-0.052141	0.04292
[2042]	SRC	--->	TERT	0.13979	-0.1366
[2043]	GSK3B	--->	GYS1	1.0067	-1.0067
[2044]	TRPC1	<---	RHOA	-0.16671	0.15589
[2045]	MBIP	<---	PLEKHF2	-0.14489	0.15274
[2046]	GRK5	--->	AVPR2	0.31971	-0.40764
[2047]	PIK3CA	<---	NRAS	-1.0082	0.98913
[2048]	CHRDL2	--->	BMP7	0.71403	-0.67452
[2049]	CTBP2	--->	BAZ2B	-0.0018216	-0.011662
[2050]	SORBS3	--->	ADAMTSL4	0.13832	-0.14333

[2051]	BMPR1A	<---	BMP2	-1.0007	0.99697
[2052]	AKAP9	<---	TRIP10	-0.11513	0.11957
[2053]	ZNF165	--->	DVL2	0.098498	-0.10766
[2054]	IRS1	<---	FER	-0.60886	0.60421
[2055]	GNB2	<---	TGFBR1	-0.40828	0.3741
[2056]	SMAD3	<--->	SMAD4	0.68552	0.68552
[2057]	CCNA1	--->	RBL2	0.26997	-0.28031
[2058]	RPS6KA1	--->	NR4A2	0.56871	-0.73191
[2059]	BCAR1	<---	FES	-0.27116	0.033974
[2060]	STAT1	--->	JUN	0.12698	-0.21221
[2061]	HSPB1	--->	DAXX	0.076546	-0.096092
[2062]	STK11	--->	STRADA	0.6413	-0.21779
[2063]	PCDH17	<---	UBQLN4	-0.088126	0.12226
[2064]	SLC9A3R1	--->	RDX	0.061503	-0.056659
[2065]	CDK1	<--->	CSNK2A1	0.23429	0.23429
[2066]	PRKCG	--->	DVL2	1.0137	-0.99339
[2067]	HECW1	--->	DVL1	0.056134	-0.068631
[2068]	PLXNA1	<--->	PLXNB1	0.014897	0.014897
[2069]	TSC1	<---	YWHAZ	-0.08934	0.11987
[2070]	MTOR	--->	IRS1	0.13236	-0.13875
[2071]	PTPN11	--->	CTLA4	0.63713	-0.56471
[2072]	CCNB1	<---	CDC25A	-0.3246	0.33489
[2073]	TGFBR1	--->	SMAD6	1.0115	-1.0087
[2074]	AGTR1	<---	EGFR	-0.076581	0.076801
[2075]	DLG4	--->	KCNA2	0.047736	-0.049447
[2076]	HDAC1	--->	NFKBIA	0.50219	-0.50984
[2077]	FYN	--->	CRK	0.47236	0.00040577
[2078]	PLD1	<---	CDC42	-1.0031	0.99718
[2079]	MAPK8IP1	<---	MAP3K7	-0.16562	0.16292
[2080]	NCOA3	<---	RXRB	-0.13412	0.12213
[2081]	PTPRS	--->	KAT5	0.15992	-0.16858
[2082]	SRCAP	<---	KAT2B	-0.035475	0.024788
[2083]	PAK6	--->	ESR1	0.73388	-0.56277
[2084]	FCGR3A	--->	CD247	0.47597	-0.4895
[2085]	ETS2	<---	SMARCA4	-0.041209	0.031899
[2086]	PIK3R1	<---	FYN	-0.99387	0.9987
[2087]	DAB1	--->	ITGB2	0.21401	-0.22291
[2088]	SUFU	<---	GLIS3	-0.088736	0.082486
[2089]	PRKCA	--->	RGS7	0.57282	-0.57611
[2090]	EIF3H	--->	ABI3	0.15773	-0.15746
[2091]	PDGFRB	<---	PIK3C2B	-0.0066848	-0.0061442
[2092]	CHEK2	--->	ERCC6	0.25238	-0.27074
[2093]	HNRNPL	--->	PCBP2	0.049773	-0.015394
[2094]	ACVR2B	<---	BMP7	-0.99943	1.0057
[2095]	GCK	<---	INS	-0.020961	0.036067
[2096]	HSPB6	<---	YWHAG	-0.1461	0.1839
[2097]	HOXA1	<---	TRIP6	-0.10746	0.14086
[2098]	TRAF6	--->	SRC	0.020723	-0.005385
[2099]	LYN	--->	CDK2	1.3862	-1.2776
[2100]	PLEK	--->	INPP5A	0.15291	-0.14482
[2101]	MAPK1	--->	FOS	0.53871	-0.68783
[2102]	SVEP1	<---	SMAD9	-0.0091113	-0.0037615
[2103]	CD2AP	<---	FYN	-0.44444	0.74787
[2104]	HABP2	<---	COL4A1	-0.011533	-0.0020188
[2105]	DOK1	--->	ITGB5	0.021936	-0.024109
[2106]	BIRC5	<---	CDK4	-0.69965	0.65723
[2107]	RERE	--->	PLSCR1	0.066718	-0.04805
[2108]	RGS2	<---	PRKCG	-0.87353	0.87438
[2109]	LRP8	<---	DLG4	-0.066551	0.055178
[2110]	CXCR1	--->	CXCL2	0.49595	-0.46475
[2111]	ABL1	<---	CBL	-0.21322	0.17988
[2112]	ZAP70	<---	FCGR3A	-1.0112	1.0048
[2113]	DLL1	--->	NOTCH3	-0.0073516	-0.010125
[2114]	GRIN1	<---	RAP2A	-0.31763	0.32333
[2115]	ATN1	--->	LTBP4	-0.0039514	-0.011042
[2116]	SNX6	?	SNX4	-0.040204	-0.040204
[2117]	NINL	--->	ZNF250	0.42694	-0.42617
[2118]	RBBP4	<---	HDAC1	-0.046452	0.052829
[2119]	HSPA1A	--->	NR3C1	0.5755	-0.5448
[2120]	PTH1R	<---	GNB1	-0.3213	0.32455
[2121]	JAK1	--->	BRCA1	0.31894	-0.33086
[2122]	EP300	--->	MEF2A	0.037357	-0.049058
[2123]	PIM1	--->	BAD	1.2302	-1.4217
[2124]	BTBD2	<---	IGSF21	-0.11578	0.10531
[2125]	SVIL	<---	NEB	-0.043657	0.098892
[2126]	ITGAV	<---	NID1	-0.010797	-0.0019659

[2127]	SH2D2A	<---	TXK	-0.53727	0.86783
[2128]	SMAD6	<---	SMURF2	-1.0067	0.9934
[2129]	PRG2	<---	PAPPA	-0.063059	0.094762
[2130]	TTRAP	--->	ETS1	0.24997	-0.22957
[2131]	HOXA10	<---	SIRT2	-0.11265	0.12778
[2132]	EGFR	--->	HGS	0.055598	-0.077423
[2133]	YWHAE	--->	TBP	0.0013287	-0.013882
[2134]	HDAC1	<---	BUB1	-0.42066	0.4
[2135]	PAX6	--->	RAX	-0.034251	-0.10489
[2136]	KHDRBS1	--->	PIK3R1	0.021672	-0.068892
[2137]	ITGA2B	<---	VWF	-0.024339	0.012226
[2138]	TP73	<---	BIN1	-0.07036	0.075074
[2139]	BMPR1B	<---	RAN	-0.53994	0.52577
[2140]	USP9X	--->	CTNNB1	0.54899	-0.54978
[2141]	NRBP1	<---	RAC3	-0.61665	0.61513
[2142]	PSG9	<---	PTPN12	-0.28215	0.26353
[2143]	INHBB	<---	INHA	-0.31443	-0.063724
[2144]	CHD3	--->	IVNS1ABP	-0.0004859	-0.0096165
[2145]	TANC1	--->	GRIN2B	0.033947	-0.039387
[2146]	GTF3C1	<---	DEDD	-0.11534	0.15607
[2147]	SFN	<---	TRIM25	-0.037811	0.033064
[2148]	NEK1	--->	ZNF350	0.63282	-0.59939
[2149]	STRN	<---	PPP2CA	-0.47389	0.47137
[2150]	ZAP70	--->	PLCG1	1.0054	-1.0267
[2151]	CSNK2A2	--->	IL16	0.62013	-0.64226
[2152]	DNMT3B	<---	HDAC1	-0.0084751	0.024192
[2153]	PTK2B	--->	SLC2A1	0.144	-0.25225
[2154]	PIAS1	--->	ELK3	0.16647	-0.15609
[2155]	PPP2R1A	--->	GRIN2D	0.070171	-0.079228
[2156]	ANK1	--->	SPTAN1	0.00082409	-0.015919
[2157]	TP53	<---	PPP1CA	-0.16272	0.15301
[2158]	EGFR	--->	ATP1A1	0.084674	-0.095229
[2159]	PLSCR1	<---	MED15	-0.035434	0.034679
[2160]	SVIL	--->	CAV3	0.097558	-0.046937
[2161]	GSK3A	--->	CREM	0.35976	-0.37116
[2162]	UBE2I	--->	ETS1	0.36763	-0.34734
[2163]	ABCA1	<---	LIN7B	-0.0080068	0.005539
[2164]	ARF6	--->	ARRB1	0.27431	-0.26383
[2165]	CAPNS2	<---	CAPN2	0.14257	0.24913
[2166]	CAV1	--->	GJB2	0.047144	-0.03367
[2167]	CLEC3B	<---	HGF	-0.027944	0.041731
[2168]	DUSP5	--->	MAPK3	1.0016	-1.0004
[2169]	TGFB3	?	TGFB2	-0.061425	-0.061425
[2170]	POU2F1	?	HNF1A	-0.11417	-0.11417
[2171]	NRXN1	<---	SIPA1L1	-0.37446	0.36354
[2172]	GNAQ	--->	VIPR1	0.4334	-0.43656
[2173]	YWHAZ	<---	TNFAIP3	-0.13918	0.13176
[2174]	CDK1	<---	TGFBR2	-0.99836	0.98829
[2175]	HDAC11	--->	HDAC6	0.020811	-0.0075026
[2176]	IGFBP7	--->	IGF1	0.0011358	-0.0050725
[2177]	CXCR1	--->	GNAI2	1.0129	-1.008
[2178]	MAP3K7	--->	CARD11	0.080372	-0.090612
[2179]	KCNJ12	<---	DLG4	-0.04695	0.04551
[2180]	MAPK8	--->	CRK	0.206	-0.30256
[2181]	TBK1	--->	HSP90AB1	0.019127	-0.032587
[2182]	AMFR	<---	UBE2D2	-0.11213	0.11388
[2183]	SMN1	--->	TP53	0.2172	-0.21579
[2184]	THBD	<---	F2	-0.012739	0.016969
[2185]	ATF3	--->	FGFR3	0.035925	-0.050394
[2186]	MEF2D	<---	MEF2A	-0.017289	-0.017284
[2187]	NISCH	--->	AR	0.57745	-0.58307
[2188]	SH3BP2	--->	VAV1	-0.027469	-0.26051
[2189]	ERBB2	<---	EGF	-0.36655	0.35749
[2190]	CSNK2A1	--->	YWHAQ	0.62776	-0.66368
[2191]	SFRS9	<---	DUSP11	-0.68308	0.71953
[2192]	E2F2	--->	YY1	0.082581	-0.076815
[2193]	SMAD2	--->	E2F4	0.19366	-0.13771
[2194]	ACVR1	<---	NEK8	-0.12661	0.18132
[2195]	CDK14	--->	TGFBR1	0.36359	0.02614
[2196]	SIX6	--->	TLE1	0.05243	-0.021516
[2197]	TERF1	--->	DLG5	0.050471	-0.030458
[2198]	SMAD4	<---	SNRNP70	-0.32493	0.40696
[2199]	EIF3F	--->	SMAD9	0.69911	-0.76963
[2200]	CCRL1	--->	CCL8	0.49595	-0.46475
[2201]	AR	<---	GNB2L1	-0.94858	0.94792
[2202]	PIK3CA	<---	TICAM1	-0.31674	0.3082

[2203]	NPHS1	<---	LYN	-0.0667	-0.02858
[2204]	ADAM22	<---	YWHAZ	-0.0038527	0.013329
[2205]	SNTG2	--->	DTNA	0.020806	0.0159
[2206]	MDM2	--->	NR3C1	0.14626	-0.15006
[2207]	HGS	<---	STAM2	-0.9996	0.99705
[2208]	NR0B2	--->	NR1H2	-0.0094276	-0.028437
[2209]	CCR5	--->	GNAQ	1.0129	-1.008
[2210]	BRD2	--->	E2F1	0.057671	-0.044146
[2211]	AKT1	--->	GATA1	0.49316	-0.34165
[2212]	CBLB	--->	EGFR	1.0132	-1.0135
[2213]	TWIST1	--->	GLI3	0.089588	-0.090315
[2214]	RAB13	--->	TGFBR1	0.010625	-0.037483
[2215]	PRKD1	--->	PLCG2	0.42463	-0.50259
[2216]	CYTH2	<---	ARF6	-0.18769	0.20203
[2217]	TRAT1	<---	SRC	-0.80822	0.81643
[2218]	RELA	<---	PPP2CA	-0.98625	0.99486
[2219]	AKT1	--->	KRT10	0.45101	-0.30414
[2220]	THRB	<---	TRIP12	-0.10641	0.13236
[2221]	SOX10	<---	MEOX1	-0.060464	0.063322
[2222]	SMAD1	<---	FOXG1	-0.50169	0.48112
[2223]	DOCK9	<---	CDC42	-0.020912	0.0079164
[2224]	RASGRF1	<---	NTRK3	-0.070857	0.059389
[2225]	YWHAG	<---	HSPA1A	-0.739	0.77111
[2226]	SKIL	<---	ASCC3	-0.2481	0.24513
[2227]	PCM1	--->	ABI2	0.022015	-0.017088
[2228]	ALX1	<---	CREBBP	-0.12156	0.11326
[2229]	DHX9	<---	BRCA1	0.0020759	0.0021762
[2230]	SH2B1	<---	NTRK1	-0.24591	0.043151
[2231]	JAK2	<---	RAF1	0.049279	0.06529
[2232]	DHX30	--->	NR2E3	0.042552	-0.032266
[2233]	RRAS2	--->	SMAD4	0.55322	-0.47159
[2234]	AR	<---	ESR1	-0.58072	-0.0031537
[2235]	NOXA1	<---	RAC1	-0.13569	0.12502
[2236]	PTK2	--->	ITGB1	0.055557	-0.077694
[2237]	ADAM12	<---	GRB2	-0.16008	0.16265
[2238]	FNBP1	--->	DNM1	0.082421	-0.091965
[2239]	BRCA1	<---	TOP2A	-0.06916	0.084341
[2240]	DLGAP1	<---	SHANK2	-0.068556	0.066496
[2241]	BCR	--->	YWHAB	0.078735	-0.083313
[2242]	CDK9	--->	AR	1.2465	-1.2513
[2243]	MAPK3	--->	TCF3	0.99488	-0.99939
[2244]	KCNQ2	--->	CALM1	0.32976	-0.29233
[2245]	FURIN	<---	ADAM19	-0.013374	0.01027
[2246]	SYTL5	<---	RAB3A	-0.15731	0.20092
[2247]	ACAN	<---	ADAMTS5	-0.015468	0.0043435
[2248]	GATA2	--->	JUN	0.38269	-0.42293
[2249]	AKAP9	<---	CSNK1E	-0.58225	0.71883
[2250]	DYRK1A	--->	CCNL2	0.64897	-0.66864
[2251]	DFNB31	--->	MYO7A	0.072616	-0.067932
[2252]	CDKN1A	<---	HDAC11	-0.041752	0.052598
[2253]	PRKCA	--->	EIF4E	0.94696	-0.94609
[2254]	CAV1	<---	GRB7	-0.16111	0.07337
[2255]	GRAP2	--->	SHB	0.28685	-0.18844
[2256]	CXCL13	<---	CXCR5	-0.46475	0.49595
[2257]	TES	--->	TLN1	0.19759	-0.20549
[2258]	MAGI3	<---	PTPRB	-0.087816	0.087637
[2259]	MYOG	<---	CALM3	-0.22666	0.2676
[2260]	E2F5	<---	EP300	-0.18734	0.18294
[2261]	YWHAZ	<---	PRKCI	-0.26837	0.25396
[2262]	TES	--->	VASP	0.10335	-0.085959
[2263]	TP53	<---	E2F1	-0.20932	0.21023
[2264]	SPRY2	<---	ADAMTSL4	-0.0036564	0.017674
[2265]	EGFR	--->	SGSM2	0.092695	-0.10179
[2266]	GJB1	?	GJB2	-0.032421	-0.032421
[2267]	PACSIN1	--->	WASL	0.092082	-0.11591
[2268]	HDAC2	--->	SMARCA5	0.0013206	-0.0086534
[2269]	PDGFRB	--->	PIK3CA	0.033614	-0.045851
[2270]	ATXN1	<---	TRAF2	-0.043696	0.034106
[2271]	KLK2	--->	SERPINE1	0.11833	-0.082185
[2272]	RASIP1	<---	MRAS	-0.64033	0.64447
[2273]	ITGB1	<---	IGF1R	-0.024262	0.010004
[2274]	RACGAP1	<---	MAP3K10	-0.16088	0.15751
[2275]	SMAD1	<---	INPP4A	-0.3971	0.37778
[2276]	KAT5	<---	CBX8	-0.025335	0.031942
[2277]	MATK	--->	KIT	1.0007	-1.0039
[2278]	FURIN	--->	FLNC	0.011227	-0.007489

[2279]	LYN	--->	CDKN1B	0.3974	-0.34695
[2280]	SOCS1	<---	PIM2	-0.71583	0.64694
[2281]	ITGB2	<---	RDX	-0.086221	0.074134
[2282]	PLD2	<---	AMPH	-0.29133	0.28396
[2283]	ESM1	--->	ITGB2	0.12755	-0.13849
[2284]	AKT1	--->	CREB1	0.4893	-0.45124
[2285]	KIT	--->	SOCS6	0.15769	-0.25081
[2286]	SNX9	--->	SOS2	0.015215	-0.030977
[2287]	ZAP70	--->	CRK	-0.046839	-0.28174
[2288]	ARAF	--->	PIK3R1	0.095588	-0.22343
[2289]	RAF1	--->	MAP2K1	0.9856	-1.0047
[2290]	TAL1	<---	GATA3	-0.26447	0.28682
[2291]	IL2RB	<---	GRB2	-0.54431	0.47127
[2292]	PLXNB1	<---	MLL	-0.063057	0.079857
[2293]	RET	<---	CBLB	-0.53538	0.52296
[2294]	H3F3A	<---	TLE2	-0.057829	0.10803
[2295]	SNX6	<---	ACVRL1	-0.083917	0.065877
[2296]	BMPR1B	--->	LZTR1	0.12723	-0.15552
[2297]	PPAP2B	<---	BCAR1	-0.028159	0.024141
[2298]	MAP2K6	--->	MAPK12	0.64901	-0.41806
[2299]	RGS3	--->	GNA11	0.35662	-0.36228
[2300]	CREB1	<---	SRF	-0.22638	0.23229
[2301]	CCL24	<---	CCR3	-0.46475	0.49595
[2302]	ETS1	--->	STAT6	0.15054	-0.16944
[2303]	ETV5	--->	AR	0.94517	-0.94537
[2304]	DDIT3	--->	ATF4	0.31009	-0.3007
[2305]	CALCOCO2	<---	PCGF1	-0.0041374	0.040751
[2306]	DEDD2	--->	CASP8	0.54362	-0.56644
[2307]	KCNJ2	<---	CASK	-0.1462	0.13185
[2308]	LYN	--->	BTK	1.0008	-0.98497
[2309]	GADD45G	<---	RAN	-0.4616	0.4922
[2310]	SOCS1	<---	NCK1	-0.070203	0.21075
[2311]	RBM19	<---	PTPN12	-0.56901	0.56611
[2312]	MAG	<---	COL3A1	-0.0077487	-0.0054005
[2313]	CASP3	--->	TRAF3	0.19743	-0.22497
[2314]	PRKD1	<---	EPAS1	-0.053023	0.039807
[2315]	CALCOCO2	--->	ZNF101	0.18918	-0.18295
[2316]	PRKDC	<---	CHUK	-0.21484	0.1291
[2317]	TREML1	<---	PTPN11	-0.82769	0.97001
[2318]	CRKL	<---	IGF1R	-0.11926	0.098003
[2319]	GRAP2	<---	GAB1	-0.09583	0.089757
[2320]	FRS2	<---	PRKCI	-0.37242	0.37492
[2321]	GNAI2	--->	PLSCR1	0.17832	-0.16912
[2322]	HDAC1	<---	SP1	-0.094236	0.082752
[2323]	SIGIRR	<---	TLR9	0.019325	0.21528
[2324]	DHX9	--->	KHDRBS1	0.061892	-0.064824
[2325]	NR2C2	<---	IKBKB	-0.42769	0.29669
[2326]	LDLR	<---	LDLRAP1	-0.14343	0.1387
[2327]	SETDB1	<---	ZFP64	-0.014921	0.012346
[2328]	ITGAE	<---	ITGB7	-0.030554	0.017259
[2329]	IKZF3	--->	BCL2L1	0.19742	-0.20413
[2330]	ETS1	<---	GATA3	-0.14597	0.17161
[2331]	COPS5	--->	RNF139	0.066239	-0.041316
[2332]	TLE1	<---	RNF10	-0.15934	0.17346
[2333]	GP5	--->	F2	0.042288	-0.035583
[2334]	ARHGEF18	<---	RHOA	-0.14455	0.15203
[2335]	GNAI1	--->	EPOR	0.12949	-0.12882
[2336]	CYTH2	--->	ARRB1	0.1548	-0.14696
[2337]	EIF4G1	--->	NCBP2	0.16238	-0.13175
[2338]	BLM	--->	MX1	0.15116	-0.15572
[2339]	SYNJ2BP	<---	RALBP1	-0.13035	0.14218
[2340]	ARHGEF15	<---	EPHA4	-0.25001	0.25178
[2341]	CAMKK1	--->	SGK1	1.3326	-1.3051
[2342]	RIPK1	--->	TAX1BP1	0.28296	-0.12837
[2343]	GABPB1	<---	CIC	-0.7548	0.787
[2344]	TGFA	--->	MAGI2	0.072699	-0.064389
[2345]	FBXW7	--->	LINGO1	0.070505	-0.077989
[2346]	INSR	<---	ARHGAP26	-0.097526	0.086294
[2347]	APP	<---	CRYAB	-0.36872	0.35919
[2348]	SETD7	--->	HIST2H3C	0.43678	-0.40934
[2349]	SPTA1	--->	VASP	0.061308	-0.06966
[2350]	GSK3A	--->	PXN	0.22984	-0.26539
[2351]	UBR1	--->	BTBD2	0.07015	-0.066732
[2352]	TRADD	--->	TRAF1	0.083752	-0.097415
[2353]	IL4R	--->	STAT6	0.18217	-0.2018
[2354]	TYK2	<---	PTPN1	-0.29476	0.27762

[2355]	RND1	--->	PLXNB1	0.41051	-0.37902
[2356]	WISP1	<---	DCN	-0.30201	0.29267
[2357]	MAPK8	<--->	MAPK1	0.23429	0.23429
[2358]	PRKCA	--->	GRM5	0.91747	-0.9157
[2359]	AR	<---	DAXX	-0.57605	0.56805
[2360]	DVL3	<---	CCNK	-0.20619	0.20073
[2361]	ZBTB16	<---	PGAM5	-0.027822	0.021861
[2362]	TGFB3	--->	TGFBR2	0.28625	-0.21622
[2363]	CRK	<---	KDR	-0.42452	0.54401
[2364]	PTPRE	--->	GRB2	0.70034	-0.9372
[2365]	SH3BP2	<---	FLT3	-0.54548	0.2315
[2366]	SMARCA5	--->	HIST4H4	0.049463	-0.04883
[2367]	RGS14	--->	GNAI2	0.013429	-0.011715
[2368]	SRC	--->	THRA	0.61826	-0.55261
[2369]	SYNCRIP	?	PABPC1	-0.016298	-0.016298
[2370]	NCOA3	<---	NCOR2	-0.035875	0.022175
[2371]	CD79B	<---	SYK	-0.99774	0.99897
[2372]	AR	<---	GSK3B	-1.2513	1.2465
[2373]	ADAM12	--->	IGFBP5	0.073812	-0.058279
[2374]	SMAD2	<---	ZMYND11	-0.56147	0.6382
[2375]	SETDB1	<---	GIPC2	-0.2382	0.25454
[2376]	NPHP1	--->	FLNA	0.18956	-0.17815
[2377]	RB1	<---	PAX2	-0.24155	0.23669
[2378]	FLII	<---	MYD88	-0.021704	0.0084983
[2379]	MAP4K4	--->	SLC9A1	0.043124	-0.026122
[2380]	MMP3	<---	CCL8	-0.16713	0.18806
[2381]	KLK5	--->	DSG1	0.092327	-0.076903
[2382]	SKAP1	--->	FYB	0.041735	-0.059482
[2383]	RIN1	--->	ABL2	1.0027	-0.9916
[2384]	FGFR4	<---	FGF9	-0.99871	1.0053
[2385]	PRKDC	<---	LYN	-0.33317	0.38401
[2386]	CAMKK2	--->	CAMK1	0.83082	-0.43719
[2387]	SNX6	?	SNX2	-0.040204	-0.040204
[2388]	SSTR2	--->	SHANK1	0.047849	-0.057821
[2389]	SMARCA4	<---	STK11	-0.10489	0.098695
[2390]	KAT2B	<---	CREBBP	-0.0041023	0.061921
[2391]	DDX20	--->	ETV3	0.13022	-0.10994
[2392]	FLT1	--->	PLCG1	0.42239	-0.4548
[2393]	VDR	--->	GABARAPL2	0.029552	-0.0053218
[2394]	PGR	--->	RELA	0.049708	-0.049342
[2395]	KIT	--->	JAK2	0.01848	0.014918
[2396]	SYK	<---	FCGR1A	-1.0112	1.0048
[2397]	MAP3K14	<---	MAP3K7	-0.98749	0.99376
[2398]	PRKCA	--->	DLX3	0.91929	-0.91785
[2399]	AFAP1	<---	PRKCA	-1.0819	1.2556
[2400]	ACTL6A	<---	SMARCA2	-0.029019	0.018653
[2401]	MAPKAPK2	--->	ETV1	0.7856	-0.78667
[2402]	LHCGR	--->	LHB	0.38896	-0.35802
[2403]	AR	<---	RBAK	-0.34556	0.3366
[2404]	FMOD	--->	TGFB3	0.47588	-0.44239
[2405]	AKAP10	--->	PRKAR2A	0.049772	-0.026102
[2406]	RBL2	<---	ID2	-0.21793	0.2101
[2407]	LRDD	--->	FADD	0.25387	-0.23906
[2408]	KAT5	--->	CRELD1	0.026733	-0.010751
[2409]	EGFR	--->	STAT3	1.0004	-0.98684
[2410]	JUP	<---	CTNNA1	-0.15422	0.14936
[2411]	SLC16A3	<---	BSG	-0.33701	0.33851
[2412]	BMPR1B	--->	SQSTM1	-0.019424	-0.079946
[2413]	STAT1	--->	CXCR4	0.11627	-0.2398
[2414]	PRKCZ	<---	AKT3	-0.0047278	0.082925
[2415]	CSNK2A1	--->	KIF1C	0.42625	-0.44778
[2416]	PGR	<---	UBE3A	-0.17478	0.17602
[2417]	IRAK3	<---	IRAK1	-1.0016	0.99899
[2418]	ATXN1	<---	KLHL12	-0.020717	0.017117
[2419]	OXSR1	<---	SMURF1	-0.39769	0.38555
[2420]	PLD2	--->	RPTOR	0.0019495	-0.015327
[2421]	SOS1	--->	RANBP9	0.33087	-0.33801
[2422]	MAPK8	--->	JUN	1.0022	-1.0148
[2423]	DLK1	--->	GRN	0.0098531	-0.017159
[2424]	SOCS1	<---	JAK3	-1.0005	1.0007
[2425]	HNRNPL	--->	HNRNPK	0.049773	-0.015394
[2426]	PLXNB1	<---	MET	-0.13486	0.13761
[2427]	BAG1	--->	AR	0.94841	-0.94935
[2428]	CSNK1E	--->	SOCS3	0.46375	-0.39014
[2429]	GRIN3A	<---	GRIN2B	-0.074124	-0.023447
[2430]	KAT5	--->	FAM135B	0.0163	-0.00079297

[2431]	GNB2L1	--->	IFNAR1	0.6437	-0.64496
[2432]	PLG	--->	SERPINC1	0.057207	-0.040728
[2433]	CHD9	--->	RXRA	-0.0060508	-0.0079267
[2434]	BOK	?	BCL2A1	-0.63915	-0.63915
[2435]	AKAP9	<---	PPP2R1B	-0.22048	0.22648
[2436]	CDH9	?	CDH7	-0.058742	-0.058742
[2437]	SH3GL1	--->	SH3GL3	-0.00032092	-0.018568
[2438]	CTNNB1	<---	TAX1BP3	-0.48903	0.49044
[2439]	SHB	<---	JAK3	-0.25447	0.28786
[2440]	MPP3	<---	TRIM41	-0.031732	0.025469
[2441]	JARID2	--->	NKX2-5	0.047645	-0.014945
[2442]	YWHAG	<---	IRS1	-0.049509	0.055105
[2443]	YWHAZ	--->	IRS2	0.072749	-0.080235
[2444]	BCL10	<---	UBE2V2	-0.26796	0.30144
[2445]	EP300	--->	EGR1	0.11396	-0.12213
[2446]	CPSF6	--->	KLHL12	0.23192	-0.19412
[2447]	PIAS3	--->	MITF	0.18029	-0.1816
[2448]	OTX2	<---	FOXA2	-0.39346	0.42236
[2449]	MAPKAPK3	--->	TCF3	0.99488	-0.99939
[2450]	FOXG1	--->	SMAD3	0.52503	-0.44328
[2451]	TJP2	<---	SH3KBP1	-0.094559	0.10051
[2452]	MAP2K3	--->	MAPK14	1.0074	-0.9992
[2453]	SHC1	<---	ZAP70	-0.99981	1.0049
[2454]	GRK5	--->	RHO	0.21152	-0.27051
[2455]	TNF	--->	TNFRSF1B	0.54124	-0.73443
[2456]	SMAD1	<---	SMURF1	-0.86047	0.84042
[2457]	YWHAB	--->	SFRS13A	0.092231	-0.070624
[2458]	PTGIR	--->	GNAS	1.0129	-1.008
[2459]	BTRC	--->	UBE2D2	0.25302	-0.26261
[2460]	FASLG	--->	FAS	0.99951	-1.0013
[2461]	EREG	--->	EGFR	1.005	-1.0102
[2462]	GDF9	<---	SERPINB9	-0.11264	0.15383
[2463]	ADRB1	--->	ARRB1	0.16659	-0.17135
[2464]	ARRB1	<---	HCK	-0.55024	0.64227
[2465]	EP300	--->	STAT3	0.98672	-1.0013
[2466]	YWHAG	--->	CDC5L	0.0801	-0.056124
[2467]	PAK7	--->	BAD	0.40593	-0.40769
[2468]	ATXN1	<---	MYST3	-0.040887	0.037741
[2469]	PLN	--->	ATP2A2	0.19215	-0.19449
[2470]	SFRS13A	<---	FUS	-0.02338	-0.0017915
[2471]	MMP15	--->	LRP1	0.076775	-0.087531
[2472]	RAP2A	--->	RAF1	1.0008	-0.9994
[2473]	DEDD	--->	CASP10	0.61024	-0.37846
[2474]	EED	<---	HDAC2	-0.098152	0.11313
[2475]	TRAF2	--->	CCDC33	0.070837	-0.061452
[2476]	FYN	--->	STAT1	0.72247	-0.56055
[2477]	SYNCRIP	<---	SYT1	-0.039791	0.077746
[2478]	NPHS1	--->	CASK	-0.0077927	-0.0094445
[2479]	ERBB2IP	--->	CTNND1	0.050618	-0.058533
[2480]	CD200R1	--->	CD200	0.050692	-0.032349
[2481]	TRIM37	--->	MCRS1	0.090989	-0.053713
[2482]	JUP	--->	NFKBIE	0.091956	-0.094755
[2483]	KIAA0513	<---	WWC1	-0.14588	0.13454
[2484]	SDCBP	--->	IL5RA	0.13013	-0.14223
[2485]	AVPR2	--->	GNAS	1.0129	-1.008
[2486]	DLG4	--->	NOS1	0.031601	-0.045651
[2487]	CARD9	<---	CCDC85B	-0.10552	0.11737
[2488]	SKAP2	<---	HCK	-0.99957	1.0067
[2489]	SHC2	<---	NTRK3	-0.20347	0.16097
[2490]	IL7R	<---	JAK1	-0.37866	0.37171
[2491]	PDGFRB	<---	FYN	-0.058137	0.049038
[2492]	WNK1	<---	MAP3K2	-0.38123	0.37019
[2493]	PPEF2	--->	CALM1	-0.12978	-0.15561
[2494]	TRIB1	<--->	MAP2K7	0.23429	0.23429
[2495]	SVIL	<---	MYL12A	-0.29047	0.34706
[2496]	SMAD1	<---	STARD13	-0.26881	0.26302
[2497]	TICAM2	--->	TIRAP	0.15275	0.06693
[2498]	U2AF2	?	PUF60	-0.016298	-0.016298
[2499]	MAGI3	<---	DSCAML1	-0.032976	0.027087
[2500]	NTRK1	--->	ABL1	-0.047761	-0.137
[2501]	RHOD	--->	TGFBR1	0.010625	-0.037483
[2502]	CABIN1	<---	YWHAQ	-0.049289	0.073247
[2503]	YWHAG	<---	RAF1	-0.051212	0.022119
[2504]	CSPG4	--->	GRIP2	0.027225	0.0095843
[2505]	NR4A1	<---	RPS6KA6	-0.73191	0.56871
[2506]	CCNT1	--->	AHR	0.13228	-0.11524

[2507]	ARPC1B	--->	ACTR2	0.16143	-0.1326
[2508]	ITGAV	--->	TGFB1	0.0093473	-0.035078
[2509]	CASP3	--->	SREBF2	0.32809	-0.3604
[2510]	E2F6	--->	E2F1	-0.023023	-0.025536
[2511]	CRADD	<---	LRDD	-0.029859	-0.020871
[2512]	NRG2	--->	ERBB4	0.99724	-1.0118
[2513]	ILKAP	<---	ILK	-0.12912	0.12518
[2514]	PPP1CC	--->	EIF2AK2	0.055906	-0.072979
[2515]	IL10	--->	A2M	0.18057	-0.14569
[2516]	PDK1	<---	RPS6KB1	-0.15143	0.0090483
[2517]	HOXA9	--->	MEIS2	0.03787	-0.033829
[2518]	JAK3	--->	JAK1	0.99665	-1.0003
[2519]	RNF115	<---	RAB7A	-0.27058	0.29994
[2520]	BANP	--->	TP53	0.36525	-0.35394
[2521]	EIF2C1	--->	DICER1	0.042243	-0.02712
[2522]	SLA	<---	EPHA2	-0.30775	0.18974
[2523]	ATF4	--->	JUN	-0.076848	-0.15815
[2524]	PTPRJ	--->	EGFR	0.071099	-0.077952
[2525]	PLCG1	<---	CBL	-0.11584	0.094306
[2526]	WNT1	--->	LRP5	0.55875	-0.55683
[2527]	RGS12	<---	MAP2K2	-0.057863	0.13071
[2528]	BTBD1	<---	TRIM5	-0.033059	0.029654
[2529]	FHL2	--->	ATXN1	0.037243	-0.040288
[2530]	YWHAG	--->	CTNND1	0.082332	-0.094252
[2531]	DVL2	<---	$ZBTB8$	-0.21496	0.20928
[2532]	HDAC5	<---	DDX20	-0.025084	0.023843
[2533]	BCL3	<---	HDAC1	-0.095221	0.10143
[2534]	PRKCA	--->	PEA15	0.92276	-0.91829
[2535]	MAP3K7IP2	--->	TNFRSF11A	0.11374	-0.12477
[2536]	SRC	--->	INPPL1	0.49697	-0.27975
[2537]	PTPN22	--->	LCK	0.034522	-0.05555
[2538]	SF3B1	<---	SNIP1	-0.014939	0.031772
[2539]	ULK1	--->	SDCBP	0.006396	-0.020619
[2540]	CCNF	?	CCNB1	-0.38307	-0.38307
[2541]	LIMK1	--->	CFL1	0.16176	-0.17545
[2542]	ZMIZ1	--->	CNTNAP2	0.06216	-0.029426
[2543]	SPIB	?	SPI1	-0.21463	-0.21463
[2544]	HMG20B	--->	CCDC85B	0.1487	-0.12042
[2545]	FYN	<---	PTPRF	-0.099692	0.053276
[2546]	PCBP2	--->	QKI	-0.0053675	-0.11083
[2547]	F2RL2	--->	CDH5	0.35777	-0.34417
[2548]	PIAS4	--->	HTT	0.12157	-0.10986
[2549]	FCAR	--->	PILRA	0.30524	-0.29136
[2550]	FYN	--->	LCP2	1.2119	-0.99119
[2551]	BCL11A	<---	NR2F1	-0.056216	0.04699
[2552]	FADD	<---	RALBP1	-0.087655	0.027542
[2553]	ABCA1	--->	MPP6	0.003993	-0.013301
[2554]	DNM2	<---	SNX9	-0.10091	0.080028
[2555]	SLA	<---	SYK	-0.40218	-0.1324
[2556]	LGALS3BP	<---	FN1	-0.10247	0.14237
[2557]	GNB5	<---	RGS11	-0.33708	0.34928
[2558]	JUN	<---	FOSL1	-0.31682	0.050278
[2559]	CUL4B	--->	AR	0.58227	-0.58829
[2560]	NR2F2	<---	HIPK3	-1.0079	0.99834
[2561]	SFRS17A	<---	NINL	-0.36665	0.40039
[2562]	BCL11A	<---	NR2F2	-0.056216	0.04699
[2563]	CASP8	--->	BID	1.0049	-1
[2564]	MAPK1	--->	NR3C1	0.99834	-1.0079
[2565]	TNFSF13	<---	TNFSF13B	0.19662	0.44795
[2566]	RELA	<---	TBK1	-0.66157	0.65983
[2567]	NUAK2	--->	SMAD4	0.61302	-0.61567
[2568]	CCR10	--->	CCL7	0.49595	-0.46475
[2569]	OCRL	<---	GRB2	-0.098831	0.11042
[2570]	FGFR2	<---	FGF5	-0.53789	0.54173
[2571]	PIAS1	--->	SKIL	0.17254	-0.17818
[2572]	PXN	<---	ABL1	-0.12233	0.023821
[2573]	PER3	?	PER2	-0.0031331	-0.0031331
[2574]	DOCK2	--->	CD247	0.0035628	-0.017391
[2575]	DSCAM	<---	PAK1	-0.032502	0.11978
[2576]	PDLIM1	--->	ACTN4	0.0082818	-0.010186
[2577]	CCDC85B	--->	SYT17	0.022981	0.012422
[2578]	CD163	<---	MYH9	-0.014239	0.0052076
[2579]	CCR5	--->	CCL5	0.49595	-0.46475
[2580]	EIF4E2	<---	EIF4EBP1	-1.0062	1.0033
[2581]	MKNK1	--->	EIF4G3	0.22195	-0.24086
[2582]	IL6ST	<---	ERBB2	-0.084274	0.071043

2585	[2583]	TRIO	--->	PTPRF	-0.0089119	-0.011442
2586	[2584]	PLD1	<---	RAC1	-1.0031	0.99718
2587	[2585]	PLCG1	<---	RET	-0.66968	0.49605
2588	[2586]	SMAP1	<---	SMURF2	-0.70973	0.70586
2589	[2587]	MAP3K12	--->	TGM2	0.47017	-0.46828
2590	[2588]	AURKB	--->	ANKZF1	0.36189	-0.38123
2591	[2589]	CBL	--->	PDGFRB	0.035212	-0.048002
2592	[2590]	HDAC3	<--->	HDAC4	0.015471	0.015471
2593	[2591]	CDC5L	--->	CDKN1A	0.044922	-0.034184
2594	[2592]	NCK2	--->	DOCK1	-0.0041103	-0.0080597
2595	[2593]	SMAD3	--->	ATF2	0.11254	-0.11504
2596	[2594]	SH2B3	<---	KIT	-0.45943	0.40786
2597	[2595]	TSHR	<---	JAK2	-0.10841	0.17632
2598	[2596]	RASD1	<---	GNAI2	-1.0143	1.0038
2599	[2597]	SQSTM1	<---	TGFBR1	-0.23654	0.16304
2600	[2598]	SHC1	<---	MAPK14	-0.26604	0.17517
2601	[2599]	HECTD1	<---	IGSF1	-0.20585	0.20525
2602	[2600]	RHEBL1	--->	EIF4EBP1	0.31457	-0.28483
2603	[2601]	FNBP4	<---	FYN	-0.79101	0.95113
2604	[2602]	BIRC6	--->	USP8	0.050233	-0.035608
2605	[2603]	PDLIM7	<---	UBQLN4	-0.0083895	0.025969
2606	[2604]	PPP2CA	--->	VDR	0.2818	-0.28789
2607	[2605]	ETS1	<---	NR3C1	-0.10264	0.11607
2608	[2606]	CDKN2D	<---	TBC1D17	-0.56989	0.60295
2609	[2607]	S1PR3	<---	HTR1A	0.060056	0.22286
2610	[2608]	GIT1	<---	PPFIA2	-0.16385	0.16054
2611	[2609]	PTPN21	<---	SRC	-0.084678	0.080574
2612	[2610]	SERPINA1	<---	CTSG	-0.082185	0.11833
2613	[2611]	ADAM15	<---	SRC	-0.53287	0.59624
2614	[2612]	CREB5	--->	ATF2	-0.012889	-0.050212
2615	[2613]	AXIN1	<---	SMAD3	-0.018982	0.01973
2616	[2614]	ID4	--->	TCF4	-0.054725	-0.37599
2617	[2615]	CASP8	--->	TRAF1	0.99771	-1.011
2618	[2616]	CTSG	<---	F2RL1	-0.42368	0.45462
2619	[2617]	SKIL	<---	HIPK1	-0.57024	0.56092
2620	[2618]	SMAD1	<---	SQSTM1	-0.21777	0.19388
2621	[2619]	CDK1	--->	MDM4	0.091873	-0.11293
2622	[2620]	SKIL	<---	TRIM62	-0.15996	0.15273
2623	[2621]	ABL1	--->	WASL	0.057901	-0.10978
2624	[2622]	TIE1	--->	TEK	-0.0069793	-0.0070026
2625	[2623]	MTNR1A	--->	RAC1	0.066588	-0.039125
2626	[2624]	TYK2	--->	VAV1	0.047528	-0.17281
2627	[2625]	RASA3	<---	HCK	-0.15418	0.2695
2628	[2626]	LCK	--->	GAB2	0.98856	-0.99701
2629	[2627]	DDX54	--->	NR3C1	0.042552	-0.032266
2630	[2628]	CNTN1	<---	PTPRB	-0.088236	0.096947
2631	[2629]	PTPN11	--->	IFNAR1	1.0145	-1.0055
2632	[2630]	GABPA	<---	SP1	-0.15255	0.14216
2633	[2631]	DACH2	<---	KAT2B	-0.18812	0.1891
2634	[2632]	NLRP1	--->	CASP1	-0.038798	-0.12583
2635	[2633]	PTPN13	--->	PLEKHA1	0.13747	-0.14906
2636	[2634]	ROCK1	--->	MSN	-0.0036985	-0.010475
2637	[2635]	TCERG1	--->	SF3B1	0.1587	-0.14388
2638	[2636]	HNRNPR	<---	PRMT1	-0.28644	0.28801
2639	[2637]	YWHAG	--->	MLLT4	0.26003	-0.2115
2640	[2638]	ADRB1	--->	SH3GL2	0.13854	-0.14363
2641	[2639]	KLF4	--->	ELK1	0.03687	-0.016266
2642	[2640]	PRKACA	--->	RASGRF1	0.99772	-0.99833
2643	[2641]	CD2	<---	CD48	-0.09472	0.051712
2644	[2642]	HRAS	<---	TLR9	-0.25275	0.24769
2645	[2643]	TRIP6	--->	SON	0.023414	-0.0074913
2646	[2644]	SP1	<---	SMAD4	-0.99936	0.98558
2647	[2645]	LIMS1	<--->	LPXN	0.029854	0.029854
2648	[2646]	SMARCD3	--->	NR5A1	0.052806	-0.042542
2649	[2647]	RNF7	<---	CUL1	-0.11034	0.12225
2650	[2648]	HOMER1	--->	RYR1	0.043301	-0.056142
2651	[2649]	PTPRE	--->	YES1	-0.065476	-0.14195
2652	[2650]	HRAS	<--->	RHOD	0.43466	0.43466
2653	[2651]	GTF2I	<---	STAT1	-0.27852	0.15577
2654	[2652]	JUP	--->	CDH2	0.15626	-0.1649
2655	[2653]	HGS	<---	NEDD4	-0.048827	0.031909
2656	[2654]	BAG4	--->	BCL2	0.31288	-0.3263
2657	[2655]	RNASEL	--->	AR	0.44672	-0.4574
2658	[2656]	CD93	<---	COL4A4	-0.042153	0.032217
2659	[2657]	BTRC	--->	TRIM9	0.1925	-0.20388
2660	[2658]	SHC1	<---	CALCOCO2	-0.31346	0.15553

[2659]	CSNK1A1	--->	YWHAZ	0.55799	-0.48053
[2660]	UBE2D2	<---	CBL	-0.27035	0.26107
[2661]	ICAM5	--->	PSEN2	0.066177	-0.066651
[2662]	ACTB	<---	DNASE1	-0.29329	0.29006
[2663]	PLAU	?	PLG	-0.010603	-0.010603
[2664]	NFATC4	<---	YWHAQ	-0.073365	0.077401
[2665]	BNIP2	<---	CREB3	0.055786	0.08833
[2666]	ACAN	--->	MMP8	0.022421	-0.030194
[2667]	YWHAG	<--->	YWHAH	0.069023	0.069023
[2668]	CDKN2A	<--->	ANKRD12	0.076747	0.076747
[2669]	PIAS1	--->	SP3	0.14645	-0.14387
[2670]	OPRK1	<---	OPRD1	0.24405	0.31807
[2671]	VAV3	<---	RHOG	-0.14624	0.08839
[2672]	MSN	<---	VCAM1	-0.042277	0.02951
[2673]	PRKCZ	<---	GSK3B	0.012708	0.1627
[2674]	GSK3B	--->	CCNE1	1.0544	-1.0774
[2675]	TRA2B	?	RBMX	-0.016298	-0.016298
[2676]	KLHL12	<---	CUL3	-0.15416	0.16535
[2677]	QKI	<---	RBPMS	-0.097474	0.10067
[2678]	FHOD1	<---	RNF5	-0.050133	0.053638
[2679]	SPOP	--->	H2AFY	0.15002	-0.12299
[2680]	PRKCZ	--->	GRM5	0.26626	-0.27969
[2681]	PTPN13	--->	FASLG	0.12909	-0.13921
[2682]	NCSTN	--->	APP	0.12874	-0.14122
[2683]	CFI	<---	C3	-0.084317	0.083569
[2684]	TRAF6	--->	NGFR	0.2738	-0.24989
[2685]	CDKN3	<---	CDK3	-0.56626	0.54604
[2686]	SYT1	--->	PPARG	0.060829	-0.048345
[2687]	PRPF4	<---	TGFBR1	-0.40828	0.3741
[2688]	CDC34	--->	CUL1	0.040175	-0.038342
[2689]	F2	--->	SERPINC1	0.057207	-0.040728
[2690]	PRKCA	--->	DVL2	1.0137	-0.99339
[2691]	NEDD9	--->	PXN	0.083267	-0.092609
[2692]	PTK2B	--->	ITGB2	0.081194	-0.099332
[2693]	TRIP6	<---	PTPN13	-0.18144	0.17025
[2694]	RNF11	<---	UBE2N	0.0038168	0.028353
[2695]	TP53BP1	--->	CRB2	0.062175	-0.067294
[2696]	KAT5	--->	ETV6	0.078098	-0.079148
[2697]	SNX1	<---	PDGFRB	-0.046812	0.040725
[2698]	MEGF10	--->	CUL7	-0.0040642	-0.0090146
[2699]	CEBPB	<---	HMGB1	-0.27554	0.32452
[2700]	IL2RG	<---	ICAM1	-0.11243	0.095457
[2701]	PVRL4	<---	PVRL1	-0.021316	0.00037536
[2702]	RPS6KA3	--->	CREB1	1.0082	-0.98976
[2703]	PAK2	<---	LCK	-0.66116	0.69113
[2704]	PGS1	--->	GABARAP	0.065664	-0.051948
[2705]	PDGFB	--->	PDGFRB	0.33373	-0.33498
[2706]	CASP8	--->	PSEN1	0.56121	-0.70272
[2707]	HOXB2	<---	CREBBP	-0.18943	0.18505
[2708]	NUP210	--->	TRIP6	0.051706	-0.016776
[2709]	EFEMP2	--->	ZNF426	0.19714	-0.20139
[2710]	MAPK1	--->	TNFRSF1A	0.23445	-0.27811
[2711]	PDLIM1	--->	PGS1	0.026092	-0.020862
[2712]	DVL2	<---	TIFA	-0.38498	0.38481
[2713]	ADRA1A	--->	RGS2	0.4217	-0.41683
[2714]	DOK3	<---	LCK	-0.88457	0.95892
[2715]	MMP25	?	MMP2	-0.12951	-0.12951
[2716]	RELA	--->	NFKBIA	0.10773	-0.11425
[2717]	NR2F6	?	NR2F2	-0.0089162	-0.0089162
[2718]	KAT5	--->	GADD45G	0.072949	-0.057531
[2719]	ANKRD23	<---	MYPN	-0.65168	0.68126
[2720]	MAGI2	<---	ATN1	-0.022182	0.012418
[2721]	YWHAB	--->	TSC2	0.294	-0.29138
[2722]	SMAD3	--->	MYC	0.39156	-0.40036
[2723]	GNA15	<---	CNR1	-0.99753	1.0047
[2724]	BIRC6	--->	BIRC5	-0.0037426	-0.08901
[2725]	BMPR1B	<---	OXSR1	-0.099843	0.1515
[2726]	DLX2	?	MSX2	-0.11417	-0.11417
[2727]	RASGRF1	<---	YWHAE	-0.28013	0.27719
[2728]	SH3GL3	--->	WASL	0.086472	-0.11025
[2729]	YWHAE	<---	CDK16	-0.66368	0.62776
[2730]	LILRB2	--->	FCGR1A	0.29591	0.26857
[2731]	CD2AP	--->	CD2	0.15646	-0.167
[2732]	HSP90AA1	<---	PRKCD	-0.058381	0.047327
[2733]	RAD23B	--->	PSMD4	0.076162	-0.042104
[2734]	NRAS	--->	RAF1	1.0008	-0.9994

```
[2735]  SCN2A   <---    RNF130  -0.11598        0.13028
[2736]  LRP1    --->    APP     0.0068317       -0.020511
[2737]  NID2    <---    HSPG2   -0.025974       0.01282
[2738]  SRC     --->    AFAP1   1.2771  -1.0835
[2739]  CRK     --->    ATXN1   0.03144 -0.050299
[2740]  YWHAG   --->    IRS2    0.072749        -0.080235
[2741]  ZNF446  --->    ZNF434  -0.011024       -0.16454
[2742]  RIT2    --->    MLLT4   0.99712 -0.98672
[2743]  TRIM37  <---    TRAF4   -0.0088131      0.14976
[2744]  GRIN1   <---    CALM1   -0.051624       0.059876
[2745]  MAPK3   --->    EPOR    0.44759 -0.47318
[2746]  TES     --->    ACTL7A  0.3238  -0.29249
[2747]  TAX1BP1 <---    TBC1D17 -0.030895       0.051459
[2748]  EGFR    --->    MET     0.11795 0.096582
[2749]  SMAD4   <---    MEF2A   -0.052689       0.061596
[2750]  CXCL12  --->    MMP2    0.18806 -0.16713
[2751]  MAP3K14 --->    IKBKG   0.83996 -0.91119
[2752]  DMPK    --->    RAF1    0.98888 -1.0049
[2753]  BIRC7   <---    CASP3   -0.38127        0.30534
[2754]  LAMA4   --->    ZHX1    0.11883 -0.10377
[2755]  CRHR1   <---    GNAS    -0.43656        0.4334
[2756]  RAN     --->    SPAG8   0.4016  -0.37076
[2757]  GCA     --->    LCP1    0.31529 -0.30918
[2758]  IGF1    --->    IGFBP5  0.087568        -0.072295
[2759]  PPP2R3A --->    PPP5C   -0.0026202      -0.0097349
[2760]  LIMK2   <--->   LIMK1   0.0039985       0.0039985
[2761]  UBE2G2  <---    UBE3A   -0.17948        0.18395
[2762]  XIAP    <---    TGFBR1  -0.16077        0.12707
[2763]  GP6     <--->   FCGR3A  0.45908 0.45908
[2764]  HSPA2   <---    MAP3K3  0.070258        0.18659
[2765]  TP53    <---    ACTA1   -0.023565       0.020417
[2766]  BGN     --->    TGFB1   1       -0.99971
[2767]  GP6     <---    FYN     0.015127        0.11748
[2768]  SRPK1   --->    SFRS6   0.45945 -0.481
[2769]  HDAC4   <---    MAPK3   -0.71414        0.68838
[2770]  CSNK1D  --->    TP53    0.361   -0.31701
[2771]  YWHAB   --->    MICALL1 0.046853        -0.036876
[2772]  EIF4EBP3        --->    EIF4E   1.0033  -1.0062
[2773]  TP53I3  <---    UBR1    -0.078803       0.11308
[2774]  CLCN3   --->    SLC9A3R1        0.74042 -0.72583
[2775]  PTPN11  <---    GAB1    -0.0502 0.060242
[2776]  LPHN1   <---    SHANK2  -0.094156       0.087413
[2777]  BCAR1   <---    PTPRF   -0.018184       0.0066364
[2778]  CTLA4   --->    STAT5A  0.25636 -0.27627
[2779]  ATF2    <---    NBN     -0.3562 0.35631
[2780]  RAC1    --->    EIF2AK2 1.0132  -1.0074
[2781]  CAPN1   --->    BID     0.27617 -0.27318
[2782]  BMP2    --->    ACVR2B  1.0057  -0.99943
[2783]  MYO6    --->    ACTA1   0.32138 -0.3252
[2784]  CDH10   ?       CDH6    -0.058742       -0.058742
[2785]  DLG2    --->    FZD1    0.040925        -0.056386
[2786]  NUCB1   <---    PTGS2   -0.16009        0.17762
[2787]  ID3     <---    PAX5    -0.10726        0.13137
[2788]  CAV1    --->    INSR    0.13911 -0.14955
[2789]  RGS2    --->    ARFGAP1 0.44588 -0.41625
[2790]  TFRC    --->    CD247   0.023907        -0.037436
[2791]  GRIN2B  <---    CAMK2D  -0.30134        0.28954
[2792]  TIAM1   <---    CD44    -0.10098        0.089263
[2793]  WT1     <---    AREG    -0.017527       0.011376
[2794]  RPS6KB1 --->    PPP1R9B 0.22846 -0.45034
[2795]  SMURF2  --->    DGCR2   0.43608 -0.44407
[2796]  ID3     ?       MYF5    -0.65635        -0.65635
[2797]  PIAS1   --->    ZNF76   0.029951        -0.036856
[2798]  KCNMA1  --->    MAP1A   0.014567        0.00095988
[2799]  PPP1CB  --->    RB1     0.14829 -0.16031
[2800]  SFRS9   ?       TRA2B   -0.016298       -0.016298
[2801]  RORC    <---    CHD4    -0.0098881      -0.00079799
[2802]  DDR1    --->    NCK2    0.010472        -0.088179
[2803]  LGALS3BP        <---    COL5A1  -0.087119       0.08699
[2804]  DMWD    <---    PPBP    -0.050157       0.064738
[2805]  ZBTB16  <---    TRIM27  -0.054725       0.045447
[2806]  HDAC9   --->    CBX5    0.02893 0.0052106
[2807]  MAP3K7  --->    HIPK2   0.99376 -0.98749
[2808]  RAB32   <---    PRKAR2A -0.062032       0.093879
[2809]  DNM1    <---    ASB13   -0.025223       0.014231
[2810]  NEDD9   <---    LCK     -0.19322        0.2639
```

[2811]	DEDD2	--->	CASP10	0.61024	-0.37846
[2812]	COL4A6	--->	SERPINE2	0.071404	-0.073538
[2813]	SOCS3	--->	IRS1	0.11723	-0.050782
[2814]	ITGAD	<---	ICAM3	-0.018738	0.005216
[2815]	THRA	?	RXRB	-0.0089162	-0.0089162
[2816]	AKT1	<---	MTOR	-0.99349	0.99887
[2817]	TOP2B	<--->	TOP2A	0.19589	0.19589
[2818]	CCR5	<--->	CCR2	0.49511	0.49511
[2819]	SHC1	<---	NGFR	-0.20006	0.12156
[2820]	MYPN	<---	ACTN2	-0.081334	0.083986
[2821]	PPM1F	--->	PAK1	0.15934	0.12966
[2822]	PTPN11	--->	SELE	0.58187	-0.48449
[2823]	RARA	?	THRB	-0.0089162	-0.0089162
[2824]	RARA	<---	NKX2-1	0.012013	0.012582
[2825]	TRAF5	<---	LTBR	-0.018319	0.010258
[2826]	HOPX	<---	HDAC2	-0.075969	0.090466
[2827]	TEX11	--->	COL20A1	0.0015303	-0.012022
[2828]	JUND	--->	BCL6	0.11317	-0.14009
[2829]	DUSP10	--->	MAPK10	0.87425	-0.89445
[2830]	RHOD	<---	SMURF2	-1.0084	1.0007
[2831]	GRIN1	<---	MYH9	-0.019321	0.007114
[2832]	PTPN11	--->	IRS1	0.58189	-0.41492
[2833]	SNTB2	<---	DMD	-0.052248	0.048407
[2834]	GRB7	--->	PTK2	-0.044378	-0.1636
[2835]	UBE2A	--->	TP53	0.43525	-0.42405
[2836]	SERPINA5	<---	F10	-0.016469	0.01376
[2837]	PAK2	--->	SH3RF1	0.082023	0.012028
[2838]	SMYD1	--->	HDAC2	0.039173	-0.022235
[2839]	DLG2	--->	WNT3A	0.14258	-0.13955
[2840]	HSPG2	--->	FBLN1	0.039181	-0.050966
[2841]	PRPF40A	<--->	WBP4	0.34574	0.34574
[2842]	GRB10	<---	EGFR	-0.42553	0.37299
[2843]	CBL	--->	RET	0.52296	-0.53538
[2844]	GDF6	--->	BMPR1A	0.99697	-1.0007
[2845]	TRAF5	<---	CD27	-0.1819	0.17914
[2846]	FURIN	<---	NAGPA	-0.010292	-0.00123
[2847]	CAV1	<---	ILK	-0.41101	0.39727
[2848]	MAST2	--->	SLC9A3	0.24426	-0.25789
[2849]	HDAC2	<--->	HDAC1	0.0081462	0.0081462
[2850]	ATN1	--->	LTBP1	-0.0039514	-0.011042
[2851]	HRAS	<---	NF1	-0.99605	1.0036
[2852]	SOX9	<---	TRAF2	-0.05242	0.039818
[2853]	PPP2R1B	--->	CFTR	0.13437	-0.14352
[2854]	TAX1BP1	<---	ARHGAP1	-0.046313	0.052926
[2855]	PARD6A	<---	PARD6B	-0.039467	-0.0067604
[2856]	NCKIPSD	<---	MAPK3	-0.50306	0.44174
[2857]	INSR	--->	SHC1	0.10914	-0.14379
[2858]	PDE9A	<---	PHYHIPL	-0.13482	0.16587
[2859]	CIB1	<---	NBR1	-0.22599	0.23137
[2860]	IL6	--->	SH3GL2	0.07496	-0.070483
[2861]	CACNB4	--->	TBL3	0.12567	-0.092378
[2862]	FZD2	<---	DLG4	-0.14014	0.11867
[2863]	MAPK8IP1	--->	MAPK8IP3	0.0007298	-0.0080062
[2864]	MINK1	--->	SPTBN2	0.028672	-0.037008
[2865]	BRCA1	--->	SMAD3	0.25767	-0.21264
[2866]	CRKL	<---	KIT	-0.5027	0.10598
[2867]	TAF1	<---	MDM2	-0.049188	0.039184
[2868]	NFKBIB	<---	CHUK	-1.0011	1.0006
[2869]	SCUBE3	?	SCUBE1	-0.013711	-0.013711
[2870]	TNFRSF11B	--->	THBS1	0.091293	-0.073731
[2871]	TLE1	<---	CCL18	-0.077632	0.11072
[2872]	AKT1	--->	BRAF	0.65966	-0.67061
[2873]	RSU1	--->	LIMS1	0.050645	-0.047088
[2874]	SHC1	<---	CD22	-0.063757	0.014474
[2875]	PDPK1	--->	RPS6KA3	1.0929	-1.1695
[2876]	BRD3	<--->	BRD7	0.055107	0.055107
[2877]	IFNAR2	--->	STAT2	0.09213	-0.12667
[2878]	CLK1	--->	PRPF4	0.83716	-0.86422
[2879]	ACACA	<---	BRCA1	-0.25516	0.24466
[2880]	YWHAG	--->	RNPS1	0.092231	-0.070624
[2881]	DTNA	<---	DRP2	-0.039526	0.036041
[2882]	GRAP2	--->	CD28	0.44787	-0.4617
[2883]	DLG4	<---	LYN	-0.27518	0.41392
[2884]	CTNND2	--->	CTNNB1	-0.0025414	-0.33209
[2885]	CD36	<---	YES1	-0.94926	1.109
[2886]	FLNB	<---	PSEN1	-0.033635	0.067884

[2887]	PSEN2	--->	BCL2L1	0.78024	-0.77257
[2888]	HDAC1	--->	STAT3	0.34132	-0.37904
[2889]	PRKCA	--->	GRIA1	0.19252	-0.20227
[2890]	GSK3B	<---	AKT2	-1.0071	1.0168
[2891]	JAK1	--->	CXCR4	0.52384	-0.53456
[2892]	YWHAE	--->	CDC25B	0.14141	-0.11975
[2893]	CCBP2	--->	CCL7	0.49595	-0.46475
[2894]	MAP4K5	--->	BCR	0.079818	-0.055203
[2895]	CHUK	--->	NCOR2	-0.0069968	-0.022849
[2896]	RASAL2	--->	NFKBIB	0.27579	-0.25877
[2897]	QKI	<---	PCBP4	-0.11083	-0.0053675
[2898]	CHUK	--->	FOXO3	0.59827	-0.60135
[2899]	CDK2	--->	CDKN1A	0.51838	-0.55156
[2900]	HGS	<---	UBQLN1	-0.061475	0.0099058
[2901]	CDK7	--->	E2F1	0.44723	-0.46975
[2902]	NR1H3	<---	ACTN1	-0.022484	0.017718
[2903]	RALA	<---	PPP2R1B	-0.13281	0.13801
[2904]	MYC	<---	RELA	-0.027622	0.022396
[2905]	ANK3	--->	PIK3R1	0.026216	-0.061984
[2906]	BCR	--->	YWHAG	0.078735	-0.083313
[2907]	KLHL3	<---	SYNCRIP	-0.036142	0.066603
[2908]	TFEB	?	MITF	-0.083565	-0.083565
[2909]	FOS	<---	DDIT3	-0.41862	0.28837
[2910]	RNF130	<---	TGFBR1	-0.2038	0.17727
[2911]	TSC2	<---	GSK3B	-1.0077	1.0125
[2912]	PRKAR1A	--->	PRPF40A	0.081111	-0.050971
[2913]	CHUK	--->	TNFRSF1A	0.20401	-0.33958
[2914]	TEK	--->	PIK3R1	0.003312	-0.022596
[2915]	NONO	--->	SPI1	0.20319	-0.16348
[2916]	BACE2	--->	APP	0.36802	-0.37802
[2917]	GNA13	<---	S1PR3	-0.51255	0.50718
[2918]	PHLDA1	<---	HSPA4L	-0.78532	0.82746
[2919]	CCL5	--->	VCAN	0.010191	-0.021003
[2920]	SMARCD3	--->	CCNE1	0.39134	-0.38869
[2921]	TBL1XR1	--->	CORO2A	-0.053138	-0.062871
[2922]	RACGAP1	<---	RND2	-0.3141	0.32343
[2923]	GRK1	--->	RHO	0.29762	-0.3178
[2924]	GUCY2C	--->	PDZD3	0.13965	-0.151
[2925]	TGFBR1	--->	WDR61	0.3741	-0.40828
[2926]	ZBTB16	<---	GATA2	-0.086971	0.087346
[2927]	EFEMP2	--->	CATSPER1	0.025848	0.0091944
[2928]	PLK3	<---	CHEK2	-0.00064284	0.1132
[2929]	SMAD5	--->	BMP6	0.060031	-0.055306
[2930]	MAPK8IP3	<---	MAPK8IP2	-0.0080062	0.0007298
[2931]	ABL1	--->	RB1	0.015042	-0.059161
[2932]	PPP1CA	--->	H2AFX	0.25877	-0.26197
[2933]	A1CF	--->	KHSRP	0.036464	-0.0048144
[2934]	MAP4K4	--->	ITGB1	0.0073229	-0.011138
[2935]	PIK3CA	--->	RPS6KB1	1.0159	-0.98663
[2936]	MAPKAPK2	--->	YWHAZ	0.4819	-0.50916
[2937]	GRM4	<---	FLNA	0.0058519	0.028788
[2938]	DOK1	--->	ITGB2	0.21218	-0.22108
[2939]	NTNG1	<---	LRRC4C	-0.037819	0.035622
[2940]	SETDB1	<---	HIST1H3A	-0.15166	0.17341
[2941]	LAMA3	--->	PLG	-0.00023876	-0.007205
[2942]	MX1	--->	TRPC5	0.019766	-0.032989
[2943]	RGS12	--->	CACNA1B	0.016049	-0.027666
[2944]	COMP	<---	COL2A1	-0.023245	0.013585
[2945]	MMP14	<---	CAV1	-0.19134	0.20837
[2946]	GRIN2B	<---	ACTN2	-0.070956	0.061729
[2947]	PLCD4	<---	TRIM39	-0.097807	0.093734
[2948]	EZH2	--->	WSB2	0.11772	-0.10187
[2949]	CHAF1B	<---	CDK1	-0.86422	0.83716
[2950]	SERPINI2	<---	UBQLN4	-0.14362	0.17983
[2951]	PKN2	--->	PLCG1	0.031458	-0.049236
[2952]	AMFR	<---	UBE2G2	-0.0077172	0.021366
[2953]	ITGA2B	<---	FGA	-0.037668	0.027138
[2954]	PRKACA	--->	ETV1	1.1285	-1.1212
[2955]	MAPK1	--->	MCL1	1.2421	-1.2851
[2956]	RUNX2	--->	JUN	0.43801	-0.48322
[2957]	PXN	<---	MAPK1	-0.59278	0.57323
[2958]	CD22	<---	LCP2	-0.10427	0.058573
[2959]	PRKCA	--->	CASR	0.55548	-0.55976
[2960]	PRKACA	--->	RYR2	1.0028	-0.99449
[2961]	SPI1	<---	FUS	-0.13112	0.14996
[2962]	NCOA3	<---	PPARG	-0.084046	0.071285

[2963]	CALCOCO1	<---	EP300	-0.187	0.18273
[2964]	NXF1	--->	STRAP	0.16628	-0.13408
[2965]	FYN	--->	CTLA4	0.99605	-0.98986
[2966]	CDK9	--->	CCNK	0.64437	-0.6658
[2967]	SYNJ1	<---	PPP3CA	-0.16793	0.16162
[2968]	TP53BP2	--->	BCL2	0.30334	-0.31189
[2969]	CRKL	--->	ETV6	0.071068	-0.089709
[2970]	CSNK1A1	--->	CREM	0.30471	-0.29126
[2971]	TCF12	<---	CREBBP	-0.040649	0.02796
[2972]	PVRL3	--->	MLLT4	0.092305	-0.083375
[2973]	DOCK8	<---	RHOJ	-0.041827	0.031606
[2974]	NRK	--->	CFL1	0.34542	-0.29737
[2975]	HTT	<---	TRIP10	-0.11641	0.12087
[2976]	PIK3R1	<---	IL2RB	-0.47835	0.373
[2977]	RIPK1	<---	TICAM1	-0.73839	-0.024648
[2978]	DEF6	<---	RAC1	-0.26331	0.26775
[2979]	MEOX2	<---	RND2	-0.47042	0.49898
[2980]	PPBP	<---	CTSG	-0.024098	0.058311
[2981]	CREBBP	--->	YWHAH	0.17985	-0.18448
[2982]	PRKCZ	<---	CDC42	-1.0135	0.99149
[2983]	TSHR	--->	GNAQ	0.65587	-0.65379
[2984]	IL1R2	<---	IL1A	-0.021437	0.026308
[2985]	CARD17	--->	CASP1	-0.076001	-0.18686
[2986]	IGSF21	--->	GIPC2	0.24012	-0.24239
[2987]	ERBB2	--->	GRB2	1.0102	-1.0038
[2988]	ITGA4	<---	PRKAR1A	-0.11857	0.11189
[2989]	HDAC1	--->	ARID4A	0.036281	-0.031925
[2990]	BLNK	<---	BTK	-0.24918	0.40931
[2991]	ERBB4	--->	CRK	0.80872	-0.80583
[2992]	NCOR2	--->	RELA	0.052857	-0.06493
[2993]	ATN1	--->	ALG13	-0.0015197	-0.0099854
[2994]	LHB	<---	GNAS	-0.09586	0.11125
[2995]	ATXN1	<---	ACOT7	-0.056594	0.052176
[2996]	BRAF	<---	YWHAH	-0.19664	0.18212
[2997]	HDAC10	<---	HDAC2	0.0010361	0.021264
[2998]	PTPN3	--->	ZAP70	0.13099	-0.18486
[2999]	TRAF3	<---	CD27	-0.13411	0.1285
[3000]	ITGA4	<---	ITGB7	-0.014391	0.003305
[3001]	PRMT2	--->	RXRA	0.073326	-0.20664
[3002]	ACVR2B	<---	MSTN	-0.99943	1.0057
[3003]	YWHAG	--->	LMO7	0.0048613	-0.013105
[3004]	PTPN6	--->	LYN	0.3856	-0.16887
[3005]	GFI1B	<---	RCHY1	-0.17975	0.17558
[3006]	HCLS1	--->	CD79A	0.1214	-0.12342
[3007]	IGF2	--->	IGFBP6	0.11742	-0.083423
[3008]	CDC25A	<---	YWHAE	-0.11975	0.14141
[3009]	MEP1A	--->	FN1	0.08568	-0.071738
[3010]	C1QC	<---	C1QA	-0.0046012	0.011291
[3011]	PLK1	<---	WEE1	0.0029423	0.19171
[3012]	TEC	--->	KIT	0.28948	-0.1651
[3013]	BAIAP2	--->	SHANK1	0.011324	-0.022782
[3014]	FGF13	<---	SCN8A	0.0085351	0.024966
[3015]	PPP1R12A	<---	RHOA	-0.044789	0.046504
[3016]	LAMC2	--->	NID1	0.033982	-0.036574
[3017]	IL2RB	<---	PTPN6	-1.0077	0.99507
[3018]	IL4R	--->	DOK2	0.070339	-0.054153
[3019]	CCL2	--->	VCAN	0.010191	-0.021003
[3020]	PLAGL2	<---	RCHY1	-0.28677	0.3209
[3021]	SYK	--->	BTK	1.0133	-1.0127
[3022]	BUB1	--->	RAE1	0.83716	-0.86422
[3023]	MCF2L	<---	RAC1	-0.014357	0.0012594
[3024]	NOTCH2NL	<---	ELANE	-0.017846	0.0046348
[3025]	AR	<---	RUNX2	-0.9578	0.010403
[3026]	SOS1	--->	PIK3R2	0.0016367	-0.015104
[3027]	PRKACA	--->	LCP1	0.62336	-0.63109
[3028]	CCNA1	--->	RB1	0.99958	-1.0044
[3029]	CASP8	--->	PARK2	0.53428	-0.67568
[3030]	TCERG1	<---	HSPA5	-0.31176	0.33101
[3031]	ATP2A2	<---	IRS1	-0.14511	0.13686
[3032]	CNN1	--->	ACTA1	0.51886	-0.51601
[3033]	SOX8	--->	NR5A1	0.0063633	-0.0075123
[3034]	MARK4	--->	MAP4	0.063532	-0.077186
[3035]	KRT15	--->	ABI2	0.0016695	-0.0040478
[3036]	ZNF398	<---	ESR1	-0.094297	0.096115
[3037]	AURKB	--->	TBL3	0.83716	-0.86422
[3038]	TSC2	<---	FBXL6	-0.30052	0.30532

[3039]	EPB41L3	<---	YWHAQ	-0.057222	0.05792
[3040]	NOTCH1	<---	NUMBL	-0.60943	0.60106
[3041]	PIAS2	--->	AR	0.5819	-0.58792
[3042]	CHEK1	--->	XIAP	0.06991	-0.12533
[3043]	CREBBP	--->	EGR1	0.11396	-0.12213
[3044]	MAP2K4	<--->	MAP3K8	0.23429	0.23429
[3045]	CLK1	--->	SFRS1	0.72994	-0.75755
[3046]	NCR1	--->	FCER1G	0.29882	-0.30643
[3047]	SMAD3	<---	PARD3	-0.12329	0.14536
[3048]	NR4A1	--->	FXYD3	0.03761	-0.0087257
[3049]	KLF1	--->	FLI1	0.0012399	-0.0011529
[3050]	CSNK2A1	--->	HOXB7	0.79979	-0.82765
[3051]	FOXG1	--->	GDF9	0.56093	-0.52506
[3052]	SERPINB8	<---	F2	-0.040728	0.057207
[3053]	PTPN11	--->	PRLR	0.042363	-0.04607
[3054]	SYNCRIP	--->	INSR	0.14005	-0.14861
[3055]	SYT3	<---	SYT5	0.011511	0.022828
[3056]	EHBP1	--->	EHD1	0.043222	-0.028219
[3057]	XAF1	<---	XIAP	-0.091572	0.092968
[3058]	MTOR	--->	RPS6KB2	1.0159	-1.0002
[3059]	ZBTB32	<---	GATA2	-0.20717	0.20796
[3060]	DLG4	--->	WNT3A	0.22514	-0.23036
[3061]	ATN1	--->	RBM9	0.038991	-0.041558
[3062]	SAV1	<---	STK3	-0.13326	0.13679
[3063]	GRID2	<---	GOPC	-0.17792	0.18588
[3064]	GSK3B	--->	CCND1	1.0544	-1.0774
[3065]	FBXW7	--->	CCNE1	0.38997	-0.39495
[3066]	PTK2B	--->	RASA1	-0.096326	-0.16442
[3067]	PPM1A	--->	CACNA1C	0.31658	-0.32848
[3068]	RBBP4	--->	RBBP7	-0.087404	-0.10491
[3069]	OPRM1	--->	CALM1	0.42043	-0.40552
[3070]	DVL2	<---	SMURF1	-0.16055	0.14913
[3071]	MFNG	--->	NOTCH2	0.99253	-0.99707
[3072]	YWHAG	<---	PRKCQ	-0.61814	0.66375
[3073]	AR	<---	STAT3	-0.10732	0.092863
[3074]	CAV1	--->	PDGFRA	0.11429	-0.11483
[3075]	MYLK	--->	CTTN	0.23126	-0.23776
[3076]	MMP1	<---	SERPINA3	-0.13073	0.15283
[3077]	FCGR2B	<---	PTPN6	-0.077948	0.26319
[3078]	VASP	<---	NOL3	-0.015311	0.031841
[3079]	NCOA1	--->	CCND1	0.03342	-0.04366
[3080]	IGFBP4	<---	HK3	-0.024856	0.039936
[3081]	RHOXF2	<---	RBM11	-0.071742	0.10141
[3082]	CCNA2	<---	CDK2	-1.0774	1.0544
[3083]	MAP4K3	--->	SH3GL2	0.27127	-0.1674
[3084]	F2	<---	FGA	-0.0052456	0.020403
[3085]	SYK	--->	ITGB2	0.17338	-0.18438
[3086]	CRKL	<---	FCGR1A	-0.27408	0.2249
[3087]	BCAR1	<---	PTK2B	-0.15041	-0.01537
[3088]	TBC1D3F	<---	RAB5A	-0.35867	0.38941
[3089]	CCBP2	--->	CCL2	0.49595	-0.46475
[3090]	PYY	--->	NPY5R	0.20502	-0.19468
[3091]	SASH3	--->	SMAD4	0.26998	-0.21835
[3092]	BACE1	--->	MMP2	0.33668	-0.31691
[3093]	CSNK2A1	--->	MEF2C	0.43947	-0.44112
[3094]	ATM	<---	STK11	-0.14308	0.13159
[3095]	DSTN	--->	ACTG1	0.30201	-0.30587
[3096]	NCOR2	--->	NR2F2	0.13736	-0.14734
[3097]	PRKCD	--->	GNAZ	0.77273	-0.76398
[3098]	TP73	<---	GNB2L1	-0.011231	0.028214
[3099]	SYT11	--->	SYNCRIP	0.077746	-0.039791
[3100]	PICK1	--->	GRM3	0.0626	-0.025484
[3101]	TSC2	<---	PAM	-0.28517	0.29006
[3102]	CEBPE	--->	SPI1	0.11084	-0.052983
[3103]	PKD1	<---	JAK2	-0.11022	0.17814
[3104]	CCNB2	<---	CDKN1A	-0.99961	0.98767
[3105]	TIRAP	<---	TICAM1	-0.10289	0.093965
[3106]	MLXIPL	?	MLX	-0.65635	-0.65635
[3107]	HSPA13	--->	UBQLN4	0.78427	-0.7421
[3108]	CTTN	<---	ANKZF1	-0.063263	0.10164
[3109]	KHDRBS1	<---	PTPN6	-0.39754	0.45935
[3110]	LDLR	<---	DAB1	-0.14343	0.1387
[3111]	MTNR1A	--->	HNRNPA0	0.46527	-0.43669
[3112]	RAD54B	--->	ZNF580	0.021981	-0.0063093
[3113]	NOTCH1	<---	RELA	-0.16634	0.15143
[3114]	AR	<---	CTNNB1	-0.02683	0.01418

[3115]	ERAS	--->	PIK3R1	0.21	-0.30832
[3116]	PPM1F	--->	ARHGEF7	0.065558	-0.069928
[3117]	MAPK8IP3	<---	MAPK9	-0.16592	0.14982
[3118]	PBX2	--->	PKNOX1	0.012187	-0.0083903
[3119]	CREBBP	--->	MLL	0.10533	-0.1134
[3120]	TRIP6	<---	PTK2	-0.4799	0.41189
[3121]	DMPK	--->	UBQLN4	0.6472	-0.67308
[3122]	FLI1	--->	SMAD9	0.03171	-0.04522
[3123]	CASP10	--->	RB1	0.33695	-0.35751
[3124]	CFTR	<---	EZR	-0.17892	0.17092
[3125]	SYK	--->	FCER1G	0.82866	-0.8746
[3126]	GNAI2	<---	ADORA1	-1.008	1.0129
[3127]	GIGYF1	<---	GRB10	-0.15218	0.073782
[3128]	MMP3	<---	SERPINE1	-0.13073	0.15283
[3129]	JAK2	--->	GTF2I	0.29776	-0.17667
[3130]	RNF4	--->	GSC2	0.20315	-0.18996
[3131]	BCL2L1	<---	MAPK8	-1.2078	1.1791
[3132]	FGFR3	<---	FGF3	-0.61087	0.60861
[3133]	NF2	--->	CTNNB1	0.30607	-0.3116
[3134]	HSPB1	<---	EIF4G1	-0.20723	0.25404
[3135]	BAX	<---	ERN1	-0.78594	0.75438
[3136]	GPR50	<--->	MTNR1A	0.49511	0.49511
[3137]	OPRD1	--->	GNAZ	1.0129	-1.008
[3138]	CREBBP	--->	FHL2	0.18135	-0.18563
[3139]	RPS6KA5	--->	BAD	0.58958	-0.91274
[3140]	CD3E	<---	SYK	-1.047	1.0695
[3141]	ERBB4	--->	SHC1	0.66297	-0.66369
[3142]	DLG4	<---	HTR2A	-0.056804	0.049427
[3143]	RUNX2	--->	ETS1	0.22693	-0.20626
[3144]	PPP1CA	--->	APAF1	0.1544	-0.16434
[3145]	CAPN3	--->	NECAB2	0.33523	-0.33403
[3146]	CDC42	--->	ARHGEF6	0.032222	-0.0405
[3147]	ARIH1	<---	UBE2L3	-0.026177	0.029733
[3148]	APLP1	--->	CDKN2C	0.079611	-0.082763
[3149]	SPARC	--->	PLAT	0.027998	-0.02429
[3150]	MAPK3	--->	RPS6KA1	1.002	-1.0109
[3151]	ITGA2B	<---	COL2A1	-0.013702	0.00020191
[3152]	TCF20	<---	RNF4	-0.128	0.14237
[3153]	ATXN1	<---	NUMBL	-0.01263	0.0027183
[3154]	KAT5	<---	EP300	-0.11551	0.1074
[3155]	E2F5	--->	RBL2	0.28436	-0.29297
[3156]	DSG1	<---	PKP3	-0.319	0.31989
[3157]	GNB2L1	--->	STAT3	0.54356	-0.59697
[3158]	LYN	--->	FCGR2B	0.11748	0.015127
[3159]	CASP3	--->	CDC2L2	0.56282	-0.63251
[3160]	SLC9A3R2	--->	PLCB3	0.33907	-0.33561
[3161]	YWHAZ	<---	TRIM21	-0.0055368	0.0059592
[3162]	MAPKAPK2	--->	CREB1	0.99947	-1.0056
[3163]	AGTR2	--->	AGT	0.55597	-0.52284
[3164]	CARD11	<---	WDR1	-0.087671	0.083119
[3165]	PRKG1	--->	MRVI1	0.99161	-1.0025
[3166]	ESR1	<---	NPPA	-0.040788	0.066582
[3167]	PPP1CA	<---	PRKCD	-0.16217	0.15344
[3168]	NRAS	--->	BCL2	0.9966	-0.99111
[3169]	NTRK1	--->	PIK3R1	0.1821	-0.30206
[3170]	$ZBTB8$	<---	ZNF408	-0.16767	-0.055896
[3171]	FHL1	--->	HIVEP3	0.23062	-0.21659
[3172]	RAC2	<--->	CDC42	0.43466	0.43466
[3173]	IGFBP6	<---	UBQLN4	-0.097913	0.13195
[3174]	UBR5	--->	CIB1	0.45013	-0.41242
[3175]	PRKACA	--->	RGS14	0.55444	-0.55865
[3176]	GNAI3	<---	AGTR2	-1.008	1.0129
[3177]	ATF3	--->	JUND	0.050278	-0.31682
[3178]	MYH7	<---	TEAD4	-0.018323	0.0060129
[3179]	CNOT1	--->	RARA	0.010054	0.00055002
[3180]	PIK3C2B	--->	LTK	0.0060475	-0.014062
[3181]	NCALD	<---	DTX2	-0.12829	0.12358
[3182]	TP53BP2	<---	MAGI1	-0.05198	0.041747
[3183]	NCOA1	--->	AR	0.11931	-0.13146
[3184]	PPP2CA	--->	PRKCD	0.070905	-0.079371
[3185]	EGFR	--->	ACTA1	0.28114	-0.28993
[3186]	DOK3	<---	RET	-0.50436	0.48844
[3187]	MAPK8	<--->	MAPK3	0.23429	0.23429
[3188]	NCR3	<---	CD59	-0.10246	0.12213
[3189]	SMAD2	<---	ESR1	-0.17818	0.22689
[3190]	BTRC	--->	NFKBIB	0.98859	-1.0006

[3191]	HTRA1	<---	FCHSD2	-0.053554	0.043501
[3192]	AKAP13	--->	RXRB	0.035086	-0.033914
[3193]	IKBKB	--->	NFKBIB	1.0006	-1.0011
[3194]	MEIS1	--->	HOXB8	0.012899	-0.0089845
[3195]	MYF6	?	ID3	-0.65635	-0.65635
[3196]	GLS2	<---	SNTA1	-0.57593	0.61152
[3197]	ARHGEF11	--->	MAP1B	0.0033578	-0.0053863
[3198]	DHX9	<---	PRMT1	-0.1729	0.16898
[3199]	AXIN1	--->	LRP5	0.024486	-0.036099
[3200]	PLEC1	--->	MAP2	0.062541	-0.046094
[3201]	PLD2	<---	ARF1	-0.50221	0.49859
[3202]	SH3GL3	<---	CD101	-0.1645	0.13993
[3203]	MEF2D	<---	NFATC2	-0.033051	0.02038
[3204]	KLF1	<---	CSNK2A2	-0.64027	0.63385
[3205]	MAPKBP1	<---	MAPK8	-0.86422	0.83716
[3206]	SOX8	<---	OLIG2	-0.01203	0.0043059
[3207]	MTOR	--->	EIF4EBP1	1.0129	-0.9999
[3208]	SMAD7	<---	ASH1L	-0.36252	0.40404
[3209]	SLC8A1	<---	YWHAE	-0.092105	0.10171
[3210]	ATF2	<---	CSNK2A1	-1.0006	1.0089
[3211]	EMID2	<---	EMID1	-0.006623	0.00089178
[3212]	HTT	<---	GAPDH	0.005969	0.0091961
[3213]	LDB3	<---	ACTN2	-0.062619	0.060394
[3214]	SYNJ2	<---	NCK1	-0.32331	0.32714
[3215]	ANK3	<---	SCN1B	-0.040108	0.027284
[3216]	UNC5C	<---	NTN1	-0.024157	0.0080805
[3217]	ADRBK1	--->	ARRB1	0.29745	-0.31779
[3218]	ARHGAP17	--->	CTTN	0.014762	0.0020228
[3219]	ITPKB	--->	CALM1	1.1467	-1.1219
[3220]	SRRM2	<---	YWHAG	-0.064614	0.088655
[3221]	HSP90AA1	--->	APOB	0.049327	-0.057262
[3222]	MXD3	?	MAX	-0.65635	-0.65635
[3223]	DLG1	--->	FZD4	0.18534	-0.22894
[3224]	TP53	--->	SMAD3	0.016573	0.007933
[3225]	HIF3A	<---	EGLN3	-0.012006	0.0022157
[3226]	SH3BP4	<---	YWHAG	-0.00061213	0.0085301
[3227]	NTSR1	--->	ARRB2	0.27159	-0.25831
[3228]	GAB2	<---	LAT	-0.46587	0.46108
[3229]	BAD	<---	PIM2	-1.4217	1.2302
[3230]	CEBPD	<---	EP300	-0.18961	0.18576
[3231]	ARFGAP1	--->	COPA	0.15762	-0.12479
[3232]	ICAM2	--->	EZR	0.029602	-0.025235
[3233]	HTR6	--->	GNAS	0.9872	-0.98627
[3234]	SMAD3	<---	FOXO3	-0.27389	0.2939
[3235]	PDGFRB	--->	PIK3R2	0.09386	-0.11269
[3236]	TP53	<---	TEC	-0.32733	0.30446
[3237]	PAK2	<---	FYN	-0.92039	1.0799
[3238]	MKI67	<---	CBX3	0.014534	0.019326
[3239]	KCNQ3	?	KCNQ2	-0.025901	-0.025901
[3240]	USP16	<---	CDK1	-0.78947	0.76367
[3241]	CABLES2	<---	CDK3	-1.1093	1.083
[3242]	ELF1	--->	REL	0.15538	-0.16476
[3243]	UBQLN1	--->	CSTF2T	0.089135	-0.057469
[3244]	USP13	--->	MYO15A	0.054944	-0.058232
[3245]	FER	<---	EGFR	0.041233	0.1145
[3246]	DNMT3B	<--->	DNMT3A	0.012904	0.012904
[3247]	ARL4D	--->	PRKCSH	0.42358	-0.39644
[3248]	CASP3	--->	PSEN1	0.58173	-0.69269
[3249]	RAB38	--->	SMAD2	0.55322	-0.47159
[3250]	RPS6KA1	--->	ESR1	0.56871	-0.73191
[3251]	PAK4	<---	CDC42	-0.9928	0.99291
[3252]	FGFR1	<---	NCAM1	-0.21241	0.010174
[3253]	CAMK2G	--->	ADCY3	0.35746	-0.37383
[3254]	THRB	--->	TP53	0.19807	-0.19275
[3255]	TGFB1	<---	ITGB6	-0.038865	0.0065129
[3256]	TF	--->	IGFBP1	0.072099	-0.038154
[3257]	EEF1G	<---	FOXG1	-0.30104	0.3328
[3258]	ACTN4	<---	CAMK2B	-0.21773	0.202
[3259]	EIF4EBP1	--->	EIF4E	1.0033	-1.0062
[3260]	SRC	--->	IL6R	0.46174	-0.43951
[3261]	PRKACA	<---	MAP3K3	-0.99622	0.99233
[3262]	RBBP4	<---	CREBBP	-0.11752	0.10953
[3263]	PRKCSH	<---	PTPRC	-0.40779	0.41064
[3264]	LDLR	<---	PF4	-0.14751	0.14259
[3265]	PAK1	--->	MYLK	0.53897	-0.35021
[3266]	IL6R	<---	IL6	-1.0005	0.9947

[3267]	C6	<---	C5	-0.10762	0.10581
[3268]	TNFRSF14	<---	LTA	-0.94009	0.80002
[3269]	CCR5	--->	STAT3	0.62118	-0.67486
[3270]	FFAR2	--->	GNAQ	1.0129	-1.008
[3271]	HDAC5	--->	GATA2	0.048777	-0.036921
[3272]	PRKCD	--->	PTPRA	0.42074	-0.41964
[3273]	UNC5CL	--->	RELA	0.11381	-0.12068
[3274]	LYN	--->	CSF3R	0.64184	-0.56719
[3275]	EPAS1	--->	ARNT	0.063735	-0.008162
[3276]	RASGRP2	--->	NRAS	1.0003	-1.007
[3277]	EPHB1	--->	NCK1	0.064938	-0.1433
[3278]	MAP3K4	<---	MAP2K1	-0.38866	0.14638
[3279]	PLCG1	<---	RHOU	-0.6651	0.59761
[3280]	SIGLEC12	<---	PTPN6	-0.097899	0.11179
[3281]	RAB4A	--->	SOD2	0.40358	-0.39039
[3282]	IL7R	<---	JAK3	-0.19329	0.19116
[3283]	YWHAB	--->	RALGPS2	0.18217	-0.17019
[3284]	TRIM23	--->	ZNF250	0.27863	-0.28527
[3285]	YAP1	--->	SLC9A3R1	0.66145	-0.65327
[3286]	COL6A2	--->	ABCD3	0.024073	-0.030894
[3287]	LPAR2	--->	SLC9A3R2	0.44636	-0.41277
[3288]	RB1	<---	TOP2A	-0.5195	0.51782
[3289]	GPS2	--->	TBL1X	0.013311	-0.01657
[3290]	FGFR2	<---	FGF18	-0.53789	0.54173
[3291]	NCK1	--->	FRS2	0.42373	-0.42803
[3292]	DAZ4	--->	QKI	0.10067	-0.097474
[3293]	GNB2L1	<---	PRKD1	-0.80211	0.76887
[3294]	CDON	<--->	BOC	0.018698	0.018698
[3295]	EP300	--->	RORA	0.17826	-0.18496
[3296]	MAPK1	--->	GJA1	0.49049	-0.48658
[3297]	PIAS2	--->	NAV2	0.038066	-0.042524
[3298]	MAPK1	--->	VAV1	0.064184	-0.26169
[3299]	LRDD	--->	CASP2	0.018651	-0.036219
[3300]	NFATC4	<---	CREBBP	-0.074436	0.064147
[3301]	ZNF521	<---	EBF1	-0.24949	0.25206
[3302]	TCL1B	--->	AKT2	0.99137	-0.99692
[3303]	SVIL	--->	ACTN4	0.034373	-0.021527
[3304]	HIST4H4	<---	HDAC8	-0.14284	0.16904
[3305]	MAP3K13	--->	IKBKB	0.10674	-0.15399
[3306]	PPFIBP1	<---	PTPRM	-0.12113	0.11923
[3307]	MYH9	<---	ASCC2	-0.0080895	-0.0019092
[3308]	IGF2	--->	IGFBP3	0.11742	-0.083423
[3309]	MAP3K2	--->	MAP2K7	0.99233	-0.99622
[3310]	KRT5	<---	KRT1	-0.012665	0.01846
[3311]	KIR2DL2	--->	CD93	0.14759	-0.14847
[3312]	TANC1	--->	GRIA1	0.18341	-0.18726
[3313]	MTA2	<---	RBBP4	-0.011108	0.035218
[3314]	MEP1B	<---	NPY	-0.27935	0.31554
[3315]	AXIN1	<---	SMAD2	-0.018982	0.01973
[3316]	GIPC1	--->	ITGA6	0.0087575	-0.016665
[3317]	RGS19	--->	GNAI1	1.0012	-1.0015
[3318]	AURKB	<---	RASA1	-0.21762	0.054854
[3319]	MAPK3	--->	ELK1	1.0428	-0.98814
[3320]	ROR2	--->	WNT5A	0.20097	-0.20779
[3321]	FYN	--->	KIT	1.0007	-1.0039
[3322]	TP53BP2	<---	YAP1	-0.56207	0.55443
[3323]	RASA1	<---	CSK	-0.57122	0.21292
[3324]	SKIL	<---	TFPI2	-0.4023	0.40635
[3325]	WDR6	<---	CDC42EP4	-0.065332	-0.017174
[3326]	CCR5	--->	CCL7	0.49595	-0.46475
[3327]	CRKL	--->	CD34	0.087032	-0.11719
[3328]	VWF	<---	HSPA5	-0.48588	0.50356
[3329]	YWHAG	--->	FOXO3	1.0083	-1.0122
[3330]	TLE2	?	TLE1	-0.1554	-0.1554
[3331]	BMPR1B	--->	CHN1	0.083183	-0.11902
[3332]	GC	--->	ACTA2	0.47181	-0.46982
[3333]	IL1R1	<---	IL1B	-1.0071	0.99815
[3334]	RHOQ	--->	PARD6B	0.11085	-0.11319
[3335]	GRIN1	--->	GRIN2B	-0.037501	-0.03762
[3336]	CALB1	--->	TRPV5	0.082402	-0.077145
[3337]	CSNK1A1	--->	ACTB	0.39781	-0.3591
[3338]	CBLB	--->	NCK1	0.32751	-0.34039
[3339]	USP7	--->	CREB3L3	0.093396	0.052737
[3340]	HDAC5	--->	SUV39H1	0.010137	-0.014278
[3341]	SLC9A3R2	--->	SLC22A4	0.37001	-0.34102
[3342]	PLD1	<---	ARF1	-0.47015	0.46122

[3343]	EHMT2	<---	HIST3H3	-0.11384	0.11647
[3344]	UBQLN4	<---	C1QTNF1	-0.083513	0.073114
[3345]	PRKACA	<---	STK11	-0.21779	0.6413
[3346]	RGS10	--->	CALM1	0.78674	-0.75366
[3347]	QKI	<---	RNF138	-0.10221	0.10661
[3348]	GSC	<---	FOXA2	-0.39346	0.42236
[3349]	PBK	--->	RAF1	0.98888	-1.0049
[3350]	NEDD4	--->	CDC25C	0.10698	-0.11718
[3351]	SLC16A1	<---	BSG	-0.33701	0.33851
[3352]	PYCARD	<---	MEFV	-0.067369	0.082651
[3353]	SPTB	--->	MAPT	0.12225	-0.11543
[3354]	GPSM2	<---	GNAO1	-0.20473	0.22114
[3355]	PDLIM5	--->	ACTB	0.33332	-0.33567
[3356]	CFLAR	--->	TRAF1	0.50472	-0.49225
[3357]	GALNT1	<---	PTPRF	-0.075229	0.068107
[3358]	CDKN3	--->	CDC25A	0.2005	-0.16931
[3359]	TGFBR1	<---	TSSK1B	0.02614	0.36359
[3360]	MPP6	--->	DNM2	0.019981	-0.030889
[3361]	YWHAG	--->	CEP170	0.043809	-0.034813
[3362]	NF2	<---	TSC1	-0.015695	0.02248
[3363]	ZFAND6	--->	PKN1	-0.05193	-0.061875
[3364]	CREBBP	--->	MYBL1	0.035202	-0.047005
[3365]	DHX9	<---	NXF1	0.00021303	0.014755
[3366]	FYN	--->	HRAS	1.0876	-0.91427
[3367]	SNRPB2	?	HTATSF1	-0.016298	-0.016298
[3368]	SYNJ1	<---	SH3GL3	-0.07064	0.084721
[3369]	ACTR1A	<---	SPTBN2	-0.024114	0.027362
[3370]	ZFYVE9	<---	TGFBR2	-0.070042	0.066121
[3371]	AKT1	--->	SMAD7	0.26467	-0.21636
[3372]	TGM2	--->	ACTB	0.53711	-0.53435
[3373]	UBR2	<---	UBE2B	-0.059235	0.074354
[3374]	CREBBP	--->	UBTF	0.17645	-0.1808
[3375]	VAV3	<---	IGF1R	-0.16158	0.14215
[3376]	HOXA9	<---	RBPMS	-0.071742	0.10141
[3377]	FES	--->	RASA1	0.39499	-0.37271
[3378]	HSPA9	--->	HSP90B1	0.28661	-0.28004
[3379]	SQSTM1	<---	NTRK3	-0.084966	0.077388
[3380]	LAMB3	--->	BMP1	0.025488	-0.021277
[3381]	MAPK8IP1	<---	MAPK9	-0.30961	0.2909
[3382]	PRKCB	--->	RRAD	1.0123	-0.99154
[3383]	FGFR2	<---	FGF8	-0.53789	0.54173
[3384]	SNTG1	--->	DTNB	0.020806	0.0159
[3385]	PTPN1	--->	STAT5A	1.0055	-1.005
[3386]	SMARCD3	--->	RXRA	0.02933	-0.16217
[3387]	CASP7	--->	BIRC2	0.10574	-0.16105
[3388]	PTPN2	--->	GHR	0.5032	-0.52008
[3389]	RAC1	--->	TRIO	0.020587	-0.034233
[3390]	RAB13	--->	SMAD4	0.55322	-0.47159
[3391]	SMURF2	--->	ARHGAP5	0.36194	-0.37365
[3392]	BCL10	--->	CASP9	-0.076001	-0.18686
[3393]	TBC1D5	--->	GABARAPL2	0.14569	-0.11414
[3394]	RBBP4	--->	SUV39H1	0.0051491	-0.0047816
[3395]	GLI3	<---	SKI	-0.14596	0.15383
[3396]	NONO	--->	FXR2	0.036464	-0.0048144
[3397]	FER	--->	CALM1	0.25483	-0.30772
[3398]	LPAR1	--->	GNAI3	1.0129	-1.008
[3399]	MTNR1B	--->	GNAI2	1.0129	-1.008
[3400]	CTBP2	--->	TGIF1	0.12019	-0.12688
[3401]	FSTL3	--->	SMAD4	0.22108	-0.19913
[3402]	CDC42BPA	--->	LIMK2	0.011701	-0.014076
[3403]	LIN7B	--->	GRIN2B	0.11324	-0.11391
[3404]	FYN	--->	GRIN2B	0.27342	-0.24765
[3405]	RAP1A	--->	FADD	0.14354	-0.23083
[3406]	KCNIP3	--->	PSEN2	0.2532	-0.23708
[3407]	GNAQ	<---	HTR2A	-0.98627	0.9872
[3408]	ANK1	<---	SLC4A3	-0.046616	0.032784
[3409]	EED	<---	HDAC3	-0.13292	0.16594
[3410]	CNKSR2	--->	TJP1	0.016798	-0.036197
[3411]	VIPR1	<---	GNAS	-0.43656	0.4334
[3412]	DAB2IP	<---	MAP3K5	0.090423	0.16804
[3413]	SMAD3	<---	CUL1	-0.99833	1.0155
[3414]	GAPDH	--->	PCDHA4	0.034062	-0.018885
[3415]	CCNA1	<---	GNB2L1	-0.54356	0.55627
[3416]	GNA12	--->	RASA2	1.0015	-1.0021
[3417]	MYD88	<---	IL1R1	-0.0036516	0.17593
[3418]	HSP90AA1	--->	MMP2	0.056603	-0.066155

[3419]	CCL19	<---	CCRL1	-0.46475	0.49595
[3420]	BGN	<---	COL4A2	-0.01099	0.0030847
[3421]	PAX9	<---	MSX1	-0.0073189	0.041579
[3422]	PICK1	<---	MLLT4	-0.22525	-0.058146
[3423]	NECAB3	<---	APBA2	-0.01778	0.022892
[3424]	ARHGAP17	<---	SH3GLB1	-0.016169	0.0011264
[3425]	SRC	--->	PAK2	0.98977	-0.89147
[3426]	LYN	<---	CASP9	-0.99432	1.0005
[3427]	FN1	<---	COL2A1	-0.041492	0.033176
[3428]	UBE2E1	--->	UBE2G1	0.29354	0.066829
[3429]	RASSF1	--->	MAP1S	0.078219	-0.060863
[3430]	JDP2	<---	CREBBP	-0.12724	0.11829
[3431]	CREBBP	--->	MYB	0.018722	-0.03137
[3432]	PTPN2	--->	STAT3	0.99961	-0.99956
[3433]	PPARG	<---	KIF1A	-0.063843	0.055415
[3434]	PIN1	--->	APP	0.37109	-0.38109
[3435]	SUFU	--->	ZNF219	0.0090887	-0.0091921
[3436]	PPARGC1A	--->	NCL	-0.00071687	-0.012459
[3437]	BCR	<---	RASA1	-0.012303	0.019897
[3438]	CBLC	--->	SPRY2	0.31167	-0.31357
[3439]	GHRL	--->	GHRHR	0.31094	-0.30506
[3440]	FLNA	--->	GRIK1	0.096074	-0.079231
[3441]	PTPN6	--->	EPOR	0.60329	-0.52352
[3442]	ADAM17	--->	TIMP3	0.056211	-0.051705
[3443]	CXCR2	--->	CXCL6	0.49595	-0.46475
[3444]	ABTB1	--->	SMAD9	0.093228	-0.11098
[3445]	MYO16	<---	NRXN1	-0.33584	0.35046
[3446]	TRIM27	<--->	TRIM11	0.0045386	0.0045386
[3447]	KLF4	<---	SP1	-0.064923	0.089665
[3448]	ASCL3	?	MYOD1	-0.65635	-0.65635
[3449]	CHEK1	--->	CUL4A	0.30021	-0.32466
[3450]	LHB	<---	GNAI2	-0.09586	0.11125
[3451]	TGFB1I1	<---	PTK2	-0.60363	0.60368
[3452]	GADD45B	<---	CDK1	-0.76568	0.74161
[3453]	EP300	--->	PPARD	0.17826	-0.18496
[3454]	PRKCA	--->	RARA	0.6447	-0.65411
[3455]	NR0B1	--->	ESRRA	-0.0089663	-0.044425
[3456]	ABI2	<---	TRIM32	-0.14847	0.13961
[3457]	NCOA1	--->	JUN	0.0094504	-0.027639
[3458]	ILK	--->	ITGB1	0.08666	-0.10051
[3459]	NF2	<--->	EZR	0.004857	0.004857
[3460]	L1CAM	--->	RANBP9	0.12745	-0.13615
[3461]	SMARCD1	--->	CALCOCO2	0.031085	-0.014888
[3462]	$ZBTB8$	--->	ZNF250	-0.012541	-0.094298
[3463]	RALGDS	<---	RAP2B	-1.0084	0.98547
[3464]	DTX3L	<---	UBE2D1	-0.02412	0.017553
[3465]	MTNR1A	<--->	MTNR1B	0.49511	0.49511
[3466]	FFAR1	--->	GNAQ	1.0129	-1.008
[3467]	EGLN3	--->	WDR83	0.025788	-0.01149
[3468]	ARHGEF2	--->	YWHAG	0.15751	-0.15298
[3469]	TLE1	<---	SERPINB9	-0.017617	0.052635
[3470]	PDX1	--->	TCF3	0.34775	-0.35072
[3471]	NOTCH3	<---	CHUK	-0.065738	0.051666
[3472]	RELA	<---	SOCS1	-0.075277	0.074761
[3473]	CYP11A1	--->	SMAD9	0.2957	-0.31436
[3474]	KCNG2	?	KCNB1	-0.025901	-0.025901
[3475]	BTK	<---	JAK1	-1.0003	0.99303
[3476]	RERE	--->	TRIP6	0.056974	-0.035531
[3477]	MATK	<--->	LYN	0.37153	0.37153
[3478]	CD4	<---	LCK	-0.67126	0.68982
[3479]	GEM	--->	GMIP	0.70845	-0.67799
[3480]	MLST8	<---	MTOR	-0.1642	0.16199
[3481]	FGFR4	--->	STAT1	-0.026735	-0.027648
[3482]	RGS9	--->	GNAT1	0.60077	-0.60607
[3483]	FZR1	<---	CDC14A	-0.80601	0.8437
[3484]	MAP1B	--->	ACTB	0.50313	-0.50002
[3485]	CASP7	--->	CASP2	0.17851	0.10027
[3486]	MAPK1	--->	CEBPB	0.97322	-0.93471
[3487]	SETDB1	<---	BTBD2	-0.079069	0.075766
[3488]	ABCD2	?	ABCD1	-0.062455	-0.062455
[3489]	LEPR	--->	STAT3	0.14842	-0.17747
[3490]	PPIA	<---	BSG	-0.35632	0.35889
[3491]	CDC20	--->	CCNA2	0.55627	-0.54356
[3492]	MAPK8	--->	CDKN1A	0.51838	-0.55156
[3493]	CCNE1	<---	CDC25A	-0.3246	0.33489
[3494]	YWHAQ	<---	KRT1	-0.033015	0.018236

[3495]	SP1	--->	NR2F1	0.10251	-0.11174
[3496]	PITX2	<---	PROP1	-0.19656	-0.024292
[3497]	DLG1	--->	FZD7	0.11867	-0.14014
[3498]	GAPDH	--->	BTBD2	0.017895	-0.02138
[3499]	TNFRSF10B	--->	FADD	0.89012	-0.86277
[3500]	SMARCD3	--->	JUN	0.28391	-0.31689
[3501]	PRKAG1	<---	PRKAA1	-0.22052	-0.13077
[3502]	EP300	--->	RAD23A	0.18025	-0.18457
[3503]	THBS1	<---	CTSG	-0.30323	0.39805
[3504]	OCLN	<---	TGFBR1	-0.38134	0.34747
[3505]	ESR1	--->	POU4F2	0.0076091	0.0018176
[3506]	MAGI1	--->	IL6R	0.03076	-0.041661
[3507]	AKT1	<---	RAB3D	0.060359	0.09142
[3508]	INPPL1	--->	SHC1	0.20287	-0.22762
[3509]	LPHN1	<---	DLG4	-0.10924	0.1027
[3510]	FRS2	<---	RND1	-0.47916	0.51129
[3511]	PRKCA	--->	OPRD1	0.87512	-0.87377
[3512]	DDX24	--->	GIT1	0.10674	-0.11476
[3513]	LRP12	<---	ACTN2	-0.12861	0.13265
[3514]	CDKN1B	<---	TRAF2	-0.061508	0.054856
[3515]	CAMK2A	--->	SYNGAP1	0.21355	-0.21755
[3516]	RANBP9	<---	RAN	-0.21869	0.23174
[3517]	ACVR1B	--->	SMAD4	1.0115	-1.0087
[3518]	HIST3H3	<---	HDAC8	-0.14284	0.16904
[3519]	GIT1	<---	BARD1	-0.1454	-0.048542
[3520]	ADRBK2	--->	BDKRB2	0.20207	-0.22265
[3521]	PTPRC	--->	INSR	0.032808	-0.062922
[3522]	FADD	<---	MYD88	-0.41719	0.44258
[3523]	PDPK1	<---	HSP90AA1	-0.0026425	0.0099435
[3524]	DLG1	--->	WNT3A	0.22514	-0.23036
[3525]	TRIM37	<---	TRAF2	-0.0093461	0.14237
[3526]	ARHGAP15	--->	CD93	0.11505	-0.12163
[3527]	RBPMS	<---	C9orf86	-0.06492	0.083233
[3528]	SNTB2	<---	UTRN	-0.052248	0.048407
[3529]	PCNT	--->	CALM2	0.34679	-0.30915
[3530]	YWHAE	<---	SRC	-0.89808	1.0093
[3531]	CXCL5	<---	CXCR2	-0.46475	0.49595
[3532]	HRAS	--->	RAF1	1.0008	-0.9994
[3533]	DUSP16	--->	MAPK8IP1	0.31547	-0.31091
[3534]	OPRD1	--->	ADRB2	0.31807	0.24405
[3535]	SIGLEC7	<---	PTPN11	-0.12796	0.14945
[3536]	CHEK1	--->	CDC25B	0.69298	-0.67632
[3537]	PRKACA	--->	CREM	0.96654	-0.95401
[3538]	MAP3K14	<---	TRAF2	-0.9866	1.0023
[3539]	NCK1	<---	CBL	-0.34039	0.32751
[3540]	CTNNB1	<---	SALL1	-0.1899	0.1814
[3541]	ACAN	<---	FBLN2	-0.015047	0.0061745
[3542]	CUL2	--->	TCEB1	0.087099	-0.076122
[3543]	TCF4	<---	NEUROD1	-0.37599	-0.054725
[3544]	PPP1R13B	<---	TP63	-0.05245	0.050275
[3545]	FLRT3	--->	FGFR1	0.2629	-0.26198
[3546]	GREM2	--->	BMP4	0.56685	-0.51806
[3547]	NRAP	--->	TLN1	0.19759	-0.20549
[3548]	BCL10	<---	CHUK	-0.61976	0.41027
[3549]	NR0B2	<---	HNF4A	-0.023084	0.1947
[3550]	GC	--->	ACTC1	0.47181	-0.46982
[3551]	CAV1	<---	GRK1	-0.73372	0.70803
[3552]	CPNE2	<---	UBE2O	-0.12637	0.16628
[3553]	PRKCB	--->	CASR	0.55548	-0.55976
[3554]	EIF2AK2	<---	HSPA1A	-0.99901	1.0063
[3555]	SMARCA4	<---	RELB	-0.05639	0.04446
[3556]	OTX2	--->	MITF	0.34514	-0.33302
[3557]	BACE1	--->	NCSTN	0.075966	-0.072907
[3558]	MAPK1	<---	PAK1	-1.3014	1.4008
[3559]	SOS1	--->	ERBB2	-0.0022102	-0.0094276
[3560]	FBXO18	<---	CUL1	-0.045528	0.057263
[3561]	HOXA1	<---	LPXN	-0.10746	0.14086
[3562]	JUP	<---	ZFYVE9	-0.03994	0.027506
[3563]	PALLD	<---	ACTN2	-0.081334	0.083986
[3564]	TRAF2	<---	TRADD	-0.10256	0.074549
[3565]	TGFB1I1	<---	CRKL	-0.15227	0.125
[3566]	PTPN11	--->	MET	0.54634	-0.3029
[3567]	ADORA2A	--->	ACTN2	0.0066716	-0.01063
[3568]	RXRG	<---	RARA	-0.99549	1.003
[3569]	F2	--->	PROS1	0.005203	-0.0080779
[3570]	BMPR1B	--->	DCAF6	0.13865	-0.1649

[3571]	CD79A	<---	PTPN6	-1.0144	1.0068
[3572]	KRAS	--->	BCL2	0.9966	-0.99111
[3573]	MAP4K1	--->	MAP3K11	1.0012	-1.003
[3574]	DLG3	--->	SCN4A	0.047736	-0.049447
[3575]	CAV1	--->	TNFRSF1B	0.00023291	-0.12489
[3576]	SKP1	--->	DDX19B	0.072324	-0.071284
[3577]	SIGLEC7	--->	GRB2	0.02153	-0.040448
[3578]	PRKCD	--->	MAPT	0.50001	-0.49874
[3579]	RIT1	--->	SMAD2	0.55322	-0.47159
[3580]	GZMA	--->	APEX1	0.017674	0.016057
[3581]	CD48	<---	IL18RAP	0.15493	0.20214
[3582]	CD209	--->	ITGAM	0.043438	-0.055942
[3583]	PRLR	<---	ZAP70	-0.054931	0.043423
[3584]	HOXB6	<---	EP300	-0.18943	0.18505
[3585]	BTRC	--->	NFKB2	0.093233	-0.10694
[3586]	SCN5A	<---	SNTB2	-0.013537	0.050181
[3587]	AGTR1	<---	ADRBK1	-0.22265	0.20207
[3588]	TNFRSF10A	<--->	TNFRSF10B	0.52751	0.52751
[3589]	ACVRL1	<---	XIAP	-0.049954	0.025777
[3590]	PGR	--->	BATF3	0.1653	-0.12194
[3591]	RNF11	--->	RABGEF1	0.0030565	-0.013718
[3592]	ARF6	--->	EZR	0.1968	-0.19524
[3593]	QKI	<---	RBM14	-0.097474	0.10067
[3594]	FYN	--->	CAV1	1.0636	-0.97511
[3595]	PTK2	--->	IRS1	0.078693	-0.1611
[3596]	GFRA1	--->	NRTN	0.39482	-0.35487
[3597]	FGFR1	<---	TENC1	-0.042904	0.010001
[3598]	CASP3	--->	KCNIP3	0.20167	-0.27393
[3599]	PIK3R1	<---	IRS1	-0.1531	0.097154
[3600]	ENOX1	<---	LNX1	-0.0053675	0.021194
[3601]	CARD17	?	CARD16	-0.10452	-0.10452
[3602]	TRAF2	--->	LNX1	0.057686	-0.022434
[3603]	SKP1	--->	FBXO6	0.055251	-0.044878
[3604]	EPAS1	--->	ARNTL	0.063735	-0.008162
[3605]	PXN	<---	VCL	-0.52828	0.52939
[3606]	NCF1	<---	PAK1	-0.068931	0.15322
[3607]	CD40	<---	HSPA4	-0.68286	0.67874
[3608]	JAK3	--->	CXCR4	0.34689	-0.32974
[3609]	IQGAP1	--->	CDH1	0.089227	-0.091577
[3610]	NEUROD1	<---	CALM2	-0.22666	0.2676
[3611]	MAP1A	--->	MAP1B	-0.033133	-0.040116
[3612]	SFRS4	<---	SMURF1	-0.71228	0.70809
[3613]	BIRC3	--->	TRAF1	0.19555	-0.19908
[3614]	MEGF10	<---	GRB10	-0.008274	-0.0068533
[3615]	SLC9A3R2	<---	PDPK1	-0.24388	0.39108
[3616]	PAK6	<---	CDC42	-1.0053	0.99704
[3617]	DAZ1	?	BOLL	-0.016298	-0.016298
[3618]	PARD3	<---	PRKCG	-0.36857	0.3615
[3619]	GPR37	--->	PARK2	0.2465	-0.23599
[3620]	CASP7	--->	CDC42	0.36319	-0.47633
[3621]	DVL3	<---	AXIN1	-0.061523	-0.036269
[3622]	SYNE2	--->	ACTC1	0.29277	-0.29627
[3623]	TRIP4	<---	GTF2A1	-0.061204	0.080514
[3624]	MYB	<---	FBXW2	-0.015531	0.0053621
[3625]	NFATC2	<---	CREBBP	-0.016462	0.0034296
[3626]	NUCB1	<---	GNAS	-0.49311	0.51194
[3627]	MMP9	<---	CXCL1	-0.16713	0.18806
[3628]	FBXW7	--->	SHPRH	0.074357	-0.082552
[3629]	BNIP3L	--->	BCL2	0.68788	-0.67957
[3630]	TSSK6	<---	HSPA1A	-0.99901	1.0063
[3631]	PRKAA1	--->	MTOR	0.99912	-0.99491
[3632]	BCR	--->	PLCG1	-0.0036243	-0.20525
[3633]	GABBR1	<---	YWHAZ	-0.09181	0.11629
[3634]	LIMK1	--->	CDKN1C	0.23693	-0.2508
[3635]	EPHB2	--->	PTK2	0.22346	-0.086913
[3636]	SMAD3	<---	ACTB	-0.0089894	0.049237
[3637]	VASP	<---	PFN2	-0.023349	0.039151
[3638]	CD244	--->	LAT	0.31893	-0.28312
[3639]	PC	<---	MDH2	-0.023782	0.01655
[3640]	GMIP	<---	RHOA	-0.67799	0.70845
[3641]	IFNAR1	<---	FBXW11	-0.49071	0.47806
[3642]	AKAP13	<---	RHOA	-0.098286	0.10183
[3643]	PTPN13	--->	PRKACA	0.14484	-0.15597
[3644]	TRIM27	--->	HDAC1	0.0089585	-0.01176
[3645]	RAC1	--->	ACTB	0.99207	-0.99507
[3646]	PTGIR	--->	GNAQ	1.0129	-1.008

[3647]	ZRANB2	--->	YY1	0.11228	-0.10555
[3648]	STAT1	--->	IFNGR1	0.13232	-0.21392
[3649]	FOS	<---	NFKB1	-0.18564	0.17421
[3650]	SVIL	--->	SPTAN1	0.05653	-0.058822
[3651]	CAPN3	--->	DYSF	0.25044	-0.24884
[3652]	PPBP	--->	STRN4	0.11072	-0.077632
[3653]	ITGA2B	<---	ITGB3	-0.012215	-0.00095394
[3654]	CXCL13	<---	CCR10	-0.46475	0.49595
[3655]	USP7	--->	TRAF1	0.072661	-0.014241
[3656]	ACACA	<---	ATXN1	-0.097481	0.084795
[3657]	LAMB3	<---	COL7A1	-0.011192	-0.0013797
[3658]	NUCB1	<---	GNAZ	-0.49311	0.51194
[3659]	ABLIM1	--->	KCNJ12	0.041011	-0.024531
[3660]	RNF128	<---	CD40LG	-0.16733	0.17786
[3661]	WNT1	?	WNT3A	-0.58807	-0.58807
[3662]	CPS1	<---	RAF1	-0.026177	0.0052031
[3663]	TBP	<---	MDM2	-0.014692	0.0011841
[3664]	RHOD	--->	DIAPH2	0.047484	-0.055375
[3665]	TESC	--->	SLC9A1	0.33362	-0.3273
[3666]	ZBTB16	<---	HDAC7	-0.071268	0.064989
[3667]	INSR	<---	PTPN6	-0.13143	0.15547
[3668]	PPP1CC	--->	TP53	0.0988	-0.11003
[3669]	MAGI2	<---	CTNNB1	-0.26247	0.25284
[3670]	PRKACA	--->	CACNA1C	0.99121	-1.0053
[3671]	GABPA	<---	SP3	-0.01846	0.027464
[3672]	LCK	--->	PRKCQ	0.073791	-0.063362
[3673]	HCK	--->	AGK	1.0649	-0.90413
[3674]	ATN1	<---	NCK2	-0.025507	0.014004
[3675]	TRRAP	--->	E2F1	-0.0031438	-0.0084023
[3676]	SH3GLB1	<--->	BIN1	0.0094254	0.0094254
[3677]	MAPK1	--->	MYB	0.092606	-0.10814
[3678]	SOCS3	<--->	SOCS2	0.07748	0.07748
[3679]	DCN	<---	COL4A1	-0.024545	0.014583
[3680]	PRKCD	--->	PLSCR1	0.78169	-0.77232
[3681]	DLG4	--->	GLS2	0.26144	-0.26501
[3682]	IFNAR1	--->	STAT3	0.21302	-0.25763
[3683]	MAPK8IP2	<---	MAPK9	-0.30961	0.2909
[3684]	FOXH1	--->	SMAD2	0.52503	-0.44328
[3685]	MMP3	<---	HAPLN1	-0.093464	0.097177
[3686]	CALCOCO2	--->	ZNF426	0.19639	-0.20055
[3687]	MAP3K7IP1	--->	XIAP	0.23033	-0.21901
[3688]	TFG	--->	PLSCR1	0.047368	-0.039186
[3689]	TRPM7	--->	HIST3H3	0.014118	-0.013401
[3690]	PCSK5	<---	REN	-0.13375	0.12947
[3691]	MITF	<---	MAPK14	-0.98857	0.9908
[3692]	TRAF2	--->	APPL1	0.16011	-0.15406
[3693]	FBLIM1	--->	FLNB	0.066481	-0.030995
[3694]	KHDRBS3	<---	SFRS9	-0.042052	0.055977
[3695]	FBXO18	<---	SKP1	-0.044878	0.055251
[3696]	GIT2	<---	PXN	-0.066907	0.057095
[3697]	RELA	<---	BTK	-0.18559	0.16682
[3698]	BOC	--->	CDH15	0.16197	-0.17057
[3699]	SMAD3	<---	CAMK2G	-1.0107	1.0038
[3700]	RXRG	<---	THRA	-0.99549	1.003
[3701]	CHAF1B	--->	HIST1H3E	0.10803	-0.057829
[3702]	ADRBK1	--->	FPR1	0.093699	-0.11125
[3703]	ELK3	--->	ID2	0.12676	-0.084944
[3704]	PRKCA	--->	NOS1	0.1484	-0.16067
[3705]	TRIM27	--->	GFI1B	0.055478	-0.065703
[3706]	POGZ	<---	SP1	-0.1593	0.14922
[3707]	PPARA	--->	RXRG	1.003	-0.99549
[3708]	PAPPA	--->	PLG	0.020602	-0.0054458
[3709]	HPCAL1	<---	ATXN1	-0.042644	0.040063
[3710]	ZEB2	--->	SMAD9	-0.0031961	-0.015716
[3711]	TLR2	--->	CD14	0.14143	0.071606
[3712]	SKIL	<---	UBE2I	-0.74511	0.75613
[3713]	JAK1	--->	STAT3	0.99633	-1.0023
[3714]	SPTBN2	<---	CELSR3	-0.0076483	-0.003754
[3715]	INPP5D	<---	FGR	-0.52338	0.86692
[3716]	GRIN1	--->	GRIN3A	-0.033971	-0.11785
[3717]	PSEN1	--->	NOTCH2	0.99249	-0.99286
[3718]	SRC	--->	PRKCE	0.06379	-0.051826
[3719]	ARHGAP8	--->	CTTN	0.020364	-0.0035364
[3720]	NFKB1	--->	LYL1	0.78572	-0.77767
[3721]	SUFU	--->	SETDB1	0.042081	-0.036253
[3722]	MX1	--->	TRPC1	0.041026	-0.053855

[3723]	SOX8	<---	PAX3	-0.029062	0.025489
[3724]	FHL3	<---	PHC2	-0.17497	0.21449
[3725]	ATF5	<---	CDC34	-0.2686	0.28344
[3726]	NGFR	--->	NFKBIB	0.57142	-0.38774
[3727]	IL1RAP	<---	IL1A	-0.18407	0.18683
[3728]	PTPRN2	--->	UBQLN4	1.2138	-1.2073
[3729]	TNFSF4	--->	TNFRSF4	0.89674	-0.85556
[3730]	SYNPO	<---	MAGI1	-0.021964	0.017006
[3731]	CCL8	<---	CCR3	-0.46475	0.49595
[3732]	TBC1D10A	--->	EZR	0.035016	-0.030605
[3733]	BAX	--->	BCL2L1	-0.23385	-0.7984
[3734]	PPP2CA	--->	AXIN1	0.998	-0.99843
[3735]	SMURF1	--->	SQSTM1	0.42147	-0.43091
[3736]	WEE1	--->	CRK	0.206	-0.30256
[3737]	SNX9	--->	SOS1	0.015215	-0.030977
[3738]	MEOX2	--->	PAX1	0.041579	-0.0073189
[3739]	SCN4A	<---	SNTA1	-0.013537	0.050181
[3740]	MAP3K4	<---	CDC42	-0.54804	0.54389
[3741]	PDLIM4	--->	RBPMS	0.03533	-0.019231
[3742]	CDKN1A	--->	CCND2	0.98767	-0.99961
[3743]	PTGIR	--->	GNA11	1.0129	-1.008
[3744]	CXCL9	<---	CXCR3	-0.46475	0.49595
[3745]	ITPR2	<---	BANK1	-0.12807	0.11559
[3746]	ACVR1	--->	WDR61	0.082233	-0.10071
[3747]	NCAN	<---	HMGB1	-0.028524	0.016443
[3748]	AHR	--->	NCOA2	-0.022773	-0.035755
[3749]	PPP3CB	--->	SLC8A1	0.10266	-0.11189
[3750]	PDE5A	<---	CASP3	-0.48639	0.4126
[3751]	F10	<---	GAD2	-0.015975	0.012987
[3752]	HAVCR1	<---	UBQLN4	-0.10922	0.13389
[3753]	CDKN1C	--->	MYOD1	0.57932	-0.54324
[3754]	TSC1	--->	MSN	0.011265	-0.017586
[3755]	PTPN11	--->	SIRPA	0.97001	-0.82769
[3756]	SERPINA5	<---	FGB	-0.083807	0.11995
[3757]	RRAGC	<--->	RRAGA	0.010143	0.010143
[3758]	MLLT6	<---	UBQLN4	-0.050606	0.084635
[3759]	CDK1	<---	RAB5B	-1.0074	1.0132
[3760]	PELI1	<---	IRAK1	-0.98981	1.0139
[3761]	PPP1R9A	--->	ACTG1	0.38986	-0.38133
[3762]	APBA1	<---	CASK	-0.00599	0.0050958
[3763]	TP53	<---	HDAC3	-0.35801	0.36943
[3764]	SRC	--->	DOK1	1.2026	-1.146
[3765]	DAB1	<---	CRK	-0.13738	0.12903
[3766]	TNFRSF13C	<---	TNFSF13B	-0.9993	1.008
[3767]	UBQLN4	--->	C1orf94	0.14474	-0.11005
[3768]	SP1	--->	MSX1	0.17126	-0.18177
[3769]	THRA	--->	MEF2A	0.027114	-0.030148
[3770]	HOXD4	<---	CREBBP	-0.18943	0.18505
[3771]	STRN3	--->	CALM1	0.082506	-0.077801
[3772]	TFEB	?	TFE3	-0.083565	-0.083565
[3773]	HDAC1	--->	RB1	0.29317	-0.29943
[3774]	BLK	--->	CD79A	1.0799	-1.0399
[3775]	UBE2D4	--->	DTX2	0.028583	-0.037834
[3776]	PTK2	<---	PTPN11	-0.99764	0.99625
[3777]	ABL2	<---	CRK	-0.99908	0.99616
[3778]	SH3BP1	<---	RHOA	-0.41737	0.43038
[3779]	RGS3	--->	ESR1	0.13202	-0.13034
[3780]	CXCR2	--->	GNA14	1.0129	-1.008
[3781]	RIT2	<---	SOS1	-0.99973	0.99534
[3782]	FLNB	?	FLNA	-0.026589	-0.026589
[3783]	CTBP1	<---	MECOM	-0.16004	0.15176
[3784]	LYN	--->	PLCG2	0.59106	-0.8605
[3785]	ELF5	<---	SIRT6	-0.24048	0.2611
[3786]	PTPN1	--->	LTK	0.57473	-0.59775
[3787]	FBXO5	--->	CDC20	0.084765	-0.070176
[3788]	SLC9A3R2	<---	P2RY1	-0.40599	0.42206
[3789]	HTR2A	--->	GNAI1	0.9872	-0.98627
[3790]	TFEC	--->	MITF	-0.069637	-0.16359
[3791]	SCRIB	--->	LPP	0.076735	-0.06172
[3792]	RFX1	<---	RFX2	-0.0094131	-0.0041411
[3793]	STARD13	--->	TAX1BP1	0.11484	-0.096873
[3794]	CTGF	<---	AP3D1	-0.47098	0.46155
[3795]	INS	--->	IGF1R	0.65928	-0.66772
[3796]	EP300	--->	CCND1	0.12677	-0.13493
[3797]	NR4A1	--->	BCL2L10	0.52125	-0.50885
[3798]	MDM2	--->	UBQLN4	0.11491	-0.11807

[3799]	HIST1H3C	?	CENPA	-0.23226	-0.23226
[3800]	NFIX	--->	SKI	0.17204	-0.16571
[3801]	MAP2K5	--->	PRKCI	0.069627	0.025152
[3802]	DNMT1	<---	TRIM27	-0.0027737	-0.00027066
[3803]	ASAP1	<---	SH3KBP1	-0.031988	0.036302
[3804]	PTPRZ1	--->	GRLF1	0.21289	-0.22013
[3805]	APP	<---	NUMBL	-0.14057	0.12812
[3806]	CTTNBP2	<---	CTTN	-0.091449	0.11077
[3807]	RPS6KA1	--->	FOS	0.29655	-0.38418
[3808]	HOXB6	<---	PLSCR1	-0.080977	0.10682
[3809]	TGFB2	--->	TGFB1	0.99583	-0.98835
[3810]	GDF9	<---	TRIB3	-0.66907	0.65231
[3811]	LIN7B	--->	KCNJ12	0.044422	-0.0070376
[3812]	CDKN1B	--->	STMN1	0.025557	-0.017804
[3813]	ADCY10	<---	SSTR5	-0.30707	0.31994
[3814]	NCOR1	--->	MECP2	0.073474	-0.079407
[3815]	NCOA2	<---	SMARCE1	-0.0651	0.054906
[3816]	UBE2I	<---	PRPF40A	-0.097401	0.12918
[3817]	BTK	<---	PRKCQ	-0.13507	0.1413
[3818]	ACVR1	--->	IKBKB	0.19684	-0.21102
[3819]	MAP1LC3A	<---	MAP1A	-0.05011	0.064082
[3820]	KCNV2	?	KCNB1	-0.025901	-0.025901
[3821]	AXL	--->	PLCG1	0.41235	-0.47161
[3822]	FGR	--->	ARRB1	0.64227	-0.55024
[3823]	ZXDC	<---	NR1H2	-0.049739	0.052753
[3824]	PTPRF	--->	CTNNB1	0.071698	-0.083619
[3825]	SP1	--->	CCND1	0.22877	-0.24076
[3826]	FBN1	--->	CALR	0.020226	-0.035097
[3827]	GNB2L1	--->	INSR	0.12996	-0.13842
[3828]	CRIP2	<---	SRF	-0.037159	0.05921
[3829]	YWHAZ	<---	CDK16	-0.66368	0.62776
[3830]	RNF138	--->	PCBP4	0.052601	-0.017679
[3831]	CSNK2A1	--->	NFKBIA	1.454	-1.5082
[3832]	YAP1	--->	SMAD7	1.0151	-0.99492
[3833]	CD19	<---	GRB2	-0.47363	0.43776
[3834]	GIT1	<---	SRC	-0.99965	0.99834
[3835]	PRKCI	<---	CDC42	-1.0135	0.99149
[3836]	PRKAG2	<--->	PRKAG1	0.82198	0.82198
[3837]	SNTA1	--->	GLS	0.61152	-0.57593
[3838]	SMAD7	--->	ZBTB11	0.071259	-0.07911
[3839]	AKT2	--->	XIAP	0.30869	-0.23198
[3840]	DOK1	<---	NCK1	-0.42803	0.42373
[3841]	CBLB	--->	CRKL	0.35632	-0.37379
[3842]	ERBB3	--->	GRB2	1.1215	-1.1271
[3843]	CSNK2A1	--->	HDAC2	0.4	-0.42066
[3844]	PTPN6	<---	SRC	0.083854	0.2006
[3845]	OSR1	<---	TGFBR1	-0.31217	0.31662
[3846]	NBEA	--->	STRN4	-0.061007	-0.062181
[3847]	INADL	--->	PARD3	0.0035079	-0.0028861
[3848]	CBLB	--->	NEDD4	0.011259	-0.024467
[3849]	TSC1	--->	CCNB1	0.61948	-0.60211
[3850]	RGS10	--->	GNAI3	1.0012	-1.0015
[3851]	RAB11FIP2	<---	RAB11A	-0.15731	0.20092
[3852]	SKAP1	<---	FYN	-0.99957	1.0067
[3853]	NOTCH1	<---	PSEN2	-1.0047	0.99454
[3854]	APAF1	<---	HSPA1A	-0.62942	0.64874
[3855]	SERPINB13	<---	ELANE	-0.082185	0.11833
[3856]	MAPK8	--->	MAP1B	0.77181	-0.77493
[3857]	FGB	--->	CALR	0.087176	-0.067164
[3858]	YY1	--->	MYC	0.068938	-0.072889
[3859]	DLG4	--->	GRIK5	0.060317	-0.068607
[3860]	MSX1	?	MSX2	-0.11417	-0.11417
[3861]	MAPK8IP1	--->	LRP1	0.08872	-0.099755
[3862]	PRKD3	--->	KPNA2	0.74061	-0.77407
[3863]	PRKCA	--->	PAM	0.90745	-0.90584
[3864]	PODXL2	--->	SELL	0.012256	-0.0093683
[3865]	CBX5	<---	TRIM24	-0.030467	0.064029
[3866]	AGTR1	<---	ADRBK2	-0.22265	0.20207
[3867]	SLC2A1	<---	COPS6	-0.57136	0.59004
[3868]	SP1	--->	AR	0.21513	-0.22644
[3869]	LMO3	--->	MBIP	0.026132	0.0091776
[3870]	ESR1	--->	RXRA	1.003	-0.99549
[3871]	NCOA1	<---	TBP	-0.0084554	-0.0052655
[3872]	KCNJ12	<---	DLG1	-0.04695	0.04551
[3873]	HDAC4	--->	ANKRA2	0.15653	-0.13768
[3874]	APBB3	--->	APP	0.22101	-0.23254

[3875]	RRAS2	--->	RAF1	1.0008	-0.9994
[3876]	OCRL	<---	RAB6A	-0.25397	0.25628
[3877]	JDP2	--->	JUNB	0.050278	-0.31682
[3878]	RASSF1	<---	MDM2	-0.072745	0.065768
[3879]	ACVR1	<---	RAP2A	-0.32935	0.31819
[3880]	XAB2	<---	ERCC6	-0.004874	0.020491
[3881]	CLSTN1	<---	APBA2	-0.033307	0.024385
[3882]	ROCK1	<---	LIMK1	-0.053928	0.039587
[3883]	KDR	--->	CSF2RB	0.45158	-0.42896
[3884]	EP300	--->	RELA	0.038481	-0.050014
[3885]	PAK2	<---	YES1	-0.92039	1.0799
[3886]	TNFRSF1A	<---	JAK2	-0.10841	0.17701
[3887]	STAT2	<---	HDAC1	-0.32685	0.30366
[3888]	SRGAP3	--->	SMARCC2	0.081416	-0.078393
[3889]	SERPINF2	<---	FGA	-0.083807	0.11995
[3890]	SPOCK3	--->	MMP3	0.25303	-0.23217
[3891]	PUF60	--->	PPP1R16A	0.58758	-0.557
[3892]	CD226	--->	ITGB2	0.14099	-0.15288
[3893]	DACH1	<---	NCOR1	-0.0549	0.04576
[3894]	JAK1	--->	SOCS3	1.0118	-1.0098
[3895]	CD93	<---	C1QA	-0.043446	0.032253
[3896]	ESR1	--->	SREBF1	0.18403	-0.18581
[3897]	YWHAG	<--->	YWHAB	0.069023	0.069023
[3898]	PPP1CA	--->	BAX	0.32856	-0.33945
[3899]	SFPQ	?	SNRPA	-0.016298	-0.016298
[3900]	PTK2B	<---	STAP1	-0.23452	-0.098115
[3901]	PAK4	<---	LIMK1	-0.13019	0.17988
[3902]	STAT4	<---	JUN	-0.22034	0.18355
[3903]	YWHAB	<---	WEE1	-0.66368	0.62776
[3904]	HSPA8	--->	BAG3	0.39751	-0.3826
[3905]	MAPK8IP3	<---	MAP2K4	-0.16592	0.14982
[3906]	ABL1	--->	CD19	0.037898	-0.14493
[3907]	EVL	<---	ABI1	-0.062083	0.051702
[3908]	ACTG1	<---	CFL1	-0.30587	0.30201
[3909]	UBQLN1	<---	RASSF5	0.0022381	0.014784
[3910]	REM1	--->	YWHAZ	0.32694	-0.30645
[3911]	PIWIL2	--->	DDX4	0.042243	-0.02712
[3912]	NEDD9	<---	ITCH	-0.14876	0.13658
[3913]	SHC1	<---	DDR1	-0.517	0.23668
[3914]	VPS8	--->	SMAD9	0.2994	-0.31807
[3915]	HEYL	<---	HAND2	-0.43036	-0.38951
[3916]	NR2F2	<---	EP300	-0.18496	0.17826
[3917]	MAP3K14	--->	HSP90AB1	0.028434	-0.045733
[3918]	GRM7	--->	CALM1	0.31782	-0.27991
[3919]	MAP3K12	--->	MAP3K11	0.16257	0.10311
[3920]	PLSCR4	?	PLSCR1	-0.011559	-0.011559
[3921]	NR5A1	--->	JUN	0.24409	-0.27062
[3922]	AP3B1	<---	BUB1	-0.33636	0.27287
[3923]	RACGAP1	--->	ECT2	0.074601	-0.068613
[3924]	SMG1	<---	PRKCI	-0.15427	0.14144
[3925]	RANBP9	--->	CALB1	0.10609	-0.10067
[3926]	IKBKG	<---	CASP8	-0.53808	0.44189
[3927]	RELB	--->	NFKB2	-0.0006231	-0.052631
[3928]	EPB41	--->	ACTA1	0.1724	-0.17979
[3929]	HSP90AA1	--->	BIRC5	0.059947	-0.068328
[3930]	BCL11B	<---	NR2F2	-0.08573	0.079553
[3931]	UBE2L3	--->	PTEN	0.33126	-0.30891
[3932]	HDAC7	<---	YWHAQ	-0.050481	0.060077
[3933]	MAPK10	--->	HNRNPK	0.43781	-0.56248
[3934]	GRM4	<---	GNAQ	-0.19572	0.21162
[3935]	CCNA1	<---	RBM4	-1.0044	0.99975
[3936]	ACTG1	<---	CFL2	-0.30587	0.30201
[3937]	PPARGC1A	--->	ESR1	0.013278	-0.021031
[3938]	ESR1	--->	SP3	0.092475	-0.080305
[3939]	TES	--->	ENAH	0.0093472	-0.019031
[3940]	SMAD4	--->	MAX	1.0041	-1.0094
[3941]	FUBP1	<---	CUGBP1	-0.0048144	0.036464
[3942]	HPCAL1	<---	HMG20A	-0.50177	0.53842
[3943]	CBX1	<---	CHAF1A	0.00039271	0.0033443
[3944]	TNFSF11	--->	MMP7	0.44952	-0.42626
[3945]	MERTK	--->	GAS6	0.1665	-0.1688
[3946]	DEDD2	--->	CFLAR	0.61024	-0.37846
[3947]	CUL3	--->	CCNE1	0.45733	-0.45665
[3948]	RRAD	--->	CALM1	0.73657	-0.70265
[3949]	MAPK3	--->	SREBF1	0.54159	-0.54127
[3950]	SMAD3	<---	ANK3	-0.066496	0.070266

[3951]	CCNB1	<---	CDKN1A	-0.99961	0.98767
[3952]	PRKAG3	<---	PRKAA1	-0.22052	-0.13077
[3953]	JAK1	--->	CSF3R	0.35799	-0.37099
[3954]	LYN	--->	FCAR	0.11748	0.015127
[3955]	DNMT3B	<---	EZH2	-0.059476	0.095626
[3956]	SYTL3	<---	RAB27A	-0.15731	0.20092
[3957]	PPP1R9A	<---	PRKAR1B	-0.10351	0.13877
[3958]	TNIP1	<---	TTF2	-0.046888	0.046179
[3959]	NCF1	--->	SFRS2	0.03659	-0.042059
[3960]	PTPN1	--->	INSR	0.26902	-0.28101
[3961]	CSNK2A2	--->	BID	0.77321	-0.7866
[3962]	GATA2	<---	CEBPA	0.007993	0.050146
[3963]	KAT2B	--->	RARA	0.15014	-0.14993
[3964]	PTPRN	--->	UBQLN4	1.2138	-1.2073
[3965]	CASP9	<---	DCC	-0.20326	0.19438
[3966]	GATA1	--->	FLI1	0.17161	-0.14597
[3967]	GHRL	--->	LEP	0.22582	-0.20756
[3968]	ARHGAP4	<---	NRAS	-0.084228	0.087638
[3969]	SHC1	<---	FLT4	-0.29858	0.19627
[3970]	TOM1L1	--->	PIK3R1	0.17284	-0.27759
[3971]	DHX9	<---	SSRP1	-0.063858	0.067398
[3972]	EFCAB6	--->	AR	0.58937	-0.59518
[3973]	SRPK1	--->	YWHAG	0.62776	-0.66368
[3974]	WWTR1	--->	NKX2-1	0.51864	-0.51881
[3975]	MATN2	<---	COL4A4	-0.028289	0.018624
[3976]	FES	--->	DOK1	0.72849	-0.81545
[3977]	IRAK1	--->	MAPK14	0.38787	-0.30391
[3978]	MAPK3	<---	DUSP3	-0.99819	1.011
[3979]	CASP1	--->	AR	0.99444	-1.0025
[3980]	AXIN1	--->	CTNNB1	0.058263	-0.069945
[3981]	SOX10	<---	HIVEP1	-0.050936	0.054534
[3982]	OPTN	<---	HTT	-0.12681	0.15659
[3983]	SOCS6	--->	PIK3R2	0.12705	-0.18873
[3984]	ADAP1	--->	PURA	0.028947	0.0050024
[3985]	SLC8A2	<---	YWHAE	-0.092105	0.10171
[3986]	PKN1	--->	TRAF2	-0.007131	-0.026005
[3987]	PPARG	--->	RXRA	0.60524	-0.60641
[3988]	TICAM2	--->	IRAK4	0.094487	-0.091207
[3989]	UPF3A	--->	RBM8A	0.039537	-0.0078563
[3990]	PPID	<---	MYST2	-0.010789	0.046835
[3991]	CHUK	--->	HIST3H3	0.41691	-0.61557
[3992]	CEBPB	--->	RELA	0.083071	-0.073817
[3993]	DLG2	<---	DLG4	-0.020129	-0.0093747
[3994]	HESX1	?	PROP1	-0.11417	-0.11417
[3995]	CARD16	?	CARD8	-0.10452	-0.10452
[3996]	TIAM1	<---	ANK1	-0.10129	0.088698
[3997]	CASP1	<---	CDC2L2	-0.57299	0.5681
[3998]	SMAD5	<---	SNRPA	-0.30655	0.28646
[3999]	DNMT1	<---	EZH2	0.0021912	0.014085
[4000]	NR0B2	--->	RXRB	-0.0094276	-0.028437
[4001]	USP7	--->	TP53	0.3914	-0.37879
[4002]	NRAP	--->	KBTBD10	0.10605	-0.071863
[4003]	EDAR	--->	TRAF2	0.093284	-0.12983
[4004]	DBP	<---	EP300	-0.18961	0.18576
[4005]	RGS7	--->	YWHAB	0.22227	-0.21545
[4006]	TICAM1	<---	TLR3	-0.12078	0.11896
[4007]	HIST2H2BE	<---	HIRA	-0.057829	0.10803
[4008]	NR4A2	?	RXRB	-0.0089162	-0.0089162
[4009]	BIRC6	<---	PLK1	-0.10467	0.080695
[4010]	PVRL3	--->	PARD3	0.042062	-0.053929
[4011]	SUFU	--->	GLI3	0.15344	-0.14553
[4012]	IPO5	<---	RAN	-0.094094	0.098188
[4013]	PRLR	<---	JAK2	-0.99828	0.99727
[4014]	MAPK7	--->	RXRA	0.61286	-0.69248
[4015]	IRS4	<---	CRK	-0.095511	0.072791
[4016]	PIK3R1	<---	GRB2	-0.62532	0.55159
[4017]	PAK2	--->	RPS6	0.78136	-0.45611
[4018]	TRIP10	--->	CTNNB1	0.33247	-0.33791
[4019]	ATN1	--->	LRP2	0.055337	-0.066829
[4020]	HDAC7	<---	NCOR2	-0.14391	0.1331
[4021]	CREBBP	--->	HDAC1	0.107	-0.1151
[4022]	DHX9	--->	HNRNPC	0.015842	-0.0019217
[4023]	SMARCA4	--->	NR3C1	0.0094759	-0.018029
[4024]	CCNB1	<---	MEF2C	-0.01407	0.0070079
[4025]	CDK2	--->	HIRA	0.83716	-0.86422
[4026]	CLNK	--->	LCP2	0.44219	-0.36879

[4027]	TBK1	<---	TRAF2	-0.59652	0.60306
[4028]	PPA1	--->	WIPI1	0.1554	-0.12257
[4029]	MAPK3	--->	PXN	0.57323	-0.59278
[4030]	PVRL3	<--->	PVRL1	0.0013613	0.0013613
[4031]	RAB3B	--->	CALM1	0.73657	-0.70265
[4032]	TNR	--->	VCAN	-0.0048741	-0.0088523
[4033]	TRADD	--->	KRT14	0.089075	-0.10859
[4034]	SMARCA4	--->	ACTB	0.070094	-0.081811
[4035]	KAT2B	--->	NOTCH3	0.15078	-0.16506
[4036]	WWOX	--->	TP73	0.12289	-0.11819
[4037]	GZMB	<---	CHRM3	-0.2044	0.21722
[4038]	SOX17	<---	CTNNB1	-0.017651	0.023932
[4039]	KAT2B	--->	CDC25B	0.11462	-0.11508
[4040]	SMAD3	<---	JUN	-0.035159	0.057717
[4041]	CDH9	<---	CTNNB1	-0.32695	0.32855
[4042]	ITGB2	<---	HP	-0.22647	0.21748
[4043]	CSNK2A2	--->	PTEN	1.0172	-1.002
[4044]	CDK6	--->	CCND3	1.0544	-1.0774
[4045]	DDX54	--->	PGR	0.042552	-0.032266
[4046]	CCL21	<---	CCRL1	-0.46475	0.49595
[4047]	PDZD2	<---	PKP4	-0.0046437	0.0069313
[4048]	PTPRN	<---	PTPRT	0.54111	0.55154
[4049]	PELI3	<---	MAP3K7	-0.26773	0.27455
[4050]	EP300	--->	TP53BP1	0.18012	-0.18462
[4051]	NFKB2	--->	HDAC4	0.010804	-0.028956
[4052]	MYF5	<---	CALM3	-0.22666	0.2676
[4053]	CCR1	--->	CREB3	0.31404	-0.17181
[4054]	LIFR	<---	CNTF	-0.11239	0.14194
[4055]	ITGB4	<---	YES1	-0.12001	0.12583
[4056]	CD28	--->	PIK3R1	0.14986	-0.25804
[4057]	XIAP	--->	BIRC5	0.036117	-0.091614
[4058]	MAPK8	--->	ATF2	1.0089	-1.0006
[4059]	PHF21A	<---	HMG20B	-0.17755	0.21079
[4060]	AURKAIP1	<---	AURKA	-0.74005	0.71415
[4061]	AKT1	--->	BAD	0.99987	-0.9934
[4062]	HNF4G	--->	GAPDH	0.19423	-0.19431
[4063]	HRAS	--->	PRKCI	0.99149	-1.0135
[4064]	CD8A	<---	LCK	-1.102	1.1416
[4065]	CPNE4	--->	RDX	0.052591	-0.045879
[4066]	CDK7	--->	CCNH	1.083	-1.1093
[4067]	MAP1LC3B	<---	UPF2	-0.019777	0.017356
[4068]	ONECUT1	<---	FOXA2	-0.39346	0.42236
[4069]	NSD1	--->	ESR1	0.12772	-0.10069
[4070]	HNF1B	--->	ATF1	0.30153	-0.25208
[4071]	TNFRSF1A	--->	GNB2L1	0.29828	-0.11449
[4072]	TRIP6	<---	NCK2	-0.25608	0.24792
[4073]	FBXL12	--->	CDKN1C	0.15077	-0.11847
[4074]	BMPR1B	<---	BMP7	-1.0007	0.99697
[4075]	PIK3R2	<---	SYK	-0.54248	0.45781
[4076]	BMPR1A	<---	BMP7	-1.0007	0.99697
[4077]	THRB	--->	RXRG	1.003	-0.99549
[4078]	CHUK	<---	MAP3K11	-0.16507	0.092342
[4079]	IGFBP3	<---	COL1A1	-0.032233	0.024387
[4080]	SKP1	--->	CENPE	0.12528	-0.11476
[4081]	FLNA	<---	RALA	-0.39396	0.4246
[4082]	S1PR4	--->	GNAI1	1.0129	-1.008
[4083]	ADAM15	<---	SH3RF3	-0.030333	0.027108
[4084]	NUMB	<---	L1CAM	-0.16568	0.1567
[4085]	SMURF2	--->	PRICKLE2	0.68232	-0.68627
[4086]	PDPK1	--->	SGK3	0.66384	-0.71595
[4087]	RAPGEF6	--->	RAP2A	1.0006	-0.99699
[4088]	MYOD1	<---	HSP90AA1	-0.13829	0.13081
[4089]	CASP14	--->	CASP1	0.1604	-0.089889
[4090]	CRADD	<---	CASP8	-0.48667	0.2142
[4091]	MYLIP	--->	MYL12B	0.11915	-0.11454
[4092]	RING1	<--->	BMI1	0.003228	0.003228
[4093]	VDR	--->	JUN	0.24409	-0.27062
[4094]	FOS	<---	STAT1	-0.096153	0.047047
[4095]	KAT2B	--->	NOTCH1	0.2543	-0.26983
[4096]	SHB	--->	EPS8	0.041547	-0.17834
[4097]	CRKL	<---	CBL	-0.37379	0.35632
[4098]	YWHAG	--->	GIT2	0.19425	-0.20074
[4099]	THRA	?	NR2F1	-0.0089162	-0.0089162
[4100]	RPS6KA1	--->	CEBPB	0.34196	-0.54207
[4101]	PDGFRB	--->	RASA1	0.010772	-0.055545
[4102]	PPYR1	<---	NPY	-0.19468	0.20502

[4103]	CCL22	?	CCL19	-0.047195	-0.047195
[4104]	MAPK8IP2	<---	MAP3K10	-0.090912	0.30425
[4105]	DAXX	--->	PAX5	0.044577	-0.062848
[4106]	F10	--->	PROS1	-0.007533	-0.0076005
[4107]	USP5	--->	DVL2	0.23053	-0.23594
[4108]	ABL1	--->	PLCG1	0.14898	-0.21216
[4109]	MAPK1	--->	TP53	1.0002	-0.99962
[4110]	GRK6	--->	BDKRB2	0.13522	-0.19362
[4111]	SOS1	--->	PLCG1	0.071806	-0.098834
[4112]	ERBB2	--->	SERPINA3	0.28428	-0.28844
[4113]	HDAC3	--->	BCOR	0.37055	-0.35594
[4114]	SH3RF1	<---	AKT2	-0.10192	0.14794
[4115]	YWHAG	<---	RACGAP1	-0.050693	0.05293
[4116]	NEUROD2	<---	PKN1	-0.075715	0.0046336
[4117]	MAPK8IP1	<---	AKT1	-0.1697	0.25408
[4118]	AR	<---	PIAS3	-0.36146	0.35279
[4119]	RHOG	--->	VAV2	0.08839	-0.14624
[4120]	TLE3	<---	FOXG1	-0.43004	0.45973
[4121]	PGR	<---	HMGB2	-0.48525	0.518
[4122]	SORBS3	--->	SAFB2	0.21756	-0.21698
[4123]	APBB1IP	--->	VASP	1.0039	-0.99848
[4124]	JAK2	--->	STAT5A	0.99488	-1.0064
[4125]	PCGF6	<--->	RNF2	0.003228	0.003228
[4126]	JUNB	<---	NINL	-0.74145	0.69597
[4127]	RNF11	--->	IKBKG	0.38348	-0.38235
[4128]	PAWR	<---	RRAS2	-0.40525	0.43624
[4129]	CDH9	?	CDH10	-0.058742	-0.058742
[4130]	HTRA1	<---	COL3A1	-0.021896	0.0096398
[4131]	EGFR	--->	ERBB2	0.0023711	-9.0961e-005
[4132]	KCNA3	<---	IL16	-0.013537	0.050181
[4133]	CPNE1	--->	ACTB	0.56315	-0.55654
[4134]	PPP2CA	--->	AKT1	0.35089	-0.342
[4135]	PPP2CB	--->	MAPT	0.29647	-0.30306
[4136]	KDR	--->	IQGAP1	0.42014	-0.39076
[4137]	SRC	--->	ADRB3	0.5564	-0.49364
[4138]	ELF5	<---	UBQLN4	-0.26725	0.28784
[4139]	F11R	<---	CASK	-0.021136	0.0040222
[4140]	USP7	--->	NECAB2	0.42587	-0.38506
[4141]	SOX8	--->	JUN	0.045434	-0.053286
[4142]	CALM1	<---	GRB7	-0.27129	0.19413
[4143]	FGF8	--->	FGFR4	1.0053	-0.99871
[4144]	NR4A2	<---	COPS5	-0.19181	0.21292
[4145]	PPP2R1A	--->	RAB7A	0.13801	-0.13281
[4146]	CDKN2A	<---	CDK4	-0.26299	0.25926
[4147]	FAS	<---	MET	-0.048345	0.13788
[4148]	RAB11FIP5	--->	RAB11FIP4	-0.0011865	-0.021155
[4149]	HDAC1	<---	NFKB1	-0.47011	0.46951
[4150]	CSNK1G2	--->	COL4A3BP	0.37965	-0.35275
[4151]	CADM1	--->	EPB41L3	0.017779	-0.029783
[4152]	SMAD6	<---	MAP3K7	-0.21256	0.23582
[4153]	RUNX2	<---	EP300	-0.21752	0.21368
[4154]	SLC9A2	--->	PIK3R1	-0.0088114	-0.024142
[4155]	PAX5	<---	CREBBP	-0.12275	0.11484
[4156]	RPH3A	--->	NRXN1	0.04918	-0.030139
[4157]	PRKCG	--->	DAB2	0.91797	-0.91615
[4158]	FLNA	<---	ARHGAP24	-0.19742	0.20476
[4159]	PI4KB	<---	YWHAG	-0.068047	0.092991
[4160]	PRMT1	--->	YLPM1	0.25061	-0.24539
[4161]	APPL1	<---	DCC	-0.26867	0.26291
[4162]	CIB1	<---	PSEN1	-0.34233	0.37985
[4163]	IRS1	<---	CHUK	-0.6179	0.56786
[4164]	NR5A2	<---	NPPA	-0.040788	0.066582
[4165]	MTNR1A	--->	RAP1A	0.066588	-0.039125
[4166]	GRK6	--->	LTB4R	0.31971	-0.40764
[4167]	MDM4	--->	TP53	0.40822	-0.41316
[4168]	KLK5	--->	SERPINA1	0.11833	-0.082185
[4169]	STK39	--->	MAPK14	0.30909	-0.32159
[4170]	PPM1G	--->	GRIA3	0.32843	-0.32078
[4171]	GSK3B	--->	MUC1	0.42246	-0.44429
[4172]	NOS3	<---	HSP90AA1	-0.076347	0.063245
[4173]	NR1H4	--->	RXRG	1.003	-0.99549
[4174]	GRHL1	?	GRHL3	-0.13135	-0.13135
[4175]	HSP90AB1	--->	STAT3	0.12931	-0.15209
[4176]	CDK2	--->	TP53	1.0002	-0.99962
[4177]	MDS1	--->	HDAC1	0.28508	-0.28349
[4178]	NSMAF	?	GNB2L1	-0.1554	-0.1554

[4179]	NPAS2	<---	EP300	-0.079572	0.069203
[4180]	FN1	<---	COL4A4	-0.061633	0.057845
[4181]	PTPN6	--->	GRB2	0.43839	-0.5864
[4182]	E2F3	--->	YY1	0.082581	-0.076815
[4183]	RAP1A	<---	RASA1	-1.0119	1.0001
[4184]	CYTIP	<---	CYTH1	-0.22934	0.25364
[4185]	BMPR1B	<---	RAB6B	-0.53994	0.52577
[4186]	PAK2	--->	SORBS1	0.22836	-0.066216
[4187]	MYC	<---	MAP2K1	-0.50038	0.65026
[4188]	TNK2	<---	RASGEF1C	-0.012241	-0.0013049
[4189]	CEBPB	<---	SP1	-0.18311	0.17655
[4190]	AKT1	--->	HMOX1	0.45236	-0.30551
[4191]	FOS	<---	SMAD3	-0.076892	0.036542
[4192]	FGFR3	--->	STAT3	0.10807	-0.14294
[4193]	PDCD6IP	<---	ATN1	-0.043089	0.040858
[4194]	CHUK	--->	ESR1	0.29669	-0.42769
[4195]	RASGRP3	<---	PRKCD	-0.4904	0.51446
[4196]	CASP3	--->	MDM4	0.092589	-0.12838
[4197]	PRKACA	--->	BAD	1.2302	-1.4217
[4198]	SORBS3	--->	ABL1	-0.098797	-0.22378
[4199]	CTSG	--->	IGFBP3	0.070438	-0.036479
[4200]	HNRNPL	<---	PJA1	-0.22785	0.22891
[4201]	CASP3	--->	MAPT	0.50832	-0.57833
[4202]	SPAG9	--->	MAX	0.13591	-0.13824
[4203]	ARRB1	--->	NFKBIA	0.48867	-0.49665
[4204]	ATXN1	--->	ZHX1	0.00068007	-0.0082126
[4205]	ERBB2	--->	MUC1	0.13324	-0.14446
[4206]	AXL	--->	SHC1	0.11493	-0.22569
[4207]	ATF3	<---	DDIT3	-0.2972	0.29414
[4208]	FCER1G	<---	FCGR3A	-0.4895	0.47597
[4209]	IRS4	<---	CRKL	-0.095511	0.072791
[4210]	ACR	--->	ZP3	0.12216	-0.088039
[4211]	PAK6	--->	AR	1.0096	-1.0048
[4212]	MAPKAPK5	<---	MAPK9	-0.45114	0.8761
[4213]	TNNT1	<---	PLEKHF1	-0.12519	0.13005
[4214]	CDK7	--->	LASP1	0.16334	-0.17365
[4215]	RET	--->	GRB2	0.15965	-0.34138
[4216]	NCOA1	<---	NKX2-1	-0.06802	0.06031
[4217]	SREBF1	<---	CREBBP	-0.056808	0.045169
[4218]	IGF1R	--->	IRS1	0.98642	-1.0008
[4219]	GMFB	<---	MAPK14	-0.48604	0.31596
[4220]	PEBP4	<---	MAP2K1	-0.17448	0.56179
[4221]	WASF1	--->	ACTB	0.26671	-0.2714
[4222]	ACVR1	<---	CDK14	-0.12661	0.18132
[4223]	KAT5	<---	C1orf174	-0.0092849	0.025151
[4224]	KCNF1	?	KCNG4	-0.025901	-0.025901
[4225]	NLK	--->	CREBBP	0.32272	-0.32425
[4226]	PPP6C	--->	TGFBR1	0.094059	-0.10679
[4227]	SMAD3	<---	CSH2	-0.21558	0.26049
[4228]	MAP2K1	--->	CASP9	0.66416	-0.42184
[4229]	SERPINE1	<---	PLG	-0.040728	0.057207
[4230]	CSF2RB	--->	STAT3	0.40372	-0.44604
[4231]	SRPK1	--->	SNRNP70	0.72994	-0.75755
[4232]	SUV39H1	--->	MBD1	0.0087871	-0.011533
[4233]	NSMAF	--->	CD40	0.093643	-0.10338
[4234]	MATK	<---	SRC	-0.016792	0.60459
[4235]	PCBP1	<---	RBM42	-0.0048144	0.036464
[4236]	TCF21	--->	TCF3	-0.13552	-0.39218
[4237]	KLKB1	--->	HGFAC	-0.0039613	-0.0063334
[4238]	ITPKA	--->	CALM1	1.1467	-1.1219
[4239]	NCR1	--->	CD59	0.25688	-0.2361
[4240]	MCL1	?	BOK	-0.63915	-0.63915
[4241]	CSTF1	<---	BRCA1	-0.10329	0.11835
[4242]	RGS12	--->	GNAI2	-0.00079669	-0.0029535
[4243]	NCF1	<---	NCF4	-0.0074756	-0.0017533
[4244]	PPP2R1A	--->	PPP2R2A	0.18879	-0.17841
[4245]	GNA12	--->	CDH1	0.20416	-0.20669
[4246]	C1QL3	--->	SERPINH1	0.031811	-0.038334
[4247]	NCOA1	<---	NR2F6	-0.043163	0.034596
[4248]	LYST	<---	HGS	-0.034151	-0.017685
[4249]	TP53	<---	SP3	-0.1417	0.14433
[4250]	MAPK8	--->	RASSF1	0.28192	-0.30531
[4251]	ZNF263	<---	EFEMP2	-0.18523	0.1912
[4252]	PRKCB	--->	PEBP1	1.0121	-1.008
[4253]	SKIL	<---	TRIM69	-0.15996	0.15273
[4254]	CAPN1	--->	TP53	0.17223	-0.17927

[4255]	CREBBP	--->	DACH1	0.12882	-0.13662
[4256]	CD79B	<---	CD19	-0.047792	0.047421
[4257]	APLP1	<---	PCDHA4	-0.040845	0.037941
[4258]	SCHIP1	--->	FOS	0.31132	-0.44491
[4259]	HES6	--->	HES1	-0.025997	-0.15188
[4260]	PTEN	<---	MAST2	-0.61289	0.61732
[4261]	RAB8B	--->	CDH1	0.2868	-0.28378
[4262]	HCRTR2	--->	CD200	0.23224	-0.21667
[4263]	TLR6	<---	TLR2	0.066482	0.28866
[4264]	PAK4	--->	ITGAV	0.023	-0.019356
[4265]	EFEMP1	<---	ARAF	-0.03651	0.011308
[4266]	F11R	--->	MPDZ	0.14909	-0.15671
[4267]	PRKCA	--->	ITPKB	0.87955	-0.8782
[4268]	APBB1	<---	FASLG	-0.08479	0.10232
[4269]	STK4	<---	CASP3	-0.59512	0.58465
[4270]	CBX5	<---	FSHR	-0.229	0.23994
[4271]	KLF4	--->	TP53	0.14433	-0.1417
[4272]	CCL16	<---	HRH4	-0.46475	0.49595
[4273]	NCOA2	<---	NR3C1	-0.043163	0.034596
[4274]	LRP5	<---	FZD8	-0.13464	0.12212
[4275]	RAC2	--->	SMAD1	0.52759	-0.54836
[4276]	SEC31A	?	SEC13	-0.1554	-0.1554
[4277]	FEM1B	<---	FAS	-0.11055	0.21889
[4278]	SMARCC1	<---	RELB	-0.38007	0.38113
[4279]	HNRNPU	--->	GRIN2D	0.11347	-0.11471
[4280]	CDK10	--->	ETS2	1.0002	-0.99981
[4281]	RASSF5	<---	RRAS2	-0.98684	1.0025
[4282]	HTR4	<--->	ADRB2	0.016768	0.016768
[4283]	BMPR1B	<---	STK35	-0.20564	0.30344
[4284]	PRKCD	--->	MUC1	0.29152	-0.29695
[4285]	TP53	--->	BCL2	0.061271	-0.062634
[4286]	SHC1	<---	ITGB4	-0.045901	0.011931
[4287]	ACVR1B	<---	INHBC	-0.71391	0.72054
[4288]	CXCR3	--->	CXCL10	0.49595	-0.46475
[4289]	ATXN1	<---	ZYX	-0.040288	0.037243
[4290]	GRIA2	--->	GRIA3	-0.020824	-0.080356
[4291]	STAT1	--->	IFNAR2	0.16145	-0.24585
[4292]	MYL2	--->	LRSAM1	0.0032816	0.0010171
[4293]	MSC	--->	TCF12	-0.091025	-0.33696
[4294]	ABCC9	<---	LDHA	-0.042256	0.039604
[4295]	KRAS	--->	FNTA	0.47292	-0.44041
[4296]	PRPF40A	--->	ZHX1	0.30261	-0.28863
[4297]	NIN	<---	GSK3B	-0.66014	0.63519
[4298]	RAPGEF1	<---	GRB2	-0.50299	0.50435
[4299]	YWHAZ	<---	BCAR1	-0.0086968	0.009842
[4300]	RFXANK	<---	CIITA	-0.63397	0.67163
[4301]	ZFPM1	<---	GIPC1	-0.12106	0.14031
[4302]	TRAF1	<---	TNFRSF8	-0.062073	0.081714
[4303]	BMPER	--->	BMP4	0.71403	-0.67452
[4304]	SMAD2	<---	SNRNP70	-0.32493	0.40696
[4305]	TCF3	<---	ASCL3	-0.39218	-0.13552
[4306]	PPP2R1A	--->	CDK1	0.30999	-0.30289
[4307]	PBX3	--->	MEIS1	0.024726	-0.0094151
[4308]	VAV1	<---	ZNF655	-0.048212	-0.0046722
[4309]	BCR	<---	IGSF21	-0.0071143	-0.0026803
[4310]	MASP1	<---	C3	-0.072287	0.071793
[4311]	THBS1	--->	LRP1	-0.0009446	-0.0072655
[4312]	CAMK2D	--->	ACTN2	-0.0067734	-0.0078992
[4313]	CUL7	--->	PLXNB3	0.069749	-0.036353
[4314]	RGS14	--->	GNAI3	0.013429	-0.011715
[4315]	CNKSR3	--->	DLL1	0.39712	-0.40361
[4316]	ARHGAP17	<---	GRB2	-0.1599	0.18798
[4317]	IRAK2	<---	TRAF6	-0.59748	0.62876
[4318]	PDK4	--->	PDHA1	1.0004	-0.99606
[4319]	CARD8	<---	NLRP3	-0.14874	0.18479
[4320]	FBXW7	--->	CUL1	0.11251	-0.11805
[4321]	KLF13	<---	SERPINH1	-0.24666	0.24832
[4322]	MATN4	?	MATN1	-0.013202	-0.013202
[4323]	NIN	<---	AURKA	-0.66014	0.63519
[4324]	INPP5D	<---	MET	-0.36899	0.24297
[4325]	PBX1	--->	PAX6	-0.043846	-0.099649
[4326]	RASD2	--->	PIK3CA	0.98913	-1.0082
[4327]	AKAP13	<---	YWHAE	-0.0046727	0.0055202
[4328]	RIN1	<---	RRAS2	-0.99801	1.0021
[4329]	EFEMP2	<---	LRDD	-0.04582	0.032721
[4330]	YWHAG	<---	WEE1	-0.66368	0.62776

[4331]	SP3	<---	E2F1	-0.10763	0.11252
[4332]	SMAD3	<---	WEE1	-1.0074	1.012
[4333]	IQGAP1	<---	CDC42	-0.13647	0.1548
[4334]	ROCK1	--->	PPP1R12A	0.99868	-0.99668
[4335]	TJP1	--->	GJA1	0.031431	-0.038078
[4336]	ACTG1	?	ACTB	-0.33964	-0.33964
[4337]	SLC9A3R2	--->	EZR	0.061503	-0.056659
[4338]	FADD	<---	DAPK1	-0.099015	0.115
[4339]	SLC9A3R1	--->	TRPC5	0.078517	-0.090326
[4340]	PAK1	--->	CDC2L2	0.8645	-0.80608
[4341]	TRIP6	--->	GFI1B	0.15476	-0.15855
[4342]	POU3F4	?	POU3F3	-0.11417	-0.11417
[4343]	CRIP2	<---	ATN1	-0.03622	0.034156
[4344]	MARK4	<---	STK11	-0.0050123	0.024635
[4345]	WAS	--->	PLCG1	0.20268	-0.23857
[4346]	LILRB4	--->	INPP5D	0.40645	-0.40243
[4347]	G3BP2	--->	NFKBIA	0.8008	-0.80634
[4348]	SH3GL3	--->	WDR33	0.050125	-0.053897
[4349]	MMP9	<---	IL8	-0.16713	0.18806
[4350]	DUSP6	--->	MAPK3	1.0016	-1.0004
[4351]	SMO	--->	PTCH1	1.0035	-0.99705
[4352]	MAP4K4	--->	DOK1	0.15272	-0.029751
[4353]	SH2D3C	<---	CARD11	-0.035406	0.01707
[4354]	KAT5	--->	HDAC7	0.016238	-0.012088
[4355]	HNRNPK	<---	RBM42	-0.0048144	0.036464
[4356]	COL4A3BP	<---	COL4A3	-0.0093832	0.00069712
[4357]	RBMX	<---	CCDC33	-0.039791	0.077746
[4358]	SOCS1	--->	CSF1	0.017899	0.0036002
[4359]	SMAD4	--->	HMG20A	0.18625	-0.10994
[4360]	ATR	<---	CHEK1	-0.15963	0.14398
[4361]	PTPN12	--->	WAS	0.99884	-0.98948
[4362]	ELANE	<---	F2RL1	-0.42368	0.45462
[4363]	SMAD4	<---	MAPK13	-0.8927	0.88461
[4364]	XRN1	<---	ALDOA	-0.0034694	0.0071858
[4365]	ZBTB43	--->	ZNF417	-0.027711	-0.050787
[4366]	FCAR	<--->	FCGR1A	0.45908	0.45908
[4367]	RARB	--->	RXRA	1.003	-0.99549
[4368]	MMP10	?	MMP9	-0.12951	-0.12951
[4369]	PRPF40A	<---	SRPK2	-0.49485	0.46953
[4370]	HIPK2	--->	SMAD1	1.2525	-1.3294
[4371]	ATF1	<---	CSNK2A2	-1.1667	1.2086
[4372]	HDAC2	--->	IKZF1	0.065285	-0.060684
[4373]	PDK2	--->	HIC2	0.18494	-0.19411
[4374]	COL5A3	<---	COL1A2	-0.0044333	0.011376
[4375]	PLD2	<---	FYN	-0.29631	0.30015
[4376]	TP53BP2	--->	TP53	0.12088	-0.1242
[4377]	CTNNB1	<---	PSEN1	-0.99666	1.0019
[4378]	CREBBP	--->	GLI3	0.0047264	-0.017445
[4379]	BTRC	--->	RELA	0.17009	-0.17982
[4380]	FBXW7	--->	NOTCH1	0.25441	-0.26994
[4381]	PTCH1	<---	IHH	-1.0096	0.99953
[4382]	TNIP2	<---	TEK	-0.015575	0.0016919
[4383]	SIK2	<---	CRTC2	-0.075794	0.069802
[4384]	ATN1	--->	TEP1	0.031383	-0.038326
[4385]	NCK2	--->	PAK1	0.32954	-0.31023
[4386]	ERBB2IP	--->	CDH1	0.080001	-0.083248
[4387]	PPM1B	--->	MAP3K7	0.99683	-1.0147
[4388]	CBLC	--->	LYN	0.23569	-0.21308
[4389]	TRIP10	<---	CDC42	-0.10219	0.10374
[4390]	MAPK3	--->	RPS6KA3	1.002	-1.0109
[4391]	DVL2	<---	PARD6A	-0.14636	-0.011932
[4392]	CDK5	--->	EZR	0.28628	-0.30395
[4393]	DLG4	--->	KCNJ4	0.04551	-0.04695
[4394]	TJP3	<---	GJC1	-0.012642	0.011892
[4395]	CTNNB1	<---	EP300	-0.032118	0.019033
[4396]	VAV2	<---	RAC1	-0.14624	0.08839
[4397]	ESR1	--->	CCNT1	0.17019	-0.14481
[4398]	AR	<---	CCNE1	-1.004	0.99358
[4399]	MAPK3	--->	TGIF1	1.0017	-1.0013
[4400]	OPTN	<---	TBC1D17	-0.13183	0.16163
[4401]	ICAM1	--->	EZR	0.048902	-0.056818
[4402]	SVIL	<---	SH3PXD2A	-0.02872	0.044413
[4403]	RBMX	?	RBM7	-0.016298	-0.016298
[4404]	PTK2B	--->	SHC1	0.060324	-0.26424
[4405]	MAPK3	--->	MYC	1.0154	-1.0065
[4406]	DVL1	--->	CTNNB1	0.041299	-0.052812

[4407]	RASSF5	--->	STK4	0.99683	-1.0036
[4408]	TNK2	<---	EGFR	-0.0095055	-0.0055006
[4409]	SLC2A1	<---	CALR	-0.26418	0.27658
[4410]	SH2B2	<---	BTK	-0.55049	0.37083
[4411]	AXIN2	--->	APC	-0.0036235	-0.010123
[4412]	RGL1	<---	RIT1	-1.0084	0.98547
[4413]	E2F1	<---	GSK3B	-0.46975	0.44723
[4414]	GRB2	<---	CBL	-0.58879	0.57629
[4415]	FLOT1	<---	SORBS1	-0.10071	0.11542
[4416]	RHPN1	<---	RHOA	-0.17126	0.18859
[4417]	CCR2	--->	CCL7	0.49595	-0.46475
[4418]	PTPRF	--->	IRS1	0.038807	-0.049156
[4419]	PFKM	--->	NOS1	0.27356	-0.28318
[4420]	HNRNPC	<---	LMO3	-0.021109	0.054056
[4421]	PBX1	<---	MYH10	-0.0092595	-0.00084646
[4422]	PTPN13	--->	CTNNB1	0.067318	-0.07944
[4423]	REPS1	<---	CRK	-0.11069	0.080507
[4424]	CDK7	--->	GTF2H2	0.70901	-0.73498
[4425]	PPARD	<---	RANBP9	-0.0368	0.046971
[4426]	EPHB2	--->	RYK	0.21303	0.078436
[4427]	MBTPS1	--->	ATF6B	0.25059	-0.21726
[4428]	PLCB1	<---	GNAQ	-1.0016	0.99447
[4429]	DLG4	<---	PTPRG	-0.34428	0.33629
[4430]	TBC1D3C	<---	RAB5A	-0.35867	0.38941
[4431]	DLAT	<---	PDK4	-0.31137	0.32371
[4432]	BMP1	<---	YLPM1	-0.083544	0.10412
[4433]	SIK2	--->	IRS1	0.5492	-0.60947
[4434]	HRAS	--->	CDC25A	0.33547	-0.30986
[4435]	MMP3	<---	CCL2	-0.16713	0.18806
[4436]	RCN1	<---	ATXN1	-0.019423	0.012532
[4437]	GNB2L1	--->	IGF1R	0.017777	-0.028991
[4438]	KAT5	<---	CREBBP	-0.11551	0.1074
[4439]	CDH5	<---	CSK	-0.58183	0.67397
[4440]	CASP3	--->	XIAP	0.30534	-0.38127
[4441]	PTPN11	--->	CAT	0.5826	-0.47371
[4442]	HSPB3	<---	MRPL38	-0.043091	0.091224
[4443]	NCF1	<---	NCF2	0.010724	0.030471
[4444]	SORBS2	<---	AKT1	-0.063369	0.13405
[4445]	PRKACA	--->	MAP2	0.56962	-0.58579
[4446]	ELAVL2	?	ELAVL4	-0.016298	-0.016298
[4447]	TSC1	<---	YWHAH	-0.08934	0.11987
[4448]	HDAC3	--->	MBD1	0.05057	-0.034081
[4449]	SMYD3	--->	PBX2	0.073287	-0.069552
[4450]	ZNF24	<---	COPS6	-0.37172	0.36975
[4451]	PPIL2	<---	BSG	-0.35632	0.35889
[4452]	MAP2K7	<--->	MAPK9	0.23429	0.23429
[4453]	TOX4	<---	HIPK3	-0.89929	0.92033
[4454]	CBLC	--->	CRK	0.19562	-0.21387
[4455]	DIXDC1	--->	AXIN1	-0.022639	-0.14331
[4456]	NRXN2	--->	MACF1	0.14785	-0.14256
[4457]	KHDRBS1	--->	STAT3	0.16476	-0.19303
[4458]	HTT	<---	RASA1	-0.29228	0.14973
[4459]	CXCR2	--->	CXCL2	0.49595	-0.46475
[4460]	CEBPB	<---	NR3C1	0.008943	0.026385
[4461]	DLG2	<---	ERBB4	-0.10094	0.091947
[4462]	RAB11FIP2	--->	REPS1	0.068394	-0.028045
[4463]	C1RL	?	HP	-0.00064986	-0.00064986
[4464]	TRIM23	--->	LNX1	0.17789	-0.17567
[4465]	TRPV4	--->	PACSIN3	0.035277	-0.038389
[4466]	RGS10	<---	GNRHR	0.011049	0.015577
[4467]	POU1F1	<---	CREBBP	-0.18943	0.18505
[4468]	RIMS2	<---	RAB3A	-0.0067445	0.016184
[4469]	GRIN1	<---	DUSP4	-0.31328	0.31254
[4470]	KCNA2	<---	CTTN	-0.017104	0.054466
[4471]	MTOR	--->	YWHAQ	0.16113	-0.16338
[4472]	HERC5	--->	CCNE1	0.47579	-0.47291
[4473]	YAP1	--->	TP73	0.28729	-0.28438
[4474]	ZHX1	<---	PIAS4	-0.13493	0.13648
[4475]	PDCD6	--->	FASLG	0.035949	-0.01742
[4476]	PTPN6	--->	SIRPA	0.97001	-0.82769
[4477]	HSPA8	--->	ALDOB	0.84403	-0.80192
[4478]	PTPN1	--->	LAT	1.2623	-1.2506
[4479]	DLGAP3	<---	DLG1	-0.040133	0.038414
[4480]	TNFRSF4	--->	TRAF1	0.081714	-0.062073
[4481]	DAAM1	<---	RHOA	-0.99591	0.99276
[4482]	HRH4	--->	GNAO1	1.0129	-1.008

```
[4483]  PTPN11  --->    KIR2DL5A        0.35299 -0.23556
[4484]  LIMK2   --->    CFL1    0.16176 -0.17545
[4485]  PRKCA   --->    INSR    0.19651 -0.20082
[4486]  SHC1    --->    LRP1    -0.0015337      -0.019167
[4487]  RALBP1  --->    CCNB1   0.46936 -0.46968
[4488]  SIGLEC7 <---    TRAF4   -0.011707       0.0017322
[4489]  RARA    <---    GADD45G 0.0079001       0.017932
[4490]  CCND1   <---    ESR1    -0.31113        0.3199
[4491]  ARF3    --->    ATN1    0.020039        -0.023318
[4492]  EGFR    <---    PRKD1   -1.0026 0.99527
[4493]  MDM2    --->    USP7    0.11459 -0.11667
[4494]  EMID2   --->    SERPINH1        0.042795        -0.037448
[4495]  FLNB    <---    PSEN2   -0.033635       0.067884
[4496]  RPS6KA5 <---    MAPK11  -0.84011        0.84931
[4497]  YWHAG   <---    PAK1    -0.29172        0.56543
[4498]  MUC1    <---    GRB2    -0.46617        0.45373
[4499]  CASP7   --->    EIF2AK2 0.87492 -0.98085
[4500]  CDKN2C  <---    CDK6    -1.3222 1.3062
[4501]  CASK    <---    RAB3A   -0.22997        0.22271
[4502]  UPF2    <---    MAP1A   -0.012868       0.0066187
[4503]  BIRC5   <---    CASP7   -0.74701        0.6341
[4504]  HNRNPK  ?       PCBP1   -0.037462       -0.037462
[4505]  INSR    --->    JAK1    0.090431        -0.090115
[4506]  KLF11   <---    HDAC1   -0.13401        0.14006
[4507]  MAPK1   --->    PPARG   0.60373 -0.61504
[4508]  KLHL20  <---    ECT2    -0.081601       0.11566
[4509]  KIT     <---    PRKCB   -0.99338        1.0004
[4510]  ALB     <---    ST13    -0.083563       0.11628
[4511]  SERPINA1        <---    PRTN3   -0.082185       0.11833
[4512]  MAP3K5  --->    RAF1    0.3634  -0.21862
[4513]  GNAI2   <---    CNR1    -0.99753        1.0047
[4514]  PIK3R1  <---    IGF1R   -0.083012       0.046534
[4515]  RET     --->    DOK6    0.31842 -0.35228
[4516]  ADAM12  <---    PACSIN3 -0.040125       0.043372
[4517]  SOCS3   <---    PPP3CB  -0.096714       0.088321
[4518]  MFAP4   <---    SFTPD   -0.041737       0.032301
[4519]  SHC1    <---    MST1R   -0.5154 0.23511
[4520]  PTEN    <---    STK11   -0.79908        0.84207
[4521]  SGSM3   --->    NF2     0.052611        -0.061503
[4522]  DTNA    <---    SNTA1   0.0159  0.020806
[4523]  SMAD5   <---    SOX5    -0.075374       0.055924
[4524]  CCR5    <---    JAK2    -0.17451        0.2955
[4525]  CSNK2A1 --->    PSEN2   0.67463 -0.70045
[4526]  GHR     <---    CISH    -0.038535       0.031349
[4527]  TGFBRAP1        <---    TGFBR1  -0.37522        0.30018
[4528]  SKIL    <---    ZBTB6   -0.051878       0.041548
[4529]  TAL2    <---    LMO2    -0.58677        0.62562
[4530]  HGS     <---    UBQLN4  -0.061475       0.0099058
[4531]  SHB     <---    CRK     -0.087097       0.12519
[4532]  RAN     --->    AR      0.97383 -0.97461
[4533]  BTC     --->    MMP9    0.26537 -0.24473
[4534]  CCND3   <---    EFEMP2  -0.61656        0.63059
[4535]  PPM1A   --->    SMAD2   0.99247 -0.99033
[4536]  STRN4   <---    CAV1    -0.11755        0.14419
[4537]  RALBP1  --->    REPS1   0.14798 -0.13774
[4538]  EGFR    --->    ICAM1   0.071062        -0.083289
[4539]  CCNA1   <---    E2F1    -0.36974        0.37157
[4540]  SH3D19  <---    SH3GL1  0.0017459       0.071738
[4541]  HDAC1   --->    NR2F2   0.085304        -0.074402
[4542]  SPTA1   --->    EVL     0.061308        -0.06966
[4543]  CHEK1   <---    HSP90AA1        -0.056762       0.040193
[4544]  CXCL13  ?       CCL19   -0.047195       -0.047195
[4545]  RB1     <---    E2F1    -0.28973        0.28304
[4546]  TAOK2   --->    ELK1    1.0428  -0.98814
[4547]  FNBP1L  --->    SGSM2   0.027498        -0.035725
[4548]  YWHAG   --->    TBC1D4  0.0064027       -0.013805
[4549]  CDK8    --->    CCNH    1.083   -1.1093
[4550]  MBD2    <---    HDAC1   -0.074216       0.091026
[4551]  CDK7    --->    PCGF6   0.5807  -0.60171
[4552]  GNB2L1  <---    JAK1    -0.54505        0.5344
[4553]  RAX2    ?       CRX     -0.11417        -0.11417
[4554]  SMAD4   <---    STK35   -1.0074 1.012
[4555]  OPRM1   --->    FLNA    0.23122 -0.21829
[4556]  PARVA   <---    LIMS1   -0.030995       0.066481
[4557]  CSK     --->    MAPK15  1.3862  -1.2776
[4558]  SMARCA4 --->    IKZF1   0.0099508       -0.019864
```

[4559]	DHX9	--->	TBP	-0.0030668	-0.0098461
[4560]	EGFR	--->	SLC3A2	0.089972	-0.099302
[4561]	SMAD2	<---	HGS	-0.096565	0.086433
[4562]	ZNF521	<---	SMAD1	-0.10753	0.095653
[4563]	PPP2CA	--->	PXN	0.29391	-0.30152
[4564]	MDM2	--->	UBE2A	0.085469	-0.088702
[4565]	PPP2R1A	--->	PPP5C	0.064649	-0.071413
[4566]	SHC1	<---	FLT1	-0.17617	0.10881
[4567]	INADL	--->	GRIN2C	0.11324	-0.11391
[4568]	NCOA1	<---	PRMT2	-0.070867	0.060713
[4569]	VASP	<---	TRIM9	-0.02355	0.019876
[4570]	RNF111	--->	SMAD7	1.0166	-0.99999
[4571]	PIAS1	--->	DDX21	0.070795	-0.069357
[4572]	SLC2A4	<---	PSMD7	-0.40316	0.4136
[4573]	GPS2	<---	PBK	-0.72853	0.7025
[4574]	CDH1	<---	VCL	-0.3322	0.33273
[4575]	SNX1	--->	INSR	0.13241	-0.14074
[4576]	CCNB1	<---	BRCA1	-0.39925	0.40227
[4577]	RGS18	--->	GNAQ	1.0012	-1.0015
[4578]	CASP3	--->	RB1	0.41973	-0.45426
[4579]	RAPGEF5	<---	RAP2A	-0.99657	0.99903
[4580]	CEACAM3	<---	SRC	-1.0576	1.0651
[4581]	A1CF	?	CUGBP2	-0.016298	-0.016298
[4582]	RAB4A	--->	APBB3	0.077953	-0.065182
[4583]	PKN1	<---	MAP2K6	-0.34453	0.33329
[4584]	ACTR3	<---	CDC42	-0.74828	0.773
[4585]	HP	--->	ITGAM	0.077373	-0.088946
[4586]	BRCA1	--->	GFI1B	0.092878	-0.1007
[4587]	CBL	--->	VAV1	0.027285	-0.065069
[4588]	NEK6	--->	CCDC85B	0.56074	-0.68539
[4589]	MTA1	<---	HDAC1	-0.094626	0.11539
[4590]	ABCD2	?	ABCD3	-0.062455	-0.062455
[4591]	ALB	<---	F7	-0.040089	0.043268
[4592]	LEP	--->	A2M	0.264	-0.23147
[4593]	TMEM189-UBE2V1	<--->	UBE2N	0.21837	0.21837
[4594]	DLG4	--->	BAI1	0.069136	-0.076242
[4595]	PTPRS	--->	UBR1	0.2501	-0.25535
[4596]	KAT2B	--->	ONECUT1	0.17193	-0.17199
[4597]	BCL6B	?	BCL6	-0.085302	-0.085302
[4598]	ERBB2IP	--->	SMAD3	0.17801	-0.15234
[4599]	RANBP9	--->	AR	0.57676	-0.58261
[4600]	LMX1B	--->	TCF3	0.21146	-0.21856
[4601]	PDCD6IP	<---	TTF2	-0.0074612	0.011209
[4602]	RIMS2	<---	RAB3D	-0.0067445	0.016184
[4603]	PTK2B	--->	PIK3R1	0.046022	-0.2003
[4604]	PRG2	<---	PRKCZ	-0.28009	0.26631
[4605]	MAPK3	--->	GTF2I	0.71989	-0.74424
[4606]	DAB2	<---	SRC	-0.90972	1.0524
[4607]	PRSS3	--->	SERPINA1	0.11833	-0.082185
[4608]	PACSIN3	<--->	PACSIN2	0.019599	0.019599
[4609]	HDAC2	--->	STAT3	0.34132	-0.37904
[4610]	PLCG1	<---	DOK1	-0.66729	0.59738
[4611]	OPRD1	--->	ARRB1	0.27159	-0.25831
[4612]	CMA1	--->	SERPINA3	0.11833	-0.082185
[4613]	DDX20	<---	NCOR1	-0.04274	0.033531
[4614]	CDC14A	--->	TP53	0.61877	-0.60562
[4615]	ALX4	?	ALX1	-0.12441	-0.12441
[4616]	GATA4	<---	MAPK3	-0.99372	1.0021
[4617]	PCYT1B	--->	ACTA2	0.54914	-0.54501
[4618]	RAB6B	--->	RABGAP1	0.15635	-0.15297
[4619]	CDKN1B	<---	GRB2	-0.12901	0.12513
[4620]	FSHR	<---	ADRBK1	-0.13993	0.1237
[4621]	IGF1R	<---	EEA1	-0.041185	0.030065
[4622]	PXN	<---	SELE	-0.10943	0.10751
[4623]	DLL1	<---	MAGI2	-0.15208	0.14094
[4624]	BRD8	--->	PPARG	0.06778	-0.057557
[4625]	CCRL1	--->	CCL7	0.49595	-0.46475
[4626]	GAB2	<---	CRKL	-0.47659	0.44936
[4627]	RYBP	--->	RNF2	0.065528	-0.030268
[4628]	PIK3CD	<---	HRAS	-1.0082	0.98913
[4629]	PAK2	<---	RAC1	-1.0053	0.99704
[4630]	VEGFA	--->	KDR	0.99212	-0.9972
[4631]	LRP5	<---	THBS1	-0.0076735	0.0093146
[4632]	SMAD1	--->	ZNF76	0.010639	-0.02457
[4633]	PSMD7	<---	TRAF6	-0.29961	0.28937
[4634]	MOBKL3	--->	KIDINS220	0.045612	-0.028163

4637	[4635]	MAPK14	--->	GRB2	0.15728	-0.29064
4638	[4636]	MICALL1	<---	YWHAG	-0.036876	0.046853
4639	[4637]	APBB1	--->	APP	0.22101	-0.23254
4640	[4638]	TRADD	--->	TRAF3	0.0665	-0.080187
4641	[4639]	NTRK2	--->	FRS3	0.20066	-0.2014
4642	[4640]	SERPINA5	<---	FGG	-0.083807	0.11995
4643	[4641]	PPP2CA	--->	MAPT	0.29647	-0.30306
4644	[4642]	SPOCK1	--->	MMP14	0.25303	-0.23217
4645	[4643]	CCR3	--->	CXCL10	0.49595	-0.46475
4646	[4644]	CDK2	--->	ID3	1.5563	-1.5271
4647	[4645]	TRIM6	<---	TRIM5	-0.00071882	0.0019784
4648	[4646]	SCN4A	<---	SNTB2	-0.013537	0.050181
4649	[4647]	ADRA1A	--->	FLNC	0.59171	-0.58518
4650	[4648]	TGM2	<---	LTBP1	-0.094206	0.058372
4651	[4649]	LUC7L3	--->	SFRS1	0.045772	-0.014362
4652	[4650]	SGK1	--->	KCNJ1	0.34518	-0.55991
4653	[4651]	SNX4	<---	BIN1	-0.091257	0.10562
4654	[4652]	FN1	<---	CTSD	-0.40338	0.41015
4655	[4653]	PLK1	--->	TSC1	0.33483	-0.33879
4656	[4654]	SKAP1	<---	GRB2	-0.49159	0.44305
4657	[4655]	ECT2	<---	RHOG	-0.29753	0.32945
4658	[4656]	MAPK14	--->	MEF2A	0.63663	-0.66297
4659	[4657]	AKT1	--->	BRCA1	0.2728	-0.18845
4660	[4658]	PDGFRA	<---	PDGFA	-0.99672	1.0139
4661	[4659]	YWHAE	<--->	YWHAH	0.069023	0.069023
4662	[4660]	NR3C1	--->	CALM1	0.15612	-0.12827
4663	[4661]	PYCARD	--->	NLRC4	-0.04463	-0.087394
4664	[4662]	DNM1	<---	SH3GL1	-0.10584	0.084936
4665	[4663]	PDGFB	--->	THBS1	0.24307	-0.19101
4666	[4664]	SOCS3	<---	IGF1R	-0.04286	0.019972
4667	[4665]	CCRL1	--->	CCL5	0.49595	-0.46475
4668	[4666]	PXN	<---	PTPN12	-0.49509	0.4699
4669	[4667]	MECP2	<---	SMARCA2	-0.013594	0.0037111
4670	[4668]	SREBF2	<---	RAB11B	-0.42398	0.41766
4671	[4669]	HIPK2	--->	NKX2-1	0.79979	-0.82765
4672	[4670]	CASP3	--->	CDKN1A	0.38241	-0.45789
4673	[4671]	TLN1	<---	VCL	-0.23428	0.2253
4674	[4672]	PTK2	--->	PLCG1	0.2822	-0.40233
4675	[4673]	NR6A1	<---	NCOR1	-0.065644	0.063164
4676	[4674]	LMO2	--->	SMAD2	0.42147	-0.33807
4677	[4675]	PRKCQ	<---	AKT1	0.056202	0.14023
4678	[4676]	MAP2K4	<--->	MAP3K14	0.23429	0.23429
4679	[4677]	FOXA1	--->	AR	0.97002	-0.97078
4680	[4678]	WAS	--->	PIK3R1	0.0085291	-0.063144
4681	[4679]	PIK3R3	--->	RB1	0.27367	-0.28878
4682	[4680]	TRIM24	--->	RXRA	0.066907	-0.27692
4683	[4681]	KCNQ5	<---	SRC	-0.89226	1.0056
4684	[4682]	CASP3	--->	BCL2	0.81871	-0.89609
4685	[4683]	PILRB	<---	SAMSN1	-0.057312	0.068718
4686	[4684]	BAZ1A	--->	SMAD9	0.26735	-0.28631
4687	[4685]	RAPH1	--->	DNMBP	-0.0050956	-0.009897
4688	[4686]	FHL2	--->	IGFBP5	0.096866	-0.061552
4689	[4687]	PARK2	--->	CASP1	0.097493	-0.089017
4690	[4688]	NEBL	--->	ACTB	0.61659	-0.6148
4691	[4689]	WWP2	--->	IL2RG	0.12039	-0.13496
4692	[4690]	RASSF5	<---	RAP2A	-0.98684	1.0025
4693	[4691]	SIRT1	<---	EP300	-0.027305	0.014726
4694	[4692]	MPP5	<---	LIN7A	-0.020783	0.0077703
4695	[4693]	PKMYT1	--->	BCL2	1.1791	-1.2078
4696	[4694]	DAPK2	<---	CAMK2A	0.091111	0.2652
4697	[4695]	FBLN2	<---	COL18A1	-0.017901	0.0090977
4698	[4696]	PPFIA1	<---	PTPRD	-0.071311	0.064659
4699	[4697]	SLC2A4	<---	LEPRE1	-0.3292	0.35549
4700	[4698]	DNM2	<---	AMPH	-0.080916	0.071535
4701	[4699]	DHX9	<---	TOP2A	-0.096352	0.11115
4702	[4700]	PRKACA	--->	ATF1	1.2086	-1.1667
4703	[4701]	BAMBI	--->	SOX30	0.071575	-0.035829
4704	[4702]	DYRK1B	<--->	MAPK1	0.23429	0.23429
4705	[4703]	FRS2	<---	RET	-0.50436	0.48844
4706	[4704]	LAMA3	--->	LAMB3	-0.0059794	-0.006461
4707	[4705]	PLAT	--->	GRIN1	0.061804	-0.068056
4708	[4706]	CDKN1A	<---	TEX11	-0.067233	0.067693
4709	[4707]	CDC42EP1	<---	FHL3	-0.17707	0.097039
4710	[4708]	CDC42	--->	PARD6B	0.11085	-0.11319
4711	[4709]	TGFBR1	--->	UBE2E3	0.14121	-0.16772
4712	[4710]	PRKCI	--->	CHUK	0.25124	-0.25473

[4711]	SH3BP2	--->	DBNL	0.12545	-0.19104
[4712]	PTPRS	--->	MOBKL3	0.3091	-0.31489
[4713]	PABPC1	--->	GNB2L1	0.13878	-0.10831
[4714]	PRPF40A	--->	ENAH	0.032269	-0.042208
[4715]	RGS4	--->	GNAI1	1.0012	-1.0015
[4716]	CLIP1	<---	MAPRE2	-0.013075	0.047474
[4717]	CLIP1	<---	MTOR	-0.16387	0.16166
[4718]	SERPINB9	--->	ZHX1	0.057064	-0.040689
[4719]	CASP4	--->	APP	0.21556	-0.22729
[4720]	CTNND2	<---	FYN	-0.63172	0.7169
[4721]	DYRK1B	--->	MAPK14	1.0074	-0.9992
[4722]	GTF3C1	--->	IGFBP3	0.053382	-0.019519
[4723]	ITGA1	<---	TLN1	-0.037263	0.024265
[4724]	ESR1	--->	ZBTB17	0.14623	-0.15777
[4725]	GRIA4	<---	IQGAP1	-0.064722	0.07026
[4726]	KCNJ12	<---	SNTB1	-0.13918	0.14939
[4727]	SNIP1	<---	CREBBP	-0.11427	0.10619
[4728]	RGS7	--->	PKD1	0.16337	-0.15908
[4729]	HSPB1	<---	PPA1	-0.075126	0.12289
[4730]	HIRIP3	--->	HIST1H4A	0.23103	-0.17959
[4731]	CALCOCO2	--->	GABARAPL1	0.12444	-0.09126
[4732]	CARD8	<---	FHL2	-0.099561	0.13481
[4733]	PXN	<---	REPS2	-0.21231	0.21965
[4734]	SOCS2	<---	IGF1R	-0.04286	0.019972
[4735]	MAPK7	--->	MEF2A	0.15063	-0.20504
[4736]	TES	--->	EVL	0.10335	-0.085959
[4737]	MITF	<---	RPS6KA1	-1.0134	0.99254
[4738]	EPHB6	<---	CBL	-0.31938	0.3081
[4739]	ARHGEF4	--->	APC	0.014284	-0.028011
[4740]	JAK3	--->	IL2RG	0.2024	-0.20694
[4741]	RELA	<---	EGR1	-0.017715	0.015281
[4742]	PPP2R1A	--->	ZFYVE9	-0.0041302	-0.0071465
[4743]	PRKCZ	--->	PSEN1	0.25781	-0.27139
[4744]	INSR	<---	CSK	-0.03	0.10959
[4745]	CASP3	<---	PPP3CA	-0.024542	-0.0018284
[4746]	BAG3	--->	BCL2	0.52549	-0.52948
[4747]	FHL5	--->	SMAD1	0.31038	-0.32822
[4748]	BNC2	--->	RB1	0.28662	-0.29287
[4749]	MCRS1	--->	DAXX	0.13007	-0.15806
[4750]	DDX20	<---	HDAC2	-0.011157	0.015318
[4751]	CCNL1	<---	SFRS2	-0.31519	0.32379
[4752]	F2RL3	--->	F2	0.28486	-0.27153
[4753]	STAT1	--->	IL2RG	0.029702	-0.088804
[4754]	YWHAG	--->	HNRNPAB	0.092231	-0.070624
[4755]	CYTH2	--->	ITGB2	0.2297	-0.23928
[4756]	RHOXF2	<---	RBM9	-0.071742	0.10141
[4757]	ZMYND11	<---	NCOR1	-0.059686	0.053444
[4758]	PRMT3	--->	PABPN1	0.18339	-0.17035
[4759]	MAPK8	<---	DUSP1	-1.0004	1.0016
[4760]	SCHIP1	--->	NF2	0.033219	-0.028562
[4761]	UPF3A	<---	NXF1	0.01377	0.01964
[4762]	CASP14	--->	CASP4	0.1604	-0.089889
[4763]	PRKCSH	<---	RGS2	-0.38573	0.41618
[4764]	PLSCR1	--->	ZNF581	0.20563	-0.20263
[4765]	MAPK8IP2	--->	LRP1	0.08872	-0.099755
[4766]	ATM	<---	MDM4	-0.056244	0.043154
[4767]	HDAC1	--->	PPARD	0.085304	-0.074402
[4768]	IGFBP3	<---	FN1	0.0040995	0.024628
[4769]	GNAQ	<---	TBXA2R	-1.008	1.0129
[4770]	ACP5	--->	EGR2	0.26789	-0.25472
[4771]	TGFBR1	--->	CD44	0.99792	-1.0003
[4772]	HIST2H2BE	<---	KPNA1	-0.15414	0.20377
[4773]	RAB11A	--->	PSEN1	0.37382	-0.34322
[4774]	CBX8	--->	BMI1	0.066008	-0.045755
[4775]	MAPK1	--->	ARRB2	0.42184	-0.44306
[4776]	AGTR1	--->	ARRB1	0.25543	-0.25215
[4777]	HNRNPC	<---	KRAS	-0.38407	0.41218
[4778]	RGL4	<---	RALA	-0.76787	0.79809
[4779]	SMAD3	--->	AXIN2	0.01973	-0.018982
[4780]	PTK2B	<---	SRC	-1.0004	0.9912
[4781]	GNA12	<---	TSHR	-0.65379	0.65587
[4782]	TNFRSF11B	--->	FN1	0.06578	-0.016647
[4783]	KIT	<---	CBLB	-0.10729	0.089475
[4784]	CAMK1	--->	ATF1	1.2086	-1.1667
[4785]	NCOA3	<---	E2F1	-0.13636	0.12378
[4786]	HNRNPK	<---	SORBS3	-0.25155	0.2537

[4787]	MMP14	<---	TIMP2	-0.19321	0.21399
[4788]	STAP1	<---	KIT	-1.0064	1.0101
[4789]	CASP8	--->	FAS	0.18641	-0.38436
[4790]	SRC	--->	ESR1	0.61826	-0.55261
[4791]	REST	<---	HTT	-0.10231	0.1379
[4792]	LRP12	<---	ZFYVE9	-0.13873	0.13121
[4793]	CYTH1	--->	ACTA1	0.6814	-0.68593
[4794]	HTT	<---	GRB2	-0.29458	0.31443
[4795]	DDB2	<---	CUL4B	-0.19793	0.20913
[4796]	PIWIL4	--->	DICER1	0.042243	-0.02712
[4797]	CDK4	--->	BRCA1	0.32242	-0.34057
[4798]	CSPG4	<---	PTK2	-0.11678	0.011406
[4799]	IKBKG	<---	UBE2D3	-0.34369	0.3382
[4800]	BMPR1B	<---	BMP4	-1.0007	0.99697
[4801]	MMP2	<---	IGFBP3	-0.15977	0.18055
[4802]	MAPK8IP2	<---	MAP3K11	-0.090912	0.30425
[4803]	NTRK1	--->	SQSTM1	0.0025423	-0.013203
[4804]	TRPM7	--->	PLCB1	0.10364	-0.1136
[4805]	CASP3	<---	AKT1	-0.21376	0.25651
[4806]	HDAC1	<---	TOP2A	-0.074374	0.089479
[4807]	PDLIM1	<---	RLIM	-0.036433	0.033745
[4808]	SCN5A	<---	NEDD4L	-0.53576	0.53682
[4809]	EPHB2	--->	MLLT4	0.095031	-0.086065
[4810]	ATXN1	<---	ZNF488	-0.029824	0.026666
[4811]	PARVA	--->	PXN	0.20653	-0.1991
[4812]	MAPK8IP3	<---	PTK2	-0.033993	-0.00016415
[4813]	EEF1G	<---	HLTF	-0.027851	0.020334
[4814]	TRIM37	<---	TRAF5	-0.0059313	0.056497
[4815]	MAPK8	--->	PAX2	1.0032	-1.0033
[4816]	DLG4	--->	GRIK1	0.058844	-0.064781
[4817]	EPHB3	--->	RYK	0.21303	0.078436
[4818]	CEBPE	<---	SIPA1L1	-0.37545	0.36471
[4819]	IRS2	--->	PLCG1	0.096019	-0.12335
[4820]	TBP	--->	RELA	-0.0047485	-0.0085521
[4821]	IGF2	--->	IGFBP4	0.11742	-0.083423
[4822]	COPS6	--->	RBX1	0.23949	-0.21315
[4823]	L1CAM	--->	ITGA5	0.034223	-0.047563
[4824]	USP7	--->	BCOR	0.40532	-0.38879
[4825]	FFAR1	--->	GNAI1	1.0129	-1.008
[4826]	ARVCF	--->	CDH15	0.51654	-0.52601
[4827]	RAP1A	--->	HDAC1	0.21754	-0.20399
[4828]	RAP1A	--->	TNFRSF10C	0.40706	-0.38376
[4829]	PTPN22	--->	VAV1	0.027206	-0.11022
[4830]	PDGFRB	--->	GRB7	0.098098	-0.11309
[4831]	CHEK1	--->	TP53	1.0002	-0.99962
[4832]	PAX2	--->	ID2	0.13137	-0.10726
[4833]	GFI1B	<---	KALRN	-0.026201	0.013934
[4834]	IL8	<---	CXCR1	-0.46475	0.49595
[4835]	ECT2	<---	F2RL2	-0.38763	0.41981
[4836]	THBS1	<---	ITGB3	-0.066626	0.07704
[4837]	SMAD3	<---	CTCF	0.0043727	0.016992
[4838]	VIPR1	<---	GNAI1	-0.43656	0.4334
[4839]	IGF1	--->	IGF1R	0.99962	-1.0053
[4840]	SCRIB	--->	TRIP6	0.076735	-0.06172
[4841]	HSPA9	--->	TP53	0.6691	-0.65475
[4842]	PRKCA	--->	CD163	0.92534	-0.92282
[4843]	ZEB1	<---	EP300	-0.050022	0.038185
[4844]	CD3E	--->	SHC1	0.10772	-0.27137
[4845]	CDK2	--->	CEBPA	0.97322	-0.93471
[4846]	BTK	--->	STAT5A	0.22463	-0.23196
[4847]	LNX1	?	LNX2	-0.0041133	-0.0041133
[4848]	SFTPA2	?	SFTPA1	-0.0069911	-0.0069911
[4849]	CSRP3	--->	MYOG	0.62562	-0.58677
[4850]	DLG1	--->	KCNJ4	0.04551	-0.04695
[4851]	ARHGAP8	<---	RHOA	-0.40165	0.41486
[4852]	BTK	<---	PRKCA	-0.99198	0.99524
[4853]	SLC9A3R1	<---	ADRB2	-0.19778	0.21221
[4854]	SYNGAP1	<---	DLG3	-0.005916	-0.0033168
[4855]	SMAD2	<---	RXRA	-0.11079	0.053826
[4856]	FZD7	<---	MAGI3	-0.0054494	0.0026755
[4857]	PLCG1	<---	DAPP1	-0.72522	0.3591
[4858]	SMAD4	<---	CNKSR1	-0.3078	0.35086
[4859]	ZBTB16	<---	HDAC6	-0.067538	0.061181
[4860]	DDX5	--->	CALM1	0.071405	-0.066574
[4861]	HRAS	--->	BRAF	1.0064	-0.99496
[4862]	CTNNB1	<---	PSEN2	-0.99666	1.0019

[4863]	MAP2K4	--->	MAPK10	0.64901	-0.41806
[4864]	CTNNB1	<---	CHD8	-0.99476	0.99618
[4865]	BCL2L10	--->	BCL2	-0.23385	-0.7984
[4866]	CLK1	--->	PPIG	0.75438	-0.77211
[4867]	KPNA2	--->	CASP2	0.015167	-0.0079866
[4868]	FIZ1	<---	FLT3	-0.2366	0.22588
[4869]	ACVR1	<---	NRAS	-0.32935	0.31819
[4870]	ITCH	--->	NOTCH1	0.014558	-0.028638
[4871]	KLF6	<---	PNO1	-0.27198	0.27286
[4872]	MTSS1	<---	PTPRD	-0.17991	0.17157
[4873]	SMYD2	--->	TP53	0.39751	-0.38538
[4874]	IQGAP1	<---	RAC1	-0.13647	0.1548
[4875]	ENAH	<---	FAT1	-0.0069974	-0.0065934
[4876]	CCNB1	<---	PBK	-1.0774	1.0544
[4877]	GSK3B	<---	PTPN1	-1.009	1.0061
[4878]	GNAO1	<---	ADRA2C	-1.008	1.0129
[4879]	CXCL13	<---	FGF2	0.0061408	0.027574
[4880]	PLD1	<---	RALA	-1.0031	0.99718
[4881]	PPP1R9B	<---	RGS2	-0.41116	0.44344
[4882]	RB1	--->	ATF2	0.034772	-0.045438
[4883]	CREBBP	--->	NCOA2	0.020953	-0.033602
[4884]	DDX4	<---	RANBP9	-0.018321	0.022448
[4885]	CDC42BPG	<---	RAC1	-0.1141	0.11487
[4886]	TENC1	<---	ITGB7	-0.063621	0.052044
[4887]	NELL1	--->	GFI1B	0.051749	-0.060266
[4888]	MASP2	<---	FCN2	-0.018669	0.0114
[4889]	RAP1GDS1	<---	NRAS	-0.41126	0.44004
[4890]	DLL1	--->	NOTCH1	-0.0072029	-0.066584
[4891]	PTPN14	--->	CTNNA2	0.18825	-0.19834
[4892]	SFN	--->	NR3C1	0.11204	-0.092332
[4893]	RALA	<---	ARF1	-0.99631	1.0019
[4894]	CDKN1A	--->	STAT3	0.33257	-0.37096
[4895]	YWHAZ	--->	CSF2RB	0.21874	-0.23617
[4896]	ANKS1B	--->	LRP2	0.067037	-0.078009
[4897]	LFNG	--->	NOTCH1	0.98927	-1.0072
[4898]	CSRP1	--->	ACTN1	0.014972	-0.010035
[4899]	RPS6KA5	--->	ATF2	0.40436	-0.41895
[4900]	PDK2	<---	SGK1	-0.15143	0.0090483
[4901]	VDR	--->	GABARAPL1	0.029552	-0.0053218
[4902]	SORBS2	--->	ABL1	-0.098797	-0.22378
[4903]	GRIN1	<---	DLG2	-0.051152	0.045953
[4904]	CSNK2A1	--->	C1R	0.45691	-0.47778
[4905]	AKT1	--->	AR	0.99897	-1.012
[4906]	RANBP10	<---	IGSF1	-0.15034	0.15866
[4907]	TBC1D17	--->	ASCC2	0.028535	0.0049192
[4908]	TSC2	<---	FBXW5	-0.18318	0.18035
[4909]	TGFBR1	--->	WDR33	0.1308	-0.15217
[4910]	AATF	<---	CHEK1	-0.60594	0.58784
[4911]	MMP11	<---	SERPINA1	0.012498	0.025482
[4912]	TRIO	<---	RHOA	-0.034233	0.020587
[4913]	IKZF4	--->	IKZF5	-0.0099063	-0.10207
[4914]	SH3BP2	--->	SH3KBP1	-0.068859	-0.083622
[4915]	LYN	--->	CRKL	0.47236	0.00040577
[4916]	CASR	<---	FLNA	-0.013533	0.030024
[4917]	ELF3	<---	NFKB1	-0.47489	0.47438
[4918]	MAPK1	--->	SMAD2	1.012	-1.0074
[4919]	CCNE1	<---	CDKN1A	-0.99961	0.98767
[4920]	MYC	--->	RBL1	0.073478	-0.085443
[4921]	UBE2I	<---	EP300	-0.1806	0.17626
[4922]	THRB	?	RXRB	-0.0089162	-0.0089162
[4923]	YWHAG	--->	LRCH3	0.0046442	-0.0015785
[4924]	MAPK3	--->	STMN2	1.0058	-0.99021
[4925]	NCF1	<---	RAC1	-0.10303	0.098698
[4926]	NOS1	<---	ADRB2	-0.19182	0.18031
[4927]	PARK2	<---	RGS3	-0.14352	0.14901
[4928]	HOXB9	<---	CREBBP	-0.18943	0.18505
[4929]	CELSR3	<---	SGSM2	-0.016827	0.0053205
[4930]	MALT1	<---	TRAF6	-0.32728	0.31586
[4931]	RARA	--->	NCOA1	0.034596	-0.043163
[4932]	TERF1	--->	GIT1	0.18358	-0.18784
[4933]	EFEMP2	<---	PLSCR4	0.0049686	0.023845
[4934]	PTPN11	--->	PIK3CG	0.1863	-0.14752
[4935]	FHL2	--->	ITGB6	0.079016	-0.085085
[4936]	STK11	--->	TP53	0.74211	-0.72341
[4937]	RBL2	<---	CCNE1	-0.28031	0.26997
[4938]	IL1R1	--->	TOLLIP	0.087649	-0.095211

[4939]	KAT5	--->	BMI1	0.016613	-0.00071569
[4940]	NCK2	<---	NTRK2	-0.041953	0.011969
[4941]	TJP2	<---	CTNNA1	-0.2343	0.23706
[4942]	NKIRAS1	--->	TGFBR1	0.010625	-0.037483
[4943]	CCL21	<---	CCR7	-0.43752	0.45214
[4944]	SPRY2	<---	GRB2	-0.32592	0.33891
[4945]	LIN7C	<---	BAIAP2	0.00089845	0.0030996
[4946]	PLCG1	<---	LCK	-0.3769	0.28213
[4947]	GDF9	<---	BTBD2	-0.050911	0.05607
[4948]	DAXX	<---	USP7	-0.20087	0.17502
[4949]	CAV1	<---	PTPN11	-0.62585	0.80079
[4950]	ATP2B4	<---	PPP3CA	-0.066967	0.054474
[4951]	SAV1	<---	STK4	-0.095877	0.092729
[4952]	CSNK2A2	--->	GTF2A1	0.69606	-0.7619
[4953]	SMAD5	<---	SNRNP70	-0.30655	0.28646
[4954]	CNNM3	--->	RBPMS	1.0145	-1.0036
[4955]	ATXN1	--->	CCNK	0.022522	-0.030122
[4956]	CDK2	--->	FZR1	0.83716	-0.86422
[4957]	BCR	--->	YWHAH	0.078735	-0.083313
[4958]	USP7	--->	TRIM22	0.032903	-0.015511
[4959]	MYLK	--->	CALM1	1.0041	-1.0022
[4960]	CDK11B	<---	PAK1	-1.3014	1.4008
[4961]	TAF1	--->	RBL2	0.10394	-0.11583
[4962]	TRIM63	--->	USP13	0.092161	-0.077235
[4963]	DAZ4	<---	DZIP1	-0.022843	0.05464
[4964]	PIAS4	--->	SETDB1	0.19053	-0.18797
[4965]	HES1	<---	ID1	-0.29914	-0.087238
[4966]	LATS1	<---	ZYX	-0.23946	0.23864
[4967]	NEURL2	--->	CTNNB1	0.54605	-0.5448
[4968]	KCNQ3	?	KCNQ4	-0.025901	-0.025901
[4969]	CDK5	--->	ACTN1	0.25801	-0.27569
[4970]	FRS2	<---	DDR1	-0.5506	0.53468
[4971]	SKP2	--->	CDKN1B	0.072187	-0.068261
[4972]	DVL1	<---	SMAD4	-0.090479	0.09242
[4973]	STAT3	<---	STAT5A	-0.13786	-0.08806
[4974]	CAMK4	--->	RELA	1.0015	-1.0004
[4975]	HSPG2	--->	PRPF40A	0.012765	-0.024766
[4976]	PIK3R1	<---	NFKBIA	-0.058482	-0.014234
[4977]	EPHA1	<---	SMURF2	-0.21599	0.20444
[4978]	PBX1	--->	NR3C1	0.012582	0.012013
[4979]	TRAF6	--->	IRAK1	0.21807	-0.21443
[4980]	PIAS1	--->	TP73	0.068649	-0.068273
[4981]	SLAMF6	<---	PTPN11	-0.54137	0.63132
[4982]	EGFR	--->	CD44	0.073073	-0.083826
[4983]	PKN2	<---	NCK1	-0.062726	0.013011
[4984]	TEAD4	<---	WWTR1	-0.46736	0.46792
[4985]	CSNK1A1	--->	TGFBR1	0.2177	-0.137
[4986]	GRB7	<---	EGFR	-0.42553	0.37299
[4987]	ACAN	<---	FBLN1	-0.030871	0.025213
[4988]	TNNT1	--->	FXR2	0.099236	-0.070138
[4989]	RB1	<---	RUNX2	-0.47187	0.47044
[4990]	KANK1	<---	BAIAP2	-0.22175	0.22437
[4991]	PAK7	--->	PDLIM7	0.053286	0.046322
[4992]	CCNA2	<---	HIRA	-0.54356	0.55627
[4993]	HIF1A	--->	HSP90AA1	0.13772	-0.14878
[4994]	CCL26	<---	CCR3	-0.46475	0.49595
[4995]	TRAF2	<---	TNF	-0.0019203	0.0025552
[4996]	YY1	--->	SREBF1	0.096441	-0.10381
[4997]	PKP2	--->	JUP	-0.016548	-0.031038
[4998]	ERF	<---	CSNK1A1	-0.58262	0.66972
[4999]	HDAC1	<---	BUB1B	-0.42066	0.4
[5000]	TRAF2	--->	MAP3K7IP2	0.12146	-0.12622
[5001]	PPP3CB	--->	NFATC2	0.99656	-0.99518
[5002]	PDGFRB	--->	CRK	0.050426	-0.097965
[5003]	CAMK2A	--->	ITPKA	0.3199	-0.34167
[5004]	ARNT2	<---	HIF1A	-0.0073388	0.30742
[5005]	IKBKB	<---	MAP3K11	-0.16507	0.092342
[5006]	SFN	<---	CDK1	-0.66368	0.62776
[5007]	PTPN6	--->	MYH9	0.061742	-0.058886
[5008]	EGFR	--->	GAPDH	0.16396	-0.17
[5009]	SH3GL3	--->	GABBR2	0.10535	-0.090824
[5010]	GP1BA	--->	F2	0.031979	-0.036279
[5011]	KAT5	--->	PITX2	0.11976	-0.11953
[5012]	CDX2	<---	GSK3B	-0.82765	0.79979
[5013]	SLC24A1	<---	CNGA1	-0.13526	0.13863
[5014]	KIF1B	--->	DLG2	0.046286	-0.055268

[5015]	MAP3K3	--->	YWHAG	0.54958	-0.30237
[5016]	MAPK1	<---	PPP2CA	-0.5252	0.52309
[5017]	SNX2	?	SNX1	-0.040204	-0.040204
[5018]	PLK1	<---	BUB1	0.0029423	0.19171
[5019]	IGFBP7	--->	INS	0.017892	-0.015418
[5020]	ZNF521	<---	GATA1	-0.21566	0.22001
[5021]	ESR1	--->	TSC2	0.15464	-0.15481
[5022]	PDE3B	<---	YWHAB	-0.15524	0.15866
[5023]	PKMYT1	--->	PIN1	0.46953	-0.49485
[5024]	CAV1	--->	GJA3	0.047144	-0.03367
[5025]	PLSCR1	<---	CCDC33	-0.034379	0.068511
[5026]	DUSP7	--->	MAPK1	1.0016	-1.0004
[5027]	NCKIPSD	--->	DIAPH2	0.011713	-0.022252
[5028]	GNB2L1	--->	GRIN2B	0.080475	-0.08198
[5029]	TRADD	<---	FAS	-1.0095	1.0063
[5030]	SMARCD1	--->	FOS	0.18888	-0.2755
[5031]	TGFBR2	<---	TGFB1	-0.99452	0.99798
[5032]	RECK	--->	MMP9	0.25303	-0.23217
[5033]	EP300	--->	TAL1	0.11852	-0.12657
[5034]	ARR3	<---	RHO	-0.12587	0.12815
[5035]	CCBP2	--->	CCL3	0.49595	-0.46475
[5036]	TJP2	?	TJP1	-0.011542	-0.011542
[5037]	ZNF521	<---	SMAD4	-0.03962	0.048771
[5038]	APBB1	--->	APLP1	0.057799	-0.065079
[5039]	PIAS1	--->	ESR1	0.095399	-0.090133
[5040]	CREBBP	--->	RBCK1	0.17662	-0.18099
[5041]	MARK1	--->	MAP2	0.059939	-0.0736
[5042]	GRB10	<---	PDGFRB	-0.11309	0.098098
[5043]	TSHR	--->	GNAI2	0.65587	-0.65379
[5044]	ERBB3	--->	RNF41	0.26577	-0.2663
[5045]	PTPRJ	--->	PLCG1	0.99691	-0.99847
[5046]	CHRNB1	<---	COPS6	-0.19292	0.21856
[5047]	MX1	--->	TRPC4	0.019766	-0.032989
[5048]	PPM1A	<---	AKT1	0.027342	0.060396
[5049]	HNRNPUL1	<---	NXF1	0.0055216	0.0092417
[5050]	SNCAIP	--->	SH2D3C	0.020063	-0.050246
[5051]	KAT5	<---	APBB1	-0.077848	0.081628
[5052]	RNF11	<---	NEDD4L	-0.49056	0.49131
[5053]	PLG	--->	F3	0.43052	-0.43634
[5054]	HNRNPUL1	--->	TP53	0.2208	-0.21938
[5055]	BTRC	--->	CTNNB1	0.99974	-0.99998
[5056]	BATF	--->	JUND	0.36727	-0.73152
[5057]	LYN	--->	FCER1G	0.99791	-0.98519
[5058]	CBS	--->	HTT	0.75146	-0.73943
[5059]	TRIM37	--->	TRAF1	0.072661	-0.014241
[5060]	COL1A2	--->	CD44	0.02036	-0.032408
[5061]	KLRD1	?	KLRC1	-0.090136	-0.090136
[5062]	APBB1	--->	APLP2	0.063813	-0.073499
[5063]	MBL2	--->	CALCR	0.038118	-0.050409
[5064]	MTNR1B	--->	GNB1	0.87375	-0.84712
[5065]	PIAS4	--->	SMAD2	0.9933	-1.0027
[5066]	PRKCG	--->	GJA1	0.22576	-0.23456
[5067]	RTKN	<---	SMURF1	-0.41164	0.40256
[5068]	HCN4	<--->	HCN2	0.047191	0.047191
[5069]	CCNK	<---	RBPMS	-0.30204	0.31068
[5070]	PLCB3	<---	CALM3	-0.28967	0.29322
[5071]	MEF2D	--->	MEF2C	-0.0092469	-0.024058
[5072]	PAK2	--->	SNX9	0.23346	-0.085436
[5073]	CUL5	<---	SMURF1	-0.41748	0.40921
[5074]	ITK	--->	PLCG1	0.99957	-0.98745
[5075]	MRE11A	--->	CCNE1	0.69202	-0.67866
[5076]	NR1I2	--->	NCOA1	0.034596	-0.043163
[5077]	TAOK2	<--->	MAP2K6	0.23429	0.23429
[5078]	NUMB	<---	MDM2	-0.092111	0.084941
[5079]	THBS1	<---	ELANE	-0.30323	0.39805
[5080]	IGF1R	--->	INSR	-0.011388	-0.023675
[5081]	NCOA2	<---	VDR	-0.043163	0.034596
[5082]	MMP12	<---	LPA	-0.11212	0.11927
[5083]	RBM41	<---	CCDC85B	-0.050711	0.077385
[5084]	WNT7A	--->	FZD9	0.99836	-1.0014
[5085]	CDK9	<---	STK36	0.10305	0.15543
[5086]	BMPR1B	--->	VEPH1	-0.0052972	-0.063463
[5087]	CD8A	<---	PTPRC	-0.44433	0.43997
[5088]	PRKCB	--->	BTK	0.99524	-0.99198
[5089]	CDC7	--->	CDKN1A	0.51838	-0.55156
[5090]	NLK	--->	SKP2	0.58677	-0.61295

[5091]	CACNB4	--->	MAP1A	0.012219	0.0035638
[5092]	ATN1	--->	MEGF8	0.037189	-0.039436
[5093]	SOSTDC1	--->	BMP4	0.43337	-0.38277
[5094]	PPP1CB	--->	PRKAR2A	0.12463	-0.13217
[5095]	ITGA2	<---	LAMA1	-0.15237	0.14233
[5096]	GAPDH	--->	SLC2A1	0.21502	-0.20452
[5097]	YWHAH	<---	MST1R	-0.41561	0.39852
[5098]	PPARGC1A	--->	ESRRG	0.013278	-0.021031
[5099]	BRCA1	<---	BRCC3	-0.19112	0.20159
[5100]	CRKL	<--->	CRK	0.036866	0.036866
[5101]	NEK1	--->	TSC2	1.0125	-1.0077
[5102]	SIGLEC10	<---	PTPN6	-0.011511	0.0066768
[5103]	F12	<---	GP1BA	-0.023569	0.015463
[5104]	ITSN1	<---	SOS1	0.0014134	0.13091
[5105]	SOCS1	<---	TIRAP	-0.02063	0.040172
[5106]	PITX1	--->	NEUROD1	0.17074	-0.15942
[5107]	YWHAB	--->	RAI14	0.6652	-0.64224
[5108]	ATN1	--->	CHRD	0.00039499	-0.0080229
[5109]	PPARGC1B	<---	HNF4A	-0.078514	0.069019
[5110]	SMURF2	--->	TXNIP	0.42567	-0.43351
[5111]	RANBP9	--->	TFRC	-0.0026074	-0.0073665
[5112]	MMP1	<---	BSG	-0.33235	0.33113
[5113]	SFRP1	--->	WNT2	0.99043	-0.98922
[5114]	PRKCZ	--->	CASP9	0.2012	-0.21571
[5115]	RAB8A	--->	PQBP1	0.069256	-0.040959
[5116]	TEK	--->	SHC1	-0.00038501	-0.021663
[5117]	MAP4K5	<---	TRAF2	-1.0041	0.99389
[5118]	BRCA1	<---	PPP1CA	-0.12679	0.11715
[5119]	WIPF2	<---	NCK2	-0.2528	0.24626
[5120]	ESR2	<---	AKAP13	-0.033914	0.035086
[5121]	KCNA2	<---	RHOA	-0.39331	0.42382
[5122]	NR4A1	--->	ATXN1	0.0039346	-0.0093575
[5123]	DCN	<---	COL5A1	-0.0049704	0.00097089
[5124]	MAPK1	--->	SP1	0.12886	-0.14656
[5125]	PRPF40A	--->	U2AF2	0.3333	-0.30347
[5126]	GRIN2B	<---	CAMK2B	-0.30134	0.28954
[5127]	SRC	--->	PTPN11	0.2006	0.083854
[5128]	PTPN18	<---	GAB1	-0.1598	0.14817
[5129]	HOMER1	--->	RYR2	0.21835	-0.22732
[5130]	BCR	--->	TP53	0.11154	-0.11951
[5131]	CD300LF	<---	PTPN6	-0.53233	0.60799
[5132]	PTK2	--->	TLN1	-0.010686	-0.085504
[5133]	SOCS3	<---	EGFR	-0.58054	0.56683
[5134]	SH3BP1	<---	CRK	-0.062337	0.045225
[5135]	LYN	--->	LIME1	1.1153	-1.0056
[5136]	CREB1	<---	HPS6	-0.31338	0.32534
[5137]	S1PR5	--->	GNAO1	1.0129	-1.008
[5138]	CNOT1	--->	RXRA	0.0033993	-0.13603
[5139]	RAPGEF5	<---	MRAS	-0.99657	0.99903
[5140]	GPR183	--->	MTA1	0.27342	-0.26732
[5141]	ARR3	<---	PTAFR	-0.25215	0.25543
[5142]	SFRS7	<---	CDK19	-0.75755	0.72994
[5143]	MAPK3	<---	DUSP1	-1.0004	1.0016
[5144]	GRM7	--->	CALM3	0.31782	-0.27991
[5145]	DHX30	<---	NCOR1	-0.04274	0.033531
[5146]	SART3	?	RNPS1	-0.016298	-0.016298
[5147]	CARD11	<---	PDPK1	-0.18928	0.20995
[5148]	GATA4	<---	NR5A1	-0.0060739	0.036935
[5149]	CTNND1	--->	ZBTB33	0.014238	-0.025416
[5150]	PRMT2	--->	PGR	0.12534	-0.11586
[5151]	FGFR1	<---	FRS2	-0.19152	0.18926
[5152]	MELK	--->	CDC25B	0.41934	-0.41468
[5153]	RBBP4	<---	BRCA1	-0.049568	0.055992
[5154]	PARD3	<---	YWHAH	-0.030232	0.031301
[5155]	BRWD1	<---	SMARCA4	-0.0060424	0.0022515
[5156]	TYK2	--->	STAT2	0.3348	-0.3798
[5157]	RNF5	--->	CFTR	0.30545	-0.30978
[5158]	HSP90B1	<---	CSNK2A2	-0.22149	0.20285
[5159]	CAMK2A	--->	CEBPB	0.7187	-0.69952
[5160]	YWHAG	--->	HNRNPM	0.092231	-0.070624
[5161]	CIT	--->	GRIN2D	0.15586	-0.17389
[5162]	SMAD5	<---	SMAD4	-0.9962	1.0013
[5163]	SH2D2A	<---	KDR	-1.0018	1.0006
[5164]	LIN7A	<---	DLG4	-0.012492	-0.0023615
[5165]	PRPF4B	--->	SFRS1	0.72994	-0.75755
[5166]	TRAF2	--->	CALCOCO2	0.14877	-0.1427

```
[5167]  ATN1     --->    FBLN2   0.025006        -0.031741
[5168]  TCL1A    --->    AKT1    0.99137 -0.99692
[5169]  NCOA2    --->    MEF2C   6.7763e-005     -0.012428
[5170]  ETS1     <---    SP100   -0.34351        0.36359
[5171]  CEP72    --->    ZNF417  0.12071 -0.12719
[5172]  CTNNA1   --->    PSEN1   0.49868 -0.47484
[5173]  SMURF2   --->    RNF111  0.38464 -0.39275
[5174]  DAZ1     ?       DAZL    -0.016298       -0.016298
[5175]  PSEN2    --->    FLNA    0.067884        -0.033635
[5176]  TRAIP    --->    TNFRSF1B        0.069363        -0.19225
[5177]  PPP4C    --->    REL     0.052217        -0.064905
[5178]  PRTN3    --->    SERPINB1        0.11833 -0.082185
[5179]  LCP2     --->    VAV3    0.14728 -0.13638
[5180]  RIMS1    --->    CACNA1C 0.16845 -0.18114
[5181]  CD247    <---    CD244   -0.4895 0.47597
[5182]  OPHN1    <---    RHOA    -0.67799        0.70845
[5183]  CYTH3    --->    CALCOCO1        0.28271 -0.25899
[5184]  CRKL     <---    SYK     -0.28174        -0.046839
[5185]  ABCA1    <---    RIMS2   -0.01222        0.0030236
[5186]  RRAS2    --->    SMAD3   0.55322 -0.47159
[5187]  MAPK3    --->    ETS1    1.0002  -0.99981
[5188]  SRGAP2   --->    YWHAG   0.061286        -0.070163
[5189]  FGF9     --->    FGFR2   0.54173 -0.53789
[5190]  SOCS2    --->    GHR     0.031349        -0.038535
[5191]  SORBS2   --->    FLOT1   0.2819  -0.27915
[5192]  SMAD1    <---    TNNT1   -0.38042        0.35869
[5193]  CASP3    --->    PRKCZ   0.19066 -0.19986
[5194]  CSNK2A1  --->    IRS1    0.5492  -0.60947
[5195]  KCNJ5    ?       KCNJ3   -0.01825        -0.01825
[5196]  SPEN     --->    RBBP4   0.033892        -0.028719
[5197]  SMG1     --->    CDH5    0.049273        -0.045014
[5198]  LCK      --->    ITK     0.98538 -0.99553
[5199]  CIT      <---    RHOA    -0.44942        0.42021
[5200]  TINAGL1  --->    LMO4    0.026222        0.0093489
[5201]  CTNND2   <---    PSEN1   -0.065576       0.074964
[5202]  PECAM1   --->    PIK3R1  0.39108 -0.49196
[5203]  CCNE2    <---    CDKN1B  -1.0151 1.0016
[5204]  HSP90AA1         --->    NOS1    0.035548        -0.048954
[5205]  CCR5     --->    GNAI2   1.0129  -1.008
[5206]  PLXNA1   <---    AKT1    -0.30469        0.45163
[5207]  THBS1    <---    CALR    -0.29395        0.38688
[5208]  ITGA4    --->    TGFB1I1 0.0071815       -0.019748
[5209]  PLEC1    --->    SPTAN1  0.035689        -0.043253
[5210]  NCOA2    <---    DLX2    -0.06802        0.06031
[5211]  ZYX      --->    VAV1    0.033881        -0.22364
[5212]  CAV1     <---    RCVRN   -0.25858        0.27087
[5213]  EPHA3    --->    ADAM10  0.01034 -0.023028
[5214]  SRPK1    --->    U2AF2   0.72994 -0.75755
[5215]  CACNA1H  ?       KCNMA1  -0.025901       -0.025901
[5216]  BTK      --->    FAS     0.40657 -0.4142
[5217]  CAMK2A   --->    DLG1    0.13725 -0.15924
[5218]  PAWR     <---    DAPK3   -0.8015 0.77764
[5219]  MARK3    --->    CDC25C  0.070066        -0.081031
[5220]  CDH24    <---    CTNNB1  -0.32695        0.32855
[5221]  YWHAG    --->    ALB     0.18081 -0.15809
[5222]  PRKCA    --->    HMGB1   0.92918 -0.92373
[5223]  PTPRR    --->    MAPK1   0.36571 -0.37559
[5224]  PSMD7    <---    RAB1A   -0.02926        0.042202
[5225]  CASP4    <---    IL1B    -0.084506       0.083786
[5226]  PTPRN    --->    PPIA    1.2981  -1.2897
[5227]  RBBP6    <---    ZFYVE9  -0.11389        0.10626
[5228]  SMAD2    --->    GLI3    0.098419        -0.097905
[5229]  HDAC7    --->    PPARD   0.041345        -0.03076
[5230]  PIAS2    --->    GTF2I   0.13053 -0.11791
[5231]  TGFB1    <---    DCN     -0.99971        1
[5232]  CDK1     --->    BRCA1   0.32242 -0.34057
[5233]  RET      --->    MAPK3   0.20607 -0.24517
[5234]  DEDD     <---    CASP3   -0.21225        -0.18821
[5235]  PIK3C3   --->    CALM1   0.088254        -0.077506
[5236]  NCOR1    --->    HTT     0.060537        -0.066714
[5237]  MAPKAPK5         <---    MAPK4   -0.45114        0.8761
[5238]  PIK3R1   <---    JAK1    -0.10851        0.11209
[5239]  ANKS1A   <---    YWHAG   -0.15511        0.14633
[5240]  LYN      <---    CASP7   -1.0017 1.0113
[5241]  RAD54B   <---    UBE2I   -0.1086 0.1235
[5242]  VCL      --->    TRIP6   0.50132 -0.47625
```

[5243]	RAP1A	<---	BMX	-0.54991		0.47751
[5244]	PSG9	--->	SMAD2	0.33439	-0.29658	
[5245]	DOCK1	<---	CRK	-0.0082083		-0.0062159
[5246]	MTNR1A	--->	GNAI2	1.0129	-1.008	
[5247]	EIF2AK2	--->	ELF2	1.1285	-1.1212	
[5248]	DGKZ	--->	LEPR	0.13941	-0.14783	
[5249]	DVL3	<---	DAB2	-0.37508		0.37541
[5250]	OPRD1	--->	GNA15	1.0129	-1.008	
[5251]	GIPC1	--->	TGFBR3	0.12267	-0.10946	
[5252]	CDK7	--->	ERCC3	0.70011	-0.72596	
[5253]	MARK4	--->	MAP2	0.035402		-0.049174
[5254]	CAPNS1	--->	RAB1A	0.025992		-0.02885
[5255]	ZNF446	--->	ZSCAN16	-0.011024		-0.16454
[5256]	OCLN	--->	SMAD4	0.41186	-0.32882	
[5257]	FOXO3	--->	SMAD2	0.2939	-0.27389	
[5258]	SMURF2	--->	ACOX3	0.43691	-0.44468	
[5259]	TRIM27	<---	TRIM23	-0.092791		0.088114
[5260]	MATN4	?	MATN3	-0.013202		-0.013202
[5261]	PRKCA	--->	ITGB2	0.054379		-0.07038
[5262]	PPP1R8	--->	SETDB1	0.25044	-0.23511	
[5263]	PEA15	<---	FADD	-0.53447		0.38361
[5264]	TTBK1	--->	MAP2	0.071705		-0.080373
[5265]	CDK2	--->	UBTF	0.92033	-0.89929	
[5266]	PTPRD	<---	PTPRA	0.01284	0.035587	
[5267]	ACAP1	<---	IFFO1	-0.096745		0.095812
[5268]	CSNK1E	--->	DVL2	0.99947	-0.9989	
[5269]	CD93	<---	RANBP1	-0.062522		0.06555
[5270]	FZD5	--->	LRP6	0.38615	-0.39454	
[5271]	KSR1	--->	RAF1	1.0003	-0.99555	
[5272]	EPB41L3	<---	MBIP	-0.036138		0.040507
[5273]	CRHR1	<---	GNAI1	-0.43656		0.4334
[5274]	AKAP13	<---	YWHAB	-0.0046727		0.0055202
[5275]	SFN	--->	HNRNPD	0.092231		-0.070624
[5276]	SPTAN1	<---	PRKCB	-0.23377		0.22221
[5277]	TRPC1	<---	HOMER1	-0.099818		0.087824
[5278]	RIPK2	<---	TRAF6	-0.61499		0.59229
[5279]	NEK6	--->	SMAD4	1.012	-1.0074	
[5280]	PTPN13	--->	APC	0.00027571		-0.014168
[5281]	SKAP1	<---	LYN	-0.99957		1.0067
[5282]	ERRFI1	<---	CDC42	-0.14045		0.14285
[5283]	KRAS	--->	CALM1	0.73657	-0.70265	
[5284]	IFNGR1	<---	RAC1	-0.71581		0.72261
[5285]	IGF1R	<---	PRKCD	-0.10134		0.091707
[5286]	GATAD2B	<---	MBD3	-0.014778		0.059037
[5287]	GIT2	<---	PAK3	-0.19118		0.24364
[5288]	FGFR1	<---	FGF1	-0.99871		1.0053
[5289]	SCYL2	--->	GTF3A	0.80449	-0.81037	
[5290]	CDH5	<---	CTNNB1	-0.32695		0.32855
[5291]	EP300	--->	ZBTB16	0.038777		-0.050528
[5292]	GNAQ	--->	PIK3CA	0.49589	-0.49854	
[5293]	PLK3	--->	TP53	0.55158	-0.55633	
[5294]	KAT2B	--->	TP53	0.17897	-0.18472	
[5295]	IL4R	<---	JAK2	-0.16446		0.23344
[5296]	MBL2	--->	CD93	-0.0018666		-0.016999
[5297]	CSF3R	<---	GRB2	-0.29959		0.28703
[5298]	TGFBR2	--->	TSSK1B	0.98829	-0.99836	
[5299]	CASP3	--->	BMX	0.049596		-0.14137
[5300]	GRB10	<---	RAF1	-0.089514		-0.042543
[5301]	SETDB1	<---	DNMT3A	-0.23951		0.25491
[5302]	NCOA2	<---	ACTN2	-0.049643		0.038115
[5303]	DLGAP2	<---	GRK5	-0.56676		0.47996
[5304]	FLNB	--->	ITGB1	0.13605	-0.14529	
[5305]	HTRA1	<---	COL2A1	-0.016442		0.0049615
[5306]	ABL1	<---	CDKN1B	-0.033446		-0.030457
[5307]	HNRNPK	--->	GFI1B	0.14679	-0.15076	
[5308]	PORCN	--->	WNT7B	1.0005	-0.9854	
[5309]	WEE1	<--->	CDK2	0.23429	0.23429	
[5310]	SYT3	--->	SYNCRIP	0.0088762		-0.0036008
[5311]	IL1F7	--->	IL18R1	0.056055		-0.04452
[5312]	ZFAND5	--->	IKBKG	0.33097	-0.33225	
[5313]	PARK2	<---	RAC1	-0.27945		0.31027
[5314]	SPINT2	<---	HGFAC	0.00099252		0.0026828
[5315]	TLR4	--->	RIPK2	0.11087	-0.18459	
[5316]	PRL	--->	PRLR	0.00085557		-0.012375
[5317]	MST1R	--->	PLCG1	0.49669	-0.67031	
[5318]	DEDD2	<--->	DEDD	0.10443	0.10443	

```
[5319]  ITGA3   <---    BIN1    -0.016654       0.0048441
[5320]  KCNJ9   <---    ADRB2   -0.21396        0.22725
[5321]  PKD1    <---    COL2A1  -0.024574       0.014907
[5322]  RANBP2  <---    OPN1LW  -0.12658        0.13007
[5323]  MYB     --->    NCL     0.00085727      -0.012563
[5324]  TP53    <---    BTBD2   -0.12237        0.11759
[5325]  GNA13   <---    TBXA2R  -1.008  1.0129
[5326]  CASP3   --->    SP1     0.032319        -0.072072
[5327]  ITGB4   <---    COL17A1 -0.013406       0.00081241
[5328]  SKP1    --->    NFKBIB  0.47933 -0.46943
[5329]  SERPINA4        <---    KLK1    -0.082185       0.11833
[5330]  CASP1   <---    EGFR    -0.098197       0.085857
[5331]  PDGFRB  --->    GAB1    -0.0020165      -0.010238
[5332]  DRD1    --->    FLNA    0.47769 -0.44658
[5333]  DLG4    <---    YES1    -0.27518        0.41392
[5334]  TLR1    --->    DHX36   0.12206 -0.12004
[5335]  SP1     --->    HTT     0.15013 -0.1602
[5336]  SOX8    <---    CEBPA   -0.057257       0.06251
[5337]  MET     --->    SPSB1   0.21004 -0.22275
[5338]  TGFB1   <---    BMP2    -0.98835        0.99583
[5339]  USF2    ?       USF1    -0.65635        -0.65635
[5340]  SKIL    <---    TTF2    -0.15309        0.14577
[5341]  PAK1    --->    FLNA    0.62467 -0.34479
[5342]  RAF1    --->    MAP2K2  0.9856  -1.0047
[5343]  SHANK1  <---    DLG4    -0.010738       0.0020577
[5344]  POU2F1  --->    STAT5B  0.45894 -0.47646
[5345]  DAB2    <---    PIN1    -0.36029        0.39414
[5346]  PIK3R1  <---    FCGR2A  -0.49196        0.39108
[5347]  E2F4    --->    TFDP1   -0.0088085      -0.064857
[5348]  IL12RB2 <---    IL12RB1 -0.12732        0.033591
[5349]  CCR5    --->    GNAO1   1.0129  -1.008
[5350]  SYK     <---    TLR4    -0.098929       0.037322
[5351]  CXXC5   --->    SMAD5   0.1889  -0.20563
[5352]  CD247   <---    CD48    -0.30375        0.28119
[5353]  CDK9    --->    TP53    1.0002  -0.99962
[5354]  MAPK3   --->    HSPB8   1.0388  -0.99926
[5355]  ATXN1   <---    KLHL8   -0.020717       0.017117
[5356]  ARHGAP17        <---    LCK     -0.47248        0.5611
[5357]  GRB14   <---    INSR    -0.17642        0.14373
[5358]  GSK3B   --->    NOTCH2  0.99811 -1.0133
[5359]  CBX1    <---    DNMT3B  0.0073508       0.026698
[5360]  IL16    --->    EFEMP2  0.042372        -0.004721
[5361]  SPEN    <---    NCOR1   -0.043991       0.034555
[5362]  TLE1    <---    ARL3    -0.56872        0.59393
[5363]  GGA2    <---    ARF1    -0.4338 0.46028
[5364]  ACVR1   --->    CREBBP  0.063595        -0.074824
[5365]  E2F3    --->    CCNA2   0.62054 -0.6085
[5366]  MPP6    <---    LIN7A   -0.020783       0.0077703
[5367]  YWHAB   --->    IGF1R   0.024667        -0.036569
[5368]  CSF1R   <---    FYN     0.27207 0.51153
[5369]  IL1RAP  --->    IL1R2   0.20741 0.11748
[5370]  CSNK2A1 --->    HNRNPC  0.72994 -0.75755
[5371]  BAZ2A   <---    HDAC1   -0.020398       0.017676
[5372]  BLK     --->    FCGR2A  0.11748 0.015127
[5373]  CDK9    --->    CCNT1   1.083   -1.1093
[5374]  DLG2    <---    SCN4A   -0.034928       0.041469
[5375]  ARHGEF12        <---    RHOA    -0.068467       0.066324
[5376]  SLC9A2  <---    SPTAN1  -0.065546       0.055106
[5377]  BMPR2   --->    LMX1A   0.10843 -0.12692
[5378]  RPS6KA1 --->    ETV1    0.39908 -0.61069
[5379]  CSNK2A2 --->    HNRNPC  0.72994 -0.75755
[5380]  SIT1    <---    ZAP70   -0.82307        0.8319
[5381]  ITGB3   <---    FBLN2   -0.024019       0.017182
[5382]  IGF1R   --->    DOK4    0.98642 -1.0008
[5383]  LRP8    <---    GRIN1   -0.036368       0.026174
[5384]  SUFU    <---    ZNF446  -0.11057        0.11939
[5385]  SOCS7   <---    GRB2    -0.11903        0.22866
[5386]  EZR     <---    ADRA1B  -0.13654        0.12943
[5387]  PABPC4  --->    SMAD9   0.28646 -0.30655
[5388]  MAP2    --->    ACTB    0.27936 -0.28243
[5389]  ILK     --->    PPP1R12A        0.081762        -0.072065
[5390]  CDKN1B  --->    CCNA2   1.0016  -1.0151
[5391]  PRKACA  --->    NR3C1   0.99834 -1.0079
[5392]  CEBPB   --->    SPI1    0.11084 -0.052983
[5393]  HIF1A   --->    JUN     0.51421 -0.5187
[5394]  SMAD2   <---    COPS5   -0.99775        0.99487
```

[5395]	CADPS2	<---	DMD	-0.0064966	-0.002678
[5396]	SLIT3	<---	ROBO2	-0.011388	-0.0012333
[5397]	FCGR2A	<---	HCK	0.015127	0.11748
[5398]	PXN	<---	PTPN11	-0.99483	1.0082
[5399]	CLK1	--->	SFRS6	0.45945	-0.481
[5400]	TFEC	--->	TFEB	-0.069637	-0.16359
[5401]	FGFR1	--->	PLCG1	0.21807	-0.28799
[5402]	PCDH8	<---	UBQLN4	-0.088126	0.12226
[5403]	CXXC5	--->	SKIL	0.24954	-0.25255
[5404]	ZHX1	--->	TP53	0.17781	-0.17643
[5405]	ARRB2	<---	SMARCC2	-0.0016756	0.016809
[5406]	EGFR	--->	SLC9A3R1	0.27299	-0.27331
[5407]	DAXX	<---	DNMT1	-0.12799	0.10549
[5408]	ZNF76	<---	EP300	-0.047566	0.035879
[5409]	LPAR3	--->	GNAI2	1.0129	-1.008
[5410]	UPF2	<---	SMG1	-0.01776	0.011604
[5411]	NEDD9	<---	PTPN12	-0.1196	0.10802
[5412]	MYC	<---	HSP90AA1	-0.064334	0.053485
[5413]	CALCOCO2	<---	MYST2	-0.0088567	0.044642
[5414]	TNFRSF21	<---	TRADD	-0.029431	-0.0048665
[5415]	SFRS5	--->	TNK2	0.0057633	-0.01903
[5416]	SOCS1	<---	JAK2	-0.17676	0.23165
[5417]	HDAC1	--->	TGIF1	0.16287	-0.15464
[5418]	CD28	--->	PIK3R2	0.033605	-0.10848
[5419]	AGGF1	<---	TNFSF12	-0.36917	0.40713
[5420]	YWHAB	--->	MLLT4	0.26003	-0.2115
[5421]	POU3F3	<---	SOX11	-0.26954	0.30083
[5422]	PTPN1	--->	IGF1R	0.15684	-0.17158
[5423]	KAT2B	--->	CCND1	0.31199	-0.31771
[5424]	ATF6	<---	SRF	-0.34479	0.39604
[5425]	LAMA1	--->	FBLN2	0.013301	-0.016221
[5426]	SPAG9	<---	MAP3K3	-0.075657	0.12059
[5427]	MTNR1A	<---	HSPA5	-0.36596	0.40505
[5428]	YWHAG	<---	SHPRH	-0.024942	0.024754
[5429]	SNRNP200	<---	YWHAB	-0.035857	0.045156
[5430]	TLR4	--->	TLR1	0.20394	0.048464
[5431]	CDK6	--->	ATXN1	0.18965	-0.2064
[5432]	EZR	--->	CD44	0.016243	-0.025631
[5433]	RECQL5	?	DDX42	-0.0054159	-0.0054159
[5434]	CDK1	--->	RPS6KB1	1.002	-1.0109
[5435]	HSPA4	--->	DDIT4	0.761	-0.71916
[5436]	CREBBP	--->	ATF1	0.1184	-0.12545
[5437]	PRKCA	--->	HSPA1A	0.77163	-0.7673
[5438]	RASGRP4	--->	HRAS	0.2121	-0.20949
[5439]	CHD3	--->	TAL1	0.023244	-0.034629
[5440]	PRKAA1	--->	PFKFB2	0.086531	-0.28854
[5441]	PPP2R1B	--->	RAB7A	0.13801	-0.13281
[5442]	COPS6	--->	PBX2	0.20684	-0.19754
[5443]	RAD23B	--->	ATXN3	0.076162	-0.042104
[5444]	PRKCA	--->	KCNQ2	0.9144	-0.91279
[5445]	FYB	<---	EVL	-0.039177	0.031117
[5446]	TGFBR1	--->	DCAF12	0.3741	-0.40828
[5447]	UBE2I	--->	JUN	0.57871	-0.6243
[5448]	PTPN6	--->	CSF2RB	0.99989	-1.0072
[5449]	FGFR3	<---	FGF4	-0.61087	0.60861
[5450]	MLXIP	--->	MLX	-0.39659	-0.4284
[5451]	PRKCG	--->	TOP2A	0.89006	-0.88857
[5452]	AFAP1	<---	PRKCD	-1.0023	1.033
[5453]	BMPR2	<---	MOS	-0.14114	0.30588
[5454]	SMAD4	<---	TGFBR2	-0.57747	0.59933
[5455]	IRAK1	<---	HRAS	-0.13201	0.22339
[5456]	MEIS1	<---	HOXB4	-0.0066279	0.010455
[5457]	SUV39H1	--->	RBL2	0.096044	-0.10743
[5458]	HSP90B1	--->	ASGR1	0.063236	-0.069118
[5459]	ARRB2	--->	NFKBIA	0.48867	-0.49665
[5460]	PPP2CA	--->	TSC2	0.12037	-0.13109
[5461]	CHD3	--->	FUBP1	-0.0029599	-0.007045
[5462]	DLST	--->	RUSC2	0.087293	-0.053588
[5463]	KPNA2	--->	EPB41	0.016397	-0.012551
[5464]	AKT1	--->	S1PR1	0.25532	-0.17208
[5465]	NCK1	--->	PLCG1	0.29725	-0.46696
[5466]	CABLES1	<---	CDK5	-1.1093	1.083
[5467]	OCRL	<---	RAB8A	-0.25397	0.25628
[5468]	C1QB	<--->	C1QA	0.0041468	0.0041468
[5469]	TRAF7	<---	MAP3K3	-0.036391	0.080908
[5470]	NR0B1	--->	RORA	-0.0089663	-0.044425

[5471]	TRPC1	<---	TRPC5	-0.0093674	-0.0093671
[5472]	PPP2R1A	--->	RAB11A	0.13801	-0.13281
[5473]	GATA1	<---	CASP3	-0.7885	0.68257
[5474]	HDAC2	--->	RBBP4	0.052829	-0.046452
[5475]	CDK5RAP2	<---	STK36	-0.46877	0.44716
[5476]	ADCY5	<---	PRKCZ	-0.20392	0.19013
[5477]	CBLN3	<--->	CBLN1	0.012763	0.012763
[5478]	HSPA1B	--->	PGAM5	0.71825	-0.69624
[5479]	SHC1	<---	LYN	-0.65926	0.73568
[5480]	PRPF40A	--->	WASL	0.17651	-0.2061
[5481]	PPP5C	<---	HSP90AA1	-0.017944	0.0061088
[5482]	VWF	<---	COL1A2	-0.047696	0.038126
[5483]	SPI1	--->	ERG	-0.088177	-0.24955
[5484]	RUNX2	--->	DVL2	0.3796	-0.37946
[5485]	HIF1A	--->	MTA1	0.8482	-0.84583
[5486]	MMP14	<---	TIMP3	-0.19321	0.21399
[5487]	VAV1	<---	CDC42	-0.22722	0.041359
[5488]	EIF3H	--->	CCDC85B	0.19324	-0.17187
[5489]	JUP	--->	CDH15	0.07062	-0.077057
[5490]	DNM2	<---	PTK2	-0.079046	0.041247
[5491]	MST1R	<---	SRC	-0.17507	0.7585
[5492]	TRAF2	--->	DVL2	0.10019	-0.10979
[5493]	SMAD3	<---	MECOM	-0.30165	0.32615
[5494]	MOBKL1B	<---	STRN3	-0.017885	0.020544
[5495]	YWHAG	--->	FOXO1	0.080297	-0.089786
[5496]	BMPR1A	<---	BMPR2	-0.18789	-0.093581
[5497]	PDGFB	--->	HNRNPC	0.046416	-0.032467
[5498]	AR	<---	FLNA	-0.95414	0.95355
[5499]	PLK1	--->	PIN1	0.13974	-0.1596
[5500]	GNAO1	<---	ADRA2A	-0.98627	0.9872
[5501]	PLAU	--->	SERPINB2	0.057207	-0.040728
[5502]	CAMK1	--->	CALM1	1.007	-1.0109
[5503]	PCSK7	--->	HNRNPD	0.015398	-0.0017254
[5504]	PTPN2	--->	STAT1	-0.010277	-0.080675
[5505]	CDON	--->	CDH15	0.16197	-0.17057
[5506]	MBD2	<---	DHX9	-0.052545	0.069048
[5507]	GNAI2	<---	EGFR	-0.24006	0.235
[5508]	CD44	<---	ARHGEF12	-0.021071	0.010038
[5509]	DVL3	<---	PPM1A	-0.33505	0.33032
[5510]	NRXN1	<---	APBA2	-0.037128	0.041981
[5511]	MPDZ	<---	CSPG4	0.0095843	0.027225
[5512]	IRS1	<---	ALK	-0.60416	0.55733
[5513]	PDPK1	<---	MTOR	-0.19124	0.18488
[5514]	TSC2	--->	RAB5A	1.0033	-0.99668
[5515]	CRELD1	<---	EEF1G	0.011446	0.024505
[5516]	GABPB1	<---	ATF1	0.0072015	0.042797
[5517]	GRAP	<---	KIT	-0.5027	0.10598
[5518]	DIP2A	--->	DLX2	0.082349	-0.053756
[5519]	SNX9	--->	MPP6	0.070199	-0.064992
[5520]	AR	<---	CALR	-0.95188	0.95058
[5521]	RNF41	<---	ERBB4	-0.1627	0.15872
[5522]	IRS4	<---	PRKCZ	-0.14329	0.13541
[5523]	DEDD	<---	FADD	-0.53447	0.38361
[5524]	MAST3	--->	PTEN	0.61732	-0.61289
[5525]	AKT1	--->	NCF1	0.13449	-0.097497
[5526]	ADAMTS4	<---	FURIN	-0.0067844	-0.00093443
[5527]	EFEMP2	--->	CCNK	0.22053	-0.205
[5528]	BRCA1	--->	CCNA1	0.40227	-0.39925
[5529]	IGF1R	<---	MDM2	-0.02215	0.0088875
[5530]	NR2E3	?	RXRB	-0.0089162	-0.0089162
[5531]	APBB1	--->	LRP1	0.093776	-0.10482
[5532]	CSNK2A1	--->	MYF5	1.5563	-1.5271
[5533]	RAB11B	--->	RAB11FIP5	0.12837	-0.10724
[5534]	NEUROD1	<---	CALM3	-0.22666	0.2676
[5535]	RXRB	?	RARA	-0.0089162	-0.0089162
[5536]	UBE2I	--->	DAXX	0.24731	-0.2755
[5537]	TNFRSF10C	<---	TNFSF10	-0.42882	0.44704
[5538]	XAB2	--->	SMAD9	0.28307	-0.30126
[5539]	ELK4	--->	FOS	0.13423	-0.21537
[5540]	AKT1	--->	ATXN1	0.12082	-0.098954
[5541]	L1CAM	--->	ITGAV	0.020574	-0.034164
[5542]	HDAC2	<---	CTBP1	-0.031773	0.022723
[5543]	HIPK2	--->	TRADD	0.14651	-0.3945
[5544]	PTPN11	--->	CSF3R	0.73024	-0.64998
[5545]	KHDRBS1	<---	LCK	-0.39443	0.41674
[5546]	SOCS3	--->	GHR	0.031349	-0.038535

[5547]	STK11	--->	SNRK	0.6413	-0.21779
[5548]	NTRK3	--->	MAPK1	0.0093651	-0.019271
[5549]	FADD	--->	MOBKL3	0.10558	-0.19204
[5550]	GSC	<---	SMAD2	-0.14259	0.20304
[5551]	SMURF2	--->	SMAD3	0.9934	-1.0067
[5552]	ROBO2	--->	PPP1R1B	0.15043	-0.14612
[5553]	STAT1	--->	FADD	0.08173	-0.17023
[5554]	SHARPIN	--->	SHANK1	0.023075	-0.03271
[5555]	APP	<---	FLOT1	-0.36828	0.35837
[5556]	VEGFC	<---	KDR	-0.28835	0.34247
[5557]	GEM	--->	YWHAB	0.32694	-0.30645
[5558]	INHBE	<---	FST	-0.99524	1.0085
[5559]	NOTCH4	<---	PSEN2	-0.98992	0.99563
[5560]	CXADR	--->	LNX1	0.14289	-0.14942
[5561]	MTA1	<---	ESR1	-0.0060739	0.036935
[5562]	PTGFR	<--->	PTGER1	0.49511	0.49511
[5563]	PIM1	<---	EPHA3	-0.19867	0.18166
[5564]	CD36	<---	LYN	-0.94926	1.109
[5565]	FGFR1	<---	NCK2	-0.077069	0.04677
[5566]	NSD1	--->	RARA	0.12772	-0.10069
[5567]	VAV3	<---	ROS1	-0.25875	0.16231
[5568]	PPFIA1	--->	GIT1	0.16054	-0.16385
[5569]	HEYL	<---	HAND1	-0.43036	-0.38951
[5570]	SERPINB13	<---	CTSG	-0.082185	0.11833
[5571]	RFX3	<---	TRAF2	-0.13965	0.14539
[5572]	NFASC	<---	NRCAM	0.0078499	0.010648
[5573]	POU3F2	--->	SOX10	0.015013	-0.022372
[5574]	PTK2	<---	TRIO	-0.0222	-0.008627
[5575]	IRS1	--->	GHR	0.014133	-0.019603
[5576]	CABLES1	<---	ABL1	-0.097172	-0.052501
[5577]	ATOH1	<---	HMGB1	-0.81406	0.8544
[5578]	TRAF6	--->	SQSTM1	0.39913	-0.4115
[5579]	TLR4	--->	IRAK2	0.13945	-0.15459
[5580]	GNAI3	<---	HTR1A	-1.008	1.0129
[5581]	BIN1	--->	MYC	0.20031	-0.19987
[5582]	PIGR	--->	CD79A	0.013923	-0.18476
[5583]	CPNE1	<---	UBE2O	-0.12637	0.16628
[5584]	UBE2D3	<---	UBE3A	-0.048727	0.045324
[5585]	SVIL	<---	BUB1	-0.65542	0.74546
[5586]	PTPN13	--->	YWHAZ	0.15464	-0.16592
[5587]	GRIN1	<---	MAP2	-0.083474	0.082677
[5588]	SP1	--->	NOS3	0.11682	-0.13018
[5589]	FCER1A	--->	FCER1G	0.47597	-0.4895
[5590]	IQGAP1	--->	CTNNB1	0.32758	-0.33225
[5591]	KCNJ11	?	KCNJ2	-0.01825	-0.01825
[5592]	CBL	--->	MET	0.51829	-0.53068
[5593]	IFNGR2	<---	NR3C1	-0.40393	0.40455
[5594]	APBB1	--->	VASP	0.13617	-0.13165
[5595]	TGFBRAP1	<---	TGFBR2	-0.8182	0.81086
[5596]	PACSIN1	--->	WASF2	0.11644	-0.11432
[5597]	SNX6	<---	EGFR	-0.32869	0.32794
[5598]	GRID2	<---	PTPN4	-0.12674	0.11501
[5599]	RNF2	--->	ABCB1	0.11571	-0.098461
[5600]	FYN	--->	CD44	0.26037	-0.23486
[5601]	YWHAZ	<---	GP1BA	-0.036526	0.036242
[5602]	PEA15	<---	MAPK1	-0.53527	0.57162
[5603]	TWIST1	<---	RELA	-0.01356	0.007141
[5604]	PELI2	<---	MYD88	-0.25688	0.23378
[5605]	STAT1	--->	DUSP3	0.11857	-0.24301
[5606]	EPS8	<---	SRC	-0.99797	0.99669
[5607]	CSNK2A1	--->	HDAC3	0.68838	-0.71414
[5608]	MBD1	--->	SMAD5	0.15567	-0.17171
[5609]	PTPN12	--->	JAK2	0.99878	-0.99232
[5610]	SNTA1	--->	SCN5A	0.050181	-0.013537
[5611]	SCN5A	<---	SNTB1	-0.14312	0.15288
[5612]	GP1BA	--->	CTSG	0.052885	-0.050941
[5613]	SGK1	--->	CREB1	1.0082	-0.98976
[5614]	SLC9A2	<---	ABL1	-0.045316	0.0018151
[5615]	CEBPB	<---	EGFR	-0.29925	0.30186
[5616]	BMP1	<---	COL1A1	-0.025745	0.012412
[5617]	TP63	<---	GRB2	-0.15294	0.20846
[5618]	MBL2	--->	MASP2	0.017757	-0.028918
[5619]	NCOA2	<---	CDK4	-0.31801	0.30243
[5620]	SPDEF	--->	AR	0.56448	-0.57006
[5621]	RAF1	<---	TBXA2R	-0.15563	0.12892
[5622]	UBE2I	<---	PHC1	-0.012883	0.029981

[5623]	CAMTA2	--->	NKX2-5	0.12796	-0.095445
[5624]	NEDD9	<---	LYN	-0.24387	0.43979
[5625]	PICK1	--->	GRIK2	0.11658	-0.098237
[5626]	ERBB2IP	<---	ZFYVE9	-0.041765	0.03163
[5627]	MYO7A	--->	CALM1	0.064234	-0.059058
[5628]	SH2B2	<---	FYN	-0.89003	0.98544
[5629]	REM1	--->	YWHAQ	0.32694	-0.30645
[5630]	SHC1	<---	CD247	-0.1376	0.024934
[5631]	WWP1	--->	KLF2	0.20646	-0.2172
[5632]	ANKRD1	<---	MYPN	-0.65168	0.68126
[5633]	ITGAV	<---	ITGB3	-0.01057	-0.0026958
[5634]	ZBTB16	<---	RARA	-0.059674	0.055651
[5635]	NR2C2	<---	HNF4A	-0.087134	0.20637
[5636]	TNFRSF14	--->	TRAF5	0.19415	-0.20124
[5637]	NKRF	<---	NFKB2	-0.029463	0.011311
[5638]	NTRK2	<---	FYN	0.010748	0.23394
[5639]	FAS	<---	PRKCA	-0.65815	0.65297
[5640]	RNF11	--->	GGA1	0.19846	-0.16573
[5641]	SMARCD1	--->	JUN	0.28391	-0.31689
[5642]	NR5A1	<---	NFKB2	-0.020746	0.0043561
[5643]	DKK1	--->	LRP5	0.61034	-0.60665
[5644]	ITGAV	<---	CALR	-0.024533	0.012424
[5645]	SRC	--->	CD33	0.74656	-0.70289
[5646]	MAGI1	<---	RPS6KA2	-0.073246	0.032672
[5647]	MAPK8IP3	<---	MAPK8	-0.16592	0.14982
[5648]	NEDD9	<---	BCAR1	-0.0069242	-0.0042734
[5649]	RAF1	--->	SPRY2	0.037199	-0.062206
[5650]	MAPK8	--->	JDP2	0.97462	-1.029
[5651]	SHANK1	<---	RPS6KA2	-0.071398	0.030611
[5652]	SCARF2	--->	SCARF1	-0.0069773	-0.0069791
[5653]	SRI	--->	CALCOCO2	0.28253	-0.26543
[5654]	CHD3	--->	KPNA2	-0.0024559	-0.0076929
[5655]	PLCG1	<---	BLNK	-0.52589	0.35939
[5656]	NCOA1	<---	BCL3	-0.033054	0.023538
[5657]	RXRA	<---	RARG	-0.99549	1.003
[5658]	PRKDC	<---	HMGB2	-0.071357	0.075255
[5659]	CSNK2A1	--->	RELA	1.0015	-1.0004
[5660]	EPHA4	<---	FGFR2	-0.048678	-0.034567
[5661]	TIMP2	--->	ITGB1	0.13807	-0.14732
[5662]	CDX2	<---	MAPK1	-0.82765	0.79979
[5663]	TRIM37	--->	APEX2	0.091528	-0.054382
[5664]	WASF2	<---	CDC42	-0.3163	0.32786
[5665]	DHX37	<---	PLXNA2	0.0014649	0.014183
[5666]	PTPN11	--->	FRS2	0.90858	-0.72183
[5667]	GNA13	--->	CDH1	0.20416	-0.20669
[5668]	SFRS17A	--->	SFRS1	0.030608	0.00099173
[5669]	RHOJ	<--->	CDC42	0.43466	0.43466
[5670]	PRKCA	--->	ATP2B1	0.14677	-0.15808
[5671]	SMAD2	<---	TBC1D1	-0.011985	0.0052988
[5672]	JAG2	--->	NOTCH2	-0.0068496	-0.014162
[5673]	MAP3K5	<---	TRAF2	-1.007	0.9969
[5674]	OBSCN	--->	ANK1	0.025414	-0.056867
[5675]	SSH3	--->	ACTB	0.63171	-0.63329
[5676]	TRAF3	<---	TBK1	-0.045413	0.03719
[5677]	MAPK1	<---	PTPN7	-1.009	1.0061
[5678]	PLXNA1	<---	RHOD	-0.37902	0.41051
[5679]	MAPK3	--->	INSR	0.22073	-0.23473
[5680]	ADAM15	<---	NCK1	-0.18624	0.18318
[5681]	FYN	<---	ACP1	0.010724	0.13856
[5682]	FHL3	--->	ITGA7	0.0067069	-0.017023
[5683]	CAMTA2	--->	HDAC5	0.050217	-0.043169
[5684]	KCND3	<---	KCNIP2	-0.26746	0.28353
[5685]	MST1R	--->	RELA	0.14335	-0.15133
[5686]	EVL	--->	WAS	-0.019182	-0.1162
[5687]	SPARC	<---	COL1A2	-0.06613	0.059945
[5688]	NCK2	--->	HNRNPK	0.24919	-0.25351
[5689]	DVL1	<---	SMAD7	-0.090479	0.09242
[5690]	ZBTB25	<---	TRAF2	-0.098503	0.096422
[5691]	MEP1B	<---	MEP1A	0.045249	0.051688
[5692]	CRK	<---	INSR	-0.077994	0.057748
[5693]	SRGAP1	--->	ROBO1	0.028311	-0.029679
[5694]	SMAD3	--->	TFE3	0.42594	-0.43382
[5695]	TLR2	--->	HMGB1	0.39113	-0.38393
[5696]	ENAH	--->	FYB	-0.0023229	-0.010822
[5697]	RAB37	<--->	RAB5A	0.43466	0.43466
[5698]	TNKS2	<--->	TNKS	0.0072404	0.0072404

[5699]	EPHA2	--->	GRB2	0.18974	-0.30775
[5700]	SAFB2	--->	ESR1	0.064959	-0.040633
[5701]	SAMD4A	--->	YWHAG	-0.0058122	-0.0070023
[5702]	ESRRA	--->	NCOA1	0.034596	-0.043163
[5703]	PLCG1	<---	SELE	-0.40835	0.36046
[5704]	KLF6	--->	TP53	0.024453	-0.03013
[5705]	SGK1	--->	BRAF	0.42615	-0.56641
[5706]	TNFRSF10C	--->	ZHX1	0.1095	-0.098808
[5707]	SPINT2	<---	ITIH3	0.0087426	0.025264
[5708]	SMAD2	<---	HUWE1	-0.17227	0.16987
[5709]	CDKN1B	<---	CDK4	-0.36687	0.34497
[5710]	PTK2	<---	FLT1	-0.40503	0.36159
[5711]	MINK1	--->	YWHAZ	0.066249	-0.065987
[5712]	MST1R	--->	SFN	0.39852	-0.41561
[5713]	CCKBR	<--->	CCKAR	0.016768	0.016768
[5714]	PILRA	<---	PTPN6	-0.52299	0.60343
[5715]	ETS1	<---	SRC	-0.59343	0.65791
[5716]	UBE2I	--->	ZNF646	0.21809	-0.20997
[5717]	OCRL	<---	RAB14	-0.25397	0.25628
[5718]	MLN	--->	MLNR	1.0107	-0.98958
[5719]	ADORA2B	--->	NTN1	0.027282	-0.034241
[5720]	CAPN1	--->	COL1A1	0.024459	-0.036581
[5721]	TRADD	<---	DAPK1	-0.0095403	0.01939
[5722]	CXCR4	<---	PTPRC	-0.31825	0.32024
[5723]	CASP8	--->	EDA2R	0.57343	-0.71763
[5724]	PTPRN	--->	SNX19	0.79417	-0.7956
[5725]	SMAD1	<---	TTF1	-0.31391	0.29525
[5726]	PPARGC1A	--->	RXRA	0.0053256	-0.064657
[5727]	CAV3	--->	SLC8A1	0.057912	-0.046008
[5728]	CBLN3	<--->	CBLN4	0.012763	0.012763
[5729]	IGFBP5	<---	PNO1	-0.0039803	0.037996
[5730]	EPHB6	<---	EPHB1	0.0074393	0.036221
[5731]	NPPB	--->	NPR3	0.041351	-0.0068276
[5732]	MTNR1B	--->	FLNA	0.47769	-0.44658
[5733]	VEGFA	<---	AKT1	-0.10793	0.15303
[5734]	CCR5	--->	CCL11	0.49595	-0.46475
[5735]	HIST2H2AC	<---	RBBP4	-0.059328	0.088873
[5736]	CTRB1	--->	SERPINA3	0.11833	-0.082185
[5737]	CCNL2	<---	CDK11B	-1.0774	1.0544
[5738]	ELK4	<---	MAPK7	-1.0045	0.99134
[5739]	CD101	--->	PUF60	0.28624	-0.31803
[5740]	ANK3	--->	FADD	0.14908	-0.13152
[5741]	STAT3	<---	CSF3R	-0.34335	0.30331
[5742]	NXF1	<---	UBQLN4	-0.029973	0.063368
[5743]	ATF5	<---	GABBR1	-0.23122	0.26486
[5744]	MMP2	<---	HAPLN1	-0.093464	0.097177
[5745]	EP300	--->	KLF5	0.11387	-0.12188
[5746]	CBX4	<---	CTBP2	-0.061494	0.055286
[5747]	ESR1	<---	SP1	-0.11174	0.10251
[5748]	PIK3CA	<---	GRIN2B	-0.096052	0.085454
[5749]	YWHAB	--->	RNPS1	0.092231	-0.070624
[5750]	TRAF6	--->	NTRK2	0.16014	-0.17094
[5751]	PTK2B	<---	PRKCD	-0.287	0.28882
[5752]	TNR	--->	BCAN	-0.0018788	-0.011468
[5753]	AKT1	--->	MAP2K4	1.0168	-1.0071
[5754]	BRSK2	--->	CDC25B	0.41582	-0.41122
[5755]	KSR1	--->	YWHAZ	0.2559	-0.1402
[5756]	CDK4	--->	SMAD2	1.012	-1.0074
[5757]	IL1RAP	--->	RAC1	0.26158	-0.25226
[5758]	PDGFRA	--->	STAT5B	0.060186	-0.073579
[5759]	HSP90AA1	--->	WASL	0.021064	-0.03719
[5760]	THBS2	<---	MMP9	-0.15886	0.21165
[5761]	E2F3	--->	RB1	0.47244	-0.47441
[5762]	MAPK3	--->	CCDC6	0.71754	-0.7435
[5763]	KSR1	--->	EPHB2	0.15104	-0.060701
[5764]	PRKCD	--->	HNRNPK	0.78324	-0.77356
[5765]	CASP10	<---	TNFRSF1A	-0.10402	0.043232
[5766]	MAP3K7	--->	SMAD3	0.23582	-0.21256
[5767]	MAPK1	--->	ESR1	0.99834	-1.0079
[5768]	SYK	--->	SH3BP2	1.004	-0.99817
[5769]	KCNA2	--->	ETV1	0.19396	-0.15183
[5770]	NCOA3	<---	NR4A1	-0.13412	0.12213
[5771]	RIN2	<---	RAB5A	-0.99801	1.0021
[5772]	ERBB3	--->	PIK3R1	0.2254	-0.29745
[5773]	FGFR3	<---	ARAP1	-0.022766	0.0091355
[5774]	NCOA3	<---	NR1I2	-0.13412	0.12213

[5775]	LRRC7	<---	ACTN2	-0.0077947	-0.0021541
[5776]	CIB1	<---	RAC3	-0.70265	0.73657
[5777]	ENAH	<---	DNMBP	-0.011955	-0.0022814
[5778]	GRM5	<---	FLNA	0.0058519	0.028788
[5779]	SKP1	--->	FBXO3	0.06478	-0.063478
[5780]	ICAM3	--->	EZR	0.029789	-0.038721
[5781]	HIRIP3	--->	HIST2H2BE	0.23103	-0.17959
[5782]	REL	<---	CALM1	-0.16227	0.15281
[5783]	VAV2	<---	RHOA	-0.14624	0.08839
[5784]	CDC42EP4	<---	RHOQ	-0.87115	0.57849
[5785]	PXN	<---	CEACAM1	-0.11527	0.10727
[5786]	MMP7	<---	FASLG	-0.42626	0.44952
[5787]	TRIB3	--->	EEF1G	0.71212	-0.73693
[5788]	PIAS4	--->	BARD1	0.15512	-0.15134
[5789]	CALR	--->	APP	0.35937	-0.36974
[5790]	ANKS4B	<---	USH1C	-0.22426	0.24286
[5791]	FGB	<--->	FGA	0.0011944	0.0011944
[5792]	GNAI2	<---	DRD2	-1.008	1.0129
[5793]	LAMC1	--->	NID1	0.0083042	-0.018071
[5794]	KLRK1	<---	RAET1G	-0.0029242	0.054213
[5795]	CASP1	<---	PSEN2	-0.048154	0.056436
[5796]	SOCS3	<---	JAK2	-0.17676	0.23165
[5797]	ETS1	<---	MEIS1	-0.095271	0.10307
[5798]	GNA12	<---	TEC	-0.68342	0.63988
[5799]	ANKRD6	--->	AXIN2	0.092329	-0.099233
[5800]	SOS1	--->	RRAS	0.99534	-0.99973
[5801]	PLEK	<---	TGFBR1	-0.19598	0.1542
[5802]	GNAQ	<---	BDKRB1	-1.008	1.0129
[5803]	DAPK2	--->	TGFBR1	0.36359	0.02614
[5804]	PDGFRA	<---	ITGB3	-0.028658	0.018014
[5805]	IL10RA	<---	IL10	-0.5028	0.5324
[5806]	ITGB1	<---	HSPG2	-0.022166	0.0084493
[5807]	DNMT3B	--->	SUV39H1	0.051582	-0.044259
[5808]	FGFR4	<---	CDH2	-0.063205	0.054283
[5809]	SMURF2	--->	DAB2	0.71632	-0.71997
[5810]	DVL2	<---	SORBS3	-0.26543	0.25823
[5811]	WNT7A	<---	STRN4	-0.36252	0.38585
[5812]	PRKCA	--->	PTPN12	0.27145	-0.27861
[5813]	MFNG	--->	DLL1	0.99944	-0.99547
[5814]	TRAT1	--->	CD3E	0.052256	-0.053704
[5815]	OCRL	<---	RAB1A	-0.25397	0.25628
[5816]	ZEB2	<---	COPS6	-0.3461	0.35467
[5817]	NFKB2	<---	MAP3K8	-0.092566	0.07198
[5818]	PAK2	--->	SH3GL2	0.20445	-0.042153
[5819]	RGS12	--->	GNAI1	-0.00079669	-0.0029535
[5820]	ACVR2B	--->	SNX1	0.23826	-0.2683
[5821]	RBMX	?	CIRBP	-0.016298	-0.016298
[5822]	EP300	--->	HOXB4	0.10958	-0.11773
[5823]	POU5F1	--->	AR	0.94974	-0.95044
[5824]	AGTR2	<---	ERBB3	-0.17809	0.17651
[5825]	SP1	--->	PRKDC	0.051675	-0.064093
[5826]	CPAMD8	--->	SCTR	0.39886	-0.39515
[5827]	JPH3	--->	SMAD3	0.48133	-0.39835
[5828]	HCK	--->	DOK2	1.3209	-1.1853
[5829]	PIK3R2	<---	TGFBR1	-0.16383	0.12279
[5830]	GNAI2	<---	ADRA2B	-0.98627	0.9872
[5831]	GGCX	--->	PROC	0.1313	-0.11522
[5832]	CBX5	<---	CHAF1A	0.00039271	0.0033443
[5833]	PRKCA	--->	PLCB1	0.26658	-0.27591
[5834]	MAPK1	--->	ETS1	1.0002	-0.99981
[5835]	PFN2	<---	PTPRS	-0.29496	0.28936
[5836]	KCNJ12	<---	SNTB2	-0.0070376	0.044422
[5837]	INPPL1	--->	SORBS3	-0.016723	-0.025522
[5838]	OPTN	--->	FOS	0.27918	-0.4139
[5839]	PHOX2A	<---	PRKACA	-0.82765	0.79979
[5840]	NLK	--->	FBXW5	0.36141	-0.38391
[5841]	DUSP7	--->	MAPK8	1.0016	-1.0004
[5842]	AKT1	--->	PPARGC1B	0.1037	-0.081786
[5843]	HDAC1	<---	FOXG1	-0.15732	0.17107
[5844]	CXCR2	--->	ST13	0.45456	-0.42361
[5845]	BTRC	--->	CDC25A	0.33885	-0.34518
[5846]	MAPK8	--->	REL	0.60973	-0.61546
[5847]	PAK1	<---	NCK1	-0.47107	0.51692
[5848]	BAG1	<---	PDGFRB	-0.051074	0.044641
[5849]	ZFYVE9	--->	SMAD2	0.99525	-0.99685
[5850]	HDAC1	--->	MYOD1	0.38449	-0.36669

[5851]	ESR2	--->	CALM3	0.15612	-0.12827
[5852]	IL12RB2	--->	STAT4	0.079189	-0.10192
[5853]	UBTF	--->	IRS1	0.024365	-0.013168
[5854]	PRKCB	--->	OCLN	0.94019	-0.93859
[5855]	TTRAP	<---	UBE2I	-0.17742	0.21073
[5856]	TRPC3	<---	PLCB2	-0.016685	0.003491
[5857]	SLC9A3R2	<---	PRKACA	-0.64226	0.62013
[5858]	KLF6	<---	GTF3C1	-0.2777	0.27852
[5859]	SMAD3	<---	TGFBR1	-1.0087	1.0115
[5860]	SMAD7	<---	TTF2	-0.12551	0.1472
[5861]	GJC1	--->	TJP1	0.011892	-0.012642
[5862]	IGFBP7	--->	CCL21	0.050367	-0.047815
[5863]	AKT1	--->	PAK1	0.41901	-0.44285
[5864]	PRELP	<---	COL2A1	-0.0059794	-0.0044767
[5865]	BLK	--->	CD79B	1.0799	-1.0399
[5866]	CDK5RAP2	<---	SENP2	0.0099228	0.026329
[5867]	USP8	<---	GRB2	-0.25594	0.27045
[5868]	RGS19	--->	GNAI3	1.0012	-1.0015
[5869]	FGFR3	<---	FGF1	-0.61087	0.60861
[5870]	FLNA	<---	DRD2	-0.44658	0.47769
[5871]	ASH2L	<---	SMURF2	-0.7089	0.70504
[5872]	APBB1	--->	LRP2	0.26643	-0.27254
[5873]	SETDB1	<---	HDAC2	-0.15393	0.15797
[5874]	DRD1	<--->	ADORA1	0.49511	0.49511
[5875]	PEA15	--->	CASP8	0.54362	-0.56644
[5876]	PPP2CA	--->	RACGAP1	0.15866	-0.16819
[5877]	KIAA0513	<---	INTS4	-0.0078902	0.042914
[5878]	FSD1	--->	PPP1R16A	0.33967	-0.3283
[5879]	CHEK1	<--->	TLK1	0.23429	0.23429
[5880]	REG3A	?	REG1A	-0.090136	-0.090136
[5881]	MAPK14	<---	MAP3K7	-1.0033	0.99812
[5882]	TNIP2	<---	SMARCD1	-0.038574	0.053793
[5883]	NR0B2	--->	PPARG	-0.0084242	-0.02056
[5884]	HNRNPD	<---	LDHA	-0.00031078	0.014007
[5885]	MAPT	<---	PSEN1	-0.24866	0.2685
[5886]	MMP2	<---	A2M	-0.11161	0.132
[5887]	TYK2	<---	PTPN6	-0.26938	0.35779
[5888]	FES	--->	STAT3	0.29709	-0.39447
[5889]	IL16	--->	GRIN2B	0.11324	-0.11391
[5890]	AATF	--->	ATM	0.12835	-0.13995
[5891]	CAV3	<---	EGFR	-0.28119	0.27464
[5892]	CHEK1	--->	CUL1	0.30021	-0.32466
[5893]	CAV1	<---	GNAI2	-0.16912	0.17832
[5894]	FN1	<---	C1QA	-0.081266	0.074357
[5895]	RUNX2	--->	AXIN1	0.182	-0.18512
[5896]	JAK3	--->	IL2RB	0.50106	-0.48285
[5897]	CHEK2	--->	MDC1	0.1491	-0.16914
[5898]	VSNL1	<---	DTX2	-0.12829	0.12358
[5899]	CHAF1A	--->	HIST1H3E	0.087658	-0.077532
[5900]	GTF2IRD1	<---	PIAS2	-0.11791	0.13053
[5901]	ITGB2	<---	ICAM2	-0.22586	0.21687
[5902]	ARHGEF11	<---	PLXNB3	-0.010587	0.0085303
[5903]	NFKBIA	<---	IKBKG	-0.33406	0.3181
[5904]	PPP1R9A	<---	RPS6KB1	-0.23619	0.10302
[5905]	RARA	--->	TRIP4	0.041496	-0.016159
[5906]	PPP1CA	--->	MYO16	0.20251	-0.21243
[5907]	SERPING1	<---	SELE	-0.015384	0.03038
[5908]	MITF	<---	PAX3	-0.17658	0.17894
[5909]	PCLO	--->	GIT1	0.19102	-0.19386
[5910]	TICAM2	<---	TLR3	-0.33121	0.35273
[5911]	TBP	--->	CREM	0.0021013	-0.015228
[5912]	SKAP2	<---	FYN	-0.99957	1.0067
[5913]	PTK2B	<---	LYN	-0.65057	0.81773
[5914]	PRKACA	<---	LCK	-0.99895	0.99734
[5915]	SH3GL1	--->	SH2D4A	0.022175	-0.11352
[5916]	GNB2L1	<---	PTPRM	-0.31311	0.3077
[5917]	SYT9	--->	SYNCRIP	0.0093975	0.0083273
[5918]	SH2D1A	<---	LY9	-0.051869	0.077004
[5919]	PBX2	<---	UBE2E1	-0.21066	0.21244
[5920]	NGF	--->	PLG	0.089923	-0.07571
[5921]	DOCK1	--->	RHOG	0.00098902	-0.014022
[5922]	EP300	--->	STAT5B	0.54507	-0.55643
[5923]	GRIA2	<---	GRIP2	-0.098237	0.11658
[5924]	MST1	--->	SERPINA3	0.057207	-0.040728
[5925]	TRIP10	<---	LYN	-0.43911	0.68012
[5926]	HSPB3	<---	OLFML3	-0.33569	0.37525

[5927]	LCP2	<---	LYN	-0.99119	1.2119
[5928]	IFNAR2	<---	GNB2L1	-0.64496	0.6437
[5929]	UBE2D3	--->	MEX3B	0.1331	-0.13116
[5930]	AKAP10	--->	PRKAR2B	0.049772	-0.026102
[5931]	MAPKAPK5	--->	EIF4E	0.63658	-0.86897
[5932]	CASP3	<---	MAP4K1	-0.018328	0.03452
[5933]	SFRS1	<---	ZRSR2	-0.030095	0.061943
[5934]	CASP3	--->	YWHAE	0.5533	-0.67039
[5935]	ANK1	--->	CD44	0.0014557	-0.015093
[5936]	RXFP2	--->	RLN2	0.15462	-0.14912
[5937]	USP7	<---	PLEKHO1	-0.15405	0.18006
[5938]	NFKBIA	<---	MAP3K2	-1.0014	1.0023
[5939]	NFKBIA	<---	NKIRAS2	-1.0482	1.0419
[5940]	MYCN	<---	MAX	-0.43013	-0.38957
[5941]	RBPJ	<---	NCOR1	-0.1313	0.12062
[5942]	CASP4	<---	APAF1	-0.09754	-0.067536
[5943]	ARHGAP1	<---	RHOA	-0.40165	0.41486
[5944]	BAT4	<---	CCDC85B	-0.62233	0.65036
[5945]	BPTF	--->	MAZ	0.020395	-0.031709
[5946]	EPB41L2	--->	SPTAN1	0.0027288	-0.014532
[5947]	KAT2A	--->	TRRAP	0.0042535	-0.016927
[5948]	SNX1	<---	EGFR	-0.32869	0.32794
[5949]	PPP1R9B	<---	CHRM3	-0.19778	0.21221
[5950]	EGFR	--->	CALM3	0.34959	-0.34973
[5951]	TRIP6	--->	PXN	0.10243	-0.21198
[5952]	SIPA1L1	--->	DLG4	0.10627	-0.11902
[5953]	NCOA3	<---	ESR1	-0.13412	0.12213
[5954]	PIN1	<---	RPS6KB1	-0.4529	0.2338
[5955]	PPFIA3	<---	PTPRS	-0.11777	0.11578
[5956]	HSPA4	--->	IKBKG	0.77676	-0.76987
[5957]	E2F3	<---	CDK3	-0.75826	0.73009
[5958]	GRAP2	--->	ZBTB7B	0.29462	-0.30848
[5959]	SH2D3C	<---	EPHB2	-0.31661	0.21974
[5960]	GRAP2	--->	LCP2	0.55945	-0.50026
[5961]	SHC1	<---	MAPK1	-0.61003	0.441
[5962]	PTPN1	--->	NFKBIA	1.5719	-1.5927
[5963]	FOXO3	<---	IKBKB	-0.60135	0.59827
[5964]	IFNAR2	--->	JAK1	1.0008	-1.0061
[5965]	RELA	--->	STAT3	0.10654	-0.12805
[5966]	ACVR1	--->	FBXO30	0.047656	-0.065872
[5967]	MDM4	--->	TP73	0.13713	-0.14303
[5968]	CTNND1	<---	FER	-0.15367	0.12058
[5969]	NOTCH3	<---	RBPJ	-0.093292	0.080448
[5970]	POU2F1	--->	RELA	0.08441	-0.079104
[5971]	NCOA1	<---	NR2F1	-0.043163	0.034596
[5972]	AHR	<---	CUL4B	-0.28729	0.29032
[5973]	RNF11	<---	UBE2D4	-0.17305	0.18537
[5974]	CD86	--->	CD80	0.055892	-0.098762
[5975]	STAT5A	<---	IL2RB	-0.4655	0.43165
[5976]	MYPN	<---	NEB	-0.040427	0.069388
[5977]	CYTH1	<---	ARF1	-0.18769	0.20203
[5978]	SMAD3	<---	PLAGL1	-0.12495	0.20566
[5979]	SUFU	--->	ZNF764	0.062865	-0.065836
[5980]	DUSP3	--->	MAPK1	1.011	-0.99819
[5981]	CDK1	--->	E2F1	0.44723	-0.46975
[5982]	ARR3	<---	ADRB2	-0.12587	0.12815
[5983]	ITGAM	<---	ICAM4	-0.056518	0.044009
[5984]	$ZBTB8$	<---	LNX1	-0.097156	0.101
[5985]	PRKCE	--->	TRPV1	0.42132	-0.46647
[5986]	ARID3A	--->	E2F1	0.011183	0.0027071
[5987]	DNM2	<---	HCK	-0.2853	0.35603
[5988]	SORBS2	--->	ABL2	-0.098797	-0.22378
[5989]	CTNNB1	<---	PLK1	-0.6004	0.5962
[5990]	NR2F2	<---	FAM46A	-0.043362	0.069121
[5991]	SPI1	--->	ATF1	0.24589	-0.19035
[5992]	CUL4A	--->	RBX1	0.12225	-0.11034
[5993]	BMPR1B	<---	RAB38	-0.53994	0.52577
[5994]	GRAP2	<---	KIT	-0.47434	-0.1804
[5995]	GAB1	--->	SHC1	0.039391	-0.063016
[5996]	TRA2B	<---	NGFR	-0.040954	0.22397
[5997]	EPS8	<---	CCDC85B	-0.1561	0.072585
[5998]	DLG1	--->	MAP1A	0.028718	-0.035109
[5999]	LAMA4	<---	COPS6	-0.21895	0.24525
[6000]	PUF60	?	SFRS11	-0.016298	-0.016298
[6001]	CCL5	--->	PIGR	0.031299	-0.0064564
[6002]	ITGB2	<---	CD14	-0.089208	0.081174

[6003]	SNX2	--->	LEPR	0.3803	-0.38118
[6004]	MAPK1	--->	IRS1	0.5492	-0.60947
[6005]	PPARD	--->	RXRA	1.003	-0.99549
[6006]	SOCS1	--->	VAV2	0.16187	-0.15263
[6007]	SMG1	--->	SMAD9	0.17181	-0.18822
[6008]	GRIN1	<---	DLG4	-0.084559	0.076324
[6009]	CCND3	<---	CDK4	-1.0774	1.0544
[6010]	PURA	<---	SP1	-0.16097	0.15089
[6011]	TLR4	--->	HSP90B1	0.11185	-0.15712
[6012]	TJP2	<---	OCLN	-0.043104	0.049764
[6013]	EED	<---	ITGB7	-0.019834	0.023504
[6014]	CSTF2	<---	EEF1G	0.011855	0.020685
[6015]	WASF3	--->	PIK3R1	0.080499	-0.15052
[6016]	HGS	<---	BEGAIN	-0.047623	-0.0039467
[6017]	NTRK1	<---	PTPN1	-0.60499	0.59653
[6018]	PEF1	<---	PDCD6	-0.24636	0.11988
[6019]	CDK1	--->	TP73	0.45486	-0.4556
[6020]	EDIL3	--->	ITGAV	0.008471	-0.020594
[6021]	BRCA1	<---	MAP3K3	-0.17392	0.32041
[6022]	KCND2	<---	IL16	-0.013537	0.050181
[6023]	DYRK1A	--->	YWHAG	0.39808	-0.42581
[6024]	APLP2	--->	PIK3CA	0.04715	-0.059156
[6025]	MPP5	<---	INADL	-0.027566	0.015158
[6026]	PTPN11	<--->	PTPN6	0.66019	0.66019
[6027]	UBTF	--->	TAF1	0.053694	-0.050432
[6028]	SH3GLB1	--->	SYNJ1	0.084721	-0.07064
[6029]	PRKCG	--->	CASR	0.55548	-0.55976
[6030]	PCBP1	--->	QKI	-0.0053675	-0.11083
[6031]	HOMER1	--->	SHANK1	0.032716	-0.042422
[6032]	ZEB2	--->	SMAD3	0.072366	-0.029947
[6033]	CXCL12	--->	FN1	0.01648	0.0125
[6034]	STAT5A	<---	MAPK1	-0.80262	0.7916
[6035]	TRIP4	--->	JUN	0.3726	-0.41837
[6036]	IKBKG	<---	TNFAIP3	-0.28058	0.26629
[6037]	TP53BP1	<---	NFKB1	-0.77017	0.77811
[6038]	MMP7	<---	HBEGF	-0.16726	0.174
[6039]	YWHAG	--->	BAT1	0.049821	-0.049431
[6040]	DOK2	<---	TEK	-0.020626	0.0066319
[6041]	ARNTL	<---	AHR	-0.061404	-0.060412
[6042]	MYOD1	<---	CALM2	-0.22666	0.2676
[6043]	RING1	--->	RB1	0.47982	-0.4811
[6044]	CAV2	<---	NCK1	-0.34888	0.32617
[6045]	HIF1A	--->	ARNTL	0.30742	-0.0073388
[6046]	NET1	--->	MAGI1	0.013211	-0.022387
[6047]	FN1	<---	COL4A3	-0.048751	0.04098
[6048]	CREBBP	--->	MSX1	0.18505	-0.18943
[6049]	ZBTB16	<---	HBEGF	-0.091882	0.085344
[6050]	NCK1	<---	FLT3	-0.21439	0.060028
[6051]	GRIN1	<---	NF1	-0.14735	0.13674
[6052]	LRP1	<---	A2M	-0.15004	0.14086
[6053]	TEAD3	<---	WWTR1	-0.46736	0.46792
[6054]	PTPN6	<---	EGFR	-0.25411	0.37037
[6055]	STAT1	<---	EP300	-0.5941	0.5772
[6056]	RIT2	--->	RGL1	0.98547	-1.0084
[6057]	INSR	--->	PLCG1	0.18107	-0.20582
[6058]	GAB1	--->	CRKL	0.057403	-0.069249
[6059]	RALGPS1	<---	RALA	-0.37677	0.38164
[6060]	FHL2	--->	ITGA7	0.0067069	-0.017023
[6061]	CDK1	<---	RAB4A	-1.0074	1.0132
[6062]	ARHGEF12	<---	PLXNB2	-0.010587	0.0085303
[6063]	NCBP2	?	HNRNPF	-0.016298	-0.016298
[6064]	TRAF2	--->	RALBP1	0.052448	-0.055581
[6065]	YWHAG	--->	EPB41L3	0.05792	-0.057222
[6066]	TENC1	<---	ITGB3	-0.030112	0.017774
[6067]	FRS2	<---	PRKCZ	-0.37242	0.37492
[6068]	CREBBP	<---	CSNK2A1	-0.32425	0.32272
[6069]	TBC1D4	<---	ZMIZ1	-0.012852	0.0061769
[6070]	TRDN	--->	RYR1	0.075908	-0.088029
[6071]	ICAM5	--->	PSEN1	0.066177	-0.066651
[6072]	IFT172	--->	LHX3	0.070191	-0.055821
[6073]	TNFSF9	--->	TNFRSF9	0.89674	-0.85556
[6074]	CRHR1	<---	GNAQ	-0.43656	0.4334
[6075]	EPS15	<---	HGS	-0.024648	0.0091155
[6076]	SVIL	--->	GPSM3	0.14038	-0.082549
[6077]	PTPN1	--->	STAT5B	1.0055	-1.005
[6078]	SOS1	--->	MUC1	0.22149	-0.23178

[6079]	CSF1R	--->	INPP5D	0.4412	-0.409
[6080]	AKAP11	<---	PRKAR2A	-0.35498	0.38689
[6081]	BNIP3	--->	BCL2	0.68788	-0.67957
[6082]	FRS3	<---	GRB2	-0.46631	0.46592
[6083]	ACVR1	--->	PLEKHJ1	-0.005073	-0.039192
[6084]	RBM39	--->	JUN	0.30791	-0.33899
[6085]	GRB14	<---	PDGFRB	-0.11309	0.098098
[6086]	HRAS	--->	MAP2K1	0.91853	-0.51286
[6087]	JAG1	--->	NOTCH1	1.0093	-0.99617
[6088]	EGFR	--->	FAS	0.13251	-0.10107
[6089]	LTBP1	<---	FBN1	-0.011353	-0.0099732
[6090]	MAPKAPK2	--->	HNRNPA0	0.54388	-0.5661
[6091]	PARD3	<---	LIMK2	-0.038276	0.027249
[6092]	HDAC4	--->	ATRX	0.041901	-0.051516
[6093]	CKAP5	<---	AURKA	-0.74005	0.71415
[6094]	KLHL12	<---	DRD4	-0.24308	0.25521
[6095]	STAT1	--->	ADRA1B	0.024631	-0.078785
[6096]	APBA1	<---	HTR2C	-0.058908	0.055497
[6097]	CD40	--->	PIK3R1	0.068767	-0.14164
[6098]	GRB2	--->	SH2B2	0.50707	-0.51768
[6099]	EP300	--->	KPNA2	0.18327	-0.18765
[6100]	KLF6	--->	EHMT2	0.021748	-0.032167
[6101]	TP53BP1	--->	H2AFX	0.30406	-0.25841
[6102]	PKP2	--->	DSG2	0.31989	-0.319
[6103]	IKBKG	<---	HSP90AB1	-0.10825	0.097619
[6104]	CD4	<---	TFRC	-0.0092896	-0.00146
[6105]	LUC7L3	<---	YWHAG	-0.039464	0.063256
[6106]	UBE2I	<---	FAS	0.087935	0.096234
[6107]	CPNE1	--->	SKIL	0.48698	-0.48061
[6108]	CDC25B	<---	MAPK14	-1.001	1.0024
[6109]	MAPRE1	--->	APC	0.042639	-0.055657
[6110]	PIK3CG	--->	IL6ST	0.012035	-0.023435
[6111]	CHAF1A	<---	CDK2	-0.28437	0.26665
[6112]	GAN	<---	MAP1B	0.0026527	0.030786
[6113]	MAPK14	--->	MAX	0.99106	-0.98599
[6114]	PAX6	<---	SIX3	-0.037249	-0.024315
[6115]	HDAC9	--->	MEF2A	0.072487	-0.072318
[6116]	AXL	<---	CBL	-0.18425	0.17263
[6117]	CDH7	?	CDH15	-0.058742	-0.058742
[6118]	RBPMS	--->	ZNF581	0.20364	-0.19923
[6119]	TGFBRAP1	<---	BMPR1A	-0.11039	0.016772
[6120]	ESR1	<---	RBM23	-0.040633	0.064959
[6121]	CBX8	--->	RING1	0.066008	-0.045755
[6122]	SMAD3	<---	PAX8	-0.11532	0.16263
[6123]	PRTN3	<---	F2RL1	-0.42368	0.45462
[6124]	MEF2D	<---	EP300	-0.032519	0.019933
[6125]	VAV1	<---	GRB2	-0.57842	0.5996
[6126]	PIN1	--->	DDX24	0.19137	-0.17708
[6127]	RALYL	--->	AEBP2	0.054367	-0.039253
[6128]	TRAF4	--->	MAP3K4	0.21579	-0.22531
[6129]	NBN	<---	BRCA1	-0.020991	0.036669
[6130]	MAPK7	--->	RAF1	0.39902	-0.48402
[6131]	INSR	--->	PIK3R1	0.031725	-0.06783
[6132]	NOL3	--->	CASP2	-0.076001	-0.18686
[6133]	TLL1	<---	CHRD	-0.16477	0.17437
[6134]	PIAS1	--->	SMAD7	1.0161	-0.98478
[6135]	ARHGEF3	<---	RHOA	-0.29753	0.32945
[6136]	OTX1	<---	RBPMS	-0.071742	0.10141
[6137]	THBS3	--->	THBS2	-0.0066825	-0.014637
[6138]	KHDRBS1	<---	HNRNPK	-0.11083	-0.0053675
[6139]	HTATSF1	?	SNRNP70	-0.016298	-0.016298
[6140]	CNTNAP2	<---	CNTN2	-0.14755	0.14383
[6141]	MAGI2	<---	DSCAML1	-0.077155	0.069415
[6142]	IL1B	--->	A2M	0.19125	-0.17688
[6143]	TP53	<---	HSPB1	-0.31524	0.33168
[6144]	NINL	--->	ZNF426	0.25124	-0.25567
[6145]	LIN7B	<---	DLG4	-0.018249	0.0028049
[6146]	TRPC6	<---	FYN	-0.11001	0.1001
[6147]	MSN	<---	EZR	-0.0027583	-0.0024871
[6148]	PAK2	--->	SH3PXD2A	0.23346	-0.085436
[6149]	GIT1	<---	PPFIA3	-0.16385	0.16054
[6150]	PEA15	--->	PANK4	0.13327	-0.092431
[6151]	ITGB3	<---	FGG	-0.048962	0.046421
[6152]	SAG	<---	RHO	-0.12587	0.12815
[6153]	PTPN11	--->	SPRY2	0.83412	-0.64372
[6154]	APP	<---	PSEN2	-1.0006	0.99505

[6155]	PRKD1	--->	HDAC7	0.44235	-0.46746
[6156]	NEK6	<---	SMURF2	-0.6445	0.64008
[6157]	SRC	--->	TNFRSF11A	0.68579	-0.63231
[6158]	THRA	<---	NCOR1	-0.065644	0.063164
[6159]	HNRNPK	--->	KHDRBS2	-0.0053675	-0.11083
[6160]	JAK2	--->	STAT1	1.0102	-0.99843
[6161]	FBXO9	<---	SKP1	-0.044878	0.055251
[6162]	MYD88	<---	TLR2	0.011261	0.29772
[6163]	SMAD4	<---	CEBPB	-0.28428	0.37299
[6164]	TGFBR3	<---	TGFBR2	-1.0004	0.99477
[6165]	CDC25C	<---	CDK1	-0.67632	0.69298
[6166]	TERT	<---	RPS6KB1	-0.41976	0.37453
[6167]	RIPK2	--->	CD40	0.32412	-0.35718
[6168]	HEY2	<---	HAND1	-0.43036	-0.38951
[6169]	RGS12	<---	CXCR2	-0.22172	0.22398
[6170]	GPR1	--->	GNAI1	1.0129	-1.008
[6171]	SLC9A3R2	--->	PTH1R	0.37858	-0.37394
[6172]	HDAC2	<---	DNMT1	-0.013043	0.016989
[6173]	ACVR1B	--->	IGSF1	0.14057	-0.16505
[6174]	ARRB2	--->	FZD4	0.28782	-0.34737
[6175]	PTK2B	<---	FYN	-0.65057	0.81773
[6176]	MDM4	--->	YWHAB	0.040034	-0.045818
[6177]	NFKB2	--->	HDAC5	0.005213	-0.021922
[6178]	HDAC4	<---	YWHAZ	-0.043467	0.067622
[6179]	GNB2L1	--->	ITGB1	0.12677	-0.13612
[6180]	OPRM1	--->	GNA15	0.9872	-0.98627
[6181]	F12	--->	CD93	0.026359	-0.034104
[6182]	SELL	<---	GRB2	-0.25658	0.25089
[6183]	LPXN	<---	PTK2	-0.4799	0.41189
[6184]	WIZ	--->	EHMT1	0.045355	-0.050681
[6185]	ZBTB16	<---	AGTR2	-0.21621	0.21403
[6186]	HDAC3	--->	TNFRSF14	0.18467	-0.19362
[6187]	MRAS	--->	BRAF	1.0064	-0.99496
[6188]	SRCIN1	<---	BCAR1	-0.016648	0.019146
[6189]	DLX1	<---	SMAD4	-0.14259	0.20304
[6190]	ARHGEF7	<---	TGFBR2	-0.18253	0.17602
[6191]	MAPK3	<---	PPP2CA	-0.5252	0.52309
[6192]	PKP2	--->	SMAD9	0.14616	-0.16653
[6193]	CSF2RB	<---	STAT1	-0.13018	0.047167
[6194]	NCF4	--->	CORO1A	0.10089	-0.11311
[6195]	SCN5A	<---	RGS3	-0.18541	0.19078
[6196]	CX3CL1	<---	CX3CR1	-0.46475	0.49595
[6197]	CXCL1	<---	CXCR2	-0.46475	0.49595
[6198]	SP100	<---	HIPK2	-0.89929	0.92033
[6199]	PARD6A	<---	TGFBR1	-0.18749	0.13918
[6200]	YWHAB	--->	FRMD6	0.05792	-0.057222
[6201]	PPP2R1B	<---	PPP2CA	-0.089956	0.079333
[6202]	LYN	<---	PRKCD	-0.3255	0.34363
[6203]	NTRK1	--->	GRB2	-0.037932	-0.114
[6204]	KAT2B	--->	MYOD1	0.35027	-0.34832
[6205]	U2AF2	?	SFRS1	-0.016298	-0.016298
[6206]	HDAC2	--->	SUV39H1	0.034642	-0.035506
[6207]	PPP5C	--->	MAPT	0.12638	-0.12832
[6208]	RGS3	--->	YWHAQ	0.15514	-0.15346
[6209]	CSTF2T	<---	UBQLN4	-0.057469	0.089135
[6210]	RFX3	--->	RFX1	-0.0041411	-0.0094131
[6211]	PRMT8	<---	PRMT1	0.01685	0.16484
[6212]	ENAH	<---	ABI1	-0.015262	0.0018099
[6213]	UBE2V2	--->	MOBKL3	0.37664	-0.34522
[6214]	NXF1	--->	NUP214	0.029559	0.0037648
[6215]	PDK2	<--->	PDK1	0.18546	0.18546
[6216]	ROCK1	--->	CDC25A	0.9899	-0.99632
[6217]	SMAD7	<---	ACVR1B	-1.0087	1.0115
[6218]	APBA3	--->	APP	0.12974	-0.14219
[6219]	ASAP1	<---	SRC	-0.12704	0.44583
[6220]	BMPR1B	<---	GDF9	-0.34476	0.32284
[6221]	ACTN4	--->	ACTC1	0.18997	-0.19732
[6222]	GABBR1	<---	CHFR	-0.12196	0.13649
[6223]	DTNA	<---	SNTB1	-0.12526	0.13506
[6224]	SYT7	--->	SYNCRIP	0.077746	-0.039791
[6225]	BRAF	<---	YWHAQ	-0.19664	0.18212
[6226]	NFIB	?	NFIX	-0.1934	-0.1934
[6227]	HDAC3	<---	PPP4C	-0.36273	0.35578
[6228]	IKZF2	?	IKZF1	-0.026262	-0.026262
[6229]	PRKACA	<---	PTPN12	-0.46863	0.46317
[6230]	ROBO2	--->	UBQLN4	0.042727	-0.030717

[6231]	CPSF6	--->	UBQLN4	0.15666	-0.11813
[6232]	BTK	--->	TLR4	0.094492	-0.12746
[6233]	SMAD3	--->	NOTCH1	0.25414	-0.26964
[6234]	PXN	<---	PTK2B	-0.99895	1.009
[6235]	BIRC5	<---	CASP9	0.0029245	0.0050222
[6236]	ALB	<---	KLK3	-0.083503	0.11621
[6237]	FN1	<---	COL4A1	-0.057266	0.047596
[6238]	GRM7	<---	FLNA	0.0058519	0.028788
[6239]	VASN	--->	TGFB2	0.45796	-0.41124
[6240]	UBE2E1	<---	UBE3A	-0.048727	0.045324
[6241]	CALM2	<---	MYO6	-0.1952	0.2124
[6242]	FGF2	<---	RPS6KA3	-0.47744	0.25344
[6243]	NUP153	<---	RAN	-0.32029	0.35116
[6244]	MAP3K14	--->	TRAF1	0.35273	-0.36329
[6245]	MYL6	--->	MOBKL3	0.25366	-0.22061
[6246]	SMURF2	--->	TSSK4	0.64008	-0.6445
[6247]	SIT1	<---	GRB2	-0.41139	0.4
[6248]	CRKL	--->	STAT5A	0.32804	-0.36563
[6249]	MYF6	<---	CALM1	-0.22666	0.2676
[6250]	EIF3I	<---	TGFBR2	-0.87271	0.93273
[6251]	CD247	<---	CD28	-0.20612	0.19126
[6252]	TRIP4	<---	CREBBP	-0.19086	0.18651
[6253]	CREBBP	--->	SPIB	0.18812	-0.1922
[6254]	NEDD4L	--->	UBE2L3	0.48868	-0.48772
[6255]	MKNK2	<--->	MAPK1	0.23429	0.23429
[6256]	AR	<---	TCF4	-0.066751	0.054169
[6257]	HSPA1A	--->	APEX1	0.8464	-0.80453
[6258]	YWHAG	--->	PRKDC	0.030448	-0.029947
[6259]	CALM1	--->	PTH2R	0.25258	-0.24765
[6260]	KPNA2	<---	ITK	-0.76215	0.72008
[6261]	DAPK1	--->	MAPK1	0.17015	-0.14879
[6262]	INHA	<---	FST	-0.6043	0.61193
[6263]	STAT1	<---	CREBBP	-0.5941	0.5772
[6264]	PPP1R9A	--->	PPP1R9B	0.044713	-0.027792
[6265]	IKBKG	<---	HSP90AA1	-0.076976	0.064877
[6266]	HDAC9	<--->	HDAC4	0.015471	0.015471
[6267]	TP53	<---	TOP2A	-0.41701	0.42803
[6268]	FBLN2	<---	COL4A2	-0.033412	0.023756
[6269]	ZNF408	<---	CHAF1A	-0.04532	0.049625
[6270]	DAXX	--->	MAP3K5	0.99897	-0.99739
[6271]	GATAD2B	<---	MBD2	-0.014778	0.059037
[6272]	DEF6	<---	RAC2	-0.26331	0.26775
[6273]	PTPN12	--->	GRB2	0.31332	-0.40658
[6274]	PLEC1	--->	GNB2L1	0.1289	-0.09589
[6275]	BCL3	--->	CTNNB1	0.32025	-0.32481
[6276]	PRKACA	--->	EGFR	0.29741	-0.32251
[6277]	SYK	--->	SHC1	1.0049	-0.99981
[6278]	AR	<---	TGIF1	-0.57013	0.56462
[6279]	PTK2B	--->	JAK2	0.062957	0.052768
[6280]	EVL	--->	WASL	-0.014273	-0.073329
[6281]	SON	<---	PRPF40A	-0.17875	0.19278
[6282]	CASP3	--->	EIF4B	0.15504	-0.17985
[6283]	STAT2	<---	CXCR4	-0.62412	0.59407
[6284]	GNAQ	<---	CHRM3	-0.98627	0.9872
[6285]	CCR8	--->	CCL17	0.49595	-0.46475
[6286]	ITGA2B	<---	COL1A2	-0.016967	0.0035823
[6287]	HMGB2	--->	APEX1	0.20407	-0.1711
[6288]	SNX9	--->	TNK2	0.00024965	-0.014373
[6289]	BIRC6	<---	MAP2K1	-0.0055362	0.22821
[6290]	SMAD5	<---	ACVR1	-1.5009	1.5013
[6291]	SGK1	<---	GSK3B	-1.0109	1.002
[6292]	ESR1	--->	BDNF	0.027304	-0.032123
[6293]	PFN1	--->	ACTB	0.99385	-1.0067
[6294]	SMAD3	<---	DDX5	-0.10567	0.12731
[6295]	JUNB	--->	BCL6	0.11317	-0.14009
[6296]	NR3C1	--->	CREB1	0.1808	-0.17665
[6297]	GAB2	<---	PIK3R1	-0.10349	0.077508
[6298]	EGFR	--->	DCN	0.13803	-0.14107
[6299]	SRC	--->	BCAR1	0.99683	-0.98488
[6300]	PDPK1	--->	PKN1	0.68989	-0.69757
[6301]	NFASC	--->	ANK1	0.055421	-0.071273
[6302]	PKP2	--->	DSC2	0.61098	-0.61498
[6303]	RACGAP1	--->	MAP3K4	0.11164	-0.12329
[6304]	RIT2	--->	RLF	0.31566	-0.30104
[6305]	RHOQ	--->	WASL	0.22215	-0.29848
[6306]	NINL	--->	LRSAM1	0.0032816	0.0010171

[6307]	IFNG	--->	IFNGR1	0.99985	-0.99847
[6308]	TRPC3	--->	ITPR3	-0.0071319	-0.016919
[6309]	PTPN6	--->	ROS1	0.66467	-0.44862
[6310]	KPNA4	<---	TGM2	-0.030402	0.062673
[6311]	HNRNPK	--->	TBP	-0.0016018	-0.010648
[6312]	SMAD1	<---	SUV39H1	-0.092045	0.078555
[6313]	CDKN3	<---	CDK2	-0.56626	0.54604
[6314]	SIT1	<---	LCK	-1.0153	1.0748
[6315]	TAX1BP1	<---	TNFAIP3	-0.13342	0.13038
[6316]	PHF23	<---	LRSAM1	-0.082901	0.087249
[6317]	ITGA3	--->	CALR	-0.0030469	-0.0082117
[6318]	CTNNB1	<---	SMAD7	-0.18358	0.17916
[6319]	SPHK1	<---	TRAF2	-0.13885	0.14454
[6320]	PAX8	--->	ID2	0.13137	-0.10726
[6321]	MTA1	<---	GRB2	-0.37216	0.39013
[6322]	PTPRC	--->	JAK1	0.085657	-0.11378
[6323]	PKNOX1	<---	HOXB7	-0.0094151	0.024726
[6324]	PTK2	<---	RIPK1	-0.28028	0.29415
[6325]	EGFR	--->	SFN	0.28431	-0.28735
[6326]	NEBL	--->	ZYX	0.16668	-0.13223
[6327]	PPARG	--->	NCOA1	0.018111	-0.028796
[6328]	HEYL	--->	SKIL	0.15953	-0.16202
[6329]	ITGA5	<---	COL1A1	-0.013468	0.00013769
[6330]	IL1R2	<---	IL1B	-0.021437	0.026308
[6331]	DYRK2	--->	HIST3H3	1.0832	-1.0289
[6332]	SYNE1	--->	ACTC1	0.29277	-0.29627
[6333]	LAMC2	<---	COL7A1	-0.011192	-0.0013797
[6334]	ITGA4	--->	ITGB1	-0.0099538	-0.039347
[6335]	LIMK1	--->	CFL2	0.16176	-0.17545
[6336]	CXXC1	<---	SMURF1	-0.70457	0.70072
[6337]	ASAP1	<---	REPS2	0.0014538	0.002835
[6338]	TICAM2	<---	TRAF6	-0.38587	0.37253
[6339]	SFRS5	<---	SMURF1	-0.71228	0.70809
[6340]	SMAD1	--->	HOXA13	0.010712	-0.04291
[6341]	IL2RB	<---	SYK	-0.95875	0.90605
[6342]	FLNB	<---	TSHR	-0.31324	0.33122
[6343]	FOXO1	--->	RARA	0.057946	-0.066978
[6344]	VAV1	<---	BCR	-0.073594	-0.012772
[6345]	INSR	<---	IGF2	-1.0034	0.99535
[6346]	BMPR1B	<---	PPP3CC	-0.026795	0.010469
[6347]	PRMT1	--->	HNRNPK	0.29604	-0.29359
[6348]	DTNA	--->	ACTA1	0.53711	-0.5343
[6349]	BAIAP3	--->	BAI1	0.16834	-0.15092
[6350]	ARID3A	--->	E2F2	0.01732	0.01461
[6351]	USP7	--->	ZMYND8	0.041195	-0.0042183
[6352]	PPP2CA	--->	BCL2	0.33848	-0.34606
[6353]	PCCB	--->	PCCA	0.010822	-0.013952
[6354]	RB1	<---	FOS	-0.042701	0.0024136
[6355]	LRRC7	<---	CNKSR2	-0.0086982	-0.0049689
[6356]	LPHN2	<---	SHANK1	-0.01098	0.00131
[6357]	GRIA4	<---	ACTN1	-0.036635	0.034438
[6358]	RNF11	<---	RIPK1	-0.075476	0.32806
[6359]	FSHB	<--->	CGB	0.073889	0.073889
[6360]	ITGA3	--->	ITGB1	-0.0080652	-0.019088
[6361]	JUB	<---	GRB2	-0.34579	0.34758
[6362]	COLQ	<---	MUSK	-0.029676	0.018668
[6363]	SMAD2	<---	NUP214	-0.32954	0.41245
[6364]	PBK	--->	SMAD4	1.012	-1.0074
[6365]	ZNF174	--->	ZNF24	-0.040055	-0.15885
[6366]	DOK2	<---	ABL1	-0.12751	-0.027909
[6367]	EPHB2	--->	NCK1	0.064938	-0.1433
[6368]	TENC1	<---	ITGB5	-0.030112	0.017774
[6369]	REPS2	<---	GRB2	-0.294	0.30689
[6370]	SMAD3	--->	ZNF8	0.12829	-0.12271
[6371]	PIN1	--->	RAF1	0.080443	-0.10668
[6372]	SERPINB9	<---	BRD7	-0.13182	0.16757
[6373]	KCNA5	?	KCNA2	-0.025901	-0.025901
[6374]	SVIL	<---	MYLK	-0.44162	0.468
[6375]	PXN	<---	PTEN	-0.0066783	0.010173
[6376]	SH3GL3	--->	CALCOCO1	0.096825	-0.081545
[6377]	AVPR1A	<---	PRKCA	-0.53729	0.53258
[6378]	ACTN4	--->	ACTN3	-0.021733	-0.025796
[6379]	CPNE4	<---	PDCD6	-0.22356	0.24347
[6380]	KRT15	<---	KRT6B	-0.017396	0.033003
[6381]	RNF11	--->	SDCBP	0.01785	-0.0026965
[6382]	UBE3A	--->	UBE2G1	0.18395	-0.17948

[6383]	PIK3CD	<---	ASCC2	-0.47314	0.47292
[6384]	FAS	<---	RAP1A	-0.017839	0.20018
[6385]	RNF11	--->	USP5	0.1181	-0.10386
[6386]	KAT2B	--->	ETV1	0.20937	-0.20573
[6387]	KHDRBS1	<---	BTK	-0.38206	0.35852
[6388]	SHC1	<---	SOS2	-0.04276	0.014124
[6389]	SYTL1	<---	NCF1	-0.018599	0.014406
[6390]	ITGA1	<---	MATN1	-0.025531	-0.0063827
[6391]	LMO4	<---	BRCA1	-0.0048072	0.021368
[6392]	SHC1	<---	MAPK8	-0.61003	0.441
[6393]	FGFR3	<---	GTF3C1	-0.15255	0.14588
[6394]	SRY	--->	ZNF208	0.41962	-0.41652
[6395]	PTPRF	--->	INSR	-0.0055525	-0.043408
[6396]	DAB2	<---	CSK	-0.94185	1.1509
[6397]	KPNA6	<---	STAT1	-0.28474	0.16081
[6398]	GRB14	<---	EGFR	-0.42553	0.37299
[6399]	GDF9	--->	ORAI2	0.026151	0.019776
[6400]	NCAM1	<---	GFRA1	-0.0054625	0.017999
[6401]	JUN	<---	ELF3	-0.16285	0.13316
[6402]	ATXN1	<---	RBPMS	-0.032318	0.028442
[6403]	HIP1R	?	HIP1	-0.0074951	-0.0074951
[6404]	LPAR2	--->	ARHGEF11	0.10316	-0.106
[6405]	GRAP2	<---	CSF1R	-0.99864	1.0022
[6406]	NOS1AP	--->	NOS1	0.26769	-0.27718
[6407]	MAP3K10	--->	CDC42	0.36613	-0.36833
[6408]	ARHGAP17	--->	ABL1	-0.039252	-0.047794
[6409]	LAMB3	?	LAMC2	-0.0035326	-0.0035326
[6410]	FXYD2	--->	ATP1A1	0.097298	-0.097228
[6411]	NOD1	--->	CASP2	-0.038798	-0.12583
[6412]	RAB3B	--->	TGFBR1	0.010625	-0.037483
[6413]	CCNE1	<---	CDK3	-1.0774	1.0544
[6414]	RIPK2	--->	CASP1	-0.011534	-0.66076
[6415]	CASP10	--->	BID	0.49065	-0.45075
[6416]	COL4A5	--->	APP	0.076846	-0.089884
[6417]	ZNF557	<---	SMAD9	-0.19115	0.17167
[6418]	TRAF3	<---	RRAS2	-0.14052	0.14278
[6419]	RORC	<---	EIF4EBP1	-0.099837	0.12515
[6420]	CDH5	<---	CTNND1	-0.52601	0.51654
[6421]	PIN1	--->	MAPT	0.43702	-0.41832
[6422]	MAGI1	<---	RPS6KA3	-0.073246	0.032672
[6423]	LCP2	--->	WAS	0.18774	-0.26391
[6424]	RARA	?	NR3C1	-0.0089162	-0.0089162
[6425]	POU2F1	--->	NR3C1	0.012582	0.012013
[6426]	LTB	--->	LTA	0.44745	0.33731
[6427]	ATR	<---	RHEB	-0.099507	0.095546
[6428]	HOXA13	--->	MEIS2	0.03787	-0.033829
[6429]	TRAF2	--->	FLNA	0.14931	-0.14359
[6430]	CCBP2	--->	CCL8	0.49595	-0.46475
[6431]	TF	--->	CALR	0.087214	-0.067218
[6432]	YWHAZ	--->	RALGPS2	0.18217	-0.17019
[6433]	MAPK9	--->	JUN	1.0022	-1.0148
[6434]	SRGAP3	--->	WASF1	0.074932	-0.079967
[6435]	JUP	--->	DSG1	0.07062	-0.077057
[6436]	CEBPE	<---	MLLT6	-0.082242	0.13197
[6437]	DNMBP	<---	HSPA1B	-0.086172	0.069808
[6438]	PRKCA	--->	GJB1	0.55904	-0.56302
[6439]	RAB3B	--->	PIGR	0.42399	-0.40837
[6440]	PURA	<---	CDK5	-0.7435	0.71754
[6441]	MKNK2	--->	MAPK14	1.0074	-0.9992
[6442]	PIK3CD	<---	PIK3R1	-0.034565	0.0096742
[6443]	AKAP6	--->	RYR2	0.3645	-0.37025
[6444]	EPHB2	--->	KALRN	0.0015108	-0.0070644
[6445]	CRKL	--->	ABL1	0.99616	-0.99908
[6446]	CCR4	--->	CCL22	0.45214	-0.43752
[6447]	SHC1	<---	PAK1	-0.34296	0.27489
[6448]	TSC2	<---	CDKN1B	-0.099249	0.092292
[6449]	MTNR1A	--->	LRRC59	0.099908	-0.091553
[6450]	PRKACA	--->	RGS10	0.28697	-0.32153
[6451]	RASSF2	<---	KRAS	-0.16806	0.16144
[6452]	BATF	--->	JUN	0.36727	-0.73152
[6453]	HNF4G	--->	NR0B2	0.1947	-0.023084
[6454]	ZHX1	<---	BAT1	-0.027303	0.024257
[6455]	SOX9	<---	HSPA1A	-0.22765	0.21713
[6456]	KHDRBS1	<---	SRC	-0.49991	0.53341
[6457]	TRAP1	--->	RB1	0.48098	-0.48246
[6458]	PIM1	<---	HSP90AA1	-0.056762	0.040193

[6459]	KEAP1	--->	MYO7A	0.039471	-0.035976
[6460]	IL2RG	--->	IL7R	-0.021798	-0.069843
[6461]	GSK3B	--->	CREM	0.96654	-0.95401
[6462]	CAV1	<---	GRK5	-0.57144	0.48233
[6463]	SFPQ	<---	FUS	-0.02338	-0.0017915
[6464]	MAGI2	<---	CTNND2	-0.035713	0.033986
[6465]	TWIST2	--->	SREBF1	-0.13859	-0.34582
[6466]	BCL2L1	?	BCL2	-0.42958	-0.42958
[6467]	FYN	<---	CBLB	-0.7389	0.74409
[6468]	CALCOCO2	<---	RHPN1	-0.0041854	0.022218
[6469]	JAK3	--->	SOCS3	1.0007	-1.0005
[6470]	ATXN1	--->	VSNL1	0.040063	-0.042644
[6471]	USP7	<---	TRAF2	-0.0093461	0.14237
[6472]	SFRS2	?	SNRNP70	-0.016298	-0.016298
[6473]	CUL1	--->	NFKBIE	0.48134	-0.47008
[6474]	TNXB	--->	VEGFA	0.0083342	-0.021172
[6475]	NBN	<---	MRE11A	-0.071207	0.10501
[6476]	GRB2	<---	FYN	-0.091982	0.77154
[6477]	CSNK2A1	--->	RNF7	0.69391	-0.71981
[6478]	SOCS1	<---	FLT3	-0.37268	0.26243
[6479]	IL1RL1	--->	MYD88	0.17593	-0.0036516
[6480]	YWHAB	<---	FER	-0.26988	0.21524
[6481]	CD3G	?	CD3D	-0.22052	-0.22052
[6482]	MAPKAPK5	<---	MAPK1	-0.45114	0.8761
[6483]	FOXO3	<---	EP300	-0.060941	0.050393
[6484]	YWHAB	<---	STK38L	-0.2472	0.13548
[6485]	$ZBTB8$	<---	TRIM41	-0.078711	0.075019
[6486]	ACP1	--->	INSR	0.23047	-0.23973
[6487]	FGF6	--->	FGFR3	0.60861	-0.61087
[6488]	RASA1	<---	EPHA2	-0.21105	-0.0081541
[6489]	HTR2B	--->	GNA11	0.9872	-0.98627
[6490]	DLG4	<---	MAPK12	-0.14764	0.087106
[6491]	CSNK2A1	--->	GYS1	1.0067	-1.0067
[6492]	PRSS23	<---	PHF23	0.01683	0.017126
[6493]	FYN	--->	BCL3	0.83462	-0.75084
[6494]	TRAF2	--->	WDR65	0.05972	-0.06654
[6495]	CXXC5	--->	SMAD3	0.25495	-0.21011
[6496]	TSC2	<---	CCND1	-0.028546	0.025619
[6497]	PICK1	<---	JAM3	-0.15671	0.14909
[6498]	TERT	--->	RELA	-0.0022036	-0.0091699
[6499]	LCK	--->	VAV1	1.0032	-1.0051
[6500]	TEK	<---	PTPRB	-0.014931	0.0020693
[6501]	CREBBP	--->	SMAD3	1.0006	-0.99697
[6502]	OPRM1	--->	GNAI2	0.9872	-0.98627
[6503]	PBX1	?	HOXA5	-0.11417	-0.11417
[6504]	KIT	--->	PLCE1	0.048803	-0.062106
[6505]	ADRB1	--->	GOPC	0.27699	-0.27482
[6506]	DLG4	--->	GUCY1A2	0.10173	-0.10343
[6507]	CDH9	?	CDH15	-0.058742	-0.058742
[6508]	NIN	--->	ABI1	0.04049	-0.047659
[6509]	MPDZ	<---	CAMK2A	-0.52215	0.50454
[6510]	BTK	--->	TLR6	0.38523	-0.41375
[6511]	PTK2B	<---	EGFR	-0.023945	0.12749
[6512]	BHLHE40	?	BHLHE41	-0.11925	-0.11925
[6513]	RND3	--->	ARHGAP5	0.35378	0.11034
[6514]	CAV1	<---	ERBB2	-0.28231	0.27589
[6515]	JAK1	--->	CSF2RB	0.34782	-0.36133
[6516]	RAB5A	<---	AGTR1	-0.17937	0.19171
[6517]	NFKBIA	<---	HSPB1	-1.0138	0.99919
[6518]	MTOR	--->	RPS6KB1	1.0159	-1.0002
[6519]	DTX2	<---	DTX1	-0.0032275	0.023717
[6520]	RAC2	--->	NCF2	0.12502	-0.13569
[6521]	CIITA	--->	ZXDC	0.15016	-0.14087
[6522]	ABL1	--->	MAPT	-0.01756	-0.082623
[6523]	DAXX	--->	TGFB1	0.12105	-0.2353
[6524]	GRK1	<---	RCVRN	-0.4439	0.43787
[6525]	EPB41L2	<---	DRD3	-0.19189	0.19515
[6526]	KCNA5	<---	SRC	-0.89226	1.0056
[6527]	BLM	<---	TP53BP1	-0.030501	0.042412
[6528]	MECR	--->	RARA	0.074075	-0.048313
[6529]	TNFSF11	<---	MAPK1	-0.29485	0.27854
[6530]	SMAD4	<---	HNF4A	-0.24224	0.26585
[6531]	LTBP3	<---	PRPF40A	-0.052088	0.015603
[6532]	JAK1	--->	STAT6	1.0006	-1.0137
[6533]	SRF	<---	CASP8	-0.41058	0.32148
[6534]	CCR5	--->	AFP	0.55385	-0.52416

[6535]	TNPO2	--->	NXF1	0.044367	-0.010158
[6536]	EDAR	<---	EDA	-0.18385	0.13993
[6537]	PDPK1	<---	ILK	-0.30915	0.35968
[6538]	CUL1	--->	SKP1	0.00027252	-0.0013415
[6539]	MEF2A	--->	ASCL1	0.060707	-0.059789
[6540]	IL8	<---	CXCR2	-0.46475	0.49595
[6541]	FBXL3	--->	SKP1	0.018801	-0.018355
[6542]	SH3RF1	<---	MAP3K11	-0.047173	0.16486
[6543]	ANGPTL3	--->	ITGA5	0.02492	-0.033901
[6544]	RPL30	<---	MAP3K14	-0.76568	0.74161
[6545]	PTK2	<---	FYN	-0.65057	0.81773
[6546]	ATF4	<---	CEBPG	-0.3007	0.31009
[6547]	RERE	--->	ALG13	-0.0028234	-0.0037907
[6548]	HNF4G	--->	COPS5	0.23695	-0.23085
[6549]	TOLLIP	<---	IL1RAP	-0.17047	0.16816
[6550]	FXYD3	<---	FBXL12	-0.23309	0.27196
[6551]	SERPINA3	<---	HOXB1	0.015262	0.018957
[6552]	AKT1	--->	TSC2	1.0085	-0.99774
[6553]	PPP4C	--->	RELA	0.6192	-0.61946
[6554]	PRKCA	--->	GRM1	0.91747	-0.9157
[6555]	CDK18	--->	CCDC85B	0.56074	-0.68539
[6556]	MAPK8IP3	<---	RAF1	-0.013575	-0.0027651
[6557]	CPNE4	--->	SKIL	0.48698	-0.48061
[6558]	LAMA5	<---	COL7A1	-0.0084486	-0.0049935
[6559]	AR	<---	PRMT1	-0.36996	0.36085
[6560]	PXN	<---	MATK	-0.91096	0.99333
[6561]	GLIS2	--->	CTNNB1	0.115	-0.12538
[6562]	ENAH	<---	ROBO4	-0.019243	0.0075546
[6563]	SNIP1	--->	MYC	0.18685	-0.18556
[6564]	MDM4	--->	RB1	0.1145	-0.12573
[6565]	DTNA	<---	UTRN	-0.022378	0.014832
[6566]	SVIL	<---	HSPA5	-0.72444	0.78939
[6567]	VRK2	--->	MAP2K7	0.34137	-0.059318
[6568]	ABI1	--->	SPTAN1	0.021593	0.0020083
[6569]	STAT5A	<---	JAK3	-1.0017	0.99962
[6570]	SMAD4	<---	FOXO4	-0.27389	0.2939
[6571]	ITGA5	<---	FN1	-0.02819	0.018501
[6572]	LRFN1	--->	DLG4	0.01134	-0.021348
[6573]	TTRAP	--->	TNFRSF8	0.021581	0.0049679
[6574]	FGFR1	<---	CREBBP	-0.054423	0.042697
[6575]	NCL	<---	CSNK2A2	-0.33816	0.31892
[6576]	RIPK2	<---	TRAF2	-0.59784	0.6021
[6577]	MCRS1	--->	TERT	0.043335	-0.051202
[6578]	FBXW7	--->	SKP1	0.11275	-0.11879
[6579]	ABL1	<---	JAK1	-0.1883	0.17191
[6580]	KLKB1	--->	HGF	0.0012258	-0.0091243
[6581]	DLG5	--->	CTNNB1	0.20409	-0.20847
[6582]	SKAP2	--->	FYB	0.041735	-0.059482
[6583]	MAPK3	--->	SREBF2	0.54159	-0.54127
[6584]	NTRK2	--->	SHC1	0.0897	-0.19951
[6585]	AUP1	--->	ITGA1	0.025399	-0.038359
[6586]	YWHAH	<---	PARK2	-0.0096756	0.033994
[6587]	MAPK1	--->	TGIF1	1.0017	-1.0013
[6588]	NCK2	--->	NCKIPSD	0.23696	-0.09833
[6589]	MAPK1	<---	PPP1CA	-0.067136	0.046927
[6590]	AMH	<---	EGFR	-0.20736	0.20212
[6591]	CRKL	--->	BCR	0.068429	-0.062951
[6592]	C7	<---	C5	-0.21163	0.21762
[6593]	PTPN1	--->	GRB2	0.70034	-0.9372
[6594]	$ZBTB8$	<---	LMO3	-0.16584	0.1799
[6595]	LYN	<---	MAP4K1	0.0088646	0.018188
[6596]	CBS	--->	FXR2	0.78118	-0.76918
[6597]	TRAF3	<---	MAP3K14	-0.06688	0.058215
[6598]	SOS1	--->	EPS8L1	-0.0024186	-0.017445
[6599]	TCF12	<---	ID2	-0.33696	-0.091025
[6600]	PAK1	<---	RAC1	-1.0053	0.99704
[6601]	ACVR1	--->	UBXN1	0.039846	-0.058064
[6602]	YWHAE	<---	MAP3K10	-0.5794	0.57548
[6603]	GIT1	<---	ADRBK1	-0.16512	0.14915
[6604]	HAT1	--->	SMAD7	0.37619	-0.33502
[6605]	DHX9	--->	SMN2	0.0045634	-0.00071951
[6606]	GATA3	<---	ZFPM2	-0.0069687	0.029291
[6607]	PTPN11	--->	JAK2	0.99157	-1.0118
[6608]	MAP4K1	--->	EGFR	0.15378	-0.15443
[6609]	RECQL4	--->	PRSS23	0.0093835	0.0059725
[6610]	RANGAP1	<---	RAN	-0.14398	0.14203

[6611]	DUSP9	--->	MAPK14	0.99169	-0.98663
[6612]	RASL12	--->	TGFBR1	0.010625	-0.037483
[6613]	PLD2	<---	OPRM1	-0.30236	0.29487
[6614]	CTNNB1	--->	MAGI1	0.00078961	-0.012485
[6615]	ITGB5	--->	ITGA5	-0.0024803	-0.010331
[6616]	KAT2B	--->	TAL1	0.21156	-0.21587
[6617]	TRAF6	--->	BMPR1B	0.39348	-0.41456
[6618]	TAOK1	<--->	MAP2K3	0.23429	0.23429
[6619]	TSSK6	--->	HSP90AB1	0.028434	-0.045733
[6620]	FZR1	--->	CCNB1	0.55627	-0.54356
[6621]	FOXO1	--->	THRB	0.057946	-0.066978
[6622]	PRKACA	--->	LIPE	0.53776	-0.55596
[6623]	CASP1	<---	IL1A	-0.084506	0.083786
[6624]	AKT1	--->	FOXO3	0.99774	-1.0046
[6625]	IRS1	<---	BCR	0.019127	0.059963
[6626]	GSC	<---	SMAD3	-0.14259	0.20304
[6627]	BMPR1B	<---	GDF5	-1.0007	0.99697
[6628]	RPS6KA1	--->	L1CAM	0.03878	-0.13396
[6629]	IL1RAP	--->	STAT3	0.48074	-0.51647
[6630]	MAPK1	--->	FRS2	1.0012	-1.0103
[6631]	SMAD7	<---	MAP3K7IP1	-0.74587	0.8096
[6632]	SIX3	<---	TLE3	-0.0020443	0.0047914
[6633]	SHC1	<---	RAPGEF1	-0.010092	-0.009051
[6634]	CHD3	--->	GIT1	0.0082204	-0.021375
[6635]	IL20RA	<---	IL20	-0.74442	0.74479
[6636]	ARNT	?	ARNT2	-0.019553	-0.019553
[6637]	RAF1	--->	YWHAH	0.022119	-0.051212
[6638]	CCNT1	--->	MYC	0.14807	-0.1379
[6639]	EPB41	--->	CRYAB	0.0078575	0.0015977
[6640]	BMP7	--->	ACVR1	0.60319	-0.60916
[6641]	PIAS3	--->	SKIL	0.17254	-0.17818
[6642]	PAX6	<---	RGS3	-0.16006	0.15789
[6643]	FGF7	<---	COL1A1	-0.019192	0.0089023
[6644]	LPHN2	<---	SHANK2	-0.094156	0.087413
[6645]	CTCF	<---	THRB	-0.094297	0.096115
[6646]	RET	--->	PRKAR2A	0.098881	-0.11284
[6647]	NCOA2	--->	CTNNB1	0.0637	-0.075596
[6648]	GRIN1	<---	HNRNPU	-0.17925	0.18626
[6649]	TEC	--->	DOK1	0.98722	-0.99462
[6650]	MAPK1	<---	DUSP6	-1.0004	1.0016
[6651]	HOXB7	<---	EP300	-0.18943	0.18505
[6652]	RYBP	--->	E2F3	0.095489	-0.06337
[6653]	RAD23A	--->	PSMD4	0.076162	-0.042104
[6654]	FCN2	--->	MASP1	0.0114	-0.018669
[6655]	ESR2	<---	PIAS1	-0.090133	0.095399
[6656]	NOV	<---	FBLN1	-0.45962	0.43394
[6657]	ELK1	<---	MAP2K3	-0.98814	1.0428
[6658]	PLD2	<---	PRKCA	-0.49286	0.48291
[6659]	CDK7	--->	ESR1	0.99834	-1.0079
[6660]	RAG1	<---	HMGB2	-0.22024	0.2138
[6661]	CEBPD	--->	SPI1	0.11084	-0.052983
[6662]	FOXG1	<---	FOXO1	0.010425	0.085024
[6663]	GRIN1	<---	PRKCE	-0.31676	0.28435
[6664]	AGFG2	--->	EPS15L1	0.016296	-0.0011105
[6665]	GSK3B	--->	AXIN2	0.26825	-0.28011
[6666]	CEP72	<---	LNX1	0.00098733	0.005777
[6667]	NRAS	<---	SMURF2	-1.0084	1.0007
[6668]	DAB2	<---	TGFBR1	-0.46556	0.43771
[6669]	TGFBR1	--->	VEPH1	0.28679	-0.34676
[6670]	SLC9A3R2	--->	RDX	0.061503	-0.056659
[6671]	DOK7	<---	MUSK	-0.18107	0.17894
[6672]	CCDC85B	--->	DOK5	0.1765	-0.14581
[6673]	FGF2	--->	CD44	0.068458	-0.07054
[6674]	TNFRSF18	--->	TRAF3	0.071664	-0.042613
[6675]	RELN	--->	VLDLR	0.096805	-0.10525
[6676]	LAMA1	<---	HSPG2	-0.0089712	-0.0047348
[6677]	SYVN1	--->	TP53	0.24323	-0.24209
[6678]	TCL1A	--->	HIST3H2A	0.33727	-0.29074
[6679]	HSP90B1	<---	ERBB2	-0.10588	0.095462
[6680]	ZBTB16	<---	WDR33	-0.042494	0.03316
[6681]	PLCD1	<---	RALA	-0.30267	0.31625
[6682]	RB1	<---	TBP	-0.020022	0.0065035
[6683]	PPP1R12C	<---	PPP1CB	-0.21108	0.20056
[6684]	KLK2	--->	SERPINA5	0.11833	-0.082185
[6685]	RAPSN	--->	CHRNB1	0.16858	-0.13534
[6686]	CTNNB1	<---	PPM1A	-0.3682	0.36371

[6687]	RHOXF2	--->	PITX1	-0.024292	-0.19656
[6688]	CTNND1	--->	ACTA1	0.14994	-0.16498
[6689]	SH2B2	<---	JAK3	-0.24383	0.21568
[6690]	FYN	--->	IRS1	1.0898	-0.93215
[6691]	AR	<---	GAPDH	-0.58216	0.5763
[6692]	BIRC6	<---	CDK1	-0.31134	0.27987
[6693]	HEY2	<---	PLSCR1	-0.37338	0.38715
[6694]	PSMD4	<---	UBQLN1	-0.042104	0.076162
[6695]	RBMX	--->	FXR2	0.036464	-0.0048144
[6696]	WASF2	--->	ACTR2	0.089124	-0.077881
[6697]	CCL15	<---	CCR1	-0.46475	0.49595
[6698]	NF2	--->	PXN	0.13306	-0.131
[6699]	SPOCK3	<--->	SPOCK2	0.074364	0.074364
[6700]	DLG1	--->	KCNA2	0.047736	-0.049447
[6701]	TFPI	--->	MMP12	0.20851	-0.1877
[6702]	USP7	--->	MARCH7	0.086379	-0.048977
[6703]	AXIN1	--->	APC	-0.0036235	-0.010123
[6704]	BMPER	--->	BMP2	0.71403	-0.67452
[6705]	PLXNB3	<---	RIT2	-0.37902	0.41051
[6706]	ABL1	<---	YWHAG	-0.08167	-0.068919
[6707]	SOX10	<---	UBE2I	-0.099835	0.1029
[6708]	PRKCA	--->	GRIN2B	0.22109	-0.2311
[6709]	WIPF1	<---	CTTN	-0.039333	0.03683
[6710]	CNOT1	<---	CNOT4	-0.04672	0.06238
[6711]	ACLY	<---	PRKACA	-0.72114	0.69525
[6712]	SERPINA1	<---	PRSS1	-0.082185	0.11833
[6713]	RHEBL1	--->	AKT1	0.09142	0.060359
[6714]	DLL1	--->	NOTCH2	-0.0071412	-0.011286
[6715]	TRAF6	--->	UBE2N	0.63617	-0.64329
[6716]	KCNJ11	<---	LDHA	-0.0019722	0.017576
[6717]	EPS15L1	<---	AGFG1	-0.0011105	0.016296
[6718]	TGFB2	--->	TGFBR2	0.28625	-0.21622
[6719]	MAPK12	--->	MAPT	0.60023	-0.67052
[6720]	DLG3	--->	CALM1	0.16268	-0.16314
[6721]	GRB2	--->	IRS2	0.2002	-0.20453
[6722]	PDPK1	--->	STRAP	0.47092	-0.32554
[6723]	SHC1	<---	IL4R	-0.19404	0.09263
[6724]	TRAF2	--->	RGS14	0.093164	-0.097869
[6725]	WASF2	<---	GRB2	-0.32992	0.32085
[6726]	GRAP2	--->	SOS2	1.0047	-0.99897
[6727]	IGFALS	--->	IGF1	0.032074	-0.030067
[6728]	PRDM2	--->	RB1	0.19005	-0.19907
[6729]	ANGPTL1	--->	NR2F6	0.073838	-0.048075
[6730]	CDK11B	--->	YWHAQ	0.62776	-0.66368
[6731]	ATXN1	<---	TOLLIP	-0.0064965	-0.003443
[6732]	PPIG	<---	YWHAG	-0.078442	0.10442
[6733]	RTKN	--->	GOPC	0.16894	-0.15896
[6734]	RARA	--->	RXRA	1.003	-0.99549
[6735]	AKT1	--->	ESR1	1.0083	-1.0039
[6736]	DDIT3	--->	BATF	0.26178	-0.18303
[6737]	PHLDA1	--->	PLSCR1	0.036678	-0.0088698
[6738]	SERPINA3	--->	APP	0.35887	-0.36875
[6739]	ADAMTS13	<---	VWF	-0.057225	0.071521
[6740]	PTK2	--->	STAT1	-0.039508	-0.060425
[6741]	NEUROG2	?	ASCL1	-0.65635	-0.65635
[6742]	GNAZ	<---	DRD2	-1.008	1.0129
[6743]	CD244	<---	CD48	0.27815	0.30986
[6744]	MET	<---	GAB1	-0.038307	0.026098
[6745]	TICAM2	--->	TICAM1	0.17817	0.023606
[6746]	NUMBL	--->	EPS15	0.022359	-0.034919
[6747]	NCOA3	<---	BMP7	-0.11664	0.10588
[6748]	SKP1	--->	FBXO8	0.055251	-0.044878
[6749]	TRAF3	--->	LTBR	-0.038149	-0.06112
[6750]	APBB1	--->	ATXN1	0.047889	-0.055423
[6751]	ATR	<---	FLT1	-0.054258	0.047044
[6752]	LPAR2	--->	ARHGEF12	0.10316	-0.106
[6753]	SRCAP	<---	ATG9A	-0.0065139	-0.001001
[6754]	SDCBP2	<---	NFASC	-0.22146	0.21712
[6755]	TSHR	--->	CALR	0.35882	-0.3494
[6756]	SPI1	<---	DNMT3B	-0.18595	0.22812
[6757]	NEK1	--->	ATRX	0.19614	-0.2142
[6758]	MAP4K1	--->	CRK	0.12568	-0.125
[6759]	IGSF5	--->	LNX1	0.20348	-0.20684
[6760]	CSNK2A1	--->	PPP1R8	0.7025	-0.72853
[6761]	FBLN1	<---	FGB	0.0019349	0.033072
[6762]	DFNB31	--->	MYO15A	0.099775	-0.095328

[6763]	IL1R2	--->	IL1RN	0.1537	-0.13578
[6764]	ATF3	<---	NFKB1	-0.47747	0.47635
[6765]	PPP1CA	--->	EIF2AK2	0.046927	-0.067136
[6766]	TIMP1	--->	MMP2	0.21399	-0.19321
[6767]	CREBBP	--->	DDX5	0.064201	-0.074615
[6768]	CASP3	--->	CAD	0.36691	-0.43968
[6769]	ZNF238	<---	DNMT3A	-0.25966	0.24764
[6770]	GIT1	<---	KIF1A	-0.094935	0.083511
[6771]	NLRC4	<---	NOD2	-0.065505	-0.019771
[6772]	GRIN1	<---	PRKCG	-0.37606	0.36861
[6773]	UBE2L3	--->	PARK2	0.12521	-0.091377
[6774]	CAMKK1	--->	AKT1	1.007	-1.0026
[6775]	FHL3	--->	ACTB	0.54878	-0.54521
[6776]	YWHAE	--->	TGFB1	0.32837	-0.48526
[6777]	MRAS	--->	RALGDS	0.98547	-1.0084
[6778]	RB1	<---	CDK14	-1.0015	0.98937
[6779]	NIN	<---	UBE2I	-0.10642	0.14117
[6780]	DDR2	<---	SRC	-0.17141	0.74642
[6781]	KHDRBS3	--->	KHDRBS1	-0.01402	-0.071085
[6782]	TYRO3	--->	PIK3R1	0.20209	-0.27441
[6783]	TES	--->	ACTN1	0.014972	-0.010035
[6784]	GRAP2	--->	BLNK	0.56823	-0.44654
[6785]	MAPK3	--->	UBTF	0.92033	-0.89929
[6786]	HNF4A	--->	HNF1A	0.36058	-0.35236
[6787]	FBXO32	<---	SKP1	-0.15846	0.15247
[6788]	VWF	<---	COL1A1	-0.010208	-0.0013471
[6789]	SLC2A4	<---	C1QTNF5	-0.16718	0.18483
[6790]	CCR8	--->	CCL16	0.49595	-0.46475
[6791]	STMN1	--->	STAT3	0.54446	-0.59635
[6792]	SFN	--->	BAX	0.62138	-0.61447
[6793]	GRIN1	<---	SPTAN1	-0.028817	0.017968
[6794]	SLC9A3R1	--->	SLC22A4	0.37001	-0.34102
[6795]	CCR2	--->	CCL11	0.49595	-0.46475
[6796]	SMAD3	<---	PCDH1	-0.31274	0.39568
[6797]	PACSIN3	<---	SOS1	-0.32202	0.31511
[6798]	PRKACA	<---	PTPN7	-1.009	1.0061
[6799]	BAG1	--->	BCL2	0.68684	-0.68331
[6800]	ATN1	--->	PFKL	0.037322	-0.03957
[6801]	MST1R	--->	YWHAE	0.39852	-0.41561
[6802]	GALR2	--->	GNAQ	1.0129	-1.008
[6803]	NGF	--->	NTRK1	1.0012	-1.0001
[6804]	PPM1B	--->	CDK2	1.0075	-0.9963
[6805]	RNF216	<---	RIPK1	-0.14565	0.39951
[6806]	RET	--->	STAT3	0.51895	-0.63828
[6807]	E4F1	--->	RB1	0.014272	-0.025849
[6808]	SRF	--->	RXRG	0.047035	-0.1777
[6809]	GRB2	--->	CD22	0.48894	-0.54698
[6810]	EZH2	--->	VAV1	0.039229	-0.22837
[6811]	CPSF6	--->	ARMC7	0.21728	-0.17983
[6812]	LEPR	<---	LEP	-1.007	0.99687
[6813]	GALNT1	--->	MUC1	0.17779	-0.17757
[6814]	PLK3	<---	MAPK1	0.0029423	0.19171
[6815]	DVL1	<---	CSNK1D	-0.9989	0.99947
[6816]	TRA2B	--->	IVNS1ABP	0.066603	-0.036142
[6817]	CD8A	--->	CD3D	0.13452	-0.11652
[6818]	ARNT	<---	SIM2	-0.05797	0.043453
[6819]	YWHAB	--->	WDR77	0.2164	-0.19355
[6820]	DAB1	--->	APP	0.35975	-0.36965
[6821]	HNF4A	--->	TP53	0.24837	-0.25108
[6822]	RIPK3	--->	TICAM1	0.030203	-0.047494
[6823]	GNAZ	<---	RAP1GAP	-0.34717	0.34131
[6824]	PTPN2	--->	STAT5A	1.0055	-1.005
[6825]	MBD2	<---	RBBP4	0.009301	0.010856
[6826]	SOX7	--->	SMAD5	0.20729	-0.075397
[6827]	CASP4	<---	IL18	-0.065744	0.073886
[6828]	PIK3R1	<---	IL1R1	-0.1818	0.15294
[6829]	MAG	<---	COL9A2	-0.0093685	-0.0036591
[6830]	PRPF40A	--->	PIAS4	0.077172	-0.066585
[6831]	S1PR1	<---	HTR1A	0.15705	0.35144
[6832]	NPHS1	<---	FYN	-0.0667	-0.02858
[6833]	PSEN1	--->	JUP	0.076926	-0.079155
[6834]	SHC1	<---	GRB7	-0.19651	0.17995
[6835]	GULP1	--->	LRP1	0.14403	-0.15309
[6836]	DMPK	--->	GABARAP	0.82871	-0.85717
[6837]	CSNK1E	--->	PER3	0.72042	-0.58384
[6838]	RAET1G	?	ULBP1	-0.082834	-0.082834

[6839]	PSTPIP2	<---	PTPN18	-1.2369	1.243
[6840]	CALR	--->	LRP1	0.14339	-0.15324
[6841]	CSNK2A1	--->	BRCA1	0.32242	-0.34057
[6842]	SH3BP2	--->	PLCG1	0.2112	-0.43897
[6843]	NR1H2	<---	UBE2I	-0.16643	0.1925
[6844]	HOMER1	--->	GRM1	0.12984	-0.11469
[6845]	RNPS1	<---	UBQLN4	-0.057469	0.089135
[6846]	SMAD1	--->	ZNF510	0.17167	-0.19115
[6847]	MAPK14	--->	NFATC4	0.2651	-0.3326
[6848]	SHC1	<---	DDR2	-0.517	0.23668
[6849]	PTPN18	<---	ERBB2	-0.15162	0.14571
[6850]	CAV1	<---	TRAF6	-0.68806	0.67965
[6851]	HSPH1	--->	PAFAH1B1	0.58812	-0.56869
[6852]	MINK1	--->	ABI1	0.0035476	-0.047061
[6853]	SFTPA1	<---	C1QA	-0.0067805	0.023513
[6854]	PTPRH	--->	LCK	0.99893	-0.99669
[6855]	CREBBP	<---	GAK	-0.19044	0.18399
[6856]	EIF2AK2	--->	TP53	1.0002	-0.99962
[6857]	P2RY2	--->	ITGB3	0.1188	-0.12597
[6858]	CIT	--->	DLG4	0.053508	-0.08154
[6859]	MYST2	--->	WDR33	0.043199	-0.039799
[6860]	DOK3	<---	LYN	-1.1853	1.3209
[6861]	TP53	<---	ERCC6	-0.21719	0.21861
[6862]	CHUK	--->	TNFAIP3	0.15626	-0.29571
[6863]	FN1	<---	COL13A1	-0.061633	0.057845
[6864]	CCBP2	--->	CCL28	0.49595	-0.46475
[6865]	NCOA3	<---	RARB	-0.13412	0.12213
[6866]	NR0B2	--->	RXRA	-0.00165	-0.38321
[6867]	ATRNL1	<---	MC4R	-0.067047	0.068057
[6868]	PIAS1	--->	GATA4	0.17317	-0.15881
[6869]	ABCD4	<---	PEA15	-0.15288	0.19423
[6870]	CAV1	<---	RHOA	-0.37866	0.40294
[6871]	LYN	--->	PILRB	0.843	-0.80069
[6872]	AXL	--->	PIK3R1	0.19756	-0.27099
[6873]	NCOA3	<---	TBP	-0.013187	-0.00068014
[6874]	PJA1	--->	ASCC2	0.23044	-0.22972
[6875]	SHOC2	--->	RAF1	0.014135	-0.028819
[6876]	DNASE1	--->	ACTA1	0.29006	-0.29329
[6877]	PORCN	--->	WNT3A	1.0005	-0.9854
[6878]	CSNK2A2	--->	ARRB2	0.42184	-0.44306
[6879]	SOS1	<---	CRK	-0.61023	0.60392
[6880]	EP300	--->	SMAD3	1.0006	-0.99697
[6881]	PPFIA1	<--->	PPFIA3	0.049612	0.049612
[6882]	UBE2I	--->	MITF	0.45272	-0.44005
[6883]	GABARAPL1	<---	OPRK1	-0.28257	0.29387
[6884]	MYH2	<---	DGKD	-0.014039	0.00043366
[6885]	SPHK2	<---	FYN	-0.90413	1.0649
[6886]	CHEK2	--->	E2F1	0.26761	-0.2868
[6887]	TNFRSF1A	<---	SYK	-0.24296	0.35942
[6888]	NPPC	--->	NPR3	0.041351	-0.0068276
[6889]	GNAI2	<---	CXCR2	-1.008	1.0129
[6890]	SPEN	<---	HDAC2	-0.016764	0.019906
[6891]	SLC9A3R2	--->	SLC22A5	0.37001	-0.34102
[6892]	BAK1	--->	BCL2	-0.23385	-0.7984
[6893]	GRK5	--->	TACR1	0.31971	-0.40764
[6894]	PPM1A	--->	PIK3R1	0.20182	-0.27016
[6895]	SMURF2	--->	SMAD7	0.9934	-1.0067
[6896]	EGFR	--->	ALCAM	0.36169	-0.37708
[6897]	AATF	<---	SP1	-0.11363	0.10222
[6898]	GAPDH	<---	RAB2A	-0.22784	0.24085
[6899]	FLNA	--->	GRIK3	0.096074	-0.079231
[6900]	KCNA2	<---	CNTNAP2	-0.021947	0.055838
[6901]	MAPK14	<---	CSNK2A2	-0.9992	1.0074
[6902]	LCK	--->	CD48	0.45518	-0.46844
[6903]	DCN	<---	C1QA	-0.030712	0.023329
[6904]	LNX2	--->	NUMB	0.032459	-0.026869
[6905]	ITGAE	<---	CHFR	-0.046728	0.033852
[6906]	PKP4	--->	CDH5	0.31989	-0.319
[6907]	GNAO1	<---	RGS1	-1.0015	1.0012
[6908]	CD19	<---	SOS1	-0.35365	0.34739
[6909]	RALA	--->	CALM1	0.73657	-0.70265
[6910]	SCN5A	<---	INADL	-0.013537	0.050181
[6911]	PZP	--->	TGFB2	0.34309	-0.29287
[6912]	CDK20	--->	CCNH	1.083	-1.1093
[6913]	VCAN	<---	HAPLN1	-0.0096314	-0.007525
[6914]	BCAR1	<---	ABL1	-0.22732	0.1318

[6915]	KLK6	--->	SERPINC1	0.11833	-0.082185
[6916]	UBTF	<---	CSNK2A1	-0.89929	0.92033
[6917]	RNF8	<---	UBE2E1	-0.10861	0.11055
[6918]	KHDRBS1	<---	CREBBP	-0.076111	0.065712
[6919]	TOPORS	--->	TP53	0.36538	-0.35369
[6920]	DYRK1B	--->	RANBP9	0.41421	-0.43537
[6921]	NRXN1	--->	MACF1	0.14785	-0.14256
[6922]	CASP9	<---	XIAP	-0.036843	0.035713
[6923]	ATXN1	<---	U2AF2	-0.032318	0.028442
[6924]	CCND3	<---	MAPK4	-1.0774	1.0544
[6925]	ATN1	--->	JAG2	0.050515	-0.063562
[6926]	ATN1	--->	MEGF11	-0.0067573	-0.0070629
[6927]	F2RL2	--->	F2	0.28486	-0.27153
[6928]	MAPK14	--->	FLNA	0.30428	-0.47425
[6929]	MAP4K4	--->	MAP3K7	0.02742	0.0093314
[6930]	JAK2	--->	IFNGR1	0.15015	-0.082156
[6931]	CREM	<---	CREB3L1	-0.31158	0.12426
[6932]	EEF1G	<---	ADAP2	0.0042122	0.030689
[6933]	PIK3R2	<---	CRKL	-0.11093	0.10927
[6934]	THRB	<---	JMJD1C	-0.069928	0.10027
[6935]	IKBKB	--->	TNFAIP3	0.15626	-0.29571
[6936]	GNB2L1	--->	ITGB5	0.015726	-0.018518
[6937]	TCF12	<---	TCF3	-0.064851	-0.033747
[6938]	PGR	<---	APEX2	-0.046671	0.072293
[6939]	RPH3A	<---	YWHAH	-0.0059882	0.018907
[6940]	NCSTN	--->	ABL1	-0.028119	-0.035812
[6941]	CTBP2	<--->	CTBP1	0.012716	0.012716
[6942]	BNIP2	--->	FGFR1	0.24882	-0.24946
[6943]	CBLB	--->	VAV1	0.027285	-0.065069
[6944]	WAS	<---	ENAH	-0.021569	-0.0088126
[6945]	GIPC1	<---	TPBG	-0.1018	0.13232
[6946]	LATS2	<---	JUB	-1.0005	0.98784
[6947]	RND1	--->	GRB7	0.38266	-0.44893
[6948]	FYN	--->	CNN3	1.0728	-0.91195
[6949]	UVRAG	<---	VPS11	-0.071616	0.090791
[6950]	SKIL	<---	SETDB1	-0.097523	0.092803
[6951]	SDCBP	<---	SOX4	-0.0060162	0.021545
[6952]	TRIM33	--->	TRIM24	0.070423	-0.0093699
[6953]	ZNF263	<---	SUFU	-0.0091921	0.0090887
[6954]	SYT11	<---	PARK2	-0.02416	0.064826
[6955]	EGFR	--->	EPS15	0.99691	-0.9942
[6956]	S1PR1	<---	PDGFRB	-0.02399	0.014674
[6957]	PRKCA	--->	GSK3A	0.22826	0.12473
[6958]	EZH2	--->	CCDC85B	0.052772	-0.021629
[6959]	DHX9	<---	SRPK1	-0.44264	0.42167
[6960]	MAP3K3	--->	IKBKB	1.0006	-1.0022
[6961]	CDH23	--->	MYO7A	0.046865	-0.042915
[6962]	SIRT1	--->	HES1	0.049867	-0.062291
[6963]	NLK	--->	MYB	0.092606	-0.10814
[6964]	SRC	--->	RXRA	0.37227	-0.38708
[6965]	CFP	<---	CFB	-0.051328	0.064899
[6966]	ATXN1	<---	ZNF609	-0.034447	0.031107
[6967]	GADD45G	<---	CDK1	-0.76568	0.74161
[6968]	KPNA2	--->	TP53	0.34944	-0.33871
[6969]	DMXL2	<---	RAB3A	-0.10712	0.10875
[6970]	PCBP2	<---	FLNA	0.0076104	0.026577
[6971]	MCL1	<---	HUWE1	-0.18362	0.17412
[6972]	KDR	--->	YES1	0.84641	-0.66543
[6973]	ABL1	<---	BTK	-0.098628	-0.018782
[6974]	INPP4A	--->	PIK3R1	0.19162	-0.29653
[6975]	RAP2A	--->	TGFBR1	0.010625	-0.037483
[6976]	WWTR1	--->	YWHAE	0.45536	-0.45849
[6977]	ITPKA	--->	ACTA1	0.7654	-0.76451
[6978]	RGPD5	<---	RAN	-0.30502	0.33562
[6979]	CDK9	--->	CCNT2	1.083	-1.1093
[6980]	TJP1	<---	CTTN	-0.03301	0.039657
[6981]	TGFBRAP1	--->	SMAD4	0.42399	-0.34146
[6982]	MYOG	?	ID1	-0.65635	-0.65635
[6983]	MAPK8IP2	<---	MAP2K7	-0.30961	0.2909
[6984]	ROCK1	--->	EZR	-0.0022587	-0.012307
[6985]	PLSCR1	<---	DTX2	-0.055999	0.049136
[6986]	JAG1	--->	NOTCH2	0.0042068	-0.014204
[6987]	OPRM1	--->	GNAI1	0.9872	-0.98627
[6988]	SMURF2	--->	SPG20	0.70647	-0.71034
[6989]	WDR65	--->	FOS	0.09914	-0.15726
[6990]	RIN3	<---	RAB5C	-0.33568	0.34965

[6991]	CEACAM1	<---	INSR	-0.09114	0.079165
[6992]	RPS6KA5	--->	STAT1	0.020187	-0.03925
[6993]	PPP1R15B	<---	PPP1CA	-0.2011	0.19414
[6994]	PRKCA	--->	CYTH2	1.2556	-1.0819
[6995]	RIPK2	<---	CASP8	-0.69678	0.68902
[6996]	UBTF	--->	RBL2	0.31365	-0.32205
[6997]	GDF5	--->	ACVR1	0.60319	-0.60916
[6998]	LPHN1	<---	ROS1	-0.34299	0.33004
[6999]	CNTFR	<---	PLSCR1	-0.019844	0.028024
[7000]	ABL1	<---	CRK	-0.99908	0.99616
[7001]	HIST3H3	<---	CBX1	-0.19484	0.24617
[7002]	ABTB2	<---	CUL3	-0.11192	0.10856
[7003]	ITGB8	--->	ITGAV	0.0021599	-0.015012
[7004]	IKZF3	<---	HRAS	-0.27115	0.27242
[7005]	PTPN11	--->	GRB2	0.43839	-0.5864
[7006]	PTPRJ	--->	JUP	0.21235	-0.2222
[7007]	PRG2	<---	CSF2	-0.14565	0.17654
[7008]	WASF1	<---	CRK	-0.16017	0.12946
[7009]	CDH5	<---	FGA	-0.077887	0.093312
[7010]	PIK3R1	<---	ERBB2	-0.17801	0.14447
[7011]	MYLK	--->	ACTC1	0.35726	-0.37945
[7012]	DLG4	--->	FZD7	0.11867	-0.14014
[7013]	E2F2	<---	CDK3	-0.75826	0.73009
[7014]	MEGF10	<---	CADPS2	-0.0069731	-0.0065688
[7015]	SMARCC1	--->	CCNE1	0.64939	-0.63597
[7016]	ARHGEF12	<---	GNA12	-0.047502	0.041194
[7017]	ERBB3	<---	EGF	-0.99332	0.99244
[7018]	CCRL1	--->	CCL11	0.49595	-0.46475
[7019]	SMURF1	--->	SMAD6	0.9934	-1.0067
[7020]	LYN	--->	HCLS1	0.99631	-1.0146
[7021]	AKT1	--->	CDKN1A	0.99717	-0.99814
[7022]	MXI1	?	MAX	-0.65635	-0.65635
[7023]	BCL2	<---	PSEN1	-0.77257	0.78024
[7024]	RAB34	--->	SMAD1	0.52759	-0.54836
[7025]	S1PR5	--->	GNAI1	1.0129	-1.008
[7026]	PGR	<---	SRC	-0.55261	0.61826
[7027]	TGM2	<---	SPARC	-0.041752	0.07584
[7028]	DAB1	<---	PTPN11	-0.6558	0.8821
[7029]	MAPK10	--->	MAPK8IP3	0.086975	-0.12532
[7030]	CTBP2	<---	MECOM	-0.16004	0.15176
[7031]	DAZL	?	DAZAP1	-0.016298	-0.016298
[7032]	RXFP1	--->	RLN1	0.15462	-0.14912
[7033]	MAP3K7IP2	--->	MAP3K7	0.99986	-0.99934
[7034]	TIAM1	<---	PRKCE	-0.18992	0.18813
[7035]	CCBP2	--->	CCL27	0.49595	-0.46475
[7036]	TANC1	<---	DLGAP1	-0.13089	0.14964
[7037]	SMAD4	--->	AR	0.33034	-0.33543
[7038]	PRKCG	--->	NOXA1	0.58363	-0.60991
[7039]	IL1RAP	--->	PRPF40A	0.20531	-0.18956
[7040]	FGFR4	<---	FGF1	-0.99871	1.0053
[7041]	IKBKG	<---	PRKCQ	-0.44613	0.46236
[7042]	SYK	<---	FYN	-1.0044	1.0025
[7043]	CDK12	--->	SFRS1	0.72994	-0.75755
[7044]	CSNK1G1	--->	FOXO1	0.12051	-0.13229
[7045]	TYRO3	--->	PROS1	0.094139	-0.10105
[7046]	PTPN6	--->	CD72	1.0035	-1.0141
[7047]	ICAM5	--->	ITGAL	0.08302	-0.094837
[7048]	PCSK9	--->	LDLR	0.14845	-0.15341
[7049]	ATN1	--->	FBLN1	0.039508	-0.041614
[7050]	CCL22	<---	VCAM1	-0.22304	0.21853
[7051]	NAIP	--->	HPCA	0.2903	-0.25599
[7052]	SKIL	<---	SNRNP70	-0.40157	0.4051
[7053]	TP73	<---	ITCH	-0.10774	0.095712
[7054]	BMP15	--->	BMPR1B	0.32284	-0.34476
[7055]	PBK	--->	ARAF	0.98888	-1.0049
[7056]	MAPK1	--->	GRB10	0.17357	-0.34335
[7057]	ADCYAP1R1	<---	CALM1	-0.24765	0.25258
[7058]	PKMYT1	<--->	CDK1	0.23429	0.23429
[7059]	RNF111	--->	SMAD6	1.0166	-0.99999
[7060]	RNF40	<---	UBE2L6	-0.17337	0.20782
[7061]	PITX1	--->	EGR1	0.012207	-0.013281
[7062]	DVL2	<---	FZD4	-0.081485	0.060144
[7063]	PSTPIP1	--->	WAS	0.15587	-0.18611
[7064]	CDKN1B	<---	COPS5	-0.11352	0.11472
[7065]	KDR	--->	TIMP3	0.6974	-0.64029
[7066]	SH3GLB1	--->	DNM2	0.071535	-0.080916

[7067] SKP1 ---> NFKBIE 0.47933 -0.46943
[7068] DLG2 <--- MAPK12 -0.21461 0.1619
[7069] FADD <--- TNFRSF10A -0.86277 0.89012
[7070] DAZ4 ? DAZL -0.016298 -0.016298
[7071] ABCA1 <--- SNTB2 -0.0080068 0.005539
[7072] PTPN1 ---> TRPV6 1.1419 -1.144
[7073] EPB41L2 ---> ACTA1 0.1724 -0.17979
[7074] PARVB <--- ARHGEF6 -0.047373 0.02323
[7075] ZAP70 <--- PTPN6 -1.0074 1.0106
[7076] GREM2 ---> BMP2 0.56685 -0.51806
[7077] FBLN1 ---> NID1 0.01137 -0.025285
[7078] GALNT12 ---> MUC1 0.17779 -0.17757
[7079] DUSP16 ---> MAPK8 1.0016 -1.0004
[7080] FGFR4 <--- FGF5 -0.99871 1.0053
[7081] DOCK10 <--- RHOJ -0.032551 0.020149
[7082] MET ---> DAPK3 0.23722 -0.27602
[7083] PRKCB ---> IKBKB 0.99661 -0.98908
[7084] RAF1 <--- STK3 -0.15284 0.15521
[7085] NCK2 ---> EEF1G 0.21371 -0.21594
[7086] RAP1GAP ---> MLLT4 0.056117 -0.02271
[7087] CD209 <--- ICAM2 -0.095662 0.11146
[7088] HNRNPA2B1 <--- CSNK2A1 -0.75755 0.72994
[7089] SOCS3 ---> RASA1 0.34543 -0.15328
[7090] LYN ---> DAPP1 1.4511 -1.0668
[7091] MAPK3 ---> PRKCZ 0.1627 0.012708
[7092] INHBC ---> ACVR2A 1.0057 -0.99943
[7093] GRM2 <--- GNAQ -0.19572 0.21162
[7094] UHRF2 <--- CDK2 -0.78141 0.75574
[7095] PTPN2 <--- SRC -0.37818 0.26821
[7096] ETV4 <--- EP300 -0.1922 0.18812
[7097] UBTF ---> TBP 0.004514 -0.016791
[7098] BLM <--- CHEK1 -0.44264 0.42167
[7099] PRKACA ---> HNRNPD 0.72994 -0.75755
[7100] EP300 ---> NFATC2 0.0034296 -0.016462
[7101] MAPK8 ---> JUNB 1.0022 -1.0148
[7102] MYC <--- ELF3 -0.081924 0.084693
[7103] GLI1 <--- DYRK1A -1.0035 0.98544
[7104] GRK5 ---> AVPR1A 0.21152 -0.27051
[7105] PRKAA2 ---> HNF4A -0.009204 -0.11591
[7106] BAG3 ---> PLCG1 0.36472 -0.41247
[7107] ANKRD11 <--- HDAC3 -0.13768 0.15653
[7108] LAT <--- SLA -0.11406 0.15259
[7109] FBN1 ---> VCAN -0.0011734 -0.012762
[7110] MAPK3 <--- LYN -1.2776 1.3862
[7111] CSNK1E ---> PER2 0.72042 -0.58384
[7112] PRKAR1A ---> AKAP1 0.28956 -0.27544
[7113] CIC <--- SETD2 -0.11812 0.13298
[7114] ARHGAP5 ---> ITGA5 0.025488 -0.037852
[7115] PTPN6 ---> JAK2 0.99157 -1.0118
[7116] BCAR1 ---> RAPGEF1 0.0064049 -0.01783
[7117] CDC5L <--- PPP1CA -0.21487 0.20779
[7118] MAPK3 ---> MAPK14 1.0074 -0.9992
[7119] WAS <--- FGR -1.0011 0.99511
[7120] MEF2C <--- EP300 -0.034917 0.022427
[7121] PDGFB ---> PDGFRA 1.0139 -0.99672
[7122] MTNR1B ---> GNB4 0.87375 -0.84712
[7123] MYH7 <--- TEAD3 -0.018323 0.0060129
[7124] FLII ---> ACTB 0.24681 -0.24807
[7125] BCL2L13 <--- GABARAPL2 -0.41352 0.42789
[7126] STAMBPL1 ---> SMAD7 0.99487 -0.99775
[7127] SYTL4 <--- CCDC85B 0.012422 0.022981
[7128] CREBBP ---> NFIA 0.41326 -0.41533
[7129] STAT5A <--- SRC -0.56898 0.57328
[7130] MAPK1 <--- DUSP2 -1.0004 1.0016
[7131] CAMK2B ---> COL4A3 0.045455 -0.059968
[7132] UBE2I ---> ATF2 0.37217 -0.3721
[7133] DUSP1 ---> HSPA4 0.067029 -0.045606
[7134] ERBB2 ---> PIK3C2A 0.013224 -0.025394
[7135] E2F1 <--- CREBBP -0.11647 0.10832
[7136] SMAD4 <--- PARD3 -0.12329 0.14536
[7137] SHC1 <--- EPOR -0.19034 0.086569
[7138] CSNK1E ---> BID 0.72833 -0.59006
[7139] PPM1A ---> GRM3 0.76481 -0.74426
[7140] FLNA ---> CCNB1 0.62484 -0.61134
[7141] DTX3L <--- UBE2D2 -0.02412 0.017553
[7142] TRIP10 ---> ARHGAP17 0.049653 -0.054714

7145	[7143]	CSNK2A2	--->	MYCN	1.1385	-1.1233
7146	[7144]	CSNK2A1	--->	STMN1	1.0058	-0.99021
7147	[7145]	SKIL	<---	ZFP106	-0.37387	0.37767
7148	[7146]	TESK1	--->	YWHAB	0.62776	-0.66368
7149	[7147]	CSNK2A1	--->	RGS19	0.28697	-0.32153
7150	[7148]	PLCG2	<---	LIME1	-0.38594	0.2238
7151	[7149]	UBE2I	--->	AR	1.0017	-0.99481
7152	[7150]	TNFRSF10B	--->	RIPK1	0.15215	-0.2843
7153	[7151]	PPP1R9B	<---	PPP1CA	-0.21277	0.20627
7154	[7152]	PTPN6	--->	PIK3R1	0.32212	-0.46461
7155	[7153]	LAT	--->	SH3BP2	-0.0075158	-0.12994
7156	[7154]	HIF1A	--->	NR4A1	0.51159	-0.51561
7157	[7155]	SLC8A3	<---	YWHAE	-0.092105	0.10171
7158	[7156]	NFIC	<---	RFX1	-0.054792	0.051672
7159	[7157]	APBB1IP	--->	PFN1	1.0022	-0.99183
7160	[7158]	SVIL	--->	ACTN1	0.034373	-0.021527
7161	[7159]	SIGLEC11	<---	PTPN11	-0.13709	0.14161
7162	[7160]	ABI3	<---	PAK2	-0.13366	0.32915
7163	[7161]	SLC9A3	<---	SHANK2	-0.04293	0.033672
7164	[7162]	FRS3	--->	FGFR1	0.18926	-0.19152
7165	[7163]	YWHAQ	<---	MST1R	-0.41561	0.39852
7166	[7164]	ALB	--->	RYR2	0.3645	-0.37025
7167	[7165]	CBX5	<---	SMARCA4	-0.010114	0.0026037
7168	[7166]	RAN	<---	SMURF2	-1.0084	1.0007
7169	[7167]	CDK14	--->	YWHAQ	0.62776	-0.66368
7170	[7168]	GNB2L1	<---	DDX19B	-0.076481	0.091156
7171	[7169]	STAT3	<---	HES1	-0.032191	0.014285
7172	[7170]	TGFB1	<---	CCL3	-0.44261	0.28949
7173	[7171]	RASGRP2	--->	KRAS	1.0003	-1.007
7174	[7172]	FGFR4	<---	FGF3	-0.99871	1.0053
7175	[7173]	PXN	<---	FYN	-0.91096	0.99333
7176	[7174]	CDK8	--->	CDKN2B	1.3062	-1.3222
7177	[7175]	TRIM24	--->	ESR2	0.11471	-0.088819
7178	[7176]	MET	--->	PLCG1	0.49669	-0.67031
7179	[7177]	FHL3	<---	SRF	-0.037159	0.05921
7180	[7178]	TNC	--->	FN1	0.0030629	-0.01967
7181	[7179]	BMPR1A	<---	GDF5	-1.0007	0.99697
7182	[7180]	ITGB3	<---	FN1	-0.039546	0.035837
7183	[7181]	SNTG1	--->	DGKZ	0.0034744	0.0034359
7184	[7182]	ARHGAP1	--->	PIK3R1	0.0030351	-0.17191
7185	[7183]	MET	<---	CASP3	-0.40402	0.30934
7186	[7184]	IGFALS	<---	PDCD6	-0.046686	0.048977
7187	[7185]	EP300	--->	POU3F2	0.075802	-0.085965
7188	[7186]	PARD6A	<---	PRKCH	-0.29922	0.24953
7189	[7187]	HUWE1	--->	CDKN2A	0.077553	-0.086915
7190	[7188]	CDON	--->	CDH2	0.12937	-0.13991
7191	[7189]	PRKCG	--->	EIF4E	0.94696	-0.94609
7192	[7190]	JUN	<---	ESR1	-0.27062	0.24409
7193	[7191]	PILRA	<---	PTPN11	-0.52299	0.60343
7194	[7192]	PRKCA	--->	RRAD	1.0123	-0.99154
7195	[7193]	TJP1	--->	ACTA1	0.16431	-0.17038
7196	[7194]	CARD6	<---	RIPK2	-0.4509	0.13986
7197	[7195]	BCL2A1	<---	GRB2	-0.63098	0.6335
7198	[7196]	HSP90AB1	--->	ESR1	0.052711	-0.058028
7199	[7197]	UBE2L3	<---	UBE3A	-0.17948	0.18395
7200	[7198]	RPS6KA3	<---	STK11	-0.74727	0.78966
7201	[7199]	ESR2	<---	YWHAH	-0.092332	0.11204
7202	[7200]	ADAMTS4	<---	HP	-0.0055546	0.0085445
7203	[7201]	CASP8	--->	BCL2L1	0.62187	-0.71598
7204	[7202]	CBL	--->	TRAF6	0.016971	-0.029553
7205	[7203]	PRAM1	<---	LYN	-1.0646	1.2078
7206	[7204]	LILRB2	<---	PTPN6	-0.17152	0.29453
7207	[7205]	GDF9	--->	ACVR2A	0.21823	-0.24164
7208	[7206]	RNF7	<---	CUL5	-0.11034	0.12225
7209	[7207]	ELF1	<---	HOXC13	-0.076678	0.11684
7210	[7208]	CUL9	<---	UBE2L3	-0.11067	0.14316
7211	[7209]	ARHGEF3	<---	RHOB	-0.29753	0.32945
7212	[7210]	THBS2	<---	MMP2	-0.15886	0.21165
7213	[7211]	PRPF40B	--->	HTT	0.32543	-0.29315
7214	[7212]	MAPK8	--->	YWHAZ	0.62776	-0.66368
7215	[7213]	GRIN2B	<---	CAPN1	-0.08811	0.077763
7216	[7214]	TGFBR3	<---	ARRB2	-0.06695	0.068501
7217	[7215]	CSPG4	<---	C1QA	-0.072562	0.066388
7218	[7216]	NCOA1	<---	RARG	-0.043163	0.034596
7219	[7217]	CXCR1	--->	CXCL6	0.49595	-0.46475
7220	[7218]	GDF5	--->	BMPR2	1.0057	-0.99943

[7219]	RBAK	--->	RB1	0.12611	-0.13512
[7220]	NCOA2	<---	ESR1	-0.043163	0.034596
[7221]	CAPN2	--->	F2RL1	0.075727	-0.071443
[7222]	TRIM54	<--->	TRIM63	0.15517	0.15517
[7223]	CD4	<---	PTPRC	-0.27246	0.26433
[7224]	SHANK2	--->	DLG4	0.0055553	-0.010663
[7225]	YWHAG	--->	SPTBN1	0.14367	-0.14732
[7226]	SFRS2IP	--->	SFRS3	0.12373	-0.11096
[7227]	SHC1	<---	BCR	-0.073138	0.03781
[7228]	GRM1	--->	ITPR1	0.32693	-0.33685
[7229]	CCR5	--->	PTK2	0.037413	-0.14397
[7230]	UMOD	<---	LTA	-0.13011	0.21052
[7231]	CDKN2A	<---	UBE2A	-0.3223	0.34219
[7232]	PHLDA1	<---	HSPA4	-0.78532	0.82746
[7233]	SUGT1	<---	SKP1	-0.022833	0.014102
[7234]	PRMT2	--->	CPSF7	0.05698	-0.052044
[7235]	RAB27A	--->	COPS6	0.16293	-0.14012
[7236]	INHBB	--->	ACVR2A	1.0057	-0.99943
[7237]	PXN	<---	PKD1	-0.12814	0.12905
[7238]	RASSF5	<---	RRAS	-0.98684	1.0025
[7239]	SMAD3	<---	FOXO1	-0.083636	0.082517
[7240]	MYO7A	<---	PRKAR1A	-0.19666	0.19982
[7241]	BMPR1B	<---	CDK4	-0.20564	0.30344
[7242]	STRAP	<---	SMN1	-0.072234	0.086693
[7243]	KCNJ3	?	KCNJ9	-0.01825	-0.01825
[7244]	EGFR	--->	CRK	0.99929	-0.99608
[7245]	PRKCD	--->	ADRB2	0.46078	-0.46051
[7246]	CCND3	<---	VDR	-0.31113	0.3199
[7247]	IRS1	<---	FES	-0.9979	1.0005
[7248]	MAPK1	--->	TNFRSF25	0.40179	-0.79895
[7249]	RASD2	--->	SMAD2	0.55322	-0.47159
[7250]	PHC1	--->	BMI1	0.14598	-0.12808
[7251]	ATAD2	--->	NCOA3	0.12359	-0.13608
[7252]	NEK2	<--->	MAPK1	0.23429	0.23429
[7253]	PRKACA	--->	BRAF	1.0065	-1.0057
[7254]	RB1	<---	RAF1	-0.18878	0.17289
[7255]	OBSCN	--->	MYH2	0.0041249	-0.018069
[7256]	PTPN11	--->	STAT1	0.63902	-0.4446
[7257]	SKP2	--->	CCNE1	0.70868	-0.69543
[7258]	RAB11B	--->	OPTN	0.54384	-0.51762
[7259]	MEP1B	--->	FN1	0.13141	-0.099269
[7260]	HGS	<---	CCDC33	-0.057531	0.0073646
[7261]	CD4	<---	CTSD	-0.25497	0.25207
[7262]	PRKCE	<---	MAPK1	-0.033187	0.63541
[7263]	GNA14	<---	CHRM2	-0.98627	0.9872
[7264]	SLC16A10	<---	RACGAP1	-0.032363	0.025764
[7265]	DVL2	<---	ZNF263	-0.11966	0.11114
[7266]	FGFR4	<---	FGF4	-0.99871	1.0053
[7267]	PLEC1	--->	ITGA6	0.0041816	-0.012424
[7268]	TRIM27	--->	PRAM1	0.042733	-0.039191
[7269]	PIAS1	--->	NR2F2	0.095399	-0.090133
[7270]	TERT	<---	NCL	-0.015307	0.0034455
[7271]	MAP1A	--->	ACTG1	0.30819	-0.31177
[7272]	COL4A6	--->	FN1	0.057845	-0.061633
[7273]	GUCA1B	<---	GUCY2F	-0.25699	0.2459
[7274]	CHUK	--->	PEBP1	0.37377	-0.58309
[7275]	ZNF148	--->	STAT3	0.30982	-0.34787
[7276]	RBBP7	--->	RB1	0.44879	-0.45052
[7277]	FRS3	<---	RND1	-0.47916	0.51129
[7278]	PPP2R1A	--->	STRN	0.25641	-0.2507
[7279]	KDR	--->	BMX	0.35527	-0.29996
[7280]	UBE2D1	--->	MDM4	0.024986	-0.033069
[7281]	ESR1	--->	USF1	0.30604	-0.27813
[7282]	STAT5B	<---	IL2RB	-0.4655	0.43165
[7283]	PARD3	<---	PRKCI	-0.10721	0.093637
[7284]	NGF	<---	NGFR	0.037444	0.14607
[7285]	BCL3	<---	HDAC3	-0.13768	0.15653
[7286]	HAND2	<---	PRKCA	-1.0076	1.0168
[7287]	SNTG1	<---	DMD	-0.052248	0.048407
[7288]	EIF3G	<---	EIF3H	-0.23464	0.25857
[7289]	TANC1	--->	SHANK1	-0.041459	-0.063773
[7290]	PLSCR1	--->	DLK2	0.077273	-0.086284
[7291]	C8B	<---	C5	-0.24379	0.26528
[7292]	F11R	<---	PTPRB	-0.25089	0.23122
[7293]	SRC	--->	FASLG	0.91374	-0.79946
[7294]	NRG2	--->	ERBB3	0.99529	-1.0003

[7295]	APBA2	--->	LRP2	0.15184	-0.16087
[7296]	PAWR	<---	HSPA5	-0.83781	0.88041
[7297]	COL1A2	--->	APP	0.045133	-0.058504
[7298]	RNF168	--->	RABGEF1	-0.00046479	-0.010127
[7299]	GSK3B	--->	AXIN1	0.26825	-0.28011
[7300]	PDIA5	<---	UBQLN4	-0.14671	0.18415
[7301]	ELAVL1	<---	TNPO2	-0.037654	0.070134
[7302]	NCOA2	<---	HNF4A	-0.077273	0.066423
[7303]	TIRAP	<---	EIF2AK2	-0.3752	0.31754
[7304]	PRKCA	--->	ATP2B2	0.14677	-0.15808
[7305]	THBS1	<---	SPARC	-0.31216	0.40698
[7306]	MAPK4	<---	GAB1	-0.19052	0.17127
[7307]	SH2D1A	--->	ARHGEF7	0.039003	0.0030364
[7308]	SMAD4	<---	HDLBP	-0.31689	0.39981
[7309]	ZBTB17	<---	GTF2I	-0.26778	0.25565
[7310]	MKNK1	--->	PLA2G4A	0.50475	-0.50164
[7311]	NINL	--->	SYT17	0.35961	-0.31952
[7312]	ZHX1	--->	TAL1	0.15254	-0.14761
[7313]	GRIP1	--->	NR3C1	0.07947	-0.052103
[7314]	BCAR1	<---	PTPN1	-0.16364	0.16317
[7315]	ERN1	<---	HSP90AA1	0.012231	0.058057
[7316]	PPFIA2	<--->	PPFIBP1	0.049612	0.049612
[7317]	TRIM37	--->	ZNF417	0.2162	-0.2182
[7318]	RORB	<---	NPPA	-0.040788	0.066582
[7319]	TEK	--->	DOK4	0.0049397	-0.01946
[7320]	TFPI	--->	MMP8	0.20851	-0.1877
[7321]	FASLG	--->	PIN1	0.099539	-0.063247
[7322]	EPHB2	--->	ERF	0.44317	-0.44675
[7323]	SMARCE1	--->	ESR2	0.30074	-0.28692
[7324]	CSK	--->	ARRB1	0.64227	-0.55024
[7325]	SH3BP2	<---	ABL1	-0.19209	-0.00085066
[7326]	ITGA4	<---	ICAM4	-0.018248	0.0089757
[7327]	MMP1	<---	CCL7	-0.16713	0.18806
[7328]	DLG1	--->	PTEN	0.097178	-0.10096
[7329]	UNC13D	<---	RAB27B	-0.15731	0.20092
[7330]	MKNK1	--->	MAPK1	0.16997	0.13867
[7331]	SMAD1	<---	COL4A1	-0.061511	0.047645
[7332]	IKBKG	<---	HSPA1A	-0.76987	0.77676
[7333]	MPRIP	--->	YWHAG	0.18747	-0.17549
[7334]	ATXN1	--->	BATF2	0.043439	-0.040313
[7335]	NR2E3	--->	TBL3	0.034681	-0.0096005
[7336]	PDZD3	--->	SLC22A5	0.37001	-0.34102
[7337]	KAT2B	<---	EP300	-0.0041023	0.061921
[7338]	EPHB3	<---	FYN	0.046336	0.39258
[7339]	RYR2	<---	CACNA1C	-0.025062	-0.0066723
[7340]	MAPK1	<---	LCK	-0.99895	0.99734
[7341]	RYBP	--->	E2F6	0.095489	-0.06337
[7342]	TRIM24	<---	HSPA1A	-0.75437	0.79606
[7343]	SOCS1	--->	IFNGR1	0.42311	-0.42099
[7344]	DLG4	<---	ERBB4	-0.19014	0.18045
[7345]	CDKN1B	<---	KPNA3	-0.011578	0.014967
[7346]	DLG4	--->	KCNJ2	0.04551	-0.04695
[7347]	CSK	<--->	FYN	0.37153	0.37153
[7348]	CFLAR	--->	TRAF2	-0.0038991	-0.0080675
[7349]	PTEN	<---	SLC9A3R1	-0.19658	0.22078
[7350]	HIP1R	--->	ACTA1	0.52908	-0.5263
[7351]	ARNTL	<---	CSNK1E	-0.26109	0.30526
[7352]	RBPMS	<---	C9orf100	-0.030847	0.063672
[7353]	SERPINA1	<---	CTRB1	-0.082185	0.11833
[7354]	MAPK1	--->	GAB2	0.99738	-0.99704
[7355]	MAP3K7	--->	MAP3K5	0.51664	-0.36367
[7356]	CCL15	<---	CCR3	-0.46475	0.49595
[7357]	PAK7	<---	CDC42	-0.9928	0.99291
[7358]	CASP1	<---	ATN1	-0.032147	0.024361
[7359]	FARP2	<---	SRC	-0.1676	0.20544
[7360]	HOXA9	<---	PLSCR1	-0.080977	0.10682
[7361]	YWHAB	<---	HSP90AB1	-0.056628	0.050767
[7362]	PTPN12	--->	PTK2B	0.29598	-0.3482
[7363]	GRIA1	<---	GRIA4	-0.19915	-0.0077421
[7364]	TRAF2	<---	FADD	-0.10543	0.064051
[7365]	DNM2	<---	ZFAND6	-0.12304	0.11453
[7366]	TNFAIP3	--->	RIPK1	1.0062	-0.99852
[7367]	GRB7	<---	INSR	-0.17642	0.14373
[7368]	ARHGEF11	--->	MAP1A	-0.0028209	-0.0038931
[7369]	LPA	<---	FGB	-0.0052456	0.020403
[7370]	RYBP	--->	E2F2	0.095489	-0.06337

[7371]	GPS2	<---	EP300	-0.18644	0.18211
[7372]	PRKACA	--->	GRIA4	0.66332	-0.67945
[7373]	E2F4	--->	RBL1	0.27168	-0.2819
[7374]	GALNT6	--->	FN1	0.041589	-0.029161
[7375]	CSNK2A2	--->	PPP1R1B	1.0061	-1.0155
[7376]	GGCX	--->	F9	0.071085	-0.066849
[7377]	CFL1	--->	ACTA2	0.30201	-0.30587
[7378]	NOTCH1	<---	CBL	-0.18016	0.16682
[7379]	SRF	--->	CEBPB	0.14789	-0.1171
[7380]	NLK	--->	CUL1	0.30021	-0.32466
[7381]	PTK2B	--->	SH2D3C	-0.041645	-0.13169
[7382]	CAMK4	--->	CREBBP	0.32272	-0.32425
[7383]	XRN1	<---	CHD4	-0.0064065	-0.0060915
[7384]	MAPK1	--->	SLC9A1	1.0078	-0.99872
[7385]	SKIL	<---	ZZEF1	-0.40553	0.4096
[7386]	MAP2K5	--->	PRKCZ	0.069627	0.025152
[7387]	DLG4	--->	GRIN2C	0.044742	-0.055228
[7388]	DAPK3	--->	CDKN1A	0.51838	-0.55156
[7389]	AHR	<---	TBP	-0.013801	0.00094649
[7390]	YWHAB	--->	SLC9A1	0.3324	-0.32858
[7391]	IRS2	<---	PTPN6	-0.21679	0.2463
[7392]	BCL6	<---	WNK4	-0.61458	0.60369
[7393]	ATF4	<---	CEBPB	-0.3007	0.31009
[7394]	CDK5RAP2	--->	STARD9	0.012623	0.0021812
[7395]	DLG4	--->	IL13RA1	0.11975	-0.12928
[7396]	BAZ2A	<---	SMARCA5	-0.008923	-0.00096593
[7397]	SRC	<---	AXL	-0.014604	0.070053
[7398]	RAP1A	--->	SMARCA4	0.04163	-0.049612
[7399]	GRK6	--->	CHRM3	0.21152	-0.27051
[7400]	NRXN1	<---	SYT4	-0.043035	0.083467
[7401]	ADRA2A	<---	ADRB1	-0.026077	0.09842
[7402]	RNF11	--->	TNIP1	0.20347	-0.18195
[7403]	MAP3K3	--->	YWHAE	0.54958	-0.30237
[7404]	PRKCA	--->	KIT	1.0004	-0.99338
[7405]	LPP	--->	ACTN1	0.014972	-0.010035
[7406]	APBA1	--->	PSEN1	0.025765	-0.020908
[7407]	IL13RA1	<---	TYK2	-0.26217	0.25162
[7408]	SOX8	<---	SP1	-0.041897	0.03089
[7409]	WDR77	<---	YWHAQ	-0.19355	0.2164
[7410]	PRKACA	--->	PPP1R9B	0.62013	-0.64226
[7411]	DNMT3A	--->	EED	0.16371	-0.13078
[7412]	EIF2AK2	--->	NFKBIA	1.454	-1.5082
[7413]	ITK	--->	TGFBR1	0.14028	-0.16822
[7414]	RAC1	--->	TIAM1	0.99931	-1.0019
[7415]	SMAD3	--->	CTNNB1	0.17916	-0.18358
[7416]	PRKCQ	--->	WIPF1	0.9992	-1.0006
[7417]	TIMP3	--->	MMP2	0.21399	-0.19321
[7418]	PARD6B	<---	PARD3B	-0.010294	-0.0054265
[7419]	CSNK1A1	--->	YWHAQ	0.55799	-0.48053
[7420]	YLPM1	<---	PRPF40A	-0.19186	0.23164
[7421]	SYK	<---	CBL	-1.0008	0.99213
[7422]	PAK2	--->	SH3RF3	0.082023	0.012028
[7423]	CDK8	--->	CCNC	1.083	-1.1093
[7424]	CSNK2A1	--->	KLF1	0.63385	-0.64027
[7425]	XRN1	<---	HNRNPA1	0.0010642	0.001779
[7426]	CDK5	--->	WASF1	0.5158	-0.53847
[7427]	PDGFC	--->	PDGFRA	0.56462	-0.5545
[7428]	ZNF76	<---	HDAC1	-0.019943	0.014938
[7429]	CIB1	<---	PTK2	-0.24557	0.14139
[7430]	ZDHHC17	<---	HTT	-0.12753	0.14632
[7431]	ADAM15	--->	ABL1	-0.039179	-0.057139
[7432]	THRA	--->	RXRA	1.003	-0.99549
[7433]	CHRDL2	--->	GDF5	0.71403	-0.67452
[7434]	MAP3K7IP1	--->	MAP3K7IP2	0.10577	-0.09944
[7435]	CBL	--->	YWHAZ	0.48957	-0.49673
[7436]	WASF2	<---	ABI1	-0.074825	0.060813
[7437]	MAP2K7	--->	GADD45B	0.74161	-0.76568
[7438]	SH2B2	<---	LYN	-0.89003	0.98544
[7439]	ABCA1	--->	ARHGEF12	-0.0052012	-0.006075
[7440]	TNFRSF1A	--->	TNFRSF25	0.74968	-0.83986
[7441]	AKT1	--->	RAF1	1.0008	-1.0133
[7442]	SMAD4	--->	HOXC8	0.13335	-0.10211
[7443]	CDK2	--->	MYBL2	0.24948	-0.26781
[7444]	TRIM31	<---	TRIM23	-0.28502	0.29563
[7445]	COPB2	?	COPA	-0.1554	-0.1554
[7446]	CARD10	--->	IKBKG	0.11247	-0.11848

[7447]	SETD7	--->	TP53	0.32543	-0.32337
[7448]	F2	<---	AMBP	0.00022919	0.014946
[7449]	SELL	?	SELE	-0.016027	-0.016027
[7450]	WT1	--->	PAX2	0.013121	-0.011504
[7451]	VAV1	<---	RHOG	-0.22722	0.041359
[7452]	INPP5B	<---	SMURF1	-0.26294	0.25263
[7453]	YWHAQ	<---	FSHR	-0.20604	0.2133
[7454]	EP300	--->	OLIG2	0.066164	-0.076626
[7455]	TRAF1	<---	TNFRSF1B	-0.096451	0.022494
[7456]	PTK2B	--->	TLN1	-0.010686	-0.085504
[7457]	KLRD1	?	KLRC3	-0.090136	-0.090136
[7458]	SH3BP2	--->	VAV2	0.0037184	-0.09675
[7459]	CNOT4	<---	UBE2E3	-0.28011	0.29304
[7460]	PDGFRA	<---	PDGFRB	-0.017692	-0.0077331
[7461]	HERC5	--->	CCND1	0.47579	-0.47291
[7462]	GAB2	<---	EPOR	-0.27745	0.26765
[7463]	FXR2	<---	RALYL	-0.0048144	0.036464
[7464]	FAS	--->	CALM1	0.42072	-0.23418
[7465]	CD8A	?	CD8B	-0.077629	-0.077629
[7466]	PCNT	<---	AKAP9	0.012396	0.021878
[7467]	CARD10	<---	BCL10	-0.095466	-0.034803
[7468]	CDC25A	<---	CDK2	-0.67632	0.69298
[7469]	GAB1	--->	PLCG2	0.028063	-0.058481
[7470]	POU3F2	--->	TBP	-0.0073617	-0.0079359
[7471]	AKT1	--->	CCDC88A	0.45026	-0.3034
[7472]	MYC	<---	CDK1	-1.0065	1.0154
[7473]	CCR3	--->	CCL5	0.49595	-0.46475
[7474]	PRDM16	--->	INADL	0.0049248	-0.014044
[7475]	JAM3	--->	ITGAX	0.025067	-0.038226
[7476]	NR0B2	--->	ESR2	-0.0094276	-0.028437
[7477]	PIAS1	--->	PIAS2	0.066672	0.023557
[7478]	CCL21	--->	VCAN	0.010191	-0.021003
[7479]	KAT5	--->	STAT3	0.33951	-0.37727
[7480]	PRKCZ	--->	IRS1	0.38015	-0.35091
[7481]	LAMA5	?	LAMC1	-0.0068785	-0.0068785
[7482]	CCBP2	--->	CCL4	0.49595	-0.46475
[7483]	CDKN1A	<---	CSNK2A1	-0.55156	0.51838
[7484]	TWF1	<---	BMPR2	-0.26483	0.2342
[7485]	ENC1	--->	RB1	0.4649	-0.46663
[7486]	ZNF689	<---	HNRNPF	-0.2001	0.19681
[7487]	STRADB	--->	XIAP	0.06991	-0.12533
[7488]	ESR1	<---	PRMT1	-0.19368	0.19359
[7489]	ZNF250	<---	CCDC85B	-0.31174	0.31077
[7490]	CDK16	--->	YWHAH	0.62776	-0.66368
[7491]	DOK5	<---	NTRK2	-0.2014	0.20066
[7492]	NR4A1	<---	YWHAZ	-0.092332	0.11204
[7493]	SMAD2	<---	FBN2	-0.1278	0.14455
[7494]	SPARC	--->	FN1	0.12359	-0.094743
[7495]	LYL1	--->	TCF3	-0.13552	-0.39218
[7496]	FHL3	--->	RBM42	0.054056	-0.021109
[7497]	ITGA2	<---	MMP1	-0.035788	0.022884
[7498]	PELI3	<---	IRAK1	-0.98981	1.0139
[7499]	ARHGEF7	--->	PXN	0.072291	-0.082458
[7500]	SYNGAP1	<---	ULK1	-0.019797	0.00663
[7501]	CPNE1	<---	MAP2K1	-0.17777	0.59194
[7502]	CALCOCO1	<---	CREBBP	-0.187	0.18273
[7503]	PIK3R1	<---	TGFBR2	-0.2338	0.18406
[7504]	MCF2L	<---	RHOG	-0.014357	0.0012594
[7505]	PLEC1	<---	EGFR	-0.28085	0.27998
[7506]	RBPJ	--->	NOTCH4	0.12107	-0.13218
[7507]	MARK4	--->	PARD6A	0.030474	-0.050196
[7508]	SMAD9	<---	HUWE1	-0.15743	0.14209
[7509]	RAB27A	--->	RIMS1	0.016184	-0.0067445
[7510]	HSPA8	--->	CXCR4	0.40505	-0.36596
[7511]	TFPI	--->	MMP7	0.20851	-0.1877
[7512]	ARL1	--->	GOLGA1	0.51273	-0.48711
[7513]	TP53	<---	MDM2	-0.9968	0.9983
[7514]	GSK3B	--->	HNRNPD	0.72994	-0.75755
[7515]	HOXA10	<---	FOXO1	-0.07443	0.06395
[7516]	MTNR1B	--->	GNAI3	1.0129	-1.008
[7517]	SETDB1	<---	SNIP1	-0.16387	0.16814
[7518]	MAPKAPK2	--->	SRF	0.28633	-0.30066
[7519]	SMAD7	<---	EP300	-0.99697	1.0006
[7520]	ADAMTS4	--->	SERPINA1	0.031729	-0.027936
[7521]	GIPC1	--->	SLC2A1	0.37001	-0.34102
[7522]	SPIB	--->	RB1	0.43649	-0.43615

[7523]	TICAM2	--->	IRAK2	0.58357	-0.54271
[7524]	NCK2	--->	CPSF7	0.11256	-0.12005
[7525]	F12	--->	APP	0.12866	-0.14113
[7526]	TCERG1	--->	SFRS1	0.19241	-0.17953
[7527]	RNF11	--->	HERC1	0.079032	-0.076132
[7528]	PLCB2	<---	MAP2K3	-0.64776	0.65475
[7529]	DIAPH3	--->	SMAD9	0.05061	-0.065528
[7530]	PTAFR	<---	TYK2	-0.011003	-0.00043892
[7531]	HIF1A	<---	EGLN3	-0.99517	0.99907
[7532]	REL	--->	NOTCH1	0.005975	-0.020004
[7533]	KPNA4	<---	RECQL	-0.018416	0.032742
[7534]	DLG3	<---	KIF1B	-0.013466	-0.0064478
[7535]	PTPN2	--->	JAK3	0.47422	-0.51771
[7536]	SMURF1	--->	UBTF	0.68132	-0.68529
[7537]	NR1H4	--->	NCOA1	0.034596	-0.043163
[7538]	FGFR4	<---	FGF17	-0.99871	1.0053
[7539]	HBP1	--->	RB1	0.52072	-0.52236
[7540]	MAPK1	--->	IQGAP1	0.41429	-0.43407
[7541]	AKT1	--->	YWHAZ	0.44249	-0.29699
[7542]	JUB	<---	BCAR1	-0.013863	0.016543
[7543]	HSPA1A	--->	APOB	0.82587	-0.78356
[7544]	RYBP	<---	FADD	-0.058912	-0.026024
[7545]	SERPING1	<---	ELANE	-0.082185	0.11833
[7546]	TNFRSF1A	--->	PTK2	0.035927	-0.043572
[7547]	GJB3	--->	TJP1	0.011892	-0.012642
[7548]	SFRS11	?	U2AF2	-0.016298	-0.016298
[7549]	HIST2H2BE	<---	TBL1X	-0.033407	0.034046
[7550]	LGALS3BP	<---	NID1	0.00032936	0.0085481
[7551]	EIF4E2	<---	EIF4EBP3	-1.0062	1.0033
[7552]	ELK3	--->	TCF3	0.16943	-0.17098
[7553]	NINL	--->	MBIP	0.38609	-0.35236
[7554]	SFRP1	--->	FZD6	0.6931	-0.62
[7555]	MAPK14	--->	NCOA3	0.22463	-0.24638
[7556]	USP7	--->	RADIL	0.014383	-0.0077294
[7557]	SYK	--->	CSF3R	0.5535	-0.55422
[7558]	ZAP70	--->	VAV1	1	-1.0027
[7559]	SYK	--->	MAPK3	0.99941	-1.0086
[7560]	SNX1	<---	F2R	-0.45859	0.48977
[7561]	MAGI2	--->	PTEN	0.0070401	-0.0057729
[7562]	RBBP7	<---	APPL1	-0.047478	0.081967
[7563]	KSR2	<---	MAPK1	-0.045194	0.056629
[7564]	EP300	--->	GABPA	0.11504	-0.12299
[7565]	MAPK12	<---	DUSP1	-0.89445	0.87425
[7566]	NIN	<---	PRKACA	-0.66014	0.63519
[7567]	FASLG	--->	PSTPIP1	0.14108	-0.12462
[7568]	NUMB	--->	ITGB5	0.015753	-0.02256
[7569]	MAPK3	--->	JUN	1.0022	-1.0148
[7570]	EGFR	--->	SOCS5	0.31029	-0.33386
[7571]	SHC1	<---	PRKCD	-0.14355	0.1262
[7572]	CTNND2	<---	CTTN	-0.034561	0.048153
[7573]	TGFBR3	--->	TGFB1	0.1408	-0.24051
[7574]	JUN	<---	MAPK11	-0.73927	0.67694
[7575]	SMAD2	<---	CSH1	-0.21558	0.26049
[7576]	ZHX1	<---	WDR33	-0.018469	0.015004
[7577]	MAPK3	--->	RALGDS	0.45836	-0.47603
[7578]	MAGI3	<---	PTEN	-0.0021203	0.0032934
[7579]	CTBP2	--->	ZEB1	0.043292	-0.055921
[7580]	NCL	<---	SSB	-0.030661	0.0097534
[7581]	MBD1	<---	HIPK3	-0.54149	0.52508
[7582]	EEF1G	<---	CBX8	-0.051859	0.072138
[7583]	THRB	<---	BRD8	-0.088819	0.11471
[7584]	COPB2	<---	RGS4	-0.55801	0.58652
[7585]	RAC2	--->	ACTB	0.99207	-0.99507
[7586]	FNTA	<---	PTP4A3	-1.3382	1.3467
[7587]	FXR1	<---	PRSS23	-0.015462	0.049479
[7588]	FGFR1	<---	SH3BP2	-0.089181	-0.033924
[7589]	SGSM3	--->	GJA1	0.093143	-0.099161
[7590]	CHUK	--->	TP53	0.231	-0.31433
[7591]	CDC25C	<---	YWHAH	-0.11975	0.14141
[7592]	AR	<---	NFKB1	-0.062533	0.05023
[7593]	SNAI1	<---	GSK3B	-0.51628	0.55651
[7594]	HSPB3	?	HSPB2	-0.096038	-0.096038
[7595]	MMP2	<---	PZP	-0.11161	0.132
[7596]	UBXN1	<---	SMURF1	-0.7022	0.69833
[7597]	PFN1	--->	PIK3R1	0.16226	-0.26719
[7598]	MAP2K1	--->	ELK1	0.69098	-0.41603

[7599]	PLAU	--->	NID1	0.028815	-0.031395
[7600]	VASP	<---	ABI1	-0.062083	0.051702
[7601]	NCF4	<---	NCF2	0.0038997	0.0068784
[7602]	MAPK3	--->	ARRB1	0.42184	-0.44306
[7603]	EP300	--->	NCOA2	0.020953	-0.033602
[7604]	CDK6	--->	TGFBR1	0.36359	0.02614
[7605]	SREBF2	<---	CREBBP	-0.056808	0.045169
[7606]	USP8	<---	STAM	0.0087086	0.062318
[7607]	MPDZ	<---	KIT	-0.24298	0.19883
[7608]	KAT2B	--->	TTF1	0.13554	-0.13488
[7609]	RHEBL1	--->	SMAD4	0.55322	-0.47159
[7610]	PDK2	--->	PDHA1	1.0004	-0.99606
[7611]	CBLC	--->	GRB2	0.30116	-0.31024
[7612]	AP3D1	<---	ARF1	-0.40724	0.43364
[7613]	SVIL	--->	FLNA	0.11922	-0.062233
[7614]	IRAK2	<---	HRAS	-0.46598	0.72678
[7615]	TRAF2	--->	ABLIM1	0.10182	-0.10309
[7616]	SLC9A3R1	<---	AKAP10	-0.41116	0.44344
[7617]	SRC	--->	CTNND1	0.44243	-0.4172
[7618]	TGIF1	<---	HMGB1	-0.34069	0.3623
[7619]	PPP1CA	<---	PPP2CA	0.0082476	0.094949
[7620]	PRKCA	--->	PTPN6	0.6319	-0.62682
[7621]	SYT1	--->	NR1H3	0.081234	-0.068641
[7622]	SELL	--->	EZR	0.0037543	-0.0071199
[7623]	SORBS1	--->	SH2B2	-0.040279	-0.071879
[7624]	RGS12	--->	PLCB3	0.053331	-0.062682
[7625]	MYBL2	<---	NCOR1	-0.0087578	0.013867
[7626]	PPP2R1B	--->	CDK1	0.30999	-0.30289
[7627]	SPARC	<---	COL13A1	-0.036678	0.034083
[7628]	BACE1	--->	PLSCR1	0.22234	-0.19625
[7629]	DMPK	--->	HSPB2	1.0388	-0.99926
[7630]	PTPN6	--->	KIT	0.98781	-1.0015
[7631]	GRIN1	<---	CAMK2A	-0.48906	0.47865
[7632]	TGFBR3	--->	INHA	0.0387	-0.024029
[7633]	RASD2	--->	SMAD1	0.52759	-0.54836
[7634]	CTNND1	--->	CDH3	0.51654	-0.52601
[7635]	DLG1	<---	BCR	-0.016059	-3.4888e-005
[7636]	BCL2L10	?	BAX	-0.63915	-0.63915
[7637]	DOCK3	--->	PSEN1	0.12827	-0.14024
[7638]	AXL	--->	SHC3	0.2696	-0.33279
[7639]	RAF1	<---	HSPA4	-0.29539	0.27279
[7640]	FYN	--->	FASLG	0.92233	-0.76006
[7641]	GDF6	--->	BMPR2	1.0057	-0.99943
[7642]	NOD1	--->	CASP1	-0.038798	-0.12583
[7643]	RPS6KB1	<---	MAPK1	-1.0109	1.002
[7644]	EGFR	--->	STAM2	0.095034	-0.090392
[7645]	RIMS2	--->	YWHAH	0.0025914	0.0007618
[7646]	PPP2R1B	--->	RELA	1.0003	-0.99542
[7647]	CAV3	--->	NOS1	0.2728	-0.28293
[7648]	WT1	<---	CREBBP	-0.076585	0.066291
[7649]	MTNR1A	--->	CALR	0.52319	-0.5062
[7650]	EPS15	<---	ELF3	-0.04262	0.031182
[7651]	RUSC1	<---	NTRK1	-0.01964	0.012772
[7652]	GAB3	<---	GRB2	-0.33688	0.35049
[7653]	IRS2	<---	IGF1R	-0.24793	0.23436
[7654]	CAMK2G	--->	TIAM1	0.32578	-0.34106
[7655]	A1CF	?	SYNCRIP	-0.016298	-0.016298
[7656]	GADD45B	--->	PPARD	0.017932	0.0079001
[7657]	PPARD	--->	SMAD9	0.14264	-0.15299
[7658]	DDX19B	<---	XPO7	0.0076288	0.0076582
[7659]	TP53	<---	CHEK2	-0.60852	0.60338
[7660]	BACH1	<---	RBCK1	-0.22847	0.25993
[7661]	EPB41L1	--->	GRIA1	0.11545	-0.12348
[7662]	SMAD5	--->	RYR2	0.21831	-0.22729
[7663]	YWHAZ	--->	FOXO1	0.080297	-0.089786
[7664]	AURKAIP1	<---	JUB	-0.00033202	0.035782
[7665]	DLG4	--->	HTT	0.042206	-0.04193
[7666]	ID1	--->	TCF4	-0.054725	-0.37599
[7667]	MAST1	--->	SNTA1	0.1406	-0.092397
[7668]	INSR	<---	ADRB2	-0.11081	0.10006
[7669]	BAIAP2	<---	CDC42	-0.13661	0.13835
[7670]	BTK	--->	CD19	0.5753	-0.62253
[7671]	RAD23A	--->	ATXN3	0.076162	-0.042104
[7672]	CD36	--->	ITGB3	0.018321	-0.021496
[7673]	AP3B1	<---	CSNK1A1	-0.20078	0.2112
[7674]	SMURF1	--->	MAP3K2	0.11726	-0.093073

[7675]	PRKACA	--->	ITGA4	0.026402	-0.20724
[7676]	ZBTB7A	<---	SP3	-0.23148	-0.020406
[7677]	IRS2	<---	UBTF	-0.095469	0.089232
[7678]	SKP2	<---	SKP1	-0.044878	0.055251
[7679]	PI4KB	--->	NCS1	0.34158	-0.30321
[7680]	HOMER1	<---	HOMER3	0.0061138	0.16988
[7681]	TRERF1	<---	CREBBP	-0.073443	0.063039
[7682]	EIF2AK2	<---	JAK1	-0.5173	0.50431
[7683]	CAV1	--->	ESR1	0.06824	-0.046234
[7684]	PRKCD	--->	ACTA2	0.48294	-0.48348
[7685]	SFN	--->	EIF4B	0.13702	-0.14209
[7686]	PRKACB	--->	NGFR	0.23445	-0.27811
[7687]	CNOT4	<---	UBE2E1	-0.28011	0.29304
[7688]	HK2	<---	UBQLN4	-0.02975	0.044875
[7689]	CDC42	--->	CDC42EP1	0.57849	-0.87115
[7690]	PPM1A	--->	CACNA1A	0.20004	-0.20923
[7691]	TF	--->	IGFBP3	0.072099	-0.038154
[7692]	TIAM1	<---	PRKCA	-0.17606	0.18829
[7693]	SHC3	<---	NTRK2	-0.21545	0.15654
[7694]	PLCG2	<---	BLK	-0.8605	0.59106
[7695]	MICAL1	<---	PTK2	-0.29942	0.25272
[7696]	INADL	--->	KCNJ10	0.044422	-0.0070376
[7697]	ADCY6	<---	GNAS	-0.63771	0.64323
[7698]	PTPN1	--->	IRS1	0.68409	-0.69025
[7699]	CXCR4	--->	GNAI1	1.0129	-1.008
[7700]	PTPRA	--->	FYN	-0.050335	-0.080843
[7701]	GAB1	--->	FRS2	0.28309	-0.29086
[7702]	RECQL5	<---	STAMBP	-0.13534	0.14604
[7703]	NPY5R	<---	NPY	-0.19468	0.20502
[7704]	MAPK10	--->	TP53	0.88459	-0.89006
[7705]	BCL2L11	--->	BCL2L2	0.16739	-0.17223
[7706]	BACH2	--->	PATZ1	0.022194	-0.15821
[7707]	IQGAP1	<---	PRKACA	-0.43407	0.41429
[7708]	LMO1	--->	NHLH1	0.62562	-0.58677
[7709]	CD40	<---	BIRC3	-0.18226	0.16508
[7710]	RAC1	--->	PLXNB1	0.41051	-0.37902
[7711]	MARK3	--->	VPS11	0.035149	-0.049183
[7712]	SQSTM1	--->	GABARAPL1	0.1048	-0.10304
[7713]	APC2	--->	CTNNB1	-0.0067549	-0.051022
[7714]	SKIV2L	<---	CDC2L2	-0.23633	0.21815
[7715]	CREBBP	--->	HTT	0.18219	-0.18651
[7716]	CBX1	<---	DNMT3A	0.0073508	0.026698
[7717]	HIPK2	--->	PAX6	0.64333	-0.66021
[7718]	UBE2D1	--->	TRIM39	0.085435	-0.089161
[7719]	CCNE2	<---	CDKN1A	-0.99961	0.98767
[7720]	SFMBT1	<---	PHC1	0.088113	0.090008
[7721]	RERE	--->	EFEMP2	0.071521	-0.050192
[7722]	CEBPG	--->	ATF2	0.34768	-0.34627
[7723]	PDPK1	--->	PRKACA	0.5367	-0.39311
[7724]	YWHAH	--->	EPB41L2	0.05792	-0.057222
[7725]	ENOX1	--->	ZBTB16	0.11348	-0.11551
[7726]	CDK5	--->	MAPT	0.99929	-1.0027
[7727]	PRKD1	--->	MAPK8	0.57016	-0.033526
[7728]	GRB7	<---	TEK	-0.014867	-0.0050415
[7729]	CISH	<---	PRKCB	-0.60234	0.60269
[7730]	PRKCA	--->	ITPKA	0.87955	-0.8782
[7731]	DNM2	<---	KDR	-0.16828	0.16958
[7732]	ITGA6	--->	ITGB1	-0.0084401	-0.026788
[7733]	SH3D19	--->	ADAM12	0.029655	-0.049519
[7734]	RIN1	--->	ABL1	1.0027	-0.9916
[7735]	FRS3	<---	PTPN11	-0.72183	0.90858
[7736]	RASA1	<---	SHC1	0.0081641	0.021019
[7737]	CCL3L1	<---	CCR3	-0.46475	0.49595
[7738]	WASL	<---	PFN1	-0.12007	0.090584
[7739]	UBE2E2	--->	CUL9	0.25501	-0.24249
[7740]	EP300	--->	JUN	0.12142	-0.13136
[7741]	MEF2A	--->	MYOD1	0.060707	-0.059789
[7742]	USP7	--->	ZNF423	0.26552	-0.26229
[7743]	EIF2AK2	<---	MAP3K7	-0.98749	0.99376
[7744]	RGS7	--->	GNAQ	0.60077	-0.60607
[7745]	HDAC1	<---	SENP1	-1.0027	1.0037
[7746]	LCK	--->	EZR	0.33022	-0.2959
[7747]	TRPS1	<---	RNF4	-0.14487	0.15096
[7748]	SPIB	<---	MAPK8	-1.1212	1.1285
[7749]	RFWD2	--->	TP53	0.1134	-0.11805
[7750]	CSNK2A1	--->	PPP1R1B	1.0061	-1.0155

[7751]	CHD3	--->	TNFRSF14	0.0076767	-0.021221
[7752]	SLA	--->	CD247	0.51177	-0.58583
[7753]	GTF2A1	--->	TBP	-0.0031141	-0.0095587
[7754]	FGR	<---	SRC	-0.016792	0.60459
[7755]	PRKACA	--->	ABCA1	0.16549	-0.18176
[7756]	BRCA1	--->	SMAD4	0.25767	-0.21264
[7757]	GIPC1	<---	DRD2	-0.41277	0.44636
[7758]	DLG4	<---	FYN	-0.27518	0.41392
[7759]	PFKM	<--->	PFKL	0.0012563	0.0012563
[7760]	RAC1	--->	NCF2	0.12502	-0.13569
[7761]	COTL1	--->	ACTA1	0.49943	-0.49675
[7762]	HSPA4	--->	HDAC3	0.83174	-0.78946
[7763]	ABLIM1	--->	CALCOCO2	0.043508	-0.02644
[7764]	PLEC1	--->	ITGB4	0.013443	-0.021421
[7765]	MTNR1A	--->	RAB10	0.066588	-0.039125
[7766]	ITGA2	<---	ACTA1	-0.032235	0.019131
[7767]	MAPKAPK5	--->	GYS1	0.55762	-0.76891
[7768]	JDP2	<---	EP300	-0.12724	0.11829
[7769]	SYNJ1	<---	BIN1	-0.07064	0.084721
[7770]	NGFR	--->	TRADD	1.0063	-1.0095
[7771]	DDX20	?	DHX9	-0.0054159	-0.0054159
[7772]	BCL2L2	<---	BID	-0.71278	0.72799
[7773]	SMAD5	<---	SFPQ	-0.30655	0.28646
[7774]	F2RL1	<---	CBL	-0.44624	0.44002
[7775]	PPARGC1A	--->	SFRS5	-0.0021891	-0.0095939
[7776]	YWHAG	--->	HDAC4	0.067622	-0.043467
[7777]	IKBKG	<---	CHUK	-0.4146	0.27526
[7778]	MXD4	?	MAX	-0.65635	-0.65635
[7779]	MELK	--->	SF3B1	0.38122	-0.40043
[7780]	ITCH	--->	CPSF6	0.1536	-0.16435
[7781]	RAB11A	--->	MYO5A	0.24373	-0.23098
[7782]	RGS2	--->	CALM1	0.78674	-0.75366
[7783]	MAP2K2	--->	CASP9	0.66416	-0.42184
[7784]	EGFR	--->	PLCG1	1.0042	-1.0044
[7785]	ENG	<---	ACVR2A	-0.50546	0.48265
[7786]	YWHAB	--->	INSR	0.13685	-0.14599
[7787]	ETS1	<---	CREBBP	-0.12299	0.11504
[7788]	SOCS7	<---	SORBS3	-0.056815	0.011345
[7789]	PGK1	--->	GAPDH	0.0086096	0.0065008
[7790]	MEIS1	<---	HOXD12	-0.0094151	0.024726
[7791]	HCK	--->	PLCG2	0.59106	-0.8605
[7792]	TNFSF13	--->	FAS	0.99951	-1.0013
[7793]	CDKN2C	<---	COPS6	-0.80626	0.83154
[7794]	CREB1	<---	SMARCA4	-0.049577	0.039125
[7795]	C1QTNF1	--->	AVPR2	0.0549	-0.065435
[7796]	TNK2	<---	GRB2	-0.015091	-0.00035839
[7797]	RFXANK	<---	RAF1	-1.003	0.99328
[7798]	ATAD2	--->	ESR1	0.033318	-0.022603
[7799]	CTNND1	<---	GRIK2	-0.0055935	-0.0042977
[7800]	ATN1	--->	RBPMS	0.038991	-0.041558
[7801]	ZMYND8	<---	ZFYVE9	-0.12583	0.11821
[7802]	YWHAB	<---	DAPK1	-0.020856	0.061934
[7803]	AKT1	--->	EP300	0.048622	-0.055988
[7804]	KLRD1	?	KLRC2	-0.090136	-0.090136
[7805]	HNRNPC	--->	ZNF581	0.20364	-0.19923
[7806]	PTPN22	--->	CD247	0.42182	-0.45942
[7807]	CCBP2	--->	CCL14	0.49595	-0.46475
[7808]	CSRP3	--->	NHLH1	0.62562	-0.58677
[7809]	LTBP3	--->	TGFB2	0.66401	-0.63698
[7810]	RAF1	--->	INSR	-0.01618	-0.062797
[7811]	ISL1	--->	ESR1	0.013803	-0.0038765
[7812]	CDK1	--->	RUNX2	0.74736	-0.77314
[7813]	SOX8	--->	TCF12	0.027151	-0.038902
[7814]	BCL2A1	?	BAX	-0.63915	-0.63915
[7815]	NPR1	--->	NPPA	0.14651	-0.15882
[7816]	RASD1	--->	NOS1	0.29504	-0.30468
[7817]	TNFAIP6	--->	ACAN	0.0012038	-0.019645
[7818]	RAP1A	--->	MLLT4	0.99712	-0.98672
[7819]	E2F5	--->	TFDP1	-0.0088085	-0.064857
[7820]	KDM2A	--->	HIST2H3C	0.28796	-0.23643
[7821]	AKAP1	<---	CAV1	-0.047653	0.059257
[7822]	JAK2	--->	BRCA1	0.17728	-0.10937
[7823]	ZFYVE16	<---	PPP1CA	-0.073136	0.061706
[7824]	PIN1	<---	NEK6	-0.49485	0.46953
[7825]	AATF	--->	RELA	0.04782	-0.049632
[7826]	CAPN2	<---	ADRBK1	-0.091658	0.074704

[7827]	RTKN	<---	TGFBR1	-0.20908	0.16739
[7828]	CSNK2A1	--->	SHOX	0.60726	-0.63441
[7829]	HIPK3	--->	FAS	0.23445	-0.27811
[7830]	MYO18A	--->	SFTPA1	-0.00026791	-0.0096926
[7831]	HEYL	<---	SMAD3	-0.62633	0.61773
[7832]	NOTCH3	<---	PSEN2	-1.0138	0.9973
[7833]	CNTN1	<---	CNTN2	0.0078499	0.010648
[7834]	GRIN1	<---	HSPA1A	-0.47675	0.48673
[7835]	SP1	--->	SREBF1	0.1015	-0.11338
[7836]	CASP2	--->	BID	0.067368	-0.028077
[7837]	SDCBP	--->	GRIK1	0.12457	-0.11938
[7838]	SEC62	<---	CIC	-0.19222	0.23362
[7839]	SKIL	<---	PAPPA	-0.40438	0.40844
[7840]	BMPR1B	<---	RHOD	-0.53994	0.52577
[7841]	STAT6	<---	NFKB1	-0.1162	0.10493
[7842]	WAS	<---	GRB2	-0.59586	0.54185
[7843]	MAPK12	--->	DLG1	0.087106	-0.14764
[7844]	TLR1	<---	TLR10	0.11276	0.13155
[7845]	SH3D19	--->	ADAM9	0.015714	-0.033248
[7846]	CAV1	--->	NOS2	0.45512	-0.46313
[7847]	FHOD1	--->	ACTA1	0.32267	-0.32649
[7848]	PFN1	--->	MLLT4	0.25515	-0.20649
[7849]	PDPK1	<---	PAK1	-0.20725	0.22288
[7850]	ASAP2	<---	ARF1	-0.13844	0.12847
[7851]	MTNR1B	--->	GNAI1	1.0129	-1.008
[7852]	CCBP2	--->	CCL25	0.49595	-0.46475
[7853]	PTPN12	--->	PTK2	0.29598	-0.3482
[7854]	ATXN1	--->	SREBF1	0.060398	-0.070769
[7855]	TLR2	--->	TICAM1	0.23289	-0.24233
[7856]	LCK	--->	AXL	0.11156	0.021933
[7857]	RASA1	<---	KIT	-0.22803	0.077443
[7858]	INHBC	--->	ACVR1	0.60319	-0.60916
[7859]	ESR1	<---	KIF1A	-0.10016	0.095314
[7860]	GNAZ	<---	ADORA1	-1.008	1.0129
[7861]	CHN2	<---	RAC1	-0.15063	0.15564
[7862]	ADAM12	<---	ACTN2	-0.058015	0.055053
[7863]	PDCD6IP	<---	SH3GL2	-0.084834	0.099128
[7864]	ROCK2	--->	PPP1R12A	0.13459	-0.14024
[7865]	ZMYND11	<---	TRAF6	-0.67538	0.66826
[7866]	HNRNPU	--->	POU3F4	0.12637	-0.094265
[7867]	SMAD4	--->	GLI3	0.098419	-0.097905
[7868]	NEDD9	--->	TCF3	0.08423	-0.095808
[7869]	DDX39	<---	HIPK2	-0.29616	0.27819
[7870]	RB1	<---	SERPINB2	-0.45799	0.457
[7871]	ATF2	<---	ATF7	-0.050197	-0.01286
[7872]	SYK	--->	VAV1	1	-1.0027
[7873]	ID1	?	MYF5	-0.65635	-0.65635
[7874]	GTF3C1	<---	ASCC2	0.0091327	0.024115
[7875]	PTPN11	--->	BDKRB2	0.26077	-0.15667
[7876]	SRC	--->	ADAM12	0.59624	-0.53287
[7877]	NUMB	<---	PRKCB	-0.55841	0.55403
[7878]	MAP3K13	--->	MAPK8IP1	0.29172	-0.29846
[7879]	SERPING1	<---	MASP2	-0.042489	0.059553
[7880]	CSNK2A1	--->	EIF4EBP1	1.0012	-1.0031
[7881]	SOCS3	--->	IL6ST	0.13396	-0.15842
[7882]	KHDRBS3	<---	LNX1	-0.035217	0.040351
[7883]	CCR3	<---	HCK	-0.74253	0.90219
[7884]	IL6ST	<---	CNTF	-0.17092	0.15837
[7885]	PGR	<---	TRIM24	-0.088819	0.11471
[7886]	NFASC	--->	SCN1B	0.22872	-0.23346
[7887]	MYOD1	?	ID4	-0.65635	-0.65635
[7888]	CD28	<---	GRB2	-0.4617	0.44787
[7889]	MAP3K2	--->	YWHAE	0.54958	-0.30237
[7890]	PRAM1	<---	LCP2	-0.12402	0.02562
[7891]	RIN2	<---	BIN1	-0.16286	0.16101
[7892]	HIVEP1	<---	RAB1A	-0.49312	0.52514
[7893]	INPP5D	<---	PTPN11	-0.79614	0.6345
[7894]	LCK	--->	LCP2	1.0058	-0.99925
[7895]	TFPI2	<---	KLKB1	0.0096062	0.024385
[7896]	TNFRSF1A	<---	LTB	-1.0013	0.99951
[7897]	AXL	--->	GRB2	0.16646	-0.23252
[7898]	HERC3	--->	UBQLN1	0.23253	-0.22787
[7899]	TSSK1B	--->	CHEK2	0.13988	0.13882
[7900]	MAP4K1	--->	SHC1	0.091849	-0.11729
[7901]	HDAC5	<---	NCOR2	-0.14637	0.13483
[7902]	PIAS3	--->	SMAD4	1.0161	-0.98478

[7903]	NCKIPSD	<---	NCK1	-0.14951	0.38788
[7904]	BCAR1	--->	GRB2	0.018615	-0.01142
[7905]	C1QA	--->	COL2A1	-0.0029299	-0.0063243
[7906]	NPAS2	<---	RXRA	-0.06928	-0.016321
[7907]	NCF1	--->	CTTN	0.029629	-0.026462
[7908]	JAK1	<---	IL10RA	-0.052954	0.045309
[7909]	FOS	<---	CSNK2A1	-0.68783	0.53871
[7910]	TRAF6	--->	XIAP	0.32867	-0.33856
[7911]	CSF2RB	<---	IL3RA	-0.23067	-0.085657
[7912]	ST8SIA4	<---	NCAM1	-0.085249	0.094891
[7913]	ADRA1D	--->	SNTG1	0.343	-0.34434
[7914]	KHDRBS1	--->	PLCG1	0.21219	-0.24657
[7915]	RDX	<--->	EZR	0.004857	0.004857
[7916]	SH3GLB1	--->	HTT	0.085985	-0.071715
[7917]	RIPK3	--->	TNFRSF1A	0.63307	-0.42168
[7918]	IRAK2	--->	PELI1	0.85311	-0.63354
[7919]	CASP8	--->	APP	0.14591	-0.16277
[7920]	ZFPM2	<---	CTBP2	-0.063447	0.05497
[7921]	ARL4A	--->	KPNA2	0.49762	-0.47298
[7922]	WEE1	<--->	CDK1	0.23429	0.23429
[7923]	ARHGAP9	--->	SMAD9	0.23001	-0.23894
[7924]	CCR4	<---	ADRBK2	-0.22265	0.20207
[7925]	YWHAG	--->	TSC2	0.294	-0.29138
[7926]	ABL1	<---	YAP1	-0.10901	0.012183
[7927]	TIMP2	--->	MMP2	0.21399	-0.19321
[7928]	SORBS1	<---	INPPL1	-0.064752	0.056822
[7929]	SLC9A3R2	--->	CFTR	0.48971	-0.48738
[7930]	CEBPB	<---	ESR1	0.008943	0.026385
[7931]	WNT2	--->	FZD9	0.99836	-1.0014
[7932]	KAT5	--->	CDK5RAP2	0.015893	-0.0005401
[7933]	SORBS3	<---	MAPK3	-0.034505	-0.0080417
[7934]	TAOK3	<---	TRAF2	-0.9866	1.0023
[7935]	GRAP	<---	FASLG	-0.061935	0.033244
[7936]	SMN2	--->	SNRPB2	0.011379	0.0023256
[7937]	RPS6KA5	--->	EP300	0.11396	-0.12955
[7938]	HIVEP3	<---	TNFRSF1B	-0.07777	-0.0026438
[7939]	SGK1	--->	KPNA2	0.26102	-0.48542
[7940]	RFWD2	--->	JUND	0.13872	-0.16423
[7941]	IL7R	<---	FYN	-0.42775	0.44363
[7942]	HNRNPK	<---	RBMX	-0.0048144	0.036464
[7943]	MBIP	<---	CCDC85B	-0.020425	0.049461
[7944]	CBLB	--->	SORBS3	0.31754	-0.32919
[7945]	PRKCZ	<---	HRAS	-1.0135	0.99149
[7946]	CUL1	<---	CHUK	-0.33522	0.20221
[7947]	ENC1	<---	TGFBR1	-0.4026	0.36718
[7948]	ROR2	--->	FZD2	0.064759	-0.13628
[7949]	ELANE	--->	SERPINA3	0.11833	-0.082185
[7950]	F2	--->	SERPINE1	0.057207	-0.040728
[7951]	YAP1	<---	ERBB4	-0.015638	0.0068046
[7952]	DYRK1A	--->	YWHAB	0.39808	-0.42581
[7953]	SYT1	--->	GNB2L1	0.20192	-0.16272
[7954]	GP9	--->	GP1BB	0.12678	0.011867
[7955]	TP53	<---	HTT	-0.35185	0.36324
[7956]	SMAD2	<---	TGM2	-0.33148	0.41439
[7957]	IL23R	<---	JAK2	-0.3083	0.42544
[7958]	HIST3H3	<---	GADD45A	-0.10198	0.15343
[7959]	PLD2	--->	PLCG1	-0.014665	-0.058372
[7960]	F2	--->	PROZ	-0.0058289	-0.0067573
[7961]	GRIN1	<---	CDH2	-0.015956	0.009938
[7962]	TRPM7	<---	TRPM6	-0.0086682	-0.0084682
[7963]	CDH16	--->	CTNNB1	0.25376	-0.24971
[7964]	GHR	--->	SHC1	0.049696	-0.11813
[7965]	HDAC5	<---	SFN	-0.08049	0.08119
[7966]	MAP3K5	--->	YWHAZ	0.23745	0.17403
[7967]	EDA	--->	EDA2R	0.20082	-0.20241
[7968]	EP300	--->	CEBPB	0.18576	-0.18961
[7969]	HSPB1	--->	ACTC1	0.47908	-0.46795
[7970]	PGR	--->	ZBTB17	0.14623	-0.15777
[7971]	NCOR1	--->	PPARG	0.035695	-0.042687
[7972]	CREBBP	--->	KPNA2	0.18327	-0.18765
[7973]	SNX17	--->	LRP1	0.14576	-0.1549
[7974]	HSP90AA1	<---	SRC	-0.11357	0.11673
[7975]	MYOD1	<---	CDK4	-1.5271	1.5563
[7976]	LCP2	<---	GRB2	-0.50026	0.55945
[7977]	HIST3H3	<---	CHD1	-0.04883	0.049463
[7978]	PIK3R3	--->	PIK3CA	0.22525	-0.23451

[7979]	CSRP3	--->	MYF6	0.62562	-0.58677
[7980]	BIN1	--->	ITGA1	0.013218	-0.026571
[7981]	CTSD	--->	IGFBP3	0.41796	-0.40802
[7982]	FYN	--->	NEDD9	0.43979	-0.24387
[7983]	SMAD2	<---	TRIM62	-0.13684	0.15868
[7984]	NOTCH1	<---	EP300	-0.028609	0.014531
[7985]	ESR1	<---	EGFR	-0.18218	0.17852
[7986]	MMP9	--->	CD44	-0.0016332	-0.0037852
[7987]	MYBL2	--->	RBL1	0.096812	-0.10932
[7988]	GRAP2	--->	RBPMS	0.31256	-0.30113
[7989]	YWHAZ	<---	PAK4	0.020227	0.14174
[7990]	ZNF165	<---	ZNF446	-0.16454	-0.011024
[7991]	DAXX	<---	TGFBR2	-0.51623	0.51166
[7992]	STAT3	<---	GATA1	-0.59647	0.54434
[7993]	SCRIB	<---	ARHGEF7	-0.0069811	-0.0012714
[7994]	YWHAB	<---	HSPA5	-0.739	0.77111
[7995]	TRPV4	<---	SRC	-0.30344	0.33627
[7996]	ZNF764	<---	CCDC85B	-0.31174	0.31077
[7997]	TNFRSF1A	<---	IKBKB	-0.33958	0.20401
[7998]	CDK11B	--->	YWHAG	0.62776	-0.66368
[7999]	SNX9	--->	DNM1	0.080028	-0.10091
[8000]	ESR1	<---	RNF4	-0.12208	0.1317
[8001]	STK36	--->	GLI1	0.6053	-0.61388
[8002]	PKN1	--->	CCDC85B	-0.058369	-0.059264
[8003]	RPS6KA1	--->	NR4A3	0.56871	-0.73191
[8004]	SRPK1	--->	SFRS4	0.72994	-0.75755
[8005]	RTN4R	<---	MAG	-0.15987	0.15628
[8006]	NFATC2	<---	PRKCZ	-0.032373	0.019058
[8007]	AKT1	--->	PFKFB2	0.30563	-0.22251
[8008]	ASAP2	<---	RAN	-0.19165	0.18213
[8009]	APEX1	<---	HDAC1	-0.016259	0.031782
[8010]	KCNB1	?	KCNS3	-0.025901	-0.025901
[8011]	TLE1	<---	TNRC4	-0.07818	0.091195
[8012]	CBL	--->	ASAP1	0.12353	-0.13498
[8013]	MAPKBP1	<---	MAPK10	-0.62525	0.49987
[8014]	EGFR	--->	VAV2	0.99895	-0.99724
[8015]	PLEKHF2	--->	CCDC85B	0.13005	-0.12519
[8016]	STRAP	<---	C6orf203	-0.12061	0.15345
[8017]	CACNB4	--->	CACNA1A	0.16653	-0.17372
[8018]	RAI14	<---	YWHAG	-0.64224	0.6652
[8019]	PIK3R1	<---	RET	-0.40533	0.20684
[8020]	PKN1	--->	ACTN1	-0.02272	-0.02757
[8021]	HK2	--->	ZBTB17	0.15362	-0.1666
[8022]	PCBP1	<---	SFRS3	-0.0048144	0.036464
[8023]	ABL1	?	ABL2	-0.20182	-0.20182
[8024]	AR	<---	NCOR2	-0.09607	0.083273
[8025]	ENOX2	?	ENOX1	-0.016298	-0.016298
[8026]	ITGB2	<---	ICAM4	-0.13882	0.12788
[8027]	PRKACA	<---	ADRBK1	-0.049315	0.28927
[8028]	HIPK3	--->	C1QA	0.032326	-0.046185
[8029]	PITPNM2	<---	CPNE4	-0.032928	0.073246
[8030]	CNKSR1	--->	RALGDS	0.26615	-0.27483
[8031]	MAP3K10	--->	HTT	0.56202	-0.56234
[8032]	KDR	--->	ITGB3	0.1567	-0.15469
[8033]	STAMBP	<---	STAM2	0.031641	0.03798
[8034]	ROCK1	<---	RHOA	-1.0003	0.99991
[8035]	ZNF423	<---	EBF1	-0.24949	0.25206
[8036]	CXCR4	--->	PTK2	0.037413	-0.14397
[8037]	TNFSF11	--->	ADAM17	0.091154	-0.085137
[8038]	FYN	--->	MAP2	0.66812	-0.57565
[8039]	SMAD2	<---	DAB2	-0.30255	0.38606
[8040]	CUL1	--->	E2F1	0.086786	-0.085884
[8041]	PIK3R1	<---	PSEN1	-0.28152	0.17677
[8042]	ERN1	<---	MAP3K5	-0.6063	0.59704
[8043]	LCK	--->	RASA1	0.096327	-0.22768
[8044]	PAK1	--->	MAP2K1	1.0033	-1.0022
[8045]	BATF3	--->	ATF3	0.042106	-0.45144
[8046]	YWHAG	--->	PARD3	0.031301	-0.030232
[8047]	NRAP	<---	VCL	-0.47625	0.50132
[8048]	WWP1	--->	CPSF6	0.14734	-0.15788
[8049]	CEBPE	?	DDIT3	-0.11448	-0.11448
[8050]	INSR	<---	PTK2	-0.041494	-0.016988
[8051]	CREBBP	--->	HOXB4	0.10958	-0.11773
[8052]	RGS16	--->	GNAI1	1.0012	-1.0015
[8053]	PRKCE	--->	PECAM1	0.6326	-0.70025
[8054]	HOMER3	--->	ITPR1	0.11654	-0.12901

[8055]	RNF11	<---	WWP2	-0.26336	0.25647
[8056]	TGFBR1	--->	PIK3R1	0.10123	-0.14439
[8057]	FUBP1	<---	SMN1	-0.0094575	0.024632
[8058]	NID1	<---	COL4A1	-0.028712	0.016583
[8059]	EPS8	<---	ITGB1	-0.014778	-0.0048492
[8060]	KCNJ8	--->	ABCC8	0.027911	-0.029689
[8061]	LUM	--->	TRA2B	0.14379	-0.11631
[8062]	NCOA1	<---	VDR	-0.043163	0.034596
[8063]	AGGF1	<---	CCDC85B	-0.03795	0.066891
[8064]	NCK2	--->	AXIN1	0.064675	-0.076378
[8065]	RBL1	<---	CEBPA	-0.27423	0.26549
[8066]	TRAF3	<---	TNFRSF4	-0.13411	0.1285
[8067]	HDAC3	--->	PPARG	0.055257	-0.044928
[8068]	KIDINS220	<---	NTRK3	-0.17084	0.16577
[8069]	HIST4H4	<---	GADD45A	-0.10198	0.15343
[8070]	FZD4	<---	DLG4	-0.22894	0.18534
[8071]	EZR	--->	CTNNB1	0.30607	-0.3116
[8072]	RBX1	<---	CUL4B	-0.11034	0.12225
[8073]	ETV1	--->	ERG	-0.088177	-0.24955
[8074]	ATN1	<---	WWP1	-0.040646	0.027457
[8075]	HDAC5	--->	RFXANK	0.31873	-0.31259
[8076]	SPRY2	<---	CBL	-0.51277	0.50698
[8077]	CD70	--->	CD27	0.89674	-0.85556
[8078]	RBMXL2	?	TRA2B	-0.016298	-0.016298
[8079]	SETDB1	<---	TRIB3	-1.0006	1.0153
[8080]	CASP7	--->	RASA1	-0.026536	-0.078902
[8081]	VAV3	<---	ZYX	-0.13519	0.074728
[8082]	PBX3	--->	HOXB8	-0.059044	-0.084876
[8083]	MATN4	--->	MATN2	-0.01029	-0.011059
[8084]	NFATC1	--->	EP300	-0.0058464	-0.0074671
[8085]	HIST3H3	<---	FOXA1	-0.50372	0.55191
[8086]	ITCH	--->	DTX1	0.048559	-0.061809
[8087]	GRK5	--->	BDKRB2	0.13522	-0.19362
[8088]	DLG4	--->	ATP2B4	0.12627	-0.13697
[8089]	EDAR	--->	TRAF1	0.049773	-0.061642
[8090]	YES1	<---	JAK2	-0.085727	0.54485
[8091]	GNA12	--->	GNB1	0.31173	-0.29746
[8092]	USF1	<---	PRKDC	-0.2062	0.21168
[8093]	F2RL2	--->	CTSG	0.45462	-0.42368
[8094]	SOCS3	--->	IRS2	0.14262	-0.13554
[8095]	SHC1	<---	FGFR1	-0.14664	0.036624
[8096]	HRAS	<---	SOS1	-0.99973	0.99534
[8097]	MCL1	<---	BAD	-0.28245	0.2727
[8098]	MAPKAPK5	--->	PLA2G4A	0.45873	-0.58026
[8099]	JUP	--->	MUC1	0.019109	-0.029649
[8100]	C1QTNF5	?	MFRP	-0.010873	-0.010873
[8101]	PTPRS	--->	SETDB1	0.15112	-0.15928
[8102]	CASP3	--->	ACIN1	0.54334	-0.64977
[8103]	CASP8	--->	CHD3	0.040731	-0.065154
[8104]	AKT1	--->	YAP1	0.27513	-0.18807
[8105]	WASF2	<---	ABI2	-0.045711	0.042336
[8106]	FGD1	--->	CTTN	0.038253	-0.040989
[8107]	TLR1	<---	TLR9	0.054939	0.1252
[8108]	NCOA2	<---	OPN1LW	-0.14084	0.13299
[8109]	SFN	--->	TBL3	0.2164	-0.19355
[8110]	SYTL5	<---	RAB6A	-0.15731	0.20092
[8111]	LCK	--->	LAT	0.94263	-0.86359
[8112]	KAT2A	<---	EP300	-0.0041023	0.061921
[8113]	TP53	<---	BTK	-0.32733	0.30446
[8114]	VAV2	<---	GRB2	-0.11041	0.2794
[8115]	POU3F4	?	POU3F1	-0.11417	-0.11417
[8116]	ZAP70	--->	MUC1	0.98926	-1.0023
[8117]	AR	<---	MDM2	-1.0039	1.01
[8118]	PRKCA	--->	GNA15	0.89037	-0.89029
[8119]	IRAK3	--->	MYD88	0.10463	-0.095609
[8120]	RAB4A	--->	MAPK3	1.0132	-1.0074
[8121]	CASP1	--->	BCL2L1	0.43666	-0.44173
[8122]	BIN1	--->	ITGA6	0.012058	-0.022681
[8123]	GADD45G	--->	GDF9	0.16244	-0.12332
[8124]	SCARF1	--->	CALR	-0.0013817	-0.010882
[8125]	SFRS2IP	--->	SFRS1	0.12373	-0.11096
[8126]	PTPN6	--->	LIFR	0.99881	-0.85824
[8127]	PTPN11	--->	KIT	0.98781	-1.0015
[8128]	HNRNPK	<---	ITK	-0.77247	0.73153
[8129]	MYF5	<---	CALM1	-0.22666	0.2676
[8130]	CUBN	--->	ALB	0.016224	-0.012193

```
[8131]  PRKG2    --->    MYLK     0.34841 0.0043358
[8132]  FBLN1    --->    MOBKL3   0.17016 -0.13728
[8133]  HSH2D    <---    FES      -0.30065        0.37215
[8134]  PIAS4    --->    SMAD7    0.9933  -1.0027
[8135]  DLG2     --->    GRID2    0.045953        -0.051152
[8136]  SPDEF    <---    NKX3-1   -0.17089        0.19026
[8137]  BCOR     --->    BCL6     0.098697        -0.11304
[8138]  SMARCC1  --->    NR3C1    0.083516        -0.057729
[8139]  ZNF205   <---    CCDC85B  -0.31174        0.31077
[8140]  FLT4     --->    ITGA5    0.073952        -0.082971
[8141]  FOXG1    --->    SMAD2    0.52503 -0.44328
[8142]  MYO10    --->    CALM1    0.093674        -0.098954
[8143]  FST      --->    BMP5     1.0085  -0.99524
[8144]  FOS      <---    HNF1A    -0.42391        0.28971
[8145]  YAP1     --->    YWHAQ    0.62175 -0.61853
[8146]  RBM9     --->    ESR1     0.064959        -0.040633
[8147]  CSNK2A1  --->    NKX2-5   0.79979 -0.82765
[8148]  MAPK8IP1         <--->    MAPK8IP2        0.004063        0.004063
[8149]  PRKCB    --->    EIF4E    0.94696 -0.94609
[8150]  HMOX2    --->    APP      0.36191 -0.37186
[8151]  MMP13    --->    LRP1     0.076775        -0.087531
[8152]  JAK2     --->    FES      0.048415        -0.11697
[8153]  ERF      <---    HMGB1    -0.36128        0.40261
[8154]  MARK4    <---    CDC42    -0.034246       0.021753
[8155]  PLK1     --->    CDC25C   0.33126 -0.32604
[8156]  PRKD1    <---    BTK      0.201   0.34925
[8157]  PTPN12   --->    ABL1     0.049094        -0.14288
[8158]  MYOD1    <---    SMAD4    -1.0094 1.0041
[8159]  DLL1     <---    POFUT1   -1.0071 1.0038
[8160]  CRADD    <---    RIPK1    -0.16538        -0.10773
[8161]  BMPR2    <---    BMP7     -0.99943        1.0057
[8162]  SMAD2    --->    SP1      0.98558 -0.99936
[8163]  CASP7    --->    PRKCZ    0.19066 -0.19986
[8164]  EGFR     --->    GAB1     0.9971  -0.98971
[8165]  TBC1D4   --->    NFAT5    -0.00054624     -0.0088325
[8166]  ITGAX    <---    FCER2    -0.088435       0.076846
[8167]  TF       --->    IGFBP4   0.072099        -0.038154
[8168]  SETDB1   <---    CBX8     -0.16569        0.17121
[8169]  CDH4     ?       CDH2     -0.059787       -0.059787
[8170]  COL9A3   <---    COL2A1   -0.00020668     -0.00019769
[8171]  CD244    --->    SH2D1B   1.0361  -1.0439
[8172]  IKBKB    --->    PEBP1    0.37377 -0.58309
[8173]  TSHR     <---    HSPA5    -0.18048        0.20329
[8174]  PHIP     <---    IRS1     -0.086549       0.087634
[8175]  SNIP1    --->    SMAD2    0.26592 -0.22069
[8176]  THRB     ?       NR2F6    -0.0089162      -0.0089162
[8177]  IL1R1    <---    IL1RN    -0.28221        0.28289
[8178]  EFEMP2   <---    RHPN1    -0.00087176     0.018478
[8179]  CYTH2    --->    ARRB2    0.1548  -0.14696
[8180]  RIT1     --->    CUL3     0.11776 -0.10799
[8181]  RNF216   <---    TLR9     -0.19872        0.19705
[8182]  MTSS1    <---    HTT      -0.045873       0.079806
[8183]  SNX1     --->    LEPR     0.3803  -0.38118
[8184]  ADAM17   --->    DLG1     0.00068077      -0.0098264
[8185]  CD40LG   --->    CD40     0.99433 -1.0079
[8186]  UPF3A    --->    THOC4    0.021343        -0.0076165
[8187]  DAB1     --->    LRP1     0.14403 -0.15309
[8188]  RAB1A    --->    SLC16A8  0.36707 -0.3359
[8189]  TSHR     --->    FN1      0.34184 -0.32714
[8190]  VSNL1    --->    ACTB     0.33163 -0.32679
[8191]  MPP3     <---    SCN2A    -0.034928       0.041469
[8192]  SOS1     --->    EPS8     0.0028479       -0.026389
[8193]  PRKACA   --->    LRP1     0.22111 -0.23243
[8194]  DLG1     ?       DLG3     -0.0075176      -0.0075176
[8195]  VDR      --->    SMAD3    0.22689 -0.17818
[8196]  YWHAG    <---    MAP3K7IP1        -0.4329 0.45906
[8197]  PRKCE    --->    BRAF     0.38645 -0.51763
[8198]  TP53     <---    HSPA1A   -0.65475        0.6691
[8199]  ATN1     --->    GAPDH    0.020037        -0.026889
[8200]  STRN     <---    ESR1     -0.0096005      0.034681
[8201]  TIMP4    --->    MMP14    0.21399 -0.19321
[8202]  HUWE1    --->    CCL1     0.10666 -0.11382
[8203]  TRPV4    <---    HCK      -0.29213        0.34238
[8204]  ARHGEF12         --->     IGF1R   -6.0094e-007    -0.013163
[8205]  KCNC1    --->    ANK3     0.026829        -0.040665
[8206]  TTF2     --->    SMAD1    0.10411 -0.11977
```

[8207]	TRIM17	<---	TRIM39	-0.0037292	0.012462
[8208]	PAK1IP1	<---	SMURF1	-0.40962	0.40156
[8209]	EGFR	--->	PIK3R2	0.23248	-0.28371
[8210]	BCL2A1	<---	BCL2L11	-0.021317	0.020672
[8211]	RAD18	<---	UBE2B	-0.17337	0.20782
[8212]	SLC9A3R1	--->	SLC22A5	0.37001	-0.34102
[8213]	THBS1	<---	CD36	-0.2868	0.38122
[8214]	MAPK1	--->	GRB2	0.22814	-0.32468
[8215]	PPIB	<---	UBQLN4	-0.065287	0.10133
[8216]	PPP2CA	--->	RHO	0.25904	-0.26619
[8217]	ANKRD11	--->	NCOA2	0.023538	-0.033054
[8218]	ACVR1	--->	USP39	0.065834	-0.084034
[8219]	MAGI1	<---	RPS6KA1	-0.073246	0.032672
[8220]	ZBTB16	<---	BMI1	-0.11838	0.11708
[8221]	MAPK8	--->	PPARG	0.60373	-0.61504
[8222]	SRC	--->	NOS2	0.23189	-0.22855
[8223]	VSX2	<---	PAX6	-0.10489	-0.034251
[8224]	PGF	<---	VEGFA	0.00053249	0.062251
[8225]	ZBTB16	<---	EPN1	-0.1184	0.11691
[8226]	RBMX	--->	KHDRBS3	0.055977	-0.042052
[8227]	PYY	--->	NPY1R	0.20502	-0.19468
[8228]	PPARGC1A	--->	SFRS4	-0.0021891	-0.0095939
[8229]	SELP	--->	VCAN	-0.0092596	-0.018576
[8230]	NID1	--->	APP	0.12701	-0.13946
[8231]	PRKCA	--->	CREM	0.54759	-0.54466
[8232]	CFL1	<---	YWHAZ	-0.067982	0.076969
[8233]	ARPC1B	--->	WAS	0.13229	-0.1718
[8234]	CCR5	--->	CD4	0.30758	-0.29998
[8235]	ATXN1	<---	TOX4	-0.076453	0.072938
[8236]	MECR	--->	RXRA	0.04226	-0.25235
[8237]	MYL1	--->	ACTA2	0.57525	-0.57177
[8238]	EP300	--->	ALX1	0.11326	-0.12156
[8239]	BRSK2	<---	WEE1	0.13714	0.14515
[8240]	TRPC3	<---	CALM1	-0.13987	0.12925
[8241]	NCAN	<---	TNC	-0.0087187	-0.0051562
[8242]	MAP2K1	--->	EP300	0.19604	-0.19015
[8243]	CADPS	<---	DMD	-0.01098	-0.0020506
[8244]	GATA6	<---	NKX2-1	-0.015702	0.056147
[8245]	ST13	<---	HSP90AA1	-0.059965	0.052004
[8246]	HOXA5	--->	TWIST1	0.18332	-0.17905
[8247]	CAPN3	--->	FLNC	0.2655	-0.26527
[8248]	DUSP9	--->	MAPK1	1.0016	-1.0004
[8249]	TP53	<---	EGR1	-0.14061	0.14131
[8250]	INADL	--->	KCNJ15	0.044422	-0.0070376
[8251]	APBB1	--->	ABL1	-0.032024	-0.057879
[8252]	ADCY8	<---	PPP2CA	-0.30964	0.30269
[8253]	GADD45B	<---	MAP3K5	0.13651	0.27933
[8254]	MET	--->	STAT3	0.52364	-0.64294
[8255]	PTPN1	--->	BCR	0.18717	-0.19601
[8256]	SPAG16	<---	SPAG6	-0.073824	0.10509
[8257]	SNX6	--->	INSR	0.13241	-0.14074
[8258]	CASP8	--->	MAPT	0.43362	-0.52325
[8259]	GFI1B	<---	HGS	-0.040336	0.022069
[8260]	CBX3	<---	PIM1	-0.72609	0.70015
[8261]	UTRN	<---	ACTN2	-0.014003	-0.012336
[8262]	NKIRAS1	--->	NFKBIB	0.98515	-0.95568
[8263]	AKT1	--->	MDM2	0.99136	-0.99587
[8264]	RHOD	--->	SMAD4	0.55322	-0.47159
[8265]	SOX7	<---	SMAD7	0.024132	0.10834
[8266]	NKX2-1	--->	RELA	0.08441	-0.079104
[8267]	TCF20	--->	ESR1	0.075814	-0.050029
[8268]	CALB1	--->	TRPV6	0.082402	-0.077145
[8269]	ADAP1	--->	NCL	0.062691	-0.060154
[8270]	PTPN6	--->	CD33	0.65471	-0.5666
[8271]	GOPC	<---	RHOQ	-0.2357	0.2721
[8272]	IP6K2	--->	PLSCR1	0.40196	-0.37727
[8273]	TRAF6	--->	TNFAIP3	0.36781	-0.38124
[8274]	PRKCZ	<---	PRKCD	-0.0071611	0.068471
[8275]	MYC	<---	CEBPB	-0.23335	0.24948
[8276]	MAPK1	--->	GATA1	1.0021	-0.99372
[8277]	DDX20	--->	SMN1	0.0045634	-0.00071951
[8278]	HDAC6	<---	RELB	-0.2365	0.23166
[8279]	PLAT	<---	LAMA1	-0.0052647	-0.0021936
[8280]	LPXN	--->	ITGA4	0.034763	-0.040684
[8281]	DHX30	--->	TBL3	0.091156	-0.076481
[8282]	UBE2I	<---	STAT1	-0.25988	0.13656

[8283]	SMAD1	--->	AR	0.56034	-0.56788
[8284]	CASP6	--->	PSEN1	0.58173	-0.69269
[8285]	ELANE	--->	TFPI	0.024385	0.0096062
[8286]	NEUROG1	<---	SMAD1	-1.0051	0.98546
[8287]	HTR6	--->	GNAQ	0.9872	-0.98627
[8288]	BMP3	<---	COL4A2	-0.1033	0.098437
[8289]	MMP2	<---	COL6A3	-0.037985	0.028819
[8290]	RHOXF2	--->	TOLLIP	0.013128	-0.0096635
[8291]	GFI1B	<---	EGFL7	-0.061525	0.051302
[8292]	BCL2L1	<---	CRYAB	-0.6937	0.70968
[8293]	LPHN1	<---	DLG3	-0.10924	0.1027
[8294]	ITGB2	<---	ICAM1	-0.093233	0.081031
[8295]	YWHAG	--->	PGAM5	0.25479	-0.24377
[8296]	SLC9A3R1	<---	P2RY1	-0.40599	0.42206
[8297]	JUND	<---	MAPK3	-1.0148	1.0022
[8298]	UBE2I	--->	TRIM63	0.072109	-0.039081
[8299]	SIRT1	--->	RELA	0.037635	-0.048408
[8300]	CEACAM6	--->	CEACAM1	-0.00010158	-0.022352
[8301]	FNTA	<---	ACVR1	-0.30066	0.2968
[8302]	TP73	<---	NEDD4	-0.074876	0.063701
[8303]	PRKCH	<---	SRC	-0.051826	0.06379
[8304]	MST1R	--->	PIK3R1	0.21153	-0.41
[8305]	PIK3R1	<---	SYK	-0.74025	0.60059
[8306]	WAS	<---	C5AR1	-0.38351	0.3433
[8307]	ZBTB16	<---	TXNIP	-0.074558	0.068158
[8308]	RAC2	--->	SMAD4	0.55322	-0.47159
[8309]	DYRK1A	--->	FOXO1	0.19737	-0.20874
[8310]	SNX6	<---	ACVR2B	-0.2683	0.23826
[8311]	HNRNPK	<---	CSK	-0.97195	1.133
[8312]	ATXN1	--->	ZBTB32	0.0395	-0.050885
[8313]	HOMER2	--->	ITPR1	0.32691	-0.33683
[8314]	TAF1	--->	FOS	0.11403	-0.17209
[8315]	MAPK1	--->	CASP9	0.99863	-0.99771
[8316]	CDX2	<---	CREBBP	-0.18943	0.18505
[8317]	UBE3A	<---	BLK	-0.24329	0.29397
[8318]	CCL8	<---	CCR1	-0.46475	0.49595
[8319]	SFRS7	<---	CPSF6	-0.16333	0.20038
[8320]	CTBP1	<---	BMPR2	-0.29717	0.2481
[8321]	ARHGEF6	--->	ARHGEF7	-0.0030535	-0.0039409
[8322]	PIAS4	--->	HDAC1	0.055864	-0.053948
[8323]	CABP1	--->	CACNA1A	0.12519	-0.13198
[8324]	PTGS1	--->	NUCB1	0.17762	-0.16009
[8325]	P2RY2	--->	ITGAV	0.043335	-0.055528
[8326]	SMAD2	<---	GATAD2B	-0.27788	0.36388
[8327]	CCNC	<---	CDK3	-1.1093	1.083
[8328]	CNKSR1	--->	CADM4	0.016058	-0.026971
[8329]	MYOG	--->	TCF3	-0.13552	-0.39218
[8330]	ITSN1	--->	CDC42	0.0049639	-0.0061254
[8331]	LPXN	--->	PHF21A	0.050813	-0.015467
[8332]	ITSN1	--->	LMO4	0.049234	-0.050713
[8333]	SMAD4	<---	FOXO1	-0.083636	0.082517
[8334]	DNM2	<---	ITSN1	-0.038616	0.026398
[8335]	WRN	--->	TP53	0.21885	-0.21736
[8336]	TRIB1	<---	MAP2K1	-0.99672	1.0159
[8337]	NFKBIA	<---	HOXB7	-0.75369	0.74842
[8338]	SMAD2	<---	HNF4A	-0.24224	0.26585
[8339]	MEGF6	--->	GFI1B	-0.0058332	-0.0080491
[8340]	HOXA9	?	PBX1	-0.11417	-0.11417
[8341]	IRAK2	<---	SMURF1	-0.3668	0.39401
[8342]	CD93	<---	COL1A1	-0.026834	0.013094
[8343]	MMP11	<---	PRPF40A	-0.39628	0.43032
[8344]	KCNIP3	--->	PSEN1	0.2532	-0.23708
[8345]	CRKL	--->	WAS	0.28805	-0.33826
[8346]	MAPKAPK5	--->	TP53	0.58544	-0.66628
[8347]	PLG	<---	HMGB1	-0.11778	0.13248
[8348]	SYK	--->	PLCG2	1.0007	-1.0015
[8349]	CAMKK1	--->	CAMK1D	0.83082	-0.43719
[8350]	SHH	<---	HHIP	-0.99342	0.98842
[8351]	HDAC3	<---	NCOR1	-0.064436	0.058272
[8352]	CHUK	<---	TGFBR1	-0.18636	0.21752
[8353]	HCK	--->	CSF3R	0.64184	-0.56719
[8354]	NKX2-1	<---	MAPK1	-0.82765	0.79979
[8355]	BCL3	<---	GSK3B	-0.26299	0.25926
[8356]	CDK9	--->	PIN1	0.46953	-0.49485
[8357]	ATRX	<---	PTPN4	-0.076917	0.062034
[8358]	MAPK1	--->	STAT3	1.0077	-1.0166

[8359]	RBM39	--->	BAT1	0.031664	-0.033526
[8360]	CUGBP1	<---	TOM1L1	0.0051365	0.026597
[8361]	PTK2	--->	SELE	0.025081	-0.09449
[8362]	ATF1	<---	BRCA1	-0.20871	0.2343
[8363]	CDK9	--->	IL6ST	0.29225	-0.31878
[8364]	TRAP1	<---	TNFRSF1A	-0.028014	0.21246
[8365]	ELANE	--->	SERPINF2	0.11833	-0.082185
[8366]	IRS2	--->	BCL2L1	0.063272	-0.073389
[8367]	ITGA5	<---	ERBB2	-0.082313	0.067256
[8368]	RAB11FIP5	<---	RAB11A	-0.10724	0.12837
[8369]	STAT5B	<---	GHR	-0.18191	0.16532
[8370]	CDKN2A	<---	ZFP91	-0.050276	0.057328
[8371]	ELK1	--->	FOS	0.13423	-0.21537
[8372]	PARD6B	<---	PARD3	-0.0089531	-0.0060714
[8373]	DLGAP1	<---	DLG4	-0.040133	0.038414
[8374]	TCF4	<---	ID3	-0.37599	-0.054725
[8375]	PRKCG	--->	OCLN	0.94019	-0.93859
[8376]	SOS2	<---	GRB2	-0.99897	1.0047
[8377]	FGFR3	<---	FGF23	-0.61087	0.60861
[8378]	DRD2	--->	ADORA2A	0.31807	0.24405
[8379]	PGR	<---	RBM39	-0.12846	0.16436
[8380]	PAK1	--->	NF2	0.23985	-0.14532
[8381]	RB1	<---	CDKN1A	-0.28611	0.27875
[8382]	CALM1	--->	AR	0.93818	-0.93861
[8383]	PTPN11	--->	SOCS3	0.57542	-0.72435
[8384]	HSPH1	<--->	HSPA8	0.94322	0.94322
[8385]	ARL8B	<---	TGFBR1	-0.2793	0.24366
[8386]	PRKCD	--->	KCNJ4	0.81962	-0.8066
[8387]	FXR2	<---	CCDC85B	-0.070138	0.099236
[8388]	ITGA1	<---	LAMA1	-0.095289	0.083714
[8389]	PAX5	--->	RB1	0.23669	-0.24155
[8390]	CASP6	--->	HTT	0.60022	-0.71121
[8391]	NONO	<---	PLEKHF2	-0.16326	0.16967
[8392]	UBR5	--->	KPNA1	0.15361	-0.12103
[8393]	CD274	<---	PDCD1	-0.057081	-0.042802
[8394]	F2	?	PLAU	-0.010603	-0.010603
[8395]	TP53	<---	FBXO11	-0.39539	0.40666
[8396]	SKP1	--->	TBL1X	0.031647	-0.040124
[8397]	RGS2	<---	PRKCB	-0.87353	0.87438
[8398]	PTK2B	<---	ARHGAP5	-0.095994	0.047041
[8399]	ACVR2B	<---	INHBA	-0.99943	1.0057
[8400]	PLCG2	<---	TEC	-1.0027	1.0063
[8401]	AKAP1	<---	PRKAR1B	-0.27544	0.28956
[8402]	TFDP2	<---	YWHAE	-0.09793	0.10009
[8403]	NCOA1	--->	STAT6	0.023993	-0.039199
[8404]	PRKACA	<--->	GSK3B	0.23429	0.23429
[8405]	KHDRBS1	<---	LYN	-0.61607	0.6674
[8406]	ATN1	--->	RBM10	0.030733	-0.032927
[8407]	ASGR2	--->	ASGR1	-0.019543	-0.15638
[8408]	CASP3	--->	SRF	0.32345	-0.39257
[8409]	YWHAZ	<---	BCR	-0.083313	0.078735
[8410]	HIPK1	--->	SNIP1	0.30806	-0.32435
[8411]	THRA	<---	FUS	-0.049691	0.059211
[8412]	TCL1A	--->	CALCOCO2	0.13092	-0.10001
[8413]	ELF2	<---	LMO2	-0.1992	0.24264
[8414]	CRKL	--->	SOS1	0.60392	-0.61023
[8415]	SMAD2	--->	HOXD13	0.20304	-0.14259
[8416]	MAP3K2	--->	MAP2K4	0.99233	-0.99622
[8417]	GSK3B	--->	CDH1	0.39746	-0.41519
[8418]	EBF1	--->	PAX5	0.25193	-0.22968
[8419]	DLG1	--->	KCNJ2	0.04551	-0.04695
[8420]	MAP2K4	--->	MAPK14	1.0074	-0.9992
[8421]	SH3KBP1	--->	CD2	0.12598	-0.1175
[8422]	BMPER	--->	BMP6	0.71403	-0.67452
[8423]	ZMYND8	<---	PRKCB	-0.9063	0.90788
[8424]	RHOJ	--->	TGFBR1	0.010625	-0.037483
[8425]	TRIB3	--->	RELA	1.0015	-1.0004
[8426]	CRK	--->	STAT5A	0.32804	-0.36563
[8427]	YWHAB	--->	MAPT	0.29081	-0.2771
[8428]	SMURF1	--->	STRAP	0.71781	-0.72185
[8429]	ADAM10	--->	DLL1	1.0058	-0.99787
[8430]	COL17A1	--->	CLSTN1	0.011338	-0.022411
[8431]	SH3RF1	<---	MAP3K10	-0.047173	0.16486
[8432]	CASP1	--->	MAPT	0.12628	-0.12396
[8433]	CABP2	?	PPP3R1	-0.37586	-0.37586
[8434]	FAM46A	<---	ZFYVE9	-0.13332	0.12576

[8435]	PLG	--->	MMP3	0.11927	-0.11212
[8436]	GADD45B	--->	PPARA	0.017932	0.0079001
[8437]	CREBBP	--->	RXRG	0.10538	-0.12158
[8438]	YWHAB	<---	PRKCG	-0.90439	0.90517
[8439]	CASP7	--->	MAPT	0.50832	-0.57833
[8440]	SMAD2	<---	MEF2C	-0.004081	0.0051714
[8441]	RAC1	<---	PRKCA	-0.99154	1.0123
[8442]	LMO2	--->	MAPRE2	0.066481	-0.030995
[8443]	PAK2	--->	NPHP1	0.34188	-0.089306
[8444]	ARHGEF7	<---	CBLB	-0.11155	0.099184
[8445]	ERN1	<---	HSPA5	-0.60495	0.6039
[8446]	SYK	--->	CTTN	0.78127	-0.61639
[8447]	AFAP1L2	<---	RASA1	-0.044914	0.17574
[8448]	IRS2	<---	PTPN11	-0.21679	0.2463
[8449]	RORA	<---	LMO3	-0.062299	0.088883
[8450]	TSHR	--->	GNAS	0.65587	-0.65379
[8451]	TRDN	<---	HRC	-0.060013	0.091383
[8452]	IRS2	--->	PIK3R1	-0.010246	-0.012384
[8453]	STAT1	--->	STAT5B	-0.053818	-0.057776
[8454]	CEACAM8	--->	CEACAM1	-0.00010158	-0.022352
[8455]	ABCA1	<---	LIN7A	-0.0097741	0.0027931
[8456]	VPS4B	<--->	VPS4A	0.00085354	0.00085354
[8457]	TNNI3K	--->	ACTA1	0.38604	-0.40491
[8458]	SHOC2	<---	NRAS	-0.0046468	0.015643
[8459]	RIPK1	--->	FADD	0.16806	-0.34782
[8460]	SYK	--->	CD22	0.35328	-0.39759
[8461]	MAPK8IP2	<---	MAPK8	-0.30961	0.2909
[8462]	CDK2	--->	BRCA1	0.32242	-0.34057
[8463]	ATXN1	<---	YWHAZ	-0.051337	0.045761
[8464]	ADCY7	<---	PRKCD	-0.78256	0.79222
[8465]	GNAI1	<---	CNR1	-0.99753	1.0047
[8466]	PRKCA	--->	DGKZ	0.50196	-0.17365
[8467]	LIMS1	<---	SMURF1	-0.68627	0.68232
[8468]	VAV3	--->	PLCG1	0.0092125	-0.04917
[8469]	TRPV4	--->	PACSIN1	0.035277	-0.038389
[8470]	TRIM37	<---	TRAF6	-0.030264	0.67424
[8471]	RGS14	<---	RAP1A	-0.99028	1.0151
[8472]	IKBKE	--->	HSP90AB1	0.019127	-0.032587
[8473]	DYSF	--->	CAV3	0.068512	-0.034382
[8474]	MDC1	<---	BRCA1	-0.020991	0.036669
[8475]	PSD	<---	RAB40C	-0.26817	0.28141
[8476]	CCNA1	<---	MYBL2	-0.23718	0.2333
[8477]	DAXX	<---	HIPK3	-0.48387	0.3655
[8478]	PRKCD	--->	ACTB	0.48294	-0.48348
[8479]	RASSF5	<---	RAP1B	-0.98684	1.0025
[8480]	FNTA	<---	RHOB	-0.44041	0.47292
[8481]	MCL1	--->	BCL2L1	-0.23385	-0.7984
[8482]	DAB2	<---	GRB2	-0.33801	0.38689
[8483]	IBTK	<---	BTK	-0.55104	0.51738
[8484]	CSNK2A2	--->	KIF1C	0.42625	-0.44778
[8485]	CTBP1	--->	ZEB1	0.043292	-0.055921
[8486]	SMAD3	<---	KAT2B	-1.0011	0.99663
[8487]	RASGRP2	--->	PTCHD2	0.37184	-0.39054
[8488]	ODZ3	--->	SMAD4	0.11936	-0.11108
[8489]	IL6ST	--->	STAT3	0.13331	-0.15767
[8490]	LPHN1	--->	ANKS1A	0.064599	-0.074918
[8491]	BCR	--->	YWHAQ	0.078735	-0.083313
[8492]	HSPB8	<---	BAG3	-0.15881	0.17717
[8493]	DNMBP	--->	WASL	0.03134	-0.050671
[8494]	ESR1	?	NR2F6	-0.0089162	-0.0089162
[8495]	TRAF1	--->	GFI1B	0.086117	-0.092754
[8496]	CDK4	--->	SETDB1	1.0153	-1.0006
[8497]	GRK4	--->	CALM1	0.89196	-0.90051
[8498]	PTPRJ	--->	CTNNB1	0.37022	-0.37559
[8499]	FOS	<---	RELA	-0.080712	0.042033
[8500]	ITGB2	<---	GNB2L1	-0.21976	0.21072
[8501]	RBBP5	--->	RB1	0.27034	-0.27686
[8502]	CSNK2A1	--->	SP1	0.12886	-0.14656
[8503]	TFDP2	<---	E2F2	-0.064857	-0.0088085
[8504]	GDF9	<---	RAB27A	-0.60202	0.63754
[8505]	TRAF6	--->	TLR3	0.34304	-0.34966
[8506]	KIT	<---	LCK	-0.37583	0.44251
[8507]	TF	--->	IGFBP5	0.072099	-0.038154
[8508]	VASP	--->	ACTG1	0.9951	-1.0004
[8509]	MDM4	--->	YWHAZ	0.040034	-0.045818
[8510]	EGFR	<---	EPHA2	0.059875	0.09263

[8511]	CFLAR	--->	RAF1	0.11654	-0.16284
[8512]	TRAF6	--->	MAP3K3	0.17832	-0.13621
[8513]	DLG1	--->	ACTA1	0.21438	-0.22546
[8514]	GRN	--->	ELANE	0.018634	0.015329
[8515]	CASP3	--->	FYN	1.0113	-1.0017
[8516]	DAB2	--->	ITGB3	0.030477	-0.03265
[8517]	LILRB4	<---	PTPN6	-0.17152	0.29453
[8518]	EP300	--->	MYOD1	0.19969	-0.20391
[8519]	FARP2	--->	YWHAG	0.019193	-0.028794
[8520]	PARD6A	<---	PRKCI	-0.058941	0.043604
[8521]	ARR3	<---	MAP2K7	-0.44306	0.42184
[8522]	MPP6	<---	EIF3G	-0.039212	0.044868
[8523]	KCNQ5	?	KCNQ3	-0.025901	-0.025901
[8524]	HDAC1	--->	CDC20	0.11313	-0.098152
[8525]	TP53	<---	WDR33	-0.11996	0.11521
[8526]	PPP1CA	--->	CDH1	0.10417	-0.11545
[8527]	BSPRY	<---	YWHAZ	-0.018028	0.027292
[8528]	IGF1	--->	IGFBP6	0.087568	-0.072295
[8529]	YWHAG	<---	PLEKHA5	-0.17951	0.17447
[8530]	EFEMP1	<---	RAF1	-0.03651	0.011308
[8531]	EGFR	<---	MAPK1	-0.32251	0.29741
[8532]	CASP9	--->	BCL2L1	0.43666	-0.44173
[8533]	RBBP4	--->	RB1	0.27528	-0.2808
[8534]	FHL2	--->	ITGB1	0.1401	-0.14924
[8535]	ATN1	--->	AGRN	0.0021556	-0.013512
[8536]	CCR9	--->	CCL25	0.49595	-0.46475
[8537]	SYK	--->	DUSP3	1.0053	-1.0087
[8538]	PAX8	<---	EP300	-0.12275	0.11484
[8539]	E2F6	<---	BMI1	-0.015216	0.047847
[8540]	CREBBP	--->	H3F3A	0.18896	-0.19276
[8541]	CD2AP	<---	BCAR1	-0.012591	0.02037
[8542]	NR0B1	--->	AR	0.010881	-0.95554
[8543]	SMAD5	<---	FLNA	-0.29902	0.28046
[8544]	RELA	<---	HMGB1	-0.14261	0.14817
[8545]	BRD4	--->	CCNT1	0.27778	-0.26313
[8546]	ESR2	<---	PPARGC1A	-0.021031	0.013278
[8547]	CBLB	--->	SPRY2	0.50698	-0.51277
[8548]	JAK2	--->	STAT3	0.99602	-0.98887
[8549]	BMP6	--->	ACVR2A	1.0057	-0.99943
[8550]	RAB11B	--->	IKBKG	0.50736	-0.50751
[8551]	NBN	--->	H2AFX	0.24372	-0.19249
[8552]	CCNA1	--->	RBL1	0.42536	-0.43637
[8553]	RAPGEF6	--->	MRAS	1.0006	-0.99699
[8554]	SMAD4	--->	SMAD9	1.0013	-0.9962
[8555]	RARA	--->	CCND3	0.3199	-0.31113
[8556]	CRYAA	--->	ALB	0.027716	0.018811
[8557]	F11	--->	SERPINF2	0.11833	-0.082185
[8558]	MAPK9	--->	BCL2L11	0.43567	-0.45207
[8559]	SPINT1	<---	HGFAC	-0.012923	0.0097419
[8560]	BIN1	<--->	AMPH	0.0094254	0.0094254
[8561]	BCL2	<---	MYC	-0.10873	0.10673
[8562]	THRB	<---	PPARGC1A	-0.021031	0.013278
[8563]	RRAS2	<---	SMURF2	-1.0084	1.0007
[8564]	TRIP4	--->	AR	0.94738	-0.94802
[8565]	JAK3	--->	ALK	0.52724	-0.23559
[8566]	FGF5	--->	FGFR1	1.0053	-0.99871
[8567]	NCKIPSD	--->	ARHGEF7	0.049924	-0.060281
[8568]	PTPN6	--->	CEACAM1	0.31282	-0.27672
[8569]	SPINK5	--->	PRSS1	0.083014	-0.048933
[8570]	PSEN2	<---	SRI	-0.23708	0.2532
[8571]	PRKCG	--->	GRIN2B	0.22109	-0.2311
[8572]	PDZD3	<---	SLCO1A2	-0.038024	0.07475
[8573]	HOXD10	<---	CREBBP	-0.18943	0.18505
[8574]	C7	<---	PLG	-0.044943	0.047838
[8575]	LYST	<---	HNRNPM	-0.10831	0.13878
[8576]	CDK17	--->	TGFBR1	0.36359	0.02614
[8577]	PHC1	--->	PHC2	0.15283	0.14921
[8578]	PIAS1	--->	DNMT3A	0.11786	-0.10655
[8579]	LAMA1	<---	C3	-0.041699	0.035698
[8580]	IKZF1	<---	HDAC1	-0.060684	0.065285
[8581]	TRIO	--->	FLNA	0.046884	-0.062072
[8582]	LCK	--->	STAT1	0.324	-0.25356
[8583]	SHANK1	--->	DNM2	-0.0024063	-0.010528
[8584]	PRKCA	--->	RAF1	0.99386	-0.99255
[8585]	NCOR1	--->	RARA	0.063164	-0.065644
[8586]	LYN	--->	CSF2RA	1.0933	-1.0787

[8587]	APBA2	--->	RELA	0.042414	-0.048869
[8588]	SIX1	<---	CCDC85B	-0.13707	0.16418
[8589]	MAPK8IP1	<---	MAP3K12	-0.30003	0.29445
[8590]	MAPT	--->	ACTB	0.17518	-0.17617
[8591]	ARHGEF6	<---	TGFBR2	-0.15359	0.14649
[8592]	CSF2RB	<---	PRKCB	-0.57071	0.56393
[8593]	GSK3B	--->	DYNC1I1	0.50151	-0.52686
[8594]	VDR	--->	BAG1	0.025363	-0.0036637
[8595]	PRKCA	--->	ACTA1	0.56912	-0.57516
[8596]	CCND3	<---	ATF5	-0.21338	0.22707
[8597]	AKT1	--->	EZH2	0.45566	-0.30774
[8598]	VAV1	<---	RAF1	-0.2614	-0.013086
[8599]	LCK	--->	FAS	0.50256	-0.4346
[8600]	SOX6	<---	CTBP2	-0.45523	0.46848
[8601]	NR2E3	?	NR1D1	-0.0089162	-0.0089162
[8602]	IGFBP7	--->	INHBA	0.23511	-0.22577
[8603]	NRXN2	<---	CASK	-0.14214	0.1271
[8604]	YWHAB	--->	SFRS3	0.092231	-0.070624
[8605]	TP73	<---	KAT2B	-0.077285	0.073753
[8606]	IGF1R	<---	CSK	0.010255	0.10882
[8607]	GHRL	--->	MLNR	1.0107	-0.98958
[8608]	CBX5	<---	DNMT3B	0.0073508	0.026698
[8609]	CFLAR	--->	DAXX	0.20967	-0.27062
[8610]	PDK3	--->	PDHA1	1.0004	-0.99606
[8611]	GIPC1	--->	IGF1R	0.029832	-0.040736
[8612]	MMP14	<---	ITGAV	-0.007497	-0.0041766
[8613]	ATN1	--->	SYVN1	0.010716	-0.017666
[8614]	GZMB	--->	CASP9	0.039556	-0.031345
[8615]	RIN2	<---	RAB5C	-0.99801	1.0021
[8616]	CSH1	--->	PRLR	0.0031206	-0.015544
[8617]	PVRL1	--->	MLLT4	0.092305	-0.083375
[8618]	MMP7	?	MMP9	-0.12951	-0.12951
[8619]	SKAP2	<---	LYN	-0.99957	1.0067
[8620]	PVRL2	--->	MLLT4	0.092305	-0.083375
[8621]	HDAC2	--->	CDYL	0.030032	-0.014369
[8622]	PRKAA1	--->	CFTR	0.32875	-0.37854
[8623]	SMAD1	<---	PSMD1	-0.34025	0.32211
[8624]	ACP5	<---	TNFSF10	-0.080361	0.096651
[8625]	MARK4	--->	YWHAB	0.047165	-0.061733
[8626]	NUMB	<---	PRKCG	-0.55841	0.55403
[8627]	CDK5	<---	SRC	-1.3225	1.3946
[8628]	SSH1	--->	CFL2	0.3315	-0.32562
[8629]	LHX9	<---	SMURF1	-0.42733	0.41928
[8630]	SOCS1	<---	TEC	-0.12165	0.27706
[8631]	LDLR	<---	HSPA5	-0.26416	0.2603
[8632]	SPOCK1	--->	MMP3	0.25303	-0.23217
[8633]	BEGAIN	<---	DEF6	-0.1466	0.1545
[8634]	CD79A	<---	CD19	-0.047792	0.047421
[8635]	CHD3	--->	PUF60	-0.0047536	-0.0054503
[8636]	DLG2	<---	CNKSR2	-0.036197	0.016798
[8637]	SRC	--->	ADRB2	0.5564	-0.49364
[8638]	MOBKL3	--->	DNM1	0.055971	-0.063762
[8639]	BACE1	--->	APP	0.36802	-0.37802
[8640]	YWHAQ	--->	SFRS3	0.092231	-0.070624
[8641]	NFKBIE	<---	NFKB2	-0.019218	-0.0066871
[8642]	TJP1	--->	ARVCF	0.0025158	-0.013369
[8643]	PIAS1	<---	PTPN1	-0.35081	0.35196
[8644]	GP1BA	--->	SELP	0.069805	-0.068365
[8645]	NEO1	--->	NTN1	0.047334	-0.059841
[8646]	DCAF6	<---	TGFBR1	-0.40828	0.3741
[8647]	S1PR2	--->	GNAQ	1.0129	-1.008
[8648]	FGR	--->	YWHAQ	1.0799	-0.92266
[8649]	CTNNB1	<---	NFKB1	-0.054902	0.04228
[8650]	PRKACA	<---	SRC	-1.3225	1.3946
[8651]	APBB1	--->	ENAH	0.013236	-0.024683
[8652]	OSTF1	<---	SMN1	-0.15695	0.15286
[8653]	NLRP1	--->	PYCARD	-0.04958	-0.082219
[8654]	EIF3I	<---	ESR1	-0.0096005	0.034681
[8655]	PRKCA	--->	RALBP1	0.53812	-0.54304
[8656]	GRB7	<---	ERBB3	-0.42553	0.37299
[8657]	NCK2	<---	EGFR	-0.39985	0.38824
[8658]	PTPN11	--->	GAB2	0.99997	-1.0165
[8659]	DUSP4	--->	MAPK8	1.0016	-1.0004
[8660]	CASP9	--->	NAIP	0.0050222	0.0029245
[8661]	SORBS1	--->	ACTA1	0.34287	-0.34738
[8662]	MAP3K7	--->	BCL10	0.22838	-0.224

[8663]	WIPF1	<---	HCK	-0.25671	0.27956
[8664]	LONRF3	<---	SMURF1	-0.69192	0.68798
[8665]	PTPN2	--->	EGFR	0.074053	-0.079237
[8666]	PRKDC	<---	SRF	-0.026612	0.025906
[8667]	PTPN6	--->	SHC1	0.47605	-0.54121
[8668]	TBP	--->	BCL3	-0.0055587	-0.0072521
[8669]	PAK2	<---	SORBS3	-0.11716	0.25826
[8670]	SHC1	<---	BCL3	-0.17393	0.072671
[8671]	FGF19	--->	FGFR4	1.0053	-0.99871
[8672]	PSMD10	--->	RB1	0.25734	-0.26292
[8673]	ERBB4	<---	ERBB3	0.0070923	0.0082662
[8674]	WASF1	<---	CDC42	-0.3163	0.32786
[8675]	YWHAB	--->	SRRM2	0.088655	-0.064614
[8676]	FLNA	<---	FURIN	-0.007489	0.011227
[8677]	TRIP4	--->	TBP	-0.0050538	-0.0072189
[8678]	FBXO30	<---	SMURF1	-0.70778	0.70392
[8679]	MAP3K7	--->	GPR25	0.080988	-0.074123
[8680]	LDLR	<---	APOB	-0.15579	0.15086
[8681]	GTF2I	--->	YY1	0.087739	-0.080566
[8682]	KLF5	<---	NFKB1	-0.47018	0.46956
[8683]	ESR1	<---	FHL2	-0.062299	0.088883
[8684]	PRKCA	--->	TP53	0.39209	-0.39877
[8685]	PPFIA2	<---	PTPRF	-0.017812	0.012075
[8686]	MTA1	<---	SH3GLB1	-0.14901	0.16833
[8687]	SCMH1	--->	MOBKL3	0.40617	-0.37013
[8688]	TRAF2	<---	TNFRSF1B	-0.99681	0.9886
[8689]	YAP1	--->	YWHAG	0.62175	-0.61853
[8690]	RASSF5	<---	HRAS	-0.98684	1.0025
[8691]	FXR2	<---	CCDC33	-0.059218	0.099597
[8692]	STAT1	--->	MAPK14	0.13868	-0.14909
[8693]	SIT1	<---	SYK	-0.82307	0.8319
[8694]	MYOG	?	ID3	-0.65635	-0.65635
[8695]	GPHB5	<---	GPHA2	-0.15578	0.18674
[8696]	LYN	--->	EPOR	0.56402	-0.48964
[8697]	NCK2	<---	PDGFRB	-0.038895	0.003963
[8698]	SHC1	<---	KDR	-0.30166	0.24452
[8699]	SNRK	--->	JUN	1.0022	-1.0148
[8700]	TNIP2	<---	STK11	-0.80985	0.85519
[8701]	PIAS4	<---	IMPDH2	-0.33727	0.33557
[8702]	JAK2	--->	STAT5B	0.99488	-1.0064
[8703]	TLR10	--->	LUC7L3	0.19384	-0.18081
[8704]	UBE2I	--->	TCF3	0.413	-0.41563
[8705]	TNFRSF10B	--->	CFLAR	0.043232	-0.10402
[8706]	LASP1	--->	ZYX	0.053659	-0.048067
[8707]	NT5E	--->	ACTB	0.58605	-0.58334
[8708]	ENO2	<---	HK1	0.0018238	0.0018389
[8709]	ALG9	--->	H2AFX	0.24405	-0.19271
[8710]	CASP3	--->	BCAR1	0.10829	-0.13063
[8711]	TOLLIP	<---	IRAK1	-0.99511	0.99759
[8712]	ASPM	<---	PLK1	-0.30272	0.28296
[8713]	SCN4A	<---	DLG1	-0.049447	0.047736
[8714]	DNMT3A	<---	EZH2	-0.059476	0.095626
[8715]	ASH2L	<---	SMURF1	-0.7089	0.70504
[8716]	PRKCD	--->	FYN	0.34363	-0.3255
[8717]	TGFA	--->	ERBB2	0.19086	-0.19678
[8718]	HSPA8	--->	RB1	0.66404	-0.6637
[8719]	CD2	?	CD4	-0.016784	-0.016784
[8720]	NTRK1	<---	NEDD4L	-0.19332	0.18386
[8721]	TBXA2R	--->	RAB11A	0.066588	-0.039125
[8722]	PTPN6	--->	IFNAR1	1.0145	-1.0055
[8723]	RND3	--->	ROCK1	0.99991	-1.0003
[8724]	SYT1	--->	CALM1	0.40214	-0.35837
[8725]	CASP3	--->	MEF2A	0.17117	-0.20473
[8726]	NTRK3	--->	NGFR	0.056379	-0.023889
[8727]	SPARC	--->	PDGFA	0.015478	-2.0483e-005
[8728]	TCF4	<---	CALM3	-0.33491	0.33066
[8729]	CORO2B	<---	VCL	-0.63862	0.66121
[8730]	PCGF6	--->	E2F6	0.047847	-0.015216
[8731]	CCRL1	--->	CCL13	0.49595	-0.46475
[8732]	MAPK1	--->	CALCOCO1	0.74955	-0.76834
[8733]	LYN	--->	CD22	0.9934	-1.0055
[8734]	SPHK2	<---	LYN	-0.90413	1.0649
[8735]	CREB5	--->	JUN	-0.074402	-0.097881
[8736]	LPP	<---	SHPRH	-0.053514	0.057955
[8737]	PARD6A	<---	YWHAH	-0.090102	0.086336
[8738]	PFN1	--->	ACTA1	0.99385	-1.0067

```
[8739]  SALL2    <---    ZMIZ2   -0.30711        0.30755
[8740]  TJP1     <---    GJA8    -0.012642       0.011892
[8741]  AHR      <---    HSP90AA1        -0.086917       0.076893
[8742]  SMAD3    <---    NR3C1   -0.17818        0.22689
[8743]  CHRDL2   --->    BMP5    0.71403 -0.67452
[8744]  BIN1     --->    DNM2    0.071535        -0.080916
[8745]  ESR1     <---    ARHGEF15        -0.068206       0.094715
[8746]  TRAF2    --->    ZBTB43  0.1053  -0.11336
[8747]  HDAC4    --->    BCL6    0.3836  -0.39654
[8748]  NCOA1    <---    THRA    -0.043163       0.034596
[8749]  NPPA     <---    UBQLN4  -0.054793       0.088881
[8750]  PKP1     --->    DSC1    0.61098 -0.61498
[8751]  MRAS     --->    PIK3CA  0.98913 -1.0082
[8752]  NUCB1    <---    GNAO1   -0.49311        0.51194
[8753]  PDX1     --->    CREB1   0.30055 -0.289
[8754]  PRKACA   --->    ITPR1   0.3288  -0.33886
[8755]  CTSE     --->    INS     0.35706 -0.34709
[8756]  NRXN1    --->    PDZD2   0.058097        -0.039252
[8757]  ATP7A    <---    PDZD11  -0.042181       0.059517
[8758]  TLN1     <---    ITGB5   -0.049547       0.036854
[8759]  CD7      --->    PIK3R1  0.14986 -0.25804
[8760]  SOCS1    <---    TEK     -0.014076       -0.0030111
[8761]  SNAPC4   --->    TBP     -0.00057729     -0.011674
[8762]  NFKBIA   <---    SKP1    -0.56996        0.5592
[8763]  CNTNAP2 <---     CASK    -0.13483        0.11974
[8764]  ZFYVE9   <---    TGFBR1  -0.028474       0.011797
[8765]  YWHAZ    <---    HSPA1B  -0.739  0.77111
[8766]  CAPN1    --->    IL1A    0.13209 -0.13754
[8767]  CAPN1    --->    SPTAN1  0.062177        -0.07387
[8768]  MZF1     <---    FHL3    -0.20808        0.20561
[8769]  FHL3     <--->   FHL2    0.029854        0.029854
[8770]  FRS2     <---    INSR    -0.35555        0.35356
[8771]  APP      <---    TGFB1   -0.13205        0.11483
[8772]  SETDB1   <---    BARD1   -0.09373        0.098896
[8773]  HSPA5    --->    DDX24   0.50862 -0.48833
[8774]  PTCH2    <---    SMO     -0.58585        0.58322
[8775]  PFN1     --->    TLE1    0.072385        -0.04081
[8776]  MYH9     --->    MYL9    0.018656        -0.028322
[8777]  PDP1     --->    PDHA1   0.52403 -0.50713
[8778]  HNRNPK   <---    GRAP2   -0.33569        0.3487
[8779]  SMAD2    <---    KHDRBS1 -0.087265       0.10909
[8780]  SYNCRIP <---     SYT4    -0.039791       0.077746
[8781]  ITLN1    --->    LTF     0.017209        0.01674
[8782]  PARD3    <---    CDC42   -0.099762       0.10443
[8783]  SPTBN1   <---    EPB41   -0.045272       0.036087
[8784]  FAM46A   <---    PSG3    -0.25274        0.24886
[8785]  SLC9A3R1         --->    SLC9A3  0.065872        -0.059787
[8786]  BMP7     --->    ACVR2A  1.0057  -0.99943
[8787]  MAP3K7   --->    MAP2K6  0.99376 -0.98749
[8788]  GRIN2D   <---    TJP1    -0.034965       0.026321
[8789]  NR3C1    <---    CALR    -0.0055003      0.022801
[8790]  TOP2A    <---    SRPK1   -0.54823        0.52133
[8791]  IRAK4    --->    PELI3   0.48758 -0.44131
[8792]  BUB3     <---    BUB1    -0.86422        0.83716
[8793]  TRAF2    --->    MAST2   0.60429 -0.60002
[8794]  MYB      --->    PAX5    0.0248  -0.033869
[8795]  KDR      --->    GRB2    0.86659 -0.66055
[8796]  SVIL     --->    MYH9    0.0053955       -0.013468
[8797]  HEY2     <---    NCOR1   -0.094657       0.085742
[8798]  AR       <---    CDC25B  -0.57745        0.57222
[8799]  ADRB1    --->    GRB2    0.05645 -0.062033
[8800]  CCDC85B --->     ZNF417  0.22518 -0.22906
[8801]  TGFBR3   --->    INHBA   0.18653 -0.16345
[8802]  BRD2     --->    E2F2    0.093965        -0.062638
[8803]  PORCN    --->    WNT5B   1.0005  -0.9854
[8804]  NCK1     --->    RRAS    0.40902 -0.38442
[8805]  FADD     --->    DDX24   -0.00095819     -0.056042
[8806]  TRAF6    <---    PRKCZ   -0.094801       0.080934
[8807]  CD209    ?       CLEC4M  -0.034109       -0.034109
[8808]  HDAC3    <---    MAP3K7IP2       -0.079101       0.084692
[8809]  RGS2     <---    PRKCA   -0.87353        0.87438
[8810]  PCDHA4   <---    IGSF21  -0.27549        0.27168
[8811]  PRDM1    --->    TLE1    0.0034961       -0.0068753
[8812]  HAND2    <---    PRKACA  -1.5271 1.5563
[8813]  IRS2     <---    TYK2    -0.253  0.24109
[8814]  LRP1B    --->    APP     0.015878        -0.029382
```

[8815]	OCLN	--->	GJB1	0.051936	-0.034486
[8816]	KLK2	--->	SERPINB6	0.11833	-0.082185
[8817]	CALCOCO2	--->	ZNF408	0.12169	-0.084439
[8818]	CXCR2	<--->	CXCR1	0.49511	0.49511
[8819]	ARF1	<---	ARHGAP21	-0.1095	0.10443
[8820]	GRAP2	--->	YWHAE	0.26086	-0.25405
[8821]	DDR1	--->	COL2A1	0.026834	-0.040546
[8822]	TP63	--->	TP53	-0.020978	-0.14021
[8823]	DVL2	<---	MCRS1	-0.38498	0.38481
[8824]	CDK2	--->	FOXM1	0.85888	-0.87871
[8825]	CDKN1A	<---	MAP3K5	0.049001	0.19292
[8826]	BLNK	--->	CD72	0.33424	-0.41274
[8827]	PTPRJ	--->	PDGFRB	0.076677	-0.086055
[8828]	RHOXF2	--->	DVL2	0.37141	-0.37157
[8829]	PTK2B	<---	FLT1	-0.40503	0.36159
[8830]	ETS2	<---	NCOR1	-0.078881	0.071387
[8831]	ABCG8	?	ABCG5	-0.062455	-0.062455
[8832]	FZD9	<---	WNT1	-1.0014	0.99836
[8833]	SMAD1	<---	SMAD4	-0.9962	1.0013
[8834]	IGFBP7	--->	IGF2	0.017892	-0.015418
[8835]	SMAD7	--->	SOX5	0.10836	-0.067676
[8836]	SLC9A2	<---	RASA1	-0.096067	0.053975
[8837]	SRC	--->	STAT3	0.9865	-0.9998
[8838]	CD93	<---	COL4A1	-0.03102	0.018746
[8839]	PTPRA	--->	CALM1	0.95254	-0.9517
[8840]	MARK3	<---	PRKCQ	0.0057333	0.022752
[8841]	DRD5	--->	NCS1	0.78966	-0.75527
[8842]	SRC	--->	CD59	1.0294	-0.87521
[8843]	RGL2	<---	MYST2	-0.15549	0.1483
[8844]	NCAN	<---	NCAM1	-0.01798	0.0053826
[8845]	ARHGEF18	--->	GNB1	0.26679	-0.25652
[8846]	LRP12	<---	MYOT	-0.11496	0.14585
[8847]	DVL3	<---	PPP2CA	-0.11058	0.098946
[8848]	AHR	<---	EP300	-0.1265	0.11845
[8849]	PITPNM2	<---	CPNE1	-0.032928	0.073246
[8850]	EED	--->	ITGAE	0.058224	-0.070399
[8851]	LTBP3	--->	TGFB3	0.66401	-0.63698
[8852]	FZD4	<---	DLG2	-0.19553	0.15497
[8853]	FBN1	?	FBN2	-0.0090854	-0.0090854
[8854]	MYO5B	--->	SMAD3	0.24687	-0.2021
[8855]	CREBBP	--->	MECOM	0.048114	-0.058293
[8856]	CAPNS1	--->	GNB2	0.13375	-0.13675
[8857]	CAMK4	--->	HDAC4	0.68838	-0.71414
[8858]	SOS1	<---	ZAP70	-0.10445	0.15874
[8859]	MAPK14	--->	JUNB	0.55135	-0.62628
[8860]	SHC1	<---	SP1	-0.097365	0.050311
[8861]	DEF6	<---	CDC42	-0.26331	0.26775
[8862]	YWHAG	--->	SFRS13A	0.092231	-0.070624
[8863]	YWHAG	--->	USP37	0.079258	-0.055259
[8864]	ZNF24	<---	SETDB1	-0.017375	0.013174
[8865]	CDK13	--->	PRPF40A	0.23542	-0.25641
[8866]	PVRL3	--->	PVR	0.00037536	-0.021316
[8867]	GRIN1	<---	PPP2R2A	-0.23678	0.25102
[8868]	FGFR2	<---	FGF23	-0.53789	0.54173
[8869]	CDK5	--->	TP53	1.0002	-0.99962
[8870]	CCNT1	--->	NCOA2	0.022194	-0.029697
[8871]	RIN3	<---	BIN1	-0.076012	0.079408
[8872]	PTPN6	--->	CUZD1	0.8651	-0.67472
[8873]	UCHL1	<---	HSP90AA1	-0.055488	0.047545
[8874]	ATF6	<---	GTF2I	-0.28662	0.35119
[8875]	STAT5B	<---	PPP2CA	-0.1082	0.095005
[8876]	ELK1	<---	CEBPB	-0.1529	0.20213
[8877]	TRIB3	<---	AKT2	-1.0071	1.0168
[8878]	TNFAIP6	--->	THBS1	0.39635	-0.30152
[8879]	UBE2D3	--->	TRIM39	0.085435	-0.089161
[8880]	TBP	<---	POU2F1	-0.0086868	-0.0036228
[8881]	DGKZ	--->	RBL1	0.096262	-0.1086
[8882]	PAK1	<---	BMX	-0.44839	0.43965
[8883]	PPIL2	<---	HSP90AA1	-0.062795	0.055103
[8884]	NOS1	<---	BDKRB2	-0.20891	0.19753
[8885]	EPHA4	--->	FGFR1	0.027285	-0.097243
[8886]	FOXL1	<---	BMPR2	-0.32274	0.29589
[8887]	SRC	--->	PRKCZ	0.0965	-0.093142
[8888]	DOK1	<---	SH2D1A	-0.1722	0.16699
[8889]	DUSP4	--->	MAPK14	0.99169	-0.98663
[8890]	PECAM1	<---	LCK	-0.44297	0.51622

[8891]	CCNK	<---	NECAB2	-0.010568	0.027549
[8892]	CDK1	--->	APLP2	0.17087	-0.18588
[8893]	RHOA	--->	TGFBR1	0.010625	-0.037483
[8894]	SIRT2	<---	KAT2B	-0.12902	0.12972
[8895]	KCNMA1	<---	HMOX2	-0.0069646	0.040937
[8896]	EPB41L1	<---	DRD3	-0.19189	0.19515
[8897]	MMP9	<---	PZP	-0.11161	0.132
[8898]	PTP4A2	--->	ATF7	0.40886	-0.41153
[8899]	NFKBIA	<---	CUL1	-0.57036	0.56042
[8900]	TJP1	<---	CTNNA1	-0.2343	0.23706
[8901]	VCAM1	--->	EZR	0.084798	-0.095532
[8902]	FXR2	--->	NECAB2	0.31957	-0.28212
[8903]	DUSP3	--->	PDGFA	0.39508	-0.37675
[8904]	RARA	--->	KLF5	0.092475	-0.080305
[8905]	ITGA3	<---	FN1	-0.0090949	-0.001635
[8906]	HNRNPK	--->	CEBPB	0.10568	-0.055891
[8907]	UBE2I	--->	FMR1	0.24253	-0.20893
[8908]	DUSP1	--->	SKP2	0.49464	-0.4783
[8909]	SMAD4	<---	JUND	-0.035159	0.057717
[8910]	HEYL	<---	HIPK3	-0.95979	0.97225
[8911]	KRT15	--->	CALCOCO1	0.0096969	0.0066793
[8912]	PRKCB	--->	PTPN11	0.6319	-0.62682
[8913]	PRTN3	<---	IL1B	-0.095903	0.11066
[8914]	COL4A6	--->	DCN	0.00097089	-0.0049704
[8915]	ADAM12	<---	EGF	-0.048799	0.036179
[8916]	ERBB4	--->	DLG3	0.18045	-0.19014
[8917]	RGL4	<---	HRAS	-0.76787	0.79809
[8918]	CDK9	--->	GRN	0.70069	-0.72663
[8919]	TRIM39	<---	UBE2D4	-0.089161	0.085435
[8920]	SH3BP1	--->	SYNJ1	0.040462	-0.026074
[8921]	HIPK1	--->	HOXD4	0.79979	-0.82765
[8922]	PIK3CG	<---	LYN	-0.11673	0.15115
[8923]	GADD45B	--->	CCNB1	0.58381	-0.57034
[8924]	PTPN1	--->	PDGFRB	0.11574	-0.12276
[8925]	PTPRE	--->	FYN	-0.065476	-0.14195
[8926]	CORO1A	--->	THBS1	0.37965	-0.28499
[8927]	EPHB3	--->	MLLT4	0.095031	-0.086065
[8928]	HIST2H4A	<---	TBL1XR1	-0.034106	0.043907
[8929]	SKP2	--->	E2F1	0.097758	-0.084136
[8930]	CAV1	<---	S1PR1	-0.11858	0.12737
[8931]	AKTIP	<---	PDPK1	-0.20451	0.35015
[8932]	CSNK2A1	--->	SRF	0.37272	-0.3873
[8933]	MAP4K1	--->	DAPP1	0.22433	-0.22977
[8934]	ACVR1	--->	VDR	0.15721	-0.17116
[8935]	BAK1	<---	ERN1	-0.78594	0.75438
[8936]	MAPK11	--->	MAPKAPK5	0.73174	-0.53091
[8937]	ARNT	<---	AHR	-0.061404	-0.060412
[8938]	CPNE2	<---	PPP5C	-0.043387	0.048759
[8939]	YWHAG	--->	ABLIM1	0.024208	-0.014257
[8940]	TNFRSF10D	<---	TNFSF10	-0.42882	0.44704
[8941]	CDK1	--->	MEF2C	0.43947	-0.44112
[8942]	HDAC1	--->	RBL2	0.17541	-0.18586
[8943]	ERBB4	<---	BTC	-0.99014	0.98479
[8944]	CSNK2A1	--->	CDC25B	0.69298	-0.67632
[8945]	HIPK2	--->	NKX3-1	0.79979	-0.82765
[8946]	CSNK2A1	<---	RRAD	-1.0074	1.0132
[8947]	SERPINA5	<---	PLAT	-0.032776	0.037242
[8948]	LCP2	<---	HCK	-0.99119	1.2119
[8949]	NKIRAS2	--->	NFKBIB	0.98515	-0.95568
[8950]	CHEK1	--->	SMAD4	1.012	-1.0074
[8951]	RIT2	--->	POU4F1	0.17195	-0.16996
[8952]	AKT2	<---	HSP90AA1	-0.99454	0.99649
[8953]	RELA	<---	FUS	-0.068991	0.066713
[8954]	ARHGEF2	<---	RRAS2	-0.14455	0.15203
[8955]	SUPV3L1	<---	ADAP1	0.013472	0.020562
[8956]	PORCN	--->	WNT1	1.0005	-0.9854
[8957]	JUNB	<---	FOSL1	-0.31682	0.050278
[8958]	ELK1	--->	ID3	0.12676	-0.084944
[8959]	HPN	<---	F7	-0.0064733	-0.0028364
[8960]	F2	<---	F2RL1	-0.27153	0.28486
[8961]	CTBP1	<---	KAT2B	-0.034272	0.022057
[8962]	CD84	--->	SH2D1A	0.077004	-0.051869
[8963]	GGCX	--->	F2	0.11438	-0.09847
[8964]	ZFYVE20	<---	RAB2A	-0.3573	0.3878
[8965]	BAD	<---	YWHAQ	-1.0088	0.9978
[8966]	GGA1	<---	SORT1	0.0083229	0.025913

[8967]	IL23A	--->	IL12RB1	0.37936	-0.34799
[8968]	FURIN	--->	DSG3	0.030103	-0.033247
[8969]	KPNA1	<---	STAT1	-0.28474	0.16081
[8970]	SPARC	<---	HSPG2	-0.044437	0.03259
[8971]	STAT5A	<---	PPIB	-0.48656	0.46957
[8972]	SMARCE1	--->	NCOA3	0.1296	-0.14219
[8973]	SMAD3	--->	E2F4	0.19366	-0.13771
[8974]	RABGEF1	--->	GGA2	0.001291	-0.011829
[8975]	SRC	--->	ATP2B4	0.15418	-0.15631
[8976]	CNOT1	--->	THRB	0.010054	0.00055002
[8977]	TNFRSF4	--->	TRAF4	0.24665	-0.24298
[8978]	PICK1	<---	PVRL2	-0.12596	0.11758
[8979]	SNTA1	--->	NOS1	0.097325	-0.10797
[8980]	ASCL2	<---	CALM1	-0.22666	0.2676
[8981]	CPNE4	--->	SPTBN1	0.14044	-0.14044
[8982]	PTPRE	--->	MAPK1	1.0061	-1.009
[8983]	MMP9	<---	HAPLN1	-0.093464	0.097177
[8984]	HOXB6	<---	KRT15	-0.042127	0.056278
[8985]	DTX1	<---	NOTCH1	-0.26293	0.2537
[8986]	NR1D2	<---	NCOR1	-0.065644	0.063164
[8987]	CDH1	<---	ITGB7	-0.045865	0.038779
[8988]	CACNB1	--->	CACNA1A	0.16653	-0.17372
[8989]	PRKCG	--->	IRS1	0.9708	-0.84897
[8990]	SSRP1	--->	CHD1	0.02048	-0.028041
[8991]	STAT1	--->	BRCA1	0.086807	-0.16699
[8992]	SRPK1	--->	SFRS2IP	0.29432	-0.31766
[8993]	CASP7	<---	CFLAR	-0.9907	0.99569
[8994]	SLIT1	--->	ROBO1	-0.0017935	-0.0095225
[8995]	MAPK3	--->	TAL1	0.95173	-0.93955
[8996]	CLSTN1	<---	APBB1	-0.082541	0.076167
[8997]	SRC	--->	MET	0.7585	-0.17507
[8998]	ITGB3	<---	PDGFRB	-0.017752	0.0055888
[8999]	DDR1	--->	COL5A2	-0.0016035	-0.01188
[9000]	SMARCD1	<---	CCDC85B	-0.014602	0.026755
[9001]	SMAD6	--->	HOXC8	0.13335	-0.10211
[9002]	KCNQ5	--->	CALM3	0.32976	-0.29233
[9003]	RAPGEF3	--->	RRAS2	1.0115	-1.0014
[9004]	ZIC1	<---	GLI1	-0.0068888	0.013977
[9005]	SRC	--->	RASA1	0.19211	-0.35582
[9006]	ERBB4	--->	STAT5B	1.0062	-1.0106
[9007]	IL6ST	<---	TYK2	-0.10893	0.092516
[9008]	MAP2K6	<---	TRAF6	-1.0149	0.98609
[9009]	CDC25B	<---	SFN	-0.11975	0.14141
[9010]	PIAS4	--->	AR	0.5819	-0.58792
[9011]	KDR	--->	SHC2	1.0026	-1.0154
[9012]	CDK5	--->	PSEN1	0.67463	-0.70045
[9013]	TGFBR1	--->	ENG	1.0038	-1.008
[9014]	EEF1G	--->	CXCL13	0.051368	-0.016209
[9015]	VWA1	<---	HSPG2	-0.035491	0.022441
[9016]	VASP	<---	WWP2	-0.18788	0.17852
[9017]	PPP1R9B	--->	TIAM1	-0.00020496	-0.46939
[9018]	NOS3	<---	BDNF	-0.16391	0.15123
[9019]	TNFRSF14	--->	TRAF2	0.64735	-0.64504
[9020]	ABL1	<---	SFN	-0.08167	-0.068919
[9021]	APPL1	--->	ADIPOR1	0.0055498	-0.00090415
[9022]	RYR2	<---	RYR1	-0.0069923	-0.006978
[9023]	GFI1B	<---	PDLIM5	-0.099409	0.091835
[9024]	PDGFD	--->	PDGFRB	0.14586	-0.14886
[9025]	HRAS	--->	CDC25C	0.33547	-0.30986
[9026]	TLR3	--->	MYD88	0.22743	0.04141
[9027]	SMARCA4	--->	AHR	0.047171	-0.056922
[9028]	HNF1B	?	HNF1A	-0.11417	-0.11417
[9029]	CREBBP	--->	POU2F3	0.18505	-0.18943
[9030]	TSHR	--->	PIK3R1	0.1441	-0.21276
[9031]	DNAJC1	<---	SERPINA3	-0.00095945	0.0053818
[9032]	WAS	<---	SRC	-0.73342	0.73218
[9033]	BMPR1A	--->	HOXC8	0.085961	-0.10748
[9034]	TGFBR1	<---	TSSK4	0.02614	0.36359
[9035]	PTPRF	--->	LCK	0.20326	-0.21657
[9036]	CASP3	--->	VAV1	0.075929	-0.20093
[9037]	RPS6KA5	--->	RELA	1.0165	-0.99047
[9038]	ESRRB	--->	NCOA3	0.12213	-0.13412
[9039]	PGR	--->	NCOA3	0.12213	-0.13412
[9040]	EIF2AK4	--->	SMAD1	0.75909	-0.80912
[9041]	MTNR1B	--->	HNRNPA0	0.46527	-0.43669
[9042]	PTPN6	<---	LCK	-0.17366	0.056644

9045	[9043]	VAV1	<---	EGFR	-0.53136	0.4261
9046	[9044]	TRPC3	?	TRPC1	-0.010919	-0.010919
9047	[9045]	DVL2	<---	ARRB1	-0.2363	0.23088
9048	[9046]	IL16	<---	PPP1R12A	-0.090946	0.094916
9049	[9047]	CBL	--->	LCK	0.98682	-1.0036
9050	[9048]	SSTR2	--->	SHANK2	0.0020815	-0.0093332
9051	[9049]	CHD1	<---	NCOR1	-0.020164	0.007947
9052	[9050]	TP53	<---	ABL1	-0.057309	-0.0059906
9053	[9051]	ERBB4	--->	SNTB2	0.16309	-0.16695
9054	[9052]	CFL1	--->	ACTA1	0.30201	-0.30587
9055	[9053]	EEF1G	--->	HDAC5	0.037795	-0.030464
9056	[9054]	PTPN11	--->	PIK3R1	0.32212	-0.46461
9057	[9055]	RELA	<---	PIN1	-0.16945	0.17479
9058	[9056]	RNPS1	--->	SFRS6	-0.0092275	-0.015971
9059	[9057]	MATN2	<---	MATN1	-0.011059	-0.01029
9060	[9058]	CTCF	<---	HMGB1	-0.45633	0.46894
9061	[9059]	KIT	--->	STAT1	1.0099	-1.0004
9062	[9060]	HRAS	--->	MLLT4	0.99712	-0.98672
9063	[9061]	NCKIPSD	--->	WASL	0.22676	-0.26759
9064	[9062]	STK25	--->	YWHAZ	0.62776	-0.66368
9065	[9063]	MAP3K7IP1	--->	MAP3K7	0.99989	-0.99428
9066	[9064]	DLG1	--->	MYO6	0.020571	-0.027105
9067	[9065]	MAPK3	--->	GJA1	0.49049	-0.48658
9068	[9066]	YWHAZ	<---	RRAD	-0.30645	0.32694
9069	[9067]	IGSF1	--->	ACVR2A	0.038789	-0.058494
9070	[9068]	PAK2	--->	SH3KBP1	0.33445	-0.057884
9071	[9069]	TNFRSF14	<---	TRAF1	-0.034181	0.029707
9072	[9070]	PTPN21	--->	KIF1C	0.18243	-0.19272
9073	[9071]	SNX17	--->	SELP	0.057897	-0.025964
9074	[9072]	RELA	<---	CEBPD	-0.073817	0.083071
9075	[9073]	LATS1	<---	LIMK1	-0.18938	0.17763
9076	[9074]	JAK3	--->	LCK	0.14024	-0.050999
9077	[9075]	SOS1	--->	BIN1	0.32181	-0.32863
9078	[9076]	USP9X	--->	SMAD2	0.45045	-0.3825
9079	[9077]	ESR1	<---	SOS1	-0.35498	0.34827
9080	[9078]	CNKSR1	<---	MAP3K10	-0.26902	0.25616
9081	[9079]	YES1	--->	RASA1	0.21292	-0.57122
9082	[9080]	PTPN6	--->	KLRB1	0.97265	-0.80298
9083	[9081]	PPARG	<---	FOXO1	-0.043496	0.03253
9084	[9082]	ABCA1	<---	DLG3	-0.016269	0.0051204
9085	[9083]	REPS1	<---	RALB	-0.37232	0.40283
9086	[9084]	PRKDC	<---	HMGB1	-0.071357	0.075255
9087	[9085]	ZNF397	<---	ZNF396	-0.097566	-0.030746
9088	[9086]	CASP8	--->	BCL2	0.62187	-0.71598
9089	[9087]	ITGAV	<---	F2R	-0.066333	0.055122
9090	[9088]	GATA4	<---	ZFPM2	-0.0069687	0.029291
9091	[9089]	IGFBP3	<---	PLG	-0.013006	0.028162
9092	[9090]	SHB	--->	VAV1	0.35276	-0.34046
9093	[9091]	ARNT	--->	HEY1	-0.14881	-0.1616
9094	[9092]	TNIP2	--->	IKBKG	0.23896	-0.23693
9095	[9093]	TWF1	--->	ACTC1	0.49943	-0.49675
9096	[9094]	TNNT1	<--->	CCDC85B	0.054031	0.054031
9097	[9095]	ITGA3	<---	FHL2	-0.014484	0.0042947
9098	[9096]	HDAC2	--->	PPP1R8	0.031455	-0.015853
9099	[9097]	MFNG	--->	NOTCH1	0.98927	-1.0072
9100	[9098]	YWHAG	<---	CRTC3	-0.0028743	0.0062851
9101	[9099]	PDP2	<---	PRKCD	-0.73033	0.74075
9102	[9100]	PEG3	<---	USP7	-0.097459	0.11675
9103	[9101]	CSH1	--->	SMAD9	0.19803	-0.21465
9104	[9102]	SCN5A	<---	DLG3	-0.049447	0.047736
9105	[9103]	GZMB	--->	GRIA3	0.13835	-0.13152
9106	[9104]	FURIN	<---	BACE1	-0.072907	0.075966
9107	[9105]	KIT	--->	SOCS5	0.074236	-0.14213
9108	[9106]	IRS2	<---	SOCS1	-0.13554	0.14262
9109	[9107]	TGFA	--->	ELANE	0.10335	-0.06964
9110	[9108]	ZBTB32	<---	TXNIP	-0.15983	0.15294
9111	[9109]	SPAG9	--->	MYC	0.061703	-0.070187
9112	[9110]	SP1	--->	RB1	0.13521	-0.1479
9113	[9111]	LNX1	--->	NUMB	0.032459	-0.026869
9114	[9112]	GRB10	<---	KDR	-0.42479	0.42333
9115	[9113]	OXTR	--->	ARRB2	0.12815	-0.12587
9116	[9114]	SMAD4	<---	JUN	-0.035159	0.057717
9117	[9115]	BFAR	<---	CASP8	-0.38333	0.29293
9118	[9116]	RAC1	--->	WAS	0.36914	-0.48596
9119	[9117]	SH3RF1	<---	RACGAP1	-0.009087	0.0024237
9120	[9118]	FBLN2	<---	FN1	-0.0037303	0.016534

[9119]	LRP1	<---	SKIL	-0.019979	0.0074629
[9120]	RAB27A	--->	RIMS2	0.016184	-0.0067445
[9121]	IRF2BP1	--->	TGIF1	0.25114	-0.22852
[9122]	MBL2	--->	KRT1	0.0027108	-0.016214
[9123]	PTPN11	--->	IL6ST	0.41197	-0.42337
[9124]	PWP2	<---	TOM1L1	-0.11779	0.15077
[9125]	PTPN11	--->	IFNGR1	0.79817	-0.71727
[9126]	SLK	--->	CLSTN1	0.26874	-0.30174
[9127]	DGKZ	--->	PLCG1	0.28631	-0.31603
[9128]	PHC2	<--->	SCMH1	0.25361	0.25361
[9129]	NCOR2	--->	ESR2	0.13736	-0.14734
[9130]	MMP9	<---	COL4A4	-0.078498	0.072684
[9131]	HNRNPU	--->	SMN1	0.017464	-0.002282
[9132]	NHP2L1	--->	CDKN2C	0.50778	-0.47467
[9133]	RUNX2	<---	UBTF	-0.20909	0.2425
[9134]	ELANE	--->	LPA	0.0012258	-0.0091243
[9135]	PLCB1	<---	SLC9A3R1	-0.5217	0.52612
[9136]	CDK2	--->	UBE2A	0.50887	-0.53523
[9137]	ERBB3	--->	ERBB2	0.0023711	-9.0961e-005
[9138]	GRIN1	<---	TRAF3	-0.022878	0.015919
[9139]	HNRNPK	<---	RBM7	-0.0048144	0.036464
[9140]	FYN	--->	CSF2RB	0.98931	-1.0003
[9141]	TRPC6	--->	ITPR3	-0.0074077	-0.013
[9142]	MLL	--->	WDR5	0.16955	-0.15372
[9143]	CBL	--->	KDR	0.035874	-0.03994
[9144]	H3F3A	<---	SMARCA1	-0.04883	0.049463
[9145]	DHH	--->	PTCH2	0.56021	-0.56975
[9146]	HTR2C	--->	CALM1	0.42043	-0.40552
[9147]	EP300	--->	DTX1	0.029864	-0.041885
[9148]	TANC1	<---	DLG1	-0.083347	0.077256
[9149]	MCF2	<---	RHOA	-0.017594	0.0050659
[9150]	HNRNPK	<---	HCK	-0.97195	1.133
[9151]	ZYG11B	<---	CUL2	-0.027118	0.029208
[9152]	EHD3	?	EHD1	-0.018744	-0.018744
[9153]	TRIM24	--->	AR	1.1345	-1.1301
[9154]	ITGA11	<---	ITGB1	-0.013186	-0.00054527
[9155]	CCNK	<---	CCDC85B	-0.24694	0.25885
[9156]	APEX1	<---	ANP32C	-0.048591	0.087031
[9157]	PHKG1	<--->	PHKG2	0.23429	0.23429
[9158]	PTPRA	--->	KCNA2	0.76285	-0.76407
[9159]	YWHAE	<---	TOP2A	-0.092745	0.1158
[9160]	EGFR	<---	CBL	-1.0135	1.0132
[9161]	OCRL	<---	RAC1	-0.25397	0.25628
[9162]	RAC1	--->	DVL2	0.41768	-0.41752
[9163]	FBXW2	<---	SKP1	-0.03566	0.035778
[9164]	YWHAG	--->	HECTD1	0.20501	-0.20526
[9165]	SRC	--->	KIT	0.98487	-0.98796
[9166]	ZHX2	?	ZHX1	-0.033918	-0.033918
[9167]	BCAR1	--->	TRIP6	0.016543	-0.013863
[9168]	CAMK2G	--->	ATF1	0.80987	-0.78916
[9169]	UBE2R2	<---	BTRC	-0.28531	0.27621
[9170]	CCL20	<---	CCR6	-0.46475	0.49595
[9171]	HNRNPU	<---	EP300	-0.18607	0.18175
[9172]	HDAC2	--->	MBD2	0.091026	-0.074216
[9173]	AREG	<---	UBQLN4	-0.1025	0.10589
[9174]	PLCB1	<---	RGS12	-0.092861	0.084261
[9175]	BCR	--->	ERCC3	0.094879	-0.097211
[9176]	PDLIM5	<---	PRKCG	-0.57553	0.57562
[9177]	ADORA2B	--->	SLC9A3R2	0.21221	-0.19778
[9178]	TNIK	<---	NCK1	-0.0017092	-0.00039255
[9179]	PRKACA	--->	PDE3B	0.53012	-0.53853
[9180]	DLL4	--->	NOTCH4	-0.0069764	-0.011246
[9181]	ADAM15	<---	ARHGEF6	-0.052451	0.042729
[9182]	ESR1	--->	RELA	0.049708	-0.049342
[9183]	PAK4	--->	ITGB5	0.049873	-0.023488
[9184]	KAT5	<---	HDAC1	-0.0059564	0.010113
[9185]	NR1D1	<---	NCOR1	-0.065644	0.063164
[9186]	MAPK14	--->	MEF2C	0.99836	-0.99617
[9187]	TRERF1	<---	EP300	-0.073443	0.063039
[9188]	CAPN1	--->	SPTBN1	0.08188	-0.092264
[9189]	TNK2	<---	ITFG2	-0.019099	0.0058481
[9190]	MAP3K7IP1	--->	BMPR1A	0.18247	-0.2027
[9191]	CDH24	--->	PPP1R16A	0.27708	-0.26227
[9192]	HSPA8	--->	HSP90AA1	0.05218	-0.059042
[9193]	DNMT3B	--->	EED	0.16371	-0.13078
[9194]	GTF3C1	--->	NHP2L1	0.090589	-0.05661

[9195]	TFG	<---	TRIM25	-0.045761	0.038871
[9196]	BCAR1	--->	PXN	0.038155	-0.043184
[9197]	CCND1	--->	RBL1	0.42536	-0.43637
[9198]	CAPNS1	--->	LRRCC1	0.049719	-0.056156
[9199]	CPNE4	<---	MAP2K1	-0.17777	0.59194
[9200]	HIRA	--->	PAX7	0.060724	-0.046115
[9201]	MAPK9	--->	NFATC3	1.0021	-0.99987
[9202]	XRN1	--->	DPP7	0.012945	-0.0087947
[9203]	CCR2	--->	CCL2	0.49595	-0.46475
[9204]	PACSIN3	--->	WASL	0.092082	-0.11591
[9205]	IL8	?	PF4	-0.047195	-0.047195
[9206]	PRELP	--->	FN1	0.1011	-0.090765
[9207]	PRKACA	--->	PLN	1.0045	-0.98691
[9208]	CNTN1	--->	NOTCH1	0.25433	-0.26986
[9209]	FSTL3	--->	INHBA	1.0085	-0.99524
[9210]	PRKCQ	--->	RASGRP3	0.99988	-0.99797
[9211]	PDPK1	--->	YWHAH	0.39388	-0.25176
[9212]	HDAC1	--->	ATRX	0.026068	-0.03729
[9213]	GDF9	<---	CRELD1	-0.24006	0.28516
[9214]	PRKCA	--->	MYOD1	1.0168	-1.0076
[9215]	TCERG1	--->	U2AF2	0.19241	-0.17953
[9216]	GOLGA1	<---	RAB6A	-0.42539	0.45515
[9217]	CDKN1B	<---	UCHL1	-0.037888	0.041957
[9218]	RGS2	--->	WDR74	0.58652	-0.55801
[9219]	SKIV2L2	--->	MPP6	0.028046	-0.029486
[9220]	NOTCH1	<---	MEF2C	-0.10361	0.089085
[9221]	SAMD4B	<---	YWHAG	-0.014652	0.0014835
[9222]	DLG3	--->	GRIK2	0.058844	-0.064781
[9223]	ONECUT1	<---	CREBBP	-0.18943	0.18505
[9224]	PKD1	<---	SRC	-0.59004	0.64957
[9225]	FOSL1	<---	CALCOCO1	-0.35816	0.34658
[9226]	TFDP2	<---	E2F3	-0.064857	-0.0088085
[9227]	ZBED1	--->	GPSM1	0.063341	-0.028365
[9228]	LAMA4	<---	BRD7	-0.029989	0.063556
[9229]	RHOXF2	<---	RADIL	-0.049343	0.054032
[9230]	PRKACA	--->	PPP1R1B	1.0061	-1.0155
[9231]	COL1A1	--->	CD44	0.0088215	-0.021236
[9232]	VEGFA	--->	FLT1	0.41525	-0.42349
[9233]	CSNK2A1	--->	ACACA	0.41949	-0.42896
[9234]	PAK1	--->	BAD	1.001	-0.99724
[9235]	IGFBP7	<---	VEGFA	-0.055565	0.046844
[9236]	GPR77	--->	C3	0.16026	-0.15448
[9237]	HSP90AB1	--->	ARHGAP1	0.028704	-0.036519
[9238]	FOXH1	--->	NKX2-5	0.42236	-0.39346
[9239]	KCNA3	--->	BAX	0.53452	-0.51759
[9240]	STAT3	<---	FLT1	-0.20727	0.17827
[9241]	ZBTB16	<---	HDAC5	-0.066557	0.058293
[9242]	IKBKG	<---	IKBKB	-0.4146	0.27526
[9243]	GNA11	<---	CHRM2	-0.98627	0.9872
[9244]	CPNE2	<---	PDCD6	-0.22356	0.24347
[9245]	SP1	--->	RELA	0.052088	-0.06317
[9246]	HRAS	<---	GRB2	-0.3944	0.44166
[9247]	CAPN1	--->	TINAGL1	0.25276	-0.25251
[9248]	PSD	--->	GMFG	0.048904	-0.014888
[9249]	MMP9	<---	COL4A2	-0.055587	0.046581
[9250]	UBE2I	--->	RANGAP1	0.071581	-0.072403
[9251]	SLAMF7	<---	SH2D1A	-0.071479	0.066041
[9252]	SSTR2	--->	PIK3R1	0.13057	-0.19991
[9253]	KAT5	<---	CDK1	-0.43407	0.41309
[9254]	NLK	--->	STAT3	1.0077	-1.0166
[9255]	CD22	--->	CD19	0.004714	-0.019002
[9256]	SH3GL1	--->	ARHGAP8	0.031042	-0.035967
[9257]	PGAM1	<---	PKP4	-0.061249	0.070245
[9258]	CDC25A	<---	YWHAB	-0.11975	0.14141
[9259]	PARD6A	<---	RHOQ	-0.17886	0.17837
[9260]	PAFAH1B1	<---	MAP1B	-0.040995	0.055856
[9261]	CDK2	--->	CCNH	1.083	-1.1093
[9262]	TCERG1	--->	SETDB1	0.21484	-0.2113
[9263]	EP400	--->	MYC	0.10189	-0.10691
[9264]	ULK1	--->	GABARAP	0.033175	-0.047831
[9265]	RARA	<---	POU2F1	0.012013	0.012582
[9266]	AKT2	--->	CHUK	1.0001	-0.99768
[9267]	CCR1	--->	CCL7	0.49595	-0.46475
[9268]	HRAS	--->	BCL2	0.9966	-0.99111
[9269]	AKT1	--->	BCL2L11	0.18668	-0.14121
[9270]	KIT	--->	INPP5D	0.16033	-0.25706

[9271]	CAMKK2	--->	CAMK4	0.83082	-0.43719
[9272]	AR	<---	NR3C1	-0.58072	-0.0031537
[9273]	PLK2	--->	CIB1	0.46574	-0.47258
[9274]	SMURF2	--->	TGFBR1	0.99923	-0.98798
[9275]	SVIL	--->	LPP	0.071071	-0.012903
[9276]	INADL	--->	GRIN2B	0.11324	-0.11391
[9277]	KCND2	--->	FLNC	0.017799	0.01637
[9278]	GDF6	<---	SMAD5	-0.055306	0.060031
[9279]	PTPN1	--->	CDH2	0.60823	-0.61405
[9280]	VAV3	<---	INSR	-0.15914	0.14
[9281]	ACVR2B	<---	INHBB	-0.99943	1.0057
[9282]	HK1	<---	SCMH1	-0.12494	0.14258
[9283]	MAPK3	<---	ADRBK1	-0.049315	0.28927
[9284]	AR	<---	RB1	-0.19464	0.18375
[9285]	DCAF6	--->	SMAD2	0.46428	-0.38187
[9286]	TLR2	--->	IRAK1	0.090527	-0.068126
[9287]	ELAVL2	?	ELAVL3	-0.016298	-0.016298
[9288]	HDAC3	--->	RELA	0.10974	-0.10396
[9289]	TNFRSF10A	<---	TNFSF10	-0.55375	0.5464
[9290]	BUB1B	--->	CENPE	0.70223	-0.72817
[9291]	MALT1	--->	TRAF2	0.0050262	-0.0087436
[9292]	PRKACA	<---	RAP1A	-1.0074	1.0132
[9293]	CHN1	--->	GTF3C1	0.059551	-0.076438
[9294]	ITGAV	<---	ITGB6	-0.0076372	-0.0058749
[9295]	DLG4	--->	KCNJ10	0.04551	-0.04695
[9296]	EEF1G	<---	C1orf174	-0.034878	0.070639
[9297]	STK35	--->	TGFBR1	0.36359	0.02614
[9298]	FES	--->	IRS2	1.0134	-1.0021
[9299]	FZD4	<---	MAGI3	-0.037554	0.024785
[9300]	STAT5B	?	STAT5A	-0.083032	-0.083032
[9301]	DDX5	<---	ESR1	-0.01017	0.010763
[9302]	ACTR3	--->	CDH1	0.040703	-0.037875
[9303]	PIK3C2B	<---	GRB2	-0.03082	0.025062
[9304]	HOXA10	<---	EP300	-0.11913	0.11102
[9305]	WNT7A	--->	FZD5	0.24992	-0.2481
[9306]	SMAD3	<---	PRTN3	-0.32884	0.41175
[9307]	STRADB	<---	STK11	-0.21779	0.6413
[9308]	ERBB2	--->	JAK2	0.36341	-0.37602
[9309]	AFAP1L2	<---	SRC	-1.0835	1.2771
[9310]	TRAF2	<---	TNFRSF1A	-0.99906	1.001
[9311]	ADCY6	<---	RAF1	-0.13977	0.11884
[9312]	ANGPTL1	<---	TEK	-0.014198	0.00032334
[9313]	DNASE1	--->	ACTA2	0.29006	-0.29329
[9314]	YWHAB	--->	TJP2	0.073422	-0.070569
[9315]	TLX1	<---	PPP2CA	-0.47089	0.4683
[9316]	PIK3R5	<---	PIK3CG	-0.075263	0.080491
[9317]	IL27RA	<---	STAT1	-0.17414	0.092446
[9318]	E2F1	--->	TFDP1	-0.0088482	-0.041319
[9319]	CREB1	<---	CHD3	-0.031771	0.01995
[9320]	NCOA2	<---	DLX5	-0.06802	0.06031
[9321]	AKTIP	<---	AKT1	-0.2796	0.4264
[9322]	SH3RF1	<---	MAP3K12	-0.16706	0.15788
[9323]	UBE2N	--->	RNF128	0.014609	-0.00058435
[9324]	HIST1H2AL	<---	GADD45A	-0.10198	0.15343
[9325]	F9	--->	LRP1	-0.0056796	-0.062493
[9326]	ZNF384	<---	TAF15	-0.13063	0.1232
[9327]	CSNK1D	--->	PSEN2	0.71499	-0.5784
[9328]	OPRD1	--->	GNAO1	1.0129	-1.008
[9329]	KCNQ3	--->	CALM2	0.32976	-0.29233
[9330]	PPFIA1	<---	PTPRS	-0.11777	0.11578
[9331]	CSNK2A1	--->	SPIB	1.1285	-1.1212
[9332]	USP20	<---	PRKCI	-0.2787	0.26512
[9333]	SOX10	<---	CEBPA	-0.057257	0.06251
[9334]	ARNT	--->	HEY2	-0.14881	-0.1616
[9335]	GAB2	<---	SRC	-0.67321	0.66426
[9336]	RERE	--->	EFEMP1	0.071521	-0.050192
[9337]	ADRBK1	--->	OPRD1	0.39714	-0.42229
[9338]	TBC1D1	--->	YWHAG	0.0046156	-0.0029444
[9339]	CXCL13	?	CCL21	-0.047195	-0.047195
[9340]	YWHAE	<---	MAP3K5	0.17403	0.23745
[9341]	SOX8	<---	DLX5	-0.060464	0.063322
[9342]	CDC34	--->	ROCK1	0.0059098	-0.020975
[9343]	SNX4	--->	PDGFRA	0.11348	-0.11276
[9344]	CYB5R1	<---	UBQLN4	-0.09609	0.13014
[9345]	OLIG3	--->	TCF12	-0.057221	-0.20787
[9346]	GATA6	<---	CRIP2	-0.13822	0.18194

[9347]	HCLS1	<---	SH2D4A	-0.28132	0.31595
[9348]	DOK2	<---	EGFR	-0.41169	0.40977
[9349]	SLAMF6	<---	SH2D1A	-0.071479	0.066041
[9350]	PLSCR1	--->	SHC1	0.15479	-0.31638
[9351]	CEBPD	?	CEBPE	-0.11448	-0.11448
[9352]	PTPRF	<---	PTPRA	-0.042173	0.035592
[9353]	DLL1	<---	PSEN1	-1.0003	1.0135
[9354]	KAT5	--->	HIST2H2AC	0.15564	-0.12976
[9355]	STAT1	<---	KDR	-0.1437	0.118
[9356]	CCL13	<---	CCR5	-0.46475	0.49595
[9357]	CDC25A	--->	SMAD3	0.2168	-0.16737
[9358]	TFPI2	--->	PLG	0.014946	0.00022919
[9359]	NGFR	<---	MAPK3	-0.27811	0.23445
[9360]	HDAC2	--->	CDC20	0.11313	-0.098152
[9361]	JAK1	--->	IL4R	1.0004	-0.9993
[9362]	MAPK3	<---	PTPN5	-1.009	1.0061
[9363]	DYNC1I1	<---	DYNLRB1	-0.060711	0.071824
[9364]	SIK3	<---	CRTC2	-0.075794	0.069802
[9365]	BUB3	<---	HDAC1	-0.098152	0.11313
[9366]	JAK2	--->	GRB2	1.0016	-1.0005
[9367]	FN1	<---	ADAMTS4	-0.015045	0.016265
[9368]	ARRB2	<---	AGTR1	-0.25215	0.25543
[9369]	HOXA11	<---	CREBBP	-0.18943	0.18505
[9370]	GRB2	--->	CD247	0.51177	-0.58583
[9371]	NCOR2	--->	ESR1	0.13736	-0.14734
[9372]	SPI1	<---	HOXA10	0.0013001	0.01868
[9373]	NCOA2	<---	ARNT	-0.031473	-0.010013
[9374]	LHCGR	--->	CGB	0.38896	-0.35802
[9375]	RASL12	--->	SMAD3	0.55322	-0.47159
[9376]	MED15	--->	SMAD2	0.18248	-0.16165
[9377]	NCOA1	<---	AHR	-0.035755	-0.022773
[9378]	BMPR1B	?	BMPR1A	-0.088422	-0.088422
[9379]	CASP8	--->	GRIA1	0.19681	-0.22459
[9380]	STK36	--->	GLI2	0.39927	-0.41367
[9381]	CSNK2A1	--->	NCF1	0.21063	-0.24169
[9382]	DUSP14	--->	CD28	0.76428	-0.73483
[9383]	TGM2	--->	SERPINF2	0.12551	-0.089358
[9384]	PDLIM7	<---	GEM	-0.131	0.14841
[9385]	GRIN1	<---	HRAS	-0.31763	0.32333
[9386]	RNF11	<---	TNFAIP3	-0.068997	0.066804
[9387]	EIF4B	<---	EIF4E	-0.073178	0.068275
[9388]	SHC1	<---	RET	-0.51074	0.23042
[9389]	BIRC5	<---	RASA1	-0.29717	0.15342
[9390]	TYK2	<---	FYN	0.081386	0.19726
[9391]	TGFB1	<---	BMP3	-0.98835	0.99583
[9392]	CASK	--->	ATP2B4	0.21676	-0.22708
[9393]	IL1F5	--->	SKIL	0.42063	-0.4166
[9394]	BIRC2	<---	CASP3	-0.16105	0.10574
[9395]	ACTN1	<---	VCL	-0.19198	0.19265
[9396]	PHKG1	--->	MAPT	0.99929	-1.0027
[9397]	LAMB1	--->	GFI1B	0.089252	-0.097152
[9398]	F2RL3	<--->	F2RL2	0.49511	0.49511
[9399]	SVIL	--->	CTTN	0.044409	0.0067907
[9400]	CBX3	<---	TRIM24	-0.030467	0.064029
[9401]	MEGF10	--->	SART3	-0.0026444	-0.010459
[9402]	PICK1	--->	CTNNB1	0.49044	-0.48903
[9403]	ADRA1D	--->	SNTB2	0.343	-0.34434
[9404]	GNA13	<---	PTK2B	-0.064753	-0.047525
[9405]	GRB2	--->	PAK4	0.52464	-0.48008
[9406]	RASL12	--->	SMAD2	0.55322	-0.47159
[9407]	PRKD1	<---	PRKCE	-0.52651	0.55695
[9408]	PACSIN3	<--->	PACSIN1	0.019599	0.019599
[9409]	MTNR1B	<---	HSPA5	-0.36596	0.40505
[9410]	MYH9	?	MYH10	-0.007305	-0.007305
[9411]	PER1	<---	CSNK1E	-0.58384	0.72042
[9412]	USP6NL	--->	ACTA1	0.53418	-0.53136
[9413]	LCK	--->	CD44	0.1979	-0.18991
[9414]	FGFR1	--->	PIK3R2	-0.025995	-0.034864
[9415]	TRAF2	--->	DTNB	0.14258	-0.13688
[9416]	GNA13	--->	MCF2	-0.00027838	-0.012719
[9417]	LRP1B	<---	PLAT	-0.038908	0.027764
[9418]	SHD	--->	ABL1	-0.18596	-0.32072
[9419]	SKIL	<---	SNX17	-0.39611	0.40021
[9420]	NELL2	--->	GFI1B	0.086979	-0.091974
[9421]	COPS6	--->	CUL1	0.02459	-0.017361
[9422]	HNRNPD	--->	PCBP1	0.036464	-0.0048144

[9423]	PRKCD	--->	GSK3A	0.26908	-0.016385
[9424]	HNRNPK	--->	VAV1	0.034383	-0.22359
[9425]	CASP3	--->	CDK11B	0.87492	-0.98085
[9426]	CCNE1	--->	RBL1	0.42536	-0.43637
[9427]	GNA12	<---	BTK	-0.68342	0.63988
[9428]	PDGFB	<---	HSPG2	-0.017166	0.0046555
[9429]	ITGA10	--->	ITGB1	-0.0043081	-0.0094516
[9430]	ABL2	--->	BCR	0.0049372	-0.030184
[9431]	LRP2	<---	APOB	-0.41586	0.41488
[9432]	BAG3	<---	HSPA1A	-0.3826	0.39751
[9433]	ABL1	<---	TRAF6	-0.10648	0.062539
[9434]	MAPK14	--->	ZFYVE20	0.29074	-0.46077
[9435]	CASP8	--->	PSEN2	0.56121	-0.70272
[9436]	SMAD3	<---	SMURF1	-1.0067	0.9934
[9437]	IRS1	<---	GRB2	-0.35997	0.37527
[9438]	GHRL	--->	GHSR	1.0107	-0.98958
[9439]	FBLN1	<---	HBEGF	-0.078894	0.093897
[9440]	FGFR3	<---	FGF18	-0.61087	0.60861
[9441]	BATF3	<---	CEBPE	-0.18303	0.26178
[9442]	PRKACA	<---	RAP1GAP	-0.21348	0.19914
[9443]	TCF4	<---	MYOD1	-0.37599	-0.054725
[9444]	JAM3	<---	F2RL2	-0.0099791	0.00028002
[9445]	TIFAB	?	TIFA	-0.00097565	-0.00097565
[9446]	JAK1	<---	IGF1R	-0.090153	0.092435
[9447]	STAT3	--->	HIF1A	1.0038	-1.0012
[9448]	ACTN4	<---	ADORA2A	-0.08121	0.077572
[9449]	RGL2	<---	RAP1B	-0.61434	0.59497
[9450]	DHH	--->	PTCH1	0.99953	-1.0096
[9451]	PRKAR2A	--->	SMAD3	0.54864	-0.46649
[9452]	BMX	--->	TNFRSF1B	0.19052	-0.25762
[9453]	SMAD4	<---	TRAP1	-0.33191	0.41481
[9454]	COPS5	--->	NCOA1	0.10501	-0.1135
[9455]	RPS6KA5	--->	CREB1	1.0082	-0.98976
[9456]	COL4A1	--->	APP	0.024788	-0.038387
[9457]	GRHL3	?	GRHL2	-0.13135	-0.13135
[9458]	SETDB1	<---	PABPC4	-0.23125	0.24574
[9459]	CHEK2	--->	MDM2	0.040027	-0.058926
[9460]	RASGRF1	<---	TNK2	-0.0089364	-0.0049306
[9461]	PIK3CB	<---	HCK	-0.34581	0.3613
[9462]	SNTB1	<---	DMD	-0.006502	-0.0028739
[9463]	GRB10	<---	RET	-0.2946	0.087937
[9464]	TCF3	--->	CTNNB1	0.097446	-0.10797
[9465]	TJP1	<---	GJA3	-0.012642	0.011892
[9466]	EIF4G1	--->	PABPC1	0.16238	-0.13175
[9467]	DOK4	<---	FYN	-0.93215	1.0898
[9468]	ESR1	<---	NFKB1	-0.47154	0.47113
[9469]	FRS3	<---	PRKCI	-0.37242	0.37492
[9470]	GRIN2B	--->	PLCG1	0.12702	-0.15333
[9471]	PIM1	--->	SNX6	0.76386	-0.78894
[9472]	EGFR	--->	SEC13	0.30491	-0.30607
[9473]	MOBKL3	--->	EPS15	0.067034	-0.077393
[9474]	PRPF40A	<---	FASLG	-0.063247	0.099539
[9475]	MST1R	<---	YES1	-0.0064537	0.77229
[9476]	PDGFRB	<---	SRC	-0.028637	0.0065541
[9477]	ADH5	--->	CHD3	-0.0042166	-0.0057929
[9478]	CCDC33	--->	NECAB2	0.40214	-0.35837
[9479]	VDR	--->	RXRG	1.003	-0.99549
[9480]	SPTAN1	--->	SPTBN1	-0.0094908	-0.03238
[9481]	HIPK3	--->	TGFB1I1	0.29365	-0.34731
[9482]	HSPA2	--->	MEOX2	0.94315	-0.90298
[9483]	LIN7A	<---	DLG3	-0.012492	-0.0023615
[9484]	CD2AP	<---	HCK	-0.44444	0.74787
[9485]	HSPA8	--->	BAG2	0.96516	-0.92976
[9486]	TRAF4	<---	NGFR	-0.55378	0.59613
[9487]	CD40	<---	JAK3	-0.2423	0.24563
[9488]	BDKRB2	<--->	AGTR1	0.17598	0.17598
[9489]	RASSF1	<---	TNFRSF1A	0.011695	0.095942
[9490]	SMAD2	--->	ZNF41	0.12829	-0.12271
[9491]	BMPR2	<---	PRKCB	0.26042	0.31069
[9492]	FYN	--->	CD79A	1.0799	-1.0399
[9493]	STRAP	--->	SMAD6	0.46428	-0.38187
[9494]	UBXN1	--->	SMAD1	0.33166	-0.35027
[9495]	WWOX	--->	EZR	0.096155	-0.099356
[9496]	HNRNPM	<---	LMO3	-0.021109	0.054056
[9497]	SH3D19	--->	SH3YL1	0.085123	0.01669
[9498]	MAPK13	--->	ATF2	1.0147	-1.0156

[9499]	RNF216	<---	UBE2L3	-0.15877	0.19238
[9500]	DHX29	--->	TBC1D17	0.008027	0.0073952
[9501]	LFNG	--->	NOTCH2	0.99253	-0.99707
[9502]	CEBPD	?	CEBPA	-0.11448	-0.11448
[9503]	ARHGEF1	<---	CNKSR1	-0.077189	0.068791
[9504]	LMO7	<---	UBAC2	-0.011325	0.0044424
[9505]	GDF9	--->	BMPR2	0.21823	-0.24164
[9506]	FLNA	<---	PSEN1	-0.033635	0.067884
[9507]	CRYZ	--->	CRYAA	0.12131	-0.073529
[9508]	AURKB	--->	CENPA	1.0832	-1.0289
[9509]	FN1	--->	SKIL	0.39778	-0.39489
[9510]	EML3	<---	YWHAG	-0.19355	0.2164
[9511]	MYOD1	--->	TCF3	-0.13552	-0.39218
[9512]	YWHAG	<---	ARHGEF6	-0.079984	0.07062
[9513]	GRM5	--->	RGS12	0.1187	-0.11565
[9514]	PLK1	--->	RECQL5	0.1667	-0.18431
[9515]	DDR1	--->	COL3A1	-0.0016035	-0.01188
[9516]	TLR4	--->	MYD88	0.2166	0.0038095
[9517]	SERPINA5	<---	F2	-0.040728	0.057207
[9518]	NCOA1	--->	FOS	0.018891	-0.049054
[9519]	PIK3R1	<---	EGFR	-0.29745	0.2254
[9520]	ATXN1	<---	PQBP1	-0.10329	0.099551
[9521]	TRIP4	<---	NFKB1	-0.77395	0.78199
[9522]	ZEB2	--->	SMAD5	-0.0031961	-0.015716
[9523]	MYOG	<---	CALM2	-0.22666	0.2676
[9524]	EPHA2	<---	CBL	-0.30479	0.29257
[9525]	RANBP2	<---	OPN1MW	-0.12658	0.13007
[9526]	PRKCA	--->	OCLN	0.94019	-0.93859
[9527]	DMTF1	--->	CCND1	0.64939	-0.63597
[9528]	PTPN6	--->	CD247	0.98974	-1.0093
[9529]	PLCG1	<---	FYN	-1.0097	0.81503
[9530]	TANC1	<---	DLG4	-0.083347	0.077256
[9531]	ATXN1	<---	TBC1D5	-0.035193	0.03187
[9532]	CRKL	--->	RAPGEF1	0.99873	-0.99825
[9533]	DGKZ	<---	HRAS	-0.3271	0.33412
[9534]	ZEB2	<---	CTBP2	-0.1174	0.10744
[9535]	RET	--->	MAPK8	0.20607	-0.24517
[9536]	ABL1	<---	YWHAB	-0.08167	-0.068919
[9537]	USP7	<---	TRAF5	-0.0059313	0.056497
[9538]	PPIA	--->	RB1	0.47448	-0.47551
[9539]	DDX21	--->	JUN	0.27835	-0.31119
[9540]	EP300	--->	MDM2	0.031284	-0.043084
[9541]	BCOR	<---	CPNE2	-0.37401	0.39237
[9542]	PDLIM5	--->	ACTN1	0.0082818	-0.010186
[9543]	PXN	--->	TLN1	0.098361	-0.10833
[9544]	KLK13	--->	SERPINF2	0.11833	-0.082185
[9545]	ACTN1	--->	GFI1B	0.050848	-0.061027
[9546]	YWHAG	<--->	YWHAQ	0.069023	0.069023
[9547]	HSPG2	--->	ITGA2	0.0075402	-0.021131
[9548]	ITGA5	--->	FGFR2	-0.0044394	-0.008527
[9549]	CXCR4	<---	PTPN11	-0.34558	0.53404
[9550]	CDC34	--->	NFKBIA	1.0081	-1.011
[9551]	MCAM	<---	FYN	-0.27678	0.3537
[9552]	CSNK2A1	--->	MYB	0.092606	-0.10814
[9553]	UBE2D1	--->	RNF126	0.18537	-0.17305
[9554]	NOS3	<---	AGTR1	-0.34063	0.3307
[9555]	LCK	--->	DAPP1	1.2932	-0.84987
[9556]	CDH7	?	CDH12	-0.058742	-0.058742
[9557]	TLE1	<---	PCDHA4	-0.075188	0.10813
[9558]	PKD1	--->	JUP	0.018206	-0.024536
[9559]	TICAM2	<---	TLR4	-0.99954	0.98673
[9560]	CTSG	<---	VCAM1	-0.20793	0.20333
[9561]	PIAS1	--->	SNIP1	0.052889	-0.051099
[9562]	PRF1	--->	GZMB	0.067098	-0.026776
[9563]	C1S	?	C1R	-0.013338	-0.013338
[9564]	TBP	<---	PIAS1	-0.012093	-0.0010036
[9565]	SPRY1	<---	GRB2	-0.32592	0.33891
[9566]	TLE1	<---	HNF1A	-0.021516	0.05243
[9567]	CACNB1	<---	ATN1	-0.043333	0.041147
[9568]	JUN	--->	STAT3	0.26847	-0.31444
[9569]	CREB1	<---	RAB1A	-0.39733	0.40853
[9570]	UBQLN1	<---	MTOR	-0.1634	0.16119
[9571]	GRM5	--->	CALM1	0.31782	-0.27991
[9572]	MOBKL3	<---	CCDC85B	-0.20643	0.23334
[9573]	FYN	--->	DOK1	1.3209	-1.1853
[9574]	FZD7	<---	UBQLN1	-0.33534	0.3099

[9575]	RRH	<---	TGFBR1	-0.1852	0.15862
[9576]	CDH12	?	CDH18	-0.058742	-0.058742
[9577]	EPS8	<---	ITGB3	-0.038477	0.0090569
[9578]	CD93	<---	COL1A2	-0.043446	0.032253
[9579]	PRKCA	--->	EGFR	0.99523	-0.99423
[9580]	ATM	<---	MDM2	-0.056522	0.043434
[9581]	APAF1	<---	CASP3	-0.51809	0.44382
[9582]	CREBBP	--->	CTNNB1	0.019033	-0.032118
[9583]	TNFSF10	--->	TNFRSF11B	0.22303	-0.25953
[9584]	FMNL3	<---	PRPF40A	-0.027603	0.016035
[9585]	PPP2CA	--->	EIF4EBP1	0.45316	-0.45565
[9586]	PHC1	--->	SCMH1	0.15283	0.14921
[9587]	CCL5	<---	GRB2	-0.31815	0.33117
[9588]	FBLN1	--->	SMAD4	0.40681	-0.32352
[9589]	ABL1	--->	BCR	0.0049372	-0.030184
[9590]	SOSTDC1	--->	BMP6	0.43337	-0.38277
[9591]	KPNA1	--->	STAT3	0.5471	-0.60142
[9592]	SON	<---	SNIP1	-0.028545	0.032947
[9593]	ATXN1	<---	RHOXF2	-0.012951	0.0091738
[9594]	MAP3K8	<---	RIPK1	-0.99512	1.0097
[9595]	NFASC	--->	SCN3B	0.21708	-0.21492
[9596]	KCNA4	?	KCNA2	-0.025901	-0.025901
[9597]	GATA1	<---	LMO2	-0.13822	0.18194
[9598]	SRC	--->	EGFR	0.58287	-0.14267
[9599]	FRMD4B	<---	CYTH3	-0.13994	0.14006
[9600]	DLG1	--->	MPP2	-0.0093747	-0.020129
[9601]	RCOR3	<---	KIAA0182	-0.07819	0.096115
[9602]	CDK5	<---	PAK1	-1.3014	1.4008
[9603]	BNIPL	--->	BCL2	0.77054	-0.76289
[9604]	YWHAG	--->	GIT1	0.19425	-0.20074
[9605]	YWHAG	--->	FLNA	0.10466	-0.080511
[9606]	STAMBP	<---	MAP2K5	-0.32962	0.43437
[9607]	CAV1	--->	GJA1	0.123	-0.12021
[9608]	IL1RAP	--->	IRAK1	0.30614	-0.26401
[9609]	GRK5	--->	ADRB2	0.21152	-0.27051
[9610]	ABLIM3	--->	ACTC1	0.3346	-0.33787
[9611]	ECT2	--->	PARD6A	0.075771	-0.075527
[9612]	HIF1A	--->	APEX1	0.84728	-0.84496
[9613]	HOMER3	--->	RYR1	0.023528	-0.036806
[9614]	GATA2	<---	RARA	-0.0060739	0.036935
[9615]	CD2AP	--->	SOS1	0.031811	-0.046797
[9616]	CSNK2A2	--->	PPP1R8	0.7025	-0.72853
[9617]	TBP	--->	JUN	0.004785	-0.01957
[9618]	PPP2R1B	--->	HDAC1	0.12934	-0.13109
[9619]	INPP5D	--->	SHC1	0.33353	-0.36452
[9620]	GGA2	<---	ARF3	-0.4338	0.46028
[9621]	LYN	--->	KIT	1.0007	-1.0039
[9622]	SMARCA4	--->	BRCA1	-0.0039336	-0.006023
[9623]	CDK1	--->	CDC25A	0.69298	-0.67632
[9624]	HCK	--->	RASA1	0.21292	-0.57122
[9625]	EP300	--->	HERC1	0.062911	-0.07333
[9626]	SMARCA2	--->	ESR1	-0.0020412	-0.0086988
[9627]	IRS1	<---	GRB14	-0.016754	0.12075
[9628]	HAND1	?	MYOD1	-0.65635	-0.65635
[9629]	DLG2	--->	ATP2B4	0.1195	-0.12951
[9630]	EGFR	<---	HBEGF	-1.0084	0.99292
[9631]	SPI1	<---	NFATC1	-0.21738	0.21662
[9632]	CSF1R	--->	INPPL1	0.25388	-0.24005
[9633]	WIPF1	--->	WAS	-0.022738	-0.050624
[9634]	NFKBIA	<---	PRKCI	-0.28443	0.26369
[9635]	FURIN	<---	VWF	-0.003526	-0.00060265
[9636]	RIT2	--->	CALM1	0.73657	-0.70265
[9637]	MAG	<---	COL9A1	-0.0077481	-0.004686
[9638]	GRID2	<---	EML1	-0.12528	0.13185
[9639]	NEDD9	<---	NCK1	-0.034419	0.052578
[9640]	CTBP1	--->	RB1	0.18396	-0.19552
[9641]	MAPK8	--->	PRKDC	0.2473	-0.26447
[9642]	ZBTB22	<---	UBQLN4	-0.28073	0.27845
[9643]	BTRC	--->	HNRNPU	0.52571	-0.53173
[9644]	SKIV2L2	--->	PBX2	0.03886	-0.035743
[9645]	INPP5D	<---	FLT3	-0.40069	0.27426
[9646]	VCL	--->	PRPF40A	0.21896	-0.197
[9647]	PAK2	<---	LYN	-0.92039	1.0799
[9648]	CHEK2	--->	PPP2R2B	0.22227	-0.19469
[9649]	ZNF24	<---	EEF1G	-0.28104	0.28224
[9650]	CBLB	--->	ZAP70	0.99213	-1.0008

[9651]	CARD8	--->	IKBKG	0.40581	-0.39725
[9652]	CTNNB1	<---	RXRA	-0.20202	0.18301
[9653]	ARHGEF2	--->	YWHAE	0.15751	-0.15298
[9654]	UBE2K	--->	TP53	0.27448	-0.27316
[9655]	ESR1	--->	CCNH	0.17019	-0.14481
[9656]	CPNE1	--->	SPTBN1	0.14044	-0.14044
[9657]	SPTAN1	<---	CASP3	-0.26287	0.22253
[9658]	GNB2L1	<---	TYK2	-0.2367	0.23069
[9659]	AR	<---	JMJD1C	-0.95717	0.95694
[9660]	ETV6	--->	FLI1	-0.080617	-0.084284
[9661]	IRS2	<---	IL4R	-0.044764	0.035564
[9662]	GATA4	--->	FOS	0.28285	-0.41474
[9663]	PARVA	<---	ILK	-0.41044	0.40608
[9664]	FER	--->	JUP	0.048291	-0.071127
[9665]	TP53	<---	EPHA3	-0.23115	0.22532
[9666]	MPDZ	<---	HTR2C	-0.19778	0.21221
[9667]	CDC42	--->	WAS	0.36914	-0.48596
[9668]	DGKQ	<---	RHOA	-0.60272	0.60674
[9669]	NCKIPSD	--->	DLG4	0.052342	0.038444
[9670]	FBXL8	<---	SKP1	-0.044878	0.055251
[9671]	PROC	<---	PF4	0.0040583	0.011422
[9672]	CASP3	--->	DSG3	0.41171	-0.48435
[9673]	CARD14	<---	BCL10	-0.049275	-0.045256
[9674]	KAT5	--->	GAPDH	0.0081772	-0.0043531
[9675]	DAB1	--->	ITGB5	0.030477	-0.03265
[9676]	SPI1	<---	GATA2	-0.035588	0.086082
[9677]	NEDD9	--->	ZYX	0.032788	-0.036363
[9678]	GRID2	<---	GRIK2	-0.089633	-0.017265
[9679]	EVL	<---	SRC	-0.61049	0.67068
[9680]	DDB2	<---	CUL4A	-0.19793	0.20913
[9681]	FXYD1	<---	DMPK	-1.0061	1.0012
[9682]	UBE3A	--->	UBQLN2	0.23253	-0.22787
[9683]	THAP11	<---	CASP3	-0.12038	0.09327
[9684]	GIT1	<---	HTT	-0.18347	0.17923
[9685]	ZFPM2	--->	GATA1	0.029291	-0.0069687
[9686]	TNFRSF11A	--->	TRAF5	0.19415	-0.20124
[9687]	NFASC	--->	SDCBP	0.094342	-0.10273
[9688]	CAV1	<---	KDR	-0.64382	0.69751
[9689]	RRP9	<---	SMURF1	-0.72185	0.71781
[9690]	BIRC5	<---	CASP3	-0.74701	0.6341
[9691]	EIF2AK3	<---	HSPA5	-0.99901	1.0063
[9692]	SP1	--->	CD2	0.095658	-0.10853
[9693]	MAP2K4	--->	MAPK12	0.64901	-0.41806
[9694]	L3MBTL	--->	ETV7	0.21332	-0.20468
[9695]	YWHAG	--->	PRLR	0.0098763	-0.021831
[9696]	RGS3	--->	YWHAZ	0.15514	-0.15346
[9697]	GNA13	<---	PRKCE	-0.65126	0.58114
[9698]	NR5A1	--->	CTNNB1	0.32434	-0.32855
[9699]	MTUS1	<---	AGTR2	-0.40363	0.43479
[9700]	SYNCRIP	?	HNRNPD	-0.016298	-0.016298
[9701]	FAT1	<---	HOMER3	-0.031199	0.018675
[9702]	MEF2D	<---	YWHAQ	-0.055451	0.048699
[9703]	ATM	<---	TP53BP1	-0.22691	0.2168
[9704]	YWHAG	<---	BAIAP2L1	-0.058218	0.080614
[9705]	PDE10A	<---	CASP3	-0.82625	0.71663
[9706]	PCOLCE	<---	COL2A1	-0.0079791	-0.0013657
[9707]	NCOA3	<---	CHUK	-0.11492	0.091219
[9708]	PTPRC	--->	CD22	0.10592	-0.11812
[9709]	ERRFI1	<---	EGFR	-0.10571	0.096552
[9710]	PTPN18	--->	PSTPIP1	0.66773	-0.67043
[9711]	PIK3R2	<---	EGF	-0.025309	0.010685
[9712]	CACNA1E	<---	HSPA1A	-0.28837	0.28222
[9713]	CBX5	--->	MBD1	0.043323	-0.026942
[9714]	FBXO2	--->	ITGB1	0.1474	-0.15667
[9715]	YWHAZ	<---	MARK3	-0.092392	0.077417
[9716]	TP53	<---	PPP2R2B	-0.41477	0.43338
[9717]	SORBS3	--->	CPSF7	0.096537	-0.098887
[9718]	ACTN3	<---	ACTN2	-0.035392	-0.012181
[9719]	PAK2	<---	HCK	-0.92039	1.0799
[9720]	BCAN	--->	MMP10	0.022421	-0.030194
[9721]	SELL	<---	PODXL	-0.02978	0.025597
[9722]	RAN	--->	ABL1	-0.067047	-0.079959
[9723]	IKZF4	<---	CHD4	-0.01143	-0.0009186
[9724]	MAPK7	--->	GJA1	0.99636	-0.99797
[9725]	LYN	--->	CD19	1.0564	-1.0123
[9726]	CSNK2A1	--->	JUN	1.0022	-1.0148

[9727]	EP300	--->	TP53	0.073526	-0.083705
[9728]	NCOA3	--->	AR	0.03014	-0.043428
[9729]	ESR1	<---	BRCA1	-0.048527	0.059045
[9730]	SSH2	--->	CFL1	0.3315	-0.32562
[9731]	CREBBP	--->	GATA1	0.18629	-0.19036
[9732]	NUMB	--->	ITGB3	0.015753	-0.02256
[9733]	MRAS	--->	RASSF5	1.0025	-0.98684
[9734]	PTPRC	--->	IRS1	0.24879	-0.25355
[9735]	ITSN1	--->	SNX5	0.05469	-0.056578
[9736]	RRAS	--->	MLLT4	0.99712	-0.98672
[9737]	AMOT	<---	MAGI1	-0.01597	0.011127
[9738]	SORBS1	--->	INSM1	0.057865	-0.043376
[9739]	WASF2	<---	ABI3	-0.045711	0.042336
[9740]	SERPING1	<---	PLG	-0.040728	0.057207
[9741]	ADCY5	<---	ADCY2	-0.19185	-0.035829
[9742]	CRKL	--->	STAT5B	0.32804	-0.36563
[9743]	RASL12	--->	SMAD4	0.55322	-0.47159
[9744]	USP6	<---	MYL2	-0.36729	0.40113
[9745]	KATNB1	<---	TGFBR1	-0.21847	0.19212
[9746]	SYK	--->	CD79A	0.99897	-0.99774
[9747]	EGFR	--->	STAT1	0.99452	-0.99826
[9748]	SYNJ1	--->	EPS15	0.075389	-0.085654
[9749]	UBQLN1	--->	SERPINI2	0.17983	-0.14362
[9750]	TLR2	--->	TLR10	0.16414	0.040783
[9751]	RABGEF1	<---	RAB33B	-0.038393	0.027632
[9752]	HOXB3	<---	EP300	-0.073943	0.063516
[9753]	CASP7	<---	NEDD4	-0.072983	0.051513
[9754]	RARA	?	NR2E3	-0.0089162	-0.0089162
[9755]	PYCARD	<---	SRF	-0.10867	0.12986
[9756]	RUNX2	--->	FOS	0.3032	-0.43692
[9757]	GP6	--->	FCER1G	0.47597	-0.4895
[9758]	FBLN1	--->	APP	0.36146	-0.37137
[9759]	CSK	--->	ADRB2	0.56481	-0.47363
[9760]	PARK2	--->	RANBP2	0.0027719	0.0019563
[9761]	SFRS13A	?	SNRNP70	-0.016298	-0.016298
[9762]	ADAM15	<---	ARHGEF7	-0.025675	0.01734
[9763]	PAK3	--->	MYO6	0.36482	-0.20271
[9764]	ZNF165	<---	MYST2	-0.28649	0.28738
[9765]	JMY	<---	MDM2	-0.036383	0.024856
[9766]	NEBL	--->	ACTN1	0.056099	-0.052241
[9767]	RAB38	--->	SMAD3	0.55322	-0.47159
[9768]	RPS6KB1	--->	EIF4EBP1	0.35226	-0.56724
[9769]	RGS13	--->	GNAQ	1.0012	-1.0015
[9770]	FGFR2	<---	FGF7	-0.33023	0.32707
[9771]	PTPN6	--->	NOS1	0.22824	-0.2252
[9772]	TRAF6	--->	MAP2K7	0.98609	-1.0149
[9773]	PLCG2	<---	FLT1	-0.24105	0.17639
[9774]	HDAC5	--->	GABARAP	0.041393	-0.036169
[9775]	CBLB	--->	FLT3	0.52016	-0.53306
[9776]	TYK2	--->	STAT1	0.12795	-0.18775
[9777]	CD4	<---	CD28	-0.050189	-0.049466
[9778]	RALYL	<---	LMO3	-0.021109	0.054056
[9779]	NR5A2	<---	NR0B2	-0.028437	-0.0094276
[9780]	PRKCA	--->	TOP2A	0.89006	-0.88857
[9781]	CSNK2A1	--->	MAZ	0.70307	-0.63523
[9782]	BID	--->	BCL2A1	0.49845	-0.46902
[9783]	CTNND1	<---	COL17A1	-0.027152	0.014554
[9784]	CDKN2A	<---	HIF1A	-0.48949	0.486
[9785]	DUSP16	--->	MAPK11	0.87425	-0.89445
[9786]	SYT13	--->	NRXN1	0.083467	-0.043035
[9787]	CAMK2G	--->	CREM	0.61276	-0.61036
[9788]	CCNA1	<---	CDK1	-1.0774	1.0544
[9789]	TRHR	--->	ARRB1	0.27159	-0.25831
[9790]	MAPK8	--->	TP53	1.0002	-0.99962
[9791]	CDC14B	--->	TP53	0.61877	-0.60562
[9792]	TRAT1	<---	FYN	-0.99229	0.99353
[9793]	YWHAZ	--->	GP1BB	0.01833	0.0093578
[9794]	CTNNB1	<---	FOXO4	-0.22409	0.2155
[9795]	TIE1	--->	PIK3R1	0.0023727	-0.019396
[9796]	IGFBP3	<---	CAV1	-0.035066	0.062832
[9797]	IGF1	--->	IGFBP1	0.087568	-0.072295
[9798]	SERPINB13	<---	PLG	-0.040728	0.057207
[9799]	DGKZ	<---	SNTB1	-0.037771	0.038662
[9800]	PRKACA	--->	WT1	0.81966	-0.82415
[9801]	CAMK2A	--->	NOS1	0.30424	-0.31365
[9802]	CDK1	--->	RB1	0.98937	-1.0015

[9803]	HNRNPL	--->	APEX1	0.017968	0.016125
[9804]	YWHAG	--->	AKAP13	0.0055202	-0.0046727
[9805]	NFKB1	--->	NFKBIA	0.47023	-0.035892
[9806]	PTPN4	--->	ZZEF1	0.28387	-0.2925
[9807]	ETS1	--->	SMAD3	0.15961	-0.11281
[9808]	ARL4C	--->	UBQLN4	0.39767	-0.37127
[9809]	RNF8	<---	UBE2E2	-0.10861	0.11055
[9810]	PTPN14	--->	CDH1	0.13952	-0.15107
[9811]	EPHA4	--->	FGFR3	0.020297	-0.065129
[9812]	RGS16	--->	GNAT1	1.0012	-1.0015
[9813]	PHLDB2	--->	FLNC	0.28513	-0.263
[9814]	CD28	--->	CD80	-0.042802	-0.057081
[9815]	IL13RA1	<---	IL4R	-0.091274	0.10076
[9816]	SHC1	<---	NTRK3	-0.19951	0.0897
[9817]	YWHAG	--->	TIAM1	0.45747	-0.4627
[9818]	PIK3CB	--->	PRKCE	0.30043	-0.29528
[9819]	IL6ST	--->	VAV1	-0.0065353	-0.048025
[9820]	CTNNB1	<---	ZFYVE9	-0.085864	0.073963
[9821]	PAK1	--->	CSNK2A1	1.4008	-1.3014
[9822]	PKD1	--->	CTNNB1	0.3269	-0.33148
[9823]	CDK1	--->	CREM	0.96654	-0.95401
[9824]	CDH1	<---	JUP	-0.1649	0.15626
[9825]	MAGI3	<---	ADRB1	-0.037356	0.032002
[9826]	RALYL	<---	CCDC85B	-0.050711	0.077385
[9827]	PIAS1	--->	AR	0.35279	-0.36146
[9828]	NEDD9	--->	SMAD1	0.079582	-0.095055
[9829]	JAG1	<---	MLLT4	-0.010182	-0.0029131
[9830]	GDF5	--->	ACVR2A	1.0057	-0.99943
[9831]	BAZ1B	<---	SMARCC2	-0.028472	0.044187
[9832]	TTRAP	<---	TRAF6	-0.68657	0.67949
[9833]	RCOR1	--->	REST	0.11358	-0.097415
[9834]	RGS4	<---	ERBB3	-1.0133	0.99759
[9835]	GRIN1	<---	DNM1	-0.0093992	-0.0013726
[9836]	SPAG9	<---	MAP2K4	-0.16592	0.14982
[9837]	DNM2	<---	CDK1	-0.29072	0.24668
[9838]	CLSTN3	<---	APBA2	-0.030515	0.03533
[9839]	EEA1	--->	TOM1	0.034946	0.02489
[9840]	MAP4K3	--->	BIN1	0.27127	-0.1674
[9841]	JAK3	--->	IL4R	0.38138	-0.37332
[9842]	PTPRM	--->	PCSK5	0.093389	-0.10377
[9843]	PRKG1	--->	TRPC3	0.62998	-0.63462
[9844]	PARD6B	--->	MPP5	-0.0046672	-0.015151
[9845]	FHL5	--->	CREB1	0.32862	-0.31634
[9846]	GRIN3B	<---	DLG4	-0.099564	0.090685
[9847]	PCBP1	<---	PUF60	-0.0048144	0.036464
[9848]	CAPN1	--->	SLIT3	0.0022423	-0.015568
[9849]	CAMK2A	--->	ETS1	0.68344	-0.68792
[9850]	ERBB2IP	--->	DLG4	-0.00028142	-0.012884
[9851]	TRIB3	<---	AKT1	-1.0071	1.0168
[9852]	CHD3	--->	ACTR1B	0.017389	-0.027727
[9853]	GPSM3	<---	GNAI1	-0.20473	0.22114
[9854]	JAG2	--->	NOTCH1	-0.0069973	-0.26198
[9855]	RASGRP1	--->	HRAS	1.0048	-1.0011
[9856]	MAPK14	--->	EEA1	0.29074	-0.46077
[9857]	RB1	<---	ZBTB16	-0.086324	0.075549
[9858]	GRIN2D	<---	RAP2A	-0.19864	0.19661
[9859]	ARID3A	<---	UBE2E3	-0.29904	0.31231
[9860]	AXIN2	--->	CTNNB1	0.058263	-0.069945
[9861]	PIK3CA	<---	IRS2	-0.21631	0.20663
[9862]	CAV1	<---	PTPRF	-0.079001	0.071036
[9863]	KLK3	--->	SERPINA1	0.11833	-0.082185
[9864]	VCL	--->	SELE	0.35374	-0.34628
[9865]	MAPK1	--->	UBTF	0.92033	-0.89929
[9866]	TRPM7	--->	PLCG1	0.1284	-0.15474
[9867]	ZNF205	<---	UBQLN4	-0.30654	0.30742
[9868]	PIAS4	<---	UBE2K	-0.023001	0.024453
[9869]	SMAD2	--->	HOXA9	0.20304	-0.14259
[9870]	GRIN2D	<---	FUS	-0.075793	0.069276
[9871]	AKT1	--->	PRKDC	0.14012	-0.093823
[9872]	LHX1	?	ISL1	-0.017692	-0.017692
[9873]	HSPA13	--->	UBQLN1	0.78427	-0.7421
[9874]	RYR1	<---	CACNA1S	-0.017946	-0.0067789
[9875]	KCNV1	?	KCNB2	-0.025901	-0.025901
[9876]	GAB2	<---	PLCG2	-0.027128	-0.016241
[9877]	TCF7L2	<---	EP300	-0.10716	0.097235
[9878]	MTA2	--->	RBBP7	0.021671	0.019573

[9879]	PPP1CC	--->	ZFYVE9	-0.0035309	-0.0098159
[9880]	LRP2	--->	TLN1	0.00064157	-0.01353
[9881]	HNRNPA2B1	?	SNRNP70	-0.016298	-0.016298
[9882]	ITGB3	<---	FGA	-0.048962	0.046421
[9883]	NR0B2	--->	AR	0.0037525	-0.5822
[9884]	SMARCA5	--->	CREB1	0.066689	-0.07485
[9885]	ID1	<---	CASK	-0.31676	0.31398
[9886]	IL1F10	--->	IL1R1	0.28289	-0.28221
[9887]	NBN	--->	CCNE1	0.63857	-0.62524
[9888]	HOXB3	<---	CREBBP	-0.073943	0.063516
[9889]	CPNE2	<---	UBE2M	-0.12637	0.16628
[9890]	PRKCQ	--->	FYN	0.032722	0.027224
[9891]	CDK14	--->	CCND3	1.0544	-1.0774
[9892]	PLD2	<---	ACTN1	-0.1753	0.16544
[9893]	ITGB4	<---	YWHAB	-0.034638	0.025286
[9894]	ISL1	?	LMX1A	-0.017692	-0.017692
[9895]	AMBP	--->	A2M	0.14247	-0.10912
[9896]	ERG	?	ETS2	-0.13143	-0.13143
[9897]	SCEL	<---	TXNIP	0.0063251	0.009004
[9898]	RPS6KA2	<---	MAPK1	-1.0109	1.002
[9899]	PTPN11	--->	PTK2B	0.99625	-0.99764
[9900]	PRKAR2A	--->	SMAD4	0.54864	-0.46649
[9901]	MSN	<---	ICAM2	-0.021095	0.013885
[9902]	ESR2	<---	TRIM59	-0.16971	0.19453
[9903]	HNRNPU	<---	SYK	-0.69768	0.7308
[9904]	MYLK	--->	ACTA1	0.35726	-0.37945
[9905]	DLG4	--->	KCNA3	0.047736	-0.049447
[9906]	EPHB6	--->	CRKL	0.13036	-0.17576
[9907]	EPS15	<---	REPS2	-0.08655	-0.0050484
[9908]	THRA	<---	NCOR2	-0.14734	0.13736
[9909]	VAV1	<---	FYN	-0.99968	1.0003
[9910]	CCR1	--->	CCL3	0.49595	-0.46475
[9911]	THBS1	<---	COL5A1	-0.079817	0.090221
[9912]	RARA	--->	BAG1	0.025363	-0.0036637
[9913]	CDH1	<---	CTNNB1	-0.98928	0.99241
[9914]	GRIA3	<---	LYN	-0.38586	0.43735
[9915]	SOCS1	--->	IL2RB	0.17341	-0.22593
[9916]	INSR	<---	CALM3	-0.1135	0.10567
[9917]	CTNNA1	--->	CDH3	0.4013	-0.39209
[9918]	SMAD2	--->	MLLT4	0.087312	-0.077357
[9919]	CD36	<---	FYN	-0.94926	1.109
[9920]	ATXN1	--->	KLF11	0.021749	-0.028521
[9921]	CRKL	<---	TYK2	-0.1562	0.10815
[9922]	PPYR1	<---	PYY	-0.19468	0.20502
[9923]	OSMR	<---	JAK1	-0.5537	0.54255
[9924]	MAPK3	--->	STAT5A	0.7916	-0.80262
[9925]	PRKCE	--->	FYN	0.15252	-0.11897
[9926]	EPOR	--->	SH2B2	-0.042363	-0.076002
[9927]	ARRB1	--->	ZBTB43	0.15294	-0.15983
[9928]	PRSS1	<---	TST	-0.041795	0.078466
[9929]	OCRL	<---	RAB5A	-0.25397	0.25628
[9930]	SRC	--->	ERBB2	0.9962	-0.99408
[9931]	RCN3	--->	PCSK6	0.054625	-0.061242
[9932]	DLGAP3	<---	DLG3	-0.040133	0.038414
[9933]	STAT3	<---	CXCR4	-0.67486	0.62118
[9934]	UBE2L6	<---	UBE3A	-0.17948	0.18395
[9935]	RNF123	--->	SMAD9	0.235	-0.25234
[9936]	PLD2	<---	BIN1	-0.29133	0.28396
[9937]	STK36	--->	SUFU	0.44964	-0.46495
[9938]	DAB2	--->	AXIN1	0.17782	-0.18074
[9939]	CBX7	<---	RING1	0.016956	0.017911
[9940]	TCF7L1	<---	CTNNB1	-1.0087	0.9959
[9941]	NRAS	--->	SMAD4	0.55322	-0.47159
[9942]	DMTF1	--->	CCND3	0.64939	-0.63597
[9943]	INHBA	<---	ENG	-0.30966	0.36037
[9944]	HEYL	<---	SMAD7	-0.62633	0.61773
[9945]	NR1H3	<---	RXRB	-0.0098959	-0.0063998
[9946]	BLK	--->	FCGR2B	0.11748	0.015127
[9947]	CASP3	--->	IL16	0.59353	-0.70295
[9948]	ESR1	--->	CDC25B	0.026116	-0.0083302
[9949]	IRAK3	<---	TRAF6	-0.59748	0.62876
[9950]	ATXN1	<---	TRIM32	-0.064741	0.053477
[9951]	KLF5	<---	CREBBP	-0.12188	0.11387
[9952]	GRB14	<---	TEK	-0.014867	-0.0050415
[9953]	GNA13	<---	TSHR	-0.65379	0.65587
[9954]	SVIL	--->	FLOT1	0.21286	-0.15484

[9955]	CAV1	<---	NTRK1	-0.043173	0.032422
[9956]	PTPRS	--->	EGFR	0.085254	-0.099172
[9957]	CASP8	--->	MAPK1	0.99111	-0.99197
[9958]	CSK	<--->	LYN	0.37153	0.37153
[9959]	SMARCD3	--->	SREBF1	0.19867	-0.20283
[9960]	PPP2R1B	--->	CDK4	0.30999	-0.30289
[9961]	ABL1	<---	MDM2	-0.038456	-0.0058126
[9962]	HAND2	--->	TCF3	-0.13552	-0.39218
[9963]	MMP1	<---	COL2A1	-0.051184	0.041848
[9964]	NGF	--->	SORT1	0.1256	-0.093056
[9965]	ZRANB2	--->	SFRS2	0.078392	-0.046406
[9966]	STAT1	<---	SYK	-0.57368	0.67225
[9967]	EPB41L1	<---	YWHAB	-0.057222	0.05792
[9968]	HDAC4	--->	TNFRSF14	0.18467	-0.19362
[9969]	TLR8	--->	MYD88	0.11043	0.0073348
[9970]	CD40	<---	BIRC2	-0.18226	0.16508
[9971]	YWHAB	<---	MPRIP	-0.17549	0.18747
[9972]	ELP2	<---	JAK2	-0.17685	0.29786
[9973]	BUB1	--->	APC	0.13984	-0.15381
[9974]	TP63	--->	DAXX	0.0054877	-0.016258
[9975]	KCNA5	<---	DLG1	-0.049447	0.047736
[9976]	SERPINA5	<---	FGA	-0.083807	0.11995
[9977]	MTNR1A	--->	GNA11	1.0129	-1.008
[9978]	TSC1	<---	YWHAE	-0.08934	0.11987
[9979]	TSC2	<---	RPS6KA1	-1.0083	1.002
[9980]	GAB2	<---	INPP5D	-0.5466	0.53493
[9981]	ID2	--->	TCF3	-0.13552	-0.39218
[9982]	MACF1	--->	ACTA1	0.10035	-0.1072
[9983]	BAIAP2	--->	EPS8	0.021661	-0.061246
[9984]	INADL	--->	GRIN2D	0.11324	-0.11391
[9985]	PPP1R13L	--->	TP53	0.11808	-0.11725
[9986]	TNFRSF1A	<---	LTA	-0.51583	0.7202
[9987]	USF2	--->	FOS	0.18276	-0.31574
[9988]	CYR61	<---	ITGAV	-0.041504	0.027796
[9989]	PARD6A	<---	MAP2K5	-0.21477	0.086506
[9990]	GRK4	--->	BDKRB2	0.17121	-0.19042
[9991]	CXCR2	--->	CXCL3	0.49595	-0.46475
[9992]	HSPB3	<---	ZZEF1	-0.078599	0.12648
[9993]	FBXW7	--->	MYC	0.19831	-0.20399
[9994]	EP300	--->	MDC1	0.18211	-0.18644
[9995]	TAF1	--->	GTF2A1	0.043644	-0.045238
[9996]	SH3GL2	--->	DNM1	0.071535	-0.080916
[9997]	KLHL12	<---	SNX20	-0.011646	0.044705
[9998]	FCAR	--->	CD79A	0.46042	-0.45529
[9999]	MMP1	<---	TFPI	-0.1877	0.20851
[10000]	PRDM1	--->	ATXN1	-0.0034945	-0.00808
[10001]	ID3	--->	TCF12	-0.091025	-0.33696
[10002]	GNA11	<---	ADRB2	-0.98627	0.9872
[10003]	TIRAP	<---	TLR4	-0.98566	0.98744
[10004]	HES1	<---	JAK2	-0.031054	0.035378
[10005]	NCOA1	<---	NFKB1	-0.11397	0.10304
[10006]	MDM4	--->	USP7	0.042625	-0.045125
[10007]	ARF6	--->	MPP5	0.21231	-0.20858
[10008]	FERD3L	--->	TCF3	-0.13552	-0.39218
[10009]	KLRK1	<---	ULBP3	-0.0029242	0.054213
[10010]	CNKSR1	<---	RHOA	-0.12754	0.13451
[10011]	MLL	--->	RBBP5	0.097755	-0.093606
[10012]	THBS1	<---	COL1A1	-0.048568	-0.0032258
[10013]	EPHB2	--->	ABL1	0.0059484	-0.07631
[10014]	MAPK14	--->	ATF2	1.0024	-0.99433
[10015]	SRF	--->	PRRX1	0.154	-0.15021
[10016]	MAP2K2	<---	BRAF	-1.0053	1.0017
[10017]	DHX30	<---	CDK9	-0.44264	0.42167
[10018]	TAL1	--->	TCF3	-0.085357	-0.2402
[10019]	MAP4K4	--->	NCK1	0.04177	-0.0063627
[10020]	CCNK	<---	HMG20A	-0.36989	0.38735
[10021]	RELA	<---	CALM1	-0.026091	0.032593
[10022]	CSNK2A1	--->	CAV1	0.70376	-0.73589
[10023]	TRAT1	--->	PIK3R1	0.080048	-0.15352
[10024]	PPFIA4	--->	GIT1	0.16054	-0.16385
[10025]	DOCK3	--->	CRK	0.041346	-0.0084299
[10026]	MMP26	<---	SERPINA1	0.012498	0.025482
[10027]	WASL	<---	CTTN	-0.095415	0.076798
[10028]	CASP8	--->	CTNNB1	0.22398	-0.23958
[10029]	NTRK2	<---	BDNF	-0.14001	0.13278
[10030]	F2RL2	--->	F11R	0.00028002	-0.0099791

```
[10031] S1PR5   --->    GNA12   1.0129  -1.008
[10032] DMTF1   --->    CCND2   0.64939 -0.63597
[10033] UBE2E3  <---    CHUK    -0.31245        0.17962
[10034] UNC13B  --->    DOC2A   0.13698 -0.017962
[10035] CSNK1A1 --->    FADD    0.29197 -0.30689
[10036] SMARCD3 --->    NR5A2   0.052806        -0.042542
[10037] TANC1   <---    CAMK2A  -0.24102        0.23406
[10038] FGFR3   <---    FGF7    -0.3745 0.36763
[10039] PRKAR1A --->    PYCARD  0.48048 -0.44864
[10040] CDK1    --->    CDKN1A  0.51838 -0.55156
[10041] PLD2    <---    ACTB    -0.24185        0.23353
[10042] BCOR    <---    CPNE4   -0.37401        0.39237
[10043] CYTH2   <---    TRIM23  -0.14363        0.14692
[10044] CRK     <---    NCK1    0.13954 0.1738
[10045] SP1     <---    EP300   -0.042454       0.030443
[10046] HCLS1   --->    QKI     0.045754        -0.043136
[10047] GEMIN5  <---    DDX20   -0.076481       0.091156
[10048] UBQLN1  --->    SMAD9   0.33166 -0.35027
[10049] ARHGEF4 <---    RHOA    -0.053017       0.048357
[10050] IRAK1   <---    TLR4    -0.080178       0.066397
[10051] RBL1    <---    BEGAIN  -0.28412        0.27409
[10052] PIK3R1  <---    CSF1R   -0.51757        0.49923
[10053] CDK1    --->    IL16    0.62013 -0.64226
[10054] BTK     --->    PLCG2   1.0063  -1.0027
[10055] FYN     --->    IL2RB   1.2619  -1.172
[10056] ESR1    <---    HNF4A   -0.087134       0.20637
[10057] IGF1R   <---    GNAI1   -0.054709       0.042064
[10058] CAV3    <---    PFKM    0.0076915       0.020073
[10059] TFR2    <---    TF      0.0012324       0.0024573
[10060] ELP2    --->    STAT3   0.54356 -0.59697
[10061] EP300   --->    MYB     0.018722        -0.03137
[10062] FYN     --->    CTNNB1  0.24969 -0.25034
[10063] SRC     --->    LRP1    0.077907        -0.08422
[10064] ARL4D   --->    SMAD1   0.56252 -0.5858
[10065] UBE2D2  <---    PJA1    -0.033933       0.028297
[10066] PABPC1  --->    PCBP2   0.036464        -0.0048144
[10067] BCL6    <---    PPARD   -0.2268 0.21581
[10068] CAMK2G  --->    GRIA1   0.62485 -0.62407
[10069] PRPF40A --->    UBQLN4  0.26122 -0.22895
[10070] UBE2D3  --->    RNF126  0.18537 -0.17305
[10071] F11     --->    F2      0.0012258       -0.0091243
[10072] CCNE1   <---    CABLES1 -0.65097        -0.11778
[10073] CD79B   <---    PTK2    -0.21367        0.10195
[10074] PGR     --->    KLF9    0.066132        -0.064125
[10075] EPB41L1 <---    YWHAQ   -0.057222       0.05792
[10076] THRA    --->    CREB1   0.1808  -0.17665
[10077] THRA    --->    CCND1   0.3199  -0.31113
[10078] SP4     <---    SP1     -0.043223       0.034089
[10079] MMP14   ?       MMP2    -0.12951        -0.12951
[10080] ACVR1   <---    RHOD    -0.32935        0.31819
[10081] SH2D1A  <---    FYN     -0.85193        1.4334
[10082] ELF4    <---    FBXO7   -0.2897 0.33154
[10083] MDM2    --->    ARRB2   0.074964        -0.082358
[10084] JUB     <---    CTNNA1  -0.47625        0.50132
[10085] MAP2K4  <--->   MAPK9   0.23429 0.23429
[10086] PPFIA1  <---    PTPRC   -0.20187        0.20471
[10087] PRKDC   <---    HSP90AA1        -0.027061       0.01538
[10088] PDGFRA  --->    STAT5A  0.060186        -0.073579
[10089] NCK2    --->    SF3B4   0.20992 -0.21492
[10090] SMPD2   --->    PIK3CG  0.069695        -0.062794
[10091] CUL1    <---    HIPK2   -0.32466        0.30021
[10092] PRKCE   --->    ACTC1   0.40221 -0.45121
[10093] BRCA1   --->    POU2F1  0.09568 -0.081108
[10094] PTPN22  --->    CSK     -0.028523       -0.056604
[10095] INSR    --->    DOK4    1.0063  -1.003
[10096] BMP2    <---    ASCL1   -0.020325       0.073076
[10097] MYPN    <---    NEBL    -0.040427       0.069388
[10098] BAG1    <---    MET     -0.46058        0.44519
[10099] PTK2    <---    EZR     -0.057198       -0.049595
[10100] RBM39   --->    ESR2    0.16436 -0.12846
[10101] CAPN1   --->    CASP14  0.20931 -0.21258
[10102] PPP4C   <---    NFKB1   -0.022572       0.010142
[10103] SFN     --->    PLK4    0.064981        -0.084695
[10104] SVIL    --->    ACTB    0.58077 -0.56416
[10105] EIF4A3  <---    YWHAQ   -0.035857       0.045156
[10106] MALT1   --->    SQSTM1  0.20665 -0.21804
```

```
[10107] HSPA4   --->    KPNA2   0.88421 -0.84382
[10108] GRASP   <---    CYTH3   -0.22934        0.25364
[10109] CHD3    --->    IKZF1   0.0004744       -0.011955
[10110] SHC1    <---    CSF2RB  -0.14829        0.035579
[10111] ERBB2IP --->    PKP4    0.066333        -0.065208
[10112] PFN2    <---    RAP1GAP -0.40057        0.39858
[10113] PIAS1   --->    NR3C2   0.095399        -0.090133
[10114] ADAMTS1 --->    VCAN    5.5806e-005     -0.012914
[10115] CSNK2A2 <---    SMURF1  -0.6445 0.64008
[10116] MAPT    <---    HSPA8   -0.72957        0.75427
[10117] BCAR1   --->    FXYD6   0.038836        -0.035643
[10118] HIST3H3 <---    TAF1    -0.091998       0.10193
[10119] PHKG1   --->    CALM1   1.007   -1.0109
[10120] DLST    --->    PFN2    0.090829        -0.058096
[10121] ADAP1   <---    PRKCI   -0.2732 0.2596
[10122] TAOK1   <---    MARK1   0.012035        0.045095
[10123] ARHGEF2 <---    RAC1    -0.14455        0.15203
[10124] SHC1    <---    FCGR3A  -0.46105        0.30147
[10125] DAB2    <---    TGFBR2  -0.85166        0.91305
[10126] HES1    <---    ID4     -0.29914        -0.087238
[10127] YWHAZ   <---    WEE1    -0.66368        0.62776
[10128] HNF4A   --->    NPPA    0.32855 -0.31961
[10129] GAB1    --->    GRB2    0.089757        -0.09583
[10130] PPFIA3  <---    PTPRU   -0.20119        0.20611
[10131] MYB     <---    SKP2    -0.022924       0.015461
[10132] TNFRSF11A       <---    MAP3K7  -0.2252 0.21637
[10133] DOCK2   --->    VAV1    -0.006288       -0.010857
[10134] CASP3   --->    PLA2G4A 0.4809  -0.54601
[10135] PRKCA   --->    EZR     0.33349 -0.34166
[10136] YES1    --->    CDKN1B  0.3974  -0.34695
[10137] RIPK4   <---    PRKCB   0.27226 0.31425
[10138] PPP4R1  <---    PPP4C   -0.36131        0.3544
[10139] STAT5A  <---    EGFR    -0.99025        0.99454
[10140] KLK2    --->    SERPINC1        0.11833 -0.082185
[10141] DOK1    <---    RASA1   -0.32041        0.17841
[10142] KIF1B   --->    INADL   0.086963        -0.089252
[10143] RNF40   --->    RB1     0.47982 -0.4811
[10144] GRB10   <---    EPHB1   -0.24782        0.11635
[10145] CSRNP2  <---    PPP1CA  -0.23597        0.22914
[10146] DUSP10  --->    MAPK11  0.87425 -0.89445
[10147] AUP1    --->    ITGAV   0.018392        -0.029406
[10148] TNFRSF11A       <---    CBLB    -0.33561        0.32332
[10149] ITGAD   --->    ITGB2   -0.0059371      -0.0079211
[10150] ERBB2IP --->    SMAD2   0.17801 -0.15234
[10151] PAX6    <---    HOMER3  -0.20998        0.206
[10152] KAT5    <---    EDNRA   -0.24931        0.26283
[10153] PPFIBP2 --->    DTNB    0.13759 -0.12093
[10154] PIK3CB  <---    AGTR2   -0.53402        0.53348
[10155] INSR    <---    CALM2   -0.1135 0.10567
[10156] BCL2A1  ?       BAK1    -0.63915        -0.63915
[10157] BTLA    <---    PTPN6   -0.077948       0.26319
[10158] HCLS1   --->    ACTA1   0.29489 -0.28941
[10159] IGSF5   --->    MAGI1   0.029486        -0.039844
[10160] F8A1    <---    TLR10   -0.13791        0.15066
[10161] RYR2    <---    PRKAR2A -0.37028        0.36453
[10162] TNFRSF1A        <---    HSP90AA1        -0.010706       0.023305
[10163] LYN     --->    PLCG1   0.81503 -1.0097
[10164] SKIL    <---    ZBTB3   -0.096341       0.087678
[10165] PRKACA  --->    VASP    0.5801  -0.59926
[10166] RAB17   <---    SMURF2  -1.0084 1.0007
[10167] DOK1    <---    RET     -0.50436        0.48844
[10168] NR1D2   <---    UBE2I   -0.16643        0.1925
[10169] PIK3R1  <---    KIT     -1.0037 0.99342
[10170] RB1     <---    TRIM27  -0.18816        0.17889
[10171] ITGB4   <---    ITGA6   -0.0071201      -0.0061495
[10172] RAF1    <---    LCK     -0.061898       0.38207
[10173] DLGAP1  <---    DLG1    -0.040133       0.038414
[10174] CD2AP   --->    SOS2    0.031811        -0.046797
[10175] SOX10   <---    OLIG2   -0.01203        0.0043059
[10176] PPFIA2  <---    PTPRD   -0.071311       0.064659
[10177] SORBS1  --->    ABL1    -0.085717       -0.1336
[10178] MYD88   --->    SPOP    0.083126        -0.0989
[10179] SHC1    --->    STAT5A  0.0062057       -0.041328
[10180] MERTK   --->    VAV1    0.10006 -0.22066
[10181] PTF1A   --->    TCF12   -0.091025       -0.33696
[10182] PXN     <---    PDPK1   -0.22442        0.30798
```

```
[10183] CTSG    <---    CXCR4   -0.42368        0.45462
[10184] WWOX    <---    MAPK8   -0.27576        0.25499
[10185] MMP17   <---    TNF     -0.35472        0.3692
[10186] RBBP4   --->    SP3     0.10701 -0.098467
[10187] CASP3   --->    RAC1    0.36319 -0.47633
[10188] COL4A5  --->    SERPINE2        0.071404        -0.073538
[10189] BPTF    --->    KEAP1   0.004424        -0.012063
[10190] RAB27A  --->    RPH3A   0.11783 -0.096859
[10191] PRKCB   --->    GRM5    0.91747 -0.9157
[10192] CD28    --->    PIK3CG  0.055449        -0.050588
[10193] KRIT1   <---    RAP1A   -0.99591        0.99542
[10194] CALR    <---    APOB    -0.085378       0.1056
[10195] CLEC2D  <---    KLRB1   -0.11173        -1.7905e-006
[10196] KLK2    --->    IGFBP2  0.070438        -0.036479
[10197] ITGA9   --->    ITGB1   -0.0084417      -0.018859
[10198] MLL     --->    RNF2    0.086432        -0.069374
[10199] TRAF6   --->    TIRAP   0.64777 -0.65562
[10200] PRMT1   --->    FGF2    0.29521 -0.29296
[10201] WASF2   <---    DOCK1   -0.012623       -0.00081121
[10202] STAM    <---    JAK1    -0.35847        0.34904
[10203] TLE1    <---    ATN1    -0.058195       0.055796
[10204] DTNB    <---    CCDC85B -0.030513       0.059654
[10205] MAPK9   --->    XPO7    0.69945 -0.7255
[10206] GC      --->    ACTA1   0.47181 -0.46982
[10207] CABIN1  <---    PPP3CA  -0.16668        0.16039
[10208] ELK4    --->    ID1     0.12676 -0.084944
[10209] YWHAG   --->    NUMBL   0.045962        -0.045339
[10210] KHDRBS1 --->    PLCG2   0.065953        -0.13344
[10211] F11     <---    GP1BA   -0.050941       0.052885
[10212] TRAF1   <---    TNFRSF1A        -0.12843        0.23727
[10213] TRAF1   --->    CD40    0.029707        -0.034181
[10214] BTK     --->    DAPP1   1.0024  -1.001
[10215] MAPK1   --->    LIFR    0.74193 -0.77508
[10216] RIMS1   <---    RAB26   -0.0067445      0.016184
[10217] TIFA    <---    LNX1    0.0022788       0.013749
[10218] PARVA   --->    ACTA1   0.51886 -0.51601
[10219] MAPKAPK5        --->    JUN     0.57465 -0.71264
[10220] SERPINA5        <---    KLKB1   -0.082185       0.11833
[10221] CSNK2A1 --->    WAS     0.18552 -0.50928
[10222] CDK1    --->    KHDRBS1 0.35791 -0.37587
[10223] NKX3-1  <---    SRF     -0.11346        0.13352
[10224] NCK2    --->    ABI1    0.14877 -0.070932
[10225] SHB     --->    PDGFRA  0.024988        -0.03522
[10226] SKP1    --->    FBXO5   0.073456        -0.072534
[10227] NFKB1   --->    IKBKG   0.47756 -0.47909
[10228] JUN     --->    ATF2    -0.059804       -0.17016
[10229] HDAC5   --->    BCL6    0.21845 -0.23368
[10230] PTPN12  --->    FLT3    0.30202 -0.31794
[10231] PROS1   --->    PROC    -0.015753       -0.016256
[10232] SHOC2   <---    HRAS    -0.0046468      0.015643
[10233] HDAC9   <---    NCOR1   -0.064436       0.058272
[10234] CASP9   <---    APAF1   -0.09754        -0.067536
[10235] PPARGC1A        <---    GSK3B   -0.16752        0.14666
[10236] CASP7   --->    TNFRSF1A        0.35625 -0.32424
[10237] IKBKG   <---    BIRC2   -0.98653        0.99943
[10238] YWHAQ   --->    RAI14   0.6652  -0.64224
[10239] ERBB2   --->    HSP90AA1        0.026699        -0.039391
[10240] RAPGEF4 --->    MAP1A   0.097076        -0.10761
[10241] PACSIN3 <---    SOS2    -0.32202        0.31511
[10242] FXR2    <---    RBPMS   -0.0048144      0.036464
[10243] CTNND1  <---    JUP     -0.021448       -0.021015
[10244] ITGA2B  <---    AUP1    -0.036779       0.026198
[10245] AKT1    --->    PEA15   0.73681 -0.61247
[10246] PBX1    --->    HOXA10  -0.053398       -0.090184
[10247] MAP2K4  --->    FLNA    0.72542 -0.75118
[10248] EIF4G1  --->    EIF4A2  0.087815        -0.073047
[10249] MAPK3   --->    SP1     0.12886 -0.14656
[10250] DNMBP   --->    WASF1   0.027326        -0.044241
[10251] TNK2    <---    NCK1    -0.012677       -0.0067431
[10252] TGFBR1  --->    SNX6    0.35774 -0.39149
[10253] TGFBR2  --->    TGFBR1  0.99398 -1.0032
[10254] FGFR1   <---    FGF17   -0.99871        1.0053
[10255] FBXO7   <---    BIRC2   -0.13666        0.13194
[10256] DLG4    --->    KCNA1   0.047736        -0.049447
[10257] CDK1    --->    MAPT    0.99929 -1.0027
[10258] RASSF1  --->    CDC20   0.13    -0.1137
```

[10259]	THRB	<---	HDAC2	-0.074402	0.085304
[10260]	SOCS3	--->	LEPR	0.2583	-0.26195
[10261]	IKBKB	--->	HSP90AA1	0.023686	-0.064239
[10262]	ARHGAP17	<---	FNBP1	-0.040952	0.043569
[10263]	RASD2	<---	SMURF2	-1.0084	1.0007
[10264]	LMO2	<---	STAT1	-0.28284	0.15805
[10265]	ACTN4	<---	PTPN11	-0.2308	0.29245
[10266]	KPNA2	--->	NFKBIB	0.56039	-0.52914
[10267]	PTPN11	--->	INSR	0.15547	-0.13143
[10268]	MAP3K4	<---	MAP2K3	-0.38277	0.050282
[10269]	EPB41	<---	YWHAB	-0.057222	0.05792
[10270]	E2F4	--->	RBL2	0.28436	-0.29297
[10271]	BAX	<---	NOL3	-0.44847	0.46531
[10272]	RAF1	<---	KRAS	-0.9994	1.0008
[10273]	CASP8	--->	CDC2L2	0.61956	-0.62555
[10274]	IRAK2	--->	PELI3	0.85311	-0.63354
[10275]	LIMK1	--->	BMPR2	0.21001	-0.23343
[10276]	PIK3R2	<---	AXL	-0.20761	0.15014
[10277]	SIX2	--->	TLE1	0.05243	-0.021516
[10278]	SPIB	<---	TBP	-0.006393	-0.0056132
[10279]	TSHR	--->	GNAI3	0.65587	-0.65379
[10280]	SMAD3	<---	DAB2	-0.30255	0.38606
[10281]	MYST2	<---	PACSIN1	-0.091209	0.10524
[10282]	MUC1	--->	APC	0.0082812	-0.021954
[10283]	PRKCB	--->	RASGRP3	1.0021	-1.004
[10284]	NR1I2	?	RXRB	-0.0089162	-0.0089162
[10285]	PIAS1	--->	TP53	0.17386	-0.17571
[10286]	SMAD3	<---	PIAS3	-0.98478	1.0161
[10287]	TRPV6	<---	CALM1	-0.060397	0.076798
[10288]	NCOR2	--->	NR3C1	0.13736	-0.14734
[10289]	DLG1	--->	ATP2B4	0.12627	-0.13697
[10290]	SRY	--->	SLC9A3R2	0.19512	-0.15926
[10291]	CASP10	--->	CASP8	1.0068	-0.99651
[10292]	PRKACA	--->	SLC2A2	1.0445	-1.078
[10293]	SPTB	--->	EPB41	0.0012181	-0.0038625
[10294]	GNA13	<---	RGS16	-1.0015	1.0012
[10295]	ITGAV	--->	MMP2	-0.0041766	-0.007497
[10296]	SRC	--->	MPZL1	1.0651	-1.0576
[10297]	REL	<---	NFKBIB	-0.16049	0.15087
[10298]	MARK4	--->	YWHAZ	0.047165	-0.061733
[10299]	MTA1	<---	RBBP4	-0.011108	0.035218
[10300]	WAS	<---	ARHGEF7	-0.088384	0.068447
[10301]	NEK3	--->	VAV1	0.064184	-0.26169
[10302]	STAT5A	<---	IL2RG	-0.17525	0.14932
[10303]	NCK1	--->	DRD4	0.11749	-0.12156
[10304]	PIK3R3	<---	TEC	-0.17828	0.33474
[10305]	USP9Y	--->	MAPT	0.31543	-0.32032
[10306]	PAX5	--->	ETS1	0.010125	0.0010023
[10307]	RFX4	?	RFX2	-0.0064988	-0.0064988
[10308]	PIK3CA	<---	LCK	-0.19173	0.19312
[10309]	VSNL1	--->	GFI1B	0.1063	-0.10982
[10310]	TP53BP1	--->	BRCA1	0.015603	-0.0033089
[10311]	PAK2	<---	CDC42	-1.0053	0.99704
[10312]	ANAPC2	--->	CDC20	0.21762	-0.18583
[10313]	CAMK2G	--->	ACTN2	-0.0067734	-0.0078992
[10314]	TIE1	<---	PTPN11	-0.015615	0.0059345
[10315]	RHOXF2	--->	ZBTB32	0.21927	-0.22229
[10316]	RIPK2	--->	UBE2I	0.24905	-0.27066
[10317]	QKI	<---	RBM9	-0.097474	0.10067
[10318]	RIPK2	<---	BIRC2	0.041459	0.21483
[10319]	NCOR1	--->	ESR2	0.063164	-0.065644
[10320]	GNA13	<---	GPR17	-0.98627	0.9872
[10321]	PRKACA	--->	GP1BB	0.63909	-0.66131
[10322]	OTX2	--->	ATXN1	0.0091738	-0.012951
[10323]	UBE2I	--->	APEX1	0.21073	-0.17742
[10324]	PRKACA	--->	CACNB2	0.72893	-0.75441
[10325]	SH2D3A	<---	EGFR	-0.63942	0.60849
[10326]	LHX2	<---	RLIM	-0.061039	0.057332
[10327]	MKNK1	--->	EIF4E	0.72573	-0.75265
[10328]	ODF2	--->	ODF1	0.031337	-0.0075842
[10329]	PJA2	<---	UBE2D2	-0.063637	0.065039
[10330]	CTNNBIP1	--->	CTNNB1	0.999	-0.99527
[10331]	KCNMA1	<---	KCNMB1	0.0065032	0.027875
[10332]	MYOG	?	ID2	-0.65635	-0.65635
[10333]	CSNK2A1	--->	RANGAP1	0.22517	-0.25741
[10334]	PTPRH	--->	MAPK1	0.67215	-0.67274

```
[10335] ACTR1A  --->    SPTBN1  0.065801        -0.068112
[10336] KLK3    --->    EPOR    0.032493        -0.020557
[10337] SSTR2   --->    GNA14   0.9872  -0.98627
[10338] STARD13 --->    BAI2    0.16815 -0.1544
[10339] WEE1    <---    FBXW11  -0.38147        0.36207
[10340] PXN     <---    PAK3    -0.2934 0.45862
[10341] ESR1    --->    JUND    0.24409 -0.27062
[10342] TNFSF11 <---    MAPK8   -0.29485        0.27854
[10343] TBXA2R  --->    GNA11   1.0129  -1.008
[10344] YWHAZ   <---    NFATC2  -0.1691 0.16254
[10345] PIK3R2  --->    PIK3CD  0.062757        -0.081967
[10346] EIF2AK2 <---    PDGFRB  -0.013343       0.0048431
[10347] CDK5    --->    PPP1R1B 1.0061  -1.0155
[10348] MMP1    <---    CCL2    -0.16713        0.18806
[10349] FGFR2   --->    PLCG1   0.20707 -0.25132
[10350] PRKCD   --->    PTPN22  0.25179 -0.25663
[10351] HERC6   <--->   HERC4   0.080858        0.080858
[10352] TRAF6   --->    STRADB  0.98609 -1.0149
[10353] SLC9A3R1        --->    PLCB2   0.33907 -0.33561
[10354] SRC     --->    GTF2I   1.0671  -0.94248
[10355] ZBTB44  --->    SMAD1   0.0033944       -0.019569
[10356] ILK     <---    CASP8   -0.18683        0.12085
[10357] SRC     --->    FYB     0.73309 -0.73407
[10358] SYT9    <---    CSNK1A1 -0.31969        0.36877
[10359] RASSF1  --->    MAP1B   0.078219        -0.060863
[10360] PPP5C   --->    MAP3K5  0.092599        0.050577
[10361] ZIC2    <---    GLI3    -0.013552       0.33841
[10362] HES6    <---    TLE1    -0.29246        0.30931
[10363] PRKCA   --->    TRPC3   0.044618        -0.057753
[10364] ITGB2   <---    CYTH1   -0.23928        0.2297
[10365] SLCO3A1 --->    SLC9A3R1        0.07475 -0.038024
[10366] HNF4A   <---    SIRT1   -0.0094487      -0.0005598
[10367] DLAT    <---    PDK2    -0.31137        0.32371
[10368] RERE    --->    TRIM22  0.030253        -0.022961
[10369] FLNB    <---    GP1BA   -0.056051       0.058034
[10370] CBL     <---    ITCH    -0.028146       0.014801
[10371] ATN1    --->    RERE    0.0098458       0.0080363
[10372] TIRAP   --->    MYD88   0.029196        -0.053807
[10373] NCOR2   --->    MYOD1   0.26995 -0.27851
[10374] LGALS3BP        <---    PPIC    -0.12021        0.16735
[10375] LCK     --->    SH3BP2  0.85163 -0.59275
[10376] TMCO7   --->    MACF1   0.15387 -0.14861
[10377] SLC9A3R1        --->    MSN     0.025199        -0.032712
[10378] UHMK1   --->    STMN1   1.0058  -0.99021
[10379] PTPN11  --->    TEK     0.014995        -0.021985
[10380] SMARCA4 --->    CCNE1   0.084436        -0.094712
[10381] VDR     <---    FGR     -0.57087        0.66681
[10382] GFI1B   <---    JUB     -0.15855        0.15476
[10383] CTNNB1  --->    CDH3    0.32855 -0.32695
[10384] BRD8    --->    RXRA    0.066907        -0.27692
[10385] MAPK12  --->    SNTB1   0.14066 -0.26313
[10386] ITGA1   <---    ITGB1   -0.0090115      -0.007585
[10387] GAPDH   --->    SERPINB9        0.068981        -0.052543
[10388] CSK     <--->   HCK     0.37153 0.37153
[10389] EGFR    --->    CAV1    0.27464 -0.28119
[10390] SPEN    --->    DLX5    0.059243        -0.046733
[10391] CAMK2B  <--->   CAMK2D  0.12964 0.12964
[10392] EEF1G   <---    CHFR    -0.10543        0.12022
[10393] NPHS1   <---    SRC     -0.087433       -0.056409
[10394] GNB2L1  <---    NFATC1  -0.21566        0.21462
[10395] EP300   --->    SOX9    0.013199        -0.026396
[10396] CAV1    <---    PTPN6   -0.62585        0.80079
[10397] SMAD1   <---    SOX5    -0.075374       0.055924
[10398] PXN     <---    RASA1   -0.22071        0.12419
[10399] PPP1R13B        <---    PPP1CA  -0.19228        0.17963
[10400] FBXL2   <---    SKP1    -0.002664       0.0042561
[10401] RAD23A  <--->   UBL7    0.069208        0.069208
[10402] ZFPM2   <---    CTBP1   -0.063447       0.05497
[10403] CCL14   <---    CCR5    -0.46475        0.49595
[10404] PPARGC1B        --->    ESR1    0.006502        -0.01266
[10405] TLE1    <---    BTBD2   -0.015956       0.018843
[10406] COPB2   <---    PRKCE   -0.70164        0.63388
[10407] F2      <---    HGFAC   -0.0096131      -0.0087417
[10408] WIPF3   <---    VSTM2L  0.0067478       0.017734
[10409] PPM1B   --->    IKBKG   0.61004 -0.61123
[10410] GRAP2   --->    LAT     0.15259 -0.11406
```

[10411] TRIM63 ---> ENO3 0.090943 -0.076137
[10412] PIK3R1 <--- SOCS7 -0.3011 0.19561
[10413] MACF1 <--- CNTNAP2 -0.15471 0.15993
[10414] RIPK1 <--- MAP3K3 -0.16384 0.12036
[10415] CMA1 <--- EDN3 0.015296 0.018665
[10416] NEDD9 ---> TRIP6 0.032788 -0.036363
[10417] BCAR1 <--- LCK -0.37364 0.35403
[10418] PAK7 ---> FHL3 0.12182 0.050059
[10419] NCOR1 ---> PPARA 0.063164 -0.065644
[10420] HSPA8 ---> YWHAQ 0.77111 -0.739
[10421] FBXO30 ---> SMAD1 0.30661 -0.32523
[10422] IGF2 ---> IGFBP5 0.11742 -0.083423
[10423] PDK1 ---> PDHA1 1.0004 -0.99606
[10424] RASD2 ---> SMAD4 0.55322 -0.47159
[10425] SOS1 <--- FYN -0.71705 0.76941
[10426] TGFA ---> NKD2 0.22973 -0.20753
[10427] NEK1 ---> MRE11A 0.6091 -0.63479
[10428] WASL <--- WIPF2 -0.12843 -0.0086954
[10429] ERN1 ---> TNFRSF1A 0.14172 -0.17368
[10430] AURKB ---> TGFBR1 0.36359 0.02614
[10431] SRC ---> ESR2 0.61826 -0.55261
[10432] PRKAR1A <--- CDK4 -0.33631 0.27068
[10433] CAMKK1 ---> CAMK4 0.83082 -0.43719
[10434] GNA12 <--- F2R -1.008 1.0129
[10435] EGFR ---> HOXC10 0.29657 -0.29791
[10436] HK2 <--- PRKCE -0.41716 0.37066
[10437] HEY2 ---> HES1 -0.025997 -0.15188
[10438] SMAD5 <--- PPIL4 -0.17947 0.16253
[10439] MAP3K8 <--- TRAF6 -1.0149 0.98609
[10440] RNF8 ---> RXRA 0.066656 -0.19994
[10441] GGCX ---> F7 0.054476 -0.050377
[10442] PPP4C ---> DHX38 0.21339 -0.22309
[10443] TCERG1 <--- HSPA1A -0.31176 0.33101
[10444] PDPK1 ---> SGK1 1.0929 -1.1695
[10445] ERBB2IP <--- SHOC2 0.023573 0.023602
[10446] PTPRH ---> PTK2 0.49671 -0.57319
[10447] LRP1 <--- ELANE -0.15665 0.1475
[10448] CAMK2G ---> PEA15 0.47512 -0.45726
[10449] CXCL12 ---> CD4 0.017225 -0.0076379
[10450] BMPR1B <--- BMP6 -1.0007 0.99697
[10451] KAT5 ---> H3F3B 0.15564 -0.12976
[10452] SECISBP2 ---> SMAD9 0.23943 -0.25802
[10453] GRB2 ---> PAK1 0.11327 -0.0073877
[10454] ATM <--- CHEK1 -0.22927 0.21896
[10455] PCM1 ---> CEP72 0.011224 0.00683
[10456] YWHAG <---> SFN 0.069023 0.069023
[10457] YWHAZ ---> PARD6B 0.018815 -0.024432
[10458] KCNJ11 ---> KCNJ8 -0.0056346 -0.035054
[10459] SORBS1 ---> INSR 0.068467 -0.079456
[10460] BCAR1 <--- HCK -1.0003 1.0002
[10461] NEK6 <---> CDK7 0.23429 0.23429
[10462] FGF1 <--- CSNK2A1 -0.75902 0.73258
[10463] IGF1R <--- EHD1 -0.020585 0.0083727
[10464] DNM2 <--- CTTN -0.066084 0.061861
[10465] COL7A1 <--- HSPA8 -0.038319 0.027042
[10466] TGFB1I1 <--- TGFBR2 -0.33465 0.33508
[10467] DLG4 <--- PTK2B -0.067529 -0.03771
[10468] UBE2Z <--- TGFBR1 -0.33487 0.30085
[10469] PTPRD <--- PTPRE 0.17316 0.18723
[10470] F10 ---> F3 0.15223 -0.16315
[10471] CD2AP <--- YES1 -0.44444 0.74787
[10472] MAP3K14 ---> NFKB2 0.07198 -0.092566
[10473] TNFRSF10A ---> TNFRSF10D 0.24159 -0.034841
[10474] DOK5 <--- RET -0.50436 0.48844
[10475] MYOG <--- CALM1 -0.22666 0.2676
[10476] FADD ---> CFLAR 1.0007 -1.0021
[10477] NCOA1 <--- SRF -0.060221 0.051041
[10478] CCL3L1 <--- CCR5 -0.46475 0.49595
[10479] BGN <--- COL1A2 -0.030712 0.023329
[10480] SPOCK3 ---> MMP14 0.25303 -0.23217
[10481] CMA1 <--- EDN1 0.015296 0.018665
[10482] EPHA4 <--- FYN -0.0073266 0.4779
[10483] PRKAR1A <--- EGFR -0.18864 0.18685
[10484] HDAC4 ---> CBX5 0.02893 0.0052106
[10485] PRKCA ---> ADRBK1 0.23691 0.014495
[10486] POU2F2 ---> NR3C1 0.012582 0.012013

[10487] SIGLEC1 ---> MUC1 0.089523 -0.10837
[10488] MAG ---> COL9A3 0.016703 -0.028685
[10489] SMAD4 <--- RALA -0.47159 0.55322
[10490] SMURF1 ---> ARHGEF9 0.26456 -0.27556
[10491] PRKAR2B ---> LASP1 0.13443 -0.12098
[10492] CDK5 ---> APP 0.38256 -0.39431
[10493] PRKCA ---> GJA1 0.22576 -0.23456
[10494] PAX3 ---> CIB1 0.075322 -0.057947
[10495] MAP3K14 <--- TRAF6 -1.0149 0.98609
[10496] ARHGAP10 <--- PAK2 -0.016889 0.17878
[10497] RPS6KB1 ---> POLDIP3 0.32452 -0.54666
[10498] SRF ---> NKX2-5 0.13352 -0.11346
[10499] SIRT1 ---> TP53 1.0032 -0.99659
[10500] ZNF446 ---> ZNF232 -0.011024 -0.16454
[10501] RNF8 <--- UBE2E3 -0.10861 0.11055
[10502] MCL1 <--- TNKS -0.1718 0.18284
[10503] CASP7 ---> RAC1 0.36319 -0.47633
[10504] EPS8 <--- PCM1 -0.15482 0.071451
[10505] NKX2-1 <--- CALR -0.013817 0.031904
[10506] ACTA2 <--- EP300 -0.13205 0.12357
[10507] CHD3 <--- PIAS4 -0.015662 0.0038179
[10508] HTR1D <---> HTR1A 0.49511 0.49511
[10509] NLRP1 ? NOD1 -0.009482 -0.009482
[10510] PAK1 ---> TGFBR1 0.49924 -0.30837
[10511] EPOR <--- CBL -0.31313 0.30369
[10512] NCOA1 <--- ESR2 -0.043163 0.034596
[10513] PXN <--- CRKL -0.25245 0.21073
[10514] SGSM2 ---> KHDRBS3 0.030126 -0.033127
[10515] IL16 ---> GRIN2D 0.11324 -0.11391
[10516] SWAP70 ---> CELSR3 0.052921 -0.061952
[10517] FN1 <--- COL1A2 -0.081266 0.074357
[10518] IL2RG <--- CAPNS1 -0.084798 0.069543
[10519] CPNE4 <---> CPNE1 0.058592 0.058592
[10520] UBE2D3 ---> BRCA1 0.1593 -0.15644
[10521] ERBB4 <--- HBEGF -1.0115 0.99285
[10522] PAPPA ---> SMAD9 0.29554 -0.31419
[10523] JAK2 ---> AGTR1 0.17269 -0.0085464
[10524] INS ---> INSR 0.99535 -1.0034
[10525] C1orf94 <--- RBPMS -0.03797 0.070213
[10526] ITGB8 ---> TGFB1 0.10411 -0.16924
[10527] RYBP <--- CASP10 -0.24517 0.19699
[10528] YWHAQ ---> CDC25B 0.14141 -0.11975
[10529] TGS1 <--- CREBBP -0.11628 0.10816
[10530] STAT5B <--- CXCR4 -0.54929 0.53162
[10531] NCR1 ---> CD247 0.29882 -0.30643
[10532] PECAM1 ---> PLCG1 0.69467 -0.76153
[10533] GRN ---> HK3 0.0085719 0.0065101
[10534] ANGPTL3 ---> ITGAV 0.019331 -0.030295
[10535] GFRA4 ---> PSPN 0.39482 -0.35487
[10536] STRN3 <--- PPP2R2A -0.10264 0.11334
[10537] CCR3 ---> CCL11 0.49595 -0.46475
[10538] TERT <--- MTOR -0.028373 0.016009
[10539] PIK3CB <--- YWHAQ -0.48448 0.48299
[10540] PTPN6 ---> KIR2DL3 0.29453 -0.17152
[10541] HERC5 ---> CCNA2 0.47579 -0.47291
[10542] E2F5 ---> RBL1 0.27168 -0.2819
[10543] TRAF2 <--- NGFR -0.99906 1.001
[10544] TCF3 <--- CALM3 -0.2977 0.29593
[10545] PDGFRB ---> SOCS1 0.059585 -0.076185
[10546] DNM1 <--- GRB2 -0.17195 0.17923
[10547] KAT5 ---> E2F1 0.02043 -0.017424
[10548] TSHR ---> LHCGR 0.31383 0.28298
[10549] CCNB3 <--- CDK2 -0.42361 0.4093
[10550] PPP1CB <--- ZFYVE9 -0.0065953 -0.0064486
[10551] SMARCA4 ---> ESR1 0.0094759 -0.018029
[10552] BMPR1B <--- SMAD7 -1.01 1.0109
[10553] CCR5 <--- STAT1 -0.2398 0.11627
[10554] PTK2 ---> ACTN1 0.0035192 -0.050849
[10555] KDR ---> GNA11 0.67347 -0.61887
[10556] APEX2 <--- NINL -0.37056 0.40406
[10557] CTNND2 <--- ERBB2IP -0.065208 0.066333
[10558] RGS12 ---> GNAI3 -0.00079669 -0.0029535
[10559] SMARCA2 <--- BRCA1 -0.0071803 -0.0043439
[10560] FBXW2 <--- CUL1 -0.036054 0.036998
[10561] YWHAH <--- MAP3K3 -0.30237 0.54958
[10562] PDGFRA ---> PLCG1 0.14737 -0.16861

[10563]	MAPK10	--->	GTF3C1	0.44094	-0.56589
[10564]	PBX1	?	HOXD4	-0.11417	-0.11417
[10565]	FOXS1	--->	RBPMS	0.33556	-0.30711
[10566]	CBL	--->	PIK3R1	0.12788	-0.15408
[10567]	AMIGO3	--->	AMIGO2	-0.0074271	-0.030794
[10568]	SQSTM1	?	NBR1	-0.0033302	-0.0033302
[10569]	F11	--->	HGF	0.0012258	-0.0091243
[10570]	SPIB	--->	JUN	0.3457	-0.38611
[10571]	DDX17	--->	NCOA1	0.015459	-0.027033
[10572]	PLN	<---	DMPK	-0.98691	1.0045
[10573]	CSNK1E	--->	APC	0.98885	-0.99835
[10574]	GRIK5	<---	GRIK3	-0.080356	-0.020824
[10575]	SERPINA5	<---	THBD	-0.015828	0.033127
[10576]	DTX2	--->	HPCAL4	0.12358	-0.12829
[10577]	SSTR5	<---	DRD2	0.24405	0.31807
[10578]	ITGB5	<---	PALLD	-0.067748	0.064443
[10579]	ATN1	--->	FBLN5	0.025006	-0.031741
[10580]	DUSP19	--->	MAP2K7	1.011	-0.99819
[10581]	GIT1	<---	GRK6	-0.20018	0.1705
[10582]	COPS6	<---	STK40	-0.49734	0.46374
[10583]	CDK5	<---	MAP2K1	-0.99672	1.0159
[10584]	AKT1	--->	MST1R	0.1934	-0.10784
[10585]	SP1	--->	NCOR1	0.00023142	-0.013374
[10586]	CREB1	<---	DYRK1A	-0.58596	0.58797
[10587]	BNIP2	<---	ARHGAP1	-0.0078982	0.017753
[10588]	FAM46A	--->	HDLBP	0.043326	-0.0093144
[10589]	RAC1	--->	PRKCI	0.99149	-1.0135
[10590]	RPS6KB1	<---	CDC42	-0.56975	0.3588
[10591]	PRKCA	--->	RGS19	0.87438	-0.87353
[10592]	NEK6	--->	TGFBR1	0.36359	0.02614
[10593]	UBQLN4	--->	SERPINH1	0.17983	-0.14362
[10594]	SERPINE2	<---	F2	-0.040728	0.057207
[10595]	JDP2	--->	JUN	0.050278	-0.31682
[10596]	PTEN	<---	COPS6	-0.33629	0.35424
[10597]	DLG2	--->	FZD7	0.068598	-0.085061
[10598]	NFKB1	<---	CHUK	-1.0129	1.001
[10599]	MBD1	<---	PIAS2	-0.088505	0.090856
[10600]	BCAN	--->	MMP3	0.022421	-0.030194
[10601]	TP63	<---	HNRNPAB	-0.056911	0.073436
[10602]	MBD1	--->	SMAD7	0.23479	-0.18953
[10603]	RELA	--->	NFKBIE	0.01764	-0.0125
[10604]	BCL2L1	<---	IRS1	-0.54713	0.54465
[10605]	CHD3	<---	CTTN	-0.0070742	-0.0026616
[10606]	LHCGR	--->	GNAI2	1.0129	-1.008
[10607]	BMPR1B	--->	SOCS6	0.15875	-0.19559
[10608]	TLR2	--->	HSP90B1	0.15741	-0.16352
[10609]	DUSP1	--->	MAPK14	0.99169	-0.98663
[10610]	PLK3	<---	MAP2K1	-0.69003	0.64519
[10611]	ATF2	--->	AR	0.054477	-0.066829
[10612]	SP1	--->	RBL1	0.068157	-0.081795
[10613]	CASP7	--->	SRF	0.32345	-0.39257
[10614]	NR0B1	--->	NR5A1	-0.0089663	-0.044425
[10615]	CDK9	--->	CUL1	0.30021	-0.32466
[10616]	DLST	--->	ZBTB16	0.11542	-0.11691
[10617]	KCNQ3	--->	CALM3	0.32976	-0.29233
[10618]	REPS1	<---	GRB2	-0.294	0.30689
[10619]	SRC	--->	SH2D3C	0.9938	-1.007
[10620]	CBL	--->	STAT5B	0.16839	-0.18275
[10621]	PTPN6	--->	VAV1	0.38489	-0.38383
[10622]	TLR2	--->	SFTPA1	0.17109	-0.17742
[10623]	LOXL4	<---	COL2A1	-0.056949	0.050853
[10624]	BMPR1B	<---	BMP2	-1.0007	0.99697
[10625]	SOX11	--->	POU3F2	0.13789	-0.13218
[10626]	CALM1	<---	GRM3	-0.27991	0.31782
[10627]	FHL3	--->	LNX1	0.018556	-0.00047163
[10628]	CDK1	--->	ECT2	0.67984	-0.70437
[10629]	CTSD	--->	IGFBP4	0.41796	-0.40802
[10630]	CFLAR	--->	PSEN2	0.21074	-0.25908
[10631]	TICAM1	<---	TRAF6	-0.39629	0.38031
[10632]	PRKACA	--->	IFNAR1	1.1418	-1.1788
[10633]	PTPRO	--->	PIM1	0.67215	-0.67274
[10634]	LAT	<---	SYK	-1.0126	0.99842
[10635]	IMPDH2	--->	UBQLN4	0.68725	-0.67523
[10636]	LRP2	<---	DLG3	-0.16223	0.15231
[10637]	E2F3	--->	TFDP1	-0.0088085	-0.064857
[10638]	RYBP	--->	RING1	0.065528	-0.030268

[10639]	PRTN3	<---	F2R	-0.42368	0.45462
[10640]	PRKACA	--->	KCNH2	0.18176	-0.22705
[10641]	AP3B1	<---	BUB1B	-0.33636	0.27287
[10642]	CAV1	<---	PTPN1	-1.2764	1.2767
[10643]	NEDD4	--->	SH3KBP1	0.17897	-0.1883
[10644]	LMO1	--->	GATA3	0.18194	-0.13822
[10645]	PITX1	<---	POU1F1	-0.19656	-0.024292
[10646]	FHL5	<---	SRF	-0.037159	0.05921
[10647]	AKT1	--->	APPL1	0.46359	-0.31639
[10648]	TCERG1	--->	PCBP1	0.20566	-0.19132
[10649]	BMPR1B	<---	RASD2	-0.53994	0.52577
[10650]	LCK	--->	DEF6	0.71858	-0.52901
[10651]	TCF7L2	<---	JUP	-0.021804	0.00097213
[10652]	BAD	<---	RPS6KA3	-0.91274	0.58958
[10653]	PTPRC	--->	ITGAL	0.083879	-0.095687
[10654]	BAIAP2	<---	RAC1	-0.13661	0.13835
[10655]	PPP2R2A	<---	PPP2R1B	-0.17841	0.18879
[10656]	MAGI2	<---	ADRB1	-0.089062	0.082775
[10657]	SYNJ2BP	<---	ACVR2A	-0.17933	0.15108
[10658]	INPP5D	<---	PECAM1	-0.63997	0.62918
[10659]	MAP3K7	--->	XIAP	0.10998	-0.11249
[10660]	ACTL6B	<---	CTBP1	-0.16693	0.15924
[10661]	ATM	<---	BRCA1	-0.14033	0.12872
[10662]	GRIN1	<---	EPHB2	-0.20791	0.19789
[10663]	TAF1	--->	GFI1B	0.05345	-0.063751
[10664]	ARL4D	<---	TGFBR1	-0.2793	0.24366
[10665]	CDK9	<---	NFKB1	-0.6564	0.6581
[10666]	LIMK1	--->	YWHAZ	0.24577	-0.26263
[10667]	NR1I2	--->	NUCB2	0.15612	-0.12827
[10668]	CD3E	--->	PIK3R1	0.12264	-0.23288
[10669]	CTRC	--->	SERPINA3	0.11833	-0.082185
[10670]	CAV3	--->	SLC22A11	0.20823	-0.20149
[10671]	MCL1	?	BAX	-0.63915	-0.63915
[10672]	PFN2	<---	HTT	-0.059658	0.092438
[10673]	GRK6	--->	ADRB2	0.21152	-0.27051
[10674]	PTEN	<---	CAV1	-0.19071	0.20955
[10675]	CDK7	--->	THRA	0.99834	-1.0079
[10676]	PER3	--->	ARNTL	0.074763	-0.087389
[10677]	MAPK1	<---	MAP2K1	-0.99672	1.0159
[10678]	CBL	--->	YWHAQ	0.48957	-0.49673
[10679]	GRIN2B	<---	CAMK2A	-0.30134	0.28954
[10680]	SVIL	<---	CTBP2	-0.033177	0.035377
[10681]	PTPN1	--->	JAK2	1.0128	-1.0014
[10682]	CPNE4	<---	UBE2M	-0.12637	0.16628
[10683]	HIPK2	--->	NKX2-5	0.79979	-0.82765
[10684]	SLC2A4	--->	ACOX1	0.14967	-0.15287
[10685]	TGFBRAP1	<---	ACVRL1	-0.067656	0.0083125
[10686]	C3AR1	--->	C3	0.16026	-0.15448
[10687]	SHC1	<---	MET	-0.5154	0.23511
[10688]	SMAD2	<---	SQSTM1	-0.22445	0.26503
[10689]	CELA1	--->	SERPINA3	0.11833	-0.082185
[10690]	CD79A	<---	LCK	-0.99051	0.99528
[10691]	SREBF1	<---	ESR2	-0.18581	0.18403
[10692]	JUN	<---	BCL3	-0.22507	0.19428
[10693]	SKAP1	<---	SRC	-0.78336	0.77338
[10694]	LINGO1	--->	RTN4R	0.043324	0.0076592
[10695]	PHC2	--->	MCRS1	0.23972	-0.20165
[10696]	DSTN	--->	SMAD9	0.15741	-0.17426
[10697]	PRKCZ	--->	NCF1	0.14477	-0.15995
[10698]	TRAF4	--->	TBC1D8	0.15184	-0.1427
[10699]	MECR	--->	PPARA	0.074075	-0.048313
[10700]	CD40	<---	HSPA8	-0.68286	0.67874
[10701]	PAK1	--->	FOXL2	0.70562	-0.42214
[10702]	KIAA0182	--->	RBPMS	0.20038	-0.16333
[10703]	CDK11B	--->	MYST2	0.69541	-0.72127
[10704]	RASA3	--->	RAP1A	1.0058	-0.99979
[10705]	CRHR1	<---	GNAO1	-0.43656	0.4334
[10706]	UBE2I	--->	PTEN	0.33126	-0.30891
[10707]	PPP1R13L	<---	PPP1CA	-0.19398	0.18385
[10708]	DYRK1A	--->	YWHAE	0.39808	-0.42581
[10709]	MAPK1	--->	SMAD4	1.012	-1.0074
[10710]	ZAP70	<---	FYN	-1.0044	1.0025
[10711]	PPP1CA	--->	AURKA	0.046927	-0.067136
[10712]	SPEN	<---	NCOR2	-0.14346	0.13249
[10713]	CD247	<---	CSK	-0.98519	0.99791
[10714]	TLK1	<--->	TLK2	0.23429	0.23429

[10715]	ZFYVE16	--->	TOM1	0.050181	-0.037021
[10716]	MAP3K4	<---	PTPN11	-0.11638	0.10708
[10717]	RGS16	--->	GNAI2	1.0012	-1.0015
[10718]	TRIM23	--->	GGA2	0.30356	-0.29291
[10719]	TRPV4	<---	FYN	-0.29213	0.34238
[10720]	SDCBP	--->	RAB5A	0.098964	-0.08204
[10721]	MAPK11	<---	MAP3K7IP1	-0.062916	-0.052553
[10722]	WASF1	<---	ABI1	-0.074825	0.060813
[10723]	BARD1	<---	CSTF1	-0.076079	0.093614
[10724]	DYRK2	--->	RAD54B	0.42211	-0.44328
[10725]	LGALS3BP	<---	CD14	-0.086839	0.1768
[10726]	DNAJC1	<---	ITIH4	-0.03856	0.042186
[10727]	NKTR	<---	RNPS1	0.0067944	0.026806
[10728]	NRXN1	<---	SYT5	-0.043035	0.083467
[10729]	BNIPL	<---	ARHGAP1	-0.0078982	0.017753
[10730]	CD226	--->	PVR	0.038911	-0.066096
[10731]	TRIM24	<---	HNF4A	-0.29042	0.29942
[10732]	PPP2R1B	--->	STRN	0.25641	-0.2507
[10733]	RORB	<---	UBE2I	-0.16643	0.1925
[10734]	PTPN6	--->	KLRC1	0.97265	-0.80298
[10735]	ETV6	<---	SMURF1	-0.28226	0.27249
[10736]	EIF3G	--->	EPB41	0.029365	-0.025865
[10737]	SMAD1	<---	SNRNP70	-0.30655	0.28646
[10738]	PAK1	--->	ARPC1B	0.69245	-0.41336
[10739]	PVRL1	--->	PARD3	0.042062	-0.053929
[10740]	MAP2K5	<---	MAP3K3	-1.0008	0.99307
[10741]	PTPN1	--->	ACTN1	0.46815	-0.47383
[10742]	PPM1B	<---	IKBKB	-0.15493	0.026748
[10743]	PAX6	<---	TBP	-0.0072121	-0.0057029
[10744]	ANKRD2	--->	TP53	0.14916	-0.13817
[10745]	PRKDC	--->	TP53	0.12799	-0.13244
[10746]	TRAF6	--->	SYK	0.39399	-0.3971
[10747]	SMAD2	<---	CYP11A1	-0.32966	0.41257
[10748]	HDAC3	--->	RUNX2	0.075349	-0.040912
[10749]	RBPMS	<---	C12orf12	0.01137	0.020254
[10750]	DUSP3	--->	MAP2K2	0.9172	-0.50725
[10751]	IRAK4	<---	TRAF6	-0.36381	0.3618
[10752]	HNRNPK	--->	QKI	-0.0053675	-0.11083
[10753]	HDAC1	--->	SUV39H1	0.034642	-0.035506
[10754]	FGFR4	<---	FGF6	-0.99871	1.0053
[10755]	ELANE	<---	VCAM1	-0.20793	0.20333
[10756]	CAMKK2	--->	CALM1	1.0175	-0.89542
[10757]	HIST2H3C	<---	RBBP4	-0.059328	0.088873
[10758]	SETDB1	<---	BHLHE40	-0.0040373	0.0087585
[10759]	PTPN6	--->	ESR1	0.52288	-0.40649
[10760]	WIPF1	<---	NCK1	-0.1072	0.096445
[10761]	JDP2	--->	JUND	0.050278	-0.31682
[10762]	IL23R	--->	IL12RB1	0.059484	-0.030751
[10763]	LCP2	--->	VAV1	0.33724	-0.32689
[10764]	PTPN14	--->	TJP1	0.10178	-0.11313
[10765]	CLK2	<---	PTPN1	-1.009	1.0061
[10766]	RIMS2	<---	RAB3B	-0.0067445	0.016184
[10767]	CCR1	--->	CCL4	0.49595	-0.46475
[10768]	MAGI2	--->	SMAD3	0.13218	-0.12255
[10769]	NFATC2	--->	YWHAQ	0.16254	-0.1691
[10770]	PLCL2	<---	PPP1CA	-0.10551	0.096638
[10771]	GAK	--->	CCNG1	0.26393	-0.2828
[10772]	MAPK14	--->	JDP2	0.5412	-0.63148
[10773]	STK11	--->	MARK2	0.024641	0.014931
[10774]	TLX1	--->	PKNOX1	0.024726	-0.0094151
[10775]	DLGAP2	--->	NOS1	0.27365	-0.28327
[10776]	PPID	<---	SMURF2	-0.71645	0.71369
[10777]	OPRM1	<---	OPRD1	0.24405	0.31807
[10778]	PAPPA	--->	IGFBP4	0.07239	-0.038431
[10779]	DRD3	<---	NCK1	-0.16206	0.16469
[10780]	SRC	--->	STAT6	0.49268	-0.46929
[10781]	SLC22A11	<---	PDZK1	-0.20736	0.21946
[10782]	PRELP	<---	HSPG2	-0.015837	0.003344
[10783]	YWHAG	<---	SRC	-0.89808	1.0093
[10784]	VWF	--->	CALR	0.053425	-0.049138
[10785]	MMP1	<---	IGFBP3	-0.15977	0.18055
[10786]	SLCO3A1	--->	SLC9A3R2	0.07475	-0.038024
[10787]	UBE2I	--->	TP53	0.43525	-0.42405
[10788]	NLRP4	--->	NLRC4	0.1678	-0.15074
[10789]	PPP2R3A	--->	HMGB1	0.0037064	-0.013236
[10790]	RASD2	--->	SMAD3	0.55322	-0.47159

[10791]	MCRS1	<---	CCDC85B	-0.03795	0.066891
[10792]	MTNR1A	--->	GNAI3	1.0129	-1.008
[10793]	RELA	<---	STAT1	-0.075086	0.037477
[10794]	SYTL2	<---	PPP1CA	-0.12792	0.11901
[10795]	CABLES2	<---	ABL1	-0.097172	-0.052501
[10796]	PIN1	<---	CDK1	-0.49485	0.46953
[10797]	LRP12	--->	GNB2L1	0.10568	-0.072485
[10798]	CREM	<---	CREBBP	-0.056352	0.045008
[10799]	PGR	--->	NCOA1	0.034596	-0.043163
[10800]	STRN4	--->	CALM1	0.21607	-0.1798
[10801]	SIRPA	<---	IL1RAP	-0.41072	0.4289
[10802]	MYF5	--->	TCF3	-0.13552	-0.39218
[10803]	GADD45G	--->	PPARA	0.017932	0.0079001
[10804]	CHAF1B	<---	CDK2	-0.86422	0.83716
[10805]	PRLR	<---	PPIA	-0.01698	0.0056227
[10806]	CLK4	--->	HSPB2	0.7368	-0.67755
[10807]	TP53BP2	<---	YES1	-0.50994	0.80689
[10808]	SERPINB9	<---	PRPF40A	-0.49699	0.53811
[10809]	UBQLN1	--->	PSEN2	0.050774	-0.016623
[10810]	SLC2A4	<---	CTSD	-0.58772	0.59304
[10811]	FGF10	--->	FGFR2	0.54173	-0.53789
[10812]	FURIN	--->	LRP1	0.049899	-0.062083
[10813]	MET	--->	CTTN	0.3106	-0.27091
[10814]	SASH3	--->	SMAD7	0.26998	-0.21835
[10815]	PIK3R1	<---	TEC	-0.3557	0.35771
[10816]	MST1R	--->	MST1	0.2305	-0.24289
[10817]	PLCG2	<---	KDR	-0.46965	0.42937
[10818]	RASD2	<---	SMURF1	-1.0084	1.0007
[10819]	AR	<---	GRIP1	-0.9557	0.95523
[10820]	SFRS17A	<---	CCDC85B	-0.034716	0.063764
[10821]	RNF216	--->	AGFG1	0.028808	0.0050732
[10822]	GNB2	<---	RAF1	-0.082089	0.056081
[10823]	SFN	--->	BAD	0.9978	-1.0088
[10824]	KCNA5	<---	DLG4	-0.049447	0.047736
[10825]	PRKD1	--->	PLCG1	1.0014	-1.0056
[10826]	SUFU	<---	BTRC	-0.39185	0.38391
[10827]	LASS2	<---	ASGR2	-0.0065592	0.03634
[10828]	SRC	--->	TRAF1	0.58415	-0.51787
[10829]	GNB2L1	<---	EPOR	-0.04996	0.061216
[10830]	DAXX	<---	HDAC1	-0.11277	0.090678
[10831]	MMP7	<---	SERPINA1	-0.13073	0.15283
[10832]	CACNA1A	<---	SYT1	-0.18324	0.17685
[10833]	TRPC4	--->	ITPR2	-0.0070644	-0.013012
[10834]	JAK1	--->	GRB2	0.62462	-0.5053
[10835]	PIN1	--->	BCL2	0.94876	-0.94223
[10836]	TTF2	--->	MYB	-0.0044126	-0.0071361
[10837]	PIK3R2	<---	GRB2	-0.40813	0.38647
[10838]	TTC14	<---	CCDC85B	-0.010732	0.012676
[10839]	JUN	--->	BCL6	0.11317	-0.14009
[10840]	HSP90AB1	--->	AKT1	0.55691	-0.53851
[10841]	SPTBN2	--->	ACTA1	0.1445	-0.15167
[10842]	PCBP1	<---	PDLIM7	-0.031014	0.048584
[10843]	RBMX	<---	LNX1	-0.0053675	0.021194
[10844]	LIN7B	<---	APBA1	-0.0085874	0.042863
[10845]	CRK	--->	PTK2	-0.04842	-0.21492
[10846]	MKNK2	--->	ESR1	0.99834	-1.0079
[10847]	ITCH	--->	RNF11	0.2604	-0.27028
[10848]	MBL2	--->	CALR	0.038063	-0.048644
[10849]	NR2C2	<---	HSP90AB1	-0.058028	0.052711
[10850]	IRAK1	<---	TRAF4	-0.10554	0.12381
[10851]	BMPR2	<---	BMP4	-0.99943	1.0057
[10852]	PRKG1	--->	ITPR1	0.11859	-0.13105
[10853]	MAPK3	<---	PRKCD	0.043445	0.65991
[10854]	NCOR2	--->	NR4A1	0.13736	-0.14734
[10855]	IGF1R	--->	JAK2	0.12738	-0.11206
[10856]	NR3C2	<---	PIAS3	-0.090133	0.095399
[10857]	RGS2	--->	SCN5A	0.474	-0.44432
[10858]	NCOA3	<---	IKBKG	-0.1241	0.11093
[10859]	NOV	?	CTGF	-0.071381	-0.071381
[10860]	IL21R	<---	JAK1	-1.0011	1.0065
[10861]	MAPK1	--->	IFNAR1	1.1418	-1.1788
[10862]	PIAS1	--->	SMAD4	1.0161	-0.98478
[10863]	SHB	--->	PLCG1	0.36719	-0.53438
[10864]	GRAP	--->	SOS1	0.60392	-0.61023
[10865]	RPS6KA4	<---	MAPK1	-1.0109	1.002
[10866]	PUF60	--->	FUBP1	0.036464	-0.0048144

[10867]	ESR2	--->	ZBTB17	0.14623	-0.15777
[10868]	SUV39H2	--->	SMAD5	0.078555	-0.092045
[10869]	NCK1	--->	FASLG	0.18483	-0.17959
[10870]	DLGAP1	--->	APC	0.043109	-0.05613
[10871]	BMP1	<---	COL7A1	-0.013155	0.00062382
[10872]	F10	<---	TFPI	-0.0072007	0.0040125
[10873]	YWHAG	<---	PPFIA1	-0.16906	0.19937
[10874]	KIRREL	<---	NPHS1	-0.0044807	-0.00011006
[10875]	GRM7	<---	PICK1	-0.025484	0.0626
[10876]	ERBB2IP	--->	NOD2	0.038958	0.0088436
[10877]	KLK2	--->	AR	0.95469	-0.95529
[10878]	MYBL2	<---	NCOR2	-0.0084055	0.076825
[10879]	ARHGEF6	<---	TGFBR1	-0.06828	0.043961
[10880]	SYK	--->	CD19	1.0127	-1.0065
[10881]	CCDC85B	--->	LNX1	0.036858	-0.022613
[10882]	TGFB3	--->	CTGF	0.18381	-0.19139
[10883]	PDGFRB	--->	STAT5A	0.15757	-0.16879
[10884]	STAT5A	<---	SYK	-0.82452	0.62688
[10885]	IKBKB	--->	HSP90AB1	0.059478	-0.11716
[10886]	MMP9	<---	COL4A1	-0.051594	0.040965
[10887]	PRKCI	<---	RAB2A	-1.0135	0.99149
[10888]	VASN	--->	TGFB3	0.45796	-0.41124
[10889]	PAK3	--->	RAF1	0.65894	-0.56404
[10890]	PTPN11	--->	KLRC1	0.97265	-0.80298
[10891]	TRIM24	--->	NR2F2	0.11471	-0.088819
[10892]	YWHAG	--->	PKP2	0.089709	-0.086535
[10893]	JUNB	--->	FOSB	-0.057338	-0.12398
[10894]	KSR2	--->	RAF1	0.1966	-0.2171
[10895]	IGF2	--->	IGFBP2	0.11742	-0.083423
[10896]	SMAD2	<---	SRI	-0.29841	0.34355
[10897]	CDK14	--->	SEPT8	0.69517	-0.7211
[10898]	DVL3	<---	MYST2	-0.3857	0.38581
[10899]	ADAM15	<---	SORBS3	-0.10904	0.10503
[10900]	AGAP2	--->	EPB41L1	0.12031	-0.13222
[10901]	HIF1A	--->	MDM2	0.18276	-0.19308
[10902]	BTBD2	<---	TRIM5	-0.026097	0.018558
[10903]	FHL2	<---	SRF	-0.037159	0.05921
[10904]	CASP8	--->	RALBP1	0.29066	-0.38381
[10905]	PARD6A	<---	MARK2	-0.082143	0.063239
[10906]	ARHGAP17	--->	PIK3R1	0.002574	-0.17145
[10907]	SP3	<---	SP1	-0.064923	0.089665
[10908]	NFKB2	<---	CHUK	-0.99594	0.99702
[10909]	USP7	<---	TRAF4	-0.0088131	0.14976
[10910]	SUFU	--->	NCOA3	0.11035	-0.1226
[10911]	CNKSR2	--->	DLG4	-0.0026013	-0.020819
[10912]	CASP1	<---	PSEN1	-0.048154	0.056436
[10913]	HIST3H3	<---	CBX3	-0.19484	0.24617
[10914]	OPRD1	--->	GNAI2	1.0129	-1.008
[10915]	IL2RG	<---	JAK1	-0.39107	0.38331
[10916]	MX1	--->	TRPC6	0.072894	-0.085464
[10917]	WEE1	<---	CASP3	-0.98085	0.87492
[10918]	SMAD3	<---	EPAS1	-0.085213	0.075495
[10919]	CUL3	--->	RBX1	0.12225	-0.11034
[10920]	CISH	--->	IL3RA	0.21779	-0.21911
[10921]	EHD2	--->	SLC2A4	0.16529	-0.15501
[10922]	TFEC	--->	TFE3	-0.069637	-0.16359
[10923]	ARPC1B	--->	ACTB	0.4503	-0.44823
[10924]	HDAC3	--->	GTF2I	0.04867	-0.012939
[10925]	GJA5	--->	GJA1	-0.0078479	-0.078897
[10926]	ITGA2B	<---	CALR	-0.031906	0.020231
[10927]	PPP1R9B	<---	ADRA1A	-0.57274	0.58018
[10928]	PIK3R1	--->	CTNNB1	0.0696	-0.086027
[10929]	CCNA2	<---	CDKN1A	-0.99961	0.98767
[10930]	CREBBP	--->	RELA	0.038481	-0.050014
[10931]	NR2C1	?	NR2C2	-0.0089162	-0.0089162
[10932]	SERPINH1	<---	UBTF	-0.26934	0.30473
[10933]	PDPK1	--->	RPS6KA1	1.0929	-1.1695
[10934]	PLCB1	<---	RGS4	-0.61782	0.62068
[10935]	EPB41L1	--->	ITPR1	0.11581	-0.12828
[10936]	PPP1R13L	<---	SP1	-0.59919	0.58044
[10937]	FNBP1	<---	RND2	-0.12591	0.13778
[10938]	IRAK1	--->	MAP3K7	0.99033	-1.0144
[10939]	SMARCC1	--->	CEBPB	0.1557	-0.10593
[10940]	TRIM24	--->	ZNF10	0.20562	-0.21006
[10941]	PTPRJ	--->	IL2RG	0.17996	-0.29789
[10942]	CALCOCO2	<---	CCDC33	-0.045677	0.087747

[10943] ATN1 ---> LYST 0.055796 -0.058195
[10944] NID1 <--- COL1A1 -0.023028 0.0095288
[10945] ATXN1 <--- SIX5 -0.012951 0.0091738
[10946] ADAM22 <--- YWHAB -0.0038527 0.013329
[10947] ACTG1 <--- WASF1 -0.2714 0.26671
[10948] GAB3 <--- GRAP2 -0.33688 0.35049
[10949] SMAD2 <--- ANAPC2 -0.68264 0.75332
[10950] SRF ---> CASP9 0.061802 -0.058599
[10951] MRAS ---> RAF1 1.0008 -0.9994
[10952] ACVR1B <--- INHBA -0.71391 0.72054
[10953] PAX3 <--- MEOX1 -0.099649 -0.043846
[10954] ATXN1 <--- WNK2 -0.12782 0.10564
[10955] BAZ1A ---> SMARCA5 0.0048807 -0.012364
[10956] RALGDS ---> MLLT4 -0.0081416 -0.081548
[10957] RIPK2 <--- TRAF5 -0.13857 0.13062
[10958] RAB3B ---> CALM3 0.73657 -0.70265
[10959] BTK ---> PIK3AP1 0.9964 -1.0013
[10960] SHC1 <--- ALK -0.84376 0.39063
[10961] SATB1 ---> GATA3 0.066181 -0.058645
[10962] HIF1A <--- EGLN1 -0.99517 0.99907
[10963] GRB2 ---> RPS6KA1 -0.002202 -0.1302
[10964] DTX1 ---> GRB2 0.032212 -0.044544
[10965] ATP1B1 <--- BACE1 -0.21605 0.24854
[10966] MAPKAPK2 ---> TCF3 0.62386 -0.63209
[10967] TBC1D10C ---> PPP3R2 0.21092 -0.19365
[10968] CCR4 ---> CCL5 0.45214 -0.43752
[10969] FCHO1 <--- SMURF1 -0.70619 0.70228
[10970] BTK ---> TLR9 0.12466 -0.14446
[10971] NCK2 ---> SOS1 0.37616 -0.38229
[10972] ADCY6 <--- CHRNA7 -0.32985 0.34477
[10973] TCERG1 ---> PIAS4 0.039813 -0.038718
[10974] DCN ---> COL14A1 0.026916 -0.031969
[10975] IKBKB <--- CASP8 -0.66258 0.65864
[10976] PRMT2 ---> ESR1 0.12534 -0.11586
[10977] ITK ---> CD28 0.78153 -0.85158
[10978] MST1R ---> GRB2 0.1807 -0.36229
[10979] SMAD4 ---> SKIL 0.0719 -0.06155
[10980] CREBBP ---> STAT3 0.98672 -1.0013
[10981] HMGB1 ---> DNMT1 0.072272 -0.057726
[10982] ARHGAP15 <--- SMURF1 -0.40937 0.40011
[10983] MAPK3 ---> ATP1A1 0.49725 -0.50785
[10984] MAGI3 <--- NRCAM -0.02371 0.01506
[10985] XCL2 <--- XCR1 -0.46475 0.49595
[10986] RXRA ---> MYOD1 -0.037427 -0.17137
[10987] FGFR4 ---> STAT3 0.13994 -0.19056
[10988] CTNNA1 ---> CDH1 0.33273 -0.3322
[10989] MAP4K4 ---> RASA1 0.00075936 -0.0062044
[10990] MAPK1 ---> LIPE 0.53776 -0.55596
[10991] RIT1 <--- SMURF1 -1.0084 1.0007
[10992] $ZBTB8$ <--- ZNF276 -0.13791 0.15064
[10993] TBK1 ---> NFKBIA 1.0116 -0.99754
[10994] SMAD1 <--- SMARCE1 -0.062155 0.042339
[10995] CASP7 ---> MEF2A 0.17117 -0.20473
[10996] ATP2A2 <--- IRS2 -0.025724 0.013329
[10997] PPP2CA ---> TP53 0.19467 -0.20375
[10998] PTK2 ---> ROCK1 0.0053916 -0.028944
[10999] GSK3A ---> YWHAG 0.26506 -0.31931
[11000] IKBKB <--- PRKCQ -0.40526 0.42813
[11001] MYD88 <--- TLR5 0.020105 0.14052
[11002] DVL3 ---> CTNNB1 0.041299 -0.052812
[11003] MAPK9 ---> TP53 1.0002 -0.99962
[11004] FCAR <--- PPP2CA -0.42938 0.42662
[11005] PTPN6 ---> PILRB 0.60343 -0.52299
[11006] RAPGEF6 ---> RAP1A 1.0006 -0.99699
[11007] RPS6KA3 ---> MAPT 0.35614 -0.49081
[11008] ITSN1 ---> SMARCC2 0.042759 -0.044691
[11009] HABP2 <--- COL4A2 -0.011113 -0.0022521
[11010] IL31RA ---> IL6ST -0.043585 -0.063694
[11011] CFLAR ? CASP10 -0.2897 -0.2897
[11012] LAT ---> PLCG1 0.98913 -0.99214
[11013] RTKN <--- RHOA -0.24337 0.24852
[11014] ZP4 <--- FURIN -0.037698 0.041432
[11015] LRP2 <--- LPA -0.25174 0.24565
[11016] YWHAB <--- PIK3R4 -0.1979 0.17545
[11017] SMN2 ---> TIAL1 0.011379 0.0023256
[11018] ABI3 <--- HOMER1 -0.072036 0.069366

[11019]	BMPR1B	<---	BMPR2	-0.18789	-0.093581
[11020]	AP3B1	<---	ARF6	-0.12845	0.11437
[11021]	CXCL10	--->	VCAN	0.010191	-0.021003
[11022]	FBXW7	--->	PSEN1	0.25446	-0.25253
[11023]	HOXA1	--->	TCF3	0.34775	-0.35072
[11024]	ITGB3	--->	SHC1	0.023499	-0.069532
[11025]	ESR1	<---	FOXO3	-0.26658	0.26449
[11026]	PTPN11	--->	JAK1	1.0117	-0.99645
[11027]	SLC2A4	<---	CFL1	-0.17434	0.18476
[11028]	EP300	--->	TCF3	0.046434	-0.058216
[11029]	PTGFR	<--->	PTGER2	0.49511	0.49511
[11030]	TBL1XR1	<---	GPS2	-0.039544	0.042918
[11031]	CD93	<---	SCYL1	-0.35429	0.33523
[11032]	PDCD6	--->	SH3KBP1	0.18522	-0.17013
[11033]	ADCY8	<---	CALM1	-0.24388	0.26107
[11034]	SRC	--->	HRAS	0.9988	-0.893
[11035]	PLCG2	<---	DAPP1	-0.52159	0.32939
[11036]	AKAP9	<---	PPP2R3A	-0.028584	0.019122
[11037]	KIT	--->	PIK3CG	0.016231	-0.033762
[11038]	PTPN11	--->	CSF2RB	0.99989	-1.0072
[11039]	HDAC1	--->	RELA	0.067213	-0.0691
[11040]	SMAD3	<---	PAPPA	-0.32956	0.41247
[11041]	PRKACA	--->	GYS1	1.0067	-1.0067
[11042]	CCL28	<---	CCR3	-0.46475	0.49595
[11043]	CHN1	<---	TGFBR1	-0.20451	0.17393
[11044]	NFATC2	--->	FOXP3	0.14313	-0.14913
[11045]	TGM2	--->	HTT	0.026939	0.0071118
[11046]	ZYX	--->	ACTN1	0.014972	-0.010035
[11047]	RARA	<---	NCOR2	-0.14734	0.13736
[11048]	NFATC2	--->	EGR1	0.11655	-0.12568
[11049]	PCBP1	<---	RALY	-0.00035796	0.014078
[11050]	FADD	--->	ABCA1	-0.001537	-0.028184
[11051]	KCNJ8	<---	KCNJ2	-0.035054	-0.0056346
[11052]	NEK3	--->	PRLR	0.040782	-0.055222
[11053]	UBE2V2	--->	SUV39H2	0.11822	-0.11108
[11054]	SYTL4	<---	INS	0.020154	0.02021
[11055]	FOXH1	--->	SMAD4	0.52503	-0.44328
[11056]	SVIL	--->	TRIP6	0.071071	-0.012903
[11057]	MYO3A	--->	TGFBR1	0.21777	0.013124
[11058]	TRAF6	--->	CD40	0.35782	-0.3718
[11059]	PDGFB	<---	COL1A1	-0.013521	0.0033105
[11060]	CCND2	<---	CDK4	-1.0774	1.0544
[11061]	CNTROB	--->	LYST	0.16264	-0.17284
[11062]	NFKB1	--->	NR3C1	0.47113	-0.47154
[11063]	SMAD2	--->	DVL1	0.09242	-0.090479
[11064]	ZBTB16	<---	TOLLIP	-0.037395	0.028142
[11065]	NFKBIA	<---	NFKBIB	-0.85341	-0.23461
[11066]	PRKCZ	<---	WWC1	-0.03202	0.018488
[11067]	TNXB	--->	VEGFB	0.042102	-0.052923
[11068]	ARHGAP27	--->	SH3KBP1	0.17653	-0.1768
[11069]	NR2C1	--->	AR	-0.0031537	-0.58072
[11070]	HIRIP3	--->	ABCF3	0.059154	-0.024569
[11071]	GUCA1A	--->	GUCY2D	0.04939	-0.062085
[11072]	PPARA	<---	STAC3	-0.07283	0.057973
[11073]	ATF3	--->	TP53	0.079028	-0.087834
[11074]	FLNA	--->	F3	0.71226	-0.71274
[11075]	ANAPC2	--->	FZR1	0.21762	-0.18583
[11076]	NR1H3	<---	RARA	-0.0098959	-0.0063998
[11077]	CXCL5	<---	CXCR1	-0.46475	0.49595
[11078]	PAX6	<---	INADL	-0.1329	0.14979
[11079]	NEUROD1	<---	CALM1	-0.22666	0.2676
[11080]	PRKCD	--->	DAB2	0.78567	-0.77586
[11081]	FGFR3	--->	STAT1	0.014039	-0.050707
[11082]	HNRNPC	<---	GRB2	-0.30113	0.31256
[11083]	SRPK1	--->	SFRS1	0.72994	-0.75755
[11084]	CDH24	<---	CTNND1	-0.52601	0.51654
[11085]	TICAM2	<---	OPN1LW	-0.11104	0.10823
[11086]	PICK1	<---	F11R	-0.15671	0.14909
[11087]	TFE3	<---	HMGB1	-0.46171	0.47559
[11088]	CCL3L3	<---	CCBP2	-0.46475	0.49595
[11089]	STAT5A	<---	TEK	-0.022459	0.0068876
[11090]	TRPV4	<---	YES1	-0.29213	0.34238
[11091]	SHANK2	--->	DNM2	0.030798	-0.043023
[11092]	GIT1	<---	RAN	-0.27068	0.26564
[11093]	CCDC33	--->	$ZBTB8$	0.1965	-0.17935
[11094]	CSF3R	<---	CISH	-0.18498	0.18267

```
[11095] YY1     <---    SP1     -0.03481        0.048953
[11096] HDAC7   <---    PRKD2   -0.46746        0.44235
[11097] UBQLN1  --->    EPS15   0.078497        -0.088747
[11098] AKT1    --->    IRS1    0.29996 -0.33499
[11099] LPAR2   --->    TRIP6   0.42955 -0.39725
[11100] FGG     <--->   FGA     0.0011944       0.0011944
[11101] MBD1    --->    SMAD9   0.15567 -0.17171
[11102] SFN     <---    MAP3K5  0.17403 0.23745
[11103] GRB2    <---    ZAP70   -0.40218        -0.1324
[11104] FURIN   <---    TNFSF13 -0.13923        0.14441
[11105] LUC7L3  <---    CLK1    -0.70995        0.6842
[11106] CDK1    <---    RAP1GAP -0.21348        0.19914
[11107] RIT2    --->    RALGDS  0.98547 -1.0084
[11108] HGS     <---    SNX1    -0.039926       -0.011609
[11109] MPL     <---    PTPN11  -0.52352        0.60329
[11110] BTBD2   --->    SMAD2   0.13507 -0.11353
[11111] IGF2    --->    PLG     0.047917        -0.032735
[11112] FLI1    <---    TTRAP   -0.22957        0.24997
[11113] HHEX    --->    JUN     0.40134 -0.44785
[11114] STAT5B  <---    TEK     -0.022459       0.0068876
[11115] PPARD   ?       RXRB    -0.0089162      -0.0089162
[11116] LRP1B   <---    SERPINE1        -0.24247        0.23656
[11117] GADD45B --->    MAP3K4  1.0051  -0.99563
[11118] INS     --->    RB1     0.48961 -0.49108
[11119] BMP1    <---    COL5A1  -0.041937       0.035051
[11120] UPF3A   <---    GSK3B   -0.71637        0.69052
[11121] RHOG    --->    SMAD1   0.52759 -0.54836
[11122] CDC42   --->    TP53    0.5057  -0.49562
[11123] CD14    --->    TLR3    0.28891 0.074216
[11124] BMPR1A  <---    BMP4    -1.0007 0.99697
[11125] RNF11   <---    SMURF2  -0.68612        0.68206
[11126] PLCG1   <---    VAV1    -0.059393       0.015696
[11127] KAT2B   --->    TCF3    0.16163 -0.17206
[11128] HNRNPAB --->    ACTB    0.52486 -0.52355
[11129] CREBBP  --->    BRCA1   0.10685 -0.11494
[11130] SKIL    <---    PLCD3   -0.054859       0.048675
[11131] CD163   <---    HP      -0.14305        0.18818
[11132] TGFBR2  --->    CCNB1   0.7651  -0.73456
[11133] PLK1    --->    CCNB1   0.55049 -0.56928
[11134] MAPK1   --->    SH2D3C  0.98948 -1.0007
[11135] ST13    <---    HSPA1A  -0.80354        0.84564
[11136] CTNNB1  --->    CDH2    0.99241 -0.98928
[11137] MAPK8   <---    HRAS    -1.0074 1.0132
[11138] CDH5    --->    PARD6A  0.0041353       -0.0095653
[11139] PLK4    --->    SMAD4   0.25338 -0.25966
[11140] ITGAM   <---    C3      -0.057453       0.044893
[11141] GNA13   <---    PRKCB   -0.89029        0.89037
[11142] TRIP10  <---    SRC     -0.31531        0.67718
[11143] CAMK2G  --->    RYR1    0.12968 -0.14062
[11144] CSNK2A1 --->    PGR     0.99834 -1.0079
[11145] HSPG2   --->    NID1    0.01282 -0.025974
[11146] JAK2    --->    CSF2RB  0.46863 -0.41001
[11147] FGFR2   --->    CACNA1D 0.053153        -0.066359
[11148] SLA     <---    ZAP70   -0.40218        -0.1324
[11149] MAPKAPK2        --->    PHC2    0.24899 -0.25972
[11150] FAM84B  <---    CTNNA1  -0.61611        0.63953
[11151] GDF6    --->    BMPR1B  0.99697 -1.0007
[11152] NUB1    --->    PSMD4   0.076162        -0.042104
[11153] ZNF263  <---    ZNF446  -0.089704       -0.01004
[11154] PSMD10  <---    MDM2    -0.1119 0.10528
[11155] HTRA1   --->    GDF5    0.21916 -0.20846
[11156] IHH     <---    HHIP    -0.99342        0.98842
[11157] COL4A6  --->    APP     0.076846        -0.089884
[11158] CADM1   <---    SDCBP   -0.026361       0.016309
[11159] NPAS2   ?       ARNTL   -0.019553       -0.019553
[11160] PDPK1   --->    PRKCD   0.24482 -0.071276
[11161] OBSCN   --->    CALM1   0.61302 -0.61711
[11162] ERN1    <---    TRAF2   -0.60031        0.60433
[11163] ADAM9   --->    ITGA3   -0.0020477      -0.010539
[11164] PIAS1   --->    NR5A1   0.095399        -0.090133
[11165] STAMBP  <---    STAM    0.031641        0.03798
[11166] OXSR1   --->    TGFBR1  0.19971 0.029867
[11167] SKP1    --->    CCNA2   0.45611 -0.45626
[11168] SH3KBP1 <---    SOS1    -0.53571        0.53351
[11169] RGS2    --->    GIT1    0.27683 -0.28206
[11170] SETDB1  <---    ERG     -0.055992       0.061822
```

[11171]	FBLN2	--->	FBN1	-0.0027601	-0.014407
[11172]	THRB	<---	CAMK2B	-0.69285	0.68233
[11173]	TRIM38	<---	RIPK1	-0.048784	0.1335
[11174]	ABCA1	<---	PDLIM3	-0.010698	0.0038951
[11175]	USP7	<---	TRAF6	-0.030264	0.67424
[11176]	DLG1	--->	EPB41	0.031325	-0.041318
[11177]	APC	<---	IQGAP1	-0.036923	0.023562
[11178]	ZBTB16	<---	CCDC85B	-0.13474	0.13214
[11179]	HEYL	<---	SMAD9	-0.63105	0.61382
[11180]	DTX3L	<---	DTX1	0.0017841	0.044917
[11181]	APC2	<---	MAPRE3	-0.017246	0.0071203
[11182]	CTBP1	--->	BRCA1	0.020833	-0.029855
[11183]	DLST	--->	ZHX1	0.1146	-0.099527
[11184]	CDH7	<---	CTNNB1	-0.32695	0.32855
[11185]	SERPINA5	<---	KLK1	-0.082185	0.11833
[11186]	RNF216	<---	TLR4	-0.42328	0.32429
[11187]	THBS1	<---	MMP2	-0.15886	0.21165
[11188]	ABL1	<---	CASP9	-0.41968	0.30417
[11189]	BMPR2	<---	BMP2	-0.99943	1.0057
[11190]	TGIF1	--->	JUN	0.13487	-0.16364
[11191]	NCOA2	--->	AR	0.11931	-0.13146
[11192]	MUSK	--->	RAPSN	0.20357	-0.1967
[11193]	SYK	--->	RPS6KA1	1.0087	-0.99081
[11194]	GTF3C1	<---	RBM23	0.010571	0.020924
[11195]	OPRD1	--->	ARRB2	0.27159	-0.25831
[11196]	WNT1	--->	LRP6	0.99882	-0.99193
[11197]	GDF9	<---	FXYD6	-0.074332	0.11849
[11198]	MAPKAPK3	--->	MAPK14	1.0074	-0.9992
[11199]	GAS1	--->	SHH	1.0121	-0.99528
[11200]	TICAM2	--->	IRAK3	0.58357	-0.54271
[11201]	TCF4	<---	CALM1	-0.33491	0.33066
[11202]	UBQLN4	--->	SMAD9	0.33166	-0.35027
[11203]	CDH1	<---	PTPRM	-0.14031	0.12974
[11204]	CHD3	<---	KIF15	-0.0070472	-0.002952
[11205]	ORAI1	--->	TRPC6	0.21846	-0.22842
[11206]	SMAD2	<---	KAT2B	-1.0011	0.99663
[11207]	HCLS1	--->	ACTR2	0.13559	-0.10757
[11208]	CDC7	--->	CDKN2A	0.25926	-0.26299
[11209]	CDH5	<---	CTNNA1	-0.39209	0.4013
[11210]	JUN	<---	HOXA9	-0.44785	0.40134
[11211]	SOCS2	--->	PRLR	0.0057133	-0.017809
[11212]	USF1	<---	HDAC9	-0.57566	0.61334
[11213]	HDAC3	--->	GATA2	0.16966	-0.12711
[11214]	PTK2	--->	ITGB2	0.081194	-0.099332
[11215]	PRKCA	--->	GRIA2	0.56969	-0.57379
[11216]	RDH5	<---	RGR	-0.39723	0.4277
[11217]	HACE1	<---	PLXNA2	-0.10908	0.11316
[11218]	ERBB2IP	<---	ERBB2	-0.10063	0.091088
[11219]	KLF6	<---	HDAC3	-0.28908	0.29
[11220]	EVL	<---	CRKL	-0.12455	0.099229
[11221]	CHAF1A	--->	CHAF1B	0.053748	-0.050424
[11222]	UBE2I	--->	SNIP1	0.074392	-0.058332
[11223]	SENP6	--->	ARNT	0.22576	-0.21987
[11224]	ATXN1	<---	SMARCC2	-0.038607	0.035269
[11225]	PEA15	<---	MAPK3	-0.53527	0.57162
[11226]	VAV3	<---	EGFR	-0.99724	0.99895
[11227]	EGFR	--->	MAP2K1	1	-1.0032
[11228]	PDPK1	--->	RPS6KB1	1.0929	-1.1695
[11229]	RASA1	<---	GRB2	-0.12459	0.10455
[11230]	HIPK3	--->	FLNC	0.72542	-0.75118
[11231]	NEUROG1	<---	CREBBP	-0.20391	0.19969
[11232]	MRPS9	<---	RBM4B	-0.0051128	0.039857
[11233]	PRMT1	--->	FUS	0.16119	-0.16564
[11234]	GNAI1	--->	GPSM2	0.22114	-0.20473
[11235]	IL7R	<---	PTK2B	-0.11665	0.064294
[11236]	DLG4	--->	GRIK2	0.058844	-0.064781
[11237]	MAPK14	--->	NCF1	0.088462	-0.1645
[11238]	PDLIM7	<---	PRKCB	-0.57553	0.57562
[11239]	BSG	--->	PDLIM7	0.22074	-0.22549
[11240]	RPTOR	--->	EIF4EBP1	1.0068	-1.0068
[11241]	LRP2	<---	SERPINE1	-0.39946	0.39875
[11242]	SETDB1	<---	RNF10	-0.17644	0.18016
[11243]	MBD1	--->	SMAD1	0.15567	-0.17171
[11244]	FBXW11	<--->	BTRC	0.12303	0.12303
[11245]	PIAS1	--->	SMAD1	0.62904	-0.6365
[11246]	TP53	<---	SMARCD1	-0.29484	0.29712

[11247]	DGKZ	--->	RBL2	0.1014	-0.11299
[11248]	MAPK3	--->	LIPE	0.53776	-0.55596
[11249]	SMAD1	--->	HOXD13	0.010712	-0.04291
[11250]	ELK1	--->	ID1	0.12676	-0.084944
[11251]	SKIL	<---	TSKU	-0.25888	0.25695
[11252]	CCDC33	<---	NINL	-0.31952	0.35961
[11253]	LCK	--->	KIR2DL3	0.36137	-0.27195
[11254]	MDM4	<---	EP300	-0.045257	0.033444
[11255]	NCOR2	--->	NR1H2	0.13736	-0.14734
[11256]	CASKIN1	<---	CASK	-0.028865	0.0065714
[11257]	ABCA1	<---	MPDZ	-0.0080068	0.005539
[11258]	MAPK8	--->	NR3C1	0.99834	-1.0079
[11259]	H3F3A	<---	SMARCA5	-0.04883	0.049463
[11260]	NONO	?	PSPC1	-0.016298	-0.016298
[11261]	PER3	--->	CLOCK	0.074763	-0.087389
[11262]	HDAC5	<---	YWHAB	-0.08049	0.08119
[11263]	LIN7B	<---	BAIAP2	0.00089845	0.0030996
[11264]	HSP90AA1	--->	ASGR1	0.048202	-0.059464
[11265]	RAD18	<---	UBE2A	-0.17337	0.20782
[11266]	RAC1	--->	CHN1	0.15564	-0.15063
[11267]	PRPF40A	--->	CHD3	0.020709	-0.030863
[11268]	WASF2	--->	ACTB	0.26671	-0.2714
[11269]	IGF1R	<---	IGFBP3	-0.03567	0.024548
[11270]	PARD3	--->	PLCB3	0.11916	-0.12669
[11271]	ITGB1	<---	FN1	-0.14385	0.13417
[11272]	TEAD1	--->	MAX	0.63969	-0.60212
[11273]	EPB41L1	<---	ARHGAP1	-0.036367	0.033329
[11274]	SFN	<---	MARK3	-0.092392	0.077417
[11275]	ASAP2	<---	SH3KBP1	-0.0047018	0.056508
[11276]	GRB2	--->	RHOU	0.44166	-0.3944
[11277]	RUNX2	--->	CEBPB	0.099202	-0.049267
[11278]	PAK1	--->	SMAD1	0.87062	-0.7402
[11279]	CSF2RA	<---	KIT	-0.19237	0.15508
[11280]	PRKCZ	--->	KRT10	0.25913	-0.27271
[11281]	FAM175A	--->	BRCA1	0.074128	-0.057632
[11282]	SERPINB13	<---	PRTN3	-0.082185	0.11833
[11283]	CCNT1	<---	SKP2	-0.4796	0.51293
[11284]	PEX14	<---	HDAC1	-0.030037	0.046151
[11285]	CDK1	<---	PTPN2	-1.009	1.0061
[11286]	YWHAZ	--->	TJP2	0.073422	-0.070569
[11287]	RAB13	<---	SMURF2	-1.0084	1.0007
[11288]	EP300	--->	PAX6	0.1118	-0.11991
[11289]	THRA	<---	CREBBP	-0.18496	0.17826
[11290]	GIT1	<---	ADRBK2	-0.16512	0.14915
[11291]	RPS6KA1	--->	NFKBIA	0.53101	-0.68424
[11292]	SKIV2L2	--->	XRN1	0.0019114	-0.005057
[11293]	MAPK14	--->	DDIT3	1.0113	-0.99712
[11294]	NOS1AP	--->	LRP2	0.40272	-0.4035
[11295]	CAMK1	--->	NOS1	0.48839	-0.49502
[11296]	MARK1	--->	MAPT	0.095209	-0.10782
[11297]	ZNF653	<---	NR5A1	-0.085284	0.094269
[11298]	TTRAP	--->	TNFRSF1B	0.0026914	-0.12515
[11299]	DHX9	<---	PRPF8	-0.13534	0.14604
[11300]	TJP3	?	TJP1	-0.011542	-0.011542
[11301]	SDCBP	--->	GRIA1	0.21545	-0.21967
[11302]	RGS14	--->	GNAI1	0.013429	-0.011715
[11303]	PTK2	<---	PDGFRB	-0.14154	0.10352
[11304]	MYC	<---	KIAA1524	-0.17037	0.17113
[11305]	IGF1R	--->	PIK3R2	0.024958	-0.05517
[11306]	IL4R	<---	SOCS5	-0.094117	0.066673
[11307]	PDPK1	--->	YWHAQ	0.39388	-0.25176
[11308]	CUL1	<---	RAC2	-0.10799	0.11776
[11309]	CORO1B	<---	SSH1	-0.58449	0.60262
[11310]	ADRB2	--->	IL1B	0.066257	-0.06418
[11311]	PAK2	<---	ABL1	-0.12443	0.043133
[11312]	KLHL2	?	KLHL12	-0.073466	-0.073466
[11313]	PRKACA	<---	RRAD	-1.0074	1.0132
[11314]	HDAC3	--->	CCND1	0.6473	-0.6338
[11315]	PDPK1	--->	PRKCI	0.27769	-0.26638
[11316]	ARR3	<---	RCOR2	-0.01607	0.019817
[11317]	ARHGAP1	--->	SMARCD3	0.020501	-0.017139
[11318]	HDAC5	--->	ANKRA2	0.061031	-0.060221
[11319]	GNA15	<---	CHRM2	-0.98627	0.9872
[11320]	MFN1	--->	BAK1	0.47818	-0.46166
[11321]	NFKBIB	<---	RELA	-0.0125	0.01764
[11322]	YY1	--->	NOTCH1	0.25428	-0.2698

[11323]	DRD4	<---	GRB2	-0.053429	0.054359
[11324]	MMP10	<---	HAPLN1	-0.093464	0.097177
[11325]	PLAU	--->	SERPINE1	0.057207	-0.040728
[11326]	YWHAG	--->	SHROOM2	0.032445	-0.021305
[11327]	USP7	--->	ATXN1	0.047825	-0.050411
[11328]	RRAS2	--->	ARAF	1.0008	-0.9994
[11329]	PRKCE	--->	RPS6KB2	-0.031433	-0.1639
[11330]	L1CAM	<---	CNTN2	0.0078499	0.010648
[11331]	GNAI2	--->	ADCY1	1.0075	-1.0077
[11332]	SHC1	<---	GRB2	-0.40614	0.39868
[11333]	MAP2K4	--->	FLNC	0.72542	-0.75118
[11334]	RNF126	<---	UBE2D4	-0.17305	0.18537
[11335]	LPA	--->	FN1	0.02035	-0.0083667
[11336]	YWHAG	<---	PRMT1	-0.25789	0.25665
[11337]	IL2RG	<---	IL7	-0.19892	0.17723
[11338]	KHDRBS1	<---	GRB2	-0.28063	0.27717
[11339]	HOXB1	<---	CREBBP	-0.18943	0.18505
[11340]	SNX2	<---	PDGFRB	-0.046812	0.040725
[11341]	ZNF295	<---	YWHAG	-0.090415	0.099295
[11342]	IRAK1	--->	ITGAM	0.019052	-0.031773
[11343]	BAI1	<---	MAGI1	-0.035785	0.027059
[11344]	CASP2	--->	RB1	0.27918	-0.28711
[11345]	CSNK2A1	--->	MYCN	1.1385	-1.1233
[11346]	EP300	--->	COPS6	0.1749	-0.17946
[11347]	SRC	--->	KCNB1	1.0056	-0.89226
[11348]	FLT4	--->	SHC3	0.63416	-0.65949
[11349]	CD4	--->	CD44	0.033954	-0.041904
[11350]	CREBBP	--->	CREB1	0.045008	-0.056352
[11351]	CPNE1	<---	PPP5C	-0.043387	0.048759
[11352]	SFRP1	--->	WNT1	0.99043	-0.98922
[11353]	PTPRK	--->	JUP	0.089247	-0.10181
[11354]	BMPR1A	--->	SF3B4	0.0992	-0.12565
[11355]	CAV1	--->	PTGS2	0.036418	-0.024728
[11356]	DVL1	<---	CSNK1E	-0.9989	0.99947
[11357]	KCNQ3	--->	CALM1	0.32976	-0.29233
[11358]	EPHA2	--->	PIK3R2	0.17224	-0.26621
[11359]	OPHN1	<---	CDC42	-0.67799	0.70845
[11360]	DMBT1	<---	SFTPA1	-0.050899	0.04973
[11361]	ATXN1	<---	MED15	-0.024865	0.014334
[11362]	DLAT	?	PDHX	-0.0055237	-0.0055237
[11363]	MATK	--->	NTRK1	0.1206	-0.19437
[11364]	PPP1CC	--->	YWHAZ	0.13714	-0.14788
[11365]	UBE2I	--->	WT1	0.19566	-0.18826
[11366]	EIF2C2	--->	DICER1	0.042243	-0.02712
[11367]	SOX10	<---	PRRX2	-0.022513	0.018113
[11368]	LRRC23	--->	CD28	0.086858	-0.062906
[11369]	SMAD3	<---	MDM4	-0.10035	0.1079
[11370]	SMAD2	--->	HOXA13	0.20304	-0.14259
[11371]	EIF2C2	--->	DDX20	0.042243	-0.02712
[11372]	RNF11	--->	UBAC1	0.13586	-0.10319
[11373]	GAB2	<---	GRB2	-0.79676	0.76817
[11374]	ZNF689	<---	NCL	-0.065797	0.055708
[11375]	NCOR2	--->	NR1H4	0.13736	-0.14734
[11376]	MAPK3	--->	TOP2B	0.52133	-0.54823
[11377]	TNFRSF10C	<---	LRSAM1	-0.085768	0.088515
[11378]	CASP1	<---	HTT	-0.02966	0.037921
[11379]	KCNJ12	<---	APBA1	-0.036942	0.042042
[11380]	RIPK1	<---	TRAF2	-1.0071	0.99521
[11381]	BAD	<---	YWHAG	-1.0088	0.9978
[11382]	MAPK9	<---	TRAF2	-0.9866	1.0023
[11383]	TRIM11	<---	MED15	-0.0048051	-0.0032986
[11384]	HSPG2	--->	FBLN2	0.023365	-0.03602
[11385]	ABCA1	--->	DLG5	0.00071402	-0.0069696
[11386]	CASP3	--->	EIF2AK2	0.87492	-0.98085
[11387]	BAIAP2	<---	LIN7A	-0.0044474	0.0013915
[11388]	SDCBP	--->	GRIA3	0.11794	-0.11896
[11389]	CCR5	--->	ARRB1	0.27159	-0.25831
[11390]	KRT15	<---	AMOTL2	-0.03007	0.045904
[11391]	PLG	--->	CXCL2	0.020806	-0.0054969
[11392]	LYN	--->	CSF2RB	0.98931	-1.0003
[11393]	CXCR2	--->	GNA15	1.0129	-1.008
[11394]	ETV6	<---	UBE2I	-0.19098	0.198
[11395]	NR4A2	<---	PIAS4	-0.10153	0.10957
[11396]	UBE2I	--->	C18orf25	0.054457	-0.021373
[11397]	TBK1	<---	NCK1	-0.11887	0.076812
[11398]	TP73	<---	HRAS	-0.01994	0.052964

11401 [11399] PRKCA ---> PEBP1 1.0121 -1.008
11402 [11400] GATA4 <--- JARID2 -0.045704 0.088735
11403 [11401] TNFRSF11A <--- TRAF1 -0.034181 0.029707
11404 [11402] RXRA <--- FUS -0.16651 0.033215
11405 [11403] GRK6 ---> FSHR 0.086299 -0.1244
11406 [11404] RIMS1 <--- RAB10 -0.0067445 0.016184
11407 [11405] FURIN ---> SORL1 0.021414 -0.032828
11408 [11406] HK2 <--- UBQLN1 -0.02975 0.044875
11409 [11407] FNBP4 <---> PRPF40A 0.34574 0.34574
11410 [11408] SERPINB9 <--- COPS6 -0.32079 0.34926
11411 [11409] THRB <--- NCOR2 -0.14734 0.13736
11412 [11410] SOX9 <--- NR5A1 -0.06735 0.056557
11413 [11411] YWHAG <--- FGD6 -0.099777 0.097041
11414 [11412] ESR1 ? NR2F1 -0.0089162 -0.0089162
11415 [11413] ARR3 <--- SRC -0.54011 0.60338
11416 [11414] HIST1H4A <--- PAK1 -0.51322 0.84168
11417 [11415] NOD1 <--- CASP8 -0.43933 0.34784
11418 [11416] CUL7 <--- SKP1 -0.074907 0.08479
11419 [11417] INSR ---> DOK1 0.35356 -0.35555
11420 [11418] PIAS4 ---> MAP1LC3A 0.19653 -0.1864
11421 [11419] CCNG1 <--- PPP2CA -0.30921 0.30227
11422 [11420] HDAC2 <--- TOP2A -0.074374 0.089479
11423 [11421] DAB1 ---> PAFAH1B1 0.043119 -0.027625
11424 [11422] RALGPS1 <--- GRB2 -0.43896 0.5088
11425 [11423] SLC12A6 <--- OXSR1 -0.42108 0.37034
11426 [11424] SRC ---> GRIN2B 0.24276 -0.22786
11427 [11425] ITGAV <--- ICAM4 -0.018913 0.0067704
11428 [11426] SOX18 ---> HMGB1 0.77715 -0.67319
11429 [11427] PKN2 <--- PTPN13 -0.02075 -0.0011132
11430 [11428] MTOR ---> PLD2 0.0087583 -0.021717
11431 [11429] PER3 ? PER1 -0.0031331 -0.0031331
11432 [11430] P2RY1 ---> ADORA1 0.43766 0.24045
11433 [11431] EIF2AK2 <--- HSP90AA1 -0.056762 0.040193
11434 [11432] MAP3K5 <--- TRAF5 -0.33509 0.38408
11435 [11433] LCK ---> CD79B 0.99528 -0.99051
11436 [11434] CHD3 <--- SIRT6 -0.0070472 -0.002952
11437 [11435] STRN ---> CALM1 0.21607 -0.1798
11438 [11436] VDR ? NR1H2 -0.0089162 -0.0089162
11439 [11437] ZBTB48 <--- $ZBTB8$ -0.14508 -0.026277
11440 [11438] PTPN18 ---> ABL1 0.12235 -0.35431
11441 [11439] SMEK2 <--- PPP4C -0.22611 0.21642
11442 [11440] CDKN2AIP ---> CDKN2A 0.080629 -0.062368
11443 [11441] YAF2 ---> MYCN 0.42441 -0.40463
11444 [11442] NFATC1 <--- PIM1 -1.007 0.99987
11445 [11443] NTF4 <--- NGFR 0.037444 0.14607
11446 [11444] EPHA8 <--- FYN 0.046336 0.39258
11447 [11445] NID2 <--- LAMC1 -0.018071 0.0083042
11448 [11446] SMAD2 ---> ZNF8 0.12829 -0.12271
11449 [11447] BAG1 ---> BCL2L1 0.68684 -0.68331
11450 [11448] C1QC <--- C1QB -0.0046012 0.011291
11451 [11449] MAPK1 ---> NCOA1 0.30243 -0.31801
11452 [11450] TGFB1 ---> CTGF 0.077807 -0.11204
11453 [11451] GRAP2 <--- MAP4K1 -0.1786 0.19604
11454 [11452] IFT172 ---> LHX4 0.070191 -0.055821
11455 [11453] CSNK1A1 ---> HNRNPC 0.58079 -0.50147
11456 [11454] SQSTM1 ---> NR2F2 0.054691 -0.051711
11457 [11455] PRKCB ---> RB1 0.26671 -0.27682
11458 [11456] PRKACA ---> RAF1 0.98888 -1.0049
11459 [11457] TEC ---> VAV1 0.98501 -1.0133
11460 [11458] CASP1 <--- NEDD4 -0.12469 0.113
11461 [11459] MAP3K6 <--- MAP3K5 0.24555 0.56058
11462 [11460] EIF3B ---> EIF3I 0.13878 -0.10831
11463 [11461] CYTH3 <--- CNKSR1 0.12048 0.21789
11464 [11462] FYN ---> CDK1 1.3862 -1.2776
11465 [11463] CDK1 <--- PTPN1 -1.009 1.0061
11466 [11464] GFI1B <--- HECW1 -0.047898 0.034985
11467 [11465] MKI67IP <--- MKI67 0.004119 0.027374
11468 [11466] EP300 ---> BRCA1 0.10685 -0.11494
11469 [11467] SSTR2 <--- PTPN6 -0.24618 0.35472
11470 [11468] E2F1 <--- SP1 -0.10437 0.092459
11471 [11469] FN1 <--- PRPF40A -0.33626 0.36323
11472 [11470] WRN <--- TERF2 -0.0012546 0.016635
11473 [11471] RELA ---> REL -0.013421 -0.043838
11474 [11472] SOX8 <--- POU5F1 -0.060464 0.063322
11475 [11473] INADL ---> SCN4A 0.050181 -0.013537
11476 [11474] CDKN2B <--- CDK4 -1.3222 1.3062

[11475]	MAPK8IP2	<---	MAP2K3	-0.30961	0.2909
[11476]	IL10	--->	IL10RB	0.74479	-0.74442
[11477]	NUMB	--->	APP	0.21546	-0.22695
[11478]	SFRS2IP	--->	U2AF2	0.12373	-0.11096
[11479]	GAB3	<---	FYN	-0.64147	0.75598
[11480]	SH2B3	--->	CD247	0.015079	-0.14815
[11481]	HDAC1	--->	TAL1	0.23353	-0.22822
[11482]	CRIP1	<---	SH3GL3	-0.075786	0.091263
[11483]	PIAS2	--->	STAT4	0.54276	-0.55325
[11484]	MECR	--->	THRB	0.074075	-0.048313
[11485]	MEGF10	--->	HDAC4	-0.0030951	-0.0099613
[11486]	VEGFA	--->	SPARC	0.043752	-0.04081
[11487]	UBE2I	--->	SREBF2	0.37111	-0.36902
[11488]	GIT2	?	GIT1	-0.022111	-0.022111
[11489]	BMP1	<---	COL5A2	-0.037281	0.024115
[11490]	CREBBP	--->	SUV39H1	0.063953	-0.074254
[11491]	NCOA3	<---	DDX17	-0.083398	0.070309
[11492]	BMPR1A	<---	BMP5	-1.0007	0.99697
[11493]	TAL1	<---	LMO2	-0.3637	0.38177
[11494]	PTPN22	<---	CBL	-0.12773	0.1154
[11495]	SOSTDC1	--->	BMP7	0.43337	-0.38277
[11496]	DLG4	<---	MAP3K10	-0.22886	0.20116
[11497]	MSN	<---	L1CAM	-0.044651	0.031787
[11498]	NF1	--->	APP	0.015491	-0.029099
[11499]	NOTCH4	<---	SMAD3	-0.14179	0.1305
[11500]	YWHAZ	--->	PRLR	0.0098763	-0.021831
[11501]	RBMX	--->	KHDRBS2	0.10067	-0.097474
[11502]	FYN	--->	SHC1	0.73568	-0.65926
[11503]	ZNF167	?	ZNF434	-0.093486	-0.093486
[11504]	PTPRS	--->	RAB35	0.19147	-0.19879
[11505]	PECAM1	--->	CTNNB1	0.56398	-0.56295
[11506]	PIK3R1	<---	CDH2	-0.087176	0.039902
[11507]	CAMK2G	--->	PLCB3	0.41213	-0.4132
[11508]	RAG1	<---	KPNA1	-0.20171	0.1952
[11509]	VAV1	<---	CRK	-0.35104	0.36032
[11510]	SPI1	<---	TBP	-0.006393	-0.0056132
[11511]	TRPV4	--->	PACSIN2	0.035277	-0.038389
[11512]	GNA12	<---	TBXA2R	-1.008	1.0129
[11513]	DMD	--->	ACTC1	0.14504	-0.15537
[11514]	SYNGAP1	--->	GRIN2B	0.045949	-0.05399
[11515]	HDAC5	<---	CIITA	-0.074461	0.084073
[11516]	CBLC	--->	SRC	0.065303	-0.052314
[11517]	ZIC3	<---	GLI3	-0.6065	0.6023
[11518]	PTPRK	--->	PTPRM	0.0029541	0.00081878
[11519]	SKI	--->	GLI2	0.23559	-0.24244
[11520]	GNB5	<---	RGS6	-0.33708	0.34928
[11521]	ELK1	<---	MAPK8	-0.98814	1.0428
[11522]	GNAI2	<---	FPR1	-0.65925	0.65603
[11523]	PARD6G	<---	PARD3	-0.010302	-0.0054402
[11524]	RAB1A	--->	TBC1D17	0.38941	-0.35867
[11525]	H3F3A	<---	SMARCA4	-0.038783	0.033623
[11526]	DAZ1	<---	DZIP3	-0.12546	0.13843
[11527]	LRRC1	--->	ERBB2IP	0.013738	0.0066074
[11528]	TEAD1	--->	TBP	0.00066752	-0.012917
[11529]	FGFR3	<---	GRB2	-0.078027	0.024925
[11530]	PRKACA	--->	PDE4B	0.99264	-1.0111
[11531]	STAT5A	<---	CCR5	-0.54929	0.53162
[11532]	RIN3	<---	RAB5B	-0.33568	0.34965
[11533]	GRB7	<---	GRB2	-0.32673	0.34015
[11534]	MAPK14	--->	PPARGC1A	0.058876	-0.10921
[11535]	SALL1	<---	HDAC1	-0.18352	0.17875
[11536]	UBR5	<---	MAPK1	-0.61734	0.59218
[11537]	REM1	--->	YWHAE	0.32694	-0.30645
[11538]	CCBP2	--->	CCL5	0.49595	-0.46475
[11539]	PRKACA	--->	APC	0.13984	-0.15381
[11540]	TLR7	--->	MYD88	0.14052	0.020105
[11541]	SMTN	--->	ACTA1	0.51886	-0.51601
[11542]	DLG2	<---	KCNA4	-0.034928	0.041469
[11543]	ENG	--->	BMP7	0.36037	-0.30966
[11544]	KIF1B	--->	YWHAG	0.061745	-0.066344
[11545]	ADAM9	<---	TNF	-0.13274	0.1341
[11546]	CCL13	<---	CCR2	-0.46475	0.49595
[11547]	DLG2	<---	DLG1	-0.020129	-0.0093747
[11548]	SYT16	<--->	SYT14	0.058592	0.058592
[11549]	NGEF	<---	EPHA4	-0.17814	0.13962
[11550]	USP7	--->	PICK1	0.082571	-0.042549

[11551]	MAPK1	--->	PRKCZ	0.1627	0.012708
[11552]	NRCAM	?	CNTN2	-0.0017969	-0.0017969
[11553]	SYNGAP1	--->	GRIN2D	0.045949	-0.05399
[11554]	RNF11	<---	UBE2D2	-0.17305	0.18537
[11555]	SRI	--->	CACNA1C	0.293	-0.3051
[11556]	CSNK2A1	--->	IGFBP3	0.7558	-0.78174
[11557]	LDLR	<---	SNX17	-0.14834	0.14342
[11558]	DOK3	<---	FYN	-1.1853	1.3209
[11559]	ABL1	<---	JAK2	-0.028405	0.1162
[11560]	SMARCA4	--->	STAT2	0.064777	-0.080818
[11561]	CD226	<---	FYN	-0.17174	0.1294
[11562]	CTTN	--->	ACTA1	0.34064	-0.33098
[11563]	HSPB3	--->	SETDB1	0.21798	-0.19753
[11564]	RNF11	<---	STAM2	0.02479	0.045684
[11565]	RPS6KA5	--->	PLA2G4A	0.24128	-0.35828
[11566]	FUZ	<---	C1orf89	0.0076475	0.02637
[11567]	VEGFA	<---	EPHB2	-0.079612	0.06757
[11568]	BIN1	--->	ASAP2	0.030013	-0.0050146
[11569]	REM1	--->	CACNB2	0.42812	-0.39718
[11570]	ELANE	--->	CXCL12	0.058311	-0.024098
[11571]	MXD1	--->	MYC	-0.23569	-0.31161
[11572]	PBK	--->	TP53	1.0002	-0.99962
[11573]	MYOM1	--->	MYH9	0.0025322	-0.014313
[11574]	TNFRSF1A	<---	JAK1	-0.32617	0.31731
[11575]	NOS3	<---	BDKRB2	-0.34063	0.3307
[11576]	PSMD1	--->	BAG1	0.18221	-0.15409
[11577]	CALM1	--->	VIPR1	0.25258	-0.24765
[11578]	PPP1CA	--->	CAD	0.12592	-0.13568
[11579]	LCK	<---	PRKCD	-0.09907	0.095887
[11580]	ERMAP	--->	VASP	0.020005	-0.024741
[11581]	CSNK2A1	<---	PTPN1	-1.009	1.0061
[11582]	ZAP70	--->	DUSP3	1.0053	-1.0087
[11583]	CEBPG	<---	MLLT6	-0.082242	0.13197
[11584]	STAT3	<---	PTK2B	-0.34159	0.23533
[11585]	PTPN13	--->	PDLIM4	0.061338	-0.03057
[11586]	GGA3	<---	SYNRG	0.011562	0.022527
[11587]	SAMD8	<---	TGFBR1	-0.16628	0.14256
[11588]	HIF3A	<---	EGLN2	-0.012006	0.0022157
[11589]	CABLES1	--->	CCNA1	-0.11778	-0.65097
[11590]	XRN2	--->	TARDBP	0.041399	-0.027487
[11591]	CSNK2A1	--->	SPTBN1	0.28219	-0.29697
[11592]	PPP2R1B	--->	RAB18	0.13801	-0.13281
[11593]	CGN	--->	TJP2	0.050357	-0.043829
[11594]	NR1D2	<---	RGN	-0.047128	0.072896
[11595]	GADD45B	--->	PPARG	0.0090803	0.0011057
[11596]	WAS	<---	CIB1	-0.098149	0.060317
[11597]	CDC20	<---	BUB1B	-0.86422	0.83716
[11598]	GRM7	--->	CALM2	0.31782	-0.27991
[11599]	PTPN4	--->	PRSS1	0.28445	-0.29309
[11600]	NEK6	--->	RPS6KB1	1.002	-1.0109
[11601]	MYC	<---	SKP2	-0.32044	0.33067
[11602]	PIK3CG	<---	HRAS	-0.43686	0.43193
[11603]	HNRNPR	<---	SMN1	0.0023256	0.011379
[11604]	MAP4	--->	CCNB1	0.56737	-0.55273
[11605]	PF4	<---	FGF2	0.0061408	0.027574
[11606]	ACTN4	<---	CAMK2A	-0.21773	0.202
[11607]	TNFRSF1A	<---	GRB2	-0.053252	0.15828
[11608]	YAP1	--->	TEAD1	0.64247	-0.63315
[11609]	SHB	<---	JAK1	-0.4182	0.50446
[11610]	MKNK1	--->	MAPK14	0.61952	-0.60904
[11611]	HDAC5	--->	MEF2C	0.065789	-0.07375
[11612]	SGSM2	<---	GDPD2	-0.037102	0.041512
[11613]	PIN1	<---	TRAF2	-0.070276	0.075566
[11614]	NOTCH2	<---	PSEN2	-0.99286	0.99249
[11615]	TNK2	<---	CDC42	-0.02265	0.0093665
[11616]	PIN1	--->	UBQLN4	0.26122	-0.22895
[11617]	TP53	<---	TFDP1	-0.19715	0.19601
[11618]	ATN1	--->	TRIP6	0.034156	-0.03622
[11619]	IL6ST	<---	LIFR	-0.12732	0.033591
[11620]	SRC	--->	FOXO1	0.11512	-0.11191
[11621]	PRKCA	--->	APLP2	0.12402	-0.13582
[11622]	BMI1	<---	MLL	-0.069374	0.086432
[11623]	BMP2	<---	CHRD	-1.0007	1.0034
[11624]	PRKCZ	<---	AKT1	-0.0047278	0.082925
[11625]	NFKB2	--->	GTF2I	0.011209	-0.029347
[11626]	DKK3	<---	UBQLN4	-0.028874	0.063055

[11627]	PIAS1	--->	SATB2	0.067311	-0.065506
[11628]	KLK2	--->	SERPINA3	0.11833	-0.082185
[11629]	TBC1D3F	<---	RAC1	-0.35867	0.38941
[11630]	CSNK2A2	--->	HDAC1	0.4	-0.42066
[11631]	RHEB	<---	TSC2	-0.99668	1.0033
[11632]	ATXN1	<---	STAC2	-0.062918	0.056139
[11633]	OLIG2	<---	NKX2-2	-0.082613	0.083862
[11634]	KAT5	--->	CCNB1	0.39097	-0.38819
[11635]	HGS	<---	UBE2I	-0.090937	0.039257
[11636]	RELA	<---	BRCA1	-0.070247	0.068376
[11637]	RAB11FIP2	<---	YWHAG	-0.0074903	0.037827
[11638]	PDGFRB	--->	STAT1	0.13199	-0.14713
[11639]	PARD3	--->	TIAM2	-0.014941	-0.052872
[11640]	MAPK8	--->	MAPK8IP1	0.2909	-0.30961
[11641]	CDK4	--->	ZNF655	0.55651	-0.51628
[11642]	MAPK9	--->	ELK1	1.0428	-0.98814
[11643]	FHL3	--->	ADAMTSL4	0.045064	-0.030346
[11644]	ARHGEF2	<---	RHOA	-0.14455	0.15203
[11645]	ADRBK1	--->	PEBP1	0.53237	-0.55593
[11646]	AGAP2	--->	PIK3R1	0.047022	-0.063829
[11647]	LRP2	<---	PLAU	-0.25174	0.24565
[11648]	INSR	<---	CAV3	-0.14955	0.13911
[11649]	XRN2	<---	MOCS3	-0.04531	0.082023
[11650]	CHRM4	<---	GRB2	-0.053429	0.054359
[11651]	LPAR1	--->	ARHGEF11	0.10316	-0.106
[11652]	SFRS2IP	--->	PSEN1	0.10047	-0.086186
[11653]	CPNE1	<---	UBE2M	-0.12637	0.16628
[11654]	PRKCD	--->	NCF1	0.99964	-1.0057
[11655]	TLR1	<---	TLR5	0.11276	0.13155
[11656]	PKP4	--->	GABARAP	0.035348	-0.02765
[11657]	FXR2	<---	AMOTL2	-0.060378	0.095248
[11658]	LRP6	<---	WNT3A	-0.99193	0.99882
[11659]	NXF1	--->	MAP1B	0.080903	-0.046972
[11660]	PTAFR	--->	ARRB2	0.25543	-0.25215
[11661]	SNX6	--->	LEPR	0.3803	-0.38118
[11662]	CASP6	--->	CHD3	0.043109	-0.065023
[11663]	AKT1	--->	MAP3K5	0.98638	-0.9981
[11664]	ATN1	--->	STXBP4	-0.0049883	-0.006158
[11665]	RREB1	--->	NEUROD1	0.023635	-0.016749
[11666]	BMPR1B	<---	RHOJ	-0.53994	0.52577
[11667]	SPI1	<---	CREBBP	-0.1922	0.18812
[11668]	GAB2	<---	CD247	-0.24828	0.23533
[11669]	KCNJ10	<---	DLG1	-0.04695	0.04551
[11670]	FCER1A	--->	ITIH2	0.42235	-0.39237
[11671]	F10	<---	PLAT	-0.006567	-0.0060678
[11672]	MYBPH	<--->	MYBPC2	0.0649	0.0649
[11673]	ICAM3	--->	ZBTB16	0.037059	-0.047616
[11674]	PIN1	--->	CCNB1	0.82413	-0.8118
[11675]	SYNJ2	<---	GRB2	-0.29365	0.30666
[11676]	GNAZ	<---	DRD5	-1.008	1.0129
[11677]	CUEDC2	--->	ESR1	0.066894	-0.041506
[11678]	HEYL	<---	PPP1CA	-0.21846	0.20921
[11679]	MLL2	--->	SMAD1	0.34922	-0.36661
[11680]	FOXH1	--->	SMAD3	0.52503	-0.44328
[11681]	SLC9A3R1	--->	PTH1R	0.37858	-0.37394
[11682]	SMAD5	<--->	SMAD1	0.004047	0.004047
[11683]	XRN1	<---	STAM2	-0.0049017	0.022082
[11684]	NUCB1	<---	GNAI2	-0.49311	0.51194
[11685]	CEBPE	<---	E2F1	-0.036105	0.059437
[11686]	EPHB2	--->	RASA1	-0.0081541	-0.21105
[11687]	VCAM1	--->	ITGB1	0.062871	-0.075774
[11688]	HDAC3	<---	HDAC1	0.0010361	0.021264
[11689]	MAPK8IP1	<---	MAP3K11	-0.090912	0.30425
[11690]	SRF	--->	MYOD1	0.42755	-0.40418
[11691]	TNFRSF10A	--->	BCL10	0.24261	-0.060782
[11692]	FOXG1	--->	HES1	0.32366	-0.32905
[11693]	SH3BP2	<---	ZAP70	-0.99817	1.004
[11694]	ELANE	<---	CXCR4	-0.42368	0.45462
[11695]	NFKB2	--->	TNIP2	0.01166	-0.029813
[11696]	SMAD2	<---	ANTXR2	-0.32916	0.41207
[11697]	GNA13	--->	CTNNB1	0.55172	-0.55141
[11698]	KDR	--->	PTPN11	0.52325	-0.39752
[11699]	CHD9	--->	NR3C1	-0.0054507	-0.0084696
[11700]	GNAQ	<---	RGS2	-1.0015	1.0012
[11701]	RAP1GDS1	<---	KRAS	-0.41126	0.44004
[11702]	TRAF4	--->	UBE2I	0.08149	-0.072545

[11703]	FGF6	--->	FGFR1	1.0053	-0.99871
[11704]	TRAF4	--->	TGFB1I1	0.11462	-0.12191
[11705]	CSF1R	--->	RASA1	0.18559	-0.26109
[11706]	U2AF2	--->	DVL2	0.3831	-0.38332
[11707]	ARR3	--->	DVL2	0.23088	-0.2363
[11708]	LYN	--->	PPP1R8	1.0644	-0.90362
[11709]	BEGAIN	<---	DLG4	-0.041151	0.041422
[11710]	SHC2	<---	GRB2	-0.22485	0.22505
[11711]	PAK1	--->	SMAD2	0.68	-0.57197
[11712]	PER2	?	PER1	-0.0031331	-0.0031331
[11713]	CAMK2G	--->	FLNA	0.5684	-0.58821
[11714]	DNM2	<---	BAT1	-0.036138	0.024714
[11715]	ZBTB7A	?	BCL6	-0.085302	-0.085302
[11716]	CDK2	--->	RB1	0.98937	-1.0015
[11717]	CASP7	--->	HSPA5	0.10083	-0.20692
[11718]	KLK3	--->	A2M	0.14985	-0.11653
[11719]	OSR1	<---	TGFBR2	-0.63395	0.71543
[11720]	HDAC3	--->	YY1	0.098812	-0.092158
[11721]	MYST4	--->	RUNX2	0.036314	-0.020868
[11722]	SDCBP	--->	GRIA4	0.12457	-0.11938
[11723]	PRMT1	--->	WDR33	0.11437	-0.1224
[11724]	HTR2A	--->	MAP1A	0.14145	-0.13892
[11725]	PRKCD	--->	KLF5	0.49984	-0.49851
[11726]	GUCY1A2	?	GUCY1B3	-0.19281	-0.19281
[11727]	NCKIPSD	--->	WAS	0.3776	-0.43587
[11728]	FXR2	?	FXR1	-0.037462	-0.037462
[11729]	TRAF6	--->	TRAF4	0.24374	0.00080821
[11730]	CEBPA	<---	CREBBP	-0.18961	0.18576
[11731]	PLEC1	--->	SMAD9	0.28046	-0.29902
[11732]	CDKN1B	<---	YWHAH	-0.053185	0.053525
[11733]	DVL3	<---	NKD1	-0.9964	0.99226
[11734]	CDX2	?	HNF1A	-0.11417	-0.11417
[11735]	MEIS1	<---	HOXB1	-0.0094151	0.024726
[11736]	EPB41L3	<---	YWHAH	-0.057222	0.05792
[11737]	MAPK3	--->	FCGR2B	0.28348	-0.31361
[11738]	CSNK1A1	--->	HMGB1	0.62328	-0.5331
[11739]	DLG3	--->	ATP2B4	0.12627	-0.13697
[11740]	SORBS1	<---	CBL	-0.30431	0.29514
[11741]	PTPRC	--->	FCGR3A	0.33764	-0.33587
[11742]	TRIP6	<---	TLR2	-0.44278	0.45076
[11743]	RXRA	<---	NR1H3	-0.60548	0.60453
[11744]	MINK1	--->	ACTA2	0.060287	-0.066644
[11745]	RGS4	<---	PTAFR	-0.14893	0.16077
[11746]	FAM46A	<---	COL4A2	-0.043384	0.03632
[11747]	WASF1	<---	FYN	-0.74439	0.82271
[11748]	CDC25B	<---	YWHAH	-0.11975	0.14141
[11749]	HTR1B	<---	HTR1A	0.24405	0.31807
[11750]	JAK2	--->	CCR2	0.2955	-0.17451
[11751]	ARF1	--->	INSR	0.17741	-0.1864
[11752]	ANTXR2	<---	COL4A2	-0.042432	0.035371
[11753]	NRXN2	<---	INADL	-0.007542	0.044258
[11754]	HEY1	<---	MYOD1	-0.43036	-0.38951
[11755]	IGF2	--->	IGF1R	0.65928	-0.66772
[11756]	PIK3CD	<---	RALY	-0.28888	0.28312
[11757]	HDAC3	--->	CORO2A	0.16594	-0.13292
[11758]	GNA13	--->	CDH2	0.20416	-0.20669
[11759]	GADD45B	--->	ESR1	0.017932	0.0079001
[11760]	RGS19	--->	GNAO1	1.0012	-1.0015
[11761]	PPEF1	--->	CALM1	-0.12978	-0.15561
[11762]	SNTA1	<---	GRB2	-0.28662	0.30109
[11763]	PSMD4	--->	RASSF8	0.037791	-0.006083
[11764]	AR	<---	CDK7	-1.2513	1.2465
[11765]	KIT	--->	CISH	0.15769	-0.25081
[11766]	PRKCG	--->	YWHAE	0.90517	-0.90439
[11767]	NCOA3	<---	JUN	-0.11477	0.099237
[11768]	NXF1	--->	SFRS3	0.042109	-0.011155
[11769]	AKT1	--->	FOXO4	0.99774	-1.0046
[11770]	PARD3	--->	PLCB1	0.18721	-0.19438
[11771]	PLD1	<---	RHOA	-1.0031	0.99718
[11772]	FLII	--->	GRIP1	0.089851	-0.07441
[11773]	PRKAA2	--->	ACACA	0.14518	-0.16304
[11774]	MARK1	--->	MAP4	0.1044	-0.11787
[11775]	NCOA2	<---	THRB	-0.043163	0.034596
[11776]	SVIL	--->	MYH10	0.0053955	-0.013468
[11777]	UBXN1	<---	TGFBR1	-0.36563	0.33171
[11778]	TRPM7	--->	PLCB2	0.065296	-0.075433

[11779]	C3	--->	LRP1	0.09196	-0.10337
[11780]	CARD8	?	CARD18	-0.10452	-0.10452
[11781]	HTRA1	--->	LRP10	0.025963	-0.021142
[11782]	STK36	--->	GLI3	0.59875	-0.61141
[11783]	CALCR	<---	FLNA	-0.36241	0.36614
[11784]	YWHAZ	<---	ADRA2C	-0.35907	0.38003
[11785]	EP300	--->	TWIST1	0.069167	-0.079539
[11786]	ABL1	<---	PLSCR1	-0.085324	-0.080616
[11787]	ANGPT2	--->	ANGPT1	-0.006398	-0.0073307
[11788]	RNF123	--->	UBAC1	0.067864	-0.053686
[11789]	SSH1	--->	CFL1	0.3315	-0.32562
[11790]	PTK2	--->	ITGAV	0.016199	-0.038841
[11791]	EEF1G	<---	YWHAG	-0.064551	0.089473
[11792]	FLOT1	<---	FLOT2	-0.082537	-0.08228
[11793]	BLNK	<---	ITK	-0.24918	0.40931
[11794]	CALCOCO2	<---	FBXL18	-0.11749	0.15296
[11795]	FHL3	<---	MED15	-0.022915	0.024936
[11796]	BTK	<---	PRKCZ	-0.076653	0.070695
[11797]	SMAD3	--->	SKIL	0.0719	-0.06155
[11798]	FHOD1	<---	PRKG1	-0.63232	0.63128
[11799]	GRIN1	<---	RPS6KA3	-0.3417	0.25402
[11800]	NFKB1	--->	BCL3	0.48241	-0.0063102
[11801]	MEIS1	<---	HOXA2	-0.0094151	0.024726
[11802]	PURA	--->	RB1	0.47775	-0.47923
[11803]	PPARD	--->	RXRG	1.003	-0.99549
[11804]	YWHAZ	<---	PRKCA	-0.90439	0.90517
[11805]	USP9X	--->	MLLT4	0.26048	-0.21229
[11806]	CREBBP	--->	FOXO4	0.050393	-0.060941
[11807]	PTPN11	--->	PDGFRA	0.14519	-0.12516
[11808]	AKAP13	<---	GNA12	-0.042898	0.040587
[11809]	ARHGEF7	<---	PAK3	-0.068611	0.1051
[11810]	HOPX	<---	SRF	-0.11346	0.13352
[11811]	MAP3K2	--->	YWHAZ	0.54958	-0.30237
[11812]	MATN2	<---	COL1A1	-0.018425	0.0048494
[11813]	BTBD2	<---	COPS6	-0.11853	0.11901
[11814]	RAB25	--->	ITGB1	0.1695	-0.17904
[11815]	TP53	<---	SSTR3	-0.51498	0.52523
[11816]	RGS7	--->	MSX1	0.32661	-0.3149
[11817]	GPR77	<--->	C5AR1	0.49511	0.49511
[11818]	PTK2B	<---	SYK	-0.48288	0.50478
[11819]	TYRO3	--->	YES1	0.073301	-0.078384
[11820]	PIK3R1	<---	JAK2	-0.033749	0.049472
[11821]	NCOA3	<---	CCND1	-0.10595	0.093343
[11822]	CACNB4	<---	SYT1	-0.052901	0.093683
[11823]	HRAS	--->	PIK3R1	0.21	-0.30832
[11824]	BAIAP2	--->	BAI1	0.048945	-0.05317
[11825]	MYO5B	<---	RAB11B	-0.23098	0.24373
[11826]	IL16	--->	KCNJ10	0.044422	-0.0070376
[11827]	RPS6KA5	--->	HIST3H3	1.009	-0.99586
[11828]	EEF1G	<---	LARS	0.012637	0.022346
[11829]	SMURF2	--->	SMAD2	0.9934	-1.0067
[11830]	AMH	--->	AMHR2	0.11301	-0.13238
[11831]	KRT15	--->	ABI3	0.0016695	-0.0040478
[11832]	CCL3L3	<---	CCR5	-0.46475	0.49595
[11833]	AKT1	--->	IMPDH2	0.40617	-0.27011
[11834]	NCALD	--->	ACTB	0.33163	-0.32679
[11835]	FAS	<---	PTPN6	-0.246	0.45864
[11836]	MTNR1B	--->	IRS4	0.22622	-0.22287
[11837]	THBS1	<---	COL3A1	-0.033756	-0.0031145
[11838]	YWHAG	<---	PRKCB	-0.90439	0.90517
[11839]	UBQLN1	<--->	UBQLN4	0.069208	0.069208
[11840]	DAB1	--->	PLCG1	0.60126	-0.66968
[11841]	CDC25A	<---	RAF1	-0.05239	0.036016
[11842]	MAPK3	--->	GAB2	0.99738	-0.99704
[11843]	PTPRN	--->	PTPRS	0.31036	0.29284
[11844]	MCL1	<---	BCL2L11	-0.021317	0.020672
[11845]	DDX39	?	BAT1	-0.0081699	-0.0081699
[11846]	SH3GL3	<---	SH3KBP1	0.084895	0.094299
[11847]	CASP8	--->	AR	0.99767	-1.0153
[11848]	PRKACA	<---	NFKB1	-0.6564	0.6581
[11849]	CDK2	--->	CCNE1	1.0544	-1.0774
[11850]	PPM1A	<---	PRKCD	-0.39783	0.39982
[11851]	PARD6G	<---	PRKCI	-0.068765	0.075804
[11852]	SMAD2	<---	MAPK9	-1.0074	1.012
[11853]	TP53	<---	SERPINB9	-0.31559	0.32775
[11854]	YES1	--->	CSF1R	0.51153	0.27207

[11855]	MEF2C	<---	SP1	-0.054635	0.042379
[11856]	SRCAP	--->	NCOA2	0.011098	-0.023597
[11857]	RNF11	--->	HERC2	0.030264	-0.026939
[11858]	FCRL3	--->	ZAP70	0.59346	-0.55508
[11859]	NR2C2	?	RXRB	-0.0089162	-0.0089162
[11860]	CCR5	--->	CCL3	0.49595	-0.46475
[11861]	SNTA1	--->	ACTA2	0.53937	-0.53502
[11862]	PREB	--->	SMAD1	0.26668	-0.28606
[11863]	SYVN1	<---	ERN1	-0.22291	0.20338
[11864]	CAV2	--->	FLOT2	0.073391	-0.061294
[11865]	MAP3K7IP2	--->	XIAP	0.018402	-0.022438
[11866]	SMAD1	<---	SMAD3	-0.9962	1.0013
[11867]	TERF1	--->	TNKS2	0.041759	-0.024514
[11868]	SELE	<---	ACTN2	-0.084677	0.081032
[11869]	SHC4	<---	GRB2	-0.26269	0.30399
[11870]	AURKB	--->	HIST1H3A	1.0832	-1.0289
[11871]	PTPN11	--->	STAT3	0.73925	-0.96892
[11872]	SOX10	--->	TCF12	0.027151	-0.038902
[11873]	MAPKAPK5	<---	MAPK6	-0.45114	0.8761
[11874]	NR2E3	--->	RBL1	0.15905	-0.16975
[11875]	LAIR1	<---	CSK	0.015127	0.11748
[11876]	BMPR1B	--->	UBXN1	0.068762	-0.094577
[11877]	FADD	<---	FAS	-0.86277	0.89012
[11878]	NRCAM	--->	ANK2	0.073658	-0.087821
[11879]	GIT1	<---	PTPRZ1	-0.30123	0.29118
[11880]	TEC	--->	PRLR	0.031504	-0.046781
[11881]	SMURF2	--->	CUEDC1	0.70793	-0.71181
[11882]	GRIK5	<---	GRIK2	-0.080356	-0.020824
[11883]	UBE2Z	<---	PLEKHF2	-0.038275	0.045869
[11884]	MAP2K2	--->	MAPK3	1.0159	-0.99672
[11885]	ANTXR1	--->	BCL2L1	0.76389	-0.75628
[11886]	PTK2B	--->	DLG3	-0.03771	-0.067529
[11887]	HERC6	<---	HERC3	0.012937	0.13675
[11888]	VEGFC	<---	FLT4	-0.18233	0.21449
[11889]	TRPC1	<---	BMPR2	-0.18399	0.172
[11890]	PRKACA	--->	HAND1	1.5563	-1.5271
[11891]	PTPN6	--->	CTNNB1	0.37447	-0.37112
[11892]	CDK20	--->	GTF2IRD1	0.71989	-0.74424
[11893]	CELA1	--->	LPA	0.0012258	-0.0091243
[11894]	ABI2	<---	SH3KBP1	0.053384	0.1266
[11895]	CHN1	<---	CDK5	-0.43051	0.36149
[11896]	TRIM63	<---	PDK4	-0.22542	0.23742
[11897]	SERPINE2	<---	COL4A3	-0.055972	0.049204
[11898]	HSP90AA1	--->	NR3C1	0.037639	-0.046517
[11899]	SMAD4	--->	CTNNB1	0.17916	-0.18358
[11900]	EHMT2	<---	CCDC33	-0.17426	0.17443
[11901]	SMAD4	<---	CD59	-0.41306	0.494
[11902]	FGD4	--->	ACTA1	0.26597	-0.27543
[11903]	AKT1	--->	CASP9	1.0121	-1.0107
[11904]	AXL	--->	RANBP9	0.14469	-0.15016
[11905]	TAF1	<---	HMGB1	-0.050432	0.053694
[11906]	HOXA9	--->	MEIS1	0.024726	-0.0094151
[11907]	FOXA2	--->	HOXA5	0.42236	-0.39346
[11908]	CYTH2	<---	ARF1	-0.18769	0.20203
[11909]	ADIPOR2	<---	ADIPOQ	-0.60258	0.59916
[11910]	CTBP1	--->	HTT	0.057114	-0.063292
[11911]	WASF1	<---	ABL1	-0.085162	-0.01425
[11912]	CPNE7	--->	CALCOCO2	0.087747	-0.045677
[11913]	TNFSF11	--->	MMP3	0.44952	-0.42626
[11914]	APLP1	--->	ZNF24	0.040108	-0.050632
[11915]	PKD1	--->	ACTN1	-0.00071178	-0.00089714
[11916]	ESR1	<---	TRIM59	-0.16971	0.19453
[11917]	TRPC3	<---	PLCB1	-0.02414	0.011125
[11918]	EP300	--->	APEX1	0.18213	-0.18646
[11919]	CHFR	<---	AURKA	-0.3195	0.29617
[11920]	CREBBP	--->	JUN	0.12142	-0.13136
[11921]	CD96	--->	PVR	0.1212	-0.12415
[11922]	PTPN11	--->	STAT5A	1.0012	-1.0146
[11923]	HIF1A	--->	EP300	0.017606	-0.031011
[11924]	HIST4H4	<---	BRD7	-0.24229	0.29322
[11925]	AP3B1	--->	ARR3	0.019138	-0.031465
[11926]	S1PR3	--->	GNAQ	0.50718	-0.51255
[11927]	SVIL	--->	GNB2	0.23096	-0.17527
[11928]	PTK2	<---	ERBB3	-0.023945	0.12749
[11929]	PTPN11	--->	HNRNPU	0.79411	-0.60378
[11930]	STAT2	--->	STAT1	-0.054325	-0.09772

```
[11931] SMURF1  --->    WEE1    0.64008 -0.6445
[11932] NOTCH1  <---    XIAP    -0.4443 0.42781
[11933] EHD2    <---    EHBP1   -0.028219       0.043222
[11934] SOX8    <---    HHEX    -0.060464       0.063322
[11935] PHF21A  <---    RCOR1   -0.02785        0.043097
[11936] SYNJ1   <---    CDK5    -0.7292 0.70304
[11937] PSTPIP1 --->    WASF1   0.11644 -0.11432
[11938] MAP2K4  <---    MAP3K10 -0.99593        0.99711
[11939] CDC20   <---    ANAPC1  -0.048858       0.052121
[11940] ERBB4   --->    PIK3R2  0.18962 -0.22617
[11941] CD2AP   --->    PDCD6IP 0.22593 -0.22384
[11942] ZNF384  <---    PCBP1   -0.19523        0.19248
[11943] SMARCC2 <---    RELB    -0.38007        0.38113
[11944] BTK     --->    TLR8    0.12505 -0.1499
[11945] PPP1R12C        <---    MYL2    -0.59747        0.60975
[11946] SERPINB1        <---    ELANE   -0.082185       0.11833
[11947] SH3GL3  --->    DNM1    0.071535        -0.080916
[11948] PDZK1   --->    ABCC2   0.11782 -0.1184
[11949] PEX14   <---    SMARCC2 -0.024199       0.058944
[11950] CCNT2   --->    RB1     0.7707  -0.77141
[11951] F2      <---    ST13    -0.0042986      0.019461
[11952] WDR1    <---    SKIV2L  -0.076481       0.091156
[11953] PPP1R15A        --->    BAG1    0.19584 -0.18309
[11954] RIMS1   --->    CACNA1B 0.061971        -0.073003
[11955] TRAF6   --->    MAPK14  0.31518 -0.36843
[11956] CHEK2   --->    CDC25A  0.41667 -0.41209
[11957] HNRNPC  <---    PIN1    -0.30347        0.3333
[11958] SHB     <---    KDR     -0.64445        0.69239
[11959] AATF    --->    MPP6    0.004157        -0.0053605
[11960] PRSS2   --->    SERPINA1        0.11833 -0.082185
[11961] SYTL1   <---    RAB27A  -0.15731        0.20092
[11962] WASF2   <---    BTK     -0.52724        0.49182
[11963] CREBBP  --->    HOXA9   0.18505 -0.18943
[11964] KPNA3   <---    TGM2    -0.030402       0.062673
[11965] SHC1    <---    CSF3R   -0.16202        0.058351
[11966] CDKN1B  --->    CCND1   1.0016  -1.0151
[11967] BMP2    --->    ACVR1   0.60319 -0.60916
[11968] PPIE    <---    XAB2    -0.0025581      0.017908
[11969] PRMT1   --->    HNRNPA1 0.28801 -0.28644
[11970] MAPK1   <---    HSP90AA1        -0.056762       0.040193
[11971] SMAD6   <---    MAP3K7IP1       -0.74587        0.8096
[11972] SERPINA5        <---    COPS6   -0.32079        0.34926
[11973] PPFIA3  <---    PTPRF   -0.017812       0.012075
[11974] FASLG   --->    FNBP1   0.14108 -0.12462
[11975] ADAP1   --->    AP3B2   0.026167        0.0077654
[11976] PRKCI   --->    GAPDH   0.15501 -0.16874
[11977] MERTK   --->    LMO4    0.3361  -0.33121
[11978] LRG1    --->    FN1     0.086995        -0.070109
[11979] IRS2    --->    ATP2A1  0.013329        -0.025724
[11980] PGR     <---    POU2F1  0.012013        0.012582
[11981] CHUK    ?       IKBKB   -0.14626        -0.14626
[11982] SP1     --->    JUN     0.18922 -0.21438
[11983] HIF1A   --->    NCOA1   0.10864 -0.0075489
[11984] REL     <---    MAP3K8  -0.61546        0.60973
[11985] HP      --->    CD22    0.035416        -0.047476
[11986] LYST    --->    CALM1   0.21607 -0.1798
[11987] STK11   --->    MARK1   0.045757        -0.0047098
[11988] EGFR    --->    ATP1B1  0.28099 -0.28185
[11989] CAV1    <---    SOS1    -0.55582        0.55226
[11990] TAF5    <---    TAF15   -0.097664       0.11089
[11991] YWHAZ   --->    CDC25B  0.14141 -0.11975
[11992] DNAJC1  <---    HSPA5   -0.34012        0.34673
[11993] LASP1   --->    ACTA1   0.38007 -0.38414
[11994] LTBP1   <---    FBN2    -0.011353       -0.0099732
[11995] SIGLEC12        <---    PTPN11  -0.097899       0.11179
[11996] DGKZ    <---    SRC     -0.28135        0.31822
[11997] CGN     <---    YWHAG   -0.055704       0.079681
[11998] CDK4    --->    CDKN1A  0.51838 -0.55156
[11999] NR1H3   <---    NCOR1   -0.045434       0.038456
[12000] PTPRS   <---    PTPRE   0.29284 0.31036
[12001] KAT2B   --->    NR4A1   0.15014 -0.14993
[12002] JAK1    --->    FES     0.99431 -0.99719
[12003] RASD2   --->    TGFBR1  0.010625        -0.037483
[12004] PRKACA  <--->   AURKA   0.23429 0.23429
[12005] DLG2    --->    GRIN2B  0.026321        -0.034965
[12006] FNDC6   <---    IL20    -0.74442        0.74479
```

[12007]	PRKCG	--->	GRIA4	0.56969	-0.57379
[12008]	IRAK4	--->	MYD88	0.15296	-0.12108
[12009]	SYK	--->	PLCG1	1.0054	-1.0267
[12010]	CCL21	<---	UBQLN4	-0.085532	0.11982
[12011]	CDKN1B	<---	YWHAG	-0.053185	0.053525
[12012]	RALGDS	<---	KRAS	-1.0084	0.98547
[12013]	CASP3	--->	PRKDC	0.21872	-0.26808
[12014]	MLNR	--->	GHRHR	0.26344	-0.26115
[12015]	ATF3	--->	JUN	0.050278	-0.31682
[12016]	EMX2	--->	EIF4E	0.35218	-0.32897
[12017]	RAB11FIP4	<---	RAB11FIP2	-0.039559	0.080156
[12018]	NOTCH1	<---	GFI1B	-0.10361	0.08908
[12019]	PTPN11	--->	PILRB	0.60343	-0.52299
[12020]	GRIA4	<---	PICK1	-0.098237	0.11658
[12021]	GRIN2D	<---	MAP2	-0.055345	0.049371
[12022]	AKAP10	--->	PRKAR1A	0.049772	-0.026102
[12023]	BAZ2A	<---	TTF1	-0.015547	0.019774
[12024]	EGFR	--->	CAV2	0.27464	-0.28119
[12025]	E2F3	<---	SP1	-0.16629	0.15571
[12026]	RAP1A	<---	RASGRP2	-1.007	1.0003
[12027]	IL18R1	--->	IL1RAP	0.072678	0.052003
[12028]	ESR1	?	THRA	-0.0089162	-0.0089162
[12029]	SMAD1	<---	BMPR1A	-1.0029	1.0036
[12030]	CRKL	<---	PTPN11	-0.40446	0.29309
[12031]	DLL1	<---	NOV	-0.092151	0.079845
[12032]	ADAM17	--->	TGFA	0.024962	-0.020542
[12033]	LRP6	<---	AXIN1	-0.082628	0.071016
[12034]	ATR	--->	E2F1	-0.0011743	-0.0063021
[12035]	LCP2	--->	PLCG1	0.35777	-0.52615
[12036]	PHC1	<---	HSPA8	-0.35038	0.37267
[12037]	HRAS	--->	INSR	0.18395	-0.19271
[12038]	PRKCD	--->	CYTH1	1.033	-1.0023
[12039]	PAX6	<---	DLG1	-0.077532	0.071063
[12040]	IKZF4	<---	IKZF2	-0.022398	-0.01724
[12041]	LTBP3	--->	ITGB5	0.025738	-0.044308
[12042]	CTBP2	<---	AKTIP	-0.015082	0.0087197
[12043]	GADD45G	--->	PPARD	0.017932	0.0079001
[12044]	CDH2	<---	CREBBP	-0.077308	0.066916
[12045]	AUP1	--->	ITGA5	0.023371	-0.032435
[12046]	MAP4K5	--->	GRB2	0.21465	-0.13809
[12047]	PRKCA	--->	CBL	0.29229	-0.30458
[12048]	YWHAG	<---	ARAF	-0.051212	0.022119
[12049]	SRGAP1	<---	RHOA	-0.093021	0.10009
[12050]	FADD	<---	LRRCC1	-0.02892	-0.026271
[12051]	FGF2	--->	FGFR3	0.60861	-0.61087
[12052]	TRAF6	<---	PPP4C	-0.052608	0.040031
[12053]	WDR33	<---	EEF1G	-0.034345	0.038065
[12054]	SMAD3	<---	PLAG1	-0.067164	0.1114
[12055]	YWHAB	<---	TNFAIP3	-0.13918	0.13176
[12056]	SMAD4	--->	ATF2	0.11254	-0.11504
[12057]	DKK2	--->	LRP6	1.009	-1.0005
[12058]	ZBTB16	<---	FCHO1	-0.12508	0.12351
[12059]	PGR	<---	PIAS3	-0.090133	0.095399
[12060]	ZNF205	<---	CALCOCO2	-0.27634	0.27784
[12061]	COL2A1	--->	BMP2	0.10897	-0.11545
[12062]	TESK1	--->	CFL1	0.45348	-0.47476
[12063]	RUFY1	<---	EPHA3	-0.25658	0.25702
[12064]	SLC2A4	<---	UBE2N	-0.52623	0.55215
[12065]	JAK1	--->	STAT2	1.0942	-1.0882
[12066]	NOVA1	?	PCBP1	-0.037462	-0.037462
[12067]	GNA12	<---	PPP2R1A	-0.18467	0.18651
[12068]	ARHGEF7	--->	SHANK1	-0.0035135	-0.015617
[12069]	SPARC	--->	PLG	0.05947	-0.044241
[12070]	STARD13	--->	EPB41L1	0.070879	-0.067034
[12071]	DUSP22	--->	MAP2K7	1.011	-0.99819
[12072]	SORBS1	--->	MLLT4	0.15495	-0.13095
[12073]	ZYG11B	--->	TCEB1	0.042589	-0.027765
[12074]	LASP1	--->	ACTB	0.38007	-0.38414
[12075]	WIZ	--->	EHMT2	0.097045	-0.10329
[12076]	GSK3B	--->	AKAP11	0.70015	-0.72609
[12077]	SMAD3	<---	RANBP9	-0.20553	0.25035
[12078]	CCNA1	<---	CDKN1B	-1.0151	1.0016
[12079]	CCL20	--->	VCAN	0.010191	-0.021003
[12080]	CASP8	--->	STK24	0.99111	-0.99197
[12081]	FMR1	<---	RANBP9	-0.02118	0.03638
[12082]	SOCS1	<---	CSF1R	-0.40275	0.43856

```
[12083] MNT     ?       MAX     -0.65635        -0.65635
[12084] IKBKB   <---    TRAF2   -0.077891       0.024674
[12085] PIAS1   --->    PRPF40A 0.0091068       0.0015163
[12086] GRM5    --->    CALM2   0.31782 -0.27991
[12087] CCR1    --->    STAT3   0.62118 -0.67486
[12088] PPP2R2A --->    GRIN2D  0.15275 -0.14961
[12089] SRC     --->    GRLF1   0.37553 -0.33012
[12090] YWHAG   <---    PTPN14  -0.29759        0.28756
[12091] EIF2AK3 <---    HSP90AA1        -0.056762       0.040193
[12092] AKT2    --->    ESR1    1.0083  -1.0039
[12093] ESR1    --->    CTNNB1  0.32434 -0.32855
[12094] MAPK8IP1        --->    LRP2    0.25269 -0.25877
[12095] ATM     <---    PRKDC   -0.087809       -0.0069752
[12096] GCKR    --->    GCK     0.29277 -0.29204
[12097] TRAF6   --->    FHL2    0.99829 -0.99734
[12098] PRKD1   --->    YWHAQ   0.69446 -0.73102
[12099] DOCK7   <---    RHOA    -0.041827       0.031606
[12100] STAMBP  <---    GRB2    -0.15316        0.16138
[12101] NCK1    --->    ABL1    0.36174 -0.44357
[12102] L1CAM   --->    NCAM1   0.057605        0.022333
[12103] PTPN14  --->    JUP     0.079157        -0.091752
[12104] RPS6KA3 --->    ESR1    0.56871 -0.73191
[12105] BCAN    --->    MMP1    0.022421        -0.030194
[12106] LRSAM1  <---    GSK3A   -0.04069        0.020107
[12107] KLF10   <---    SP1     -0.043592       0.05218
[12108] RASD1   <---    GNAI1   -1.0143 1.0038
[12109] CCRL1   --->    CCL2    0.49595 -0.46475
[12110] RBBP7   <---    APPL2   -0.1817 0.18996
[12111] GNAI2   <---    CHRM1   -0.98627        0.9872
[12112] KCNB1   ?       KCNG1   -0.025901       -0.025901
[12113] DUSP22  --->    MAPK8   1.011   -0.99819
[12114] CEBPB   <---    NFKB1   -0.7732 0.78153
[12115] HSPA4   --->    OLR1    0.92153 -0.88169
[12116] CAV2    <---    DRD1    -0.43128        0.45604
[12117] PTPRU   --->    GNAI3   0.40483 -0.41277
[12118] CABP1   --->    ITPR3   0.32619 -0.3361
[12119] SH3D19  --->    SOS2    0.013507        -0.030722
[12120] RAB11FIP3       <---    ARF4    -0.47544        0.48818
[12121] COL8A2  <---    COL8A1  -0.0043908      0.011501
[12122] GRIN2D  <---    HSPA1A  -0.29516        0.29572
[12123] PAX6    --->    SHANK3  0.00068577      -0.01033
[12124] CSF3R   <---    LCK     -0.32405        0.36831
[12125] NLK     <--->   HIPK2   0.23429 0.23429
[12126] SERPINA10       <---    F10     -0.016469       0.01376
[12127] SMAD3   --->    ZNF329  0.12829 -0.12271
[12128] ACVRL1  <---    TGFBR1  -0.049446       -0.0024623
[12129] DSG3    <---    PKP3    -0.319  0.31989
[12130] NGFR    <---    MAG     -0.0541 0.10698
[12131] ATXN1   <---    GCA     -0.090004       0.082747
[12132] RTKN    <---    RHOC    -0.24337        0.24852
[12133] SOCS1   <---    JAK1    -1.0098 1.0118
[12134] ADAM12  --->    PIK3R1  0.10677 -0.17574
[12135] HIC2    <---    CCNT1   -0.11421        0.10909
[12136] SKIL    <---    TTRAP   -0.40379        0.40779
[12137] TNIP1   <---    TNFAIP3 -0.14096        0.14277
[12138] WWP2    --->    IL6R    0.12783 -0.13937
[12139] NCL     <---    NR3C1   -0.075993       0.075093
[12140] MSR1    <---    HSPA1A  -0.47081        0.49104
[12141] NTRK3   <---    NTF3    -0.13418        0.13151
[12142] HNRNPK  <---    RBM41   -0.0048144      0.036464
[12143] TBP     <---    WDR62   -0.008057       -0.0042132
[12144] INSM1   --->    MPP3    0.04194 -0.035101
[12145] RAB1A   --->    MAPRE3  0.4246  -0.39396
[12146] ZEB2    <---    CTBP1   -0.1174 0.10744
[12147] BMX     --->    RUFY1   0.47808 -0.56023
[12148] EP400   <---    TRRAP   -0.0067065      -0.0057168
[12149] LTBP1   --->    IGFBP3  0.080676        -0.11651
[12150] IL12RB2 <---    JAK2    -1.013  1.001
[12151] RAP1A   --->    BIN1    0.16316 -0.15105
[12152] BGN     <---    TNF     -0.15485        0.17426
[12153] HEY2    <---    HDAC1   -0.24383        0.24931
[12154] TOM1L1  <---    RAB18   -0.37208        0.4027
[12155] PTK2    <---    SYK     -0.48288        0.50478
[12156] PRKCG   --->    CDC42   1.0123  -0.99154
[12157] CSNK2A1 --->    ABCA1   0.16549 -0.18176
[12158] PBX1    --->    HOXB8   -0.059044       -0.084876
```

[12159]	PLCG1	<---	ERBB2	-0.39155	0.3832
[12160]	ATXN1	<---	PIM2	-0.2064	0.18965
[12161]	BCAR1	--->	CRKL	1.0122	-0.99965
[12162]	TOLLIP	<---	TLR4	-0.17215	0.12701
[12163]	ITGB3	<---	NID1	-0.0094892	-2.0965e-005
[12164]	CSNK1D	--->	GJA1	0.20086	-0.17844
[12165]	RHOH	<---	KCNH2	-0.025535	0.039355
[12166]	VASP	--->	WAS	-0.019182	-0.1162
[12167]	RPS6KB1	<---	RAC1	-0.56975	0.3588
[12168]	PICK1	--->	GRIA1	0.33891	-0.33666
[12169]	CBX4	<---	FHL1	-0.0059015	0.041217
[12170]	TEP1	--->	TP53	0.18707	-0.18591
[12171]	RXRA	<---	GADD45A	-0.21826	0.0082071
[12172]	RIPK1	--->	EGFR	0.29953	-0.27657
[12173]	PRKCB	--->	DVL2	1.0137	-0.99339
[12174]	NAGPA	--->	EPS15	0.0048128	-0.018258
[12175]	SKIL	<---	PSG3	-0.305	0.29773
[12176]	SPHK1	<---	LYN	-0.90413	1.0649
[12177]	CHD3	<---	PTPRS	-0.029298	0.016058
[12178]	RB1	<---	DNMT1	-0.30503	0.29862
[12179]	ITGA2B	<---	CIB1	-0.010033	-0.00021457
[12180]	SETDB1	<---	MOBKL3	-0.17387	0.18846
[12181]	GABARAP	<---	TFRC	-0.01925	0.011502
[12182]	LY6G6F	<---	GRB7	-0.21503	0.12732
[12183]	CNOT1	--->	PPARG	0.0039289	-0.002982
[12184]	CSF2RB	<---	EPOR	-0.10715	0.013527
[12185]	CYTIP	<---	CYTH2	-0.22934	0.25364
[12186]	CASP10	--->	CASP3	0.99569	-0.9907
[12187]	AKT1	--->	GATA2	0.49316	-0.34165
[12188]	TGIF1	--->	SMAD2	0.16064	-0.11329
[12189]	PCBP1	<---	CUGBP2	-0.0048144	0.036464
[12190]	CD3E	?	CD3D	-0.22052	-0.22052
[12191]	FOXP4	?	FOXP2	-1.9914e-005	-1.9914e-005
[12192]	EPS15	<---	AGFG1	-0.085978	0.075724
[12193]	SIGIRR	<---	IRAK1	0.026746	0.056796
[12194]	WIPI1	<---	ESR2	-0.0096005	0.034681
[12195]	GSK3A	--->	MAPT	0.38658	-0.41618
[12196]	TEP1	--->	TERT	0.012117	-0.02246
[12197]	RCHY1	--->	AR	1.1462	-1.1482
[12198]	FUBP3	--->	QKI	-0.0053675	-0.11083
[12199]	EP300	--->	GATA5	0.18629	-0.19036
[12200]	TRIB2	--->	UBQLN4	0.6472	-0.67308
[12201]	NCF1	<---	NOXA1	0.010724	0.030471
[12202]	MATN2	--->	FBN2	-0.0044429	-0.011459
[12203]	FHOD1	<---	RAC1	-0.22543	0.23844
[12204]	DVL1	<---	USP13	-0.23594	0.23053
[12205]	PRKCB	--->	GRIN2B	0.22109	-0.2311
[12206]	GIPC1	--->	LRP1	0.14805	-0.15706
[12207]	SIK1	<---	CRTC2	-0.075794	0.069802
[12208]	BMP4	<---	ZFYVE9	-0.13467	0.12939
[12209]	WNK1	--->	SYT2	0.28469	-0.30397
[12210]	VAV2	<---	CD19	-0.09306	0.053576
[12211]	YWHAG	--->	SON	0.046568	-0.037524
[12212]	SQSTM1	<---	PRKCI	-0.060181	0.075293
[12213]	ERBB2IP	--->	SMAD4	0.17801	-0.15234
[12214]	HOXA9	--->	NFKBIA	0.74842	-0.75369
[12215]	GRK6	--->	EDNRA	0.31971	-0.40764
[12216]	GNA13	--->	RDX	0.092987	-0.095447
[12217]	NPR3	<---	NPPA	-0.0068276	0.041351
[12218]	GRK5	--->	AVPR1B	0.21152	-0.27051
[12219]	NFKB2	--->	NFKBIB	-0.0066871	-0.019218
[12220]	EP300	--->	ELK1	0.11551	-0.12289
[12221]	PPP2R1B	--->	STRN3	0.088301	-0.094979
[12222]	INHBC	?	INHBB	-0.061425	-0.061425
[12223]	STAT5B	<---	NCOR2	-0.077181	0.063117
[12224]	ATN1	--->	EFEMP1	0.039508	-0.041614
[12225]	SUV39H1	--->	RB1	0.16254	-0.17109
[12226]	EP300	--->	ETV1	0.18812	-0.1922
[12227]	SOX10	<---	SP1	-0.041897	0.03089
[12228]	BEGAIN	<---	DTNB	0.0093216	0.02481
[12229]	IRS4	<---	SOCS7	-0.080805	0.078985
[12230]	RYBP	<---	CASP8	-0.69428	0.55287
[12231]	ITCH	--->	TRPC4	0.00058429	-0.014424
[12232]	CSNK1A1	--->	NFATC3	0.37393	-0.34882
[12233]	CARD11	<---	PRKCQ	-1.0125	1.004
[12234]	CSPG4	--->	GRIP1	0.027225	0.0095843

[12235]	IFNGR2	<---	IFNGR1	-0.42221	-0.22376
[12236]	JAM3	--->	ITGAM	0.025067	-0.038226
[12237]	P2RY12	--->	GNAI2	0.99249	-0.98951
[12238]	EPB41L1	<---	YWHAZ	-0.057222	0.05792
[12239]	KIT	--->	STAT5A	0.99911	-0.99301
[12240]	NFKB1	<---	FBXW11	-0.99914	1.0055
[12241]	INHBB	?	INHBA	-0.061425	-0.061425
[12242]	DVL3	<---	PITX1	-0.21121	0.20498
[12243]	PRKCD	--->	NCF4	0.51755	-0.53024
[12244]	LCP2	<---	PTPRC	-0.46882	0.39702
[12245]	GDF9	<---	SMN1	-0.12987	0.14807
[12246]	ZAP70	--->	DBNL	0.9915	-0.99905
[12247]	SMURF2	--->	TRAF2	0.14265	-0.15283
[12248]	IL16	--->	KCND1	0.050181	-0.013537
[12249]	KCNJ16	?	KCNJ15	-0.01825	-0.01825
[12250]	BMP15	?	GDF9	-0.24667	-0.24667
[12251]	MAPK14	<---	PTPN7	-0.33377	0.17221
[12252]	DVL2	<---	RBPMS	-0.38332	0.3831
[12253]	CCNE1	<---	CALM1	-0.42212	0.4376
[12254]	RIPK1	<---	ZFAND5	-0.14554	0.33696
[12255]	SNX9	--->	ADAM15	0.021174	-0.025517
[12256]	SMAD2	--->	RBL1	0.069298	-0.075304
[12257]	KAT2B	--->	TWIST1	0.12586	-0.13396
[12258]	SLC2A4	<---	BSG	-0.55581	0.55255
[12259]	ZNF592	--->	SMAD9	0.1603	-0.17676
[12260]	CHEK2	--->	PPP2R2A	0.22227	-0.19469
[12261]	TRAF4	<---	BCKDK	-0.026516	0.025517
[12262]	JAK2	--->	HES5	0.17918	-0.11126
[12263]	BCL11B	<---	SUV39H1	-0.053963	0.044342
[12264]	PRKCD	--->	ITGA6	0.096253	-0.11177
[12265]	MEIS1	<---	HOXA7	-0.0094151	0.024726
[12266]	PRKCB	--->	CHUK	0.99661	-0.98908
[12267]	CAV1	<---	NGFR	-0.037673	0.21837
[12268]	CBLB	--->	PLCG2	0.075994	-0.10984
[12269]	RB1	<---	EP300	-0.061481	0.049731
[12270]	SQSTM1	<---	IRAK1	-0.1075	0.1057
[12271]	CSNK2A1	--->	ZHX1	0.5908	-0.60913
[12272]	SENP2	--->	AXIN1	0.19527	-0.19802
[12273]	MAP3K2	--->	PKN2	0.11596	-0.1344
[12274]	SRC	--->	SPTAN1	0.25187	-0.12836
[12275]	SNIP1	<---	SP100	-0.054499	0.070358
[12276]	HAND2	<---	CENPB	-0.49313	0.51667
[12277]	JUB	--->	ACTA1	0.54878	-0.54521
[12278]	LHX3	<---	RLIM	-0.061039	0.057332
[12279]	CASP3	--->	MCL1	0.92594	-1.0473
[12280]	DBP	?	TEF	-0.11448	-0.11448
[12281]	SLC2A4	<---	GAPDH	-0.20452	0.21502
[12282]	FURIN	--->	ADAMTS1	-0.00093443	-0.0067844
[12283]	CDK1	--->	PLEC1	0.72542	-0.75118
[12284]	TIFA	<---	SDCBP2	0.0083082	0.026262
[12285]	TCL1A	--->	AKT2	0.99137	-0.99692
[12286]	PAPPA2	--->	SMAD4	0.41247	-0.32956
[12287]	PLSCR1	--->	OGDH	0.11635	-0.088429
[12288]	ADAM15	<---	LCK	-0.47182	0.5171
[12289]	EIF3B	?	EIF3G	-0.016298	-0.016298
[12290]	PTPN6	--->	KIR2DL5A	0.35299	-0.23556
[12291]	ICAM1	--->	FGG	0.11036	-0.10822
[12292]	PPEF2	--->	CALM2	-0.12978	-0.15561
[12293]	CD2AP	<---	RAB4A	-0.0073743	0.0097879
[12294]	SMAD3	<---	SPTBN1	-0.030643	0.038981
[12295]	SLC16A2	--->	THRA	0.039128	-0.028872
[12296]	COL4A3	--->	APP	0.044125	-0.057522
[12297]	PTPRF	--->	JUP	0.017356	-0.029876
[12298]	E2F4	<---	BRCA1	-0.03902	0.053404
[12299]	SYTL4	<---	RAB8A	-0.15731	0.20092
[12300]	FYN	--->	GRB10	0.85304	-0.6448
[12301]	MITF	<---	EP300	-0.084188	0.074054
[12302]	TGFBR1	--->	TNNT1	0.31242	-0.36838
[12303]	JAK2	--->	CSF3R	0.59532	-0.53917
[12304]	TEAD1	--->	MEF2C	0.12802	-0.13023
[12305]	SIM2	<---	PARK2	-0.042945	0.058397
[12306]	CASP3	--->	PKN1	0.066252	-0.14308
[12307]	MMP7	?	MMP1	-0.12951	-0.12951
[12308]	SF1	--->	DDX17	0.0075185	-0.010781
[12309]	NCOA1	<---	RXRB	-0.043163	0.034596
[12310]	NBEA	<---	PRKAR2A	-0.18426	0.18669

[12311]	PAX3	--->	DAXX	-0.0059114	-0.016407
[12312]	DYRK2	--->	HIST2H2BE	1.0832	-1.0289
[12313]	FZD8	<---	WNT1	-0.15329	0.1489
[12314]	CENPB	--->	GFI1B	0.14001	-0.14588
[12315]	GRASP	--->	GABBR2	0.0626	-0.025484
[12316]	RGS5	--->	GNAI3	1.0012	-1.0015
[12317]	PAX6	<---	CDX2	-0.099649	-0.043846
[12318]	APBA1	--->	CACNA1B	0.062773	-0.074105
[12319]	CSF2RA	<---	CHUK	-0.37913	0.24345
[12320]	BMPR1B	<---	RRAS2	-0.53994	0.52577
[12321]	RAB27A	--->	MYO5A	0.24373	-0.23098
[12322]	YWHAZ	<---	STK38	-0.2472	0.13548
[12323]	KCNIP1	--->	KCND1	0.28353	-0.26746
[12324]	PRPF40A	--->	MECP2	0.42102	-0.38693
[12325]	RAB5A	--->	APPL1	0.46313	-0.43085
[12326]	PTK2	<---	EGFR	-0.023945	0.12749
[12327]	MBD1	--->	SETDB1	-0.016907	-0.1792
[12328]	TRIM24	--->	VDR	0.11471	-0.088819
[12329]	EZH2	--->	HDAC1	0.039173	-0.022235
[12330]	CRKL	--->	PIK3R1	0.0060627	-0.3721
[12331]	PRKCD	--->	PLCB1	0.21205	-0.21981
[12332]	ZBTB16	<---	CDK1	-0.3943	0.37815
[12333]	FURIN	<---	PDGFA	-0.012903	0.0097438
[12334]	GRIN2D	<---	PRKCE	-0.19531	0.17016
[12335]	ARFGAP1	<---	ARF1	-0.42695	0.45328
[12336]	YWHAG	--->	IRS4	0.08899	-0.088202
[12337]	BAZ1B	<---	SMARCA1	-0.0096565	0.0023298
[12338]	IKZF5	<---	IKZF2	-0.17388	-0.0079103
[12339]	DDR1	--->	SNRNP40	0.27759	-0.29046
[12340]	PRKACA	--->	CFTR	1.0007	-1.0004
[12341]	BOC	--->	CDH2	0.12937	-0.13991
[12342]	SHANK2	--->	CTTN	0.034159	-0.035953
[12343]	HDAC4	--->	RUNX2	0.075349	-0.040912
[12344]	ARF1	--->	PICK1	0.45084	-0.42186
[12345]	YWHAG	<---	HSPA9	-0.739	0.77111
[12346]	LMO4	--->	NHLH1	0.62562	-0.58677
[12347]	KHDRBS1	<---	NCK1	-0.40252	0.39608
[12348]	GNA12	<---	DRD5	-1.008	1.0129
[12349]	POU2F1	<---	FDXR	-0.014945	0.047645
[12350]	HIPK3	--->	LIMS2	0.5736	-0.58259
[12351]	SYK	--->	DBNL	0.9915	-0.99905
[12352]	BACH1	--->	PATZ1	0.022194	-0.15821
[12353]	MICAL3	<---	RAB1B	-0.22699	0.24091
[12354]	ACVR2B	<---	IGSF1	-0.058494	0.038789
[12355]	ADAP1	<---	PRKCZ	-0.2732	0.2596
[12356]	SYNJ1	<---	EPHB2	-0.37811	0.3712
[12357]	SFRP1	--->	WNT4	0.99043	-0.98922
[12358]	CDC42EP1	<---	PLSCR1	-0.15058	0.063008
[12359]	SOCS1	--->	TCEB1	0.024927	-0.0040225
[12360]	TRIM23	--->	ZNF581	0.23231	-0.23626
[12361]	IL16	--->	GRIN2C	0.11324	-0.11391
[12362]	ACVR1	<---	RASD2	-0.32935	0.31819
[12363]	PRKCQ	--->	MSN	0.089314	-0.09498
[12364]	BAD	--->	BCL2A1	0.2727	-0.28245
[12365]	MAPK1	--->	PLCB1	1.0018	-1.001
[12366]	DTX3	<---	DTX1	0.0017841	0.044917
[12367]	FRMPD4	<---	STK36	-0.19542	0.17957
[12368]	PTPN6	--->	CD22	0.31368	-0.29575
[12369]	SIX3	<---	TLE1	-0.0020443	0.0047914
[12370]	MOBKL3	<---	ARHGEF15	-0.18656	0.21963
[12371]	RYR1	<---	CALM1	-0.088015	0.075894
[12372]	BCAR1	--->	CSPG4	0.017459	-0.014935
[12373]	CNN1	<---	PRKCE	-0.68634	0.61876
[12374]	SNX18	<---	SOS1	-0.33072	0.32391
[12375]	KAT5	--->	APP	0.21705	-0.22856
[12376]	COL4A6	--->	MMP9	0.072684	-0.078498
[12377]	SMAD3	<---	HSPA4	-0.63072	0.71662
[12378]	HDAC1	--->	CDH1	0.09416	-0.096836
[12379]	CTNNA1	--->	DLG1	0.14625	-0.15174
[12380]	SMAD2	<---	PAPPA	-0.32956	0.41247
[12381]	ANK2	<---	DNAJB1	-0.070269	0.0565
[12382]	MAGI3	<---	PLXNB3	-0.028004	0.038014
[12383]	EP300	--->	USF2	0.19969	-0.20391
[12384]	PARK2	<---	CASK	-0.11921	0.10424
[12385]	MAPK10	--->	STMN2	0.60216	-0.70167
[12386]	BHLHA15	--->	TCF3	-0.13552	-0.39218

```
[12387] DLG1    --->    EZR     0.031325        -0.041318
[12388] ELK1    <---    BRCA1   -0.15727        0.17622
[12389] CD247   <---    FYN     -0.98519        0.99791
[12390] PRKCD   --->    YWHAZ   0.77791 -0.76873
[12391] FASLG   <---    GRB2    -0.056691       0.072042
[12392] CDK9    --->    HTATSF1 0.72994 -0.75755
[12393] PPARA   <---    PRKCD   -0.53137        0.52878
[12394] MTNR1B  --->    YWHAQ   0.38003 -0.35907
[12395] ACTL6A  <---    SMARCA4 -0.048903       0.04085
[12396] SLC8A2  <---    YWHAB   -0.092105       0.10171
[12397] UBE2I   --->    NR3C1   0.1925  -0.16643
[12398] GRB10   <---    INSR    -0.17642        0.14373
[12399] SP1     <---    SMAD3   -0.99936        0.98558
[12400] TP53    <---    ERCC3   -0.3537 0.36508
[12401] PABPC4  --->    GDF9    0.23998 -0.20333
[12402] HMGB1   --->    HES1    0.31653 -0.32111
[12403] CARD17  ?       CARD18  -0.10452        -0.10452
[12404] EFEMP2  <---    APEX2   0.0042499       0.030525
[12405] ARRB2   <---    CXCR4   -0.25831        0.27159
[12406] CHUK    --->    CTNNB1  0.14295 -0.16439
[12407] PIAS4   --->    GADD45G 0.17487 -0.16315
[12408] FGD1    <---    CDC42   -0.061461       0.05557
[12409] NR2E3   <---    HDAC3   -0.067996       0.094064
[12410] SKIL    <---    USP25   -0.40447        0.40852
[12411] SLAMF6  <---    PTPN6   -0.54137        0.63132
[12412] IKBKB   --->    NFKB1   1.001   -1.0129
[12413] MAPKAPK2        <---    AKT1    -0.60008        0.60882
[12414] AHR     <---    NR2F1   -0.17306        0.18451
[12415] PPEF2   --->    CALM3   -0.12978        -0.15561
[12416] PPP1CA  --->    BCL2L1  0.29213 -0.30359
[12417] AR      <---    CASP3   -1.0225 1.0094
[12418] PLEKHO1 <---    CSNK2A1 -0.5885 0.44589
[12419] FOXO3   --->    SIRT1   0.00087169      -0.012205
[12420] STAT5B  <---    MAPK1   -0.80262        0.7916
[12421] CSF1R   --->    GRB2    1.0022  -0.99864
[12422] YWHAB   --->    BRAF    0.18212 -0.19664
[12423] RANBP10 <---    MEGF10  -0.0087767      -0.0046328
[12424] PRX     <---    DRP2    -0.1455 0.14851
[12425] TGFBRAP1        <---    ACVR2B  -0.22409        0.081783
[12426] CASP3   --->    CDC42   0.36319 -0.47633
[12427] TRIP6   --->    CATSPER1        0.0657  -0.030347
[12428] ARFGAP1 <---    CYTH2   -0.23435        0.25616
[12429] PBX2    <---    PBX1    -0.072035       -0.071956
[12430] COPS5   --->    CUL5    0.02459 -0.017361
[12431] DMD     ?       UTRN    -0.0066167      -0.0066167
[12432] HNRNPUL1        <---    PRPF40A -0.17178        0.18586
[12433] NFIC    <---    ZCCHC14 -0.38046        0.39957
[12434] EXOC2   <---    RALA    -0.98466        1.0001
[12435] IKBKB   <---    EIF2AK2 -1.003  1.0117
[12436] SRC     --->    YWHAH   1.0093  -0.89808
[12437] NTF3    --->    BDNF    0.055518        -0.063078
[12438] ZRSR2   --->    U2AF2   0.061943        -0.030095
[12439] ABCC9   <---    EEF1G   -0.062258       0.067271
[12440] ABCC2   <---    PDZD3   -0.1184 0.11782
[12441] SIRT1   --->    NR1H2   0.083315        -0.090495
[12442] CCR5    --->    CCL4    0.49595 -0.46475
[12443] SETDB1  <---    GPS2    -0.23511        0.25044
[12444] FBLN1   --->    VCAN    0.0093299       -0.020156
[12445] CBL     --->    LTK     0.51963 -0.53202
[12446] PTK2    --->    PXN     1.009   -0.99895
[12447] DLG4    <---    ADRB1   -0.093156       0.082793
[12448] RAPGEF1 --->    RHOQ    1.0045  -0.99301
[12449] MAP2K6  --->    MAPK14  1.0074  -0.9992
[12450] GIPC1   <---    LHCGR   -0.41277        0.44636
[12451] MMP2    <---    COL18A1 -0.040076       0.031923
[12452] GLS2    <---    CASK    -0.27103        0.25819
[12453] LAMA5   <---    MEP1A   -0.014379       0.0073722
[12454] BSPRY   --->    ZYX     0.012004        0.004138
[12455] CEBPB   --->    EGR1    0.15068 -0.12791
[12456] DOCK7   <---    CDC42   -0.041827       0.031606
[12457] MMP2    <---    IL1B    -0.18207        0.18897
[12458] YWHAQ   <---    MAP3K3  -0.30237        0.54958
[12459] ROCK1   <---    LIMK2   -0.053928       0.039587
[12460] KCND2   <---    KCNIP1  -0.26746        0.28353
[12461] DBNL    <---    SH2D4A  -0.40866        0.39487
[12462] PDLIM5  <---    PRKCE   -0.39954        0.3589
```

[12463] PTK2B <--- MATK -0.65057 0.81773
[12464] GRAP2 ---> FASLG 0.072042 -0.056691
[12465] SNX18 <--- SOS2 -0.33072 0.32391
[12466] UBE2D3 ---> RNF5 0.12227 -0.11995
[12467] CFB <--- C3 -0.067025 0.066153
[12468] IKZF1 <--- UBE2I -0.18962 0.20545
[12469] UBE2I ---> SALL1 0.35582 -0.35511
[12470] CSF2RB <--- FES -0.40477 0.32212
[12471] PTCH1 <--- SHH -1.0096 0.99953
[12472] EP300 ---> MN1 0.21428 -0.21816
[12473] COL4A6 ---> FBLN2 0.035051 -0.041937
[12474] DLG2 <--- LIN7A -0.020783 0.0077703
[12475] NGFR ---> SORT1 0.21289 -0.028353
[12476] PECAM1 <--- YES1 0.015127 0.11748
[12477] TNNI3K <--- MYBPC3 -0.023154 -0.011189
[12478] RXRA <--- PPARA -0.99549 1.003
[12479] EPHB2 ---> FOS 0.19129 -0.25278
[12480] BRD4 ---> CCNT2 0.27778 -0.26313
[12481] IL6ST <--- JAK1 -0.20912 0.1933
[12482] CCNG1 <--- CDK5 -0.75888 0.73793
[12483] SMAD2 <--- STAMBP -0.99775 0.99487
[12484] SMAD5 ---> HOXD13 0.010712 -0.04291
[12485] SH2B2 <--- INSR -0.14528 0.092287
[12486] MYC <--- CEBPA -0.23335 0.24948
[12487] SHC1 <--- IL2RB -0.29211 0.11736
[12488] ADAM17 ---> NOTCH1 0.99876 -1.0036
[12489] POU1F1 ---> ETS1 0.19026 -0.17089
[12490] SOS2 <--- ABL1 -0.14615 0.12333
[12491] LIN7B ---> KCNJ4 0.044422 -0.0070376
[12492] TCF4 <--- ID2 -0.37599 -0.054725
[12493] IGF1R <--- MAP3K5 -0.015817 0.036421
[12494] CSK <--- SRC -0.016792 0.60459
[12495] ENAH <--- ABI3 -0.01221 -5.6818e-005
[12496] RBBP4 <--- SP1 -0.10639 0.095829
[12497] NEK9 ---> SSRP1 0.56249 -0.55527
[12498] GRB7 <--- MAP3K14 -0.34335 0.17357
[12499] PPBP <--- CXCR2 -0.46475 0.49595
[12500] DHX9 ---> SMN1 0.0045634 -0.00071951
[12501] RNF216 <--- UBE2L6 -0.15877 0.19238
[12502] HOXA9 <--- TRIP6 -0.10746 0.14086
[12503] SMARCE1 <--- RELB -0.21955 0.2145
[12504] IVNS1ABP <--- SYNCRIP -0.036142 0.066603
[12505] STAT6 <--- HIST2H3C -0.29268 0.27166
[12506] PIK3R3 ---> IRS1 0.043693 -0.0050431
[12507] RIMS1 <--- RAB3A -0.0067445 0.016184
[12508] RAPGEF5 <--- RAP1A -0.99657 0.99903
[12509] RIMS2 ---> CACNA1S 0.061971 -0.073003
[12510] MECP2 ---> SKI 0.22212 -0.20309
[12511] NEK1 ---> YWHAH 0.62776 -0.66368
[12512] MAG ---> COL2A1 0.0068841 -0.019836
[12513] ACTN2 <--- CAPN1 0.022046 0.094446
[12514] CEBPA <--- EP300 -0.18961 0.18576
[12515] CEBPB ? DDIT3 -0.11448 -0.11448
[12516] SVIL <--- HSP90AA1 -0.049261 0.044391
[12517] BRCA1 ---> ABL1 -0.030619 -0.065362
[12518] MAPKAPK5 <--- MAPK12 -0.53091 0.73174
[12519] TRAF6 ---> MAPK8 0.98609 -1.0149
[12520] GRM5 ---> CALM3 0.31782 -0.27991
[12521] NR2E3 <--- CDK9 -1.0079 0.99834
[12522] PRSS2 ---> APP 0.36186 -0.37181
[12523] STAT1 ---> EIF2AK2 1.0031 -1.0075
[12524] CEBPE ---> GATA1 0.050146 0.007993
[12525] GADD45G <--- PTPRK -0.40004 0.39301
[12526] YWHAH <--- GREM1 -0.15683 0.1778
[12527] IKBKB <--- TGFBR1 -0.18636 0.21752
[12528] CCL28 <--- CCR10 -0.46475 0.49595
[12529] PDCD6IP <--- EGFR -0.28117 0.28029
[12530] MBD2 <--- CREBBP -0.18934 0.18507
[12531] PPP1CA ---> AKAP1 0.13605 -0.14578
[12532] STARD13 ---> HMGCR 0.22505 -0.22236
[12533] DAPK1 ---> UNC5C 0.004408 -0.013127
[12534] CAPN1 ---> BCL2L1 0.32381 -0.33086
[12535] YWHAB <--- CRTC2 -0.070272 0.071582
[12536] PELI2 <--- IRAK1 -0.98981 1.0139
[12537] MAPK3 ---> STMN1 1.0058 -0.99021
[12538] BCAR1 ---> PKD1 0.011253 -0.01521

```
[12539] IL2RB   <---    LYN     -1.172  1.2619
[12540] CDKN1C  <---    CDK4    -0.77518        0.74419
[12541] PPP2CA  --->    CAD     0.27734 -0.28429
[12542] PRKCA   --->    DNM1    0.28727 -0.25947
[12543] NGFR    <---    GRB2    -0.053252       0.15828
[12544] FIGF    <---    KDR     -0.28835        0.34247
[12545] CTBP2   --->    TEAD3   0.041222        -0.047403
[12546] PTPRK   --->    CTNNB1  0.13525 -0.14608
[12547] HIST2H2AA3      <---    UBE2H   -0.38612        0.43703
[12548] SOCS1   --->    VAV1    0.35622 -0.34335
[12549] JUNB    <---    BRCA1   -0.32745        0.29484
[12550] TGFB1   --->    THBS1   0.029924        -0.023311
[12551] TRAF2   <---    CD27    -0.53992        0.5393
[12552] MDM2    --->    ESR1    0.14626 -0.15006
[12553] PRKCE   --->    SLC4A3  0.61534 -0.68295
[12554] IRAK2   <---    IRAK1   -1.0016 0.99899
[12555] GP6     <---    YES1    0.015127        0.11748
[12556] YWHAZ   <---    TBXA2R  -0.35907        0.38003
[12557] ETS2    <---    CREBBP  -0.12299        0.11504
[12558] CREBBP  --->    HDAC3   0.18168 -0.186
[12559] ZNF148  <---    EP300   -0.12213        0.11396
[12560] BAG1    <---    HSPA1A  -0.92976        0.96516
[12561] CDC5L   --->    PPP1R8  0.018619        0.015243
[12562] FURIN   --->    ZP3     0.041432        -0.037698
[12563] CSK     --->    GJA1    0.27853 -0.25076
[12564] TP53    <---    NCL     -0.11263        0.10757
[12565] EIF2AK2 <---    TYK2    -0.17059        0.15428
[12566] CASP7   --->    XIAP    0.30534 -0.38127
[12567] CDC25C  <---    YWHAQ   -0.11975        0.14141
[12568] IL7R    --->    STAT5A  0.17619 -0.19577
[12569] YWHAG   --->    USP8    0.022808        0.0038773
[12570] GATA4   --->    MEF2C   0.10493 -0.10601
[12571] CEBPB   <---    HOMER3  -0.29828        0.30646
[12572] PTPN6   <---    CBL     -0.39261        0.40648
[12573] EPHB2   --->    GRB2    0.18974 -0.30775
[12574] DLST    <---    FLII    -0.080007       0.094487
[12575] INHBC   ?       INHBA   -0.061425       -0.061425
[12576] JUN     <---    ETS1    -0.16285        0.13316
[12577] NCOA1   <---    RXRA    -0.028914       -0.011576
[12578] SETDB1  <---    PLEKHA4 -0.32974        0.34069
[12579] TAP2    ?       TAP1    -0.071121       -0.071121
[12580] RGS7    --->    GNAI1   0.60077 -0.60607
[12581] C5AR1   --->    ARRB1   0.27159 -0.25831
[12582] ZBTB16  <---    NR3C1   -0.059674       0.055651
[12583] SIX1    --->    TLE1    0.05243 -0.021516
[12584] ELP2    <---    STAT1   -0.28689        0.1635
[12585] CAMK1   --->    EIF4G3  0.33156 -0.35333
[12586] PEG3    <---    TRAF2   -0.10064        0.099066
[12587] FBLIM1  <---    FERMT2  -0.05215        0.055796
[12588] CD2     <---    FYN     -0.72305        0.76723
[12589] STAT5A  <---    CD247   -0.24277        0.21556
[12590] ZMYND8  --->    FHOD1   0.033656        -0.018793
[12591] CHEK2   --->    NBN     0.1491  -0.16914
[12592] CASP6   --->    TRAF1   0.72974 -0.75525
[12593] CDK2    --->    SMAD3   1.012   -1.0074
[12594] STAT5B  <---    ETS2    -0.26685        0.25162
[12595] MAP3K2  --->    MAP3K5  0.11998 -0.16749
[12596] PEBP1   <---    MAPK1   -0.78886        0.76438
[12597] RPS6KA4 --->    ATF1    0.98749 -1.0017
[12598] KCNJ2   ?       KCNJ4   -0.01825        -0.01825
[12599] TMPRSS6 <---    LAMA1   -0.018532       0.011049
[12600] JUND    <---    MAPK1   -1.0148 1.0022
[12601] SLA2    <---    CBL     -0.58879        0.57629
[12602] SETD5   --->    CCDC85B 0.052772        -0.021629
[12603] ITGA2   <---    COL1A1  -0.01415        0.00041402
[12604] MDC1    --->    TP53    0.36414 -0.35282
[12605] TCERG1  --->    BARD1   0.19519 -0.1898
[12606] ESR1    <---    GNAI1   -0.15535        0.16985
[12607] DOK2    <---    RET     -0.50436        0.48844
[12608] BIN1    <---    PTK2    -0.071491       -0.0012201
[12609] SRC     --->    PECAM1  0.39601 -0.32901
[12610] PRKACA  --->    KLF1    0.63385 -0.64027
[12611] ITGA1   <---    COL8A1  -0.014328       0.00060242
[12612] TGFBR1  <---    TRAF6   -0.18887        0.16774
[12613] ITPR2   <---    TRPC1   -0.016919       -0.0071319
[12614] HDAC2   --->    RELA    0.067213        -0.0691
```

[12615]	CACNA1A	<---	GNB1	-0.15907	0.15167
[12616]	ETS2	<---	SRC	-0.59343	0.65791
[12617]	MAP2K7	<--->	MAPK8	0.23429	0.23429
[12618]	MERTK	--->	BMPR2	0.11504	-0.12666
[12619]	TNFSF11	<---	TRAF6	-0.59934	0.59323
[12620]	DFNB31	<---	LRRC4C	-0.055166	0.061557
[12621]	FGFR3	<---	RADIL	-0.063882	0.052899
[12622]	PTPN11	--->	PDGFRB	0.13294	-0.12047
[12623]	MAPK3	<---	PTPN7	-1.009	1.0061
[12624]	KCNJ12	<---	DTNA	-0.0058577	0.040695
[12625]	CUL9	--->	TP53	0.38845	-0.37748
[12626]	HNRNPC	?	RALYL	-0.016298	-0.016298
[12627]	TNIP2	<---	MAP3K8	-0.81814	0.79521
[12628]	BAG1	<---	NR3C1	-0.0036637	0.025363
[12629]	SHC1	<---	ERBB2	-0.47563	0.47338
[12630]	CREBBP	--->	MYOD1	0.19969	-0.20391
[12631]	CSNK2A2	--->	CALM1	1.007	-1.0109
[12632]	TP53	<---	FXYD6	-0.3015	0.31475
[12633]	ACTN4	--->	TJP1	0.012884	-0.01902
[12634]	CTNNB1	<---	HIF1A	-0.052313	0.039257
[12635]	DUSP13	<---	SMURF1	-0.61688	0.61366
[12636]	UBE2B	--->	UBR1	0.11223	-0.078375
[12637]	PLCG1	<---	SHC1	-0.11439	0.06568
[12638]	HGF	?	PLAU	-0.010603	-0.010603
[12639]	CEBPB	--->	CREB1	0.29921	-0.28371
[12640]	RIN1	<---	HRAS	-0.99801	1.0021
[12641]	IL1RAP	--->	MYD88	0.17483	0.026315
[12642]	TIMP3	--->	MMP9	0.21399	-0.19321
[12643]	CBX1	<---	ARL5A	-0.43058	0.45692
[12644]	PRKG2	--->	PLCB3	0.62876	-0.62602
[12645]	MYO10	--->	CALML3	0.093674	-0.098954
[12646]	LIMS1	--->	EHMT2	0.16914	-0.17009
[12647]	MAP3K8	<--->	MAP3K14	0.23429	0.23429
[12648]	EIF4G1	--->	EIF4E	0.5863	-0.56213
[12649]	SYK	<---	PRKCA	-0.60642	0.70021
[12650]	IL8	<---	GNAI2	-0.20259	0.21824
[12651]	E2F6	--->	TFDP1	-0.0088085	-0.064857
[12652]	EPHB2	--->	RRAS	0.17617	-0.18451
[12653]	CAMK4	--->	NOS1	0.48839	-0.49502
[12654]	RAC1	--->	NOS2	0.50819	-0.51583
[12655]	NCOR2	--->	PPARG	0.080647	-0.092189
[12656]	RAB11FIP2	<---	RAB11B	-0.15731	0.20092
[12657]	CCL8	--->	VCAN	0.010191	-0.021003
[12658]	TNFRSF9	--->	TRAF2	0.5393	-0.53992
[12659]	TAL2	--->	TCF3	-0.13552	-0.39218
[12660]	SPARC	<---	COL1A1	-0.016078	0.0082154
[12661]	MDM4	<---	MDM2	-0.0029923	0.015384
[12662]	PRKCA	--->	ITGB1	0.075699	-0.093315
[12663]	CDK7	<--->	CDK1	0.23429	0.23429
[12664]	SMYD3	<---	HSP90AA1	-0.0049084	0.023571
[12665]	GNB2L1	<---	PRKCE	-0.70164	0.63388
[12666]	PDCD6IP	<---	PDCD6	-0.26872	0.28484
[12667]	SMARCD1	--->	NR3C1	0.052806	-0.042542
[12668]	RUNX2	--->	MSX2	0.075507	-0.043266
[12669]	MMP2	<---	DCN	-0.18966	0.20515
[12670]	RPS6KA5	--->	ATF1	0.98749	-1.0017
[12671]	ID2	?	MYOD1	-0.65635	-0.65635
[12672]	RAPGEF2	--->	RAP2B	1.0152	-1.003
[12673]	GNB2L1	<---	RASA1	-0.30266	0.15908
[12674]	DLG4	<---	CASK	-0.012439	-0.0091843
[12675]	SLC9A5	<---	ARRB1	-0.02919	0.044822
[12676]	ESR2	<---	MAPK11	-0.67374	0.59966
[12677]	TIAM1	<---	PRKCD	-0.13142	0.12393
[12678]	BCAR1	--->	SH2D3C	0.0074719	-0.058292
[12679]	MYST2	<---	NINL	-0.36262	0.39643
[12680]	ITGAM	<---	ELANE	-0.088946	0.077373
[12681]	CASP1	<---	CDK11B	-0.99771	0.99863
[12682]	P2RY2	--->	ITGB5	0.1188	-0.12597
[12683]	NOS2	<---	ACTB	-0.21268	0.20117
[12684]	RIN2	<---	RAB5B	-0.99801	1.0021
[12685]	PSEN1	--->	NOTCH3	0.9973	-1.0138
[12686]	PGR	<---	PIAS1	-0.090133	0.095399
[12687]	ZAP70	--->	SH2B3	1.0101	-0.99632
[12688]	LYN	--->	RGS16	0.94045	-0.7821
[12689]	KPNA2	<---	RECQL	-0.018416	0.032742
[12690]	TGM2	<---	RHOA	-0.35844	0.38895

[12691]	FOS	<---	BCL3	-0.24172	0.15591
[12692]	SPTBN4	<---	PTPRN	-0.54881	0.54341
[12693]	CCR1	<---	JAK1	-0.53456	0.52384
[12694]	CBL	--->	CRK	0.35632	-0.37379
[12695]	IL6ST	<---	ERBB3	-0.11128	0.096368
[12696]	RASIP1	<---	RAP1A	-0.64033	0.64447
[12697]	HIPK3	--->	AR	1.2465	-1.2513
[12698]	PRDM2	--->	HIST3H3	0.1669	-0.15529
[12699]	CASP1	<---	IL18	-0.065744	0.073886
[12700]	F10	<---	F7	-0.0069337	-0.006575
[12701]	AKT1	--->	WNK1	0.40131	-0.41216
[12702]	ATXN1	<---	SETD2	-0.077057	0.069583
[12703]	GNB2L1	<---	PRKCD	-0.77871	0.78833
[12704]	CD48	<---	IL18R1	0.094034	0.14071
[12705]	CDK6	--->	CDKN2D	1.3062	-1.3222
[12706]	SMURF2	--->	ACBD3	0.42581	-0.43365
[12707]	FN1	<---	FASLG	-0.41473	0.44755
[12708]	MOS	<---	HSPA1A	-0.99901	1.0063
[12709]	RNF11	--->	PDCD6IP	0.22174	-0.18902
[12710]	PLD2	<---	ALDOA	-0.46543	0.46244
[12711]	CSNK1A1	--->	CTNNB1	0.99847	-0.98639
[12712]	SPOCK1	--->	MMP2	0.25303	-0.23217
[12713]	IGF1R	--->	IRS4	0.15482	-0.16797
[12714]	PTPN12	--->	INSR	0.10269	-0.11556
[12715]	STMN1	<---	UBQLN4	-0.17374	0.21794
[12716]	AKT1	--->	SH2B2	0.092343	-0.068454
[12717]	HSPA8	--->	SP1	0.025312	-0.033583
[12718]	CCR5	<---	ADRBK1	-0.42229	0.39714
[12719]	SH2B3	<---	FLNA	-0.12031	-0.059425
[12720]	ADAM28	--->	ITGA4	0.010504	-0.021553
[12721]	MATN2	<---	FN1	-0.0054899	0.0077716
[12722]	SH3KBP1	--->	PIK3R1	0.17874	-0.28474
[12723]	RASA1	<---	SYK	-0.99912	1.0053
[12724]	NCOA2	<---	PPARG	-0.028796	0.018111
[12725]	TIRAP	--->	CD247	0.31313	-0.3265
[12726]	DHX30	--->	RBBP7	0.091156	-0.076481
[12727]	RARG	?	RXRB	-0.0089162	-0.0089162
[12728]	RIT1	--->	MLLT4	0.99712	-0.98672
[12729]	TLN1	<---	ITGB3	-0.049547	0.036854
[12730]	ATP1A3	<---	ATXN1	-0.026708	0.01561
[12731]	CASP7	--->	BIRC3	0.10574	-0.16105
[12732]	TIFA	<---	IRAK1	-0.16525	0.24952
[12733]	MET	<---	CNR1	-0.15228	0.14229
[12734]	SCN2A	<---	ARHGEF10L	-0.040085	0.075316
[12735]	BAG2	<---	HSPA1A	-0.92976	0.96516
[12736]	LPXN	--->	ZNF426	0.20253	-0.20674
[12737]	MAPK8IP1	<---	VRK2	-0.242	0.24958
[12738]	PPIL5	--->	TNFRSF9	0.060806	-0.046825
[12739]	FHL3	--->	CDC25B	0.11826	-0.089716
[12740]	SLC2A4	<---	ESYT2	-0.19815	0.21239
[12741]	WASF2	--->	ACTR3	0.089124	-0.077881
[12742]	ATM	<---	EIF4EBP1	-0.22623	0.21616
[12743]	PAX5	--->	ID2	0.13137	-0.10726
[12744]	KHDRBS1	<---	NCF1	-0.056096	0.04573
[12745]	NOS1	<---	HMOX1	-0.28319	0.27356
[12746]	SDCBP	--->	GRM3	0.12855	-0.11226
[12747]	LCP2	<---	CBL	-0.50201	0.47731
[12748]	ARAF	<---	HRAS	-0.9994	1.0008
[12749]	PRKACA	<---	CETN1	-1.0113	1.0111
[12750]	PTCH2	<---	SHH	-0.56975	0.56021
[12751]	BMPR1B	--->	DCAF12	0.13865	-0.1649
[12752]	CHD3	--->	TP73	-0.00020956	-0.011165
[12753]	GAPDH	<---	CAMK4	-0.44451	0.4233
[12754]	TWIST1	--->	TCF3	-0.054581	-0.14837
[12755]	ACTN3	<---	ADORA2A	-0.15434	0.15695
[12756]	SMAD3	<---	NUP153	-0.34737	0.43042
[12757]	PTPRC	--->	EZR	0.16704	-0.17508
[12758]	ARF1	--->	EEF1G	0.467	-0.43972
[12759]	GNB2L1	<---	ITGB7	-0.019834	0.023504
[12760]	PRKAR1B	--->	SMAD4	0.54864	-0.46649
[12761]	INHBA	--->	ACVR2A	1.0057	-0.99943
[12762]	PTPRF	--->	IRS2	0.015314	-0.028177
[12763]	PPP6C	--->	NFKBIE	0.5082	-0.51072
[12764]	CCR1	--->	CCL2	0.49595	-0.46475
[12765]	DAB2	<---	CDK1	-0.80702	0.78366
[12766]	BLNK	<---	PTPN6	-1.0412	0.99357

[12767]	HSPG2	--->	COL13A1	-0.0010265	-0.012405
[12768]	PAK2	--->	MYLK	0.53897	-0.35021
[12769]	CCL3L1	<---	CCR1	-0.46475	0.49595
[12770]	APP	<---	HOMER2	-0.37062	0.36069
[12771]	TP53	<---	PLK1	-0.55633	0.55158
[12772]	LIN7A	<---	CASK	-0.019766	0.014905
[12773]	CASP3	--->	RASA1	-0.026536	-0.078902
[12774]	TCERG1	--->	CHAF1A	0.05053	-0.053946
[12775]	GRID1	<---	GOPC	-0.17792	0.18588
[12776]	PICK1	<---	UBQLN4	-0.033544	0.070224
[12777]	RIMS2	<---	RAPGEF4	-0.042153	0.033559
[12778]	TIAL1	<---	SMN1	0.0023256	0.011379
[12779]	CHN2	<---	TGFBR1	-0.20451	0.17393
[12780]	HNRNPD	<---	UBE2I	-0.18951	0.22068
[12781]	DLG1	--->	GRIK2	0.058844	-0.064781
[12782]	THRB	--->	CCND1	0.3199	-0.31113
[12783]	CUGBP1	<---	DMPK	-0.75755	0.72994
[12784]	PPFIA3	<--->	PPFIBP1	0.049612	0.049612
[12785]	RHOH	--->	TGFBR1	0.010625	-0.037483
[12786]	OCLN	--->	ACTA1	0.53293	-0.52996
[12787]	EEF1G	--->	GADD45G	0.1007	-0.06568
[12788]	EGR1	<---	CDKN2A	-0.0070279	0.011085
[12789]	YWHAB	<---	ALS2	-0.027479	0.028247
[12790]	PPEF1	--->	CALM2	-0.12978	-0.15561
[12791]	ITGA4	<---	FN1	-0.026035	0.018644
[12792]	MMP1	<---	TNFSF11	-0.42626	0.44952
[12793]	MYL1	--->	ACTA1	0.57525	-0.57177
[12794]	TICAM2	<---	IL1RAP	-0.20182	0.20236
[12795]	PTPRE	<---	SRC	-0.37818	0.26821
[12796]	GNAI2	<---	EDNRA	-1.008	1.0129
[12797]	HIST3H3	<---	KPNA1	-0.15414	0.20377
[12798]	WIPI1	<---	ESR1	-0.0096005	0.034681
[12799]	CTSD	--->	SERPINA4	0.447	-0.43574
[12800]	PRKCG	--->	ADRBK1	0.23691	0.014495
[12801]	BATF	--->	JUNB	0.36727	-0.73152
[12802]	FLNA	<---	RHOA	-0.39396	0.4246
[12803]	CABP1	--->	CACNA1C	0.44954	-0.46035
[12804]	CAMKK1	--->	CALM1	1.0175	-0.89542
[12805]	PALLD	--->	EZR	0.06611	-0.063376
[12806]	SMARCE1	--->	NR3C1	0.30074	-0.28692
[12807]	KIT	--->	STAT5B	0.99911	-0.99301
[12808]	ATF7	<---	PTP4A1	-0.3305	0.33221
[12809]	H3F3A	<---	KAT2B	-0.21255	0.21959
[12810]	EPB41L2	<---	DRD1	-0.19189	0.19515
[12811]	RAP1A	--->	RAF1	1.0008	-0.9994
[12812]	CLEC1B	<---	NINL	-0.44772	0.4792
[12813]	STMN2	<---	RGS20	-0.55135	0.59116
[12814]	MMP3	<---	CCL13	-0.16713	0.18806
[12815]	DMD	<---	FASLG	-0.035659	0.033134
[12816]	INSM1	--->	CCND1	0.62601	-0.61209
[12817]	PKN1	<---	MAPK12	-0.24206	0.23247
[12818]	SCTR	<---	PRKAR2A	-0.50854	0.51145
[12819]	RCOR1	--->	HDAC2	0.0065674	-0.0024837
[12820]	TRAF4	--->	TICAM1	0.091536	-0.10593
[12821]	PTPN11	--->	MPZL1	0.97001	-0.82769
[12822]	SRC	--->	HNRNPK	1.0415	-0.92798
[12823]	JUN	<---	RELA	-0.052901	0.037403
[12824]	KDR	--->	HSP90AA1	0.08077	-0.085172
[12825]	SMAD9	--->	HEY1	0.61382	-0.63105
[12826]	MCF2L	<---	RHOA	-0.014357	0.0012594
[12827]	SHANK1	<---	RPS6KA3	-0.071398	0.030611
[12828]	BHLHA15	?	MYOD1	-0.65635	-0.65635
[12829]	AATF	<---	DAPK3	-0.60594	0.58784
[12830]	CDK1	--->	AMPH	0.2474	-0.26764
[12831]	KIF1C	<---	YWHAB	-0.040995	0.049737
[12832]	TNFSF11	<---	NFKB1	-1.0088	1.0201
[12833]	DVL1	<---	SMAD3	-0.090479	0.09242
[12834]	CD1D	<---	PTPRC	-0.41618	0.41885
[12835]	MAP2K3	<--->	DYRK1B	0.23429	0.23429
[12836]	FHOD1	--->	ACTG1	0.32267	-0.32649
[12837]	BARD1	<--->	BCL3	0.076747	0.076747
[12838]	KCNQ1	<---	AKAP9	-0.0046269	0.038696
[12839]	SERPINF2	<---	PLG	-0.040728	0.057207
[12840]	KCNIP3	--->	CREM	0.25156	-0.24957
[12841]	APBA1	--->	NOTCH1	0.37677	-0.38955
[12842]	NCOA3	<---	PIN1	-0.23727	0.22559

[12843]	YWHAG	--->	SVIL	0.025272	0.021556
[12844]	ADAM9	<---	PRKCD	-0.28743	0.28219
[12845]	AKAP13	<---	PRKAR2A	-0.11497	0.11855
[12846]	PFN2	--->	ACTB	0.99385	-1.0067
[12847]	TRAF3	<---	OTUD5	-0.076039	0.077894
[12848]	GHR	--->	PIK3R1	0.14374	-0.18664
[12849]	OTUD7B	<---	TRAF6	-0.66545	0.65723
[12850]	PFN2	<---	IVNS1ABP	0.00020341	0.031364
[12851]	YWHAZ	--->	INPP5A	0.082277	-0.058532
[12852]	PDPK1	--->	PKN2	0.68989	-0.69757
[12853]	GUCY2C	<---	SRC	-0.11555	0.46393
[12854]	MICAL1	<---	RAB1B	-0.22699	0.24091
[12855]	MDH1	--->	MPP3	-0.00067805	-0.006164
[12856]	MPP6	<---	DYNLRB1	-0.034814	0.039328
[12857]	CTNNAL1	--->	AKAP13	0.16916	-0.1684
[12858]	HIPK2	--->	SMAD2	1.012	-1.0074
[12859]	ROCK1	<---	RHOC	-1.0003	0.99991
[12860]	BCL2	<---	PPP3CA	-0.20783	0.19751
[12861]	ZFYVE20	<---	RAB14	-0.3573	0.3878
[12862]	MAPK7	--->	ETS1	0.57541	-0.6753
[12863]	SRC	--->	CDH5	0.62261	-0.55927
[12864]	XRN1	--->	UPF2	0.0040317	-0.013387
[12865]	DOK5	<---	INSR	-0.35555	0.35356
[12866]	DAPK3	--->	DAXX	0.3655	-0.48387
[12867]	NPHP1	<---	PTK2B	-0.15413	-0.11085
[12868]	TLR1	--->	TRAP1	0.31346	-0.28434
[12869]	BARD1	<---	BRCA1	-0.098326	0.10458
[12870]	CSNK2A1	--->	CALM1	1.007	-1.0109
[12871]	PRKD1	--->	HDAC5	0.43108	-0.46538
[12872]	HDAC1	<---	BRCA1	-0.000943	0.005336
[12873]	ROBO2	<---	SSRP1	-0.021034	0.022518
[12874]	PRKACA	--->	KCNJ12	0.73999	-0.76275
[12875]	KIT	<---	ABL1	-0.22904	0.15955
[12876]	PKD1	<---	VCL	-0.31076	0.32289
[12877]	CCNA2	<---	CDK1	-1.0774	1.0544
[12878]	PTPRM	--->	PTPRA	0.20738	0.20483
[12879]	PAK1	<---	ERBB2	-0.031059	0.042103
[12880]	PARVG	<---	ILK	-0.41044	0.40608
[12881]	CRNKL1	<---	HSP90AA1	-0.062493	0.054633
[12882]	SH2B2	<---	KIT	-0.45943	0.40786
[12883]	MAPK8	--->	PIK3R1	0.31528	-0.45297
[12884]	TANC1	<---	CASK	-0.046266	0.035543
[12885]	RASSF5	?	RASSF1	-0.03947	-0.03947
[12886]	TSHR	--->	GNAI1	0.65587	-0.65379
[12887]	RNF11	<---	STAMBP	-0.041316	0.066239
[12888]	MAP3K14	--->	CHUK	1.0117	-1.003
[12889]	STAT5A	<---	NR3C1	-0.29089	0.27638
[12890]	TERT	<---	YWHAQ	-0.061318	0.052112
[12891]	NFKB1	--->	NFKBIB	0.77463	-0.041138
[12892]	RFX4	?	RFX3	-0.0064988	-0.0064988
[12893]	HNF4A	--->	COPS5	0.23695	-0.23085
[12894]	GRB7	<---	RET	-0.2946	0.087937
[12895]	EDAR	--->	TRAF3	0.080013	-0.092937
[12896]	ZHX1	<---	ZHX3	-0.090184	-0.053398
[12897]	RELA	--->	NFKB2	-0.010922	-0.031705
[12898]	TRAF4	--->	NCF1	0.058052	-0.065722
[12899]	RANBP9	<---	UCHL1	-0.0033515	0.018608
[12900]	KDR	--->	NCK1	0.50592	-0.38691
[12901]	IRAK1	--->	NTRK3	0.044374	-0.038092
[12902]	HIST1H3A	<---	SUV39H1	-0.029918	0.043597
[12903]	TRPC4	--->	ITPR1	-0.0070644	-0.013012
[12904]	IGF1R	<---	GNAI2	-0.054709	0.042064
[12905]	CASP1	--->	BID	0.067368	-0.028077
[12906]	DSCAML1	--->	PLCL2	0.1514	-0.15651
[12907]	INPP5D	<---	GRB2	-0.19081	0.28774
[12908]	COL6A1	<---	COL2A1	-0.0028706	-0.0028642
[12909]	SUFU	--->	GLI2	0.23545	-0.24228
[12910]	SOCS3	--->	EPOR	0.055717	-0.058518
[12911]	SOX10	<---	ALX4	-0.022513	0.018113
[12912]	NEDD9	--->	ID2	0.16014	-0.16339
[12913]	TCL1B	--->	TNPO2	0.068657	-0.038606
[12914]	CREBBP	--->	NFIC	0.41326	-0.41533
[12915]	F12	<---	KRT1	-0.028694	0.015965
[12916]	BAD	<---	YWHAZ	-1.0088	0.9978
[12917]	TRADD	--->	FADD	0.98478	-0.99837
[12918]	LASP1	--->	FHL3	0.053659	-0.048067

[12919] CHUK ---> HSP90AB1 0.059478 -0.11716
[12920] GNA14 <--- ADRB2 -0.98627 0.9872
[12921] PDZD3 <--- SLCO3A1 -0.038024 0.07475
[12922] CBL ---> SH2B2 0.16849 -0.20483
[12923] PRPF4B ---> KLF13 0.96685 -0.90544
[12924] C6 <--- F2 -0.015759 0.012052
[12925] RB1 <--- PPP1CA -0.16031 0.14829
[12926] PTPN11 <--- CHUK -0.35322 0.22566
[12927] CASP4 <--- CASP3 0.10027 0.17851
[12928] MDC1 ---> H2AFX 0.24372 -0.19249
[12929] PRKCE ---> ADAP1 0.61485 -0.68245
[12930] VAV3 <--- RHOA -0.14624 0.08839
[12931] SF1 <--- WWP2 -0.18587 0.17634
[12932] CAMK2G ---> EGFR 1.0058 -1.0028
[12933] PPARGC1B ---> ESRRG 0.006502 -0.01266
[12934] PSEN1 ---> CTNND1 0.073388 -0.081539
[12935] MAP3K7IP3 <--- MAP3K7IP1 -0.50733 0.54286
[12936] ESR1 ---> CALM2 0.15612 -0.12827
[12937] TP73 <--- MDM2 -0.3559 0.35226
[12938] RIMS1 ---> YWHAH 0.0025914 0.0007618
[12939] SRF ---> RELA 0.07682 -0.077466
[12940] CREBBP ---> SMAD4 1.0006 -0.99697
[12941] INHA ---> INHBA -0.063724 -0.31443
[12942] SIRT2 <--- SP140 -0.015238 0.048926
[12943] TNFRSF10B ---> TRADD 1.0063 -1.0095
[12944] GRK6 ---> TBXA2R 0.31971 -0.40764
[12945] CAMK2G <--- RRAD -0.48262 0.48194
[12946] RIPK4 <--- PRKCD 0.015172 0.40777
[12947] TRAF1 <--- TRAF2 -0.022408 0.089
[12948] EPB41L3 <--- YWHAB -0.057222 0.05792
[12949] KAT2B ---> RELA 0.064754 -0.072138
[12950] KLHL3 <--- GNAO1 -0.22528 0.23954
[12951] EFEMP2 ---> HHEX 0.101 -0.067922
[12952] PDGFRB ---> ARAF 0.015632 -0.046798
[12953] LRP6 <--- ANTXR1 -0.6819 0.68261
[12954] TGFB3 <--- ENG -0.30966 0.36037
[12955] CSNK1A1 ---> BID 0.59045 -0.50796
[12956] RHOG ---> TGFBR1 0.010625 -0.037483
[12957] PXN <--- CRK -0.25245 0.21073
[12958] CAMK2A ---> ACTN2 -0.0067734 -0.0078992
[12959] WASL <--- WIPF1 -0.033488 -0.016497
[12960] DLGAP1 ---> SHANK1 0.036253 -0.043141
[12961] PTPRE ---> MAPK3 1.0061 -1.009
[12962] TGM2 ---> FN1 0.06474 -0.036003
[12963] MAPK14 ---> ESR1 0.38823 -0.49258
[12964] MST1R <--- GAB1 -0.038307 0.026098
[12965] MYOM2 ---> MYH9 0.013105 -0.026322
[12966] FHL5 ---> CREM 0.32862 -0.31634
[12967] CRTC2 ---> YWHAZ 0.071582 -0.070272
[12968] ATXN1 <--- ZNF276 -0.029824 0.026666
[12969] L1CAM ---> EZR 0.090989 -0.10199
[12970] IKBKG <--- FADD -0.34023 0.25275
[12971] ICAM2 ---> ITGAM 0.077289 -0.088862
[12972] NCF1 ---> IL4R 0.018494 -0.026745
[12973] NCOR2 ---> FOS 0.069886 -0.093411
[12974] IRAK1 <--- BTK -0.15264 0.13824
[12975] SPTAN1 <--- CTNNA1 -0.16431 0.1545
[12976] CAPN1 ---> CAPNS1 0.040543 0.0017592
[12977] BMPR1B <--- SRGAP1 -0.007431 0.012373
[12978] BHLHE40 ---> STAT3 0.20842 -0.24129
[12979] TAX1BP3 <--- RTKN -0.15896 0.16894
[12980] GSK3B ---> NOTCH1 0.70744 -0.72575
[12981] DOK3 <--- CSK -1.1853 1.3209
[12982] PSEN2 <--- CAPN1 -0.25395 0.25417
[12983] PRKACA ---> HMGCR 0.54046 -0.56003
[12984] HNF4A ---> NCOA1 0.066423 -0.077273
[12985] SMAD5 <--- WWP1 -0.32416 0.30797
[12986] PRKCA ---> RHOA 1.0123 -0.99154
[12987] MAP4K1 ---> HCLS1 0.22295 -0.19429
[12988] CACNA1C <--- GNB1 -0.47032 0.45928
[12989] ARHGAP1 <--- RASA1 -0.17636 0.087311
[12990] YWHAB <--- MAP3K3 -0.30237 0.54958
[12991] AMHR2 <--- TGFBR1 -0.062144 -0.022886
[12992] DCAF6 ---> AR 0.94792 -0.94858
[12993] SMAD2 ---> MYC 0.39156 -0.40036
[12994] ARAF ---> MAP2K1 0.9856 -1.0047

[12995]	FBXW8	--->	RBX1	0.035455	-0.032537
[12996]	GNB2L1	<---	PRKCA	-0.92085	0.92236
[12997]	FBLN2	<---	COL4A1	-0.038144	0.027121
[12998]	PLG	<---	F2R	-0.27153	0.28486
[12999]	PRKCA	--->	HES1	0.14857	-0.15922
[13000]	RB1	<---	MYOD1	-0.35069	0.35001
[13001]	HDAC1	<---	DNMT1	-0.013043	0.016989
[13002]	ANKRA2	--->	LRP2	0.22874	-0.23435
[13003]	CMA1	--->	PFN1	0.095133	-0.062435
[13004]	PIM3	--->	BAD	0.75379	-0.87574
[13005]	AHRR	--->	ARNT	-0.060412	-0.061404
[13006]	TNIK	<---	TRAF2	-0.11664	0.11187
[13007]	CDC42	--->	EEF1G	0.40942	-0.378
[13008]	PTK2B	<---	ZAP70	-0.48288	0.50478
[13009]	SRF	--->	MYOG	0.42755	-0.40418
[13010]	CABP1	--->	ITPR1	0.32619	-0.3361
[13011]	DLG4	--->	GRIN2B	0.044742	-0.055228
[13012]	PELI1	<---	IRAK4	-0.44131	0.48758
[13013]	TRAIP	<---	FLII	-0.012092	0.026055
[13014]	PRKD3	--->	GSK3A	0.28762	-0.077068
[13015]	BRAF	<---	RAP1A	-0.99496	1.0064
[13016]	FYB	<---	SH2D1A	-0.26135	0.23548
[13017]	TAL1	<---	SKP2	-0.41859	0.4357
[13018]	ABI1	<---	NCK1	-0.10752	0.24536
[13019]	KCNQ5	--->	CALM1	0.32976	-0.29233
[13020]	SRC	--->	NFKBIA	1.0084	-1.0021
[13021]	NECAB3	--->	APP	0.36296	-0.37288
[13022]	SKIL	<---	FBLN1	-0.40072	0.405
[13023]	SYT9	--->	TRPV1	0.14554	-0.13949
[13024]	RASA1	--->	CAV2	0.14846	-0.29786
[13025]	HDAC10	<---	NCOR2	-0.22984	0.22101
[13026]	ABL1	--->	ACTA1	-0.011285	-0.086082
[13027]	MMP13	<---	COL2A1	-0.051184	0.041848
[13028]	EP300	--->	ETS1	0.11504	-0.12299
[13029]	SMURF1	--->	WDR61	0.71781	-0.72185
[13030]	STRAP	--->	SMAD2	0.46428	-0.38187
[13031]	NOTCH2	<---	SMAD1	-0.39121	0.38008
[13032]	ZFYVE20	<---	RAB5A	-0.3573	0.3878
[13033]	GABBR2	--->	ATF5	0.26486	-0.23122
[13034]	SPI1	<---	DNMT3A	-0.18595	0.22812
[13035]	GRK5	<---	RCVRN	-0.24096	0.19057
[13036]	BARD1	<---	POU2F1	-0.07011	0.087681
[13037]	S1PR1	<---	HTR1D	0.15705	0.35144
[13038]	PKNOX1	<---	HOXB6	-0.0094151	0.024726
[13039]	GRAP	<---	LAT	-0.013083	-0.0015555
[13040]	HDAC3	<---	PPP4R1	-0.010498	0.045525
[13041]	GAK	--->	AXIN1	0.11959	-0.13243
[13042]	PATZ1	--->	MITF	0.0070915	-0.015719
[13043]	TNF	--->	CSF1	0.36051	-0.33358
[13044]	ABCA1	<---	LMO7	-0.0068988	-0.0054502
[13045]	SMAD4	--->	TFE3	0.42594	-0.43382
[13046]	SRC	--->	PIK3R1	0.67123	-0.63842
[13047]	BCAN	<---	FBLN2	-0.015047	0.0061745
[13048]	ITCH	--->	SH3GL2	0.99755	-1.0001
[13049]	YWHAZ	<---	SIK3	-0.66368	0.62776
[13050]	GRASP	--->	GRM2	0.0626	-0.025484
[13051]	PPARA	--->	RELA	0.049708	-0.049342
[13052]	DOK3	<---	INPP5D	-0.10368	0.094457
[13053]	SMAD3	<---	TGFBR2	-0.57747	0.59933
[13054]	SLC8A1	<---	YWHAB	-0.092105	0.10171
[13055]	CRK	<---	FLT1	-0.17632	0.18782
[13056]	GRAP2	--->	KHDRBS1	0.27717	-0.28063
[13057]	PRKCD	--->	EGFR	0.40309	-0.40693
[13058]	CXCR2	--->	GRIA1	0.39666	-0.39518
[13059]	NEDD9	<---	PTK2	-0.039175	0.0030428
[13060]	CEBPA	--->	CTNNB1	0.53851	-0.53649
[13061]	MECP2	<---	HMGB1	-0.26665	0.30175
[13062]	MAPKAPK2	--->	HSPB1	0.73124	-0.7127
[13063]	TIFA	<---	TRAF6	-0.68642	0.67937
[13064]	MYOD1	<---	CALM1	-0.22666	0.2676
[13065]	BMPR1B	--->	KLHL1	0.12723	-0.15552
[13066]	HIVEP3	<---	TRAF1	-0.10652	0.11472
[13067]	SERPINA1	<---	CELA1	-0.082185	0.11833
[13068]	GIPC1	<---	RGS19	-0.41116	0.44344
[13069]	NCOR1	--->	SKI	0.070703	-0.07957
[13070]	SHC1	<---	EPHA2	-0.33226	0.15648

[13071]	BCL2	<---	MAPK3	-1.2078	1.1791
[13072]	CCNG1	<---	MDM2	-0.15283	0.14549
[13073]	CSF1R	--->	SOS1	0.01128	-0.017264
[13074]	APBA2	--->	APP	0.12974	-0.14219
[13075]	VEGFB	--->	FLT1	0.038499	-0.020768
[13076]	SPRED1	<---	PPP1CA	-0.10392	0.092859
[13077]	EDNRB	--->	NOS3	0.51537	-0.52296
[13078]	SMARCA2	--->	NR3C1	-0.0020412	-0.0086988
[13079]	BEGAIN	<---	TRIM27	-0.029515	0.033637
[13080]	UNC13B	--->	SPTBN2	0.11071	-0.10244
[13081]	DAB1	--->	VLDLR	0.1387	-0.14343
[13082]	FLNA	<---	PRKCA	-0.91157	0.91318
[13083]	GFI1B	<---	PDZD4	-0.096181	0.088337
[13084]	RIN1	<---	YWHAZ	-0.24433	0.24721
[13085]	DVL2	<---	RNPS1	-0.38332	0.3831
[13086]	SOCS3	--->	INSR	0.025183	-0.046398
[13087]	SMURF2	--->	PRICKLE1	0.68232	-0.68627
[13088]	FHL1	<---	RBPJ	-0.089863	0.09262
[13089]	PRKG1	--->	NPR1	0.29513	-0.30735
[13090]	HIPK2	--->	TP63	0.45486	-0.4556
[13091]	COPA	<---	PDGFRB	-0.051561	0.045406
[13092]	SHC1	<---	ESR1	-0.18573	0.085747
[13093]	ATXN3	--->	CASP1	0.040981	-0.032755
[13094]	TIAM1	<---	PRKCG	-0.17606	0.18829
[13095]	ITGB2	<---	TLN1	-0.010254	-0.0032203
[13096]	RABGGTA	<---	BMPR2	-0.2778	0.26219
[13097]	RPS6KA2	--->	BAD	0.58958	-0.91274
[13098]	CASP8	--->	TOPORS	0.57059	-0.71181
[13099]	ATF2	<---	CEBPA	-0.34627	0.34768
[13100]	SYNGAP1	--->	TRIP6	0.035611	-0.025886
[13101]	RASGRF1	<---	NTRK2	-0.070857	0.059389
[13102]	CXXC4	--->	DVL1	1.0006	-0.994
[13103]	APC	<---	GSK3B	-0.15381	0.13984
[13104]	IKBKG	<---	CARD11	-0.036627	0.027076
[13105]	RBBP6	--->	ETS1	0.33388	-0.31365
[13106]	PLSCR3	?	PLSCR1	-0.011559	-0.011559
[13107]	BMPR1B	<---	SMAD6	-1.01	1.0109
[13108]	TYRO3	--->	FYN	0.073301	-0.078384
[13109]	ITGA2	<---	ITGB1	-0.010285	-0.0079812
[13110]	PLD1	<---	ARF6	-0.47015	0.46122
[13111]	COPS5	--->	JUND	0.60169	-0.65167
[13112]	MAPK14	--->	SLC9A1	0.37568	-0.41499
[13113]	NR1H3	<---	FOXO3	-0.24075	0.23509
[13114]	MAPK9	--->	IRS1	0.5492	-0.60947
[13115]	GTF2IRD1	--->	SMAD3	0.40588	-0.32239
[13116]	PDLIM5	<---	TRIM39	-0.012675	0.010563
[13117]	MAP3K7	--->	IKBKB	1.0099	-0.99837
[13118]	RPS6KA3	<---	MAPK1	-1.0109	1.002
[13119]	LGALS3BP	--->	ITGB1	0.12479	-0.13313
[13120]	SPI1	<---	NFKB1	-0.77477	0.78296
[13121]	COPS5	--->	BCL3	0.28802	-0.27394
[13122]	PRPF4	<---	SFRS1	-0.10831	0.13878
[13123]	ZIC1	<---	GLI2	-0.0093948	0.078952
[13124]	PTPRO	--->	KIT	0.38384	-0.41648
[13125]	DLGAP2	<---	ADRB1	-0.27781	0.27902
[13126]	CCR1	--->	CCL5	0.49595	-0.46475
[13127]	PTK2	--->	ERBB2	0.05046	0.038896
[13128]	ATN1	--->	SSPO	0.031894	-0.038927
[13129]	ATF7	<---	MAPK9	-0.71045	0.71012
[13130]	RALB	--->	CALM1	0.73657	-0.70265
[13131]	NR2C2	?	ESR1	-0.0089162	-0.0089162
[13132]	YES1	--->	KIT	1.0007	-1.0039
[13133]	SMAD3	<---	SKI	-0.10174	0.14755
[13134]	KCNJ12	<---	SNTA1	-0.0070376	0.044422
[13135]	TCF20	--->	JUN	0.4633	-0.50863
[13136]	PKN3	--->	PLD1	0.056976	-0.074382
[13137]	TP53	<---	TOP2B	-0.41701	0.42803
[13138]	STRAP	--->	SMAD3	0.46428	-0.38187
[13139]	SMAD1	<---	HBP1	-0.11467	0.091961
[13140]	RAB33B	--->	TGFBR1	0.010625	-0.037483
[13141]	HOXB6	<---	CREBBP	-0.18943	0.18505
[13142]	EGFR	<---	PAK1	-0.16913	0.21518
[13143]	PGR	?	ESR1	-0.0089162	-0.0089162
[13144]	NCOR2	--->	BCL6	0.075513	-0.089123
[13145]	TNK2	--->	MCF2	0.99631	-0.99902
[13146]	FGF2	<---	CSNK2A1	-0.75902	0.73258

[13147]	KDR	--->	SYNGAP1	0.25234	-0.23912
[13148]	ITGB1	<---	VCAN	-0.01696	0.0033275
[13149]	PLSCR1	--->	C12orf12	0.016782	0.011019
[13150]	PRMT1	--->	NCOA3	0.087035	-0.10018
[13151]	PLK1	--->	MYT1	0.1704	-0.18815
[13152]	SF3B1	--->	SMAD9	0.32211	-0.34025
[13153]	RET	--->	MAPK14	0.058583	-0.16526
[13154]	CSNK2A1	--->	BDP1	0.69994	-0.72579
[13155]	DLG4	<---	SRC	-0.1977	0.4105
[13156]	AR	<---	CASP7	-1.0225	1.0094
[13157]	RAD23A	<--->	UBQLN4	0.069208	0.069208
[13158]	CIRBP	<---	PRMT1	-0.28644	0.28801
[13159]	ADRA1A	--->	ADRA1B	0.17875	0.075526
[13160]	L1CAM	--->	RDX	0.090989	-0.10199
[13161]	CDKN2D	<---	CDK4	-1.3222	1.3062
[13162]	SNTA1	--->	CALM1	0.36293	-0.32288
[13163]	ELP2	<---	JAK1	-0.54505	0.5344
[13164]	DNMT3B	<---	HDAC2	-0.0084751	0.024192
[13165]	ACVR2B	<---	GDF5	-0.99943	1.0057
[13166]	LYN	--->	FCGR2A	0.11748	0.015127
[13167]	NKIRAS1	<---	SMURF2	-1.0084	1.0007
[13168]	HRAS	--->	VAV1	0.041359	-0.22722
[13169]	MATN2	<---	COL4A3	-0.023075	0.01173
[13170]	ADRB2	--->	ARRB1	0.12815	-0.12587
[13171]	CSNK2A1	--->	TOP2A	0.52133	-0.54823
[13172]	PRAM1	--->	MXD1	0.57232	-0.53633
[13173]	HDAC1	--->	SPEN	0.019906	-0.016764
[13174]	FLT3	--->	GRB2	0.22877	-0.47074
[13175]	TGFBR3	--->	TGFB2	0.18653	-0.16345
[13176]	BUB3	<---	BUB1B	-0.86422	0.83716
[13177]	MAPK4	--->	GAB2	0.99738	-0.99704
[13178]	HIPK3	--->	RGS3	0.11222	-0.11559
[13179]	SHC1	<---	EGFR	-0.987	0.99728
[13180]	VSIG4	<---	C3	-0.09081	0.088879
[13181]	STAT6	<---	CREBBP	-1.0041	0.99741
[13182]	TRPC6	<---	SRC	-0.077836	0.06644
[13183]	BFAR	<---	CASP10	-0.084638	0.05136
[13184]	HSPB1	<---	IGSF21	-0.30268	0.30719
[13185]	TTRAP	<---	TRAF5	-0.057745	0.058225
[13186]	HOXB13	--->	MEIS1	0.024726	-0.0094151
[13187]	DACH1	<---	UBE2I	-0.22081	0.23729
[13188]	ADRA1D	--->	SNTB1	0.17556	-0.18342
[13189]	CSNK1D	--->	CDK5	0.9966	-0.99147
[13190]	TNFSF15	--->	TNFRSF25	0.62384	-0.7724
[13191]	TGFB1I1	<---	EP300	-0.076507	0.065899
[13192]	GSK3B	<---	MARK2	0.0039491	0.058458
[13193]	RHOJ	--->	SMAD4	0.55322	-0.47159
[13194]	SMAD2	<---	RANBP9	-0.20553	0.25035
[13195]	CASP5	--->	MAX	0.32179	-0.31143
[13196]	TNFAIP3	--->	YWHAH	0.13176	-0.13918
[13197]	IGF1R	--->	CRK	0.098003	-0.11926
[13198]	PTPRC	--->	GRB2	0.77792	-0.79846
[13199]	SYNJ1	<---	SH3GL1	-0.094045	0.095249
[13200]	GABARAPL2	<---	ULK1	-0.047831	0.033175
[13201]	WASF3	--->	ACTB	0.26671	-0.2714
[13202]	LMOD1	--->	ACTB	0.092222	-0.10225
[13203]	PPARA	<---	HSP90AA1	-0.046517	0.037639
[13204]	AURKA	--->	CDC25B	0.69298	-0.67632
[13205]	ACTN1	<---	CAMK2A	-0.21773	0.202
[13206]	TRPC1	<---	TRPC4	-0.0093674	-0.0093671
[13207]	CCL27	<---	CCR10	-0.46475	0.49595
[13208]	YWHAE	--->	CALM1	0.41662	-0.38921
[13209]	BMPR1A	<---	BMP6	-1.0007	0.99697
[13210]	TLR2	--->	MAPK8IP3	0.095048	-0.10411
[13211]	ZBTB16	<---	SPOP	-0.053262	0.011037
[13212]	PRG2	<---	AKT1	-0.3163	0.46285
[13213]	TRRAP	<---	BRD8	-0.0074731	-0.0023635
[13214]	ACVR1	--->	FCHO1	0.044958	-0.063252
[13215]	RPS6KA1	--->	SRF	0.12564	-0.26444
[13216]	TP53BP2	--->	RELA	0.0097856	-0.014883
[13217]	HNF4A	--->	SP1	0.0089233	-0.020282
[13218]	RCOR3	--->	NECAB2	0.24182	-0.2245
[13219]	RAC3	--->	AR	0.97383	-0.97461
[13220]	YWHAG	--->	DDX17	0.079181	-0.079288
[13221]	CDK1	--->	EPN1	0.70002	-0.72593
[13222]	GPD2	<---	ALDOA	-0.31629	0.3537

[13223]	UBE2J2	<--->	UBE2G2	0.21837	0.21837
[13224]	SMARCD3	--->	PBX1	0.077665	-0.063695
[13225]	SH3GL1	--->	AXIN1	0.12265	-0.13191
[13226]	RAGE	--->	JUN	1.0022	-1.0148
[13227]	PDLIM1	<---	ACTN2	-0.062619	0.060394
[13228]	NUAK2	--->	TGFBR1	0.21822	0.012691
[13229]	DAB1	--->	APLP2	0.068664	-0.075344
[13230]	PTPN6	--->	PECAM1	0.26319	-0.077948
[13231]	PRKAR2A	--->	WASF1	0.3203	-0.30788
[13232]	MSN	<---	ICAM3	-0.020306	0.0082012
[13233]	FIGF	<---	FLT4	-0.18233	0.21449
[13234]	EP300	--->	SMAD1	0.62712	-0.62984
[13235]	ARF5	--->	RAB11FIP3	0.48818	-0.47544
[13236]	LIMK1	--->	RLIM	0.05293	-0.066833
[13237]	C21orf7	--->	GPS2	0.026044	0.011368
[13238]	SH3GL2	<--->	AMPH	0.0094254	0.0094254
[13239]	FLT4	--->	GRB2	0.52045	-0.4666
[13240]	RASSF1	<---	HRAS	-0.98684	1.0025
[13241]	PRKACA	--->	CUL5	0.30021	-0.32466
[13242]	PGF	--->	FLT1	0.038499	-0.020768
[13243]	MYST3	--->	SPI1	0.22322	-0.18106
[13244]	FYN	<--->	TXK	0.37153	0.37153
[13245]	PPP2R2A	<---	PPP2CA	-0.4473	0.44649
[13246]	PPP2R2A	<---	TGFBR1	-0.99919	1.0077
[13247]	ANTXR1	<---	HDAC2	-0.015166	0.030838
[13248]	DNM1	<---	BIN1	-0.080916	0.071535
[13249]	MAPK8	--->	IRS1	0.5492	-0.60947
[13250]	BEGAIN	--->	ZNF417	0.19959	-0.20234
[13251]	NFKBIE	<---	IL1A	-0.44458	0.4588
[13252]	SKIL	<---	NID1	-0.14021	0.13313
[13253]	DHX30	<---	HDAC3	0.00091556	0.01455
[13254]	ASCC1	--->	ASCC3	0.010259	-0.018028
[13255]	THRB	<---	TRIP10	-0.10572	0.10739
[13256]	MAP3K14	--->	PEBP1	0.76438	-0.78886
[13257]	SENP2	<---	NUP153	-0.020638	0.057275
[13258]	MUC1	--->	CTNNB1	0.18706	-0.1956
[13259]	YWHAG	--->	TFE3	0.40547	-0.39631
[13260]	HSPA5	--->	A2M	0.97857	-0.93712
[13261]	MAP3K5	--->	EIF2AK2	0.9934	-0.99683
[13262]	RBPJ	<---	HDAC1	-0.60406	0.60565
[13263]	CDK9	--->	SERPINH1	0.80369	-0.82745
[13264]	ADRA1D	--->	SNTA1	0.343	-0.34434
[13265]	UBE2E1	--->	RNF5	0.12227	-0.11995
[13266]	ZRANB1	<---	TRAF6	-0.50449	0.49675
[13267]	RIN1	<---	YWHAE	-0.24433	0.24721
[13268]	WAS	--->	ACTC1	0.1702	-0.20434
[13269]	MAPK14	<---	MAPK1	-0.9992	1.0074
[13270]	SMAD3	--->	SREBF2	0.29212	-0.30243
[13271]	SKIL	<---	HIPK3	-0.57024	0.56092
[13272]	USH1G	<---	USH1C	-0.22426	0.24286
[13273]	MYO7A	--->	ACTA1	0.15467	-0.16233
[13274]	CREB3L1	<--->	CREB3L3	0.45817	0.45817
[13275]	BSG	--->	CAV1	0.3459	-0.3483
[13276]	AKT1	--->	ESR2	1.0083	-1.0039
[13277]	PHC2	--->	GFI1B	0.1813	-0.18471
[13278]	AXIN1	<---	SMAD7	-0.018982	0.01973
[13279]	NOV	<---	IGF2	-0.47388	0.46446
[13280]	FGFR4	<---	EPHA4	-0.097243	0.027285
[13281]	PPP2R1B	--->	BSG	0.056162	-0.061094
[13282]	TRAF6	--->	TRAFD1	0.70458	-0.71167
[13283]	PLAT	--->	SERPINI1	0.037242	-0.032776
[13284]	AKT1	--->	CDKN1B	1.0151	-1.0131
[13285]	HSP90AA1	--->	MAP3K14	0.040193	-0.056762
[13286]	VCL	--->	ACTA1	0.84173	-0.84519
[13287]	IL7R	--->	PIK3R1	0.35895	-0.39111
[13288]	SMAD2	<---	DYNC1H1	-0.32837	0.41128
[13289]	GAB2	<---	ETV6	-0.15462	0.1439
[13290]	FYN	--->	CDK5	1.3862	-1.2776
[13291]	YWHAB	<---	CSNK2A1	-0.66368	0.62776
[13292]	CSNK2A1	--->	HMOX2	0.70065	-0.72661
[13293]	TRIM37	--->	ZNF655	0.26552	-0.26229
[13294]	SRC	--->	CEACAM1	0.32027	-0.20338
[13295]	KDR	--->	GNAQ	0.67347	-0.61887
[13296]	PAK1	--->	ACTA1	0.50905	-0.3561
[13297]	MBD3	--->	RBBP7	0.055321	-0.020592
[13298]	TBP	--->	TP53	0.0022556	-0.01535

[13299]	DDIT3	<---	TRIB3	-0.93471	0.97322
[13300]	SKIL	<---	SKI	-0.25142	-0.043312
[13301]	PTPN14	--->	CTNNB1	0.19978	-0.20894
[13302]	NEO1	--->	NTN3	0.047334	-0.059841
[13303]	NPHS1	--->	NCK1	0.0073429	-0.034402
[13304]	FIGF	--->	ITGA9	0.013841	-0.024825
[13305]	IKBKG	<---	GSK3B	-0.91119	0.83996
[13306]	SH3BP2	<---	GRB2	-0.46269	0.48053
[13307]	HABP2	<---	COL4A6	-0.012629	-0.00035712
[13308]	GIPC1	<---	NTRK1	-0.030169	0.0297
[13309]	GRM1	<---	ADORA1	-0.45788	0.48933
[13310]	FEM1B	<---	APAF1	-0.012153	0.017367
[13311]	ARAF	--->	CPS1	0.0052031	-0.026177
[13312]	DLST	--->	DLG5	0.037198	-0.017188
[13313]	IKBKB	--->	IRS1	0.56786	-0.6179
[13314]	COPS6	--->	TP53	0.44919	-0.44065
[13315]	FOXM1	<---	STAT3	-0.02811	-0.016166
[13316]	PCBP2	?	HNRNPK	-0.037462	-0.037462
[13317]	PRKCB	--->	NCF1	0.51538	-0.52599
[13318]	BEGAIN	--->	CATSPER1	0.038696	-0.0046269
[13319]	ABL1	<---	HCK	-0.78293	0.63857
[13320]	APPL1	--->	MTA2	0.085684	-0.041663
[13321]	PKD1	<---	PTK2	-0.087868	0.020202
[13322]	IL6ST	<---	HCK	-0.40397	0.39113
[13323]	PAWR	<---	PRKCZ	-0.27969	0.26626
[13324]	PAK3	<---	NCK1	-0.47107	0.51692
[13325]	GAPDH	--->	ACTB	0.32036	-0.32418
[13326]	DNM3	<---	HOMER1	-0.065632	0.055895
[13327]	PALLD	--->	ACTN1	0.035561	-0.032381
[13328]	RIN1	<---	RAB5B	-0.99801	1.0021
[13329]	ATM	<---	CREB1	-0.054665	0.041638
[13330]	PICK1	--->	PVRL4	0.064042	-0.062655
[13331]	CRKL	--->	ITGB1	0.05275	-0.067754
[13332]	PTK2B	--->	MAP3K4	0.13745	-0.16342
[13333]	CREBBP	--->	HOXB7	0.18505	-0.18943
[13334]	PKP3	--->	DSC1	0.61098	-0.61498
[13335]	SP1	--->	MYOD1	0.27942	-0.28872
[13336]	NFKB1	--->	RELB	0.12864	0.0031239
[13337]	SKP2	--->	RB1	0.50486	-0.50639
[13338]	YAP1	--->	HNRNPU	0.65979	-0.65047
[13339]	HNRNPK	<---	FYN	-0.97195	1.133
[13340]	NCOA3	<---	YWHAH	-0.22568	0.21359
[13341]	PARD3	<---	STK11	-0.2738	0.28207
[13342]	IFT140	--->	CELSR3	-0.003168	-0.0068881
[13343]	NR2E3	<---	NCOR1	-0.065644	0.063164
[13344]	PRKACA	--->	PPP1R8	0.7025	-0.72853
[13345]	IPO5	--->	NUP214	0.05083	-0.045448
[13346]	RNF11	--->	SNRNP70	0.20794	-0.17771
[13347]	CASP3	--->	TGM2	0.59419	-0.70521
[13348]	MAPK1	<---	PAK2	-1.3014	1.4008
[13349]	PTPRA	--->	GRB2	0.40905	-0.558
[13350]	MAST1	--->	SNTB2	0.1406	-0.092397
[13351]	THRA	<---	HDAC2	-0.074402	0.085304
[13352]	EP300	--->	NR4A1	0.17826	-0.18496
[13353]	FOXO4	--->	ESR1	0.26449	-0.26658
[13354]	RAB11FIP4	--->	RAB11FIP3	0.063365	-0.086359
[13355]	RECQL5	--->	MTSS1	0.049124	-0.033834
[13356]	TP53	<---	LAMA4	-0.35386	0.36521
[13357]	ABL1	--->	CDK5	-0.013323	-0.14811
[13358]	ARR3	<---	OPN1MW	-0.25831	0.27159
[13359]	TAGLN	--->	ACTA1	0.51886	-0.51601
[13360]	FOXG1	<--->	FOXH1	0.3478	0.3478
[13361]	ITCH	--->	NUMB	0.15983	-0.17126
[13362]	SPRY4	<---	RAF1	-0.062206	0.037199
[13363]	IL12RB1	--->	STAT4	0.47869	-0.51446
[13364]	MYO16	<---	PPP1CC	-0.14575	0.13449
[13365]	VRK1	--->	PLK3	0.23606	-0.12147
[13366]	SVIL	--->	FLOT2	0.12602	-0.096306
[13367]	MMP7	<---	PLG	-0.11212	0.11927
[13368]	BCL2L11	--->	BCL2	0.16739	-0.17223
[13369]	CASP7	--->	MEF2D	0.10147	-0.12731
[13370]	BAZ1B	<---	SMARCA5	-0.0096565	0.0023298
[13371]	AFAP1	<---	PRKCZ	-0.33344	0.34665
[13372]	CNKSR1	--->	STK3	0.099408	-0.10472
[13373]	MAGI3	<---	BAI1	-0.0073329	0.007752
[13374]	LIN7C	<---	HTR2C	-0.19778	0.21221

```
[13375] ROCK1   --->    RDX     -0.0022587      -0.012307
[13376] TAOK2   --->    MEGF10  0.0027118       -0.016874
[13377] PTPRC   --->    LYN     1.007   -0.99313
[13378] BNIP3   --->    BCL2L1  0.68788 -0.67957
[13379] SERPINF2        <---    CFD     -0.082185       0.11833
[13380] IRS1    --->    ATP2A1  0.13686 -0.14511
[13381] PTPN2   --->    ITGA1   0.033943        -0.047085
[13382] CEBPB   --->    ATF2    0.34768 -0.34627
[13383] PLCB2   <---    MAP2K6  -0.64776        0.65475
[13384] CASP3   --->    IL18    0.56414 -0.67524
[13385] LCP2    <---    SYK     -1.0144 1.0092
[13386] NPHS1   <---    YES1    -0.0667 -0.02858
[13387] HEY1    <---    FBXW7   -0.30257        0.29912
[13388] HS1BP3  --->    HCLS1   0.04375 -0.013168
[13389] SFRS11  ?       SFRS1   -0.016298       -0.016298
[13390] UBE3A   --->    SMAD9   0.37907 -0.39667
[13391] CD28    <---    PTPRC   -0.44433        0.43997
[13392] ELK1    <---    MAPK11  -0.88927        0.89407
[13393] RAVER1  <---    VCL     -0.51569        0.53704
[13394] EIF2AK2 <---    CDC42   -1.0074 1.0132
[13395] DRD3    <---    GRB2    -0.02779        0.038831
[13396] SH3GL2  <--->   BIN1    0.0094254       0.0094254
[13397] SYNJ2BP --->    ACTB    0.53937 -0.53502
[13398] GNA11   <---    BDKRB2  -0.98951        0.99249
[13399] ARHGAP1 --->    BAI2    0.10624 -0.10384
[13400] AKT2    <--->   AKT1    0.20101 0.20101
[13401] PTPN11  <---    MAPK3   -1.0142 1.0027
[13402] VAV1    <---    INSR    -0.11857        0.066056
[13403] INHBA   --->    ACVR1   0.60319 -0.60916
[13404] CD2     <---    LCK     -0.67126        0.68982
[13405] CASP7   --->    RB1     0.41973 -0.45426
[13406] PRPF4B  --->    HSPB3   1.0388  -0.99926
[13407] SART3   --->    AR      0.95404 -0.95476
[13408] CALM1   --->    GLP1R   0.25258 -0.24765
[13409] RAP1GDS1        <---    MBIP    -0.040506       0.072762
[13410] CCR3    --->    CCL7    0.49595 -0.46475
[13411] ITGB5   <---    FLNA    -0.043452       0.040949
[13412] AXL     --->    NCK2    0.036899        -0.070771
[13413] SHB     --->    IL2RB   0.099417        -0.17887
[13414] SRC     <---    CBL     -0.40259        0.40313
[13415] MAPK9   --->    ATF2    1.0089  -1.0006
[13416] HRH4    --->    GNA15   1.0129  -1.008
[13417] TNFSF11 --->    ADAM19  0.21069 -0.19267
[13418] PIAS3   --->    NCOA2   0.039014        -0.049546
[13419] GRAP2   --->    USP8    0.27045 -0.25594
[13420] GSK3B   --->    CTNNB1  0.99917 -0.99713
[13421] CSH1    --->    SMAD4   0.26049 -0.21558
[13422] PTPRZ1  --->    CTNNB1  0.37022 -0.37559
[13423] HSPB8   <---    CSNK2A1 -0.99926        1.0388
[13424] AGAP2   --->    PLCG1   0.038365        -0.057144
[13425] GFRA1   <---    RET     -0.26357        0.25259
[13426] NBN     --->    PRKDC   0.006588        -0.0024509
[13427] RAB22A  <---    SMURF2  -1.0084 1.0007
[13428] WFIKKN2 --->    MSTN    0.17311 -0.17467
[13429] GSK3B   --->    PSEN1   0.67463 -0.70045
[13430] RAB11FIP2       --->    MYO5B   0.038221        -0.019313
[13431] GHR     <---    TYK2    -0.083461       0.071588
[13432] PRKACA  --->    STMN1   1.0058  -0.99021
[13433] UBE2I   <---    TNFRSF1A        0.087935        0.096234
[13434] UBTF    --->    RB1     0.52072 -0.52236
[13435] HNRNPA1 ?       PABPN1  -0.016298       -0.016298
[13436] COPS5   --->    TP53    0.44919 -0.44065
[13437] PTPRB   --->    MET     0.44701 -0.46104
[13438] NPHP1   --->    UBQLN4  0.099201        -0.08803
[13439] SMARCA4 --->    CEBPB   0.019297        -0.024673
[13440] YWHAE   <---    SORBS2  -0.1699 0.16586
[13441] ARL4D   --->    UBR1    0.36131 -0.33466
[13442] PPP1CA  --->    ID2     0.33803 -0.34432
[13443] GNA15   <---    ADRB2   -0.98627        0.9872
[13444] PVRL3   <--->   PVRL2   0.0013613       0.0013613
[13445] RAB34   --->    TGFBR1  0.010625        -0.037483
[13446] KPNA1   --->    PAX5    0.21934 -0.1984
[13447] TRAF2   --->    ZBTB16  0.05308 -0.062258
[13448] IKZF1   --->    TBP     -0.0055364      -0.0073621
[13449] ABCE1   <---    RNASEL  -0.15704        0.1369
[13450] RELA    <---    ACTL6A  -0.038283       0.043124
```

[13451] ACTB <--- CFL2 -0.30587 0.30201
[13452] ANKRD11 <--- HDAC4 -0.13768 0.15653
[13453] DLGAP3 <---> DLGAP1 0.0025079 0.0025079
[13454] IL17RB <--- TRAF6 -0.69822 0.69125
[13455] IL2RG <--- IL4R -0.11231 0.094332
[13456] GAB1 ---> PIK3R1 0.04059 -0.059832
[13457] TNFRSF1B <--- LTA -1.0119 1.0002
[13458] YWHAE <--- RAF1 -0.051212 0.022119
[13459] TGFB2 ---> TGFBR1 0.72054 -0.71391
[13460] SPARC <--- COL3A1 -0.043682 0.034121
[13461] AATF ---> MAPT 0.1116 -0.10456
[13462] MAP3K5 ---> ARRB2 0.1574 0.088914
[13463] STAT5B <--- PTPN6 -1.0146 1.0012
[13464] EPB41L3 <--- CNTNAP2 -0.035916 0.040248
[13465] TBP <--- TAF1 -0.0094054 -0.0039181
[13466] AKAP6 ---> PDE4D 0.18877 -0.15233
[13467] MAPK1 ---> FRS3 1.0012 -1.0103
[13468] POMT2 <---> POMT1 0.0010064 0.0010064
[13469] EPS8 <--- ITGB5 -0.038477 0.0090569
[13470] ESR1 <--- PRKACA -1.0079 0.99834
[13471] PEBP1 <--- MAP2K1 -0.17448 0.56179
[13472] ESR1 ---> POU4F1 0.0055155 -0.0053295
[13473] FN1 <--- COL4A2 -0.048751 0.04098
[13474] PKD1 ---> CDH1 0.08425 -0.085942
[13475] HDAC4 ---> MEF2A 0.072487 -0.072318
[13476] MAPK12 ---> GRB2 0.15548 -0.28096
[13477] IFNAR1 <--- ZAP70 -0.98188 0.96597
[13478] MOBKL1B <--- MST1 -0.082921 0.096789
[13479] PIAS2 ---> MSX2 0.17899 -0.1685
[13480] ATXN1 <--- TRIP6 -0.040288 0.037243
[13481] IFNAR1 <--- LCK -0.59947 0.6379
[13482] COL7A1 <--- FBXL2 -0.0068727 -0.0056444
[13483] ATXN1 <--- RAD54L2 -0.024042 0.016533
[13484] MATK ---> CD36 1.109 -0.94926
[13485] TRAF3 <--- TICAM1 -0.064443 0.05457
[13486] FYN ---> CD79B 1.0799 -1.0399
[13487] TGFB2 ? BMP2 -0.061425 -0.061425
[13488] PIK3CB <--- PIK3R1 -0.034565 0.0096742
[13489] RELB ---> KPNA6 0.38498 -0.38418
[13490] NOV <--- FN1 -0.46204 0.4519
[13491] DLG3 <--- CNKSR2 -0.020819 -0.0026013
[13492] RCHY1 ---> TP53 0.43056 -0.41971
[13493] ZFYVE9 ---> PPP1CA -0.0064486 -0.0065953
[13494] SH3GL3 ---> TERF1 0.073662 -0.059418
[13495] MAP3K7IP3 ---> C21orf7 0.05679 -0.019505
[13496] PEA15 <--- RPS6KA3 -0.50242 0.29142
[13497] FGFR1 <--- FGF7 -0.61054 0.60904
[13498] COL14A1 ---> CD44 0.010519 -0.022042
[13499] HGF <--- HPN -0.0091243 0.0012258
[13500] DLG4 <--- ACTN2 -0.031653 0.022285
[13501] NCOA1 ---> STAT3 0.057151 -0.076389
[13502] PKP4 ---> DSC3 0.61098 -0.61498
[13503] RNF216 <--- TLR5 -0.18096 0.19409
[13504] C8B ---> C8A -0.012945 -0.057269
[13505] ARL5A ---> KPNA2 0.49762 -0.47298
[13506] EPOR ---> STAT5B 0.2772 -0.29551
[13507] JUN ---> TCF4 0.1497 -0.18479
[13508] CTBP1 <--- EP300 -0.071355 0.060646
[13509] LIMS1 <--- ILK -0.43923 0.43884
[13510] NRAP ---> ACTB 0.54878 -0.54521
[13511] PARD3B <--- YWHAG -0.044889 0.0557
[13512] ID1 ? MYOD1 -0.65635 -0.65635
[13513] GTF2I <--- SRF -0.062868 0.085062
[13514] SORBS2 ---> PAK1 0.25826 -0.11716
[13515] CKAP5 <--- PTPRN2 -1.2611 1.2676
[13516] CCND3 <--- CDKN1A -0.99961 0.98767
[13517] STARD13 <--- HSP90AB1 -0.013744 0.010104
[13518] KAT5 <--- MDM2 -0.080351 0.073011
[13519] MATK ---> ERBB2 0.63574 0.0073254
[13520] CREBBP ---> CSK 0.079863 -0.085392
[13521] ITGB4 <--- GRB2 -0.052861 0.039217
[13522] MAPK13 <--- MAPKAPK3 -0.41806 0.64901
[13523] CFL1 ---> ACTC1 0.30201 -0.30587
[13524] THBS1 <--- LAMB3 -0.18697 0.23898
[13525] MMP2 ---> FGFR1 0.10201 -0.10663
[13526] YWHAH ---> PARD6B 0.018815 -0.024432

```
[13527] CASP3   --->    CTNNB1  0.35648 -0.3675
[13528] SOX10   <---    POU3F3  -0.060464       0.063322
[13529] FGG     --->    ITGAM   0.077384        -0.088956
[13530] NTN4    --->    ADAMTSL4        0.051635        -0.058198
[13531] SLA2    <---    ZAP70   -0.40218        -0.1324
[13532] DTX3    --->    DTX2    0.0017289       -0.0016883
[13533] MAPK8   --->    BCL2    1.1791  -1.2078
[13534] GRK6    --->    RHO     0.21152 -0.27051
[13535] NUMB    <---    PRKCH   -0.42361        0.37732
[13536] PRKCD   --->    PEBP1   0.84277 -0.83157
[13537] MMP14   <---    CCL7    -0.16713        0.18806
[13538] SMAD2   <---    JUN     -0.035159       0.057717
[13539] SRC     --->    IKBKB   0.99783 -0.99597
[13540] TYK2    --->    PIK3R1  0.085841        -0.16481
[13541] NFKB1   --->    NFKBIE  0.77463 -0.041138
[13542] ATN1    --->    GRN     0.037326        -0.039573
[13543] CRTC2   <---    PPP3CA  -0.017183       0.0056399
[13544] LRP2    <---    GC      -0.39974        0.39857
[13545] SRC     --->    CDKN1B  0.36964 -0.33668
[13546] ITGB1   <---    ICAM4   -0.08989        0.078785
[13547] RHPN2   <---    SMURF1  -0.71122        0.70741
[13548] GRB10   --->    ELK1    0.13704 -0.18107
[13549] CASP6   <--->   CASP3   0.35273 0.35273
[13550] GIT1    <---    GRK5    -0.20018        0.1705
[13551] AKT1    --->    TERT    1.0008  -1.0008
[13552] UBE2L6  --->    PARK2   0.12521 -0.091377
[13553] EIF3I   <---    EIF4G1  -0.25468        0.28655
[13554] CXCR4   <---    ADRBK2  -0.42229        0.39714
[13555] PRMT1   --->    KHDRBS1 0.13822 -0.146
[13556] CD79B   <---    ZAP70   -0.99774        0.99897
[13557] CSNK2A1 --->    L1CAM   0.088336        -0.11794
[13558] CXCL12  <---    CXCR4   -0.46475        0.49595
[13559] ITGA2B  <---    CD36    -0.027564       0.016801
[13560] LMO2    --->    NHLH1   0.62562 -0.58677
[13561] MAP2K1  --->    RPS6KA4 0.92747 -1.0338
[13562] MAPK3   --->    RPS6KB1 1.002   -1.0109
[13563] MYO1E   --->    ACTB    0.37528 -0.38746
[13564] ATXN3   <---    ARHGAP19        -0.040834       0.073833
[13565] IFNG    --->    IFNGR2  0.92292 -0.92615
[13566] ACTL6B  <---    SMARCA2 -0.029019       0.018653
[13567] SMURF1  --->    RHOA    1.0007  -1.0084
[13568] CTRC    <---    PHKG1   -0.72828        0.70234
[13569] BTK     --->    VAV1    0.98501 -1.0133
[13570] KCNG3   ?       KCNB1   -0.025901       -0.025901
[13571] MAGI3   --->    GRIN2B  -0.0021991      -0.0062499
[13572] SNX6    <---    TGFBR2  -0.84501        0.90577
[13573] GOPC    --->    FZD8    0.16667 -0.18664
[13574] BMP4    <---    BGN     -0.66809        0.75395
[13575] RALGDS  <---    RRAS2   -1.0084 0.98547
[13576] MID2    --->    MID1    0.0028182       0.0025083
[13577] ERBB2   --->    ITGB4   0.033404        -0.046181
[13578] FN1     --->    SMAD4   0.3949  -0.31392
[13579] TBL1X   --->    TBL1XR1 -0.0027564      -0.0078643
[13580] CNKSR1  --->    STK4    0.037259        -0.045637
[13581] CREBBP  <---    RPS6KA3 -0.12955        0.11396
[13582] ATN1    --->    MEGF6   -0.0067506      -0.00712
[13583] EIF4G3  --->    EIF4A1  0.05612 -0.052606
[13584] PPP4C   --->    CCDC6   0.35678 -0.36374
[13585] RNF11   --->    UBQLN2  0.13586 -0.10319
[13586] AMBP    --->    FN1     0.050185        -0.021425
[13587] MYBPC3  --->    MYH9    0.0025322       -0.014313
[13588] TBP     --->    RXRA    -0.007501       -0.012303
[13589] PRPF40A --->    ZFHX3   0.16204 -0.15941
[13590] UBE2I   --->    PIAS2   0.008864        0.002536
[13591] PDK2    --->    PDHX    0.32371 -0.31137
[13592] GATA4   <---    NKX2-5  -0.015702       0.056147
[13593] ANK1    --->    ITPR3   0.067436        -0.080515
[13594] BID     <---    YWHAB   -0.11593        0.15245
[13595] RBM10   <---    UBQLN4  0.0063104       0.028117
[13596] MXD1    --->    MYCL1   -0.38957        -0.43013
[13597] USH1C   --->    CDH23   0.071664        -0.034955
[13598] TGFB1I1 <---    UBQLN4  -0.1371 0.13426
[13599] CTGF    <---    LRP1    -0.0091604      -0.004239
[13600] SPOCK2  --->    PLXNB3  0.069958        -0.035396
[13601] GNAI2   <---    AGTR2   -1.008  1.0129
[13602] RAF1    --->    ARRB2   0.00066958      -0.021683
```

[13603]	EFEMP2	<---	UBQLN4	-0.057902	0.09298
[13604]	NCL	<---	GZMA	-0.058011	0.060551
[13605]	TRAF2	<---	TNFRSF4	-0.53992	0.5393
[13606]	CDK20	<--->	CDK2	0.23429	0.23429
[13607]	LYST	<---	YWHAB	-0.19355	0.2164
[13608]	FHL3	--->	KLF8	0.13538	-0.12918
[13609]	RASIP1	<---	RRAS	-0.64033	0.64447
[13610]	BRD7	--->	DVL1	0.39153	-0.39148
[13611]	RPS6KA1	--->	GMFB	0.26248	-0.48635
[13612]	MYBL2	--->	TTF2	-0.0022011	-0.0052466
[13613]	STRN	<---	CAV1	-0.11755	0.14419
[13614]	PPP2CA	--->	RHOB	0.42845	-0.43116
[13615]	UBE2E1	<---	NEDD4	-0.086802	0.07541
[13616]	RELA	<---	DHX9	-0.064265	0.062309
[13617]	GABPA	--->	ATF1	0.072618	-0.044114
[13618]	RFFL	--->	SFN	0.14429	-0.15526
[13619]	SRC	--->	STAT5B	0.57328	-0.56898
[13620]	ITGA8	<---	FN1	-0.028333	0.016313
[13621]	GFI1B	<---	C20orf12	-0.041387	0.033253
[13622]	AXIN1	<---	CSNK1E	-0.12095	0.12779
[13623]	SERPINE2	<---	COL4A1	-0.061646	0.052584
[13624]	ISL2	?	ISL1	-0.017692	-0.017692
[13625]	RAPGEF6	--->	PTPN13	0.019046	0.0043839
[13626]	C2	<---	C5	-0.12343	0.13077
[13627]	HSPA1A	--->	DYX1C1	0.8467	-0.80459
[13628]	DGKZ	--->	RB1	0.17136	-0.18027
[13629]	SVIL	<---	GNAI2	-0.08235	0.12059
[13630]	RHEB	--->	RPTOR	0.99036	-1
[13631]	LYN	--->	SYK	1.0025	-1.0044
[13632]	TGFA	--->	MAGI3	0.038536	-0.028593
[13633]	NCOA2	<---	ESRRB	-0.043163	0.034596
[13634]	NRXN1	<---	SYT6	0.00636	0.012867
[13635]	HSP90B1	--->	APOB	0.014544	-0.01089
[13636]	CCL14	<---	CCR1	-0.46475	0.49595
[13637]	CSTF2	<---	CHD3	-0.0054503	-0.0047536
[13638]	CHUK	<---	EIF2AK2	-1.003	1.0117
[13639]	RBM7	<---	CCDC85B	-0.050711	0.077385
[13640]	SHC1	<---	GRAP	-0.2424	0.22879
[13641]	GTF3C2	--->	RB1	0.44879	-0.45052
[13642]	SPARC	<---	COL2A1	-0.026355	0.018764
[13643]	HIPK3	--->	FADD	0.18204	-0.46216
[13644]	MYF6	<---	CALM3	-0.22666	0.2676
[13645]	SLC9A3R2	<---	LPAR1	-0.41277	0.44636
[13646]	PRELP	--->	NID1	0.048394	-0.052301
[13647]	HSPA4	--->	NR3C2	0.5755	-0.5448
[13648]	POU2F1	--->	SOX2	-0.089966	-0.1696
[13649]	MECR	<---	HNF4A	-0.31507	0.32399
[13650]	CASP4	<---	CASP10	-0.40694	0.26698
[13651]	LATS2	<---	STK3	-0.99703	0.99704
[13652]	CSNK2A1	--->	CUX2	0.79979	-0.82765
[13653]	KLHL1	<---	TGFBR1	-0.4026	0.36718
[13654]	CEBPB	<---	CREBBP	-0.18961	0.18576
[13655]	QKI	<---	USH1C	-0.10443	0.10947
[13656]	FBXW8	<---	SKP1	-0.024382	0.018964
[13657]	PBRM1	--->	SMARCA4	0.0036916	-0.0074712
[13658]	HSP90AA1	--->	IKBKE	0.020358	-0.034367
[13659]	RANBP2	<---	RAN	-0.10722	0.11054
[13660]	ADCY6	<---	ATXN1	-0.055044	0.04757
[13661]	PLAU	--->	SERPINB6	0.057207	-0.040728
[13662]	FBLIM1	--->	FLNA	0.066481	-0.030995
[13663]	WASF1	<---	IL8	-0.055875	0.069701
[13664]	YAP1	--->	RUNX2	0.69072	-0.68132
[13665]	FXR2	<---	CALCOCO2	0.0051862	0.030578
[13666]	ZNF689	<---	HNRNPU	-0.20513	0.20238
[13667]	AXL	--->	SOCS1	0.18064	-0.22188
[13668]	SH3GL3	--->	HTT	0.085985	-0.071715
[13669]	TOPORS	<---	UBE2I	-0.17337	0.20782
[13670]	FBXO25	<---	CUL1	-0.1587	0.15321
[13671]	EPHA2	<---	PTPN11	-0.16915	0.29856
[13672]	SH3KBP1	<---	CBL	-0.48677	0.48074
[13673]	RIT1	--->	KLHL12	0.46417	-0.43482
[13674]	PRKAR1A	--->	CCNE1	0.86441	-0.85226
[13675]	ITGA9	--->	PXN	0.0012691	-0.014059
[13676]	PTK2	--->	JAK2	0.062957	0.052768
[13677]	PRKCD	--->	ADRA2A	0.46078	-0.46051
[13678]	CHD4	<---	SMARCA4	-0.0069954	-0.0069328

[13679]	BMX	--->	RUFY2	0.47808	-0.56023
[13680]	CAMK2A	--->	EGFR	1.0058	-1.0028
[13681]	CTRL	--->	SERPINA3	0.11833	-0.082185
[13682]	MAPK8	--->	MAPK14	1.0074	-0.9992
[13683]	RRAGD	<--->	RRAGB	0.010143	0.010143
[13684]	DBN1	--->	PFN1	0.040193	-0.0076096
[13685]	RND1	--->	PLXNA1	0.41051	-0.37902
[13686]	MYC	<---	TBP	-0.013086	-3.9148e-005
[13687]	CASP10	--->	TRAF1	0.50472	-0.49225
[13688]	EGR1	<---	SP1	-0.037055	0.10897
[13689]	SLC2A4	<---	STRN3	-0.084472	0.085112
[13690]	MYBL2	--->	CCNA2	0.2333	-0.23718
[13691]	PDP1	<---	PRKCD	-0.73033	0.74075
[13692]	ARHGEF11	<---	PLXNB2	-0.010587	0.0085303
[13693]	ADAMTS1	--->	ACAN	0.0043435	-0.015468
[13694]	CXCR3	--->	PF4	0.49595	-0.46475
[13695]	YWHAE	<---	IRS1	-0.049509	0.055105
[13696]	ERBB4	--->	CTGF	0.078694	-0.091593
[13697]	GRAP2	--->	GNB2L1	0.38787	-0.37569
[13698]	SPI1	<---	CEBPA	-0.052983	0.11084
[13699]	MTA3	<---	GRB2	-0.37216	0.39013
[13700]	HOMER2	--->	DBN1	0.032965	0.001493
[13701]	RIPK3	<---	TRAF2	-0.87248	0.88498
[13702]	E4F1	<---	RASSF1	-0.13133	0.12343
[13703]	LMO2	--->	EHMT2	0.16914	-0.17009
[13704]	TMEM189-UBE2V1	<---	TRAF6	-0.64329	0.63617
[13705]	TEC	<---	JAK1	-1.0003	0.99303
[13706]	CCBP2	--->	CCL11	0.49595	-0.46475
[13707]	UBE2I	--->	RANBP2	0.040093	-0.035646
[13708]	SVIL	--->	ODF2	0.094107	-0.064874
[13709]	TBL1XR1	<---	NCOR1	-0.034426	0.023295
[13710]	PRKAA2	--->	ACACB	0.13992	-0.15543
[13711]	TCF4	<---	HBP1	-0.38312	0.37883
[13712]	WASL	<---	GRB2	-0.36444	0.32619
[13713]	DLG1	<---	ACTN2	-0.031653	0.022285
[13714]	ITGB7	<---	FN1	-0.011166	0.013324
[13715]	VPS4A	<---	RND2	-0.36973	0.4002
[13716]	KPNA2	--->	NR3C1	0.048465	-0.023733
[13717]	KIT	--->	GRB2	-0.1804	-0.47434
[13718]	CCNB2	<---	CDK1	-1.0774	1.0544
[13719]	ZNF24	?	ZNF434	-0.093486	-0.093486
[13720]	TRPV4	<---	LCK	-0.27527	0.29717
[13721]	TGFA	--->	EGFR	1.005	-1.0102
[13722]	SPG20	--->	EPS15	0.075501	-0.085754
[13723]	PTPN11	--->	BCR	0.19284	-0.1134
[13724]	PTPN21	<---	BMX	-0.045315	-0.029523
[13725]	SYK	--->	GRB2	-0.1324	-0.40218
[13726]	AR	<---	HMGB1	-0.99126	0.99123
[13727]	CCNY	<---	CDK14	-1.1093	1.083
[13728]	HDAC4	<---	YWHAE	-0.043467	0.067622
[13729]	MAPK1	<---	DUSP16	-1.0004	1.0016
[13730]	MMP17	?	MMP2	-0.12951	-0.12951
[13731]	NCOR1	--->	NR3C1	0.063164	-0.065644
[13732]	VDR	--->	RXRA	1.003	-0.99549
[13733]	NFKBIA	<---	UBE2D3	-0.99915	0.99947
[13734]	ZEB2	--->	SMAD2	0.072366	-0.029947
[13735]	CSNK2A1	--->	MAX	1.5563	-1.5271
[13736]	YWHAG	<---	CDK16	-0.66368	0.62776
[13737]	JUN	<---	HOXC8	-0.25302	0.21945
[13738]	AKT1	--->	APLP2	0.066296	-0.058409
[13739]	SMARCD3	--->	NR1H4	0.052806	-0.042542
[13740]	CDK1	--->	TSC1	0.79117	-0.7903
[13741]	ARFRP1	--->	SMAD4	0.5744	-0.4943
[13742]	FYN	<---	CBL	-0.7389	0.74409
[13743]	PRKCD	--->	SRC	0.15359	-0.14943
[13744]	FLNA	<---	GP1BA	-0.056051	0.058034
[13745]	SPRED2	<---	KIT	-1.0166	1.0163
[13746]	TEK	--->	GRB2	-0.0041166	-0.015192
[13747]	CXXC5	--->	SMAD7	0.25495	-0.21011
[13748]	TGFBR2	--->	ENG	1.1279	-1.082
[13749]	MPDZ	<---	RNF5	-0.075273	0.091712
[13750]	NF2	<---	SDCBP	-0.071711	0.068892
[13751]	PPP3CA	--->	BAD	0.22491	-0.23594
[13752]	CDH2	<---	CTNND2	-0.61498	0.61098
[13753]	GNA13	--->	ARHGEF1	0.049948	-0.05671
[13754]	CDK1	--->	FOXM1	0.85888	-0.87871

```
[13755] EZH2    --->    GTF3C1  0.12441 -0.088442
[13756] RIPK1   <---    FAS     -0.2843 0.15215
[13757] UBQLN4  <---    DTX2    -0.040579       0.035113
[13758] SORBS2  <---    CBL     -0.32919        0.31754
[13759] GJA3    --->    GJA1    -0.0078479      -0.078897
[13760] RNF11   --->    ZNF350  0.26185 -0.26541
[13761] CRKL    <---    GRB2    0.22272 0.23706
[13762] HGS     <---    MAP3K7  -0.075158       0.048319
[13763] CSNK1G2 --->    NCK1    0.086175        -0.082356
[13764] MIA     <---    FN1     -0.03411        0.06413
[13765] ROBO2   <--->   ROBO1   0.0649  0.0649
[13766] ARL15   --->    UBR1    0.36131 -0.33466
[13767] GAB3    <---    LYN     -0.64147        0.75598
[13768] AKAP13  --->    ESR1    0.035086        -0.033914
[13769] ADRBK2  --->    OPRM1   0.25048 -0.27132
[13770] DAPK1   --->    UNC5A   0.009997        -0.015275
[13771] WWP2    --->    CPSF6   0.14567 -0.15206
[13772] RAC1    --->    PPP2R2B 0.23328 -0.18308
[13773] H3F3A   <---    CHD1    -0.04883        0.049463
[13774] TERF1   --->    ZBTB16  0.1203  -0.1218
[13775] FADD    --->    CASP10  1.0007  -1.0021
[13776] NFKB1   --->    HMGB1   0.77588 -0.76785
[13777] CNTN1   <---    TNC     -0.0079648      -0.0059918
[13778] KHDRBS1 <---    GRAP    -0.14707        0.12752
[13779] SMAD4   <---    PSG9    -0.29658        0.33439
[13780] PRKCA   --->    TRPV6   0.59235 -0.59608
[13781] PLD2    <---    GAPDH   -0.28495        0.27765
[13782] ATXN1   <---    NLK     -0.2064 0.18965
[13783] CCL4    ?       CCL3    -0.047195       -0.047195
[13784] IFNAR1  <---    PTPRC   -0.42852        0.41121
[13785] PPARGC1A        <---    FBXW7   -0.06257        0.050538
[13786] SOCS1   <---    FYN     -0.49174        0.82968
[13787] FZD8    <---    RYK     -0.25919        0.23116
[13788] SETDB1  <---    PCDHA4  -0.21906        0.23447
[13789] TRAF1   <---    TNFAIP3 -0.12362        0.12298
[13790] AGER    --->    HMGB1   0.0066998       -0.011729
[13791] BMPR1B  <---    RAB25   -0.53994        0.52577
[13792] BHLHE40 <---    ARNTL   -0.1616 -0.14881
[13793] CSPG4   <---    CDC42   -0.35255        0.38323
[13794] ACTR2   ?       ACTR3   -0.32501        -0.32501
[13795] NR1I2   <---    NCOR2   -0.14734        0.13736
[13796] MTNR1B  --->    COPA    0.87375 -0.84712
[13797] MEIS1   <---    HOXD4   -0.0094151      0.024726
[13798] PRKACA  --->    PDE4D   0.99264 -1.0111
[13799] ATN1    --->    MYST3   0.036611        -0.038848
[13800] XCR1    --->    XCL1    0.49595 -0.46475
[13801] BMPR1B  <---    PPP2CA  -0.21116        0.20095
[13802] CTSG    <---    F2R     -0.42368        0.45462
[13803] PLSCR1  --->    ADAMTSL4        0.024423        -0.014281
[13804] E4F1    <---    CDKN2A  -0.077211       0.069036
[13805] SFRS13A ?       TRA2A   -0.016298       -0.016298
[13806] CASP8   --->    CDH1    0.25858 -0.31925
[13807] UBQLN1  <---    PRPF40A -0.22895        0.26122
[13808] TRADD   <---    STAT1   -0.16329        0.080165
[13809] PXN     <---    ILK     -0.44478        0.44112
[13810] CLK1    --->    PIAS4   0.34781 -0.36824
[13811] DLGAP1  <--->   DLGAP2  0.0025079       0.0025079
[13812] RIPK2   --->    BCL10   0.13986 -0.4509
[13813] PRKCB   --->    ITGB2   0.054379        -0.07038
[13814] P2RY2   <---    KDR     -0.33448        0.36236
[13815] GOPC    <---    CLCN3   -0.72583        0.74042
[13816] TLE1    <---    FUBP1   -0.086464       0.11936
[13817] ITGB1   <---    ITGA5   -0.026593       -0.0095743
[13818] CSNK2A2 --->    MYC     1.0154  -1.0065
[13819] MYC     <---    SP1     -0.12555        0.1145
[13820] DLG2    --->    NOS1    0.029108        -0.042908
[13821] FYN     --->    PLCG2   0.59106 -0.8605
[13822] NR2E3   <---    CRX     0.012013        0.012582
[13823] IRS1    --->    RPTOR   0.12289 -0.13361
[13824] SP1     --->    MYOG    0.27942 -0.28872
[13825] NCOA1   <---    TEAD2   -0.086679       0.079238
[13826] ERRFI1  <---    ERBB2   -0.10747        0.097059
[13827] EPS15   <---    NEDD4   -0.022028       0.0084075
[13828] PTPN6   --->    TLR10   0.17555 -0.10865
[13829] PTPN1   --->    MAPK1   1.0061  -1.009
[13830] FGF8    --->    FGFR3   0.60861 -0.61087
```

[13831]	ACIN1	--->	ABCF3	0.17069	-0.12864
[13832]	MAPK8IP1	--->	TIAM1	0.27495	-0.28283
[13833]	GRB2	--->	SYP	0.30815	-0.29529
[13834]	KAT5	--->	CSTF2	0.024416	-0.010504
[13835]	RGS16	--->	GNAO1	1.0012	-1.0015
[13836]	SMAD4	<---	CTCF	0.0043727	0.016992
[13837]	LTBP1	--->	ITGB5	0.025738	-0.044308
[13838]	MET	--->	SNX2	0.39749	-0.40866
[13839]	BUB1B	--->	MAP3K4	0.050282	-0.38277
[13840]	SCN4A	<---	SNTB1	-0.14312	0.15288
[13841]	PRKACA	--->	NOS1	0.48839	-0.49502
[13842]	CMA1	--->	MMP1	0.21298	-0.1922
[13843]	SKP2	--->	CCNA2	0.70868	-0.69543
[13844]	CDY1	--->	HIST4H4	0.24617	-0.19484
[13845]	TOLLIP	<---	RBPMS	-0.041595	0.044896
[13846]	CUBN	--->	GC	0.016224	-0.012193
[13847]	VAV1	<---	CD19	-0.173	0.056138
[13848]	AKT1	--->	EIF4EBP1	0.49303	-0.34292
[13849]	FNTA	<---	HRAS	-0.44041	0.47292
[13850]	LTF	--->	CALM1	0.35368	-0.31629
[13851]	MAP3K7	--->	MAPK8	0.99376	-0.98749
[13852]	RGS20	--->	GNAI3	1.0012	-1.0015
[13853]	PPP2R1A	--->	TRADD	0.10858	-0.11822
[13854]	TLR4	--->	BCL10	0.34862	-0.44765
[13855]	TRPC1	--->	ITPR3	-0.0071319	-0.016919
[13856]	NOTCH1	<---	LCK	-0.10396	0.089451
[13857]	TP53	<---	CDKN2C	-0.13817	0.14916
[13858]	DDX18	<---	INADL	0.0043716	0.012589
[13859]	NCF1	<---	NFKB1	-0.28744	0.28141
[13860]	PTPN12	--->	PSTPIP1	0.30035	-0.30748
[13861]	AFAP1	<---	PRKCG	-1.0819	1.2556
[13862]	LCP2	--->	PLCG2	0.33399	-0.37787
[13863]	PRKCE	--->	ITGB1	0.056164	-0.082667
[13864]	WASF3	--->	ACTR3	0.089124	-0.077881
[13865]	ATXN1	<---	UBE2I	-0.078596	0.075361
[13866]	CNKSR2	--->	SHANK3	0.0022939	-0.005684
[13867]	PPM1A	--->	MSN	0.10633	-0.11549
[13868]	MEF2D	<---	SP1	-0.036919	0.023951
[13869]	FYN	--->	JUP	0.251	-0.22621
[13870]	LAMA1	<---	COL7A1	-0.0094033	-0.0037082
[13871]	PKN1	--->	CDC25C	0.0050881	-0.076912
[13872]	MPL	--->	SHC1	0.086569	-0.19034
[13873]	PDLIM1	--->	AGK	0.029896	-0.012335
[13874]	SOCS1	--->	GHR	0.031349	-0.038535
[13875]	ILK	--->	ITGB2	0.10006	-0.11317
[13876]	MAGI1	<---	CLCN5	-0.084799	0.07686
[13877]	KDM2A	--->	SMAD3	0.42793	-0.34495
[13878]	TGFA	--->	ADAM10	-0.0047281	-0.0052027
[13879]	AFAP1	--->	ACTA1	0.6814	-0.68593
[13880]	SP1	--->	BCOR	0.59916	-0.6177
[13881]	GRK5	--->	CHRM2	0.21152	-0.27051
[13882]	SYNJ1	<---	ITSN1	-0.04882	0.046828
[13883]	KCNJ9	<---	DRD4	-0.21396	0.22725
[13884]	CAPNS1	--->	CDK4	0.06607	-0.080926
[13885]	ZYX	--->	VASP	0.10335	-0.085959
[13886]	WIPF2	<---	DNMBP	-0.055138	0.037296
[13887]	PTK2B	--->	ERBB2	0.05046	0.038896
[13888]	SFRS2	<---	FUS	-0.02338	-0.0017915
[13889]	LRP8	<---	RELN	-0.10525	0.096805
[13890]	CRK	--->	PLSCR1	0.079404	-0.13604
[13891]	ETS1	<---	NCOR1	-0.078881	0.071387
[13892]	PIM2	--->	FBXO18	0.58677	-0.61295
[13893]	MTNR1A	--->	GNB4	0.87375	-0.84712
[13894]	HTR2A	<---	NTRK3	-0.043582	0.038109
[13895]	ARID3A	<---	SP100	-0.19758	0.23099
[13896]	NFIB	<---	RFX1	-0.054792	0.051672
[13897]	TNFSF13	--->	TNFRSF1A	0.99951	-1.0013
[13898]	PRKCE	--->	GRM5	0.62582	-0.69315
[13899]	RNF40	<---	SPRYD5	-0.0099979	0.025868
[13900]	PDE4A	<---	LYN	-0.63279	0.72686
[13901]	AKT1	--->	ITGB3	0.10202	-0.079994
[13902]	SFRS1	?	SNRNP70	-0.016298	-0.016298
[13903]	PDK1	--->	YWHAQ	0.27583	-0.26958
[13904]	FBLN2	<---	COL4A4	-0.041937	0.035051
[13905]	PRKAA1	--->	ACACA	0.14518	-0.16304
[13906]	HMOX1	--->	POR	0.27728	-0.28037

[13907]	FOS	<---	ATF2	-0.063012	-0.036232
[13908]	KCNJ12	--->	DLG2	0.04386	-0.037047
[13909]	IRAK1	--->	JUN	0.27776	-0.2302
[13910]	E2F2	<---	BCAR1	-0.021497	0.023691
[13911]	TSSK2	--->	TFG	0.38235	-0.75899
[13912]	BACE1	--->	LRP1	0.15766	-0.16689
[13913]	FOXO3	--->	SMAD4	0.2939	-0.27389
[13914]	BRD2	--->	HIST4H4	0.29322	-0.24229
[13915]	ARR3	--->	ZNF496	0.08602	-0.087683
[13916]	NEDD4	--->	IGF1R	0.010531	-0.0242
[13917]	PLEC1	--->	MSN	0.01246	-0.019645
[13918]	ARHGEF2	--->	YWHAZ	0.15751	-0.15298
[13919]	WRN	--->	H2AFX	0.14706	-0.12117
[13920]	NRXN1	<---	CASK	-0.14214	0.1271
[13921]	SMAD4	--->	HOXA9	0.20304	-0.14259
[13922]	ZMYND11	--->	SMARCA4	0.0014404	0.001416
[13923]	SH3D19	--->	ADAM10	-0.0028598	-0.011873
[13924]	ELF1	<---	NFKB1	-0.77477	0.78296
[13925]	BLNK	--->	VAV1	0.33991	-0.32645
[13926]	SERPINE2	<---	COL4A4	-0.073538	0.071404
[13927]	ATN1	--->	PCSK5	0.0076295	-0.01708
[13928]	CSNK2A2	<---	HSPH1	-0.99901	1.0063
[13929]	JUP	--->	DSG2	0.07062	-0.077057
[13930]	GPS2	--->	TP53	0.36414	-0.35282
[13931]	ABI1	--->	EPS8	0.12665	-0.17549
[13932]	PRKCE	--->	ACTA1	0.40221	-0.45121
[13933]	SOS2	<---	CRKL	-0.61023	0.60392
[13934]	CDC42EP2	<---	RHOQ	-0.87115	0.57849
[13935]	PDPK1	--->	AKT3	0.99822	-0.99934
[13936]	HDAC2	--->	MTA1	0.11539	-0.094626
[13937]	CDC42EP2	<---	CDC42	-0.87115	0.57849
[13938]	EP300	--->	MYBL2	0.062447	-0.072857
[13939]	STAT1	--->	TNFRSF1A	0.086379	-0.15275
[13940]	GRB10	<---	MAP3K14	-0.34335	0.17357
[13941]	YWHAE	--->	IGF1R	0.024667	-0.036569
[13942]	ENAH	<---	ZYX	-0.019031	0.0093472
[13943]	NFATC2	--->	EGR4	0.11655	-0.12568
[13944]	SMAD3	<---	HIVEP1	-0.12495	0.20566
[13945]	IKZF5	<---	IKZF3	-0.10358	-0.033634
[13946]	CDK9	--->	NBN	0.7025	-0.72853
[13947]	SRC	--->	AKT1	0.99355	-0.99803
[13948]	HSPA4	<---	TRAF6	-0.50145	0.49624
[13949]	GAPDH	<---	CAMK1	-0.44451	0.4233
[13950]	KAT5	<---	PTPN4	-0.17966	0.16895
[13951]	PLAU	<---	PLAT	-0.0096131	-0.0087417
[13952]	RBL2	<---	RAF1	-0.11816	0.10232
[13953]	TNFSF14	--->	TNFRSF6B	0.45861	-0.52454
[13954]	PZP	<---	KLK3	-0.11653	0.14985
[13955]	PIK3CB	--->	PRKCD	0.036254	-0.037311
[13956]	KIF15	--->	MAP1LC3A	0.16076	-0.1292
[13957]	KLK3	--->	SERPINA3	0.11833	-0.082185
[13958]	IGSF1	--->	INHBB	0.41678	-0.38696
[13959]	MPDZ	<---	DRD3	-0.41277	0.44636
[13960]	MYBL2	--->	CCND1	0.2333	-0.23718
[13961]	CCR4	--->	CCL3	0.45214	-0.43752
[13962]	RAP1GAP	--->	MEX3B	0.21246	-0.21918
[13963]	GATA4	<---	SRF	-0.13212	0.15844
[13964]	ABI3BP	--->	ABI3	0.0092269	-0.013249
[13965]	PAM	--->	FXYD1	0.20355	-0.1643
[13966]	GABPB1	<---	BAI2	-0.21666	0.2296
[13967]	PKP2	--->	CTNNB1	-0.0025414	-0.33209
[13968]	CD93	<---	MSN	-0.010107	-0.0014566
[13969]	E4F1	<---	TP53	-0.030171	0.020776
[13970]	GNB2L1	--->	CSF2RB	0.097106	-0.10916
[13971]	RAB25	<---	SMURF2	-1.0084	1.0007
[13972]	SHC1	<---	FCGR2A	-0.46105	0.30147
[13973]	MAPK1	--->	SREBF1	0.54159	-0.54127
[13974]	MBD3	?	MBD2	-0.10949	-0.10949
[13975]	HDAC3	--->	STAT3	0.56387	-0.61711
[13976]	TRAF2	--->	FXR2	0.15142	-0.14572
[13977]	BAD	--->	BCL2	1.0001	-0.99613
[13978]	FGF9	--->	FGFR3	0.60861	-0.61087
[13979]	THOC1	--->	RB1	0.47495	-0.48865
[13980]	ZAP70	<---	PTPRC	-0.78502	0.68417
[13981]	MAPK12	--->	INADL	0.36823	-0.49063
[13982]	EP300	--->	TP63	0.06489	-0.074886

[13983]	PPP2R1B	--->	RAB11A	0.13801	-0.13281
[13984]	POU5F1	<---	HMGB1	-0.26954	0.30083
[13985]	TGM2	--->	ALDOA	0.022486	0.011489
[13986]	GADD45A	<---	CDKN1A	-0.0109	0.021707
[13987]	MAPK3	--->	CASP9	0.99863	-0.99771
[13988]	MAPK8IP3	<---	MAP3K5	0.028845	0.053142
[13989]	STK11	--->	PRKAA2	0.99921	-1.002
[13990]	USP9X	--->	SMAD4	0.45045	-0.3825
[13991]	TRIM32	--->	UBQLN1	0.21727	-0.21907
[13992]	ATN1	--->	DVL2	0.051641	-0.062984
[13993]	APBB1IP	--->	EVL	1.0039	-0.99848
[13994]	ANTXR1	<---	BRCA1	-0.020304	0.036052
[13995]	SMAD2	--->	SKIL	0.0719	-0.06155
[13996]	IGF2	--->	IGFBP1	0.11742	-0.083423
[13997]	MAP2K7	<---	MAP3K11	-0.99593	0.99711
[13998]	SCN8A	<---	GNAO1	-0.18377	0.1992
[13999]	RALGDS	<---	RAP2A	-1.0084	0.98547
[14000]	NCOR1	--->	ACTN2	0.0023645	-0.013428
[14001]	CAV1	<---	TGFBR1	-0.37965	0.34433
[14002]	RHOD	--->	SMAD2	0.55322	-0.47159
[14003]	HIPK2	--->	TP53	1.0002	-0.99962
[14004]	DMPK	--->	ATXN1	0.18965	-0.2064
[14005]	CRTC2	--->	YWHAG	0.071582	-0.070272
[14006]	PTPRS	--->	PTPRM	0.034468	0.033851
[14007]	GRB10	--->	DOK1	0.15647	-0.23363
[14008]	PPARGC1A	--->	PPARA	0.013278	-0.021031
[14009]	ADAM15	<---	LYN	-0.53832	0.63049
[14010]	BTAF1	--->	TBP	-0.0030861	-0.0098329
[14011]	EZH2	--->	WDR61	0.24227	-0.20733
[14012]	MARK4	--->	HPS6	0.05029	-0.064366
[14013]	RHPN1	<---	CNKSR1	-0.17117	0.17271
[14014]	MYF6	?	ID1	-0.65635	-0.65635
[14015]	PCOLCE	<---	COL3A1	-0.032536	0.021912
[14016]	PIK3R1	<---	FASLG	-0.33	0.22579
[14017]	EPHB2	--->	ARHGEF6	0.072503	-0.089766
[14018]	SMARCA4	--->	CTNNB1	0.068088	-0.079984
[14019]	MAP3K10	--->	CEACAM1	0.13136	-0.14266
[14020]	CLEC4M	<---	ICAM3	-0.071641	0.066003
[14021]	FRS3	<---	NTRK1	-0.12806	0.12396
[14022]	PLAGL1	--->	TP53	0.31933	-0.3074
[14023]	PPARGC1A	--->	USF2	0.1323	-0.13917
[14024]	ADAM12	<---	YES1	-0.53832	0.63049
[14025]	BRCA1	--->	KPNA6	0.059934	-0.045225
[14026]	FMNL1	<---	RAC1	-0.15739	0.15996
[14027]	PDGFRA	--->	CRK	-0.0068636	-0.0074943
[14028]	RNF11	--->	OPTN	0.33959	-0.31127
[14029]	CD44	<---	PKN1	-0.029237	-0.0065749
[14030]	PRKCB	--->	IFNAR2	0.60897	-0.61477
[14031]	MAPK1	<---	GAB1	-0.19052	0.17127
[14032]	WASF1	<---	RAC1	-0.3163	0.32786
[14033]	ABL1	--->	JUN	-0.017125	-0.08419
[14034]	DSC3	<---	JUP	-0.1649	0.15626
[14035]	WASL	--->	VIPR1	0.028172	-0.047735
[14036]	NTRK1	--->	SH2B2	0.043151	-0.24591
[14037]	PFN2	--->	WDR33	0.011404	-0.0084964
[14038]	MSN	<---	NCF1	-0.018581	0.0057655
[14039]	BCR	<---	GRB2	-0.12163	0.15712
[14040]	KCNJ2	<---	DLG3	-0.04695	0.04551
[14041]	EIF4A3	--->	RBM8A	0.015842	-0.0019217
[14042]	ELK1	<---	MAPK1	-0.98814	1.0428
[14043]	SMAD3	<---	JUNB	-0.035159	0.057717
[14044]	TAF1L	--->	TBP	-0.0039181	-0.0094054
[14045]	MX1	--->	ACTB	0.038077	-0.047265
[14046]	VAV3	<---	RAC1	-0.14624	0.08839
[14047]	TGFBR1	--->	FNTA	1.0094	-1.0042
[14048]	GTF2I	--->	MYC	0.28237	-0.27146
[14049]	DLGAP2	<---	DLG4	-0.040133	0.038414
[14050]	KLK3	--->	IGFBP3	0.070438	-0.036479
[14051]	MLX	<---	GABARAPL2	-0.43534	0.47031
[14052]	RUNX2	--->	MAP3K4	0.30213	-0.31055
[14053]	GABPB1	<---	YAF2	-0.61802	0.65136
[14054]	RET	--->	GDNF	0.48119	-0.48968
[14055]	YWHAZ	<---	ADRA2B	-0.16522	0.17198
[14056]	CLEC4M	--->	ITGAM	0.043438	-0.055942
[14057]	DEGS1	<---	EGFR	-0.27911	0.27822
[14058]	KIT	<---	PTPRU	-0.2507	0.22549

[14059]	SF1	<---	FUS	-0.050464	0.053517
[14060]	MAPK3	--->	PRKCE	0.63541	-0.033187
[14061]	KIF1B	--->	DLG4	-0.0064478	-0.013466
[14062]	ARHGAP15	<---	TGFBR1	-0.20535	0.16336
[14063]	NUP153	--->	NXF1	0.052625	-0.018909
[14064]	CSNK1D	--->	BACE1	0.69091	-0.55457
[14065]	TNFSF13	--->	TNFRSF11B	0.40687	-0.45752
[14066]	NFKB1	--->	KPNA3	0.78002	-0.772
[14067]	GRIN1	<---	DRD1	-0.33699	0.34286
[14068]	UBE2K	<---	NFKB1	-0.076386	0.073425
[14069]	PACSIN1	<---	SOS1	-0.32202	0.31511
[14070]	IFNAR2	<---	TYK2	-0.18353	0.17162
[14071]	SRY	--->	AR	0.99123	-0.99126
[14072]	F11	--->	SERPINA5	0.11833	-0.082185
[14073]	OPRM1	--->	GNAO1	0.9872	-0.98627
[14074]	PRKCZ	--->	STAT6	0.11023	-0.12226
[14075]	NPAS2	--->	NCOA3	-0.0080467	-0.08503
[14076]	UBQLN4	--->	ADAM33	0.011451	0.0041262
[14077]	PRSS2	--->	MMP9	0.21298	-0.1922
[14078]	TMOD1	<---	NEB	-0.12065	0.15304
[14079]	ACAP1	<---	TFRC	-0.018272	0.0061104
[14080]	AXIN1	<---	CSNK1A1	-0.11943	0.11894
[14081]	DAB2	<---	MYO6	-0.03643	0.052546
[14082]	RERE	--->	ZMYND8	0.047937	-0.027139
[14083]	EGFR	<---	EGF	-0.99332	0.99244
[14084]	GSK3B	<--->	CDK5	0.23429	0.23429
[14085]	MAPK14	--->	STAT4	0.98606	-1.0018
[14086]	HOXA10	<---	CREBBP	-0.11913	0.11102
[14087]	SLC8A2	<---	YWHAZ	-0.092105	0.10171
[14088]	BCL2L10	<---	APAF1	-0.19166	0.1957
[14089]	TSC1	--->	EZR	0.02248	-0.015695
[14090]	GADD45G	--->	PPARG	0.0090803	0.0011057
[14091]	MAST2	<---	TRAF6	-0.61717	0.59447
[14092]	HNRNPC	--->	KPNA3	0.042476	-0.012581
[14093]	TLX1	<---	PPP1CB	-0.23737	0.22986
[14094]	CDK1	--->	PTCH1	0.50317	-0.51297
[14095]	NCOR2	--->	PPARD	0.13736	-0.14734
[14096]	PRKCB	--->	ITGB7	0.33316	-0.34136
[14097]	DOK4	<---	SRC	-1.0017	1.009
[14098]	PARVB	<---	ILK	-0.41044	0.40608
[14099]	KRT10	--->	SMAD3	0.41607	-0.33313
[14100]	ITGAV	<---	ITGB5	-0.01057	-0.0026958
[14101]	CDC7	<--->	CDK4	0.23429	0.23429
[14102]	BCR	--->	MLLT4	0.053585	-0.053555
[14103]	MDM4	--->	E2F1	0.03615	-0.044096
[14104]	DNMT3A	--->	MYC	0.29262	-0.28227
[14105]	PIK3R2	<---	CSF1R	-0.37073	0.36447
[14106]	SYT1	--->	NR3C1	0.10325	-0.073631
[14107]	ACP5	--->	ZBTB16	0.13599	-0.1375
[14108]	CASP9	<---	CASP10	-0.40694	0.26698
[14109]	CD4	--->	PIK3R1	0.08802	-0.15912
[14110]	L3MBTL	--->	RB1	0.31145	-0.31721
[14111]	INSR	<---	CBL	-0.20671	0.19508
[14112]	EFS	<---	PTK2B	-0.049536	-0.00037625
[14113]	PRKDC	--->	ABL1	-0.027382	-0.036432
[14114]	ARHGEF7	<---	SHANK2	-0.012374	-0.010098
[14115]	RET	--->	CRK	0.17744	-0.29385
[14116]	PTK2	<---	GRB2	-0.43317	0.0087435
[14117]	CRX	<---	EP300	-0.18943	0.18505
[14118]	ANKRD6	<---	CSNK1E	-0.33129	0.411
[14119]	UBE2I	--->	DNMT3B	0.19821	-0.16458
[14120]	PRPF40A	--->	HTT	0.32543	-0.29315
[14121]	KCNG3	?	KCNF1	-0.025901	-0.025901
[14122]	UBE2E3	--->	RNF5	0.12227	-0.11995
[14123]	GNA12	<---	PRKCA	-0.89029	0.89037
[14124]	PTPRF	--->	PRPF40A	0.029239	-0.036603
[14125]	PRKCZ	--->	YWHAB	0.25396	-0.26837
[14126]	PI4KA	--->	STRN4	0.018717	-0.021093
[14127]	PACSIN2	<---	FASLG	-0.12462	0.14108
[14128]	PER3	<---	CSNK1D	-0.58384	0.72042
[14129]	L3MBTL	--->	ETV6	0.11316	-0.11328
[14130]	GIPC1	--->	LRP2	0.41365	-0.41429
[14131]	CBX1	<--->	CBX3	0.0018385	0.0018385
[14132]	RALGDS	<---	RRAS	-1.0084	0.98547
[14133]	MAPK3	<---	PPP1CA	-0.067136	0.046927
[14134]	WT1	<---	TP73	-0.019382	0.018391

[14135]	KDR	--->	COL18A1	0.086883	-0.09098
[14136]	RAB4A	--->	TP53	0.5057	-0.49562
[14137]	ERBB3	--->	FLNA	0.27998	-0.28085
[14138]	CDK2	--->	MYOD1	1.5563	-1.5271
[14139]	PIAS4	--->	SMAD4	0.9933	-1.0027
[14140]	PAK1	--->	COL1A1	0.063717	-0.038147
[14141]	ABCA1	<---	GOPC	-0.0080068	0.005539
[14142]	CABIN1	<---	PPP3CC	-0.11071	0.10813
[14143]	ESRRG	--->	NCOA1	0.034596	-0.043163
[14144]	MAP2K1	<---	TRAF6	-0.59578	0.66379
[14145]	CTSD	--->	INS	0.35706	-0.34709
[14146]	KAT2B	--->	ACTN2	0.0039043	-0.012397
[14147]	PAK1IP1	<---	PAK1	-0.16671	0.33014
[14148]	CSNK2A1	--->	IL16	0.62013	-0.64226
[14149]	GFI1B	<---	LTBP4	-0.074844	0.059215
[14150]	CDC42EP1	<---	RHOQ	-0.87115	0.57849
[14151]	RPS6KA4	--->	FOS	0.29655	-0.38418
[14152]	MKI67	<---	CBX5	0.014534	0.019326
[14153]	ST13	<---	CD101	-0.31322	0.2823
[14154]	TBP	<---	POU2F2	-0.0086868	-0.0036228
[14155]	RAC3	--->	HNF1A	0.49898	-0.47042
[14156]	IL1F7	--->	SMAD3	0.43257	-0.34971
[14157]	CBL	--->	TNFRSF11A	0.32332	-0.33561
[14158]	TEX11	--->	STMN2	0.16425	-0.1439
[14159]	BHLHE41	<---	MYOD1	-0.43036	-0.38951
[14160]	MAPK14	--->	MAPKAPK5	1.008	-1.0015
[14161]	KLF1	<---	SMARCC1	-0.14726	0.15317
[14162]	CDK1	--->	ITPR1	0.3288	-0.33886
[14163]	ABI1	<---	NEB	-0.074449	0.061532
[14164]	EPAS1	--->	MAX	0.20114	-0.20998
[14165]	AATF	--->	RB1	0.27378	-0.27997
[14166]	TES	<--->	ZYX	0.029854	0.029854
[14167]	CCL4	<---	CCR3	-0.46475	0.49595
[14168]	PRKACA	--->	RELA	1.0015	-1.0004
[14169]	PPARD	<---	SRC	-0.55261	0.61826
[14170]	CDK9	--->	MYBL2	0.24948	-0.26781
[14171]	PFKFB2	<---	GCK	-0.152	0.1536
[14172]	NBN	<---	EP300	-0.18644	0.18211
[14173]	HABP2	<---	COL4A4	-0.012629	-0.00035712
[14174]	KAT2B	<---	MECOM	-0.13062	0.12485
[14175]	GADD45B	<---	CDKN1A	-0.0109	0.021707
[14176]	SNTB1	<---	UTRN	-0.006502	-0.0028739
[14177]	IL23R	<---	IL23A	-0.18117	0.21612
[14178]	CPSF6	--->	PLSCR1	0.19478	-0.16005
[14179]	HESX1	<---	NCOR1	-0.079798	0.07335
[14180]	MAP3K11	--->	RHOG	0.36613	-0.36833
[14181]	C3	--->	CFP	0.17093	-0.16487
[14182]	CBLC	<---	EGFR	-0.99978	0.99898
[14183]	TERF1	--->	ZHX1	0.12787	-0.1128
[14184]	TCF4	<---	JUP	-0.08322	0.071428
[14185]	EPOR	<---	INPP5D	-0.027885	0.0084058
[14186]	CAPN1	<---	CBL	-0.082749	0.071081
[14187]	CCIN	--->	ACTA1	0.49407	-0.49201
[14188]	YWHAZ	--->	CDC25C	0.14141	-0.11975
[14189]	PIN1	<---	CSNK2A1	-0.49485	0.46953
[14190]	MAPK3	<---	NTRK3	-0.019271	0.0093651
[14191]	ATN1	--->	EFEMP2	0.039508	-0.041614
[14192]	KCNIP3	--->	CREB1	0.25156	-0.24957
[14193]	RNF4	--->	AR	0.69378	-0.70051
[14194]	HOXA9	?	PBX3	-0.11417	-0.11417
[14195]	SLC9A3R1	--->	ABCC2	0.11782	-0.1184
[14196]	TRAF3	<---	MAP2K1	0.025134	0.055083
[14197]	FCN1	--->	TGFB1	0.069421	-0.11436
[14198]	ETV6	<---	HDAC9	-0.12494	0.13218
[14199]	NR3C2	--->	ACTA1	0.28724	-0.2846
[14200]	INSL3	<---	RXFP2	-0.14912	0.15462
[14201]	PHC2	--->	BMI1	0.23735	-0.19828
[14202]	GPSM3	--->	FGFR3	0.14234	-0.14886
[14203]	RIPK1	<---	NFKB1	-0.010397	0.2425
[14204]	GRB14	--->	FGFR1	0.019469	-0.098619
[14205]	MEIS1	<---	HOXD11	-0.0094151	0.024726
[14206]	PCYT1A	--->	ACTB	0.54914	-0.54501
[14207]	PACSIN2	<--->	PACSIN1	0.019599	0.019599
[14208]	PIK3CG	<---	KRAS	-0.43686	0.43193
[14209]	EDNRB	--->	CAV1	0.45604	-0.43128
[14210]	UBR5	--->	KPNA2	0.15361	-0.12103

[14211] SYK <--- PRKD1 -1.009 1.0005
[14212] APPL1 <--- AKT2 -0.31639 0.46359
[14213] TGM2 ---> RB1 0.48073 -0.4822
[14214] LCK ---> DOK1 0.95892 -0.88457
[14215] MEP1A ---> LAMA1 0.016671 -0.019173
[14216] ACVR1 ---> SMAD1 1.5013 -1.5009
[14217] DLAT <--- PDK1 -0.31137 0.32371
[14218] CIRBP ---> ATXN1 0.028442 -0.032318
[14219] FOXP4 ---> FOXP1 0.023813 0.023442
[14220] TRPC3 ---> TRPC6 -0.0078558 -0.013476
[14221] GFI1B <--- FBLN5 -0.091877 0.084092
[14222] GJB1 ---> CALM1 0.16868 -0.14937
[14223] NR1I2 <--- ACTN2 -0.090564 0.091365
[14224] DLG2 ---> LRP2 0.14113 -0.14994
[14225] PRKCD <--- PIK3CA -0.037311 0.036254
[14226] ASCL2 <--- CALM3 -0.22666 0.2676
[14227] PRKDC <--- PRKCD -0.28455 0.27966
[14228] ZNF496 <--- NSD1 -0.17169 0.17878
[14229] NCOA2 ---> CCND3 0.03342 -0.04366
[14230] CHD9 ---> ESR1 -0.0054507 -0.0084696
[14231] ZNF484 <--- SMAD9 -0.19115 0.17167
[14232] DAB2 ---> ITGB5 0.030477 -0.03265
[14233] MYOD1 <--- NR2F2 -0.27813 0.30604
[14234] UBE3A ---> TP53 0.22276 -0.22705
[14235] DRD3 ---> EEF1G 0.46251 -0.43062
[14236] PTK2 <--- FGR -0.65057 0.81773
[14237] ARL3 ---> UBR1 0.36131 -0.33466
[14238] YES1 ---> PDGFRB 0.049038 -0.058137
[14239] CREBBP ---> MDC1 0.18211 -0.18644
[14240] BCAR1 ---> MMP14 0.037149 -0.040356
[14241] FER ---> CTTN 0.19937 -0.20369
[14242] CREBBP <--- CSNK2A2 -0.32425 0.32272
[14243] SFPQ <--- FHL2 -0.021109 0.054056
[14244] SPTAN1 <--- EPB41 -0.014532 0.0027288
[14245] SFRP2 ---> WNT1 0.99043 -0.98922
[14246] GSK3B ---> CEBPA 0.97322 -0.93471
[14247] GLS2 <--- INADL -0.57593 0.61152
[14248] KAT5 ---> BCL3 0.093303 -0.087282
[14249] HTR1F ---> CAV1 0.45604 -0.43128
[14250] HDAC3 ---> JUN 0.47019 -0.51542
[14251] CSF2RB <--- HCK -1.0003 0.98931
[14252] EEA1 <--- RAB5A -0.3573 0.3878
[14253] HSP90AA1 ---> ESR1 0.037639 -0.046517
[14254] CRLF1 ---> CNTFR -0.024458 -0.024522
[14255] CREBBP ---> BCL3 0.11195 -0.11995
[14256] SMAD1 ---> HOXC8 0.025106 -0.050008
[14257] JAK3 ---> INPP5D 0.39763 -0.38409
[14258] GATA1 ---> ZFPM1 0.054769 -0.032025
[14259] BID ---> BAK1 0.49845 -0.46902
[14260] RACGAP1 <--- NRAS -0.3141 0.32343
[14261] CDC20 <--- AURKA -0.86422 0.83716
[14262] APBA1 ---> APP 0.12974 -0.14219
[14263] NCOR1 ---> RDBP 0.014628 -0.0087221
[14264] DNM1L <--- GSK3B -0.52541 0.50478
[14265] CD4 <--- CXCR4 -0.29998 0.30758
[14266] STAMBP ---> SMAD6 0.99487 -0.99775
[14267] HNF1A <--- BCR -0.1199 0.11712
[14268] MAPK8 ---> MYC 1.0154 -1.0065
[14269] IKZF4 <--- IKZF1 -0.022398 -0.01724
[14270] BNIP3 ? BNIP3L -0.14151 -0.14151
[14271] FHL2 <---> FHL1 0.029854 0.029854
[14272] CFLAR ---> RIPK1 0.29027 -0.19724
[14273] ATM <--- E2F1 -0.14013 0.12852
[14274] SOX8 <--- POU3F3 -0.060464 0.063322
[14275] TRAF2 <--- TNFRSF8 -0.53992 0.5393
[14276] EPOR <--- MST1R -0.24217 0.2257
[14277] UBQLN1 ---> ATXN3 0.076162 -0.042104
[14278] KHDRBS1 <--- RAPSN -0.16823 0.17179
[14279] RHEB ---> RAF1 1.0008 -0.9994
[14280] TNK1 ---> PLCG1 0.29853 -0.40934
[14281] TEAD1 ---> MEF2B 0.033226 0.0030102
[14282] GPS2 <--- NCOR1 -0.066384 0.060182
[14283] CREBBP ---> FOXO1 0.015896 -0.028568
[14284] IGF1R <--- CBL -0.1919 0.1799
[14285] CPNE1 ---> RDX 0.052591 -0.045879
[14286] FZD1 <--- WNT3 -0.15879 0.15445

```
[14287] EGFR    --->    CTNNB1  0.12967 -0.14012
[14288] CRK     --->    BCR     0.068429        -0.062951
[14289] CDC40   <---    SMURF1  -0.72185        0.71781
[14290] SH3GLB1 --->    ADAM9   0.01509 -0.018516
[14291] MARK4   --->    YWHAH   0.047165        -0.061733
[14292] VASP    <---    VCL     -0.38594        0.39629
[14293] PDPK1   --->    RALGDS  0.22578 -0.18071
[14294] AKT1    --->    MAP3K8  1.0168  -1.0071
[14295] PSMD4   <---    UBQLN2  -0.042104       0.076162
[14296] DAB1    --->    LRP8    0.1387  -0.14343
[14297] SASH3   <---    SMURF1  -0.42524        0.41992
[14298] NCOA1   <---    ARNT    -0.031473       -0.010013
[14299] TNFRSF10B       <---    CASP8   -0.38436        0.18641
[14300] TIMP3   --->    MMP3    0.21399 -0.19321
[14301] BCL11B  <---    NR2F1   -0.08573        0.079553
[14302] PCBP1   ?       PCBP2   -0.037462       -0.037462
[14303] ARL4D   --->    SMAD2   0.5744  -0.4943
[14304] JAK2    --->    SIRPA   0.29881 -0.17811
[14305] COL4A5  --->    RNF10   0.0080065       -0.015154
[14306] TRIM3   --->    ACTN4   0.011116        -0.013681
[14307] SCRIB   --->    TJP2    0.012008        -0.024056
[14308] PRKCD   --->    YWHAB   0.77791 -0.76873
[14309] RADIL   --->    SFI1    0.006441        -0.00070275
[14310] NR4A1   <---    RPS6KA5 -0.73191        0.56871
[14311] BGN     <---    COL2A1  -0.0065871      -0.0016489
[14312] IRS4    <---    PTPN11  -0.28963        0.34979
[14313] FGFR1   <---    SLA     -0.14334        0.086985
[14314] MYOT    --->    ACTN1   0.035561        -0.032381
[14315] SOX10   --->    JUN     0.045434        -0.053286
[14316] HSPG2   --->    GRN     0.037865        -0.049722
[14317] PRKCD   --->    ACTA1   0.48294 -0.48348
[14318] CDK2    --->    CDKN1B  0.34497 -0.36687
[14319] PPM1D   --->    NCL     0.25062 -0.24728
[14320] GRIN2D  <---    PRKCG   -0.2311 0.22109
[14321] CASP8   --->    CASP6   1.0138  -1.0003
[14322] SMAD2   <---    BAZ1A   -0.34689        0.38799
[14323] SYNJ2   <---    RAC1    -0.37198        0.40222
[14324] ESR1    --->    IGF1R   0.01032 -0.021681
[14325] PAK2    --->    MKNK1   0.84646 -0.77353
[14326] CDH5    <---    PTPN11  -0.42458        0.53469
[14327] NFKBIA  <---    NKIRAS1 -1.0482 1.0419
[14328] CASP6   --->    PCYT1A  0.58353 -0.69319
[14329] RPS6KB1 --->    EIF4B   1.0004  -1.0123
[14330] SLC9A3R2        --->    ATP2B2  0.34774 -0.35142
[14331] RHOJ    --->    PAK1    0.99704 -1.0053
[14332] DNM1L   <---    SIRT3   -0.25761        0.25905
[14333] SOX2    <---    POU5F1  -0.1696 -0.089966
[14334] PLD1    <---    PKN1    -0.066838       0.050505
[14335] CEBPB   <---    RPS6KA5 -0.54207        0.34196
[14336] RGS5    --->    GNAI2   1.0012  -1.0015
[14337] BLNK    <---    GRB2    -0.44654        0.56823
[14338] CCL25   <---    CCR10   -0.46475        0.49595
[14339] HSP90AA1        --->    TBK1    0.020358        -0.034367
[14340] CAV1    <---    BMX     -0.57896        0.48076
[14341] CUL1    --->    SKP2    0.057263        -0.045528
[14342] NCOR2   --->    ARNT    0.095314        -0.10727
[14343] RGS5    --->    GNAQ    1.0012  -1.0015
[14344] YWHAB   <---    IRS1    -0.049509       0.055105
[14345] APBB1IP <---    RAP1A   -1.0025 0.98956
[14346] MITF    --->    CTNNB1  0.17717 -0.18508
[14347] NCOR2   --->    YWHAE   0.21604 -0.2257
[14348] FYN     --->    GRAP    0.47236 0.00040577
[14349] SRPK1   --->    SFRS3   0.72994 -0.75755
[14350] WRN     <---    PRKAR1B -0.21755        0.23166
[14351] TP53BP2 <---    PPP1CC  -0.13353        0.12061
[14352] SLC2A4  <---    ACTN1   -0.12341        0.12505
[14353] ATXN1   <---    FAM193B -0.069307       0.066199
[14354] SMAD3   <---    TGM2    -0.33148        0.41439
[14355] NPHP1   --->    INVS    0.25068 -0.24815
[14356] AKTIP   --->    GTF3C1  0.22699 -0.19355
[14357] GIPC1   --->    ZNF408  0.1515  -0.11335
[14358] CALM1   <---    IQGAP2  -0.20253        0.22116
[14359] RPS6KA4 <---    MAPK3   -1.0109 1.002
[14360] SOCS3   --->    CSF3R   0.18267 -0.18498
[14361] RIPK2   --->    IKBKG   0.58576 -0.6305
[14362] MYC     <---    RAF1    -0.11913        0.10334
```

[14363]	ID4	?	ID3	-0.65635	-0.65635
[14364]	HCN1	<--->	HCN2	0.047191	0.047191
[14365]	BACE1	--->	KHSRP	0.25343	-0.22109
[14366]	ELP2	--->	STAT5B	0.45399	-0.4714
[14367]	PIAS1	--->	DNM1	0.056889	-0.066705
[14368]	RAB1A	--->	SYT5	0.20092	-0.15731
[14369]	BCL2L1	<---	TP53	-0.062634	0.061271
[14370]	MAZ	<---	DCC	-0.15813	0.14806
[14371]	SMAD6	--->	SMAD1	1.0013	-0.9962
[14372]	SH3GLB2	<--->	SH3GLB1	0.0094254	0.0094254
[14373]	TLE1	--->	HES1	0.26359	-0.26871
[14374]	DLG1	<---	CASK	-0.012439	-0.0091843
[14375]	E2F1	<---	BRCA1	-0.02848	0.031722
[14376]	EIF3F	<---	MTOR	-0.16271	0.16046
[14377]	BMPR1B	--->	UBE2Z	0.017874	-0.043865
[14378]	PABPC1	?	HNRNPD	-0.016298	-0.016298
[14379]	ORAI1	--->	TRPC3	0.13724	-0.14774
[14380]	GRM8	<---	PIAS2	-0.12897	0.14094
[14381]	PRKCG	--->	CYTH2	1.2556	-1.0819
[14382]	PTK6	<---	EGFR	0.18397	0.24432
[14383]	TRIP4	--->	RXRA	-0.01247	-0.19788
[14384]	MXD4	?	MLX	-0.65635	-0.65635
[14385]	NLRP4	<---	CHUK	-0.56455	0.35532
[14386]	CCL19	<---	CCR10	-0.46475	0.49595
[14387]	IKBKG	<---	RALBP1	-0.25547	0.24821
[14388]	NCR3	--->	CD247	0.19126	-0.20612
[14389]	SUV39H1	--->	MYOD1	0.17427	-0.16568
[14390]	RAP1GDS1	<---	RAC1	-0.41126	0.44004
[14391]	NF2	--->	RALGDS	0.068893	-0.078432
[14392]	PIAS1	--->	STAT1	1.0042	-0.98993
[14393]	BNIP2	--->	BCL2	0.77054	-0.76289
[14394]	NGF	--->	A2M	0.26609	-0.23433
[14395]	FGR	--->	HCLS1	0.99631	-1.0146
[14396]	PRKG1	--->	SF1	0.65757	-0.65853
[14397]	RAD54L2	--->	AR	0.57636	-0.58222
[14398]	LMO2	--->	GATA3	0.18194	-0.13822
[14399]	PFKL	<---	PRPF40A	-0.28877	0.32097
[14400]	SEMA7A	--->	PLXNC1	0.41015	-0.37971
[14401]	NCK2	--->	LIMS1	0.24792	-0.25608
[14402]	SLC2A4	<---	KBTBD10	-0.27011	0.29533
[14403]	ADAM15	<---	SH3GL2	-0.02473	0.028123
[14404]	MAPK10	--->	ARRB2	0.24999	-0.33112
[14405]	HIST2H4A	<---	RBBP4	-0.059328	0.088873
[14406]	CDK5	<---	PTPN2	-1.009	1.0061
[14407]	PAK2	--->	PACSIN3	0.18906	-0.026904
[14408]	RFX1	--->	SMAD9	0.098402	-0.11395
[14409]	CNN3	--->	LCP1	0.056374	-0.09335
[14410]	ATXN1	--->	ANKHD1	0.0046756	-0.01423
[14411]	YWHAG	--->	PIK3R1	0.18175	-0.28786
[14412]	KIAA0020	<---	HPS6	-0.0099418	0.043901
[14413]	RALBP1	<---	RALB	-0.99572	1.0059
[14414]	TP53BP1	--->	E2F1	0.064244	-0.053923
[14415]	PTEN	<---	SLC9A3R2	-0.19658	0.22078
[14416]	SMARCA4	--->	RB1	0.060292	-0.072565
[14417]	MIB1	--->	DAPK1	0.0037698	-0.0057789
[14418]	RNF11	<---	PTPRC	-0.37567	0.37802
[14419]	RGS7	--->	GNAZ	0.60077	-0.60607
[14420]	ESR1	<---	NR0B1	-0.044425	-0.0089663
[14421]	NPY2R	<---	NPY	-0.19468	0.20502
[14422]	PKP1	--->	DSG1	0.31989	-0.319
[14423]	INSR	--->	RASA1	0.13656	-0.17723
[14424]	TSHB	<---	TSHR	-0.25953	0.27741
[14425]	RALYL	<---	MCRS1	0.004119	0.027374
[14426]	SMAD7	<--->	SMAD6	0.68552	0.68552
[14427]	UCHL1	--->	CBX1	0.047946	-0.013836
[14428]	ARHGAP17	<---	BTK	-0.45155	0.43652
[14429]	ABCA1	--->	SHANK1	0.0019726	-0.014321
[14430]	REL	<---	CCNE1	-0.093329	0.082031
[14431]	KAT2A	--->	NOTCH1	0.2543	-0.26983
[14432]	SHB	<---	GRB2	-0.18844	0.28685
[14433]	GAS2	--->	ACTA1	0.51886	-0.51601
[14434]	RXRG	<---	CAMK2B	-0.5566	0.41116
[14435]	PRSS23	<---	RACGAP1	-0.074459	0.08037
[14436]	TLR2	--->	TLR1	0.28866	0.066482
[14437]	E2F2	<---	SP1	-0.16629	0.15571
[14438]	STAT3	<---	PDGFRA	-0.076775	0.058866

[14439]	GNA12	<---	S1PR4	-1.008	1.0129
[14440]	AKT1	--->	GSK3A	0.99492	-0.99817
[14441]	RXRB	--->	MAP4	0.025513	0.0014925
[14442]	BCL2	<---	PXN	-0.33203	0.32642
[14443]	CSRP3	--->	MYOD1	0.62562	-0.58677
[14444]	SMAD3	<---	CYP11A1	-0.32966	0.41257
[14445]	TRIP6	--->	PPP1R16A	0.62703	-0.59272
[14446]	CASP2	--->	CCND3	0.36107	-0.36262
[14447]	ACR	--->	ZP2	0.12216	-0.088039
[14448]	STAT3	<---	IFNAR2	-0.25763	0.21302
[14449]	TLR3	<---	CSK	-0.43643	0.53349
[14450]	CASP8	--->	PCYT1A	0.5623	-0.70302
[14451]	PLK1	<---	NINL	-0.32727	0.31052
[14452]	STAM2	--->	USP8	0.062318	0.0087086
[14453]	SMAD3	<---	EBI3	-0.19302	0.23665
[14454]	STAT5B	<---	CD247	-0.24277	0.21556
[14455]	SERPINA1	<---	ELANE	-0.082185	0.11833
[14456]	GIPC1	--->	ITGA5	0.026177	-0.0348
[14457]	MMP17	--->	LRP1	0.076775	-0.087531
[14458]	CHEK1	--->	MDM4	0.091873	-0.11293
[14459]	LCK	--->	SH2D1A	1.2859	-0.84082
[14460]	ATM	<---	AP3B2	-0.22663	0.21655
[14461]	GDF9	<---	PCDHA4	-0.17021	0.20932
[14462]	IGF1	--->	IGFBP4	0.087568	-0.072295
[14463]	PIN1	--->	CDC25C	0.28763	-0.26098
[14464]	TRIM27	--->	MCOLN1	0.025777	-0.021593
[14465]	CFTR	<---	PDZD3	-0.48738	0.48971
[14466]	THRA	<---	YWHAH	-0.092332	0.11204
[14467]	SMAD2	--->	ZEB1	0.10343	-0.10164
[14468]	RPS6KA5	--->	EIF4EBP1	0.35226	-0.56724
[14469]	TNNT1	<---	NINL	-0.31988	0.34869
[14470]	CDH5	<---	KDR	-0.40267	0.43186
[14471]	ACVR1	<---	RAB17	-0.32935	0.31819
[14472]	ATN1	<---	WWP2	-0.039345	0.027081
[14473]	HDAC1	<---	TOP2B	-0.074374	0.089479
[14474]	MYC	<---	CREBBP	-0.081442	0.071232
[14475]	ABL1	<---	YWHAH	-0.08167	-0.068919
[14476]	CASP3	--->	STK24	0.87492	-0.98085
[14477]	NR3C1	?	NR2F2	-0.0089162	-0.0089162
[14478]	KLF6	<---	SP1	-0.07405	0.063416
[14479]	PRKCQ	--->	LYN	0.032722	0.027224
[14480]	TBC1D3F	<---	CDC42	-0.35867	0.38941
[14481]	ADCY5	<---	GNAI1	-0.63771	0.64323
[14482]	MAP4K5	--->	CRKL	0.15333	-0.12435
[14483]	RAD23B	--->	NGLY1	0.074999	-0.040959
[14484]	IL1RL1	--->	TIRAP	0.21173	-0.20583
[14485]	STAT2	--->	STAT6	-0.12425	-0.1265
[14486]	MAP2K1	--->	PRKCZ	0.13614	-0.092025
[14487]	PLAA	<---	HDAC6	-0.044843	0.059463
[14488]	ACVR1	<---	NUAK2	-0.079957	0.10767
[14489]	GGA2	--->	SORL1	0.069724	-0.076758
[14490]	CSNK2A1	--->	CREM	0.96654	-0.95401
[14491]	CEBPD	<---	CREBBP	-0.18961	0.18576
[14492]	FUBP1	--->	CCNH	0.36516	-0.33153
[14493]	PPM1D	<---	MDM2	-0.016494	0.013841
[14494]	GRN	--->	HOXA1	0.11608	-0.084041
[14495]	NRAS	--->	PLCE1	0.99635	-0.99834
[14496]	F7	--->	F3	0.26572	-0.27477
[14497]	SMAD7	<---	ZBTB44	-0.025332	0.046047
[14498]	BMPR1B	<---	PAK1	-0.6575	0.64535
[14499]	RUNX2	--->	HDAC6	0.010966	0.0045162
[14500]	TP53	<---	BRCA1	-0.22722	0.22885
[14501]	BTK	--->	WAS	0.4868	-0.53132
[14502]	MAPK9	--->	SF3B4	0.72994	-0.75755
[14503]	PDGFB	--->	LRP1	0.088334	-0.099358
[14504]	NFKB1	--->	RELA	0.18462	-0.013559
[14505]	KPNA2	--->	RELA	0.097611	-0.092255
[14506]	HSPB6	?	HSPB8	-0.096038	-0.096038
[14507]	RGS19	--->	OPRL1	0.059274	-0.049072
[14508]	REPS1	?	REPS2	-0.0020812	-0.0020812
[14509]	SRC	--->	CTTN	0.75064	-0.61051
[14510]	YWHAG	<---	HSPA8	-0.739	0.77111
[14511]	JAK1	--->	IL3RA	0.54644	-0.55778
[14512]	PIAS1	--->	JUN	0.23842	-0.25931
[14513]	ATF4	<---	DAPK3	-1.0029	0.99807
[14514]	TBP	<---	HTT	-0.011546	-0.00070351

[14515]	MAPK1	--->	PLAT	0.30477	-0.32323
[14516]	HTR2C	--->	ARRB2	0.12815	-0.12587
[14517]	JUN	--->	FOS	0.17898	-0.31886
[14518]	PTGDR	--->	GNAS	1.0129	-1.008
[14519]	RPS6KA1	--->	BAD	0.58958	-0.91274
[14520]	MYO18B	--->	ACTB	0.53391	-0.53116
[14521]	NLRP12	--->	PYCARD	0.18479	-0.14874
[14522]	ABL1	<---	YWHAE	-0.08167	-0.068919
[14523]	GNA13	<---	DRD5	-1.008	1.0129
[14524]	CEBPE	<---	MYB	-0.014408	0.0091118
[14525]	RNF11	--->	ZFYVE16	0.021351	-0.018205
[14526]	ATN1	--->	GIGYF1	0.03757	-0.039831
[14527]	BAG1	--->	RB1	0.45098	-0.45395
[14528]	RET	--->	MAPK1	0.20607	-0.24517
[14529]	CUL4A	--->	CDKN1B	0.059379	-0.063622
[14530]	MAPK9	--->	JUND	1.0022	-1.0148
[14531]	WNT2	?	WNT3A	-0.58807	-0.58807
[14532]	CAV1	<---	MAPK3	-0.73589	0.70376
[14533]	PIN1	<---	NFATC2	-0.14931	0.14342
[14534]	NR1H2	--->	RXRA	1.003	-0.99549
[14535]	YWHAZ	<---	GP5	-0.0031405	0.017142
[14536]	PTPN4	--->	EEF1G	0.28532	-0.29386
[14537]	RPS6KA5	--->	HSPB2	0.36611	-0.56582
[14538]	FBXO17	<---	SKP1	-0.044878	0.055251
[14539]	SIRT1	--->	HEY2	0.12648	-0.13477
[14540]	NR1H4	<---	GAPDH	-0.031277	0.041417
[14541]	SDCBP	<---	PTPRJ	-0.44544	0.4442
[14542]	PTK2	<---	SOCS2	-0.21901	0.019531
[14543]	CD2AP	<---	SRC	-0.31855	0.71828
[14544]	ITCH	--->	OCLN	0.26589	-0.27572
[14545]	NEUROD6	<---	HMGB1	-0.81406	0.8544
[14546]	SMAD1	<---	ICK	-1.3294	1.2525
[14547]	OGDH	<---	DLST	-0.085502	0.11965
[14548]	SOCS1	<---	IGF1R	-0.04286	0.019972
[14549]	KIF15	--->	MKI67	0.034555	-0.00056863
[14550]	PTPN11	--->	IL4R	0.48562	-0.37565
[14551]	DHX9	--->	JUN	0.27835	-0.31119
[14552]	SYK	<---	FCGR2A	-1.0112	1.0048
[14553]	CREBBP	--->	AR	0.99704	-0.99484
[14554]	STMN1	<---	HSPA5	-0.93289	0.98516
[14555]	ABL1	--->	APP	0.02088	-0.038724
[14556]	PECAM1	<---	LYN	0.015127	0.11748
[14557]	CUBN	<---	TF	-0.020897	0.025384
[14558]	TP73	--->	SMAD3	0.12401	-0.097283
[14559]	RAN	--->	SMAD2	0.55322	-0.47159
[14560]	ITGA3	<---	TIMP2	-0.012564	0.0022617
[14561]	ITGB3	<---	PECAM1	-0.14014	0.1367
[14562]	PFKL	<---	COPS6	-0.21994	0.24624
[14563]	PIK3C2B	<---	EGFR	-0.018116	0.0079317
[14564]	MIB1	<---	SNX5	-0.0059012	-0.0036989
[14565]	GAB3	--->	PIK3R1	0.035855	-0.075247
[14566]	PI4KB	<---	YWHAB	-0.068047	0.092991
[14567]	PEA15	--->	PLD1	0.45079	-0.45937
[14568]	UBQLN1	--->	HES1	0.29031	-0.29527
[14569]	CAMK1	--->	HDAC5	0.4123	-0.45761
[14570]	IFNG	<---	TNF	-0.28524	0.31183
[14571]	ITSN1	<---	EPHB2	-0.080837	0.06826
[14572]	SMARCA4	--->	TP53	0.045703	-0.056245
[14573]	SDCBP	--->	GRIK2	0.12457	-0.11938
[14574]	ITGA1	<---	COL13A1	-0.014328	0.00060242
[14575]	TRAF2	--->	CHUK	0.024674	-0.077891
[14576]	GRIA1	<---	HSPA5	-0.4466	0.44959
[14577]	CD247	<---	PTPRC	-0.35868	0.3377
[14578]	PDZD3	--->	SLC22A4	0.37001	-0.34102
[14579]	PARD3	--->	SMAD7	0.14536	-0.12329
[14580]	SKIL	<---	SMAD1	-0.18376	0.17319
[14581]	TFPI2	<---	F11	0.0096062	0.024385
[14582]	CRELD1	--->	SETDB1	0.24559	-0.22984
[14583]	CUL5	--->	RBX1	0.12225	-0.11034
[14584]	PIK3R3	--->	INSR	0.013083	-0.039462
[14585]	SYK	--->	EPOR	0.42519	-0.42636
[14586]	STAT2	<---	JAK2	-0.89499	0.90272
[14587]	STAM2	--->	UBQLN4	0.061166	0.0094847
[14588]	FGFR1	<---	FGF3	-0.99871	1.0053
[14589]	PTPN11	<---	CBL	-0.39261	0.40648
[14590]	SHC1	<---	IRS1	-0.17662	0.088295

[14591]	TRIM27	--->	CHD4	0.00063094	-0.013034
[14592]	C5AR1	--->	ARRB2	0.27159	-0.25831
[14593]	BCL10	<---	PELI2	0.011012	0.01976
[14594]	COL4A2	--->	APP	0.044125	-0.057522
[14595]	SERPINE2	<---	COL4A2	-0.055972	0.049204
[14596]	MAPK14	--->	CREB1	0.56317	-0.56034
[14597]	SFRS3	<---	CHD1	-0.0049598	0.0011884
[14598]	PRPF40A	--->	SF1	0.25659	-0.24238
[14599]	GHR	<---	GRB2	-0.10805	0.094648
[14600]	TGFBR2	--->	TRAP1	0.87761	-0.81719
[14601]	NCOA3	<---	RXRA	-0.084089	0.060364
[14602]	FMOD	--->	TGFB1	0.61673	-0.62998
[14603]	GIT2	<---	ARHGEF7	-0.058731	0.046581
[14604]	BGN	--->	APOB	0.1253	-0.096261
[14605]	COL9A1	--->	COMP	0.039096	-0.044946
[14606]	ICAM5	--->	ITGB2	0.046792	-0.059499
[14607]	IL16	--->	CD4	0.048758	-0.037718
[14608]	YWHAZ	<---	EGFR	-0.28735	0.28431
[14609]	GSK3B	<---	NFKB1	-0.6564	0.6581
[14610]	EPHB3	--->	CRK	0.17048	-0.24745
[14611]	NR2E3	--->	RBBP7	0.034681	-0.0096005
[14612]	KLHL12	<---	LNX1	-0.036151	0.052728
[14613]	NF2	<---	SLC9A3R1	-0.056659	0.061503
[14614]	TCERG1	<---	HSPA8	-0.31176	0.33101
[14615]	ABL1	<---	CAV1	-0.085324	-0.080621
[14616]	STAT5A	<---	NCOR2	-0.077181	0.063117
[14617]	PTGIR	--->	ADCY10	0.62397	-0.59295
[14618]	KLF12	--->	EHMT2	0.029925	-0.041087
[14619]	STARD13	--->	ITPR1	0.19595	-0.20742
[14620]	S1PR2	--->	GNA13	1.0129	-1.008
[14621]	DUSP16	--->	MAPK9	1.0016	-1.0004
[14622]	SUFU	<---	GLI1	-0.088736	0.082486
[14623]	CD19	--->	PLCG2	0.1287	-0.23333
[14624]	MAP3K7IP3	<---	TRAF6	-1.0134	0.99787
[14625]	TRPC1	<---	CAV1	-0.15495	0.14405
[14626]	LYN	--->	CD79A	1.0799	-1.0399
[14627]	DDX27	--->	PPP1R16A	0.36337	-0.34862
[14628]	HIC1	<---	HDAC1	-0.12872	0.1203
[14629]	SIRT2	--->	HDAC6	0.048177	-0.032799
[14630]	PIAS3	<---	OPN1LW	-0.039903	0.050065
[14631]	MAG	--->	MAP1B	0.19941	-0.20537
[14632]	AFP	<---	SGSM2	-0.053053	0.056543
[14633]	BCAR1	<---	LYN	-1.0003	1.0002
[14634]	GP6	<---	LYN	0.015127	0.11748
[14635]	KBTBD7	--->	MAP1LC3B	0.079574	-0.04927
[14636]	ACVR1B	--->	SMAD2	1.0115	-1.0087
[14637]	SHE	--->	ABL1	-0.18596	-0.32072
[14638]	TRAF4	--->	SORBS2	0.044259	-0.047654
[14639]	PTPN11	--->	CEACAM1	0.31282	-0.27672
[14640]	CBX5	<--->	CBX3	0.0018385	0.0018385
[14641]	PIN1	<---	PTPN1	-0.96315	0.96784
[14642]	ADAM12	--->	IGFBP3	0.073812	-0.058279
[14643]	LCK	--->	IL2RB	0.99939	-1.0044
[14644]	TWIST1	<---	MYOD1	-0.24728	-0.24445
[14645]	CSNK2A2	--->	WAS	0.18552	-0.50928
[14646]	WIPF1	--->	ACTC1	0.080345	-0.090566
[14647]	OSMR	--->	IL6ST	0.033591	-0.12732
[14648]	RBBP7	<---	SUV39H1	-0.0028102	0.0096869
[14649]	USP7	<---	RNF220	-0.046576	0.084465
[14650]	NCOR2	--->	TBL1X	0.045382	-0.058192
[14651]	PPIE	<---	MLL	-0.059229	0.06362
[14652]	SOCS1	--->	IRS1	0.11723	-0.050782
[14653]	DAB1	<---	CRKL	-0.13738	0.12903
[14654]	DNMBP	--->	DNM1	0.013624	-0.030517
[14655]	CABLES2	<---	CDK5	-1.1093	1.083
[14656]	NFKB1	--->	REL	0.01855	-0.0095479
[14657]	ARIH1	--->	PLD1	0.16164	-0.17367
[14658]	SYNJ2BP	--->	LRP2	0.41365	-0.41429
[14659]	PRKACA	--->	HDAC8	0.4	-0.42066
[14660]	PDGFA	<---	HSPG2	-0.017166	0.0046555
[14661]	CCNG1	<---	CDKN2A	-0.10371	0.10975
[14662]	ZBTB7A	<---	SP1	-0.096361	0.038159
[14663]	BTK	<---	CBL	-0.23287	0.21359
[14664]	TLN1	<---	ACTA1	-0.103	0.091758
[14665]	MAPK9	--->	JDP2	0.97462	-1.029
[14666]	CHD1	<---	HDAC1	-0.0086534	0.0013206

[14667]	SPI1	<---	SSRP1	-0.2185	0.23807
[14668]	CUL9	<---	UBE2G2	-0.11067	0.14316
[14669]	SEC63	--->	SEC62	0.072754	-0.030532
[14670]	TNFRSF10A	<---	COL2A1	0.0042647	0.0085499
[14671]	HOXB9	<---	EP300	-0.18943	0.18505
[14672]	BRSK1	<---	WEE1	0.13714	0.14515
[14673]	CBX4	--->	RB1	0.47962	-0.48109
[14674]	MAPK3	<---	LCK	-0.99895	0.99734
[14675]	MAP3K7IP3	<---	TRAF2	-0.17777	0.18227
[14676]	CD84	--->	SH2D1B	0.077004	-0.051869
[14677]	TGFA	--->	ERBB4	0.98479	-0.99014
[14678]	MAPK14	--->	ELK1	1.0099	-1.0011
[14679]	FBXO5	--->	FZR1	0.084765	-0.070176
[14680]	PRKCE	--->	GJA1	0.17785	-0.201
[14681]	NPHP1	--->	FLNC	0.18956	-0.17815
[14682]	APBA3	<---	BCR	-0.0361	0.026617
[14683]	SH2D1A	<---	GRB2	-0.30614	0.47743
[14684]	CCNB2	<---	TGFBR2	-0.73456	0.7651
[14685]	MTA2	--->	TP53	0.31636	-0.30191
[14686]	NEK9	<--->	NEK7	0.23429	0.23429
[14687]	GTF2H2	<---	ERCC3	0.0080458	0.025965
[14688]	ASB6	<---	SH2B2	-0.11568	-0.0059636
[14689]	CASP3	--->	TRAF1	0.72974	-0.75525
[14690]	EP300	--->	ZFPM2	0.10965	-0.1177
[14691]	CBL	--->	LAT	0.48555	-0.49057
[14692]	RAB5A	<---	RASA1	-1.0119	1.0001
[14693]	CCND3	<---	AREG	-0.18507	0.18099
[14694]	PRPF4B	--->	YWHAG	0.62776	-0.66368
[14695]	FMNL2	<---	PRPF40A	-0.05499	0.047368
[14696]	PPP1CA	--->	SKP1	0.096177	-0.10699
[14697]	PPP4C	--->	PPP2R1A	0.063232	-0.075162
[14698]	CSNK2A1	--->	GTF2A1	0.69606	-0.7619
[14699]	ARL4D	--->	PGAM1	0.64301	-0.61706
[14700]	JAK1	--->	PLA2G4A	0.32169	-0.33349
[14701]	KCNA4	<---	SNTA1	-0.013537	0.050181
[14702]	PRKACA	--->	GMFB	0.75717	-0.78322
[14703]	ATXN1	<---	MYO18A	-0.013608	0.0021544
[14704]	IRAK2	--->	SMAD2	0.66542	-0.57144
[14705]	RBX1	<---	FBXO18	-0.090119	0.12388
[14706]	RXRA	<---	IGFBP3	-0.23195	0.021852
[14707]	LAMA5	<---	BCAM	-0.034373	0.025055
[14708]	CD3E	<---	TOP2B	-0.34784	0.3711
[14709]	MAPK6	<---	PRKCB	0.47633	0.49475
[14710]	KLRK1	<---	RAET1E	-0.0029242	0.054213
[14711]	SH3PXD2A	<---	SRC	-0.32216	0.68095
[14712]	JAK1	<---	IFNGR1	-0.031422	0.021249
[14713]	CD79B	<---	PTPN6	-1.0144	1.0068
[14714]	GNAS	--->	ADCY2	0.30417	-0.29639
[14715]	LEP	--->	HK3	0.076808	-0.062201
[14716]	JMY	<---	EP300	-0.047229	0.035426
[14717]	PPP2R1A	--->	STRN3	0.088301	-0.094979
[14718]	TLR2	--->	RIPK2	0.13535	-0.14978
[14719]	MEGF10	<---	ITSN2	-0.0071422	-0.0068233
[14720]	KRT2	--->	SMAD3	0.42076	-0.33768
[14721]	TBP	<---	CXXC1	-0.012951	0.00070615
[14722]	KLHL26	<---	UBQLN4	-0.10822	0.14113
[14723]	THPO	--->	MPL	0.033493	-0.021555
[14724]	COL8A1	--->	EFEMP2	0.052018	-0.054407
[14725]	HOXA1	<---	EFEMP2	-0.067922	0.101
[14726]	SRC	--->	FRS2	1.2026	-1.146
[14727]	CD2	<---	CD59	-0.065869	0.07232
[14728]	DLG2	<---	SCN5A	-0.034928	0.041469
[14729]	ACIN1	--->	KLHL26	0.17821	-0.1378
[14730]	FYN	<---	CASP8	-0.71326	0.68828
[14731]	KLK2	--->	A2M	0.14985	-0.11653
[14732]	CCNH	<---	MTA1	-0.22549	0.26747
[14733]	CCR8	--->	CCL1	0.49595	-0.46475
[14734]	TGFA	--->	SNTA1	0.095088	-0.058731
[14735]	RNF5	--->	PXN	0.20001	-0.20077
[14736]	CREBBP	--->	SRF	0.10587	-0.1138
[14737]	DDR1	--->	PLCG1	0.4969	-0.67053
[14738]	MPP1	<---	SGSM2	-0.02687	0.020554
[14739]	MMP14	--->	LRP1	0.076775	-0.087531
[14740]	LCP2	--->	BLNK	0.054968	0.0073472
[14741]	BGN	--->	APP	0.22065	-0.23176
[14742]	DHX30	--->	RBBP4	0.039499	-0.033308

[14743]	HDAC1	<---	NCOR2	-0.1409	0.13009
[14744]	EFEMP1	<---	TIMP3	0.0018781	0.033109
[14745]	GRB10	--->	GHR	0.025719	-0.067227
[14746]	H3F3B	--->	EHMT2	0.11647	-0.11384
[14747]	RAB2B	--->	SMAD1	0.52759	-0.54836
[14748]	RAB11A	--->	PSEN2	0.37382	-0.34322
[14749]	PFKFB2	<---	YWHAQ	-0.29134	0.29699
[14750]	F2	<---	F2R	-0.27153	0.28486
[14751]	MAPK12	--->	SNTA1	0.36823	-0.49063
[14752]	CDH11	<---	CTNNB1	-0.32695	0.32855
[14753]	FYN	--->	FLOT2	0.66869	-0.57642
[14754]	MAPK3	--->	DCC	0.088336	-0.11794
[14755]	NCOA1	<---	NR5A1	-0.043163	0.034596
[14756]	PRKG1	--->	CREB1	0.54384	-0.54419
[14757]	NUMB	--->	ITGB2	0.12706	-0.13795
[14758]	LEPR	<---	JAK2	-1.0017	1.0162
[14759]	PIWIL1	--->	DICER1	0.066947	-0.063843
[14760]	DUSP9	--->	MAPK3	1.0016	-1.0004
[14761]	PDGFRB	--->	EGFR	0.005423	-0.0073134
[14762]	EPC1	--->	E2F6	0.18227	-0.15164
[14763]	CEACAM1	--->	FLNA	0.048324	-0.046149
[14764]	SMARCE1	--->	ESR1	0.30074	-0.28692
[14765]	ITPR1	<---	CCNB1	-0.20554	0.19403
[14766]	ABL1	--->	TERT	-0.01369	-0.018679
[14767]	RALY	--->	WDR62	0.076139	-0.063134
[14768]	MAP2K1	--->	PARVA	0.53816	-0.15162
[14769]	HIF1A	--->	MAX	0.85119	-0.84886
[14770]	CDK2	--->	PRKAR1A	0.27068	-0.33631
[14771]	PRKDC	--->	HOXC4	0.033341	-0.029874
[14772]	MAPK3	--->	PPARA	0.99834	-1.0079
[14773]	RALGDS	<---	RAP1A	-1.0084	0.98547
[14774]	SMAD9	<---	RRBP1	-0.31807	0.2994
[14775]	PKN1	--->	MAPT	-0.005192	-0.067776
[14776]	CCND1	<---	STAT3	-0.21474	0.22363
[14777]	RALGAPA1	--->	TCF3	0.23437	-0.24125
[14778]	GRIA2	<---	SDCBP	-0.11938	0.12457
[14779]	MAPK1	--->	CAD	0.43416	-0.45528
[14780]	MAP3K9	--->	SH3RF1	0.16486	-0.047173
[14781]	MTOR	--->	STAT3	0.018483	-0.031022
[14782]	MOS	--->	MYOD1	1.5563	-1.5271
[14783]	SRC	--->	WWOX	0.55602	-0.49297
[14784]	FBN1	<---	DCN	-0.048442	0.042123
[14785]	CAMK2G	<---	MYLK	0.12121	0.16206
[14786]	LIFR	--->	CNTFR	-0.035679	-0.036024
[14787]	ITGA5	<---	EGFR	-0.14759	0.12886
[14788]	TSHR	--->	GNAO1	0.65587	-0.65379
[14789]	YWHAQ	--->	SMAD9	0.33354	-0.35825
[14790]	SP100	--->	CBX3	0.20119	-0.16798
[14791]	PTPN12	--->	PSTPIP2	0.55034	-0.55247
[14792]	RECQL5	--->	EEF1G	0.014169	0.0017615
[14793]	CREBBP	--->	HNF1A	0.18505	-0.18943
[14794]	PPP1CB	--->	PTK2	0.044129	-0.079901
[14795]	GATA6	<---	EP300	-0.19036	0.18629
[14796]	CASP8	--->	CHUK	0.65864	-0.66258
[14797]	PDLIM7	<---	PRKCA	-0.57553	0.57562
[14798]	RPS6KB1	<---	PPP2CA	-0.99976	0.99478
[14799]	BTK	--->	TNFRSF10A	0.40657	-0.4142
[14800]	LAMC2	--->	BMP1	0.025488	-0.021277
[14801]	CASP3	--->	HMGB1	0.48503	-0.59651
[14802]	PAPPA	<---	C3	-0.104	0.1116
[14803]	ARL4D	--->	EML4	0.59393	-0.56872
[14804]	IRS2	--->	PIK3CD	0.20663	-0.21631
[14805]	RBL2	<---	CCND3	-0.28031	0.26997
[14806]	SP1	--->	REL	0.032741	-0.045828
[14807]	SNX6	<---	BMPR1B	-0.1372	0.11168
[14808]	HOXB9	<---	RBPMS	-0.071742	0.10141
[14809]	ABR	<---	ADRA1A	-0.098424	0.089362
[14810]	MTOR	--->	RPTOR	-0.0030289	-0.010689
[14811]	IRS4	<---	GRB2	-0.19563	0.18694
[14812]	AURKA	<--->	GSK3B	0.23429	0.23429
[14813]	HTRA1	--->	CDYL	0.0080582	-0.0033669
[14814]	FCGR3A	<---	LCK	-0.44297	0.51622
[14815]	AXIN1	<---	RNF111	-0.15945	0.15179
[14816]	SCN2A	--->	ANK3	0.026829	-0.040665
[14817]	THBS1	<---	MMP9	-0.15886	0.21165
[14818]	FRS2	<---	NTRK1	-0.12806	0.12396

14821 [14819] NR2C2 <--- MAP3K7IP1 -0.35914 0.38623
14822 [14820] FADD <--- FASLG -0.4629 0.30026
14823 [14821] DAAM1 <--- DVL1 -0.053465 0.040131
14824 [14822] CSF2RB <--- SYK -0.81996 0.75159
14825 [14823] YWHAG <--- CAMKK1 -0.68484 0.78748
14826 [14824] CNKSR1 ---> RAF1 0.05881 -0.082858
14827 [14825] RSU1 ---> RAF1 0.0086899 -0.022887
14828 [14826] LAT <--- GRB2 -0.11406 0.15259
14829 [14827] YWHAG <--- PRKCG -0.90439 0.90517
14830 [14828] EDNRB <--- GRK6 -0.40764 0.31971
14831 [14829] DHH <--- HHIP -0.99342 0.98842
14832 [14830] RNF11 <--- WWP1 -0.26209 0.25179
14833 [14831] RNF11 ---> MYO6 0.11529 -0.10101
14834 [14832] HIPK2 ---> MYB 0.092606 -0.10814
14835 [14833] PRKACA ---> STMN2 1.0058 -0.99021
14836 [14834] KRT15 <--- RHPN1 -0.0046265 0.0095574
14837 [14835] MLL ---> MYST1 0.044329 -0.039661
14838 [14836] ZNF337 <--- CCDC85B -0.22906 0.22518
14839 [14837] HDAC3 ---> ARID4A 0.049112 -0.033325
14840 [14838] FLT1 ---> NCK1 0.14973 -0.1387
14841 [14839] PRKCD ---> BDKRB2 0.45098 -0.45062
14842 [14840] CCND1 ---> CTNNB1 0.29555 -0.30038
14843 [14841] STRN <--- GNAI1 -0.29746 0.31173
14844 [14842] CTSE ---> EDN3 0.38451 -0.37457
14845 [14843] MET ---> GRB2 0.1807 -0.36229
14846 [14844] CREBBP ---> ZNF639 0.1109 -0.11893
14847 [14845] BAG4 <--- TNFRSF25 -0.61534 0.53344
14848 [14846] PTPN6 ---> STAT6 0.52344 -0.62738
14849 [14847] NR4A1 <--- RPS6KA1 -0.73191 0.56871
14850 [14848] PTK2B <--- CRK -0.21492 -0.04842
14851 [14849] E4F1 <--- EHMT2 -0.01749 0.0057555
14852 [14850] RARB ---> NCOA1 0.034596 -0.043163
14853 [14851] XCL1 ---> VCAN 0.010191 -0.021003
14854 [14852] SCN5A <--- DLG1 -0.049447 0.047736
14855 [14853] TRPC3 <--- SLC8A1 -0.095093 0.083123
14856 [14854] U2AF2 <--- SRPK2 -0.75755 0.72994
14857 [14855] GP1BA ---> ITGAM 0.012212 -0.025598
14858 [14856] HOXA5 <--- FOXO1 -0.10716 0.098681
14859 [14857] TRAF5 <--- TNFRSF4 -0.1819 0.17914
14860 [14858] COL8A1 ---> UBQLN4 0.035083 -0.037687
14861 [14859] SFRS2 <--- ZRSR2 -0.030095 0.061943
14862 [14860] TRIP12 ---> CELSR3 0.033032 -0.040396
14863 [14861] CCNB2 <--- CDK2 -1.0774 1.0544
14864 [14862] ESR1 ? ESRRA -0.0089162 -0.0089162
14865 [14863] UBQLN1 ---> DNAJB2 0.14708 -0.15637
14866 [14864] JUN <--- NR3C1 -0.27062 0.24409
14867 [14865] TRPC4 ---> ITPR3 -0.0070644 -0.013012
14868 [14866] NCAN <--- L1CAM -0.021081 0.0079271
14869 [14867] PHF21A <--- HDAC2 -0.020171 0.035853
14870 [14868] CXCR1 ---> CXCL3 0.49595 -0.46475
14871 [14869] ZBTB48 ---> DVL3 0.10199 -0.11158
14872 [14870] BCL6 <--- MAPK1 -1.0089 1.0108
14873 [14871] RGS7 ---> YWHAQ 0.22227 -0.21545
14874 [14872] HDAC5 ---> BCOR 0.19084 -0.1926
14875 [14873] SRC ---> GNB2L1 1.0299 -0.91681
14876 [14874] CASP9 <--- CAPN2 -0.15804 0.15328
14877 [14875] CNKSR2 ---> DLG1 -0.0026013 -0.020819
14878 [14876] RORA <--- COPS5 -0.19181 0.21292
14879 [14877] MARK4 ---> PRKCI 0.0031581 -0.0051882
14880 [14878] NFIC ? NFIB -0.1934 -0.1934
14881 [14879] TJAP1 <--- DLG1 -0.041151 0.041422
14882 [14880] CXCL11 <--- CCR3 -0.46475 0.49595
14883 [14881] CDH5 <--- PTPRB -0.55724 0.55574
14884 [14882] NEDD9 <--- PTPN11 -0.13445 0.1641
14885 [14883] DLG4 <--- PRKCA -0.40275 0.39335
14886 [14884] TESK2 ---> YWHAB 0.65023 -0.66983
14887 [14885] BMPR2 ---> CRYAB 0.40505 -0.39586
14888 [14886] ESRRG ---> DAXX 0.043094 -0.059061
14889 [14887] CSNK2A1 ---> APEX1 0.70304 -0.7292
14890 [14888] TNFSF4 <--- TRAF2 -0.051462 0.058058
14891 [14889] ACTN2 ---> EPS8L1 0.014484 -0.043625
14892 [14890] ASAP2 <--- PACSIN3 -0.0090737 0.034952
14893 [14891] AR <--- UBE3A -0.36841 0.35941
14894 [14892] TNFRSF11A <--- TNFSF11 -1.0769 0.99996
14895 [14893] SETDB1 <--- PGAM5 -0.068842 0.073397
14896 [14894] HDAC3 ---> RBBP4 0.076396 -0.057276

[14895]	PDZD11	--->	ATP2B2	0.34774	-0.35142
[14896]	BPGM	<---	GAPDH	-0.10417	0.11905
[14897]	ABI1	<---	SPTA1	0.012444	0.035235
[14898]	ZBTB17	<---	ITGB5	-0.059245	0.045749
[14899]	SPAG9	<---	NFKB1	-0.0084134	0.17325
[14900]	AURKA	--->	MBD3	0.91183	-0.93004
[14901]	AKAP11	<---	PPP1CC	-0.15632	0.14694
[14902]	RBX1	<---	SKP1	-0.10969	0.12024
[14903]	PIK3AP1	--->	PIK3R1	0.0028834	-0.049744
[14904]	RIPK2	--->	NGFR	0.10391	-0.13698
[14905]	PIAS3	--->	RELA	0.061658	-0.066672
[14906]	CHD6	<---	ZFYVE9	-0.0072111	-0.0067234
[14907]	PPP2CB	--->	MID1	0.094726	-0.10622
[14908]	STAT5A	<---	PPP2CA	-0.1082	0.095005
[14909]	ITGAE	<---	CDH1	-0.029675	0.016384
[14910]	DDX18	--->	MTA1	0.09341	-0.072955
[14911]	NR4A1	--->	BCL2A1	0.52125	-0.50885
[14912]	STAM2	<---	JAK2	-0.99545	0.98976
[14913]	ABL1	<---	MAP4K1	-0.046372	0.017907
[14914]	EDNRB	--->	EDN1	0.45294	-0.42199
[14915]	GHR	<---	GH2	-0.0256	0.021287
[14916]	TSC2	<---	YWHAQ	-0.29138	0.294
[14917]	LCK	--->	CTLA4	0.99954	-1.0121
[14918]	KRT15	<---	RCOR3	-0.02322	0.027094
[14919]	EPHB6	--->	GRB2	0.12359	-0.18954
[14920]	DUSP7	--->	MAPK14	0.99169	-0.98663
[14921]	SLC22A12	<---	PDZK1	0.014565	0.022051
[14922]	CTSD	--->	CCL21	0.4106	-0.40051
[14923]	RNF5	<---	TGFBR1	-0.20601	0.17962
[14924]	GLS2	<---	DLG1	-0.26501	0.26144
[14925]	ARHGEF2	--->	CGN	0.20159	-0.19101
[14926]	FBXW7	--->	HIPK2	-0.0095111	-0.010638
[14927]	TAL1	<---	LMO1	-0.3637	0.38177
[14928]	MMP13	<---	TIMP3	-0.19321	0.21399
[14929]	THAP11	<---	HDAC3	-0.028297	0.020885
[14930]	COL4A1	--->	COL4A2	-0.0044774	-0.0066169
[14931]	ZHX2	<---	ZHX3	-0.090184	-0.053398
[14932]	MAPK8IP2	--->	TIAM1	0.27495	-0.28283
[14933]	PARK2	--->	GRIN2B	0.13477	-0.13596
[14934]	RHPN2	<---	RHOA	-0.41099	0.4427
[14935]	LAT	--->	VAV1	0.041933	-0.2286
[14936]	FYN	--->	SH3BP2	1.0676	-0.85236
[14937]	PLAT	--->	LRP1	0.049858	-0.062045
[14938]	FBXW4	<---	CUL1	-0.036054	0.036998
[14939]	FGFR2	<---	FGF1	-0.53789	0.54173
[14940]	ZSCAN4	--->	SMAD9	-0.0031961	-0.015716
[14941]	SPRY2	<---	SPRYD5	-0.044583	0.060024
[14942]	SERPINE1	<---	UBQLN4	-0.14362	0.17983
[14943]	TAF15	<---	TAF1	-0.0038354	5.1693e-005
[14944]	RELA	--->	STAT6	0.049369	-0.064195
[14945]	L3MBTL	--->	CBX3	0.11193	-0.094052
[14946]	COPS6	--->	C1orf174	0.203	-0.17586
[14947]	PAK2	<---	GRB2	-0.0073877	0.11327
[14948]	PRKACA	--->	PFKFB2	0.6735	-0.69812
[14949]	GRB14	<---	MAP3K14	-0.34335	0.17357
[14950]	KCNMA1	<---	PTK2	-0.13188	0.026448
[14951]	ABL1	<---	YWHAQ	-0.08167	-0.068919
[14952]	ARR3	<---	CAPNS1	-0.066977	0.059819
[14953]	DGKZ	<---	DMD	-0.024722	0.015095
[14954]	GZMB	--->	BID	0.087842	-0.041342
[14955]	ZBTB25	<---	MAPK9	-0.56785	0.54323
[14956]	GAB3	<---	SRC	-0.56616	0.62131
[14957]	BLNK	--->	CD79A	0.18339	-0.31363
[14958]	NFATC3	<---	TTF1	-0.26621	0.27767
[14959]	HAND1	--->	TCF3	-0.13552	-0.39218
[14960]	ITPR1	<---	LYN	-1.0037	0.99821
[14961]	PIN1	<---	RAB4A	-0.040959	0.069256
[14962]	SVIL	--->	TGFB1I1	0.14076	-0.13521
[14963]	SF3B4	<---	TCERG1	-0.17953	0.19241
[14964]	ETS2	<---	GATA3	-0.14597	0.17161
[14965]	ATXN1	<---	C1orf94	-0.019912	0.016709
[14966]	EP300	--->	SREBF2	0.045169	-0.056808
[14967]	GADD45G	--->	RXRA	0.0082071	-0.21826
[14968]	CAMKK1	--->	CAMK1	0.83082	-0.43719
[14969]	FLNB	<---	SMURF2	-0.71362	0.70977
[14970]	SYTL4	<---	RAB27A	-0.15731	0.20092

[14971]	MPHOSPH8	<---	RANBP9	-0.055276	0.053372
[14972]	RHOXF2	--->	CCNK	0.1684	-0.15467
[14973]	ENAH	<---	BAIAP2	-0.01106	-0.0014158
[14974]	ADRBK2	--->	CCR5	0.39714	-0.42229
[14975]	NCOR2	--->	RARG	0.13736	-0.14734
[14976]	MAP3K14	<--->	MAPK3	0.23429	0.23429
[14977]	SMAD4	<---	PSG2	-0.29658	0.33439
[14978]	STAT2	<---	CREBBP	-0.90452	0.89453
[14979]	MPP3	<---	KBTBD4	-0.020086	0.026197
[14980]	ENG	<---	ACVR1	-0.31327	0.30512
[14981]	USP6NL	--->	EPS8	0.071305	-0.15468
[14982]	SP4	<---	E2F1	-0.092247	0.083768
[14983]	STAT6	<---	CEBPB	-0.31266	0.29146
[14984]	DVL3	<---	KLHL12	-0.37628	0.37623
[14985]	GGA1	<---	RABGEF1	-0.011829	0.001291
[14986]	PCBP1	?	PCBP4	-0.037462	-0.037462
[14987]	PPP1R12B	<---	IL16	-0.17253	0.17662
[14988]	ATXN3	<---	PICK1	0.011067	0.025564
[14989]	SCUBE2	?	SCUBE1	-0.013711	-0.013711
[14990]	BCL2L10	?	BAK1	-0.63915	-0.63915
[14991]	CREBBP	--->	ZBTB2	0.066012	-0.076418
[14992]	RNF11	--->	ASCC2	0.2065	-0.17452
[14993]	SYK	<---	MAP4K1	-0.15553	0.18246
[14994]	PRMT1	--->	HIST2H4A	0.3676	-0.35877
[14995]	ARHGEF5	--->	PLCG1	0.36098	-0.40858
[14996]	PIAS4	--->	SMAD3	0.9933	-1.0027
[14997]	DLG2	<---	MAP1A	-0.021355	0.01997
[14998]	EPB41L3	<---	IGSF21	-0.12014	0.10952
[14999]	DLG4	--->	LRP2	0.15231	-0.16223
[15000]	TICAM1	--->	PIAS4	0.036012	-0.047762
[15001]	CDK5	--->	MAPK10	0.64901	-0.41806
[15002]	GIPC3	--->	FZD3	0.27921	-0.30309
[15003]	CD28	<---	LCK	-1.102	1.1416
[15004]	LRP4	<---	DFNB31	-0.41429	0.41365
[15005]	ESR2	<---	NR0B1	-0.044425	-0.0089663
[15006]	MX1	--->	TRPC7	0.041026	-0.053855
[15007]	WISP2	<---	IGF1	-0.13213	0.12573
[15008]	MAPK1	--->	RXRA	0.60286	-0.8344
[15009]	HSP90B1	<---	TLR1	-0.098736	0.10057
[15010]	NBL1	--->	UBQLN1	0.19096	-0.15992
[15011]	MAPK3	--->	RAF1	0.98888	-1.0049
[15012]	SMAD4	<---	ESR2	-0.17818	0.22689
[15013]	PRKCA	--->	PTPN11	0.6319	-0.62682
[15014]	FSHR	--->	FSHB	0.21659	-0.20565
[15015]	PLK4	<---	TEC	-0.37481	0.34073
[15016]	ZNF410	--->	CDKN2A	0.065817	-0.070981
[15017]	HIF1A	--->	PER1	0.84715	-0.022294
[15018]	PLCG1	<---	ALK	-1.1004	0.82322
[15019]	APBA2	--->	LRP1	0.051553	-0.063681
[15020]	JUP	--->	DSC1	0.15626	-0.1649
[15021]	PJA1	--->	MDM2	-0.002635	-0.0079477
[15022]	NR2C2	--->	AR	-0.0031537	-0.58072
[15023]	PRKCD	--->	IRS1	0.99369	-0.99616
[15024]	PORCN	--->	WNT4	1.0005	-0.9854
[15025]	EPS8	<---	MYO15A	-0.057451	0.018492
[15026]	CD14	--->	LTF	0.1132	-0.027357
[15027]	SGK3	--->	SLC9A3R2	0.12244	-0.26256
[15028]	CEBPB	<---	MAPK3	-0.93471	0.97322
[15029]	RGS10	--->	GNAI1	1.0012	-1.0015
[15030]	FGFR1	<---	FGF8	-0.99871	1.0053
[15031]	PTPRC	--->	LCK	0.99837	-0.99758
[15032]	ZNF408	<---	UPF2	-0.017268	0.016344
[15033]	CSNK2A2	--->	RGS19	0.28697	-0.32153
[15034]	RGL4	<---	RALB	-0.76787	0.79809
[15035]	MINK1	--->	MAP1B	0.078073	-0.07512
[15036]	GIT1	--->	PLCG1	0.11838	-0.14491
[15037]	EFS	<---	SORBS3	0.00027436	0.059018
[15038]	NEK11	<--->	NEK2	0.23429	0.23429
[15039]	NUAK1	<---	CASP6	-0.59442	0.52477
[15040]	AR	<---	SRC	-0.266	0.26035
[15041]	PKD1	--->	FN1	0.029037	-0.013871
[15042]	KCND2	--->	NCS1	0.32976	-0.29233
[15043]	NCOA3	<---	ESR2	-0.13412	0.12213
[15044]	ITK	--->	PLCG2	1.0063	-1.0027
[15045]	RHOQ	--->	PFN1	0.47968	-0.45047
[15046]	RAB14	<---	SMURF2	-1.0084	1.0007

[15047]	CDK1	--->	TOP2A	0.52133	-0.54823
[15048]	CCNA2	<---	BRCA1	-0.39925	0.40227
[15049]	DAB1	--->	ITGB7	0.0044867	0.00020414
[15050]	TAX1BP1	<---	TRAF6	-0.42732	0.41877
[15051]	LAMA1	--->	APP	0.12878	-0.14126
[15052]	SATB1	<---	HDAC1	-0.0020697	-0.00052083
[15053]	THY1	--->	ITGAV	0.017537	-0.028129
[15054]	RELA	<---	MAP3K8	-1.0004	1.0015
[15055]	ROBO2	--->	SLIT2	0.00065307	-0.010875
[15056]	PRKACA	--->	ITPR2	0.3288	-0.33886
[15057]	KIF1B	--->	GIPC1	0.086963	-0.089252
[15058]	LYN	--->	FOLR1	1.0426	-0.88182
[15059]	TRAF3	<---	CD40	-0.095839	0.088651
[15060]	RAP2A	--->	SMAD1	0.52759	-0.54836
[15061]	SIGIRR	<---	TRAF6	-0.63812	0.63208
[15062]	PEX10	--->	PEX2	0.15217	-0.11782
[15063]	COPS5	<---	F2RL1	-0.19274	0.21602
[15064]	RAC1	--->	ARHGEF7	0.076055	-0.082042
[15065]	CCR3	--->	CCL3	0.49595	-0.46475
[15066]	RXRA	<---	BCL3	-0.10371	-0.027877
[15067]	TP53BP2	<---	PPP1CA	-0.19228	0.17963
[15068]	KHDRBS1	<---	HCK	-0.61607	0.6674
[15069]	PIAS4	--->	SNIP1	0.039487	-0.037176
[15070]	TCF4	<---	ASCL1	-0.37599	-0.054725
[15071]	MDC1	?	NBN	-0.00097565	-0.00097565
[15072]	FYN	--->	ITGB4	0.12583	-0.12001
[15073]	SRC	--->	JUP	0.22916	-0.21486
[15074]	SH3GL3	--->	EGFL6	0.093076	-0.078222
[15075]	CDKN2C	<---	CDKN2A	-0.48608	-0.19687
[15076]	CGN	<---	F11R	-0.16002	0.15143
[15077]	CISH	<---	PRKCQ	-0.39634	0.42516
[15078]	RGS3	--->	CNKSR2	-0.0017831	-0.013537
[15079]	PCSK7	--->	PABPC1	0.015398	-0.0017254
[15080]	MYH2	<---	PTK2B	-0.014488	-0.0021551
[15081]	PRKAA2	--->	TRIP6	-0.051958	-0.26375
[15082]	APPL1	<---	APPL2	-0.10773	0.0033381
[15083]	MYBL1	--->	NCL	0.0082486	-0.01851
[15084]	RASGRP1	--->	DGKZ	0.13168	0.0092462
[15085]	PTK2B	?	PTK2	-0.13006	-0.13006
[15086]	HNRNPM	?	RBM4B	-0.016298	-0.016298
[15087]	GRB10	<---	MAP2K1	-0.098068	0.13958
[15088]	FYN	--->	MAG	0.088878	-0.12752
[15089]	SMAD7	<---	TGFBR2	-0.57747	0.59933
[15090]	HBP1	--->	RBL2	0.31365	-0.32205
[15091]	ACVR1	<---	NKIRAS1	-0.32935	0.31819
[15092]	MAPK8IP3	<---	MAP2K1	-0.034938	0.11064
[15093]	PIN1	<---	CSNK2A2	-0.49485	0.46953
[15094]	PLCG1	<---	CD22	-0.15906	0.13201
[15095]	CSNK2A2	--->	ACACA	0.41949	-0.42896
[15096]	PI4KB	<---	YWHAQ	-0.068047	0.092991
[15097]	DVL1	<---	PPM1A	-0.33505	0.33032
[15098]	RELA	<---	NR3C1	-0.049342	0.049708
[15099]	CASP10	--->	CASP2	0.26698	-0.40694
[15100]	PKN2	<---	AKT1	-0.037187	0.041211
[15101]	CSNK2A1	--->	BID	0.77321	-0.7866
[15102]	SNRPB2	<---	DHX9	-0.0019217	0.015842
[15103]	DFNB31	<---	CASK	-0.029385	0.030418
[15104]	TSC2	<---	AKT3	-0.99774	1.0085
[15105]	PHC2	--->	LMO3	0.21449	-0.17497
[15106]	BAG1	<---	ESR1	-0.0036637	0.025363
[15107]	RPH3A	<---	CASK	-0.095078	0.08666
[15108]	PARD6A	<---	PRKCZ	-0.058941	0.043604
[15109]	UBE2G1	<---	CBL	-0.47935	0.47348
[15110]	ABCA1	<---	PDLIM4	-0.010698	0.0038951
[15111]	EVL	<---	APBB1	-0.13165	0.13617
[15112]	CHRDL2	--->	INHBA	0.71403	-0.67452
[15113]	SF1	<---	PLSCR1	-0.055054	0.066581
[15114]	TLE1	<---	ARL4D	-0.56872	0.59393
[15115]	BIRC2	--->	CASP9	0.11221	-0.084679
[15116]	JAG1	--->	NOTCH3	0.076185	-0.092263
[15117]	WDR44	<---	RAB11A	-0.51481	0.54415
[15118]	NCOA1	<---	FOXA2	-0.12331	0.11543
[15119]	SP3	<---	EP300	-0.12188	0.11387
[15120]	GRIN2B	<---	PTPN11	-0.20874	0.24158
[15121]	EPHB1	--->	GRB2	0.18974	-0.30775
[15122]	JUN	<---	MYOD1	-0.15382	0.11063

[15123]	YWHAB	--->	BCL2L11	0.20731	-0.20535
[15124]	CASP6	--->	SATB1	0.20813	-0.25733
[15125]	TBL3	<---	ITSN2	-0.04286	0.028
[15126]	ACTN1	<---	SRC	-0.33112	0.36417
[15127]	AR	<---	IL6ST	-0.21155	0.2001
[15128]	CBL	--->	FGR	0.74409	-0.7389
[15129]	DAXX	<---	FASLG	-0.39221	0.36676
[15130]	CHEK2	--->	MRC1	0.46776	-0.49041
[15131]	KAT2B	--->	NCOA1	0.040892	-0.052862
[15132]	ELK1	<---	GRB2	-0.32248	0.3397
[15133]	JAK2	--->	IL3RA	0.42723	-0.31036
[15134]	SKP2	<---	CDK2	-0.61295	0.58677
[15135]	MARK2	<---	MAP3K3	-0.044307	0.042534
[15136]	TP53	--->	CCNA2	0.015421	-0.014652
[15137]	SPTBN4	<---	ANK3	-0.010032	-0.0036427
[15138]	LCP2	<---	SHB	-0.22641	0.26544
[15139]	AGFG2	--->	EPS15	0.075724	-0.085978
[15140]	ERBB2	--->	PTPN11	0.42743	-0.3855
[15141]	RGS9	--->	GNB5	0.34928	-0.33708
[15142]	JUN	<---	HNRNPM	-0.5005	0.45372
[15143]	SELL	--->	MSN	0.0014624	-0.011519
[15144]	CDK1	<---	ABL1	-0.14811	-0.013323
[15145]	RANGAP1	<---	RANBP2	-0.016915	0.0081914
[15146]	STK39	<---	WNK4	-0.083879	0.0018665
[15147]	DKK1	--->	SMAD9	0.3038	-0.32237
[15148]	MAPK1	--->	CD19	0.55021	-0.57067
[15149]	ACTN4	<---	MAGI1	-0.012922	0.0023819
[15150]	ADRA1A	--->	CXCR2	0.5907	0.10636
[15151]	ZFYVE9	--->	SMAD3	0.99525	-0.99685
[15152]	CCL8	<---	CCR2	-0.46475	0.49595
[15153]	ERBB2	--->	CTNNB1	0.05222	-0.064642
[15154]	RAC1	<---	MAP3K11	-0.36833	0.36613
[15155]	PAFAH1B1	<---	CSNK2A1	-0.52301	0.50117
[15156]	ZAP70	--->	GAB2	1.0021	-0.98622
[15157]	CBL	--->	FYB	0.19314	-0.20529
[15158]	HSPH1	--->	PGK1	0.84404	-0.80195
[15159]	TNFRSF18	--->	TRAF2	0.056894	-0.031484
[15160]	ZFYVE20	<---	RAB4A	-0.3573	0.3878
[15161]	PRKCA	--->	ADAP1	0.91081	-0.90921
[15162]	CREBBP	--->	DAXX	0.11141	-0.12082
[15163]	NUP214	--->	SMAD4	0.41245	-0.32954
[15164]	BMPR1B	--->	TNNT1	0.036956	-0.099107
[15165]	ELF1	<---	SP1	-0.20224	0.19397
[15166]	PAK1	<---	TGFBR2	-0.14423	0.066652
[15167]	YWHAQ	<---	CAPN3	-0.24575	0.24371
[15168]	PPARGC1A	--->	SFRS6	-0.0033134	-0.0093093
[15169]	GSK3B	--->	LRP6	0.91677	-0.91735
[15170]	MAP3K7IP2	<---	IRAK1	-0.12803	0.14851
[15171]	GNB2L1	?	GNB1	-0.1554	-0.1554
[15172]	DNAJB1	--->	GLB1	0.032407	0.0017785
[15173]	MELK	--->	CDC5L	0.42526	-0.44642
[15174]	UBQLN4	--->	PELI2	0.16399	-0.13308
[15175]	CNGB1	--->	ABCA4	0.018602	-0.028668
[15176]	SQSTM1	<---	PRKCZ	-0.060181	0.075293
[15177]	PIAS1	--->	PIAS4	0.066672	0.023557
[15178]	PBX1	--->	PKNOX1	0.024726	-0.0094151
[15179]	VDR	<---	CDK7	-1.0079	0.99834
[15180]	HCK	--->	RAPGEF1	0.34923	-0.34388
[15181]	MYC	<---	MAX	-0.31161	-0.23569
[15182]	ITSN2	--->	WAS	0.033179	-0.052031
[15183]	PRKACA	<--->	CDK16	0.23429	0.23429
[15184]	NKRF	--->	RELA	0.10069	-0.094893
[15185]	LIMS1	<---	TGFBR1	-0.34544	0.31529
[15186]	MAPT	<---	FYN	-0.73301	0.80788
[15187]	PRKCB	--->	TOP2A	0.89006	-0.88857
[15188]	IKZF1	<---	CTBP1	-0.069899	0.061002
[15189]	MAX	<---	PLEKHF2	-0.51464	0.525
[15190]	CDC42	<---	LCK	-0.78488	0.87311
[15191]	YWHAB	<---	SRC	-0.89808	1.0093
[15192]	EPB41L1	--->	GRIA4	0.016866	-0.019104
[15193]	PTCH1	<---	CCNB1	-0.22496	0.21637
[15194]	GSK3B	--->	PTK2	-0.06025	-0.061905
[15195]	PTPN11	--->	SOS1	0.13368	-0.098708
[15196]	GLS2	<---	RGS3	-0.3923	0.39729
[15197]	PDGFRB	--->	PIK3R3	0.079229	-0.097993
[15198]	AHR	<---	RELA	-0.017082	0.015556

[15199]	TAP1	<---	ESR1	-0.022043	0.033147
[15200]	CASP3	--->	HTT	0.60022	-0.71121
[15201]	ARHGEF7	<---	PAK1	-0.068611	0.1051
[15202]	IKBKB	--->	NCOA3	0.091219	-0.11492
[15203]	GAB1	--->	PLCG1	0.062033	-0.084037
[15204]	WASF2	<---	ABL1	-0.085162	-0.01425
[15205]	CHD3	<---	UBE2I	-0.021509	0.011465
[15206]	PAK1	<---	SMURF1	-0.36829	0.40085
[15207]	SMURF1	--->	$ZBTB8$	0.47682	-0.48433
[15208]	HOXA1	<---	PLSCR1	-0.080977	0.10682
[15209]	TNFRSF1A	<---	PTPN11	-0.246	0.45864
[15210]	INSR	<---	SRC	-0.085882	0.12818
[15211]	PRKCA	--->	HAND1	1.0168	-1.0076
[15212]	DSC3	<---	CTNNB1	-0.98928	0.99241
[15213]	SFMBT1	--->	PHC2	0.15167	0.15117
[15214]	PPP2R1B	<---	JAK2	-0.043825	0.060025
[15215]	MDM4	--->	YWHAH	0.040034	-0.045818
[15216]	CSF1R	--->	SHC1	0.20211	-0.26094
[15217]	FASLG	--->	EZR	0.17762	-0.17175
[15218]	HDAC7	<---	HDAC3	-0.0012231	0.014661
[15219]	CASP9	<---	BIRC8	-0.036843	0.035713
[15220]	ACP5	--->	SNRNP40	0.13877	-0.12432
[15221]	SOX10	<---	POU5F1	-0.060464	0.063322
[15222]	SOCS2	--->	EPOR	0.055717	-0.058518
[15223]	FOXF2	--->	TBP	0.0086767	-0.021023
[15224]	FN1	<---	DCN	-0.1362	0.16194
[15225]	TRAF3	<---	TNFRSF8	-0.13411	0.1285
[15226]	HNRNPL	--->	HNRNPA2B1	0.027922	0.0040332
[15227]	MTA1	--->	TP53	0.31636	-0.30191
[15228]	CSNK2A1	--->	SSRP1	0.56249	-0.55527
[15229]	EPS15	<---	EPN1	-0.085804	0.075552
[15230]	NTRK2	<---	NTF3	-0.13418	0.13151
[15231]	CPNE2	--->	RDX	0.052591	-0.045879
[15232]	IL5RA	<---	CSF2RB	-0.085024	-0.080624
[15233]	CRK	--->	SHC1	0.22879	-0.2424
[15234]	PPARG	--->	RXRG	0.60524	-0.60641
[15235]	GRID2	<---	GRIK5	-0.030719	-0.022933
[15236]	NOTCH1	<---	NOV	-0.10351	0.088972
[15237]	NCOR2	--->	SRF	0.12702	-0.13737
[15238]	GIT1	<---	PTK2	-0.15323	0.11778
[15239]	FHL3	<---	MAPK1	-0.58259	0.5736
[15240]	CDK5	<--->	CDK16	0.23429	0.23429
[15241]	SORBS2	--->	SYNJ1	0.21152	-0.20965
[15242]	SF1	--->	BCL2	0.42176	-0.42254
[15243]	HIRIP3	--->	HIRA	0.13885	-0.10591
[15244]	PLCG1	<---	AGTR1	-0.41825	0.37047
[15245]	CCKBR	<---	PTPN11	-0.24618	0.35472
[15246]	ADAMTS4	--->	ACAN	0.0043435	-0.015468
[15247]	NCOA2	<---	NR4A1	-0.043163	0.034596
[15248]	BMP8B	?	BMP4	-0.061425	-0.061425
[15249]	STAT5A	<---	JAK1	-0.99052	0.98525
[15250]	BCAR1	<---	INPPL1	-0.0061672	-0.00020042
[15251]	SKP1	--->	FBXW5	0.035778	-0.03566
[15252]	ACVR1	<---	RASL12	-0.32935	0.31819
[15253]	ITGB7	<---	VCAM1	-0.091177	0.080816
[15254]	VAV1	<---	DNM2	-0.066012	-0.01184
[15255]	UBE2L3	--->	BRCA1	0.096255	-0.080771
[15256]	MSX1	--->	TLE1	0.05243	-0.021516
[15257]	YWHAZ	--->	MAPT	0.29081	-0.2771
[15258]	LHCGR	--->	GNAS	1.0129	-1.008
[15259]	ABI1	--->	SOS2	0.0045411	-0.021566
[15260]	ADRB2	<---	GRB2	-0.053429	0.054359
[15261]	PLSCR1	<---	EGFR	-0.28119	0.27464
[15262]	CCND2	<---	CDK5	-1.0774	1.0544
[15263]	MAP3K5	<---	HSPA1A	-0.36101	0.78839
[15264]	TP73	<---	MAPK8	-0.4556	0.45486
[15265]	TCF7L2	--->	PSEN1	0.27754	-0.32234
[15266]	CSRP3	--->	SPTB	0.069648	-0.053191
[15267]	ARHGAP1	<---	CDC42	-0.40165	0.41486
[15268]	GFI1B	<---	ZNRF3	-0.17975	0.17558
[15269]	ZBTB16	<---	HDAC4	-0.121	0.11954
[15270]	LCK	--->	SH2B3	0.99538	-1.0003
[15271]	SRC	--->	TXK	0.60459	-0.016792
[15272]	IL7R	--->	STAT3	0.27541	-0.30414
[15273]	ASAP2	<---	ARF6	-0.13844	0.12847
[15274]	WASL	<---	CDC42	-0.29848	0.22215

[15275]	ZBTB16	<---	SP1	-0.027906	0.034779
[15276]	SHC1	<---	CSK	-0.65926	0.73568
[15277]	DNAI1	<---	PAFAH1B1	-0.062432	-0.059306
[15278]	GRIN1	<---	PRKCB	-0.37606	0.36861
[15279]	NRXN1	<---	SYT9	0.00636	0.012867
[15280]	F2R	--->	GNAI2	1.0129	-1.008
[15281]	TBC1D10A	<---	SLC9A3R1	0.011063	0.025653
[15282]	ANAPC2	<---	UBE2C	-0.11067	0.14316
[15283]	PRKCB	--->	GSK3A	0.22826	0.12473
[15284]	LCK	--->	SHC1	0.36949	-0.31772
[15285]	GRM1	<---	GAPDH	-0.01314	0.028565
[15286]	NR4A1	<---	RPS6KA3	-0.73191	0.56871
[15287]	SMAD7	--->	SOX13	0.18625	-0.10994
[15288]	CDC25A	--->	AR	0.57222	-0.57745
[15289]	AKT3	<---	TCL1A	-0.99692	0.99137
[15290]	PI4KA	--->	CD7	0.0025887	-0.0068441
[15291]	HIPK2	--->	SKI	0.69634	-0.71478
[15292]	SERPING1	<---	C1R	-0.023809	0.04046
[15293]	THBD	<---	PF4	-0.0032742	0.019403
[15294]	NPAS2	<---	RARA	-0.10974	0.11124
[15295]	SHANK1	<---	SPTAN1	-0.0058858	-0.0056091
[15296]	PCSK4	<---	IGF2	-0.019533	0.034667
[15297]	SLC9A3R1	--->	NOS2	0.45762	-0.46444
[15298]	AVPR2	--->	ARRB2	0.27159	-0.25831
[15299]	PAK1	<---	RHOU	-1.0053	0.99704
[15300]	PGR	<---	SORBS1	-0.054481	0.063929
[15301]	RELA	<---	MAP3K7	-0.094382	0.088632
[15302]	SNX2	--->	INSR	0.13241	-0.14074
[15303]	PZP	--->	TGFB1	0.25549	-0.40894
[15304]	HSPA8	--->	APOB	0.82587	-0.78356
[15305]	TERF1	--->	SMAD9	0.30501	-0.32365
[15306]	GRIK2	--->	CTNNB1	0.32204	-0.3267
[15307]	KSR2	--->	MAP2K1	0.077995	-0.027725
[15308]	LIN7C	--->	KCNJ12	0.044422	-0.0070376
[15309]	CAPN1	--->	COL3A1	0.0051297	-0.01782
[15310]	TWIST2	--->	MEF2C	0.044228	-0.04596
[15311]	AKT1	<---	HSP90AA1	-0.99454	0.99649
[15312]	KPNA6	--->	STAT3	0.5471	-0.60142
[15313]	HIF1A	--->	AR	0.042026	-0.055094
[15314]	GRIN2D	<---	PRKCB	-0.2311	0.22109
[15315]	TP53	<---	APEX1	-0.35262	0.36389
[15316]	SRF	--->	FLI1	0.18977	-0.17908
[15317]	KIF1B	--->	ARHGAP1	0.03579	-0.042041
[15318]	PRKCZ	<---	PRKCA	0.00070791	0.10014
[15319]	CCND1	<---	BRCA1	-0.39925	0.40227
[15320]	RASSF2	<---	HRAS	-0.16806	0.16144
[15321]	PRKCZ	--->	NUMB	0.15905	-0.17265
[15322]	RPS6KA2	--->	CREB1	1.0082	-0.98976
[15323]	FMNL1	<---	PRPF40A	-0.11709	0.12018
[15324]	BLNK	<---	CRKL	-0.24364	0.29586
[15325]	UBE2L3	<---	NEDD4	-0.16172	0.15222
[15326]	RAB34	--->	SMAD2	0.55322	-0.47159
[15327]	FGFR3	<---	HNRNPL	-0.15484	0.14823
[15328]	MAPK1	<---	PTPN5	-1.009	1.0061
[15329]	RXRG	<---	RARG	-0.99549	1.003
[15330]	YWHAB	--->	PARD6B	0.018815	-0.024432
[15331]	FBXW11	--->	NFKBIA	0.99943	-1.0169
[15332]	SF1	<---	U2AF2	-0.049452	0.063299
[15333]	HSPA5	--->	APOB	0.82587	-0.78356
[15334]	SBF1	--->	SUV39H1	0.13736	-0.13448
[15335]	PPARGC1A	<---	SIRT1	-0.034436	0.021851
[15336]	GAB2	<---	LYN	-0.99966	0.9993
[15337]	GSK3B	--->	APP	0.38256	-0.39431
[15338]	ATXN1	<---	FAM21C	-0.056516	0.053239
[15339]	DLAT	<---	PDK3	-0.31137	0.32371
[15340]	FBXW11	--->	CUL1	0.29027	-0.30014
[15341]	PTPRF	--->	SKIL	0.065826	-0.077265
[15342]	TP53	<---	HSP90AA1	-0.074478	0.063769
[15343]	SH2B2	<---	PDLIM7	-0.070429	-0.046257
[15344]	CDK12	--->	SFRS2	0.72994	-0.75755
[15345]	SYNJ2BP	--->	SYNJ2	0.028583	0.0077937
[15346]	HDAC3	--->	GTF2IRD1	0.04867	-0.012939
[15347]	HGFAC	--->	HGF	-0.0087417	-0.0096131
[15348]	FOXC1	--->	HMGB1	0.13776	-0.10769
[15349]	TRIM27	<---	EPC1	-0.017605	0.021296
[15350]	STAT5A	<---	ESR1	-0.29089	0.27638

[15351] NKX2-1 ---> PAX8 0.18345 -0.16266
[15352] PEX14 ---> PEX7 0.12816 -0.094579
[15353] MAGI1 <--- RASA1 -0.043891 0.012604
[15354] KCNQ2 ---> CALM3 0.32976 -0.29233
[15355] CCND1 <--- CDKN1A -0.99961 0.98767
[15356] SPTBN4 <--- PTPRN2 -0.54881 0.54341
[15357] DNM2 <--- PPIB -0.09584 0.088971
[15358] ABCA1 <--- LIMK1 -0.061417 0.047673
[15359] DUSP19 ---> MAP3K5 0.79011 -0.3607
[15360] CASP4 ---> SERPINB9 0.030125 -0.020591
[15361] RASIP1 <--- RAP2A -0.64033 0.64447
[15362] PPFIA1 ---> MBIP 0.23414 -0.19777
[15363] CREB1 <--- ATF1 -0.17244 -0.040607
[15364] FLNA ---> SELE 0.030873 -0.017095
[15365] PIN1 ---> TP53 0.47668 -0.46597
[15366] ILK ---> ITGB3 0.10084 -0.1124
[15367] CSNK2A1 ---> ATF1 1.2086 -1.1667
[15368] RAB11FIP3 <--- ARF6 -0.47544 0.48818
[15369] NUDC ---> PAFAH1B1 0.081743 -0.067254
[15370] GTF3C1 <--- TBC1D17 -0.0023129 0.036285
[15371] BLK ---> BCR 0.36363 -0.25336
[15372] DOCK2 ---> RAC1 0.016262 -0.029068
[15373] LYST <--- YWHAQ -0.19355 0.2164
[15374] ZNF423 <--- SMAD4 -0.03962 0.048771
[15375] RPS6KB1 ---> RPS6 0.98649 -0.99602
[15376] YWHAG ---> TAF15 0.023974 -0.016119
[15377] HEY2 <--- HAND2 -0.43036 -0.38951
[15378] HIP1 ---> CASP9 0.035887 -0.02764
[15379] GATA5 <--- HNF1A -0.015702 0.056147
[15380] GGA2 <--- SYNRG 0.011562 0.022527
[15381] HDAC9 <--- HDAC1 0.0010361 0.021264
[15382] SOCS1 <--- EGFR -0.58054 0.56683
[15383] CIB1 <--- PSEN2 -0.34233 0.37985
[15384] ARHGEF6 ---> BMPR1B 0.0043459 -0.030693
[15385] TGM2 ---> ITGB3 0.047661 -0.050201
[15386] PIK3CG <--- GAB1 -0.087942 0.076408
[15387] PAK1 ---> GIT2 0.24364 -0.19118
[15388] STAT3 <--- IRAK1 -0.23189 0.23272
[15389] SERPINB9 <--- UBR1 -0.17998 0.21644
[15390] TIMP2 ---> MMP8 0.21399 -0.19321
[15391] YWHAG ---> TRA2A 0.092231 -0.070624
[15392] WISP1 <--- BGN -0.30201 0.29267
[15393] PTK6 <--- ERBB3 0.18397 0.24432
[15394] TESK2 ---> CFL1 0.43358 -0.44548
[15395] CDC5L ---> CDKN2A 0.14696 -0.12821
[15396] LRDD ---> CCDC85B 0.0072843 -0.022577
[15397] BNIP2 <--- CDC42 -0.34856 0.3794
[15398] YWHAG <--- TNFAIP3 -0.13918 0.13176
[15399] FZD5 <--- WNT5A -0.2481 0.24992
[15400] BMP1 <--- ADRA1A -0.36577 0.36452
[15401] KCND2 <--- DLG4 -0.049447 0.047736
[15402] MEIS1 ---> HOXA10 0.022206 -0.017736
[15403] CASP8 ---> PIAS1 0.16431 -0.22241
[15404] ARHGEF6 ---> SMAD1 0.11418 -0.13153
[15405] GAB2 <--- GRAP2 -0.79676 0.76817
[15406] DUOX1 <--- TPO -0.035296 0.016316
[15407] SRC ---> MUC1 0.70785 -0.68334
[15408] ACP1 ---> FNBP1L 0.20281 -0.20329
[15409] MAPK14 ---> SMAD3 1.0053 -0.99469
[15410] NOS1 <--- ADRB1 -0.13158 0.11901
[15411] PPM1J ---> UBE2I 0.23817 -0.22725
[15412] JUN <--- EGR1 -0.18465 0.15051
[15413] TCF21 <--- LMO4 -0.58677 0.62562
[15414] CREB1 <--- RECQL5 -0.19157 0.19341
[15415] WASF1 <--- GRB2 -0.32992 0.32085
[15416] SRC ---> HNF1A 1.0216 -0.90865
[15417] SNTG2 ---> DTNB 0.020806 0.0159
[15418] SRF ---> TEAD1 0.0499 -0.028627
[15419] CDC42 ---> FNBP1 0.13778 -0.12591
[15420] GFRA1 ---> GDNF 0.39482 -0.35487
[15421] SOX10 <--- PAX6 -0.029062 0.025489
[15422] MLLT4 <--- CTNNA1 -0.21644 0.26497
[15423] BCR <--- SRC -0.26216 0.3195
[15424] SMAD7 <--- MAP3K7 -0.21256 0.23582
[15425] PI4K2B ---> CD247 0.22325 -0.23376
[15426] NR3C1 <--- YWHAH -0.092332 0.11204

[15427] MYD88 <--- IRAK1 -0.046878 0.23479
[15428] SERPINB8 <--- FURIN -0.035545 0.040022
[15429] MAPK3 ---> TAL2 1.5563 -1.5271
[15430] NR1I2 <--- SRC -0.55261 0.61826
[15431] LRP6 <--- ANTXR2 -0.6819 0.68261
[15432] RLN3 <--- RXFP1 -0.14912 0.15462
[15433] THBS1 <--- COL2A1 -0.04908 -0.0027015
[15434] ITGAL <--- ICAM3 -0.094837 0.08302
[15435] WIPI2 ? STRN4 -0.1554 -0.1554
[15436] UBE2I ---> SP3 0.32873 -0.31052
[15437] EZR ---> ACTC1 0.1724 -0.17979
[15438] NPAS2 <--- CREBBP -0.079572 0.069203
[15439] ESR1 ---> JUNB 0.24409 -0.27062
[15440] VAV1 <--- JAK2 -0.063533 0.060428
[15441] APAF1 ---> BCL2L1 0.37579 -0.37711
[15442] CCL17 <--- VCAM1 -0.22304 0.21853
[15443] HNRNPD ---> PCBP2 0.036464 -0.0048144
[15444] LMO2 ---> LYL1 0.62562 -0.58677
[15445] PDGFRB ---> RAF1 0.015632 -0.046798
[15446] NSD1 ---> RXRA 0.0748 -0.28412
[15447] HSP90B1 <--- CSNK2A1 -0.22149 0.20285
[15448] RALGDS <--- HRAS -1.0084 0.98547
[15449] UBE2D2 <--- NEDD4 -0.086802 0.07541
[15450] PRKCA ---> PFKFB2 0.57052 -0.57532
[15451] PTPN11 ---> PLCG1 0.3891 -0.71038
[15452] SRPK1 ---> SFRS5 0.72994 -0.75755
[15453] EGFR ---> EPB41 0.10938 -0.11225
[15454] PTPN6 <--- TRAF6 -0.24835 0.27798
[15455] CAV2 ? CAV1 -0.011563 -0.011563
[15456] ATXN1 <--- NCOR1 -0.024699 0.012467
[15457] BRD7 ---> HNRNPUL1 0.030324 -0.015416
[15458] SOCS3 ---> TCEB1 0.024927 -0.0040225
[15459] NUCB1 <--- GNAI3 -0.49311 0.51194
[15460] HSPB1 ---> TGFB1I1 0.079081 -0.076856
[15461] SFRP2 ---> PAX2 0.33557 -0.28628
[15462] PAK1 <--- CDC42 -1.0053 0.99704
[15463] SP1 ---> TAL1 0.16786 -0.17899
[15464] SIM1 ---> ARNT -0.039196 -0.040484
[15465] SOCS7 <--- NCK1 -0.070203 0.21075
[15466] PRKCH <--- CDK2 -0.033187 0.63541
[15467] RALBP1 <--- RAC1 -0.99572 1.0059
[15468] RCOR3 <--- NINL -0.19589 0.21098
[15469] PIN1 ---> CCNE1 0.82413 -0.8118
[15470] BTLA <--- PTPN11 -0.077948 0.26319
[15471] ATN1 ---> NELL1 0.010628 -0.018507
[15472] PTPRD <--- PTPRS -0.014244 -0.014175
[15473] SERPINB12 ? SERPINF2 -0.11243 -0.11243
[15474] TRRAP ---> ESR1 0.0009567 -0.011515
[15475] ADAM15 <--- HCK -0.53832 0.63049
[15476] MAPK8 ---> JUND 1.0022 -1.0148
[15477] BCL2L1 <--- CRYAA -0.6937 0.70968
[15478] DEDD ---> CASP8 0.54362 -0.56644
[15479] GRAP ---> EPOR 0.020289 -0.052551
[15480] EPHB2 ---> PICK1 0.36684 -0.37243
[15481] DVL1 <--- ARRB1 -0.2363 0.23088
[15482] ADRB3 <---> ADRB2 0.016768 0.016768
[15483] PRKACA ---> GSK3A 0.16704 0.057781
[15484] ACTR2 ---> ACTC1 -0.065578 -0.55039
[15485] PAK1 ---> SMAD4 0.68 -0.57197
[15486] TRIM3 ---> MYO5A 0.019602 -0.015582
[15487] APEX2 <--- CCDC85B -0.038619 0.067431
[15488] ATXN1 <--- GAPDH -0.023777 0.016258
[15489] ARL14 <--- SMURF1 -0.65068 0.64578
[15490] HDAC2 ---> MXD1 0.38449 -0.36669
[15491] LCK ---> CD247 1.001 -1.0054
[15492] PCSK7 <--- HSPA5 -0.48877 0.50881
[15493] PPAP2B ---> SHC1 0.078306 -0.17977
[15494] NTRK2 <--- NTF4 -0.075412 0.076577
[15495] AHR ---> AR 0.5587 -0.56444
[15496] JAK2 ---> NFKBIA 0.18178 -0.11406
[15497] SENP3 <--- EP300 -0.11677 0.10885
[15498] STAT1 <--- BMX -0.22542 0.23671
[15499] MAP3K7IP2 <--- TRAF6 -1.0029 1.0051
[15500] BARX2 <--- SRF -0.11346 0.13352
[15501] FCER2 <--- FYN -1.0651 1.2131
[15502] IFT80 ---> PLXNA2 0.14499 -0.1314

[15503]	CCR5	<---	LCK	-0.7217	0.8049
[15504]	FYN	<---	PTPRZ1	-0.55938	0.43621
[15505]	DLK2	<---	ADAMTSL4	-0.053385	0.04264
[15506]	JUP	<---	ERBB2	-0.07097	0.05922
[15507]	SNIP1	--->	SMAD4	0.26592	-0.22069
[15508]	CDK5RAP2	--->	EEF1G	0.028282	0.0066341
[15509]	TRIM27	--->	FXYD6	0.084773	-0.078783
[15510]	ACVR1	--->	RXRA	0.092623	-0.15572
[15511]	E4F1	<---	SMAD9	-0.13203	0.11854
[15512]	SPIB	<---	CEBPB	-0.052983	0.11084
[15513]	SPRED2	<---	RHOA	-0.30977	0.32428
[15514]	SMAD3	<---	SNRNP70	-0.32493	0.40696
[15515]	HIPK2	--->	TP73	0.45486	-0.4556
[15516]	MYST2	<---	CBX8	-0.043394	0.063146
[15517]	MERTK	--->	GRB2	0.1921	-0.25229
[15518]	PTK2	--->	ATG12	0.063886	-0.17094
[15519]	PAK1	--->	CASP1	0.58095	-0.43163
[15520]	LMO4	--->	SMAD9	0.31038	-0.32822
[15521]	RB1	<---	SUV420H2	-0.50032	0.49931
[15522]	PPBP	<---	CXCR1	-0.46475	0.49595
[15523]	RPS6KC1	--->	PBX2	0.16435	-0.18145
[15524]	PRKCZ	--->	GSK3A	0.050924	0.0093507
[15525]	PDCD6IP	<---	PTK2B	-0.13253	0.027133
[15526]	FN1	--->	MMP9	0.18519	-0.16758
[15527]	WWOX	--->	TNK2	0.033836	-0.046873
[15528]	ACAN	<---	HAPLN1	-0.012533	-0.010162
[15529]	IL6ST	<---	IL6R	-0.042891	-0.040767
[15530]	YWHAG	<---	RAPGEF6	-0.40143	0.37632
[15531]	LAMA1	--->	NID1	0.018222	-0.025274
[15532]	SYK	<---	PTPN6	-1.0074	1.0106
[15533]	DNM1	<---	CTTN	-0.066084	0.061861
[15534]	CRKL	<---	INSR	-0.077994	0.057748
[15535]	USP6NL	<---	RAB5A	-0.35867	0.38941
[15536]	MEGF10	--->	SNRNP200	-0.0044959	-0.0089101
[15537]	RNF11	--->	TOM1L2	0.19846	-0.16573
[15538]	BTBD3	<---	PLXNB3	-0.059845	0.093156
[15539]	GGCX	--->	F10	0.040593	-0.04351
[15540]	E2F5	<---	SMAD3	-0.13771	0.19366
[15541]	CABIN1	<---	MEF2B	0.0010503	0.035098
[15542]	PRKX	--->	PRKAR2A	0.27068	-0.33631
[15543]	MTA2	<---	SATB1	-0.058645	0.066181
[15544]	PIAS3	--->	PRPF40A	0.0091068	0.0015163
[15545]	BRSK1	--->	CDC25B	0.41582	-0.41122
[15546]	MPP3	<---	MORN2	-0.11263	0.11924
[15547]	MAPK3	--->	STAT3	1.0077	-1.0166
[15548]	CRKL	--->	EPOR	0.020289	-0.052551
[15549]	PIK3R1	<---	CTLA4	-0.14775	0.074775
[15550]	NR0B1	--->	PPARG	-0.0081193	-0.030626
[15551]	RGS4	--->	GNAI2	1.0012	-1.0015
[15552]	YWHAG	--->	RPS2	0.081652	-0.057314
[15553]	NCOR1	--->	ESR1	0.063164	-0.065644
[15554]	CSNK2A1	--->	HES6	0.97225	-0.95979
[15555]	RXRA	<---	NR2F1	-0.99549	1.003
[15556]	RNPS1	<---	CDC2L2	-0.40469	0.38252
[15557]	MAP2K1	--->	MAPK14	0.93073	-1.0267
[15558]	BCL3	<---	HDAC6	-0.062887	0.06888
[15559]	KAT2A	<---	CREBBP	-0.0041023	0.061921
[15560]	DAZ1	?	DAZAP1	-0.016298	-0.016298
[15561]	PARD3	<---	YWHAZ	-0.030232	0.031301
[15562]	LATS1	<---	STK4	-0.19851	0.19722
[15563]	RIPK1	<---	HSP90AA1	-0.004716	0.026638
[15564]	IRAK2	--->	PELI2	0.85311	-0.63354
[15565]	YWHAG	--->	IGF1R	0.024667	-0.036569
[15566]	ICAM1	--->	SLC3A2	0.027957	-0.036229
[15567]	UBE2I	--->	MYB	0.025979	-0.033467
[15568]	TRIM22	<---	CIC	-0.086302	0.10117
[15569]	TRIB3	--->	ATF4	0.99807	-1.0029
[15570]	FXYD1	--->	ATP1A1	0.097298	-0.097228
[15571]	TRPV4	--->	CALM1	0.24842	-0.24255
[15572]	TJP1	<---	EGFR	-0.16284	0.15596
[15573]	PDZK1	--->	SLC9A3	0.065872	-0.059787
[15574]	PIK3R2	<---	ERBB2	-0.21459	0.18986
[15575]	TGFB1I1	<---	NR3C1	-0.048168	0.044863
[15576]	NPY1R	<---	NPY	-0.19468	0.20502
[15577]	PLD2	<---	LCK	-0.14987	0.1452
[15578]	GIPC2	--->	FZD3	0.27921	-0.30309

[15579]	CCL16	<---	CCR5	-0.46475	0.49595
[15580]	PARD6G	<---	CDC42	-0.18257	0.18762
[15581]	CD1D	--->	CALR	0.084283	-0.064247
[15582]	CDK3	--->	KIR3DL1	0.11063	-0.11082
[15583]	EIF4G3	--->	EIF4E	0.3621	-0.35286
[15584]	USP7	--->	ARNT2	0.24824	-0.24193
[15585]	SH3GLB1	<--->	AMPH	0.0094254	0.0094254
[15586]	TYK2	--->	IFNAR1	0.17162	-0.18353
[15587]	FYN	--->	SNX26	0.4469	-0.38316
[15588]	ANGPT4	<---	TEK	-0.014198	0.00032334
[15589]	WASF1	--->	BAD	0.20314	-0.21075
[15590]	ATN1	<---	HSPG2	-0.0093904	-0.0038491
[15591]	CSNK2A1	<---	HIF1A	-0.84006	0.84197
[15592]	DTNA	--->	GFI1B	0.15215	-0.15612
[15593]	ST14	<---	SPINT1	-0.021112	-0.013372
[15594]	SOSTDC1	--->	CDC20	0.16715	-0.13431
[15595]	KIRREL	--->	TJP1	0.054633	-0.064221
[15596]	FGFR2	<---	FGF17	-0.53789	0.54173
[15597]	GADD45G	--->	MAP3K4	1.0051	-0.99563
[15598]	OCLN	<---	SMURF1	-0.71048	0.70665
[15599]	THBS1	<---	IGFBP5	-0.28782	0.38263
[15600]	PRKCA	<---	PRKG1	-0.4183	0.6366
[15601]	ATXN1	<---	CPSF7	-0.034367	0.027552
[15602]	SYT4	<--->	SYT1	0.058592	0.058592
[15603]	EP300	--->	DDX24	0.10766	-0.11577
[15604]	CSF2RB	<---	CSF2	-0.24221	0.23035
[15605]	NOVA1	<---	SMARCC2	-0.033002	0.067059
[15606]	CRK	<---	NTRK1	-0.12933	0.031701
[15607]	WAS	<---	ITK	-0.53132	0.4868
[15608]	SLC8A1	<---	YWHAQ	-0.092105	0.10171
[15609]	ESR1	?	RARA	-0.0089162	-0.0089162
[15610]	CD2AP	<---	CBL	-0.32919	0.31754
[15611]	SPOP	--->	PDX1	0.071447	-0.056119
[15612]	FHL2	--->	PFKM	0.041726	-0.0064134
[15613]	MAP2K7	<---	MAP3K5	-0.99683	0.9934
[15614]	RIPK2	--->	TRAF1	0.18737	-0.19913
[15615]	RHOXF2	<---	RBPMS	-0.071742	0.10141
[15616]	GAPDH	--->	APP	0.21673	-0.22826
[15617]	MYC	<---	BRCA1	-0.18176	0.18276
[15618]	NPPB	<---	NPR1	-0.15882	0.14651
[15619]	SNX4	--->	INSR	0.13241	-0.14074
[15620]	HGS	--->	EHMT2	0.020711	-0.042611
[15621]	KLF4	--->	KLF6	-0.040055	-0.15885
[15622]	MAPK1	--->	NCOA3	0.61693	-0.63156
[15623]	NEK6	--->	NECAB2	1.007	-1.0109
[15624]	NEDD1	<---	EP300	-0.19054	0.18618
[15625]	IGFALS	--->	IGFBP5	0.10584	-0.093579
[15626]	NCOA2	<---	PRMT1	-0.072651	0.060923
[15627]	CCNG1	--->	TP53	0.10251	-0.10101
[15628]	SFTPD	--->	DCN	0.0074081	-0.017488
[15629]	SERPINB9	<---	GZMB	-0.082185	0.11833
[15630]	PRAM1	<---	SKAP2	-0.049292	0.033299
[15631]	GNAO1	<---	RGS10	-1.0015	1.0012
[15632]	CASP8	--->	EIF2AK2	0.99111	-0.99197
[15633]	NT5E	--->	FN1	0.14543	-0.11676
[15634]	CSF2RB	<---	KIT	-0.18865	0.14982
[15635]	ATN1	<---	EFCAB4B	-0.014773	0.012495
[15636]	PTPN11	--->	ARHGAP1	0.46853	-0.34679
[15637]	CSTF2	<---	UBQLN4	-0.057469	0.089135
[15638]	WNT2	--->	FZD1	0.15445	-0.15879
[15639]	EPAS1	--->	EP300	-0.0066632	-0.0068904
[15640]	RNF11	<---	CBLB	-0.48107	0.47507
[15641]	PRKCZ	--->	RAF1	0.23215	-0.27161
[15642]	SLC9A2	<---	SRC	-0.27246	0.28899
[15643]	EIF4E2	<---	ARIH1	-0.20479	0.20515
[15644]	ACP1	--->	EPHA2	0.023989	-0.034598
[15645]	ARAF	--->	KLHL12	0.029526	-0.055538
[15646]	DNM2	<---	SRC	-0.28153	0.3136
[15647]	HSP90AA1	--->	STAT3	0.077565	-0.096904
[15648]	FOXG1	<---	FOXO3	0.034125	0.39933
[15649]	PARD3	--->	SMAD2	0.14536	-0.12329
[15650]	YWHAB	<---	RABGEF1	-0.0064412	-0.0049278
[15651]	PIAS3	--->	SMAD2	1.0161	-0.98478
[15652]	CDK14	--->	YWHAB	0.62776	-0.66368
[15653]	MAPK3	<---	MKNK1	0.13867	0.16997
[15654]	PTK2	--->	SHC1	0.060324	-0.26424

```
[15655] AKT2    --->    SLC2A4  0.49889 -0.35306
[15656] GHRL    --->    NPY     0.071009        -0.052988
[15657] CDKL2   <---    CBL     -0.34518        0.32905
[15658] BAK1    --->    TP53    0.15938 -0.1543
[15659] ERN1    --->    PSEN1   0.41017 -0.43131
[15660] PAK2    --->    EIF4B   0.29371 -0.26131
[15661] PIK3R2  <---    IRS1    -0.058683       0.032789
[15662] PVRL1   --->    PVR     0.00037536      -0.021316
[15663] ZFP2    <---    PTPN12  -0.31711        0.30651
[15664] AKT1    --->    GSK3B   1.0168  -1.0071
[15665] NCF2    <---    CDC42   -0.13569        0.12502
[15666] CD93    <---    COL4A3  -0.031485       0.019975
[15667] ACVR1   <---    PLEK    -0.022831       -0.0094324
[15668] PDGFB   --->    A2M     0.12055 -0.10555
[15669] LAMB1   <---    COL6A1  -0.022736       0.012961
[15670] CHD3    --->    PRG2    0.00059694      -0.010792
[15671] DNMT3A  <---    DNMT1   -0.033562       0.048618
[15672] KCNA4   <---    DLG1    -0.049447       0.047736
[15673] TRPC3   --->    ITPR1   -0.0071319      -0.016919
[15674] EGFR    <---    PTPN1   -0.079237       0.074053
[15675] ZHX1    --->    BHLHE40 0.16833 -0.16322
[15676] RBBP6   --->    LRCH4   0.031429        -0.024878
[15677] GSK3B   --->    CTNND1  0.31524 -0.34547
[15678] SDCBP   --->    WDR91   0.18927 -0.17397
[15679] AR      <---    PTEN    -0.57187        0.56639
[15680] ARFGAP1 <---    CSNK1D  -0.58138        0.71795
[15681] NCAM1   --->    GDNF    0.22314 -0.20811
[15682] RANBP9  <---    MET     -0.23016        0.21784
[15683] PELI3   <---    MAP3K14 -0.91867        0.87996
[15684] ADRA1B  --->    FLNC    0.33997 -0.33598
[15685] GHR     <---    JAK1    -0.21159        0.19869
[15686] DLL4    --->    NOTCH1  -0.0069767      -0.036833
[15687] APC     <---    JUP     -0.017995       -0.0075596
[15688] TRAF4   <---    LTBR    -0.02119        0.016885
[15689] MAP2K2  --->    MAPK1   1.0159  -0.99672
[15690] SCN3A   --->    LRP1    0.1464  -0.15554
[15691] CHD3    <---    HTT     -0.0053996      -0.0046032
[15692] PDCD6IP <---    YES1    -0.91248        1.0733
[15693] MSC     ?       ID2     -0.65635        -0.65635
[15694] NPHP1   --->    FLNB    0.18956 -0.17815
[15695] GFI1B   <---    CCDC85B -0.16538        0.16074
[15696] PTPN6   --->    ACTN1   0.29245 -0.2308
[15697] ESR1    <---    PIAS3   -0.090133       0.095399
[15698] PLCG2   <---    FCER1G  -0.21135        0.10278
[15699] FRS2    <---    SOS1    -0.57326        0.57066
[15700] DLG4    <---    HTR2C   -0.056804       0.049427
[15701] ITK     --->    SMAD4   0.31089 -0.32408
[15702] EFEMP1  --->    GFI1B   0.14936 -0.1532
[15703] ATXN1   <---    QRICH1  -0.019912       0.016709
[15704] PPARD   <---    NR0B2   -0.028437       -0.0094276
[15705] GAPDH   <---    ARL15   -0.26528        0.27577
[15706] KRT15   <---    PCM1    -0.0071777      0.022499
[15707] PTPRC   --->    FYN     1.007   -0.99313
[15708] AR      <---    HSP90AA1        -0.13812        0.12595
[15709] CSNK2A1 --->    VDR     0.99834 -1.0079
[15710] RBL2    <---    CDK2    -0.47326        0.45745
[15711] GIT1    <---    WDR33   -0.066936       0.056406
[15712] PSEN1   --->    CDH1    0.15557 -0.15122
[15713] SIGIRR  <---    TLR5    0.042913        0.22804
[15714] RAGE    --->    MYC     1.0154  -1.0065
[15715] RASSF8  <---    YWHAG   -0.078347       0.10004
[15716] ELF3    <---    EP300   -0.12299        0.11504
[15717] INPPL1  <---    MET     -0.17831        0.095438
[15718] SNRPA   <---    C19orf43        -0.0031348      0.034771
[15719] ADRA1A  --->    ADRA1D  0.10332 0.10046
[15720] MYF5    <---    CALM2   -0.22666        0.2676
[15721] IRS2    <---    PIK3R2  -0.041802       0.026325
[15722] DIAPH1  <---    PRPF40A -0.066411       0.05891
[15723] SMARCA4 --->    PAX6    0.01737 -0.027391
[15724] GSK3B   --->    MITF    1.0118  -0.98821
[15725] EIF2AK2 --->    MAP3K7IP2       0.18133 -0.19831
[15726] NCOA2   <---    RORA    -0.043163       0.034596
[15727] AKT2    --->    TSC2    1.0085  -0.99774
[15728] IL5RA   <---    JAK1    -0.38108        0.37392
[15729] PRKCD   --->    ADRBK1  0.20529 0.0040446
[15730] TCF3    <---    CREBBP  -0.058216       0.046434
```

[15731]	BAG4	<---	TNFRSF1A	-1.0013	1.0009
[15732]	HSPA2	--->	CDK1	1.0063	-0.99901
[15733]	HIPK2	--->	CREBBP	0.32272	-0.32425
[15734]	LAIR1	<---	PTPN11	-0.077948	0.26319
[15735]	NR2E3	<---	HDAC1	-0.074402	0.085304
[15736]	HOMER1	--->	GRIA1	0.21562	-0.22
[15737]	ATM	<---	RHEB	-0.22838	0.21827
[15738]	PRMT1	<---	STAT1	-0.082675	0.028398
[15739]	RORB	--->	NCOA1	0.034596	-0.043163
[15740]	SMAD2	<---	UBR5	-0.46159	0.54423
[15741]	THRB	<---	NCOR1	-0.065644	0.063164
[15742]	SHC1	<---	DOK1	-0.29232	0.13337
[15743]	JAK1	--->	STAT1	1.0095	-1.0128
[15744]	PLCG1	<---	KDR	-1.0003	1
[15745]	HDAC6	<---	EP300	-0.11672	0.10861
[15746]	MLST8	--->	RPTOR	-0.00608	-0.21415
[15747]	H2AFX	<---	BRCA1	-0.14798	0.17426
[15748]	RAB3B	--->	SMAD4	0.55322	-0.47159
[15749]	SNIP1	--->	TOPORS	0.057671	-0.040806
[15750]	EP300	--->	YY1	0.065354	-0.075677
[15751]	ADAM9	--->	ITGA6	0.00087399	-0.012698
[15752]	EP300	--->	RAN	0.17039	-0.17482
[15753]	SLC8A2	<---	YWHAQ	-0.092105	0.10171
[15754]	TGFBR3	--->	TGFB3	0.18653	-0.16345
[15755]	MYD88	<---	TLR10	0.020105	0.14052
[15756]	ZBTB17	<---	MSX2	-0.25697	0.24436
[15757]	AGT	<---	AGTR1	-0.47275	0.48854
[15758]	FZD6	<---	WNT4	-0.2481	0.24992
[15759]	NTRK3	<---	PTPN1	-0.20877	0.2053
[15760]	ESR2	<---	PRMT2	-0.11586	0.12534
[15761]	NR3C1	--->	STAT3	0.3307	-0.36706
[15762]	SMAD3	<---	SVEP1	-0.0091406	-0.0011456
[15763]	ADAM15	<---	SORBS2	-0.10904	0.10503
[15764]	EGFR	--->	RASA1	1.0038	-0.99351
[15765]	BCL2	--->	ITPR1	0.1937	-0.20523
[15766]	RASA1	<---	LYN	-0.57122	0.21292
[15767]	RB1	<---	BRCA1	-0.30058	0.29434
[15768]	UBQLN2	<--->	UBQLN4	0.069208	0.069208
[15769]	USF2	<---	BRCA1	-0.37182	0.38971
[15770]	DDX5	--->	TP53	0.10983	-0.11451
[15771]	XRN1	<---	PABPC4	0.0010642	0.001779
[15772]	RHOJ	--->	WAS	0.36914	-0.48596
[15773]	PAK2	<---	LIMK1	-0.08975	0.17985
[15774]	CASP9	<---	BIRC3	-0.084679	0.11221
[15775]	HNRNPC	<---	CCDC85B	-0.050711	0.077385
[15776]	PIK3CD	<---	TNPO2	-0.48002	0.48
[15777]	LCK	--->	ESR1	0.53046	-0.4838
[15778]	RGS18	--->	GNAI1	1.0012	-1.0015
[15779]	BAZ1B	<---	SMARCE1	-0.072271	0.075468
[15780]	STMN2	--->	TRPC5	0.075949	-0.087621
[15781]	HNRNPL	<---	HMG20B	-0.16988	0.20345
[15782]	CASP7	--->	STK24	0.87492	-0.98085
[15783]	RHPN2	<---	TGFBR1	-0.37858	0.34978
[15784]	ANKRD11	<---	SRC	-0.73527	0.78397
[15785]	ADAP1	--->	SFRS2	0.033447	-0.0018603
[15786]	DACH1	<---	SIX1	-0.021357	0.036066
[15787]	SVIL	<---	MLL	-0.023252	0.053621
[15788]	ZMYND11	<---	BMPR1A	-0.099706	0.073759
[15789]	GSK3A	--->	MCL1	0.49108	-0.54794
[15790]	DLG2	<---	KCNJ4	-0.037047	0.04386
[15791]	RGS12	<---	NPY2R	-0.13722	0.13311
[15792]	CTNNA1	--->	CDH2	0.33273	-0.3322
[15793]	CAPN1	--->	FHL2	0.25476	-0.25427
[15794]	MEGF10	--->	MCF2L2	-0.0062286	-0.0075185
[15795]	SPRY2	<---	PLSCR1	-0.035506	0.063476
[15796]	SERPINB2	<---	PRPF8	-0.32079	0.34926
[15797]	ZBTB16	<---	ESR1	-0.059674	0.055651
[15798]	PRKCB	--->	EPB41	0.33349	-0.34166
[15799]	KRT1	--->	CALB2	0.08484	-0.096028
[15800]	IRS1	--->	ATP2A3	0.13686	-0.14511
[15801]	HMOX2	--->	EEF1G	0.026536	0.0083461
[15802]	RBM9	--->	ESR2	0.064959	-0.040633
[15803]	ZNF165	<---	PDLIM7	-0.18191	0.17784
[15804]	KIDINS220	<---	NTRK2	-0.17084	0.16577
[15805]	CASP14	--->	CASP2	0.1604	-0.089889
[15806]	DOCK3	--->	PSEN2	0.12827	-0.14024

[15807]	WAS	<---	HCLS1	-0.20758	0.17746
[15808]	TGFB1	<---	MMP2	-0.20485	0.10852
[15809]	MMP1	--->	CD44	-0.0016332	-0.0037852
[15810]	GRM1	<---	ADRBK1	-0.53628	0.51226
[15811]	TARDBP	--->	ZHX1	0.045785	-0.042886
[15812]	CACNA1G	<---	UBQLN4	-0.06887	0.084075
[15813]	BRD2	--->	HIST3H2BB	0.29322	-0.24229
[15814]	ADAP1	<---	CSNK1A1	-0.49364	0.57334
[15815]	PDGFRB	--->	GRB2	0.034846	-0.10537
[15816]	CASP3	<---	NEDD4	-0.072983	0.051513
[15817]	EP300	--->	STAT5A	0.54507	-0.55643
[15818]	SLC22A11	<---	SLC9A3R1	-0.20736	0.21946
[15819]	MAP4K5	--->	CRK	0.15333	-0.12435
[15820]	YWHAB	--->	IRS2	0.072749	-0.080235
[15821]	ITGB2	<---	FCER2	-0.2227	0.2136
[15822]	PLCB2	<---	GNAQ	-0.63757	0.63397
[15823]	RBM7	?	SFRS3	-0.016298	-0.016298
[15824]	MTMR4	<---	HIPK3	-0.51441	0.49278
[15825]	GADD45G	--->	ESR1	0.017932	0.0079001
[15826]	HSPG2	--->	GFI1B	0.011827	-0.025209
[15827]	TIAM1	<---	EPHA2	-0.21119	0.20059
[15828]	GATA1	<---	MAPK3	-0.99372	1.0021
[15829]	FOSB	<---	JUND	-0.12398	-0.057338
[15830]	EEA1	<---	RAB5C	-0.3573	0.3878
[15831]	SYK	--->	STAT3	0.78889	-1.0582
[15832]	DVL2	<---	BAG3	-0.25166	0.24517
[15833]	ITGA6	<---	GRB2	-0.052075	0.041345
[15834]	HOMER2	--->	RYR1	0.075907	-0.088028
[15835]	BMP2	<---	GREM1	-0.51806	0.56685
[15836]	ADCY5	<---	GNAS	-0.63771	0.64323
[15837]	ZBTB43	<---	LNX1	-0.16116	0.15506
[15838]	KCNJ12	<---	LIN7A	-0.016752	0.033961
[15839]	RAB3B	--->	SMAD1	0.52759	-0.54836
[15840]	SH3D19	--->	SH3GL3	0.049844	0.0048783
[15841]	KCNA5	<---	ACTN2	-0.11983	0.12376
[15842]	CHUK	--->	HSP90AA1	0.023686	-0.064239
[15843]	FLNA	<---	ARRB2	-0.0077442	0.022957
[15844]	AXIN1	--->	DVL1	-0.036269	-0.061523
[15845]	JAK2	<---	PDGFRB	0.0001069	0.003212
[15846]	HES1	<---	ID2	-0.29914	-0.087238
[15847]	LMO2	--->	MAPRE3	0.066481	-0.030995
[15848]	LAT	<---	ITK	-0.9883	1.0001
[15849]	PAX6	<---	HOXB1	-0.099649	-0.043846
[15850]	SRC	--->	DAB1	1.0524	-0.90972
[15851]	MAP3K5	--->	YWHAH	0.23745	0.17403
[15852]	EPHB1	--->	PXN	0.42431	-0.42927
[15853]	JUN	<---	BRCA1	-0.32745	0.29484
[15854]	PPID	<---	HSPA8	-0.82444	0.86854
[15855]	YWHAG	<---	PPFIBP1	-0.16906	0.19937
[15856]	MDM4	<---	CSNK1A1	-0.10359	0.10954
[15857]	PACSIN1	--->	HTT	0.11113	-0.097098
[15858]	WWP1	--->	RASA1	0.012886	-0.002901
[15859]	ADAMTS1	--->	A2M	0.043324	-0.040571
[15860]	TNIP2	<---	NFKB1	-0.77233	0.78038
[15861]	TOLLIP	<---	TOM1L1	-0.045081	0.049305
[15862]	HIPK1	--->	NKX2-5	0.79979	-0.82765
[15863]	CSNK2A1	--->	TP53	1.0002	-0.99962
[15864]	CREBBP	--->	SH3GL1	0.10466	-0.11319
[15865]	PECAM1	<---	CSK	0.015127	0.11748
[15866]	BMPR1B	<---	NKIRAS1	-0.53994	0.52577
[15867]	GABBR2	<---	HTR1A	-0.45788	0.48933
[15868]	RAC1	--->	STAT3	0.54946	-0.59641
[15869]	STAM	<---	JAK3	-1.0043	0.99888
[15870]	DLG3	--->	KCNA4	0.047736	-0.049447
[15871]	PLCG1	<---	RHOA	-0.6651	0.59761
[15872]	RHOXF2	<---	EFEMP2	-0.067922	0.101
[15873]	HNF1A	--->	ALB	0.016842	0.013944
[15874]	NOG	--->	BMP2	0.99953	-1.0058
[15875]	CSNK2A2	--->	CAV1	0.70376	-0.73589
[15876]	PJA1	--->	ZNF440	0.15512	-0.163
[15877]	GNA14	<---	OPRL1	-0.98627	0.9872
[15878]	ZNF174	--->	ZNF20	-0.026316	-0.10962
[15879]	ZMYND11	--->	ETS2	0.2804	-0.2601
[15880]	ATF2	<---	CSNK2A2	-1.0006	1.0089
[15881]	STAP1	<---	TEC	-1.001	1.0024
[15882]	SMURF2	--->	ZBTB44	0.289	-0.29887

```
[15883] GTF2I    --->    USF1    0.58324 -0.54415
[15884] FN1      <---    FSTL3   -0.21547        0.21796
[15885] EGFR     --->    STAT5B  0.99454 -0.99025
[15886] DDX17    <---    HDAC1   -0.042746       0.039624
[15887] ZNF426   <---    CCDC85B -0.21947        0.2144
[15888] CHUK     <---    CHEK1   -1.003  1.0117
[15889] NEK9     <---    RAN     -1.0074 1.0132
[15890] USP33    <---    DDX56   -0.03114        0.046579
[15891] MAP3K7   --->    CHUK    1.0099  -0.99837
[15892] TEX11    <---    DUSP6   -0.29154        0.29549
[15893] EFEMP2   <---    LINGO1  -0.064118       0.06992
[15894] PPARGC1A         --->    NR3C1   0.013278        -0.021031
[15895] TGFBR3   <---    ACVR2A  -0.38596        0.36043
[15896] PRKCA    --->    RHO     0.53258 -0.53729
[15897] EDNRA    --->    EDN3    0.45294 -0.42199
[15898] ID3      --->    TCF3    -0.13552        -0.39218
[15899] OPRK1    --->    GNA15   0.9872  -0.98627
[15900] PLAT     <---    FGA     -0.00063786     0.0043026
[15901] CDC2L2   --->    MYST2   0.36157 -0.38269
[15902] EP300    --->    HAND2   0.19969 -0.20391
[15903] FRS2     <---    GRB2    -0.46631        0.46592
[15904] CDKL1    --->    TGFBR1  0.36359 0.02614
[15905] GDF9     --->    BMPR1A  0.32284 -0.34476
[15906] LRRC7    <---    CAMK2A  -0.05314        0.04106
[15907] FBXW7    --->    PARK2   0.23888 -0.23676
[15908] MAPK8IP2         --->    FGF12   0.066065        -0.051178
[15909] ANGPTL3 --->     ITGB3   0.046421        -0.048962
[15910] NF2      --->    HGS     -0.012869       -0.014772
[15911] SP2      <---    E2F1    -0.10763        0.11252
[15912] DVL2     <---    PPM1A   -0.33505        0.33032
[15913] FYN      --->    NCAM1   0.47822 -0.44392
[15914] ATXN3    <---    TEX11   -0.064059       0.078255
[15915] YWHAE    <---    KCNH2   -0.16536        0.17316
[15916] IQGAP1   --->    CLIP1   0.05034 -0.033628
[15917] MAPKBP1 <---     MAPK9   -0.86422        0.83716
[15918] FLT1     --->    GRB2    0.27934 -0.25133
[15919] ATXN1    <---    ZC3H7B  -0.035472       0.032126
[15920] CPNE2    --->    SKIL    0.48698 -0.48061
[15921] ABL1     <---    RASA1   -0.080986       -0.049696
[15922] DCAF7    <---    DYRK1B  -0.86422        0.83716
[15923] YWHAZ    --->    PFKFB2  0.29699 -0.29134
[15924] UTRN     --->    ACTA1   0.14504 -0.15537
[15925] PBX2     <---    BAIAP2  -0.026846       0.022762
[15926] TP53     <---    HMGB1   -0.42172        0.43281
[15927] TP73     --->    TP53    -0.020978       -0.14021
[15928] G3BP1    <---    RASA1   -0.29317        0.14948
[15929] EPS15L1 <---     EPN1    0.0010124       0.014192
[15930] HRAS     <--->   RAP1B   0.43466 0.43466
[15931] ATXN1    <---    CLEC4F  -0.017774       0.013922
[15932] NR4A2    --->    RXRA    1.003   -0.99549
[15933] CFL1     <---    YWHAG   -0.067982       0.076969
[15934] SEMA4D   <---    PTPRC   -0.43102        0.4347
[15935] GRIA1    <---    GRIK2   -0.19915        -0.0077421
[15936] PTPN3    --->    YWHAB   0.27832 -0.28778
[15937] PITX1    <---    RBPMS   -0.17593        0.18367
[15938] PPP1CA   --->    BCL2    0.29213 -0.30359
[15939] ZNF384   <---    ZYX     -0.20808        0.20561
[15940] MEIS1    <---    HOXD13  -0.0094151      0.024726
[15941] HCK      --->    DOK1    1.3209  -1.1853
[15942] PPEF1    --->    CALM3   -0.12978        -0.15561
[15943] HOMER1   --->    ITPR1   0.19585 -0.20736
[15944] SERPING1         <---    MASP1   -0.042489       0.059553
[15945] LAMC2    --->    FBLN2   0.025488        -0.021277
[15946] TP53BP2 <---     WWP1    -0.18062        0.16813
[15947] JUND     --->    SMAD3   0.057717        -0.035159
[15948] SH3GL3   <--->   SH3GL2  0.0094254       0.0094254
[15949] RAB11FIP3        <---    RAB25   -0.43801        0.45325
[15950] HDAC1    --->    AR      0.9992  -0.99909
[15951] PRKCD    --->    PTPN6   0.54021 -0.53464
[15952] SMURF1   --->    SMAD7   0.9934  -1.0067
[15953] INSR     <---    CALM1   -0.1135 0.10567
[15954] CCR5     --->    PTK2B   0.037413        -0.14397
[15955] BIRC7    --->    CASP9   0.035713        -0.036843
[15956] JAK2     --->    EPOR    0.27406 -0.20849
[15957] ABCA1    --->    TIAM1   0.094901        -0.10671
[15958] ACTR8    <---    BMPR2   -0.33418        0.29939
```

```
[15959] BAT1     --->    STRN4   0.027606        -0.023956
[15960] CDKN1C   --->    CCNE2   0.89594 -0.88886
[15961] ASCC3    --->    TRIP4   0.097971        -0.08304
[15962] KLK3     --->    FN1     0.057559        -0.02883
[15963] APPL1    --->    PIK3CA  0.4648  -0.46471
[15964] PRKCQ    --->    GRM5    0.68403 -0.63679
[15965] FBLN1    --->    FN1     0.044135        -0.014362
[15966] PIK3R3   <---    IGF1R   -0.05496        0.026909
[15967] TRPC3    <---    PLCB3   -0.016685       0.003491
[15968] CDC25C   <---    LCK     -0.48751        0.53444
[15969] RASSF1   --->    STK4    0.99683 -1.0036
[15970] EP300    --->    NCOA3   0.0089653       -0.022438
[15971] TNIP1    <---    MAPK1   -0.4293 0.42383
[15972] AKAP13   --->    PPARA   0.035086        -0.033914
[15973] FXR2     <---    MBIP    -0.032739       0.06675
[15974] KHDRBS1 <---     CBL     -0.20421        0.19331
[15975] RASL12   --->    SMAD1   0.52759 -0.54836
[15976] PRKAG1   <---    PRKAA2  -0.22052        -0.13077
[15977] CEACAM3 <---     CEACAM1 -0.063515       -0.031866
[15978] LRRC7    --->    CDH2    0.026983        -0.036879
[15979] TRRAP    --->    MAX     0.046549        -0.05611
[15980] SKP2     --->    TCF3    0.39566 -0.39825
[15981] JAM2     <---    F2RL2   -0.12281        0.11327
[15982] GRIN2D   <---    NF1     -0.092214       0.080291
[15983] GRIN1    <---    CIT     -0.2802 0.25952
[15984] GABPB1   <---    GABPA   -0.10406        0.12399
[15985] CTNNB1   <---    TGFBR2  -0.39864        0.39449
[15986] MAGI1    --->    LRP2    0.052584        -0.064443
[15987] PRKAA1   --->    GRIK2   0.082786        -0.28167
[15988] ARHGAP15         <---    RAC1    -0.44871        0.45312
[15989] TRPC5    --->    ITPR3   -0.0070644      -0.013012
[15990] SSRP1    --->    SRF     0.031515        -0.024412
[15991] IKBKG    <---    CREBBP  -0.12427        0.11576
[15992] CNKSR2   --->    INADL   0.042791        -0.058542
[15993] SNIP1    --->    MAX     0.40082 -0.38217
[15994] CASP7    --->    PSEN2   0.58173 -0.69269
[15995] NCK1     <---    PDGFRB  -0.060593       0.012153
[15996] KREMEN1 <---     DKK1    -0.013364       0.047551
[15997] BCR      --->    YWHAE   0.078735        -0.083313
[15998] GSC      <---    SMAD1   -0.04291        0.010712
[15999] PRPF40A --->     SNRNP70 0.3333  -0.30347
[16000] MSX1     <---    PIAS1   -0.14749        0.15805
[16001] LY6G6F   <---    GRB2    -0.4617 0.44787
[16002] TP53I3   --->    EEF1G   0.026885        0.0080237
[16003] TEC      --->    WAS     0.4868  -0.53132
[16004] ARHGEF12         <---    PLXNB1  -0.010587       0.0085303
[16005] IPO5     --->    RANBP2  0.0024939       -0.0089922
[16006] UCHL1    <---    HSPA8   -0.77045        0.81269
[16007] GNAS     --->    GCGR    0.4334  -0.43656
[16008] DAXX     --->    SLC2A4  0.049357        -0.081903
[16009] UBE2I    --->    CBX4    0.20784 -0.1743
[16010] GABARAPL2        <---    LRSAM1  -0.11169        0.11548
[16011] CTNND2   <---    PSEN2   -0.065576       0.074964
[16012] SYNCRIP <---     SMN1    0.0023256       0.011379
[16013] ITGA4    --->    VCAN    -0.0051395      -0.0083593
[16014] FLT4     --->    PTPN11  0.20004 -0.068046
[16015] FLI1     ?       ERG     -0.13143        -0.13143
[16016] SFPQ     ?       NONO    -0.016298       -0.016298
[16017] MEF2A    <---    TEAD1   -0.078222       0.078368
[16018] MAPK1    --->    SMAD3   1.012   -1.0074
[16019] YWHAQ    <---    HSPA1A  -0.739  0.77111
[16020] RANBP9   --->    SMAD9   0.18132 -0.19809
[16021] DUSP10   --->    MAPK8   1.0016  -1.0004
[16022] FHL1     --->    RING1   0.041192        -0.0049711
[16023] YWHAG    --->    INPP5E  0.082277        -0.058532
[16024] L1CAM    --->    PEA15   0.17775 -0.18054
[16025] H2AFX    <---    MRE11A  -0.28058        0.33185
[16026] AEBP2    <---    RBBP4   -0.0090258      0.016412
[16027] BMPR1B   --->    DCAF7   0.13865 -0.1649
[16028] ATF4     <---    GPS2    -0.3277 0.33355
[16029] AKT1     --->    PTPN1   0.21859 -0.068869
[16030] E2F6     --->    TFDP2   -0.0088085      -0.064857
[16031] CCR7     --->    CCR10   0.43766 0.24045
[16032] LIN7B    --->    GRIK2   0.11658 -0.098237
[16033] POU1F1   --->    JUN     0.40134 -0.44785
[16034] RAB38    --->    SMAD1   0.52759 -0.54836
```

[16035]	RAF1	--->	BRAF	-0.069957	-0.17467
[16036]	JPH2	--->	CAV3	0.20753	-0.17955
[16037]	EHMT2	<---	LNX1	-0.10363	0.097869
[16038]	NFKB2	<---	REL	-0.013964	-0.0090872
[16039]	SPG20	<---	ZFYVE9	-0.13231	0.12474
[16040]	PLAT	<---	HMGB1	-0.068891	0.072278
[16041]	CASP1	--->	PLA2G4A	0.04868	-0.044028
[16042]	CDH5	<---	JUP	-0.077057	0.07062
[16043]	RBL2	<---	CCND1	-0.28031	0.26997
[16044]	NEDD9	<---	PTK2B	-0.039175	0.0030428
[16045]	E2F6	<---	EZH2	-0.10137	0.13519
[16046]	NOS1	<---	ADRA1B	-0.13558	0.12324
[16047]	SNX6	?	SNX1	-0.040204	-0.040204
[16048]	TCL1B	--->	AKT1	0.99137	-0.99692
[16049]	ABL1	<---	YWHAZ	-0.08167	-0.068919
[16050]	TBP	<---	PIAS3	-0.012093	-0.0010036
[16051]	CDK14	--->	YWHAE	0.62776	-0.66368
[16052]	YWHAB	<---	HSPA1B	-0.739	0.77111
[16053]	THRA	<---	CUL1	-0.10593	0.11411
[16054]	HTR2B	--->	GNAQ	0.9872	-0.98627
[16055]	ILK	--->	CASP9	0.11322	-0.13171
[16056]	SCN4B	<---	SCN2A	-0.025495	0.050124
[16057]	ZRANB2	--->	SNRNP70	0.078392	-0.046406
[16058]	HSH2D	--->	TNK2	0.0040751	-0.019422
[16059]	SOX10	<---	HHEX	-0.060464	0.063322
[16060]	ZNF193	<---	ZNF446	-0.16454	-0.011024
[16061]	ID3	?	MYOD1	-0.65635	-0.65635
[16062]	UBE2L6	<---	TRIM25	-0.0091502	0.0065374
[16063]	BTBD2	<---	BRD7	-0.049016	0.052169
[16064]	CD36	--->	ITGB1	0.12773	-0.13721
[16065]	ACTB	?	ACTC1	-0.33964	-0.33964
[16066]	TF	--->	IGFBP2	0.072099	-0.038154
[16067]	ZFAND6	<---	CDC34	-0.12164	0.13371
[16068]	UBQLN4	--->	CCDC33	0.034731	0.0056646
[16069]	WAC	--->	UBQLN4	0.26122	-0.22895
[16070]	UBE2D2	--->	RNF5	0.12227	-0.11995
[16071]	LAMA1	<---	NT5E	-0.028193	0.03188
[16072]	EDNRB	--->	EDN3	0.45294	-0.42199
[16073]	NR2E3	<---	RBBP4	0.0052452	0.0072573
[16074]	NACC1	<---	HDAC4	-0.060751	0.09376
[16075]	ADRB1	--->	DLG1	0.082793	-0.093156
[16076]	CSNK2A2	<---	PTPRC	-0.35814	0.35828
[16077]	MTOR	--->	RHEB	0.1597	-0.16194
[16078]	MAPK15	--->	TGFB1I1	0.29365	-0.34731
[16079]	CAMK2G	<---	STAT1	-0.60307	0.59503
[16080]	HIF1A	--->	MAPK3	0.84197	-0.84006
[16081]	KHDRBS1	<---	PTK6	-0.99763	0.99942
[16082]	GRB14	<---	PDGFRA	-0.012912	-0.0051675
[16083]	LHX1	<---	RLIM	-0.061039	0.057332
[16084]	FNBP4	--->	SYNJ1	0.32335	-0.29138
[16085]	JAK1	--->	SH2B2	0.30795	-0.25706
[16086]	RPS6KA2	<---	MAP2K1	-1.0338	0.92747
[16087]	CASP8	--->	HIP1	0.57365	-0.71518
[16088]	YWHAZ	--->	IGF1R	0.024667	-0.036569
[16089]	PRKG1	--->	PDE5A	0.66782	-0.68921
[16090]	RASGRP4	--->	RAP1A	0.2121	-0.20949
[16091]	CCL16	<---	CCR1	-0.46475	0.49595
[16092]	CCBP2	--->	CCL21	0.49595	-0.46475
[16093]	PRKCA	--->	CDC42	1.0123	-0.99154
[16094]	HTR1D	--->	HTR1B	0.31807	0.24405
[16095]	LIN7A	--->	GRIN2B	0.068119	-0.074005
[16096]	CTSG	<---	C3	-0.10518	0.11279
[16097]	KIF1C	<---	YWHAZ	-0.040995	0.049737
[16098]	CSNK2A2	--->	CREM	0.96654	-0.95401
[16099]	HDAC4	--->	RBBP4	0.076396	-0.057276
[16100]	AFAP1	--->	ACTC1	0.6814	-0.68593
[16101]	SMAD2	<---	RNF123	-0.23844	0.2829
[16102]	PPBP	<---	CUGBP1	-0.013451	0.045295
[16103]	CLK2	<--->	CLK3	0.23429	0.23429
[16104]	THBS1	<---	F2	-0.18506	0.23708
[16105]	TIMP1	<---	RECQL5	0.0069799	0.0083656
[16106]	DLG4	--->	LRP1	0.051765	-0.064185
[16107]	NEXN	--->	ACTA1	0.58273	-0.58205
[16108]	CD2	<---	PTPRC	-0.27246	0.26433
[16109]	PEBP1	<---	MAP3K7	-0.24545	0.25355
[16110]	FBLN5	<---	LPA	-0.012739	0.016969

```
[16111] SMAD4   <---    SKI     -0.10174        0.14755
[16112] GRIK5   <---    LRSAM1  -0.064674       0.058161
[16113] PIK3R1  --->    RB1     -0.0023346      -0.031638
[16114] RAB4A   --->    PRKCI   0.99149 -1.0135
[16115] ATXN1   <---    DZIP3   -0.054478       0.046778
[16116] LMO1    --->    TAL2    0.62562 -0.58677
[16117] TFRC    <---    TF      -0.0058258      -0.0015825
[16118] HOXA13  --->    MEIS3   0.03787 -0.033829
[16119] NCOR1   --->    BCL6    0.059712        -0.073247
[16120] EDN1    <---    COPS6   -0.21994        0.24623
[16121] ACTL6A  --->    MYC     0.18361 -0.17477
[16122] APAF1   <---    NLRP1   -0.071307       -0.014274
[16123] CD2AP   --->    PSTPIP1 0.10004 0.0089745
[16124] PTPN18  <---    SRC     -0.37818        0.26821
[16125] PDHX    <---    EP300   -0.18656        0.18224
[16126] HTT     <---    EGFR    -0.2938 0.294
[16127] GSK3A   --->    SGK1    0.15763 -0.14926
[16128] PPP1R13B        <---    TP73    -0.05245        0.050275
[16129] FMOD    --->    TGFB2   0.47588 -0.44239
[16130] PYCARD  --->    CASP1   -0.076001       -0.18686
[16131] HDAC4   --->    RFXANK  0.61474 -0.58161
[16132] YWHAG   --->    TP53    0.388   -0.38031
[16133] SRF     --->    ELK1    0.19917 -0.17689
[16134] CD2     --->    CD247   0.11267 -0.12716
[16135] GRIN2D  <---    ABL1    -0.028909       -0.015191
[16136] CABIN1  --->    MEF2D   0.041082        -0.046484
[16137] MAPK8IP3        <---    MAP2K7  -0.16592        0.14982
[16138] WASF3   --->    PIK3R2  0.0050199       -0.054138
[16139] NCOA2   <---    NKX2-1  -0.06802        0.06031
[16140] TLR9    --->    MAPK8IP3        0.038821        -0.049969
[16141] PPFIA1  --->    NCOA2   0.074074        -0.081205
[16142] LTBR    <---    LTB     -0.999  1.0116
[16143] ACP5    <---    MAPK9   -0.63479        0.6091
[16144] ITGB2   <---    ICAM3   -0.059499       0.046792
[16145] CREBBP  --->    PIAS1   0.059178        -0.069359
[16146] CDKN2A  <---    MDM2    -0.1119 0.10528
[16147] MLLT6   <---    MED15   -0.034901       0.036422
[16148] ESR1    <---    ERBB2   -0.17724        0.17
[16149] NEDD4   --->    GRB10   0.044809        -0.06385
[16150] PTK2    <---    EPHA2   -0.086913       0.22346
[16151] RAF1    <---    PRKCD   -0.29448        0.44007
[16152] BIRC6   <---    CASP3   -0.38561        0.31149
[16153] MTNR1A  --->    GNAI1   1.0129  -1.008
[16154] SYNJ2   <---    ITSN2   -0.032377       0.017577
[16155] HIP1    --->    APAF1   0.14243 -0.128
[16156] PTPN22  --->    CD3E    0.56653 -0.59124
[16157] KSR1    --->    MAP2K1  0.29558 -0.15526
[16158] CEACAM8 ?       CEACAM6 -0.0055194      -0.0055194
[16159] RASAL2  --->    YWHAG   0.033651        -0.019932
[16160] MYOD1   <---    CDC34   -0.52948        0.54693
[16161] IRS1    <---    NTRK1   -0.12726        0.11277
[16162] BAX     --->    BCL2    -0.23385        -0.7984
[16163] CREBBP  --->    EBF1    0.18171 -0.18602
[16164] SDCBP2  <---    SDCBP   -0.026072       0.091264
[16165] NEUROD1 <---    PDX1    -0.46425        0.49982
[16166] RAPGEF2 <---    MAGI2   -0.032841       0.11765
[16167] FGFR3   <---    FGF5    -0.61087        0.60861
[16168] LTBR    <---    LTA     -1.0129 1.001
[16169] ACR     --->    SERPINA5        0.11833 -0.082185
[16170] FBXO5   <---    EVI5    0.0059665       0.009262
[16171] PICK1   <---    CIC     -0.15926        0.19512
[16172] PTK2    --->    PIK3R1  0.046022        -0.2003
[16173] PEX14   --->    ZHX1    0.10287 -0.087369
[16174] BMX     --->    STAT3   0.27519 -0.38748
[16175] TNNC1   --->    TNNT1   0.23685 -0.22378
[16176] YWHAG   --->    SNRNP200        0.045156        -0.035857
[16177] LEPR    <---    PIN1    -0.50721        0.5056
[16178] PIK3R1  <---    IFNAR1  -0.052954       -0.022889
[16179] DIP2A   --->    DAB2    0.056387        -0.024219
[16180] KLF12   <---    CTBP1   -0.05097        0.039185
[16181] EED     --->    YY1     0.091635        -0.084095
[16182] WDR44   ?       SEC13   -0.1554 -0.1554
[16183] USH2A   <---    COL4A3  -0.032274       0.022072
[16184] RAPGEF3 --->    MAP1A   0.3413  -0.3475
[16185] RRAS2   --->    MLLT4   0.99712 -0.98672
[16186] FLII    --->    ACTL6A  0.19604 -0.18305
```

[16187]	SRC	--->	DNM1	0.3136	-0.28153
[16188]	CASP10	--->	CASP7	0.99569	-0.9907
[16189]	F10	--->	SERPINB6	0.01376	-0.016469
[16190]	EGFR	--->	ERBB4	0.0082662	0.0070923
[16191]	SMAD9	<---	SNRNP70	-0.30655	0.28646
[16192]	HDAC2	--->	RBBP7	0.11313	-0.098152
[16193]	DOK2	<---	INPP5D	-0.10368	0.094457
[16194]	SYK	<---	CBLB	-1.0008	0.99213
[16195]	CAPN1	--->	F2RL1	0.16203	-0.16251
[16196]	KAT5	--->	CREB1	0.19532	-0.19349
[16197]	PLCG1	<---	KIT	-0.20161	0.13525
[16198]	SHB	--->	PIK3R1	0.18012	-0.28812
[16199]	CSF3R	<---	INPP5D	-0.15435	0.13536
[16200]	GDF11	--->	ACVR2B	1.0057	-0.99943
[16201]	PTK2	<---	DCC	-0.26593	0.11016
[16202]	MEP1A	--->	AGT	0.12254	-0.1041
[16203]	TLK2	--->	YWHAZ	0.62776	-0.66368
[16204]	PRPF40A	--->	YWHAG	0.24806	-0.22585
[16205]	SNX6	--->	PDGFRA	0.11348	-0.11276
[16206]	IKBKB	--->	CTNNB1	0.14295	-0.16439
[16207]	CD22	<---	INPP5D	-0.083122	0.057279
[16208]	SMAD5	<---	SF3B1	-0.34025	0.32211
[16209]	MMP7	<---	DCN	-0.18966	0.20515
[16210]	WWP2	<---	RASA1	-0.034154	0.0026628
[16211]	CDH1	<---	EGFR	-0.13773	0.12871
[16212]	ADRBK1	--->	ADRA2A	0.25048	-0.27132
[16213]	ITPR3	?	ITPR1	-0.0077936	-0.0077936
[16214]	EGFR	--->	PLD2	0.99738	-1.0064
[16215]	RTN4R	<---	NGFR	0.013514	0.17554
[16216]	SRC	--->	ITGB3	0.22832	-0.2137
[16217]	KAT5	<---	CDC42	-0.2174	0.23063
[16218]	DDX5	--->	NCOA1	0.015459	-0.027033
[16219]	DGKH	?	DGKD	-0.0034464	-0.0034464
[16220]	EZH2	--->	SUZ12	0.063759	-0.04715
[16221]	UBQLN1	--->	ASCL1	0.6607	-0.62313
[16222]	ZBTB17	<---	HK3	-0.1666	0.15362
[16223]	RIMS1	<---	UNC13B	-0.010013	0.049217
[16224]	MYB	--->	CEBPB	0.0091118	-0.014408
[16225]	E2F2	--->	RB1	0.47244	-0.47441
[16226]	AKAP6	<---	PRKAR2A	-0.38007	0.41215
[16227]	RGS3	--->	YWHAB	0.15514	-0.15346
[16228]	CTTN	--->	HIP1R	0.04465	-0.0072753
[16229]	CHRDL2	--->	BMP6	0.71403	-0.67452
[16230]	SVIL	--->	ATRX	0.05641	-0.060966
[16231]	SRC	--->	RGS16	0.92473	-0.81283
[16232]	PPP2CB	--->	PRKCB	0.15086	-0.15768
[16233]	MTNR1B	--->	PI4KA	0.10526	-0.1081
[16234]	RANBP2	<---	MDM2	-0.0067123	0.033891
[16235]	F11R	--->	MLLT4	0.092655	-0.083716
[16236]	RAB11FIP2	<---	CXCR2	-0.37356	0.41087
[16237]	STT3A	--->	HJURP	0.026648	0.0075652
[16238]	SH3D19	--->	ADAM15	0.029655	-0.049519
[16239]	NEK9	<--->	NEK6	0.23429	0.23429
[16240]	LYN	<---	JAK2	-0.085727	0.54485
[16241]	HHEX	--->	TLE1	0.05243	-0.021516
[16242]	BRCA1	--->	FHL2	0.021368	-0.0048072
[16243]	CAPN2	--->	CAPNS1	0.034704	0.027859
[16244]	RXRG	<---	NR4A1	-0.99549	1.003
[16245]	SH2B2	<---	JAK2	-0.091396	0.19985
[16246]	FHL2	<---	PSEN2	0.015695	0.019738
[16247]	PRKCD	--->	STAT1	0.55428	-0.55913
[16248]	GSK3B	<---	ILK	-0.99572	0.99289
[16249]	REPS2	--->	RELA	0.1063	-0.10056
[16250]	MAP4K4	--->	RAP2A	0.11593	0.0073108
[16251]	SPI1	--->	MITF	0.3114	-0.29558
[16252]	ATF3	<---	IGSF21	-0.38319	0.35852
[16253]	NR3C2	?	NR3C1	-0.0089162	-0.0089162
[16254]	AKAP11	<---	PRKAR2B	-0.35498	0.38689
[16255]	PLEKHJ1	<---	TGFBR1	-0.34676	0.28679
[16256]	RPH3A	<---	RAB8A	-0.096859	0.11783
[16257]	CSNK2A1	--->	UBE2R2	0.2372	-0.25823
[16258]	CDC25C	--->	CCNA2	0.33489	-0.3246
[16259]	PPP1R8	<---	PPP1CA	-0.21526	0.20815
[16260]	CARD8	<---	NLRP2	-0.14874	0.18479
[16261]	JAM3	--->	JAM2	-0.0049032	-0.015181
[16262]	CASP3	--->	CDH1	0.30281	-0.3521

```
[16263] CRKL    --->    SHC1    0.22879 -0.2424
[16264] MAPK8   --->    SMAD2   1.012   -1.0074
[16265] JAG1    <---    POFUT1  -0.99765        0.99977
[16266] SMAD4   <---    DNAJB2  -0.18867        -0.0017973
[16267] ZBTB16  <---    HEATR2  -0.091824       0.086016
[16268] DSG2    --->    DSC2    -0.018045       -0.093588
[16269] CDC42EP3        <---    RHOQ    -0.87115        0.57849
[16270] TP73    <---    CREBBP  -0.074886       0.06489
[16271] HIST3H3 <---    PIM1    -1.0289 1.0832
[16272] GATA2   <---    MAPK1   -0.99372        1.0021
[16273] CAV1    --->    DNM1    0.091922        -0.10062
[16274] RALBP1  <---    RALA    -0.99572        1.0059
[16275] AR      <---    PXN     -0.57156        0.56532
[16276] CISH    <---    PRKCA   -0.60234        0.60269
[16277] FGR     --->    DOK1    1.3209  -1.1853
[16278] GAB3    <---    PTPN11  -0.46915        0.55498
[16279] PXN     <---    LIMK1   -0.26794        0.082044
[16280] FXR2    --->    $ZBTB8$ 0.14419 -0.13092
[16281] ZFP161  <---    ZNF295  -0.13819        0.0012582
[16282] SETDB1  <---    TOLLIP  -0.07332        0.070337
[16283] RUFY2   <---    EPHA3   -0.25658        0.25702
[16284] STAT5B  <---    ETS1    -0.26685        0.25162
[16285] MKNK2   --->    EIF4E   1.1369  -1.1717
[16286] AHR     --->    RB1     0.20895 -0.21485
[16287] ZNF467  --->    STAT4   0.13529 -0.15449
[16288] FRS2    <---    CBL     -0.5201 0.51452
[16289] TRADD   <---    TNFRSF1A        -1.0095 1.0063
[16290] ADRA1A  --->    CXCR1   0.5907  0.10636
[16291] GLI2    <---    CREB1   -0.092091       0.080845
[16292] NCOA1   <---    CIITA   -0.089957       0.083144
[16293] KSR1    --->    MAPK1   0.19786 -0.090179
[16294] PDLIM1  --->    PITX2   0.12638 -0.12483
[16295] HIST4H4 <---    UBE2I   -0.38612        0.43703
[16296] EIF2AK4 --->    TGFBR1  0.21993 0.010982
[16297] DUSP4   --->    MAPK3   1.0016  -1.0004
[16298] CFL1    --->    ATP1A1  0.067712        -0.07387
[16299] DSG2    --->    DSC1    -0.018045       -0.093588
[16300] CHD3    --->    TP53    0.023267        -0.035138
[16301] ACTR10  --->    ACTA1   -0.065578       -0.55039
[16302] FHL2    --->    TNFRSF11A       0.19213 -0.20036
[16303] ZBTB16  --->    BCL6    -0.010121       -0.08598
[16304] CD79B   ?       CD79A   -0.12533        -0.12533
[16305] SMAD3   --->    GLI3    0.098419        -0.097905
[16306] RCVRN   --->    PQBP1   0.083542        -0.068562
[16307] SMAD4   <---    PIAS2   -1.0027 0.9933
[16308] NCOA2   <---    RXRA    -0.028914       -0.011576
[16309] ACVR1B  <---    INHBB   -0.71391        0.72054
[16310] ACTN4   --->    NOS2    0.16304 -0.17439
[16311] CASP8   --->    PLEC1   0.57989 -0.72138
[16312] TCEB1   --->    USP33   0.1432  -0.11059
[16313] KLF4    <---    HDAC5   -0.099015       0.099657
[16314] DNM1L   <---    MAP3K10 -0.30363        0.29381
[16315] PPP2R1A --->    GRIN1   0.11902 -0.1249
[16316] CREBBP  --->    KLF13   0.071128        -0.081623
[16317] CTSG    --->    SERPINA3        0.11833 -0.082185
[16318] WASF1   --->    ACTR2   0.089124        -0.077881
[16319] MAPK1   --->    TSC2    1.0125  -1.0077
[16320] PRLR    --->    VAV1    -0.0052605      -0.017413
[16321] SMAD2   --->    CTNNB1  0.17916 -0.18358
[16322] EPB41   <---    DRD2    -0.19189        0.19515
[16323] RHOA    <---    HSPA1A  -0.41905        0.45767
[16324] MEFV    <---    PSTPIP1 -0.048723       0.051841
[16325] CBX1    --->    SUV39H1 0.013252        -0.019388
[16326] SELE    --->    CD44    0.034315        -0.041319
[16327] RBBP7   <---    HDAC1   -0.098152       0.11313
[16328] CASP8   --->    CDK11B  0.99111 -0.99197
[16329] YWHAZ   <---    HSPA1A  -0.739  0.77111
[16330] MED15   --->    SMAD3   0.18248 -0.16165
[16331] CTNND1  <---    CTTN    -0.047374       0.045061
[16332] FURIN   <---    EDA     -0.038723       0.029066
[16333] HDAC1   <---    MECOM   -0.28349        0.28508
[16334] ZEB2    --->    SMAD1   -0.0031961      -0.015716
[16335] MYC     <---    GSK3B   -1.0065 1.0154
[16336] DVL1    --->    CSNK2A1 0.99205 -1.0024
[16337] GSK3B   --->    FOXO1   0.32546 -0.33505
[16338] TAF1    --->    RELA    0.0385  -0.045239
```

[16339]	IL31RA	<---	LIFR	-0.17226	0.026948
[16340]	GADD45A	--->	MAP3K4	1.0051	-0.99563
[16341]	COL4A5	--->	MMP9	0.072684	-0.078498
[16342]	DDX54	--->	ESR2	0.042552	-0.032266
[16343]	AFAP1	<---	PRKCB	-1.0819	1.2556
[16344]	EGFR	--->	PLD1	0.42658	-0.43815
[16345]	SFRS1	--->	CD8A	0.059362	-0.037139
[16346]	HSPA8	--->	ABI1	0.50064	-0.5036
[16347]	SOCS1	--->	PIK3R1	0.19561	-0.3011
[16348]	GATA2	<---	HHEX	-0.015702	0.056147
[16349]	IGF1R	<---	CAV1	-0.040129	0.02693
[16350]	TBP	<---	NR3C1	-0.0078454	-0.0047912
[16351]	GAB2	<---	SHC1	-0.10547	0.072259
[16352]	PARK2	<---	UBE2G1	-0.091377	0.12521
[16353]	HIPK2	--->	MDM2	0.071064	-0.09315
[16354]	AKT1	--->	SMAD3	0.26467	-0.21636
[16355]	HIF1A	--->	ARNT	0.30742	-0.0073388
[16356]	CNKSR1	--->	RASSF1	0.13353	-0.12283
[16357]	MAPK8IP3	<---	MAP3K11	-0.13143	0.12071
[16358]	ERBB3	<--->	EGFR	0.017633	0.017633
[16359]	ARHGAP5	<---	RASA1	-0.37557	0.36452
[16360]	VASP	--->	ACTA1	0.9951	-1.0004
[16361]	GIT2	<---	ADRBK1	-0.16512	0.14915
[16362]	CARD6	<---	NOD1	-0.082219	-0.04958
[16363]	PAK1	--->	PLCG1	0.48811	-0.52405
[16364]	CDC25B	<---	CDK1	-0.67632	0.69298
[16365]	BCL10	<---	TRAF2	-0.13252	0.13865
[16366]	SQSTM1	<---	MAP2K5	-0.12906	0.15892
[16367]	KLHL3	<---	SYT2	-0.090545	0.12974
[16368]	IGFBP5	<---	RASSF1	-0.063669	0.080655
[16369]	GZMB	--->	PRKDC	0.0066816	-0.0025076
[16370]	DDX1	--->	CSTF2	0.014281	-0.01143
[16371]	CDH7	?	CDH6	-0.058742	-0.058742
[16372]	ACVR1	<---	UBE2E3	-0.035815	0.018831
[16373]	FYB	<---	LCP2	-0.19484	0.14071
[16374]	FHL1	<---	MYBPC1	-0.063036	0.075077
[16375]	PAK1	--->	FOXO1	0.22002	-0.19059
[16376]	CDK5	--->	DAB1	0.78366	-0.80702
[16377]	FOXO1	--->	ESR1	0.057946	-0.066978
[16378]	DDX1	--->	HNRNPK	0.022319	-0.018577
[16379]	TLE1	<---	FOXG1	-0.43004	0.45973
[16380]	C1S	<---	C1QA	-0.052141	0.04292
[16381]	PTPN2	--->	INSR	0.26902	-0.28101
[16382]	PLD1	<---	ACTA1	-0.26381	0.25267
[16383]	GFI1B	<---	RAB11FIP4	-0.15339	0.14945
[16384]	TP73	--->	MYC	0.078575	-0.078549
[16385]	RAB9A	--->	BCL3	0.38119	-0.36457
[16386]	CDK5	--->	MEF2A	0.35609	-0.36411
[16387]	KLF11	<---	MAPK1	-0.99799	0.99575
[16388]	FAT1	<---	HOMER1	-0.019874	0.00843
[16389]	DRD2	--->	GRIA2	0.37541	-0.3605
[16390]	CDH1	<---	CTNND1	-0.99605	0.98604
[16391]	PRKG1	--->	PLCB3	0.62876	-0.62602
[16392]	ARF3	--->	PICK1	0.45084	-0.42186
[16393]	WEE1	<---	BTRC	-0.38147	0.36207
[16394]	CCND1	<---	CALM1	-0.42212	0.4376
[16395]	TRIM23	--->	GEM	0.63209	-0.63408
[16396]	TAF5	--->	TBP	-0.0042132	-0.008057
[16397]	NFATC3	--->	FOS	0.050576	-0.10687
[16398]	HCK	--->	STAT3	0.99917	-0.99055
[16399]	STAT3	<---	STAT4	-0.24505	-0.080638
[16400]	DCAF7	<---	YWHAG	-0.19355	0.2164
[16401]	CXCR4	<---	JAK2	-0.17451	0.2955
[16402]	INPP5D	<---	CRKL	-0.088536	0.12574
[16403]	MDM4	--->	YWHAG	0.040034	-0.045818
[16404]	ADAM15	<---	NPHP1	-0.063075	0.064826
[16405]	PPP1R15A	<---	PPP1CA	-0.10742	0.098019
[16406]	NAV2	<---	RRAS	-0.13811	0.14062
[16407]	CABIN1	--->	DNM1	0.096958	-0.10429
[16408]	SF3B1	--->	SMAD1	0.32211	-0.34025
[16409]	ITGB5	<---	FHL2	-0.054195	0.051956
[16410]	CRK	--->	FRS2	0.17696	-0.2152
[16411]	BARD1	--->	TP53	0.16786	-0.16512
[16412]	TNFRSF11A	<---	TRAF6	-0.3718	0.35782
[16413]	PRKCQ	--->	IRS1	0.86592	-0.78274
[16414]	RAP1GDS1	<---	RHOA	-0.41126	0.44004

[16415]	EPS8L1	<---	ABI1	-0.10541	0.075646
[16416]	UBASH3B	<---	CBL	-0.010206	0.1804
[16417]	PRKCD	--->	AKT3	0.11908	0.046737
[16418]	SF1	<---	TRIM23	-0.21852	0.21954
[16419]	DOK1	--->	ITGB1	0.12918	-0.13829
[16420]	PCBP2	<---	PRPF40A	-0.3229	0.35515
[16421]	CDK18	--->	CCNK	0.64437	-0.6658
[16422]	FHL2	--->	CTNNB1	0.54507	-0.54377
[16423]	SRC	--->	CTNNB1	0.16647	-0.1713
[16424]	ZNF238	<---	DNMT3B	-0.25966	0.24764
[16425]	LRRC7	--->	CTNND2	0.023227	-0.031438
[16426]	PRKCB	--->	CYTH2	1.2556	-1.0819
[16427]	PTK2B	<---	FGFR3	-0.050984	-0.010244
[16428]	TOM1L1	<---	GRB2	-0.3075	0.30495
[16429]	RBX1	--->	CCND1	0.64387	-0.63044
[16430]	ARHGEF7	<---	NCK1	-0.12702	0.13779
[16431]	MST1R	--->	YWHAB	0.39852	-0.41561
[16432]	MYO7A	<---	MAP2	-0.013203	0.01054
[16433]	LCP2	<---	PTPN6	-1.0045	0.99683
[16434]	HSP90AB1	--->	DDX24	0.037788	-0.045426
[16435]	ATXN1	<---	KAT5	-0.036892	0.029829
[16436]	STAM2	<---	JAK3	-1.0043	0.99888
[16437]	TAF1	--->	JUN	0.17547	-0.20105
[16438]	ASCL2	<---	CALM2	-0.22666	0.2676
[16439]	TAX1BP3	--->	GLS	0.61152	-0.57593
[16440]	SNX13	--->	GNAS	0.58198	-0.58754
[16441]	PELI3	<---	TRAF6	-0.70614	0.69841
[16442]	TRADD	<---	BIRC2	-0.22675	0.1957
[16443]	TRPM2	--->	TRPC6	-0.0064221	-0.024915
[16444]	CDK17	--->	YWHAG	0.62776	-0.66368
[16445]	MAPK3	<---	GAB1	-0.19052	0.17127
[16446]	CCNL2	<---	SFRS7	-1.0044	0.99975
[16447]	MMP7	<---	HAPLN1	-0.093464	0.097177
[16448]	CASP6	--->	RB1	0.41973	-0.45426
[16449]	SNTG1	<---	TGFBR1	-0.35876	0.32567
[16450]	HOMEZ	--->	HOXC8	0.026261	-0.010757
[16451]	RNF7	<---	CUL2	-0.11034	0.12225
[16452]	ADORA2B	<---	DCC	-0.062366	0.052813
[16453]	PDPK1	--->	AKT1	0.99822	-0.99934
[16454]	PAWR	--->	WT1	0.11206	-0.10432
[16455]	HIF1A	--->	SP1	0.18257	-0.19294
[16456]	NFAM1	<---	ZAP70	-0.99528	0.9932
[16457]	RXRG	<---	LRSAM1	-0.092492	0.0376
[16458]	ITGB2	<---	PRTN3	-0.22647	0.21748
[16459]	PTEN	<---	MAST1	-0.61289	0.61732
[16460]	PTPN2	--->	STAT5B	1.0055	-1.005
[16461]	ATXN1	<---	FAT4	-0.010442	0.00051436
[16462]	RNF25	--->	RELA	0.14681	-0.14139
[16463]	UACA	<---	CELSR3	-0.059348	0.051802
[16464]	TNFSF8	--->	TNFRSF8	0.89674	-0.85556
[16465]	PLG	<---	MEP1A	-0.036571	0.041491
[16466]	TLE1	--->	ZFP64	0.16224	-0.15724
[16467]	NR4A3	<---	COPS5	-0.19181	0.21292
[16468]	ZBTB12	<---	RAP1GAP	-0.16871	0.15915
[16469]	KIF1B	--->	DLG1	-0.0064478	-0.013466
[16470]	RPS6KA5	--->	HIST3H2BB	1.009	-0.99586
[16471]	LPHN1	<---	SHANK1	-0.01098	0.00131
[16472]	HIPK2	--->	DAXX	0.3655	-0.48387
[16473]	AKT1	--->	PDK2	0.17464	-0.091743
[16474]	SMAD7	<---	TGFBR1	-1.0087	1.0115
[16475]	CBLB	--->	GRB2	0.57629	-0.58879
[16476]	MAPK1	--->	SMAD1	1.2525	-1.3294
[16477]	DNMT3B	--->	SMARCA5	0.00060167	-0.0038251
[16478]	UBE2I	--->	ARNTL	0.29209	-0.28719
[16479]	SMAD4	<---	RMND5B	-0.34606	0.42901
[16480]	PTPRE	--->	KCNA5	1.2976	-1.2911
[16481]	SORBS2	<---	VCL	-0.10408	0.099887
[16482]	THRA	<---	POU2F1	0.012013	0.012582
[16483]	TRAF2	--->	TNFAIP3	0.043212	-0.04893
[16484]	AKAP9	<---	PRKAR2B	-0.35774	0.38972
[16485]	KIDINS220	<---	NGFR	-0.11055	0.21889
[16486]	EFEMP2	<---	UBQLN1	-0.057902	0.09298
[16487]	PPM1A	--->	SMAD3	0.99247	-0.99033
[16488]	YWHAG	--->	TSC1	0.11987	-0.08934
[16489]	TNFRSF6B	<---	FASLG	-0.52454	0.45861
[16490]	GRB2	--->	PIK3CG	0.075802	-0.076608

[16491]	KIF1B	--->	SNTA1	0.086963	-0.089252
[16492]	IGF1R	--->	RASA1	0.1424	-0.18388
[16493]	CLSTN1	--->	SETDB1	0.070317	-0.075356
[16494]	FOS	<---	JUNB	-0.31886	0.17898
[16495]	PTPRU	--->	GNAO1	0.40483	-0.41277
[16496]	TNFRSF14	<---	BTLA	-0.43397	0.42249
[16497]	VDR	<---	PPP1CA	-0.14546	0.13806
[16498]	MET	--->	HGF	0.2305	-0.24289
[16499]	DNM1	<---	NCK1	-0.13035	0.13129
[16500]	HIST1H2BG	<---	TGM2	-0.19982	0.25116
[16501]	MTNR1B	--->	CTNND1	0.21956	-0.22892
[16502]	RIPK3	--->	RIPK1	0.99901	-1.0116
[16503]	CFTR	<---	PRKAR2A	-0.53689	0.53859
[16504]	FBN1	<---	HSPG2	-0.023366	0.0090566
[16505]	VRK1	--->	TP53	0.64996	-0.64026
[16506]	PPARG	<---	COPS5	-0.12003	0.12735
[16507]	TGFBRAP1	<---	ACVR1B	-0.37522	0.30018
[16508]	SH2B1	<---	GRB2	-0.51768	0.50707
[16509]	FNBP4	--->	KHDRBS1	0.21791	-0.21449
[16510]	DVL2	<---	ZNF250	-0.10766	0.098498
[16511]	CBX1	--->	HIST1H3A	0.24617	-0.19484
[16512]	NCK2	--->	PTK2	0.030428	-0.19662
[16513]	RIPK1	<---	TNFRSF1A	-0.2843	0.15215
[16514]	TRPV4	<---	ITCH	-0.10212	0.089709
[16515]	PRKCH	--->	ITGB2	0.049804	-0.071081
[16516]	HDAC4	--->	MEF2C	0.12446	-0.12664
[16517]	FCHSD2	--->	CASK	0.0094794	-0.022424
[16518]	HDAC1	--->	RBL1	0.16548	-0.17701
[16519]	ITGA2	<---	COL8A1	-0.018968	0.0053855
[16520]	BAX	<---	YWHAQ	-0.61447	0.62138
[16521]	PLCG1	<---	INPP5D	-0.53436	0.36711
[16522]	SIX5	<---	HMGB1	-0.26954	0.30083
[16523]	CENPA	<---	DIAPH1	-0.010828	0.0059164
[16524]	CDK2	--->	TP73	0.45486	-0.4556
[16525]	PPP1R10	<---	PPP1CA	-0.22813	0.22108
[16526]	FNBP4	--->	PLXNA2	0.3096	-0.27692
[16527]	ATXN3	<---	PSMD7	-0.18962	0.20468
[16528]	REPS1	<---	RALA	-0.37232	0.40283
[16529]	RB1	<---	ID2	-0.35069	0.35001
[16530]	AKAP11	<---	PRKAR1A	-0.35498	0.38689
[16531]	PABPC1	--->	EIF4B	-0.0035406	-0.13445
[16532]	TGFB1	<---	FNTA	-0.43156	0.27909
[16533]	KLK2	--->	SERPINF2	0.11833	-0.082185
[16534]	PLAG1	<---	KPNA2	-0.071921	0.08904
[16535]	DLGAP1	--->	KCNA4	0.042047	-0.0080297
[16536]	SNRK	<---	HSPA1A	-0.99901	1.0063
[16537]	TP53	<---	CDC25C	-0.18195	0.18798
[16538]	YWHAZ	--->	FOXO4	1.0083	-1.0122
[16539]	GATA4	<---	EP300	-0.19036	0.18629
[16540]	GAB2	<---	SYK	-0.98622	1.0021
[16541]	TERT	<---	HSP90AA1	-0.020575	0.007382
[16542]	FAS	--->	EZR	0.095211	-0.03575
[16543]	SUV39H1	--->	HIST3H3	0.043597	-0.029918
[16544]	GAPDH	<---	CAMK2B	-0.35111	0.33355
[16545]	CPSF6	--->	UBAC1	0.15666	-0.11813
[16546]	RBM41	?	RALYL	-0.016298	-0.016298
[16547]	HRAS	<---	AGTR1	-0.17937	0.19171
[16548]	CD93	<---	COL4A2	-0.031485	0.019975
[16549]	GTF2I	--->	STAT3	0.55734	-0.60973
[16550]	WWP1	--->	CLCN5	0.22331	-0.23464
[16551]	PDZD3	<---	SLCO1C1	-0.038024	0.07475
[16552]	CDK11B	--->	YWHAE	0.62776	-0.66368
[16553]	SELP	--->	EZR	0.0054266	-0.0018855
[16554]	SSRP1	<---	RAN	-0.11813	0.13065
[16555]	FOXA2	--->	TLE1	0.45973	-0.43004
[16556]	RLF	<---	PRPF40A	-0.25433	0.27005
[16557]	CDON	--->	CTNNB1	0.2065	-0.21531
[16558]	MAPKAPK3	--->	HSPB1	1.0388	-0.99926
[16559]	CHUK	<---	MAP3K8	-1.003	1.0117
[16560]	SUV39H1	--->	RBL1	0.093015	-0.10523
[16561]	RASA3	<---	FES	-0.10261	0.18644
[16562]	CCR5	--->	CCL2	0.49595	-0.46475
[16563]	RAC1	--->	CAV1	0.40294	-0.37866
[16564]	ATN1	--->	ZMYND8	0.030832	-0.033133
[16565]	CEBPB	<---	FOXO1	-0.10797	0.10189
[16566]	CDKN1B	<---	KPNA5	-0.011578	0.014967

[16567]	BCL10	--->	IKBKG	0.40581	-0.39725
[16568]	RPS6KA5	--->	ETV1	0.39908	-0.61069
[16569]	EP300	--->	STAT6	0.99741	-1.0041
[16570]	ARHGAP10	--->	PKN1	-0.029107	-0.044734
[16571]	GNA12	<---	PPP5C	-0.0029914	-0.00080903
[16572]	NRG3	--->	ERBB4	1.0123	-0.99547
[16573]	MAPK1	--->	ERF	1.1285	-1.1212
[16574]	GRASP	--->	GRM3	0.0626	-0.025484
[16575]	ABLIM1	--->	BEGAIN	0.031376	-0.015325
[16576]	DRD2	--->	CADPS	0.031808	-0.041843
[16577]	PRKACA	--->	GJA5	0.53362	-0.54829
[16578]	KLKB1	--->	IGFBP3	0.070438	-0.036479
[16579]	FNBP1	--->	SNX2	0.13076	-0.11664
[16580]	BUB1	--->	EIF4EBP1	1.0012	-1.0031
[16581]	LAT	<---	FCGR2A	-0.28312	0.31893
[16582]	CDC42	--->	MYO9A	0.49518	-0.49774
[16583]	CDK11B	--->	CCND3	1.0544	-1.0774
[16584]	PCDHA4	<---	RELN	-0.087774	0.10343
[16585]	YWHAG	--->	PUF60	0.092231	-0.070624
[16586]	PGR	<---	RBM23	-0.040633	0.064959
[16587]	TESK2	--->	DSTN	0.43358	-0.44548
[16588]	ARNT	<---	SMAD9	-0.37718	0.36118
[16589]	GPSM2	<---	GNAI2	-0.20473	0.22114
[16590]	PTPN5	--->	FYN	-0.065476	-0.14195
[16591]	PITX2	<---	HDAC1	-0.12747	0.12789
[16592]	NFKBIA	--->	REL	0.080231	-0.091899
[16593]	ZSCAN4	--->	SMAD1	-0.0031961	-0.015716
[16594]	RARG	<---	NR0B2	-0.028437	-0.0094276
[16595]	CASP6	--->	PRKCZ	0.19066	-0.19986
[16596]	HCK	--->	PLCG1	0.81503	-1.0097
[16597]	CRK	<---	BUB1	-0.30256	0.206
[16598]	GRASP	--->	GRM1	0.0626	-0.025484
[16599]	TTF2	--->	HNRNPA1	0.0061326	-0.0033008
[16600]	TAF15	--->	SF1	0.053517	-0.050464
[16601]	TRIM37	--->	TRAF3	0.00096766	-0.051516
[16602]	PTPN11	--->	PECAM1	0.26319	-0.077948
[16603]	SFRS11	?	SFRS2	-0.016298	-0.016298
[16604]	BCL2L10	--->	BCL2L1	-0.23385	-0.7984
[16605]	HDAC3	--->	SUV39H1	0.052403	-0.045047
[16606]	RAF1	--->	NR3C1	0.02521	-0.042304
[16607]	MARK3	--->	PKP2	0.052067	-0.066617
[16608]	UBE2L3	<---	CBL	-0.47935	0.47348
[16609]	KRT15	<---	ABLIM1	-0.018124	0.022471
[16610]	U2AF2	<---	CPSF7	-0.032449	0.0088798
[16611]	MAG	--->	FN1	0.19573	-0.20467
[16612]	ELANE	<---	F2R	-0.42368	0.45462
[16613]	KHDRBS1	<---	RASA1	-0.13465	0.073127
[16614]	MAPKAPK5	--->	ATF2	0.60506	-0.62243
[16615]	CSNK1A1	--->	OCLN	0.57809	-0.49833
[16616]	MAPK14	--->	RPS6KA4	1.0109	-0.99775
[16617]	SFRS7	<---	CDC2L2	-0.40469	0.38252
[16618]	PLD2	<---	CAV1	-0.46518	0.46168
[16619]	SIRPA	--->	NOL3	0.043617	-0.01909
[16620]	SMAD1	--->	ZNF8	0.1194	-0.13537
[16621]	MYBL2	--->	CCNE1	0.2333	-0.23718
[16622]	IL3RA	<---	CSF2	-0.23688	0.26063
[16623]	SRC	--->	SHC1	0.5117	-0.43547
[16624]	SRCAP	<---	CREBBP	-0.047004	0.035201
[16625]	PRKCE	--->	KRT1	0.27815	-0.30092
[16626]	CASP14	<---	CASP8	-0.072167	0.41659
[16627]	CDK2	--->	CDX2	0.79979	-0.82765
[16628]	GRIN2D	<---	HRAS	-0.19864	0.19661
[16629]	GRB7	<---	KIT	-0.6082	0.60466
[16630]	HIST1H2BD	<---	HIRA	-0.057829	0.10803
[16631]	REL	<---	TNIP2	-0.16734	0.15782
[16632]	STAT1	--->	TNFRSF1B	0.054258	-0.11899
[16633]	SGK1	--->	FOXO3	0.46293	-0.56866
[16634]	MAP3K5	<---	PDCD6	-0.36981	0.62386
[16635]	PICK1	<---	PRKCA	-0.98595	0.99063
[16636]	RUFY1	<---	RAB4A	-0.3573	0.3878
[16637]	TP53	--->	DVL2	0.089168	-0.096909
[16638]	MAPK3	--->	GMFB	0.75717	-0.78322
[16639]	ACVR1	<---	RHEBL1	-0.32935	0.31819
[16640]	AP3B2	<---	CSNK1A1	-0.49706	0.57673
[16641]	E2F1	--->	CCNA2	0.37157	-0.36974
[16642]	MAP3K3	--->	ZBTB16	0.17154	-0.12661

[16643]	GNAS	<---	ADORA1	-1.008	1.0129
[16644]	NEDD9	--->	SH2D3C	0.053649	-0.07171
[16645]	NCOA1	<---	PPARA	-0.043163	0.034596
[16646]	HDAC9	--->	BCL6	0.3836	-0.39654
[16647]	SYK	<---	FGR	-1.0044	1.0025
[16648]	ASAP2	--->	PXN	0.041833	-0.053739
[16649]	IRAK4	--->	IRAK1	1.0149	-1.0046
[16650]	LPXN	<---	PTK2B	-0.4799	0.41189
[16651]	SORBS1	--->	PXN	0.20829	-0.20912
[16652]	IL1RAP	--->	PIK3R1	0.25311	-0.31923
[16653]	MBD1	<---	PIAS1	-0.082232	0.084046
[16654]	PIN1	--->	SOCS3	0.14202	-0.12128
[16655]	RXFP4	--->	RLN3	0.40768	-0.37669
[16656]	BUB3	<---	HDAC2	-0.098152	0.11313
[16657]	PRKCA	--->	GRIA4	0.56969	-0.57379
[16658]	KCNJ1	<---	SLC9A3R2	-0.0070376	0.044422
[16659]	HRAS	--->	PIK3CA	0.98913	-1.0082
[16660]	STRAP	<---	TGFBR2	-0.87271	0.93273
[16661]	KALRN	--->	PAM	0.053723	-0.067915
[16662]	PIK3R1	--->	PIK3CA	0.0096742	-0.034565
[16663]	EP300	--->	ABL1	0.0167	-0.034052
[16664]	CSK	--->	CD44	0.26037	-0.23486
[16665]	HSPG2	--->	APP	0.024599	-0.038277
[16666]	SQSTM1	<---	NTRK2	-0.084966	0.077388
[16667]	CCL25	<---	CCRL1	-0.46475	0.49595
[16668]	LRP2	<---	SLC9A3	-0.14828	0.13929
[16669]	RXRA	<---	UBQLN4	-0.27421	0.064146
[16670]	CSNK2A2	--->	TGFBR1	0.36359	0.02614
[16671]	MSC	--->	TCF4	-0.054725	-0.37599
[16672]	WASL	<---	NCK1	-0.2904	0.26053
[16673]	TP53	<---	HDAC2	-0.22533	0.22693
[16674]	ABL1	<---	ZAP70	-0.35427	-0.22276
[16675]	ELK1	<---	UBE2I	-0.34372	0.38313
[16676]	STK16	--->	ELK1	1.0428	-0.98814
[16677]	ZNF587	<---	SMAD9	-0.13537	0.1194
[16678]	GRB10	--->	BCR	0.022162	0.00013004
[16679]	GABBR2	--->	ATF4	0.32597	-0.31999
[16680]	IGFALS	--->	IGFBP3	0.10584	-0.093579
[16681]	MAP3K8	<---	NFKB1	-0.6564	0.6581
[16682]	RIT1	--->	RLF	0.31566	-0.30104
[16683]	IKBKE	--->	NFKBIA	1.0116	-0.99754
[16684]	CSRP3	--->	ACTN1	0.014972	-0.010035
[16685]	TFRC	<---	RAB5B	-0.058114	0.050236
[16686]	LCK	--->	MUC1	1.0056	-1.0032
[16687]	MDM4	--->	YWHAE	0.040034	-0.045818
[16688]	SIGIRR	<---	IL1R1	0.0012543	0.15175
[16689]	CD3E	<---	NCL	-0.0095263	0.0084695
[16690]	RAD54B	--->	LNX1	0.0035116	0.0015118
[16691]	TRPC1	<---	GRIA1	-0.016705	0.0034749
[16692]	TTF2	<---	CDC5L	0.00167	0.0020334
[16693]	ELANE	--->	CSF3R	0.24103	-0.22826
[16694]	MOBKL3	<---	EGLN2	-0.031377	0.045066
[16695]	PCGF2	<--->	RNF2	0.003228	0.003228
[16696]	REPS2	<---	CDK5RAP2	0.012308	0.021687
[16697]	CNGA2	--->	CALM1	0.42161	-0.40567
[16698]	POU2F1	--->	ESR1	0.012582	0.012013
[16699]	BCAR1	<---	VPS11	-0.0054812	0.001534
[16700]	PPAP2A	<---	FXYD1	0.018715	0.019646
[16701]	UPF2	<---	EIF4G1	-0.079058	0.077209
[16702]	NR1I2	<---	POU1F1	0.012013	0.012582
[16703]	SRC	--->	PRKD1	0.11747	-0.11367
[16704]	IQGAP2	<---	RAC1	-0.13647	0.1548
[16705]	MAPK1	--->	RB1	0.98937	-1.0015
[16706]	ZAP70	--->	WIPF1	0.20697	-0.21167
[16707]	CTNNA1	--->	CDH15	0.4013	-0.39209
[16708]	YWHAG	--->	BAIAP2	0.020651	-0.0213
[16709]	CARD11	<---	BCL10	-0.1239	-0.022282
[16710]	MATN2	<---	COL4A2	-0.023075	0.01173
[16711]	ARHGEF11	<---	PLXNB1	-0.010587	0.0085303
[16712]	USP7	<---	SYVN1	-0.016475	0.032937
[16713]	NDOR1	<---	DLG4	-0.11701	0.10585
[16714]	CYTH2	<--->	IPCEF1	0.28269	0.28269
[16715]	RAC1	--->	PIK3R1	0.21	-0.30832
[16716]	SLC22A4	<---	PDZK1	-0.34102	0.37001
[16717]	SMAD3	<---	RBX1	-0.33195	0.41487
[16718]	JAK1	<---	PTPN6	-0.99645	1.0117

```
[16719] RAPSN   <---    GRB2    -0.18398        0.19602
[16720] SP1     --->    NR5A1   0.10251 -0.11174
[16721] CPNE1   <---    PDCD6   -0.22356        0.24347
[16722] TXNIP   <---    HDAC1   -0.011412       0.015423
[16723] MAPK9   --->    WDR62   0.83716 -0.86422
[16724] ADAMTS5 --->    MMP13   0.069458        -0.071046
[16725] KLKB1   --->    F12     -0.0039613      -0.0063334
[16726] UBE2I   --->    ARNT    0.29209 -0.28719
[16727] MERTK   --->    TNK2    -0.0050621      -0.012123
[16728] AKT1    <---    RAC1    0.060359        0.09142
[16729] RGS2    --->    GNA15   1.0012  -1.0015
[16730] CASP4   --->    PLA2G4A 0.04868 -0.044028
[16731] KCNJ2   <---    INADL   -0.0070376      0.044422
[16732] MAP3K3  --->    HSP90AB1        0.093507        -0.04884
[16733] TLN1    --->    ITGA2B  1.0088  -0.99292
[16734] GFRA3   --->    ARTN    0.39482 -0.35487
[16735] ATF1    --->    CREM    -0.040607       -0.17244
[16736] FBXO25  <---    SKP1    -0.15846        0.15247
[16737] IQGAP2  --->    ACTR2   0.18802 -0.1739
[16738] INPP4A  <---    CAPN1   -0.22947        0.22942
[16739] ERBB3   <---    RASA4   -0.04787        0.039445
[16740] DAXX    <---    ATRX    -0.016218       -0.0047309
[16741] MMP26   <---    IGFBP1  -0.03539        0.071188
[16742] ATF6    <---    YY1     -0.024629       0.041704
[16743] ITGA5   <---    COL18A1 -0.012383       -0.00065137
[16744] MYC     --->    RB1     0.10943 -0.11731
[16745] ESRRG   --->    CALM1   0.15612 -0.12827
[16746] SOX10   <---    DLX5    -0.060464       0.063322
[16747] YWHAZ   <---    RAF1    -0.051212       0.022119
[16748] RAB27A  --->    UNC13D  0.20092 -0.15731
[16749] UBE2N   <--->   UBE2V1  0.21837 0.21837
[16750] STYXL1  --->    TGFBR1  0.052632        -0.084844
[16751] SYTL2   <---    RAB27A  -0.10459        0.12562
[16752] SFRS4   <---    FUS     -0.02338        -0.0017915
[16753] RBPMS   ?       RBM9    -0.016298       -0.016298
[16754] MAP3K5  --->    CDC25A  0.25715 0.0031037
[16755] CNOT4   <---    UBE2B   -0.1727 0.20587
[16756] CD247   <---    PTPN3   -0.99565        0.99129
[16757] MAPK3   --->    PLCB1   1.0018  -1.001
[16758] CCDC85B --->    FAM13C  0.056721        -0.02845
[16759] HNRNPUL1        <---    PRMT2   -0.07839        0.081435
[16760] TNFRSF10B       --->    TNFRSF10D       0.24159 -0.034841
[16761] FYB     <---    VASP    -0.039177       0.031117
[16762] CALM1   --->    CRHR1   0.25258 -0.24765
[16763] PPID    <---    HSP90AA1        -0.062795       0.055103
[16764] RNF2    <---    SMURF2  -0.71117        0.70747
[16765] UXS1    <---    AKT1    -0.30732        0.45418
[16766] RGS1    --->    GIPC1   0.44344 -0.41116
[16767] MAPK8   --->    RPS6KB1 1.002   -1.0109
[16768] RTDR1   --->    SMAD4   0.42859 -0.34537
[16769] TLR4    <---    CD14    -0.99822        0.99661
[16770] FGFR1   <---    FGF9    -0.99871        1.0053
[16771] BRD7    --->    HIST1H2AL       0.29322 -0.24229
[16772] CARD8   --->    CASP1   -0.076001       -0.18686
[16773] CHEK2   --->    KPNA2   0.45358 -0.47583
[16774] GATA4   --->    HAND2   0.46907 -0.42316
[16775] COPS6   --->    ORAI2   0.48805 -0.45484
[16776] BID     --->    BCL2    0.72799 -0.71278
[16777] HSPA8   --->    BAG1    0.96516 -0.92976
[16778] DLK1    <---    GAS1    -0.0089901      0.0018112
[16779] HSPA8   --->    GAK     0.90477 -0.90693
[16780] BAD     --->    BCL2L2  1.0001  -0.99613
[16781] CDK7    <--->   CDK2    0.23429 0.23429
[16782] ACTN4   <---    NEB     -0.052241       0.056099
[16783] RPS6KC1 --->    SPHK1   0.24177 -0.26013
[16784] LYN     --->    DOK2    1.3209  -1.1853
[16785] ACTR1B  --->    ACTA1   -0.065578       -0.55039
[16786] DLG3    --->    GRIN2C  0.044742        -0.055228
[16787] PPIH    --->    PRPF4   0.13097 -0.096121
[16788] IKBKG   <---    TNFRSF1A        -0.30588        0.38193
[16789] FGFR4   <---    FGF7    -0.61054        0.60904
[16790] RAP1GAP --->    RALGDS  0.19036 -0.20125
[16791] HIPK2   <---    BTRC    -0.38147        0.36207
[16792] SHANK2  --->    BAI2    0.067053        -0.07414
[16793] PTPN11  --->    LIFR    0.99881 -0.85824
[16794] ATF5    <---    CEBPG   -0.17879        0.22169
```

[16795]	NISCH	--->	IRS4	0.045298	-0.046338
[16796]	MAPK14	--->	EIF4EBP1	0.40574	-0.56693
[16797]	HMGB1	--->	HOXD9	0.30083	-0.26954
[16798]	SUFU	<---	HNRNPA1	-0.27719	0.2963
[16799]	TRIM2	--->	MYO5A	0.069004	-0.065506
[16800]	HIPK2	--->	PTCH1	0.50317	-0.51297
[16801]	DGKZ	<---	SNTA1	0.0034359	0.0034744
[16802]	PLD2	<---	CSK	-0.29631	0.30015
[16803]	NOTCH1	<---	POFUT1	-1.0101	0.99698
[16804]	ESR1	--->	CALM1	0.15612	-0.12827
[16805]	KSR2	<---	MAPK3	-0.045194	0.056629
[16806]	MAPK14	--->	SCN8A	0.30973	-0.47974
[16807]	MAP3K2	--->	YWHAG	0.54958	-0.30237
[16808]	ARNTL2	?	CLOCK	-0.019553	-0.019553
[16809]	PTK2B	<---	CBL	-0.35239	0.32679
[16810]	PKP1	<---	KRT14	-0.041253	0.050704
[16811]	EPS15	<---	ITSN1	-0.025597	-0.0056558
[16812]	CALCOCO2	<---	LNX1	-0.0089375	0.02726
[16813]	YWHAZ	--->	TP53	0.388	-0.38031
[16814]	RGS7	--->	GNAI3	0.60077	-0.60607
[16815]	GRK6	--->	PRSS23	0.48181	-0.56864
[16816]	PARD6A	<---	RAC1	-0.17886	0.17837
[16817]	GRM7	<---	GNAQ	-0.19572	0.21162
[16818]	HDAC2	<---	SP1	-0.094236	0.082752
[16819]	ADAM17	<---	ERBB4	-0.058927	0.048672
[16820]	EZH2	<---	NINL	-0.27917	0.315
[16821]	GNB5	<---	RGS7	-0.33708	0.34928
[16822]	APBB3	--->	APLP2	0.063813	-0.073499
[16823]	SP1	--->	RXRA	0.059448	-0.11968
[16824]	HDAC4	<---	CIITA	-0.033526	0.072376
[16825]	GADD45G	<---	CDKN1A	-0.0109	0.021707
[16826]	IRS1	--->	CAV1	0.1012	-0.093306
[16827]	SLC9A3R2	--->	SLC9A3	0.065872	-0.059787
[16828]	ITSN1	--->	HRAS	0.0049639	-0.0061254
[16829]	NCAN	<---	CNTN2	-0.016308	0.0030879
[16830]	GFI1B	<---	MYH14	-0.026735	0.014139
[16831]	ATN1	--->	NELL2	0.024909	-0.028854
[16832]	SALL1	<---	TERF1	-0.29039	0.29126
[16833]	DLGAP1	--->	GRIN2C	0.11143	-0.11268
[16834]	PIK3R1	<---	CHRNA7	-0.28174	0.17689
[16835]	KCNA4	<---	CASK	-0.14695	0.13191
[16836]	RAF1	--->	BCL2	0.27923	-0.30301
[16837]	CBLB	--->	SH3KBP1	0.48074	-0.48677
[16838]	ACTG1	<---	PRSS23	-0.53	0.53275
[16839]	HCK	--->	WAS	0.99511	-1.0011
[16840]	ACP1	--->	SFMBT1	0.19329	-0.17541
[16841]	RFWD2	<---	SIPA1	-0.15204	0.14174
[16842]	RB1	<---	SPI1	-0.43615	0.43649
[16843]	SRC	--->	VCL	0.88389	-0.77281
[16844]	DLG1	--->	GRIA1	0.12481	-0.13367
[16845]	UBE2I	--->	SMAD4	0.76473	-0.70088
[16846]	CDK4	--->	RB1	0.98937	-1.0015
[16847]	ARHGAP1	--->	ITPR1	0.1957	-0.20721
[16848]	GC	<---	C3	-0.16613	0.17297
[16849]	WASF1	<---	PFN1	-0.033541	0.046449
[16850]	FURIN	--->	NOTCH1	0.99505	-1.0035
[16851]	ITGB6	<---	FN1	-0.0762	0.069239
[16852]	RASIP1	<---	RRAS2	-0.64033	0.64447
[16853]	BCAN	--->	MMP8	0.022421	-0.030194
[16854]	NCOA1	<---	ESR1	-0.043163	0.034596
[16855]	PTPN11	--->	EPOR	0.60329	-0.52352
[16856]	NOTCH1	--->	RBPJ	0.60413	-0.6021
[16857]	RAF1	--->	BAD	1.0053	-0.99427
[16858]	PLEKHO1	<---	SMURF1	-0.67813	0.67205
[16859]	LTB4R	--->	GNA15	1.0129	-1.008
[16860]	MAP3K7	--->	TGFBR1	0.13905	-0.15486
[16861]	PDIK1L	<---	TNF	-0.059898	0.062342
[16862]	TBP	<---	ESR1	-0.0078454	-0.0047912
[16863]	RARB	--->	RXRG	1.003	-0.99549
[16864]	JAK1	--->	PDGFRA	0.025864	-0.03096
[16865]	SLK	<---	CASP3	-0.98085	0.87492
[16866]	GSK3B	--->	MAPT	0.99929	-1.0027
[16867]	NR1H2	<---	FOXO3	-0.26658	0.26449
[16868]	CDK9	<---	HSPA1A	-0.99901	1.0063
[16869]	PRKAR1A	<---	GRB2	-0.067546	0.07917
[16870]	TRAF2	--->	SETD5	0.12445	-0.1183

```
[16871] OCRL    <---    CDC42   -0.25397        0.25628
[16872] PTPN1   --->    CAPN2   0.23046 -0.23518
[16873] LHX9    <---    SMAD2   -0.083819       0.1153
[16874] GNA13   <---    S1PR4   -1.008  1.0129
[16875] FER     <---    JAK1    -0.60754        0.6002
[16876] MMP13   <---    CCL7    -0.16713        0.18806
[16877] WASL    <---    VASP    -0.073329       -0.014273
[16878] SNX27   --->    DGKZ    0.009322        -0.018742
[16879] RGS19   <---    TGFBR1  -0.28719        0.25134
[16880] TP73    <---    HMGB1   -0.12968        0.1468
[16881] FCGR1A  <--->   FCGR3A  0.45908 0.45908
[16882] EED     <---    PPP1R8  -0.11881        0.15155
[16883] CDX4    ?       HOXA5   -0.11417        -0.11417
[16884] FXYD1   <---    PRKACA  -1.0061 1.0012
[16885] PAK1    <---    PRKCD   0.086409        0.32578
[16886] KHDRBS2 <---    PRMT1   -0.146  0.13822
[16887] CSNK1A1 --->    PSEN2   0.56845 -0.48873
[16888] PPP3CB  --->    SLC8A2  0.10266 -0.11189
[16889] MAP4K2  --->    MAP3K11 0.046903        0.012956
[16890] PKNOX1  ?       MEIS1   -0.025279       -0.025279
[16891] EGFR    <---    ADRBK1  -0.088489       0.072039
[16892] SMARCA5 <---    SATB1   -0.0076601      -0.0021804
[16893] HDAC3   --->    TBL1X   0.016523        -0.019719
[16894] TGFBR1  --->    TRAP1   0.34066 -0.37461
[16895] JDP2    --->    ATF2    -0.036813       -0.19685
[16896] DUSP22  --->    MAPK14  0.99677 -1.0053
[16897] DEF6    <---    RHOA    -0.26331        0.26775
[16898] TNFRSF8 <---    ALK     -0.63053        0.61325
[16899] SMAD4   <---    JUNB    -0.035159       0.057717
[16900] MBD3    <---    RBBP4   0.009301        0.010856
[16901] CSNK2A2 --->    CABP1   1.007   -1.0109
[16902] CD3E    <---    NCK2    -0.31417        0.29617
[16903] TJP1    --->    TRPC4   0.023578        -0.036707
[16904] SP3     --->    RB1     0.24307 -0.24871
[16905] NRXN1   <---    SYT1    -0.043035       0.083467
[16906] ARFRP1  --->    CYTH1   0.20203 -0.18769
[16907] KANK1   <---    PPP1CA  -0.34565        0.33834
[16908] SHC1    <---    CBL     -0.15929        0.12586
[16909] SPTA1   --->    ACTA1   0.21933 -0.23217
[16910] FSHR    --->    ZBTB16  0.091712        -0.099463
[16911] PPP1R9A <---    PPP1CA  -0.10771        0.10247
[16912] ATXN1   <---    RAPGEF4 -0.045273       0.032569
[16913] TSHR    --->    GNA11   0.65587 -0.65379
[16914] HDLBP   <---    DHX9    -0.013705       0.029095
[16915] CDK19   --->    EIF3F   0.46374 -0.49734
[16916] RARA    <---    UBE3A   -0.17478        0.17602
[16917] MOBKL1B <---    CCDC85B -0.20643        0.23334
[16918] AKT1    --->    STAT1   1.013   -1.0034
[16919] CDKN3   <---    CDK1    -0.56626        0.54604
[16920] CDK2    <--->   CDC7    0.23429 0.23429
[16921] RERE    --->    MYST3   0.063647        -0.042673
[16922] JAK2    --->    FYN     0.54485 -0.085727
[16923] EPS15   <---    CRK     -0.041512       0.027234
[16924] HDAC4   <---    NCOR2   -0.22984        0.22101
[16925] ATXN1   <---    VSTM2L  -0.022333       0.016895
[16926] OPTN    <---    RAB8A   -0.51762        0.54384
[16927] BCAR1   --->    PIK3R1  0.15655 -0.071429
[16928] TRAF2   --->    HINFP   0.10832 -0.10968
[16929] GRB10   <---    KIT     -0.6082 0.60466
[16930] EPB41L2 --->    GRIA1   0.11545 -0.12348
[16931] CSNK2A2 --->    TCF7L2  1.0076  -0.99098
[16932] KCNJ9   <---    DRD2    -0.43943        0.47115
[16933] TGFB1   <---    COL2A1  -0.1037 0.070136
[16934] CDC42BPA        --->    LIMK1   0.011701        -0.014076
[16935] FBXW7   --->    JUN     0.32482 -0.3514
[16936] CCRL1   --->    CXCL13  0.49595 -0.46475
[16937] SAG     <---    ADRA1B  -0.2009 0.19779
[16938] E2F3    --->    TFE3    0.39997 -0.38788
[16939] ISL1    --->    NR2F1   0.013803        -0.0038765
[16940] ATXN1   <---    MTERFD2 -0.019393       0.012933
[16941] PPP1CA  --->    BAD     0.31263 -0.32754
[16942] LRP1    <---    WNT3A   -0.2095 0.20143
[16943] TRPC3   <---    GNA11   -0.16038        0.14911
[16944] MAP3K14 <---    RIPK1   -0.99512        1.0097
[16945] PRKCE   --->    RAF1    0.63927 -0.76202
[16946] BRD4    <---    CHFR    -0.040258       0.04326
```

[16947]	CD36	<---	COL1A1	-0.041822	0.033591
[16948]	INSR	--->	STAT5A	0.13049	-0.14657
[16949]	ASAP2	<---	ARF5	-0.13844	0.12847
[16950]	RGS14	--->	GNAO1	0.013429	-0.011715
[16951]	BCOR	<---	HDAC6	-0.19527	0.19869
[16952]	STAT5A	<---	EPOR	-0.29551	0.2772
[16953]	IGSF1	--->	IGF1	0.13539	-0.127
[16954]	SERPINF2	<---	PLAU	-0.040728	0.057207
[16955]	RGS1	<---	MAP3K12	-0.39617	0.39659
[16956]	VPREB1	?	VPREB3	-0.077629	-0.077629
[16957]	IKBKB	--->	YWHAB	0.35528	-0.56614
[16958]	SMARCE1	<---	SRC	-0.57475	0.63145
[16959]	RBM39	--->	ESR1	0.16436	-0.12846
[16960]	RPS6KB1	--->	TRAF4	0.088757	-0.16797
[16961]	TRAF2	<---	BIRC2	-0.1025	0.095718
[16962]	SKIL	<---	NID2	-0.14021	0.13313
[16963]	CARD16	--->	CASP1	-0.076001	-0.18686
[16964]	PTPRU	--->	GNAI2	0.40483	-0.41277
[16965]	THY1	--->	ITGB3	0.041544	-0.043074
[16966]	TRIM55	--->	USP13	0.092161	-0.077235
[16967]	SNAPC4	--->	POU2F1	0.11591	-0.083872
[16968]	FOXO1	<---	SIRT1	-0.011886	-0.00097782
[16969]	PBX1	?	HOXB1	-0.11417	-0.11417
[16970]	EBI3	--->	IL27RA	-0.024139	-0.02496
[16971]	MPL	<---	SOCS1	-0.058518	0.055717
[16972]	TNFAIP3	--->	TICAM1	0.13385	-0.17435
[16973]	CASP6	<---	NEDD4	-0.072983	0.051513
[16974]	HSPB1	?	CRYAA	-0.096038	-0.096038
[16975]	SMAD2	<---	CISH	-0.22094	0.2682
[16976]	ABCA1	<---	SNTB1	-0.038572	0.030033
[16977]	PTPN1	--->	CTNNB1	0.60956	-0.61
[16978]	CDK12	--->	PRPF40A	0.46953	-0.49485
[16979]	RAB22A	--->	APPL2	0.30433	-0.29881
[16980]	TNFRSF9	--->	TRAF3	0.1285	-0.13411
[16981]	SOX8	<---	EGR2	-0.0046139	0.0013215
[16982]	RAF1	<---	ERBB2IP	-0.028501	0.014197
[16983]	ZBTB16	<---	NCOR1	-0.035957	0.023976
[16984]	ZMIZ1	--->	AR	0.95777	-0.95843
[16985]	LAMA3	--->	PLAT	-0.0040784	-0.0059348
[16986]	AGTR1	<---	GRK5	-0.19362	0.13522
[16987]	JAK1	<---	PRKCZ	-1.0001	1.0002
[16988]	HDAC2	--->	PPARD	0.085304	-0.074402
[16989]	CEBPB	?	CEBPA	-0.11448	-0.11448
[16990]	SNX4	<---	EGFR	-0.32869	0.32794
[16991]	MBL2	<---	PTPRC	-0.033091	0.020447
[16992]	PRMT1	--->	IFNAR1	0.43392	-0.44371
[16993]	SH3KBP1	<---	HCK	-1.0528	1.1813
[16994]	ID1	--->	TCF3	-0.13552	-0.39218
[16995]	STAT1	--->	IL2RB	0.16708	-0.30043
[16996]	FGFR4	<---	FGF2	-0.99871	1.0053
[16997]	CREBBP	--->	VDR	0.17826	-0.18496
[16998]	BRD7	--->	HIST3H3	0.29322	-0.24229
[16999]	ADIPOQ	--->	CDH13	0.079953	-0.090335
[17000]	PDZK1	<---	CLCN3	-0.72583	0.74042
[17001]	RNF11	--->	CALCOCO2	0.21794	-0.1836
[17002]	MTOR	--->	RHEBL1	0.1597	-0.16194
[17003]	KAT5	<---	TRIB3	-0.43407	0.41309
[17004]	GRIN2D	<---	MAP2K2	-0.20393	0.28279
[17005]	RYBP	--->	YY1	0.11228	-0.10555
[17006]	NTRK2	--->	NGFR	0.056379	-0.023889
[17007]	FGFR1	--->	PIK3R1	0.025123	-0.10487
[17008]	BFAR	--->	BCL2	0.54574	-0.54505
[17009]	PRKD1	--->	KIDINS220	0.41912	-0.43688
[17010]	CDK2	<---	PTPN2	-1.009	1.0061
[17011]	BTRC	--->	JUP	0.18765	-0.19886
[17012]	MAP2K2	<---	ARAF	-1.0047	0.9856
[17013]	NLRC4	<---	BCL10	-0.087394	-0.04463
[17014]	PECAM1	--->	JUP	0.12634	-0.1298
[17015]	FGF7	<---	HSPG2	-0.028988	0.016341
[17016]	ZBTB16	<---	RXRA	-0.038922	-0.018147
[17017]	NR3C1	<---	LCK	-0.4838	0.53046
[17018]	PPFIA1	--->	PPFIBP2	0.027796	0.0269
[17019]	PLCG1	<---	GRB2	-0.76596	0.49194
[17020]	NUP153	<---	IPO5	-0.027473	0.032999
[17021]	EP300	--->	NEUROD1	0.19969	-0.20391
[17022]	TANC1	--->	ACTB	0.23743	-0.23905

```
[17023] CNTF    --->    CNTFR   0.083818        -0.071381
[17024] RADIL   --->    ASCC2   0.021571        -0.016401
[17025] LCK     --->    NFKBIA  0.68922 -0.65792
[17026] DVL1    ?       DVL3    -0.059754       -0.059754
[17027] STAT1   <---    FLT1    -0.089725       0.056619
[17028] SHC1    <---    IL2RG   -0.086361       0.0071273
[17029] BOC     --->    CTNNB1  0.2065  -0.21531
[17030] SHC1    <---    PTPN11  -0.54121        0.47605
[17031] ACVR1   --->    SOCS6   0.094078        -0.12014
[17032] TRAF2   --->    SOX30   0.1024  -0.096889
[17033] MBD1    --->    SMAD3   0.23479 -0.18953
[17034] SYK     <---    JAK1    -1.0043 1.0096
[17035] RNF11   <---    ZFYVE9  -0.10809        0.10034
[17036] ATXN1   <---    FAM46A  -0.033322       0.029973
[17037] AKAP1   <---    PRKAR2A -0.27544        0.28956
[17038] DOCK8   <---    CDC42   -0.041827       0.031606
[17039] ACP1    <---    KDR     -0.4932 0.54883
[17040] CRKL    <---    PDGFRA  -0.0074943      -0.0068636
[17041] PIK3CG  <---    ADRBK1  -0.023174       0.018158
[17042] GNAI1   <---    DRD3    -1.008  1.0129
[17043] RBPMS   <---    DTX2    -0.057207       0.051197
[17044] IRAK2   --->    MYD88   0.10463 -0.095609
[17045] RHOA    --->    DIAPH1  0.10067 -0.10807
[17046] FFAR2   --->    GNAO1   1.0129  -1.008
[17047] GHR     <---    PTPN6   -0.32057        0.36347
[17048] CXCR2   --->    MYO5B   0.27327 -0.26005
[17049] JAK2    --->    PPP2CA  0.99934 -0.99423
[17050] TNFSF13 --->    TNFRSF14        0.99996 -1.0769
[17051] CRK     <---    GRB2    0.22272 0.23706
[17052] ACTG1   <---    SH3GL2  -0.3713 0.36694
[17053] ETS2    <---    NR3C1   -0.10264        0.11607
[17054] CREM    <---    CDC34   -0.23273        0.23455
[17055] PRMT1   --->    NCOA1   0.060923        -0.072651
[17056] NCK1    --->    RASA1   0.065403        -0.079232
[17057] EIF2AK3 --->    IKBKB   1.0117  -1.003
[17058] SKP2    --->    CDKN1C  0.15077 -0.11847
[17059] RAB2B   --->    SMAD4   0.55322 -0.47159
[17060] JAK2    --->    PLCG2   0.082672        -0.09501
[17061] AHR     <---    ESR1    -0.17306        0.18451
[17062] MAPKAPK2        --->    BAG2    0.59959 -0.62486
[17063] SPEN    --->    RARA    0.036945        -0.027678
[17064] HDAC5   --->    MEF2A   0.029756        -0.037031
[17065] DAB2    --->    LRP2    0.40272 -0.4035
[17066] MAPK8IP1        <---    MAP2K7  -0.30961        0.2909
[17067] TP53    <---    CDK1    -0.99962        1.0002
[17068] AR      <---    RELA    -0.21321        0.20268
[17069] SGK1    <---    NEDD4   -0.045856       0.015448
[17070] ZIC1    <---    GLI3    -0.013552       0.33841
[17071] VASP    <---    LPP     -0.085959       0.10335
[17072] SORBS2  --->    MLLT4   0.15672 -0.13285
[17073] CREBBP  --->    ZBTB17  0.026511        -0.039308
[17074] TADA2A  --->    HGS     0.00010426      -0.051681
[17075] HIST1H3C        <---    CBX1    -0.19484        0.24617
[17076] GRB10   <---    AKT1    -0.067255       0.15007
[17077] HSPB1   ?       HSPB8   -0.096038       -0.096038
[17078] EIF3I   --->    TGFB1   0.2546  -0.40822
[17079] CMA1    --->    AGT     0.11833 -0.082185
[17080] SMAD2   --->    INSR    0.020361        -0.025754
[17081] HABP2   <---    COL4A3  -0.011113       -0.0022521
[17082] JAK3    --->    IRS1    0.99889 -1.0106
[17083] GRIN2D  <---    RPS6KA3 -0.21119        0.15252
[17084] ADRB2   <---    NEDD4   -0.093233       0.081814
[17085] HDAC4   --->    MEF2D   0.041869        -0.047246
[17086] CDKN3   --->    CEBPA   0.30014 -0.2447
[17087] TAOK3   <---    ERN1    0.13189 0.14223
[17088] RAB38   --->    TGFBR1  0.010625        -0.037483
[17089] SREBF2  <---    HNF4A   -0.19516        0.18831
[17090] ZBTB16  <---    GATA1   -0.086971       0.087346
[17091] PPARA   <---    PRKCA   -0.65411        0.6447
[17092] SFRS5   <---    CD101   -0.31803        0.28624
[17093] KCNA4   <---    ACTN2   -0.11983        0.12376
[17094] MAPK8   --->    BCL2L11 0.43567 -0.45207
[17095] DLST    <---    PAK4    -0.0051224      0.17077
[17096] ZFAND5  --->    RAD23B  0.049279        -0.020584
[17097] PTPRC   --->    TYK2    0.14022 -0.1486
[17098] TBC1D4  <---    CDK5RAP2        -0.0079622      0.0013798
```

[17099]	GRIN1	<---	FUS	-0.11719	0.1155
[17100]	NR2C2	<---	TRAF2	-0.098931	0.099265
[17101]	EGFR	--->	EZR	0.10938	-0.11225
[17102]	MBTPS1	--->	BDNF	0.083504	-0.088859
[17103]	SGK3	--->	ITCH	0.21645	-0.23446
[17104]	RBL1	<---	ID2	-0.23638	0.22664
[17105]	CSK	--->	LCK	0.99631	-0.99108
[17106]	ATXN1	<---	CIC	-0.076453	0.072938
[17107]	MPP1	<---	AMOTL2	-0.059652	0.066494
[17108]	PDE10A	<---	WDR3	-0.17096	0.18621
[17109]	PXN	<---	ITGA4	-0.034471	0.023469
[17110]	MTPN	<---	RELA	-0.0125	0.01764
[17111]	MAP2K6	--->	STAT4	1.0227	-1.0418
[17112]	CSNK2A1	<---	HSPH1	-0.99901	1.0063
[17113]	PDLIM7	<---	PRKCZ	-0.14463	0.13399
[17114]	ABCB1	<---	CAV1	-0.096259	0.10921
[17115]	MMP3	<---	DCN	-0.18966	0.20515
[17116]	CRADD	--->	CASP2	-0.055149	-0.097951
[17117]	GNB2L1	<---	FYN	-0.95354	1.1139
[17118]	NR1H4	?	ESR1	-0.0089162	-0.0089162
[17119]	CASP6	<---	CFLAR	-0.9907	0.99569
[17120]	PTPRS	--->	EEF1G	0.27633	-0.28119
[17121]	SYNGAP1	--->	MPDZ	0.043213	-0.032953
[17122]	PCDHA6	<---	RELN	-0.087774	0.10343
[17123]	PRKG1	--->	CFTR	0.47148	-0.4767
[17124]	GLS2	<---	DLG2	-0.16542	0.17158
[17125]	GLS2	<---	DLG3	-0.26501	0.26144
[17126]	SMAD2	<---	STAMBPL1	-0.99775	0.99487
[17127]	MPP4	--->	MPP5	-0.0093747	-0.020129
[17128]	UBE2I	<---	HIPK2	-0.53523	0.50887
[17129]	RAC1	--->	PARD6B	0.11085	-0.11319
[17130]	TOM1L1	--->	HGS	0.12908	-0.13845
[17131]	PTH1R	<---	YWHAH	-0.39834	0.39833
[17132]	VRK2	<---	MAP3K7	-0.55566	0.56879
[17133]	CASP1	<---	IL1B	-0.084506	0.083786
[17134]	MACF1	<---	PLXND1	-0.15338	0.15882
[17135]	GRIN1	<---	AKAP9	-0.17464	0.18165
[17136]	RHOG	--->	SMAD4	0.55322	-0.47159
[17137]	YAP1	--->	TEAD3	0.64247	-0.63315
[17138]	TRIM54	<--->	TRIM55	0.15517	0.15517
[17139]	C5	<---	C5AR1	-0.22186	0.23997
[17140]	SPHK1	<---	TRAF6	-0.68673	0.67971
[17141]	SPRED1	<---	KIT	-0.99899	1.0002
[17142]	PSEN1	--->	NOTCH1	0.99454	-1.0047
[17143]	FNBP1	--->	WASL	0.092082	-0.11591
[17144]	MAGI2	--->	GRID2	0.013733	-0.018729
[17145]	MLL2	--->	SMAD9	0.34922	-0.36661
[17146]	EDARADD	?	EDAR	-0.062038	-0.062038
[17147]	CTNNAL1	<---	NEK1	-0.21887	0.18269
[17148]	TRPC4	?	TRPC5	-0.0075092	-0.0075092
[17149]	YWHAG	--->	SFRS3	0.092231	-0.070624
[17150]	TIMP1	--->	MMP3	0.21399	-0.19321
[17151]	DLG3	<---	MAPK12	-0.14764	0.087106
[17152]	AKT1	--->	CHUK	1.0001	-0.99768
[17153]	SNX9	--->	ADAM9	0.010876	-0.018993
[17154]	SOX2	--->	NR5A1	0.018836	-0.17467
[17155]	DLGAP3	<---	PTK2	-0.12257	0.017126
[17156]	G3BP1	<---	CSK	-0.90832	1.0682
[17157]	IL2RB	<---	SOCS3	-0.22593	0.17341
[17158]	TRAF3	<---	EDA2R	-0.13411	0.1285
[17159]	ERBB2IP	--->	ITGB4	0.013061	-0.023356
[17160]	PLCD1	<---	TGM2	-0.23659	0.24924
[17161]	BAD	<---	MAP2K5	-0.89025	0.89927
[17162]	PRKCB	--->	GJA1	0.22576	-0.23456
[17163]	MAPK8	--->	BAD	1.2302	-1.4217
[17164]	DLG2	--->	ATP2B2	0.1195	-0.12951
[17165]	DLG1	--->	KCNA1	0.047736	-0.049447
[17166]	TSC1	<---	YWHAQ	-0.08934	0.11987
[17167]	AKAP9	--->	CALM3	0.35144	-0.31397
[17168]	PLK1	<---	PKMYT1	0.0029423	0.19171
[17169]	DUSP13	--->	TGFBR1	0.010136	-0.035422
[17170]	SMAD7	<---	MAPK14	-0.99469	1.0053
[17171]	TRAIP	--->	TRAF1	0.1472	-0.12358
[17172]	EPOR	<---	GAB1	-0.17314	0.16265
[17173]	CSNK2A2	--->	UBE2R2	0.2372	-0.25823
[17174]	SRC	--->	PRKCI	0.0965	-0.093142

```
[17175] WASF1   <---    NCK1    -0.28925        0.27913
[17176] PRKCB   --->    SYT6    0.48164 -0.070652
[17177] NOV     <---    IGF1    -0.29273        0.28155
[17178] PRKCB   --->    DAB2    0.91797 -0.91615
[17179] TRIM24  --->    NR3C1   0.11471 -0.088819
[17180] ELK1    <---    PIAS2   -0.21027        0.22675
[17181] SNX2    <---    ACVR2B  -0.2683 0.23826
[17182] SNIP1   --->    RELA    0.073383        -0.075125
[17183] HNF1A   --->    CTNNB1  0.53881 -0.53767
[17184] HSP90AA1        --->    CALM1   0.097354        -0.10485
[17185] RNF111  --->    SMAD3   1.0166  -0.99999
[17186] CD40    <---    CAV1    -0.18429        0.17148
[17187] MAP2K3  --->    MAPK12  0.64901 -0.41806
[17188] TGFB1   <---    SPARC   -0.58597        0.43327
[17189] CSK     --->    DOK1    1.3209  -1.1853
[17190] STAT5A  <---    BMX     -0.28638        0.20849
[17191] COL5A3  <--->   COL5A1  0.0041938       0.0041938
[17192] CASP8   <---    CFLAR   -0.99651        1.0068
[17193] BNIP2   <--->   BNIPL   0.012831        0.012831
[17194] THRA    <---    AKAP13  -0.033914       0.035086
[17195] PKP4    <---    PSEN1   -0.065576       0.074964
[17196] YAF2    --->    YY1     0.11228 -0.10555
[17197] CISH    --->    EPOR    0.055717        -0.058518
[17198] IL4R    <---    PRKCZ   -0.16905        0.15532
[17199] CEBPE   --->    RB1     0.45562 -0.45357
[17200] ACTA2   <---    CREBBP  -0.13205        0.12357
[17201] PTPRC   --->    JAK2    0.19532 -0.20418
[17202] NFKB1   <---    UBE2D3  -0.99769        0.9883
[17203] COPS5   --->    UCHL1   0.21491 -0.18847
[17204] RAC1    <---    RASGRF1 -1.0025 1
[17205] PEA15   --->    PLD2    0.47233 -0.47475
[17206] BUB1    --->    CDC20   0.83716 -0.86422
[17207] ATP7B   --->    ZBTB16  0.057865        -0.064126
[17208] DAPK3   --->    PRKCZ   0.1627  0.012708
[17209] BIRC2   --->    GAS1    0.12263 -0.12691
[17210] CACNB3  --->    CACNA1B 0.16653 -0.17372
[17211] KCNJ16  ?       KCNJ2   -0.01825        -0.01825
[17212] FSTL3   --->    MSTN    1.0085  -0.99524
[17213] THY1    <---    FYN     -1.0419 1.2764
[17214] DAZ4    ?       BOLL    -0.016298       -0.016298
[17215] UBE2N   <---    AURKA   -0.53523        0.50887
[17216] DDX6    --->    U2AF2   0.015842        -0.0019217
[17217] PTK2    <---    LYN     -0.65057        0.81773
[17218] SKIL    <---    NCOR2   -0.089988       0.077776
[17219] PCSK1   <---    REN     -0.22971        0.23025
[17220] ENAH    <---    PFN1    -0.010558       0.00065906
[17221] C2      --->    MASP1   -0.0025717      -0.0065822
[17222] ZMYND11 --->    HDAC1   0.014167        0.001254
[17223] FLT1    --->    YES1    0.23463 -0.2101
[17224] PECAM1  --->    PRPF40A 0.11578 -0.087577
[17225] YWHAG   <---    CHEK1   -0.66368        0.62776
[17226] HIST1H4A        <---    TBL1X   -0.033407       0.034046
[17227] HTR2A   --->    ANKS1B  0.13165 -0.13922
[17228] MPRIP   <---    RHOA    -0.42614        0.44248
[17229] CORO2B  --->    ACTA1   0.4503  -0.44823
[17230] ERBB4   --->    CD44    0.041635        -0.053239
[17231] ATN1    --->    RHOXF2  0.050738        -0.053245
[17232] TSC1    --->    TSC2    0.27251 -0.26619
[17233] PPFIA3  <---    PTPRD   -0.071311       0.064659
[17234] KCNJ4   <---    CASK    -0.1462 0.13185
[17235] YWHAB   <---    RAF1    -0.051212       0.022119
[17236] TEAD1   --->    MYH7    0.0060129       -0.018323
[17237] PDX1    --->    SLC2A2  0.25973 -0.23531
[17238] LYN     --->    CD79B   1.0799  -1.0399
[17239] SCAP    --->    SREBF2  0.16164 -0.16597
[17240] IKBKB   <---    TBK1    -0.61213        0.61363
[17241] NCOA1   <---    NR3C1   -0.043163       0.034596
[17242] DDX19B  --->    NUP214  0.00823 0.0071275
[17243] DLG2    --->    GRIN2D  0.026321        -0.034965
[17244] NCOR1   --->    AR      0.12804 -0.13969
[17245] FLT1    --->    FYN     0.23463 -0.2101
[17246] APAF1   <---    CASP8   -0.48671        0.39449
[17247] NUAK2   --->    SMAD2   0.61302 -0.61567
[17248] DDR1    --->    COL11A1 0.060375        -0.073217
[17249] FLT1    --->    CTNNB1  0.079539        -0.090285
[17250] LTF     --->    LRP1    0.14759 -0.15674
```

```
[17251] GRN     --->    SMAD9   0.29535 -0.31401
[17252] PRKG1   --->    PPP1R12A        0.99718 -1.0017
[17253] DMPK    --->    PPP1R12A        0.35178 -0.366
[17254] ARR3    <---    CSNK2A1 -0.44306        0.42184
[17255] PTPN11  --->    IGF1R   0.087425        -0.065062
[17256] EPOR    <---    GRB2    -0.17313        0.16008
[17257] PTPN6   --->    JAK3    0.9903  -0.98654
[17258] RXFP1   --->    RLN2    0.15462 -0.14912
[17259] MDC1    --->    ATM     0.21656 -0.22664
[17260] CACNA1B <---    GNB1    -0.15907        0.15167
[17261] CDC42BPG        <---    CDC42   -0.1141 0.11487
[17262] S1PR1   --->    GNAI1   0.46515 -0.46841
[17263] NR1H4   <---    CASP8   -0.49913        0.41161
[17264] BTRC    --->    UBQLN2  0.51465 -0.52067
[17265] CABP2   <---    CAMK2G  -0.74572        0.73919
[17266] FGF3    <---    RPS2    -0.013688       0.047514
[17267] TRPM2   --->    ITPR3   -0.0064875      -0.034054
[17268] CCNK    <---    CDK2    -0.6658 0.64437
[17269] GNAQ    <---    BTK     -0.68342        0.63988
[17270] TFDP2   <---    E2F4    -0.064857       -0.0088085
[17271] OXSR1   <---    PAK1    -0.84122        0.89243
[17272] SLC2A4  <---    HK2     -0.20456        0.21502
[17273] SIGIRR  <---    TLR4    0.014121        0.34485
[17274] COPS6   --->    CUL5    0.02459 -0.017361
[17275] PPFIA1  <---    PTPRF   -0.017812       0.012075
[17276] HES1    <---    ID3     -0.29914        -0.087238
[17277] XAB2    --->    ERCC8   0.13319 -0.099596
[17278] DTX2    <---    NOTCH1  -1.0124 1.0135
[17279] KAT5    --->    RGL2    0.088579        -0.098381
[17280] CHD3    <---    GPS2    -0.0056149      -0.0044028
[17281] DUSP19  --->    MAPK9   1.011   -0.99819
[17282] MAPK15  <---    SRC     -1.3225 1.3946
[17283] CCND2   <---    CDKN1B  -1.0151 1.0016
[17284] CASP3   --->    STK3    1.0019  -1.0107
[17285] LUM     <---    COL1A2  -0.030712       0.023329
[17286] SIGLEC6 <---    LEP     -0.023728       0.017484
[17287] PDPK1   --->    PRKCZ   0.27769 -0.26638
[17288] GRAP2   --->    SHC1    0.39868 -0.40614
[17289] ESR2    --->    CALM1   0.15612 -0.12827
[17290] PTPN6   --->    PTK2B   0.99625 -0.99764
[17291] USF1    --->    FOSL1   0.007062        -0.01755
[17292] PPP1R13L        --->    RELA    0.0080874       -0.010665
[17293] RNF8    --->    CCDC85B 0.082271        -0.071039
[17294] PTPN11  --->    CD33    0.65471 -0.5666
[17295] DYRK1A  --->    SF3B1   0.39838 -0.41811
[17296] PRKACA  --->    ITPKB   0.3157  -0.34471
[17297] PAX6    --->    MITF    0.17894 -0.17658
[17298] PDPK1   --->    PRKCB   0.99669 -0.99581
[17299] PLK1    <---    CHEK2   -0.00064284     0.1132
[17300] SFRS17A <---    ZRANB2  -0.030411       0.064771
[17301] YWHAG   <---    PRKCD   -0.76873        0.77791
[17302] CLPB    <---    TTF2    -0.052157       0.050249
[17303] DBNL    <---    MAP4K1  -0.22316        0.23302
[17304] MAP3K7  <---    TRAF6   -0.99941        0.99968
[17305] PRKCG   --->    PEBP1   1.0121  -1.008
[17306] RAE1    <---    DDX24   -0.076481       0.091156
[17307] ACTN4   --->    SERPINE1        0.036472        -0.031231
[17308] UBE2I   --->    SREBF1  0.37111 -0.36902
[17309] AKAP11  <---    PPP1CA  -0.21471        0.20763
[17310] LMO1    --->    LYL1    0.62562 -0.58677
[17311] ST14    <---    PLAU    -0.012425       0.00068251
[17312] SOS1    <---    GRB2    -0.99897        1.0047
[17313] STAT5B  <---    CTLA4   -0.27627        0.25636
[17314] YWHAE   <---    TNFAIP3 -0.13918        0.13176
[17315] SERPING1        <---    F2      -0.040728       0.057207
[17316] XPO7    <---    NUP153  -0.030611       0.064851
[17317] UBE2I   --->    PDLIM7  0.10576 -0.088475
[17318] ASH2L   --->    SMAD9   0.3016  -0.32021
[17319] ARRB2   --->    SLC9A5  0.044822        -0.02919
[17320] ADAM12  <---    FSTL3   -0.11077        0.10811
[17321] CAPN1   --->    MYBPC3  0.13831 -0.14438
[17322] SETDB1  <---    ULK2    -0.056636       0.04412
[17323] ARNT    <---    ESR1    -0.10974        0.11124
[17324] AXL     --->    PIK3R3  0.20639 -0.26123
[17325] PER1    <---    CSNK1D  -0.58384        0.72042
[17326] INSR    <---    KHDRBS1 -0.036533       0.024722
```

[17327]	DAPK1	--->	UNC5B	0.004408	-0.013127
[17328]	ARID3A	--->	E2F4	0.01732	0.01461
[17329]	PPARA	<---	GADD45A	0.0079001	0.017932
[17330]	CSNK1A1	--->	TP53	0.3735	-0.34877
[17331]	RXRA	--->	RELA	-0.021752	-0.032655
[17332]	EGFR	--->	AR	0.22159	-0.23249
[17333]	IGFBP7	--->	CCL5	0.050367	-0.047815
[17334]	IL2RG	<---	IL2RB	-0.13003	-0.024456
[17335]	NEK2	<---	PPP1CA	-0.067136	0.046927
[17336]	LCP2	<---	NCK1	-0.30064	0.4181
[17337]	SFRP2	--->	WNT4	0.99043	-0.98922
[17338]	CXXC5	--->	SMAD9	0.1889	-0.20563
[17339]	EGFR	--->	RGS16	0.99759	-1.0133
[17340]	DOK1	<---	ABL1	-0.12751	-0.027909
[17341]	STAT3	<---	IGF1R	-0.17044	0.15031
[17342]	MAP3K1	--->	RAF1	0.35918	-0.26764
[17343]	STAM2	<---	JAK1	-0.35847	0.34904
[17344]	TMPRSS11A	--->	ZBTB17	0.14912	-0.16215
[17345]	UBE2G2	<---	CBL	-0.47935	0.47348
[17346]	ATR	<---	BRCA1	-0.012028	0.0049915
[17347]	PTPN2	--->	PDGFRB	0.11574	-0.12276
[17348]	PKP3	--->	DSC2	0.61098	-0.61498
[17349]	RHOQ	--->	PRKCI	0.99149	-1.0135
[17350]	CDC40	--->	SMAD2	0.46428	-0.38187
[17351]	STAT5A	<---	BRCA1	-0.30452	0.2887
[17352]	TBP	--->	REL	-0.0020661	-0.011747
[17353]	PDZD2	<---	CTNND2	-0.0046437	0.0069313
[17354]	SMAD7	<---	MAP2K6	-1.0074	1.012
[17355]	IGFBP5	<---	PAPPA2	-0.038431	0.07239
[17356]	SREBF2	<---	SP1	-0.11338	0.1015
[17357]	SLC9A3R2	<---	ADRB2	-0.19778	0.21221
[17358]	CAV1	--->	APP	0.36163	-0.37176
[17359]	NRCAM	<---	PTPRB	-0.059828	0.060744
[17360]	RYR2	<---	ITPR1	-0.0078762	-0.0070005
[17361]	PPM1B	--->	CDK6	1.0075	-0.9963
[17362]	STAT2	<---	IFNAR1	-0.12667	0.09213
[17363]	EPOR	--->	PLCG1	0.52619	-0.56072
[17364]	LTBP3	--->	HBEGF	0.0054525	-0.03295
[17365]	ACTC1	<---	CAPN1	-0.28049	0.27236
[17366]	HGS	<---	STAM	-0.9996	0.99705
[17367]	CENPA	<---	DIAPH2	-0.03689	0.031778
[17368]	MAP2	<---	GRB2	-0.22163	0.22469
[17369]	RAB38	--->	SMAD4	0.55322	-0.47159
[17370]	ATN1	--->	BAIAP2	0.0087436	-0.018477
[17371]	MET	--->	PIK3R1	0.21153	-0.41
[17372]	GRK6	<---	RCVRN	-0.24096	0.19057
[17373]	ELK4	--->	ID3	0.12676	-0.084944
[17374]	PHIP	--->	STMN4	0.03822	-0.01787
[17375]	SHC3	<---	EGFR	-0.95742	0.95146
[17376]	MPP3	--->	CCDC137	0.060022	-0.054969
[17377]	ARHGEF7	<---	CD244	-0.054428	0.05088
[17378]	AXIN1	<---	JUP	-0.042854	0.031408
[17379]	ATM	<---	MRE11A	-0.227	0.21692
[17380]	SNTG1	--->	KCNA4	0.050181	-0.013537
[17381]	PDLIM1	--->	ACTN1	0.0082818	-0.010186
[17382]	BTRC	--->	SMAD4	1.0078	-1.0017
[17383]	GFI1	<---	PICK1	-0.14183	0.15108
[17384]	FER	--->	STAT3	0.17746	-0.24207
[17385]	ADAMTS4	--->	SERPINA3	0.031729	-0.027936
[17386]	MUC1	<---	LYN	-0.91849	0.955
[17387]	TGM2	--->	ITGB1	0.13852	-0.14777
[17388]	SFRS2IP	--->	SFRS2	0.12373	-0.11096
[17389]	CCND1	<---	CDK4	-1.0774	1.0544
[17390]	MMP2	<---	COL7A1	-0.021313	0.0090209
[17391]	ATXN1	<---	ZHX3	-0.012951	0.0091738
[17392]	PRKCD	--->	ITGB7	0.28454	-0.28978
[17393]	MAPK3	<---	MYLK	0.12804	0.30099
[17394]	EP300	--->	CHD4	0.0085638	-0.021725
[17395]	TNK2	<---	FYN	-0.013149	0.0028058
[17396]	GP1BA	--->	VWF	0.02785	-0.032687
[17397]	CASP3	--->	HNRNPU	0.59134	-0.70233
[17398]	CRKL	--->	WIPF1	0.041384	-0.059712
[17399]	NCK1	<---	MAP4K1	-0.16145	0.16473
[17400]	ARVCF	--->	CDH1	0.98604	-0.99605
[17401]	MLL	--->	ASH2L	0.080573	-0.064023
[17402]	EFS	<---	PTK2	-0.049536	-0.00037625

```
[17403] RANBP2  --->    HDAC4   0.030392        -0.025724
[17404] CSNK2A2 --->    NCF1    0.21063 -0.24169
[17405] EPOR    --->    PLCG2   0.19635 -0.29494
[17406] FGFR4   --->    PLCG1   0.21807 -0.28799
[17407] ABCC9   <---    KCNJ8   -0.029689       0.027911
[17408] RHEBL1  --->    SMAD1   0.52759 -0.54836
[17409] RASL12  <---    SMURF2  -1.0084 1.0007
[17410] SNX2    <---    ACVR1B  -0.39149        0.35774
[17411] KLHL17  --->    ACTC1   0.49407 -0.49201
[17412] RAB11FIP3       <---    RAB11B  -0.43801        0.45325
[17413] JUN     <---    SKI     -0.14459        0.11329
[17414] WWP2    --->    PDLIM7  0.1524  -0.16215
[17415] MAP2K4  <---    MAP3K12 -0.9926 1.0104
[17416] SMAD2   <---    ROCK1   -0.068769       0.056017
[17417] PPARGC1A        --->    NCOA1   0.011583        -0.024678
[17418] CDC42   --->    CDC42EP3        0.57849 -0.87115
[17419] FYN     --->    ATXN1   0.26551 -0.24413
[17420] ACVR1   <---    RAB25   -0.32935        0.31819
[17421] WNT3A   --->    FZD1    0.15445 -0.15879
[17422] MEX3B   --->    SMAD4   0.273   -0.22853
[17423] CASP8   --->    MAPK3   0.99111 -0.99197
[17424] NCS1    <---    ADRBK1  -0.57187        0.55237
[17425] YWHAG   <---    NCKIPSD -0.090647       0.091898
[17426] MAP1B   <---    GSK3B   -0.77493        0.77181
[17427] RXRG    <---    PNO1    -0.23966        0.029584
[17428] CNKSR1  --->    MAP2K2  0.1394  0.052938
[17429] CASP6   --->    APP     0.23458 -0.24991
[17430] OSMR    <---    JAK2    -0.30826        0.42522
[17431] AR      <---    POU2F1  -0.95044        0.94974
[17432] VASP    <---    PLSCR1  -0.069894       0.082708
[17433] KDR     --->    RASA1   0.10932 -0.16289
[17434] CCND2   --->    RB1     0.99958 -1.0044
[17435] NOV     <---    GJA1    -0.092643       0.080706
[17436] YWHAZ   --->    CDC25A  0.14141 -0.11975
[17437] PIAS1   --->    RELA    0.061658        -0.066672
[17438] PDPK1   --->    AKT2    0.99822 -0.99934
[17439] MAP3K5  --->    YWHAQ   0.23745 0.17403
[17440] CDKN1B  <---    CDK3    -0.36687        0.34497
[17441] BCL10   <---    UBE2N   -0.26796        0.30144
[17442] FADD    <---    IRAK1   -0.18823        0.47953
[17443] CDC42BPA        --->    MYL2    0.11462 -0.12425
[17444] BIRC6   --->    CASP9   0.069243        -0.070067
[17445] PRKACA  --->    MC4R    0.21381 -0.23401
[17446] NLRC4   --->    CASP1   -0.061733       -0.10304
[17447] HMGB1   <---    RBPJ    -0.031012       0.032995
[17448] GDF6    <---    SMAD1   -0.055306       0.060031
[17449] VAV3    <---    SHC1    -0.021897       0.012069
[17450] IQGAP2  <---    RHOG    -0.13647        0.1548
[17451] CDK1    --->    NUP210  0.64216 -0.66848
[17452] JUP     <---    CTNNBIP1        -0.075945       0.073576
[17453] CDK2    --->    ID2     1.5563  -1.5271
[17454] BLNK    <---    MAP4K1  -0.26273        0.2595
[17455] ACVR1   <---    RAB33B  -0.32935        0.31819
[17456] GABPB1  <---    RYBP    -0.61802        0.65136
[17457] ERBB4   --->    MUC1    0.11556 -0.12727
[17458] CSNK2A2 --->    EIF4EBP1        1.0012  -1.0031
[17459] SKIL    <---    EEF1G   -0.40219        0.40645
[17460] ZBTB7A  <---    SP4     -0.088103       -0.019253
[17461] DLG1    <---    ERBB4   -0.19014        0.18045
[17462] PRKAR2A --->    EZR     0.17104 -0.16742
[17463] IGF1    --->    IGFBP2  0.087568        -0.072295
[17464] RGS2    --->    NINL    0.065091        -0.035677
[17465] USP6    --->    ANKRD44 0.14937 -0.13057
[17466] PAK2    --->    ARHGEF6 0.035613        -0.0010098
[17467] DOK4    <---    RASA1   -0.047053       0.11766
[17468] RAB11FIP4       <---    RAB11A  -0.38384        0.41459
[17469] ZNF423  <---    SMAD1   -0.10753        0.095653
[17470] GAB1    --->    INPP5D  0.14126 -0.14483
[17471] ENG     --->    BMP2    0.36037 -0.30966
[17472] ADRBK1  --->    BDKRB2  0.20207 -0.22265
[17473] LAT     --->    PLCG2   0.38292 -0.51638
[17474] CCNE2   <---    CDK2    -1.0774 1.0544
[17475] FN1     <---    COL7A1  -0.013192       -0.0054466
[17476] PRKCG   --->    GSK3A   0.22826 0.12473
[17477] RIT1    --->    SMAD3   0.55322 -0.47159
[17478] MAPK7   --->    MYC     0.40878 -0.48462
```

[17479]	ETS1	<---	NFKB1	-0.47489	0.47438
[17480]	CDK9	--->	ZMYM6	0.69999	-0.72592
[17481]	IL27RA	<---	MAPK8	-0.47533	0.44975
[17482]	CD2AP	<---	GRB2	-0.10655	0.3004
[17483]	PDZK1	--->	SCARB1	0.14936	-0.1156
[17484]	MAPK8	--->	ELK3	1.0428	-0.98814
[17485]	CSNK1E	--->	AXIN2	0.12779	-0.12095
[17486]	PIAS4	--->	SMAD1	0.62814	-0.67658
[17487]	HIPK2	--->	BAT1	0.27819	-0.29616
[17488]	IRS4	--->	PIK3CA	0.27653	-0.28366
[17489]	ATXN1	<---	LRSAM1	-0.031921	0.022049
[17490]	BMPR1B	<---	RHEBL1	-0.53994	0.52577
[17491]	CFLAR	<---	MAP2K7	-0.077064	0.090997
[17492]	ABI3	<---	PAK1	-0.13366	0.32915
[17493]	IRS1	<---	PDPK1	-0.23162	0.35854
[17494]	ICAM1	--->	ITGAM	0.024789	-0.037876
[17495]	PIK3R1	<---	RASA1	-0.0086165	0.028886
[17496]	CDKN2A	<---	NCL	-0.016333	0.013719
[17497]	NCK2	--->	FXR2	0.24919	-0.25351
[17498]	SCN10A	--->	ACTB	0.51926	-0.51648
[17499]	EPS8	<---	ACTA1	-0.08066	0.024228
[17500]	HRAS	--->	PLCE1	0.99635	-0.99834
[17501]	SKIL	<---	CBX4	-0.40444	0.40849
[17502]	CEBPE	<---	UBE2I	-0.28186	0.33117
[17503]	PIAS3	--->	HDAC1	0.062822	-0.061272
[17504]	IL16	--->	KCNJ4	0.044422	-0.0070376
[17505]	SMAD3	<---	ZBTB16	-3.2377e-005	0.007722
[17506]	INSR	--->	JAK2	0.12693	-0.11206
[17507]	KIT	<---	CBL	-0.10729	0.089475
[17508]	SHB	--->	IL2RG	0.14649	-0.212
[17509]	CD48	<---	FYN	-0.17174	0.1294
[17510]	DUSP16	--->	MAPK10	0.87425	-0.89445
[17511]	AMBP	--->	ALB	0.10884	-0.076098
[17512]	KLK2	--->	SERPINA4	0.11833	-0.082185
[17513]	DOCK8	<---	RHOQ	-0.041827	0.031606
[17514]	TNFRSF13C	--->	TRAF3	0.018195	-0.015355
[17515]	MKNK2	--->	ESR2	0.99834	-1.0079
[17516]	ACP1	--->	EPHB1	0.023989	-0.034598
[17517]	ACVRL1	<---	TGFBR2	-0.54629	0.4833
[17518]	APPL1	<---	FSHR	-0.2555	0.26704
[17519]	DOK4	<---	RET	-0.35228	0.31842
[17520]	NCKIPSD	<---	GRB2	-0.19873	0.4846
[17521]	CD2AP	--->	PIK3R1	0.12189	-0.29979
[17522]	PABPC1	--->	PCBP1	0.036464	-0.0048144
[17523]	SRF	--->	RXRA	0.047035	-0.1777
[17524]	PDCD6IP	<---	SH3GL1	-0.10824	0.10966
[17525]	STAT5A	<---	ERBB4	-1.0106	1.0062
[17526]	MMP3	<---	CCL7	-0.16713	0.18806
[17527]	CSK	--->	RGS16	0.94045	-0.7821
[17528]	HSP90AA1	--->	GUCY1B3	0.07493	-0.082884
[17529]	SKAP1	<---	LCK	-0.55395	0.61951
[17530]	AXIN1	<---	PPP2CB	-0.99843	0.998
[17531]	CDH8	<---	CTNNB1	-0.32695	0.32855
[17532]	SOCS6	<---	RBCK1	-0.044893	0.065868
[17533]	FYN	--->	FCGR2A	0.11748	0.015127
[17534]	PAX5	<---	TBP	-0.010047	-0.0026666
[17535]	TNFSF9	--->	TRAF1	0.27438	-0.24744
[17536]	MEOX1	--->	PAX1	0.041579	-0.0073189
[17537]	BTBD2	<---	TGFBR1	-0.15459	0.13275
[17538]	PRF1	--->	CALR	0.13567	-0.1093
[17539]	RASSF1	--->	DAXX	0.10091	-0.1222
[17540]	CASK	--->	GRIK2	0.1257	-0.13919
[17541]	HOMER2	<---	GRM1	0.01285	0.022232
[17542]	SH3BP2	--->	YWHAQ	-0.067846	-0.088071
[17543]	MINK1	--->	CALM3	0.097379	-0.094487
[17544]	TIMP1	--->	MMP1	0.21399	-0.19321
[17545]	SRGAP2	--->	ROBO1	0.010069	-0.020968
[17546]	F2R	--->	GNA15	1.0129	-1.008
[17547]	HDAC2	--->	SALL1	0.17875	-0.18352
[17548]	DOK3	<---	ABL1	-0.12751	-0.027909
[17549]	NCOR2	--->	PPARA	0.13736	-0.14734
[17550]	RFX4	--->	ESR1	0.069681	-0.043772
[17551]	SRC	--->	RET	0.76133	-0.17792
[17552]	PPM1A	--->	CACNA1B	0.20004	-0.20923
[17553]	PTK2	--->	TSC2	0.10208	-0.13346
[17554]	KAT5	<---	PLEKHA4	-0.13797	0.14604

[17555]	IRS1	<---	ROCK1	-0.074229	-0.0014004
[17556]	UBE2L3	--->	SMAD4	0.76473	-0.70088
[17557]	CD86	--->	CD28	0.089425	-0.15128
[17558]	JDP2	<---	KAT2B	-0.22403	0.20873
[17559]	PLG	<---	F2RL1	-0.27153	0.28486
[17560]	ATR	<---	NBN	-0.0048261	0.0020217
[17561]	GIT1	<---	TGFB1I1	-0.047634	0.03579
[17562]	ZBTB17	<---	MYC	-0.065034	0.052572
[17563]	DZIP1	--->	DAZ1	0.05464	-0.022843
[17564]	DCN	--->	COL1A1	-0.00034351	-0.0080368
[17565]	TJP3	<---	INADL	-0.027566	0.015158
[17566]	BTRC	--->	CUL1	0.29027	-0.30014
[17567]	RPS6KA5	--->	STAT3	0.6387	-0.6669
[17568]	YWHAG	--->	LIMA1	0.05651	-0.03118
[17569]	MAPK7	<---	MAP2K5	-1.0007	0.9943
[17570]	PLSCR1	<---	NTN4	-0.080461	0.077147
[17571]	IL2RB	<---	RAF1	-0.10577	0.066581
[17572]	ARHGEF1	<---	PRKCA	-0.99959	1.0018
[17573]	DCN	<---	COL4A3	-0.01099	0.0030847
[17574]	DLG4	--->	KCNA4	0.047736	-0.049447
[17575]	RECQL5	<---	MAPK8IP1	-0.021369	0.025242
[17576]	PTPRU	--->	CTNNB1	0.22012	-0.22884
[17577]	SKP2	<---	CDC34	-0.10854	0.1237
[17578]	TRPV2	<---	TRPV1	-0.044626	-0.042787
[17579]	SYK	--->	PIK3AP1	1.0011	-0.99209
[17580]	GJB1	<---	SRC	-0.54981	0.61355
[17581]	RIPK1	--->	SQSTM1	0.17282	-0.10256
[17582]	CCL13	<---	CCR3	-0.46475	0.49595
[17583]	PTPN18	--->	ARHGAP5	0.30918	-0.31741
[17584]	SOS2	--->	EGFR	0.070156	-0.07959
[17585]	CBLB	--->	PIK3R1	0.12788	-0.15408
[17586]	INPPL1	--->	ABL1	-0.070949	-0.12346
[17587]	RARA	<---	CDK7	-1.0079	0.99834
[17588]	BCL2	<---	MAPK14	-0.56609	0.45471
[17589]	MASP1	<---	MBL2	-0.028918	0.017757
[17590]	NCS1	<---	DRD2	-0.75527	0.78966
[17591]	DGKA	<---	SRC	-0.26021	0.29653
[17592]	MAP3K14	<---	CASP8	-0.99197	0.99111
[17593]	JAK2	--->	MPL	0.27406	-0.20849
[17594]	HSPG2	--->	CTGF	0.037021	-0.05085
[17595]	CLK1	<---	PTPN1	-1.009	1.0061
[17596]	CFLAR	--->	BCL2L1	0.59369	-0.63309
[17597]	YWHAB	<---	HSPA1A	-0.739	0.77111
[17598]	F10	<---	PLG	-0.0067573	-0.0058289
[17599]	HSPA4	--->	GATA1	0.98424	-0.93373
[17600]	BMP3	<---	COL4A1	-0.080535	0.072418
[17601]	CDK4	--->	CEBPA	0.97322	-0.93471
[17602]	PFN1	<---	DLG5	-0.0083595	0.027582
[17603]	SYT7	--->	NRXN1	0.083467	-0.043035
[17604]	GATA6	<---	HHEX	-0.015702	0.056147
[17605]	LRP1	<---	SERPINA1	-0.15161	0.14257
[17606]	PLN	--->	ATP2A1	0.19215	-0.19449
[17607]	RAP1GDS1	<---	HRAS	-0.41126	0.44004
[17608]	MAPK13	--->	MAP3K4	-0.047686	-0.16593
[17609]	FRS2	<---	PTPRF	-0.096726	0.089863
[17610]	SMAD3	<---	FLNA	-0.32036	0.40332
[17611]	PAK2	--->	ABI1	0.33366	-0.15272
[17612]	RNF11	<---	UBE2D1	-0.17305	0.18537
[17613]	KCNIP2	--->	KCND2	0.28353	-0.26746
[17614]	CADM1	--->	MPP3	0.020963	-0.031695
[17615]	STRAP	<---	TGFBR1	-0.40828	0.3741
[17616]	SF3B4	--->	FAM115A	0.026324	-0.013866
[17617]	PRKACA	<---	PTPRR	-0.37559	0.36571
[17618]	PTPRM	--->	CTNNB1	0.13052	-0.14124
[17619]	RBM14	<---	EP300	-0.18682	0.18243
[17620]	ADAM15	<---	SNX33	-0.015338	0.018773
[17621]	EP300	--->	ESR1	0.17826	-0.18496
[17622]	RFWD2	--->	JUN	0.13872	-0.16423
[17623]	CXCR4	--->	GNA13	1.0129	-1.008
[17624]	MAPK1	--->	EIF4EBP1	1.0012	-1.0031
[17625]	PTPRR	--->	MAPK7	0.020191	-0.10294
[17626]	MORF4L1	--->	RB1	0.48069	-0.48215
[17627]	AKT1	--->	DLC1	0.25476	-0.16872
[17628]	CTBP1	--->	NOS1	0.10063	-0.1138
[17629]	VAV1	<---	RHOA	-0.22722	0.041359
[17630]	ACTB	<---	DSTN	-0.30587	0.30201

[17631]	NKX2-3	<---	SRF	-0.073419	0.072004
[17632]	NCK1	--->	WAS	0.43378	-0.47397
[17633]	DMBT1	<---	SFTPD	-0.041445	0.030797
[17634]	CTNND1	--->	MUC1	0.032997	-0.046126
[17635]	GZMB	<---	PTK2	-0.12359	0.01814
[17636]	GRAP2	--->	STAMBP	0.16138	-0.15316
[17637]	CSF3	--->	CSF3R	0.99871	-0.98782
[17638]	GFI1B	<---	PICK1	-0.15628	0.15266
[17639]	NFATC1	--->	PPP3R1	0.22072	-0.22162
[17640]	MAPK1	--->	FCGR2B	0.28348	-0.31361
[17641]	ANGPT2	<---	TEK	-0.01137	-0.0025354
[17642]	RAF1	<---	FYN	-0.14067	0.45621
[17643]	AXL	--->	ADAM10	0.010932	-0.023686
[17644]	SORBS1	<---	VCL	-0.27056	0.27832
[17645]	DAB1	<---	RELN	-0.10353	0.12012
[17646]	GIPC1	<---	DRD3	-0.41277	0.44636
[17647]	SOX10	<---	HOXA3	-0.060464	0.063322
[17648]	PDPK1	--->	RPS6KB2	1.0929	-1.1695
[17649]	TNFRSF10B	<---	TNFSF10	-0.55375	0.5464
[17650]	PIAS2	--->	SNIP1	0.039487	-0.037176
[17651]	LDLRAP1	--->	LRP2	0.40272	-0.4035
[17652]	KAT5	--->	BARD1	0.093303	-0.087282
[17653]	ATXN1	<---	EFEMP2	-0.031466	0.028351
[17654]	ACTA1	<---	SPTAN1	-0.097921	0.085109
[17655]	TJP1	<---	MLLT4	-0.087541	-0.025679
[17656]	MAP2K6	--->	MAPK13	0.64901	-0.41806
[17657]	CASP3	--->	LYN	1.0113	-1.0017
[17658]	DDIT3	?	CEBPG	-0.11448	-0.11448
[17659]	RHOA	--->	ARHGAP5	0.35378	0.11034
[17660]	CNKSR2	<---	RALA	-0.061587	0.062721
[17661]	CDKN1B	<---	YWHAB	-0.053185	0.053525
[17662]	SMAD7	--->	MYOD1	1.0041	-1.0094
[17663]	NTN1	<---	DCC	-0.059841	0.047334
[17664]	MNT	<---	HMGB1	-0.81406	0.8544
[17665]	MAPK7	--->	YWHAB	0.33428	-0.5474
[17666]	FNBP1	--->	AKAP9	0.10983	-0.095812
[17667]	SH3BP1	<---	HCK	-0.53864	0.67237
[17668]	SYTL1	<---	NCF2	-0.094726	0.084837
[17669]	RAB22A	--->	EEA1	0.3878	-0.3573
[17670]	CAV3	<---	GNAS	-0.16912	0.17832
[17671]	CTTN	<---	GRB2	-0.21104	0.28868
[17672]	PTPN6	--->	BCR	0.19284	-0.1134
[17673]	SHC1	--->	PLCG2	0.045374	-0.06681
[17674]	HTR2A	<---	JAK2	-0.10882	0.17671
[17675]	PLAT	--->	PLG	-0.0087417	-0.0096131
[17676]	EVL	<---	LYN	-0.66629	0.75322
[17677]	SLC2A4	<---	ARF3	-0.78251	0.80123
[17678]	SMAD2	<---	ABTB1	-0.1527	0.19692
[17679]	CDH11	--->	CDH2	-0.018045	-0.093588
[17680]	CHD3	<---	HSPH1	-0.072985	0.063644
[17681]	TP53	<---	NR4A1	-0.19275	0.19807
[17682]	SSTR5	--->	GOPC	0.21221	-0.19778
[17683]	MAP3K7	--->	STAT3	0.31754	-0.39263
[17684]	SLC2A4	<---	MAP1S	-0.28331	0.31027
[17685]	CSNK2A1	--->	CASP2	0.99863	-0.99771
[17686]	RGS12	<---	HRAS	-0.59215	0.60317
[17687]	TNFSF11	--->	NFKBIA	1.0438	-1.0454
[17688]	RPS6KA5	--->	HIST4H4	1.009	-0.99586
[17689]	ATXN1	<---	SAR1A	-0.13505	0.13
[17690]	NFKBIA	<---	CHUK	-1.0087	1.0136
[17691]	NCK1	<---	NTRK2	-0.066523	0.026639
[17692]	CSF2RA	<---	IKBKB	-0.37913	0.24345
[17693]	YWHAB	<---	STK38	-0.2472	0.13548
[17694]	CHRDL2	--->	BMP4	0.71403	-0.67452
[17695]	SPEN	--->	MSX2	0.059243	-0.046733
[17696]	GPR26	<--->	HTR1A	0.49511	0.49511
[17697]	YWHAG	--->	DYNC1H1	0.082878	-0.058878
[17698]	HDAC3	<---	SMYD1	-0.057245	0.093486
[17699]	PLRG1	<---	CDC5L	-0.12045	0.15328
[17700]	BCL10	<---	TRADD	-0.13649	0.11413
[17701]	WDR5	<---	MBIP	-0.13624	0.16907
[17702]	DDX17	?	DDX5	-0.0098667	-0.0098667
[17703]	BLM	<---	TERF1	-0.0012546	0.016635
[17704]	RIPK2	--->	SMAD4	0.59014	-0.59288
[17705]	YWHAZ	--->	EPB41L3	0.05792	-0.057222
[17706]	CREBBP	--->	ESR1	0.17826	-0.18496

[17707] INPPL1 ---> FLNC 0.079061 -0.086371
[17708] BAK1 ? BAX -0.63915 -0.63915
[17709] CNTN1 ---> FYN 0.070912 -0.10775
[17710] MAPRE1 <--- PIK3R4 -0.23009 0.21138
[17711] SPON1 ---> APP 0.35889 -0.36891
[17712] PIN1 ---> RBPMS 0.3333 -0.30347
[17713] ACVR2A ---> ACVRL1 -0.059533 -0.11619
[17714] PF4 ? CCL5 -0.047195 -0.047195
[17715] RIN1 <--- RAB5A -0.99801 1.0021
[17716] RGS10 ---> GNAZ 1.0012 -1.0015
[17717] CHD4 ---> ATR -0.0063705 -0.0066622
[17718] PYCARD <--- NLRP3 -0.14874 0.18479
[17719] PLAT ---> SERPINE1 0.037242 -0.032776
[17720] MDC1 <--- RNF8 -0.043386 0.036407
[17721] SNX17 ---> VLDLR 0.14342 -0.14834
[17722] PHLDA1 ---> PABPC4 0.045914 -0.014282
[17723] TINAGL1 ---> SMAD9 0.31516 -0.33367
[17724] RASD1 ---> GNB1 0.54415 -0.51481
[17725] RDX <--- ICAM2 -0.025235 0.029602
[17726] GNB1 <--- HDAC5 -0.040448 0.046523
[17727] ABL1 ---> PSTPIP1 -0.039964 -0.058413
[17728] MMP2 <--- CCL7 -0.16713 0.18806
[17729] KHDRBS1 <--- FYN -0.61607 0.6674
[17730] KLHL20 ---> ACTA1 0.49407 -0.49201
[17731] YWHAB ---> GAPVD1 0.069314 -0.058536
[17732] SHC3 <--- RET -0.48588 0.32368
[17733] FES ---> BCR 0.16974 -0.14841
[17734] SFRS3 ---> PCBP2 0.036464 -0.0048144
[17735] PIAS1 ---> AXIN1 0.080773 -0.08972
[17736] HIF3A <--- HIF1A -0.0069781 0.10863
[17737] GRIN1 <--- SP3 -0.033736 0.033718
[17738] CABLES1 <--- TP73 -0.061942 0.079194
[17739] MINK1 <--- NCK1 -0.013599 0.0011448
[17740] EGFR ---> CALM2 0.34959 -0.34973
[17741] PDPK1 ---> ITGB3 0.10613 -0.084177
[17742] ZBED1 <--- UBE2I -0.17436 0.2079
[17743] TRIM8 <--- SOCS1 -0.036123 0.057971
[17744] HCLS1 <--- CASP3 -0.65101 0.58167
[17745] TNFRSF11B ---> VWF 0.030179 -0.0056869
[17746] SLA ---> VAV1 0.5996 -0.57842
[17747] VTCN1 <--- BTLA -0.26934 0.28328
[17748] CDH1 <--- PTPRF -0.052428 0.041072
[17749] SMAD7 <--- TTF1 -0.32939 0.4123
[17750] ASB2 ---> SMAD9 0.063356 -0.077649
[17751] MAPK3 ---> ZNF7 0.53059 -0.51252
[17752] TRIP4 ---> RELA 0.073837 -0.068258
[17753] UBE2I ---> HDAC1 0.10139 -0.085985
[17754] PAK2 <--- SORBS2 -0.11716 0.25826
[17755] IGF1R <--- PRKD1 -0.05257 0.035316
[17756] WASF2 <--- FYN -0.74439 0.82271
[17757] TFDP2 <--- E2F1 -0.041319 -0.0088482
[17758] SCYL3 ---> EZR 0.28628 -0.30395
[17759] RAB11FIP1 <--- RAB11A -0.15731 0.20092
[17760] TP53BP1 ---> TP53 0.38633 -0.37707
[17761] AKT1 ---> CHEK1 1.0168 -1.0071
[17762] TP53 <--- ARL3 -0.51833 0.52688
[17763] ARAF <--- RRAS -0.9994 1.0008
[17764] CREBBP ---> CDC25B 0.11057 -0.1183
[17765] RARA <--- SP1 -0.11174 0.10251
[17766] NBEA <--- PRKAR2B -0.18426 0.18669
[17767] NCOA2 <--- HIF1A -0.0075489 0.10864
[17768] C3AR1 ---> C4A 0.23997 -0.22186
[17769] GFI1B <--- IFFO1 -0.15555 0.15156
[17770] FCGR2A <---> GP6 0.45908 0.45908
[17771] NCOA2 <--- YWHAH -0.091303 0.082501
[17772] TCERG1 ---> CHD3 0.0092409 -0.020867
[17773] CSNK2A2 ---> HDAC2 0.4 -0.42066
[17774] DVL2 <--- CPSF7 -0.24253 0.23753
[17775] DVL1 ---> EPS8 -0.0078971 -0.015221
[17776] CSNK1A1 ---> CHRM3 0.2985 -0.25616
[17777] TNNI3K ---> ACTC1 0.38604 -0.40491
[17778] RAPGEF1 ---> RRAS2 1.0045 -0.99301
[17779] TLX2 <--- YWHAH -0.15698 0.17903
[17780] DNASE1 ---> ACTG1 0.29006 -0.29329
[17781] RRAS2 ---> PIK3R1 0.21 -0.30832
[17782] RAF1 <--- JAK1 -0.17422 0.23365

[17783]	AKAP9	<---	CSNK1D	-0.58225	0.71883
[17784]	CAV1	<---	CSK	-0.97511	1.0636
[17785]	FES	--->	IL4R	0.21182	-0.26391
[17786]	IGF1	--->	IGFBP3	0.087568	-0.072295
[17787]	YWHAG	--->	PFKFB2	0.29699	-0.29134
[17788]	CDKN2A	<---	MYC	-0.53052	0.52637
[17789]	SMARCD3	--->	RORA	0.052806	-0.042542
[17790]	MAP2K7	<---	MAP3K12	-0.9926	1.0104
[17791]	TRPC4	<---	SLC9A3R1	-0.090326	0.078517
[17792]	PLSCR1	--->	C10orf54	0.064775	-0.046375
[17793]	TTRAP	--->	CD40	0.17554	-0.18473
[17794]	CSNK2A1	--->	MYC	1.0154	-1.0065
[17795]	KDR	--->	FLT1	0.12048	-0.0019912
[17796]	FBXL5	<---	SKP1	-0.002664	0.0042561
[17797]	BAD	--->	BCL2L1	1.0001	-0.99613
[17798]	YWHAE	--->	KIF1C	0.049737	-0.040995
[17799]	HSP90B1	--->	LRP1	0.05085	-0.063039
[17800]	NR5A1	--->	AR	-0.0031537	-0.58072
[17801]	RANBP9	--->	ITGAL	0.23718	-0.24535
[17802]	GALNT13	--->	SKIV2L	0.018265	-0.002887
[17803]	SPAG8	<---	UBQLN4	-0.044159	0.078556
[17804]	BTRC	--->	AXIN1	0.10809	-0.12001
[17805]	PIAS1	--->	UBE2I	0.051031	-0.039915
[17806]	ADAM15	<---	FYN	-0.53832	0.63049
[17807]	RALGDS	<---	ARRB1	-0.15446	0.14726
[17808]	ADAMTS4	--->	BCAN	0.0043435	-0.015468
[17809]	PPP3CB	--->	SLC8A3	0.10266	-0.11189
[17810]	SMURF2	--->	SKIL	1	-1.0003
[17811]	PRTN3	--->	RELA	0.099888	-0.094153
[17812]	KCNJ3	--->	GNB1	0.13605	-0.10243
[17813]	PRKG1	--->	RGS2	1.0011	-1.0099
[17814]	FGFR1	<---	FGF18	-0.99871	1.0053
[17815]	CD36	<---	ITGA6	-0.0095428	0.0008925
[17816]	TIFA	<---	TRAF2	-0.13854	0.1442
[17817]	COL2A1	--->	MATN1	0.017871	-0.027944
[17818]	PSG5	--->	PRPF40A	0.058667	-0.063606
[17819]	MAP3K5	<---	TRAF6	-0.45172	0.51665
[17820]	RNF11	--->	TAX1BP1	0.1594	-0.13973
[17821]	CAV1	<---	HRAS	-0.37866	0.40294
[17822]	MAP3K4	<---	AXIN1	-0.024629	0.011925
[17823]	YWHAE	--->	CDKN1B	0.053525	-0.053185
[17824]	CSNK1E	--->	RAD54B	0.43304	-0.35569
[17825]	DDX17	<---	SORBS3	-0.11389	0.10575
[17826]	SMURF2	--->	FAM175B	0.68562	-0.68933
[17827]	IGFBP3	--->	CD44	0.063388	-0.065359
[17828]	MAP2K3	<--->	MAPK3	0.23429	0.23429
[17829]	RGS20	--->	GNAI1	1.0012	-1.0015
[17830]	TJP3	<---	OCLN	-0.043104	0.049764
[17831]	DDR1	--->	RGS2	0.18009	-0.19562
[17832]	FLNA	<---	DCN	-0.12231	0.15132
[17833]	NR1I2	<---	PPARGC1A	-0.021031	0.013278
[17834]	WASF2	<---	BAIAP2	-0.037191	0.033008
[17835]	FLNA	<---	ARRB1	-0.0077442	0.022957
[17836]	DVL1	<---	RAC1	-0.41752	0.41768
[17837]	ATXN1	<---	UBE2E3	-0.088933	0.081101
[17838]	CDK5RAP2	--->	ABCA2	0.037268	-0.021804
[17839]	FNBP1L	--->	WASL	0.13882	-0.16911
[17840]	KAT5	--->	MYC	0.17591	-0.17505
[17841]	NTRK3	--->	JUN	0.18672	-0.20517
[17842]	KCNV1	?	KCNB1	-0.025901	-0.025901
[17843]	CASP3	--->	PSEN2	0.58173	-0.69269
[17844]	CD40	<---	IL4R	-0.1127	0.10176
[17845]	ACVR1	<---	RHOJ	-0.32935	0.31819
[17846]	TGFBR1	--->	SMAD2	1.0115	-1.0087
[17847]	PDPK1	--->	PRKCE	0.86338	-0.85829
[17848]	KHDRBS3	<---	PRMT1	-0.1942	0.19028
[17849]	APP	<---	HOMER3	-0.14908	0.13656
[17850]	RAB1B	--->	RUSC2	0.47475	-0.44454
[17851]	ATP1B1	--->	FXYD7	0.12923	-0.089911
[17852]	ACVRL1	<---	TGFB1	-0.59518	0.56265
[17853]	LYN	--->	ACTB	0.83599	-0.74852
[17854]	DLG1	--->	KCNJ6	0.04551	-0.04695
[17855]	NOS1	<---	ADRA1A	-0.15603	0.1439
[17856]	GRB7	<---	ERBB2	-0.33539	0.31209
[17857]	SMAD2	<---	RARB	-0.17818	0.22689
[17858]	SIRT2	<---	KAT2A	-0.12902	0.12972

[17859]	CTNNB1	--->	CDH15	0.32855	-0.32695
[17860]	DAB1	--->	LRP2	0.40272	-0.4035
[17861]	CD300LF	<---	INPP5D	-0.088902	0.065508
[17862]	NCOA3	<---	ESRRA	-0.13412	0.12213
[17863]	CTNNB1	<---	CTNNA1	-0.56839	0.56912
[17864]	RARA	?	NR1H2	-0.0089162	-0.0089162
[17865]	MAP4K4	--->	CASP8	0.02694	0.0096868
[17866]	EEF1G	<---	PLEKHA4	-0.2488	0.2719
[17867]	NFKBIE	--->	REL	0.15087	-0.16049
[17868]	FBLN2	--->	VCAN	0.00056946	-0.012608
[17869]	CD28	--->	PLCG1	0.6733	-0.74
[17870]	ACVR1	<---	DUSP13	-0.32729	0.3177
[17871]	CSK	--->	PXN	0.99333	-0.91096
[17872]	ITK	--->	GRB2	0.13441	-0.027891
[17873]	PTPRD	--->	PTPRF	-0.0068368	-0.023703
[17874]	PPA1	--->	SETDB1	0.25185	-0.23649
[17875]	TCF12	<---	ID1	-0.33696	-0.091025
[17876]	ACVR1	<---	TGFBR1	-0.049446	-0.0024623
[17877]	SPTAN1	<---	TES	-0.053957	0.046734
[17878]	ARIH1	<---	UBE2L6	-0.026177	0.029733
[17879]	MAP3K2	--->	MAPK8	0.99233	-0.99622
[17880]	SHC3	<---	ALK	-0.82443	0.566
[17881]	CAMK2G	--->	GRIA4	0.47913	-0.49361
[17882]	CUL9	--->	PLXNB3	0.069749	-0.036353
[17883]	PIK3R2	<---	SOCS1	-0.18873	0.12705
[17884]	YWHAB	--->	RADIL	0.01098	-0.0092562
[17885]	SNIP1	--->	SMAD1	0.207	-0.22308
[17886]	PEBP1	<---	RAF1	-0.054366	0.029303
[17887]	NR4A1	<---	ARHGEF10L	-0.068206	0.094715
[17888]	NR3C1	<---	FYN	-0.57087	0.66681
[17889]	CDK1	--->	AR	1.2465	-1.2513
[17890]	YWHAZ	<---	MAP3K3	-0.30237	0.54958
[17891]	ACAN	--->	MMP14	0.022421	-0.030194
[17892]	HIP1	<---	HTT	0.013708	0.020393
[17893]	NRXN1	<---	SYTL2	-0.022343	0.04145
[17894]	PLG	<---	LAMA1	-0.0073623	0.0041402
[17895]	YWHAG	<---	PRKCA	-0.90439	0.90517
[17896]	GRIN1	<---	MAP2K2	-0.32844	0.46752
[17897]	RXRA	<---	KIF1A	-0.14454	0.055094
[17898]	IL18R1	<---	IL18	-0.04452	0.056055
[17899]	THBS1	<---	PLG	-0.18506	0.23708
[17900]	RCVRN	--->	ADRBK1	0.014955	-0.030571
[17901]	PTPN11	<---	LCK	-0.17366	0.056644
[17902]	RARG	--->	SMAD3	0.22689	-0.17818
[17903]	HNRNPA1	--->	NFKBIA	0.8008	-0.80634
[17904]	HDAC3	<---	NCOR2	-0.22984	0.22101
[17905]	KRT9	<---	YWHAQ	-0.057314	0.081652
[17906]	CEBPB	--->	RB1	0.45562	-0.45357
[17907]	ASCL4	--->	TCF12	-0.091025	-0.33696
[17908]	SRF	<---	CASP6	-0.39257	0.32345
[17909]	CDR2	<---	PKN1	-0.061396	-0.051322
[17910]	SH2D1A	--->	ARHGEF6	0.017661	0.013587
[17911]	CTSG	--->	AGT	0.11833	-0.082185
[17912]	HDAC7	<---	YWHAG	-0.050481	0.060077
[17913]	ASCL1	<---	EP300	-0.20391	0.19969
[17914]	ITGB5	<---	MYO10	-0.021851	0.0098233
[17915]	PARD6B	<---	PRKCZ	-0.044435	0.043217
[17916]	TGFB1	<---	ENG	-0.41912	0.26597
[17917]	FGB	--->	NID1	0.059798	-0.055029
[17918]	MYCL1	<---	MAX	-0.43013	-0.38957
[17919]	SMAD5	<---	U2AF2	-0.30655	0.28646
[17920]	HSPA8	--->	REL	0.17406	-0.18344
[17921]	YWHAQ	--->	UBQLN4	0.020149	0.0038986
[17922]	XRN1	<---	SMARCD2	-0.010295	0.014023
[17923]	TP53	<---	ESR1	-0.19275	0.19807
[17924]	MEGF8	--->	GFI1B	0.15154	-0.15552
[17925]	IGSF1	--->	ACVR1	0.021501	-0.03646
[17926]	INPP5D	<---	LYN	-0.52338	0.86692
[17927]	ARHGAP21	<---	PTK2B	-0.029888	-0.022185
[17928]	MAPK3	--->	TSC2	1.0125	-1.0077
[17929]	DLG4	--->	ATP2B2	0.12627	-0.13697
[17930]	NBN	<---	TERF1	-0.00081361	0.034714
[17931]	MMP8	<---	CCL2	-0.16713	0.18806
[17932]	MYO1A	--->	ACTG1	0.53391	-0.53116
[17933]	TGFBR1	--->	CTNNB1	0.22712	-0.23837
[17934]	ABLIM1	--->	ACTG1	0.3346	-0.33787

[17935]	CCNA2	<---	CDK3	-1.0774	1.0544
[17936]	RAPGEF2	--->	RAP1A	1.0152	-1.003
[17937]	RB1	<---	MDM2	-0.1417	0.13056
[17938]	MMP2	<---	EPHB2	-0.30455	0.29717
[17939]	TBK1	--->	TICAM1	0.025166	-0.17891
[17940]	LAT	--->	LCP2	1.006	-0.99741
[17941]	NFKBIA	<---	NCOR2	-0.21016	0.19772
[17942]	PRKCA	--->	CFTR	0.19214	-0.20268
[17943]	DLG4	<---	ERBB2	-0.22341	0.21404
[17944]	TRAF6	--->	TRAF7	0.14764	-0.0030374
[17945]	CRK	--->	IRS2	0.10487	-0.11739
[17946]	SQSTM1	<---	GRB14	-0.086985	0.029576
[17947]	EIF4G1	<---	ZFYVE9	-0.11425	0.10656
[17948]	THRB	--->	RXRA	1.003	-0.99549
[17949]	NCOR2	--->	RXRA	0.080576	-0.1102
[17950]	NOD1	--->	CASP9	-0.038798	-0.12583
[17951]	BTK	<---	HCK	-0.98497	1.0008
[17952]	PPFIA3	<--->	PPFIA2	0.049612	0.049612
[17953]	DTNA	<---	DMD	-0.022378	0.014832
[17954]	ITGAM	<---	THY1	-0.088779	0.077236
[17955]	MYOT	--->	FLNA	0.12245	-0.091736
[17956]	IQGAP1	--->	ACTA1	0.32669	-0.32964
[17957]	CTBP1	--->	HDAC1	0.022723	-0.031773
[17958]	GEMIN5	<---	SMN1	-0.072234	0.086693
[17959]	TNFRSF25	<---	TRADD	-0.082096	0.039901
[17960]	CHD4	<---	HDAC1	-0.0080668	-0.0034483
[17961]	TNFRSF9	--->	TRAF1	0.081714	-0.062073
[17962]	ARL4D	<---	SMURF1	-0.65068	0.64578
[17963]	HNRNPD	--->	IGF2BP2	-0.0048895	-0.020357
[17964]	DLG2	--->	GRID1	0.045953	-0.051152
[17965]	PRPF40A	--->	MAP1LC3A	0.44902	-0.41934
[17966]	CBL	--->	CSF1R	0.10835	-0.11729
[17967]	JAG2	<---	GFI1B	-0.061457	0.048391
[17968]	SHC1	<---	SH2B2	-0.068075	0.053784
[17969]	PAK1	--->	PXN	0.45862	-0.2934
[17970]	CLEC3B	<---	PLG	-0.027944	0.041731
[17971]	WRN	?	BLM	-0.0054159	-0.0054159
[17972]	BLNK	<---	SH3KBP1	-0.19651	0.032417
[17973]	PRMT2	--->	THRB	0.12534	-0.11586
[17974]	ANTXR2	<---	COL4A4	-0.051216	0.048598
[17975]	FZD1	<---	WNT5A	-0.15879	0.15445
[17976]	SHB	<---	SRC	-0.4651	0.82869
[17977]	DAB1	--->	ITGB3	0.030477	-0.03265
[17978]	PYY	--->	NPY2R	0.20502	-0.19468
[17979]	YWHAG	<---	CLK2	-0.66368	0.62776
[17980]	RHOA	--->	DVL2	0.41768	-0.41752
[17981]	SF3B1	--->	PPP1R8	0.062904	-0.02819
[17982]	CTNND1	<---	PTPRM	-0.19511	0.17674
[17983]	IL2RB	--->	STAT3	0.52122	-0.59107
[17984]	PLK4	--->	TGFBR1	0.060991	0.015693
[17985]	PIWIL2	--->	DICER1	0.042243	-0.02712
[17986]	TGFBR1	--->	SMAD1	1.6219	-1.6278
[17987]	PRKCG	--->	PARD6B	0.35239	-0.37011
[17988]	PAK1	--->	ARHGEF6	0.035613	-0.0010098
[17989]	FLT4	--->	ITGB1	0.070176	-0.080719
[17990]	EP300	--->	TP73	0.06489	-0.074886
[17991]	FEM1A	<---	PPM1F	-0.44126	0.46133
[17992]	PTK2B	<---	ERBB3	-0.023945	0.12749
[17993]	PRKCA	--->	VCL	0.86863	-0.86787
[17994]	ITGB4	<---	PRKCA	-0.13119	0.11929
[17995]	FLNA	--->	ITGB7	0.021752	-0.017606
[17996]	PTPRA	--->	BCAR1	0.093327	-0.099054
[17997]	ZNF467	--->	STAT3	0.17629	-0.20444
[17998]	PITPNM2	<---	PTK2B	-0.12585	0.020402
[17999]	MDM4	--->	YWHAQ	0.040034	-0.045818
[18000]	ADRBK1	--->	MC4R	0.1689	-0.18714
[18001]	ABL1	<---	PRKD1	-0.17642	0.11013
[18002]	JAK1	--->	IRS1	1.0117	-1.0143
[18003]	SPTAN1	--->	ACTB	0.085109	-0.097921
[18004]	WWOX	--->	TP53	0.29636	-0.29532
[18005]	TGFB2	--->	APP	0.20523	-0.21568
[18006]	MAPK3	--->	TP53	1.0002	-0.99962
[18007]	NCL	<---	CSNK2A1	-0.33816	0.31892
[18008]	CDK9	--->	SKP2	0.58677	-0.61295
[18009]	ABL1	<---	ABI1	-0.23076	-0.10747
[18010]	ATR	<---	KDR	-0.1478	0.14984

[18011]	CAMK2A	--->	MAPT	0.68291	-0.68964
[18012]	PTPRE	--->	KCNB1	1.2976	-1.2911
[18013]	ESR1	<---	KAT5	-0.06693	0.078305
[18014]	RB1	<---	CDK6	-1.0015	0.98937
[18015]	WT1	<---	FHL2	-0.12631	0.13437
[18016]	CREM	?	CREB1	-0.057915	-0.057915
[18017]	SMAD2	--->	SMAD9	1.0013	-0.9962
[18018]	SELL	<---	CD4	-0.00062328	-1.3545e-005
[18019]	BTK	--->	MYD88	0.3919	-0.42658
[18020]	RB1	<---	CCND1	-1.0044	0.99958
[18021]	TP53BP1	--->	HIST4H4	0.30406	-0.25841
[18022]	TBP	--->	FOS	0.001774	-0.019302
[18023]	MYOD1	<---	CALM3	-0.22666	0.2676
[18024]	PKD1	<---	CTNNA1	-0.31076	0.32289
[18025]	SHC1	<---	PIK3R1	-0.045259	0.0061788
[18026]	PPP2CA	--->	CCNG2	0.30227	-0.30921
[18027]	GSK3B	--->	CREB1	0.96654	-0.95401
[18028]	SPHK1	--->	CTNND2	0.049527	-0.040271
[18029]	SLC9A3R1	<---	SLCO1A2	-0.038024	0.07475
[18030]	DVL3	<---	ZNF408	-0.36803	0.36834
[18031]	PRKACA	--->	GRIA1	1.0055	-0.99542
[18032]	SMARCD3	--->	SMARCC1	0.0079227	0.0072386
[18033]	ADCY5	<---	PRKCA	-0.58968	0.58511
[18034]	TRAF2	--->	SPRY2	0.15623	-0.15051
[18035]	NCOA3	<---	RELA	-0.049581	0.036604
[18036]	NONO	<---	PIN1	-0.30347	0.3333
[18037]	SMAD5	<---	SUV39H1	-0.092045	0.078555
[18038]	SMAD2	<--->	SMAD4	0.68552	0.68552
[18039]	ESR1	<---	CDKN1A	-0.0091886	0.016673
[18040]	GATA1	--->	HEY1	0.31283	-0.29021
[18041]	PARD6A	<---	YWHAZ	-0.090102	0.086336
[18042]	BCAR1	--->	NCK1	0.0049959	0.0034161
[18043]	SMAD1	<---	ANKRD27	-0.050672	0.036468
[18044]	EPN1	--->	REPS2	0.020625	0.013386
[18045]	AKT1	<---	MAP3K11	0.0045156	0.046402
[18046]	TCERG1	--->	HNRNPM	0.19241	-0.17953
[18047]	RTN4RL1	<---	OMG	-0.014789	0.12321
[18048]	ARNTL	?	CLOCK	-0.019553	-0.019553
[18049]	FCHSD2	--->	CCDC85B	0.11805	-0.12415
[18050]	PDGFRB	--->	STAT5B	0.15757	-0.16879
[18051]	PAFAH1B1	<---	MAP2	-0.0040216	0.0080706
[18052]	PAK1	--->	CSNK2A2	1.4008	-1.3014
[18053]	YAP1	--->	TEAD2	0.64247	-0.63315
[18054]	DGKD	--->	IGSF21	0.03212	-0.042942
[18055]	SPEN	--->	PPARD	0.036945	-0.027678
[18056]	GDF9	<---	MOBKL3	-0.047376	0.084264
[18057]	CCBP2	--->	CCL19	0.49595	-0.46475
[18058]	PTPN22	--->	ZAP70	0.10398	-0.33186
[18059]	RAC1	--->	OPHN1	0.70845	-0.67799
[18060]	POU3F3	<---	PPP1R15A	-0.16685	0.1825
[18061]	KAT2B	--->	KLF13	0.14975	-0.15936
[18062]	EDN1	<---	UBQLN4	-0.042769	0.076804
[18063]	EP300	--->	EMB	0.097101	-0.10563
[18064]	RBL2	<---	SUV420H2	-0.30868	0.30066
[18065]	HNF4A	<---	CREBBP	-0.10418	0.094585
[18066]	CCNL2	<---	SFRS2	-1.0044	0.99975
[18067]	SSTR2	--->	GNAI1	0.9872	-0.98627
[18068]	YWHAG	--->	RGS12	0.14075	-0.14004
[18069]	ARHGAP9	--->	RBPMS	0.21372	-0.19978
[18070]	UBE2D3	<---	NEDD4	-0.086802	0.07541
[18071]	ELF1	--->	RB1	0.43649	-0.43615
[18072]	SYK	--->	HCLS1	0.98897	-0.99533
[18073]	SLC2A4	<---	PRKAR1A	-0.7069	0.7312
[18074]	BMPR1B	<---	TGFBR1	-0.077309	0.00058115
[18075]	FADD	<---	PRKCZ	-0.19592	0.15968
[18076]	PTPN1	--->	ESR1	0.83604	-0.82697
[18077]	IRS2	<---	PIK3R3	-0.12533	0.12621
[18078]	CPNE2	--->	ACTB	0.56315	-0.55654
[18079]	ZBTB16	<---	NCOR2	-0.066506	0.053838
[18080]	NPHP1	<---	BCAR1	-0.031524	0.041872
[18081]	SP1	--->	POU2F1	0.17126	-0.18177
[18082]	DEDD	--->	CFLAR	0.61024	-0.37846
[18083]	RB1	<---	GNB2L1	-0.45052	0.44879
[18084]	RASGRF1	<---	NTRK1	-0.10931	0.099393
[18085]	GPS2	<---	HDAC3	0.0027644	0.031275
[18086]	PCDH18	--->	DAB1	0.044793	-0.0090783

18089	[18087]	DAXX	<---	HDAC3	-0.16662	0.1388
18090	[18088]	VAV2	<---	CD44	-0.033722	0.0095541
18091	[18089]	STAT5B	<---	NR3C1	-0.29089	0.27638
18092	[18090]	BAD	<---	YWHAH	-1.0088	0.9978
18093	[18091]	CD36	<---	COL1A2	-0.09189	0.085305
18094	[18092]	BMPR1B	<---	SH3KBP1	-0.021953	-0.0033303
18095	[18093]	CHD3	--->	IKZF3	0.0041182	-0.016712
18096	[18094]	LRP2	<---	INS	-0.41953	0.41853
18097	[18095]	TEP1	<---	PRKCA	-0.56144	0.55687
18098	[18096]	HIPK3	--->	ZNF107	0.63282	-0.59939
18099	[18097]	RPS6KB1	--->	MAPT	0.35614	-0.49081
18100	[18098]	PTPRC	--->	RASA1	0.046394	-0.090838
18101	[18099]	NCSTN	<---	PSEN1	-0.0074558	0.011175
18102	[18100]	DAXX	<---	TCF7L2	-0.309	0.25019
18103	[18101]	NEDD9	--->	CRK	-0.026897	-0.20886
18104	[18102]	KAT2B	--->	HNF1A	0.17193	-0.17199
18105	[18103]	GRIA2	--->	SPTAN1	-0.00046302	-0.0092274
18106	[18104]	MEIS1	<---	HOXC13	-0.0094151	0.024726
18107	[18105]	ARHGEF6	--->	SMAD2	0.11916	-0.11292
18108	[18106]	PTGER3	<--->	PTGER1	0.49511	0.49511
18109	[18107]	RARA	<---	NR0B2	-0.028437	-0.0094276
18110	[18108]	PTPN6	--->	CXCR4	0.53404	-0.34558
18111	[18109]	WIPI1	--->	AR	0.94792	-0.94858
18112	[18110]	NTRK1	--->	RASA1	-0.032431	-0.1896
18113	[18111]	MEIS1	<---	HOXA13	-0.0094151	0.024726
18114	[18112]	SLC9A3R1	--->	CTNNB1	0.49044	-0.48903
18115	[18113]	CCNA1	<---	CDK2	-1.0774	1.0544
18116	[18114]	FGFR1	<---	RPS6KA1	-0.13422	0.089884
18117	[18115]	GRIN1	<---	ACTN2	-0.11049	0.10433
18118	[18116]	RACGAP1	--->	CHN2	0.015634	-0.0015215
18119	[18117]	VDR	<---	NR0B2	-0.028437	-0.0094276
18120	[18118]	PRKACA	--->	FOS	0.53871	-0.68783
18121	[18119]	PIN1	--->	TOP2A	0.14833	-0.11704
18122	[18120]	SRC	--->	GRB10	0.7343	-0.48402
18123	[18121]	NXF1	--->	MAP1A	0.036848	-0.021731
18124	[18122]	CBLN3	<--->	CBLN2	0.012763	0.012763
18125	[18123]	ADAM22	<---	LGI1	0.0004255	0.019707
18126	[18124]	GNA13	<---	MTNR1A	-1.008	1.0129
18127	[18125]	RHOC	--->	DIAPH1	0.10067	-0.10807
18128	[18126]	USP7	--->	KLHL8	0.15347	-0.11723
18129	[18127]	EVL	<---	DNMBP	-0.042401	0.026319
18130	[18128]	HLF	<---	CREBBP	-0.18961	0.18576
18131	[18129]	SP1	<---	NCOR2	-0.044557	0.031184
18132	[18130]	MYL2	--->	TRIM63	0.26544	-0.23222
18133	[18131]	CLK1	--->	YWHAG	0.62776	-0.66368
18134	[18132]	PRSS3	--->	TFPI	0.024385	0.0096062
18135	[18133]	GRM5	<---	HOMER1	-0.11469	0.12984
18136	[18134]	LCK	--->	ESR2	0.53046	-0.4838
18137	[18135]	GNA13	<---	TGFBR1	-0.34227	0.30419
18138	[18136]	DLG4	--->	MAP1A	0.028718	-0.035109
18139	[18137]	PRKCD	--->	IL6ST	0.176	-0.18517
18140	[18138]	PRL	<---	PPIB	-0.076762	0.11148
18141	[18139]	GGA2	<---	BACE1	-0.18968	0.2221
18142	[18140]	YWHAE	--->	BAD	0.9978	-1.0088
18143	[18141]	SLC9A3R1	--->	CFTR	0.48971	-0.48738
18144	[18142]	JAK1	--->	TYK2	0.012969	-0.0068414
18145	[18143]	KPNA1	<---	LMO4	-0.048303	0.081919
18146	[18144]	SRC	--->	PTPRA	0.16479	-0.2369
18147	[18145]	EGF	--->	GRB2	0.033115	-0.045317
18148	[18146]	SPTBN1	<---	SMAD9	-0.018011	0.0041277
18149	[18147]	KCNJ16	?	KCNJ10	-0.01825	-0.01825
18150	[18148]	MINK1	--->	CASKIN1	0.017928	-0.02806
18151	[18149]	TCF7L2	<---	CTNNB1	-1.0087	0.9959
18152	[18150]	FBXW7	--->	NOTCH4	0.13067	-0.14197
18153	[18151]	NXF1	--->	RAE1	0.16628	-0.13408
18154	[18152]	ERG	--->	JUN	0.13316	-0.16285
18155	[18153]	CTBP2	--->	KLF8	0.061778	-0.072132
18156	[18154]	SP1	--->	BRCA1	0.081606	-0.093072
18157	[18155]	EEA1	<---	RAB5B	-0.3573	0.3878
18158	[18156]	PTK2B	--->	VAV1	0.045986	-0.26113
18159	[18157]	NCK2	--->	ABI2	0.10797	-0.063821
18160	[18158]	MAPK10	--->	BCL2L11	0.25961	-0.31312
18161	[18159]	HOXD4	<---	EP300	-0.18943	0.18505
18162	[18160]	SH3GL2	<---	SH3KBP1	0.084895	0.094299
18163	[18161]	ABL2	<---	HCK	-0.78293	0.63857
18164	[18162]	SQSTM1	--->	GRIA1	0.20471	-0.21065

[18163]	INHBB	--->	ACVR1	0.60319	-0.60916
[18164]	TCF12	<---	EP300	-0.040649	0.02796
[18165]	GNAI1	<---	ADRA2A	-0.98627	0.9872
[18166]	HDAC10	<--->	HDAC3	0.015471	0.015471
[18167]	MTCP1	--->	AKT1	0.99137	-0.99692
[18168]	PDCD6IP	<---	SH3GL3	-0.084834	0.099128
[18169]	ATN1	--->	MYST2	0.036611	-0.038848
[18170]	PAK2	--->	RAF1	0.65894	-0.56404
[18171]	HGS	<---	PAK1	-0.10724	0.12951
[18172]	PTPN11	--->	FYN	0.3856	-0.16887
[18173]	NFKBIB	<---	RXRA	-0.17356	-0.03691
[18174]	YWHAB	--->	PIK3R2	0.086177	-0.15766
[18175]	SHOC2	<---	KRAS	-0.0046468	0.015643
[18176]	MACF1	<---	NRCAM	-0.12733	0.11837
[18177]	NR6A1	--->	GNB5	0.034681	-0.0096005
[18178]	PRKCA	--->	ADRA1B	0.3038	-0.31201
[18179]	SMAD2	<---	ST13	-0.32887	0.41177
[18180]	CHAD	--->	COL2A1	-0.0014143	-0.009855
[18181]	HTRA1	<---	COL1A1	-0.012661	0.001081
[18182]	ATXN1	<---	SPEN	-0.022453	0.014613
[18183]	CDK1	<--->	BUB1	0.23429	0.23429
[18184]	FLII	<---	PRMT1	-0.074528	0.070574
[18185]	GPSM2	<---	HRAS	-0.46286	0.49748
[18186]	PTK2B	--->	MCAM	-0.0084763	-0.050374
[18187]	HDAC3	--->	PPARD	0.094064	-0.067996
[18188]	PPP1R15A	<---	LYN	-0.50815	0.6008
[18189]	NCF4	--->	MSN	0.0082852	-0.022076
[18190]	TBP	--->	JUND	0.004785	-0.01957
[18191]	DGKZ	<---	SNTB2	0.0034359	0.0034744
[18192]	YWHAG	<---	MARK3	-0.092392	0.077417
[18193]	RABGGTA	<---	RAB1A	-0.26241	0.27625
[18194]	SKIV2L	--->	XRN1	0.0019114	-0.005057
[18195]	ITGA2B	<---	GRB2	-0.050201	0.038141
[18196]	COMP	<---	FN1	-0.078708	0.11926
[18197]	CDK5	--->	DNM1	0.24668	-0.29072
[18198]	ARL4D	--->	MAPK8IP3	0.089603	-0.09428
[18199]	FAM46A	--->	EGLN2	0.067231	-0.052256
[18200]	CRKL	<---	MAP4K1	-0.125	0.12568
[18201]	EPOR	<---	EPO	-0.021555	0.033493
[18202]	ERCC6	--->	ERCC8	0.090516	-0.076041
[18203]	HOMER2	<---	AGAP2	-0.35058	0.34221
[18204]	DNAJB1	<---	HSPA1A	-0.80138	0.84348
[18205]	TRPV1	--->	CALM1	0.069592	-0.052783
[18206]	SH2D2A	<---	LCK	-0.51928	0.77761
[18207]	HNRNPK	<---	UBE2I	-0.20893	0.24253
[18208]	TEC	<---	BMX	-0.20499	-0.055822
[18209]	CEBPB	?	CEBPD	-0.11448	-0.11448
[18210]	YWHAB	--->	SON	0.046568	-0.037524
[18211]	PRKACA	--->	PRKAR1A	0.27068	-0.33631
[18212]	NRTN	<---	RET	-0.48968	0.48119
[18213]	INSR	--->	IRS2	0.2388	-0.24842
[18214]	ERBB3	<---	SOS1	-0.07959	0.070156
[18215]	PURA	--->	E2F1	0.024073	-0.01031
[18216]	FZR1	<---	SKP2	-0.2343	0.26737
[18217]	SOCS1	<---	KIT	-0.25081	0.15769
[18218]	PREB	<---	TGFBR1	-0.40828	0.3741
[18219]	MAPKAPK5	<---	MAPK13	-0.53091	0.73174
[18220]	NOD1	<---	RIPK2	-0.26618	0.072332
[18221]	JUND	<---	BRCA1	-0.32745	0.29484
[18222]	PRDM1	--->	TLE2	0.0034961	-0.0068753
[18223]	EVL	<---	HCK	-0.66629	0.75322
[18224]	NFKBIZ	<---	NFKB1	-0.041138	0.77463
[18225]	PPP1CC	--->	AURKA	0.055906	-0.072979
[18226]	MYF6	?	ID2	-0.65635	-0.65635
[18227]	CASP3	--->	MAPK8IP3	0.12331	-0.15875
[18228]	LMO3	--->	GABPB1	0.62703	-0.59272
[18229]	GYG2	--->	GYS1	0.089304	-0.084666
[18230]	MAPK9	--->	RB1	0.98937	-1.0015
[18231]	LCK	--->	STAT5A	0.36018	-0.35032
[18232]	NOTCH4	<---	SMAD2	-0.14179	0.1305
[18233]	PRKCD	--->	CREBBP	0.032186	-0.044787
[18234]	SOSTDC1	--->	BMP2	0.43337	-0.38277
[18235]	GNA13	<---	F2R	-1.008	1.0129
[18236]	CREBBP	--->	E2F5	0.18294	-0.18734
[18237]	SMARCA2	--->	KHDRBS1	0.0024909	-0.01497
[18238]	EPAS1	<---	EGLN2	-0.22558	0.21644

[18239]	CDC42	--->	ARHGEF7	0.076055	-0.082042
[18240]	SKP1	--->	FBXO15	0.055251	-0.044878
[18241]	SRC	<---	KDR	-1.0068	0.99457
[18242]	PICK1	--->	GRIK1	0.11658	-0.098237
[18243]	NOS1	<---	ADRA1D	-0.096907	0.08403
[18244]	RGS14	<---	RAP2A	-0.99028	1.0151
[18245]	ITGB2	--->	ITGAX	-0.0059638	-0.0078919
[18246]	CHEK2	--->	CDC25C	0.41667	-0.41209
[18247]	MAPK14	--->	HNF4A	0.1013	-0.17053
[18248]	BMP1	<---	HSPG2	-0.03602	0.023365
[18249]	PSMD10	<---	CDK4	-0.26299	0.25926
[18250]	CDC7	--->	DIAPH1	0.1103	-0.12566
[18251]	YWHAZ	<---	IRS1	-0.049509	0.055105
[18252]	GRIN2B	<---	CAMK2G	-0.30134	0.28954
[18253]	COL8A1	--->	KLHL12	0.062767	-0.06564
[18254]	MAP3K14	<---	BIRC3	-0.28014	0.20869
[18255]	HIPK2	--->	RANBP9	0.41421	-0.43537
[18256]	CDKL1	<---	SMURF1	-0.6445	0.64008
[18257]	LYN	--->	CDK1	1.3862	-1.2776
[18258]	IRS1	<---	CRK	-0.17891	0.16295
[18259]	BIRC6	<---	RAB8A	-0.14947	0.16034
[18260]	SFTPA2	--->	CD93	-0.0091981	-0.027227
[18261]	BCL6	<---	PRKD3	-0.48323	0.47186
[18262]	TBP	<---	RNF4	-0.014318	0.0013715
[18263]	PHOX2A	<---	CREBBP	-0.18943	0.18505
[18264]	GAB1	--->	RET	0.028928	-0.041155
[18265]	RXRA	<---	NFKB1	-0.29278	0.28301
[18266]	SPTB	--->	SPTAN1	-0.0080708	-0.01632
[18267]	SLC9A3R1	<---	LPAR2	-0.41277	0.44636
[18268]	BRD7	--->	BHLHE40	0.42163	-0.40434
[18269]	TNFRSF10A	--->	TRADD	1.0063	-1.0095
[18270]	BRSK2	--->	CDC25C	0.41582	-0.41122
[18271]	BCL2L11	--->	BCL2L1	0.16739	-0.17223
[18272]	SHC3	--->	APP	0.21295	-0.22602
[18273]	THBS1	<---	DCN	-0.20236	0.25625
[18274]	ARR3	<---	FSHR	-0.14161	0.14276
[18275]	PKN1	<---	RHOA	-0.17601	0.06529
[18276]	JAK3	--->	PIK3R1	0.081001	-0.10601
[18277]	NFKBIA	<---	CAPN1	-0.9974	1.0011
[18278]	DUSP4	--->	GRIN2D	0.18879	-0.19473
[18279]	GRIN1	--->	CTNNB1	0.18683	-0.19503
[18280]	UBE2Z	--->	SMAD1	0.56175	-0.59141
[18281]	TNC	--->	ITGA5	-0.0019846	-0.011758
[18282]	BCL2L11	<---	MEX3D	-0.13699	0.13458
[18283]	PRKACA	--->	SPTBN1	0.28219	-0.29697
[18284]	SOX5	--->	SOX6	0.087568	0.083948
[18285]	NCOR2	--->	NR1D1	0.13736	-0.14734
[18286]	MAST2	--->	SNTB2	0.1406	-0.092397
[18287]	PLSCR1	--->	ZBTB16	0.11468	-0.11757
[18288]	AKT1	--->	FOXO1	1.0157	-0.99901
[18289]	SPEN	<---	RBPJ	-0.065874	0.061228
[18290]	ABI3	<---	SOS1	-0.34178	0.33371
[18291]	PRKG1	--->	CRIP2	1.0027	-0.98912
[18292]	LYN	--->	IL7R	0.44363	-0.42775
[18293]	TOM1L1	<---	UBXN4	0.012976	0.021132
[18294]	ACVR2B	<---	INHBC	-0.99943	1.0057
[18295]	ELAVL1	--->	KPNA1	0.042476	-0.012581
[18296]	PECAM1	<---	HCK	0.015127	0.11748
[18297]	USP8	<---	YWHAQ	0.0038773	0.022808
[18298]	TCERG1	--->	HTT	0.18763	-0.17327
[18299]	PRKCH	--->	PTPN11	0.44605	-0.47505
[18300]	HTR6	<---	FYN	-0.47363	0.56481
[18301]	NF2	--->	CD44	0.016243	-0.025631
[18302]	PDLIM5	<---	PRKCB	-0.57553	0.57562
[18303]	MAPK8	--->	PXN	0.57323	-0.59278
[18304]	TNFRSF11A	--->	GAB2	0.24284	-0.25552
[18305]	MYOD1	--->	STAT3	0.35503	-0.40022
[18306]	PLCG1	<---	TEC	-0.98745	0.99957
[18307]	JUN	<---	MAPK10	-0.73927	0.67694
[18308]	CSNK2A1	--->	CTNNB1	0.99917	-0.99713
[18309]	BIRC7	<---	CASP7	-0.38127	0.30534
[18310]	IL7R	--->	STAT5B	0.17619	-0.19577
[18311]	PIK3R1	<---	SOCS6	-0.3011	0.19561
[18312]	FAM46A	<---	HTRA1	-0.0064366	0.011126
[18313]	ATM	<---	CHEK2	-0.1418	0.13005
[18314]	CISH	--->	IL2RB	0.17341	-0.22593

```
[18315] NCF1    <---    FASLG   -0.10929        0.1062
[18316] SMAD2   <---    LAMA5   -0.079301       0.086968
[18317] APPL2   --->    MTA2    0.19655 -0.18261
[18318] UBE2D1  <---    UBE3A   -0.048727       0.045324
[18319] DVL2    <---    SNIP1   -0.24306        0.23764
[18320] MAPK3   <---    DAPK1   -0.14879        0.17015
[18321] DVL2    <---    CCDC33  -0.44039        0.44302
[18322] CDK1    --->    MAP4    0.9565  -0.97405
[18323] CRK     <---    BCAR1   -0.99965        1.0122
[18324] SHC2    <---    NTRK2   -0.20347        0.16097
[18325] WAS     <---    FYN     -1.0011 0.99511
[18326] ST14    <---    HGF     -0.012425       0.00068251
[18327] GAPDH   <---    LAMA4   0.0058959       0.0092233
[18328] PDCD6IP <---    SH3KBP1 -0.14715        0.17965
[18329] TOM1L1  --->    CWF19L1 0.044583        -0.013926
[18330] WDR47   <---    CHFR    -0.18367        0.19721
[18331] MLL2    --->    ASH2L   0.095572        -0.059441
[18332] NFKB1   --->    STAT3   0.10962 -0.12144
[18333] MAPK8IP1        <---    MAP3K10 -0.090912       0.30425
[18334] AR      <---    CCNH    -1.1373 1.1347
[18335] PLD1    <---    RHOG    -1.0031 0.99718
[18336] RUNX2   --->    SMAD6   0.39743 -0.31445
[18337] SOCS1   <---    GRB2    -0.11903        0.22866
[18338] GAD2    <---    PRKCE   -0.69479        0.6277
[18339] OLA1    --->    ASCC2   0.024471        0.0088952
[18340] DLAT    --->    PDHA1   0.20804 -0.19245
[18341] MAPK1   <---    SOS1    -0.40733        0.394
[18342] ITGB5   --->    CYR61   0.09044 -0.10329
[18343] DMD     --->    ACTA1   0.14504 -0.15537
[18344] ERBB4   --->    ERBB2   -0.0016223      -0.0026838
[18345] MAP3K7IP2       --->    NUMBL   0.036126        -0.042683
[18346] ZBTB16  <---    HDAC1   -0.079433       0.073156
[18347] HDAC5   <---    HDAC3   -0.012263       0.022428
[18348] HUWE1   --->    TP53    0.081814        -0.09224
[18349] SPTB    <---    NCAM1   -0.071622       0.07371
[18350] JAK2    --->    SH2B1   0.19985 -0.091396
[18351] GRIN2D  <---    TRAF3   -0.017346       0.0076351
[18352] ZAP70   --->    DEF6    0.56671 -0.34242
[18353] TNIK    <---    RAP2A   -0.035656       0.067479
[18354] CDK9    --->    RELA    1.0015  -1.0004
[18355] STAT3   <---    HES5    -0.24129        0.20842
[18356] MMP16   --->    LRP1    0.076775        -0.087531
[18357] PACSIN3 --->    ADAM15  0.043372        -0.040125
[18358] PITX2   --->    CTNNB1  0.3176  -0.32264
[18359] PTK2B   <---    LCK     -0.24948        0.51061
[18360] C8A     ?       C9      -0.028773       -0.028773
[18361] THRA    <---    TRIM24  -0.088819       0.11471
[18362] ESR2    --->    SMAD3   0.22689 -0.17818
[18363] STK38L  <---    STK24   -0.35503        0.47046
[18364] GALNT2  --->    MUC1    0.17779 -0.17757
[18365] CTNND2  <---    LCK     -0.50618        0.54889
[18366] ZMYND11 <---    EZH2    -0.022146       0.057808
[18367] RHOT2   <---    ZFYVE9  -0.034282       0.022515
[18368] FBXL12  <---    TGFBR1  -0.35222        0.31823
[18369] ZBTB17  <---    CDKN2B  -0.14193        0.1286
[18370] RRAGC   <--->   RRAGB   0.010143        0.010143
[18371] PRKCA   --->    DGKD    0.43581 -0.10826
[18372] PHC1    --->    RNF2    0.14598 -0.12808
[18373] RAC1    --->    FLNA    0.4246  -0.39396
[18374] SPOP    --->    TRAF1   0.0324  -0.0026086
[18375] CUL4B   --->    ESR1    0.11411 -0.10593
[18376] TRIM23  --->    CYTH1   0.14692 -0.14363
[18377] TF      --->    IGFBP6  0.072099        -0.038154
[18378] PRSS3   --->    APP     0.36186 -0.37181
[18379] ATN1    --->    PLSCR1  0.037741        -0.040826
[18380] PIAS1   --->    CEBPE   0.16677 -0.1497
[18381] UMOD    --->    MMP8    0.082055        -0.074496
[18382] ASAP1   --->    PXN     0.045458        -0.050087
[18383] SOS2    --->    PLCG1   0.071806        -0.098834
[18384] RHEB    --->    BRAF    1.0064  -0.99496
[18385] VAV2    <---    CBL     -0.046669       0.0265
[18386] CBX5    --->    SUV39H1 0.013252        -0.019388
[18387] PAK2    --->    SNX33   0.22355 -0.060976
[18388] TSHR    --->    STAT3   0.38001 -0.41743
[18389] ESR1    <---    HSPA4   -0.5448 0.5755
[18390] NFKBIA  <---    MAP3K3  -1.0014 1.0023
```

```
[18391] SPEN    --->    MBD3    0.059803        -0.04498
[18392] FBXL12  --->    SMAD1   0.41909 -0.43793
[18393] ARF4    <---    EGFR    -0.17709        0.17448
[18394] DUSP4   --->    MAPK9   1.0016  -1.0004
[18395] ARF1    --->    ASAP1   0.10177 -0.099189
[18396] DDX5    --->    NCOA3   0.070309        -0.083398
[18397] MX1     --->    TRPC3   0.041026        -0.053855
[18398] ITSN1   --->    DNM1    0.026398        -0.038616
[18399] GRM6    <---    GNAO1   -0.19572        0.21162
[18400] GNAI2   <---    ADRA2A  -0.98627        0.9872
[18401] BCAM    --->    EPB41   0.059162        -0.065371
[18402] SYNJ1   <---    SH3GL2  -0.07064        0.084721
[18403] JAK2    --->    CTLA4   0.17943 -0.11187
[18404] PGR     --->    JDP2    0.18128 -0.18797
[18405] MYST2   --->    BARD1   0.15221 -0.13342
[18406] EED     <---    TGS1    -0.059761       0.07426
[18407] MAP2K5  <---    MAP3K2  -1.0008 0.99307
[18408] ESRRG   <---    ARHGEF15        -0.068206       0.094715
[18409] ARHGEF6 --->    SMAD3   0.11916 -0.11292
[18410] IKBKG   <---    IRAK1   -0.32468        0.33772
[18411] MYB     <---    FBXW7   -0.042899       0.031105
[18412] PLK3    --->    CDC25C  0.33126 -0.32604
[18413] EFEMP2  <---    PLSCR1  0.0049686       0.023845
[18414] NR1H3   <---    NR0B2   -0.024731       -0.0036982
[18415] JAM2    --->    TJP1    0.027639        -0.038512
[18416] COPS5   --->    HAND2   0.83013 -0.8003
[18417] SH3GL3  --->    SNX24   0.10562 -0.091257
[18418] FBLN2   --->    NID1    -0.0085722      -0.017485
[18419] RARB    ?       RXRB    -0.0089162      -0.0089162
[18420] CTNND2  <---    ABL1    -0.060021       -0.037858
[18421] CFL1    --->    ATXN1   0.0095078       -0.017042
[18422] MMP14   <---    F12     -0.073981       0.072802
[18423] PIAS1   --->    PTK2    -0.0055934      -0.039223
[18424] TOLLIP  <---    TOM1    -0.045081       0.049305
[18425] SERPINA5        <---    KLK3    -0.082185       0.11833
[18426] CCR3    <---    FGR     -0.74253        0.90219
[18427] EGFR    --->    GRB2    1.1215  -1.1271
[18428] BCL11B  <---    CBX5    -0.15169        0.14818
[18429] NR1H2   --->    RXRG    1.003   -0.99549
[18430] DVL2    <---    C9orf100        -0.38949        0.38972
[18431] EIF4G3  --->    PABPC1  0.10498 -0.091818
[18432] ACTG1   <---    PFN2    -1.0067 0.99385
[18433] MDM4    <---    ARF1    -0.043676       0.038419
[18434] LUM     --->    ACAN    0.043333        -0.049293
[18435] HSP90AA1        <---    MAP3K3  -0.016598       0.038069
[18436] RAF1    <---    HSP90AA1        -0.025844       0.010135
[18437] RB1     <---    JUN     -0.19293        0.17306
[18438] TAF5    <---    TAF1    -0.06489        0.068015
[18439] CAMK2D  --->    CREM    0.61276 -0.61036
[18440] INPPL1  <---    EGFR    -0.34337        0.3256
[18441] JAK3    <---    PTPRC   -0.14818        0.12992
[18442] DTNA    <---    SNTB2   0.0159  0.020806
[18443] CDH18   ?       CDH6    -0.058742       -0.058742
[18444] MYO10   --->    ACTA1   0.12695 -0.13887
[18445] TGFB3   --->    TGFBR1  0.72054 -0.71391
[18446] ABL1    --->    CREB1   0.0022038       -0.045218
[18447] SPTBN4  <---    CELSR3  -0.0076483      -0.003754
[18448] TRIP6   <--->   FHL3    0.029854        0.029854
[18449] HLF     ?       DBP     -0.11448        -0.11448
[18450] ARFGAP3 --->    SMAD2   0.41389 -0.33099
[18451] PRKCA   --->    LCK     0.16189 -0.15354
[18452] REPS2   <---    RALB    -0.37232        0.40283
[18453] GNA12   --->    CDH15   0.18933 -0.18517
[18454] ACVR1   <---    RRAS2   -0.32935        0.31819
[18455] TTK     --->    AZU1    0.70234 -0.72828
[18456] MAPKAPK5        --->    HSPB1   1.0072  -1.0117
[18457] BAP1    --->    BRCA1   0.047451        -0.049923
[18458] TCF3    <---    CALM1   -0.2977 0.29593
[18459] PRKCB   --->    ADRBK1  0.23691 0.014495
[18460] TGFBR2  --->    PIK3R2  0.2278  -0.26602
[18461] TRAF4   --->    PLSCR1  0.15318 -0.14636
[18462] TGFBRAP1        --->    SMAD2   0.42399 -0.34146
[18463] WEE1    <--->   CSNK2A1 0.23429 0.23429
[18464] CHEK2   --->    AATF    0.3529  -0.3694
[18465] ZBTB5   <---    CCDC85B -0.28095        0.28001
[18466] FOS     <---    CEBPG   -0.41862        0.28837
```

```
[18467] CASP3   --->    BLM     0.35933 -0.43201
[18468] MALT1   --->    BCL10   0.43597 -0.43559
[18469] LAT     --->    CD8A    0.18251 -0.15207
[18470] CAV1    --->    PLD1    0.44764 -0.45653
[18471] SPRY4   <---    TESK1   -0.78218        0.75644
[18472] HNRNPK  <---    LYN     -0.97195        1.133
[18473] GRIA3   <---    PICK1   -0.13456        0.14237
[18474] GRIN1   <---    DLG3    -0.084559       0.076324
[18475] MAP3K14 --->    EGFR    0.29741 -0.32251
[18476] TICAM2  <---    IL1R1   -0.12608        0.12091
[18477] HIST2H4A        <---    BRDT    -0.24229        0.29322
[18478] TRRAP   --->    MYC     0.018643        -0.03054
[18479] BLK     --->    BCL2    0.92083 -0.83157
[18480] SMAD3   <---    BTRC    -1.0017 1.0078
[18481] GRM4    <---    GNA15   -0.19572        0.21162
[18482] SATB1   <---    KAT2B   -0.04931        0.041013
[18483] AR      <---    JUN     -0.55955        0.55069
[18484] TBP     <---    HMGB1   -0.016791       0.004514
[18485] CCL4    ?       IL8     -0.047195       -0.047195
[18486] SRC     --->    ABL1    0.99536 -1.0073
[18487] ZBTB12  <---    HRAS    -0.25472        0.25529
[18488] FBXO30  --->    SMAD4   0.41918 -0.33626
[18489] ATM     <---    ABL1    -0.025238       0.011033
[18490] SRC     --->    IGF1R   0.12772 -0.061466
[18491] COL4A1  --->    COL7A1  -0.0044833      -0.0066409
[18492] RAB3GAP1        --->    WDR7    0.056462        -0.052839
[18493] TANC1   --->    SPTAN1  0.010703        -0.020542
[18494] HCK     --->    BCR     0.36363 -0.25336
[18495] DAXX    --->    MX1     0.089533        -0.11001
[18496] MAPKAPK2        --->    SHC1    0.27879 -0.38681
[18497] PLSCR1  <---    RASD1   -0.37866        0.40294
[18498] CSNK1D  --->    CTNNB1  0.62178 -0.61528
[18499] NAIP    <---    MAP3K7  -0.25783        0.26467
[18500] CDK11B  --->    YWHAB   0.62776 -0.66368
[18501] YWHAB   --->    ITGB1   0.14323 -0.15331
[18502] WDR6    <---    G2E3    -0.21676        0.24992
[18503] SRC     --->    PLCG1   1.0088  -0.9985
[18504] TNIP2   <---    FLNA    -0.0179 0.052165
[18505] EPHA2   --->    CDH5    0.25278 -0.26214
[18506] CDK1    --->    STMN1   1.0058  -0.99021
[18507] DVL3    --->    CSNK2A1 0.99205 -1.0024
[18508] TGS1    <---    EP300   -0.11628        0.10816
[18509] ITK     --->    PPIA    0.70937 -0.75012
[18510] FLT4    --->    PTK2    0.63426 -0.66392
[18511] MAS1    --->    AGT     0.55597 -0.52284
[18512] QKI     <---    PRMT1   -0.146  0.13822
[18513] TREML1  <---    PTPN6   -0.82769        0.97001
[18514] ATP1A1  <---    EZH2    -0.1481 0.1475
[18515] SP1     --->    BCL6    0.038159        -0.096361
[18516] THY1    <---    LCK     -0.83183        0.94256
[18517] CCR1    <---    STAT1   -0.2398 0.11627
[18518] UBE2I   --->    SP100   0.02288 0.0099088
[18519] PCIF1   --->    PDX1    0.4201  -0.38982
[18520] ZBTB43  <---    LMO3    -0.26736        0.26555
[18521] MARK3   --->    ANKRD7  0.13311 -0.14644
[18522] MMP12   <---    F12     -0.073981       0.072802
[18523] EVL     <---    FYN     -0.66629        0.75322
[18524] PPFIA2  --->    PPFIBP2 0.027796        0.0269
[18525] MPP3    <---    RPS2    -0.043104       0.049764
[18526] PDGFRA  --->    STAT1   0.032288        -0.051325
[18527] NCOA1   <---    NR4A1   -0.043163       0.034596
[18528] CAMK2A  --->    SMAD2   1.0038  -1.0107
[18529] AATF    <---    ATR     -0.015565       0.0085869
[18530] CHEK1   --->    RELA    1.0015  -1.0004
[18531] GZMA    <---    HMGB2   -0.17017        0.20338
[18532] NOG     --->    BMP4    0.99953 -1.0058
[18533] SOCS6   <---    TGFBR1  -0.33501        0.30165
[18534] SKP1    --->    FBXO16  0.055251        -0.044878
[18535] BRCA1   --->    STAT3   0.34303 -0.38069
[18536] BAG1    <---    HSPA1B  -0.92976        0.96516
[18537] SETDB1  <---    HIST3H3 -0.15166        0.17341
[18538] FGF1    <---    CSNK2A2 -0.75902        0.73258
[18539] GAPDH   <---    PPM1E   -0.30454        0.32098
[18540] TYK2    <---    CBL     -0.24303        0.23062
[18541] SRPK1   --->    FERMT1  0.5238  -0.54463
[18542] KCNJ9   ?       KCNJ6   -0.01825        -0.01825
```

[18543]	SERPINB8	<---	PRSS1	-0.082185	0.11833
[18544]	USH1C	--->	MYO7A	0.072616	-0.067932
[18545]	CCNA2	--->	RBL1	0.42536	-0.43637
[18546]	RASA3	--->	GNB2L1	0.062361	-0.060571
[18547]	PTK2B	<---	PDPK1	-0.02836	0.034992
[18548]	GIPC1	<---	ADRB1	-0.27482	0.27699
[18549]	SQSTM1	<---	RAD23A	-0.0091048	0.012437
[18550]	GP1BA	--->	GP9	0.023317	0.020939
[18551]	SLC9A3R1	<---	OPRK1	-0.19778	0.21221
[18552]	HOMER1	--->	GRIK1	0.12535	-0.12085
[18553]	CDKN2A	<---	HSP90AB1	-0.076352	0.069468
[18554]	PAK1	--->	FRS2	0.79195	-0.50198
[18555]	ITK	?	BTK	-0.2242	-0.2242
[18556]	PRKCA	--->	PTGIR	0.87512	-0.87377
[18557]	NTRK3	--->	PLCG1	0.22525	-0.29514
[18558]	FN1	--->	IGFBP5	0.024628	0.0040995
[18559]	STAM	<---	PPP1CA	-0.018643	0.023054
[18560]	F2	--->	PROC	-0.0018565	-0.0083748
[18561]	NF2	<---	ERBB2	-0.10988	0.10178
[18562]	ID3	--->	SREBF1	-0.13859	-0.34582
[18563]	SNTG1	--->	DTNA	0.020806	0.0159
[18564]	RIMS2	<---	RAB8A	-0.0067445	0.016184
[18565]	GRASP	--->	GRM5	0.0626	-0.025484
[18566]	ANKRD11	<---	RAC3	-0.36457	0.38119
[18567]	L1CAM	--->	ANK1	0.055421	-0.071273
[18568]	PTPN11	<---	EGFR	-0.25411	0.37037
[18569]	SMAD3	<---	RUNX2	-0.31445	0.39743
[18570]	PRKCZ	--->	NCL	0.10252	-0.11622
[18571]	RHOQ	--->	TRIP10	0.10374	-0.10219
[18572]	ASAP1	<---	PACSIN3	-0.031598	0.028577
[18573]	TGFB2	<---	ENG	-0.30966	0.36037
[18574]	MAP3K3	--->	MAP3K5	0.11998	-0.16749
[18575]	MAST2	--->	STK36	0.070896	0.04976
[18576]	WDR12	--->	NOTCH1	0.70309	-0.72133
[18577]	YWHAQ	--->	ITGB4	0.025286	-0.034638
[18578]	COL9A2	--->	COL2A1	-0.00021365	-0.0060522
[18579]	TP53BP1	--->	RELA	0.11999	-0.11552
[18580]	RMND5A	--->	SMAD9	0.32281	-0.34138
[18581]	NGFR	--->	ZNF274	0.25476	-0.14952
[18582]	ATM	<---	H2AFX	-0.22572	0.2157
[18583]	RPS6KB1	<---	AKT1	-0.15752	0.34577
[18584]	CHUK	<---	PRKCQ	-0.40526	0.42813
[18585]	MYC	<---	EP300	-0.081442	0.071232
[18586]	FRK	--->	RB1	0.40892	-0.38323
[18587]	PAX2	<---	MAPK8IP1	-0.16156	0.16945
[18588]	PRKCD	--->	OPRD1	0.75968	-0.75016
[18589]	SKIL	<---	HEY1	-0.16202	0.15953
[18590]	RAB5A	--->	APPL2	0.30433	-0.29881
[18591]	EPHB2	--->	ABL2	0.0059484	-0.07631
[18592]	MEGF10	--->	DHX16	-0.0067145	-0.0072024
[18593]	ITGB4	<---	MET	-0.068266	0.053858
[18594]	EPHB3	--->	RASA1	-0.0081541	-0.21105
[18595]	HDAC5	<---	YWHAE	-0.08049	0.08119
[18596]	MFNG	--->	JAG1	1.0097	-0.98741
[18597]	PIK3R1	<---	ESR1	-0.16238	0.094794
[18598]	HTR2A	--->	PPP5C	0.036889	-0.041381
[18599]	KPNA2	--->	SLC2A2	0.31889	-0.29424
[18600]	CUL5	<---	TGFBR1	-0.20525	0.17846
[18601]	PKNOX1	<---	HOXD9	-0.0094151	0.024726
[18602]	SEMA4D	<---	PLXNB1	-0.016309	0.055285
[18603]	PARK2	<---	RGS2	-0.33046	0.36045
[18604]	SH3BP2	--->	VAV3	0.0037184	-0.09675
[18605]	SOX10	<---	HOXC4	-0.060464	0.063322
[18606]	NUMB	<---	PRKCD	-0.47333	0.47379
[18607]	PPP1CA	--->	AKAP9	0.20826	-0.21533
[18608]	RASGRF1	--->	RRAS2	1	-1.0025
[18609]	GRIA1	<---	GRID2	-0.12364	-0.012247
[18610]	TNFRSF18	<---	TRAF1	-0.011528	0.069487
[18611]	SHC1	<---	TEC	-0.23274	0.26093
[18612]	DDX54	--->	ESR1	0.042552	-0.032266
[18613]	ZNF165	?	ZNF250	-0.093486	-0.093486
[18614]	YWHAZ	<--->	YWHAE	0.069023	0.069023
[18615]	EGFR	--->	PIK3C2A	0.0079317	-0.018116
[18616]	SERPINF2	<---	CTRB1	-0.082185	0.11833
[18617]	ABL1	<---	GRB2	-0.74268	0.58868
[18618]	BNIP3L	<---	DOK5	0.0022969	0.034488

[18619]	MXD1	?	MAX	-0.65635	-0.65635
[18620]	RERE	--->	RBM9	0.070116	-0.050041
[18621]	GRB10	<---	JAK2	-0.077416	0.26658
[18622]	ZNF148	--->	TP53	0.14131	-0.14061
[18623]	FOXE1	--->	PLCB3	0.37977	-0.37711
[18624]	E2F2	<---	SETD8	-0.10137	0.13519
[18625]	SPINT1	<---	UBQLN4	-0.063485	0.078871
[18626]	HSPA1A	--->	RAP1A	0.45767	-0.41905
[18627]	GNAI3	<---	CD48	-0.15201	0.13687
[18628]	PVRL2	<---	CD226	0.045072	0.05258
[18629]	ADRBK1	<---	SRC	-0.41592	0.42921
[18630]	BCL2L2	<---	PPP1CA	-0.30359	0.29213
[18631]	STAT1	--->	STAT5A	-0.053818	-0.057776
[18632]	GGA1	<---	ARF1	-0.4338	0.46028
[18633]	SMAD7	<---	MAP2K3	-1.0074	1.012
[18634]	AKT1	--->	GAB2	0.062026	-0.15811
[18635]	SOCS1	<---	CXCR4	-0.20208	0.22206
[18636]	MMP2	<---	COL1A1	-0.04494	0.03546
[18637]	ENAH	<---	ABL1	-0.015311	-0.0099442
[18638]	MAPK1	<---	PRKCD	0.043445	0.65991
[18639]	JUP	--->	DSG3	0.07062	-0.077057
[18640]	RBL1	<---	CDK2	-0.47579	0.46185
[18641]	EN2	<---	FOXA2	-0.39346	0.42236
[18642]	BDP1	--->	RB1	0.47968	-0.48114
[18643]	RUNX2	<---	PRKCD	-0.77523	0.78492
[18644]	SKP2	<---	WEE1	-0.61295	0.58677
[18645]	AUP1	--->	ITGA2	0.045804	-0.058139
[18646]	HSP90AA1	--->	IRAK1	0.0039187	-0.0047954
[18647]	IGSF9	--->	ASCC2	0.22354	-0.22908
[18648]	HIPK2	--->	KAT2B	0.11967	-0.14102
[18649]	ACTB	<---	SMAD9	-0.15026	0.12595
[18650]	MARK4	--->	MAPT	0.056794	-0.069929
[18651]	SMAD2	<---	TNNT1	-0.41306	0.494
[18652]	BLNK	--->	PLCG2	0.3384	-0.37715
[18653]	CALM1	<---	GRM4	-0.27991	0.31782
[18654]	MYLK	--->	PRKCA	0.18885	0.18136
[18655]	RABGEF1	<---	RAB4A	-0.038393	0.027632
[18656]	SRC	--->	CD36	1.0269	-0.91422
[18657]	RASA1	--->	SPSB1	0.082536	-0.17742
[18658]	HOXB7	?	HOXB6	-0.11417	-0.11417
[18659]	FOXH1	<--->	FOXG1A	0.3478	0.3478
[18660]	RAB11FIP5	<---	RAB25	-0.10724	0.12837
[18661]	LYN	--->	TRPV4	0.34238	-0.29213
[18662]	CDK4	--->	RBL1	0.46185	-0.47579
[18663]	CAV1	<---	ADRBK1	-0.52164	0.49578
[18664]	MAPK3	<---	PAK2	-1.3014	1.4008
[18665]	NCKIPSD	--->	ITGB1	0.1502	-0.16132
[18666]	FHL2	<---	FOXO1	-0.090133	0.082099
[18667]	GNAS	<---	ADRB3	-0.98627	0.9872
[18668]	FLT1	--->	PTPN11	0.085004	-0.0095962
[18669]	SERPING1	<---	SELP	-0.0063551	0.040239
[18670]	FGFR1	<---	FGF4	-0.99871	1.0053
[18671]	PARD6A	--->	PLCB1	0.17385	-0.1821
[18672]	ITCH	--->	ATN1	0.028854	-0.042091
[18673]	KAT2B	--->	NCOA3	0.079666	-0.092899
[18674]	PRKACA	--->	CCND1	1.0544	-1.0774
[18675]	ALK	<---	PTPRZ1	-0.76248	0.74811
[18676]	PRKCH	--->	PRKD1	0.55695	-0.52651
[18677]	SMARCE1	--->	NCOA1	0.054906	-0.0651
[18678]	CDC42SE2	<---	RAC1	-0.87115	0.57849
[18679]	SPHK1	<---	FYN	-0.90413	1.0649
[18680]	SMAD3	--->	SMAD9	1.0013	-0.9962
[18681]	GGA2	--->	LRP3	0.062428	-0.027973
[18682]	ANKRD11	<---	HDAC5	-0.060221	0.061031
[18683]	UBE2E3	--->	NFKBIA	0.99947	-0.99915
[18684]	SRPK2	--->	SFRS1	0.72994	-0.75755
[18685]	GFI1B	<---	AGRN	-0.040843	0.029101
[18686]	MSN	<---	ICAM1	-0.028035	0.016304
[18687]	SHC1	<---	JAK2	-0.10887	0.053547
[18688]	IKZF5	<---	CCDC85B	-0.31174	0.31077
[18689]	CASP3	--->	ROCK1	0.098051	-0.12151
[18690]	HCK	<---	CBL	-0.7389	0.74409
[18691]	RPH3A	<---	RAB3A	-0.096859	0.11783
[18692]	CREB3	--->	NFIL3	0.16894	-0.015309
[18693]	SLC9A3R1	--->	EZR	0.061503	-0.056659
[18694]	TRAF2	--->	UBQLN4	0.13046	-0.12476

[18695]	CREB3L3	--->	ATF6	0.51642	-0.73364
[18696]	THBS1	<---	FGA	-0.30345	0.39827
[18697]	HSP90AA1	<---	LCK	-0.10417	0.10336
[18698]	PDCD6	--->	FAS	0.13639	-0.029433
[18699]	AKT1	--->	HTT	0.45296	-0.3061
[18700]	SYNGAP1	--->	GRIN1	0.078313	-0.082518
[18701]	MAPK1	--->	PPP1R9B	0.62013	-0.64226
[18702]	YWHAZ	<---	ADRA2A	-0.16522	0.17198
[18703]	SMAD3	<---	CEBPB	-0.28428	0.37299
[18704]	AKT1	--->	IRAK1	0.19598	-0.16806
[18705]	JAG2	--->	NOTCH3	-0.007157	-0.031997
[18706]	IKBKB	--->	RELA	1.0019	-0.99319
[18707]	RAB8B	<--->	RALA	0.43466	0.43466
[18708]	TNFSF11	--->	TNFRSF11B	0.40687	-0.45752
[18709]	CDKN1B	<---	YWHAQ	-0.053185	0.053525
[18710]	CDKN2A	<---	E2F1	-0.068834	0.073896
[18711]	ADCY1	<---	ADCY2	-0.32592	-0.010401
[18712]	F8A1	--->	HTT	0.15451	-0.12143
[18713]	LCK	--->	PIK3R1	1.0077	-0.99637
[18714]	PPP1CC	--->	RAF1	0.045094	-0.061326
[18715]	LAMA3	--->	BMP1	0.0053173	-0.012578
[18716]	PRKCD	--->	EP300	0.032186	-0.044787
[18717]	SH2B3	<---	GRB2	-0.51768	0.50707
[18718]	HTT	<---	PDK2	-0.3104	0.32276
[18719]	TNFRSF1A	<---	PTPN6	-0.246	0.45864
[18720]	HDAC4	<---	MAPK1	-0.71414	0.68838
[18721]	PRKCD	--->	ITGB2	0.041771	-0.056258
[18722]	TP53	<---	CD40LG	-0.35955	0.36542
[18723]	ARHGEF12	<---	GNA13	-0.047502	0.041194
[18724]	CARD9	<---	BCL10	-0.089569	-0.05715
[18725]	ATXN1	<---	BEND2	-0.034989	0.031641
[18726]	CNKSR2	--->	SNTA1	0.042791	-0.058542
[18727]	GRM5	<---	HOMER3	-0.2721	0.27466
[18728]	YWHAG	<---	CBL	-0.49673	0.48957
[18729]	PAK2	<---	NCK1	-0.47107	0.51692
[18730]	HNF1A	--->	CEBPA	0.038752	0.0090572
[18731]	ATN1	--->	MYST4	0.019795	-0.026638
[18732]	JAK2	<---	MST1R	0.088603	0.11457
[18733]	MYO5B	<---	RAB25	-0.23098	0.24373
[18734]	ADAM17	<---	PTPN3	-0.1006	0.088797
[18735]	NLRP1	--->	CASP2	-0.038798	-0.12583
[18736]	EPS15	<---	DNM2	-0.019797	0.0064683
[18737]	MAPK8	--->	STAT3	1.0077	-1.0166
[18738]	SOX10	<---	EGR2	-0.0046139	0.0013215
[18739]	CABIN1	--->	CALM1	0.36034	-0.32295
[18740]	NOG	--->	BMP5	0.99953	-1.0058
[18741]	SMAD7	--->	ZNF107	0.13543	-0.13005
[18742]	CD3D	--->	CD247	-0.087677	-0.26463
[18743]	DLG4	--->	GRIN2D	0.044742	-0.055228
[18744]	VAV1	<---	EPOR	-0.14134	0.020328
[18745]	E2F1	<---	NFKB1	-0.47094	0.4703
[18746]	CDKN2A	<---	WRN	-0.082076	0.088102
[18747]	TRIB1	<--->	MAP2K4	0.23429	0.23429
[18748]	RAB30	--->	SMAD1	0.52759	-0.54836
[18749]	DRP2	--->	UTRN	-0.0030966	-0.0038653
[18750]	FLNA	<---	TLR10	-0.19591	0.20907
[18751]	TCF4	<---	EP300	-0.032499	0.019503
[18752]	SHC2	<---	NTRK1	-0.25539	0.18649
[18753]	DAXX	<---	FAS	-1.0072	1.0095
[18754]	PRKDC	--->	CIB1	0.12075	-0.11531
[18755]	HOXB1	<---	EP300	-0.18943	0.18505
[18756]	PTPN11	--->	STAT5B	1.0012	-1.0146
[18757]	CASP3	--->	MDM2	0.049195	-0.084734
[18758]	TRIM24	--->	ESR1	0.11471	-0.088819
[18759]	OBSCN	--->	ANK2	0.046364	-0.088708
[18760]	DDIT3	<---	SPOP	-0.059758	0.085818
[18761]	PRMT1	--->	TP53	0.23342	-0.23852
[18762]	SLC2A4	<---	FKBP10	-0.36828	0.39543
[18763]	PDPK1	--->	PRSS23	0.42132	-0.27553
[18764]	NEDD9	--->	SMAD2	0.098174	-0.0908
[18765]	EPB41	--->	APP	0.12776	-0.14021
[18766]	RPS6KA1	--->	CREBBP	0.11396	-0.12955
[18767]	ZFYVE16	--->	EEA1	0.042183	-0.0051998
[18768]	YWHAE	--->	TSC2	0.294	-0.29138
[18769]	COPS5	<---	HIF1A	-0.8434	0.84566
[18770]	SQSTM1	--->	GRIA2	0.076448	-0.078954

[18771]	NFKB2	--->	DDX3X	0.0039182	-0.020417
[18772]	RXRA	<---	TMPRSS3	-0.1445	0.011657
[18773]	TAF1	--->	MSX1	0.054267	-0.051435
[18774]	SVIL	--->	KIF14	0.058569	-0.029909
[18775]	ERBB3	--->	MUC1	0.1949	-0.20625
[18776]	PPFIA2	<---	PTPRS	-0.11777	0.11578
[18777]	MYB	<---	FBXW5	-0.015531	0.0053621
[18778]	CCNT2	<---	PKN1	-0.080369	-0.032369
[18779]	CTNNB1	--->	SOX1	0.0049916	-0.023984
[18780]	NID2	<---	COL13A1	-0.035878	0.026339
[18781]	POLR2G	--->	TAF15	0.046624	-0.027336
[18782]	BMPR2	<---	BMP6	-0.99943	1.0057
[18783]	EP300	?	CREBBP	-0.0013284	-0.0013284
[18784]	CASP1	<---	FGF2	-0.013004	0.020913
[18785]	FZR1	--->	SKIL	0.37767	-0.37387
[18786]	MAP3K3	--->	WNK1	0.37019	-0.38123
[18787]	PHF21A	<---	HDAC1	-0.020171	0.035853
[18788]	MAP3K7	--->	FOS	0.163	-0.22125
[18789]	SLC9A3R1	--->	PDGFRA	0.11605	-0.1152
[18790]	PPP1CB	--->	PPP1R12B	0.11026	-0.12181
[18791]	FAM46A	--->	KEAP1	0.073466	-0.040642
[18792]	SMAD2	<---	CUL5	-0.99833	1.0155
[18793]	ADORA2B	--->	EZR	0.095103	-0.098782
[18794]	MAPK7	--->	SGK1	0.23945	-0.1834
[18795]	PXN	<---	SYK	-1.0242	1.0228
[18796]	KCNJ2	--->	DLG2	0.04386	-0.037047
[18797]	SLC9A3R2	<---	PODXL	-0.017662	0.02166
[18798]	ADAM15	<---	SORBS1	-0.00081604	0.0040601
[18799]	NOD1	--->	CASP4	-0.038798	-0.12583
[18800]	KCNQ2	--->	CALM2	0.32976	-0.29233
[18801]	ITGB3	--->	ITGA5	-0.0024803	-0.010331
[18802]	ABCA1	<---	LIN7C	-0.0080068	0.005539
[18803]	ITGA2	<---	COL1A2	-0.014706	0.0009885
[18804]	CCNE1	<---	CDK1	-1.0774	1.0544
[18805]	NOS2	<---	RAC2	-0.51583	0.50819
[18806]	YWHAE	--->	ATXN1	0.045761	-0.051337
[18807]	GAS2	<---	CAPN2	-0.24087	0.24632
[18808]	PPP1R1B	--->	PPP1CA	0.99756	-0.99939
[18809]	RIT2	<--->	HRAS	0.43466	0.43466
[18810]	NR1I2	--->	RXRG	1.003	-0.99549
[18811]	TRAF2	--->	TRIM31	0.14151	-0.13581
[18812]	PTK2	<---	NEO1	-0.26593	0.11016
[18813]	PAK1	--->	ESR1	0.73388	-0.56277
[18814]	HOXB2	<---	EP300	-0.18943	0.18505
[18815]	SFRS4	<---	SFRS2IP	-0.11096	0.12373
[18816]	TRAF2	<---	TNFRSF25	-0.12983	0.093284
[18817]	EVL	<---	ABL1	-0.067735	-0.029754
[18818]	NEDD4L	--->	UBE2E1	0.25514	-0.26042
[18819]	NUP153	--->	SMAD2	0.43042	-0.34737
[18820]	ZHX1	<---	CHD3	-0.014408	0.0028431
[18821]	SH2D2A	<---	ITK	-0.1384	0.2911
[18822]	SMURF2	--->	SOCS6	0.41956	-0.42181
[18823]	SLC8A3	<---	YWHAB	-0.092105	0.10171
[18824]	FBXO7	<---	SKP1	-0.044878	0.055251
[18825]	GLS	<---	PARD6A	-0.16488	0.16064
[18826]	NCK1	<---	DCC	-0.07349	0.048604
[18827]	ATR	--->	TP53	0.074664	-0.082792
[18828]	ATN1	--->	MAGI1	-0.0034443	-0.0085838
[18829]	HDAC2	--->	SP3	0.14006	-0.13401
[18830]	SPTA1	--->	ENAH	0.0027775	-0.015962
[18831]	PPP2R3A	--->	RBL1	0.018076	-0.031661
[18832]	ERBB2IP	--->	ARVCF	0.050618	-0.058533
[18833]	RORA	--->	MYOD1	0.30604	-0.27813
[18834]	CEACAM3	<---	CSNK1A1L	-0.50939	0.587
[18835]	HNRNPC	<---	UBE2I	-0.18951	0.22068
[18836]	NFKBIA	<---	NFKB2	-0.019372	-0.0048972
[18837]	GAB3	<---	CSF1	-0.044914	0.045334
[18838]	PCNT	<---	PCM1	0.0045768	0.029635
[18839]	RELA	<---	TNIP2	-0.087887	0.093652
[18840]	PHOX2A	--->	HAND2	0.49982	-0.46425
[18841]	ACTB	<---	CFL1	-0.30587	0.30201
[18842]	INSR	--->	IRS1	1.0063	-1.003
[18843]	RGS2	<---	HSPA8	-0.36888	0.40666
[18844]	SFRS4	<---	SMURF2	-0.71228	0.70809
[18845]	HDAC5	--->	MEF2D	0.015697	-0.025592
[18846]	GIPC1	<---	NTRK2	-0.14203	0.14349

[18847]	TNF	--->	PRTN3	0.4078	-0.38056
[18848]	NR0B1	--->	SREBF1	0.32119	-0.3185
[18849]	PTK2	--->	ITGB3	0.016172	-0.050274
[18850]	ARHGAP17	<---	SRC	-0.53307	0.62164
[18851]	NR3C2	<---	HSP90AA1	-0.046517	0.037639
[18852]	CASP6	--->	PSEN2	0.58173	-0.69269
[18853]	TCF4	<---	CALM2	-0.33491	0.33066
[18854]	YWHAG	--->	CAD	0.057654	-0.048494
[18855]	LRP6	<---	FZD8	-0.24204	0.23146
[18856]	IKBKB	--->	NFKBIA	1.0136	-1.0087
[18857]	KLF3	<---	CTBP2	-0.11438	0.10635
[18858]	PKMYT1	--->	CCNB1	1.0544	-1.0774
[18859]	PIK3R2	<---	CBL	-0.20723	0.18586
[18860]	EHD1	<---	ZFYVE20	-0.05054	0.065453
[18861]	EED	<---	EZH1	-0.20733	0.24227
[18862]	PIK3R1	<---	DNM1	-0.047631	0.018617
[18863]	SPAG9	<---	MAPK8	-0.16592	0.14982
[18864]	KAT2A	--->	TADA2A	0.12963	-0.12896
[18865]	JUN	<---	DDIT3	-0.43348	0.3977
[18866]	ENO2	<---	UBE2C	-0.10983	0.12469
[18867]	HDAC4	--->	MAP1S	0.080563	-0.045813
[18868]	CFLAR	--->	IKBKB	0.010091	-0.10259
[18869]	CAMK2G	--->	CALM1	0.73919	-0.74572
[18870]	ITGB2	--->	ITGAM	-0.0059638	-0.0078919
[18871]	VEGFA	--->	CTGF	0.17307	-0.18546
[18872]	PAX3	<---	MEOX2	-0.099649	-0.043846
[18873]	CRK	<---	KIT	-0.5027	0.10598
[18874]	NCF1	--->	TRIM9	0.0051689	-0.016899
[18875]	BCAR1	<---	FYN	-1.0003	1.0002
[18876]	BID	--->	MCL1	0.49845	-0.46902
[18877]	TP53	<---	ZNF24	-0.03013	0.024453
[18878]	NEK1	--->	TP53BP1	0.54968	-0.67505
[18879]	NR2C1	?	ESR1	-0.0089162	-0.0089162
[18880]	GTF2I	--->	SMAD2	0.40588	-0.32239
[18881]	AURKA	--->	TP53	1.0002	-0.99962
[18882]	RNF11	--->	RNF168	0.059365	-0.027288
[18883]	EGFR	--->	CDC25A	0.18507	-0.1883
[18884]	CSNK2A1	--->	PTEN	1.0172	-1.002
[18885]	CLEC4A	<---	PTPN6	-0.80298	0.97265
[18886]	NCK1	--->	RHOU	0.40902	-0.38442
[18887]	SYNCRIP	<---	PCSK7	-0.0017254	0.015398
[18888]	SMAD5	<---	RBM4	-0.30655	0.28646
[18889]	BCL10	<---	AKT1	-0.62371	0.74011
[18890]	ZRANB2	<---	EP300	-0.1849	0.18058
[18891]	BMPR1B	<---	AMHR2	-0.18789	-0.093581
[18892]	IQGAP1	--->	CALM1	0.22116	-0.20253
[18893]	RBM7	<---	HMG20A	-0.18319	0.21403
[18894]	NFAM1	<---	SYK	-0.99528	0.9932
[18895]	TP53	<---	KAT5	-0.22051	0.222
[18896]	PLD2	<---	PRKCD	-0.20365	0.1928
[18897]	PIAS3	--->	GFI1	0.093975	-0.096955
[18898]	INPP5D	<---	IL4R	-0.0081723	-0.00072301
[18899]	SH3GLB1	--->	BAX	0.4117	-0.40783
[18900]	SMAD2	<---	SKI	-0.10174	0.14755
[18901]	PRKCZ	--->	RELA	1.0022	-0.99892
[18902]	DLG4	--->	SCN5A	0.047736	-0.049447
[18903]	TNFSF11	<---	AKT1	-0.24707	0.39523
[18904]	MMP14	?	MMP13	-0.12951	-0.12951
[18905]	STK11	--->	MOBKL3	1.0098	-0.97158
[18906]	PPIB	<---	APOB	-0.023689	0.059867
[18907]	PRKCZ	--->	PPP3CA	0.01439	-0.028159
[18908]	ZDHHC17	<---	SYT1	-0.15575	0.17835
[18909]	NCOR1	--->	TBL1X	0.010034	-0.02225
[18910]	TRAF6	--->	PSMD1	0.66932	-0.67615
[18911]	LAMA1	--->	ITGB1	0.046522	-0.058743
[18912]	GNA12	<---	HSP90AA1	-0.038331	0.027855
[18913]	CYTH2	<---	ADORA2A	-0.05575	0.0613
[18914]	NUMB	--->	NOTCH1	0.9955	-1.0002
[18915]	BAZ1A	--->	SATB1	0.0053489	-0.0081871
[18916]	SFI1	<---	CETN2	-0.23404	0.25065
[18917]	SOS1	--->	FGFR1	0.14678	-0.15855
[18918]	TESC	<---	HMG20A	-0.1001	0.13452
[18919]	PBX1	--->	MEIS1	0.024726	-0.0094151
[18920]	GRB2	--->	ESR1	0.22252	-0.21374
[18921]	RPS6KA4	--->	CREB1	1.0082	-0.98976
[18922]	ITGAV	--->	ITGB1	-0.0089044	-0.018744

[18923]	PTK2B	<---	JAK1	-0.17484	0.2558
[18924]	KLKB1	--->	MST1	0.0012258	-0.0091243
[18925]	COL6A2	--->	FN1	0.022104	-0.029703
[18926]	LYN	--->	TEC	1.0008	-0.98497
[18927]	PIK3R2	<---	HCK	-0.70354	0.78626
[18928]	MAP1B	--->	STRAP	0.10297	-0.069521
[18929]	PTK2B	<---	GRB2	-0.43317	0.0087435
[18930]	SPEN	<---	UBE2L6	-0.11721	0.13063
[18931]	LCK	<---	GRAP	-0.050399	-0.025585
[18932]	AKT1	--->	PIK3R1	0.067204	-0.070636
[18933]	ABI2	<---	ABL1	-0.08522	-0.017847
[18934]	IRAK1	--->	PRKCZ	0.068486	-0.015265
[18935]	SMAD9	<---	ACVR1	-1.5009	1.5013
[18936]	C1S	--->	SERPING1	0.04046	-0.023809
[18937]	RANBP9	--->	ITGB2	0.12962	-0.14056
[18938]	HNRNPU	--->	NR3C1	0.080098	-0.054294
[18939]	DDX39	<---	CCL14	-0.0067869	0.010943
[18940]	GHR	--->	STAT3	0.19827	-0.22811
[18941]	GTF3C2	--->	GTF3A	0.072821	-0.070178
[18942]	VDR	<---	STAT1	-0.17447	0.095073
[18943]	CABIN1	<---	PPP3CB	-0.16668	0.16039
[18944]	ZBTB16	<---	FHL2	-0.12348	0.12228
[18945]	RPS6KA1	--->	YWHAB	0.21487	-0.44237
[18946]	GUCY1A2	<---	DLG1	-0.10343	0.10173
[18947]	BCL2	<---	CDK2	-1.2078	1.1791
[18948]	HNF4A	--->	PPARGC1A	0.063942	-0.074404
[18949]	TNF	--->	TNFRSF1A	0.9951	-1.0019
[18950]	RAVER1	<---	ACTN1	0.00046173	0.0031059
[18951]	PTPN6	--->	IL4R	0.48562	-0.37565
[18952]	HINFP	<---	MBD2	-0.035614	0.054911
[18953]	CDKN2C	<---	GDF9	-0.3293	0.36731
[18954]	RGS19	--->	GNAZ	1.0012	-1.0015
[18955]	ILK	--->	AKT1	0.04085	0.0092002
[18956]	TGFB1I1	<---	PTPN12	-0.2966	0.27516
[18957]	SMAD4	<---	EP300	-0.99697	1.0006
[18958]	YWHAG	--->	DHX15	0.045156	-0.035857
[18959]	CEBPG	<---	CREB3L3	-0.015309	0.16894
[18960]	SYNCRIP	<---	SYT2	-0.039791	0.077746
[18961]	UPF2	<---	XRN2	-0.027824	0.026342
[18962]	EREG	--->	ERBB4	0.98479	-0.99014
[18963]	PARD6A	<---	PARD3	-0.032899	-0.0044046
[18964]	CISH	--->	PRLR	0.0057133	-0.017809
[18965]	CASP7	--->	CTSG	0.59854	-0.70957
[18966]	MATN2	<---	COL4A5	-0.028289	0.018624
[18967]	KAT2B	--->	RBPJ	0.01197	-0.021015
[18968]	CAMK2A	--->	SRF	0.30287	-0.31641
[18969]	SNX2	<---	EGFR	-0.32869	0.32794
[18970]	TLX1	--->	MEIS1	0.024726	-0.0094151
[18971]	CUL3	--->	CCND1	0.45733	-0.45665
[18972]	RARA	<---	TRIM24	-0.088819	0.11471
[18973]	GRB2	--->	SH3KBP1	0.29628	-0.29982
[18974]	SNX4	<---	AGK	-0.016245	0.050478
[18975]	NEDD9	--->	CRKL	-0.026897	-0.20886
[18976]	KAT2B	<---	MDM2	-0.022934	0.012285
[18977]	AKT1	--->	XIAP	0.30869	-0.23198
[18978]	NCOR2	--->	RBPJ	0.60125	-0.61067
[18979]	FOXP1	<---	FOXP2	0.023442	0.023813
[18980]	SETDB1	<---	HDAC1	-0.15393	0.15797
[18981]	PPP1CA	--->	PCDH7	0.21789	-0.22494
[18982]	IL16	--->	KCNJ2	0.044422	-0.0070376
[18983]	MAPK1	--->	EPOR	0.44759	-0.47318
[18984]	BLM	--->	TP53	0.21885	-0.21736
[18985]	PIK3R1	<---	CD19	-0.16666	0.10126
[18986]	DAPK1	<---	TNFRSF1A	-0.083357	0.084854
[18987]	MYPN	--->	ACTN3	0.10359	-0.090032
[18988]	MAPK3	--->	SMAD2	1.012	-1.0074
[18989]	CDC42	--->	UBR1	0.27889	-0.24852
[18990]	MAPK1	--->	PPARA	0.99834	-1.0079
[18991]	ABL1	<---	C3	-0.063651	-0.033318
[18992]	ZNF263	<---	PLEKHF2	-0.17825	0.17348
[18993]	RBMX	<---	SORBS3	-0.21698	0.21756
[18994]	IFFO1	<---	RNF183	-0.027024	0.061004
[18995]	BLNK	<---	SYK	-1.0075	1.0124
[18996]	PPP2R1A	<---	PPP2CA	-0.089956	0.079333
[18997]	PLCG1	<---	SH2D2A	-0.53426	0.36686
[18998]	MAPK7	--->	MEF2D	0.08921	-0.12769

[18999]	CTNND2	--->	CDH1	0.61098	-0.61498
[19000]	TSC2	<---	SMAD3	-0.014015	0.024661
[19001]	GRB2	--->	SHC3	0.37019	-0.2872
[19002]	AR	<---	EP300	-0.99484	0.99704
[19003]	OPRM1	--->	DNAJB4	0.21588	-0.20305
[19004]	WNT3A	<---	RYK	-0.70876	0.69175
[19005]	AGTR1	--->	RAF1	0.13207	-0.15654
[19006]	MAPK3	--->	SPIB	1.1285	-1.1212
[19007]	LEPR	<---	PTPN11	-0.47077	0.52911
[19008]	KHDRBS3	--->	KHDRBS2	-0.01402	-0.071085
[19009]	MAP3K7IP1	<---	TRAF2	-0.023197	0.029367
[19010]	PTPN6	--->	SOS1	0.13368	-0.098708
[19011]	MAPK9	--->	SMAD3	1.012	-1.0074
[19012]	ARHGEF7	<---	PAK2	-0.068611	0.1051
[19013]	XRN1	<---	LDHA	-0.0044644	0.0012363
[19014]	BCOR	<---	HDAC1	-0.2276	0.23124
[19015]	CEP72	--->	ZBTB16	0.068653	-0.074397
[19016]	UPF3A	--->	SMG1	0.021991	-0.0061794
[19017]	LCP2	<---	ITK	-0.30539	0.41768
[19018]	PIAS3	--->	STAT3	0.8829	-0.86933
[19019]	MTNR1A	--->	EEF1G	0.46251	-0.43062
[19020]	LTBP1	--->	FN1	0.075938	-0.1137
[19021]	CSNK2A1	<---	HSP90AA1	-0.056762	0.040193
[19022]	TXK	--->	LCP2	1.2119	-0.99119
[19023]	GIPC1	--->	ACTN1	0.0092631	-0.0038511
[19024]	DNMT1	--->	EED	0.13756	-0.12324
[19025]	RGS5	--->	GNAO1	1.0012	-1.0015
[19026]	KAT5	--->	ATM	0.12865	-0.14025
[19027]	TOX2	--->	ZNF461	0.48123	-0.46893
[19028]	SMAD2	<---	PRKAR1A	-0.46649	0.54864
[19029]	FGFRL1	--->	FGF2	0.034867	-0.0036324
[19030]	TNFRSF25	<---	TNFSF12	-0.7724	0.62384
[19031]	CAMK2B	--->	ITPKA	0.3199	-0.34167
[19032]	ZAP70	--->	LCP2	1.0092	-1.0144
[19033]	FHL1	<---	SRF	-0.037159	0.05921
[19034]	AKT1	--->	MDM4	0.41208	-0.39989
[19035]	GATA2	--->	RXRA	-0.0063531	-0.20065
[19036]	BRCA1	--->	H2AFY	0.17426	-0.14798
[19037]	HDAC1	--->	DDB2	0.11313	-0.098152
[19038]	TBL1XR1	<---	HDAC3	-0.044737	0.048214
[19039]	PRKX	--->	PRKAR1A	0.27068	-0.33631
[19040]	PTPN11	--->	RET	0.56725	-0.32395
[19041]	LHX2	--->	MSX1	-0.066587	-0.077101
[19042]	PRKCQ	--->	ICAM3	0.15325	-0.15312
[19043]	SNTG2	<---	DMD	-0.052248	0.048407
[19044]	HSPA13	--->	UBQLN2	0.78427	-0.7421
[19045]	PICK1	<---	ERBB2	-0.27941	0.27529
[19046]	BEGAIN	<---	DLG1	-0.041151	0.041422
[19047]	PPP2R1A	--->	RAP1A	0.13801	-0.13281
[19048]	JUND	--->	FOS	0.17898	-0.31886
[19049]	U2AF2	--->	EPB41	0.029365	-0.025865
[19050]	BAG1	<---	RAF1	-0.078488	0.050429
[19051]	RAB11FIP1	<---	RAB4A	-0.15731	0.20092
[19052]	DDX20	<---	NCOR2	-0.14271	0.13187
[19053]	CDC42	--->	MCF2L	0.0012594	-0.014357
[19054]	SORBS2	--->	DLGAP2	0.20943	-0.2074
[19055]	CIT	<---	RHOC	-0.44942	0.42021
[19056]	ELK1	<---	CREBBP	-0.12289	0.11551
[19057]	SOCS6	--->	IRS2	0.14262	-0.13554
[19058]	PPP1CC	--->	RB1	0.092497	-0.10519
[19059]	TICAM1	--->	TRAF1	0.11411	-0.11915
[19060]	CABLES2	--->	TP53	0.22407	-0.21285
[19061]	RAB34	--->	SMAD4	0.55322	-0.47159
[19062]	RIMS1	--->	SYT1	0.020213	0.01606
[19063]	NFATC4	<---	GATA4	0.0050395	0.0057634
[19064]	ZNF408	<---	PDLIM4	-0.085375	0.10385
[19065]	KLK6	--->	SERPINF2	0.11833	-0.082185
[19066]	SMAD4	--->	RBL1	0.069298	-0.075304
[19067]	FLT4	<---	KDR	0.013594	0.19862
[19068]	PLG	<---	PCOLCE	-0.13445	0.14183
[19069]	PPP3R1	<---	PPP3CA	-0.21268	0.20687
[19070]	FSD1	<---	CDK5	-0.46695	0.4414
[19071]	PDZK1	<---	SLCO1A2	-0.038024	0.07475
[19072]	PDZRN3	--->	BMPR2	0.019703	-0.037206
[19073]	ENO2	<---	SIGIRR	-0.12094	0.13872
[19074]	PGR	<---	CUEDC2	-0.041506	0.066894

[19075]	TNFRSF10A	--->	RIPK1	0.15215	-0.2843
[19076]	TRIM3	<---	UBE2K	-0.073529	0.077458
[19077]	PPP1R9B	<---	CCKBR	-0.19778	0.21221
[19078]	TBC1D4	<---	NAV1	-0.0086462	0.0020679
[19079]	PIAS1	<---	MDM2	-0.02603	0.017751
[19080]	DUSP10	--->	MAPK3	1.0016	-1.0004
[19081]	TGFB1I1	--->	AR	0.34101	-0.35038
[19082]	ZNF446	--->	ZNF496	0.0002948	-0.028208
[19083]	ARHGAP1	--->	HMGCR	0.1875	-0.19169
[19084]	ATP1A1	<---	FXYD7	-0.097228	0.097298
[19085]	PER3	<---	SP1	-0.16009	0.15002
[19086]	CSNK2A1	--->	CDH1	0.39746	-0.41519
[19087]	MYO1C	--->	BAZ1B	0.03468	-0.018988
[19088]	TIAM1	<---	HRAS	-1.0019	0.99931
[19089]	YWHAB	<---	RACGAP1	-0.050693	0.05293
[19090]	CNN2	--->	ACTB	0.51886	-0.51601
[19091]	DSG2	<---	PKP3	-0.319	0.31989
[19092]	MAPK8IP2	<---	DUSP16	-0.31091	0.31547
[19093]	USP7	<---	WWP2	-0.27815	0.27191
[19094]	BAD	<---	YWHAB	-1.0088	0.9978
[19095]	ARID3A	--->	TP53	0.35415	-0.34272
[19096]	SOCS6	--->	TCEB1	0.024927	-0.0040225
[19097]	NOL3	<---	CASP8	-0.74711	0.60453
[19098]	NPHS1	--->	NCK2	-9.4397e-005	-0.021976
[19099]	PTPN13	--->	FADD	0.086782	-0.10685
[19100]	VCL	<--->	CTNNA1	0.57393	0.57393
[19101]	DUSP3	<---	EGF	-0.064303	0.052622
[19102]	ADRB1	--->	SH3GL3	0.13854	-0.14363
[19103]	PRKDC	--->	NR3C1	0.025211	-0.024204
[19104]	CARD8	--->	CASP9	-0.076001	-0.18686
[19105]	NTRK1	<---	NGFR	-0.030739	0.086123
[19106]	SMURF1	--->	STK35	0.64008	-0.6445
[19107]	PSEN1	--->	NOTCH4	0.99563	-0.98992
[19108]	MAP3K14	<---	TRAF5	-0.28436	0.27903
[19109]	GRM2	<---	GNA15	-0.19572	0.21162
[19110]	ZIC2	<---	GLI2	-0.0093948	0.078952
[19111]	NTRK1	<---	MAPK3	-0.045919	0.027697
[19112]	IL4R	<---	IRS1	-0.058838	0.060463
[19113]	CUL4A	--->	SKP2	0.057263	-0.045528
[19114]	IGF2BP1	--->	CTNNB1	0.32562	-0.33043
[19115]	CDK1	--->	LATS1	0.18008	-0.12423
[19116]	RNF2	<--->	BMI1	0.003228	0.003228
[19117]	NR1H4	--->	RXRA	1.003	-0.99549
[19118]	DHX30	--->	RBL1	0.16368	-0.17524
[19119]	CDK3	--->	CABLES1	1.083	-1.1093
[19120]	NCF1	--->	ACTB	0.21028	-0.22311
[19121]	DTNB	<---	UTRN	-0.022378	0.014832
[19122]	NFKB1	--->	NFKB2	1.0089	-0.99422
[19123]	HDAC3	--->	NFKBIA	0.81727	-0.82127
[19124]	MAP3K7IP1	<---	TRAF6	-0.57108	0.56454
[19125]	PRKCG	--->	PICK1	0.99063	-0.98595
[19126]	NUP153	--->	KPNA2	0.10626	-0.0736
[19127]	TNIP2	<---	TNFAIP3	-0.21351	0.21223
[19128]	SMAD3	<---	MAPK8	-1.0074	1.012
[19129]	LRP1	<---	CAV1	-0.15658	0.14712
[19130]	PPFIA1	<--->	PPFIBP1	0.049612	0.049612
[19131]	CCDC33	--->	KHDRBS3	0.094269	-0.07505
[19132]	TEC	?	BTK	-0.2242	-0.2242
[19133]	TIAM1	<---	MYC	-0.12853	0.11929
[19134]	LMO2	--->	MLLT4	0.2596	-0.21095
[19135]	SLC2A4	<---	ACTN4	-0.12341	0.12505
[19136]	ABCA1	--->	ARHGEF11	-0.0052012	-0.006075
[19137]	FZD1	<---	DLG4	-0.089793	0.071296
[19138]	BNIP3L	--->	BCL2L1	0.68788	-0.67957
[19139]	CSNK1A1	--->	HMGB2	0.62328	-0.5331
[19140]	ESR1	<---	MAPK3	-1.0079	0.99834
[19141]	TP63	?	TP73	-0.020631	-0.020631
[19142]	CREBBP	--->	TRIP10	0.104	-0.11254
[19143]	ARHGEF11	<---	RHOA	-0.068467	0.066324
[19144]	RNF11	--->	ERCC6	0.11566	-0.10134
[19145]	PIAS2	--->	BAT1	0.054382	-0.058677
[19146]	FMNL1	--->	PFN1	0.024761	-0.021487
[19147]	RDX	<---	TSC1	-0.015695	0.02248
[19148]	FGF7	<---	COL2A1	-0.025436	0.01529
[19149]	CDK4	--->	TGFBR1	0.36359	0.02614
[19150]	SLA2	--->	CD247	0.51177	-0.58583

[19151]	E2F6	<---	RING1	-0.015216	0.047847
[19152]	GNB2L1	--->	IL2RB	0.010016	-0.0065932
[19153]	GADD45G	--->	CCNB1	0.58381	-0.57034
[19154]	CSNK2A2	--->	SPIB	1.1285	-1.1212
[19155]	ERN1	--->	COPS5	0.28225	-0.30812
[19156]	PTK2B	--->	KCNA2	0.026448	-0.13188
[19157]	ERBB2IP	--->	SMAD7	0.17801	-0.15234
[19158]	DUSP16	--->	MAPK14	0.99169	-0.98663
[19159]	FOXG1	--->	SMAD4	0.52503	-0.44328
[19160]	SFPQ	?	PSPC1	-0.016298	-0.016298
[19161]	TNFSF11	<---	MAPK3	-0.29485	0.27854
[19162]	MAPK1	--->	ARRB1	0.42184	-0.44306
[19163]	SMAD3	<---	HGS	-0.096565	0.086433
[19164]	TRAF2	--->	MAP3K8	1.0023	-0.9866
[19165]	DAXX	--->	TP53	0.15721	-0.17165
[19166]	TXK	--->	CTLA4	0.99605	-0.98986
[19167]	SIGLEC10	--->	FLNA	0.085121	-0.10583
[19168]	RB1	<---	CEBPD	-0.45357	0.45562
[19169]	HRH4	--->	GNAI1	1.0129	-1.008
[19170]	MAP1B	<---	U2AF2	-0.021204	0.053407
[19171]	SH3KBP1	<---	BCAR1	-0.0032617	0.0046398
[19172]	CFTR	<---	PRKCE	-0.21974	0.20067
[19173]	MUC1	<---	ABL1	-0.15799	0.090973
[19174]	PRKAR1A	--->	PLEKHF2	0.070321	-0.063716
[19175]	PTGS2	--->	TP53	0.20598	-0.20446
[19176]	HECW2	--->	TP73	0.072698	-0.083498
[19177]	RGS18	--->	GNAI2	1.0012	-1.0015
[19178]	PTK2B	<---	JAK3	-0.1203	0.21102
[19179]	DNAJB6	<---	USP8	-0.15476	0.14707
[19180]	CCNT1	<---	GRN	-0.36568	0.39925
[19181]	S1PR1	--->	GNAI3	0.46515	-0.46841
[19182]	CCNB1	<---	CDK1	-1.0774	1.0544
[19183]	PRKCB	--->	CARD11	1.0012	-1.0075
[19184]	CBLB	--->	NCK2	0.1941	-0.20757
[19185]	YLPM1	<---	PPP1CA	-0.1932	0.18781
[19186]	TRAF3	<---	TNFRSF1B	-0.15253	0.1203
[19187]	YWHAZ	<---	SIK1	-0.66368	0.62776
[19188]	STAT3	<---	DAXX	-0.35929	0.31567
[19189]	CDH9	?	CDH18	-0.058742	-0.058742
[19190]	BTK	--->	TIRAP	0.68713	-0.72865
[19191]	DLG1	--->	LRP2	0.15231	-0.16223
[19192]	DLG1	--->	GRIN2B	0.044742	-0.055228
[19193]	PATZ1	<---	RNF4	-0.15415	0.14544
[19194]	SNTG2	--->	SCN5A	0.050181	-0.013537
[19195]	TAOK2	<--->	MAP2K3	0.23429	0.23429
[19196]	ATN1	--->	SLIT1	-0.0062944	-0.0067765
[19197]	HIPK3	--->	SNIP1	0.30806	-0.32435
[19198]	NR3C2	--->	ACTG1	0.28724	-0.2846
[19199]	ACTN4	<---	CAMK2G	-0.21773	0.202
[19200]	SYK	--->	VAV2	0.46323	-0.42786
[19201]	KLF2	<---	EP300	-0.12188	0.11387
[19202]	PRKG1	--->	GTF2I	1.0565	-1.0486
[19203]	ARHGAP18	--->	MPP6	0.071153	-0.065536
[19204]	EPB41	--->	CD44	0.016243	-0.025631
[19205]	CDK9	--->	CDC34	0.2372	-0.25823
[19206]	LHX3	?	ISL1	-0.017692	-0.017692
[19207]	MBD4	<---	FADD	-0.14822	0.064192
[19208]	TRIM32	--->	PDE9A	0.27442	-0.27572
[19209]	INPP5D	<---	HCK	-0.52338	0.86692
[19210]	STMN2	<---	RGS6	-0.33305	0.35209
[19211]	COPS6	--->	WIPI2	0.38274	-0.35757
[19212]	CLPB	--->	SMAD9	0.092834	-0.10744
[19213]	PYY	--->	MEP1B	0.31554	-0.27935
[19214]	CSNK2A1	<---	PTPRC	-0.35814	0.35828
[19215]	EPHB2	<---	SRC	-0.076788	0.41298
[19216]	IRS4	<---	PTPN6	-0.28963	0.34979
[19217]	MYL1	--->	EPB41	0.059931	-0.055114
[19218]	DLG1	--->	CALM2	0.16268	-0.16314
[19219]	EIF2AK1	<---	HSP90AA1	-0.056762	0.040193
[19220]	SRC	--->	PTK2	0.9912	-1.0004
[19221]	MAP4K1	--->	LCP2	0.24673	-0.24131
[19222]	HIF1A	--->	PGK1	1.0002	-1.0034
[19223]	SNX4	<---	PDGFRB	-0.046812	0.040725
[19224]	CD22	--->	ATP2B4	0.068209	-0.080639
[19225]	P2RY2	<---	SRC	-0.44703	0.5032
[19226]	RGL1	<---	HRAS	-1.0084	0.98547

[19227]	AKAP9	<---	PRKCE	-0.68332	0.61572
[19228]	AEBP2	--->	EED	0.011152	0.0054936
[19229]	NCOA3	<---	BMP6	-0.11664	0.10588
[19230]	ACP1	--->	CTNNB1	0.56985	-0.56875
[19231]	IL5RA	<---	JAK2	-0.13462	0.16905
[19232]	YWHAZ	--->	ENO1	0.046783	-0.037723
[19233]	ATM	<---	TP53	-0.086307	0.073809
[19234]	IKZF4	<---	HDAC2	-0.050145	0.04822
[19235]	PREX2	<---	TGFBR1	-0.11238	0.091664
[19236]	COL25A1	--->	APP	0.044125	-0.057522
[19237]	SKIV2L2	--->	UPF2	0.014692	-0.020995
[19238]	MMP9	<---	PLG	-0.11212	0.11927
[19239]	GRN	--->	GFI1B	0.1514	-0.15538
[19240]	TNNT1	<---	PPFIA1	-0.17806	0.21145
[19241]	CD4	<---	FCGR3A	-0.27801	0.29204
[19242]	RLIM	--->	LMO2	0.062395	-0.05875
[19243]	SFRS2	<---	UBQLN4	-0.057469	0.089135
[19244]	SMAD5	--->	HOXA13	0.010712	-0.04291
[19245]	AMIGO2	<---	AMIGO1	-0.0082553	-0.0055479
[19246]	THEM4	--->	AKT1	0.99218	-0.99773
[19247]	HNF4G	--->	RANBP9	0.19088	-0.19094
[19248]	CDK1	--->	BIRC5	0.65723	-0.69965
[19249]	GNAI3	<---	CNR1	-0.99753	1.0047
[19250]	PRKAA1	--->	RAF1	0.26426	-0.39064
[19251]	SETDB1	<---	OLFML3	-0.33235	0.34469
[19252]	FZD3	<---	GIPC1	-0.30309	0.27921
[19253]	CCL16	<---	CCR2	-0.46475	0.49595
[19254]	RASGRF1	--->	RHOA	1	-1.0025
[19255]	NFATC2	--->	PPP3R1	0.19616	-0.20162
[19256]	JAK3	--->	JAK2	0.075973	-0.079325
[19257]	PPARG	<---	RANBP9	-0.025636	0.02632
[19258]	PRKCD	--->	STAT3	0.99971	-0.99944
[19259]	ITGAX	<---	C3	-0.057453	0.044893
[19260]	KAT5	--->	ZEB1	0.058263	-0.066345
[19261]	UNC5CL	<---	NFKB1	-0.025875	0.77823
[19262]	PCBP1	<---	RBM11	-0.0048144	0.036464
[19263]	CSNK2A2	--->	MYF5	1.5563	-1.5271
[19264]	PRKCG	--->	GRM5	0.91747	-0.9157
[19265]	TRAF5	<---	CD40	-0.20124	0.19415
[19266]	CSNK2A1	--->	SERPINF1	0.80369	-0.82745
[19267]	DUSP2	--->	MAPK14	0.99169	-0.98663
[19268]	IFNAR1	?	IFNAR2	-0.42827	-0.42827
[19269]	CREBBP	--->	CRX	0.18505	-0.18943
[19270]	EGFR	--->	ELF3	0.21816	-0.22299
[19271]	S1PR2	--->	GNAI1	1.0129	-1.008
[19272]	MRE11A	<---	EP300	-0.18378	0.17945
[19273]	HCK	--->	VAV1	1.0003	-0.99968
[19274]	ARHGAP10	--->	PKN3	-0.019941	-0.077099
[19275]	EPAS1	<---	EGLN3	-0.22558	0.21644
[19276]	NCOR2	--->	AHR	0.16121	-0.17189
[19277]	NTRK2	<---	PTPN1	-0.20877	0.2053
[19278]	TNFAIP3	--->	ZFAND5	0.16762	0.018795
[19279]	TRAF2	--->	RAD23A	0.13046	-0.12476
[19280]	CASP9	--->	RB1	0.27918	-0.28711
[19281]	ARNTL2	<---	EPAS1	-0.008162	0.063735
[19282]	CBX1	<---	TRIM24	-0.030467	0.064029
[19283]	PCBP2	<---	SFRS7	-0.0048144	0.036464
[19284]	ZBTB25	<---	SORBS3	-0.16154	0.15717
[19285]	KDR	--->	CTNNB1	0.10248	-0.11116
[19286]	TBC1D4	<---	RBM12	-0.0068088	3.1379e-005
[19287]	ADAM15	<---	GRB2	-0.16008	0.16265
[19288]	SOCS7	<---	EGFR	-0.58054	0.56683
[19289]	NFKB2	--->	NR3C1	0.0043561	-0.020746
[19290]	IL7R	<---	IL7	-0.16012	0.038334
[19291]	A1CF	<---	TNPO2	-0.037654	0.070134
[19292]	RND1	--->	PLXNB2	0.41051	-0.37902
[19293]	CCNT1	<---	PURA	-0.35788	0.39145
[19294]	E2F2	--->	TFDP1	-0.0088085	-0.064857
[19295]	RGS19	--->	GNAI2	1.0012	-1.0015
[19296]	PRKAR2A	--->	MAP2	0.30212	-0.28702
[19297]	BLM	<---	CHAF1A	-0.0023226	-0.0008092
[19298]	ACVR1B	--->	SNX1	0.35774	-0.39149
[19299]	LASP1	--->	PLSCR1	0.10681	-0.095871
[19300]	PTPN12	--->	CSK	0.0095777	-0.093042
[19301]	EIF4G1	--->	EIF4A1	0.087815	-0.073047
[19302]	RAB6B	--->	TGFBR1	0.010625	-0.037483

[19303]	CFTR	<---	SLC9A6	-0.47826	0.48035
[19304]	EZH2	--->	ATRX	0.058932	-0.068118
[19305]	NRXN2	<---	DDX24	-0.0038891	0.019311
[19306]	FGA	--->	NID1	0.059798	-0.055029
[19307]	KIT	<---	BTK	-0.1651	0.28948
[19308]	ELK1	<---	MOBKL3	-0.12532	0.1637
[19309]	SFN	<---	PLEKHF2	-0.11618	0.11798
[19310]	NACC1	<---	HDAC3	-0.060751	0.09376
[19311]	LYN	--->	CTLA4	0.99605	-0.98986
[19312]	CASP7	--->	MAX	0.95167	-1.0606
[19313]	DOCK8	<---	RAC1	-0.041827	0.031606
[19314]	SSTR5	<--->	SSTR1	0.016768	0.016768
[19315]	SLC12A6	<---	STK39	-0.25753	0.18285
[19316]	MBD3	<---	HDAC1	-0.074216	0.091026
[19317]	HSPA8	<---	JAK2	-0.17291	0.29395
[19318]	CREBBP	--->	GABPA	0.11504	-0.12299
[19319]	SRC	--->	TRAF3	0.19356	-0.17646
[19320]	YWHAG	<---	CLK3	-0.66368	0.62776
[19321]	SYNJ2BP	<---	ACVR2B	-0.17933	0.15108
[19322]	TCF4	--->	CTNNB1	0.020437	-0.032869
[19323]	AKT1	--->	NOS3	0.99914	-1.0011
[19324]	PIM1	--->	CDC25A	0.69298	-0.67632
[19325]	SGK1	<---	NEDD4L	-0.16119	0.11149
[19326]	NOS1AP	--->	LRP1	0.14403	-0.15309
[19327]	ITCH	<---	RIPK1	-0.37477	0.36785
[19328]	CHEK2	--->	BRCA1	0.19191	-0.20845
[19329]	UBE2Z	--->	SMAD4	0.76473	-0.70088
[19330]	ETS2	?	ETS1	-0.13143	-0.13143
[19331]	MAP3K7	--->	MAP2K4	0.99376	-0.98749
[19332]	CDH9	?	CDH6	-0.058742	-0.058742
[19333]	RGS3	--->	GNAI3	0.35662	-0.36228
[19334]	RHOA	--->	ITPR1	0.32796	-0.33789
[19335]	PLCG1	<---	BTK	-0.98745	0.99957
[19336]	COL1A1	<---	COL7A1	-0.0067221	-0.0055036
[19337]	CRKL	--->	IFNAR1	0.36728	-0.4096
[19338]	CDC20	<---	TGFBR1	-0.40828	0.3741
[19339]	FYN	--->	ITPR1	0.99821	-1.0037
[19340]	LHCGR	--->	SLC9A3R1	0.44636	-0.41277
[19341]	CASK	--->	EPS8	0.013444	-0.042972
[19342]	DTX3L	<---	UBE2D3	-0.02412	0.017553
[19343]	KPNA2	<---	CSNK1A1	-0.53648	0.61639
[19344]	PIK3R1	<---	EZR	-0.098879	0.051241
[19345]	PARD6A	<---	CDC42	-0.17886	0.17837
[19346]	WRN	--->	PRKDC	0.0019808	-0.0048476
[19347]	SOX10	<---	PRRX1	-0.022513	0.018113
[19348]	RAC1	<---	NCK1	-0.38442	0.40902
[19349]	BDNF	<---	SORT1	-0.090848	0.085542
[19350]	ACTG1	<---	PTPRO	-0.83206	0.82287
[19351]	HDAC3	<--->	HDAC9	0.015471	0.015471
[19352]	IKBKG	<---	TBK1	-0.56113	0.51929
[19353]	NRXN1	<---	SYT2	-0.043035	0.083467
[19354]	SPTAN1	<---	DDX24	-0.032431	0.022432
[19355]	MDM2	--->	CTBP2	0.028603	-0.040782
[19356]	PTPN12	--->	SMAD5	0.44375	-0.46289
[19357]	CSNK2A1	--->	HCLS1	0.42702	-0.44058
[19358]	PTPN6	--->	GAB2	0.99997	-1.0165
[19359]	PRKG1	--->	RYR1	0.075945	-0.088067
[19360]	DAXX	--->	ETS1	0.048707	-0.067841
[19361]	SETDB1	<---	ORAI2	-0.14682	0.16474
[19362]	SVIL	--->	AMOTL2	0.051229	0.0064384
[19363]	SQSTM1	--->	GRIA3	0.088751	-0.094441
[19364]	HGS	<---	DLG4	-0.062512	0.0028646
[19365]	ARHGAP1	--->	WAS	0.12319	-0.15259
[19366]	E2F6	<---	EZH1	-0.10137	0.13519
[19367]	NCF1	<---	MYBPC3	-0.020189	0.0093042
[19368]	GAS6	<---	TYRO3	-0.1688	0.1665
[19369]	TNFRSF18	<---	TNFSF18	-0.72971	0.73243
[19370]	PDE4D	<---	GNB2L1	-0.040579	0.075733
[19371]	MTA2	<---	MBD3	-0.014778	0.059037
[19372]	AKT1	--->	PDE3B	0.19946	-0.15291
[19373]	HSPA1A	--->	ATXN1	0.21626	-0.21779
[19374]	BAIAP2	--->	DIAPH1	0.0026261	-0.014205
[19375]	RGL4	<---	RAP1A	-0.76787	0.79809
[19376]	RXRB	<---	SPOP	-0.033371	0.044347
[19377]	MMP1	<---	CCL13	-0.16713	0.18806
[19378]	PARD3	--->	TIAM1	-0.0047459	-0.17683

[19379]	TRPV4	--->	MAP7	0.12082	-0.11619
[19380]	DDX17	<---	SNRNP70	-0.047931	0.050627
[19381]	CIAO1	--->	WT1	0.12267	-0.11416
[19382]	FGFR2	--->	PTK2B	0.073624	-0.1825
[19383]	SRI	--->	RYR2	0.21836	-0.22734
[19384]	ERCC8	<---	GTF2H2	-0.11229	0.1451
[19385]	PACSIN1	--->	DNM1	0.082421	-0.091965
[19386]	EIF3B	--->	IL2RB	0.13298	-0.13073
[19387]	MMP9	<---	COL1A2	-0.075308	0.066825
[19388]	PPIC	<---	UBQLN4	-0.065287	0.10133
[19389]	SKIL	<---	NCOR1	-0.074672	0.063442
[19390]	STIM2	--->	PRPF40A	0.16677	-0.1472
[19391]	DVL3	<---	LNX1	-0.23742	0.0029433
[19392]	YWHAG	<---	PHLDB2	-0.17549	0.18747
[19393]	ARNT	<---	EP300	-0.079572	0.069203
[19394]	PTPN18	--->	CSK	-0.065476	-0.14195
[19395]	GRIN1	<---	EPHB4	-0.20791	0.19789
[19396]	NOTCH4	<---	SMAD4	-0.14179	0.1305
[19397]	DUSP10	--->	MAPK14	0.99169	-0.98663
[19398]	HSPG2	--->	FGF2	0.032705	-0.044505
[19399]	CCR5	<---	TXK	-0.74253	0.90219
[19400]	KIF13A	<---	PPP1CA	-0.13401	0.12415
[19401]	MAPK1	--->	NR5A1	0.99834	-1.0079
[19402]	POU3F2	<---	PQBP1	-0.17643	0.18177
[19403]	F9	<---	F2	-0.0065348	-0.005167
[19404]	HMOX2	--->	SETDB1	0.25114	-0.23579
[19405]	CREBBP	--->	SMAD2	1.0006	-0.99697
[19406]	MAPK1	<---	DUSP1	-1.0004	1.0016
[19407]	CBLB	--->	FYB	0.19314	-0.20529
[19408]	JMJD1C	--->	RXRA	0.058149	-0.26546
[19409]	NEDD9	--->	SMAD3	0.098174	-0.0908
[19410]	CD3E	<---	ZAP70	-1.047	1.0695
[19411]	PLXDC1	--->	NID1	0.097902	-0.10809
[19412]	RALBP1	--->	REPS2	0.14798	-0.13774
[19413]	PKP3	--->	DSC3	0.61098	-0.61498
[19414]	RAP2A	--->	MLLT4	0.99712	-0.98672
[19415]	HDAC3	<---	PIAS2	-0.099651	0.11141
[19416]	PRKG1	--->	RAF1	0.99567	-1.0079
[19417]	NGF	--->	MMP7	0.28348	-0.26364
[19418]	CASP3	--->	BID	0.86165	-0.88437
[19419]	GRIN1	<---	TJP1	-0.051152	0.045953
[19420]	TNFSF9	<---	TRAF2	-0.051462	0.058058
[19421]	DMPK	--->	ATN1	0.13466	-0.073681
[19422]	RABGAP1	<---	RAB6A	-0.15297	0.15635
[19423]	SYNJ1	<---	AMPH	-0.07064	0.084721
[19424]	ACP1	--->	SPTAN1	0.1578	-0.16593
[19425]	CREBBP	--->	CTBP1	0.060646	-0.071355
[19426]	TNFRSF14	--->	TRAF3	0.088651	-0.095839
[19427]	AKAP13	<---	YWHAZ	-0.0046727	0.0055202
[19428]	ESR1	<---	BCAR1	-0.020718	0.018185
[19429]	NPAS2	<---	KAT2B	-0.13438	0.1263
[19430]	NR1I2	--->	RXRA	1.003	-0.99549
[19431]	CAPNS1	--->	TERF1	0.099755	-0.1025
[19432]	CTNNB1	<---	MET	-0.3513	0.34524
[19433]	EN1	--->	PAX6	-0.043846	-0.099649
[19434]	TRAT1	--->	CD247	0.097133	-0.1158
[19435]	IL6ST	<---	IL6	-0.24647	0.23419
[19436]	MTNR1A	--->	GNB1	0.87375	-0.84712
[19437]	HSPA8	<--->	HSPA1A	0.94322	0.94322
[19438]	MLXIP	<---	YWHAB	-0.33861	0.32576
[19439]	RPH3A	<---	RAB3B	-0.096859	0.11783
[19440]	CDH10	<---	COPS6	-0.22162	0.2324
[19441]	CSNK2A2	<---	PTPN1	-1.009	1.0061
[19442]	ELK4	--->	ID2	0.12676	-0.084944
[19443]	SERPINI1	<---	PLG	-0.040728	0.057207
[19444]	CEBPG	?	CEBPD	-0.11448	-0.11448
[19445]	PICK1	<--->	GRIP1	0.012784	0.012784
[19446]	AUP1	--->	ITGAM	0.077306	-0.088883
[19447]	PIK3R2	--->	SHC1	0.08236	-0.11472
[19448]	HDAC3	--->	RB1	0.48228	-0.48372
[19449]	KLF6	<---	CELSR2	-0.056632	0.045985
[19450]	LCK	--->	PLCG2	0.1769	-0.32378
[19451]	ROCK1	--->	PFN2	0.026003	-0.041655
[19452]	TNFRSF11A	--->	TRAF2	0.64735	-0.64504
[19453]	MXD1	--->	MYCN	-0.38957	-0.43013
[19454]	TGFBR1	<---	TGFB1	-0.99838	1.0002

[19455]	CCR10	--->	CCL2	0.49595	-0.46475
[19456]	HSP90AA1	--->	NR2C2	0.037639	-0.046517
[19457]	RALGDS	<---	ARRB2	-0.15446	0.14726
[19458]	DIAPH1	<---	RAC1	-0.10807	0.10067
[19459]	UBE2I	--->	UCHL1	0.17702	-0.14333
[19460]	UBE2I	--->	DNMT3A	0.19821	-0.16458
[19461]	SLAMF6	<---	ZAP70	-0.61226	0.61757
[19462]	ZFHX3	<---	PIAS3	-0.062933	0.058114
[19463]	KRT15	<---	WAC	-0.18929	0.20354
[19464]	KCNA4	<---	INADL	-0.013537	0.050181
[19465]	JUN	<---	ATF1	-0.41878	0.14839
[19466]	IRS4	--->	PIK3R1	0.045751	-0.093339
[19467]	RXRA	<---	POU2F1	-0.21576	0.0049626
[19468]	CCL23	<---	CCR1	-0.46475	0.49595
[19469]	AEBP1	--->	TCF3	0.33215	-0.33467
[19470]	CDKN1A	<---	RAB1A	-0.26864	0.27726
[19471]	BAIAP2	<---	NCKIPSD	-0.06953	0.061735
[19472]	NCOR1	--->	ZBTB33	0.0549	-0.064396
[19473]	MAP3K7	--->	STRADB	0.99376	-0.98749
[19474]	NFKB2	--->	RPL30	0.030905	-0.048828
[19475]	LCK	--->	STAT3	0.46642	-0.43959
[19476]	PPP1CA	--->	CUL1	0.096032	-0.10654
[19477]	RASSF4	<---	HMGB1	-0.06826	0.081832
[19478]	UBE2I	--->	SLC2A1	0.55215	-0.52623
[19479]	CBL	--->	FNBP1	0.28637	-0.29554
[19480]	MMP26	--->	MMP9	-0.025733	-0.20541
[19481]	AGAP1	--->	GUCY1A3	0.34095	-0.34945
[19482]	GNA12	--->	ACTB	0.62975	-0.63827
[19483]	DOK1	<---	PTPN6	-0.72183	0.90858
[19484]	DDB2	<---	E2F1	-0.054649	0.067734
[19485]	UNC13B	--->	DOC2B	0.13698	-0.017962
[19486]	SHC3	<---	NTRK1	-0.22613	0.12507
[19487]	SMAD2	<---	TP73	-0.097283	0.12401
[19488]	ABCC9	<---	KCNJ11	-0.059078	0.064029
[19489]	PPP1CA	--->	HSPA4	0.02502	-0.030287
[19490]	ITGAD	<---	VCAM1	-0.027394	0.014008
[19491]	TTF2	--->	HNRNPA2B1	0.0061326	-0.0033008
[19492]	APC	<---	CTBP1	-0.026215	0.012424
[19493]	WT1	--->	TP53	0.085834	-0.089047
[19494]	BAK1	--->	BCL2L1	-0.23385	-0.7984
[19495]	ACAN	--->	MMP2	0.022421	-0.030194
[19496]	AMOT	--->	PLG	0.047179	-0.031505
[19497]	EIF4G1	<---	MKNK1	-0.40182	0.37962
[19498]	TLE1	<---	CDK1	-0.86422	0.83716
[19499]	TNFSF14	--->	TNFRSF14	0.99996	-1.0769
[19500]	IL6	--->	ZBTB16	0.11207	-0.11797
[19501]	CDK9	--->	RB1	0.98937	-1.0015
[19502]	NOXA1	--->	NOXO1	0.05417	0.03092
[19503]	HDAC2	<---	BRCA1	-0.000943	0.005336
[19504]	TRIP4	<---	ESR1	-0.016159	0.041496
[19505]	NCOA3	<---	NR1H3	-0.08303	0.070275
[19506]	FHL2	--->	ITGB2	0.2188	-0.22772
[19507]	TEC	<---	JAK2	-0.10245	0.25294
[19508]	PIAS1	--->	SUFU	0.1805	-0.17716
[19509]	PTK2	<---	CSK	-0.65057	0.81773
[19510]	CASP10	--->	CASP6	0.99569	-0.9907
[19511]	NOG	--->	BMP7	0.99953	-1.0058
[19512]	SHC1	--->	EPS8	-0.0055154	-0.040261
[19513]	RBPMS	--->	HEYL	0.38387	-0.36777
[19514]	DCN	<---	COL4A4	-0.0049704	0.00097089
[19515]	PTPN2	--->	JAK1	0.38802	-0.46748
[19516]	ITGB4	<---	PTK2	-0.028979	0.0021518
[19517]	BRCA1	--->	TRRAP	-0.0055608	-0.0057614
[19518]	COPS5	--->	JUN	0.60169	-0.65167
[19519]	RAC1	<---	TLR2	-1.002	0.99581
[19520]	BRD4	<---	PRPF40A	-0.13954	0.15337
[19521]	RRAS	--->	BCL2	0.9966	-0.99111
[19522]	MAPK1	--->	RPS6KA1	1.002	-1.0109
[19523]	JAK1	--->	IL2RB	1.0065	-1.0011
[19524]	SMAD4	<---	SQSTM1	-0.22445	0.26503
[19525]	PGR	<---	UBR5	-0.10641	0.13236
[19526]	CDK5	--->	AMPH	0.2474	-0.26764
[19527]	LAT	<---	FCGR1A	-0.28312	0.31893
[19528]	KHDRBS3	--->	ZNF408	0.026464	-0.010167
[19529]	LMO3	<---	CCDC85B	-0.011268	0.041664
[19530]	TAF1	--->	CCNA2	0.24062	-0.24459

[19531]	RNF11	<---	NEDD4	-0.16276	0.15319
[19532]	PBX1	?	HOXD10	-0.11417	-0.11417
[19533]	TRIM32	--->	UBQLN4	0.21727	-0.21907
[19534]	KLF4	<---	CREBBP	-0.12188	0.11387
[19535]	PKN1	--->	AKAP9	-0.051287	-0.061428
[19536]	WISP2	<---	IGF2	-0.20183	0.20025
[19537]	PIK3CG	<---	EPHA8	-0.018189	0.014687
[19538]	TRIB3	--->	COPS6	0.46374	-0.49734
[19539]	UHMK1	--->	PAM	0.65556	-0.68136
[19540]	JAK3	--->	CD247	0.25379	-0.25126
[19541]	SPEN	--->	SOX9	0.087082	-0.099478
[19542]	CARD16	<---	RIPK2	-0.4509	0.13986
[19543]	PRKACA	--->	ATP2B1	1.006	-1.0042
[19544]	RBBP4	--->	CHAF1B	-0.087404	-0.10491
[19545]	CDX2	<---	MAPK14	-0.50238	0.33164
[19546]	PLEC1	--->	ACTC1	0.51886	-0.51601
[19547]	TSSK6	<---	HSPA8	-0.99901	1.0063
[19548]	CASP2	<---	CFLAR	-0.40694	0.26698
[19549]	BIRC2	--->	UBE2J1	0.10328	-0.10806
[19550]	SVIL	--->	LIMA1	0.071071	-0.012903
[19551]	HSPA8	--->	HDAC3	0.83174	-0.78946
[19552]	MAP3K1	--->	MAPK1	1.0041	-0.98638
[19553]	JAK3	--->	STAT3	1.0004	-0.98602
[19554]	FBXO44	<---	SKP1	-0.044878	0.055251
[19555]	MAPK14	<---	MAPKAPK2	-0.62285	0.64491
[19556]	PTPN6	--->	ACTN4	0.29245	-0.2308
[19557]	PLEKHG2	<---	RAC1	-0.29753	0.32945
[19558]	PIK3R2	<---	FYN	-0.70354	0.78626
[19559]	RAB27A	--->	ZBTB16	0.20219	-0.20446
[19560]	PTPN11	--->	NTRK2	0.18536	-0.094392
[19561]	MSX1	?	POU2F1	-0.11417	-0.11417
[19562]	PTH1R	<---	ARRB1	-0.22822	0.22496
[19563]	TNFRSF10A	<---	CASP8	-0.38436	0.18641
[19564]	ZFPM1	<---	GATA2	-0.032025	0.054769
[19565]	FYN	--->	TOM1L1	1.0637	-0.91225
[19566]	SH2D3A	<---	BCAR1	-0.034259	0.02891
[19567]	KCNJ3	?	KCNJ6	-0.01825	-0.01825
[19568]	NTRK1	--->	SHC1	0.12173	-0.29661
[19569]	PAX3	<---	WWTR1	-0.34507	0.33998
[19570]	YWHAG	--->	TRA2B	0.092231	-0.070624
[19571]	JAM3	--->	TJP1	0.069964	-0.080827
[19572]	ATF2	<---	HIST2H2BE	-0.33758	0.33912
[19573]	RIPK2	--->	TNFRSF1A	0.10391	-0.13698
[19574]	EDNRB	--->	GNA11	1.0129	-1.008
[19575]	CNN1	--->	CALM1	0.32911	-0.29155
[19576]	DKK1	--->	LRP6	1.009	-1.0005
[19577]	DBP	?	CEBPA	-0.11448	-0.11448
[19578]	NCOA2	<---	DDX17	-0.027033	0.015459
[19579]	PIK3CG	--->	NOTCH1	0.15145	-0.16635
[19580]	HIF1A	--->	CREBBP	0.017606	-0.031011
[19581]	NR0B2	--->	ESR1	-0.0094276	-0.028437
[19582]	EIF3B	<---	EIF4G1	-0.13175	0.16238
[19583]	JAK1	--->	PDGFRB	0.026285	-0.028464
[19584]	HDAC4	<---	MECOM	-0.47211	0.48351
[19585]	SMAD2	<---	HDAC1	-0.21075	0.25575
[19586]	LCK	--->	LIME1	1.0047	-1.0037
[19587]	AKAP1	<---	PRKAR2B	-0.27544	0.28956
[19588]	PRKACA	--->	GJB1	0.53362	-0.54829
[19589]	HAND1	--->	TCF12	-0.091025	-0.33696
[19590]	EPS15	<---	STAMBP	-0.097568	0.086932
[19591]	YWHAG	--->	PIK3C3	0.068249	-0.062439
[19592]	FCAR	<--->	OSCAR	0.45908	0.45908
[19593]	GFI1B	<---	MID2	-0.11018	0.10216
[19594]	NFKB1	--->	BRCA1	0.46973	-0.47039
[19595]	PRKAR1A	<--->	PRKAR1B	0.42105	0.42105
[19596]	GOPC	--->	FZD5	0.27921	-0.30309
[19597]	ATP2B4	<---	RASSF1	-0.21866	0.21097
[19598]	RASSF5	<---	KRAS	-0.98684	1.0025
[19599]	ASAP2	<---	REPS2	-0.037797	0.029539
[19600]	CCR4	<---	ADRBK1	-0.22265	0.20207
[19601]	PWP1	<---	SMURF1	-0.43949	0.43156
[19602]	VCL	--->	ABI2	0.32663	-0.32792
[19603]	BRD8	<---	WEE1	-0.68702	0.66078
[19604]	EED	--->	ITGA4	-0.0014674	-0.0050066
[19605]	FER	--->	CTNNB1	0.078601	-0.092823
[19606]	ARHGEF1	--->	CD44	0.007983	-0.019118

[19607]	GTF2A1	--->	BCL3	0.0979	-0.087623
[19608]	FN1	<---	FST	-0.21547	0.21796
[19609]	CSTF2	<---	UBQLN1	-0.057469	0.089135
[19610]	PRKCE	--->	MYH2	0.01332	-0.028866
[19611]	ZMYND11	<---	EZH1	-0.022146	0.057808
[19612]	HTR2A	--->	GNA11	0.9872	-0.98627
[19613]	BACE1	--->	PSEN1	0.19322	-0.16082
[19614]	HTRA1	--->	BMP4	0.21916	-0.20846
[19615]	SRF	--->	ELK4	0.19917	-0.17689
[19616]	DNM2	<---	GRB2	-0.17195	0.17923
[19617]	CELA1	--->	A2M	0.14985	-0.11653
[19618]	CASP3	--->	PKN2	0.066252	-0.14308
[19619]	PTEN	--->	TP53	0.14492	-0.14077
[19620]	BMPR2	<---	MAP3K8	-0.14114	0.30588
[19621]	NCOA3	<---	RARA	-0.13412	0.12213
[19622]	MAP4K5	<---	ABL1	-0.033903	-0.018045
[19623]	INSR	--->	GAB1	0.13532	-0.14561
[19624]	KCNF1	<---	KCNH1	-0.08117	0.050261
[19625]	CADM1	<---	CASK	-0.061102	0.043926
[19626]	NEDD9	<---	ABL1	-0.028066	0.02193
[19627]	SLC9A2	<---	SPTA1	-0.13122	0.12278
[19628]	PDLIM4	--->	TRIP6	0.017308	0.014007
[19629]	FYN	--->	CD19	1.0564	-1.0123
[19630]	CBL	--->	YWHAB	0.48957	-0.49673
[19631]	MPRIP	--->	PPP1R12C	0.50829	-0.50367
[19632]	MAP4K1	--->	GRB2	0.19604	-0.1786
[19633]	NEDD9	--->	CDH1	0.038728	-0.048892
[19634]	SH2B1	<---	INSR	-0.14528	0.092287
[19635]	PFN2	<---	TERF1	-0.079974	0.11271
[19636]	PTPN2	--->	SHC1	0.34818	-0.56392
[19637]	ELK4	<---	MAPK11	-0.88927	0.89407
[19638]	REM1	--->	YWHAH	0.32694	-0.30645
[19639]	PITX1	<---	NR5A1	-0.073763	0.080302
[19640]	CORO1A	--->	ACTB	0.4503	-0.44823
[19641]	DNMT3A	--->	ZBTB17	0.25942	-0.27176
[19642]	SMAD3	--->	MYOD1	1.0041	-1.0094
[19643]	AURKA	--->	BRCA1	0.32242	-0.34057
[19644]	DNMBP	--->	ACTA1	0.083425	-0.10118
[19645]	YAP1	--->	TEAD4	0.64247	-0.63315
[19646]	DLG4	?	DLG3	-0.0075176	-0.0075176
[19647]	GDF9	<---	STRN4	-0.079162	0.11705
[19648]	THBS1	<---	COL7A1	-0.022867	0.014315
[19649]	CSNK1E	--->	OCLN	0.72084	-0.58422
[19650]	LILRB4	<---	PTPN11	-0.17152	0.29453
[19651]	HNF1A	--->	STAT3	0.54851	-0.60203
[19652]	PLCG1	<---	LIFR	-0.91995	0.87208
[19653]	PPARGC1A	--->	PPARG	0.0055204	-0.01571
[19654]	GFI1B	<---	COPS5	-0.18745	0.18243
[19655]	PDPK1	--->	SGK2	1.0929	-1.1695
[19656]	ISL2	?	LHX3	-0.017692	-0.017692
[19657]	ACVR1	--->	DCAF6	0.082233	-0.10071
[19658]	CNGA2	--->	SLC9A3R1	0.21309	-0.1966
[19659]	PAK2	--->	EIF4G1	0.52649	-0.24747
[19660]	MMP13	<---	F12	-0.073981	0.072802
[19661]	PTPRC	--->	SKAP1	0.14419	-0.15643
[19662]	TP53	--->	SMAD2	0.016573	0.007933
[19663]	TP53	<---	NR3C1	-0.19275	0.19807
[19664]	CDX1	--->	CEBPA	0.038752	0.0090572
[19665]	ARHGAP29	<---	PTPN13	-0.094942	0.083439
[19666]	EGFR	<---	AREG	-1.0074	0.99592
[19667]	GPS2	--->	CCNA1	0.63857	-0.62524
[19668]	NCK2	--->	DOK1	0.26634	-0.27569
[19669]	CDKN2A	--->	ZNF227	0.049559	-0.057083
[19670]	UPF2	<---	EIF4A1	-0.020995	0.014692
[19671]	RFX1	--->	ABL1	-0.028934	-0.034979
[19672]	CASK	--->	EPB41	0.053423	-0.067739
[19673]	FGFR3	<---	FGF17	-0.61087	0.60861
[19674]	GJB6	?	GJB2	-0.032421	-0.032421
[19675]	TIMP4	--->	MMP2	0.21399	-0.19321
[19676]	ZAP70	--->	LAT	0.99842	-1.0126
[19677]	MINK1	--->	PKP4	0.045293	-0.050602
[19678]	CAV1	<---	BTK	-0.77363	0.706
[19679]	SUFU	<---	RCN3	-0.2124	0.21167
[19680]	EP300	--->	SMAD2	1.0006	-0.99697
[19681]	PIAS4	--->	SKIL	0.26978	-0.27359
[19682]	AP3D1	<---	ARF6	-0.40724	0.43364

```
[19683] ESR1     <---    PIK3R3  -0.016463       0.0028628
[19684] RAD54B   <---    CCDC33  -0.017795       0.036811
[19685] SIT1     <---    FYN     -1.0988 1.2193
[19686] ADAM17   <---    TNF     -0.11106        0.11277
[19687] SRC      --->    DOK2    1.2026  -1.146
[19688] RIMS2    <---    RAB3C   -0.0067445      0.016184
[19689] NINL     --->    ZNF646  0.28921 -0.28102
[19690] ZNF165   <---    CCDC85B -0.31174        0.31077
[19691] MTNR1A   --->    FLNA    0.47769 -0.44658
[19692] NEDD9    --->    BCAR3   0.053649        -0.07171
[19693] COPS6    --->    NR3C1   0.21292 -0.19181
[19694] FBXW8    --->    CUL7    0.013956        -0.011438
[19695] APP      <---    MAPK8IP2        -0.22947        0.21795
[19696] KSR1     --->    MARK3   0.0028075       -0.0066004
[19697] FLOT1    <---    FYN     -0.94604        1.1072
[19698] PACSIN2 --->    WASL    0.092082        -0.11591
[19699] PTK2     --->    TP53    0.13558 -0.18037
[19700] CD4      <---    TNFRSF10A       -0.047854       0.15082
[19701] SMAD4    <---    TGFBR1  -1.0087 1.0115
[19702] CDH19    ?       CDH6    -0.058742       -0.058742
[19703] TNR      --->    ACAN    -0.0018788      -0.011468
[19704] CAV1     --->    FLNA    0.032447        -0.0045083
[19705] NGFR     <---    MAPK1   -0.27811        0.23445
[19706] STAT1    --->    STAT3   -0.050869       -0.095384
[19707] ZBTB16   <---    HDAC9   -0.121  0.11954
[19708] SHC1     <---    ERBB3   -0.987  0.99728
[19709] TRAF3    <---    TRAF5   -0.029555       -0.00095543
[19710] RANBP10 <---    MET     -0.23016        0.21784
[19711] TLR4     --->    MAPK8IP3        0.069139        -0.102
[19712] PBK      --->    DLG1    0.15126 -0.17925
[19713] KAT5     --->    AR      1.0146  -1.0053
[19714] IRS2     <---    JAK1    -0.9987 0.99718
[19715] PSMD4    --->    MYOD1   0.60154 -0.56402
[19716] DOCK2    --->    CRKL    -0.0057038      -0.0080124
[19717] ARHGAP1 <---    SRC     -0.53101        0.61959
[19718] FCER1G   <---    KIR2DL4 -0.4895 0.47597
[19719] CUBN     --->    LRP2    0.14449 -0.15358
[19720] SMURF1   --->    TNNT1   0.696   -0.70097
[19721] SERPINE1        <---    IGFBP5  -0.028727       0.064867
[19722] TIRAP    --->    IRAK2   0.29323 -0.1123
[19723] ABI1     --->    SOS1    0.0045411       -0.021566
[19724] TP53     <---    CCL18   -0.33767        0.34912
[19725] UTRN     <---    PTPN12  -0.11216        0.1008
[19726] PPARD    <---    PEBP1   -0.029851       0.055828
[19727] GRIK4    <---    GRIK2   -0.080356       -0.020824
[19728] ANK2     --->    SPTBN1  0.027238        -0.040471
[19729] DSG1     --->    DSC2    -0.018045       -0.093588
[19730] POU5F1   <---    HMGB2   -0.26954        0.30083
[19731] NCK2     --->    DNM1    0.079581        -0.084778
[19732] MAPK1    --->    KHDRBS1 0.35791 -0.37587
[19733] HOXA9    --->    PBX2    -0.071956       -0.072035
[19734] SMAD2    <---    ESR2    -0.17818        0.22689
[19735] CDK6     --->    CDKN1A  0.51838 -0.55156
[19736] PABPC1   --->    PXN     0.21252 -0.20664
```

```
[Section 4] Predictions on NetPath-PI (physical PPIs)

[TGFBeta]
	NUP214--->SMAD3
	SMAD3--->E2F4
	SMAD4--->ATF2
	CDK2--->CDKN1A
	TGFBR2--->SMAD7
	TGFBR1--->PPP2R2A
	TGFBR3--->TGFB1
	NUP214--->SMAD2
	ESR1--->SMAD2
	SMAD3--->RBL1
	TGFBR2--->DAXX
	MAP2K6--->SMAD7
	EP300--->SNIP1
	CREBBP--->SMAD3
	IRF2BP1--->TGIF1
	TGFBR2--->SMAD7
	DDX5--->SMAD3
	CDK2--->CCNE1
	SMAD3--->SP1
	JUND--->SMAD4
	CREBBP--->SNIP1
	TGFBR2--->TGFBR1
	CCNE1--->RBL1
	BTRC--->SMAD3
	SKI--->SMAD3
	TGFBR2--->TGFBR3
	SMAD2--->ZEB1
	KAT2B--->SMAD2
	TRAF6--->MAP3K7
	EP300--->SMAD2
	E2F4--->RB1
	RBX1--->SMAD3
	TGFB3--->TGFBR2
	SMAD4--->RBL1
	HDAC1--->SMAD2
	HNF4A--->SMAD4
	MAP4K1--->MAP3K7
	TGFBR1--->TRAP1
	TRAP1--->SMAD4
	CDKN1A--->CCND1
	HGS--->SMAD3
	E2F5--->TFDP1
	DAB2--->SMAD2
	BRCA1--->SMAD3
	TGFBR2--->PIK3R2
	PRKAR2A--->SMAD3
	ATF3--->SMAD3
	TGFBR1--->SMAD2
	TGFBR2--->ZFYVE9
	TGFBR1--->SMAD3
	SMURF2--->SMAD2
	ERBB2IP--->SMAD4
	TGFBR1--->SMAD6
	FOXH1--->SMAD3
	SMAD3--->E2F5
	SMAD2--->SP1
	EP300--->SMAD7
	PARD3--->SMAD7
	ANAPC2--->FZR1
	JUNB--->SMAD4
	SMAD4--->SP1
	TGFB1--->TGFBR1
	SKI--->SMAD2
	SMAD3--->SP1
	SMAD4--->SKIL
	SMAD2--->DVL1
	TGFBR2--->EIF3I
	ESR1--->SMAD4
	PARD3--->SMAD3
	SPTBN1--->SMAD4
	JUND--->SMAD3
	TP73--->SMAD3
	STRAP--->SMAD2
```

E2F4--->RBL1
JUN--->SMAD4
TGFBR2--->PIK3R1
TP53--->SMAD2
TGFBR1--->ENG
TGFBR2--->ENG
ZEB2--->SMAD3
TGFBR1--->FNTA
SMAD7--->CTNNB1
DYNLRB1--->DYNC1I1
PTK2--->PXN
SPTBN1--->SMAD3
CREBBP--->SMAD4
SKI--->SMAD4
TGFBR2--->TGFBR3
ESR1--->SMAD3
STAMBPL1--->SMAD2
GIPC1--->TGFBR3
SMAD3--->CTNNB1
E2F5--->RBL2
E2F5--->RBL1
STRAP--->SMAD6
TRAF6--->TGFBR1
TGFBR2--->SNX6
SMAD3--->AXIN2
HGS--->SMAD2
ZEB2--->SMAD2
TGFB1--->TGFBR2
E2F4--->RBL2
TGFBR1--->CD44
TGFBR2--->TGFBR1
EP300--->SMAD3
BCAR1--->CRK
TGIF1--->SMAD2
ROCK1--->CDC25A
SKI--->SMAD3
E2F4--->TFDP1
TGFBR1--->DAB2
SMAD3--->FOS
SMAD4--->DVL1
SMAD3--->AXIN1
E2F4--->RBL2
SMAD2--->SKIL
TP73--->SMAD2
SOS1--->SHC1
UBE2I--->SMAD4
MEF2C--->SMAD2
TGIF1--->JUN
TGFBR2--->TRAP1
SMAD7--->DVL1
TGIF1--->SMAD2
TGFBR3--->TGFB2
SMURF1--->SMAD7
DAB2--->SMAD3
JUN--->SMAD2
E2F4--->RBL1
COPS5--->SMAD4
JUNB--->SMAD3
TGFBR2--->EIF3I
CUL1--->SMAD3
ETS1--->SMAD3
TGFBR1--->PARD6A
FOXO4--->SMAD4
PRKAR1B--->SMAD3
TGFBR2--->CTNNB1
JUND--->FOSB
E2F4--->TFDP1
SMURF1--->SMAD7
MAP2K3--->SMAD7
NUP214--->SMAD4
EP300--->NCOA1
JUND--->FOSB
TGFBR1--->STRAP
HNF4A--->SMAD3
TGFBR2--->CCNB2
BTRC--->SMAD4

```
SMAD3--->FOS
STAMBPL1--->SMAD7
E2F5--->TFDP1
SMAD3--->E2F5
MAPK14--->SMAD7
FOXO4--->SMAD3
TGFBR1--->SNX6
ERBB2IP--->SMAD7
SMAD3--->AR
SMAD3--->ZEB1
SMAD4--->HOXA9
CTCF--->SMAD4
RUNX2--->SMAD3
JUND--->SMAD3
SMAD2--->MYC
TGFBR1--->SMAD7
HDAC1--->TGIF1
PRKAR1B--->SMAD4
TGIF1--->JUN
TGFBR1--->PIK3R1
GRB2--->SHC1
CDK2--->RBL1
FZR1--->SKIL
KAT2B--->SMAD3
TGFB1--->TGFBR2
CDK2--->RB1
PARD3--->SMAD4
E2F5--->RBL2
TGFBR1--->PIK3R2
STRAP--->SMAD7
PARD3--->SMAD2
FOXH1--->SMAD2
EP300--->SMAD4
SMAD3--->SKIL
EP300--->SMAD3
SKI--->SKIL
CTCF--->SMAD3
NUP153--->SMAD3
TGFB2--->TGFBR2
SMAD4--->SP1
JUN--->FOS
PRKAR2A--->SMAD4
SMAD3--->ATF2
TGFBR1--->ZFYVE9
E2F4--->TFDP2
SMAD2--->SKIL
RUNX2--->SMAD2
TGFBR3--->TGFB1
HDAC1--->TGIF1
TGFBR1--->STRAP
SMURF2--->TGFBR1
MEF2A--->SMAD2
SMAD4--->CTNNB1
ZFYVE9--->SMAD2
ERBB2IP--->SMAD2
TP53--->SMAD3
TGFB1--->ACVRL1
TGFBR2--->CCNB2
JUND--->SMAD4
SMAD3--->SKIL
TGFBR2--->ACVRL1
TGFBR2--->DAB2
TGFB1--->TGFBR1
SNIP1--->SMAD4
RHOA--->CAV1
SMAD3--->RBL1
SNIP1--->SMAD2
SMAD2--->AXIN1
YAP1--->SMAD7
SMURF2--->SMAD7
SMAD3--->MYC
ERBB2IP--->SMAD3
TGFBR1--->CAV1
SMAD3--->E2F4
PPM1A--->SMAD2
HGS--->SMAD2
```

```
        STRAP--->SMAD3
        SP1--->ETS1
        NUP153--->SMAD2
        ARRB2--->TGFBR3
        RNF111--->SMAD7
        VDR--->SMAD3
        JUN--->SMAD3
        HDAC1--->RBL2
        SMAD3--->DVL1
        TGFBR3--->TGFB3
        PDPK1--->STRAP
        TGFBR1--->SMAD2
        FOXH1--->SMAD4
        TGFBR2--->STRAP
        TGFBR1--->SMAD7
        TGFB2--->TGFBR1
        SMURF2--->SKIL
        ZFYVE9--->SMAD3
        MAP3K7--->SMAD7
[TNF]
        PPM1B--->MAP3K7
        RIPK1--->PTK2
        IKBKB--->RELA
        TRAF6--->PSMD1
        FADD--->CASP8
        BIRC2--->IKBKG
        TRAF2--->CHUK
        RPS6KA5--->RELA
        MAP3K3--->YWHAH
        MAP3K3--->YWHAQ
        MAP3K7--->TNFRSF11A
        TNFAIP3--->IKBKG
        RELB--->SMARCA4
        RIPK3--->RIPK1
        MAP3K8--->NFKB2
        CHUK--->NFKBIB
        RELB--->SMARCC1
        RELB--->NFKB2
        RIPK1--->PTK2
        NFKB1--->REL
        NFKB2--->GTF2I
        CFLAR--->TRAF1
        HSPA4--->IKBKG
        MAP3K3--->IKBKB
        MAP3K3--->NFKBIA
        CHUK--->NFKB1
        PPM1B--->MAP3K7
        TRAF2--->MAP3K5
        TRAF2--->MAP4K2
        TRAF6--->TNFRSF11A
        MAP3K8--->RELA
        GAB1--->MAP3K3
        NFKB1--->NFKBIZ
        IKBKB--->HSP90AB1
        TNFRSF1A--->TRAF2
        CHUK--->RELA
        TNFRSF1A--->TRAF2
        TRAF2--->RIPK1
        BTRC--->NFKBIA
        TNFRSF1B--->TRAF2
        RELA--->NFKBIA
        TNFRSF1A--->RASSF1
        BIRC2--->TRADD
        TRAF2--->MAP3K14
        CASP8--->TRAF1
        MAP3K14--->TRAF3
        CHUK--->HSP90AA1
        FADD--->TRAF2
        GRB2--->SOS1
        TRAF2--->TBK1
        MAP3K3--->YWHAZ
        CFLAR--->TRAF1
        NFKB1--->KPNA3
        NKIRAS1--->NFKBIA
        CAV1--->TNFRSF1B
        G3BP2--->NFKBIA
```

305 TNFRSF11A--->TRAF2
306 TRAF2--->IKBKB
307 NFKB2--->NFKBIA
308 TRAF2--->CHUK
309 CASP8--->RIPK1
310 BIRC2--->TRAF2
311 HSP90AA1--->IKBKG
312 RIPK1--->RNF11
313 CFLAR--->CASP3
314 TRADD--->TRAF2
315 FADD--->CASP10
316 PRKCD--->NCF1
317 NFKB1--->NFKBIE
318 MAP3K14--->NFKB2
319 CASP8--->TRAF2
320 IKBKE--->HSP90AB1
321 CASP10--->TRAF1
322 TRAF2--->TIFA
323 RELB--->KPNA2
324 GRB2--->PTK2
325 TRAF2--->TRAF1
326 MAP3K3--->NFKBIA
327 CREBBP--->RELA
328 BIRC2--->TRADD
329 RELA--->NFKB2
330 TNFRSF1A--->TRADD
331 IKBKB--->NFKBIB
332 MAP3K14--->CHUK
333 BCL3--->NFKB2
334 CUL1--->NFKBIE
335 MAP3K14--->IKBKG
336 TNFRSF1A--->BAG4
337 HSPB1--->NFKBIA
338 CFLAR--->TRAF2
339 NFKB1--->TNIP2
340 NFKB2--->NFKBIB
341 EP300--->RELA
342 NFKB1--->IKBKG
343 NFKB1--->RIPK1
344 NFKB1--->SPAG9
345 IKBKB--->TNFRSF1A
346 TNFAIP3--->TRAF1
347 RIPK1--->IKBKG
348 TRADD--->TRAF1
349 NFKBIE--->REL
350 MAP3K7--->IKBKB
351 NFKB2--->TNIP2
352 TNFRSF1A--->TRAP1
353 TNFAIP3--->RIPK1
354 CHUK--->NFKB2
355 MAP3K14--->HSP90AB1
356 TRADD--->TRAF2
357 RELB--->NFKB2
358 NFKB1--->BCL3
359 BTRC--->CUL1
360 TNFRSF1A--->UBE2I
361 HDAC1--->RELA
362 MAP3K3--->YWHAB
363 CHUK--->NFKB2
364 STAT1--->TRADD
365 JAK1--->TNFRSF1A
366 HSP90AA1--->TBK1
367 PPP1R13L--->RELA
368 RIPK1--->TRAF1
369 HSP90AA1--->RIPK1
370 IKBKB--->NFKBIA
371 TBK1--->IKBKG
372 NFKB1--->NFKB2
373 GRB2--->SOS1
374 PPP6C--->NFKBIE
375 SRC--->CHUK
376 NFKBIB--->REL
377 CHUK--->NFKBIB
378 TNFRSF1A--->GNB2L1
379 TNFRSF1A--->IKBKG
380 TNF--->TRAF2

381 MAP3K7--->MAP3K3
382 HSP90AA1--->TNFRSF1A
383 NFKB2--->NFKBIA
384 BIRC3--->TRAF2
385 TNFRSF8--->TRAF2
386 TRAF6--->PSMD7
387 RIPK1--->CRADD
388 FBXW11--->CUL1
389 IKBKB--->NFKB2
390 NFKB1--->NFKBIB
391 HDAC2--->RELA
392 MAP3K3--->YWHAE
393 TNFRSF1B--->TRAF2
394 IKBKB--->NFKB1
395 REL--->NFKB2
396 BID--->BAX
397 SRC--->NFKBIA
398 MAP3K3--->MARK2
399 BTRC--->SKP1
400 HDAC1--->RELA
401 CFLAR--->RIPK1
402 ERBB3--->ERBB2
403 NFKB2--->NFKBIE
404 RELB--->KPNA6
405 CUL1--->NFKBIB
406 SRC--->IKBKB
407 IKBKB--->TNFRSF1A
408 MAP3K8--->REL
409 TRAF2--->RIPK1
410 CHUK--->NFKB1
411 SKP1--->NFKBIB
412 IKBKG--->NFKBIA
413 APAF1--->CASP9
414 TRAF2--->SPHK1
415 RNF11--->TAX1BP1
416 NFKB1--->RELA
417 TRAF2--->RIPK3
418 CHUK--->TNFRSF1A
419 RELB--->SMARCE1
420 CUL1--->SKP1
421 MAP3K3--->RIPK1
422 TNFRSF1A--->RIPK1
423 RIPK1--->TRADD
424 TRAF2--->TNFAIP3
425 CHUK--->TNFRSF1A
426 ERBB3--->PIK3R1
427 NFKB1--->IKBKG
428 TNF--->TNFRSF1A
429 TNFAIP3--->RNF11
430 NFKB1--->BCL3
431 TRADD--->FADD
432 RIPK3--->RIPK1
433 STAT1--->TNFRSF1A
434 MAP3K3--->HSP90AA1
435 TRAF2--->IKBKB
436 RASAL2--->NFKBIB
437 TRAF5--->MAP3K14
438 CHUK--->PTPN11
439 ERBB3--->PIK3R2
440 IKBKB--->NFKBIA
441 MAP3K3--->MAP2K5
442 TNFRSF1A--->IKBKG
443 CASP8--->TRAF2
444 TBK1--->HSP90AB1
445 MAP3K7--->MAP3K5
446 NFKB2--->RPL30
447 TNFAIP3--->TNIP2
448 RELA--->REL
449 CHUK--->NFKBIA
450 RIPK1--->ITCH
451 IKBKB--->NFKB1
452 TNIP2--->RELA
453 BTRC--->NFKBIB
454 HSP90AA1--->MAP3K14
455 G3BP2--->NFKBIA
456 MAP3K7--->CHUK

457 STAT1--->TNFRSF1B
458 NFKB2--->IKBKG
459 TRADD--->FADD
460 CASP10--->CASP8
461 MAP3K14--->IKBKB
462 TRADD--->TRAF1
463 NFKB1--->RELB
464 NFKB1--->RELB
465 RIPK3--->TNFRSF1A
466 NFKBIB--->NFKBIA
467 MAP3K3--->TRAF7
468 FADD--->CASP8
469 NFKB1--->NFKB2
470 TNFRSF1A--->NSMAF
471 TNFRSF1A--->TRADD
472 CHUK--->NFKBIA
473 GRB2--->TNFRSF1A
474 TNIP2--->REL
475 NFKB1--->RELA
476 STAT1--->FADD
477 FADD--->CFLAR
478 TRIB3--->RELA
479 CFLAR--->CASP8
480 RELB--->HDAC6
481 NKIRAS2--->NFKBIA
482 CHUK--->HSP90AB1
483 CHUK--->IKBKG
484 IKBKG--->NFKBIB
485 HSP90AB1--->IKBKG
486 SKP1--->NFKBIE
487 NFKB2--->DDX3X
488 GRB2--->TNFRSF1A
489 NFKB1--->NFKBIA
490 RIPK1--->FADD
491 MAP3K3--->HSP90AB1
492 MAP3K14--->RPL30
493 HSP90AA1--->RIPK1
494 MAP3K7--->IKBKB
495 IKBKB--->IKBKG
496 NFKB1--->MAP3K8
497 FADD--->IKBKG
498 TRAF2--->FLNA
499 AKT1--->EP300
500 GRB2--->SOS2
501 SYK--->TNFRSF1A
502 NFKB1--->NFKBIB
503 CRADD--->CASP2
504 TNFRSF1A--->RIPK1
505 BIRC2--->TRAF2
506 RIPK1--->TRADD
507 HSP90AA1--->IKBKE
508 PIAS3--->RELA
509 NFKBIA--->REL
510 CHUK--->RELA
511 MAP3K8--->TNIP2
512 RIPK1--->IKBKG
513 RELA--->NFKBIE
514 KPNA2--->NFKBIB
515 MAP3K7--->CHUK
516 CASP8--->RIPK1
517 IKBKG--->NFKBIA
518 CDK9--->CCNT1
519 TRAF6--->KCNQ1
520 IKBKB--->HSP90AA1
521 FBXW11--->NFKBIA
522 TRAF6--->MAP3K14
523 FADD--->TRAF2
524 TNF--->TNFRSF1A
525 SKP1--->NFKBIA
526 MAP3K3--->YWHAG
527 RELB--->RELA
528 RELA--->STAT3
529 BIRC2--->TRAF1
530 CUL1--->NFKBIA
531 TRAF2--->CAV1
532 RNF25--->RELA

```
        TNF--->TNFRSF1B
        TNFAIP3--->RIPK1
        RIPK1--->FADD
        RELA--->NFKBIB
        BIRC2--->IKBKG
        TRAF2--->PEG3
        RELB--->SMARCC2
[TCR]
        GRB2--->PLCG1
        CBLB--->CRKL
        ZAP70--->SHC1
        SLA--->LAT
        PTPN12--->WAS
        SHB--->VAV1
        NCK1--->CD3E
        LCK--->CD8A
        CBL--->ZAP70
        CD247--->STAT5B
        GRB2--->PLCG1
        CBL--->ZAP70
        ZAP70--->SLA
        GRB2--->PTK2B
        CTLA4--->PIK3R1
        FYN--->ZAP70
        SH3BP2--->VAV3
        MAP4K1--->LAT
        CD86--->CTLA4
        CD28--->PIK3R1
        LAT--->GRAP
        PAK1--->ARHGEF6
        ARHGEF7--->GIT2
        FYN--->SYK
        LYN--->SKAP1
        NCK1--->WAS
        CRK--->PIK3R1
        SOS1--->PLCG1
        GRAP--->KHDRBS1
        PAK1--->ARHGEF7
        ZAP70--->RASA1
        ZAP70--->GRB2
        MAP4K1--->DBNL
        ABI1--->WASF2
        CRKL--->WIPF1
        PSTPIP1--->WAS
        ZAP70--->MUC1
        FYN--->ITPR1
        FYN--->GRAP
        ZAP70--->SOS1
        LCP2--->VAV3
        GRAP--->LCK
        CBL--->FYN
        NEDD9--->CRK
        ZAP70--->CD3E
        PTPRH--->LCK
        PTPN3--->CD247
        PTPN12--->PSTPIP1
        AKT1--->PRKCQ
        CBL--->YWHAQ
        SH3BP2--->VAV1
        PTPN12--->PSTPIP1
        LCK--->SH3BP2
        PTK2B--->SH2D3C
        FYN--->PTK2B
        ZAP70--->PLCG1
        NFATC2--->EGR1
        LCK--->SH2D2A
        ZAP70--->WIPF1
        GRB2--->PIK3R1
        CBLB--->ZAP70
        WIPF1--->WAS
        LCP2--->WAS
        SRC--->SKAP1
        LCP2--->VAV1
        LCK--->LIME1
        LCK--->CD4
        ZAP70--->PTK2B
```

609 NCK1--->LCP2
610 JAK3--->CD247
611 CBL--->SYK
612 CBLB--->SYK
613 GRB2--->SHC1
614 GRB2--->LAT
615 GRB2--->SHC1
616 CRKL--->WAS
617 GRB2--->CD247
618 ITK--->GRB2
619 GRAP2--->SHB
620 NCK1--->PAK1
621 CD28--->CD80
622 RASGRP2--->RAP1A
623 PTPN11--->GAB2
624 CD80--->CTLA4
625 PAK1--->GIT2
626 PRKCQ--->CISH
627 ZAP70--->NFAM1
628 PTPN11--->SIT1
629 SYK--->SLA
630 ZAP70--->GAB2
631 VASP--->FYB
632 RAPGEF1--->NEDD9
633 CBL--->GRB2
634 TRAT1--->CD3E
635 MAP4K1--->GRB2
636 RIPK2--->BCL10
637 JUN--->FOS
638 FCRL3--->ZAP70
639 EGR1--->RELA
640 MAP4K1--->NCK1
641 CREBBP--->CREB1
642 DNM2--->VAV1
643 CRKL--->WIPF1
644 FYN--->WAS
645 GRAP--->SHC1
646 FYN--->FYB
647 CDC42--->WAS
648 ARHGEF7--->GIT2
649 HOMER3--->CEBPB
650 ZAP70--->DUSP3
651 LCK--->PTK2
652 SYK--->NFAM1
653 MUC1--->CTNNB1
654 MAP4K1--->GRAP2
655 PTPRC--->SKAP1
656 PPP3CB--->CABIN1
657 GRB2--->SKAP1
658 ZAP70--->DEF6
659 LCK--->SKAP1
660 PTK2B--->PXN
661 JUN--->FOS
662 EVL--->FYB
663 NCL--->CD3E
664 LCP2--->PLCG1
665 ZAP70--->CRK
666 ENAH--->FYB
667 SLA--->CD247
668 LAT--->GAB2
669 PAK1--->GIT2
670 GRAP--->SOS1
671 CBL--->CRK
672 LAT--->CD4
673 GRB2--->SOS1
674 SHB--->LCP2
675 ZAP70--->CD247
676 MAP4K1--->CRK
677 GRB2--->SOS2
678 LCK--->DLG1
679 ITK--->LCP2
680 SLA2--->CD247
681 LCP2--->VAV1
682 TRAT1--->PIK3R1
683 PSTPIP1--->CD2
684 PPP3CB--->NFATC2

```
        ZAP70--->ABL1
        GRAP2--->LAT
        CD247--->SHC1
        GRB2--->SOS1
        ZAP70--->SHB
        LCK--->PTK2B
        MAP3K7--->BCL10
        GRB2--->PIK3R2
        CD2AP--->PSTPIP1
        GRB2--->INPP5D
        LCK--->MUC1
        CBL--->SLA2
        LAT--->PLCG1
        LCK--->ZAP70
        CBL--->PTPN22
        SKAP2--->FYB
        LAT--->CD8A
        SOS2--->PLCG1
        PDPK1--->PRKCD
        LAT--->LCP2
        CD28--->PIK3R2
        ZAP70--->CARD11
        TRAF6--->MALT1
        PTK2B--->VAV1
        TRAT1--->CD247
        CBLB--->GRB2
        BCL10--->CARD11
        LCP2--->FYB
        MAP4K1--->CRKL
        SOS1--->PLCG1
        LAT--->SHB
        ZAP70--->PLCG1
        INPP5D--->DOK2
        FYB--->WAS
        DOCK2--->CD247
        FYN--->SKAP1
        PRKCQ--->STK39
        ZAP70--->SLA2
        LAT--->VAV1
        SOS1--->SHC1
        CD247--->STAT5A
        NCK1--->LCP2
        GRAP--->PLCG1
        MAP2K1--->MAPK1
[Notch]
        JAG2--->NOTCH2
        JAG1--->NOTCH2
        RBPJ--->NOTCH3
        PSEN1--->NOTCH1
        FBXW7--->NOTCH4
        SKP2--->TCF3
        NCOR2--->RBPJ
        NOTCH1--->RBPJ
        CNTN1--->NOTCH1
        NCOR2--->HDAC1
        JAG1--->NOTCH1
        AKT1--->PIK3R1
        NCOR1--->HEY2
        PSEN2--->NOTCH4
        DLL4--->NOTCH4
        JAG2--->NOTCH3
        RBPJ--->NOTCH3
        HES6--->HES1
        PSEN1--->DLL1
        RBPJ--->NOTCH2
        PSEN2--->NOTCH3
        GSK3B--->NOTCH2
        EP300--->SMAD1
        LCK--->PIK3R1
        ITCH--->NOTCH1
        FHL1--->RING1
        SMAD3--->NOTCH1
        JAG1--->NOTCH3
        JAK2--->STAT3
        HDAC1--->RBPJ
        HES5--->STAT3
```

```
        EP300--->NOTCH1
        RBPJ--->NOTCH2
        NOTCH1--->RBPJ
        NUMBL--->NOTCH1
        NCOR1--->RBPJ
        RBPJ--->NOTCH4
        JAK2--->HES1
        ITCH--->NUMB
        CNTN1--->NOTCH2
        FBXW7--->NOTCH1
        EP300--->ASCL1
        DLL1--->NOTCH3
        LCK--->NOTCH1
        NFKB1--->NOTCH1
        NUMB--->APP
        HES1--->STAT3
        EP300--->NOTCH1
        PSEN1--->NOTCH4
        NOV--->NOTCH1
        PSEN1--->NOTCH2
        DLL1--->NOTCH1
        WDR12--->NOTCH1
        RBPJ--->FHL1
        RBPJ--->NOTCH4
        PSEN1--->NOTCH3
        DLL1--->NOTCH2
        MEF2C--->NOTCH1
        JAK2--->HES5
        NUMBL--->APP
        YY1--->NOTCH1
        EP300--->DTX1
        RBPJ--->SPEN
        PSEN2--->NOTCH1
        NOTCH1--->DTX1
        NUMB--->NOTCH1
        JAG2--->NOTCH1
        PSEN2--->NOTCH2
        HEY2--->HES1
[Leptin]
        JAK2--->IRS2
        STAT1--->STAT3
        AKT1--->IKBKB
        JAK2--->IRS2
        JAK2--->PIK3R1
        JAK2--->IRS1
        NCOA1--->STAT3
        JAK2--->LEPR
        IRS1--->PIK3R2
        JAK2--->STAT3
        JAK2--->SH2B1
        PIK3R2--->IRS2
        EP300--->RELA
        MTOR--->AKT1
        KHDRBS1--->PIK3R1
        FYN--->IRS1
        IRS4--->PIK3R1
        JAK2--->IRS1
        JAK2--->SOCS3
        JAK2--->SH2B1
        JAK2--->STAT5B
        KHDRBS1--->STAT3
        PTPN11--->LEPR
        AKT1--->CHUK
        GRB2--->SHC1
        IRS2--->PIK3R1
        PTPN11--->GRB2
        AKT1--->STAT1
        LEP--->LEPR
        IRS1--->PIK3R1
        LEPR--->STAT3
        LEP--->LEPR
[Kit]
        TEC--->DOK1
        TEC--->KIT
        CRKL--->DOK1
        AKT1--->BAD
```

837 PTPRU--->KIT
838 LYN--->KIT
839 MATK--->KIT
840 CBL--->KIT
841 SRC--->DOK1
842 CBL--->SHC1
843 KIT--->SH2B3
844 CRK--->PIK3R1
845 FGR--->DOK1
846 KIT--->CRKL
847 CRKL--->PIK3R1
848 RASA1--->DOK1
849 CBL--->PIK3R1
850 SRC--->KIT
851 CBL--->CRKL
852 KIT--->STAT5A
853 EP300--->MITF
854 KIT--->GRB7
855 GRB2--->SOCS1
856 PTPN11--->KIT
857 KIT--->STAP1
858 KIT--->PIK3R2
859 YES1--->DOK1
860 WIPF1--->WAS
861 KIT--->PIK3R1
862 STAT1--->STAT5A
863 KIT--->CRKL
864 KIT--->GRAP
865 KIT--->RASA1
866 KIT--->GRB10
867 KIT--->CRK
868 CRKL--->PIK3R2
869 BTK--->TNFRSF10A
870 CBL--->GRB2
871 ABL1--->DOK1
872 GRB2--->SOS1
873 LYN--->DOK1
874 BTK--->KIT
875 SOS1--->SHC1
876 KIT--->PLCG1
877 CBLB--->SH3KBP1
878 FYN--->KIT
879 HRAS--->RAF1
880 KIT--->STAT5B
881 PTPN11--->GRB2
882 KIT--->SOCS6
883 KIT--->SPRED2
884 KIT--->GRB2
885 HCK--->KIT
886 STAT1--->STAT5B
887 KIT--->SOCS5
888 TEC--->SHC1
889 PTPN6--->KIT
890 YES1--->KIT
891 HCK--->DOK1
892 KIT--->SH2B2
893 JAK3--->IL2RG
894 KIT--->DOK1
895 LYN--->KIT
896 KIT--->PIK3R1
897 CBLB--->KIT
898 SRC--->PIK3R1
899 KIT--->SOCS1
900 KIT--->STAT1
901 GRB2--->SHC1
902 FES--->PIK3R1
903 PTPN11--->GAB2
904 DOK1--->PLCG1
905 KIT--->JAK2
906 CBL--->KIT
907 KIT--->INPP5D
908 KIT--->DOK1
909 KIT--->SPRED1
910 KIT--->EPOR
911 KIT--->CSF2RB
912 CBL--->CRKL

```
        INPP5D--->DOK1
        CBL--->PIK3R2
[RANKL]
        PTPN6--->LYN
        CBL--->TNFRSF11A
        MAP3K7--->TNFRSF11A
        TRAF6--->MAP3K7
        TRAF6--->TNFRSF11A
        TNFRSF11A--->TRAF5
        FCAR--->PILRA
        TNFRSF11A--->GAB2
        TRAF6--->SQSTM1
        MAPK14--->NFATC1
        TNFRSF11A--->TRAF3
        CBL--->PIK3R1
        CBLB--->TNFRSF11A
        TNFRSF11A--->TRAF3
        CBL--->TRAF6
        TRAF6--->MAP3K7
        IKBKB--->IKBKG
        TRAF6--->SQSTM1
        LYN--->SYK
        TNFSF11--->TNFRSF11A
        SRC--->TNFRSF11A
        PTPN6--->LYN
        PLCG2--->GAB2
        FHL2--->TNFRSF11A
        LYN--->GAB2
        CBL--->TNFRSF11A
        TNFSF11--->TNFRSF11B
        SRC--->TRAF3
        LYN--->GAB2
        PTPN6--->GAB2
        TRAF6--->PTPN6
        TRAF6--->SRC
        TRAF1--->TNFRSF11A
        MAP3K7--->TNFRSF11A
        TNFRSF11A--->TRAF2
        TRAF6--->SRC
        NFATC1--->SPI1
        TRAF6--->FHL2
        TRAF1--->TNFRSF11A
        TNFSF11--->TNFRSF11A
        SRC--->TRAF1
        TRAF6--->TNFRSF11A
        CBL--->PIK3R2
[Prolactin]
        SRC--->DNM2
        CBL--->FYN
        GRB2--->SOCS1
        ZAP70--->PRLR
        ZAP70--->VAV1
        PTPN6--->GRB2
        ERBB2--->JAK2
        PTPN1--->STAT5A
        PRLR--->VAV2
        PTPN1--->STAT5B
        PPIB--->STAT5A
        JAK2--->PRLR
        NEK3--->VAV2
        TEC--->PRLR
        IRS1--->PIK3R1
        CBL--->PIK3R1
        YWHAZ--->PRLR
        CBL--->PIK3R2
        ZAP70--->PRLR
        PTPN11--->JAK2
        IRS1--->PIK3R2
        JAK2--->SH2B1
        ERBB2--->GRB2
        YWHAG--->PRLR
        JAK2--->PRLR
        NCK1--->PAK1
        PIK3R2--->IRS2
        PTPN11--->STAT5A
        SOCS3--->PRLR
```

PRL--->PRLR
JAK2--->PPIA
PPP2CA--->RPS6KB1
CBL--->GRB2
GRB2--->SOS1
PRLR--->VAV1
PPIB--->PRL
SOS1--->SHC1
JAK2--->SHC1
PTPN11--->GRB2
PPP2CA--->EIF4EBP1
PLAU--->SERPINE1
JAK2--->SOCS1
FYN--->PIK3R2
ZAP70--->VAV1
NEK3--->VAV1
FYN--->PIK3R1
PPIB--->PRL
PTPN11--->PRLR
NEK3--->PRLR
JAK2--->STAT5B
PTPN11--->PRLR
TEC--->PRLR
JAK2--->STAT5A
EP300--->STAT5A
PTPN11--->JAK2
GRB2--->SHC1
PTPN11--->GAB2
IRS2--->PIK3R1
TEC--->VAV1
SOCS2--->PRLR
IRS1--->PIK3R1
CISH--->PRLR
PPIA--->PRLR
JAK2--->SIRPA
PRLR--->VAV1
[Wnt]
CTNNB1--->CDH2
PIN1--->CTNNB1
AXIN1--->APC
ROR2--->FZD2
WNT1--->FZD8
DAB2--->AXIN1
ARRB2--->DVL2
AXIN1--->DVL1
GSK3B--->CTNNB1
DAB2--->DVL3
RAC1--->DVL1
RHOA--->DVL2
CSNK1A1--->AXIN1
RYK--->FZD8
AXIN2--->APC
TCF3--->CTNNB1
WNT3A--->FZD1
DIXDC1--->MAP3K4
SFRP1--->WNT4
RHOA--->DAAM1
CCND1--->CTNNB1
CSNK1A1--->CTNNB1
DIXDC1--->AXIN1
WNT1--->FZD8
WNT7A--->FZD5
DAB2--->AXIN1
JUP--->CDH2
DAB2--->DVL3
AXIN1--->APC
RYK--->WNT1
SFRP2--->WNT4
AXIN1--->CTNNB1
WNT7A--->FZD9
ILK--->GSK3B
ARRB1--->DVL1
SFRP1--->WNT2
FZD8--->LRP6
GSK3B--->APC
ARRB1--->DVL2

WNT1--->LRP5
AXIN1--->DVL3
SFRP1--->FZD6
AXIN1--->MAP3K4
FZD4--->DVL2
GSK3B--->AXIN1
PRKCG--->DVL2
PRKCB--->DVL2
GSK3B--->AXIN2
ROR2--->WNT5A
AKT1--->GSK3B
AKT1--->GSK3B
RAC1--->DVL2
PPP2CA--->AXIN1
WNT7A--->FZD5
DKK1--->LRP6
BTRC--->AXIN1
APC--->CTNNB1
FZD8--->LRP5
SFRP1--->WNT1
BTRC--->CTNNB1
CTNNB1--->SOX1
WNT3--->FZD1
PPP1R1B--->PPP1CA
JUP--->APC
CTNNB1--->CDH1
PRKCA--->DVL2
AXIN1--->LRP6
WNT2--->FZD1
GSK3B--->APC
FZD8--->LRP6
WNT4--->FZD6
GSK3B--->AXIN1
ROR2--->FZD5
WNT1--->LRP6
TCF4--->CTNNB1
APC--->CTNNB1
DKK1--->LRP5
AXIN1--->LRP5
AXIN1--->CTNNB1
RYK--->WNT3A
WNT2--->FZD9
ARRB2--->FZD4
WNT3A--->LRP6
DIXDC1--->AXIN1
CDK6--->CTNNB1
CSNK1E--->DVL1
AXIN2--->CTNNB1
AXIN1--->DVL2
WNT3A--->LRP6
TCF4--->CTNNB1

[ID]

ID3--->HES1
ELK3--->ID2
ID4--->HES1
PAX5--->ID3
ID2--->RBL1
PAX8--->ID2
ELK1--->ID1
ID2--->RBL2
ID2--->RB1
PAX2--->ID2
ELK4--->ID3
ELK4--->ID2
ID1--->TCF3
PAX5--->ID2
ID1--->TCF12
ELK4--->ID1
PSMD4--->MYOD1
ATF3--->SMAD3
ID2--->TCF3
ID3--->TCF3
ID3--->TCF12
ID3--->SREBF1
ELK1--->ID3
PSMD4--->TCF3

```
        ID1--->TCF4
        ID1--->HES1
        PSMD4--->ID1
        ID2--->TCF12
        ID2--->HES1
        ELK1--->ID2
[Gastrin]
        BCAR1--->CRK
        PTPN11--->CCKBR
        GRB2--->SOS1
        JAK2--->PIK3R1
        PXN--->ITGB1
        PTK2--->PXN
        SRC--->PTK2
        GRB2--->IRS1
        GRB2--->SHC1
        TCF4--->CTNNB1
        IRS1--->PIK3R1
        SRC--->PLCG1
[Ghrelin]
        MAPK1--->RAF1
        MAPK3--->RAF1
        GHRL--->GHSR
        SRC--->AKT1
        IRS1--->PIK3R1
        MAPK3--->ARRB1
        RAF1--->ARRB2
        MAPK1--->ARRB1
        FYN--->DOK1
        MAPK1--->ARRB2
        GRB2--->IRS1
        SRC--->RAF1
[Hedgehog]
        SHH--->PTCH1
        DHH--->PTCH2
        GAS1--->SHH
        GLI1--->SUFU
        STK36--->SUFU
        SHH--->PTCH2
        SMO--->PTCH2
        SHH--->PTCH1
        STK36--->GLI2
        IHH--->PTCH2
        SMO--->PTCH1
        SUFU--->GLI2
        SMO--->PTCH1
        IHH--->PTCH1
        SUFU--->GLI3
        HHIP--->SHH
        HHIP--->DHH
        CREBBP--->GLI3
        GLI1--->SUFU
        STK36--->GLI1
        HHIP--->IHH
        SKI--->GLI3
        STK36--->GLI3
        DHH--->PTCH1
        SKI--->GLI2
        DYRK1A--->GLI1
[RAGE]
        CDK2--->CCNE1
        IRAK4--->MYD88
        PRKCD--->EGFR
        TIRAP--->MYD88
        EGFR--->SHC1
        PRKCD--->SHC1
        CDK2--->CDKN1B
        PRKCA--->SRC
        ROCK1--->MSN
        GRB2--->SHC1
[AR]
        JUN--->AR
        FHL2--->AR
        CDK7--->AR
        EFCAB6--->AR
        CDC25A--->AR
```

1217 RAC3--->AR
1218 SRC--->ESR1
1219 DAXX--->AR
1220 AKT1--->AR
1221 HIPK3--->AR
1222 MDM2--->AR
1223 CREBBP--->CREB1
1224 PAK6--->AR
1225 CCND1--->AR
1226 CCNH--->AR
1227 FHL2--->AR
1228 CDC25B--->AR
1229 CTNNB1--->AR
1230 IL6ST--->AR
1231 ESR1--->AR
1232 UBE3A--->AR
1233 CREBBP--->AR
1234 CCNE1--->AR
1235 SRY--->AR
1236 EFCAB6--->AR
1237 STAT3--->AR
1238 NCOA2--->AR
1239 NR2C1--->AR
1240 GAPDH--->AR
1241 PIAS1--->AR
1242 HSPA8--->HSP90AA1
1243 ERCC3--->GTF2H2
1244 TCF4--->AR
1245 CTNNB1--->AR
1246 NCOR2--->AR
1247 GNB2L1--->AR
1248 CDK7--->AR
1249 SPDEF--->AR
1250 NR3C1--->AR
1251 SMAD3--->AR
1252 RNF4--->AR
1253 ESR1--->AR
1254 TGFB1I1--->AR
1255 FLNA--->AR
1256 FOXO1--->AR
1257 BAG1--->AR
1258 RCHY1--->AR
1259 NCOA1--->AR
1260 CDK7--->CCNH
1261 ETV5--->AR
1262 CREBBP--->AR
1263 NSD1--->AR
1264 RNF4--->AR
1265 RNF4--->PATZ1
1266 RBAK--->AR
1267 RUNX2--->AR
1268 BRCA1--->AR
1269 PIAS2--->AR
1270 TCF4--->AR
1271 SP1--->AR
1272 CCNH--->AR
1273 AHR--->AR
1274 GSK3B--->AR
1275 ATF2--->AR
1276 HDAC1--->AR
1277 POU2F1--->AR
1278 PTEN--->AR
1279 EGFR--->AR
1280 NONO--->AR
1281 CDK9--->AR
1282 CAV1--->AR
1283 FOXA1--->AR
1284 RAD54L2--->AR
1285 PXN--->AR
1286 ERCC3--->CCNH
1287 BAG1--->AR
1288 SVIL--->AR
1289 MDM2--->HDAC1
1290 PRMT1--->AR
1291 ZMIZ1--->AR
1292 HSP90AA1--->AR

```
        SRC--->AR
        RB1--->AR
        PIAS4--->AR
        CDK7--->GTF2H2
        CDK11B--->CCND3
        RAN--->AR
        UBE2I--->AR
        NCOA1--->AR
        MDM2--->AR
        CDK7--->ERCC3
        NR0B2--->AR
        CALR--->AR
        NR2C2--->AR
        NCOR1--->AR
        PIAS3--->STAT3
        NCOA3--->AR
        PTK2--->PIK3R1
        SRC--->AR
        PIAS3--->AR
        TCF4--->CTNNB1
        RANBP9--->AR
[BCR]
        BCAR1--->CRK
        CRKL--->RAPGEF1
        CD22--->SHC1
        CD22--->SHC1
        PIK3R1--->GAB2
        LYN--->RASA1
        INPP5D--->CD22
        CBL--->SLA2
        LCP2--->VAV1
        CDK4--->CCND2
        CRK--->VAV1
        CBL--->SHC1
        LYN--->CD79A
        CD19--->CD79A
        CBL--->LCK
        GRB2--->INPP5D
        PRKD1--->SYK
        CBL--->CRK
        FYN--->BCAR1
        GRB2--->SOS1
        HCK--->BCAR1
        CSK--->DOK1
        GAB1--->PIK3R1
        LYN--->NEDD9
        NCK1--->BLNK
        CBL--->PIK3R1
        ZAP70--->CD79B
        CD22--->ATP2B4
        CBL--->CRKL
        CBL--->SYK
        DAPP1--->PLCG1
        BCL2L11--->BCL2
        SOS1--->CD19
        CD22--->PLCG1
        CARD11--->IKBKG
        GAB1--->SHC1
        CDK2--->CCNA2
        BTK--->CD19
        BTK--->BLNK
        CDK6--->CCND3
        PTPN6--->CD79A
        CRK--->RAPGEF1
        MAP4K1--->BLNK
        PTPN11--->PIK3R1
        GRB2--->CD22
        PRKD1--->PLCG1
        GRB2--->VAV1
        LYN--->PTK2
        BLK--->CD79B
        BLK--->CD79A
        LCP2--->PLCG1
        CBL--->LYN
        LYN--->PLCG2
        GAB1--->MAPK1
```

1369 GRB2--->BLNK
1370 SH3BP2--->VAV2
1371 TEC--->PTPN18
1372 SYK--->CD79A
1373 PTK2B--->NEDD9
1374 GRB2--->SOS1
1375 TCL1A--->AKT1
1376 GRB2--->INPP5D
1377 MAPK4--->GAB2
1378 SOS1--->SHC1
1379 BCAR1--->CRKL
1380 CD19--->VAV1
1381 MAP3K7--->CARD11
1382 SH3BP2--->PLCG1
1383 LCK--->CD79A
1384 INPP5D--->DOK3
1385 CBL--->FYN
1386 SYK--->CD22
1387 BLNK--->VAV1
1388 LIME1--->PLCG2
1389 SYK--->VAV1
1390 CD19--->PLCG2
1391 PIK3AP1--->PIK3R1
1392 BANK1--->ITPR2
1393 MAP4K1--->DAPP1
1394 PTPN6--->BLNK
1395 CD19--->PIK3R1
1396 SYK--->VAV2
1397 PTK2B--->BCAR1
1398 PTPN6--->CD22
1399 LYN--->CD19
1400 CRKL--->SOS2
1401 PTPN6--->CD79B
1402 GAB1--->MAPK3
1403 BLNK--->PLCG2
1404 DAPP1--->PLCG2
1405 LCP2--->CD22
1406 TEC--->DOK1
1407 NEDD9--->CRKL
1408 MAPK3--->FCGR2B
1409 GRB2--->SOS2
1410 RASA1--->DOK1
1411 GAB1--->MAPK4
1412 MAPK1--->FCGR2B
1413 CBL--->BLK
1414 SYK--->CD79B
1415 SOS2--->SHC1
1416 CDK4--->CCND3
1417 SOS1--->SHC1
1418 NCK1--->LCP2
1419 GRB2--->CD72
1420 CD19--->CD79B
1421 SYK--->SH3BP2
1422 CBL--->VAV2
1423 BLNK--->CD79A
1424 PTPN6--->CD72
1425 JUNB--->FOSB
1426 FYN--->CD79A
1427 PRKD1--->PLCG2
1428 SH3BP2--->VAV1
1429 MAPK3--->GAB2
1430 GRB2--->BLNK
1431 INPP5D--->CD22
1432 JUNB--->FOS
1433 BTK--->GTF2I
1434 PTPRC--->LYN
1435 GRB2--->SHC1
1436 GRB2--->SHC1
1437 PTPN11--->GAB2
1438 CRKL--->SOS1
1439 PTPN11--->GRB2
1440 CBL--->GRB2
1441 GRB2--->RAPGEF1
1442 GAB1--->PTPN11
1443 BLNK--->PLCG1
1444 PTK2--->CD79B

INPP5D--->SHC1
GRB2--->LIME1
CBL--->BTK
INPP5D--->SHC1
LYN--->BCAR1
MAPK1--->GAB2
CBL--->PIK3R2
CDK2--->CCNE1
GRB2--->CD19
[EGFR]
NCK1--->SOS1
MAPK1--->ELK1
GRB7--->CAV1
STAT1--->STAT3
SRC--->GAB2
RAB5A--->RIN1
EGFR--->PIK3R1
GRB2--->PIK3R1
EGFR--->PTK6
MAPK3--->RPS6KA3
SH3KBP1--->SH3GL2
EGF--->EGFR
EGF--->PIK3R2
PIK3R1--->GAB2
MAP2K1--->MAPK3
CBL--->SHC1
MAPK1--->PEBP1
EGFR--->TNK2
PTPN5--->MAPK1
CBL--->SHC1
SRC--->EGFR
HTT--->HIP1
HRAS--->RALGDS
GRB2--->FRS2
CAV1--->PLD1
CBLC--->SPRY2
GRB2--->REPS2
PIK3R1--->VAV3
NCK2--->PAK1
RASA1--->DOK2
EGFR--->SOCS3
NCK1--->PKN2
EGFR--->DOK2
PIK3R1--->SHC1
CBL--->CRK
PIK3R3--->PIK3CA
GRB2--->SOS1
CBLB--->PIK3R1
PIK3R1--->PIK3CA
MAP3K14--->EGFR
GRB2--->PIK3R2
GAB1--->PIK3R1
SRC--->SHC1
STAT5B--->STAT3
EGFR--->CBLC
CBL--->PIK3R1
EGFR--->PTK2
MAP3K2--->SH2D2A
EGFR--->PTPN11
SRC--->EGFR
MAPK3--->MAPK14
CBLB--->EGFR
CBL--->CRKL
MAPK7--->GJA1
PAK1--->EGFR
EGFR--->NCK1
RAC1--->STAT3
EGFR--->PLSCR1
GRB2--->HTT
EGFR--->INPPL1
EGFR--->GRB2
EGFR--->SOCS1
CBL--->PIK3R1
GRB2--->GAB2
MAPK1--->EGFR
EGFR--->GRB2

1521 SRC--->STAT1
1522 CBL--->PIK3R2
1523 MAPK1--->MAPK14
1524 ABI1--->EPS8
1525 MAP3K14--->GRB10
1526 GIT1--->PLCG1
1527 EGFR--->STAT5A
1528 JAK2--->STAT3
1529 MAP2K2--->MAPK1
1530 GRB2--->PAK1
1531 GRB2--->PIK3R1
1532 EGFR--->STAT1
1533 RAF1--->BRAF
1534 APPL2--->RBBP7
1535 CBLC--->CRK
1536 PTPN11--->SHC1
1537 GRB2--->GAB2
1538 GAB1--->PIK3CG
1539 PIK3R2--->SHC1
1540 MAPK1--->RPS6KA1
1541 PLD2--->PLCG1
1542 EGFR--->NCK2
1543 GRB2--->PIK3C2B
1544 PTPN11--->PIK3R1
1545 SOCS3--->RASA1
1546 CBLB--->PIK3R1
1547 CDC42--->MAP3K4
1548 EGFR--->PIK3R2
1549 RAF1--->PEBP1
1550 JUN--->FOS
1551 SOS2--->SHC1
1552 NCK1--->DOK2
1553 VAV3--->PLCG1
1554 PTPRR--->MAPK1
1555 INPPL1--->SHC1
1556 EGFR--->HIST3H3
1557 PTPN12--->JAK2
1558 PTPN11--->SOS1
1559 EGFR--->VAV2
1560 NCK2--->SOS1
1561 EGFR--->ERBB2
1562 EGFR--->SHC1
1563 EPN1--->EPS15L1
1564 JAK1--->STAT1
1565 CBLB--->SHC1
1566 CBL--->EGFR
1567 CBL--->GRB2
1568 RALB--->RALBP1
1569 EGFR--->CAV1
1570 GRB2--->SOS1
1571 EGFR--->VAV3
1572 MAPK3--->CASP9
1573 MAP3K2--->WNK1
1574 MAP2K1--->MAPK14
1575 EGFR--->EPS8
1576 SOS1--->SHC1
1577 APPL1--->RBBP7
1578 SRC--->CAV2
1579 EGFR--->GRB10
1580 CEBPA--->MYC
1581 RAB5A--->USP6NL
1582 NCK1--->PAK1
1583 RAF1--->RFXANK
1584 MAPK3--->CAV1
1585 USP6NL--->EPS8
1586 PRKCD--->SHC1
1587 MAPK1--->CAV1
1588 CBL--->PLCG1
1589 PIK3R2--->SHC1
1590 SRC--->DNM1
1591 SOS1--->PIK3R2
1592 EGFR--->CRK
1593 SRC--->PTK2
1594 CBL--->YWHAZ
1595 MAPK1--->CASP9
1596 EGFR--->STAT5B

1597 PTPN5--->MAPK3
1598 CRK--->SOS1
1599 GRB2--->REPS1
1600 HRAS--->RAF1
1601 EGFR--->PIK3C2B
1602 MAP2K5--->PRKCZ
1603 PTPN11--->GRB2
1604 MAP2K1--->MAPK1
1605 EGFR--->YWHAZ
1606 PTPN11--->FRS2
1607 EGFR--->PLD1
1608 RASA1--->HTT
1609 NCK1--->CAV2
1610 PIK3R1--->PIK3CB
1611 CBLC--->GRB2
1612 ADRBK1--->EGFR
1613 PTPN6--->CTNND1
1614 EGFR--->HTT
1615 EGFR--->RGS16
1616 PRKCA--->PLD2
1617 RAC1--->MAP3K4
1618 GAB1--->GRB2
1619 EGFR--->GAB1
1620 SP1--->JUN
1621 SRC--->PTK2B
1622 EGFR--->VAV3
1623 PRKCA--->PLD1
1624 MAPK1--->FRS3
1625 EGFR--->PRKAR1A
1626 CBL--->SPRY2
1627 SHC1--->VAV3
1628 MAPK1--->RPS6KA3
1629 GRB2--->VAV3
1630 EGFR--->ERRFI1
1631 SH3GL3--->DNM1
1632 GAB1--->PTPN11
1633 MAP3K3--->MAP2K5
1634 SH3KBP1--->ASAP1
1635 MAP3K14--->GRB14
1636 RAF1--->MAP2K1
1637 MAP2K1--->PEBP1
1638 NCK2--->PKN2
1639 PTPRR--->MAPK3
1640 EGFR--->EPS15
1641 MAPK3--->JUN
1642 EGF--->GRB2
1643 PIAS3--->STAT3
1644 RIPK1--->EGFR
1645 GRB2--->CRK
1646 EGFR--->SHC1
1647 PDPK1--->AKT1
1648 JAK2--->STAT1
1649 MAPK7--->YWHAB
1650 SOS2--->SHC1
1651 SOS1--->PLCG1
1652 PTPN11--->PXN
1653 EGFR--->ARF4
1654 EGFR--->VAV1
1655 PIK3R1--->SHC1
1656 SOS1--->SHC1
1657 NFKB1--->RELA
1658 GRB2--->PRKAR1A
1659 FLOT2--->FLOT1
1660 GRB2--->TNK2
1661 PTPRR--->MAPK7
1662 CEBPB--->MYC
1663 EGFR--->PTPN6
1664 EGFR--->STAT3
1665 CBL--->VAV2
1666 CAV1--->DNM1
1667 MAP2K5--->MAPK7
1668 MAP3K14--->GRB7
1669 RASA1--->CAV2
1670 SOS1--->EGFR
1671 APPL1--->MTA2
1672 MAPK3--->ELK1

MAPK3--->RPS6KA2
PIK3R1--->PIK3CD
CBL--->SH3KBP1
EGFR--->ERRFI1
MAPK1--->RPS6KA2
CRK--->REPS1
APPL2--->MTA2
SRC--->STAT5A
EGFR--->MAP2K1
EGFR--->RASA1
SRC--->STAT3
MAP2K5--->PRKCI
RALBP1--->REPS2
MAPK1--->TNIP1
PRKCA--->EGFR
GRB2--->SHC1
GRB2--->SHC1
REPS2--->ASAP1
GRB2--->SOS2
DNM1--->PLCG1
GAB1--->PLCG1
PTPN11--->GAB2
EGFR--->CAV1
PTPN1--->EGFR
EGFR--->PLD2
CEACAM1--->SHC1
PTPN11--->GRB2
CBL--->GRB2
MAPK8--->CRK
SRC--->CAV1
RAP1A--->RALGDS
HDAC1--->TGIF1
GAB1--->PTPN11
PIK3R2--->PIK3CD
MAP4K1--->GRB2
HRAS--->PLCE1
RALA--->PLD1
PRKCD--->EGFR
JUND--->FOS
MAP3K3--->WNK1
CDC42--->TNK2
SHC1--->EPS8
PTPN12--->GRB2
PLSCR1--->SHC1
EGFR--->PLCG1
EGFR--->VAV2
SRC--->PLCG1
CBLB--->GRB2
SOS2--->EGFR
SH3GL3--->WASL
EGFR--->PTPN11
CBL--->PIK3R2
MAP2K2--->MAPK3
GRB2--->VAV2
JAK1--->STAT3
MAPK3--->RPS6KA1
PTK2B--->STAT3

[IL]

CRKL--->RAPGEF1
JAK1--->SOCS3
HDAC1--->STAT3
PTPN11--->PTK2B
PIK3R1--->GAB2
STAT1--->STAT3
MAPK3--->STAT3
IL1RAP--->IRAK1
CDK2--->CCNE1
GRB2--->CRKL
PRKCD--->STAT3
JAK1--->IL2RB
JAK3--->INPP5D
CISH--->IL2RB
IL1R1--->TOLLIP
IL2RB--->STAT5A
CHUK--->NFKBIB
ETS2--->STAT5B

1749 CRKL--->GAB2
1750 DNM1--->PIK3R1
1751 MAP3K3--->IKBKB
1752 IRAK4--->IRAK1
1753 SOX4--->SDCBP
1754 PIK3R1--->GAB2
1755 PRKCZ--->IL4R
1756 IL2RB--->SHC1
1757 NR3C1--->STAT5B
1758 JAK3--->STAM
1759 SYK--->GAB2
1760 CBL--->SHC1
1761 TRAF6--->IRAK4
1762 IL6ST--->STAT3
1763 IRAK1--->MYD88
1764 IL6--->IL6R
1765 LYN--->CSF2RB
1766 IRAK4--->PELI1
1767 CBL--->SHC1
1768 IL1RAP--->IRAK1
1769 SHC1--->PLCG1
1770 SHB--->IL2RG
1771 GRB2--->INPP5D
1772 JAK2--->PIK3R1
1773 CHUK--->RELA
1774 DAXX--->STAT3
1775 PTPN11--->SIRPA
1776 IL2RB--->STAT5B
1777 SOCS3--->IL6ST
1778 IKBKB--->NFKBIB
1779 HSP90AA1--->STAT3
1780 RASA1--->DOK2
1781 FES--->IL4R
1782 JAK2--->GRB2
1783 IL1RAP--->MYD88
1784 TRAF6--->IRAK1
1785 IL1RAP--->MYD88
1786 PTPN11--->PILRB
1787 RAC1--->VAV1
1788 IL4R--->DOK2
1789 CBL--->CRK
1790 IRAK4--->MYD88
1791 GRB2--->SOS1
1792 CRKL--->PIK3R1
1793 NLK--->STAT3
1794 PTPN6--->IL4R
1795 BAD--->BCL2
1796 GAB1--->PIK3R1
1797 CBL--->HCK
1798 IL1R2--->IL1RN
1799 IL6--->IL6ST
1800 STAT5B--->STAT3
1801 IL7R--->STAT5A
1802 TRAF6--->IRAK2
1803 CBL--->PIK3R1
1804 HCK--->IL6ST
1805 IL7R--->STAT5B
1806 PTPN11--->IRS2
1807 HSP90AA1--->TERT
1808 CBL--->CRKL
1809 IL1RAP--->TOLLIP
1810 JAK3--->PIK3R1
1811 JAK1--->IRS2
1812 IRS1--->PIK3R1
1813 PTPN6--->PIK3R1
1814 GRB2--->GAB2
1815 JAK2--->IL5RA
1816 IRAK2--->MYD88
1817 PTPN11--->CRKL
1818 PTPN11--->IL6ST
1819 ETS1--->STAT5B
1820 IL1R1--->MYD88
1821 PTPN11--->SOCS3
1822 JAK1--->FES
1823 IRAK1--->PELI1
1824 IL1RAP--->SIRPA

1825 JAK1--->IL2RG
1826 PPP2CA--->STAT5A
1827 JAK1--->IL4R
1828 IL6--->IL6R
1829 CRKL--->RAPGEF1
1830 NCOA1--->STAT3
1831 IL6R--->IL6ST
1832 PTPN11--->JAK2
1833 JAK1--->IRS1
1834 HSP90AA1--->AKT1
1835 LYN--->MAPK3
1836 IL2RB--->STAT3
1837 IRS1--->PIK3R2
1838 MTOR--->TERT
1839 NCOA1--->STAT3
1840 JAK1--->CSF2RB
1841 MAP3K3--->NFKBIA
1842 CISH--->CSF2RB
1843 IL2RB--->IL2RG
1844 IKBKB--->RELA
1845 TRAF6--->MAP3K7
1846 SOCS1--->IL2RB
1847 IL1R1--->TOLLIP
1848 GNB2L1--->CSF2RB
1849 JAK1--->IL7R
1850 HRAS--->RAF1
1851 MAP3K14--->CHUK
1852 PTPN11--->PIK3R1
1853 FES--->CSF2RB
1854 GRB2--->GAB2
1855 PIK3R2--->IRS2
1856 IL4R--->SHC1
1857 TRAF6--->IRAK1
1858 PTPN11--->PIK3R1
1859 CBLB--->PIK3R1
1860 JAK1--->CSF2RB
1861 JAK1--->IRS1
1862 CEBPB--->STAT6
1863 AKT1--->TERT
1864 TRAF6--->MAP3K3
1865 CDK9--->IL6ST
1866 IL1RAP--->TOLLIP
1867 IRAK1--->SQSTM1
1868 JAK1--->IRS2
1869 MAP3K7--->STAT3
1870 AKT1--->CREB1
1871 IL1B--->IL1RAP
1872 IL1R1--->IL1RAP
1873 TRAF6--->SQSTM1
1874 IL4R--->INPP5D
1875 LYN--->SYK
1876 MAP3K14--->CHUK
1877 PTPN11--->JAK1
1878 CRKL--->PIK3R2
1879 IL2RB--->PIK3R1
1880 LCK--->PIK3R1
1881 CBLB--->SHC1
1882 JAK1--->PIK3R1
1883 JAK1--->SOCS1
1884 CBL--->GRB2
1885 JAK1--->INPP5D
1886 FYN--->PIK3R2
1887 IKBKB--->NFKBIA
1888 PPP2CA--->STAT5B
1889 GRB2--->SOS1
1890 EP300--->NCOA3
1891 STAT5A--->STAT3
1892 SOCS3--->IL2RB
1893 JUN--->STAT3
1894 SOS1--->SHC1
1895 IL4R--->IL13RA1
1896 NFKB1--->CEBPB
1897 JAK1--->TEC
1898 IL4R--->IL2RG
1899 JAK1--->PIK3R1
1900 JAK3--->IRS2

1901 ERBB3--->SHC1
1902 CRK--->STAT5A
1903 IL1R1--->IRAK2
1904 EP300--->STAT6
1905 NLK--->STAT3
1906 PTPN11--->CSF2RB
1907 PRKCZ--->TRAF6
1908 JAK3--->IRS1
1909 CSF2RB--->SHC1
1910 VCL--->PXN
1911 IRAK4--->IRAK1
1912 CBL--->FYN
1913 IL7R--->PIK3R1
1914 NFKB1--->STAT6
1915 MAP3K7--->MAP3K14
1916 IL1B--->IL1R2
1917 CBL--->PTPN11
1918 HRAS--->RAF1
1919 JAK2--->SHC1
1920 IL4R--->CD40
1921 IL6--->IL6ST
1922 CXCR4--->PTK2
1923 PTK2B--->IL7R
1924 RELA--->STAT6
1925 HCK--->CSF2RB
1926 PTPN11--->GRB2
1927 JAK1--->SHB
1928 RAPGEF1--->SHC1
1929 FYN--->IL2RB
1930 JAK1--->PTK2B
1931 YWHAZ--->CSF2RB
1932 IRAK4--->PELI1
1933 JAK2--->SOCS1
1934 JAK1--->SOCS3
1935 IL7--->IL7R
1936 IL6ST--->STAT3
1937 IL2RG--->IL7R
1938 SYK--->PLCG2
1939 RELA--->NFKBIA
1940 JAK2--->STAM
1941 IL2RB--->IL2RG
1942 IL3RA--->CSF2RB
1943 PTPN6--->GAB2
1944 GRB2--->VAV1
1945 MAP3K7--->MAP3K3
1946 YWHAB--->BCL2L11
1947 SHC1--->GAB2
1948 IL1RN--->IL1R1
1949 FYN--->GRB2
1950 BAD--->BCL2L1
1951 GAB1--->GRB2
1952 ERBB2--->IL6ST
1953 ETS1--->STAT6
1954 IL1A--->IL1R2
1955 IRAK1--->MAP3K7
1956 STAT5A--->STAT3
1957 JAK3--->IL2RB
1958 PTPN11--->IL4R
1959 IL4R--->STAT6
1960 JAK1--->IL5RA
1961 TRAF6--->MAP3K3
1962 IRS1--->IL4R
1963 GAB1--->PTPN11
1964 JAK2--->GRB2
1965 JAK2--->TEC
1966 PTK2--->PXN
1967 BAX--->BCL2
1968 PTPN6--->CSF2RB
1969 RAF1--->MAP2K1
1970 EIF3B--->IL2RB
1971 PRKCD--->IL6ST
1972 GNB2L1--->CSF2RB
1973 JAK2--->FYN
1974 MAP3K2--->NFKBIA
1975 PRKCZ--->JAK1
1976 PIAS3--->STAT3

1977 PTPN11--->IRS1
1978 ERBB2--->SHC1
1979 MAP3K7--->CHUK
1980 JAK3--->IL2RG
1981 GRB2--->IRS1
1982 STAT3--->AR
1983 IL1A--->IL1R1
1984 PTPN11--->STAT3
1985 JAK1--->IL3RA
1986 IRAK1--->PELI2
1987 SOCS5--->IL4R
1988 FES--->PIK3R1
1989 NFKBIB--->NFKBIA
1990 CSF2RB--->IL5RA
1991 CHUK--->NFKBIA
1992 SOS1--->SHC1
1993 RAF1--->IL2RB
1994 SYK--->CSF2RB
1995 LCK--->IL2RB
1996 IL1RAP--->IL1R2
1997 PTPN6--->PILRB
1998 IRAK1--->PELI1
1999 RELA--->NFKBIB
2000 CREBBP--->NCOA1
2001 MAP3K7--->MAP3K14
2002 IL6ST--->VAV1
2003 LYN--->IL2RB
2004 STAT5B--->STAT3
2005 HRAS--->PIK3CD
2006 TEC--->PIK3R1
2007 PIK3R1--->PIK3CD
2008 JAK3--->IL7R
2009 FYN--->PIK3R1
2010 JAK3--->IRS1
2011 JAK1--->IL6ST
2012 NCOA1--->AR
2013 GAB1--->PIK3R1
2014 LCK--->MAPK1
2015 SRC--->STAT3
2016 JAK2--->IL4R
2017 JAK3--->IRS2
2018 MAPK1--->VAV1
2019 YWHAQ--->BAD
2020 CRK--->STAT5B
2021 JAK3--->PTK2B
2022 FYN--->GRB2
2023 PTPN11--->INPP5D
2024 KAT5--->STAT3
2025 GRB2--->IRS1
2026 PTPN11--->IL6ST
2027 IL1A--->IL1R2
2028 JAK2--->FYN
2029 PTPN11--->JAK2
2030 IL1B--->IL1R1
2031 GRB2--->SHC1
2032 GRB2--->SHC1
2033 PTPN6--->IL2RB
2034 CRKL--->STAT5A
2035 HCK--->CSF2RB
2036 PTPN11--->GAB2
2037 FYN--->CSF2RB
2038 RPS6KB1--->TERT
2039 PTPN11--->GAB2
2040 SYK--->IL2RB
2041 IRS2--->PIK3R1
2042 TEC--->VAV1
2043 GRB2--->IRS2
2044 CHUK--->IKBKG
2045 PTPN11--->GRB2
2046 JAK2--->PPP2CA
2047 CBL--->GRB2
2048 IL6R--->IL6ST
2049 IRS2--->PIK3R1
2050 JAK2--->TEC
2051 PTPN11--->SIRPA
2052 SOX4--->SDCBP

IL4R--->IRS2
GAB1--->PTPN11
PIK3R2--->PIK3CD
MAP3K7--->CHUK
RARA--->RXRA
ADRBK2--->CXCR4
YWHAZ--->CSF2RB
CREBBP--->STAT6
CRKL--->SHC1
MAP3K7--->STAT3
JAK2--->IL3RA
CSF2RB--->IL5RA
JAK2--->CSF2RB
IRS1--->PIK3R1
HRAS--->PIK3R1
STAT1--->IL2RB
PTPN11--->SOS1
JAK3--->IL4R
SHB--->IL2RB
FYN--->IL7R
IL1A--->IL1R1
IL1B--->IL1R1
CREBBP--->STAT3
JAK3--->IL2RG
IL1A--->IL1RAP
INPP5D--->SHC1
SDCBP--->IL5RA
CBL--->CRKL
JAK2--->YES1
JAK3--->SHB
CBL--->PIK3R2
TRAF6--->IRAK4
IL7R--->STAT3
PRKCD--->STAT3
CRKL--->STAT5B

[Section 5] Predictions on Reactome

[1]	GCK	<---	PPARG	-0.048738	-0.017457	+
[2]	EP300	<---	RARG	-0.0067479	-0.0035614	-
[3]	IGF1R	--->	SHC1	0.37655	-0.22424	-
[4]	ANKRD1	<---	FOS	-0.12621	0.26835	+
[5]	PRKAR1A	--->	ZEB1	0.49287	-0.43671	-
[6]	MAP3K3	--->	MAPK14	1.3429	-1.347	+
[7]	PRKCA	--->	SHC1	0.468	-0.43947	-
[8]	INS	<---	NR3C1	-0.085453	-0.068148	-
[9]	IL10RB	<---	THPO	-0.075265	0.16814	+
[10]	SRC	--->	TRPV1	0.44853	-0.34759	+
[11]	ARHGAP28	<---	RHOD	-1.5245	1.016	-
[12]	AXIN2	<---	PSMD7	-0.13899	0.081618	+
[13]	CHRM3	<---	PKN2	-0.1993	0.11589	-
[14]	GCK	<---	STAT1	-0.19153	0.15472	+
[15]	PRKD3	<---	STAT1	-0.067215	-0.065942	+
[16]	PIK3C2A	--->	PTK2B	-0.005256	-0.019304	-
[17]	ACTG1	<---	PXN	-0.36318	0.3394	+
[18]	WIF1	--->	WNT4	0.49247	-0.8346	+
[19]	FGF4	--->	KDR	0.21774	-0.0015767	+
[20]	BAMBI	--->	FZD2	0.43771	-0.51806	+
[21]	CD79B	--->	PIK3CD	-0.0062679	-0.006908	-
[22]	SHC2	<---	SYK	-0.71996	0.36756	+
[23]	PRKCI	--->	RASAL2	0.065883	-0.018231	-
[24]	BLNK	--->	SOS1	0.52612	-0.51133	+
[25]	CCNA2	<---	TFDP1	-0.19646	0.24842	-
[26]	ITGA1	--->	NTRK1	-0.018354	-0.059751	+
[27]	FCGR2A	<---	MAPK14	0.12024	0.14714	-
[28]	GNAI3	<---	RAPGEF3	-0.19611	0.45387	-
[29]	RHOH	--->	STARD13	0.10338	-0.50393	-
[30]	IFNLR1	<---	IL10	-0.20115	0.093836	+
[31]	GNB3	--->	GRM4	0.082045	-0.075766	-
[32]	CBLB	--->	VAV1	0.024154	-0.021931	+
[33]	PLD1	<---	PPAP2A	-0.019741	0.020601	-
[34]	CABLES1	--->	TP53	0.064257	-0.038691	-
[35]	RHOB	<---	TRIP10	0.074898	0.19853	+
[36]	COL4A4	<---	PTK2	-0.11304	-0.10314	-
[37]	GLI1	--->	WNT9A	0.86373	-1.47	+
[38]	E2F6	<---	EZH1	-0.21469	0.1814	-
[39]	ATR	<---	MAPK8	-0.35989	0.42273	-
[40]	CSNK1E	--->	WNT7A	0.83912	-0.78694	-
[41]	NPY	--->	NPY1R	0.89835	-1.3205	+
[42]	MAPK3	--->	NOTCH1	0.46221	-0.33075	+
[43]	JAK3	<---	PRKCE	-0.27725	0.5325	-
[44]	MAPK10	<---	RAC2	-0.20304	1.2491	+
[45]	AKT3	<---	PLCG1	0.010979	0.019996	+
[46]	LRP3	<---	PSEN2	-0.16338	0.1035	+
[47]	GRIA2	<---	PRKACA	-1.006	1.5014	+
[48]	TLR9	--->	UBE2V1	0.22217	-0.49454	+
[49]	BCL3	<---	RELA	-0.61683	0.50341	+
[50]	IL6	<---	PTPRE	-1.1386	0.97471	+
[51]	TLR8	--->	UBE2V1	0.22217	-0.49454	+
[52]	ARRB1	--->	FZD1	0.13556	-0.17053	+
[53]	PRKCB	--->	STAT5A	0.53803	-0.49743	-
[54]	ARHGAP30	<---	RHOC	-1.5245	1.016	-
[55]	PTK2	--->	THBS1	0.16475	-0.069329	-
[56]	PRKCZ	--->	SPHK1	-0.012216	-0.065283	+
[57]	PRKCE	--->	TIAM1	0.16	-0.14114	+
[58]	MAP2K4	<---	TRADD	-0.18726	-0.055605	+
[59]	MAP2K2	<--->	PRKX	0.31014	0.31014	-
[60]	ETV4	--->	REST	0.28002	-0.15205	-
[61]	CBLB	--->	CRLF2	0.18381	-0.1079	+
[62]	CBL	--->	IL2RB	0.28248	-0.14259	-
[63]	BHLHE40	<---	WHSC1	-0.39896	0.35984	-
[64]	CASP3	--->	PRKCQ	0.53516	-0.71981	+
[65]	AKT1	--->	VLDLR	0.095281	-0.088833	-
[66]	F11R	<---	PPP2CB	-0.23504	0.36585	+
[67]	CHRM5	--->	KCNJ6	0.11779	-0.2522	-
[68]	CCNK	--->	RXRA	-0.065965	-0.111	-
[69]	GNB5	<---	HTR1B	-0.92616	1.1575	+
[70]	RHOF	<---	SOS2	-0.33772	0.48674	+
[71]	SPTBN2	<---	SRC	-0.16875	0.23238	+
[72]	MAP2K6	<---	RAC1	-0.20304	1.2491	+
[73]	CTCF	<---	MTF2	-0.027621	0.094875	-
[74]	COL6A2	--->	ITGA8	-0.0092525	-0.030111	+

[75]	BIRC3	<---	TLR3	-0.11129	0.0037413	-
[76]	PRKCA	--->	STAT3	0.53803	-0.49743	-
[77]	ELMO1	<---	FGR	-0.45391	0.63648	+
[78]	SFRP2	--->	WNT5B	1.0317	-1.1063	+
[79]	TLR3	--->	UBE2D3	0.018863	-0.28222	-
[80]	FGF11	--->	FGFR2	0.40218	-0.67149	+
[81]	ID4	<---	SMAD4	-0.34215	0.18982	+
[82]	BRSK1	<--->	STK11	0.31014	0.31014	-
[83]	ATP1B3	<---	NR3C2	-0.14879	-0.015388	+
[84]	SETDB1	--->	ZFP69	0.16497	-0.10617	+
[85]	PPP2CA	--->	YWHAZ	0.18205	-0.13344	-
[86]	CTNNA3	--->	YWHAH	1.0272	-1.0665	+
[87]	CNR1	--->	GNB3	0.69217	-0.57296	+
[88]	CD8B	--->	IL2RG	0.046886	-0.10991	+
[89]	NRG1	<---	SRC	-0.22084	0.22446	+
[90]	MAPK14	<---	RAPGEF3	-0.093777	0.86463	+
[91]	C1QA	--->	SERPING1	0.20126	-0.32562	-
[92]	BCAR1	--->	CRKL	0.036273	-0.5203	+
[93]	SEH1L	--->	SRSF4	0.31391	-0.17132	+
[94]	CCNB2	<---	CKAP5	-0.42437	0.47972	-
[95]	IL26	--->	IL4R	0.073187	-0.17721	+
[96]	FZD5	<---	WNT9A	-0.78209	0.63734	+
[97]	COL2A1	<---	GP6	-0.12794	0.089198	-
[98]	CIITA	--->	SPI1	0.036535	-0.077722	-
[99]	NUMB	<---	PSMD14	-0.11462	0.1016	+
[100]	CCL21	<---	CXCR5	-0.35895	0.21658	-
[101]	CD274	<---	TRBV12-3	-0.0347	-0.027692	-
[102]	PDE4C	<---	PRKACA	-0.95055	1.4803	+
[103]	CRLF2	<---	IL6	-0.18506	0.094523	+
[104]	CCL14	<---	CXCR1	-0.35895	0.21658	-
[105]	CAMK2G	--->	CREB1	0.43562	-0.25046	+
[106]	ARNT	<---	CEBPB	-0.16173	0.16165	+
[107]	FGD4	<---	RAC1	-0.12937	0.14242	-
[108]	ALX4	<---	CTBP2	-0.25654	0.18028	+
[109]	TGFBR2	--->	XIAP	0.045687	-0.17424	+
[110]	APOA1	<---	PPARG	-0.048738	-0.017457	+
[111]	NFKBIA	<---	TRAF6	-0.59572	0.72072	+
[112]	CTNNB1	<---	PCDHB3	-0.19204	0.11503	-
[113]	BCL2L1	<---	PAX3	-0.40891	0.25225	+
[114]	NCOA1	<---	PPARGC1B	-0.0055094	0.020955	-
[115]	ATM	<---	BRCA1	-0.10817	0.11433	-
[116]	HIVEP1	<---	TCF7L2	-0.026148	0.31266	+
[117]	HCK	--->	PDGFB	0.37363	-0.26526	-
[118]	JUNB	<---	KAT2A	-0.083122	0.057371	+
[119]	GLI3	--->	WNT7B	0.86373	-1.47	+
[120]	MNX1	--->	TCF12	0.23888	-0.32236	-
[121]	CREBBP	--->	HK1	-0.00068954	-0.0041449	+
[122]	CCL4	<---	CX3CR1	-0.35895	0.21658	-
[123]	ANGPT4	--->	CSF1R	0.33967	-0.087672	+
[124]	EGFR	--->	IRS1	0.5854	-0.36048	+
[125]	IL6	<---	NFKB1	-0.23502	0.23585	+
[126]	MYH6	<---	NEB	-0.093801	0.057586	-
[127]	DRD5	<---	GRK6	-0.28786	0.33635	+
[128]	MAPK3	--->	PLA2G4B	0.18871	-0.84715	+
[129]	AGT	<---	RXRA	-0.16627	-0.076024	+
[130]	CTNND1	<---	PIK3CA	-0.04451	0.018807	-
[131]	WIF1	--->	WNT2	0.49247	-0.8346	+
[132]	BCL11A	<---	BLK	-0.302	0.48041	-
[133]	NET1	<---	RHOV	-0.23262	0.26814	-
[134]	PLEKHG5	<---	RHOB	-0.012974	0.047696	-
[135]	GADD45B	--->	MAP3K4	0.89119	-1.126	+
[136]	ARHGEF3	--->	RHOT2	0.33144	-0.31781	+
[137]	OXTR	--->	RHOA	0.12209	-0.13328	+
[138]	GNAI1	--->	PF4	0.087289	-0.054136	-
[139]	AMH	<---	RELA	-0.38713	0.18717	+
[140]	JAK2	--->	MAP2K1	0.43202	-1.1344	+
[141]	CCR5	--->	XCL2	0.21658	-0.35895	-
[142]	ORAI1	<---	STIM1	-0.88237	0.15971	+
[143]	DSCAM	<---	MAPK12	-0.10556	0.15829	-
[144]	MYL2	<---	MYL4	-0.45619	-0.35268	-
[145]	CC2D1A	--->	E2F1	0.16904	-0.33311	-
[146]	AP2S1	<---	RPS6KA3	-0.40283	0.20087	+
[147]	CD247	--->	IGKV4-1	-0.021439	-0.042516	-
[148]	CD247	<---	LYN	-0.27641	0.38494	+
[149]	GRB2	--->	IL2RG	0.13981	-0.11713	-
[150]	CD4	<---	RGS1	-0.22622	0.18095	+

[151]	BRAF	--->	PAK2	0.68927	-0.76991	-
[152]	ITGA5	<---	LAMB1	-0.066621	-0.0090065	+
[153]	GRIN2A	<---	RAC3	-0.38938	0.54247	-
[154]	CALML5	<---	ITPKC	-0.12569	0.087809	+
[155]	BRAF	--->	MAPK10	0.99795	-1.3266	+
[156]	FZD1	<---	WNT10B	-0.78209	0.63734	-
[157]	FGF2	--->	FGFR3	0.071829	-0.16555	+
[158]	GNAT2	--->	GNB5	0.13157	-0.0025637	-
[159]	PRKACB	<---	RAF1	-1.3266	0.99795	-
[160]	FGFR1	<---	VEGFB	-0.61313	0.56088	+
[161]	MAPK8	<---	ROCK2	-0.73578	0.41942	+
[162]	FGD2	<---	RHOU	-0.12937	0.14242	-
[163]	ALYREF	<---	NUP153	-0.54167	0.60094	+
[164]	FOXO4	--->	PLK2	1.0275	-0.62691	+
[165]	KRAS	--->	MAP3K3	1.1267	-0.17545	+
[166]	FZD10	<---	WNT4	-0.78209	0.63734	+
[167]	GNAT3	<---	TAS2R14	-0.011498	0.05262	+
[168]	CDH3	<---	MMP28	-0.24168	-0.0076207	+
[169]	BAD	<---	MAPK9	-0.56837	0.82969	+
[170]	CBL	--->	ERBB2	1.4559	-0.015521	+
[171]	COL5A2	<---	PCOLCE2	-0.15873	0.062969	+
[172]	ITGB3	--->	THBS4	0.066535	-0.040844	-
[173]	PDPK1	--->	PIK3CA	0.081383	-0.030162	-
[174]	MPL	--->	PLCB1	-0.0062892	-0.012639	+
[175]	AKT2	--->	GRB2	0.14037	-0.23377	-
[176]	CCL2	<---	REL	-0.69538	0.38373	+
[177]	FYN	--->	NRAS	0.61281	0.099664	+
[178]	CHUK	<---	PRKCD	-0.6951	0.07681	+
[179]	ETV4	<---	TGFBR2	-0.35031	0.21583	-
[180]	APBB1IP	--->	FGG	0.02593	-0.056145	-
[181]	EIF4E	<---	RAE1	-0.37256	0.38207	+
[182]	BIRC5	--->	PLK1	0.24874	-0.10908	-
[183]	GRB2	--->	IRS1	0.09185	-0.22736	-
[184]	SFRP4	--->	WNT6	1.0317	-1.1063	+
[185]	KDR	--->	ROCK1	0.079714	0.01731	-
[186]	AGTR2	--->	PLCB1	0.038179	-0.065598	+
[187]	RAP1B	<---	RASGRP1	-0.17492	0.5048	+
[188]	FCER1G	--->	PIK3R3	0.09585	-0.1984	+
[189]	OBSCN	<---	RHOU	-0.11324	0.22832	-
[190]	ECT2	--->	RHOT1	0.33144	-0.31781	+
[191]	E2F1	--->	KLF13	0.23995	-0.10098	+
[192]	ARHGEF18	<---	RHOF	-0.012974	0.047696	-
[193]	AJUBA	--->	CTCF	0.27332	-0.15173	-
[194]	F2RL2	<--->	F2RL3	0.14697	0.14697	-
[195]	IGKV1-5	<---	LYN	-0.47931	0.63774	+
[196]	FZD4	<---	WNT11	-0.78209	0.63734	+
[197]	FLT4	--->	HGF	0.052126	-0.049428	-
[198]	ADRBK2	--->	GRM3	0.53935	-0.4166	-
[199]	CCNB1	<---	SEH1L	-0.33759	0.44625	-
[200]	AKT2	<---	ERBB4	0.022566	0.027633	+
[201]	DAND5	--->	ITGAL	0.0088417	-0.1724	+
[202]	PBX2	--->	ZBTB7A	0.046782	0.029172	-
[203]	LRIG1	--->	TP53	-0.0017763	-0.053753	-
[204]	CSF1	--->	ITGAV	0.020108	-0.13626	+
[205]	APOBEC3G	<---	PSMD14	-0.21016	0.1406	+
[206]	ARHGAP39	<---	RHOBTB2	-0.50443	0.26296	-
[207]	HOXC6	--->	ZEB1	-0.077956	-0.17436	-
[208]	EP300	<---	HSPA8	-0.031607	0.02936	-
[209]	CDK11A	--->	ETS1	0.24561	-0.15935	-
[210]	PLA2G4E	<---	RASGRP1	-0.029717	0.042847	+
[211]	GNAO1	--->	IGF1R	0.2732	-0.0010398	-
[212]	CALML5	<---	PPP1CC	-0.26348	0.069904	+
[213]	CDK4	--->	TFDP1	0.34787	-0.22767	+
[214]	MAP3K1	--->	MAPK13	1.5096	-1.3549	+
[215]	GRIK5	<---	RHOQ	-0.38938	0.54247	-
[216]	ADCY3	<---	CALML5	-0.63894	0.65698	+
[217]	IL2RG	<---	JAK2	-0.1451	0.11865	-
[218]	CAMK4	--->	MEF2D	1.0903	-1.4506	+
[219]	GNB4	<---	PIK3CG	-0.018881	0.015862	-
[220]	BST2	<---	FOS	0.023792	0.063444	+
[221]	ADAMTS14	<---	COL5A2	-0.054632	-0.00053524	-
[222]	CREB1	<---	GDNF	-0.02286	0.050475	-
[223]	CAMK2D	<---	JAK2	-0.59	0.1682	-
[224]	PIK3CB	<---	SRC	-0.06481	0.084876	+
[225]	CYTH2	--->	EGF	0.11286	-0.083693	-
[226]	CACNA1C	<---	GNB3	-0.095409	0.10645	+

[227]	CD244	--->	SH2D1B	0.91475	-0.91492	+
[228]	ABCB1	<---	DAND5	-0.20523	0.13754	+
[229]	CCR6	--->	CXCL5	0.16688	-0.23229	-
[230]	CBL	--->	EPHA3	0.18892	-0.074222	-
[231]	AR	<---	CDK6	-0.27508	0.26535	-
[232]	CDC42	<---	IQGAP2	-0.076889	0.12856	-
[233]	KLRC2	<---	PLCG2	-0.021591	0.04532	-
[234]	ITGA6	--->	TNR	-0.015417	-0.048468	-
[235]	IL11RA	<---	IL3	-0.033963	-0.0073275	+
[236]	IL10	--->	IL7R	0.12248	-0.29036	+
[237]	HRAS	--->	RGL1	0.86481	-0.75112	+
[238]	GRK5	--->	HTR2A	0.33635	-0.28786	+
[239]	SH2B3	--->	SOCS3	0.16699	-0.16873	+
[240]	CTNND1	<---	PAK1	-0.32218	0.58879	+
[241]	AURKA	--->	CDC25B	0.26855	-0.97911	+
[242]	CDHR1	--->	CTNNB1	0.11503	-0.19204	-
[243]	DRD4	--->	GNAI2	1.1631	-1.1303	+
[244]	CALM1	--->	CAMKK2	0.41687	-0.23601	+
[245]	PLK1	<---	SLK	-0.098976	0.29896	+
[246]	CSF2	--->	ZEB1	0.37822	-0.24728	-
[247]	CSF2RA	<---	PRKACG	-0.27385	0.44193	+
[248]	DDX4	<---	HSP90AA1	-0.15268	0.12236	+
[249]	COL6A1	<---	ITGB5	-0.01668	-0.0099212	-
[250]	GATA2	--->	ZNF280A	0.28002	-0.15205	+
[251]	PRF1	--->	STAT5B	0.47814	-0.37844	-
[252]	DAND5	<---	UTRN	-0.060107	0.017005	-
[253]	CXCL2	<---	CXCR2	-0.35895	0.21658	-
[254]	FOXA1	--->	HDAC1	0.74871	-0.64672	+
[255]	GRIN2A	<---	PRKACG	-1.006	1.5014	+
[256]	CSF2	--->	OSMR	0.16017	-0.20921	+
[257]	EPHB2	--->	MMP9	-0.015392	-0.077231	-
[258]	PLCG1	--->	TCRB	0.045786	-0.022856	-
[259]	DVL1	<---	WNT8A	-0.54867	0.38842	+
[260]	BID	<---	FASLG	-0.49686	0.31206	+
[261]	ACTR2	<---	MMP28	-0.29263	0.085904	+
[262]	FZD5	<---	WNT2	-0.78209	0.63734	+
[263]	CEBPB	<---	MIA	-0.12174	0.043175	-
[264]	VEGFA	--->	ZBTB7A	0.12847	-0.085273	-
[265]	CBL	--->	HSP90AA1	0.13547	-0.053813	-
[266]	DUSP1	--->	USF2	0.81499	-0.74592	-
[267]	PPP2R1A	--->	YWHAH	0.1764	-0.3228	-
[268]	F2	--->	SERPIND1	0.18568	-0.33063	-
[269]	DAPP1	<---	NFATC1	-0.41714	0.54272	-
[270]	TICAM2	<---	TRAF3	-0.21928	0.42938	-
[271]	EZR	<---	SLC9A1	-0.064875	-0.00012811	+
[272]	REL	--->	TNFSF10	0.93112	-1.1394	+
[273]	PIK3CA	--->	PLD2	0.0053638	-0.0095341	+
[274]	CUL3	--->	PSMD4	0.16421	-0.24949	-
[275]	PPP2R1B	--->	YAP1	0.19485	-0.34161	+
[276]	IRS4	--->	SOCS1	0.093091	-0.31853	-
[277]	ARHGEF16	<---	RHOH	-0.067065	0.085273	-
[278]	RHOU	--->	SRGAP3	0.39011	-0.42732	-
[279]	INS	<---	RPS6KB1	-0.31439	0.10614	+
[280]	LAMA2	--->	SV2B	0.099369	-0.11816	+
[281]	MRAS	--->	RAF1	0.78219	-0.86318	+
[282]	ITGA2	<---	TNXB	-0.076459	-0.047786	+
[283]	IL12RB1	<---	SLA2	-0.31251	0.22719	-
[284]	APOA4	--->	HNF4A	0.065867	-0.21743	-
[285]	ARHGEF3	<---	RHOC	-0.012974	0.047696	-
[286]	H2AFZ	<---	TAF1	-0.32305	0.21329	+
[287]	EGFR	--->	FGF4	0.25979	-0.22928	-
[288]	CSH1	--->	IL11RA	0.084246	-0.14339	+
[289]	SMAD3	<---	ZFYVE9	-0.039669	-0.020213	+
[290]	IL4R	--->	STAT6	0.14129	-0.10714	+
[291]	TFDP1	--->	ZNF263	0.098413	0.0022878	-
[292]	GRK4	--->	OPRD1	0.33635	-0.28786	+
[293]	BMP6	<---	SMAD1	-0.24379	0.060804	-
[294]	MDM2	--->	RANGAP1	0.048193	-0.088875	-
[295]	DNM1	--->	NTRK1	-0.022652	-0.056599	+
[296]	CSNK1G3	--->	WNT3A	0.50785	-0.4784	-
[297]	MYL12A	--->	ROCK1	0.31451	-0.08875	-
[298]	LCK	--->	PLCG1	0.066589	-0.05234	+
[299]	IL1B	<---	MAPK1	-0.078488	0.15109	+
[300]	FOXA1	--->	FST	0.057907	-0.12177	+
[301]	ETV4	--->	JUND	0.16075	-0.22115	-
[302]	MYH8	--->	MYL2	0.18892	-0.22718	-

[303]	BTK	--->	DAND5	0.20381	-0.049974	-
[304]	IL11RA	<---	PIK3CA	-0.01992	0.0073949	-
[305]	GNAI2	<---	KCNJ9	-0.011498	0.05262	-
[306]	ARF6	--->	VAV2	0.05913	-0.025623	-
[307]	NRAS	<---	RASGRP4	-0.10998	0.089485	+
[308]	HLF	--->	REST	0.28002	-0.15205	-
[309]	EPHA2	<---	HCK	-0.26855	0.35424	+
[310]	PLK1	<---	UBE2C	-0.15699	0.38608	-
[311]	PRKACB	--->	TTF1	0.40675	-0.26091	+
[312]	CASP1	<---	TP53	-0.087276	0.040162	+
[313]	ARNT	<---	ENO3	-0.099193	0.096943	-
[314]	ITSN1	--->	RHOF	0.16745	-0.11028	+
[315]	CTBP2	--->	WNT5A	0.18271	-0.36436	+
[316]	FXYD2	--->	PIK3CD	-0.0050831	-0.010841	-
[317]	NCOA3	<---	SPI1	-0.061263	0.057699	+
[318]	RXRA	<---	TBL1XR1	-0.12811	-0.068767	-
[319]	ACTG2	<---	RHOQ	-0.51168	0.52339	+
[320]	BATF	<---	CHD6	-0.18206	0.11598	-
[321]	TXK	--->	VAV3	0.072747	-0.065501	+
[322]	MAP2K3	<---	MAP3K1	-0.22981	0.6879	+
[323]	THBS1	<---	WT1	-0.15361	0.47374	+
[324]	ACVR2A	--->	SMAD1	0.6082	-0.49302	+
[325]	C2	--->	FCN3	-0.011958	-0.022757	-
[326]	GNB4	--->	WNT5B	0.3343	-0.42584	-
[327]	HDAC4	<---	NUP153	-0.53842	0.60929	+
[328]	DHX9	<---	NFKB1	-0.1408	0.13949	-
[329]	CCNB1	<---	NCAPG	-0.40564	0.45419	-
[330]	ARAP3	<---	RHOT2	-0.19281	0.10567	-
[331]	SMARCD3	--->	SP1	0.29622	-0.181	+
[332]	ICAM1	<---	PLCG1	-0.048094	0.051968	-
[333]	CAMK2A	--->	SCN5A	0.43397	-0.32993	+
[334]	DUSP14	--->	MAPK13	1.4184	-1.4202	+
[335]	FGR	--->	PIK3CA	0.084876	-0.06481	+
[336]	DAND5	--->	HMOX1	0.036704	-0.072158	+
[337]	IL13RA2	<---	PRL	-0.18506	0.094523	+
[338]	EPHA2	<---	RASA1	-0.09886	-0.072416	-
[339]	CDH3	<---	MMP12	-0.24168	-0.0076207	+
[340]	PRKCD	--->	TRPV4	0.34142	-0.37962	+
[341]	ARHGAP31	<---	RHOC	-1.5245	1.016	-
[342]	PRKCE	--->	RASA1	0.06376	0.037455	-
[343]	ITGAM	<---	PIK3R3	-0.12063	-0.07193	-
[344]	PDPK1	--->	STRAP	0.66642	-0.19947	+
[345]	KRAS	<---	SPTA1	-0.048099	0.21387	+
[346]	RANBP2	--->	SRSF1	0.34767	-0.31854	+
[347]	CHAD	--->	ITGB5	0.035642	-0.083698	+
[348]	IL19	--->	IL20RA	0.20113	-0.087402	+
[349]	FGD1	<---	RHOBTB1	-0.087343	0.096875	-
[350]	GLI1	--->	SUFU	0.14383	-0.059407	-
[351]	BCL2	<---	PRKAR2B	-1.0163	0.7413	+
[352]	CCR8	--->	GNAI3	1.1631	-1.1303	+
[353]	JAK2	--->	PIK3CG	0.020536	-0.05391	+
[354]	CDK1	<---	CLASP1	-0.015373	0.11246	-
[355]	COL6A2	--->	ITGA5	-0.0092525	-0.030111	+
[356]	NUMB	--->	PSMD10	0.2371	-0.194	-
[357]	CXCR4	<---	SSH1	-0.5177	0.35324	-
[358]	RHOV	<---	SOS2	-0.33772	0.48674	+
[359]	ETS1	--->	ITGA2B	-0.0090065	-0.066621	+
[360]	CD44	<---	LAMA1	-0.06897	0.0013783	+
[361]	FZD2	<---	MAPK10	-0.51612	0.5614	-
[362]	SLTM	<---	SMAD7	-0.21482	0.14684	+
[363]	FOXA2	--->	NR0B2	0.27471	-0.36003	+
[364]	ERBB3	--->	SHC1	1.2029	-1.0554	+
[365]	ADCY7	<---	RAPGEF4	-0.28599	0.53533	-
[366]	NCOA1	<---	TNF	-0.063102	0.054132	+
[367]	CCR6	--->	CXCL2	0.21658	-0.35895	-
[368]	CTCF	<---	E2F4	0.009671	0.068465	+
[369]	ATF2	<---	PPP1CC	-0.66816	0.44089	+
[370]	CREB1	<---	PRKACA	-0.35231	0.65748	+
[371]	SAG	--->	WNT7B	0.23514	-0.34616	+
[372]	PLEKHG2	<---	RHOB	-0.23262	0.26814	-
[373]	KLF1	--->	TBP	0.1822	-0.096587	-
[374]	GAB2	<---	HCK	-1.1861	0.83596	+
[375]	CD3D	--->	PIK3CA	-0.0050831	-0.010841	+
[376]	MAP2K6	<--->	PRKACA	0.31014	0.31014	-
[377]	PLA2G6	<---	PRKCA	-0.21133	0.19522	+
[378]	CD247	--->	GNAZ	0.030816	-0.0080738	-

[379]	DNER	<---	PSEN2	-0.34905	0.24464	+
[380]	ITGAX	<---	PTK2	-0.084093	-0.024145	-
[381]	MYH9	--->	USF1	0.16008	-0.19465	-
[382]	MAPK12	<---	TNF	0.048942	0.10494	-
[383]	FZD1	<---	WNT11	-0.78209	0.63734	+
[384]	ADRB2	<---	ADRBK2	-0.21301	0.25327	+
[385]	CD247	--->	JUN	0.096401	-0.13523	+
[386]	IL12RB2	<---	SOCS4	-0.10625	0.079572	+
[387]	MEF2C	<---	TLR6	-0.3634	0.073374	-
[388]	GP5	--->	PIK3R3	0.32086	-0.62431	+
[389]	CDC42	<---	NGFR	-0.26604	0.057257	+
[390]	BRCA1	--->	SPI1	0.30904	-0.32545	-
[391]	AKT1	--->	GNAI3	0.1635	-0.22536	-
[392]	ACTB	<---	MYO10	-0.10458	0.07416	+
[393]	GRK5	--->	SSTR1	0.33635	-0.28786	+
[394]	FOS	<---	SFRP1	-0.43315	0.59036	-
[395]	GRIN2C	<---	PRKX	-1.006	1.5014	+
[396]	NCOA3	<---	RARA	-0.054726	0.035483	+
[397]	WIF1	--->	WNT11	0.49247	-0.8346	+
[398]	PRKCI	--->	STAT5B	0.53838	-0.49624	-
[399]	IGHD	<---	SYK	-0.38078	0.20643	-
[400]	IL3	--->	JUND	0.069178	-0.11172	-
[401]	CDH1	<---	MMP25	-0.24168	-0.0076207	+
[402]	CASP3	--->	SREBF1	0.65399	-1.0089	+
[403]	DLK1	--->	HEY1	0.12507	-0.21211	-
[404]	NOTCH1	--->	RBPJ	0.48073	-0.35488	-
[405]	DDX20	--->	EGR1	0.18149	-0.10437	-
[406]	CASP2	<---	TRAF2	-0.28901	0.21849	+
[407]	EGFR	--->	UBE2D3	0.20569	-0.1777	-
[408]	CRKL	<---	FGFR2	-0.10344	-0.046426	+
[409]	DCN	--->	TGFB3	0.6954	-1.2337	+
[410]	E2F1	--->	MESP2	0.45267	-0.59164	+
[411]	CD44	<---	COL2A1	-0.17689	0.042749	+
[412]	JUND	<---	MAPK14	-0.47491	0.66358	+
[413]	GRB14	<---	SGK1	-0.064856	0.25933	-
[414]	MYL7	--->	MYLK	0.33394	-0.31058	-
[415]	MAPK1	--->	PTPN11	1.0948	-1.0232	-
[416]	LMOD1	<---	MYL12B	-0.26749	0.19644	+
[417]	DRD2	--->	GNB1	0.69217	-0.57296	+
[418]	SIPA1	--->	YWHAZ	0.055611	0.036859	-
[419]	EP300	--->	NFIL3	0.00067605	-0.0050208	+
[420]	CACNA1C	<---	PPP2R1A	-0.39164	0.37653	+
[421]	MYLK3	<---	PAK3	-1.2674	1.5523	+
[422]	EGF	<---	NRAS	-0.09589	0.11136	-
[423]	CPS1	<---	EP300	-0.006286	0.0019904	+
[424]	MAP3K7	--->	RGMB	0.29084	-0.37186	+
[425]	MYL12B	--->	ROCK1	0.31451	-0.08875	-
[426]	WIF1	--->	WNT7A	0.49247	-0.8346	+
[427]	CTNNA1	--->	GNA13	0.70036	-0.61326	-
[428]	FGR	--->	PIK3R5	0.79939	-0.64452	+
[429]	CSNK1A1	--->	WNT8B	0.83912	-0.78694	-
[430]	FZD7	<---	GNB4	-0.19257	0.19185	-
[431]	GNA14	--->	WNT8B	0.42087	-0.47341	-
[432]	BCL3	<---	NPAS4	-0.082782	0.17544	-
[433]	NOTCH3	<---	THRA	-0.13359	0.17288	+
[434]	GRIP2	<---	PRKCB	-0.40252	0.33758	+
[435]	CD3G	<---	SYK	-0.22013	0.087709	-
[436]	FZD10	<---	GNB3	-0.12321	0.10912	-
[437]	ESR1	--->	HES2	0.26687	-0.42437	+
[438]	ARHGAP24	<---	RHOH	-0.49576	0.47865	-
[439]	IL1R1	--->	MAP2K6	1.0044	-0.55666	+
[440]	STAT1	--->	THPO	0.25961	-0.32133	-
[441]	EGR2	<---	NFATC3	-0.57388	0.50851	-
[442]	ADRB2	--->	GNAI3	1.1631	-1.1303	+
[443]	AURKB	--->	PLK1	0.29896	-0.098976	-
[444]	ADAM17	--->	NGFR	-0.061676	-0.075813	+
[445]	PKMYT1	--->	RPS6KA3	0.9519	-1.129	-
[446]	RAP1B	<---	VAV1	0.1025	0.10902	-
[447]	ITGA3	<---	LAMB4	-0.047832	-0.013442	+
[448]	AKT2	--->	PPP6C	-0.032241	-0.11483	+
[449]	CTCF	<---	FOXS1	-0.13257	0.21979	-
[450]	CEBPB	--->	SMAD6	-0.050142	-0.10303	+
[451]	CDH1	<---	PSEN2	-0.39844	0.072753	+
[452]	CD3G	<---	CSK	-0.45391	0.63648	-
[453]	EGF	<---	EPS15	-0.14831	0.13459	-
[454]	E2F1	<---	IRF2BPL	-0.36552	0.36011	-

[455]	FGF13	--->	KIT	0.017168	-0.013685	+
[456]	COL3A1	--->	PTK2B	-0.055436	-0.087563	-
[457]	LZTR1	--->	MAX	0.26575	-0.31912	-
[458]	IL20RA	<---	THPO	-0.075265	0.16814	+
[459]	ERBB4	--->	PIK3CB	0.031013	-0.032307	+
[460]	HMGB1	<---	RELA	-0.36587	0.18582	-
[461]	GNA14	--->	WNT5A	0.42087	-0.47341	-
[462]	IL6ST	<---	PIK3CD	-0.016602	0.0042292	-
[463]	E2F6	--->	RBBP4	0.060123	-0.046997	+
[464]	MYLK	--->	NTRK1	-0.14463	-0.24987	+
[465]	COL4A6	--->	ITGA5	-0.013036	-0.10803	+
[466]	CACNA1F	<---	KCNMA1	-0.21987	-0.071398	+
[467]	GNAI3	<---	MAPK12	-0.22953	0.50225	-
[468]	BTK	--->	PIK3R2	0.039717	-0.090918	-
[469]	DAPP1	<---	MAP3K1	-0.39039	0.7413	-
[470]	CCNB2	<---	CLASP1	-0.38312	0.41421	-
[471]	KCNQ4	--->	TRPC4	-0.051301	-0.15927	+
[472]	TCF12	--->	THBS1	1.2901	-0.8282	+
[473]	ADRA2B	<---	GRK1	-0.17569	0.20291	+
[474]	ANGPT1	--->	GRB7	0.10607	0.027741	+
[475]	GNAI1	<---	RGS14	-0.094828	0.065188	+
[476]	EGR1	--->	JUN	0.094536	-0.025299	+
[477]	DVL3	<---	FZD8	-0.33521	0.24353	+
[478]	SFRP5	--->	WNT5B	1.0317	-1.1063	+
[479]	IL20RA	<---	PIK3CA	-0.012343	0.023213	-
[480]	CDH1	<---	ZBTB33	-0.17752	0.026264	+
[481]	CALML3	--->	NOS1	0.032614	-0.032827	+
[482]	PARD6A	--->	TIAM1	0.034339	-0.019415	+
[483]	EPOR	--->	SH2B3	0.10672	-0.030455	-
[484]	CEBPB	--->	ZNF280A	0.28002	-0.15205	+
[485]	DAB1	--->	VLDLR	0.19906	-0.14124	-
[486]	CCL28	<---	CXCR4	-0.35895	0.21658	-
[487]	GYS2	<---	PPP1CB	-0.9036	0.53904	+
[488]	CTBP2	--->	HOXA9	0.04296	-0.16727	+
[489]	MAPK14	<---	NRAS	-0.12425	0.75653	+
[490]	SHC3	<---	SRC	-0.49397	0.48646	-
[491]	CBLB	--->	IL12RB1	0.3001	-0.17434	+
[492]	PAFAH1B1	--->	PLK1	0.14873	0.015926	-
[493]	EP300	--->	HP	0.00067605	-0.0050208	+
[494]	COMP	<---	ITGA4	-0.021497	-0.014728	-
[495]	CDKN1B	<---	SKP1	-0.032551	0.085167	-
[496]	MAPK1	--->	SETDB1	0.099401	-0.034028	-
[497]	CCR2	<---	GRK1	-0.28786	0.33635	+
[498]	F2RL3	--->	PRSS3	0.11779	-0.2522	-
[499]	NFATC4	--->	ZEB1	0.42982	-0.47113	-
[500]	MAP3K5	<---	PPP5C	-0.43287	0.49678	+
[501]	KCNMB1	<---	PRKX	-0.31173	0.40189	+
[502]	NCK1	--->	PAK6	0.73462	-0.72841	+
[503]	GNAI2	<---	MAPK12	-0.22953	0.50225	-
[504]	PRKCI	--->	RAF1	0.1479	-0.22585	+
[505]	NR3C1	--->	TP53	0.092108	-0.12718	+
[506]	ETS1	--->	MPL	0.033006	-0.069846	+
[507]	PIK3CD	<---	SRC	-0.06481	0.084876	+
[508]	CCR5	--->	CXCL3	0.21658	-0.35895	-
[509]	CACNB1	<---	PPP2R1A	-0.20392	0.21525	+
[510]	FZR1	--->	H3F3A	0.15811	-0.17998	-
[511]	PIK3R3	--->	PLCG1	0.050029	0.0024742	+
[512]	ANAPC2	--->	CCNB3	0.54369	-0.49444	+
[513]	CDK2	--->	FOXM1	0.2499	-0.22085	+
[514]	GLI3	--->	WNT2	0.86373	-1.47	+
[515]	MAX	<---	POU2F2	-0.19662	0.14379	-
[516]	CXCL5	<---	CXCR5	-0.23229	0.16688	-
[517]	MAPK14	<---	STAT1	-1.2527	1.4568	-
[518]	CXCL2	<---	CXCR3	-0.35895	0.21658	-
[519]	PLEKHG5	<---	RHOJ	-0.012974	0.047696	-
[520]	IL13RA2	<---	SOCS7	-0.10066	-0.08965	+
[521]	ATP1A3	<---	PRKACA	-0.09775	0.14787	+
[522]	RYR1	<---	SRI	-0.013867	0.010071	+
[523]	FLT1	--->	RASA1	0.24802	-0.11395	+
[524]	GAB1	<---	PIK3R3	-0.54679	-0.10697	-
[525]	RHOB	--->	SYDE1	1.016	-1.5245	-
[526]	FGF4	<---	KRAS	-0.25726	0.38422	-
[527]	ADCY9	<---	PRKCA	-0.49495	0.42642	+
[528]	CTNNA1	--->	PTPN11	0.10008	0.0018819	+
[529]	ARHGEF17	<---	RHOQ	-0.012974	0.047696	-
[530]	APBB3	--->	BACE2	0.074889	-0.18174	-

[531]	KCNQ5	--->	TRPC3	0.011076	-0.12465	-
[532]	SNRPD3	--->	WDR77	0.074498	-0.10631	-
[533]	DUSP7	--->	MAPK12	1.353	-1.036	+
[534]	IL3RA	<---	PRKACB	-0.27385	0.44193	+
[535]	MCF2L	<---	RHOQ	-0.026438	0.029604	-
[536]	CDC42	<---	GRAP2	0.0030067	0.63892	+
[537]	MAPK3	<--->	MAPKAPK2	0.31014	0.31014	-
[538]	SIX5	--->	ZNF565	0.078944	0.049909	+
[539]	ARHGAP10	<---	RHOC	-0.65972	0.48549	-
[540]	IRF2	<---	SRF	-0.35947	0.27595	+
[541]	IL6	<---	RELA	-0.46471	0.18561	+
[542]	ASCL1	--->	STAT3	0.19378	-0.12088	-
[543]	MTOR	<---	PRKAG1	-1.2269	1.2782	+
[544]	GATA2	<---	TLR4	-0.43236	0.032528	-
[545]	CCND3	<---	NCOA2	-0.019866	0.030334	-
[546]	CRHR1	<---	GNAI2	-0.040633	0.044298	-
[547]	TAF1	<---	TRIM25	-0.031649	0.26583	-
[548]	CDC42	--->	GIT2	0.02238	-0.11262	-
[549]	LRP1B	<---	MMP14	-0.11665	0.12325	+
[550]	ESR1	--->	TIMP4	-0.015388	-0.14879	+
[551]	CCL11	<---	CXCR1	-0.35895	0.21658	-
[552]	ADCY1	<---	GNAT3	-0.63317	1.0384	-
[553]	GNA11	--->	PLA2G4E	-0.10668	-0.13723	+
[554]	AKT3	--->	PDE3B	0.55876	-0.51658	+
[555]	COL11A1	<---	PCOLCE2	-0.32363	0.21278	+
[556]	CNOT6	--->	EIF4A2	0.034994	-0.11982	+
[557]	HLA-DQA2	<---	LCK	-0.3621	0.5114	-
[558]	FGF18	--->	FGFR3	0.071829	-0.16555	+
[559]	JAM3	<---	PRKCQ	-0.1346	0.18364	+
[560]	COL2A1	--->	ITGB5	0.0024845	-0.035647	+
[561]	EGFR	--->	SOS2	0.07671	-0.044447	+
[562]	MAPK10	<---	RHOG	-0.20304	1.2491	+
[563]	CSF3R	<---	JAK1	-0.14471	0.060656	-
[564]	BIRC3	--->	FASLG	0.015949	-0.093263	+
[565]	HTR1B	<---	NPY	-1.3205	0.89835	+
[566]	CDC42	--->	MAPK12	1.2491	-0.20304	+
[567]	CER1	--->	WNT8B	0.60582	-0.57968	+
[568]	GATA1	--->	STAT5A	0.26825	-0.19721	+
[569]	ROBO2	<---	SRGAP1	-0.19806	0.11796	-
[570]	CD40LG	--->	NFKBIA	0.0082832	-0.19123	+
[571]	MAPK14	--->	PLCB2	0.13237	-0.16722	-
[572]	AXIN1	<---	CSNK1G3	-0.12485	0.13422	+
[573]	RANBP2	--->	SRSF9	0.34767	-0.31854	+
[574]	GH2	--->	GHR	0.073187	-0.17721	+
[575]	CD19	--->	VAV2	0.17916	-0.063802	-
[576]	CSF2RB	<---	SOCS4	-0.16792	0.11632	+
[577]	EP300	--->	FGFR2	0.0025327	-0.014064	+
[578]	JAK3	--->	PDPK1	0.2569	-0.48656	+
[579]	MMP23A	--->	POTEKP	0.38294	-0.43788	+
[580]	MAPK3	--->	SHC3	0.70518	-0.81557	+
[581]	GRK1	--->	HRH1	0.33635	-0.28786	+
[582]	GMIP	<---	RHOA	-1.011	0.68428	-
[583]	CRLF2	<---	PRL	-0.18506	0.094523	+
[584]	TICAM2	--->	UBE2D3	0.28095	-0.27162	-
[585]	GAB2	<---	LMO4	-0.63682	0.80353	+
[586]	AKT2	--->	CREB3L2	0.82132	-1.3257	+
[587]	ITGAE	<---	PTK2	-0.084093	-0.024145	-
[588]	GNB2	<---	MAPK12	-0.31609	0.54982	-
[589]	ADAM10	--->	JAG1	0.017463	-0.033173	+
[590]	OBSCN	<---	RHOJ	-0.11324	0.22832	-
[591]	RAPGEF4	--->	RRAS2	0.78832	-0.62373	+
[592]	FGF2	<---	HOXB7	-0.0596	0.064226	+
[593]	DAND5	--->	TFPI2	0.036704	-0.072158	+
[594]	MYH8	<---	TNNC1	-0.032012	0.012494	-
[595]	BCL11A	<---	CYR61	-0.028943	0.050795	-
[596]	CDC42	--->	NTRK2	-0.037856	-0.071723	-
[597]	CEBPB	<---	UBQLN1	-0.098952	0.044375	-
[598]	AVPR1B	--->	GNA14	1.1631	-1.1303	+
[599]	EGFR	--->	PIK3CA	0.031013	-0.032307	-
[600]	DVL2	<---	WNT16	-0.54867	0.38842	+
[601]	CALML5	--->	RASGRF2	0.29154	-0.36654	+
[602]	CD8A	--->	CREB1	0.13924	-0.092878	-
[603]	CALM1	--->	GATA1	0.11805	-0.16334	-
[604]	ATP2A2	--->	NOTCH1	0.16442	-0.12186	+
[605]	FCER1G	<---	HRAS	-0.01596	0.026206	+
[606]	ARHGAP24	<---	RHOBTB2	-0.30179	0.28922	-

[607]	DVL1	<---	FZD2	-0.33521	0.24353	+
[608]	CCNA2	<---	MDM2	-0.29258	0.26558	-
[609]	FYN	--->	RGS1	0.53308	-0.35367	+
[610]	CD247	<---	JAK2	-0.13461	0.052099	+
[611]	HLTF	<---	MAX	-0.081175	0.088104	+
[612]	ARNT	--->	FLT1	-0.036285	-0.060074	+
[613]	FCER1A	--->	LCP2	0.31147	-0.37956	+
[614]	NTRK1	--->	PLCG1	-0.018287	-0.019144	-
[615]	MYC	<---	ZMYND8	-0.28761	0.073333	-
[616]	CRK	--->	ITGA4	0.032574	-0.078547	-
[617]	SMAD3	<---	WWTR1	-1.0153	1.0527	-
[618]	FGR	--->	SLA	0.41943	0.028419	-
[619]	ALYREF	<---	RANBP2	-0.31854	0.34767	+
[620]	MAPK8	<---	NCK1	-0.31116	0.22669	+
[621]	JUP	<---	MMP24	-0.26058	0.11034	+
[622]	GRIN2B	<---	RAC3	-0.38938	0.54247	-
[623]	CCL14	<---	CCR9	-0.35895	0.21658	-
[624]	ERCC6L	--->	PLK1	0.14995	-0.036163	-
[625]	DNM1	<---	GNA14	-0.041492	0.069455	+
[626]	CD40	<---	MAPK11	-0.57721	0.64173	-
[627]	IL7R	--->	SOCS3	-0.16125	-0.20399	-
[628]	DCN	--->	JUN	0.21563	-0.34041	-
[629]	CTNNB1	<---	STRAP	-0.35456	0.3165	+
[630]	CCND2	<---	FOXO4	-0.62791	0.70491	+
[631]	PRKACA	<--->	STRADA	0.31014	0.31014	-
[632]	FOS	<---	LPAR3	-0.36998	0.41745	+
[633]	CALM1	--->	MAP2K3	0.2245	-0.20028	+
[634]	MAP2K3	--->	PEBP1	0.19282	-0.25178	-
[635]	EP300	--->	SLC9A1	0.00067605	-0.0050208	+
[636]	ARHGAP11A	<---	RHOG	-1.5245	1.016	-
[637]	ARHGAP11A	<---	RAC3	-1.5245	1.016	-
[638]	MAPK8	<---	ROCK1	-0.4474	0.43952	+
[639]	CDH3	<---	MMP20	-0.24168	-0.0076207	+
[640]	SGK1	--->	TAL1	0.31275	-0.45754	-
[641]	COL6A1	--->	ITGA3	-0.014544	-0.025688	+
[642]	CSF2	<---	PRKACB	-0.1175	0.35399	-
[643]	PIK3CD	<---	ZAP70	-0.033797	0.011356	+
[644]	CHN1	<---	RHOD	-0.4978	0.36255	-
[645]	ARRB1	--->	FZD9	0.13556	-0.17053	-
[646]	SPI1	--->	TNF	-0.056467	-0.069238	+
[647]	MYH14	<---	RHOA	-0.01596	0.026206	+
[648]	EIF4E2	<---	MKNK1	-0.73366	0.88689	+
[649]	CREB1	--->	INHBA	0.063524	-0.11958	+
[650]	ARHGEF7	<---	RAC2	-0.044321	0.055318	-
[651]	PLCB2	<---	RGS17	-0.075217	0.041005	+
[652]	CTBP1	--->	RBPJ	0.030403	-0.04043	-
[653]	GATA2	<---	SP100	-0.042674	0.055951	-
[654]	FOXA1	<--->	FOXQ1	0.41711	0.41711	-
[655]	CD3E	--->	JUN	0.16075	-0.22115	+
[656]	NFKB1	--->	TNFRSF11A	0.15339	-0.16101	-
[657]	FADD	--->	IKBKG	0.48004	-0.69432	+
[658]	MYH9	<---	RHOC	-0.01596	0.026206	+
[659]	APP	--->	JUN	0.01063	-0.020718	-
[660]	SMAD2	--->	TGFB2	0.060804	-0.24379	-
[661]	COL24A1	--->	ITGA4	-0.013036	-0.10803	+
[662]	EP300	--->	MYF6	0.024715	-0.02899	+
[663]	ROCK1	--->	VEGFA	0.068522	0.046215	-
[664]	MAP3K5	<---	RAC3	-0.38976	1.4785	+
[665]	ARNT	<---	EGLN2	-0.50456	0.44408	-
[666]	GRB2	<---	SHC3	0.018816	0.29675	+
[667]	MCF2L	<---	RAC2	-0.026438	0.029604	-
[668]	SFRP1	--->	WNT9A	1.0317	-1.1063	+
[669]	IL1RN	--->	JUN	0.18782	-0.2509	-
[670]	GNAI1	<---	HCK	-0.44236	0.67161	-
[671]	ETV4	<---	STK11	-0.26091	0.40675	+
[672]	CTNNB1	<---	PCDHA7	-0.19204	0.11503	-
[673]	FXYD2	<---	PRKX	-0.26091	0.40675	+
[674]	GNB2	--->	WNT3	0.3343	-0.42584	-
[675]	ERCC6	--->	MAX	0.141	-0.19411	-
[676]	CEACAM1	--->	HEY1	0.12772	-0.23541	-
[677]	NFATC4	--->	TNF	0.93112	-1.1394	+
[678]	JUN	<---	VEGFA	-0.1628	0.12363	-
[679]	HDAC3	<---	TNF	-0.32473	0.35108	+
[680]	GADD45B	--->	TP53	0.25242	-0.28587	-
[681]	RAC1	<---	TRAF6	-0.44185	0.67631	+
[682]	PRKAR1A	<---	PRKG1	0.12108	0.63043	+

[683]	GRK4	--->	GRM6	0.80802	-0.62351	+
[684]	EPOR	--->	VAV2	0.030275	-0.0056667	+
[685]	IL4R	<---	MAPK14	-0.30945	0.3459	-
[686]	FLT1	--->	PIK3CB	0.043149	-0.0082223	+
[687]	CDK1	--->	PSMD7	0.2441	-0.083683	-
[688]	EPHA3	--->	RHOA	0.29551	-0.29565	+
[689]	GSK3B	--->	PIK3CD	0.061458	-0.046432	-
[690]	ARHGEF11	<---	GNA12	0.011916	0.034755	+
[691]	EP300	--->	LDHA	-0.00068954	-0.0041449	+
[692]	GNAI1	--->	GRM6	0.24033	-0.15623	-
[693]	CALML6	<---	ITPKB	-0.65345	0.74578	+
[694]	ARF6	<---	HTT	-0.49203	0.32422	-
[695]	MAPK6	--->	SPIC	0.40675	-0.26091	+
[696]	PRKAR1A	--->	PXN	0.1254	-0.15337	+
[697]	FGFR3	--->	SHC4	0.23069	-0.27006	+
[698]	GHR	--->	STAT5B	0.23476	-0.17484	+
[699]	PPP3R1	<---	YWHAH	-0.13033	-0.078387	-
[700]	GHR	<---	IRS2	0.018455	0.089853	-
[701]	ARHGAP8	<---	RHOBTB2	-0.34208	0.22384	-
[702]	ARHGAP25	<---	RHOT1	-0.52838	0.35211	-
[703]	CD3E	<---	LCK	-0.45391	0.63648	+
[704]	FYN	--->	PTK2	0.34449	-0.5161	-
[705]	PRKCH	--->	SOS1	0.13325	-0.18146	-
[706]	MAP2K1	--->	YWHAE	0.079981	-0.071802	-
[707]	LPAR2	<---	NFKB1	-0.43941	0.47827	-
[708]	IL3	<---	PRKACA	-0.41189	0.58717	-
[709]	HCK	--->	TRPV6	0.44853	-0.34759	+
[710]	CD4	<---	GNAI1	-0.052939	0.061543	+
[711]	CDKN1A	<---	RBX1	-0.21238	0.29674	+
[712]	CCR6	--->	PF4V1	0.21658	-0.35895	-
[713]	CTBP1	--->	RBPJL	0.061737	-0.1027	+
[714]	MAPK1	--->	YWHAH	0.079981	-0.071802	-
[715]	PAX5	<---	WWOX	-0.20054	0.11492	-
[716]	CCL3	<---	CX3CR1	-0.35895	0.21658	-
[717]	CCND1	<---	FLI1	-0.25849	0.28986	+
[718]	EGR3	<---	SPINT1	0.0086698	0.045705	-
[719]	FOXA2	--->	ID1	0.81123	-0.8599	+
[720]	ACTR8	<---	YY1	-0.19307	0.29531	+
[721]	CD44	<---	LAMA2	-0.041536	-0.001298	+
[722]	PPARGC1A	<---	PRKAA1	-0.47902	1.2133	+
[723]	EPO	<---	IL3RA	-0.062099	-0.03581	-
[724]	CREB1	--->	MAX	0.11676	-0.084413	+
[725]	CCL24	<---	XCR1	-0.35895	0.21658	-
[726]	ADAM17	<---	PRKCD	-0.087066	0.033769	+
[727]	BIRC3	<---	RELA	-0.13423	0.00049143	+
[728]	CACNB1	<---	CAMK2G	-0.047052	0.14731	+
[729]	PSMD1	--->	SUFU	0.28298	-0.33691	+
[730]	PRKACB	--->	TRPV1	0.54889	-0.41958	+
[731]	CRKL	<---	EGFR	-0.26389	0.35602	+
[732]	CD80	<---	ITK	-0.044304	0.12709	-
[733]	MAPK9	<---	TGFB2	-0.0067309	0.13651	+
[734]	GNAT3	<---	RGS18	-1.2531	1.2063	+
[735]	IKZF3	<---	IL2RB	-0.075342	0.22454	+
[736]	CALM1	<---	ITPKC	-0.12569	0.087809	+
[737]	RHOQ	<---	TRIP10	0.074898	0.19853	+
[738]	MYO9B	<---	RHOV	-0.54674	0.38058	-
[739]	GSK3B	--->	TSC2	0.62858	-0.85611	+
[740]	POU4F1	--->	SP1	0.078944	0.049909	-
[741]	HCK	<---	PDGFRB	-0.15501	0.75705	+
[742]	CTNNB1	<---	FZD8	-0.12047	0.05338	+
[743]	IL1A	--->	IL1RAP	0.18502	-0.17739	+
[744]	MMP10	<---	PLG	-0.1166	-0.013996	+
[745]	AGTR1	--->	GNA11	1.1631	-1.1303	+
[746]	RHOG	<---	SOS1	-0.33772	0.48674	+
[747]	PLCG1	--->	TREM2	0.045786	-0.022856	-
[748]	NOTCH2	<---	PCSK5	-0.048737	0.058804	+
[749]	IGKV1-5	--->	PIK3CD	-0.0085211	-0.0095765	-
[750]	HIF1A	<---	PIK3CA	0.01233	0.01701	+
[751]	RALBP1	<---	RHOQ	-1.5245	1.016	-
[752]	IKBKB	<---	MAP4K4	-1.5263	1.1934	+
[753]	LYN	<---	PRKCA	-0.027179	0.23398	-
[754]	CCNB2	<---	SEC13	-0.33759	0.44625	-
[755]	CTNNA1	--->	MMP7	0.38719	-0.55042	-
[756]	CAMK2D	<---	PPP1CA	-0.015111	0.02592	+
[757]	SMAD4	<---	TGFBR1	-0.52231	0.72329	+
[758]	MAP3K4	<--->	MAPK10	0.31014	0.31014	-

```
[759]   CTBP2   --->    NEUROG3 0.21332 -0.33732        +
[760]   CREB3L3 <---    MAPK3   -0.57905        1.0858  +
[761]   BCL3    <---    HNRNPAB -0.058092       0.17511 -
[762]   CBL     --->    CNTFR   0.16204 -0.082304       +
[763]   HIPK2   <---    TAB1    -0.18043        0.60671 +
[764]   CCNA1   <---    E2F3    -0.39339        0.45542 -
[765]   CASP3   --->    CDH1    0.16402 -0.58792        +
[766]   CAMK2G  --->    EP300   0.010341        -0.008238       +
[767]   CHRM1   --->    GNAI2   1.1631  -1.1303 +
[768]   HDAC1   --->    SMO     0.06261 -0.024412       +
[769]   E2F6    --->    MGA     0.45267 -0.59164        +
[770]   BDNF    <---    EHD4    -0.31357        0.23297 +
[771]   CAMK2D  --->    IFNGR1  0.20978 -0.34095        -
[772]   FOXA2   --->    MTF2    0.27809 -0.34674        +
[773]   ARRB2   --->    AXIN1   0.0088597       -0.049113       +
[774]   MAPK3   --->    S1PR3   0.19743 -0.10024        -
[775]   CTBP2   --->    SATB2   0.04296 -0.16727        +
[776]   PDPK1   --->    PRKCQ   0.54086 -0.92024        -
[777]   SETDB1  --->    ZNF792  0.064599        -0.034791       +
[778]   ITGA10  --->    TNC     -0.015417       -0.048468       -
[779]   PPARG   --->    TGFB1   0.057992        -0.17749        -
[780]   F2RL2   --->    GNB1    0.69217 -0.57296        +
[781]   POTEKP  <---    RAC2    -0.51168        0.52339 +
[782]   CCL16   <---    CXCR5   -0.35895        0.21658 -
[783]   FGFR2   <---    HNF4A   -0.26109        0.079615        +
[784]   MSC     <---    TBP     -0.043597       0.00042063      +
[785]   IL12RB1 <---    IL3     -0.10994        -0.0285 +
[786]   PTK2    <---    PVRL1   -0.10899        0.030751        +
[787]   MAPK8   --->    STAT3   1.4162  -0.966  +
[788]   GNAT3   --->    PDE6A   0.45807 -0.32299        +
[789]   NCOR1   --->    RARB    -0.018841       -0.11403        -
[790]   COL4A1  <---    FYN     -0.45391        0.63648 -
[791]   ACVR1   <---    INHBE   0.095733        0.23422 +
[792]   PPP2CA  --->    WWTR1   0.78287 -0.58536        +
[793]   CCNT2   <---    NR1D2   -0.18783        0.044603        -
[794]   GNAI3   <---    RGS12   -0.022601       0.029368        +
[795]   CREB1   <---    PPARA   -0.13468        0.099807        -
[796]   BID     <---    MAPK8   -0.62086        0.60076 +
[797]   GNAQ    --->    WNT16   0.42087 -0.47341        -
[798]   FBXW7   --->    TP63    0.066076        -0.069483       +
[799]   CD3E    --->    PIK3R3  0.15984 -0.32529        +
[800]   FGFR1   <---    HGF     -0.27366        0.27202 +
[801]   DUSP12  --->    MAP2K4  1.4184  -1.4202 +
[802]   CDC20   <---    CUL1    -0.28296        0.25098 +
[803]   GNB1    <---    MAPK14  -0.28574        0.38931 -
[804]   PIK3CA  <---    PRKCB   -0.06189        0.049814        -
[805]   CXCL12  --->    STAT5B  0.42026 -0.3013 +
[806]   AXIN1   <---    TCF7    -0.7238 0.68508 +
[807]   LEP     --->    SOCS3   -0.011149       -0.30429        -
[808]   ADAM17  <---    PKN3    -0.047438       -0.0085337      +
[809]   E2F1    --->    MEF2C   0.097401        -0.26812        +
[810]   CBLC    --->    PIK3R2  0.33419 -0.25407        +
[811]   ANGPT4  --->    ARNT    0.0052685       -0.16068        -
[812]   CRKL    --->    PTK2    -0.072696       -0.30229        -
[813]   EIF3B   <---    RXRA    -0.19115        -0.11049        +
[814]   IL20RB  <---    LEP     -0.087402       0.20113 +
[815]   E2F4    --->    RNPS1   0.32902 -0.26332        +
[816]   FOS     --->    STAT5A  0.4178  -0.25647        -
[817]   BNIP3   <---    FOXO1   -0.37846        0.32839 +
[818]   BIRC3   --->    CASP1   0.072187        -0.29878        -
[819]   CTNND1  <---    PTPN11  -0.46977        0.49771 -
[820]   ARHGAP23        <---    RHOT1   -0.17007        0.096777        -
[821]   COL16A1 <---    PTK2    -0.11304        -0.10314        +
[822]   MPL     --->    STAT5A  0.1409  -0.099294       +
[823]   ITGA2B  <---    SPI1    -0.10803        -0.013036       +
[824]   CTNNB1  <---    WNT6    -0.187  0.023012        +
[825]   PRKCZ   --->    SOS2    0.088396        -0.13444        -
[826]   GNB2    <---    HTR1D   -0.92616        1.1575  +
[827]   MAP3K14 <--->   MAPK1   0.31014 0.31014 -
[828]   FASLG   --->    MAPK8   0.10494 0.048942        -
[829]   AAAS    <---    GCK     -0.060578       0.059668        -
[830]   ADCY3   <---    PRKACA  -0.63235        0.8673  -
[831]   CTCF    <---    PPP1R1B -0.094434       0.20666 -
[832]   ARL2    <---    TBCD    -0.47156        0.21207 -
[833]   SMAD4   --->    TGFB1   0.060804        -0.24379        +
[834]   PTPN6   --->    SOCS3   0.77689 -1.074  +
```

[835]	EBF1	--->	MAP2K3	0.21802	-0.072402	+
[836]	BATF	--->	KLF1	0.16224	0.14761	+
[837]	MYL12B	--->	MYL9	0.74066	-0.84911	+
[838]	MAPK1	--->	SHC1	0.70518	-0.81557	-
[839]	EGLN1	--->	EPAS1	0.87319	-0.89053	+
[840]	FOXP3	--->	RORC	0.076395	-0.21893	+
[841]	C4A	--->	NFIC	0.21087	-0.3538	-
[842]	EGF	<---	PRKCA	-0.12658	0.10812	+
[843]	ATP1B4	<---	PRKG2	-0.60022	0.54802	+
[844]	CSF1R	<---	NGF	-0.17101	0.35874	+
[845]	TNFRSF11A	<---	TRAF6	-0.085301	0.021316	-
[846]	NFKBIA	<---	PPARA	-0.23053	0.03163	+
[847]	PDE4A	<---	PRKACA	-0.95055	1.4803	+
[848]	CALM1	--->	RAB10	0.20073	-0.14858	-
[849]	DUSP12	--->	MAPK9	1.4184	-1.4202	+
[850]	PIK3CG	<---	PRKCZ	-0.014245	-0.0037836	-
[851]	DLL4	--->	MIB1	-0.0009879	-0.0037502	-
[852]	CCL3	--->	CTCF	0.043931	0.027226	-
[853]	HGF	<---	PTPRJ	-0.35709	0.24155	+
[854]	COL6A1	--->	ITGA1	-0.0037591	-0.061373	+
[855]	PLG	--->	TGFB1	0.097403	-0.23285	+
[856]	PRKCZ	--->	TIAM1	0.086046	-0.063619	+
[857]	NUP37	--->	SRSF1	0.31391	-0.17132	+
[858]	ALX4	<---	TCF12	-0.1101	0.05946	+
[859]	ARF6	<---	EXOC2	-0.08523	0.16139	-
[860]	CD247	--->	GNAI1	0.030816	-0.0080738	-
[861]	CAMK2A	--->	TRPV1	0.36975	-0.29127	+
[862]	BAX	<---	CAPN1	-0.20179	0.14315	-
[863]	FGF17	--->	KIT	0.015796	-0.016964	+
[864]	DLL4	--->	NOTCH4	-0.0032792	-0.10471	+
[865]	NCOR1	--->	SMARCD3	0.14243	-0.18931	+
[866]	FGD1	<---	RHOA	-0.14219	0.16153	-
[867]	CSNK1G1	--->	TP53	0.18812	-0.13106	+
[868]	CTBP2	<---	FLI1	-0.04934	-0.028582	-
[869]	ARHGAP35	<---	SRC	0.10251	0.62001	+
[870]	GNAI3	--->	PLA2G4F	-0.10668	-0.13723	+
[871]	ADCY9	<---	GNAI1	-0.63317	1.0384	-
[872]	MYO9B	<---	RHOH	-0.54674	0.38058	-
[873]	C1QA	<---	C4A	-0.19739	0.12796	-
[874]	GABBR1	<---	GNB4	-0.10866	0.15376	-
[875]	NCF2	--->	VCAM1	0.080684	-0.11847	-
[876]	MET	--->	SH3KBP1	0.034013	-0.10194	-
[877]	FZR1	<---	PSMD7	-0.26071	0.27742	+
[878]	BCL6	<---	CEBPB	-0.093323	0.16874	+
[879]	DAND5	--->	RGL3	0.48968	-0.3901	+
[880]	IKBKG	<---	MAP3K14	-0.45312	0.62218	+
[881]	PLCB1	<---	RGS4	-0.15809	0.12524	+
[882]	BCAR1	--->	DNAJC27	0.21814	-0.11421	+
[883]	LPAR1	--->	PIK3R5	0.56062	-0.77034	+
[884]	GABPA	--->	TBP	0.14957	-0.17847	+
[885]	COL27A1	--->	ITGA3	-0.020349	-0.077049	+
[886]	CSNK1D	--->	WNT7B	0.83912	-0.78694	-
[887]	CRKL	--->	RAC1	0.43868	-0.057401	+
[888]	GNB4	--->	WNT7B	0.3343	-0.42584	-
[889]	AKT2	--->	CREB5	0.82132	-1.3257	+
[890]	RALB	--->	RGL1	1.4276	-1.2366	-
[891]	CCL3	<---	CXCR1	-0.35895	0.21658	-
[892]	ERBB2	--->	MAPK8	0.086576	-0.027782	+
[893]	AXIN2	<---	PSMD4	-0.079161	0.01642	+
[894]	MAPK3	--->	SHC1	0.70518	-0.81557	-
[895]	CCL23	<---	CCR8	-0.35895	0.21658	-
[896]	SHC1	<---	VEGFC	-0.23434	0.14513	+
[897]	ITGA8	<---	LAMC2	-0.066621	-0.0090065	+
[898]	BMP4	--->	BMPR1A	0.27387	-0.33406	+
[899]	ATF2	<---	SMAD1	-0.080108	0.060865	+
[900]	ROCK2	--->	SHB	0.25656	-0.19456	-
[901]	IL12RB2	<---	SOCS5	-0.10625	0.079572	+
[902]	CCL17	<---	CXCR3	-0.35895	0.21658	-
[903]	PPP1CA	<---	TGFBR1	0.053734	0.1312	+
[904]	GLI1	<---	RPGRIP1L	-0.24841	0.32995	+
[905]	CAMK2B	--->	EP300	0.010341	-0.008238	+
[906]	BID	<---	TNFRSF1A	-0.48613	0.24746	+
[907]	IL11RA	<---	JAK1	-0.23205	0.12411	-
[908]	KRAS	--->	PIK3R5	0.51887	-0.55672	-
[909]	DVL2	<---	FZD5	-0.33521	0.24353	+
[910]	MAPK14	--->	NR3C1	0.29076	-0.30308	+

[911]	CAMK2B	--->	NOS3	0.066587	-0.050851	+
[912]	KCNQ5	--->	TRPV4	-0.088937	-0.17028	-
[913]	EP300	<---	FES	-0.017582	0.00096527	-
[914]	CTCFL	<---	EGR1	-0.039841	-0.037992	+
[915]	BIRC3	--->	SMPD3	0.020947	-0.083567	+
[916]	ITSN1	--->	RHOV	0.16745	-0.11028	+
[917]	BCAR1	<---	PTPN12	-0.051384	0.032818	+
[918]	CTNNB1	<---	WNT8A	-0.187	0.023012	+
[919]	IL2RG	<---	SOCS5	-0.10625	0.079572	+
[920]	FLT1	--->	PIK3CD	0.043149	-0.0082223	+
[921]	FCGR2A	--->	LAT	0.19986	-0.23925	+
[922]	ARHGAP19	<---	RAC2	-1.5245	1.016	-
[923]	FZD7	<---	WNT10A	-0.78209	0.63734	+
[924]	CCL28	<---	CCR9	-0.35895	0.21658	-
[925]	GHRL	--->	INS	0.23782	-0.29926	-
[926]	ARHGEF7	<---	FYN	-0.30715	0.22299	+
[927]	ELMO1	<---	RAC2	-0.038582	0.076038	-
[928]	ARHGAP35	<---	PTPN11	-0.26381	0.45826	+
[929]	ERBB4	--->	MYC	0.10642	-0.14183	+
[930]	IL20RA	<---	IL26	-0.087402	0.20113	+
[931]	KCNB1	--->	REST	0.1257	0.025162	-
[932]	CBL	--->	CD3G	0.27772	-0.14084	-
[933]	DSG1	<---	EP300	-0.015116	0.0030477	+
[934]	PLCB2	<---	S1PR1	-0.052724	0.014576	+
[935]	PLA2G4F	<---	PRKCA	-0.21202	0.18036	+
[936]	JUN	<---	KLF6	-0.025299	0.094536	-
[937]	HSP90AA1	--->	PRKACA	0.24936	-0.12983	-
[938]	FGF21	--->	MYC	-0.0071149	-0.17424	-
[939]	RIPK2	<---	UBE2V1	-0.25379	0.15676	+
[940]	CRLF2	<---	EPO	-0.17292	0.061531	+
[941]	MAPK3	--->	MAPT	0.40675	-0.26091	+
[942]	SKP1	--->	SMAD2	-0.031512	-0.063591	+
[943]	COL4A3BP	<---	CSNK1G2	-0.26066	0.70565	+
[944]	PIM2	--->	STAT5B	1.4162	-0.966	-
[945]	PLD1	<---	SYK	-0.3078	0.073307	+
[946]	FZD3	<---	WNT7A	-0.78209	0.63734	-
[947]	F12	<---	KLKB1	-0.055978	0.034355	-
[948]	CSF1R	<---	FGF21	-0.64493	0.77791	+
[949]	MMP9	--->	MYC	-0.014865	-0.16766	-
[950]	CAMK2B	--->	FLNA	0.30815	-0.21817	+
[951]	BCAR1	--->	PRKCD	0.0033496	-0.01579	-
[952]	MAPK13	<---	RIPK2	-0.29078	0.20201	+
[953]	GNA11	<---	PRKACA	-0.22953	0.50225	+
[954]	DAND5	--->	TIMP2	0.036704	-0.072158	+
[955]	CHUK	<---	SQSTM1	-0.29176	0.0081481	+
[956]	LFNG	--->	NOTCH3	0.28627	-0.21238	+
[957]	CCNB2	<---	PLK1	-0.071915	0.2707	-
[958]	CREB3L1	<---	CREBBP	-0.010885	0.015595	-
[959]	COL5A1	--->	ITGA3	-0.020349	-0.077049	+
[960]	CTCF	--->	GFI1B	-0.0033695	-0.0087103	+
[961]	CAMK2B	--->	PDE3A	0.95928	-0.63646	+
[962]	FGF13	<---	MET	-0.13536	0.11923	-
[963]	DDIT4	--->	TSC1	0.4913	-0.57333	+
[964]	ERBB2	<---	IGF1	-0.010237	0.15911	+
[965]	CHUK	<---	FAS	-0.16508	0.036616	+
[966]	PPP2CA	--->	YWHAB	0.18205	-0.13344	-
[967]	GNB2	--->	WNT9B	0.3343	-0.42584	-
[968]	PIK3CD	--->	SHC1	0.013662	-0.030241	+
[969]	HTT	--->	RHOQ	0.21591	-0.31065	-
[970]	FOS	<---	IL3	0.019757	0.1058	-
[971]	COL4A1	--->	ITGA9	-0.013036	-0.10803	+
[972]	CTBP2	--->	SIX3	0.04296	-0.16727	+
[973]	RBL1	<---	TFDP2	-0.13587	0.072342	-
[974]	JAK3	--->	SGMS1	0.087709	-0.22013	+
[975]	RAPGEF1	--->	VLDLR	0.13612	-0.10139	-
[976]	FGFR1	<---	PDGFA	-0.37298	0.33909	+
[977]	CLEC4M	--->	TGFB2	0.17799	-0.37709	+
[978]	INHBE	--->	TGFB1	-0.050062	-0.38124	+
[979]	FGF14	--->	FLT1	0.21774	-0.0015767	+
[980]	CXCR3	--->	MEF2A	0.21915	-0.42605	-
[981]	GNAT2	<---	OPN1LW	-1.1303	1.1631	+
[982]	PRLR	--->	SOCS2	-0.098904	-0.12483	-
[983]	HDAC8	<---	PRKACA	-0.52917	0.82707	+
[984]	MAP2K3	--->	MAPK3	1.1648	-0.18663	+
[985]	STAM	<---	TYK2	-0.043241	-0.042015	-
[986]	LRP5	<---	WNT7B	-0.28769	0.39899	+

[987]	MAPK12	--->	NCF1	0.056871	0.030415	-
[988]	RHOA	<---	SHC1	-0.13281	0.31787	-
[989]	CDC20	<---	PSMD4	-0.098063	0.10019	+
[990]	APBB1IP	--->	STAT3	0.3173	-0.39904	-
[991]	NFATC4	--->	PPP3R1	-0.039001	-0.22539	-
[992]	CASP10	--->	PTPN11	0.028467	-0.21537	-
[993]	ITGB3	--->	LAMA2	-0.0055713	-0.0064655	-
[994]	CTCF	<---	LILRB1	0.0095251	0.054431	-
[995]	LRP5	<---	SOST	-0.54894	0.76391	+
[996]	POU2F1	<---	PPARG	-0.15251	0.047328	+
[997]	IL26	--->	PRLR	0.073187	-0.17721	+
[998]	RHOBTB2	--->	STARD13	0.061607	-0.30675	-
[999]	IL1B	--->	NLRC4	0.17679	-0.19911	-
[1000]	CSNK1G3	--->	WNT1	0.50785	-0.4784	-
[1001]	ITGB3	--->	THBS3	0.01856	-0.023154	-
[1002]	STAT1	--->	TIMP1	0.2569	-0.31397	+
[1003]	NCOA2	<---	SP1	-0.017564	0.042925	-
[1004]	COL12A1	<---	LEPRE1	-0.16824	0.086087	+
[1005]	PIK3R1	<---	TRAF5	-0.25609	0.28661	+
[1006]	ARPC1A	--->	MYO10	0.017976	-0.019996	-
[1007]	GAB1	<---	PIK3R1	-0.21066	-0.044795	-
[1008]	CRLF2	<---	IL20	-0.18506	0.094523	+
[1009]	ADRA1D	<---	GRK4	-0.28786	0.33635	+
[1010]	CDC42	--->	MYO9A	0.38058	-0.54674	-
[1011]	MLST8	<---	SGK1	-0.2505	0.14791	-
[1012]	CBLC	--->	CRLF2	0.30834	-0.14884	+
[1013]	LRP6	<---	WNT10B	-0.28769	0.39899	+
[1014]	RICTOR	--->	TSC1	0.13022	-0.12111	-
[1015]	PRKCA	--->	SHC3	0.468	-0.43947	-
[1016]	INSR	<---	PIK3CG	-0.0060038	0.056262	-
[1017]	FZD2	<---	WNT3	-0.78209	0.63734	+
[1018]	RGS1	<---	YES1	-0.35367	0.53308	+
[1019]	GNA15	--->	WNT16	0.42087	-0.47341	-
[1020]	MAPK13	<---	STAT1	-1.2527	1.4568	-
[1021]	NFKB1	--->	TYK2	0.34808	-0.35909	-
[1022]	KLRC2	<---	LCK	-0.45391	0.63648	+
[1023]	E2F1	--->	RB1	0.13728	-0.24843	-
[1024]	MYH14	<---	RHOC	-0.01596	0.026206	+
[1025]	MRAS	--->	PIK3R2	0.22452	-0.33729	+
[1026]	FOS	<--->	MSR1	0.22155	0.22155	-
[1027]	AAAS	--->	SRSF1	0.31391	-0.17132	+
[1028]	CCNB1	<---	RAB1A	-0.42176	0.55184	-
[1029]	MAPK9	--->	NFATC3	0.92767	-0.99472	+
[1030]	CCL5	--->	EGR1	0.17326	-0.053263	-
[1031]	ADCY8	<---	EDNRA	-0.62364	0.57833	+
[1032]	GP1BB	--->	PIK3CA	0.022421	-0.053455	+
[1033]	FYN	--->	NGEF	0.49172	-0.08758	+
[1034]	CD14	<---	RIPK1	-0.0049718	0.0098361	-
[1035]	RNF41	--->	USP8	0.27956	-0.31865	-
[1036]	JAK1	--->	PRLR	0.10182	-0.23677	-
[1037]	F2RL3	--->	PRSS2	0.11779	-0.2522	-
[1038]	CREB1	<---	PCDHB11	-0.064967	0.092502	-
[1039]	PTPN11	--->	SOCS3	0.77689	-1.074	-
[1040]	GRIN2B	<---	PPP1CB	-0.67094	0.67498	+
[1041]	DKK1	--->	WNT11	0.54164	-0.69755	+
[1042]	FOXO1	<---	SGK2	-0.40959	0.15123	+
[1043]	NFKB1	--->	PDGFB	0.13867	-0.14039	+
[1044]	PSMD1	<---	SKP2	-0.13813	-0.076225	-
[1045]	IL13RA1	<---	JAK1	-0.23741	0.19744	-
[1046]	GARNL3	--->	RAP1B	0.57378	-0.48764	+
[1047]	MAPK1	<---	TNFRSF10A	-0.22533	-0.1429	+
[1048]	GABBR2	<---	GNAI1	-0.09586	0.14467	-
[1049]	APBB2	--->	PSEN1	0.093374	-0.20238	-
[1050]	ACACB	<---	PRKAA1	-0.374	0.41081	+
[1051]	ADRBK2	--->	CXCR6	0.25327	-0.21301	+
[1052]	HDAC2	--->	SMO	0.06261	-0.024412	+
[1053]	IL18RAP	--->	MAPK9	0.25091	-0.10136	+
[1054]	FXYD2	<---	MAPK3	-0.26091	0.40675	+
[1055]	EP300	<---	PRKACG	-0.006941	0.013728	+
[1056]	FLT1	--->	PIK3CA	0.043149	-0.0082223	+
[1057]	PPARD	<---	RELA	-0.27124	-0.010205	-
[1058]	CCR4	--->	CXCL1	0.21658	-0.35895	-
[1059]	GNAI3	<---	HCK	-0.44236	0.67161	-
[1060]	NFKB1	--->	NR4A2	0.1148	-0.14103	+
[1061]	KRAS	--->	PLCE1	0.086381	-0.093653	+
[1062]	FGF10	<---	SOS1	0.067334	0.085583	-

[1063]	SMARCD1	--->	SP2	0.29622	-0.181	-
[1064]	IFNGR2	<---	PTPN11	-0.75018	0.80704	+
[1065]	EZH2	--->	H3F3A	0.3865	-0.4548	+
[1066]	RBBP7	--->	SMO	0.19185	-0.19257	+
[1067]	CRKL	<---	PTPN11	-0.4534	0.31387	+
[1068]	GLP1R	--->	SP3	0.16874	-0.093323	-
[1069]	RACGAP1	<---	RHOT1	-0.40786	0.2043	-
[1070]	DBNL	--->	MAP3K1	0.067194	0.033228	+
[1071]	IL3RA	--->	STAT5A	0.44302	-0.302	+
[1072]	CXCL1	<---	MAPK13	-0.36767	0.50554	+
[1073]	FZD6	<---	WNT7A	-0.30406	0.25758	+
[1074]	CTCF	--->	HOXC6	0.065182	0.0092332	+
[1075]	HNF1A	<---	NLK	-0.46198	0.60871	+
[1076]	ITGA10	<---	LAMA2	-0.025761	-0.0054603	+
[1077]	BRCA1	--->	UHRF2	0.10587	-0.1182	+
[1078]	FCGR2A	--->	IGKV1-5	0.28143	-0.28221	-
[1079]	ABL1	<---	FGR	-1.0203	0.50083	+
[1080]	KCNJ9	<---	SSTR4	-0.2522	0.11779	-
[1081]	GNAI1	--->	PIK3R3	0.19122	-0.22979	+
[1082]	BRD2	--->	ZBTB7A	0.16874	-0.093323	-
[1083]	IL12RB1	<---	RELB	-0.71218	0.35097	-
[1084]	SLC2A4	<---	YWHAB	-0.85287	0.64882	+
[1085]	ITGA2	<---	SRC	-0.16015	0.14089	-
[1086]	MAPK3	--->	PLA2G4D	0.18871	-0.84715	+
[1087]	MYLK3	<--->	PRKX	0.31014	0.31014	-
[1088]	BCAR1	--->	RAC1	0.21814	-0.11421	-
[1089]	SMAD2	--->	TNC	0.0033454	-0.089303	+
[1090]	COL27A1	--->	ITGA10	-0.013036	-0.10803	+
[1091]	GATA3	--->	STAT3	0.44408	-0.32334	-
[1092]	MAPKAPK3	--->	PHC2	0.40675	-0.26091	+
[1093]	SMAD3	--->	TGFB1	0.060804	-0.24379	-
[1094]	EP300	--->	SERPINB7	0.019422	-0.029524	+
[1095]	FYN	--->	PIK3R2	0.26317	-0.26628	+
[1096]	EGFR	--->	PDGFC	0.23053	-0.20539	-
[1097]	APBB1	--->	PSEN2	0.093374	-0.20238	-
[1098]	LRP6	<---	WNT4	-0.28769	0.39899	+
[1099]	ARHGEF11	--->	RHOB	0.092701	0.0073174	+
[1100]	AKT1	--->	HIF1A	0.090304	-0.029611	+
[1101]	E2F1	<---	NR1D2	-0.17309	0.015585	-
[1102]	CHUK	<---	TLR3	-0.19289	0.066908	+
[1103]	NCK1	--->	PAK3	0.73462	-0.72841	+
[1104]	MAPK11	<--->	PRKACB	0.31014	0.31014	-
[1105]	PRKCB	--->	PTPN11	0.55493	-0.48581	-
[1106]	PIK3C3	<---	PTK2B	-0.045972	-0.036571	+
[1107]	ADRB1	<---	GRK6	-0.28786	0.33635	+
[1108]	BCL3	<---	ZNF280B	-0.04047	0.37639	-
[1109]	FGF7	<---	KRAS	-0.25726	0.38422	-
[1110]	EP300	<---	PRMT1	-0.022365	0.018573	-
[1111]	PIK3CD	<---	PRKD1	-0.039504	0.050936	-
[1112]	HDAC2	<---	IL1RN	-0.3602	0.42207	-
[1113]	AKT3	--->	RPS6KB2	0.67325	-0.79394	+
[1114]	ABL1	--->	MDM2	-0.0046292	-0.045135	+
[1115]	HOXC8	<---	TNFRSF11B	-0.46013	0.17714	-
[1116]	RANBP2	--->	SRSF4	0.34767	-0.31854	+
[1117]	MAP2K6	<--->	MAPK12	0.31014	0.31014	-
[1118]	DVL1	<---	WNT2	-0.54867	0.38842	+
[1119]	FGF9	--->	PDGFRB	0.48772	-0.57252	+
[1120]	NUP37	--->	SRSF11	0.31391	-0.17132	+
[1121]	CBLC	--->	JAK2	1.1229	-1.0039	+
[1122]	FLT4	--->	PIK3CB	0.0062554	-0.016711	+
[1123]	MAP2K1	--->	NTN1	0.044586	-0.0036971	-
[1124]	NRAS	--->	RPS6KA3	0.39663	-0.23906	-
[1125]	IL4R	<---	JAK2	-0.23677	0.10182	-
[1126]	PELI2	<---	UBE2N	-0.27351	0.12593	-
[1127]	RAP1A	<---	RAPGEF4	-0.62373	0.78832	+
[1128]	MAPK8	<---	RHOA	-0.20304	1.2491	-
[1129]	FOXO1	<---	PRKAA1	-0.22085	0.2499	+
[1130]	ARHGAP26	<---	RHOB	-0.65972	0.48549	-
[1131]	IL20RB	<---	THPO	-0.075265	0.16814	+
[1132]	GRB7	<---	PTPRB	-0.31265	0.30993	+
[1133]	GNGT2	--->	PIK3CB	-0.0073408	-0.012795	+
[1134]	CBLB	--->	LIFR	0.11039	-0.066545	+
[1135]	AKT2	--->	NFKBIB	0.28123	-0.28841	+
[1136]	HOMER2	--->	PIK3R2	-0.042301	-0.056077	+
[1137]	COL27A1	--->	PPIB	-0.024575	-0.068802	-
[1138]	INSR	<---	RPS6KB1	0.030601	0.14209	+

[1139]	CNTF	--->	IL6ST	0.056231	-0.11334	+
[1140]	MYD88	--->	UBE2V1	0.21853	-0.27431	+
[1141]	CREB1	<---	IL5RA	0.0246	0.032723	-
[1142]	ADORA3	--->	GNAI2	0.027216	-0.0021531	+
[1143]	SKI	--->	SMAD2	-0.031512	-0.063591	+
[1144]	CCL16	<---	CCR3	-0.35895	0.21658	-
[1145]	CHRM5	--->	PRKCZ	-0.029433	-0.05892	+
[1146]	PBX3	--->	ZNF281	0.046782	0.029172	+
[1147]	PIK3R1	<---	SHANK3	-0.0030957	0.0046229	+
[1148]	NFATC4	--->	TRPC6	0.21608	-0.35616	+
[1149]	HRAS	--->	ITGB3	0.014795	-0.01078	-
[1150]	ADAM10	--->	CDH1	0.044284	-0.17263	+
[1151]	COL4A5	--->	ITGA3	-0.020349	-0.077049	+
[1152]	HDAC3	<---	TCF7L2	-0.12264	0.37175	-
[1153]	CCL14	--->	HDAC2	0.27068	-0.21235	-
[1154]	PIK3CD	<---	RAC1	-0.01026	0.011011	-
[1155]	GNAI3	--->	GPSM1	-0.0080287	-0.042052	-
[1156]	ARHGAP12	<---	RHOD	-0.36596	0.22724	-
[1157]	FZD10	<---	GNAQ	-0.22143	0.24435	-
[1158]	CCNB2	<---	RAE1	-0.33759	0.44625	-
[1159]	BHLHE40	<---	MEF2A	-0.26031	0.094795	+
[1160]	PPP2R1A	--->	PRKCI	0.2396	-0.27354	+
[1161]	CRK	<---	KDR	-0.25443	0.68453	+
[1162]	CCL20	<---	RELA	-0.69538	0.38373	+
[1163]	CCL5	<---	CCR3	-0.35895	0.21658	-
[1164]	CCL19	<---	CXCR2	-0.35895	0.21658	-
[1165]	CBLB	--->	CNTFR	0.16204	-0.082304	+
[1166]	LRP5	<---	WNT2	-0.28769	0.39899	+
[1167]	RARA	--->	TMSB10	-0.015388	-0.14879	+
[1168]	FZD8	<---	WNT9B	-0.78209	0.63734	+
[1169]	CHD2	--->	KLF1	0.12224	-0.14589	+
[1170]	ARHGEF17	<---	RAC3	-0.012974	0.047696	-
[1171]	ACTN4	<---	CRKL	-0.012872	0.00051804	-
[1172]	ARR3	--->	FZD2	0.13556	-0.17053	+
[1173]	CDC42	--->	LAMC1	0.026206	-0.01596	-
[1174]	COL15A1	<---	PTK2	-0.11304	-0.10314	+
[1175]	CXCL1	<---	REL	-0.69538	0.38373	+
[1176]	CCL26	<---	CXCR3	-0.35895	0.21658	-
[1177]	EP300	--->	PRUNE2	0.00067605	-0.0050208	+
[1178]	ARAP1	<---	RHOD	-0.48615	0.36733	-
[1179]	IL4R	--->	SOCS3	-0.098904	-0.12483	-
[1180]	BTRC	--->	SMAD3	0.20235	-0.14554	-
[1181]	PIK3R3	--->	RAC1	0.30072	-0.072693	-
[1182]	CAMK2G	--->	PDE4C	0.95928	-0.63646	+
[1183]	GNAI2	--->	PIK3R1	0.060838	-0.089976	+
[1184]	GDF1	--->	LEFTY2	-0.050062	-0.38124	-
[1185]	CCNE2	<---	E2F2	-0.39339	0.45542	+
[1186]	GNAI2	<---	PLCG1	-0.0052535	0.030885	-
[1187]	EP300	<---	PIAS4	-0.0030573	7.1807e-005	-
[1188]	GRK4	--->	HTR5A	0.33635	-0.28786	+
[1189]	INSR	--->	SOCS3	0.25546	-0.083828	-
[1190]	ACADM	--->	RXRA	-0.091263	-0.26204	-
[1191]	SERPINE1	<---	SMAD4	-0.34866	0.12534	+
[1192]	CCND1	<---	PSMD1	-0.40564	0.45419	+
[1193]	SMAD6	<---	TAB2	-0.29305	0.22564	-
[1194]	RACGAP1	<---	RHOH	-1.011	0.68428	-
[1195]	FGF18	<---	HRAS	-0.15714	0.23194	-
[1196]	MCF2	--->	RHOT1	0.037885	-0.030277	+
[1197]	GNAI1	<--->	GNAQ	0.064764	0.064764	-
[1198]	ADRB1	--->	GNAL	1.1631	-1.1303	+
[1199]	LYN	--->	STAT1	0.53614	-0.81843	+
[1200]	BIRC5	<---	E2F4	-0.15842	-0.028308	+
[1201]	GLI3	<---	RAB23	-0.09407	0.30452	+
[1202]	ONECUT3	--->	ZNF263	0.11044	0.025818	-
[1203]	GAB3	<---	PRKCI	-0.15343	0.32961	-
[1204]	CER1	--->	FZD2	0.27359	-0.29711	+
[1205]	GRK5	--->	HTR2B	0.33635	-0.28786	+
[1206]	FOS	<---	SMAD4	-0.12158	0.078724	-
[1207]	PRICKLE1	--->	PSMD1	0.50477	-0.47264	-
[1208]	ADCY4	<---	EDN1	-0.41432	0.41767	+
[1209]	GNB4	<---	PIK3CB	-0.012551	0.008521	-
[1210]	FLT1	--->	PTK2	0.28509	-0.20446	+
[1211]	MAP4K4	<---	TRAF3	-0.56477	0.90152	+
[1212]	CCNK	<---	TBP	-0.032749	0.069947	+
[1213]	CTNNB1	<---	PCDHA4	-0.19204	0.11503	-
[1214]	GLI2	--->	WNT8B	0.86373	-1.47	+

[1215]	CNTFR	<---	PTPN6	-0.057663	0.065784	+
[1216]	ATXN3	--->	MAX	0.26575	-0.31912	-
[1217]	MYH9	--->	ROCK2	0.042533	-0.040702	-
[1218]	HSP90AA1	--->	RAC1	0.21721	-0.19053	+
[1219]	AKT3	--->	SOS2	0.052556	-0.15964	-
[1220]	FZD4	<---	WNT10B	-0.78209	0.63734	+
[1221]	BAMBI	--->	FZD10	0.43771	-0.51806	+
[1222]	IL10RB	<---	IL19	-0.087402	0.20113	+
[1223]	CDKN1A	<---	SMAD4	-0.055585	0.0092434	+
[1224]	ARHGAP20	<---	RHOB	-1.4209	0.96682	-
[1225]	PIK3R3	<---	TRAF3	-0.68526	0.79666	+
[1226]	CALML6	--->	CAMKK1	1.2051	-1.1603	+
[1227]	RAE1	--->	RNPS1	0.31391	-0.17132	+
[1228]	NGEF	<---	SRC	-0.08758	0.49172	+
[1229]	PLD1	<---	PPAP2B	-0.019741	0.020601	-
[1230]	CREBBP	<---	RELA	-0.005516	0.0083594	-
[1231]	IRAK4	--->	PPP2CB	0.24582	-0.27237	-
[1232]	CSF1	--->	INSR	0.31112	-0.055094	+
[1233]	PPP2R1B	--->	STAT2	0.35788	-0.28878	+
[1234]	SLC2A4	<---	YWHAZ	-0.85287	0.64882	+
[1235]	KPNA5	<---	NUP153	-0.33633	0.24182	+
[1236]	DAXX	<---	PTPN11	-0.34883	0.32068	+
[1237]	NPAS2	<---	RORB	-0.14396	0.10125	-
[1238]	RAC1	<--->	RHOB	0.1628	0.1628	-
[1239]	PLXNA4	<---	RRAS	-0.037168	0.1815	-
[1240]	CALML3	--->	CAMK2G	0.19292	-0.081704	+
[1241]	FOXO3	--->	SIRT1	0.32839	-0.37846	-
[1242]	ADAMTSL4	<---	B3GALTL	-0.071046	-0.011484	+
[1243]	CER1	--->	WNT5A	0.60582	-0.57968	+
[1244]	AR	<---	FGF13	-0.066708	0.038491	-
[1245]	LAMA4	--->	SV2A	0.45971	-0.5261	+
[1246]	GRB2	<---	PTPRB	-0.46395	0.25893	+
[1247]	CCR10	--->	CXCL9	0.21658	-0.35895	-
[1248]	HIST2H3A	<---	MAPK14	-0.92817	0.922	+
[1249]	PIAS4	--->	STAT3	1.2392	-0.72187	+
[1250]	NOTCH4	--->	RBPJ	0.10174	-0.075473	+
[1251]	ERCC6	<---	UBTF	-0.058454	0.033732	-
[1252]	EP300	<---	TP63	-0.0040742	-0.0025658	-
[1253]	EGF	--->	INSRR	0.1552	-0.058377	+
[1254]	RHOBTB1	--->	TRIO	0.069967	0.0048318	-
[1255]	CHD7	--->	E2F1	0.082463	-0.29449	-
[1256]	IL12RB1	<---	STAT1	-0.34746	0.27928	-
[1257]	RAC2	--->	TAGAP	1.016	-1.5245	-
[1258]	CCL5	<---	CCR2	-0.35895	0.21658	-
[1259]	HRAS	<--->	NRAS	0.019616	0.019616	-
[1260]	CSRP1	<---	NFKB1	-0.091069	0.10092	+
[1261]	SRCAP	--->	TBP	0.13048	-0.17793	-
[1262]	HCK	--->	STAT3	0.43807	-0.74539	+
[1263]	PRKACA	--->	RASGRF2	0.38956	-0.93223	+
[1264]	PAX5	--->	REST	0.027275	0.016594	-
[1265]	BRAF	--->	MAPK9	0.99795	-1.3266	+
[1266]	BIRC2	--->	ELK1	0.020947	-0.083567	-
[1267]	FSHR	--->	USF2	0.33842	-0.47683	-
[1268]	CDK2	<--->	PKMYT1	0.31014	0.31014	-
[1269]	CCR2	--->	CXCL9	0.21658	-0.35895	-
[1270]	CSNK1G2	--->	YWHAB	0.047412	-0.04465	+
[1271]	IL12RB1	<---	IL23A	-0.29036	0.12248	+
[1272]	CSNK1G3	--->	GLI1	0.85273	-0.60299	+
[1273]	ARHGAP12	<---	RHOBTB2	-0.22307	0.13673	-
[1274]	PPM1B	--->	UBE2L6	0.45602	-0.68882	-
[1275]	CAMK2A	--->	FLNC	0.30815	-0.21817	+
[1276]	FGF8	--->	FLT1	0.21774	-0.0015767	+
[1277]	MAP2K4	<---	PAK1	-1.2674	1.5523	+
[1278]	ADCY8	<---	GNAZ	-0.63317	1.0384	-
[1279]	ACTC1	<---	MMP21	-0.29263	0.085904	+
[1280]	DKK2	--->	WNT3A	0.54164	-0.69755	+
[1281]	FOSB	--->	GRIA2	0.3989	-0.21023	+
[1282]	CCNT2	<---	VEGFA	-0.20827	0.17502	-
[1283]	SNAI2	--->	TP53	0.062203	0.013051	-
[1284]	ETS2	<---	MYB	-0.048028	0.036068	+
[1285]	CDK1	--->	CENPK	0.45546	-0.3575	+
[1286]	KLRC2	--->	VAV3	0.06651	-0.018805	+
[1287]	IGF1	<---	MAPK3	-0.15648	0.31976	-
[1288]	FZD9	<---	WNT6	-0.78209	0.63734	+
[1289]	MAPK1	--->	PGR	0.29076	-0.30308	-
[1290]	MIA2	--->	STAT3	0.46155	-0.34323	-

```
[1291]  MAPK13  <--->   MAPK8   0.31014 0.31014 -
[1292]  ATP1B3  <---    PRKACB  -0.26091        0.40675 +
[1293]  FGF18   <---    FRS2    -0.28504        0.43716 -
[1294]  GNB2L1  <---    PRKD1   -0.21398        0.36694 -
[1295]  GHRL    --->    USF2    0.58343 -0.6546 -
[1296]  HLX     --->    TCF12   0.23888 -0.32236        -
[1297]  ACTB    <---    PRKCA   -0.43362        0.33453 +
[1298]  SRC     --->    TRPV6   0.44853 -0.34759        +
[1299]  F2RL3   --->    GNA11   1.1631  -1.1303 +
[1300]  GRB2    --->    HGS     0.026143        -0.03841        -
[1301]  PDGFRB  <---    PTPN1   -0.47934        0.3788  +
[1302]  CASP9   <---    XIAP    -0.29703        0.11242 -
[1303]  LAMB3   --->    MMP19   -0.0045452      -0.11357        -
[1304]  KCNJ3   <---    PRKACA  -0.26091        0.40675 +
[1305]  BCAR1   --->    ITGAV   0.070773        -0.089729       -
[1306]  SMAD2   --->    TGFB1   0.060804        -0.24379        -
[1307]  ETS1    --->    FOSL1   0.15465 -0.098546       +
[1308]  GRAP2   --->    KRAS    0.63892 0.0030067       +
[1309]  STAT5B  <---    TYK2    -0.77344        0.58294 +
[1310]  BCL2L1  <---    PRKAR2B -1.0163 0.7413  +
[1311]  MYH8    <---    TNNC2   -0.032012       0.012494        -
[1312]  CD4     <---    GNAO1   -0.052939       0.061543        +
[1313]  MAPK1   <---    PRKCG   -0.092669       0.0037501       +
[1314]  ARHGAP35        <---    RHOC    -0.12797        1.0383  -
[1315]  DVL1    <---    WNT5B   -0.54867        0.38842 +
[1316]  ITGAV   <---    PIK3CA  -0.015882       -0.0051451      -
[1317]  BCL3    <---    KRT1    -0.12621        0.26835 -
[1318]  TCF7L2  --->    ZNF775  0.081795        -0.0087537      +
[1319]  EGR2    --->    MEF2A   0.055487        -0.10325        -
[1320]  PPP2R2A <---    SFN     -0.1213 -0.088103       -
[1321]  EPB41   <---    TCF7L2  -0.015736       0.051767        +
[1322]  CD80    <---    EP300   -0.005167       0.000286        +
[1323]  FGFR4   <---    VEGFA   -0.36751        0.24978 +
[1324]  CXCR3   --->    MEF2C   0.21915 -0.42605        -
[1325]  GNB5    <---    HTR1D   -0.92616        1.1575  +
[1326]  CSH1    --->    IFNAR1  0.20113 -0.087402       +
[1327]  FGF5    --->    FGFR3   0.071829        -0.16555        +
[1328]  FGB     <---    SRC     -0.34123        0.43967 -
[1329]  ATP1B1  --->    PIK3R3  0.15984 -0.32529        -
[1330]  CFTR    <---    CREB1   -0.11204        0.076021        +
[1331]  ACTB    <---    MMP19   -0.29263        0.085904        +
[1332]  CACNA2D1        <---    PPP2CA  -0.13479        0.16467 +
[1333]  ATF4    <---    EP300   -0.010885       0.015595        -
[1334]  IL10RA  <---    PRL     -0.087402       0.20113 +
[1335]  AURKB   --->    CENPA   0.922   -0.92817        -
[1336]  HDAC2   <---    KAT2A   -0.11623        0.13709 -
[1337]  LTBP1   --->    TGFB2   1.0128  -1.5309 +
[1338]  HGF     <---    SH3GL2  -0.056004       0.011081        +
[1339]  CCR2    --->    XCL1    0.21658 -0.35895        -
[1340]  EPHB3   --->    MMP9    -0.015392       -0.077231       -
[1341]  PLK1    <---    PSMD4   -0.058706       0.2805  +
[1342]  CRLF2   <---    SOCS2   -0.10066        -0.08965        +
[1343]  GNAI3   <---    OPRD1   -1.1303 1.1631  +
[1344]  AR      <---    HDAC7   -0.075457       0.043796        -
[1345]  PPP2CA  --->    STAT2   0.28302 -0.1728 +
[1346]  JAK3    --->    PIK3CD  0.011356        -0.033797       +
[1347]  DKK4    --->    FZD1    0.3176  -0.35737        +
[1348]  CCNB2   <---    NUP37   -0.33759        0.44625 -
[1349]  ARHGAP26        <---    RHOA    -0.65972        0.48549 -
[1350]  IRS1    <---    TEK     -0.14126        0.19864 +
[1351]  CBL     --->    HCK     0.10935 -0.17509        -
[1352]  FGFR3   <---    PDGFB   -0.047197       0.01764 +
[1353]  HIST3H3 <---    TCF7L2  -0.12986        0.27218 +
[1354]  COL19A1 <---    PTK2    -0.11304        -0.10314        +
[1355]  NR4A1   <---    TCF7L2  -0.983  0.9955  +
[1356]  IGFALS  --->    IGFBP3  0.16947 -0.36538        -
[1357]  EGF     <---    LRIG1   -0.050399       0.013684        +
[1358]  BCR     --->    STAT5B  0.092684        -0.1407 +
[1359]  MYL4    <---    TPM4    -0.091663       0.035745        -
[1360]  FYN     --->    SPTBN5  0.30577 -0.43809        +
[1361]  PPP2CA  --->    RB1     0.17848 -0.20801        +
[1362]  MAPKAP1 <---    SGK1    -0.22729        0.094208        -
[1363]  PITX2   <---    SMAD2   -1.5172 1.4362  +
[1364]  BCAR1   --->    ITGA5   0.070773        -0.089729       -
[1365]  MRC1    --->    TGFB1   0.17799 -0.37709        +
[1366]  CCR2    --->    CEBPA   0.11779 -0.2522 -
```

[1367]	ARHGAP29	<---	RHOU	-1.011	0.68428	-
[1368]	DVL1	--->	NOTCH4	0.16335	-0.1956	+
[1369]	FGF9	<---	MET	-0.26028	0.20868	-
[1370]	ACTR2	<---	MMP12	-0.29263	0.085904	+
[1371]	NTRK1	<---	PIK3CA	-0.059227	0.015072	-
[1372]	ADRBK2	--->	CCR1	0.25327	-0.21301	+
[1373]	IRS1	<---	KIT	-0.089607	0.61204	+
[1374]	CAPN2	<---	MAPK3	-0.20981	0.22699	+
[1375]	MAPK11	--->	NFATC2	0.92767	-0.99472	+
[1376]	IGKV1-5	<---	YES1	-0.47931	0.63774	+
[1377]	ARHGAP44	<---	RHOT1	-0.35613	0.16234	-
[1378]	PF4V1	--->	PROC	0.12832	-0.15711	+
[1379]	DKK2	--->	FZD1	0.3176	-0.35737	+
[1380]	RB1	<---	TFDP1	-0.13587	0.072342	-
[1381]	MYD88	--->	TAB3	0.021913	-0.048346	+
[1382]	PGK1	--->	RXRA	-0.091263	-0.26204	-
[1383]	HOXB6	--->	TCF12	0.23888	-0.32236	-
[1384]	NCAM1	<---	SRC	-0.4124	0.36715	+
[1385]	GLI2	--->	WNT7B	0.86373	-1.47	+
[1386]	DAND5	<---	TNFRSF10B	-0.2899	0.036892	-
[1387]	GNAI1	<---	PIK3CG	0.00043444	0.0052476	-
[1388]	CEBPB	--->	HOXA4	0.15908	-0.24396	+
[1389]	ARHGAP36	<---	RHOBTB2	-0.92575	0.61511	-
[1390]	KAT2A	--->	SMAD4	-0.020213	-0.039669	+
[1391]	ARF6	<---	LHCGR	-0.34211	0.034722	+
[1392]	MAP2K3	<---	RIPK2	-0.21145	0.1049	+
[1393]	AP2S1	<---	RPS6KA4	-0.40283	0.20087	+
[1394]	CCL23	<---	CXCR4	-0.35895	0.21658	-
[1395]	ACTA1	<---	PTPN11	-0.58001	0.63618	+
[1396]	CACNA1D	<---	KCNMB3	-0.49348	0.54975	+
[1397]	NCOA3	--->	TCF12	-0.066532	-0.08315	-
[1398]	SFRP1	--->	WNT11	1.0317	-1.1063	+
[1399]	NR2F1	<---	SRF	-0.20741	0.044585	+
[1400]	ARHGAP17	<---	RHOT1	-0.35613	0.16234	-
[1401]	ERBB4	--->	PLCG2	0.05141	-0.048506	+
[1402]	ADCY4	<---	PRKCE	-0.49495	0.42642	+
[1403]	RHOT1	--->	TIAM1	0.05147	-0.016056	-
[1404]	DKK2	--->	LRP5L	0.47847	-0.43871	+
[1405]	MYC	<---	VEGFA	-0.19227	0.059892	-
[1406]	KAT2B	--->	SMAD4	-0.020213	-0.039669	+
[1407]	CCL7	<---	CCR6	-0.35895	0.21658	-
[1408]	GNAI3	--->	HRAS	0.76886	-0.56799	+
[1409]	JAK1	--->	SOS1	0.37323	-0.4515	+
[1410]	COL11A2	<---	LEPRE1	-0.18291	0.065305	+
[1411]	FGFR2	<---	PRKCH	-0.26548	0.26159	-
[1412]	GNA11	<---	RGS18	-1.2531	1.2063	+
[1413]	CCNB1	<---	PLK1	-0.071915	0.2707	-
[1414]	FZD6	<---	GNA15	-0.097804	0.11301	-
[1415]	FGR	--->	PTK2	0.34449	-0.5161	+
[1416]	CACNA1S	<---	PRKACA	-0.38391	0.51195	+
[1417]	EBF1	<---	SRC	-0.41605	0.62668	-
[1418]	ARHGAP8	<---	RAC3	-0.56218	0.37087	-
[1419]	ANGPT1	<---	EGFR	-0.22938	0.24656	-
[1420]	ITGA1	<---	LAMC2	-0.062785	-0.0050465	+
[1421]	DYRK1A	--->	RBBP4	0.54982	-0.31609	+
[1422]	ACVR2A	<---	BMPR1B	-0.20314	-0.072808	+
[1423]	CCL19	<---	CXCR1	-0.35895	0.21658	-
[1424]	HSP90AA1	--->	PTK2	0.019262	-0.15031	+
[1425]	GNB2	--->	WNT1	0.3343	-0.42584	-
[1426]	BID	<---	ETS1	-0.29746	0.19787	+
[1427]	MAPK10	<---	TAB1	-0.18043	0.60671	+
[1428]	PDGFB	--->	PDGFRA	0.20176	-0.21798	+
[1429]	IL6ST	<---	LEP	-0.11334	0.056231	+
[1430]	GNAI1	<---	HCAR2	-1.1303	1.1631	+
[1431]	NSMAF	<---	TNF	-0.11165	0.073831	+
[1432]	JUN	<---	KLF1	-0.025299	0.094536	-
[1433]	NRAS	<---	SPTAN1	-0.030273	0.12862	+
[1434]	BTK	--->	IGKV4-1	0.20824	-0.07009	-
[1435]	SP3	<---	UTRN	-0.10222	0.095586	-
[1436]	EDNRB	--->	GNA11	1.1631	-1.1303	+
[1437]	GRIN2A	<---	GRM5	-0.36981	-0.05834	+
[1438]	GH1	<---	JAK3	-0.22013	0.087709	-
[1439]	ESR2	--->	GNB1	0.036887	-0.11778	+
[1440]	LAMC1	<---	PTK2	-0.04335	-0.039708	-
[1441]	AJUBA	--->	MAFK	0.69801	-0.66354	-
[1442]	BRCA1	--->	CREBZF	0.60871	-0.44323	+

[1443]	EHMT2	<---	UBTF	-0.0033223	-0.0011318	-
[1444]	AXIN1	--->	HNF1A	-0.0048637	-0.057551	+
[1445]	DDIT3	<---	MAPK12	-0.15935	0.24561	+
[1446]	FZD6	<---	SFRP4	-0.50655	0.27565	+
[1447]	PIK3CB	<---	RAF1	-0.036513	0.0060418	+
[1448]	JUP	<---	MMP9	-0.26058	0.11034	+
[1449]	ID2	<---	PBX3	-0.32236	0.23888	+
[1450]	DRD3	--->	GNAO1	1.1631	-1.1303	+
[1451]	GNAI1	<---	MAPK1	-0.22953	0.50225	-
[1452]	ADCY3	<---	PRKACB	-0.63235	0.8673	-
[1453]	LEPR	--->	SOCS1	-0.098904	-0.12483	-
[1454]	PLCB3	<---	PRKG1	-0.40138	0.33221	+
[1455]	PDGFB	<---	PTPRJ	-0.3388	0.23567	+
[1456]	FCER1A	--->	PLD2	0.1299	-0.10889	+
[1457]	LIPE	<---	PRKACA	-0.43325	0.64376	+
[1458]	FZD8	<---	WNT5B	-0.78209	0.63734	+
[1459]	DNM2	--->	PIK3R5	0.15292	-0.17518	-
[1460]	HSPA1A	--->	NFIC	0.64576	-0.72147	-
[1461]	FGF7	--->	KIT	0.015796	-0.016964	+
[1462]	AR	<---	FGF8	-0.10048	0.054858	-
[1463]	FZD6	<---	WNT16	-0.30406	0.25758	+
[1464]	VAV1	<---	YES1	-0.019255	0.11378	+
[1465]	RYR2	<---	SRI	-0.013867	0.010071	+
[1466]	EXOC2	--->	MYH2	0.076141	-0.013059	-
[1467]	ARF4	--->	EGF	0.13882	-0.17578	-
[1468]	PRKD2	--->	RASAL2	0.19487	-0.082398	-
[1469]	ARAP3	<---	RHOF	-0.39203	0.25315	-
[1470]	EP300	--->	RBMS3	0.021187	-0.018539	+
[1471]	IL18	<---	MAPK13	-0.21628	0.35769	+
[1472]	SOCS2	--->	STAT2	0.27422	-0.74698	-
[1473]	F2	--->	PARD3	-0.0048386	-0.0061601	+
[1474]	ERBB2	--->	FIGF	0.23053	-0.20539	-
[1475]	EP300	--->	EPO	0.0016723	-0.007729	+
[1476]	GRIN1	<---	RAC3	-0.38938	0.54247	-
[1477]	PRKACG	--->	SOX9	0.51637	-0.27888	+
[1478]	ACTR2	<---	RHOJ	-0.51168	0.52339	+
[1479]	ATF3	<---	CCL5	-0.061875	0.15003	-
[1480]	NCOR1	--->	PPARA	-0.018841	-0.11403	+
[1481]	EP300	<---	MDM4	-0.012575	0.0038451	-
[1482]	FADD	--->	IKBKB	0.17249	-0.38531	+
[1483]	PLXNB1	--->	SEMA4D	0.24361	-0.26123	-
[1484]	FRS2	<---	PRKCE	-0.48425	0.40952	-
[1485]	FOXO3	--->	IL7R	0.45535	-0.90965	+
[1486]	IRAK1	--->	TAB1	0.41951	-0.039801	+
[1487]	CD247	<---	SYK	-0.13461	0.052099	-
[1488]	FGF17	--->	FLT1	0.21774	-0.0015767	+
[1489]	MYH3	<---	NEB	-0.093801	0.057586	-
[1490]	GRIN2A	<---	PRKX	-1.006	1.5014	+
[1491]	RHOB	--->	SRGAP1	0.39011	-0.42732	-
[1492]	MAP3K11	--->	MAPK8	1.2829	-1.3853	+
[1493]	LAMB3	--->	PLG	-0.020576	-0.026992	-
[1494]	AKT1	--->	BCL2L1	0.30677	-0.44298	+
[1495]	BMP6	<---	SMAD5	-0.24379	0.060804	-
[1496]	FZD1	<---	GNAQ	-0.22143	0.24435	-
[1497]	CTNND1	<---	RET	-0.31001	9.1776e-006	+
[1498]	RFFL	<---	TNFRSF1A	-0.19178	-0.11508	-
[1499]	PRKCE	--->	STAT1	-0.025941	-0.050583	-
[1500]	KCNU1	<---	PRKG1	-1.0533	0.74543	+
[1501]	GNAO1	<---	PRKACA	-0.22953	0.50225	+
[1502]	SEH1L	--->	SRSF5	0.31391	-0.17132	+
[1503]	BRCA1	--->	JUNB	0.3742	-0.39254	-
[1504]	HRAS	<--->	RALA	0.097643	0.097643	-
[1505]	ATR	--->	TP53	0.031907	-0.022247	+
[1506]	KCNH7	--->	REST	0.27672	-0.20732	-
[1507]	DUSP21	--->	MAPK10	1.4184	-1.4202	+
[1508]	FBXW11	--->	NCSTN	0.34941	-0.19209	-
[1509]	GRIA4	<---	GSK3A	-1.006	1.5014	+
[1510]	CALML3	--->	CAMK2B	0.19292	-0.081704	+
[1511]	IL20	--->	IL5RA	0.11381	-0.12599	+
[1512]	NF1	<---	PRKD1	-0.13347	0.2008	-
[1513]	MAP2K4	<--->	MAPK10	0.31014	0.31014	-
[1514]	FGF16	<---	MET	-0.26028	0.20868	-
[1515]	PRKX	<---	RASD1	-0.20304	1.2491	+
[1516]	BNIP3	<---	FOXO3	-0.37846	0.32839	+
[1517]	FGF18	<---	GRB2	-0.29505	0.31381	-
[1518]	EGR1	<---	ELK4	-0.15205	0.28002	+

[1519]	FGR	--->	RBPJL	0.45232	-0.32136	-
[1520]	FGR	--->	PIK3R3	0.79696	-0.74223	+
[1521]	ACTB	<---	MMP7	-0.29263	0.085904	+
[1522]	ACVR2B	<---	BAMBI	-0.85898	0.90858	+
[1523]	NCBP2	<---	NUP153	-0.54167	0.60094	+
[1524]	ITGAV	<---	LAMA3	-0.041507	-0.0065623	+
[1525]	PIK3CD	--->	PLD1	0.0053638	-0.0095341	+
[1526]	COLEC12	--->	JUN	0.096401	-0.13523	-
[1527]	ARRB2	--->	ROR2	-0.056708	-0.18763	+
[1528]	SOCS2	--->	STAT4	0.27422	-0.74698	-
[1529]	PIK3R1	<---	SRC	-0.27345	0.28568	+
[1530]	CBLC	--->	IL3RA	0.4878	-0.21009	+
[1531]	ARAF	<---	PRKCE	-0.23412	0.18571	+
[1532]	COL5A2	--->	ITGA9	0.0052169	-0.090529	+
[1533]	ACTBL2	<---	MMP2	-0.29263	0.085904	+
[1534]	TNFAIP3	<---	TNFRSF1A	-0.1335	-0.054445	-
[1535]	MAPK14	<--->	PRKACB	0.31014	0.31014	-
[1536]	IL2RB	--->	STAT5B	0.44302	-0.302	+
[1537]	CHRM1	<---	PRKCH	-0.37622	0.27696	-
[1538]	CABLES1	--->	CTNND1	-0.047546	-0.074954	+
[1539]	CCL24	<---	CXCR6	-0.35895	0.21658	-
[1540]	DRD3	--->	GNB5	1.1575	-0.92616	+
[1541]	BRCA1	--->	IGF1R	0.40962	-0.14169	+
[1542]	MPHOSPH8	--->	PBX3	0.032887	-0.13256	-
[1543]	CTNNB1	<---	GJA1	-0.27358	0.20637	-
[1544]	GAB3	<---	PRKCG	-0.50087	0.66104	-
[1545]	FOS	<---	LRCH4	-0.27991	0.25231	-
[1546]	FGF16	<---	GRB2	-0.29505	0.31381	-
[1547]	CXCR3	--->	TAF1	0.11779	-0.2522	-
[1548]	CD14	<---	PLCG2	-0.014111	0.027311	+
[1549]	ATP2B4	<---	PRKACG	-0.060388	0.088587	+
[1550]	OSBP	<---	PPM1L	-0.83065	1.0338	-
[1551]	PDGFRB	--->	SOS2	0.053022	0.0048838	+
[1552]	MAP3K3	--->	MAPK9	1.3429	-1.347	+
[1553]	CSNK1A1L	--->	GLI2	1.4077	-0.99235	+
[1554]	MRAS	--->	PIK3CD	0.011011	-0.01026	+
[1555]	LCP2	--->	RAC2	1.3361	-1.3049	+
[1556]	GNGT2	<---	MAPK12	-0.41411	0.50061	-
[1557]	ABCB1	<---	EGR1	0.008373	0.023053	+
[1558]	FZD8	<---	GNB1	-0.12321	0.10912	-
[1559]	FOXA1	--->	HNF4A	0.076395	-0.21893	-
[1560]	RHOG	--->	SRGAP3	0.39011	-0.42732	-
[1561]	RAC3	--->	TAGAP	1.016	-1.5245	-
[1562]	PVRL3	<---	SRC	-0.19916	0.30386	-
[1563]	CD40LG	<---	TRAF3	-0.52996	0.66546	-
[1564]	BIRC3	--->	GZMB	0.020947	-0.083567	-
[1565]	ADRA2B	<---	GRK7	-0.17569	0.20291	+
[1566]	CTNNA1	--->	CTNNB1	0.73494	-0.81423	-
[1567]	CD44	<---	LAMC3	-0.06897	0.0013783	+
[1568]	DAND5	<---	FAS	-0.2899	0.036892	-
[1569]	PIK3R1	<---	PVRL3	-0.028432	0.036969	+
[1570]	KCNJ3	<---	OPRD1	-0.2522	0.11779	-
[1571]	IGFBP1	<---	PGR	-0.14879	-0.015388	+
[1572]	ARHGAP32	<---	RHOU	-0.48747	0.29572	-
[1573]	COL3A1	--->	SMAD3	-0.0080619	-0.10301	-
[1574]	SIX5	--->	ZNF331	0.078944	0.049909	+
[1575]	CCL21	<---	CXCR6	-0.35895	0.21658	-
[1576]	FANCB	<---	FANCM	-0.10116	0.029203	+
[1577]	CTNNB1	<---	ERBB4	-0.22663	0.22796	+
[1578]	CDH1	<---	MMP23A	-0.29512	0.10165	+
[1579]	HNF4A	<---	NR0B2	-0.014718	-0.0076266	-
[1580]	ADRB2	--->	GNAL	1.1631	-1.1303	+
[1581]	ITGA3	<---	LAMC1	-0.030111	-0.0092525	+
[1582]	CBLB	--->	CD19	0.2466	-0.10039	+
[1583]	SLC22A2	<---	SP1	-0.2032	0.35054	+
[1584]	MYB	--->	SKI	0.036068	-0.048028	+
[1585]	CTCF	<---	HPN	-0.0091154	0.083205	-
[1586]	THRA	--->	WNT4	0.24686	-0.46784	+
[1587]	CREB3	<---	PRKG2	-0.79318	0.9599	+
[1588]	RHOV	--->	SYDE2	1.016	-1.5245	-
[1589]	ITGAV	<---	LAMB4	-0.066621	-0.0090065	+
[1590]	ATF1	--->	USF1	0.19431	-0.13736	-
[1591]	ADCY3	<---	GNAL	-0.63317	1.0384	-
[1592]	CXCR4	<---	GRK4	-0.28786	0.33635	+
[1593]	HOMER1	--->	PIK3R3	-0.0054307	-0.057628	+
[1594]	IL26	--->	IL6R	0.12377	-0.18387	+

[1595]	CXCR3	<---	JAK3	-0.12268	-0.039248	-
[1596]	DAND5	--->	VEGFB	0.01248	-0.048119	+
[1597]	CUL2	--->	UBE2D3	0.065927	-0.14391	+
[1598]	BMPR1B	<---	MAP3K7	-0.24273	0.024072	-
[1599]	TCF7L2	--->	TLE4	0.20358	0.0065975	-
[1600]	EIF4EBP1	<---	RPS6KA4	-0.89196	0.67958	+
[1601]	ABCC3	--->	ALB	-0.091253	-0.17229	+
[1602]	PPP2CB	<---	STAT1	-0.18258	0.16953	-
[1603]	CXCR1	--->	PPBP	0.21658	-0.35895	-
[1604]	FGFR2	<---	PDGFB	-0.23595	0.12039	+
[1605]	MYL12A	--->	PPP1R12C	0.27396	-0.40226	-
[1606]	FOS	--->	MTOR	0.3423	-0.22539	+
[1607]	TBK1	<---	TICAM1	-0.38376	0.69721	+
[1608]	SIX5	<---	ZNF227	0.0092332	0.065182	-
[1609]	CHRM1	<---	GRK7	-0.28786	0.33635	+
[1610]	MYL4	<---	NEB	-0.35332	0.30092	-
[1611]	FOXO1	--->	IL6ST	0.17869	-0.34117	-
[1612]	MYO1C	<---	RAB13	-0.038582	0.076038	+
[1613]	IFNB1	--->	IL13RA2	0.29642	-0.56068	+
[1614]	CLEC7A	<---	SYK	-0.22013	0.087709	-
[1615]	CRK	--->	RAP2B	0.43868	-0.057401	+
[1616]	ACVR1C	<---	LEFTY2	-0.33406	0.27387	+
[1617]	CAMK2B	--->	GRIA1	0.94748	-0.64695	-
[1618]	CD40LG	<---	REL	-1.1394	0.93112	+
[1619]	IGF1	--->	STAT3	0.46738	-0.34275	-
[1620]	CREB3L1	<---	RPS6KA5	-0.98092	1.2458	+
[1621]	KLRG1	<---	LYN	-0.27641	0.38494	-
[1622]	ITGA10	--->	THBS2	0.081101	-0.066168	-
[1623]	CSNK1E	--->	WNT2	0.83912	-0.78694	-
[1624]	RAP1B	--->	SKAP1	0.32282	0.0528	-
[1625]	CDC42	--->	PIK3CB	0.011011	-0.01026	-
[1626]	EGFR	<---	PRKCQ	-0.059824	0.12383	-
[1627]	HIST1H3A	<---	SUV39H1	-0.18758	0.15209	+
[1628]	PDK1	--->	RXRA	0.10013	-0.32848	-
[1629]	IL20RB	<---	IL23A	-0.087402	0.20113	+
[1630]	GSC	--->	YY1	0.078944	0.049909	-
[1631]	ENG	<---	MAPK1	-0.48426	0.70593	-
[1632]	ARF1	--->	CYTH2	0.19683	-0.33222	-
[1633]	FGD4	<---	RHOJ	-0.12937	0.14242	-
[1634]	CHUK	<---	MALT1	-0.86011	0.79	+
[1635]	MYO9B	<---	RHOJ	-0.54674	0.38058	-
[1636]	MAX	<---	TNFRSF1A	-0.38203	0.2192	-
[1637]	MAPK14	--->	SRF	0.35865	-0.36564	+
[1638]	GPR37	<---	HSPA6	-0.53064	0.43836	+
[1639]	GNB3	<---	PRKCG	-0.27923	0.22728	+
[1640]	CREBBP	<---	PRKX	-0.006941	0.013728	+
[1641]	NEUROG3	<---	PAX4	-0.19662	0.14379	-
[1642]	CBLC	--->	IFNAR1	1.4386	-0.93248	+
[1643]	FGF23	--->	KDR	0.21774	-0.0015767	+
[1644]	MAPK8	<---	RIPK2	-0.29078	0.20201	+
[1645]	PIK3CD	<---	THRA	-0.021852	-0.0056486	-
[1646]	IKZF3	<---	RASA1	-0.16125	0.23487	+
[1647]	FXYD2	<---	NR3C2	-0.14879	-0.015388	+
[1648]	NR2F2	--->	ZNF263	0.14696	-0.13354	-
[1649]	CTNNB1	<---	PCDH15	-0.19204	0.11503	-
[1650]	CALM1	--->	EGF	0.21228	-0.20736	-
[1651]	LIMK1	--->	ROCK2	0.13188	-0.07742	-
[1652]	MEF2C	--->	MYOD1	-0.0062373	-0.17031	-
[1653]	CUL3	--->	PSMD10	0.32103	-0.32277	-
[1654]	LEF1	--->	NRCAM	0.10914	-0.066772	+
[1655]	ADCY7	<---	PRKACB	-0.63235	0.8673	-
[1656]	GNA15	--->	WNT10B	0.42087	-0.47341	-
[1657]	PIK3CA	--->	SOS1	0.010347	0.003946	-
[1658]	KRAS	--->	PDGFB	0.030327	-0.0099201	-
[1659]	FSHB	<---	FSHR	-0.29972	0.16079	-
[1660]	ADORA2B	<---	GRK5	-0.28786	0.33635	+
[1661]	MAPK1	--->	NELFE	1.2133	-0.47902	-
[1662]	GNAO1	<---	RGS2	-1.2531	1.2063	+
[1663]	DVL2	<---	WNT2B	-0.54867	0.38842	+
[1664]	GRIK5	<---	RHOG	-0.38938	0.54247	-
[1665]	COL5A3	--->	ITGAV	-0.013036	-0.10803	+
[1666]	AKT3	--->	PIK3C3	0.048764	-0.083121	-
[1667]	PRKACA	--->	RPS6KA5	0.9519	-1.129	+
[1668]	NKX2-3	--->	TCF12	0.23888	-0.32236	-
[1669]	IFIH1	--->	IKBKB	0.076669	0.0020064	+
[1670]	GNB1	<---	PIK3CA	-0.010082	0.0028834	-

[1671]	LAMA1	<---	PTK2	-0.04335	-0.039708	-
[1672]	CBLB	--->	SPRY2	0.29155	-0.14969	-
[1673]	IL5RA	--->	JAK2	0.16565	0.076641	-
[1674]	CXCL9	<---	CXCR6	-0.35895	0.21658	-
[1675]	PPBP	<---	XCR1	-0.35895	0.21658	-
[1676]	MAPK6	--->	STAT6	0.85786	-0.58701	+
[1677]	PARD3	--->	PVRL2	0.07545	-0.093624	-
[1678]	ITGA8	<---	LAMB1	-0.066621	-0.0090065	+
[1679]	CREB5	<---	PRKG2	-0.79318	0.9599	+
[1680]	AKT1	--->	DEPTOR	0.1831	-0.24055	+
[1681]	HNRNPA0	<---	TAF1	-0.31439	0.36909	+
[1682]	DUSP14	--->	MAPK11	1.4184	-1.4202	+
[1683]	G6PC3	<---	HNF4A	-0.7847	0.51998	+
[1684]	BCAR1	--->	GFRA1	0.14189	-0.1329	-
[1685]	CTNNB1	<---	HNF1A	-0.32674	0.22011	-
[1686]	CSNK2A2	--->	NFKB1	0.20986	-0.059594	-
[1687]	CASP8	--->	IKBKB	0.89389	-1.332	+
[1688]	CASP3	--->	PTK2	0.10272	-0.39495	+
[1689]	BDNF	<---	CDC42	-0.48322	0.53989	-
[1690]	FOS	<---	MAPK10	-0.57905	1.0858	+
[1691]	PARD3	--->	RAC3	0.23118	0.053034	-
[1692]	PTPN11	--->	SGMS1	0.36484	-0.29307	+
[1693]	FZD7	<---	WNT5B	-0.78209	0.63734	+
[1694]	MET	--->	PLCB1	0.068415	-0.090465	+
[1695]	F2RL2	--->	PRSS1	0.11779	-0.2522	-
[1696]	OCRL	<---	RHOT2	-0.35613	0.16234	-
[1697]	PIK3C2G	<---	S1PR1	-0.029714	-0.00027016	+
[1698]	PDPK1	--->	PIK3CB	0.081383	-0.030162	-
[1699]	HOXB9	--->	RXRA	-0.16523	-0.18775	-
[1700]	CREBBP	--->	GAPDH	-0.00068954	-0.0041449	+
[1701]	MYBPC1	<---	MYH6	-0.15073	0.094518	+
[1702]	EDNRA	--->	SLC9A1	0.11779	-0.2522	+
[1703]	FN1	<---	RAC1	-0.28606	0.2414	-
[1704]	CEBPA	--->	E2F2	0.0081868	-0.082946	+
[1705]	MAX	<---	ZBTB17	-0.023698	0.070404	+
[1706]	CDC42	--->	EP300	0.00089974	0.00089005	-
[1707]	BIRC7	<---	NFKB1	-0.13417	0.10963	+
[1708]	E2F6	--->	HLTF	0.24556	-0.22836	+
[1709]	PIK3CB	<---	PRKCQ	-0.045695	0.032777	-
[1710]	DUSP9	--->	MAPK14	1.353	-1.036	+
[1711]	JAK1	--->	PIK3R2	0.18777	-0.36432	+
[1712]	PPP2R1B	--->	STK3	0.40754	-0.3445	+
[1713]	DEPDC7	<---	RHOJ	-0.058594	0.11751	-
[1714]	NFKBIA	<---	REL	-0.62901	0.2336	+
[1715]	CCR1	--->	GNAI1	1.1631	-1.1303	+
[1716]	GNAI3	--->	GRM2	0.24033	-0.15623	-
[1717]	CALML3	--->	CAMK2A	0.19292	-0.081704	+
[1718]	EPHA8	--->	RAC1	0.039661	0.014723	+
[1719]	EGF	<---	SHC1	-0.06309	0.054752	-
[1720]	NRAS	<---	SOCS3	-0.093407	0.044639	+
[1721]	GPR65	--->	MEF2A	0.21915	-0.42605	-
[1722]	CER1	--->	FZD9	0.27359	-0.29711	+
[1723]	MMP9	<---	SMAD3	-0.12754	-0.050595	+
[1724]	SRCAP	--->	ZEB1	0.15646	-0.13113	-
[1725]	NOL3	<---	RELA	-0.88183	0.6154	+
[1726]	RAB5C	<--->	RAB7A	0.1628	0.1628	-
[1727]	GNAI2	<---	RGS6	-0.736	0.71839	+
[1728]	GNAI1	<---	HTR5A	-1.1303	1.1631	+
[1729]	FYN	--->	MAPK12	0.92013	-1.0925	+
[1730]	GNAI3	--->	RASD1	1.2694	-0.93465	-
[1731]	CDKN1A	<---	RELB	-0.47368	0.37072	+
[1732]	INSRR	<---	PDGFB	-0.021519	0.16712	+
[1733]	FGF12	<---	MET	-0.26028	0.20868	-
[1734]	CNTF	--->	OSMR	0.073187	-0.17721	+
[1735]	CXCR2	<---	GRK4	-0.28786	0.33635	+
[1736]	CDK1	--->	SEC13	0.54982	-0.31609	+
[1737]	ACTB	<---	MMP28	-0.29263	0.085904	+
[1738]	IKBKB	<---	TRAF2	-0.4424	0.8755	+
[1739]	CSF2RA	--->	SOCS3	-0.014037	-0.24629	-
[1740]	EGFR	<---	YES1	-1.474	0.20438	+
[1741]	TNF	<---	TNIK	-0.22921	0.62058	-
[1742]	SRC	--->	TRIP6	0.55031	-0.33502	+
[1743]	CD3E	--->	IL2RB	-0.0077042	-0.055821	+
[1744]	PRKCD	--->	RAF1	0.20778	-0.36938	+
[1745]	CCL2	<---	SMAD3	-0.11489	-0.043113	+
[1746]	ARHGEF2	<---	CDC42	-0.093742	0.10079	-

[1747]	PRKAG3	--->	TSC2	-0.061017	-0.063939	+
[1748]	PPARA	<---	PRKX	-0.30308	0.29076	+
[1749]	MEF2A	<---	TP63	-0.14294	0.05577	-
[1750]	IRAK2	--->	MAP2K6	0.73444	-0.20293	+
[1751]	AKT1	<---	HSP90AA1	-0.59063	0.6369	-
[1752]	LPAR3	<---	NFKB1	-0.43941	0.47827	-
[1753]	AKT3	--->	GAB3	0.24623	-0.074658	-
[1754]	MAPK12	<---	PPP2R2A	-0.068084	0.2712	-
[1755]	EP300	<---	PDK1	-0.023486	0.017046	-
[1756]	BMP4	<---	TAB2	-0.45928	0.36401	-
[1757]	ADRBK2	--->	HTR1E	0.25327	-0.21301	+
[1758]	EGF	<---	YES1	-0.13921	0.19166	+
[1759]	COL5A1	<---	GP6	-0.27286	0.25603	-
[1760]	BCL3	<---	TNFRSF6B	-0.16544	0.14795	-
[1761]	FGF18	--->	PDGFRB	0.48772	-0.57252	+
[1762]	CSNK1D	--->	WNT1	0.83912	-0.78694	-
[1763]	FOXO4	--->	GABARAP	1.0193	-0.41286	+
[1764]	ARHGEF16	<---	RAC2	-0.067065	0.085273	-
[1765]	BRCA1	--->	DCAF6	0.45211	-0.38063	+
[1766]	CBL	--->	CSF2RA	0.28248	-0.14259	+
[1767]	MYLK3	<---	PAK4	-1.2674	1.5523	+
[1768]	JUND	<---	SFTPD	-0.13523	0.096401	-
[1769]	PAK7	<---	RAF1	-0.76991	0.68927	-
[1770]	CDKN1A	<---	PPARG	-0.081844	0.041128	+
[1771]	PLXNB2	<---	SEMA4C	-0.063433	0.098848	+
[1772]	ACOX2	--->	RXRB	-0.03534	-0.098167	-
[1773]	CNOT1	<---	HNF4A	-0.2804	0.10664	+
[1774]	IL10RA	<---	IL23A	-0.087402	0.20113	+
[1775]	HOXD8	--->	TCF12	0.23888	-0.32236	-
[1776]	CAMK2A	--->	RYR2	0.042152	-0.028973	+
[1777]	BMPR1A	--->	MAPK1	0.17987	-0.35209	+
[1778]	PPP1CB	--->	YAP1	0.95833	-1.0588	+
[1779]	EGFR	--->	FGF9	0.25979	-0.22928	-
[1780]	ITGB1	--->	PIK3CD	-0.0036365	-0.0057548	+
[1781]	CBL	--->	IFNLR1	0.4819	-0.29306	+
[1782]	PRKACG	<---	RASD1	-0.20304	1.2491	+
[1783]	ARAP1	<---	RAP1A	-0.48615	0.36733	+
[1784]	GRIN1	<---	RAC2	-0.38938	0.54247	-
[1785]	GRK7	--->	HTR2B	0.33635	-0.28786	+
[1786]	CD3E	--->	PIK3CG	-0.0065677	-0.016061	+
[1787]	CCNB1	<---	WEE1	-0.55661	0.75243	-
[1788]	FOXA2	--->	NR1D1	0.076395	-0.21893	+
[1789]	FZD5	<---	WNT4	-0.78209	0.63734	+
[1790]	IL10	<---	PTAFR	-0.2522	0.11779	+
[1791]	MRAS	--->	RGL2	1.4276	-1.2366	+
[1792]	FOSL2	<---	MAPK13	-0.57905	1.0858	+
[1793]	GRAP	<---	PRKCE	-0.20419	0.22188	-
[1794]	DRD5	--->	KCNJ5	0.11779	-0.2522	-
[1795]	JAK2	--->	STAM2	0.018374	-0.0056557	-
[1796]	MAPK9	<---	TRAF5	-0.47605	1.1617	+
[1797]	CSNK1A1L	--->	WNT16	0.83912	-0.78694	-
[1798]	RAC3	--->	VAV3	0.058196	0.018587	-
[1799]	GNAI2	<---	RGS18	-1.2531	1.2063	+
[1800]	ADRA1D	<---	GRK7	-0.28786	0.33635	+
[1801]	LRP6	<---	NCSTN	-0.44675	0.5925	+
[1802]	PDPK1	--->	PRKCD	0.54086	-0.92024	+
[1803]	WIF1	--->	WNT8A	0.49247	-0.8346	+
[1804]	CD44	--->	FN1	-0.017319	-0.23893	-
[1805]	FOXO4	--->	GABARAPL1	1.0193	-0.41286	+
[1806]	BIRC2	--->	GZMB	0.020947	-0.083567	-
[1807]	NFKB1	--->	PSMD7	0.19956	-0.19547	-
[1808]	IL18	<---	RELA	-0.42007	0.13655	-
[1809]	DRD4	<---	GRK5	-0.28786	0.33635	+
[1810]	CREB1	<---	LDHA	-0.060871	0.092699	-
[1811]	L1CAM	<---	SRC	-0.38095	0.11864	+
[1812]	RAC1	<---	TBXA2R	-0.13328	0.12209	-
[1813]	GNAI2	<---	RGS2	-1.2531	1.2063	+
[1814]	NFKBIA	<---	PSMD4	-0.17765	0.034641	+
[1815]	CCL4	<---	CXCR6	-0.35895	0.21658	-
[1816]	CNN2	<---	SRF	-0.35947	0.27595	+
[1817]	CTNNA1	--->	YWHAZ	1.0272	-1.0665	+
[1818]	ERBB4	--->	PSEN2	0.24864	-0.22414	-
[1819]	ITGB1	<---	PTP4A3	-0.25132	0.20519	+
[1820]	FADD	--->	MAPK3	0.081126	-0.58844	-
[1821]	ITGB7	<---	SRC	-0.27641	0.38494	-
[1822]	FOXO4	<---	NLK	-0.22085	0.2499	+

[1823]	BHLHE40	<---	ZNF460	-0.37439	0.40971	-
[1824]	ITGA5	<---	PTK2	-0.076638	-0.038051	-
[1825]	CREB1	<---	TF	-0.088144	0.12637	-
[1826]	CSNK1G3	<--->	GSK3B	0.18701	0.18701	-
[1827]	KPNA5	<---	NUP43	-0.11171	0.067727	+
[1828]	PIK3R3	--->	PTK2	-0.36936	-0.56806	-
[1829]	ERG	<---	SETDB1	-0.041135	0.02691	-
[1830]	ADCY6	<---	CAMK2B	-0.44346	0.58749	+
[1831]	ETS1	<---	PRAME	-0.04824	0.02106	-
[1832]	ABI1	--->	ACTR3	0.41622	-0.41852	+
[1833]	SMPD3	<---	TRAF2	-0.3125	0.26384	+
[1834]	CD4	<---	PSMD4	-0.088072	0.049998	+
[1835]	GNB3	--->	GRM3	0.082045	-0.075766	-
[1836]	CSNK2A1	<---	PPP6C	-0.12622	0.14153	-
[1837]	ADRBK2	--->	CCR10	0.25327	-0.21301	+
[1838]	PRKCI	<---	TRIP10	-0.038692	0.0037462	+
[1839]	CAMK2D	--->	GRIA1	0.94748	-0.64695	-
[1840]	GNAO1	--->	GRM8	0.24033	-0.15623	-
[1841]	NR3C1	--->	SMAD3	-0.024404	-0.1374	-
[1842]	CCR7	--->	PF4V1	0.21658	-0.35895	-
[1843]	RRAS	<---	SYNGAP1	-0.68738	0.87061	+
[1844]	NPY	--->	SSTR1	0.89835	-1.3205	+
[1845]	DVL2	<---	SENP2	-0.59348	0.48328	+
[1846]	CEBPB	<---	SFRP1	-0.36172	0.40861	-
[1847]	GSK3B	--->	PSME4	0.57437	-0.5963	-
[1848]	GNGT2	<---	MTNR1A	-0.26888	0.10335	+
[1849]	PLEKHG2	<---	RHOU	-0.23262	0.26814	-
[1850]	GNAI1	<---	KCNJ6	-0.011498	0.05262	-
[1851]	CCNT2	--->	TCF12	0.21622	-0.26507	+
[1852]	NR3C1	--->	SKIL	-0.010433	-0.091347	+
[1853]	ACTA2	<---	MMP12	-0.29263	0.085904	+
[1854]	CHUK	<---	TAB3	-0.20333	0.26163	+
[1855]	EP300	--->	PDGFRA	0.009775	-0.01506	+
[1856]	COL5A2	--->	ITGB1	0.0024845	-0.035647	+
[1857]	PIM1	--->	TCF7L2	1.3891	-0.77835	-
[1858]	G6PC	--->	USF1	0.25527	-0.35391	-
[1859]	CCL11	<---	CXCR2	-0.35895	0.21658	-
[1860]	F11R	<---	MLLT4	-0.067087	0.075867	+
[1861]	EGR1	<---	IRF2	-0.15205	0.28002	-
[1862]	IFNB1	--->	LEPR	0.27509	-0.55283	+
[1863]	TCF7L2	--->	ZNF595	0.31266	-0.026148	+
[1864]	CDC42	--->	PLD1	0.1299	-0.11526	+
[1865]	ANKRD1	<---	JUND	-0.073947	0.087304	+
[1866]	AKT1	--->	PIK3R1	0.18336	-0.2541	-
[1867]	GP1BB	--->	PROC	0.17287	-0.24288	-
[1868]	GNAI3	<---	MAPK13	-0.22953	0.50225	-
[1869]	CLOCK	<---	NCOR1	-0.22156	0.24046	-
[1870]	LCK	--->	SGMS1	0.63648	-0.45391	+
[1871]	ESR2	--->	GNA14	0.042535	-0.12976	+
[1872]	CSNK2A1	--->	PAFAH1B1	0.33238	-0.19282	+
[1873]	ITGAL	<---	PRKCZ	-0.051128	-0.0042668	+
[1874]	CCL3	<---	CCR7	-0.35895	0.21658	-
[1875]	CTCFL	<---	KAT2B	-0.037332	0.083726	-
[1876]	KLHL12	--->	PSME4	-0.012014	-0.084972	-
[1877]	CBL	--->	IL20RA	0.61825	-0.39145	+
[1878]	FYN	--->	VAV2	0.072747	-0.065501	+
[1879]	BIRC5	--->	CCNB1	0.37866	-0.39123	-
[1880]	NCK1	--->	RAC2	0.56021	-0.18722	+
[1881]	APP	<---	EP300	-0.0029773	-0.0025097	+
[1882]	DUSP19	--->	MAPK9	1.4184	-1.4202	+
[1883]	ABR	<---	RHOT2	-0.047245	-0.014168	-
[1884]	ADCY7	<---	PRKCA	-0.49495	0.42642	+
[1885]	CRTC2	--->	YWHAE	-0.0021827	-0.10486	-
[1886]	FOS	<---	MAPK11	-0.57905	1.0858	+
[1887]	CDK1	--->	PSMD4	0.40675	-0.26091	-
[1888]	PRKACB	--->	TRPM8	0.61419	-0.44517	+
[1889]	JUN	<---	TCF7L1	-0.51678	0.699	+
[1890]	EP300	--->	ZBTB32	0.0090612	-0.0074415	+
[1891]	SMPD1	--->	TNFRSF10B	-0.13353	-0.22431	-
[1892]	GALR1	--->	MYC	0.13244	-0.36462	-
[1893]	FGF20	<---	MAPK3	-0.47959	0.71493	+
[1894]	IL12RB1	<---	IL19	-0.29036	0.12248	+
[1895]	FGF5	<---	MAPK3	-0.47959	0.71493	+
[1896]	CDC20	<---	UBE2C	-0.2601	0.27426	+
[1897]	OPHN1	<---	RHOD	-0.9609	0.7795	-
[1898]	IL12RB1	<---	NFKBIA	-0.21284	-0.012289	-

[1899]	EDNRB	<---	MAPK14	-0.10024	0.19743	-
[1900]	MYB	--->	SIM2	0.14475	-0.13548	+
[1901]	CTCF	<---	NFATC4	-0.1643	0.16726	-
[1902]	CCND2	<---	SOS1	0.0039278	0.094104	+
[1903]	GAB1	<---	MAPK8	-0.42795	1.1652	-
[1904]	NCOA2	<---	RARA	-0.054726	0.035483	+
[1905]	FGF9	--->	FGFR3	0.071829	-0.16555	-
[1906]	PPP2R2A	--->	PRKCZ	0.38699	-0.50418	+
[1907]	USF1	<---	YY1	-0.20142	0.33079	+
[1908]	MAPK8	--->	TRPV1	0.54889	-0.41958	+
[1909]	EP300	<---	LRRK2	-0.0043024	-0.0026809	-
[1910]	ARAP3	<---	RHOV	-0.39203	0.25315	-
[1911]	MAPK1	--->	PRKCQ	-0.20738	-0.21837	-
[1912]	ITGA11	<---	LAMA2	-0.025761	-0.0054603	+
[1913]	RACGAP1	<---	RHOF	-1.011	0.68428	-
[1914]	JAK3	--->	SOCS2	1.1559	-1.1639	-
[1915]	H3F3A	<---	NFKB1	-0.29753	0.29301	-
[1916]	JUN	<---	PCDHB11	-0.18757	0.098606	-
[1917]	AKT3	--->	CREB3L2	0.82132	-1.3257	+
[1918]	FGFR1	<---	SHC1	-0.10391	-0.029269	-
[1919]	ADAM17	--->	COL17A1	0.088795	-0.17277	+
[1920]	ADRBK1	--->	HTR1A	0.25327	-0.21301	+
[1921]	HGF	<---	HGS	-0.011696	-0.0093361	+
[1922]	EP300	<---	PRKX	-0.006941	0.013728	+
[1923]	ANGPT1	--->	ARNT	0.0052685	-0.16068	-
[1924]	DHX38	--->	NUP37	0.072414	-0.071625	-
[1925]	PDGFB	--->	STAT3	0.2715	-0.20049	+
[1926]	TCF7L2	--->	ZNF805	0.31266	-0.026148	+
[1927]	PEBP1	<---	PRKCH	-0.58926	0.32382	+
[1928]	GNB2	--->	WNT5A	0.3343	-0.42584	-
[1929]	RAP1B	<---	RASGRP2	-0.24352	0.27322	+
[1930]	IL12B	--->	PIK3R1	-0.041749	-0.064276	+
[1931]	BIRC7	<---	CASP3	-0.35112	0.045424	-
[1932]	IL23R	--->	STAT3	0.26761	-0.18427	-
[1933]	HNF4A	<---	TCF7L2	-0.983	0.9955	+
[1934]	BCAR1	--->	ITGB4	0.028893	-0.034822	-
[1935]	ATF6B	<---	NPY	-0.81635	0.84205	-
[1936]	RELA	--->	VEGFA	0.066025	-0.23807	+
[1937]	ARHGAP24	<---	RHOT1	-0.22584	0.2121	-
[1938]	PEBP1	<---	PRKCQ	-0.63034	0.25209	+
[1939]	CBLB	--->	IFNAR2	0.61825	-0.39145	+
[1940]	HOMEZ	--->	SREBF1	0.045133	-0.11838	-
[1941]	ATF6B	<---	GATA2	-0.16066	0.25678	+
[1942]	EREG	<---	ZNF263	0.033936	0.099135	+
[1943]	CREB5	--->	MCL1	0.72216	-1.2248	+
[1944]	HIF1A	--->	HSP90B1	0.1387	-0.019106	+
[1945]	GNAI1	<---	RGS17	-1.2531	1.2063	+
[1946]	FYN	--->	ITGA7	0.23404	-0.20204	-
[1947]	ETS1	--->	PBX1	0.095387	-0.14907	+
[1948]	CD247	--->	CD4	0.029226	-0.054518	-
[1949]	COL6A6	--->	ITGA9	-0.0067115	-0.019363	+
[1950]	CD247	--->	IGKV1-5	-0.021439	-0.042516	-
[1951]	ESR1	<---	STAT1	-0.18995	0.13825	+
[1952]	E2F4	<---	SMAD2	-0.30541	0.36653	+
[1953]	SRC	--->	UNC5C	0.020547	-0.038954	+
[1954]	PPP3CC	<---	YWHAQ	-0.31613	0.09356	-
[1955]	MSN	--->	ROCK2	0.036127	-0.038345	-
[1956]	BCAR1	--->	ITGA11	0.070773	-0.089729	-
[1957]	ADCY9	<---	PRKCD	-0.47485	0.42125	+
[1958]	CD79B	<---	PIK3AP1	-0.042516	-0.021439	-
[1959]	AKT2	--->	GRAP	0.077189	-0.13812	-
[1960]	HLA-DQA2	--->	TCRA	0.12456	-0.36464	+
[1961]	CDK1	--->	PAFAH1B1	0.33238	-0.19282	+
[1962]	ACAP1	<---	ARF6	-0.26479	0.28761	-
[1963]	FGFR3	<---	PRKCE	-0.086608	0.13769	-
[1964]	ATF2	--->	HIST1H2BB	0.048146	0.0081543	+
[1965]	AAAS	--->	KPNA7	0.12101	-0.19114	+
[1966]	EGFR	--->	PPP2R2A	0.18956	-0.16113	+
[1967]	GJA1	--->	JUP	0.20637	-0.27358	-
[1968]	CSF1R	--->	PIK3R1	0.35687	-0.036824	+
[1969]	ARRB2	<---	CSNK1G3	-0.09775	0.14787	-
[1970]	CRK	<---	PIK3R3	0.055027	0.30578	-
[1971]	MYC	<---	POU2F2	-0.21331	-0.00063842	+
[1972]	CASP12	<---	CASP3	-0.88853	-0.039589	-
[1973]	JAK3	--->	STAT5B	0.56357	-1.1931	+
[1974]	FOXO1	--->	JAK1	-0.022797	-0.061344	-

[1975]	SP1	<---	TBL1XR1	-0.033889	0.21107	+
[1976]	CBL	--->	OSMR	0.18092	-0.10684	+
[1977]	BRCA1	--->	TAL1	0.79188	-0.80689	-
[1978]	DIAPH2	<---	RHOA	-0.01596	0.026206	+
[1979]	GNB1	<---	LCK	-0.29244	0.41365	-
[1980]	JUND	--->	MNT	0.071708	-0.13126	+
[1981]	ARHGEF26	<---	RHOG	-0.067065	0.085273	-
[1982]	FGFR1	<---	SHC2	-0.10391	-0.029269	-
[1983]	MAP2K3	<---	TRADD	-0.14866	-0.051349	+
[1984]	CREB1	<---	TPO	-0.077588	0.087444	-
[1985]	GNAI2	<---	RGS3	-0.50586	0.48145	+
[1986]	AKT2	--->	ESR1	0.7641	-0.82222	-
[1987]	FGF3	<---	GRB2	-0.29505	0.31381	-
[1988]	ITGB1	<---	RAC1	-0.01596	0.026206	-
[1989]	CLEC4E	<---	SYK	-0.22013	0.087709	-
[1990]	CCL2	<---	EP300	-0.010336	0.0055944	+
[1991]	AKAP13	<---	RHOV	-0.22586	0.072537	-
[1992]	MAPK8	<---	RAC2	-0.20304	1.2491	+
[1993]	CCNE2	<---	E2F3	-0.39339	0.45542	+
[1994]	APC	<---	RAC1	-0.019081	0.012037	-
[1995]	GLI3	--->	WNT9A	0.86373	-1.47	+
[1996]	DRD4	--->	GNB1	0.69217	-0.57296	+
[1997]	GRAP2	--->	NRAS	0.38643	0.00072409	+
[1998]	NFKB1	<---	PKN3	-0.060899	0.080625	+
[1999]	EIF2AK4	<---	PPP1CC	-0.12622	0.14153	+
[2000]	CCND1	<---	ESR1	-0.10703	0.070418	+
[2001]	CBLC	--->	IL5RA	0.62472	-0.35538	+
[2002]	GLI1	--->	WNT7A	0.86373	-1.47	+
[2003]	CCR6	<---	GRK1	-0.28786	0.33635	+
[2004]	CAMK2A	--->	FLNA	0.30815	-0.21817	+
[2005]	GRIA4	<---	PRKCB	-0.43995	0.47541	+
[2006]	CEBPA	<---	PPARG	-0.078543	-0.026969	+
[2007]	CAPN2	<---	TLN2	-0.009837	0.003375	-
[2008]	PPIA	<---	TAF1	-0.068802	-0.024575	+
[2009]	BRCA1	--->	CAND1	0.14246	-0.15045	+
[2010]	FGF6	--->	INSR	0.25346	-0.0044024	+
[2011]	INPP5D	<---	INS	-0.25116	0.15919	+
[2012]	GNB5	<---	PIK3CD	-0.012551	0.008521	-
[2013]	EP300	<---	F2RL1	-0.0097452	0.0029784	-
[2014]	RHOJ	--->	TIAM2	0.20928	-0.13672	-
[2015]	NTRK1	<---	PIK3R5	-0.26539	-0.054263	-
[2016]	GRB2	<---	MAP2K1	-0.34568	0.27364	-
[2017]	RPTOR	<---	RRAGD	-0.69387	1.0493	+
[2018]	POU2F1	<---	SFTPA1	-0.14907	0.095387	-
[2019]	ADCY4	<---	PRKACA	-0.63235	0.8673	-
[2020]	APBB1	--->	DAND5	0.057131	-0.13347	-
[2021]	SFRP1	--->	WNT7A	1.0317	-1.1063	+
[2022]	PAX5	<---	SENP1	-0.054444	0.050178	-
[2023]	CBL	--->	IRS1	0.16956	-0.066313	-
[2024]	IL23R	<---	STAT1	-0.19192	0.16257	-
[2025]	EP300	<---	WHSC1	-0.0046617	0.0040701	-
[2026]	GP9	--->	PIK3CB	0.022421	-0.053455	+
[2027]	ADCY8	<---	GNAI3	-0.63317	1.0384	-
[2028]	GPC4	--->	HS3ST3A1	-0.029045	-0.085212	-
[2029]	IL13RA1	<---	PTPN6	-0.26872	0.31912	+
[2030]	PLA2R1	--->	TGFB3	0.17799	-0.37709	+
[2031]	DKK2	--->	FZD5	0.3176	-0.35737	+
[2032]	ORAI1	<---	STIM2	-0.88237	0.15971	+
[2033]	ITGB1	<---	PRKX	-0.09775	0.14787	-
[2034]	ERBB3	<---	PRKD2	-0.057937	0.13298	-
[2035]	CD44	<---	COL4A5	-0.18268	0.0085485	+
[2036]	AKT2	<---	INSR	0.035184	0.14591	+
[2037]	PDK3	--->	RXRA	0.10013	-0.32848	-
[2038]	EDN1	<---	ESR1	-0.14879	-0.015388	+
[2039]	E2F6	--->	TCF12	0.45267	-0.59164	+
[2040]	IFNGR2	--->	JAK1	1.1771	-1.1167	-
[2041]	PLXNB2	<---	RAC2	-0.037168	0.1815	-
[2042]	PIAS3	--->	STAT5A	1.2392	-0.72187	+
[2043]	GNAI1	<---	MAPK11	-0.22953	0.50225	-
[2044]	CREB1	<---	RPS6KA3	-0.59606	0.7545	+
[2045]	GNAQ	<---	TRHR	-1.1303	1.1631	+
[2046]	CREB3L3	<---	INS	-0.24765	0.36121	-
[2047]	MYL12B	--->	RHOC	0.20073	-0.14858	-
[2048]	COL4A2	<---	GP6	-0.27286	0.25603	-
[2049]	CTNND1	<---	GDNF	-0.20942	0.0044379	+
[2050]	CHD2	--->	EZH2	0.020791	-0.082884	+

[2051]	CD3D	<---	CSK	-0.45391	0.63648	-
[2052]	ARHGEF7	<---	PAK6	-0.051206	0.17799	+
[2053]	CXCL10	--->	DNM1	0.022884	-0.019254	-
[2054]	GH1	<---	IRS1	0.023045	0.12681	-
[2055]	MAPK14	--->	USF1	0.99789	-0.87887	+
[2056]	AKT3	--->	PPP4C	-0.032241	-0.11483	+
[2057]	ATP1A4	<---	PRKACG	-0.09775	0.14787	+
[2058]	MAPK3	--->	SHC2	0.70518	-0.81557	+
[2059]	AKT3	<---	TCL1B	-1.4472	1.0764	+
[2060]	NOTCH3	<---	PSEN2	-0.70606	0.28518	+
[2061]	ARHGAP33	<---	RHOBTB2	-0.29676	0.17827	-
[2062]	ADORA2A	--->	FSHB	0.11779	-0.2522	-
[2063]	FGFR4	<---	IGF1	-0.40644	0.26408	+
[2064]	ITGB4	--->	TNXB	-0.028909	-0.031286	-
[2065]	FGD2	<---	RHOQ	-0.12937	0.14242	-
[2066]	EPHA8	--->	RHOA	0.039661	0.014723	+
[2067]	CXCL11	<---	CXCR4	-0.35895	0.21658	-
[2068]	GAB3	<---	PRKD1	-0.1056	0.2913	-
[2069]	ICAM1	<---	MYC	-0.10034	0.038571	+
[2070]	NRG3	<---	PRKCA	-0.29531	0.2389	-
[2071]	SLC2A4	<---	YWHAG	-0.85287	0.64882	+
[2072]	PIK3R1	<---	SH3KBP1	-0.20014	0.12565	-
[2073]	CACNA1A	<---	RHOA	-0.2309	0.29304	-
[2074]	INS	--->	PCSK1	0.036233	-0.079867	-
[2075]	STAT1	--->	THBS1	0.084672	-0.05189	+
[2076]	CCNA2	<---	TP53	-0.058346	0.10404	-
[2077]	FGF5	--->	FLT1	0.21774	-0.0015767	+
[2078]	SGK2	--->	WNK4	-0.11244	-0.11615	-
[2079]	GNB1	<---	PRKCA	-0.27923	0.22728	+
[2080]	ARHGAP44	<---	RHOD	-0.92575	0.61511	-
[2081]	FOXO4	--->	PCK1	0.5532	-0.58214	+
[2082]	CREM	<--->	FOS	0.22155	0.22155	-
[2083]	L1CAM	<---	RPS6KA4	-0.12605	0.076189	+
[2084]	EDN1	<---	STAT1	-0.31397	0.2569	+
[2085]	CBL	--->	SH3GL2	0.16088	-0.079462	+
[2086]	MAPK13	<---	RAC1	-0.20304	1.2491	+
[2087]	FGFR1	<---	FIGF	-0.61313	0.56088	+
[2088]	NKD1	<---	SAG	-0.37274	0.3133	-
[2089]	ITGAX	<---	SRC	-0.16015	0.14089	-
[2090]	CCR8	--->	CXCL10	0.21658	-0.35895	-
[2091]	RIMS1	<---	UNC13B	0.026968	0.098475	+
[2092]	CALM1	--->	CREB1	0.2998	-0.23478	+
[2093]	EIF2AK2	<--->	MAPK12	0.31014	0.31014	-
[2094]	FOXA1	--->	SMYD3	0.095014	0.024351	+
[2095]	EGR1	<---	IL12RB1	0.013308	0.03254	-
[2096]	CASP9	<---	EIF4E	-0.21179	0.019349	-
[2097]	CSF1	<---	NGFR	-0.28352	0.0081011	-
[2098]	EGR1	<---	TGFBR2	-0.41653	0.41168	-
[2099]	CEACAM1	--->	USF2	0.1127	-0.23041	-
[2100]	EPO	<---	NFKB1	-0.24715	0.24032	-
[2101]	FZD8	<---	GNB2	-0.19257	0.19185	-
[2102]	MAPK14	<---	NCK1	-0.31116	0.22669	+
[2103]	GNA11	<---	PTGER1	-1.1303	1.1631	+
[2104]	CD3E	<---	LAT	-0.099047	-0.0092662	-
[2105]	FGF22	--->	FGFR2	0.40218	-0.67149	-
[2106]	MAFK	--->	RBM39	0.1361	-0.090283	+
[2107]	CRKL	--->	ITGAL	0.018691	-0.085251	-
[2108]	ROCK2	<---	SH3GL2	-0.071767	0.079001	-
[2109]	LRP2	<---	PSEN2	-0.255	0.33599	+
[2110]	NFATC1	--->	PPP3R2	-0.039001	-0.22539	-
[2111]	GRK6	--->	SSTR4	0.33635	-0.28786	+
[2112]	KDR	--->	VAV1	0.66334	-0.062661	+
[2113]	NCL	--->	TCF12	0.16845	-0.11235	-
[2114]	HRAS	--->	IL5RA	0.11168	-0.075936	-
[2115]	AXIN1	--->	LRP6	0.19353	-0.15362	-
[2116]	GAB2	<---	MALT1	-0.52234	0.36685	+
[2117]	MAFK	--->	ZNF587	0.091076	0.039251	+
[2118]	CALML5	--->	PDE1B	0.40285	-0.39116	+
[2119]	GNA11	--->	PLCE1	0.0051395	-0.013272	+
[2120]	EP300	--->	RBM39	0.021187	-0.018539	+
[2121]	PIK3CG	<---	PRKCQ	-0.073525	0.055854	-
[2122]	IRS2	<---	SOCS4	-0.1721	0.068387	+
[2123]	FZD5	<---	GNAQ	-0.22143	0.24435	-
[2124]	MYC	--->	RBL1	-0.0082358	-0.077075	-
[2125]	MICALL1	<---	PTK2	0.041564	0.0884	+
[2126]	CX3CL1	<---	CXCR6	-0.35895	0.21658	-

[2127]	CD14	--->	TLR6	0.65283	-1.4467	+
[2128]	MAP2K1	<---	MAP3K5	-0.99422	1.146	-
[2129]	GNAO1	--->	RAC1	1.2694	-0.93465	+
[2130]	MYH11	<---	MYL12B	-0.10017	0.070498	+
[2131]	ITGA2B	<---	ITGB3	-0.041507	-0.0065623	+
[2132]	EPHA3	<---	LYN	-0.26002	0.33922	+
[2133]	IGHD	--->	PIK3R1	0.080373	-0.072507	+
[2134]	DRD2	--->	KCNJ9	0.11779	-0.2522	-
[2135]	ARHGAP25	<---	RAC2	-1.2097	0.92798	-
[2136]	CCNG2	--->	TP53	0.064257	-0.038691	-
[2137]	FGF7	--->	PDGFRB	0.48772	-0.57252	+
[2138]	CACNA1S	<---	PPP2R1A	-0.39164	0.37653	+
[2139]	CASP8	--->	TICAM2	-0.073061	-0.30384	-
[2140]	NOD1	<---	TNFAIP3	-0.17666	0.15817	+
[2141]	CTLA4	<---	LYN	-0.47931	0.63774	+
[2142]	FSHB	--->	INHBA	0.17799	-0.37709	-
[2143]	DRD2	--->	GNAO1	1.1631	-1.1303	+
[2144]	CDK4	--->	HDAC1	0.82707	-0.52917	+
[2145]	AKT1	--->	CSF2	0.14442	-0.21012	-
[2146]	PSEN1	--->	ZNF274	0.24598	-0.12157	+
[2147]	AMPH	--->	PIK3CB	0.01258	-0.024691	-
[2148]	FYN	--->	PIK3CB	0.084876	-0.06481	+
[2149]	DAXX	<---	MAPK8	-0.32851	0.12919	+
[2150]	ECT2	<---	RHOH	-0.012974	0.047696	-
[2151]	CAMK2G	--->	ITPR2	0.31376	-0.17692	-
[2152]	CDKN1A	--->	TFAP4	0.35967	-0.17058	-
[2153]	HOMER3	--->	PIK3CB	0.0038501	-0.0097611	+
[2154]	SMG1	--->	TAF1	0.086212	-0.11446	-
[2155]	CDC42	--->	GRIK1	0.54247	-0.38938	-
[2156]	FOXA1	--->	MYB	0.059212	-0.062176	+
[2157]	TGFB1	--->	USF2	-0.084233	-0.11402	-
[2158]	IFNLR1	<---	SOCS5	-0.65115	0.3746	+
[2159]	DVL2	<---	WNT9B	-0.54867	0.38842	+
[2160]	CREB3	<---	EP300	-0.010885	0.015595	-
[2161]	GRK5	--->	GRM6	0.80802	-0.62351	+
[2162]	RASGRP4	--->	RRAS	0.14935	-0.17952	+
[2163]	NEK6	--->	RAE1	0.54982	-0.31609	+
[2164]	ACTB	<---	PRKCB	-0.43362	0.33453	+
[2165]	PIK3C2G	--->	RAC1	-0.00086183	-0.016541	+
[2166]	BMI1	--->	MAPK14	0.35592	0.044099	-
[2167]	PPP4C	--->	YWHAZ	-0.03225	-0.31369	-
[2168]	SPI1	--->	USF1	0.43996	-0.52432	+
[2169]	ITGA8	<---	VWF	-0.05402	0.0049709	+
[2170]	RHOG	--->	WASF1	0.076038	-0.038582	+
[2171]	FOS	<---	RASGRP1	0.049686	0.23282	-
[2172]	LIFR	<---	PTPN6	-0.16409	0.19246	+
[2173]	ARHGAP1	<---	RHOT2	-0.35613	0.16234	-
[2174]	EGR1	--->	GDF15	0.1568	-0.0046291	+
[2175]	FGF1	<---	KRAS	-0.25726	0.38422	-
[2176]	ENG	<---	ETS1	-0.097441	0.05448	+
[2177]	CDKN1B	<---	PSMD7	-0.2145	0.31946	+
[2178]	NPNT	<---	SRF	-0.27707	0.23655	+
[2179]	CRKL	--->	ITGA7	0.032574	-0.078547	-
[2180]	GATA2	--->	ZNF792	0.075341	-0.0098617	+
[2181]	IL10RA	<---	IL26	-0.087402	0.20113	+
[2182]	CREB3L3	<---	PRKACG	-0.57905	1.0858	+
[2183]	DKK1	--->	WNT1	0.54164	-0.69755	+
[2184]	HNF4G	--->	PSMD1	-0.019218	-0.15739	+
[2185]	PLCZ1	<---	RGS4	-0.082548	0.03305	+
[2186]	EPHA3	<---	FGR	-0.26002	0.33922	+
[2187]	DAND5	--->	SPI1	0.036704	-0.072158	+
[2188]	MYC	<---	NCOA3	-0.045126	-0.024729	-
[2189]	CARD11	<---	TAB2	-0.27907	0.32874	+
[2190]	ARHGAP18	<---	RHOT2	-0.58534	0.26947	-
[2191]	MOB1A	<---	STK4	-1.1289	1.5226	+
[2192]	CTCF	--->	KLF1	-0.0066414	-0.044983	+
[2193]	RAF1	--->	YWHAB	-0.029863	-0.22693	-
[2194]	ITGB1	<---	PIK3R2	-0.026261	-0.023476	-
[2195]	FURIN	<---	PDGFA	-0.026453	-0.0086977	-
[2196]	CISH	--->	STAT5B	0.27422	-0.74698	-
[2197]	NTRK3	<---	SHC4	-0.080387	-0.019524	-
[2198]	CAMK2B	--->	MAP2K6	0.24715	0.164	+
[2199]	INPP5B	<---	RHOA	-0.92575	0.61511	-
[2200]	FGFR4	--->	MAPK1	0.071367	-0.33653	-
[2201]	CHN1	<---	RAC3	-0.4978	0.36255	-
[2202]	BCL2	<---	CREB3L2	-1.0502	0.64854	+

[2203]	ARRB2	--->	CXCL12	0.03281	-0.091858	+
[2204]	ID1	<---	STAT1	-0.46579	0.3797	+
[2205]	MEIS1	--->	TCF12	0.2773	-0.39532	-
[2206]	PDGFRB	--->	PIK3R2	0.15669	-0.055486	+
[2207]	RAC1	--->	WASF3	0.076038	-0.038582	+
[2208]	PPARG	--->	SMAD3	-0.025931	-0.076669	+
[2209]	KRAS	--->	NCAM1	0.31099	-0.36448	-
[2210]	EP300	--->	SLC2A4RG	0.0026	-0.0057334	+
[2211]	NR3C1	--->	ZNF595	0.18046	-0.21501	+
[2212]	FGF2	--->	FLT1	0.21774	-0.0015767	+
[2213]	KCNJ5	<---	SSTR4	-0.2522	0.11779	-
[2214]	CDK1	--->	NDC80	1.0858	-0.57905	+
[2215]	BCAR1	--->	RAP2C	0.21814	-0.11421	+
[2216]	MAPK3	<---	RRAS2	-0.20304	1.2491	+
[2217]	ADCY2	<---	EP300	-0.023514	0.023605	+
[2218]	RHOC	<--->	RHOG	0.1628	0.1628	-
[2219]	JUN	<---	POU1F1	-0.098653	0.038793	-
[2220]	FGF21	--->	FGFR1	1.1329	-1.3925	+
[2221]	IKZF3	--->	STAT3	0.25273	-0.13931	-
[2222]	GRIA1	<---	PRKACB	-1.006	1.5014	+
[2223]	ARHGEF17	<---	RHOU	-0.012974	0.047696	-
[2224]	GNAO1	--->	GPSM1	-0.0080287	-0.042052	-
[2225]	MMP8	<---	PRSS1	-0.18543	-0.0056809	+
[2226]	IQGAP1	--->	RAC1	0.062791	-0.028196	+
[2227]	KRAS	<---	RASGRF2	-0.52428	0.44606	+
[2228]	DAND5	<---	RELA	-0.38512	0.15634	-
[2229]	BMP4	<---	CHRD	-1.4437	0.57812	+
[2230]	MCF2	<---	RHOJ	-0.14219	0.16153	-
[2231]	SMYD2	--->	TCF7L2	0.14513	0.036901	-
[2232]	INS	<---	SORBS1	-0.17609	0.17146	-
[2233]	GNAI1	<---	RGS12	-0.022601	0.029368	+
[2234]	DVL1	<---	WNT8B	-0.54867	0.38842	+
[2235]	APC	--->	WNT3A	0.0022317	-0.020073	-
[2236]	ID1	<---	RAP1B	-0.9119	1.1173	+
[2237]	EBI3	--->	EGR1	0.03254	0.013308	-
[2238]	EPOR	<---	JAK1	-0.23677	0.10182	-
[2239]	ADAM12	--->	EGF	0.10995	-0.11663	+
[2240]	CRTC2	--->	SIK3	0.23894	-0.32874	-
[2241]	HCLS1	<---	SRC	0.0056949	0.31785	+
[2242]	RHOA	<---	YES1	0.16613	1.0122	+
[2243]	CREBBP	<---	TEK	-0.0072822	0.00099787	-
[2244]	GNB3	<---	PRKX	-0.28574	0.38931	+
[2245]	HOMER2	--->	PIK3R1	0.073576	-0.12217	+
[2246]	MAPK1	<---	RHOA	-0.20304	1.2491	+
[2247]	AKT3	<---	HSP90B1	-0.32359	0.3448	+
[2248]	GRIA4	<---	PPP1CA	-0.67094	0.67498	+
[2249]	EP400	--->	TP53	0.074126	-0.072897	-
[2250]	ADCY1	<---	ADRB2	-0.62364	0.57833	+
[2251]	DAPP1	<---	RAC2	0.14154	0.33406	-
[2252]	CDH1	<---	SNX1	-0.40965	0.085277	+
[2253]	ADAM17	--->	NRG4	0.20756	-0.29314	+
[2254]	MMP1	--->	RXRB	-0.1147	-0.15215	-
[2255]	IRAK2	--->	MAP2K4	0.73444	-0.20293	+
[2256]	ACTC1	<---	MMP23A	-0.43788	0.38294	+
[2257]	COL6A1	--->	ITGA6	-0.0092525	-0.030111	+
[2258]	FGF21	<---	NGFR	-0.47081	0.24157	-
[2259]	RB1	<---	SPI1	-0.25983	0.14622	-
[2260]	FOXA1	--->	MTF2	0.27809	-0.34674	+
[2261]	CTBP1	<---	MECOM	-0.22767	0.31039	+
[2262]	PTK2	<---	PTPRA	-1.1857	0.40365	-
[2263]	GATA1	--->	KLF1	0.16874	-0.093323	+
[2264]	KRAS	<---	YWHAE	-0.25073	0.15053	-
[2265]	PIK3CA	<---	PRKD2	-0.052369	0.069093	-
[2266]	ACTR2	<---	MMP7	-0.29263	0.085904	+
[2267]	MXI1	<---	ZNF653	-0.059615	0.19618	-
[2268]	CTNNA1	<---	SRC	-0.21532	0.5503	-
[2269]	ITK	--->	PIK3CB	0.026127	-0.010992	-
[2270]	CGREF1	--->	MYC	0.16137	-0.37024	-
[2271]	CTBP2	<---	PAX9	-0.079535	-0.045311	-
[2272]	MAX	<---	SP1	-0.12327	0.19953	+
[2273]	PIK3CG	<---	TRAF3	-0.11948	0.15129	+
[2274]	GNB2	<---	PIK3CB	-0.012551	0.008521	-
[2275]	LEF1	<---	SMAD3	-1.2424	1.1041	+
[2276]	ARHGAP26	<---	RND1	-0.65972	0.48549	-
[2277]	KRAS	--->	PTK2	-0.084837	-0.14524	-
[2278]	CDK1	--->	PSME4	0.57437	-0.5963	-

[2279]	EGR1	<---	EPO	-0.13204	0.22562	-
[2280]	BCL2L1	<---	PRKAR2A	-1.0163	0.7413	+
[2281]	EP300	--->	HDAC7	0.021603	-0.018377	+
[2282]	ACTA2	<---	MMP27	-0.29263	0.085904	+
[2283]	CTNNB1	<---	RET	-0.2282	0.14424	+
[2284]	RHOU	<---	SOS1	-0.33772	0.48674	+
[2285]	PDGFB	--->	STAT5A	0.2715	-0.20049	+
[2286]	CCR10	--->	PF4	0.21658	-0.35895	-
[2287]	G6PC3	<---	PRKAG2	-0.20283	0.012951	+
[2288]	CDKN1A	<---	PSMD1	-0.020988	0.10014	+
[2289]	C2	--->	IGKV1-5	-0.02225	-0.02389	-
[2290]	NET1	<---	RHOC	-0.23262	0.26814	-
[2291]	FGFR4	<---	VEGFC	-0.38721	0.2003	+
[2292]	CDC42	--->	RPS6KB1	0.65574	-0.39233	+
[2293]	FOXO4	--->	GADD45A	0.36388	-0.62421	+
[2294]	FOXO4	<---	PRKAA2	-0.22085	0.2499	+
[2295]	CHUK	<---	TNFSF10	-0.82132	0.98959	+
[2296]	CNTFR	--->	SOCS1	0.17881	-0.34174	-
[2297]	BHLHE40	<---	ZNF175	-0.38032	0.36387	-
[2298]	E2F1	--->	HES4	0.4378	-0.61701	+
[2299]	ADAM17	--->	DLL1	0.07458	-0.10897	+
[2300]	CDK5	<---	EPHA5	-0.4085	0.3522	+
[2301]	NPY	--->	OXTR	0.89835	-1.3205	+
[2302]	MAPK1	<---	PPP2R1B	-0.25679	0.26693	+
[2303]	CDKN1B	--->	TFDP2	0.069711	0.016842	+
[2304]	CAPN11	<---	HTT	-0.21113	0.18093	-
[2305]	MAP2K6	<---	RAP1A	-0.20304	1.2491	+
[2306]	RHOQ	--->	TIAM2	0.20928	-0.13672	-
[2307]	NEK9	--->	NUP37	0.54982	-0.31609	+
[2308]	AKT2	<---	PLCG2	0.010979	0.019996	+
[2309]	FN1	<---	ITGA5	-0.10408	-0.047194	-
[2310]	DNM1	<---	GIPC1	-0.027372	0.056251	-
[2311]	CSNK1E	--->	FZD2	0.5614	-0.51612	-
[2312]	NOTCH1	<---	PSEN2	-1.1415	0.45108	+
[2313]	CREBBP	--->	PPARGC1B	0.021187	-0.018539	+
[2314]	BCL3	<---	ZNF595	-0.064911	0.62238	-
[2315]	IL10RA	<---	IL19	-0.087402	0.20113	+
[2316]	FCER1G	<---	LAT	-0.061175	-0.0067198	-
[2317]	PIK3R5	<---	PLD2	-0.16007	0.13645	-
[2318]	ADAM10	--->	NOTCH2	0.06241	-0.054476	+
[2319]	CX3CL1	<---	XCR1	-0.35895	0.21658	-
[2320]	GABBR1	<---	GNAI3	-0.15623	0.24033	-
[2321]	COL9A1	--->	PPIB	-0.024575	-0.068802	-
[2322]	ADAM10	--->	COL17A1	0.02236	-0.072336	+
[2323]	GATA2	<---	GP1BB	-0.36538	0.16947	-
[2324]	E2F4	<---	KIF2C	-0.043794	-0.029425	-
[2325]	PIK3CA	<---	TLR3	-0.046452	0.0057294	+
[2326]	CSF2RB	--->	STAT5A	0.078645	-0.047643	+
[2327]	APPL1	<---	ESR1	-0.062336	0.03178	+
[2328]	ITGA6	<---	RPS6KB1	-0.19561	0.059423	-
[2329]	CDC42	--->	GRIK2	0.54247	-0.38938	-
[2330]	GNB1	--->	WNT5B	0.19155	-0.26814	-
[2331]	HRAS	<---	PTPRA	-0.61954	0.5927	+
[2332]	MMP1	<---	PLG	-0.1166	-0.013996	+
[2333]	RHOV	<---	SOS1	-0.33772	0.48674	+
[2334]	IKBKG	<---	RIPK2	-0.17793	0.13116	+
[2335]	PIAS4	--->	STAT6	0.75054	-0.43894	+
[2336]	ITGB1	--->	PIK3CB	-0.0036365	-0.0057548	+
[2337]	PRKCH	--->	RAF1	0.18571	-0.23412	+
[2338]	GNAI3	<---	LCK	-0.44236	0.67161	-
[2339]	DLG3	<---	WWTR1	-0.05901	0.042003	-
[2340]	C3	--->	CFB	0.1329	-0.21082	-
[2341]	IL5RA	<---	PTPN6	-0.38053	0.50251	+
[2342]	RXRA	<---	SFRP1	-0.28313	0.10384	-
[2343]	ADORA2B	--->	KCNJ9	0.11779	-0.2522	-
[2344]	KRAS	--->	RALGDS	1.4276	-1.2366	+
[2345]	NGFR	--->	PIK3R5	0.32324	-0.65244	+
[2346]	TAF5L	--->	ZNF263	0.49296	-0.13732	-
[2347]	MYL5	--->	ROCK2	0.24001	-0.20673	-
[2348]	RGL1	<---	RRAS	-1.2366	1.4276	+
[2349]	CEBPA	--->	SERPINC1	0.33364	-0.53505	+
[2350]	CACNA1F	<---	PPP2R3B	-0.36974	0.36606	+
[2351]	EP300	<---	STAT1	-0.018478	0.010999	-
[2352]	ATP1B1	--->	PIK3CA	-0.0050831	-0.010841	-
[2353]	MAPK3	--->	RPS6KA4	0.9519	-1.129	+
[2354]	APOA1	--->	LDLR	0.20747	-0.15861	-

```
[2355]  NGFR    --->    TP53    0.11787 -0.19685        +
[2356]  CALM1   --->    CAMK2A  0.19292 -0.081704       -
[2357]  G6PC    <---    PRKAG2  -0.20283        0.012951        +
[2358]  CHEK1   --->    UBE2T   0.24471 -0.086834       +
[2359]  PPARGC1A        <---    PRKAG3  -0.911  0.97387 +
[2360]  CCR10   <---    GRK5    -0.28786        0.33635 +
[2361]  NFKB1   --->    VEGFA   0.13181 -0.13363        +
[2362]  AEBP2   --->    MYC     0.017786        -0.08008        -
[2363]  BRCA1   --->    EP300   0.0125  -0.013392       -
[2364]  ANGPT1  <---    FOXO1   -0.43599        0.34955 +
[2365]  PSME4   <---    SMURF2  -0.35498        0.21231 -
[2366]  F2R     --->    GZMA    0.11779 -0.2522 -
[2367]  LCK     --->    STAT5B  0.43807 -0.74539        +
[2368]  COL27A1 --->    ITGA2B  -0.013036       -0.10803        +
[2369]  MYD88   <---    TRAF6   -0.043927       0.11956 -
[2370]  GRK1    --->    XCR1    0.33635 -0.28786        +
[2371]  PRKCQ   --->    SRC     0.26845 0.097057        +
[2372]  COL11A1 --->    ITGA8   -0.013036       -0.10803        +
[2373]  CBLC    --->    TYK2    0.76823 -0.20151        +
[2374]  APP     <---    NFKB2   -0.025843       0.018608        -
[2375]  GNB2    <---    PRKACA  -0.31609        0.54982 +
[2376]  IGSF5   <---    PPP2CB  -0.23928        0.38829 +
[2377]  AGAP2   <---    PIK3CB  -0.0056985      0.012149        -
[2378]  ARHGAP24        <---    RHOF    -0.49576        0.47865 -
[2379]  CUL5    <---    ERBB2   -0.08975        0.095965        -
[2380]  MAFK    --->    SERPINA10       0.10065 -0.31094        +
[2381]  FGF1    <---    NRAS    -0.15714        0.23194 -
[2382]  EP300   --->    ZNF257  0.01223 -0.0076085      +
[2383]  LAMB2   --->    SV2A    0.20521 -0.20511        +
[2384]  EIF2AK2 <---    PTPN2   -0.75249        0.74392 -
[2385]  COL4A4  --->    ITGA8   -0.013036       -0.10803        +
[2386]  PSME4   <---    SKP1    -0.052638       -0.0083864      -
[2387]  BRCA1   <---    GADD45A -0.1036 -0.037705       -
[2388]  AKT2    --->    CREB3L3 0.82132 -1.3257 +
[2389]  GRB2    <---    LCP2    -0.050615       0.40601 -
[2390]  FYN     --->    SHC3    0.48646 -0.49397        +
[2391]  CALM1   --->    CAMK2B  0.19292 -0.081704       -
[2392]  ITGA3   <---    LAMA3   -0.030111       -0.0092525      +
[2393]  GNAO1   <---    KCNJ6   -0.011498       0.05262 -
[2394]  TAB2    <---    TRAF5   -0.63372        1.3819  +
[2395]  PRKX    --->    SCN5A   0.61419 -0.44517        +
[2396]  ID2     <---    SMAD9   -0.34215        0.18982 +
[2397]  EPB41L1 --->    NGF     0.10576 -0.1637 -
[2398]  COL27A1 <---    LEPRE1  -0.18291        0.065305        +
[2399]  CD3E    --->    CD80    -0.0075134      -0.047639       -
[2400]  AGRN    --->    LRP2    0.061778        -0.047155       -
[2401]  EPX     --->    SMAD4   -0.050142       -0.10303        -
[2402]  CDK9    --->    SMAD2   0.88243 -0.89987        +
[2403]  FZD7    <---    WNT10B  -0.78209        0.63734 +
[2404]  CAMK2G  --->    SCN7A   0.43397 -0.32993        +
[2405]  NFKBIA  <---    SYK     -0.30187        0.13473 +
[2406]  MAPK9   --->    NFKB1   0.20986 -0.059594       +
[2407]  IL4R    --->    STAT3   0.23476 -0.17484        -
[2408]  FYN     --->    KLRD1   0.63648 -0.45391        +
[2409]  COL24A1 <---    PCOLCE  -0.32363        0.21278 +
[2410]  ARAP2   <---    RHOU    -0.48615        0.36733 -
[2411]  MAPK1   --->    PTPN6   1.0948  -1.0232 -
[2412]  C1R     --->    JUN     0.040859        -0.06886        -
[2413]  HES4    <---    ZBTB7A  -0.23178        0.2196  +
[2414]  CACNA1D <---    CAMK2A  -0.26963        0.34735 +
[2415]  IL12RB2 --->    STAT3   0.1409  -0.099294       +
[2416]  FCER1A  --->    PPARG   0.031418        -0.11157        -
[2417]  E2F4    <---    MYBL2   -0.092111       0.080065        +
[2418]  CIITA   --->    MAFK    0.26064 -0.31071        -
[2419]  BMP4    <---    TAB1    -0.69176        0.63763 -
[2420]  ACTB    <---    RHOJ    -0.51168        0.52339 +
[2421]  IL18    --->    PYCARD  0.43154 -0.50906        -
[2422]  PRLR    --->    STAT3   0.23476 -0.17484        -
[2423]  CD3D    <---    PTPN11  -0.29307        0.36484 -
[2424]  LRIG1   --->    NRG1    -0.045748       -0.057079       +
[2425]  GRK4    --->    SSTR1   0.33635 -0.28786        +
[2426]  CCL11   <---    CCR6    -0.35895        0.21658 -
[2427]  FIGF    --->    PIK3CA  -0.0018298      -0.01412        +
[2428]  CSNK1G1 --->    DVL1    0.31547 -0.38065        +
[2429]  PTPN6   --->    VAV3    0.050213        -0.1064 +
[2430]  MAPK8   --->    ZNF274  1.0991  -0.67282        -
```

[2431]	FGF13	<---	TEK	-0.05268	0.034632	-
[2432]	EP300	--->	VAV1	0.00094793	-0.00049258	+
[2433]	SFRP2	--->	WNT10B	1.0317	-1.1063	+
[2434]	CBL	--->	CD19	0.2466	-0.10039	+
[2435]	CBLB	--->	IL2RB	0.28248	-0.14259	+
[2436]	F11R	--->	PIK3R1	0.21013	-0.027316	+
[2437]	FGF1	--->	FGFR2	0.40218	-0.67149	+
[2438]	GMIP	<---	RHOG	-1.011	0.68428	-
[2439]	IRS1	--->	PIK3R1	-0.026224	-0.083368	+
[2440]	HRAS	<---	SOCS3	-0.093407	0.044639	+
[2441]	IL12B	--->	IL13RA1	-0.19263	-0.20337	+
[2442]	SRSF7	--->	TBP	0.083732	-0.042563	-
[2443]	CD8A	--->	JUN	0.14534	-0.21548	+
[2444]	NPAS2	<---	NR1D1	-0.14396	0.10125	-
[2445]	MAP3K2	<---	RAC1	-0.17545	1.1267	+
[2446]	AKT2	--->	RPS6KB2	0.67325	-0.79394	+
[2447]	PIK3CB	--->	SPRY2	0.002984	-0.013928	+
[2448]	CASP7	--->	ITGB4	0.043126	-0.1073	+
[2449]	GNB5	--->	SCN1A	0.07915	-0.054128	+
[2450]	GNB2	--->	RASGRF1	1.2179	-1.2056	+
[2451]	EPOR	<---	PTPN6	-0.26697	0.28395	-
[2452]	MAPK10	<---	TGFB1	-0.0067309	0.13651	+
[2453]	IL10	--->	STAT6	0.26825	-0.19721	-
[2454]	MYO1C	<---	RAB11A	-0.038582	0.076038	+
[2455]	MLXIPL	<---	PRKACA	-0.72712	1.016	+
[2456]	JAK1	<---	SOCS5	-0.73315	0.53539	+
[2457]	SMAD4	--->	TNC	0.0033454	-0.089303	+
[2458]	CRKL	--->	PIK3CA	0.010761	-0.019381	+
[2459]	INHBE	--->	TGFB3	-0.050062	-0.38124	+
[2460]	ITGB4	<---	LAMC2	-0.026399	0.0074885	+
[2461]	NFKB1	--->	PIGR	0.23794	-0.24069	+
[2462]	ARHGAP40	<---	RHOV	-1.5245	1.016	-
[2463]	COL3A1	--->	ITGA3	0.010009	-0.08725	+
[2464]	COL12A1	<---	MMP12	-0.064017	-0.031269	+
[2465]	MYD88	--->	NGF	0.69759	-0.77157	+
[2466]	CRLF2	<---	JAK2	-0.23741	0.19744	-
[2467]	COL18A1	--->	PPIB	-0.0030457	-0.073832	-
[2468]	BMPR1B	--->	NLK	0.17987	-0.35209	+
[2469]	CBL	--->	IL2RG	0.11039	-0.066545	-
[2470]	CCL21	<---	CCR7	-0.35895	0.21658	-
[2471]	APOA1	<---	EP300	-0.0041449	-0.00068954	+
[2472]	BCL2	<---	EGR1	-0.59901	0.47345	+
[2473]	PDE4D	<---	PRKACB	-0.95055	1.4803	+
[2474]	ITGB3	<---	KDR	-0.016584	0.12094	-
[2475]	BCAR1	--->	ITGA2	0.041847	-0.077667	-
[2476]	MAPK14	--->	PPARGC1A	1.2133	-0.47902	+
[2477]	ACTG2	<---	MMP25	-0.29263	0.085904	+
[2478]	ANAPC2	--->	CDKN1B	0.2477	-0.16739	-
[2479]	CTNNA1	--->	IQGAP1	0.11911	-0.12202	-
[2480]	COL6A6	--->	ITGA6	-0.0067115	-0.019363	+
[2481]	COL5A2	--->	ITGB7	0.0059092	-0.05696	+
[2482]	RHOG	--->	STARD8	1.016	-1.5245	-
[2483]	KIT	<---	VEGFB	-0.099588	0.12565	+
[2484]	GNAI1	<---	MAPK3	-0.22953	0.50225	-
[2485]	FYN	--->	PIK3CG	0.14175	-0.10504	+
[2486]	SIRT1	--->	TFDP2	0.075154	-0.095433	-
[2487]	DLC1	<---	RHOV	-0.50393	0.10338	-
[2488]	ATF2	--->	HIST1H2BN	0.048146	0.0081543	+
[2489]	DVL2	<---	WNT11	-0.54867	0.38842	+
[2490]	SPI1	--->	WAS	0.032903	-0.089878	+
[2491]	MAP2K3	<---	TLR3	-0.16162	0.015687	+
[2492]	RALBP1	<---	RHOJ	-1.5245	1.016	-
[2493]	PLN	<---	PPP1CA	-0.5028	0.29452	+
[2494]	KIT	--->	RPS6KB1	0.23742	-0.1758	+
[2495]	AKT1	--->	PIK3CG	0.028477	-0.051515	-
[2496]	GHRL	--->	USF1	0.58343	-0.6546	-
[2497]	CSF2RB	<---	SOCS3	-0.095304	0.022627	+
[2498]	MYC	<---	NFKBIA	-0.2578	0.033283	-
[2499]	CASP2	<---	MAP4K4	-1.0164	1.1279	-
[2500]	PDPK1	<---	TGFBR2	-0.38998	0.51453	-
[2501]	IRS1	--->	PIK3CD	0.016648	-0.00088766	+
[2502]	HOMER3	--->	PIK3CA	0.0038501	-0.0097611	+
[2503]	EP300	<---	IRS1	0.0018074	0.0035183	-
[2504]	CCNB3	<---	GADD45G	-0.67465	0.65424	+
[2505]	CX3CL1	<---	CX3CR1	-0.35895	0.21658	-
[2506]	MAP2K4	<--->	MAPK11	0.31014	0.31014	-

[2507]	COL4A1	--->	PPIB	-0.024575	-0.068802	-
[2508]	GRB2	<---	YWHAH	-0.071301	-0.047712	+
[2509]	CBX3	--->	CCNH	0.2481	-0.31692	+
[2510]	FOXO4	<---	SETD7	-0.30041	0.66262	+
[2511]	E2F1	<---	LHX2	-0.16404	0.19535	-
[2512]	GNAI1	<---	KCNJ9	-0.011498	0.05262	-
[2513]	CRK	--->	RAP1B	0.43868	-0.057401	-
[2514]	ADAM17	<---	PRKCQ	-0.087066	0.033769	+
[2515]	COL4A5	<---	LEPRE1	-0.18291	0.065305	+
[2516]	LTBP4	--->	ZBTB7A	0.030996	-0.05695	-
[2517]	ADRBK2	--->	DRD3	0.25327	-0.21301	+
[2518]	PAX8	<---	PRKACG	-0.15935	0.24561	+
[2519]	CRK	--->	PXN	0.098826	-0.1839	+
[2520]	CXCR1	<---	GRK5	-0.28786	0.33635	+
[2521]	HDAC2	<---	SMAD3	-0.21285	0.15191	-
[2522]	ITGB1	<---	MAPK8	-0.09775	0.14787	-
[2523]	ANAPC2	<---	PPP3CA	-0.39733	0.17898	+
[2524]	PTPN6	--->	TRBV12-3	0.37051	-0.30848	+
[2525]	ELF3	<---	SMARCA4	-0.033126	-0.00089051	+
[2526]	LPAR1	--->	PTK2B	-0.05393	-0.18014	+
[2527]	MAPK3	<---	TNFRSF10A	-0.22533	-0.1429	+
[2528]	GNB2	<---	HTR1A	-0.92616	1.1575	+
[2529]	CTBP2	--->	NKX2-1	0.04296	-0.16727	+
[2530]	E2F5	<---	SMAD4	-0.30541	0.36653	+
[2531]	CNTF	--->	MPL	0.056231	-0.11334	+
[2532]	NFKB1	<---	TLR6	-1.1204	1.0762	-
[2533]	PCK2	<---	PRKAG2	-0.22182	0.067346	+
[2534]	CRK	--->	ITGA6	0.032574	-0.078547	-
[2535]	PRKCG	--->	STAT1	-0.025941	-0.050583	-
[2536]	CTBP2	<---	LHX5	-0.11766	0.087559	-
[2537]	CISH	<---	NTRK1	-0.19861	-0.19248	-
[2538]	FOXG1	<--->	FOXO3	0.41711	0.41711	-
[2539]	COL9A1	<---	PTK2B	-0.11304	-0.10314	+
[2540]	CACNA1S	<---	GNB4	-0.14673	0.18744	+
[2541]	GNA12	--->	PLA2G4C	0.034723	-0.073024	+
[2542]	ITGA1	<---	MYL12B	-0.1321	0.025881	+
[2543]	PPP1R1A	<---	PRKX	-0.92753	1.2659	+
[2544]	FZD8	<---	GNB3	-0.12321	0.10912	-
[2545]	ANGPT2	<---	MET	-0.26054	0.17274	-
[2546]	CCL1	<---	XCR1	-0.35895	0.21658	-
[2547]	CD79B	<---	PTPRC	-0.13309	0.089475	+
[2548]	RXRA	<---	TNF	-0.26704	-0.10096	+
[2549]	FGF7	--->	IGF1R	0.25346	-0.0044024	+
[2550]	ITGA8	<---	LAMA5	-0.041507	-0.0065623	+
[2551]	AR	<---	CCNG1	-0.42506	0.36565	-
[2552]	CCL22	<---	CCR10	-0.35895	0.21658	-
[2553]	PDPK1	<---	TAB2	0.11824	0.13488	+
[2554]	ITGA9	--->	TNC	-0.015417	-0.048468	-
[2555]	APOA1	<---	PPARD	-0.091347	-0.010433	+
[2556]	IL12RB1	<---	NFKB2	-0.37463	0.31878	-
[2557]	KRAS	<---	SPTAN1	-0.048099	0.21387	+
[2558]	FGF17	<---	MAPK1	-0.47959	0.71493	+
[2559]	PPARA	--->	SREBF1	0.27747	-0.4408	+
[2560]	NRAS	--->	SPTB	0.026206	-0.01596	-
[2561]	ADRA2C	<---	GRK1	-0.28786	0.33635	+
[2562]	PAFAH1B1	<---	RHOA	-0.21926	0.18651	-
[2563]	EGR1	<---	ELK1	-0.15205	0.28002	-
[2564]	ACTG1	<---	MMP24	-0.29263	0.085904	+
[2565]	ACVR1B	<---	BAMBI	-1.3349	1.2775	+
[2566]	UBE2D1	<--->	UBE2E1	0.18452	0.18452	-
[2567]	MET	--->	PDGFB	0.077225	-0.11958	-
[2568]	BRAP	--->	KSR1	0.87466	-0.553	+
[2569]	SRPK1	--->	SRSF2	1.2133	-0.47902	+
[2570]	COL3A1	--->	ITGA9	0.0052169	-0.090529	+
[2571]	GNAI3	--->	GPSM2	-0.0080287	-0.042052	-
[2572]	NR1H3	--->	TAF1	-0.015388	-0.14879	-
[2573]	CEBPB	<---	PRMT1	-0.50568	0.43102	-
[2574]	B3GALTL	--->	CFP	-0.011484	-0.071046	+
[2575]	RELB	--->	TYK2	-0.11509	-0.25929	-
[2576]	BUB1	--->	TBP	0.81266	-0.67809	-
[2577]	FZD2	<---	WNT8B	-0.78209	0.63734	+
[2578]	FAM13B	<---	RHOA	-1.5245	1.016	-
[2579]	DSG3	--->	MEF2A	-0.060937	-0.13698	-
[2580]	GRIA4	<---	PRKACG	-1.006	1.5014	+
[2581]	ARHGEF9	<---	RHOB	0.091479	0.13323	-
[2582]	JAK3	--->	KRAS	0.579	-0.13016	+

[2583]	IGF1R	<---	MAPK1	0.083131	0.1759	-
[2584]	BTK	<---	LCK	-0.5383	0.92083	+
[2585]	MAPK11	<---	PAK3	-1.2674	1.5523	+
[2586]	FGF2	<---	GRB2	-0.29505	0.31381	-
[2587]	ACTA1	<---	MMP15	-0.29263	0.085904	+
[2588]	GP9	--->	PROC	0.17287	-0.24288	-
[2589]	BRAF	--->	PPP2CA	0.0693	-0.12371	-
[2590]	NFKB1	--->	RARA	0.1148	-0.14103	+
[2591]	FGF10	--->	FLT1	0.21774	-0.0015767	+
[2592]	ATF1	<--->	FOS	0.22155	0.22155	-
[2593]	CUL3	--->	DZIP1	0.18764	-0.25817	-
[2594]	BLNK	<---	VAV2	-0.037022	-0.0049551	-
[2595]	ELAVL1	<---	PRKAG1	-0.911	0.97387	+
[2596]	ARHGAP33	<---	RHOV	-0.48747	0.29572	-
[2597]	HEPACAM	--->	MYC	0.091782	-0.22648	-
[2598]	SFRP1	--->	WNT5A	1.0317	-1.1063	+
[2599]	CRTC2	<---	PRKAG3	-1.0463	1.4287	+
[2600]	CCL15	<---	CXCR5	-0.35895	0.21658	-
[2601]	ESR1	--->	SERPINB9	0.21298	-0.44731	+
[2602]	APOA4	--->	LRP1	0.28825	-0.24037	-
[2603]	PRLR	--->	STAT5B	0.23476	-0.17484	-
[2604]	CBX6	<---	MAPK14	-0.22919	0.35645	+
[2605]	PRKDC	--->	TP53	0.031907	-0.022247	+
[2606]	GNA15	<---	PTGFR	-1.1303	1.1631	+
[2607]	DCC	<---	TRIO	-0.0098693	-0.00028444	-
[2608]	FLT3	--->	STAT3	0.19582	-0.19819	+
[2609]	EP300	--->	HEYL	0.022073	-0.031481	+
[2610]	SMAD2	<---	UBE2D3	-0.20131	0.05544	+
[2611]	MPL	<---	SOCS5	-0.10625	0.079572	+
[2612]	CIAO1	--->	WT1	0.34981	-0.05406	-
[2613]	CD79B	--->	NTRK1	0.066193	-0.26011	+
[2614]	CSNK1G1	--->	YWHAE	0.047412	-0.04465	+
[2615]	IL12RB1	--->	SHC1	-0.01955	-0.11936	+
[2616]	KIR2DL5A	<---	PTPN6	-0.33664	0.18045	-
[2617]	MAPK11	--->	MEF2D	1.0903	-1.4506	+
[2618]	TP53	<---	WRN	-0.086478	0.092486	-
[2619]	MYH10	--->	ROCK2	0.042533	-0.040702	-
[2620]	ITGB5	<---	LAMB2	-0.011824	0.005707	+
[2621]	CREBBP	<---	NCOR1	-0.0074211	0.0056463	+
[2622]	IFNB1	--->	IFNLR1	0.46511	-0.53673	+
[2623]	SPI1	--->	USF2	0.43996	-0.52432	-
[2624]	CXCR4	--->	USF1	0.60063	-0.73364	-
[2625]	ERBB2	--->	PDGFA	0.13872	-0.12568	-
[2626]	DHX58	--->	STAT2	0.27914	-0.19666	-
[2627]	CSF2	<---	JAK2	-0.13315	0.05571	+
[2628]	POU4F1	<---	TP53BP2	-0.4162	0.44265	-
[2629]	DOCK2	<---	HCK	-0.098032	0.14215	+
[2630]	HNF4A	<---	SRC	-0.2721	0.34018	-
[2631]	IGKV1-5	<---	RAC1	-0.063985	0.085383	-
[2632]	CRKL	--->	YY1	0.20763	-0.15839	-
[2633]	PRKCE	--->	TJP1	0.14343	-0.1592	+
[2634]	IGF1R	<---	ITGAV	-0.0089829	0.075974	+
[2635]	ARAP1	<---	RHOG	-0.48615	0.36733	-
[2636]	CNOT6L	--->	PABPC1	0.32043	-0.37316	+
[2637]	CEBPB	<---	XRCC5	-0.17449	0.12202	-
[2638]	PDGFRB	--->	RAC1	0.48853	-0.2264	-
[2639]	GNB1	<---	MAP2K3	-0.19093	0.20105	-
[2640]	IL12RB1	<---	TYK2	-0.62127	0.29769	-
[2641]	ACTB	<---	PTK2B	-0.28708	0.061438	+
[2642]	ITGA1	<---	THBS3	-0.029329	-0.0096546	+
[2643]	CTNNB1	<---	MMP19	-0.26058	0.11034	+
[2644]	ADCY9	<---	CAMK2G	-0.44346	0.58749	+
[2645]	MAP4K4	--->	TRADD	0.24439	-0.21179	-
[2646]	CSNK1A1L	--->	WNT8A	0.83912	-0.78694	-
[2647]	FLT1	<---	VEGFA	-0.60455	0.76698	+
[2648]	F2R	--->	PLCB1	0.081663	-0.121	+
[2649]	DUSP14	--->	MAPK10	1.4184	-1.4202	+
[2650]	IL23R	--->	THPO	-0.022819	-0.038764	-
[2651]	CAMK4	--->	PDE4D	1.4803	-0.95055	+
[2652]	KIR3DL2	<---	PTPN11	-0.55321	0.29932	-
[2653]	IL23A	<---	NFKB1	-0.23502	0.23585	-
[2654]	PDHX	--->	RXRA	-0.10061	-0.25233	-
[2655]	FGF7	--->	FGFR1	1.1329	-1.3925	-
[2656]	CBL	--->	MET	0.3135	-0.10542	+
[2657]	IL6R	<---	PTPN11	-0.25507	0.22314	-
[2658]	NUP43	--->	RNPS1	0.31391	-0.17132	+

[2659] GLI3 ---> WNT6 0.86373 -1.47 +
[2660] MXI1 <--- SPI1 -0.52432 0.43996 -
[2661] DAND5 ---> GATA3 0.036704 -0.072158 +
[2662] CNTF ---> CRLF2 0.094523 -0.18506 +
[2663] PPP1R1A <--- PRKACG -0.92753 1.2659 +
[2664] CRKL ---> RAC2 0.43868 -0.057401 +
[2665] JUN <--- MAPK10 -0.47491 0.66358 +
[2666] GRB2 <--- LYN 0.028419 0.41943 +
[2667] DEPDC1B <--- RHOF -0.93788 0.64026 -
[2668] ACTA1 <--- AKT1 -0.29841 0.18175 +
[2669] IFNGR1 ---> JAK2 0.016586 -0.2096 -
[2670] MXD4 <--- PBX3 -0.2366 0.10664 +
[2671] DEPDC7 ---> RHOT2 0.26799 -0.26312 +
[2672] PDGFD ---> PLAT 0.10703 -0.15627 -
[2673] BATF ---> CCNK 0.40828 -0.24261 +
[2674] DAPP1 <--- LYN -0.46872 0.73349 +
[2675] BCR <--- RHOT2 -0.047245 -0.014168 -
[2676] JAK2 ---> PRKACA 0.43202 -1.1344 -
[2677] ADRBK2 ---> CXCR1 0.25327 -0.21301 +
[2678] NFKB1 ---> SIRT2 0.23585 -0.23502 +
[2679] EXOC8 <--- MYO1C -0.24915 0.28675 +
[2680] DKK2 ---> FZD8 0.3176 -0.35737 +
[2681] HNRNPDL ---> MAX 0.10107 -0.069244 -
[2682] EP300 <--- FST -0.0076683 0.0012761 -
[2683] NOD1 <--- UBE2V1 -0.33022 0.31802 +
[2684] E2F5 ---> MYC 0.3354 -0.7861 +
[2685] COL5A2 ---> ITGA3 0.010009 -0.08725 +
[2686] BCAR1 ---> GNAI1 0.21576 -0.17722 -
[2687] PIK3CA <--- VEGFC -0.01412 -0.0018298 +
[2688] MYH9 <--- ROCK1 0.024697 0.042724 +
[2689] GNAT3 ---> PDE6B 0.45807 -0.32299 +
[2690] CASP8 <--- TLR3 -0.14978 -0.082012 +
[2691] BCAR1 ---> PRKCQ 0.0033496 -0.01579 -
[2692] FGA ---> ITGA2B -0.0342 -0.10024 +
[2693] ABL1 ---> SHC3 0.040787 -0.1399 +
[2694] ITGA5 ---> THBS4 0.046345 -0.039839 -
[2695] CCL11 <--- CCR3 -0.35895 0.21658 -
[2696] RAC2 <--- WASF2 -0.076889 0.12856 -
[2697] ATF1 ---> RB1 0.071648 -0.11814 +
[2698] CACNA1C <--- GNB4 -0.14673 0.18744 +
[2699] ARHGAP25 <--- RHOD -1.2097 0.92798 -
[2700] ACTN2 <--- MYH6 -0.037584 0.019009 +
[2701] GRB2 ---> IL23A 0.12689 -0.16241 -
[2702] CDKN1A <--- PSMD4 -0.051854 0.14223 +
[2703] CREB1 ---> EGR1 0.097302 0.088432 +
[2704] CEBPB ---> DLX1 0.15908 -0.24396 +
[2705] CBL ---> IL7R 0.3001 -0.17434 +
[2706] PRKCB ---> PRKD1 0.11731 -1.1868 +
[2707] CTCF ---> WNT1 0.5734 -0.94937 +
[2708] FOS <--- VEGFB -0.18489 0.28082 -
[2709] NUP43 ---> RBM8A 0.31391 -0.17132 +
[2710] CDK1 <---> WEE1 0.31014 0.31014 -
[2711] FOS <--- SMARCA4 -0.11153 0.069245 +
[2712] EIF2AK1 <---> MAP2K4 0.31014 0.31014 -
[2713] CD80 <--- CD8A -0.0347 -0.027692 -
[2714] CDK1 <---> SLK 0.31014 0.31014 -
[2715] CUL1 ---> PSMD10 0.32103 -0.32277 -
[2716] ARHGAP36 <--- RHOA -1.5245 1.016 -
[2717] RPTOR ---> SREBF1 0.038999 -0.11391 +
[2718] DOCK1 ---> ELMO1 0.055526 -0.16405 -
[2719] ARHGEF3 ---> RHOT1 0.33144 -0.31781 +
[2720] JAG1 ---> RBPJ 0.0073315 -0.0044081 +
[2721] FHL2 ---> JUN 0.47419 -0.45628 -
[2722] FGF19 ---> KDR 0.21774 -0.0015767 +
[2723] ABL1 <--- BLK -1.0203 0.50083 +
[2724] IL1B ---> PYCARD 0.31443 -0.37463 -
[2725] ATP1B4 ---> PIK3CA -0.0050831 -0.010841 -
[2726] NFKB1 <--- PRKCA -0.10517 0.10818 +
[2727] MAPK8 ---> PAFAH1B1 0.33238 -0.19282 +
[2728] BPTF ---> ZNF263 0.23239 -0.096677 -
[2729] ATP1A2 ---> PIK3CA -0.0036365 -0.0057548 -
[2730] PARD6A <--- RAC3 0.018694 0.089672 +
[2731] MAPK8 <--- TAB2 -0.13728 0.23085 +
[2732] HNF1A ---> YY1 0.078944 0.049909 -
[2733] CCL18 <--- XCR1 -0.35895 0.21658 -
[2734] ESR1 ---> MEOX1 0.10711 -0.27075 +

[2735]	ARHGAP11A	<---	RHOV	-1.5245	1.016	-
[2736]	NFKB1	--->	WWOX	0.16973	-0.19974	+
[2737]	CD3E	--->	TCRA	-0.033535	-0.068284	-
[2738]	ABL1	<---	CBL	-0.12808	0.065466	-
[2739]	DRD5	--->	GNAL	1.1631	-1.1303	+
[2740]	CD4	<---	CSK	-0.20543	0.30675	-
[2741]	GATA1	<---	HNF4A	-0.091347	-0.010433	+
[2742]	DDX1	--->	MYC	0.031508	-0.11942	-
[2743]	PPP1CA	<---	PRKG2	-0.35613	0.22476	+
[2744]	FRS2	--->	SPRY4	0.23114	-0.13172	+
[2745]	RPL30	--->	RPS6	0.54301	-0.64425	-
[2746]	CTGF	<---	WWTR1	-0.076032	0.014964	+
[2747]	RHOC	<---	SOS1	-0.33772	0.48674	+
[2748]	PTK2	<---	VCL	-0.31846	0.12607	-
[2749]	ARHGAP24	<---	NANOG	-0.093007	0.13613	+
[2750]	MCF2L	<---	RHOC	-0.026438	0.029604	-
[2751]	APAF1	--->	CASP9	-0.17607	-0.45498	+
[2752]	GNGT2	--->	PLCB1	0.042091	-0.077521	+
[2753]	BCL3	<---	ZNF280A	-0.064911	0.62238	-
[2754]	MYOD1	<---	ZFHX3	-0.10614	0.080848	-
[2755]	MYD88	<---	TLR6	0.05829	0.13137	+
[2756]	GYS1	<---	PRKAG1	-0.41895	0.087064	+
[2757]	EGR1	--->	SERPINB9	0.22447	-0.21215	+
[2758]	ATF2	--->	BMP2	0.32646	-0.28355	-
[2759]	FRZB	--->	WNT16	1.0317	-1.1063	+
[2760]	FADD	<---	FAS	-0.76832	0.62176	+
[2761]	CXCR3	<---	GRK5	-0.28786	0.33635	+
[2762]	ATP1B2	<---	PIK3R2	-0.066584	-0.059014	+
[2763]	NEK2	--->	PLK1	0.29896	-0.098976	-
[2764]	ESR1	<---	SRC	-0.2721	0.34018	-
[2765]	ARR3	--->	WNT1	0.23514	-0.34616	-
[2766]	SMPD3	<---	TRADD	-0.30009	0.14194	+
[2767]	CXCR5	--->	GNAI3	1.1631	-1.1303	+
[2768]	COL10A1	<---	LEPREL2	-0.11204	0.03851	+
[2769]	PPP2R1A	--->	YAP1	0.19485	-0.34161	+
[2770]	ETS1	--->	PDGFRB	0.036699	-0.01322	+
[2771]	ELAVL1	<---	PRKAG3	-0.911	0.97387	+
[2772]	PPARGC1A	<---	PRKAG1	-0.911	0.97387	+
[2773]	GCKR	--->	NUP153	0.23664	-0.24455	-
[2774]	CCL2	<---	CXCR2	-0.35895	0.21658	-
[2775]	HPN	--->	REST	0.28002	-0.15205	-
[2776]	CCL1	<---	CCR5	-0.35895	0.21658	-
[2777]	ARHGEF16	<---	CDC42	-0.067065	0.085273	-
[2778]	OGT	<---	TAF1	-0.086655	-0.0025849	+
[2779]	FOXA1	--->	PCK1	0.5532	-0.58214	+
[2780]	STAT3	<---	TIMP3	-0.32334	0.44408	-
[2781]	PGR	--->	PIK3R1	0.033109	-0.12175	+
[2782]	JUND	<---	MTF2	-0.25166	0.17999	-
[2783]	MYL2	<---	MYLK4	-1.1685	1.2382	+
[2784]	FOXO3	--->	GATA1	0.19808	-0.23065	-
[2785]	CAMK2D	--->	MEF2D	0.69813	-0.91661	+
[2786]	CBLB	--->	IL20RA	0.61825	-0.39145	+
[2787]	GNAI2	<--->	GNAI3	0.064764	0.064764	-
[2788]	CUL1	--->	SKP2	-0.0051086	-0.13603	-
[2789]	CCR3	--->	CXCL1	0.21658	-0.35895	-
[2790]	ARAP2	<---	RHOJ	-0.48615	0.36733	-
[2791]	ACTG2	<---	RHOG	-0.51168	0.52339	+
[2792]	F2RL2	--->	PRSS3P2	0.11779	-0.2522	-
[2793]	HDAC1	<---	TFDP2	-0.087553	0.1595	+
[2794]	IL10	--->	IL13RA2	0.094523	-0.18506	+
[2795]	SREBF1	<---	STRN3	-0.38125	0.38477	-
[2796]	PIK3CG	--->	PTEN	0.03343	-0.063035	-
[2797]	HRAS	<---	INS	-0.0077402	0.03884	+
[2798]	BIRC3	<---	FADD	-0.12287	-0.0028914	+
[2799]	ARHGAP17	<---	RHOG	-0.92575	0.61511	-
[2800]	MYL5	<---	MYLK3	-0.29873	0.36004	+
[2801]	ANKRD1	<---	FOXA1	-0.51151	0.56498	+
[2802]	ATG12	<---	FOXO4	-0.37846	0.32839	+
[2803]	PIK3CG	--->	TPTE	0.033723	-0.052	-
[2804]	CSH1	--->	MPL	0.056231	-0.11334	+
[2805]	F2R	--->	GNAQ	1.1631	-1.1303	+
[2806]	ESRRA	--->	MEF2C	0.079825	-0.25631	+
[2807]	IL3RA	<---	PTPN11	-0.29595	0.42284	+
[2808]	NFKB2	--->	TYK2	0.49194	-0.5357	-
[2809]	FYN	--->	GIT2	0.14621	-0.11451	+
[2810]	CD3D	<---	ITK	-0.060744	0.20777	-

[2811]	MAP2K6	<--->	TAOK1	0.31014	0.31014	-
[2812]	ERBB3	--->	TAL1	0.34669	-0.32435	-
[2813]	DRD4	--->	KCNJ9	0.11779	-0.2522	-
[2814]	AKT2	--->	SOS2	0.052556	-0.15964	-
[2815]	FCGR3A	--->	VAV1	0.11008	0.093865	+
[2816]	BMP2	<---	TAB2	-0.45928	0.36401	-
[2817]	CD83	<---	PPARG	-0.087888	-0.023531	+
[2818]	FGA	--->	PROC	0.14643	-0.18567	-
[2819]	BRAF	<---	NRAS	-0.52464	0.47332	-
[2820]	CCND1	<---	E2F2	-0.39339	0.45542	-
[2821]	HRAS	<---	LCK	0.099664	0.61281	+
[2822]	PTPN11	--->	VAV2	0.050213	-0.1064	+
[2823]	ACTC1	<---	RHOQ	-0.51168	0.52339	+
[2824]	CD40	<---	RELA	-0.781	0.4206	-
[2825]	PDGFB	--->	STAT5B	0.2715	-0.20049	+
[2826]	CBL	--->	PIK3R1	0.080801	-0.12639	+
[2827]	NRAS	<---	PRKCB	-0.41664	0.51818	+
[2828]	KRAS	<---	RASAL2	-0.68738	0.87061	+
[2829]	GH1	<---	MAPK3	-0.26091	0.40675	-
[2830]	ITGA2	<---	PIK3R1	-0.047872	-0.029048	-
[2831]	GLI1	<---	PSMD14	-0.19533	0.33989	+
[2832]	STAT3	<---	TRIM25	-0.12386	0.22483	-
[2833]	MEF2C	<---	SPIB	-0.29909	0.1217	-
[2834]	FRS2	--->	SOS1	0.11735	0.037859	+
[2835]	IL6R	--->	VAV1	0.058648	0.025	+
[2836]	HDAC2	<---	PHF19	-0.36144	0.40916	-
[2837]	CACNA1C	<---	GNB5	-0.14673	0.18744	+
[2838]	AR	<---	NCOA2	-0.010612	-0.00022423	-
[2839]	IRS2	--->	SOCS3	-0.098579	-0.19619	-
[2840]	FGD2	<---	NFKB1	-0.0541	0.056978	+
[2841]	CCL2	<---	CCR8	-0.35895	0.21658	-
[2842]	SP1	--->	ZBTB17	-0.024143	-0.025264	-
[2843]	ITGA9	<---	PTK2B	-0.076638	-0.038051	+
[2844]	GRB2	--->	IL6R	0.1568	-0.17404	-
[2845]	SREBF1	<---	ZNF219	-0.059615	0.19618	-
[2846]	CREB3L4	<---	MAPK3	-0.57905	1.0858	+
[2847]	SMAD4	--->	TLE2	-0.016256	-0.083613	-
[2848]	DNM2	<---	PIK3R2	-0.068974	-0.0029759	+
[2849]	BMPR2	<---	TAB2	-0.13684	0.27363	-
[2850]	COL3A1	--->	PPIB	0.022658	-0.10782	-
[2851]	SEH1L	--->	SRSF7	0.31391	-0.17132	+
[2852]	FOS	<---	MAPK14	-0.57905	1.0858	+
[2853]	GRK6	--->	HRH2	0.33635	-0.28786	+
[2854]	FYN	--->	ITGB7	0.38494	-0.27641	-
[2855]	IL2RG	--->	PIK3R5	0.049565	-0.13438	+
[2856]	IL18RAP	--->	JUN	0.1981	-0.17954	+
[2857]	ITGA9	<---	SRC	-0.20204	0.23404	-
[2858]	COL5A1	--->	ITGA6	-0.013036	-0.10803	+
[2859]	IL12RB1	--->	STAT3	0.38886	-0.28644	-
[2860]	ALK	<---	PIK3CG	-0.023151	0.015763	-
[2861]	SMAD3	<---	UBE2D1	-0.20131	0.05544	+
[2862]	JAK1	--->	MAPK11	0.43202	-1.1344	+
[2863]	PRKCH	--->	RASAL2	0.14565	-0.025686	-
[2864]	GRIN2C	<---	GRM5	-0.36981	-0.05834	+
[2865]	H3F3A	--->	TBP	0.078479	-0.13706	+
[2866]	EGFR	--->	ITGB1	0.087202	-0.081427	-
[2867]	EBF3	--->	TCF12	0.54286	-0.55095	-
[2868]	ACTA1	<---	MMP16	-0.29263	0.085904	+
[2869]	LAMA3	--->	MMP20	-0.0038564	-0.069983	-
[2870]	KCNJ5	<---	OPRL1	-0.2522	0.11779	-
[2871]	PEBP1	<---	PPP6C	-0.54234	0.30906	-
[2872]	ITGB5	--->	LAMA2	-0.0055713	-0.0064655	-
[2873]	IL23R	<---	IL6	-0.034956	-0.0057724	+
[2874]	PRKCB	--->	TCF7L2	0.45036	-0.38915	-
[2875]	IGKV1-5	--->	VAV2	0.064425	-0.018037	+
[2876]	SEH1L	<---	SNRPD3	-0.10631	0.074498	-
[2877]	IFIH1	<---	NFKB1	-0.12462	0.13206	+
[2878]	CCR6	--->	CXCL1	0.21658	-0.35895	-
[2879]	IL3RA	<---	PRL	-0.055821	-0.0077042	+
[2880]	JAK3	--->	MAPK11	0.43202	-1.1344	+
[2881]	EGR1	<---	TLR7	-0.47252	0.15132	-
[2882]	GNAT1	<---	SAG	-0.0080738	0.030816	+
[2883]	FCER1A	--->	PTPN11	0.052018	0.00515	-
[2884]	COL6A6	<---	PTK2	-0.046347	-0.038605	-
[2885]	AXIN1	<---	CSNK2A1	-0.20403	0.2231	+
[2886]	BTK	--->	CD79B	0.1252	-0.043611	-

[2887]	ITGB7	--->	TNN	0.051076	-0.12975	-
[2888]	ARHGAP9	<---	RHOC	-0.36596	0.22724	-
[2889]	ITGA1	<---	LAMC1	-0.03918	-0.0041604	+
[2890]	AKT2	--->	NFKB1	0.086648	-0.068126	+
[2891]	JAK1	--->	STAM	0.014222	-0.010226	-
[2892]	ITGB1	<---	PTK2	-0.04335	-0.039708	-
[2893]	ARHGAP5	<---	RHOBTB1	-0.048842	0.38021	-
[2894]	BCR	<---	GATA2	-0.05687	-0.0069736	+
[2895]	GRB2	--->	RHOA	0.63892	0.0030067	-
[2896]	COL2A1	--->	ITGB7	0.0059092	-0.05696	+
[2897]	SELE	--->	STAT6	0.13474	-0.094216	-
[2898]	COL11A2	<---	GP6	-0.27286	0.25603	-
[2899]	CTNNB1	<---	PCDH20	-0.19204	0.11503	-
[2900]	PLN	<---	PPP1CB	-0.5028	0.29452	+
[2901]	NOTCH3	<---	PCSK6	-0.078069	0.10256	+
[2902]	STAT1	<---	STAT3	-0.13561	-0.1014	-
[2903]	IL6ST	<---	JAK3	-0.1451	0.11865	-
[2904]	CHRM1	--->	KCNJ9	0.11779	-0.2522	-
[2905]	CNTF	--->	LIFR	0.056231	-0.11334	+
[2906]	GP9	--->	SERPIND1	0.39784	-0.66389	-
[2907]	SOCS7	--->	STAT3	0.27422	-0.74698	-
[2908]	CD40LG	--->	MAPK9	0.10494	0.048942	+
[2909]	CD4	<---	LCP2	-0.10512	0.021607	-
[2910]	CCL25	<---	CCR5	-0.35895	0.21658	-
[2911]	PPP2CB	--->	PRKCI	0.086514	-0.097871	+
[2912]	EIF4E	<---	NUP37	-0.37256	0.38207	+
[2913]	COL4A3BP	<---	PRKD1	-0.1056	0.2913	+
[2914]	GLI2	<---	TEAD3	-1.1565	1.4387	+
[2915]	BSG	<---	EGR2	-0.068599	0.24925	+
[2916]	IFNG	--->	LEPR	0.11434	-0.28909	+
[2917]	NUP43	--->	SRSF5	0.31391	-0.17132	+
[2918]	ADRBK2	--->	SSTR1	0.25327	-0.21301	+
[2919]	GNRHR	<---	GRK1	-0.28786	0.33635	+
[2920]	CREB5	<---	RPS6KA5	-0.98092	1.2458	+
[2921]	CACNA1S	<---	CAMK2G	-0.26963	0.34735	+
[2922]	SAG	--->	WNT8B	0.23514	-0.34616	+
[2923]	COL27A1	<---	GP6	-0.27286	0.25603	-
[2924]	KPNA3	<---	RANBP2	-0.33056	0.23067	+
[2925]	BATF	<---	CHD3	-0.18206	0.11598	-
[2926]	SREBF2	<---	ZNF718	-0.20142	0.33079	-
[2927]	NCK2	<---	SRC	0.063181	0.23434	+
[2928]	RHOB	--->	SRGAP2	0.39011	-0.42732	-
[2929]	CREB3	<---	PRKCG	-0.63701	0.72046	+
[2930]	BHLHE40	<---	HCFC1	-0.47588	0.27999	-
[2931]	MMP8	<---	PLG	-0.1166	-0.013996	+
[2932]	DRD4	--->	KCNJ3	0.11779	-0.2522	-
[2933]	CDK1	<---	GADD45G	-1.126	0.89119	+
[2934]	CCL28	<---	CCR8	-0.35895	0.21658	-
[2935]	ADCY6	<---	RAPGEF3	-0.28599	0.53533	-
[2936]	CXCR1	--->	DNM1	0.082554	-0.088002	-
[2937]	BIRC3	<---	TRADD	-0.062126	-0.021809	+
[2938]	PRKCZ	--->	STAT1	-0.025727	-0.049864	-
[2939]	FARP1	<---	RHOA	-0.11502	0.058173	-
[2940]	BMPR1A	--->	SMAD6	0.7946	-0.60139	-
[2941]	FZD3	<---	GNB2	-0.19257	0.19185	-
[2942]	PPARD	<---	TCF7L1	-0.983	0.9955	+
[2943]	LCK	--->	PIK3R2	0.26317	-0.26628	+
[2944]	NOTCH2	<---	PCSK2	-0.083505	0.10143	+
[2945]	RASGRF2	--->	RHOT1	0.3454	-0.39621	+
[2946]	MYL9	<---	SORBS3	-0.5985	0.56563	-
[2947]	GNA14	<---	TACR2	-1.1303	1.1631	+
[2948]	FCGR1A	--->	VAV2	0.18158	-0.03415	+
[2949]	EP300	--->	RBPJL	0.0084282	-0.010229	+
[2950]	DUSP12	--->	MAP3K4	1.4184	-1.4202	+
[2951]	CHRM5	<---	PRKCQ	-0.2791	0.17482	-
[2952]	IRX4	--->	MEF2A	0.023045	-0.22085	-
[2953]	ARHGAP35	<---	RHOG	-0.12797	1.0383	-
[2954]	EP300	--->	MAPT	0.00067605	-0.0050208	+
[2955]	ADAM17	--->	CDH3	0.068724	-0.20957	+
[2956]	IL11RA	<---	SOCS5	-0.16657	0.10622	+
[2957]	FGFR4	--->	SOS2	0.041735	0.010746	+
[2958]	FOLR1	<---	SPI1	-0.086078	0.060492	+
[2959]	PDGFRA	<---	PIK3CD	-0.039311	0.031642	-
[2960]	GADD45B	--->	MAP2K6	0.89119	-1.126	+
[2961]	CDC42	--->	CDH1	0.12902	-0.40807	+
[2962]	CASP8	--->	DAXX	0.12709	-0.6543	-

[2963]	FZD7	<---	WNT5A	-0.78209	0.63734	+
[2964]	MRPL38	--->	MRPS33	-0.039883	-0.061045	-
[2965]	ETV4	<---	TCF7L2	0.040068	0.11681	+
[2966]	ITGA1	<---	MYH11	-0.03918	-0.0041604	+
[2967]	STAT2	<---	TRIM22	-0.20194	0.30315	-
[2968]	CCL27	<---	CCR7	-0.35895	0.21658	-
[2969]	RAC2	<--->	RHOA	0.1628	0.1628	-
[2970]	E2F1	<---	MDM2	-0.25436	0.15106	+
[2971]	GNAL	<---	HTR7	-1.1303	1.1631	+
[2972]	IL13RA2	<---	LEP	-0.18506	0.094523	+
[2973]	DLG3	--->	GRIK4	0.19945	-0.095793	-
[2974]	ITGA5	<---	LAMC2	-0.066621	-0.0090065	+
[2975]	DAB2	--->	JUN	0.16686	-0.19603	-
[2976]	CREBBP	--->	STAT3	0.01914	-0.017328	+
[2977]	DRD2	<---	PPP2R2A	-0.11654	-0.0038512	-
[2978]	CXCL11	<---	CXCR6	-0.35895	0.21658	-
[2979]	LCK	--->	MLLT4	0.12276	-0.082867	+
[2980]	PTP4A3	--->	RHOA	0.56686	-0.55945	+
[2981]	ITGA2	<---	TNN	-0.076459	-0.047786	+
[2982]	PTPRA	--->	SRC	0.28716	-0.25253	-
[2983]	AAAS	--->	KPNA2	0.12101	-0.19114	+
[2984]	GNAZ	<---	MAPK8	-0.22953	0.50225	-
[2985]	CTBP2	--->	IRX5	-0.0012926	-0.14397	+
[2986]	FGF20	--->	INSR	0.25346	-0.0044024	+
[2987]	COL5A2	--->	ITGB6	0.0024845	-0.035647	+
[2988]	IL12RB1	--->	SOS2	0.072558	0.062464	+
[2989]	CBL	--->	IL20RB	0.61825	-0.39145	+
[2990]	CCL8	<---	CXCR2	-0.35895	0.21658	-
[2991]	FZD5	<---	SFRP4	-1.3633	0.73901	+
[2992]	CALML6	--->	CNGB1	0.064313	-0.070242	+
[2993]	SHC3	<---	SPRY2	-0.15769	0.090914	-
[2994]	WIF1	--->	WNT6	0.49247	-0.8346	+
[2995]	CASP6	--->	PTBP1	0.58312	-0.79899	+
[2996]	ROBO2	<---	SRGAP2	-0.19806	0.11796	-
[2997]	CSNK1G2	<---	PPP6C	-0.077655	0.084741	-
[2998]	CTHRC1	--->	DVL2	0.45961	-0.60718	+
[2999]	FGR	<---	PTPN13	-0.03006	0.033239	+
[3000]	SMAD2	<---	SMURF2	-0.50288	0.48017	-
[3001]	IFNGR2	<---	IL20	-0.087402	0.20113	+
[3002]	CTNNB1	<---	PCDHA12	-0.19204	0.11503	-
[3003]	FGG	<---	RAP1B	-0.096109	0.097201	-
[3004]	FCER1A	--->	GAB2	0.5919	-0.47829	+
[3005]	PRKACA	--->	PTGIR	0.19743	-0.10024	-
[3006]	JUN	<---	NFATC1	-0.477	0.29934	+
[3007]	GPR183	--->	MEF2C	0.21915	-0.42605	-
[3008]	TLR4	--->	UBE2D3	-0.027081	-0.36832	-
[3009]	EXOC8	<---	RAC1	-0.50681	0.59188	+
[3010]	IL12RB2	<---	IL18RAP	-0.10194	0.10427	-
[3011]	APPL1	<---	CASP3	-0.35546	0.22868	-
[3012]	NCF1	--->	RAC1	0.12583	0.034532	-
[3013]	KCNS2	--->	REST	0.1257	0.025162	-
[3014]	ACTC1	<---	MMP8	-0.29263	0.085904	+
[3015]	DAXX	--->	REST	0.18808	-0.22487	-
[3016]	MAPK10	--->	PGR	0.29076	-0.30308	-
[3017]	GAB1	<---	PXN	-0.20135	0.26799	-
[3018]	ACVRL1	--->	SMAD9	0.49955	-0.48017	+
[3019]	ARHGAP44	<---	RHOH	-0.92575	0.61511	-
[3020]	COL24A1	--->	ITGA6	-0.013036	-0.10803	+
[3021]	MYL9	<---	TPM4	-0.57325	0.45789	-
[3022]	GHRL	--->	HCAR2	0.61472	-0.75338	+
[3023]	ACTBL2	<---	RAC1	-0.51168	0.52339	+
[3024]	CASP10	--->	FASLG	0.16302	-0.61452	-
[3025]	RHOBTB2	--->	VAV3	0.034198	0.010174	-
[3026]	CUL3	--->	PSMD7	0.065557	-0.142	-
[3027]	APOA1	--->	RXRA	-0.056454	-0.16004	-
[3028]	ACVR2A	<---	BMPR1A	-0.20314	-0.072808	-
[3029]	CTCFL	--->	STAT5A	0.25273	-0.13931	+
[3030]	IL6	<---	MAPK13	-0.26091	0.40675	+
[3031]	AHRR	<---	EP300	-0.015981	0.014019	+
[3032]	RAF1	--->	RB1	0.067028	-0.1475	+
[3033]	SOST	--->	WNT8B	0.41775	-0.59125	+
[3034]	SOCS1	<---	TLR2	-0.53321	0.20294	-
[3035]	CAMKK1	--->	PRKAG3	1.1791	-1.2499	+
[3036]	PARD3	--->	PVRL3	0.065762	-0.040193	-
[3037]	HNF4A	--->	SLC9A3R1	0.022463	-0.12284	+
[3038]	FZD3	<---	WNT11	-0.78209	0.63734	+

[3039]	DUSP14	--->	MAP3K4	1.4184	-1.4202	+
[3040]	NRAS	<--->	RALB	0.097643	0.097643	-
[3041]	ADRA1A	--->	PRKAG3	0.84337	-1.591	+
[3042]	SOS2	--->	SPRY2	1.2804	-0.54968	-
[3043]	EGF	<---	PXN	-0.10743	0.1165	-
[3044]	ITGAV	<---	VEGFA	-0.083343	0.007506	-
[3045]	ADRBK2	--->	DRD5	0.25327	-0.21301	+
[3046]	CCND1	<---	CDK7	-0.55661	0.75243	+
[3047]	PIK3R2	--->	PTK2B	-0.11451	-0.24511	-
[3048]	ARHGAP44	<---	RHOQ	-0.92575	0.61511	-
[3049]	MYH3	<---	TNNT1	-0.020839	-0.0058045	-
[3050]	CAMK4	--->	EP300	0.013728	-0.006941	+
[3051]	CBL	--->	IL6ST	0.11039	-0.066545	+
[3052]	FGD1	<---	RHOV	-0.14219	0.16153	-
[3053]	CCNE2	<---	SKP2	-0.22163	0.13223	+
[3054]	ITGA11	<---	PTK2B	-0.076638	-0.038051	+
[3055]	CRLF2	<---	THPO	-0.17292	0.061531	+
[3056]	KIF2C	<---	SMARCA4	-0.093637	0.011117	+
[3057]	ICAM1	<---	IFNG	-0.409	0.40556	+
[3058]	ADCY2	<---	GNAI3	-0.63317	1.0384	-
[3059]	AGT	<---	EGR1	-0.12977	0.13505	+
[3060]	AKT2	--->	FGFR2	0.053523	-0.092062	-
[3061]	GNAI2	<---	HCAR2	-1.1303	1.1631	+
[3062]	GLI2	<---	YAP1	-0.27643	0.32828	+
[3063]	MAPK1	--->	NRG3	0.44142	-0.35781	-
[3064]	ACTG2	<---	MYH11	-0.19158	0.14703	+
[3065]	ACTB	<---	MYL10	-0.74059	0.64379	+
[3066]	LTB	<---	NFKB1	-0.39904	0.30264	+
[3067]	ADORA2A	<---	GRK6	-0.28786	0.33635	+
[3068]	HSPB1	<---	RELA	-0.46471	0.18561	-
[3069]	COL5A3	--->	ITGA9	-0.013036	-0.10803	+
[3070]	CACNB4	<---	PPP2R3A	-0.060248	0.10014	+
[3071]	NTRK1	<---	PIK3CB	-0.059227	0.015072	-
[3072]	KCNH4	--->	REST	0.27672	-0.20732	-
[3073]	AGT	<---	CTSG	-0.32562	0.20126	+
[3074]	ATF6B	<---	PRKCG	-0.63701	0.72046	+
[3075]	PRKACG	--->	SCN4B	0.41609	-0.28631	+
[3076]	COL5A3	--->	ITGA3	-0.020349	-0.077049	+
[3077]	PLA2G4E	<---	RASGRP2	-0.071292	0.0030946	+
[3078]	ACTG1	<---	PRKCQ	-0.37374	0.26975	+
[3079]	DCC	<---	MAP2K2	-0.10556	0.15829	-
[3080]	CDKN1B	<---	CUL1	-0.25493	0.27648	-
[3081]	CSF1R	<---	FGF14	-0.64493	0.77791	+
[3082]	ERBB3	<---	PRKCZ	-0.081967	0.11234	-
[3083]	ARHGEF9	<---	RHOF	0.091479	0.13323	-
[3084]	ARF6	--->	ASAP3	0.099163	-0.17356	-
[3085]	GP9	--->	VWF	0.065267	-0.20741	-
[3086]	HOXA2	--->	TCF12	0.23888	-0.32236	-
[3087]	EDNRA	--->	GNA15	1.1631	-1.1303	+
[3088]	CREB3	<--->	CREM	0.22155	0.22155	-
[3089]	AKT1	--->	SOS2	0.052556	-0.15964	-
[3090]	PTPRC	--->	TCRB	0.14933	-0.21762	+
[3091]	DAND5	--->	GUCY2C	0.16318	-0.16622	+
[3092]	ARHGAP35	<---	RHOBTB1	-0.078715	0.62868	-
[3093]	ACTBL2	<---	MMP12	-0.29263	0.085904	+
[3094]	DUSP7	--->	MAPK11	1.353	-1.036	+
[3095]	DAXX	<---	MAP2K7	-0.32851	0.12919	-
[3096]	RASGRF2	--->	RHOQ	0.44606	-0.52428	+
[3097]	GNAI1	<---	HTR1E	-1.1303	1.1631	+
[3098]	BMP6	--->	BMPR1A	0.27387	-0.33406	+
[3099]	FZD8	<---	GNA15	-0.22143	0.24435	-
[3100]	CSNK1A1L	--->	WNT3	0.83912	-0.78694	-
[3101]	YY1	--->	ZNF585B	-0.013494	-0.067472	+
[3102]	CXCL11	<---	CXCR5	-0.35895	0.21658	-
[3103]	REST	--->	SCRT2	-0.037992	-0.039841	+
[3104]	NRAS	<---	YWHAZ	-0.15318	0.090199	-
[3105]	ITGB1	--->	TNXB	0.02988	-0.079795	-
[3106]	CCL21	<---	CXCR4	-0.35895	0.21658	-
[3107]	DEPDC1B	<---	RHOD	-0.93788	0.64026	-
[3108]	MAPK12	<--->	PRKX	0.31014	0.31014	-
[3109]	CHRM2	<---	PKN3	-0.17138	0.05983	-
[3110]	SAG	--->	WNT2	0.23514	-0.34616	-
[3111]	PLCL1	<---	RGS13	-0.082548	0.03305	+
[3112]	MYO9B	<---	RHOB	-0.54674	0.38058	-
[3113]	HRAS	<---	SOCS1	-0.093407	0.044639	+
[3114]	EGFR	--->	HGS	0.051791	-0.050488	-

[3115]	PPARA	--->	PPARGC1B	0.23448	-0.31347	+
[3116]	BRCA1	<---	FOXA1	-0.026542	0.04582	+
[3117]	PIK3R3	<---	PRKCD	-0.83874	0.97532	-
[3118]	MAPK3	--->	UBTF	0.85317	-0.45799	+
[3119]	MAP4K2	--->	TRADD	0.24439	-0.21179	-
[3120]	RHOH	<---	VAV1	0.1025	0.10902	+
[3121]	MMP2	<---	TIMP3	-0.18543	-0.0056809	+
[3122]	GNA15	--->	WNT5A	0.42087	-0.47341	-
[3123]	ACTA1	<---	MMP9	-0.29263	0.085904	+
[3124]	IRS1	--->	ITGB3	0.044885	0.0067114	-
[3125]	SHC1	--->	SOS1	0.31513	-0.30654	-
[3126]	DUSP6	--->	MAPK12	1.353	-1.036	+
[3127]	F2RL1	--->	PLG	0.065338	-0.16965	-
[3128]	IL12RB1	<---	IL18RAP	-0.26755	0.27531	-
[3129]	BCL10	<---	TAB2	-0.65842	0.65755	+
[3130]	BATF	--->	NCOA3	0.041759	-0.020708	+
[3131]	NFKBIB	<---	TAB1	-0.62263	0.79944	+
[3132]	E2F3	--->	TBP	0.22411	-0.26146	+
[3133]	SEH1L	--->	SRSF9	0.31391	-0.17132	+
[3134]	IRS2	--->	SOCS1	-0.098579	-0.19619	-
[3135]	AKT2	<---	PPP2R1A	-0.35974	0.31573	-
[3136]	MAP3K3	<---	RAC1	-0.17545	1.1267	+
[3137]	CDC42	--->	GRIN2C	0.54247	-0.38938	-
[3138]	BCL2	<---	MAX	-0.30558	0.14911	+
[3139]	CD247	<---	RGS1	-0.25487	0.18879	+
[3140]	AKT1	--->	STMN1	0.16383	-0.26287	+
[3141]	MAFK	<---	SP5	0.047027	0.072056	-
[3142]	CD79A	<---	GRB2	-0.19344	0.10389	-
[3143]	FXYD2	<---	PRKACB	-0.26091	0.40675	+
[3144]	GNB2	<---	PLCB3	-0.014636	-0.0050336	-
[3145]	BAMBI	--->	FZD5	0.43771	-0.51806	+
[3146]	NFKB1	--->	SKP1	0.14195	-0.14364	-
[3147]	EP300	--->	FGB	0.006717	-0.012918	+
[3148]	RORC	--->	STAT3	0.27087	-0.22437	-
[3149]	HRAS	--->	PRKCD	0.34126	-0.27388	-
[3150]	CTCFL	<---	ZNF554	-0.039841	-0.037992	-
[3151]	FZD10	<---	WNT10B	-0.78209	0.63734	+
[3152]	CHRM3	--->	GNAQ	1.1631	-1.1303	+
[3153]	ACVR2A	<---	BAMBI	-0.85898	0.90858	+
[3154]	MAPK3	--->	PLA2G4A	0.18871	-0.84715	+
[3155]	RHOBTB2	<---	SOS1	-0.20593	0.29412	+
[3156]	CAMK2A	--->	CREB3	0.72002	-0.41113	+
[3157]	CD3E	--->	GNAI2	0.05262	-0.011498	-
[3158]	HNF4G	--->	SRCAP	-0.0098898	-0.11044	+
[3159]	AXIN1	<---	CSNK1D	-0.20403	0.2231	-
[3160]	CSNK1E	--->	WNT9A	0.83912	-0.78694	-
[3161]	COL4A2	--->	ITGA8	-0.013036	-0.10803	+
[3162]	GLI1	--->	PTCH2	0.24988	-0.17362	+
[3163]	ARHGAP4	<---	RAC2	-0.42732	0.39011	-
[3164]	EPHA2	--->	FGF4	-0.0069714	-0.050497	-
[3165]	MYC	--->	STAT6	0.028486	-0.040883	-
[3166]	PRKACA	--->	TRPM8	0.61419	-0.44517	+
[3167]	CACNA1C	<---	PPP2CA	-0.27679	0.37996	+
[3168]	CRHR1	<---	GNA12	-0.040633	0.044298	-
[3169]	KLF5	--->	TGFB1	0.55396	-0.47503	-
[3170]	CTCF	--->	ZNF280B	-0.0051278	-0.028383	+
[3171]	COL4A2	<---	FYN	-0.45391	0.63648	-
[3172]	APC	--->	LRP6	0.021508	-0.020885	-
[3173]	EP300	<---	TLR1	-0.022171	-0.0011207	-
[3174]	IRAK1	--->	PPP2R1A	0.16464	-0.085866	-
[3175]	CCR7	--->	GNAI1	1.1631	-1.1303	+
[3176]	GNB1	--->	WNT2	0.19155	-0.26814	-
[3177]	GNAI2	<---	PLCB4	-0.023623	0.0010472	-
[3178]	FIGF	--->	KIT	0.12565	-0.099588	+
[3179]	RELA	--->	SOCS3	0.12744	-0.56014	+
[3180]	GRK7	--->	HTR1B	0.33635	-0.28786	+
[3181]	HNF4A	--->	STAT5A	0.27087	-0.22437	+
[3182]	EGR1	<---	SRF	-0.46864	0.59885	+
[3183]	NCK1	--->	SOS1	0.7292	-0.75682	-
[3184]	IL10RB	<---	SOCS7	-0.40298	0.060066	+
[3185]	HSPA6	--->	MAPK8	1.2218	-0.90384	+
[3186]	HRAS	--->	RPS6KA6	0.39663	-0.23906	-
[3187]	CXCR2	--->	PPBP	0.21658	-0.35895	-
[3188]	STAT2	<---	ZNF595	-0.25129	0.41972	-
[3189]	COL11A2	--->	ITGAV	-0.013036	-0.10803	+
[3190]	F11R	<---	PRKCG	-0.26818	0.23617	+

[3191]	MYC	<---	ZBTB17	-0.04967	0.0096881	-
[3192]	E2F4	<---	SPC24	-0.082946	0.0081868	-
[3193]	HDAC2	<---	HOXC6	-0.10918	0.17637	-
[3194]	LYN	--->	PIK3R3	0.79696	-0.74223	+
[3195]	FZD6	<---	WNT5B	-0.30406	0.25758	+
[3196]	CDK9	--->	SMAD4	0.88243	-0.89987	+
[3197]	CASP9	<---	EGR1	-0.30716	0.23987	+
[3198]	EP300	--->	SOX15	0.00068634	-0.00010033	+
[3199]	SIK1	<--->	STRADA	0.31014	0.31014	-
[3200]	GP1BA	--->	PIK3R1	0.10111	-0.20143	+
[3201]	EGFR	<---	PRKD3	-0.031152	0.11487	-
[3202]	CTNNB1	<---	PCDHGA8	-0.19204	0.11503	-
[3203]	CCR7	--->	FOS	0.41745	-0.36998	-
[3204]	FOXO3	--->	KLF2	0.65129	-0.48763	+
[3205]	EGR1	<---	VEGFA	-0.13878	0.21363	-
[3206]	DVL2	<---	GNAO1	-0.59426	0.46386	+
[3207]	ITGB1	<---	TLN1	-0.0032849	-0.0027006	+
[3208]	IL10RB	<---	SOCS2	-0.40298	0.060066	+
[3209]	EIF2AK4	<--->	MAPK14	0.31014	0.31014	-
[3210]	CACNA1A	<---	GNAO1	-0.17558	0.23994	+
[3211]	RALBP1	<---	RHOF	-1.5245	1.016	-
[3212]	RASGRP3	<---	SOS2	0.001776	0.018372	+
[3213]	COL6A5	--->	ITGA4	-0.0067115	-0.019363	+
[3214]	DAND5	--->	HK2	0.021163	-0.044865	+
[3215]	MAP3K1	--->	MAPK12	1.5096	-1.3549	+
[3216]	HSPG2	<---	MMP3	-0.00099974	0.0010462	+
[3217]	ORAI3	<---	STIM2	-0.88237	0.15971	+
[3218]	BMP4	--->	BMPR1B	0.27387	-0.33406	+
[3219]	CTNNA2	--->	YWHAE	1.0272	-1.0665	+
[3220]	EML4	<---	PIK3CB	-0.012551	0.008521	-
[3221]	MAPK14	--->	SLC9A1	0.40675	-0.26091	+
[3222]	KAT5	--->	USF1	0.26575	-0.31912	-
[3223]	EIF2AK2	--->	TP53	0.31197	-0.21427	+
[3224]	MAPK12	--->	PLA2G4A	0.18871	-0.84715	+
[3225]	ITGA10	<---	RELN	-0.10572	-0.014742	+
[3226]	SRC	--->	VEGFA	0.34411	-0.23622	-
[3227]	NOTCH3	<---	NUMBL	-0.58076	0.76973	+
[3228]	ACOX2	--->	RXRG	-0.03534	-0.098167	-
[3229]	CD14	--->	SFTPA1	0.070555	-0.20848	+
[3230]	ANAPC2	--->	CDC20	0.20566	-0.2136	-
[3231]	CDKN1B	<---	PSMD4	-0.051854	0.14223	+
[3232]	ZNF718	<---	ZZZ3	-0.19197	0.29869	+
[3233]	CDK1	--->	RXRA	0.17534	-0.43654	+
[3234]	LPAR2	--->	PTK2B	-0.05393	-0.18014	+
[3235]	CSNK1A1L	--->	CTNNB1	1.0802	-1.3965	+
[3236]	IGF1R	<---	IGF2	-0.70677	1.2868	+
[3237]	CHRM3	--->	KCNJ6	0.11779	-0.2522	-
[3238]	CDK4	--->	MAX	0.60415	-0.53416	-
[3239]	BIRC2	<---	TCF7	-0.61109	0.8289	+
[3240]	CCNB1	<---	PKMYT1	-0.55661	0.75243	+
[3241]	PLAT	<---	PLG	-0.017305	0.0023247	-
[3242]	AURKB	--->	PSMD7	0.2441	-0.083683	-
[3243]	CAPNS1	<---	HTT	-0.2265	0.19268	-
[3244]	CAMK2A	--->	CREB5	0.72002	-0.41113	+
[3245]	ATF1	<---	PRKACA	-0.35231	0.65748	+
[3246]	CCL7	<---	CCR8	-0.35895	0.21658	-
[3247]	ABL1	--->	STAT5B	0.17147	-0.39405	+
[3248]	E2F1	<---	YWHAZ	-0.40971	0.19729	-
[3249]	MYLK3	<--->	PRKACB	0.31014	0.31014	-
[3250]	DRD2	--->	GNGT2	0.10335	-0.26888	+
[3251]	ACTR2	<---	MMP9	-0.29263	0.085904	+
[3252]	ABCB1	<---	NFKB1	-0.17943	0.16792	+
[3253]	GNGT2	<---	MAPK9	-0.41411	0.50061	-
[3254]	PIK3CB	<---	PRKCA	-0.06189	0.049814	-
[3255]	PAK7	<---	RAC1	-1.1901	0.96651	+
[3256]	CAMK2A	--->	CDC25C	0.19972	-0.63064	+
[3257]	CCL3L1	<---	CXCR6	-0.35895	0.21658	-
[3258]	CSNK2A1	--->	POU2F3	0.60871	-0.46198	+
[3259]	CDC20	<---	PSME4	-0.054167	0.071127	+
[3260]	CAMK2D	--->	PDE3B	0.95928	-0.63646	+
[3261]	SAG	--->	WNT9A	0.23514	-0.34616	-
[3262]	AKT2	--->	FOXO4	0.30488	-0.37709	+
[3263]	IFNG	--->	MPL	0.081194	-0.1812	+
[3264]	DAND5	--->	IGFBP2	0.036704	-0.072158	+
[3265]	NSMAF	<---	SMPD1	-0.55798	0.43759	-
[3266]	CD33	<---	DAND5	-0.20303	0.1346	+

[3267]	CCNB1	<---	ERCC6L	-0.25795	0.27078	-
[3268]	AKT2	--->	RELA	0.35546	-0.53283	+
[3269]	RAPGEF4	--->	RIMS2	0.2044	-0.034891	+
[3270]	CCL17	<---	XCR1	-0.35895	0.21658	-
[3271]	GRB2	--->	IL6	0.12689	-0.16241	-
[3272]	ACTG1	<---	PRKCH	-0.43362	0.33453	+
[3273]	RAC3	<---	SOS2	-0.33772	0.48674	+
[3274]	FADD	--->	MAP2K4	0.081126	-0.58844	+
[3275]	NCAM1	<---	PTK2	-0.20353	0.029239	+
[3276]	ATP1A3	--->	PIK3CB	-0.0036365	-0.0057548	-
[3277]	COL4A3	<---	PTK2	-0.11304	-0.10314	-
[3278]	HSPG2	<---	MMP15	-0.00099974	0.0010462	+
[3279]	FGF5	<---	NRAS	-0.15714	0.23194	-
[3280]	CXCR1	--->	SHC1	0.228	-0.34661	+
[3281]	ARHGEF17	<---	RHOC	-0.012974	0.047696	-
[3282]	BAMBI	--->	GDF11	0.39038	-0.6158	+
[3283]	ARHGAP6	<---	CDC42	-1.5245	1.016	-
[3284]	PLCD4	<---	RGS17	-0.06854	0.054727	+
[3285]	CAMK2A	--->	RASGRF1	0.24914	-0.57965	+
[3286]	ADRA1A	<---	PRKAA1	-0.10024	0.19743	-
[3287]	ITGA3	<---	VWF	-0.065106	0.0089061	+
[3288]	HIST2H3A	<---	UBE2D1	-0.48509	0.38736	-
[3289]	FGF4	<---	SOS1	0.067334	0.085583	-
[3290]	MLST8	<---	PRKCB	-0.42438	0.35558	-
[3291]	COL6A3	<---	LEPRE1	-0.077201	0.025255	+
[3292]	CDC42	<---	MUSK	-0.076906	0.13971	-
[3293]	CISH	<---	GHR	-0.12483	-0.098904	-
[3294]	CBLB	--->	IL2RG	0.11039	-0.066545	+
[3295]	GRK4	--->	HTR1B	0.33635	-0.28786	+
[3296]	ARHGEF12	--->	RHOC	0.092701	0.0073174	+
[3297]	PAK1	<---	YES1	-0.81947	0.81303	+
[3298]	PLCB2	<---	PRKG1	-0.40138	0.33221	+
[3299]	DAND5	--->	MMP28	0.04259	-0.20319	+
[3300]	CCNT2	<---	DGCR8	-0.56297	0.37447	-
[3301]	GRAP2	<---	ITK	0.053566	0.10002	-
[3302]	CRK	<---	PRKCA	-0.20419	0.22188	+
[3303]	JUN	<---	MTF2	-0.25166	0.17999	-
[3304]	CAMK2B	--->	MDM2	-0.043766	-0.12114	+
[3305]	ACTA1	<---	MMP21	-0.29263	0.085904	+
[3306]	OPTN	<---	PPP1CB	-0.69501	0.50995	-
[3307]	GNB1	--->	WNT8A	0.19155	-0.26814	-
[3308]	DCC	<---	RAC1	0.013806	0.028841	-
[3309]	GNB5	<---	PIK3CA	-0.012551	0.008521	-
[3310]	FOXA2	--->	IRF2BPL	0.04582	-0.026542	+
[3311]	CCNB1	<---	CENPK	-0.15303	0.19277	-
[3312]	HSPG2	--->	LRP8	-0.0024025	-0.0096851	-
[3313]	MAPK14	--->	SCMH1	0.40675	-0.26091	+
[3314]	SMAD2	--->	TGIF1	-0.024788	-0.1488	-
[3315]	DLG4	--->	RHOJ	0.15149	0.1492	+
[3316]	ATP2A3	--->	NOTCH1	0.16442	-0.12186	+
[3317]	ARHGAP10	<---	RHOV	-0.65972	0.48549	-
[3318]	ESR1	--->	OTUD7B	-0.045315	-0.06093	+
[3319]	CALML3	<---	PPP3CB	-0.26348	0.069904	-
[3320]	BDNF	<---	CREB5	-0.75934	1.0339	+
[3321]	ARHGEF2	<---	RHOBTB1	-0.057957	0.060033	-
[3322]	CD79B	--->	IBTK	0.0016317	-0.063682	-
[3323]	S1PR3	<---	SRC	-0.3948	0.55948	-
[3324]	CSNK1G2	--->	WNT5B	0.50785	-0.4784	-
[3325]	CD86	<---	ITK	-0.05166	0.16906	-
[3326]	GNB4	<---	MTNR1A	-0.92616	1.1575	+
[3327]	ARF6	--->	ASAP2	0.012792	-0.060803	-
[3328]	CHD6	--->	MEF2A	0.057125	-0.1853	-
[3329]	E2F2	--->	TBP	0.22411	-0.26146	+
[3330]	EPO	--->	HDAC2	0.32305	-0.29113	-
[3331]	ADAM17	--->	PRKCZ	-0.0020561	-0.040896	-
[3332]	PAK1	<---	PDGFRB	-0.067603	0.1553	+
[3333]	KIT	--->	MAPK8	0.42368	-0.37214	+
[3334]	FERMT3	<---	ITGA2B	-0.051891	0.0095089	-
[3335]	HOXA9	--->	PYCARD	0.18583	-0.25804	+
[3336]	CDH2	<---	NTRK2	-0.080474	-0.06141	+
[3337]	ITGAM	<---	RHOA	-0.16491	0.017618	-
[3338]	IL13RA1	<---	SOCS7	-0.10066	-0.08965	+
[3339]	IFNGR2	<---	SOCS5	-1.0456	0.57217	+
[3340]	MAPK8	--->	RALGDS	0.45932	-0.42161	-
[3341]	MYL4	<---	TNNT1	-0.23303	0.19641	-
[3342]	GFRA1	<---	MAPK8	-0.030888	0.17118	-

[3343]	NTRK1	<---	PRKAR1A	-0.5916	0.25472	+
[3344]	PPARG	--->	WNT10B	0.13209	-0.27206	-
[3345]	HLF	--->	TAL1	0.43996	-0.52432	-
[3346]	E2F1	--->	ZNF696	0.23995	-0.10098	+
[3347]	ARAP1	<---	RHOC	-0.48615	0.36733	-
[3348]	CCR7	<---	GRK4	-0.28786	0.33635	+
[3349]	MAFK	<---	ZNF280A	0.047027	0.072056	-
[3350]	MAX	<---	SIRT1	-0.31912	0.26575	-
[3351]	COL2A1	--->	ITGA1	0.00018599	-0.07158	+
[3352]	CD7	--->	SP2	0.25462	-0.14271	-
[3353]	SETDB1	--->	ZNF165	0.16497	-0.10617	+
[3354]	NEK6	--->	RANBP2	0.12172	-0.089925	+
[3355]	GLI1	--->	WNT6	0.86373	-1.47	+
[3356]	ADAM17	--->	POTEKP	0.25337	-0.34681	+
[3357]	HDAC9	--->	MEF2C	-0.041005	-0.044156	-
[3358]	COL6A3	<---	ITGB3	-0.011216	-0.0071171	-
[3359]	MAPK10	<---	RAP1A	-0.20304	1.2491	+
[3360]	CXCR3	--->	TCF12	0.60063	-0.73364	-
[3361]	CCL17	<---	CCR3	-0.35895	0.21658	-
[3362]	PRKCA	--->	SFN	0.25075	-0.36987	+
[3363]	CXCR3	--->	PF4V1	0.21658	-0.35895	-
[3364]	DYRK2	--->	PPP1R13L	0.10239	0.0048035	-
[3365]	CD3D	--->	PIK3R5	0.39995	-0.56102	+
[3366]	CDC25A	--->	PSMD10	0.025074	-0.1744	-
[3367]	SFRP4	--->	WNT7B	1.0317	-1.1063	+
[3368]	NGEF	<---	RHOBTB1	0.054385	0.079711	-
[3369]	GNAI2	<---	OXTR	-1.1303	1.1631	+
[3370]	GNAI1	<---	RGS11	-0.736	0.71839	+
[3371]	DVL3	<---	WNT5B	-0.54867	0.38842	+
[3372]	FGF3	--->	FGFR1	1.1329	-1.3925	-
[3373]	MMP7	--->	POTEKP	0.085904	-0.29263	+
[3374]	JAK2	<---	PDGFRB	-0.71241	0.60182	+
[3375]	CTNNB1	<---	GFRA1	-0.59212	0.48158	+
[3376]	ADRBK1	--->	HRH4	0.25327	-0.21301	+
[3377]	CAMK2D	--->	FLNC	0.30815	-0.21817	+
[3378]	APBB2	--->	ZNF263	0.20172	-0.15352	-
[3379]	EGF	<---	NGFR	-0.16588	0.11351	-
[3380]	MTF2	--->	MYC	0.085003	-0.30612	-
[3381]	CARD10	<---	ESR1	-0.40179	0.24529	+
[3382]	TAF1	--->	TP53	0.14656	-0.13406	+
[3383]	CCL8	<---	CCR7	-0.35895	0.21658	-
[3384]	DLL1	<---	EPS15	-0.12146	0.061152	+
[3385]	CD44	<---	LAMA5	-0.06897	0.0013783	+
[3386]	IRS1	<---	PTPN1	-0.53775	0.64024	+
[3387]	FAM13A	<---	RHOT2	-0.58534	0.26947	-
[3388]	EIF2AK3	<--->	MAPK11	0.31014	0.31014	-
[3389]	CCL13	<---	CCR2	-0.35895	0.21658	-
[3390]	DVL2	<---	WNT7B	-0.54867	0.38842	+
[3391]	CXCL6	<---	CXCR4	-0.35895	0.21658	-
[3392]	DIAPH1	<---	PFN2	-0.1109	0.062206	-
[3393]	BUB3	--->	PSMD10	0.29993	-0.2684	-
[3394]	ARHGEF16	<---	RHOJ	-0.067065	0.085273	-
[3395]	CD14	<---	PTPN11	-0.19541	0.18347	+
[3396]	MYL6B	<---	VCL	-0.59258	0.56994	-
[3397]	LIMS1	--->	PARVA	0.4739	-0.43055	+
[3398]	IRS1	--->	SOCS3	-0.098579	-0.19619	-
[3399]	ADAMTS5	--->	B3GALTL	-0.02131	-0.035774	-
[3400]	CUL1	--->	SMAD3	0.70672	-0.96245	+
[3401]	ITGA9	<---	LAMC1	-0.041507	-0.0065623	+
[3402]	PTPN11	--->	SYK	0.85941	-1.1104	+
[3403]	PRKCI	--->	SRC	0.56064	-0.35659	-
[3404]	FRS3	<---	PRKCI	-0.13747	0.073646	-
[3405]	BCL10	<---	MAP3K7	-0.76071	0.59232	+
[3406]	ACTN2	<---	CALM1	-0.18224	0.14227	-
[3407]	MAPK3	<--->	MKNK1	0.31014	0.31014	-
[3408]	MAFK	<---	PRAME	-0.31071	0.26064	-
[3409]	CTBP2	<---	GATA2	-0.079535	-0.045311	-
[3410]	PPP3R1	<---	YWHAQ	-0.13033	-0.078387	-
[3411]	BRCA1	--->	RXRA	0.038201	-0.366	-
[3412]	CCL24	<---	CXCR2	-0.35895	0.21658	-
[3413]	CTNNB1	<---	MAP2K6	-1.3965	1.0802	+
[3414]	POU2F1	<---	TNF	-0.25754	0.13272	-
[3415]	ITGAM	<---	PIK3R1	-0.047872	-0.029048	-
[3416]	PRKD3	<---	RASAL2	0.017414	0.017696	+
[3417]	EPHB6	<---	RASA1	-0.090332	-0.087435	-
[3418]	NFKBIA	<---	PRKCD	-0.42826	0.35946	+

[3419]	FOXK2	--->	SREBF1	0.48214	-0.52321	-
[3420]	EP300	--->	HIST1H2BC	0.01343	-0.01897	+
[3421]	F2RL1	--->	GZMA	0.11779	-0.2522	-
[3422]	CTBP2	--->	ONECUT3	0.04296	-0.16727	+
[3423]	AKT1	--->	RPS6KB2	0.67325	-0.79394	+
[3424]	TAF1	--->	TRA2A	0.36909	-0.31439	+
[3425]	NFATC1	--->	PPARG	0.0055153	-0.18215	+
[3426]	F2R	--->	GNA12	1.1631	-1.1303	+
[3427]	CACNA1F	<---	PPP2CA	-0.27679	0.37996	+
[3428]	TP53	<---	TP73	-0.051157	-0.006114	-
[3429]	EGR1	--->	PROC	0.12744	-0.074666	+
[3430]	EIF4E	<---	MLST8	-0.37256	0.38207	+
[3431]	COL1A2	--->	ITGAV	-0.013036	-0.10803	+
[3432]	ADRA1B	--->	GNA14	1.1631	-1.1303	+
[3433]	BMP5	<---	EP300	-0.021569	0.0095214	+
[3434]	COL6A2	--->	ITGA4	-0.0092525	-0.030111	+
[3435]	GNAI1	<---	RGS1	-1.2531	1.2063	+
[3436]	GNAI2	<---	KCNJ5	-0.011498	0.05262	-
[3437]	PLSCR1	<---	STAT1	-0.31397	0.2569	+
[3438]	CSNK1D	--->	GLI3	1.4077	-0.99235	+
[3439]	ATF1	--->	IFNAR1	0.077561	0.056129	+
[3440]	PRKD2	--->	STAT3	0.99262	-0.67977	-
[3441]	CXCR3	--->	GNAI3	1.1631	-1.1303	+
[3442]	GRAP2	--->	VAV1	0.070133	0.047063	+
[3443]	CAMK2A	<---	PPP1CC	-0.015111	0.02592	+
[3444]	MAX	<---	SIX5	-0.19662	0.14379	+
[3445]	NTRK1	<---	PTPRC	-0.19887	0.12227	+
[3446]	FOS	<---	KMT2A	-0.11829	0.16666	-
[3447]	GNAI2	--->	RHOA	1.2694	-0.93465	+
[3448]	FLT1	--->	PLCG1	0.099165	-0.040562	+
[3449]	MAX	<---	MPP6	-0.04815	0.080114	-
[3450]	CDK2	--->	SKP1	0.24561	-0.15935	-
[3451]	CTNNA2	--->	YWHAH	1.0272	-1.0665	+
[3452]	MAPK14	<---	RIPK1	-1.2598	0.76413	+
[3453]	SHC3	<---	SYK	-0.71996	0.36756	+
[3454]	GLI1	<---	ITCH	-0.23107	0.21366	+
[3455]	DVL1	<---	FZD6	-0.12172	0.085851	+
[3456]	COL18A1	--->	MMP13	0.003315	-0.13237	+
[3457]	MAP2K1	--->	SHC1	0.70518	-0.81557	-
[3458]	EDN1	<---	MAPK14	-0.26091	0.40675	-
[3459]	ADIPOR1	--->	PRKAG3	-0.018145	-0.15746	+
[3460]	ITCH	--->	PSMD14	0.13685	-0.27177	-
[3461]	ACTBL2	<---	PTK2B	-0.28708	0.061438	+
[3462]	ITGA5	<---	LAMA4	-0.10803	-0.013036	+
[3463]	IL1R1	--->	MAP3K1	0.61156	-0.32196	+
[3464]	MYH11	--->	ROCK2	0.042533	-0.040702	-
[3465]	CRLF2	<---	IL19	-0.18506	0.094523	+
[3466]	BDNF	<---	GRB2	-0.4321	0.40823	-
[3467]	AAAS	<---	CDK1	-0.31609	0.54982	+
[3468]	CDK6	--->	E2F1	0.45782	-0.30098	+
[3469]	CACNA1F	<---	HTR2B	-0.36046	0.31836	+
[3470]	HRAS	--->	MAPK3	0.75653	-0.12425	-
[3471]	FN1	<---	PTK2	-0.21519	-0.053416	-
[3472]	IKBKB	<---	MAP3K1	-1.7597	1.2613	+
[3473]	CREBBP	--->	GATA1	-0.00068954	-0.0041449	-
[3474]	FYN	--->	GSK3B	0.92013	-1.0925	-
[3475]	GRK4	--->	HTR1F	0.33635	-0.28786	+
[3476]	IRS1	--->	PIK3CB	0.016648	-0.00088766	+
[3477]	ARR3	--->	NKD1	0.3133	-0.37274	-
[3478]	MEF2C	<---	RDX	-0.10124	0.014296	-
[3479]	GRK5	--->	HTR1D	0.33635	-0.28786	+
[3480]	CEBPB	--->	FN1	0.12248	-0.29036	+
[3481]	HDAC2	<---	SMAD5	-0.21285	0.15191	-
[3482]	FZR1	<---	SKP1	-0.060578	0.059668	+
[3483]	SOX17	--->	TCF12	0.69943	-0.32586	-
[3484]	STAT3	<---	TNF	-0.32637	0.4382	-
[3485]	KCNJ9	<---	OPRM1	-0.2522	0.11779	-
[3486]	MAPRE1	--->	PLK1	0.2805	-0.058706	-
[3487]	CACNA1H	--->	GRIN1	0.37674	-0.2845	-
[3488]	DUSP18	--->	MAPK13	1.4184	-1.4202	+
[3489]	IKBKG	--->	NFKBIB	0.22321	-0.1773	+
[3490]	CCR8	<---	GRK5	-0.28786	0.33635	+
[3491]	BAG4	<---	CASP10	-0.59854	0.17126	-
[3492]	GATA2	--->	ZBTB7A	0.16874	-0.093323	-
[3493]	CAV1	<---	PTPN1	-1.1386	0.97471	+
[3494]	CEBPB	<---	SETD3	-0.17463	0.13033	-

[3495]	KIT	--->	SH2B2	0.14622	-0.13877	+
[3496]	EP300	--->	KAT6A	0.00067605	-0.0050208	+
[3497]	CTNNB1	<---	ERBB2	-0.22663	0.22796	+
[3498]	CCR3	--->	XCL2	0.21658	-0.35895	-
[3499]	PLD1	<---	SP1	0.014284	0.037452	+
[3500]	SAR1B	--->	SEC13	0.010277	-0.18113	-
[3501]	COL1A2	--->	ITGA5	-0.013036	-0.10803	+
[3502]	CXCL9	<---	CXCR4	-0.35895	0.21658	-
[3503]	ARHGAP31	<---	RHOBTB2	-0.92575	0.61511	-
[3504]	IL10	--->	IL22RA2	0.20113	-0.087402	+
[3505]	ELMO1	<---	RAC1	-0.038582	0.076038	-
[3506]	CACNB3	<---	PRKACB	-0.016941	0.18215	+
[3507]	GRM5	<---	KCNJ6	-0.25173	0.20895	+
[3508]	CCNE2	<---	CDC25A	-0.245	0.10994	+
[3509]	IL6	<---	MAPK11	-0.26091	0.40675	+
[3510]	DVL2	<---	MAX	-0.32974	0.23815	+
[3511]	INPPL1	<---	PRDM1	-0.032101	0.047784	+
[3512]	IFNG	--->	IL12B	0.19033	-0.47481	+
[3513]	GNA15	<---	HTR2B	-1.1303	1.1631	+
[3514]	CD79B	--->	POU2F2	0.079979	-0.1434	-
[3515]	CDC42	<---	FYN	0.16613	1.0122	+
[3516]	HRAS	--->	PIK3CD	0.0055787	-0.0073223	+
[3517]	KALRN	<---	RHOU	-0.021179	0.042607	-
[3518]	GSK3B	--->	NR3C1	0.29076	-0.30308	+
[3519]	E2F1	--->	RBL1	0.13728	-0.24843	-
[3520]	AKT1	--->	IL2RG	0.081851	-0.12459	-
[3521]	CXCR5	<---	JAK3	-0.12268	-0.039248	-
[3522]	CCL14	<---	CCR5	-0.35895	0.21658	-
[3523]	CD47	--->	THBS4	0.17768	-0.10726	-
[3524]	PRKD1	--->	SHC1	0.65574	-0.65265	-
[3525]	BUB1B	--->	PSMD7	0.2441	-0.083683	-
[3526]	ATF3	<---	WWP2	-0.30618	0.26884	-
[3527]	ETS2	--->	STAT3	0.26825	-0.19721	-
[3528]	BIRC3	--->	CASP9	0.072187	-0.29878	-
[3529]	CRKL	--->	IGF1	0.056841	-0.079809	-
[3530]	DAB1	--->	LRP8	0.34713	-0.25115	-
[3531]	DYRK1B	--->	REST	1.0991	-0.67282	-
[3532]	CAMK2A	<---	PPP1CA	-0.015111	0.02592	+
[3533]	DUSP6	--->	MAPK11	1.353	-1.036	+
[3534]	NFKB1	--->	TACR1	0.47827	-0.43941	+
[3535]	MET	--->	VEGFA	0.028553	-0.071698	-
[3536]	JUP	<---	MMP12	-0.26058	0.11034	+
[3537]	DUSP6	--->	MAPK13	1.353	-1.036	+
[3538]	EGR2	<---	FASLG	-0.16564	0.25366	-
[3539]	CCL5	<---	CXCR3	-0.35895	0.21658	-
[3540]	ETS1	--->	SPRY2	0.034853	-0.066747	+
[3541]	GRB2	<---	PRKCG	-0.33961	0.37448	-
[3542]	RARA	--->	STAT3	0.27087	-0.22437	+
[3543]	ARPC1B	--->	PDGFRB	0.050006	0.01194	-
[3544]	CFTR	<---	PRKAA2	-0.37336	0.33589	+
[3545]	FOS	<---	TIMP1	-0.16066	0.25678	-
[3546]	JAM2	<---	PPP2R1B	-0.30896	0.28522	+
[3547]	CREB1	<---	TIMP1	-0.098546	0.15465	-
[3548]	NFATC3	--->	RNF128	0.11084	-0.40394	+
[3549]	FGF6	<---	GRB2	-0.29505	0.31381	-
[3550]	EGLN3	<---	PRKCA	-0.91465	0.45016	+
[3551]	ETV1	<---	MAPK14	-0.26091	0.40675	+
[3552]	ARHGAP8	<---	RHOQ	-0.56218	0.37087	-
[3553]	ERBB2	--->	ETS1	0.14559	-0.13244	-
[3554]	CTSD	--->	USF2	0.26575	-0.31912	-
[3555]	F2	--->	SERPINC1	0.18568	-0.33063	-
[3556]	GNAT3	--->	GNB1	0.069944	-0.012773	+
[3557]	GNB1	<---	RGS1	-0.29846	0.26681	-
[3558]	CBLB	--->	IL4R	0.18092	-0.10684	+
[3559]	AKT1	--->	FAS	-0.074983	-0.15449	-
[3560]	FGF19	<---	HRAS	-0.15714	0.23194	-
[3561]	CASP10	--->	DAXX	0.12709	-0.6543	-
[3562]	PLXNB3	<---	SEMA4G	-0.16996	0.22939	+
[3563]	MAP3K1	--->	MAPK8	1.5096	-1.3549	-
[3564]	DAND5	--->	USF1	0.51954	-0.5536	+
[3565]	GRB2	--->	MRAS	0.63892	0.0030067	+
[3566]	FGF9	<---	MAPK1	-0.47959	0.71493	+
[3567]	MYO5A	<---	RHOQ	-0.046655	0.010173	+
[3568]	COL19A1	<---	FYN	-0.45391	0.63648	-
[3569]	IRS4	--->	PIK3CG	0.03178	-0.021301	+
[3570]	AKAP13	<---	RHOQ	-0.22586	0.072537	-

[3571]	ATF6B	<--->	FOSB	0.36709	0.36709	-
[3572]	CDKN1A	<--->	CDKN1B	0.037067	0.037067	-
[3573]	FLNB	<---	PTK2B	-0.11304	-0.10314	+
[3574]	LEF1	--->	TLE4	0.20358	0.0065975	-
[3575]	FLT4	--->	SHC1	0.23069	-0.27006	+
[3576]	PRKG1	--->	SLC8A3	0.32653	-0.52118	+
[3577]	AKT1	--->	GATA3	0.16383	-0.26287	+
[3578]	SGK1	<---	SRC	-0.2822	0.51839	+
[3579]	CCR8	--->	CXCL1	0.21658	-0.35895	-
[3580]	C2CD5	--->	MYO1C	0.11797	-0.29304	+
[3581]	ARHGAP19	<---	RHOH	-1.5245	1.016	-
[3582]	MYL9	<---	PXN	-0.46104	0.42006	-
[3583]	ITGA8	--->	TNR	-0.015417	-0.048468	-
[3584]	PLCG2	--->	SPRY4	0.055284	-0.026882	+
[3585]	CXCL5	<---	NFKB1	-0.74844	0.65878	+
[3586]	JUN	<---	TNFAIP3	-0.17011	0.12682	-
[3587]	EPHA2	<---	GRB2	-0.1135	-0.10862	-
[3588]	PRKCG	--->	RASA1	0.06376	0.037455	-
[3589]	CRK	<---	MAP3K1	-0.11539	0.11526	-
[3590]	NCOA3	<---	YY1	-0.017564	0.042925	+
[3591]	EPHB3	<---	LIMK1	-0.01801	0.025848	-
[3592]	PTK2	<---	RAP1B	-0.14524	-0.084837	-
[3593]	CAMK2D	--->	STMN1	0.30815	-0.21817	+
[3594]	ARF6	--->	RHOA	0.93356	-1.3488	+
[3595]	IL10RA	<---	IL6	-0.087402	0.20113	+
[3596]	CXCR5	--->	XCL1	0.21658	-0.35895	-
[3597]	JAK1	--->	UBE2L6	-0.010571	-0.11455	-
[3598]	GH2	--->	IFNAR2	0.20113	-0.087402	+
[3599]	GNB4	--->	PIK3R5	0.34477	-0.41795	+
[3600]	CDC42	--->	DLC1	0.10338	-0.50393	-
[3601]	SMAD3	--->	TFDP1	1.0055	-0.62586	+
[3602]	ERBB3	--->	PRKACA	0.086576	-0.027782	-
[3603]	DZIP1	--->	GLI3	0.048493	-0.037946	+
[3604]	CSNK1D	--->	CTNNB1	1.0802	-1.3965	+
[3605]	AKT1	--->	SLC2A5	0.34872	-0.44064	+
[3606]	ADRBK1	--->	CXCR2	0.25327	-0.21301	+
[3607]	PRKCZ	--->	RASA1	0.055429	-0.006492	-
[3608]	IL3	--->	IL5RA	0.022237	-0.016561	+
[3609]	HRAS	<---	YWHAZ	-0.15318	0.090199	-
[3610]	PPARG	--->	PPARGC1A	0.12459	-0.17843	+
[3611]	CTCFL	<---	CXXC1	-0.11143	0.24928	-
[3612]	GRK1	--->	OPRM1	0.33635	-0.28786	+
[3613]	FGFR2	--->	SHC3	0.029603	-0.2849	+
[3614]	CAV1	--->	INSR	0.2662	-0.022349	-
[3615]	AR	<---	MAPK8	-0.27508	0.26535	+
[3616]	CSF1	<---	STAT1	-0.30178	0.24651	+
[3617]	CBLB	--->	IL20RB	0.61825	-0.39145	+
[3618]	FYN	--->	SPTAN1	0.30667	0.01761	+
[3619]	ARAF	<---	MRAS	-0.86318	0.78219	+
[3620]	GLI3	<---	IFT57	-0.10941	0.27889	+
[3621]	MYB	--->	NOL3	0.21939	-0.23082	+
[3622]	COL3A1	<---	PCOLCE2	-0.15873	0.062969	+
[3623]	ARR3	--->	FZD6	0.063196	-0.073345	-
[3624]	MAPK1	--->	SPRY2	0.5089	-0.32626	+
[3625]	SETDB1	--->	ZNF416	0.16497	-0.10617	+
[3626]	NR3C1	--->	ZNF740	0.18046	-0.21501	+
[3627]	KLF6	--->	MEF2C	0.055487	-0.10325	-
[3628]	C4B	--->	FCN3	0.1408	-0.23228	-
[3629]	APC	<---	ARRB1	-0.012802	0.0026103	+
[3630]	FZD3	<---	WNT9A	-0.78209	0.63734	+
[3631]	IL18	<---	IL18RAP	-0.09224	0.11129	-
[3632]	ERBB3	--->	KRAS	0.28807	-0.18972	+
[3633]	TNF	--->	TNFRSF1B	1.0868	-1.3016	+
[3634]	CTSG	<---	F2R	-0.2522	0.11779	-
[3635]	CBLC	--->	PIK3R3	0.93154	-0.70449	+
[3636]	MAPK3	--->	WASF2	0.20642	-0.1186	+
[3637]	GRK5	--->	HTR7	0.33635	-0.28786	+
[3638]	FLT4	<---	PDGFA	-0.047197	0.01764	+
[3639]	AKT3	--->	CREB1	0.49706	-0.80518	+
[3640]	LPAR1	--->	NFKBIA	0.19531	-0.38696	+
[3641]	CEBPA	<---	HMGB1	-0.042674	0.055951	-
[3642]	MAP3K1	<---	TNIK	-0.54177	0.52161	+
[3643]	PPP2CA	<---	PPP2R1A	-0.019382	0.092045	+
[3644]	IL20RA	<---	IL6	-0.087402	0.20113	+
[3645]	BCL11A	<---	HOXC6	0.025818	0.11044	-
[3646]	AGT	<---	GATA1	-0.1986	0.12097	+

[3647]	ADRB1	--->	ARRB2	0.070342	-0.15406	-
[3648]	GNB1	--->	VAV1	0.067133	0.0011028	+
[3649]	RXRA	<---	SCD5	-0.23787	-0.11913	-
[3650]	ACTA2	<---	MMP21	-0.29263	0.085904	+
[3651]	GNAI2	<---	HTR1B	-1.1303	1.1631	+
[3652]	CSF1R	<---	FGF7	-0.64493	0.77791	+
[3653]	IL26	--->	IL5RA	0.11381	-0.12599	+
[3654]	DVL1	--->	NOTCH3	0.47526	-0.54738	+
[3655]	CRKL	--->	EP300	0.0041175	-0.0090595	-
[3656]	BDKRB1	--->	ETV4	0.11779	-0.2522	-
[3657]	ADCY4	<---	GNAI1	-0.63317	1.0384	-
[3658]	CIITA	--->	USF1	0.51937	-0.55916	-
[3659]	NR2C2	--->	ZNF595	0.18046	-0.21501	+
[3660]	CEBPB	--->	PXDN	-0.023435	-0.074931	+
[3661]	GNAI2	<---	RGS7	-0.736	0.71839	+
[3662]	CCNT2	<---	KLHL22	-0.26662	0.21637	-
[3663]	KLRD1	--->	SOS1	0.096929	0.043876	+
[3664]	PIK3CD	<---	PRKCI	-0.0097396	-0.0033944	-
[3665]	IL2RB	<---	IL6	-0.055821	-0.0077042	+
[3666]	FGF4	--->	INSR	0.25346	-0.0044024	+
[3667]	CEBPD	--->	CFTR	0.17048	-0.29315	+
[3668]	CCNA1	<---	CDKN1B	-1.0232	1.4682	+
[3669]	APP	--->	MEF2C	0.0074247	-0.027116	-
[3670]	GNAI1	--->	PLA2G4D	-0.10668	-0.13723	+
[3671]	EPAS1	--->	TEK	0.0013543	-0.024325	+
[3672]	LRP5	<---	WNT5B	-0.28769	0.39899	+
[3673]	IFNGR2	<---	IL10	-0.087402	0.20113	+
[3674]	RHOQ	--->	SYDE1	1.016	-1.5245	-
[3675]	CCNT2	<---	EDN1	-0.26662	0.21637	-
[3676]	IL12RB1	--->	STAT4	0.38886	-0.28644	+
[3677]	CALML3	--->	CAMKK2	0.41687	-0.23601	+
[3678]	IFNAR1	<---	LEP	-0.087402	0.20113	+
[3679]	PPP1R1A	--->	PPP3R2	0.068088	-0.035483	-
[3680]	NPY	--->	SUCNR1	0.89835	-1.3205	+
[3681]	ITGB1	<---	PTK2B	-0.04335	-0.039708	-
[3682]	CSF2RA	<---	SPI1	-0.055821	-0.0077042	+
[3683]	GH1	--->	IFNLR1	0.093836	-0.20115	+
[3684]	IL26	--->	LIFR	0.056231	-0.11334	+
[3685]	SH3KBP1	--->	SHC1	0.25644	-0.34336	+
[3686]	GAB1	<---	PIK3C2B	-0.042511	0.032177	+
[3687]	MYC	<---	SP1	-0.16882	0.033171	-
[3688]	DLL1	<---	RAB11A	-0.13461	0.14494	+
[3689]	MYO9A	<---	RHOJ	-0.54674	0.38058	-
[3690]	HSPB2	<---	MAPK14	-0.26091	0.40675	+
[3691]	IGKV4-1	<---	ITK	-0.07009	0.20824	-
[3692]	PSMD1	<---	RBX1	-0.2455	0.1425	-
[3693]	AKT3	<---	ERBB2	0.022566	0.027633	+
[3694]	ANGPT2	--->	FGFR4	0.086464	-0.29412	+
[3695]	FGF6	<---	FRS2	-0.28504	0.43716	-
[3696]	NUP153	--->	SMAD4	0.090481	-0.24089	+
[3697]	CREB1	<---	PSEN1	-0.080061	0.134	-
[3698]	GSK3B	--->	PSMD7	0.2441	-0.083683	-
[3699]	FIGF	--->	SOS1	0.10018	0.040597	+
[3700]	BTK	--->	SPI1	0.20777	-0.060744	-
[3701]	ITGA6	--->	THBS3	-0.014728	-0.021497	-
[3702]	KAT7	--->	YY1	0.28002	-0.15205	-
[3703]	PITX2	<---	SMAD3	-1.5172	1.4362	+
[3704]	CRKL	--->	RAP2A	0.43868	-0.057401	+
[3705]	ANGPT1	<---	TEK	-0.099458	0.049474	-
[3706]	EPHB4	--->	RHOA	0.039661	0.014723	+
[3707]	NDC80	<---	PLK1	0.08754	0.35317	+
[3708]	CAMKK2	--->	LEPR	0.3459	-0.30945	-
[3709]	FZD3	<---	WNT3	-0.78209	0.63734	+
[3710]	EBF1	--->	WNT7A	0.49239	-0.59554	+
[3711]	BIN1	--->	EP300	0.0020219	-0.0069494	-
[3712]	HDAC9	--->	MEF2A	-0.041005	-0.044156	-
[3713]	MOB1A	<---	YAP1	-0.77343	0.57923	-
[3714]	CREBBP	--->	NPPA	0.0055378	-0.0068093	+
[3715]	DCN	--->	EP300	0.0056421	-0.017531	-
[3716]	LRP8	<---	RAPGEF1	-0.17755	0.23663	-
[3717]	EP300	<---	RAB11A	0.00089005	0.00089974	-
[3718]	MEF2C	--->	THBS1	0.26935	-0.09813	+
[3719]	PLXNA1	<---	RAC1	-0.31182	0.50205	-
[3720]	CSF2	--->	IFNGR1	0.24886	-0.38103	+
[3721]	GNAI1	<---	LPAR1	-1.1303	1.1631	+
[3722]	PDGFRB	--->	PIK3R1	0.38672	-0.068382	+

[3723]	FGF16	<---	FRS2	-0.28504	0.43716	-
[3724]	GCK	<---	MAP3K2	-0.068211	0.35673	-
[3725]	AKT2	--->	TCF7L2	0.51894	-0.38826	-
[3726]	ERBB3	--->	GRB2	0.58449	-0.45971	+
[3727]	FZD4	<---	WNT3	-0.78209	0.63734	+
[3728]	EXOC8	<---	RAB10	-0.50681	0.59188	+
[3729]	CLASP1	--->	PLK1	0.33389	-0.195	-
[3730]	PLCG1	<---	PRKCG	-0.0064763	0.024762	-
[3731]	HIF1A	<---	TERT	0.061721	0.06932	-
[3732]	JAK2	--->	PDPK1	0.2569	-0.48656	+
[3733]	CCND1	<---	FOS	-0.24261	0.40828	+
[3734]	JUN	--->	SERPINB2	0.0072239	-0.13776	+
[3735]	CDC40	--->	CPSF7	0.31391	-0.17132	-
[3736]	LATS2	<---	LIMD1	-0.694	0.8544	+
[3737]	ARAP2	<---	RHOH	-0.48615	0.36733	-
[3738]	CRK	--->	HGF	0.050944	-0.07854	-
[3739]	COL4A4	--->	ITGA11	-0.013036	-0.10803	+
[3740]	CDH1	<---	MMP14	-0.24168	-0.0076207	+
[3741]	EDNRA	<---	MAPK8	-0.10024	0.19743	-
[3742]	FGR	--->	PTK2B	0.34449	-0.5161	-
[3743]	ADCY1	<---	PRKCG	-0.49495	0.42642	+
[3744]	EP300	<---	MAPK9	-0.006941	0.013728	-
[3745]	E2F1	<---	NR2C2	-0.17309	0.015585	-
[3746]	CCNE1	<---	FOXM1	-0.62791	0.70491	-
[3747]	ADRBK2	--->	CHRM3	0.25327	-0.21301	+
[3748]	MAPK8	--->	PLA2G4A	0.18871	-0.84715	+
[3749]	BIRC5	<---	TCF7L1	-0.21066	0.34564	+
[3750]	DVL2	<---	GNAQ	-0.59426	0.46386	+
[3751]	APP	<---	MAPK1	-0.023982	0.030823	-
[3752]	ITGA10	--->	TNN	-0.025002	-0.077063	-
[3753]	CTCF	<---	NACC1	-0.0091154	0.083205	-
[3754]	APC	<---	PSMD7	-0.027392	0.01494	+
[3755]	PPP1CC	<---	PRKG1	-0.70943	0.3441	+
[3756]	MAP3K5	--->	MAPK10	1.146	-0.99422	+
[3757]	GNAI1	<---	SSTR2	-1.1303	1.1631	+
[3758]	MAP3K1	--->	TNF	0.10374	0.07797	-
[3759]	ATR	--->	CDKN2D	0.089858	-0.067512	+
[3760]	EGFR	--->	NRAS	0.17363	-0.11617	+
[3761]	PPP2R1A	--->	PRKCB	0.0058797	-0.035074	+
[3762]	AR	<---	CDK11B	-0.27508	0.26535	-
[3763]	ARHGAP31	<---	RHOD	-1.5245	1.016	-
[3764]	CEBPB	<---	IL1B	-0.12163	0.053753	-
[3765]	CEBPA	--->	SREBF2	0.43996	-0.52432	+
[3766]	MAPK11	<---	PTPRR	-1.4016	1.2548	+
[3767]	ATF1	<---	RPS6KA4	-0.59606	0.7545	+
[3768]	FCGR1A	--->	VAV1	0.24729	0.06291	+
[3769]	ACVR1C	<---	TCF7L2	-0.050123	0.062	+
[3770]	GNAT1	<--->	GNAZ	0.064764	0.064764	-
[3771]	AR	<---	TCF4	-0.061507	-0.0037714	-
[3772]	PIK3C3	<---	PRKD1	-0.10258	0.14326	-
[3773]	FGD3	<---	RHOBTB1	-0.087343	0.096875	-
[3774]	ITGB3	<---	SRC	-0.16875	0.23238	-
[3775]	H1F0	--->	TP53	0.28315	-0.25752	-
[3776]	IL1A	--->	ZEB1	0.22459	-0.189	-
[3777]	SIK1	<--->	STRADB	0.31014	0.31014	-
[3778]	FGF12	--->	PDGFRA	0.68139	-0.83032	+
[3779]	MAP4K4	--->	TNFRSF1A	0.22129	-0.26473	-
[3780]	KRIT1	<---	RAP1B	-1.0084	0.071276	+
[3781]	NFIB	--->	POU2F1	0.34916	-0.46338	+
[3782]	FRZB	--->	WNT4	1.0317	-1.1063	+
[3783]	CTNNB1	--->	DVL1	0.22164	-0.31127	-
[3784]	PDE4A	<---	PRKX	-0.95055	1.4803	+
[3785]	ITGA11	<---	LAMC1	-0.041507	-0.0065623	+
[3786]	AKT3	<---	FGFR3	-0.040847	0.067461	+
[3787]	KDR	--->	PIK3CB	0.043149	-0.0082223	+
[3788]	ARHGEF10L	<---	RHOA	-0.012974	0.047696	-
[3789]	CD79A	<---	SH3KBP1	-0.26912	0.20811	-
[3790]	ANAPC2	<---	PPP3CB	-0.39733	0.17898	+
[3791]	MAPK12	--->	TP53	0.31197	-0.21427	+
[3792]	FGF7	--->	FGFR4	0.40218	-0.67149	+
[3793]	PPP2CB	--->	RELA	0.70818	-0.71935	-
[3794]	FZD1	<---	SFRP1	-1.3633	0.73901	+
[3795]	FES	<---	PLXNA4	-0.10012	-0.046212	-
[3796]	EGFR	--->	FGF3	0.25979	-0.22928	-
[3797]	IRAK1	<---	MYD88	-0.6873	0.026112	+
[3798]	PAK4	<---	RAC3	-1.1901	0.96651	+

[3799]	FGA	--->	SERPIND1	0.27611	-0.51388	-
[3800]	KCNC2	--->	REST	0.1257	0.025162	-
[3801]	EPHA2	<---	VEGFA	-0.064113	0.021204	+
[3802]	NEK6	--->	NUP153	0.17193	-0.084754	+
[3803]	KLF1	--->	TAL1	0.33079	-0.20142	-
[3804]	E4F1	--->	TP53	0.1064	-0.015277	+
[3805]	MAPK3	--->	PLA2G4E	0.18871	-0.84715	+
[3806]	ADCY2	<---	CAMK2D	-0.44346	0.58749	+
[3807]	EGFR	--->	SPRY2	0.27575	-0.22962	-
[3808]	CREBBP	<---	STAT1	-0.018478	0.010999	-
[3809]	IL20	--->	IL20RA	0.20113	-0.087402	+
[3810]	CCL1	<---	CXCR1	-0.35895	0.21658	-
[3811]	PLCD1	<---	RGS17	-0.082548	0.03305	+
[3812]	IL12B	--->	STAT5A	0.38886	-0.28644	+
[3813]	DUSP19	--->	MAPK11	1.4184	-1.4202	+
[3814]	BMP1	<---	COL1A2	-0.19386	0.13754	-
[3815]	ARHGEF18	<---	RHOJ	-0.012974	0.047696	-
[3816]	BCAR1	--->	PDGFRB	0.029256	-0.0078377	-
[3817]	GNAQ	--->	WNT4	0.42087	-0.47341	-
[3818]	HDAC1	<---	ZBTB17	0.041167	0.09489	+
[3819]	CD3G	<---	LCP2	-0.14641	-0.021938	-
[3820]	CD44	<---	COL27A1	-0.18268	0.0085485	+
[3821]	ADCY1	<---	CAMK2D	-0.44346	0.58749	+
[3822]	CREB3	<---	MAPK3	-0.57905	1.0858	+
[3823]	SAG	--->	WNT3	0.23514	-0.34616	+
[3824]	MAP2K2	--->	PRKCD	-0.20738	-0.21837	-
[3825]	GRIK2	<---	RAC3	-0.38938	0.54247	-
[3826]	CBLB	--->	IL23R	0.17024	-0.087588	+
[3827]	FZD5	<---	GNB3	-0.12321	0.10912	-
[3828]	DDB2	--->	TP53	0.12626	-0.081433	-
[3829]	GNAT3	<---	OPRM1	-1.1303	1.1631	-
[3830]	FGR	--->	RGS1	0.53308	-0.35367	+
[3831]	HNF4G	<---	MYB	-0.043716	-0.0086978	-
[3832]	MYH10	<---	RHOC	-0.01596	0.026206	+
[3833]	ARHGAP17	<---	RHOF	-0.92575	0.61511	-
[3834]	KRAS	<---	PDGFRB	-0.2264	0.48853	+
[3835]	GNAQ	--->	WNT7A	0.42087	-0.47341	-
[3836]	CCL19	<---	NFKB2	-0.88578	0.76708	+
[3837]	AKT2	<---	PPP2R2A	-0.64728	0.4828	+
[3838]	MYH6	<---	THRA	-0.056504	-0.0074276	+
[3839]	IRS1	--->	ITGAV	0.047617	-0.048171	-
[3840]	LAT	--->	VAV3	0.070415	-0.030274	+
[3841]	IL26	--->	IL6ST	0.056231	-0.11334	+
[3842]	CDKN1A	<---	SKP2	-0.12595	-0.019483	-
[3843]	COL2A1	--->	ITGB6	0.0024845	-0.035647	+
[3844]	FZD5	<---	WNT7A	-0.78209	0.63734	+
[3845]	GNAO1	--->	PIK3R3	0.19122	-0.22979	+
[3846]	RPS13	--->	RPS6	0.25527	-0.23161	+
[3847]	EIF3B	<---	HNF4A	-0.31347	0.23448	+
[3848]	MYL7	--->	ROCK1	0.31451	-0.08875	-
[3849]	CDC42	--->	PAK1	0.96651	-1.1901	-
[3850]	HRAS	<---	RASGRP2	-0.1488	0.16462	+
[3851]	EDN1	<---	EDNRB	-0.2522	0.11779	-
[3852]	ADCYAP1R1	--->	NTRK1	0.081601	-0.26088	+
[3853]	AKT1	--->	APAF1	0.30391	-0.34088	+
[3854]	PPP2R2A	--->	YAP1	-0.090891	-0.11911	+
[3855]	DTL	<---	E2F1	-0.046997	0.060123	+
[3856]	LIPE	<---	PRKACB	-0.43325	0.64376	+
[3857]	DLG3	--->	GRIK2	0.19945	-0.095793	-
[3858]	ADAMTS3	--->	COL11A1	-0.02131	-0.035774	+
[3859]	EIF4E	<---	NUP43	-0.37256	0.38207	+
[3860]	EGLN3	--->	EP300	0.00036539	-0.0062955	-
[3861]	GNA15	<---	RGS13	-1.2531	1.2063	+
[3862]	ITCH	--->	TRIM25	0.065399	-0.021483	+
[3863]	FZD6	<---	WNT3A	-0.30406	0.25758	+
[3864]	HIF1A	<---	PIK3R3	0.0059242	0.14781	+
[3865]	EP300	<---	NCOR2	-0.0074211	0.0056463	-
[3866]	AKAP13	<---	RHOF	-0.22586	0.072537	-
[3867]	CXCL12	<---	GNAZ	-0.054136	0.087289	+
[3868]	ATF1	--->	CCNA1	0.24653	-0.14825	+
[3869]	GNAO1	<---	MAPK8	-0.22953	0.50225	-
[3870]	HDAC2	<---	NACC1	-0.31114	0.37744	-
[3871]	FGF17	<---	SOS1	0.067334	0.085583	-
[3872]	PDK3	--->	PPARD	0.16681	-0.28815	-
[3873]	ADRBK2	--->	GRM8	0.53935	-0.4166	+
[3874]	CREB3L4	<---	PRKG1	-1.2233	1.1994	+

[3875]	CALML3	<---	PPP1CC	-0.26348	0.069904	+
[3876]	MYD88	--->	SOCS1	0.26105	-0.47362	-
[3877]	MAPK12	--->	NOS2	0.088587	-0.060388	+
[3878]	IQCB1	<---	RAB8A	-0.38228	0.36949	-
[3879]	HRAS	--->	PLD1	0.077686	-0.071006	+
[3880]	KLF9	--->	ZNF263	-0.037992	-0.039841	-
[3881]	NOD2	<---	TNFAIP3	-0.13292	0.015399	+
[3882]	ACVR2B	--->	E2F1	0.19006	0.038453	-
[3883]	PIK3R1	<---	TXK	-0.27345	0.28568	-
[3884]	LIMK1	--->	PLXNA1	0.24614	-0.21791	-
[3885]	CREB1	<---	IL1B	-0.1182	0.14174	-
[3886]	CCNB1	<---	KIF2C	-0.40062	0.4569	-
[3887]	DLAT	<---	PDK3	-0.5004	0.41454	+
[3888]	ITGB8	--->	TNR	0.053378	-0.13145	-
[3889]	IGKV1-5	--->	VAV1	0.072612	0.033678	+
[3890]	BAD	<---	YWHAZ	-0.92512	0.50537	+
[3891]	EP300	<---	PPP5C	-0.015462	0.0034359	-
[3892]	GNA15	--->	GRM5	0.24033	-0.15623	-
[3893]	ACTB	<---	ARHGAP10	-0.17746	0.10822	+
[3894]	SRC	--->	TCF7L2	0.40966	-0.22346	-
[3895]	CCDC85B	--->	CTCF	0.083205	-0.0091154	-
[3896]	HDAC1	<---	SMAD5	-0.21285	0.15191	-
[3897]	CTSG	<---	F2RL1	-0.2522	0.11779	-
[3898]	ANGPT2	--->	STAT5B	0.43125	-0.31862	+
[3899]	FGFR2	<---	INS	-0.40644	0.26408	+
[3900]	NUP153	--->	SRSF4	0.60094	-0.54167	+
[3901]	BRCA1	<---	CHD2	-0.074281	-0.00087986	+
[3902]	PIK3CA	--->	VAV1	0.0074012	0.0024242	-
[3903]	AMPH	--->	PIK3CA	0.01258	-0.024691	-
[3904]	EGR1	<---	SPIB	-0.15205	0.28002	-
[3905]	CCND1	<---	JUP	-0.034577	0.033828	+
[3906]	IGF1	--->	PLG	0.031228	-0.09545	-
[3907]	BRAF	--->	MAPK11	0.99795	-1.3266	+
[3908]	MYC	<---	TFAP4	-0.30347	-0.19793	-
[3909]	GNAO1	<---	RGS4	-1.2531	1.2063	+
[3910]	MAPK14	<---	RAC1	-0.20304	1.2491	+
[3911]	CCKAR	--->	GNA11	1.1631	-1.1303	+
[3912]	PECAM1	<---	YES1	-0.94853	1.1589	+
[3913]	BTK	--->	VAV2	0.11535	-0.012489	-
[3914]	FRS3	--->	SOS1	0.11735	0.037859	+
[3915]	NF1	<---	PRKCE	-0.24229	0.18612	-
[3916]	MYO9B	<---	RHOBTB1	-0.33271	0.22974	-
[3917]	ITGAV	<---	MAP3K1	-0.1096	0.11897	-
[3918]	CARD11	<---	UBE2N	-0.28845	0.3507	-
[3919]	IL13RA1	<---	TYK2	-0.38081	0.23745	-
[3920]	PLXNA1	<---	RAC2	-0.31182	0.50205	-
[3921]	MYB	--->	NRAS	0.037027	-0.021494	+
[3922]	SIX5	--->	ZNF689	0.11044	0.025818	+
[3923]	GAB2	<---	SHC1	-0.29804	-0.087237	+
[3924]	COL18A1	--->	NFIC	-0.015308	-0.076406	-
[3925]	EXOC8	<---	RAB14	-0.50681	0.59188	+
[3926]	CXCL5	<---	CXCR2	-0.23229	0.16688	-
[3927]	CREB3L3	<---	PPP1CB	-1.0855	0.69008	+
[3928]	ARHGAP22	<---	RHOT2	-0.37357	0.26247	-
[3929]	ABCG1	<---	EP300	-0.0090562	0.0040014	+
[3930]	CCR7	--->	CXCL13	0.21658	-0.35895	-
[3931]	FAM13B	<---	RHOV	-1.5245	1.016	-
[3932]	CSF2	--->	CSF2RB	0.16398	-0.10767	+
[3933]	PRKCG	--->	PTK2B	0.09356	-0.1078	+
[3934]	PBX3	<---	TFCP2L1	-0.24396	0.15908	-
[3935]	FZD10	<---	WNT7B	-0.78209	0.63734	+
[3936]	NCOA2	--->	SREBF1	-0.066532	-0.08315	+
[3937]	IRAK1	--->	RELA	1.0385	-1.0161	+
[3938]	HNRNPUL1	<---	TCF7L2	0.08213	0.10134	+
[3939]	GNA14	--->	WNT9B	0.42087	-0.47341	-
[3940]	CSF2	<---	PDPK1	-0.079019	0.54764	-
[3941]	GRK1	--->	HTR4	0.33635	-0.28786	+
[3942]	EPHA1	<---	RASA1	-0.09886	-0.072416	-
[3943]	CCL5	<---	CCR6	-0.35895	0.21658	-
[3944]	EIF4E1B	<---	MKNK2	-0.73366	0.88689	+
[3945]	LIMK1	--->	PAK4	0.52276	-0.38894	-
[3946]	CSNK1G2	--->	FZD2	0.33941	-0.31414	-
[3947]	IL7R	--->	SOCS7	-0.16125	-0.20399	-
[3948]	BATF3	<--->	FOS	0.36709	0.36709	-
[3949]	ITGB3	--->	RELN	-0.015239	-0.019247	-
[3950]	IKZF3	--->	JUND	0.054205	0.022163	-

[3951]	INSR	--->	SOS1	0.084932	-0.0020396	+
[3952]	MAPK3	--->	NOS2	0.088587	-0.060388	+
[3953]	CRTC2	--->	PRKAA1	0.23894	-0.32874	-
[3954]	FOS	--->	ZNF689	0.16095	0.085078	+
[3955]	AKT3	--->	FRS3	0.18047	-0.25572	-
[3956]	COL2A1	--->	ITGA4	0.0052169	-0.090529	+
[3957]	CCR3	--->	PPBP	0.21658	-0.35895	-
[3958]	ELMO1	<---	RHOG	-0.038582	0.076038	+
[3959]	IL12RB2	<---	SOCS7	-0.06215	-0.055475	+
[3960]	GLI1	--->	HDAC1	0.15826	0.069686	-
[3961]	MMP13	<---	NFKB1	-0.17897	0.14673	+
[3962]	ERBB2	--->	PIK3R1	0.59225	-0.30243	+
[3963]	CACNA1S	<---	PRKACG	-0.38391	0.51195	+
[3964]	JAK1	--->	MAPK14	0.43202	-1.1344	+
[3965]	CIITA	--->	CREB1	0.20282	-0.11968	-
[3966]	IL20RA	<---	SOCS4	-1.0456	0.57217	+
[3967]	CTLA4	<---	SRC	-0.47931	0.63774	+
[3968]	ARF1	--->	ASAP2	0.012792	-0.060803	-
[3969]	MTOR	<---	RPTOR	-0.09063	-0.010081	+
[3970]	CTNNB1	<---	PCDHA1	-0.19204	0.11503	-
[3971]	NTRK1	<---	PRKAR2B	-0.5916	0.25472	+
[3972]	RAB8A	<---	TBC1D4	-0.77234	1.3656	+
[3973]	ERBB4	<---	PRKCA	-0.085576	0.11629	-
[3974]	OCRL	<---	RHOH	-0.92575	0.61511	-
[3975]	PDPK1	--->	PRKCZ	-0.022758	-0.478	+
[3976]	IL27RA	--->	STAT2	0.38886	-0.28644	+
[3977]	GLI3	--->	SUFU	0.14383	-0.059407	-
[3978]	MAGI2	<---	PTEN	-0.03035	0.081348	-
[3979]	BRAF	<---	PRKCD	-0.36938	0.20778	+
[3980]	CCR7	--->	POU2F2	0.31975	-0.45327	-
[3981]	JAK1	--->	MAPK9	0.43202	-1.1344	+
[3982]	RACGAP1	<---	RHOG	-1.011	0.68428	-
[3983]	DDX17	--->	MEF2A	0.080448	-0.18921	-
[3984]	CHRM1	--->	PRKCZ	-0.029433	-0.05892	+
[3985]	MEF2A	<---	TRAF3	-0.47781	0.49778	-
[3986]	CCL28	<---	CXCR2	-0.35895	0.21658	-
[3987]	CD3D	--->	TCRA	-0.033535	-0.068284	+
[3988]	JAK1	--->	SOCS6	1.1559	-1.1639	-
[3989]	DAPK1	--->	EP300	0.0097451	-0.011095	-
[3990]	ARF1	<---	ARFGAP3	-0.13279	-0.12595	+
[3991]	DCN	--->	MMP2	0.11468	-0.31874	-
[3992]	MAPK3	--->	YWHAB	0.079981	-0.071802	-
[3993]	ACTR1A	--->	PLK1	0.22192	-0.029158	-
[3994]	DVL1	<---	ROR2	-0.14847	0.06035	+
[3995]	ACVR1B	<---	INHBB	-0.38171	0.71751	+
[3996]	EIF4EBP1	<---	PPP3CC	-0.21908	0.090291	+
[3997]	IL11RA	<---	IL6	-0.14339	0.084246	+
[3998]	F10	<---	HNF4A	-0.1632	0.10486	+
[3999]	AXIN1	--->	PSMD10	0.08645	-0.095154	-
[4000]	IL10RA	<---	SOCS1	-0.40298	0.060066	+
[4001]	ENO1	--->	PBX3	0.095387	-0.14907	-
[4002]	RBBP7	<---	SMAD4	-0.083613	-0.016256	-
[4003]	MYL6B	<---	SORBS1	-0.20496	0.19742	-
[4004]	IL1R1	--->	MAP3K3	0.61925	-0.24738	+
[4005]	SRC	--->	TLN1	0.054004	-0.040082	+
[4006]	GNAQ	<---	PLCG1	-0.0052535	0.030885	-
[4007]	ITGA1	<---	LAMA4	-0.1017	-0.0065074	+
[4008]	SMAD5	<---	ZFYVE16	-0.063591	-0.031512	+
[4009]	NR3C1	--->	SERPINE1	0.21298	-0.44731	+
[4010]	AVPR1B	--->	GNAQ	1.1631	-1.1303	+
[4011]	RPS6KA6	--->	SRF	0.12973	-0.44125	+
[4012]	CNTF	--->	CSF2RB	0.076998	-0.10334	+
[4013]	PRKAA2	<--->	STRADA	0.31014	0.31014	-
[4014]	MAP2K2	--->	STAT3	1.4162	-0.966	+
[4015]	AKT1	--->	ESR1	0.7641	-0.82222	-
[4016]	PPP1R12C	<---	PRKG1	-1.0415	0.74248	+
[4017]	KCNJ9	<---	OPRK1	-0.2522	0.11779	-
[4018]	COL1A2	<---	PCOLCE	-0.32363	0.21278	+
[4019]	GHSR	--->	GNAI1	1.1631	-1.1303	+
[4020]	RASA1	--->	RET	-0.12691	-0.16451	-
[4021]	PRKCE	--->	RASAL2	0.14565	-0.025686	-
[4022]	ARHGAP4	<---	RHOJ	-0.42732	0.39011	-
[4023]	AKT1	<---	MTCP1	-1.4472	1.0764	+
[4024]	E2F1	<---	TEAD1	-0.41605	0.3621	-
[4025]	MAP3K1	--->	MAPK14	1.5096	-1.3549	+
[4026]	SETDB1	--->	ZIM2	0.16497	-0.10617	+

[4027] EGF <--- RAB5A -0.15628 0.18542 +
[4028] ADAM17 <--- PKN2 -0.064546 0.023414 +
[4029] PAK2 <--- RAC2 -1.1901 0.96651 +
[4030] ARHGAP4 <--- RHOT1 -0.13505 0.10836 -
[4031] HGF <--- KIT -0.053062 0.073425 -
[4032] MAPK3 <--- RHOA -0.20304 1.2491 +
[4033] GH2 ---> IL2RG 0.056231 -0.11334 +
[4034] GABBR1 <--- GNAO1 -0.15623 0.24033 -
[4035] JUP <--- MMP15 -0.26058 0.11034 +
[4036] GRIN1 <--- GRM5 -0.36981 -0.05834 +
[4037] CDC42 ---> GIT1 0.02238 -0.11262 -
[4038] COL6A6 ---> ITGA5 -0.0067115 -0.019363 +
[4039] DVL1 <--- WNT1 -0.54867 0.38842 +
[4040] GNAI1 <--- RGS2 -1.2531 1.2063 +
[4041] CACNA1C <--- KCNMB1 -0.49348 0.54975 +
[4042] ERBB2 ---> PTK2 0.12444 -0.041656 +
[4043] SOCS1 ---> STAT4 0.27422 -0.74698 -
[4044] SETDB1 ---> ZNF3 0.16497 -0.10617 +
[4045] DKK1 ---> WNT4 0.54164 -0.69755 +
[4046] ECT2 <--- RHOF -0.012974 0.047696 -
[4047] LAT <--- PRKCQ -0.32584 0.23116 -
[4048] CXCR4 ---> HGS 0.024111 -0.058444 -
[4049] EPOR ---> PLCB1 -0.0068049 -0.023786 +
[4050] BCL11A <--- GSC 0.025818 0.11044 -
[4051] GNB2 <--- OPRM1 -0.92616 1.1575 +
[4052] FGB ---> PLAT -0.0063682 -0.035752 -
[4053] PLA2G4D <--- RASGRP1 -0.029717 0.042847 +
[4054] CDKN2C <--- E2F1 -0.37456 0.50683 +
[4055] BCAR1 <--- PRKCA -0.045495 0.027208 +
[4056] ETS1 <--- TLR4 -0.26334 0.01863 -
[4057] GRB2 ---> IL12RB1 0.22719 -0.31251 -
[4058] CXCL12 <--- GNAI2 -0.054136 0.087289 +
[4059] ATF2 ---> HIST1H2BL 0.048146 0.0081543 +
[4060] ACTG1 <--- WAS -0.22029 0.18415 +
[4061] PLCG1 ---> VAV3 0.0032846 -0.00078332 +
[4062] MAPK9 ---> NR3C1 0.29076 -0.30308 +
[4063] EGLN3 <--- PRKCG -0.91465 0.45016 +
[4064] CTNNB1 <--- CTTN -0.34984 0.2815 -
[4065] ACTA1 <--- FLT1 -0.10431 0.39409 +
[4066] JUN <--- TNC -0.040107 -0.01172 -
[4067] JUND ---> THBS1 0.27574 -0.094925 +
[4068] BRCA1 ---> E2F4 0.36011 -0.36552 -
[4069] PDE4A <--- PRKACG -0.95055 1.4803 +
[4070] E2F4 <--- NEK2 -0.30098 0.45782 -
[4071] JUP <--- MMP27 -0.26058 0.11034 +
[4072] ENG <--- SMAD7 -0.12048 -0.021443 +
[4073] ADAM12 ---> DLL1 0.08448 -0.11694 +
[4074] JAK2 <--- SOCS4 -0.73315 0.53539 +
[4075] DKK4 ---> WNT8B 0.54164 -0.69755 +
[4076] CASP8 <--- TRAF2 -0.11766 -0.051288 -
[4077] LAMB3 ---> SV2C 0.27773 -0.3202 +
[4078] GNAI2 ---> GRM2 0.24033 -0.15623 -
[4079] AKT3 <--- PPP2R3A -0.7767 0.80883 +
[4080] PIK3CB ---> PTEN 0.019177 -0.039333 -
[4081] GATA2 ---> ZEB1 0.29124 -0.21528 +
[4082] NCK1 ---> PTK2 -0.03525 -0.37576 -
[4083] LTA ---> TNFRSF1A 0.191 -1.2526 +
[4084] ARHGAP10 <--- RAC3 -0.65972 0.48549 -
[4085] ATP1B1 <--- MAPK1 -0.26091 0.40675 +
[4086] MYB ---> NCL 0.21283 -0.17725 +
[4087] GNA11 ---> WNT1 0.42087 -0.47341 -
[4088] ASCL1 <--- POU2F2 -0.32236 0.23888 +
[4089] MAPKAP1 ---> TSC2 -0.015952 -0.07411 -
[4090] CBL ---> MYC 0.1127 -0.09013 +
[4091] PLD2 <--- SPHK2 -0.057765 0.061811 -
[4092] CTSG ---> MMP1 -0.0056809 -0.18543 +
[4093] BMP2 <--- GLI2 -0.76749 1.0844 +
[4094] MAPK7 <---> MKNK1 0.31014 0.31014 -
[4095] ADRA2A ---> TRPC6 0.202 -0.26712 +
[4096] COL2A1 ---> ITGA6 0.0052169 -0.090529 +
[4097] IL10RB <--- LEP -0.087402 0.20113 +
[4098] ADRB3 <--- GRK4 -0.28786 0.33635 +
[4099] GATA2 ---> HOXA3 0.15908 -0.24396 +
[4100] ACTR2 <--- MMP1 -0.29263 0.085904 +
[4101] ATF2 ---> HIST2H2BF 0.048146 0.0081543 +
[4102] MEF2A <--- WWOX -0.47884 0.21759 -

[4103]	GABPA	<---	SKAP1	0.036217	0.038495	-
[4104]	EPHB2	<---	LIMK1	-0.01801	0.025848	-
[4105]	FOS	<---	PRKCG	-0.63701	0.72046	+
[4106]	EGF	<---	MAPK1	-0.20589	0.25921	+
[4107]	TNFRSF10B	<---	TNFSF10	-1.2526	0.191	+
[4108]	HSPG2	<---	MMP14	-0.00099974	0.0010462	+
[4109]	ITK	--->	PIK3R2	0.039717	-0.090918	-
[4110]	RALA	<--->	RHOQ	0.1628	0.1628	-
[4111]	ESR1	--->	SMAD3	-0.024404	-0.1374	-
[4112]	RELA	<---	TLR2	-0.54488	0.15126	+
[4113]	FXYD2	<---	PRKG1	-0.85748	0.54017	+
[4114]	EP300	<---	NR3C1	-0.0067479	-0.0035614	+
[4115]	FOS	<---	WASF2	-0.13773	0.1954	-
[4116]	MAPK3	<---	RHOG	-0.20304	1.2491	+
[4117]	ACTA2	<---	MYH11	-0.19158	0.14703	+
[4118]	HLA-DQA2	--->	LCP2	-0.050519	-0.20169	+
[4119]	CDH3	<---	MMP15	-0.24168	-0.0076207	+
[4120]	CD3G	--->	PIK3R1	0.049293	-0.12511	+
[4121]	PPP3R1	<---	YWHAZ	-0.13033	-0.078387	-
[4122]	MET	--->	PIK3R5	0.42849	-0.53992	+
[4123]	ADORA2A	--->	KCNJ5	0.11779	-0.2522	-
[4124]	SOCS3	--->	STAT2	0.27422	-0.74698	-
[4125]	BTRC	--->	WWTR1	0.76809	-0.28452	+
[4126]	HIF1A	<---	PIK3R2	-0.027171	0.066539	+
[4127]	PIK3R5	<---	RAC2	-0.55672	0.51887	-
[4128]	MYD88	--->	VDR	0.17757	-0.2984	+
[4129]	CTCF	--->	STAT3	0.18082	-0.10898	+
[4130]	ARHGEF7	<---	CDC42	-0.044321	0.055318	-
[4131]	GRB2	--->	IL16	0.16474	-0.13646	-
[4132]	DKK1	--->	WNT10A	0.54164	-0.69755	+
[4133]	CDC42	--->	DOCK11	-0.050286	-0.28582	-
[4134]	GNA14	<---	TACR3	-1.1303	1.1631	+
[4135]	JUN	<---	ZKSCAN2	-0.025299	0.094536	-
[4136]	ITGA2B	<---	RAP1B	-0.10645	0.030711	-
[4137]	SMAD5	<---	UBE2D3	-0.20131	0.05544	+
[4138]	CNTN6	<---	NCSTN	-0.40491	0.26173	+
[4139]	FZR1	--->	HIST2H3A	0.15811	-0.17998	-
[4140]	MTA1	--->	TBP	0.22839	-0.2383	+
[4141]	BCL11A	--->	ID1	0.19618	-0.059615	+
[4142]	MTOR	<---	RELA	-0.26357	0.34797	-
[4143]	GNGT2	<---	HTR1F	-0.26888	0.10335	+
[4144]	PPP1R1A	--->	PPP2R2A	0.058373	-0.026453	-
[4145]	TCF7L1	--->	TLE2	0.20358	0.0065975	-
[4146]	DAND5	--->	POU5F1	0.23866	-0.27323	+
[4147]	ATP1A1	--->	PIK3R1	0.016367	-0.047791	-
[4148]	MAPK12	<---	PPP2R1A	-0.25679	0.26693	-
[4149]	CXCL2	<---	XCR1	-0.35895	0.21658	-
[4150]	CCR1	--->	GNAI2	1.1631	-1.1303	+
[4151]	RHOG	--->	TIAM1	0.20928	-0.13672	-
[4152]	EPAS1	--->	SLC2A1	0.12974	0.0039232	+
[4153]	PLEKHG5	<---	RHOG	-0.012974	0.047696	-
[4154]	AGT	<---	DAND5	-0.34338	0.24953	+
[4155]	MAPK12	--->	RPS6KA5	0.9519	-1.129	+
[4156]	HSPA9	<---	TLR2	-0.18128	-0.072839	-
[4157]	ACVR1	--->	MAPK14	0.36409	0.12889	+
[4158]	GLI1	--->	WNT7B	0.86373	-1.47	+
[4159]	CXCL9	<---	CXCR2	-0.35895	0.21658	-
[4160]	GABBR1	<---	GNGT2	-0.26617	0.19226	-
[4161]	EPN1	--->	PPP3CC	-0.021139	-0.11794	-
[4162]	SMAD2	--->	TGIF2	-0.024788	-0.1488	+
[4163]	FOSL1	--->	JUP	0.27062	-0.26748	+
[4164]	RHOT2	<---	TRIP10	-0.16531	0.32578	+
[4165]	CREB1	--->	PCK1	0.037806	0.031112	+
[4166]	RAP2B	<---	RAPGEF1	-0.72612	0.70033	+
[4167]	CREB3L2	<---	PBX3	0.041297	0.055709	+
[4168]	COL11A1	--->	ITGA5	-0.013036	-0.10803	+
[4169]	CCL7	<---	CXCR6	-0.35895	0.21658	-
[4170]	CALML5	<---	PPP1CB	-0.26348	0.069904	+
[4171]	KIT	<---	PGF	-0.099588	0.12565	+
[4172]	POU2F1	<---	PSG1	-0.28647	0.2348	-
[4173]	GRK1	--->	HRH2	0.33635	-0.28786	+
[4174]	CD3D	<---	PRKCQ	-0.33821	0.25182	-
[4175]	OBSCN	<---	RHOF	-0.11324	0.22832	-
[4176]	E2F1	--->	HNRNPAB	0.32902	-0.26332	+
[4177]	DAND5	<---	HTRA1	0.0083549	0.010037	-
[4178]	CAMK2A	--->	EP300	0.010341	-0.008238	+

[4179]	STAT3	<---	UBE2D3	-0.35949	0.48292	-
[4180]	PPP3R1	<---	YWHAE	-0.13033	-0.078387	-
[4181]	GNRHR	--->	KCNJ6	0.11779	-0.2522	-
[4182]	PKN1	--->	SPI1	0.1626	-0.23515	-
[4183]	GRK6	--->	HTR1E	0.33635	-0.28786	+
[4184]	EGF	<---	EPN1	-0.15724	0.15888	+
[4185]	CD47	--->	THBS2	0.25377	-0.17387	-
[4186]	ACVR2A	--->	ZFYVE16	0.09539	0.036963	+
[4187]	CACNA1I	--->	GRIN2B	0.37674	-0.2845	-
[4188]	GRK6	--->	GRM8	0.80802	-0.62351	+
[4189]	NCK2	--->	RAC2	0.56021	-0.18722	+
[4190]	DAND5	--->	PSEN1	0.067181	-0.1062	+
[4191]	PGR	--->	PIK3CD	-0.0056486	-0.021852	+
[4192]	GNAI2	<---	TBXA2R	-1.1303	1.1631	+
[4193]	PRKCZ	--->	STAT6	0.32545	-0.30209	-
[4194]	HOXD3	<---	ITGB3	-0.091513	0.056756	-
[4195]	CD3D	--->	CD4	0.049998	-0.088072	-
[4196]	AJUBA	--->	SREBF1	0.95674	-0.91199	-
[4197]	KPNB1	<---	NUP37	-0.35456	0.3165	+
[4198]	JUN	<---	KCNIP3	-0.16866	0.13635	-
[4199]	SHC2	<---	ZAP70	-0.71996	0.36756	+
[4200]	CXCL11	<---	CXCR1	-0.35895	0.21658	-
[4201]	AKT2	--->	FGFR4	0.053523	-0.092062	-
[4202]	KSR1	--->	MAPK1	0.77296	-0.77096	-
[4203]	PBX3	<---	SETD8	-0.3757	0.33229	-
[4204]	ITGB5	--->	TNR	0.01787	-0.050125	-
[4205]	GAB1	<---	NRAS	-0.3085	0.35789	-
[4206]	CAMK2D	--->	PIK3C3	0.12615	-0.096379	+
[4207]	ITGB3	--->	PLCB2	-0.0044033	-0.010741	+
[4208]	BUB1B	--->	CCNB1	0.75243	-0.55661	-
[4209]	MAPK14	--->	TCF3	0.99789	-0.87887	+
[4210]	FN1	--->	ITGA1	-0.04	-0.079323	+
[4211]	CDK7	--->	TAF1	0.40675	-0.26091	+
[4212]	VAV3	<---	VEGFA	-0.016236	0.042925	+
[4213]	KREMEN2	--->	WNT8B	0.3638	-0.57716	+
[4214]	HIP1	--->	PBX3	0.15908	-0.24396	-
[4215]	BIRC3	--->	CASP2	0.072187	-0.29878	-
[4216]	EGR1	<---	RELA	-0.57388	0.50851	-
[4217]	INSR	<---	PLCB1	0.005684	0.043268	-
[4218]	CACNA1S	<---	PRKG2	-0.53538	0.52712	+
[4219]	ATP1A4	<---	PRKG2	-0.22258	0.19984	+
[4220]	MCF2L	<---	RAC3	-0.026438	0.029604	-
[4221]	NCOA1	<---	SMARCD3	-0.065181	0.059892	-
[4222]	ARHGAP33	<---	RHOJ	-0.48747	0.29572	-
[4223]	AKT1	<---	THEM4	-1.3914	1.0709	+
[4224]	PPARA	--->	SCD5	0.024476	-0.19474	+
[4225]	BCAR1	--->	CSF1	0.17733	-0.16412	-
[4226]	FRZB	--->	FZD9	0.73901	-1.3633	+
[4227]	DEPTOR	--->	TSC1	0.36088	-0.40482	-
[4228]	GRB2	--->	KLRC2	0.12689	-0.16241	-
[4229]	APBB1IP	<---	SRC	-0.65966	0.43454	+
[4230]	EZR	<---	ROCK1	0.020811	0.044153	+
[4231]	SOST	--->	WNT8A	0.41775	-0.59125	+
[4232]	FGF5	--->	PDGFRA	0.68139	-0.83032	+
[4233]	CTCF	--->	ZNF805	-0.0066414	-0.044983	+
[4234]	JAM2	<---	PRKCH	-0.25541	0.20772	+
[4235]	FGFR2	--->	PIK3R5	-0.038369	-0.19291	+
[4236]	MYBPC2	<---	MYH6	-0.15073	0.094518	+
[4237]	GNAI1	--->	HRAS	0.76886	-0.56799	+
[4238]	CEBPB	--->	NFKBIA	0.034641	-0.17765	+
[4239]	RXRA	<---	SP1	-0.14326	-0.13142	-
[4240]	LDHA	--->	MYC	0.043379	-0.17554	-
[4241]	CDK5	--->	EGR1	1.0991	-0.67282	-
[4242]	CCND3	<---	CDKN1B	-1.0232	1.4682	-
[4243]	CEBPB	--->	CELF1	0.36909	-0.31439	+
[4244]	FES	<---	PLXNA1	-0.1358	-0.095414	-
[4245]	DKK2	--->	FZD6	0.13996	-0.14782	+
[4246]	MRAS	<---	RASA4	-1.0994	1.1737	+
[4247]	ATF2	<---	SMAD6	-0.080108	0.060865	+
[4248]	MDM2	--->	RANBP2	0.077827	0.039093	-
[4249]	CAV1	<---	TRAF2	-0.3125	0.26384	-
[4250]	CSF1R	<---	PIK3CA	-0.014221	0.044706	-
[4251]	CAMK2B	--->	MAPK3	0.24715	0.164	+
[4252]	GNB2	--->	GRM2	0.15376	-0.10866	-
[4253]	PLEKHG2	<---	RHOBTB1	-0.14219	0.16153	-
[4254]	OSBP	--->	VAPA	0.01581	-0.087437	+

[4255]	PAK3	<---	RAC2	-1.1901	0.96651	+
[4256]	MMP24	--->	POTEKP	0.085904	-0.29263	+
[4257]	FGF12	--->	FLT4	0.071829	-0.16555	+
[4258]	SMAD9	<---	UBE2D3	-0.20131	0.05544	+
[4259]	FOXA2	--->	HNF4A	0.076395	-0.21893	+
[4260]	ORAI2	<---	STIM1	-0.88237	0.15971	+
[4261]	ABR	<---	RHOG	-0.20131	0.10561	-
[4262]	HDAC2	<---	HPN	-0.31114	0.37744	-
[4263]	C3	<--->	C4B	0.26349	0.26349	-
[4264]	CCL4L1	<---	CEBPB	-0.14964	0.055907	+
[4265]	MAPK1	--->	SFN	0.079981	-0.071802	-
[4266]	FGF21	--->	KDR	0.21774	-0.0015767	+
[4267]	FCER1G	--->	PIK3R1	0.028798	-0.076981	+
[4268]	EGFR	--->	UBE2D2	0.20569	-0.1777	-
[4269]	POTEKP	<---	PTK2B	-0.28708	0.061438	+
[4270]	LAMB2	--->	SV2C	0.20521	-0.20511	+
[4271]	MAP3K2	<---	RAP1B	-0.17545	1.1267	+
[4272]	FYN	--->	SLA	0.41943	0.028419	-
[4273]	CASP8	--->	NFKBIA	0.15946	-0.57031	+
[4274]	COL6A6	--->	ITGAV	-0.0067115	-0.019363	+
[4275]	FRZB	--->	WNT3A	1.0317	-1.1063	+
[4276]	EP300	<---	LMCD1	-0.018625	0.020708	-
[4277]	F2RL3	--->	PLG	0.065338	-0.16965	-
[4278]	FCER1A	--->	PPARA	0.032502	-0.15488	-
[4279]	E2F1	<---	GPR132	-0.29226	0.16885	-
[4280]	E2F1	--->	TP53	0.13183	-0.11528	+
[4281]	CREB3	<---	PRKCA	-0.63701	0.72046	+
[4282]	IKBKG	<---	TLR6	-0.50307	0.11178	+
[4283]	IFNGR2	--->	JAK2	1.1771	-1.1167	-
[4284]	GATA3	--->	MEF2A	0.1217	-0.29909	-
[4285]	RBPJ	--->	TWIST1	0.32269	-0.43826	+
[4286]	ADRB3	<---	ADRBK1	-0.21301	0.25327	+
[4287]	RPTOR	<---	RRAGA	-0.69387	1.0493	+
[4288]	ADRBK2	--->	HTR1B	0.25327	-0.21301	+
[4289]	GATA2	--->	RBPJ	0.083738	-0.053804	+
[4290]	FZD6	<---	WNT2B	-0.30406	0.25758	+
[4291]	FOS	--->	STAT3	0.4178	-0.25647	-
[4292]	FOS	--->	KLF1	0.16224	0.14761	+
[4293]	EPHA2	<---	SRC	-0.26855	0.35424	+
[4294]	CD3G	<---	PIK3R2	-0.066584	-0.059014	-
[4295]	ITGAM	<---	PIK3R2	-0.073728	-0.0143	-
[4296]	SFRP4	--->	WNT2B	1.0317	-1.1063	+
[4297]	CCR4	<---	JAK3	-0.12268	-0.039248	-
[4298]	RAP1B	<---	VWF	-0.015029	0.00073144	+
[4299]	GNA15	--->	WNT4	0.42087	-0.47341	-
[4300]	HSP90AB1	<---	SRC	-0.18749	0.29829	+
[4301]	LPAR1	--->	PIK3R1	0.1084	-0.20211	+
[4302]	DAND5	<---	HSPA8	-0.61172	0.6184	-
[4303]	MYO5A	<---	RAB13	-0.046655	0.010173	+
[4304]	EGLN2	<---	PRKCB	-0.91465	0.45016	+
[4305]	ARHGAP44	<---	RHOT2	-0.35613	0.16234	-
[4306]	IRAK3	<---	MYD88	-0.6873	0.026112	-
[4307]	CCL22	<---	CXCR1	-0.35895	0.21658	-
[4308]	RAP1A	<--->	RAP1B	0.1628	0.1628	-
[4309]	PAK1	--->	PDGFB	0.19801	0.051375	-
[4310]	PIK3CB	<---	TXK	-0.06481	0.084876	-
[4311]	IFNLR1	<---	SOCS4	-0.65115	0.3746	+
[4312]	DAND5	--->	IGFBP3	0.036704	-0.072158	+
[4313]	CCL28	<---	CXCR1	-0.35895	0.21658	-
[4314]	ADCY8	<---	EDN1	-0.41432	0.41767	+
[4315]	ACTA1	<---	RHOJ	-0.51168	0.52339	+
[4316]	RHOT2	--->	TIAM1	0.05147	-0.016056	-
[4317]	HDAC2	--->	STAT5A	0.38423	-0.22955	+
[4318]	CREB3L2	--->	MCL1	0.72216	-1.2248	+
[4319]	GSK3B	--->	PSMD14	0.2441	-0.083683	-
[4320]	RAC2	<---	RELA	-0.34579	0.18991	-
[4321]	FZD1	<---	SOST	-0.2929	0.24246	+
[4322]	HOXB9	--->	JUN	0.038793	-0.098653	-
[4323]	CTNNB1	<---	PCDHGB7	-0.19204	0.11503	-
[4324]	TBC1D4	<---	YWHAQ	0.06516	0.21759	-
[4325]	POFUT2	--->	SPON1	-0.004569	-0.07359	+
[4326]	PRKCE	--->	RAF1	0.18571	-0.23412	+
[4327]	L1CAM	<---	RPS6KA1	-0.12605	0.076189	+
[4328]	FGG	<---	PTPN1	-1.1961	0.99587	+
[4329]	PRKACA	<--->	STRADB	0.31014	0.31014	-
[4330]	LRP1B	<---	NCSTN	-0.19529	0.24035	+

[4331]	FGF16	--->	PDGFRB	0.48772	-0.57252	+
[4332]	GNB1	--->	WNT9B	0.19155	-0.26814	-
[4333]	CTCF	<---	NFKB2	-0.10299	0.13019	-
[4334]	RHOF	--->	VAV3	0.058196	0.018587	-
[4335]	CDC42	<---	GNAI1	-0.93465	1.2694	+
[4336]	CTCF	--->	ZSCAN31	-0.0066414	-0.044983	+
[4337]	CD40LG	--->	MAPK8	0.10494	0.048942	+
[4338]	ADCY5	<---	PRKACB	-0.63235	0.8673	-
[4339]	EP300	--->	ZSCAN10	0.01223	-0.0076085	+
[4340]	ADRBK1	--->	MTNR1A	0.25327	-0.21301	+
[4341]	CACNA1C	--->	GRIN1	0.15899	-0.088421	-
[4342]	BRAF	<---	RAP2C	-0.86318	0.78219	+
[4343]	AXIN1	--->	FZD5	0.043749	-0.083935	-
[4344]	SMO	<---	STK36	-0.28413	0.34258	-
[4345]	KLF1	--->	MYC	0.033171	-0.16882	-
[4346]	GHR	<---	IRS1	0.018455	0.089853	-
[4347]	ACTB	<---	MMP2	-0.29263	0.085904	+
[4348]	MAPK12	<--->	PRKACG	0.31014	0.31014	-
[4349]	FGF8	<---	MAPK3	-0.47959	0.71493	+
[4350]	COL9A3	--->	MMP13	-0.0056809	-0.18543	-
[4351]	MYH2	<---	RAC1	-0.01596	0.026206	+
[4352]	ADCY1	<---	CAMK2A	-0.44346	0.58749	+
[4353]	PIAS3	--->	SMAD3	0.0061839	-0.83897	+
[4354]	CER1	--->	WNT6	0.60582	-0.57968	+
[4355]	RANBP2	--->	SRSF2	0.34767	-0.31854	+
[4356]	ATP1A1	<---	NR3C2	-0.056504	-0.0074276	+
[4357]	IFNB1	<---	STAT1	-0.51073	0.62285	-
[4358]	GNAI3	<---	RGS2	-1.2531	1.2063	+
[4359]	FGFR2	<---	PRKCI	-0.25567	0.25085	-
[4360]	COL3A1	--->	ITGA7	0.0052169	-0.090529	+
[4361]	HCK	--->	PTK2	0.34449	-0.5161	+
[4362]	RAC1	<---	SHB	-0.072693	0.30072	+
[4363]	CTCF	<---	LMO1	-0.15173	0.27332	-
[4364]	NFATC1	--->	RNF128	0.11084	-0.40394	+
[4365]	NFKB2	--->	TNFSF13B	0.36806	-0.55401	+
[4366]	NGF	<---	PSEN2	-0.45705	0.38692	+
[4367]	MAPK12	<---	RAPGEF4	-0.093777	0.86463	+
[4368]	ATF6B	<---	PRKACB	-0.57905	1.0858	+
[4369]	GRK4	--->	GRM5	0.80802	-0.62351	+
[4370]	MOB1B	<---	WWTR1	-0.77343	0.57923	-
[4371]	FGF17	--->	FGFR1	1.1329	-1.3925	+
[4372]	CCR3	--->	CX3CL1	0.21658	-0.35895	-
[4373]	MAPK1	--->	SMARCA4	0.015081	-0.046268	-
[4374]	ACVR1	--->	MAPK13	0.36409	0.12889	+
[4375]	ANGPT2	--->	PIK3R1	0.02813	-0.11732	+
[4376]	MAP2K4	<--->	MAPK6	0.31014	0.31014	-
[4377]	SKIL	--->	SMAD3	-0.031512	-0.063591	-
[4378]	DRD4	--->	GNAO1	1.1631	-1.1303	+
[4379]	BRAF	<---	PRKCG	-0.23412	0.18571	+
[4380]	GNAI2	<--->	GNAL	0.064764	0.064764	-
[4381]	NOS2	<---	RELA	-0.10586	0.039245	+
[4382]	FGF10	--->	KIT	0.015796	-0.016964	+
[4383]	RASGRF2	--->	RRAS2	0.44606	-0.52428	+
[4384]	ITGA9	<---	LAMA5	-0.041507	-0.0065623	+
[4385]	CSNK2A1	--->	NFIC	0.6709	-0.55941	-
[4386]	CD79A	<---	PTPRC	-0.16486	0.1118	+
[4387]	NCOA1	--->	TGFB1	-0.010501	-0.013468	-
[4388]	PRMT1	--->	STAT3	0.54982	-0.4266	-
[4389]	KRAS	--->	SPTB	0.04502	-0.024501	-
[4390]	KCNMB1	<---	PRKG1	-1.2332	0.72885	+
[4391]	CCR8	--->	GNAI2	1.1631	-1.1303	+
[4392]	BTK	--->	NRAS	0.25652	0.067809	+
[4393]	EIF2AK1	<---	PPP1CB	-0.12622	0.14153	+
[4394]	CCNB1	<---	SEC13	-0.33759	0.44625	-
[4395]	NFKBIA	<---	NR3C1	-0.23053	0.03163	+
[4396]	CRK	<---	RET	-0.11251	-0.071904	+
[4397]	COL2A1	<---	LEPRE1	-0.073376	-0.02648	+
[4398]	PKN3	--->	RELA	0.32847	-0.50044	+
[4399]	NFKB1	--->	TNIP1	0.18625	-0.19285	+
[4400]	SMAD2	<---	UBE2D1	-0.20131	0.05544	+
[4401]	PIK3R2	<---	TLR3	-0.12913	0.0075307	+
[4402]	FGD1	<---	RHOU	-0.14219	0.16153	-
[4403]	COL11A1	--->	PPIB	-0.024575	-0.068802	-
[4404]	ARHGAP44	<---	RHOV	-0.92575	0.61511	-
[4405]	ERBB3	<---	PRKCG	-0.085576	0.11629	-
[4406]	PPP1CC	--->	TRPM8	0.47173	-0.65712	+

[4407]	C1QA	--->	IGKV4-1	-0.021439	-0.042516	+
[4408]	CD3D	<---	JAK2	-0.22013	0.087709	+
[4409]	CACNA1F	<---	GNAI3	-0.17558	0.23994	+
[4410]	PTGS1	--->	TAL1	0.049189	-0.099502	-
[4411]	MUC1	--->	STAT5B	0.44408	-0.32334	-
[4412]	NCOA3	<---	RARB	-0.054726	0.035483	+
[4413]	NLRC5	<---	STAT1	-0.19409	0.12263	+
[4414]	DKK2	--->	WNT16	0.54164	-0.69755	+
[4415]	LAT	--->	MEF2A	0.14209	-0.33316	-
[4416]	MTOR	<---	NFKB1	-0.07119	0.094521	-
[4417]	CHRM4	--->	PRKCI	-0.029433	-0.05892	+
[4418]	MAPK9	<---	TGFB3	-0.0067309	0.13651	+
[4419]	ACADL	--->	RXRG	-0.091263	-0.26204	-
[4420]	CD8A	<---	CSK	-0.47931	0.63774	-
[4421]	FGF6	<---	PLCG1	-0.032479	0.060664	-
[4422]	ERBB2	--->	SHC3	1.2029	-1.0554	+
[4423]	CHAD	--->	ITGA9	0.0373	-0.11699	+
[4424]	MYL2	<---	RXRA	-0.3009	-0.055578	+
[4425]	ELMO1	--->	GNAI2	0.05262	-0.011498	-
[4426]	CDC42	<--->	RAC1	0.1628	0.1628	-
[4427]	MED26	--->	TGFB1	0.0080535	-0.22171	+
[4428]	LPAR3	--->	PIK3CG	0.029282	-0.062766	+
[4429]	ACTG1	<---	MMP1	-0.29263	0.085904	+
[4430]	MYL10	--->	ROCK1	0.31451	-0.08875	-
[4431]	GH1	--->	OSMR	0.073187	-0.17721	+
[4432]	CSNK2A2	--->	NFIC	0.6709	-0.55941	-
[4433]	CCNE2	<---	PKMYT1	-0.55661	0.75243	+
[4434]	NTRK1	--->	STAT3	0.11815	-0.23718	+
[4435]	CTNNB1	<---	TAF1	-0.44924	0.34206	+
[4436]	AKT1	--->	RALGDS	0.16506	-0.19034	+
[4437]	MAX	<---	NRBF2	-0.069589	0.10065	-
[4438]	GNAT1	--->	GNB5	0.13157	-0.0025637	-
[4439]	RHOA	<---	SHB	-0.072693	0.30072	-
[4440]	IL5RA	<---	TYK2	-0.11806	0.19541	-
[4441]	CDK4	--->	CDKN1A	0.71039	-0.49354	-
[4442]	BCAR1	--->	ITGB1	0.069768	-0.069539	-
[4443]	SOS1	<---	ZFYVE28	0.043876	0.096929	+
[4444]	NGFR	--->	PIK3CG	0.013159	-0.080249	+
[4445]	KLRK1	--->	VAV3	0.06651	-0.018805	+
[4446]	EGR3	<---	NFATC2	-0.57388	0.50851	-
[4447]	FCGR2A	<--->	FCGR3A	0.34717	0.34717	-
[4448]	AKT1	--->	ITGA2B	0.060159	-0.13176	+
[4449]	DNM2	<---	F2R	-0.088002	0.082554	-
[4450]	PRKCB	--->	SHC3	0.468	-0.43947	-
[4451]	CRKL	--->	ITGA9	0.032574	-0.078547	-
[4452]	COL4A3	--->	ITGA6	-0.013036	-0.10803	+
[4453]	CTBP2	--->	PAX5	0.014037	-0.063302	+
[4454]	ARL4A	<---	CEBPB	-0.13279	-0.12595	+
[4455]	JUP	<---	SRC	-0.4317	0.52076	+
[4456]	ARF6	<---	ITGB3	-0.050618	-0.048099	+
[4457]	CXCR6	--->	PPBP	0.21658	-0.35895	-
[4458]	ITGA1	<---	PRKAR2A	-0.15111	0.038483	+
[4459]	CCNE2	<---	SKP1	-0.25849	0.28986	+
[4460]	DLG4	<---	ERBB4	-0.077078	0.031401	-
[4461]	MPL	<---	TYK2	-0.23207	0.14292	-
[4462]	MET	--->	PIK3CD	0.021126	-0.034545	+
[4463]	EIF2AK1	<---	PPP1CA	-0.12622	0.14153	+
[4464]	NFIC	--->	POU1F1	0.34916	-0.46338	+
[4465]	IFNG	--->	PIK3R1	0.074256	-0.19296	+
[4466]	CCR1	--->	PPBP	0.21658	-0.35895	-
[4467]	GNA15	<---	OXTR	-1.1303	1.1631	+
[4468]	FGF20	--->	IGF1R	0.25346	-0.0044024	+
[4469]	NFKB1	<---	PPP2CA	-0.04343	0.077509	-
[4470]	PDPK1	--->	RPS6KA3	1.6772	-1.9829	-
[4471]	CTNNA2	--->	MMP9	0.38719	-0.55042	-
[4472]	PDGFB	<---	PTPN1	-0.67709	0.5754	+
[4473]	DLC1	<---	RHOBTB1	-0.30675	0.061607	-
[4474]	AKT2	--->	RPS6KB1	0.67325	-0.79394	+
[4475]	HGF	--->	PDGFRB	0.043622	-0.0013731	+
[4476]	GATA2	<---	PECAM1	-0.085373	0.037772	-
[4477]	PCK2	<---	PRKAG3	-0.22182	0.067346	+
[4478]	EXOC8	<---	RALA	-0.50681	0.59188	+
[4479]	GNAI2	<---	XCR1	-1.1303	1.1631	+
[4480]	HIF1A	<---	HKDC1	0.061721	0.06932	-
[4481]	CDH1	<---	IQGAP1	-0.098608	0.025681	+
[4482]	PDX1	<---	PRKX	-0.46198	0.60871	+

[4483]	MAPK8	<---	TRAF3	-0.47605	1.1617	+
[4484]	ITGA1	<---	PRKAR1A	-0.15111	0.038483	+
[4485]	AKT2	--->	GRB14	0.087326	0.081418	-
[4486]	PPM1A	--->	SMAD3	0.32113	-0.52898	+
[4487]	ARRB1	--->	FZD7	0.13556	-0.17053	-
[4488]	ROCK2	<---	SLC9A1	-0.10584	0.12042	-
[4489]	SMARCE1	--->	TCF12	0.53879	-0.52411	-
[4490]	POU2F1	<---	SPI1	-0.24396	0.15908	-
[4491]	LAMB4	--->	SV2A	0.27773	-0.3202	+
[4492]	KLRD1	<---	PLCG1	-0.021591	0.04532	-
[4493]	COL2A1	<---	PCOLCE	-0.15873	0.062969	+
[4494]	ACTG2	<---	MMP9	-0.29263	0.085904	+
[4495]	IKBKG	<---	TRAF5	-0.63055	0.80437	+
[4496]	GNAI3	<---	LYN	-0.44236	0.67161	-
[4497]	MAPK3	--->	YWHAE	0.079981	-0.071802	-
[4498]	INSR	<---	PTPRF	-0.076966	-0.041863	+
[4499]	MAPK8	<---	TAB3	-0.13728	0.23085	+
[4500]	JAK3	<---	SOCS5	-0.73315	0.53539	+
[4501]	NCOA1	<---	SMAD3	-0.041375	0.023622	-
[4502]	CD4	<---	GNAI3	-0.052939	0.061543	+
[4503]	ATF6	<---	MAPK14	-0.57905	1.0858	+
[4504]	IKBKG	<---	IRS4	-0.25857	0.34441	-
[4505]	ACVR1C	--->	NLK	0.17987	-0.35209	+
[4506]	RAF1	--->	RELA	-0.10548	-0.15822	+
[4507]	JAK2	--->	MAPK1	0.43202	-1.1344	+
[4508]	CCL2	<---	CCR9	-0.35895	0.21658	-
[4509]	CCR1	--->	XCL1	0.21658	-0.35895	-
[4510]	ITGA4	--->	THBS1	0.16838	-0.067658	-
[4511]	ITGA2B	<---	PXN	-0.084695	0.026004	-
[4512]	CSNK1A1	--->	WNT2B	0.83912	-0.78694	-
[4513]	CRKL	--->	IL2RG	0.088471	-0.078989	-
[4514]	AKAP13	<---	RHOA	-0.22586	0.072537	-
[4515]	FGF23	--->	FGFR2	0.40218	-0.67149	-
[4516]	SREBF2	<---	TGFB1	-0.11402	-0.084233	+
[4517]	MAPK12	--->	TP63	0.096521	-0.076704	+
[4518]	ADRB3	<---	GRK6	-0.28786	0.33635	+
[4519]	MAPK9	<---	RAPGEF3	-0.093777	0.86463	+
[4520]	GRB2	<---	IGF1R	0.015827	0.095474	+
[4521]	ARHGAP24	<---	RAC2	-0.49576	0.47865	-
[4522]	CCL16	<---	CXCR1	-0.35895	0.21658	-
[4523]	ATF2	--->	BDNF	0.61729	-0.43822	+
[4524]	DUSP10	--->	MAPK9	1.353	-1.036	+
[4525]	CTNNA1	--->	MMP20	0.38719	-0.55042	-
[4526]	CCNB3	<---	GADD45A	-0.67465	0.65424	+
[4527]	CTNNB1	--->	NOTCH1	0.26549	-0.18577	-
[4528]	AKT3	--->	BCL2L1	0.30677	-0.44298	+
[4529]	ARHGAP10	<---	RND2	-0.65972	0.48549	-
[4530]	CBL	--->	RAP1A	0.27831	-0.12475	+
[4531]	CCL25	<---	CCR4	-0.35895	0.21658	-
[4532]	LNX1	--->	NUMB	0.055842	-0.037664	+
[4533]	IQGAP2	--->	RAC3	0.12856	-0.076889	-
[4534]	COL5A2	--->	PPIB	0.022658	-0.10782	-
[4535]	HNF4G	--->	STAT5B	0.27087	-0.22437	+
[4536]	FGFR3	--->	SPRY4	0.16123	-0.11915	+
[4537]	ETS1	<---	VEGFC	-0.0418	-0.012527	-
[4538]	BATF	--->	KLF6	0.16224	0.14761	+
[4539]	BIRC2	--->	CTCF	0.043002	-0.029409	-
[4540]	OTX2	--->	TCF12	0.23888	-0.32236	-
[4541]	DAG1	<---	LAMB2	-0.027338	0.020315	+
[4542]	CBL	--->	EPO	0.28043	-0.1482	-
[4543]	PSMD10	<---	SPOP	-0.18082	0.18558	-
[4544]	CXCR1	--->	XCL1	0.21658	-0.35895	-
[4545]	SOX17	--->	TCF7	1.4646	-1.3296	+
[4546]	GRIN1	<---	RAC1	-0.38938	0.54247	-
[4547]	ESR1	--->	STAT3	0.27087	-0.22437	-
[4548]	PPP1CA	--->	ZFYVE9	0.10658	-0.18674	-
[4549]	GRK1	--->	SSTR4	0.33635	-0.28786	+
[4550]	IL6ST	<---	PIAS1	-0.087342	0.064164	-
[4551]	JUN	<---	SFTPA1	-0.13523	0.096401	-
[4552]	ITGA6	--->	TNXB	-0.025002	-0.077063	-
[4553]	CDK2	<--->	MAPK3	0.31014	0.31014	-
[4554]	JAK3	--->	PIK3CA	0.011356	-0.033797	+
[4555]	ITGB3	<---	KRAS	-0.01596	0.026206	-
[4556]	MYO9B	<---	RHOQ	-0.54674	0.38058	-
[4557]	PFKFB1	--->	PRKAG1	-0.012105	-0.096604	-
[4558]	IL12RB1	<---	IL20	-0.29036	0.12248	+

[4559]	ERBB3	--->	HIF1A	0.060245	-0.024893	+
[4560]	KRAS	<---	PTPRA	-1.0196	0.97901	+
[4561]	GNAI3	<---	NOS3	-0.0047367	0.00957	-
[4562]	CTCFL	<---	PHF19	-0.12719	0.25163	-
[4563]	COL11A1	<---	PCOLCE	-0.32363	0.21278	+
[4564]	ADCY1	<---	PRKACG	-0.63235	0.8673	-
[4565]	EP300	--->	NACC1	0.00067605	-0.0050208	+
[4566]	RAPGEF4	--->	RYR2	0.019562	-0.014285	+
[4567]	EP300	--->	ETS1	-0.00068954	-0.0041449	+
[4568]	NCF2	<---	RHOA	0.088846	0.11735	-
[4569]	MSC	<---	NFKB1	-0.29534	0.47748	+
[4570]	CXCR3	--->	XCL2	0.21658	-0.35895	-
[4571]	NGFR	--->	PGF	0.039957	-0.32359	-
[4572]	ACTB	<---	PXN	-0.36318	0.3394	+
[4573]	CTNNB1	<---	TCF3	-0.15362	0.05043	-
[4574]	PLD1	<---	SPHK1	-0.057765	0.061811	-
[4575]	CREB3L3	<--->	FOSB	0.36709	0.36709	-
[4576]	GNAI3	--->	RAC1	1.2694	-0.93465	+
[4577]	CSF1R	<---	FIGF	-0.30912	0.56567	+
[4578]	RHOJ	<---	VAV1	0.1025	0.10902	+
[4579]	ADORA2B	<---	ADRBK2	-0.21301	0.25327	+
[4580]	ACP5	--->	SPI1	0.29452	-0.5028	-
[4581]	FGF13	--->	FLT1	0.13624	-0.0034489	+
[4582]	EGLN1	<---	FOXA2	-0.27028	0.18836	+
[4583]	AR	<---	HRAS	-0.10317	0.077862	-
[4584]	GABBR2	<---	GNB4	-0.067008	0.092163	-
[4585]	ESR1	--->	POU4F1	0.10711	-0.27075	+
[4586]	CREBBP	--->	HK2	-0.00068954	-0.0041449	+
[4587]	ARRB1	--->	WNT6	0.23514	-0.34616	+
[4588]	MYD88	--->	RIPK2	0.64348	-0.71107	+
[4589]	MAPK12	<---	PAK2	-1.2674	1.5523	+
[4590]	POU2F1	<---	REL	-0.66578	0.38757	-
[4591]	GRB2	--->	KIT	-0.26231	-0.65509	-
[4592]	CRK	--->	ITGA3	0.055272	-0.09118	-
[4593]	OPHN1	<---	RHOH	-0.9609	0.7795	-
[4594]	IKBKG	<---	PDPK1	-0.28258	0.71031	+
[4595]	NRG2	<---	SRC	-0.22084	0.22446	+
[4596]	ARHGAP25	<---	RAC3	-1.2097	0.92798	-
[4597]	PEBP1	<---	PRKD1	-0.34948	0.35832	+
[4598]	PTPRC	--->	TCRA	0.14933	-0.21762	+
[4599]	FOXA1	--->	ZNF131	0.27644	-0.18319	+
[4600]	KIR2DL4	<---	PTPN11	-0.54038	0.44825	-
[4601]	EPS15	<---	HGS	-0.036026	-0.002804	-
[4602]	ATP1B4	--->	PIK3R5	0.39995	-0.56102	-
[4603]	CACNA1S	<---	PPP2CB	-0.27679	0.37996	+
[4604]	JUP	<---	MYC	-0.1109	-0.039597	-
[4605]	EP300	--->	THBS1	0.021825	-0.011642	+
[4606]	C2CD5	--->	MYH2	0.041632	-0.10957	+
[4607]	GRIA1	<---	PPP1CC	-0.67094	0.67498	+
[4608]	NR2C2	--->	SPI1	-0.015388	-0.14879	+
[4609]	CDC20	<---	PSMD14	-0.26071	0.27742	+
[4610]	JUP	<---	PAK3	-0.70785	0.7131	+
[4611]	HIF1A	<---	PFKFB3	0.061721	0.06932	-
[4612]	ARVCF	--->	BCL3	0.17614	-0.078689	-
[4613]	GP1BA	--->	SERPIND1	0.39784	-0.66389	-
[4614]	NTN1	<---	TRIO	-0.00039921	0.0067787	-
[4615]	JUNB	<---	PAX5	-0.038059	0.012505	+
[4616]	ITGA1	<---	PTK2	-0.052104	-0.015744	-
[4617]	DAND5	--->	PDHA1	0.28061	-0.33364	+
[4618]	E2F6	--->	ZNF75A	0.23995	-0.10098	+
[4619]	FOXO3	--->	GADD45A	0.36388	-0.62421	+
[4620]	BCAR1	<---	FGR	0.023313	1.0891	+
[4621]	ARHGEF11	--->	RHOT1	0.16719	-0.11927	+
[4622]	DEPDC7	<---	RAC3	-0.058594	0.11751	-
[4623]	CCND1	<---	TCF7L1	-0.21775	0.37715	+
[4624]	EPHB4	<---	LIMK1	-0.01801	0.025848	-
[4625]	ARR3	--->	FZD10	0.13556	-0.17053	+
[4626]	CHD9	--->	NR1D1	0.014854	-0.17168	-
[4627]	ARHGAP1	<---	RHOT1	-0.35613	0.16234	-
[4628]	CHRM1	<---	PKN2	-0.1993	0.11589	-
[4629]	JAK2	--->	PTK2	-0.060415	-0.38746	+
[4630]	FCER1G	--->	PLD2	0.036391	-0.036136	+
[4631]	GNB5	<---	HTR1F	-0.92616	1.1575	+
[4632]	RPS12	--->	TBP	0.42294	-0.54272	-
[4633]	LEP	<---	PTPN1	-1.1386	0.97471	+
[4634]	PPP1R13L	--->	SPI1	0.015015	-0.10582	-

[4635]	FGFR1	<---	PGF	-0.61313	0.56088	+
[4636]	ARHGAP29	<---	RHOQ	-1.011	0.68428	-
[4637]	GNA15	<---	RGS4	-1.2531	1.2063	+
[4638]	FGFR1	<---	SHC4	-0.10391	-0.029269	-
[4639]	DEF6	<---	RAC1	-0.65413	0.6983	-
[4640]	BRCA1	--->	GABPA	0.18634	-0.1985	-
[4641]	ABCB4	--->	JUN	-0.00086664	-0.064647	-
[4642]	AKT1	--->	YWHAB	0.043615	-0.1933	+
[4643]	RAP1GAP	<---	YWHAZ	-0.19238	-0.17593	+
[4644]	MYL12B	--->	MYLK	0.33394	-0.31058	-
[4645]	ADCY1	<---	EDN1	-0.41432	0.41767	+
[4646]	FGF16	--->	PDGFRA	0.68139	-0.83032	+
[4647]	ACTG1	<---	HTT	-0.72444	0.64593	+
[4648]	HIF1A	<---	THRB	0.034569	0.071935	+
[4649]	CNOT6L	--->	EIF4E	0.36177	-0.49521	+
[4650]	SMPD3	<---	TNFRSF1A	-0.33817	0.05465	+
[4651]	DUSP7	--->	MAPK13	1.353	-1.036	+
[4652]	ANGPT4	<---	FOXO1	-0.43599	0.34955	+
[4653]	DLC1	--->	PLCB1	0.036451	-0.097767	+
[4654]	CEACAM5	<---	NFKB1	-0.065503	0.053968	+
[4655]	LYN	--->	STAT3	0.43807	-0.74539	+
[4656]	EP300	<---	IRF2BP2	-0.013392	0.0125	-
[4657]	FGFR1	--->	PLCG1	0.0087648	-0.025406	+
[4658]	EGF	<---	SPRY3	-0.13445	0.1443	+
[4659]	CREBBP	--->	SKP2	-0.0067507	-0.0071006	+
[4660]	PGF	--->	PIK3R1	0.058137	-0.13402	+
[4661]	GAB2	<---	PRKCI	-0.15343	0.32961	-
[4662]	CD40LG	<---	TDP2	-0.069238	-0.056467	-
[4663]	JAK1	<---	PDGFRB	-0.71241	0.60182	+
[4664]	EP300	--->	TIMP1	0.00067605	-0.0050208	+
[4665]	CRLF2	<---	IL3	-0.07563	0.0029493	+
[4666]	FOXO1	<---	SGK3	-0.24953	0.090624	+
[4667]	ARAP1	<---	RHOV	-0.48615	0.36733	-
[4668]	COL4A2	--->	ITGA7	-0.013036	-0.10803	+
[4669]	CTCF	<---	PPM1F	-0.19997	0.36199	-
[4670]	LCP2	--->	TRBV12-3	-0.016269	-0.16182	-
[4671]	IRS4	<---	PTPN1	-0.9667	0.95122	+
[4672]	PRKCD	--->	SPHK1	0.25182	-0.33821	+
[4673]	CREB5	<---	EP300	-0.010885	0.015595	-
[4674]	FLI1	--->	PDGFRB	0.036699	-0.01322	+
[4675]	CTCF	--->	ZNF823	-0.0066414	-0.044983	+
[4676]	CCR3	--->	CXCL10	0.21658	-0.35895	-
[4677]	RAC3	--->	SRGAP3	0.39011	-0.42732	-
[4678]	ITGA6	--->	THBS1	0.16838	-0.067658	-
[4679]	FOS	--->	ILK	0.0515	-0.0016212	-
[4680]	HRAS	--->	ITGA6	0.017528	-0.065662	-
[4681]	CEBPB	--->	HIST2H3A	0.21329	-0.32305	-
[4682]	CDH1	<---	PLG	-0.25141	0.044891	+
[4683]	CASP7	<---	STAT1	-0.25299	0.15858	+
[4684]	NOTCH2	<---	PCSK6	-0.048737	0.058804	+
[4685]	HRAS	--->	RPS6KA1	0.39663	-0.23906	-
[4686]	CCL19	<---	CCR9	-0.35895	0.21658	-
[4687]	LEPR	<---	SOCS4	-0.1723	0.11167	+
[4688]	DAND5	--->	PDGFB	0.0064702	-0.030285	+
[4689]	DAND5	--->	IGF1R	0.28396	-0.028881	+
[4690]	MYBPC3	<---	MYL4	-0.12286	0.036048	+
[4691]	F2RL3	--->	GNA12	1.1631	-1.1303	+
[4692]	PDGFRA	<---	PIK3R5	-0.17898	0.14037	-
[4693]	CTSG	--->	SERPINB9	0.33364	-0.53505	-
[4694]	CSNK1A1L	--->	FZD7	0.5614	-0.51612	-
[4695]	NOS3	<---	PRKACA	-0.060388	0.088587	+
[4696]	PRKCZ	<---	RAP1B	-1.529	1.3756	-
[4697]	MAP3K7	--->	MAPK8	1.2193	-1.3738	+
[4698]	IL13RA1	<---	JAK3	-0.23741	0.19744	-
[4699]	ANAPC2	<---	PSMD14	-0.10006	0.018807	+
[4700]	FZD2	<---	SFRP5	-1.3633	0.73901	+
[4701]	HDAC3	--->	MEF2D	-0.041005	-0.044156	+
[4702]	GRIN2B	<---	GRM1	-0.36981	-0.05834	+
[4703]	PLK1	<---	PSMD7	-0.15735	0.388	+
[4704]	NOTCH1	<---	PCSK5	-0.12075	0.16382	+
[4705]	BMPR2	<---	MAPK13	0.14267	0.21493	-
[4706]	PRKCD	--->	PTK2B	0.041146	-0.044924	+
[4707]	FGF16	--->	FLT1	0.21774	-0.0015767	+
[4708]	TGFB1	<---	ZFYVE9	-0.14049	0.063711	+
[4709]	CYSLTR2	--->	GNA14	1.1631	-1.1303	+
[4710]	PRKCG	--->	RXRA	0.18741	-0.28891	+

[4711]	KCNQ3	--->	TRPV4	-0.088937	-0.17028	+
[4712]	APC2	<---	CSNK2A2	-0.10343	0.11983	+
[4713]	IL5RA	<---	PDPK1	-0.16176	0.43594	-
[4714]	PRKCE	--->	PTPN11	0.55493	-0.48581	-
[4715]	FABP4	--->	RXRG	-0.091263	-0.26204	-
[4716]	APBB1IP	--->	FN1	0.096402	-0.13048	-
[4717]	CHD2	--->	GFI1	0.051816	-0.055891	+
[4718]	BUB1B	--->	PSME4	0.57437	-0.5963	-
[4719]	GRB2	--->	S1PR1	-6.3665e-005	-0.064961	-
[4720]	KPNA7	<---	NUP43	-0.19114	0.12101	+
[4721]	RELA	--->	VCAM1	0.11826	-0.18235	+
[4722]	PLA2G4F	<---	RASGRP2	-0.071292	0.0030946	+
[4723]	GRIA4	<---	GSK3B	-1.006	1.5014	+
[4724]	ACTG1	--->	STK3	0.89796	-0.77607	+
[4725]	COL2A1	--->	ITGA8	0.0052169	-0.090529	+
[4726]	FGF8	--->	PDGFRB	0.48772	-0.57252	+
[4727]	EZR	<---	FAS	-0.15488	0.0289	-
[4728]	ARHGAP30	<---	RHOBTB1	-0.92575	0.61511	-
[4729]	CBLC	--->	IL23R	0.29477	-0.12853	+
[4730]	MAPK3	--->	RPS6KA2	0.9519	-1.129	+
[4731]	GNAI3	--->	RHOA	1.2694	-0.93465	+
[4732]	JAK1	<---	SRC	-1.3861	0.84298	+
[4733]	ITGA4	<---	RASSF5	-0.13486	0.053468	+
[4734]	HIST1H3A	<---	NFKB1	-0.29753	0.29301	-
[4735]	GNA11	<---	PLCB4	-0.023623	0.0010472	-
[4736]	CREB3L1	<---	PRKX	-0.57905	1.0858	+
[4737]	MYLK	<---	PAK6	-0.52045	0.62184	+
[4738]	GRB2	--->	NTF3	0.40823	-0.4321	-
[4739]	CCND1	<---	SMARCC2	-0.33683	0.38035	+
[4740]	H2AFX	--->	TBP	0.078479	-0.13706	-
[4741]	CASP8	--->	CD247	0.10277	-0.36413	-
[4742]	BCAR1	--->	PDGFB	0.11356	-0.10958	-
[4743]	CREB1	--->	ILK	0.030137	-0.0020829	-
[4744]	FGF6	--->	JUP	0.20943	-0.26232	-
[4745]	BMP2	<---	TAB1	-0.69176	0.63763	-
[4746]	CCND1	<---	MAPK12	-0.55661	0.75243	+
[4747]	NCOR2	--->	NOTCH2	0.17972	-0.14396	-
[4748]	GNAI2	<---	PRKACA	-0.22953	0.50225	+
[4749]	FOXO4	<---	STK4	0.28083	0.33755	+
[4750]	CSNK1A1	--->	TP53	0.31197	-0.21427	+
[4751]	JUNB	<---	MAPK13	-0.47491	0.66358	+
[4752]	ABL1	--->	EPHA3	0.054361	-0.15878	-
[4753]	CCR1	--->	CXCL9	0.21658	-0.35895	-
[4754]	GNAT2	<---	OPRM1	-1.1303	1.1631	-
[4755]	RHOC	--->	SEMA4D	0.22192	-0.16793	-
[4756]	ERBB3	--->	JAK2	1.1628	-0.035242	+
[4757]	CTCF	<---	SPDEF	-0.0066284	0.049367	-
[4758]	DNMT1	--->	HIST1H3A	0.24113	-0.34738	+
[4759]	CCR2	<---	GRK7	-0.28786	0.33635	+
[4760]	RASA3	--->	RRAS2	1.1737	-1.0994	+
[4761]	IL6ST	<---	PIAS3	-0.087342	0.064164	-
[4762]	CRTC2	--->	SFN	-0.0021827	-0.10486	-
[4763]	EGFR	--->	IPCEF1	0.48107	-0.44255	+
[4764]	PRKACG	--->	RYR2	0.06942	-0.04418	+
[4765]	EP300	--->	PCK2	0.011869	-0.015161	+
[4766]	MAP2K2	--->	YWHAQ	0.079981	-0.071802	-
[4767]	RHOF	--->	TAGAP	1.016	-1.5245	-
[4768]	PIK3R2	<---	RRAS	-0.33729	0.22452	+
[4769]	RXRB	<---	SORBS1	-0.17063	0.045962	-
[4770]	CTBP1	--->	HDAC1	0.1754	-0.20802	-
[4771]	TICAM1	--->	UBE2D2	0.38496	-0.36312	-
[4772]	MAP3K14	<---	TNF	0.048942	0.10494	+
[4773]	EGFR	--->	SYNJ1	0.24184	-0.21654	-
[4774]	CCL24	<---	CXCR5	-0.35895	0.21658	-
[4775]	EIF3B	<---	HNF4G	-0.31347	0.23448	+
[4776]	BLK	--->	PTK2	0.34449	-0.5161	+
[4777]	ITGA2	<---	PIK3CD	-0.024164	-0.004208	-
[4778]	FGFR4	<---	PRKCI	-0.25567	0.25085	-
[4779]	PRKD3	--->	STAT5A	0.84558	-0.79252	-
[4780]	MOB1B	<---	YAP1	-0.77343	0.57923	-
[4781]	FADD	--->	TNFRSF10D	0.2446	-0.54302	-
[4782]	GNB2	--->	WNT3A	0.3343	-0.42584	-
[4783]	GNAO1	<---	RGS17	-1.2531	1.2063	+
[4784]	PIK3R2	<---	RAC1	-0.33729	0.22452	-
[4785]	CRKL	--->	EPO	0.096203	-0.13824	-
[4786]	COL18A1	--->	PRSS2	-0.014148	-0.058109	+

[4787] SPTBN5 <--- SRC -0.43809 0.30577 +
[4788] FBXO45 <--- TP73 -0.062935 0.018792 -
[4789] LIFR <--- SOCS2 -0.06215 -0.055475 +
[4790] CNGB3 ---> PDE6C 0.36568 -0.36893 -
[4791] ADCY1 <--- PRKCD -0.47485 0.42125 +
[4792] FGF4 <--- POU5F1 -0.0596 0.064226 +
[4793] AURKA ---> PSMD14 0.2441 -0.083683 -
[4794] DUSP5 ---> MAPK13 1.353 -1.036 +
[4795] PRKCE ---> TBXA2R 0.27696 -0.37622 +
[4796] ADCY7 <--- CAMK2B -0.44346 0.58749 +
[4797] CTCFL <--- HNF4G -0.13354 0.14696 +
[4798] H3F3A <--- SUV39H1 -0.18758 0.15209 +
[4799] HES4 <--- MAX -0.36106 -0.15175 +
[4800] CBL ---> VEGFA 0.1612 -0.080451 -
[4801] CTCF <--- EDN1 -0.0091154 0.083205 -
[4802] BHLHE40 <--- NR1D1 -0.42437 0.26687 -
[4803] MYH11 <--- ROCK1 0.024697 0.042724 +
[4804] TIRAP <--- TRAF6 -0.21928 0.42938 -
[4805] EGFR ---> SLC9A1 0.24184 -0.21654 +
[4806] BCAR1 ---> ITGAM 0.041847 -0.077667 -
[4807] PLK1 <--- RAB8A -0.42856 1.1964 -
[4808] IGF1 <---> INS 0.10744 0.10744 -
[4809] NCSTN ---> ZNF274 0.28002 -0.15205 +
[4810] CDK19 ---> WNT1 0.83912 -0.78694 -
[4811] MXI1 <--- ZNF280B -0.12327 0.19953 -
[4812] ADRBK2 ---> GRM7 0.53935 -0.4166 +
[4813] IL6ST ---> STAT4 0.1409 -0.099294 +
[4814] MAPK14 <--- NCF4 -0.26379 0.013605 +
[4815] GNGT2 <--- HTR1B -0.26888 0.10335 +
[4816] GRB14 <--- PTPRB -0.31265 0.30993 +
[4817] FGFR1 <--- FGFR3 -0.27201 -0.016868 -
[4818] DGCR8 ---> E2F4 0.16628 -0.3793 -
[4819] NPC1 ---> STAT3 0.15293 -0.083314 -
[4820] KIT <--- PDGFB -0.061502 0.075112 +
[4821] ADAM17 ---> NOTCH4 0.044451 -0.039929 +
[4822] ACOX3 <--- PPARA -0.056504 -0.0074276 +
[4823] PEBP1 <--- PRKCI -0.54693 -0.012156 +
[4824] PIK3CA ---> PTEN 0.019177 -0.039333 -
[4825] MAPK10 <--- TRAF3 -0.47605 1.1617 +
[4826] NXF1 ---> SEH1L 0.26121 -0.39709 -
[4827] SYK ---> VAV3 0.032507 -0.097582 +
[4828] ARHGAP32 <--- RHOB -0.48747 0.29572 -
[4829] CDC42 ---> CSNK1A1 1.2491 -0.20304 +
[4830] MAPKAPK3 ---> PHC3 0.40675 -0.26091 +
[4831] ABCA1 ---> USF2 0.35891 -0.45753 -
[4832] MDM2 ---> UBE2I 0.07266 -0.17558 -
[4833] COL9A3 <--- PTK2B -0.11304 -0.10314 +
[4834] KLF1 <--- TCF7L2 -0.026148 0.31266 +
[4835] FZD7 <--- GNB1 -0.12321 0.10912 -
[4836] BHLHE40 <--- HIF1A -0.14108 0.077579 +
[4837] ADCY9 <--- CALML3 -0.63894 0.65698 +
[4838] PSMD10 <--- RFWD2 -0.26785 0.31028 -
[4839] MAP3K14 ---> TNFRSF13C 0.83122 -0.79026 -
[4840] CD36 ---> THBS1 0.46324 -0.17296 -
[4841] ARFGAP1 <--- PTK2 -0.11304 -0.10314 +
[4842] MAP4K1 ---> ZAP70 0.054416 -0.29641 -
[4843] GNA11 <--- TACR2 -1.1303 1.1631 +
[4844] BCAR1 ---> IGF1R 0.10077 -0.066107 -
[4845] ITGA1 <--- LAMB4 -0.062785 -0.0050465 +
[4846] EPO ---> GAB1 0.23235 -0.22914 +
[4847] RAC3 ---> SRGAP2 0.39011 -0.42732 -
[4848] PRKACB ---> RELA 0.92767 -0.99472 +
[4849] CHRM1 ---> PRKCI -0.029433 -0.05892 +
[4850] NFKB1 ---> PIK3R1 0.099912 -0.11223 -
[4851] PSMD1 <--- RELA -0.25054 -0.060722 -
[4852] COL4A1 ---> ITGA3 -0.020349 -0.077049 +
[4853] CCNE1 <--- CDKN1C -1.0232 1.4682 -
[4854] JAK3 ---> PIK3CG 0.020536 -0.05391 +
[4855] CALM1 ---> SMAD2 0.095011 -0.23927 +
[4856] ARHGAP8 <--- RHOF -0.56218 0.37087 -
[4857] HIRA <--- STAT1 -0.32144 0.27626 +
[4858] CCL22 <--- CEBPB -0.14964 0.055907 +
[4859] EP300 ---> GREM1 -0.00078159 -0.0010585 +
[4860] CXCR2 ---> GNAI1 1.1631 -1.1303 +
[4861] PTPRA ---> SPTB 0.59009 -0.69167 +
[4862] GNAI1 <--- KCNJ5 -0.011498 0.05262 -

[4863]	FSHB	<---	HTR6	-0.2522	0.11779	-
[4864]	IL23R	<---	RELA	-0.29081	0.13282	-
[4865]	ITGA11	<---	LAMB3	-0.066621	-0.0090065	+
[4866]	GNB3	--->	LRP5L	0.038734	-0.040664	-
[4867]	CTSG	--->	SERPINB10	0.33364	-0.53505	-
[4868]	CTCF	<---	ESR2	-0.051394	0.053678	-
[4869]	APC2	<---	CSNK1E	-0.10343	0.11983	+
[4870]	FLT4	<---	VEGFB	-0.076002	0.030896	+
[4871]	ATP1B3	<---	PRKACA	-0.26091	0.40675	+
[4872]	CALML5	<---	PPP3CB	-0.26348	0.069904	-
[4873]	NFKB1	--->	TDP2	0.23585	-0.23502	-
[4874]	C1S	<---	C2	-0.017809	0.006907	-
[4875]	ERBB4	<---	PRKD2	-0.057937	0.13298	-
[4876]	GP1BA	--->	PIK3R2	0.030832	-0.16134	+
[4877]	COL6A5	<---	ITGB1	-0.0058928	-0.0043847	-
[4878]	BLM	--->	E2F1	0.016613	-0.062456	-
[4879]	HCK	--->	PIK3CA	0.084876	-0.06481	+
[4880]	BMP1	<---	LAMB3	-0.11868	0.082322	-
[4881]	MPL	<---	SOCS2	-0.06215	-0.055475	+
[4882]	FLT1	--->	INS	0.31135	-0.0084341	-
[4883]	NCOA2	<---	SMARCD3	-0.065181	0.059892	-
[4884]	SMARCA2	--->	TFDP1	0.023162	-0.068048	+
[4885]	BID	<---	RIPK1	-0.22488	0.20249	+
[4886]	AKT1	--->	RPS6KA5	0.67325	-0.79394	+
[4887]	BAZ1A	--->	ZNF274	0.28002	-0.15205	-
[4888]	FYN	--->	ITGA10	0.23404	-0.20204	-
[4889]	EP300	--->	HNRNPA1	0.021187	-0.018539	+
[4890]	CD40	<---	TRAF3	-0.13883	0.036957	-
[4891]	SFRP5	--->	WNT11	1.0317	-1.1063	+
[4892]	BTK	--->	PIK3CG	0.044889	-0.01631	-
[4893]	CRLF2	<---	IL26	-0.18506	0.094523	+
[4894]	JUN	<---	PPARG	-0.12012	0.051652	-
[4895]	DMD	--->	RAC1	0.024496	-0.051331	+
[4896]	ATP2B1	<---	PRKG2	-0.1361	0.12011	+
[4897]	GRM5	<---	KCNJ5	-0.25173	0.20895	+
[4898]	CEBPA	<---	FOXA2	-0.37846	0.32839	-
[4899]	ATF2	<---	PRKACA	-0.54462	0.90502	+
[4900]	FGB	<---	RAP1A	-0.20071	0.19378	-
[4901]	CTNNB1	<---	PRKACG	-1.3965	1.0802	+
[4902]	CSNK1A1	<---	PPP2CB	-0.15489	0.41077	-
[4903]	PTPN1	--->	TYK2	1.0715	-1.2127	+
[4904]	CAMK2G	<---	STAT1	-0.75243	0.87452	-
[4905]	FGF19	--->	IGF1R	0.25346	-0.0044024	+
[4906]	ADRB3	--->	CREB1	0.2521	-0.2255	-
[4907]	GNAL	<---	HTR5A	-1.1303	1.1631	+
[4908]	KAT2B	--->	MYOD1	0.16008	-0.19465	+
[4909]	FRS2	<---	PTPN11	-0.18883	0.3506	-
[4910]	IL1A	--->	JUN	0.14545	-0.19256	-
[4911]	BLK	--->	EPHA1	0.35424	-0.26855	+
[4912]	BUB1	--->	E2F4	0.45782	-0.30098	-
[4913]	APBB1IP	--->	PFN4	0.81658	-0.79397	+
[4914]	PAX2	--->	TCF12	0.086114	-0.12036	-
[4915]	IKBKB	<---	PRKCD	-0.6951	0.07681	+
[4916]	CUX1	--->	STAT5A	0.39922	-0.27828	+
[4917]	CTCF	--->	SUFU	0.11684	-0.071491	+
[4918]	CSNK1G3	--->	CTNNB1	0.65405	-0.84813	+
[4919]	HDAC2	<---	PRAME	-0.34598	0.45686	-
[4920]	CCL15	<---	CCR10	-0.35895	0.21658	-
[4921]	ADCY3	<---	GNAI3	-0.63317	1.0384	-
[4922]	AURKB	<---	RACGAP1	-0.11458	-0.068737	-
[4923]	HLA-DQA2	<---	NFKB1	-0.19974	0.16973	+
[4924]	SNAI2	<---	TCF7L1	-0.018993	0.22714	+
[4925]	GNAO1	--->	GRM3	0.24033	-0.15623	-
[4926]	APC2	<---	CSNK1A1L	-0.10343	0.11983	+
[4927]	GNA11	--->	WNT9A	0.42087	-0.47341	-
[4928]	CALM1	--->	MYLK	0.33394	-0.31058	+
[4929]	NFKB1	--->	SREBF2	0.47748	-0.29534	+
[4930]	ARAP1	<---	RHOQ	-0.48615	0.36733	-
[4931]	FYN	--->	KLRC2	0.63648	-0.45391	+
[4932]	ELF1	--->	PRKDC	0.060853	-0.072573	+
[4933]	LMCD1	--->	PAX5	0.24687	-0.23413	-
[4934]	PDE3B	<---	PRKX	-0.95055	1.4803	+
[4935]	PTK2	--->	WAS	-0.014413	-0.06905	+
[4936]	EBF1	<---	RELA	-0.3618	0.15898	+
[4937]	LCK	--->	PXN	0.50966	-0.41502	+
[4938]	PORCN	--->	WNT2B	0.38949	-0.56891	+

[4939]	MAPK11	<---	PTPN7	-1.4016	1.2548	+
[4940]	LCP2	--->	NRAS	0.80926	-0.79256	+
[4941]	CREB5	<---	PPP1CA	-1.0855	0.69008	+
[4942]	LAMA3	--->	SV2B	0.16735	-0.19531	+
[4943]	PIK3R5	<---	RAF1	-0.4329	0.21261	+
[4944]	ITGA1	--->	THBS2	0.019923	-0.026101	-
[4945]	FGF17	<---	TEK	-0.099363	0.062698	-
[4946]	CSF1R	<---	HGF	-0.045293	0.21963	+
[4947]	PRKACB	--->	SUFU	0.58123	-0.48787	+
[4948]	JAK2	--->	XCR1	-0.039248	-0.12268	-
[4949]	EPAS1	<---	STAT3	-0.53248	0.37489	+
[4950]	HRAS	<--->	RAC1	0.097643	0.097643	-
[4951]	RHOG	--->	WASL	0.041525	-0.34784	+
[4952]	FGF22	--->	FGFR1	1.1329	-1.3925	-
[4953]	INS	--->	NKX2-2	0.26351	-0.33094	-
[4954]	COL4A3	--->	ITGA1	-0.0065074	-0.1017	+
[4955]	ATF6B	<---	PPP1CC	-1.0855	0.69008	+
[4956]	KDR	<---	PDGFC	-0.023732	0.31113	+
[4957]	BMPR1A	--->	SMAD5	0.7946	-0.60139	+
[4958]	ARNT	<---	PGK1	-0.16173	0.16165	-
[4959]	CD247	<---	LCP2	-0.089902	-0.014405	-
[4960]	COL1A1	<---	PCOLCE	-0.15873	0.062969	+
[4961]	ARHGEF4	<---	RHOB	0.091479	0.13323	-
[4962]	MAPK8	<---	TAB1	-0.18043	0.60671	+
[4963]	CAV1	--->	LRP6	0.5925	-0.44675	+
[4964]	LAT	--->	MYC	0.085699	-0.30827	-
[4965]	TAB1	--->	TGFBR2	0.68697	-0.25416	-
[4966]	RHOJ	--->	SYDE1	1.016	-1.5245	-
[4967]	KDR	--->	PIK3CA	0.043149	-0.0082223	+
[4968]	CSF2RB	<---	KRAS	-0.10238	0.10353	-
[4969]	APBB1IP	--->	ITGA2B	0.0057829	-0.064467	-
[4970]	AXIN1	--->	MYC	0.0025426	-0.095079	+
[4971]	CALM1	<---	ITPKB	-0.12569	0.087809	+
[4972]	GRAP2	<---	NFKB1	-0.10928	0.11176	+
[4973]	HOXD1	--->	YY1	0.078944	0.049909	-
[4974]	CD79A	<---	IGKV4-1	-0.12798	0.078578	+
[4975]	ARHGAP29	<---	RHOT2	-0.40786	0.2043	-
[4976]	AXIN2	<---	TEAD1	-0.2017	0.14662	+
[4977]	GCK	--->	HIF1A	0.06932	0.061721	-
[4978]	CHAD	--->	ITGA2B	0.0373	-0.11699	+
[4979]	ARRB2	--->	WNT4	0.23514	-0.34616	+
[4980]	MYO9B	<---	RHOU	-0.54674	0.38058	-
[4981]	IKBKB	<---	MYD88	-0.26344	0.25687	+
[4982]	PIK3CB	<---	PTK2	-0.018679	-0.01522	-
[4983]	KAT2A	--->	RBPJL	0.055633	-0.056694	+
[4984]	PLCZ1	<---	RGS13	-0.082548	0.03305	+
[4985]	PLCG1	<---	PRKCD	-0.027548	0.05522	-
[4986]	C4B	--->	MASP2	0.088812	-0.16376	-
[4987]	CTBP2	<---	FOXF1	-0.28308	0.17988	-
[4988]	NFKBIA	<---	PRKCA	-0.46669	0.38895	+
[4989]	ARHGAP18	<---	RHOT1	-0.58534	0.26947	-
[4990]	FGFR2	--->	MAPK3	0.071367	-0.33653	-
[4991]	ARR3	--->	GNAQ	0.030816	-0.0080738	-
[4992]	RHOV	--->	SYDE1	1.016	-1.5245	-
[4993]	PSMD1	<---	UBE2E1	-0.24701	0.16206	-
[4994]	DAG1	--->	HSPG2	0.01142	-0.00090216	-
[4995]	GRB2	--->	IL11RA	0.10389	-0.19344	-
[4996]	COL12A1	<---	PTK2B	-0.083928	-0.020315	+
[4997]	CAMK2A	--->	NOS3	0.066587	-0.050851	+
[4998]	PRKACG	<---	PTK2	-0.49205	0.28562	-
[4999]	ATF4	--->	MYC	0.030003	-0.17737	-
[5000]	IL11RA	<---	LEP	-0.14339	0.084246	+
[5001]	ATF6B	<---	PPP1CA	-1.0855	0.69008	+
[5002]	TSHB	<---	TSHR	-0.2522	0.11779	-
[5003]	CDC42	<---	DOCK1	-0.31631	-0.038445	+
[5004]	COL4A2	--->	ITGB4	0.014159	-0.041712	+
[5005]	CSF3R	--->	SOCS2	-0.061088	-0.07681	-
[5006]	GNAZ	<---	PTK2	-0.10149	-0.068003	-
[5007]	FYN	<---	PRKCD	0.097057	0.26845	-
[5008]	GPR45	--->	KCNJ9	0.11779	-0.2522	-
[5009]	CRTC2	--->	YWHAZ	-0.0021827	-0.10486	-
[5010]	CACNA1C	<---	HTR2B	-0.36046	0.31836	+
[5011]	DOCK1	--->	PTK2	-0.016505	-0.09364	-
[5012]	MAPK8	--->	NKD2	1.2382	-1.1685	+
[5013]	PDE3A	<---	PRKACG	-0.95055	1.4803	+
[5014]	MAPK14	<---	PTK2	-0.49205	0.28562	+

[5015]	ABL1	--->	CTNND1	0.28027	-0.33962	+
[5016]	MAPK1	<---	PRKAR2B	0.096385	0.19912	+
[5017]	CCNB2	<---	GADD45B	-0.67465	0.65424	+
[5018]	GNA14	--->	WNT11	0.42087	-0.47341	-
[5019]	IL1R1	--->	MAP2K3	0.60204	-0.35123	+
[5020]	MAPK9	--->	ROR2	0.5369	-1.3322	-
[5021]	CTNND1	<---	GFRA1	-0.56977	0.40621	+
[5022]	TAB2	--->	XIAP	0.20956	-0.26057	-
[5023]	LCP2	--->	TREM2	-0.016269	-0.16182	-
[5024]	FLT1	--->	PLCB2	0.042035	-0.0086242	+
[5025]	EGFR	--->	FGF19	0.25979	-0.22928	-
[5026]	PPP4C	--->	YWHAH	-0.03225	-0.31369	-
[5027]	SERPINB13	--->	USF1	-0.052208	-0.1478	-
[5028]	MAPK14	<---	PTPRR	-1.4016	1.2548	+
[5029]	IL12B	--->	IL22RA1	0.051025	0.012893	+
[5030]	CTBP2	--->	GFI1	0.064439	-0.044029	+
[5031]	CREB3L1	<---	PRKCB	-0.63701	0.72046	+
[5032]	EPHA2	--->	FGF13	-0.0091145	-0.017407	-
[5033]	AKT2	--->	FASLG	0.032197	-0.12828	-
[5034]	CASP8	--->	FASLG	0.16302	-0.61452	-
[5035]	MTOR	<---	RRAGD	-0.26816	0.38257	+
[5036]	RAC1	<---	RASGRF1	-0.52428	0.44606	+
[5037]	LHCGR	--->	STAT3	0.47993	-0.37004	-
[5038]	FCER1G	<---	SYK	-0.13461	0.052099	-
[5039]	SRF	<---	STK16	-0.36564	0.35865	-
[5040]	KLK3	<---	PGR	-0.14879	-0.015388	+
[5041]	BMPR1B	--->	MAPK14	0.17987	-0.35209	+
[5042]	CCL18	<---	CCR10	-0.35895	0.21658	-
[5043]	ACTA2	<---	RHOJ	-0.51168	0.52339	+
[5044]	FLT1	<---	PDGFB	-0.015494	0.18761	+
[5045]	RHOD	--->	SYDE1	1.016	-1.5245	-
[5046]	PTPRC	--->	SPRY3	0.17004	-0.22654	-
[5047]	PAX8	--->	THPO	-0.014971	-0.0601	+
[5048]	GNB4	<---	PLCB2	-0.014636	-0.0050336	-
[5049]	LAMC2	--->	MMP13	-0.0045452	-0.11357	-
[5050]	CTNNB1	<---	PCDHA2	-0.19204	0.11503	-
[5051]	ATAD2	--->	E2F4	0.0081868	-0.082946	-
[5052]	PRL	--->	STAT5A	0.44408	-0.32334	+
[5053]	CRKL	--->	ITGA2B	0.032574	-0.078547	-
[5054]	RHOT2	<---	STARD13	-0.14803	-0.10028	+
[5055]	CACNA1C	<---	PPP2CB	-0.27679	0.37996	+
[5056]	AKT1	--->	FOS	0.82132	-1.3257	+
[5057]	ACVR2B	<---	LEFTY2	-0.85941	1.3161	+
[5058]	CSNK1D	--->	SFN	0.079981	-0.071802	+
[5059]	PIK3CA	<---	RRAS2	-0.01026	0.011011	+
[5060]	EP300	--->	HIST1H2BN	0.01343	-0.01897	+
[5061]	CDKN1B	<---	E2F5	-0.00078709	0.10216	-
[5062]	CREBBP	--->	HMOX1	0.00067605	-0.0050208	+
[5063]	IL4R	<---	JAK3	-0.23677	0.10182	-
[5064]	RAC1	--->	RACGAP1	0.68428	-1.011	-
[5065]	BDNF	<---	MMP7	-0.27537	0.095906	+
[5066]	BUB3	<---	UBE2D1	-0.2601	0.27426	-
[5067]	EP300	<---	UBE2C	-0.0073912	0.0036459	-
[5068]	EML4	<---	NRAS	-0.21926	0.18651	-
[5069]	FGF22	--->	PDGFRB	0.48772	-0.57252	+
[5070]	CHAD	--->	ITGB8	0.10169	-0.22271	+
[5071]	ARHGAP39	<---	RHOU	-0.82986	0.43536	-
[5072]	BATF	--->	BHLHE40	0.35865	-0.39442	+
[5073]	LCP2	<---	VAV2	-0.011785	-0.011743	-
[5074]	MAP2K6	<--->	MAPKAPK2	0.31014	0.31014	-
[5075]	CDC42	--->	MTOR	0.36914	-0.27146	+
[5076]	OCRL	<---	RHOBTB2	-0.56259	0.37198	-
[5077]	IRS4	--->	PIK3CB	0.018176	-0.01402	+
[5078]	ATF6B	<---	RPS6KA4	-0.98092	1.2458	+
[5079]	LAMA3	<---	SRF	-0.13401	0.099749	+
[5080]	MAX	<---	RBBP5	-0.23234	0.23228	-
[5081]	KLF1	--->	MXI1	0.33079	-0.20142	-
[5082]	ARAP2	<---	RHOA	-0.48615	0.36733	-
[5083]	GH1	--->	IFNAR2	0.20113	-0.087402	+
[5084]	APOA4	--->	SAR1B	0.22797	-0.4659	-
[5085]	LATS1	<---	LIMD1	-0.694	0.8544	+
[5086]	ACTB	<---	PTK2	-0.28708	0.061438	+
[5087]	LAMA1	--->	SV2B	0.16735	-0.19531	+
[5088]	COL6A2	<---	ITGB5	-0.01668	-0.0099212	-
[5089]	COL18A1	--->	CTSD	-0.0096811	-0.036345	-
[5090]	ARHGAP24	<---	CDC42	-0.49576	0.47865	-

[5091]	RAC1	<---	VEGFA	-0.00043626	0.020385	+
[5092]	HRAS	--->	RPS6KA3	0.39663	-0.23906	-
[5093]	EZH1	<---	FOXA1	0.024351	0.095014	+
[5094]	COL1A2	<---	LEPREL1	-0.18291	0.065305	+
[5095]	CX3CR1	<---	GRK4	-0.28786	0.33635	+
[5096]	JUP	<---	PIK3R1	-0.1265	0.058829	-
[5097]	MLST8	--->	TSC1	0.32571	-0.30322	-
[5098]	CBX8	<---	MAPKAPK3	-0.22919	0.35645	+
[5099]	IKBKB	<---	TAB1	-0.30669	0.5692	+
[5100]	CTCFL	<---	ZNF398	-0.039841	-0.037992	-
[5101]	MAP2K7	<---	RAC3	-0.20304	1.2491	+
[5102]	AKT2	<---	RASA1	0.020878	0.14202	+
[5103]	DLG1	<---	PIK3CA	0.0026535	0.0054411	-
[5104]	CHRM4	<---	PKN1	-0.1993	0.11589	-
[5105]	CCR9	--->	CXCL9	0.21658	-0.35895	-
[5106]	IL22RA1	<---	SOCS5	-1.0456	0.57217	+
[5107]	APP	<---	DAND5	-0.0084882	0.00044769	+
[5108]	GRIN2A	<---	GRM1	-0.36981	-0.05834	+
[5109]	CREB3L4	<---	PRKACB	-0.57905	1.0858	+
[5110]	GNB1	<---	HTR7	-0.57296	0.69217	+
[5111]	ARHGAP33	<---	RHOT1	-0.17154	0.051113	-
[5112]	CCND2	--->	RBL2	0.2465	-0.47102	+
[5113]	EP300	--->	RBBP4	0.007799	-0.0077682	-
[5114]	AVPR1A	--->	GNA11	1.1631	-1.1303	+
[5115]	ARHGAP5	<---	RAC1	-0.078715	0.62868	+
[5116]	CREB3L3	<---	RPS6KA4	-0.98092	1.2458	+
[5117]	LPAR4	<---	RPS6KA5	-0.28028	0.031945	-
[5118]	COL6A2	<---	ITGB7	-0.025688	-0.014544	-
[5119]	COL5A2	--->	ITGA7	0.0052169	-0.090529	+
[5120]	PSMD1	--->	TNKS2	0.08803	-0.053465	+
[5121]	DDX60	<---	STAT1	-0.19474	0.15956	+
[5122]	PRKCA	--->	YWHAB	0.25075	-0.36987	+
[5123]	NFKB1	<---	PRKACB	-0.059594	0.20986	+
[5124]	PDPK1	--->	UBE2V1	0.48136	-0.060419	+
[5125]	CSNK1G3	<--->	CSNK2A1	0.18701	0.18701	-
[5126]	MYL2	<---	TNNT1	-0.6217	0.55026	-
[5127]	GAB2	<---	TRAF6	-0.63909	0.87363	-
[5128]	ETS1	--->	MMP2	-0.0045452	-0.11357	+
[5129]	ANGPT2	<---	FYN	-0.47507	0.64427	-
[5130]	MTA3	--->	TBP	0.22839	-0.2383	+
[5131]	ITGB3	--->	VAV3	0.022701	-0.0086843	+
[5132]	CSNK1G3	--->	LRP6	0.4586	-0.32071	-
[5133]	HIST2H3A	<---	NFKB1	-0.29753	0.29301	-
[5134]	CTNNB1	<---	HDAC2	-0.1943	0.17936	+
[5135]	AJUBA	--->	NR3C1	0.16465	-0.25052	-
[5136]	GNGT2	<---	HTR1E	-0.26888	0.10335	+
[5137]	NFKBIA	--->	SREBF1	0.33109	-0.44329	-
[5138]	AKT3	--->	NFKBIA	0.27146	-0.35293	+
[5139]	PRKCB	--->	SHC1	0.468	-0.43947	-
[5140]	GLI3	<---	PSMD4	-0.096677	0.23239	+
[5141]	COL6A1	--->	ITGA10	-0.006085	-0.062785	+
[5142]	FGF4	--->	KIT	0.015796	-0.016964	+
[5143]	RHOC	--->	TRIO	0.11717	0.0097792	-
[5144]	GH2	<---	JAK3	-0.22013	0.087709	-
[5145]	PLA2G4E	<---	PRKCA	-0.21202	0.18036	+
[5146]	FZD8	<---	WNT3A	-0.78209	0.63734	+
[5147]	CCR9	--->	PF4V1	0.21658	-0.35895	-
[5148]	CASP10	--->	XIAP	0.068027	-0.38238	-
[5149]	KCNQ3	<---	PRKG1	-1.0533	0.74543	-
[5150]	FGFR2	<---	HGF	-0.15508	0.091325	+
[5151]	ITGB1	<---	RPS6KB1	-0.14072	0.05669	-
[5152]	ARRB1	--->	WNT7A	0.23514	-0.34616	-
[5153]	MAPK9	<---	TNF	0.048942	0.10494	-
[5154]	JUND	<---	WWOX	-0.4009	0.25664	-
[5155]	AKT1	--->	PTPN11	0.46491	-0.49912	-
[5156]	DRD2	<---	PPP2CB	-0.15069	0.25329	-
[5157]	CREB1	<---	PRKCB	-0.38747	0.43588	+
[5158]	ALB	--->	USF1	0.0087214	-0.36568	-
[5159]	EBF1	--->	POU2F2	0.26198	-0.27059	+
[5160]	ADCY3	<---	CAMK2B	-0.44346	0.58749	+
[5161]	MYL2	<---	MYLK3	-1.1685	1.2382	+
[5162]	DRD1	<---	GRK7	-0.28786	0.33635	+
[5163]	ARHGAP28	<---	RHOJ	-1.5245	1.016	-
[5164]	PIK3CD	<---	PTK2	-0.018679	-0.01522	-
[5165]	PGK1	--->	STAT3	0.44408	-0.32334	-
[5166]	PHKG2	<--->	PRKACB	0.31014	0.31014	-

```
[5167]  AKT3     --->    NFKBIB  0.28123 -0.28841        +
[5168]  BMP7     --->    MYC     -0.085372       -0.1001 -
[5169]  CCL26    <---    CCR1    -0.35895        0.21658 -
[5170]  MAPK3    --->    RXRG    0.17534 -0.43654        +
[5171]  CACNA1C <---     GNAI2   -0.17558        0.23994 +
[5172]  ADCY8    <---    PRKCD   -0.47485        0.42125 +
[5173]  PDGFRB   <---    PIK3CG  -0.005476       0.01077 -
[5174]  MAPK11   --->    PLA2G4A 0.18871 -0.84715        +
[5175]  DRD3     --->    GNB4    1.1575  -0.92616        +
[5176]  CDC25C   <---    CDK2    -0.97911        0.26855 +
[5177]  FABP4    <---    PPARG   -0.078543       -0.026969       +
[5178]  CSNK1A1 --->     PSMD14  0.2441  -0.083683       -
[5179]  CTCF     --->    IKZF3   -0.0037425      -0.012548       +
[5180]  HDAC3    <---    PPARGC1A        0.10083 0.10593 -
[5181]  CCL22    <---    CXCR6   -0.35895        0.21658 -
[5182]  CD19     --->    HDAC2   0.67635 -0.54112        -
[5183]  ARRB2    --->    AXIN2   0.0088597       -0.049113       +
[5184]  B3GALTL --->     SPON1   -0.011484       -0.071046       +
[5185]  ACTG2    <---    MMP8    -0.29263        0.085904        +
[5186]  PLXNA1   <--->   PLXNA2  0.12735 0.12735 -
[5187]  COL8A2   <---    LEPRE1  -0.11204        0.03851 +
[5188]  AMHR2    <---    GDF5    -0.85941        1.3161  +
[5189]  ATP1A2   --->    PIK3R5  0.14537 -0.20815        -
[5190]  MYC      <---    PRAME   -0.30043        0.10255 -
[5191]  CHRM4    --->    KCNJ5   0.11779 -0.2522 -
[5192]  PIK3R5   <---    PRKCD   -0.52882        0.41473 -
[5193]  ITGA10   <---    LAMB2   -0.045111       0.0073643       +
[5194]  CACNB2   <---    PPP2R1B -0.20392        0.21525 +
[5195]  COL2A1   --->    ITGA11  0.0052169       -0.090529       +
[5196]  CEBPB    --->    HOXB9   0.15908 -0.24396        +
[5197]  MYL4     <---    TPM2    -0.22759        0.17706 -
[5198]  INSR     <---    PDGFA   -0.021519       0.16712 +
[5199]  SIK2     <--->   STK11   0.31014 0.31014 -
[5200]  CD14     --->    SOCS1   0.086513        -0.48565        -
[5201]  COL7A1   <---    FYN     -0.12806        0.15161 -
[5202]  ADCY9    <---    PRKX    -0.63235        0.8673  -
[5203]  GRK1     --->    HTR5A   0.33635 -0.28786        +
[5204]  GH2      --->    IL6R    0.12377 -0.18387        +
[5205]  CSNK1E   --->    NPAS2   0.3669  -0.29216        +
[5206]  MAPK8    <---    PTPN5   -1.4016 1.2548  +
[5207]  CCND3    <---    E2F3    -0.39339        0.45542 -
[5208]  FGF19    --->    KIT     0.015796        -0.016964       +
[5209]  COL8A1   <---    LEPRE1  -0.11204        0.03851 +
[5210]  KCNS3    <---    PRKACB  -0.44517        0.61419 +
[5211]  ITGA11   <---    LAMA1   -0.041507       -0.0065623      +
[5212]  ADRB3    --->    GNAI3   1.1631  -1.1303 +
[5213]  KCNJ5    <---    PRKACB  -0.26091        0.40675 +
[5214]  CSF2RA   --->    SOCS1   -0.014037       -0.24629        -
[5215]  GNB2     <---    HTR5A   -0.92616        1.1575  +
[5216]  RXRA     --->    WNT1    -0.10298        -0.28478        -
[5217]  NFKB1    --->    PSMD10  0.1433  -0.049608       -
[5218]  IGF1R    <---    PTPN11  0.12327 0.24681 -
[5219]  MAP2K6   <---    MAP3K1  -1.3549 1.5096  +
[5220]  KPNA2    <---    RANBP2  -0.33056        0.23067 +
[5221]  SP1      --->    TP53    0.1064  -0.015277       -
[5222]  FOS      <---    MAPK8   -0.57905        1.0858  +
[5223]  HTT      --->    RHOG    0.21591 -0.31065        -
[5224]  CSNK1A1L         --->    SAG     0.24561 -0.15935        -
[5225]  ERBB3    --->    NRAS    0.17363 -0.11617        +
[5226]  TAB1     <---    TRAF2   -0.033705       0.072988        +
[5227]  CDC42    <---    ITSN1   -0.11028        0.16745 +
[5228]  NFATC4   --->    NPPB    0.28326 -0.50063        +
[5229]  GNB1     --->    GRIK3   0.25774 -0.19153        -
[5230]  BDKRB2   --->    KCNJ5   0.11779 -0.2522 -
[5231]  ELANE    --->    NID1    0.17438 -0.15772        +
[5232]  IGSF1    --->    REST    0.26609 -0.052319       -
[5233]  IL5RA    <---    SOCS4   -0.39643        0.2841  +
[5234]  NCOA1    <---    NR1D1   -0.054726       0.035483        +
[5235]  CCR7     --->    CXCL3   0.21658 -0.35895        -
[5236]  BCR      <---    RHOB    -0.20131        0.10561 -
[5237]  RAP1A    <---    RASGRP1 -0.17492        0.5048  +
[5238]  AXIN1    <---    TCF7L2  -0.7238 0.68508 -
[5239]  AJUBA    --->    LATS2   0.8544  -0.694  +
[5240]  ITGA2B   --->    TNN     -0.025002       -0.077063       -
[5241]  RXRA     --->    SREBF2  -0.084418       -0.26838        +
[5242]  NFATC4   --->    PPP3R2  -0.039001       -0.22539        -
```

[5243]	IFNG	--->	IL12RB2	0.081194	-0.1812	+
[5244]	FCER1G	<---	ZAP70	-0.13461	0.052099	-
[5245]	LCP2	<---	MAP3K1	-0.3665	0.30421	-
[5246]	JAK2	--->	NRG1	0.10197	-0.1694	-
[5247]	C4B	--->	IGKV4-1	0.22213	-0.34903	-
[5248]	BAD	<---	PRKACB	-0.56837	0.82969	+
[5249]	E2F3	--->	TFDP1	-0.020679	-0.077916	-
[5250]	AKT1	--->	FRS3	0.18047	-0.25572	-
[5251]	CD4	<---	GNAZ	-0.052939	0.061543	+
[5252]	IL20RA	--->	TYK2	0.48376	-0.37166	+
[5253]	NSMAF	<---	TRADD	-0.33356	0.22872	+
[5254]	PEBP1	<---	PPP2CA	-0.27212	0.38907	-
[5255]	AKT1	--->	PDGFB	0.092861	-0.15518	-
[5256]	HIPK2	--->	SRF	0.35865	-0.36564	-
[5257]	PLCG2	<---	PRKCE	-0.0064763	0.024762	-
[5258]	PPP2CA	--->	SAG	0.22953	-0.1516	-
[5259]	IL12RB2	--->	STAT6	0.084359	-0.061324	+
[5260]	GNAI2	<---	MAPK14	-0.22953	0.50225	-
[5261]	GHR	<---	PTPN6	-0.26697	0.28395	+
[5262]	CACNA1D	<---	PPP1CC	-0.51246	0.42555	+
[5263]	BRCA1	<---	SRF	-0.007544	-0.0066241	+
[5264]	ITGB6	<---	LAMB2	-0.011824	0.005707	+
[5265]	MAP2K4	<---	MAP3K9	-0.066655	0.28419	+
[5266]	IL1R1	--->	NFKB1	0.21125	-0.11961	+
[5267]	GNAQ	--->	WNT8B	0.42087	-0.47341	-
[5268]	DTX3L	--->	NOTCH3	0.04408	0.031528	-
[5269]	CSNK1D	<---	PPP6C	-0.12622	0.14153	-
[5270]	DHX38	<---	RANBP2	-0.086689	0.051515	+
[5271]	CTNND1	--->	GRIA2	0.29364	-0.25911	+
[5272]	ITGB1	<---	RASSF5	-0.079982	0.050735	+
[5273]	CAMK2G	--->	GRIN2A	0.94748	-0.64695	-
[5274]	ARF1	--->	GRASP	-0.070386	-0.083158	-
[5275]	DOK1	<---	PDGFRB	0.0012647	0.51189	+
[5276]	CXCL1	<---	DAND5	-0.17891	0.13549	+
[5277]	CDK4	<--->	MAPK3	0.31014	0.31014	-
[5278]	ITGB3	<---	MAPK3	-0.09775	0.14787	-
[5279]	LYN	--->	MAPK11	0.92013	-1.0925	+
[5280]	PIK3CD	<---	RRAS2	-0.01026	0.011011	+
[5281]	ABI2	--->	RAC3	0.16563	-0.13126	-
[5282]	SMAD4	--->	TCF7	1.1041	-1.2424	+
[5283]	ACTB	<---	RAC2	-0.51168	0.52339	+
[5284]	NRAS	--->	PTK2B	-0.052556	-0.089194	-
[5285]	ECT2	<---	EP300	-0.0079191	0.0030842	+
[5286]	GAB3	<---	PRKCA	-0.50087	0.66104	-
[5287]	JAK2	--->	NRAS	0.35008	-0.080048	+
[5288]	RHOC	--->	ROCK1	0.59366	-0.49545	+
[5289]	KSR1	--->	YWHAG	0.11407	-0.07813	-
[5290]	MAPK13	<---	NRAS	-0.12425	0.75653	+
[5291]	GH2	--->	IFNGR2	0.20113	-0.087402	+
[5292]	CXCR4	--->	PPBP	0.21658	-0.35895	-
[5293]	NGF	<---	SHC1	-0.13914	0.061686	-
[5294]	GNAI1	<---	PRKACG	-0.22953	0.50225	+
[5295]	IL4R	--->	SOS1	0.042909	0.036787	+
[5296]	CCL14	<---	CCR10	-0.35895	0.21658	-
[5297]	CXCL12	--->	STAT5A	0.42026	-0.3013	+
[5298]	ZNF274	--->	ZNF454	-0.037992	-0.039841	+
[5299]	ITGA2	<---	LAMA2	-0.038869	-0.0043913	+
[5300]	ACVRL1	--->	SMAD1	0.49955	-0.48017	+
[5301]	ATRX	<---	SRF	-0.2506	0.16717	+
[5302]	MEF2C	<---	NEK6	-1.4506	1.0903	-
[5303]	CSNK1G3	--->	FZD7	0.33941	-0.31414	-
[5304]	FSHB	--->	INHA	0.081905	-0.21463	-
[5305]	FGFR4	--->	SHC1	0.029603	-0.2849	+
[5306]	IRS1	<---	KRAS	-0.029466	0.20812	-
[5307]	CXCR2	<---	PPP2CA	-0.15069	0.25329	+
[5308]	SMAD3	<---	SNIP1	-0.10359	-0.058937	+
[5309]	FLT4	--->	NGF	-0.074067	-0.088225	-
[5310]	IKBKG	<---	TRAF3	-0.63055	0.80437	+
[5311]	DNM2	<---	EBF1	-0.047239	0.069665	+
[5312]	PIK3CA	<---	PTK2B	-0.018679	-0.01522	+
[5313]	GATA3	--->	STAT6	0.26825	-0.19721	-
[5314]	ARRB2	--->	FZD4	0.13556	-0.17053	+
[5315]	CREBBP	--->	SP1	0.016752	-0.010456	+
[5316]	ADCY6	<---	GNAI2	-0.63317	1.0384	-
[5317]	BDNF	<---	SHC2	-0.13914	0.061686	-
[5318]	HSP90AB1	--->	STAT3	0.32218	-0.24977	-

```
[5319]  ARF1     --->    PIK3CA   -0.017251        -0.022083        +
[5320]  BRAF     --->    CAMK2G   0.54794 -0.74558         -
[5321]  CRLF2    <---    SOCS4    -0.17336         0.13301 +
[5322]  MAPK12   --->    RPS6KA4 0.9519  -1.129  +
[5323]  FST      --->    INHBC    0.51875 -0.80349         +
[5324]  PHKG2    <--->   PRKACA   0.31014 0.31014 -
[5325]  FGFR4    <---    HGF      -0.15508         0.091325         +
[5326]  EP300    --->    ZNF341   0.0063184        -0.0057144       +
[5327]  PRKAA1   --->    SREBF1   0.99789 -0.87887         +
[5328]  EXOC2    <---    RAB10    -0.73289         1.1482  +
[5329]  AURKA    --->    NFKBIA   0.60616 -0.41321         +
[5330]  STAT3    <---    TNIP1    -0.32656         0.44036 -
[5331]  GRIN3A   <---    GRM1     -0.36981         -0.05834         +
[5332]  PPP2R1A  --->    RAF1     -0.0018068       -0.11934         +
[5333]  BHLHE40  <---    CLOCK    -0.22744         -0.07309         +
[5334]  MAPK13   --->    MYC      0.75491 -0.9874 +
[5335]  CCNB2    <---    KIF2C    -0.40062         0.4569  -
[5336]  BNC1     <---    GATA2    -0.15205         0.28002 +
[5337]  ACTA2    <---    MMP25    -0.29263         0.085904         +
[5338]  BRCA1    --->    ETS2     0.18634 -0.1985 -
[5339]  EGR2     <---    SRF      -0.46864         0.59885 +
[5340]  BTK      --->    CD79A    0.1727  -0.056154        -
[5341]  PRKCD    --->    YES1     0.26845 0.097057         -
[5342]  MYC      <---    TRIP6    -0.43023         0.26345 -
[5343]  IL4R     <---    PRL      -0.17721         0.073187         +
[5344]  CDK1     --->    NCAPG    0.57437 -0.5963 +
[5345]  ACTG1    <---    MMP25    -0.29263         0.085904         +
[5346]  SOCS2    --->    STAT5B   0.27422 -0.74698         -
[5347]  RHOC     --->    SRGAP3   0.39011 -0.42732         -
[5348]  ORAI1    <---    PRKACB   -0.46035         0.48012 +
[5349]  FGG      --->    PROC     0.14643 -0.18567         -
[5350]  CCR10    <---    GRK4     -0.28786         0.33635 +
[5351]  CCR3     --->    CXCL13   0.21658 -0.35895         -
[5352]  CREB3L4  <---    RPS6KA4 -0.98092         1.2458  +
[5353]  DUSP6    --->    MAPK9    1.353   -1.036  +
[5354]  CRKL     --->    ITGB1    0.030916         -0.04526         -
[5355]  IL11RA   <---    PTPN6    -0.32886         0.27078 +
[5356]  NFATC3   <---    PRKCQ    -0.60816         0.44346 +
[5357]  MAP2K2   --->    PLA2G4A 0.18871 -0.84715         +
[5358]  COL6A6   <---    ITGB1    -0.0058928       -0.0043847       -
[5359]  GATA2    <---    NR3C1    -0.14879         -0.015388        -
[5360]  IL12RB1  <---    IRS1     0.032241         0.14995 -
[5361]  SMAD7    --->    TGFB3    0.060804         -0.24379         +
[5362]  MAP2K6   <---    RIPK2    -0.29078         0.20201 +
[5363]  FGF20    --->    FGFR4    0.40218 -0.67149         -
[5364]  IL13RA1  <---    IL6      -0.18506         0.094523         +
[5365]  CSNK1G3  --->    WNT4     0.50785 -0.4784 -
[5366]  ATF2     <---    IFNG     -0.2191 0.21924 -
[5367]  FOXA2    --->    SMYD3    0.095014         0.024351         +
[5368]  PIN1     <---    YWHAB    -0.12941         -0.092158        -
[5369]  RPS6KA2  --->    SH3GL2   0.066268         -0.19855         +
[5370]  RHOA     <---    S1PR5    -0.13328         0.12209 +
[5371]  NCSTN    --->    NOTCH3   0.28627 -0.21238         +
[5372]  CTNNB1   <---    WNT7A    -0.187  0.023012         +
[5373]  AKT3     --->    SOS1     0.052556         -0.15964         -
[5374]  IL1RAP   --->    IRAK2    0.13056 0.038971         +
[5375]  NFKB1    --->    PSMD14   0.19956 -0.19547         -
[5376]  ABL1     <---    CRKL     -0.25443         0.54622 -
[5377]  ARHGAP20         <---     RAC2    -1.4209 0.96682 -
[5378]  C5       <---    FOXA1    -0.1228 0.04764 +
[5379]  HOXA3    --->    TAL1     0.23888 -0.32236         -
[5380]  NGF      <---    SHC3     -0.13914         0.061686         -
[5381]  CBLB     --->    IL3RA    0.28248 -0.14259         +
[5382]  CEBPB    <---    MAPK3    -0.26091         0.40675 +
[5383]  CBLB     --->    CTNNB1   0.21731 -0.14365         +
[5384]  DUSP9    --->    MAPK11   1.353   -1.036  +
[5385]  NGEF     <---    RHOC     0.091479         0.13323 -
[5386]  COL10A1  <---    LEPREL1 -0.11204         0.03851 +
[5387]  CRKL     --->    KIT      -0.14409         -0.19167         -
[5388]  ARR3     --->    WNT10B   0.23514 -0.34616         -
[5389]  CCNA1    <---    CDKN1C   -1.0232 1.4682  +
[5390]  CRLF2    <---    GH1      -0.18506         0.094523         +
[5391]  BAX      <---    YY1      -0.48088         0.43349 +
[5392]  COL1A1   --->    PTK2B    -0.055436        -0.087563        -
[5393]  COL15A1  <---    FYN      -0.45391         0.63648 -
[5394]  GRIP1    <---    PRKCG    -0.40252         0.33758 +
```

[5395]	JUN	<---	TNNC1	-0.16866	0.13635	-
[5396]	CTNNA2	--->	MMP19	0.38719	-0.55042	-
[5397]	MC1R	--->	TCF7L2	0.13752	-0.010148	-
[5398]	HNRNPC	<---	TAF1	-0.31439	0.36909	+
[5399]	AKT1	--->	EDN1	0.16383	-0.26287	-
[5400]	ARHGEF11	--->	RHOA	0.092701	0.0073174	+
[5401]	ADRBK1	--->	CCR3	0.25327	-0.21301	+
[5402]	IL13RA2	<---	SLA2	-0.19129	0.23232	-
[5403]	IL12RB1	--->	PIK3R1	-0.041749	-0.064276	+
[5404]	ASCL1	<---	TAF1	-0.52432	0.43996	+
[5405]	KRAS	--->	RPS6KA2	0.65574	-0.39233	-
[5406]	FZD6	<---	GNAQ	-0.097804	0.11301	-
[5407]	ITGA4	--->	THBS3	-0.014728	-0.021497	-
[5408]	JAK1	--->	MITF	0.229	-0.31927	+
[5409]	ABL1	--->	TP73	0.071547	-0.098451	+
[5410]	PSMD14	--->	UBE2E1	-0.027689	-0.03145	+
[5411]	PCK1	<---	RARB	-0.27233	0.12096	+
[5412]	CNTFR	--->	SOCS7	0.17881	-0.34174	-
[5413]	PRKCI	--->	SLC2A4	0.099927	-0.1731	+
[5414]	ISL1	<---	NFKB1	-0.10092	0.11682	+
[5415]	CDON	--->	GLI2	0.072511	0.014458	+
[5416]	PUF60	<---	ZBTB33	0.0040614	0.15655	+
[5417]	AKT1	--->	CDKN2D	0.28123	-0.28841	+
[5418]	ITGB3	<---	RHOA	-0.01596	0.026206	-
[5419]	ITGB8	<---	SRC	-0.45391	0.63648	-
[5420]	COL5A3	<---	PCOLCE2	-0.32363	0.21278	+
[5421]	CEBPB	--->	FGFR2	0.12136	-0.30696	+
[5422]	PPARD	<---	TCF7	-0.983	0.9955	+
[5423]	FGFR2	--->	PIK3R1	0.054746	-0.13412	+
[5424]	MEF2C	<---	YAP1	-0.47884	0.21759	-
[5425]	FOXO4	--->	G6PC3	0.4988	-0.56315	+
[5426]	ERBB2	--->	PDGFC	0.23053	-0.20539	-
[5427]	ARRB1	<---	CCR5	-0.15406	0.070342	-
[5428]	HIRA	--->	SIX5	0.1039	-0.10089	-
[5429]	EBF1	--->	KLF1	0.38292	-0.17868	+
[5430]	KRAS	--->	PDGFRA	0.17783	-0.077254	-
[5431]	ATF4	<---	KHSRP	-0.16066	0.25678	-
[5432]	TREM2	--->	VAV3	0.064425	-0.018037	+
[5433]	HDAC1	<---	WWTR1	-0.6075	0.53554	-
[5434]	CIITA	--->	NFKB1	0.061822	-0.041235	-
[5435]	PDPK1	--->	RXRA	0.22813	-0.26832	-
[5436]	CTNNB1	<---	PTPRB	-0.60368	0.4685	+
[5437]	EP300	--->	HIST2H2BF	0.01343	-0.01897	+
[5438]	NTN1	--->	UNC5A	0.0062462	-0.049103	+
[5439]	RELA	--->	RIPK1	-0.015837	-0.045793	-
[5440]	CEBPA	--->	MMP2	-0.0056809	-0.18543	+
[5441]	CTGF	--->	SMAD4	-0.012247	-0.03036	-
[5442]	CRTC2	--->	SIK2	0.23894	-0.32874	-
[5443]	TNF	<---	TNFAIP3	-0.1006	0.014801	-
[5444]	GRIA4	<---	PPP1CC	-0.67094	0.67498	+
[5445]	PRKACB	<---	RASD1	-0.20304	1.2491	+
[5446]	CDK8	<---	TGFB1	-0.0067309	0.13651	+
[5447]	PPP2CB	--->	WWTR1	0.78287	-0.58536	+
[5448]	EDNRA	--->	GNA14	1.1631	-1.1303	+
[5449]	NGFR	--->	PSEN2	0.073135	-0.35882	-
[5450]	CCL2	<---	CXCR4	-0.35895	0.21658	-
[5451]	IFNGR1	<---	SOCS1	-0.31187	-0.11646	+
[5452]	DAND5	--->	GLP1R	0.021163	-0.044865	+
[5453]	CIAO1	<---	FOXA2	-0.43365	0.47146	+
[5454]	MAPK11	--->	NFATC3	0.92767	-0.99472	+
[5455]	HOMER2	--->	PIK3CD	0.0038501	-0.0097611	+
[5456]	ADAM17	--->	FURIN	0.052757	-0.10589	+
[5457]	CSNK1D	--->	FZD5	0.5614	-0.51612	-
[5458]	DAPP1	<---	PIK3R1	-0.037873	-0.020226	+
[5459]	MYO10	<---	NCKIPSD	-0.074465	0.069862	-
[5460]	MAPK9	--->	SMAD4	0.88243	-0.89987	+
[5461]	GATA2	<---	PHF19	-0.093184	-0.011158	-
[5462]	RBM39	<---	ZNF274	0.008509	0.25992	+
[5463]	NOTCH1	<---	RAB6A	-0.33225	0.45312	+
[5464]	KRAS	<---	RASGRP2	-0.24352	0.27322	+
[5465]	PPP2R1A	--->	PTPN11	0.064793	-0.028725	-
[5466]	EIF4E2	<---	EIF4EBP1	-0.67843	0.63979	+
[5467]	SOCS1	<---	TLR6	-0.36531	0.024501	-
[5468]	ARNT	<---	PFKFB1	-0.099193	0.096943	-
[5469]	GADD45B	--->	RARA	0.025734	-0.26567	-
[5470]	CRLF2	<---	IL12B	-0.20337	-0.19263	+

[5471]	DYNC1I2	<---	RAB8A	-0.35969	0.30932	-
[5472]	COL2A1	--->	ITGB1	0.0024845	-0.035647	+
[5473]	ACTG1	<---	BAIAP2	-0.47897	0.40884	+
[5474]	ARRB2	<---	PPP2R1A	-0.26645	0.22611	+
[5475]	CCL4L1	<---	CXCR2	-0.35895	0.21658	-
[5476]	GNA13	--->	PLA2G4B	-0.10668	-0.13723	+
[5477]	ITGA9	--->	THBS2	0.081101	-0.066168	-
[5478]	IKBKG	<---	PEBP1	-0.22054	0.13301	+
[5479]	BCAR1	<---	LCK	0.023313	1.0891	+
[5480]	CCL28	<---	CX3CR1	-0.35895	0.21658	-
[5481]	EIF2AK4	<--->	MAPK11	0.31014	0.31014	-
[5482]	CTNNB1	<---	FZD6	-0.030994	0.0090278	+
[5483]	PIK3R1	<---	TEK	-0.085651	0.12824	+
[5484]	MAPK3	--->	PLAU	0.30397	-0.27947	+
[5485]	FYN	--->	ITGB8	0.63648	-0.45391	-
[5486]	FGF23	--->	PDGFRB	0.48772	-0.57252	+
[5487]	EZH2	--->	HIST1H3A	0.3865	-0.4548	+
[5488]	RAC2	<---	SOS1	-0.33772	0.48674	+
[5489]	KCNQ1	<---	PRKACG	-0.44517	0.61419	-
[5490]	TCEB1	<---	UBE2D3	-0.20492	0.13119	-
[5491]	ADCY1	<---	PRKCB	-0.49495	0.42642	+
[5492]	CSF1	--->	KDR	0.48842	-0.039108	+
[5493]	ETV4	--->	WT1	0.28002	-0.15205	+
[5494]	EPHA4	<---	FGR	-0.26002	0.33922	+
[5495]	ABI1	--->	RAC1	0.34684	0.098669	-
[5496]	FOS	<---	ZNF143	-0.0074278	0.17997	-
[5497]	BCL3	<---	PPM1F	-0.62263	0.79944	-
[5498]	FOXJ3	--->	PAX5	0.19334	-0.21497	-
[5499]	CD40	<---	TRAF6	-0.13883	0.036957	-
[5500]	CACNA1D	<---	GNB5	-0.14673	0.18744	+
[5501]	CD79B	<---	NCK1	-0.18118	0.15241	-
[5502]	APP	<---	MAPK3	-0.023982	0.030823	-
[5503]	CD14	--->	CEBPB	0.11814	-0.34191	-
[5504]	CSK	--->	TCRB	0.63774	-0.47931	+
[5505]	COL26A1	--->	PPIB	-0.024575	-0.068802	-
[5506]	GH2	--->	IL23R	-0.0057724	-0.034956	+
[5507]	EIF4A1	--->	EIF4B	0.23551	-0.20283	-
[5508]	CALML3	--->	MYLK4	0.41687	-0.23601	+
[5509]	CEBPD	--->	TNF	-0.056467	-0.069238	+
[5510]	CSF1	<---	PTPN1	-1.0485	0.89796	+
[5511]	ADCY7	<---	CALM1	-0.63894	0.65698	+
[5512]	LAMB2	--->	SV2B	0.20521	-0.20511	+
[5513]	EGR1	--->	NKD1	0.40918	-0.28984	+
[5514]	BTRC	--->	YAP1	0.76809	-0.28452	+
[5515]	BCL2	<---	CEBPB	-0.79485	0.53967	+
[5516]	CCL20	<---	CCR2	-0.35895	0.21658	-
[5517]	ARHGAP40	<---	RHOH	-1.5245	1.016	-
[5518]	KPNA3	<---	NUP43	-0.19114	0.12101	+
[5519]	EGFR	--->	ITGA1	0.030696	-0.05258	-
[5520]	CDKN1B	<---	ITGB3	-0.020843	0.050557	+
[5521]	CD3E	<---	HLA-DQA2	-0.33924	0.11522	+
[5522]	BAMBI	--->	FZD7	0.43771	-0.51806	+
[5523]	CACNB1	<---	PPP2R2A	-0.06614	0.10562	+
[5524]	FGF20	<---	HRAS	-0.15714	0.23194	-
[5525]	BCL10	<---	FOS	-0.16034	0.26912	+
[5526]	FGB	<---	ITGB3	-0.076563	0.042988	-
[5527]	CDKN1B	<---	UBE2C	-0.21389	0.3163	-
[5528]	ARPC1A	<---	WASF2	-0.099765	0.10042	+
[5529]	PDGFB	--->	PIK3CA	-0.0022094	-0.0096636	+
[5530]	MAPK11	--->	TRPV1	0.54889	-0.41958	+
[5531]	BMPR2	<---	MAP3K7	-0.91029	0.97115	+
[5532]	EPHB1	--->	MAP2K1	0.3522	-0.4085	+
[5533]	ARHGAP32	<---	RAC3	-0.48747	0.29572	-
[5534]	ARHGEF1	<---	GNB1	-0.034933	0.022143	+
[5535]	GABBR2	<---	GNB5	-0.067008	0.092163	-
[5536]	EPO	<---	PLCG1	-0.024299	0.046317	-
[5537]	CSF2RB	<---	PRKACG	-0.15198	0.17732	+
[5538]	G6PC	<---	PRKAG3	-0.20283	0.012951	+
[5539]	ADCYAP1R1	--->	GNB2	0.059668	-0.060578	+
[5540]	AR	<---	HSP90B1	-0.096063	0.066601	-
[5541]	DKK4	--->	WNT10B	0.54164	-0.69755	+
[5542]	KRAS	--->	PIM1	1.2491	-0.20304	+
[5543]	GAB1	<---	PIK3R2	-0.32654	0.0213	-
[5544]	HSPG2	<---	TLL2	0.003657	0.005972	+
[5545]	CHRM4	--->	GNB2	1.1575	-0.92616	+
[5546]	CNTF	--->	IL5RA	0.11381	-0.12599	+

[5547]	CRLF2	<---	IL10	-0.18506	0.094523	+
[5548]	ABR	<---	RHOQ	-0.20131	0.10561	-
[5549]	GNAI1	--->	GRM3	0.24033	-0.15623	-
[5550]	PRKD3	--->	STAT4	0.84558	-0.79252	-
[5551]	CAPN15	--->	SIX5	0.23601	-0.28692	-
[5552]	IGF1R	<---	VEGFC	-0.033666	0.27735	+
[5553]	DMD	--->	MYL4	0.058733	-0.093061	-
[5554]	TGFA	--->	TP53	0.026194	-0.015299	-
[5555]	HIF1A	<---	NR3C1	0.034569	0.071935	-
[5556]	EP300	<---	IL1B	-0.0053955	-0.00011903	-
[5557]	ARR3	--->	GNA14	0.030816	-0.0080738	-
[5558]	BRCA1	--->	RXRB	0.038201	-0.366	+
[5559]	GRAP2	--->	TCRA	0.13256	-0.17782	-
[5560]	BIRC3	--->	NSMAF	0.073579	-0.10387	+
[5561]	CSNK1A1	--->	WNT7B	0.83912	-0.78694	-
[5562]	CCR7	--->	CXCL5	0.16688	-0.23229	-
[5563]	IL3	--->	OSMR	-0.018386	-0.067781	+
[5564]	MAPK13	<---	TRAF6	-0.47605	1.1617	+
[5565]	IL6	<---	PRKCD	-0.33821	0.25182	-
[5566]	DVL3	<---	GNAQ	-0.59426	0.46386	+
[5567]	RAC1	<---	YES1	0.16613	1.0122	+
[5568]	CTNNB1	<---	VCL	-0.81423	0.73494	-
[5569]	PRKCZ	--->	STAT5B	0.53838	-0.49624	-
[5570]	AKT2	<---	PPP2CA	-0.049067	0.049194	-
[5571]	MAPK13	--->	RPS6KA5	0.9519	-1.129	+
[5572]	EPOR	<---	IL26	-0.17721	0.073187	+
[5573]	CEACAM8	--->	ITGB1	-0.019146	-0.021049	+
[5574]	LRP5L	<---	SAG	-0.11868	0.082322	-
[5575]	GNGT2	--->	PREX1	0.11399	-0.10327	+
[5576]	ARR3	--->	FZD1	0.13556	-0.17053	+
[5577]	CAMK2B	--->	CREBBP	0.010341	-0.008238	+
[5578]	CTCF	--->	ZIM2	-0.0066414	-0.044983	+
[5579]	GSK3B	--->	SEMA3A	0.41609	-0.28631	+
[5580]	CD4	<---	TCRB	-0.078726	0.04656	-
[5581]	ATF3	<---	CEBPB	-0.16066	0.25678	+
[5582]	IL11RA	--->	SOCS2	-0.089456	-0.13382	-
[5583]	EGLN3	<---	PRKCB	-0.91465	0.45016	+
[5584]	CHEK2	--->	RNF8	0.014135	-0.032053	-
[5585]	FOS	<---	MMP1	-0.07712	-0.0036762	-
[5586]	ASAP2	<---	RAC1	-0.17153	0.091225	-
[5587]	EPHB4	<---	NCSTN	-0.03656	-0.029491	+
[5588]	PLCD1	<---	RGS4	-0.082548	0.03305	+
[5589]	CACNA1D	<---	PPP2R3B	-0.36974	0.36606	+
[5590]	ATR	<---	FANCB	-0.053787	0.046114	-
[5591]	HDAC2	<---	TCF7L1	-0.12264	0.37175	-
[5592]	EPOR	<---	TYK2	-0.37792	0.17946	-
[5593]	ATF1	<---	IL1B	-0.1182	0.14174	-
[5594]	IL22RA1	--->	TYK2	0.48376	-0.37166	+
[5595]	ETV4	--->	USF1	0.43996	-0.52432	-
[5596]	CDK2	--->	HCFC1	0.57211	-0.50839	+
[5597]	BMPR1A	--->	BMPR2	-0.072808	-0.20314	+
[5598]	RASGRF2	--->	RHOB	0.44606	-0.52428	+
[5599]	IRS1	<---	MAPK8	-0.20734	0.74413	+
[5600]	STAT2	<---	ZC3HAV1	-0.32439	0.44837	-
[5601]	NRAS	<---	SRC	0.099664	0.61281	+
[5602]	CTBP2	--->	SMAD4	-0.063311	-0.074632	+
[5603]	PLCB3	<---	PRKCA	-0.044086	0.057441	-
[5604]	PSMD4	<---	UBE2E1	-0.20492	0.13119	-
[5605]	PRKAR2B	--->	STMN1	0.28956	-0.40795	+
[5606]	AKT1	--->	RXRB	0.46238	-0.59236	+
[5607]	HIF1A	<---	UBE2D1	0.067418	0.15494	-
[5608]	PIK3CD	<---	TYK2	-0.051538	0.022717	+
[5609]	MAPK3	<---	ROCK2	-0.73578	0.41942	-
[5610]	BLK	--->	RGS1	0.53308	-0.35367	+
[5611]	PRKCA	--->	YWHAQ	0.25075	-0.36987	+
[5612]	GRIA2	<---	LYN	-0.41064	0.56795	-
[5613]	ITPR1	<---	JUN	-0.12318	0.23964	+
[5614]	HRAS	--->	PIM1	0.75653	-0.12425	+
[5615]	RASA1	--->	SPRY4	0.35823	-0.2629	+
[5616]	PIK3CA	--->	TRPV1	0.015716	-0.03037	+
[5617]	F2R	--->	GNAZ	1.1631	-1.1303	+
[5618]	GNB1	<---	PRKCB	-0.27923	0.22728	+
[5619]	FOXO1	--->	GABARAPL1	1.0193	-0.41286	+
[5620]	LAMC2	--->	MMP12	-0.0045452	-0.11357	-
[5621]	ITGAL	--->	VAV2	1.8011e-005	-0.0061352	+
[5622]	FOS	<---	NFATC3	-0.58249	0.48528	+

[5623]	CEBPA	--->	GUCY2C	0.1339	-0.15545	+
[5624]	CBX2	<---	MAPK11	-0.45438	0.48369	+
[5625]	INPP5B	<---	RHOT2	-0.35613	0.16234	-
[5626]	HIF1A	--->	SREBF1	0.09493	-0.13863	-
[5627]	PAK3	<---	RAF1	-0.76991	0.68927	-
[5628]	NCOA1	<---	SP1	-0.017564	0.042925	-
[5629]	RAC1	--->	WASL	0.041525	-0.34784	-
[5630]	LIMK1	--->	SEMA3A	0.2572	-0.28117	-
[5631]	CDKN1A	<---	UBE2D1	-0.21389	0.3163	-
[5632]	PPM1F	--->	SPI1	0.71495	-0.56168	-
[5633]	ARHGAP6	<---	RHOF	-1.5245	1.016	-
[5634]	ADCY1	<---	IL1A	-0.34692	0.33309	+
[5635]	IL7R	<---	JAK3	-0.38855	0.16968	-
[5636]	GNAI2	<---	MAPK3	-0.22953	0.50225	-
[5637]	FZD4	<---	GNA14	-0.22143	0.24435	-
[5638]	CCL25	<---	CCR6	-0.35895	0.21658	-
[5639]	MAPK3	<---	TGFB1	-0.0067309	0.13651	+
[5640]	MYL1	<---	TNNC2	0.034293	0.19015	-
[5641]	CCNT2	<---	EGR1	0.056283	0.1072	-
[5642]	RALA	<---	RALGAPA2	-0.50061	0.67654	+
[5643]	PTPRC	--->	SPRY1	0.17004	-0.22654	-
[5644]	CDH2	<---	PTPN11	-0.34741	0.19481	-
[5645]	ARHGAP36	<---	RHOT2	-0.58534	0.26947	-
[5646]	ITGB3	<---	PTPN1	-0.42062	0.35681	-
[5647]	FOXF1	--->	USF1	0.81123	-0.8599	-
[5648]	CTCFL	<---	RXRA	-0.082047	-0.064572	+
[5649]	ARHGAP31	<---	RAC2	-1.5245	1.016	-
[5650]	GNAI2	--->	GRM6	0.24033	-0.15623	-
[5651]	ELK3	<---	FOXA1	-0.37846	0.32839	+
[5652]	CCL15	<---	CCR5	-0.35895	0.21658	-
[5653]	NR3C1	--->	POU2F1	0.10711	-0.27075	+
[5654]	MAP2K1	<---	PPP2R1B	-0.25679	0.26693	+
[5655]	CDK1	--->	CPEB4	1.2133	-0.47902	+
[5656]	FZD7	<---	WNT4	-0.78209	0.63734	+
[5657]	DUSP1	<---	RASA1	-0.11384	0.18282	-
[5658]	CTBP2	--->	RBPJL	0.061737	-0.1027	+
[5659]	NGFR	--->	SHC2	0.12767	-0.32299	+
[5660]	MLYCD	<---	PRKAA1	-0.34967	0.64801	+
[5661]	CACNB2	<---	PPP2R3A	-0.060248	0.10014	+
[5662]	ATP2B1	<---	PRKACB	-0.060388	0.088587	+
[5663]	FGF13	--->	KDR	0.13624	-0.0034489	+
[5664]	HGF	--->	PIK3CA	-0.0062915	-0.0083524	+
[5665]	CD36	--->	SOCS1	-0.15494	-0.25139	-
[5666]	ARHGAP5	<---	RHOU	-0.078715	0.62868	-
[5667]	CCR8	--->	XCL1	0.21658	-0.35895	-
[5668]	CD3G	<---	LAT	-0.099047	-0.0092662	-
[5669]	IL12B	--->	JUN	0.010648	-0.12085	-
[5670]	PML	--->	SIRT1	0.078837	-0.17835	-
[5671]	MYLK3	<---	PAK2	-1.2674	1.5523	+
[5672]	CCR8	--->	GNAI1	1.1631	-1.1303	+
[5673]	ARHGAP11A	<---	RHOB	-1.5245	1.016	-
[5674]	FGF5	<---	HRAS	-0.15714	0.23194	-
[5675]	PRLR	--->	SOCS3	-0.098904	-0.12483	-
[5676]	PRKCG	--->	RAF1	0.18571	-0.23412	+
[5677]	ADCY3	<---	CAMK2A	-0.44346	0.58749	+
[5678]	CDH1	<---	ENAH	-0.24513	0.11346	-
[5679]	CCL18	<---	CCR7	-0.35895	0.21658	-
[5680]	EGF	<---	FGR	-0.13921	0.19166	+
[5681]	ITGA9	--->	THBS3	-0.014728	-0.021497	-
[5682]	AR	<---	BRCA1	-0.23229	0.18218	+
[5683]	SELE	<---	TNF	-0.23381	0.15272	+
[5684]	MYBPC2	<---	MYH8	-0.15073	0.094518	+
[5685]	ARHGAP26	<---	RHOF	-0.65972	0.48549	-
[5686]	HRAS	<---	RET	-0.063585	0.096555	+
[5687]	GNGT2	--->	PIK3R5	0.38327	-0.57546	+
[5688]	GNAI3	<---	RGS5	-1.2531	1.2063	+
[5689]	CCND1	<---	PSMD4	-0.42437	0.47972	+
[5690]	ARHGAP30	<---	RHOT1	-0.58534	0.26947	-
[5691]	CSNK2A2	--->	DVL1	0.52194	-0.62577	+
[5692]	HRAS	<---	INPP5D	-0.028509	0.10886	-
[5693]	C2	<---	C5	-0.12897	0.079508	-
[5694]	CCL16	<---	CXCR3	-0.35895	0.21658	-
[5695]	CEBPB	--->	RBPJ	0.083738	-0.053804	+
[5696]	GRK4	--->	SSTR4	0.33635	-0.28786	+
[5697]	FYN	<---	PTPN11	-0.41996	-0.082668	-
[5698]	BMP1	<---	LAMA3	-0.073083	0.048831	-

[5699]	IGF1R	<---	NRAS	0.013385	0.17663	-
[5700]	ETS1	<---	VEGFB	-0.0418	-0.012527	-
[5701]	HOXB8	--->	TCF12	0.23888	-0.32236	-
[5702]	NFKB1	<---	PPP2R1B	-0.080482	0.09153	-
[5703]	COL24A1	--->	ITGA5	-0.013036	-0.10803	+
[5704]	MET	--->	PDGFA	0.077225	-0.11958	-
[5705]	SOCS3	--->	STAT1	0.56704	-0.92188	-
[5706]	ITGA2	<---	LAMC3	-0.062785	-0.0050465	+
[5707]	CBLB	--->	FCER1A	0.2466	-0.10039	-
[5708]	CSNK1G3	--->	FZD10	0.33941	-0.31414	-
[5709]	ITGA2B	<---	PTPN1	-0.5111	0.36131	-
[5710]	IFNG	--->	IL2RG	0.081194	-0.1812	+
[5711]	EPHA2	--->	IRS1	0.2575	-0.16351	+
[5712]	MYL12A	<---	NCK2	-0.19951	0.16702	+
[5713]	EPHB1	--->	MMP2	-0.015392	-0.077231	-
[5714]	CSNK1A1	--->	FZD7	0.5614	-0.51612	-
[5715]	COL6A5	--->	ITGA7	-0.0067115	-0.019363	+
[5716]	IL12B	<---	NOS2	-0.066959	0.025158	+
[5717]	FOXO4	--->	GADD45G	0.36388	-0.62421	+
[5718]	INHA	--->	INHBA	-0.050062	-0.38124	-
[5719]	CSF2RB	<---	TYK2	-0.10716	0.10028	-
[5720]	CBL	--->	PLCG2	0.0046457	-0.010821	+
[5721]	BMP4	<---	GLI2	-0.76749	1.0844	+
[5722]	FGF21	--->	IGF1R	0.25346	-0.0044024	+
[5723]	HOXC6	<---	SPI1	-0.24396	0.15908	+
[5724]	APC	<---	SKP1	-0.012802	0.0026103	+
[5725]	CDKN1B	--->	ITGAV	-0.016338	-0.039929	-
[5726]	FZD9	<---	WNT9A	-0.78209	0.63734	+
[5727]	EGFR	--->	SH3GL2	0.13907	-0.11984	-
[5728]	CACNA1D	<---	GNAO1	-0.17558	0.23994	+
[5729]	ITGB7	<---	RHOA	-0.024501	0.04502	+
[5730]	FLI1	<---	GP6	-0.1666	0.15419	-
[5731]	GNB4	--->	WNT10B	0.3343	-0.42584	-
[5732]	AXIN1	--->	DVL2	-0.01881	-0.23402	-
[5733]	STAT1	--->	VEGFB	0.25362	-0.31072	+
[5734]	IGF2	--->	IGFBP4	0.06155	-0.12987	-
[5735]	CDKN1C	<---	DAND5	-0.081131	0.22181	+
[5736]	BDNF	<---	PLG	-0.3018	0.23865	+
[5737]	SMAD4	--->	TFE3	0.11404	-0.20862	+
[5738]	H3F3A	<---	UBE2E1	-0.48509	0.38736	-
[5739]	CDK7	--->	TAF5	0.54982	-0.31609	+
[5740]	DDIT3	<---	MAPK11	-0.15935	0.24561	+
[5741]	GATA4	--->	TCF12	0.26575	-0.31912	-
[5742]	CD36	<---	PRKAA2	-0.49798	0.49396	+
[5743]	CSNK1E	--->	SFN	0.079981	-0.071802	+
[5744]	IL13RA1	<---	PIK3CA	-0.025559	0.0087857	-
[5745]	HSPG2	--->	LRP2	-0.0024025	-0.0096851	-
[5746]	DKK1	--->	WNT2B	0.54164	-0.69755	+
[5747]	HIST2H2BE	<---	KAT2A	-0.12061	0.076698	+
[5748]	DUSP5	--->	MAPK1	1.353	-1.036	+
[5749]	AKT2	--->	GAB3	0.24623	-0.074658	-
[5750]	CASP7	--->	NAIP	0.11472	-0.37392	-
[5751]	CBL	--->	CD79A	0.18242	-0.10226	-
[5752]	PPP2R2A	<---	YWHAZ	-0.1213	-0.088103	-
[5753]	LAMC2	--->	MMP3	-0.0045452	-0.11357	-
[5754]	ARF6	--->	FBXO8	-0.027657	-0.28516	-
[5755]	MAPK1	<---	MATK	-1.0925	0.92013	+
[5756]	ITGAM	--->	PIK3R5	-0.067056	-0.12453	+
[5757]	CXCR4	--->	DNM1	0.082554	-0.088002	-
[5758]	CTNNA1	--->	RAP1B	0.60916	-0.52573	-
[5759]	JAM2	<---	PARD3	-0.059331	0.09631	+
[5760]	CEBPA	--->	USF1	0.43996	-0.52432	-
[5761]	FLT1	<---	PGF	-0.023732	0.31113	+
[5762]	CXCR4	--->	PF4V1	0.21658	-0.35895	-
[5763]	FIGF	<---	NGFR	-0.32359	0.039957	-
[5764]	CD3G	<---	HCK	-0.45391	0.63648	+
[5765]	ACACB	<---	PRKAG1	-0.95328	1.0154	+
[5766]	FZD3	<---	GNAO1	-0.22143	0.24435	-
[5767]	CSF2RA	--->	STAT3	0.44302	-0.302	+
[5768]	PREX1	<---	RHOA	-0.049683	0.1134	-
[5769]	YY1	--->	ZNF432	-0.037992	-0.039841	+
[5770]	FZD9	<---	SAG	-0.17053	0.13556	-
[5771]	CSF1R	<---	PIK3CB	-0.014221	0.044706	-
[5772]	ATM	<---	DYRK2	-0.21939	0.2553	-
[5773]	FOXO3	--->	G6PC3	0.4988	-0.56315	+
[5774]	CD19	--->	IGKV4-1	0.28143	-0.28221	-

[5775]	EDNRB	--->	GNAO1	1.1631	-1.1303	+
[5776]	PPP1R1B	--->	PPP3R1	0.068088	-0.035483	-
[5777]	GLI2	<---	WWTR1	-0.27643	0.32828	+
[5778]	RHOT2	<---	TRIO	0.024782	0.046226	+
[5779]	ITGA2B	<---	LAMC2	-0.066621	-0.0090065	+
[5780]	FASLG	--->	IKBKG	0.14619	-0.24867	+
[5781]	KLF1	<---	SMARCC1	-0.22248	0.31793	+
[5782]	CTSD	<---	HLA-DQA2	-0.20686	0.068783	-
[5783]	FGF22	<---	MAPK1	-0.47959	0.71493	+
[5784]	MAP2K1	--->	SFN	0.079981	-0.071802	-
[5785]	FLT4	--->	PIK3CA	0.0062554	-0.016711	+
[5786]	MYL5	<---	MYLK2	-0.29873	0.36004	+
[5787]	GP1BA	--->	VWF	0.065267	-0.20741	-
[5788]	FRS2	<---	GRB2	-0.058171	0.11265	-
[5789]	RPS6	<---	RPS6KA3	-0.79542	1.3472	+
[5790]	APP	<---	CASP8	-0.051696	0.011492	+
[5791]	JUNB	<---	MAPK9	-0.47491	0.66358	+
[5792]	ITSN1	--->	RHOT2	0.14654	-0.11477	+
[5793]	GSK3B	<---	PRKCZ	-0.70345	0.00041873	+
[5794]	JUND	<---	SRCAP	-0.13469	0.077313	-
[5795]	GP1BA	--->	PIK3R5	0.43806	-0.67965	+
[5796]	MAPK11	--->	PHC1	0.40675	-0.26091	+
[5797]	FRS2	--->	NGF	0.59283	-0.51101	-
[5798]	GRK4	--->	HTR6	0.33635	-0.28786	+
[5799]	RHOJ	<---	SOS1	-0.33772	0.48674	+
[5800]	TCRA	<---	ZAP70	-0.26201	0.089794	-
[5801]	EGF	<---	PTPN11	-0.15582	0.18272	-
[5802]	CDH1	<---	RAP1A	-0.40807	0.12902	+
[5803]	IFNAR1	<---	IL6	-0.087402	0.20113	+
[5804]	GSK3B	<--->	PRKACG	0.31014	0.31014	-
[5805]	CREB3L3	<---	PRKG1	-1.2233	1.1994	+
[5806]	CDKN1A	--->	JUND	0.15531	-0.10887	-
[5807]	PDGFB	--->	SP3	0.18332	-0.10802	-
[5808]	AXIN1	<---	CSNK1G1	-0.12485	0.13422	+
[5809]	MAPK6	--->	RPS6KA3	0.9519	-1.129	+
[5810]	CXCL1	--->	SP3	0.17326	-0.053263	-
[5811]	MAPK6	--->	SPIB	0.40675	-0.26091	+
[5812]	CSF1R	<---	FGF11	-0.64493	0.77791	+
[5813]	CCNB3	<---	FOXO1	-0.62791	0.70491	+
[5814]	ITGB1	<---	RAP1A	-0.01596	0.026206	+
[5815]	CCNE1	<---	CDKN1B	-1.0232	1.4682	-
[5816]	IL1A	<---	SRF	-0.13827	0.07175	+
[5817]	BMP5	--->	BMPR2	1.3161	-0.85941	+
[5818]	CSNK1G2	--->	FZD5	0.33941	-0.31414	-
[5819]	NCOA1	<---	RARA	-0.054726	0.035483	+
[5820]	KRAS	<--->	RAC3	0.1628	0.1628	-
[5821]	SETDB1	<---	ZNF134	0.012459	0.049815	-
[5822]	LAMB4	<---	PTK2	-0.06966	-0.063654	-
[5823]	PIAS4	--->	SMAD3	0.0061839	-0.83897	+
[5824]	EGR1	<---	PLAU	-0.098327	0.15316	-
[5825]	FOS	<---	MMP13	-0.07712	-0.0036762	-
[5826]	MAP2K4	<--->	MAPK3	0.31014	0.31014	-
[5827]	LRP5	<---	WNT9A	-0.28769	0.39899	+
[5828]	CEBPA	<---	NCOR1	-0.20552	0.17137	+
[5829]	CSNK1A1L	--->	WNT11	0.83912	-0.78694	-
[5830]	CCL4	<---	CCR5	-0.35895	0.21658	-
[5831]	ACTG1	<---	PRKCG	-0.43362	0.33453	+
[5832]	DVL3	<---	PSMD4	-0.60718	0.45961	+
[5833]	ACTBL2	<---	MMP13	-0.29263	0.085904	+
[5834]	COL2A1	--->	DAND5	-0.0062022	-0.043827	-
[5835]	IL1B	<---	MAPK9	-0.078488	0.15109	+
[5836]	PABPC1	<---	PPP2CA	-0.41281	0.63012	+
[5837]	ASH2L	<---	FOXA1	-0.30912	0.30288	+
[5838]	GNAL	--->	OR2C3	0.49109	-0.4306	-
[5839]	GFRA1	--->	HRAS	0.11501	-0.097858	+
[5840]	IRAK1	--->	MAP2K6	0.73444	-0.20293	+
[5841]	ARPC1A	--->	MEF2A	0.088232	-0.21232	-
[5842]	CEBPB	--->	ZNF69	0.28002	-0.15205	+
[5843]	BRCA1	--->	FUBP1	0.30904	-0.32545	+
[5844]	ACVR1C	<---	INHBB	-0.33406	0.27387	+
[5845]	SHC3	<---	SPRY4	-0.15769	0.090914	-
[5846]	IL19	--->	IL6R	0.12377	-0.18387	+
[5847]	ELAVL2	<---	REST	0.008509	0.25992	+
[5848]	CDK5	<---	PLXNA3	-0.025182	0.40618	+
[5849]	MAPK7	--->	MEF2C	1.0903	-1.4506	+
[5850]	IL20RA	<---	SOCS1	-0.40298	0.060066	+

[5851]	GRK7	--->	MTNR1B	0.33635	-0.28786	+
[5852]	HDAC2	<---	UBTF	-0.31093	0.47627	-
[5853]	PPP2R1A	--->	WWTR1	0.19485	-0.34161	+
[5854]	KLF1	--->	TCF12	0.33079	-0.20142	-
[5855]	F2	--->	F9	0.033116	-0.046014	+
[5856]	PRKCZ	<---	RHOQ	-1.529	1.3756	+
[5857]	MAP3K11	--->	RAC1	0.44037	-0.35521	-
[5858]	DCAF6	<---	ETS1	-0.060578	0.059668	+
[5859]	GRB2	--->	RAC1	0.63892	0.0030067	+
[5860]	DVL1	<---	FZD10	-0.33521	0.24353	+
[5861]	SAG	--->	WNT7A	0.23514	-0.34616	-
[5862]	COL14A1	<---	MMP9	-0.064017	-0.031269	+
[5863]	FARP2	<---	SRC	-0.20014	0.13659	+
[5864]	BUB1B	--->	BUB3	0.54982	-0.31609	-
[5865]	SIRT2	--->	SIX5	0.15908	-0.24396	-
[5866]	MAPK8	<---	RET	-0.8008	0.67629	+
[5867]	DAXX	<---	MDM2	-0.27344	0.10548	-
[5868]	AREG	<---	RARA	-0.34725	0.18042	+
[5869]	EP300	<---	SEMA7A	-0.010774	0.0098608	-
[5870]	GNA15	--->	GRM1	0.24033	-0.15623	-
[5871]	EIF2AK1	<--->	MAPK11	0.31014	0.31014	-
[5872]	CCL3L1	<---	CCR6	-0.35895	0.21658	-
[5873]	ITGA2	<---	PIK3R2	-0.073728	-0.0143	-
[5874]	EP300	<---	RPS6KB1	-0.016182	0.0029102	-
[5875]	EIF2AK3	<---	PPP1CC	-0.12622	0.14153	+
[5876]	GNAI3	<---	KCNJ3	-0.011498	0.05262	-
[5877]	MYC	<---	SMAD2	-0.18402	0.015448	-
[5878]	CD86	<---	SPI1	-0.097183	0.070359	+
[5879]	GNA14	--->	WNT10A	0.42087	-0.47341	-
[5880]	CTCF	--->	HOXC5	0.065182	0.0092332	+
[5881]	SMAD3	<---	YAP1	-1.0153	1.0527	+
[5882]	EGF	<---	SPRY1	-0.13445	0.1443	-
[5883]	GCK	--->	STAT5A	0.26825	-0.19721	-
[5884]	JUP	--->	TCF7L2	1.3086	-0.63623	+
[5885]	FOS	<---	TRIB1	-0.57905	1.0858	-
[5886]	EPAS1	<---	UBE2D3	0.039792	0.092879	-
[5887]	GH2	--->	IL20RA	0.20113	-0.087402	+
[5888]	EGFR	--->	FGF10	0.25979	-0.22928	-
[5889]	PRLR	--->	STAT5A	0.23476	-0.17484	-
[5890]	SREBF1	<---	ZNF718	-0.20142	0.33079	-
[5891]	NGFR	--->	SHC3	0.12767	-0.32299	+
[5892]	GRB2	--->	IGKV1-5	0.13256	-0.17782	-
[5893]	F11	--->	SERPIND1	0.33364	-0.53505	-
[5894]	FYN	<---	PDGFRB	-0.15501	0.75705	+
[5895]	PPP1R13L	--->	TP53	0.10329	-0.11137	+
[5896]	CTCF	--->	ZBTB32	-0.0051278	-0.028383	+
[5897]	FURIN	--->	NODAL	0.10685	-0.22982	+
[5898]	GNAI3	<---	RGS10	-1.2531	1.2063	+
[5899]	ADIPOR1	--->	PRKAG2	-0.018145	-0.15746	+
[5900]	PRKCA	--->	SRC	0.23398	-0.027179	+
[5901]	KCNQ3	<---	PRKG2	-0.76808	0.73036	-
[5902]	PIK3CG	<---	TXK	-0.10504	0.14175	-
[5903]	FCER1A	--->	SPHK1	0.25603	-0.27286	+
[5904]	CCR6	--->	GNAI1	1.1631	-1.1303	+
[5905]	HSPB2	<---	MAPK12	-0.26091	0.40675	+
[5906]	FOXO1	--->	G6PC2	0.4988	-0.56315	+
[5907]	CTNNB1	<---	IQGAP1	-0.11071	0.088702	+
[5908]	EPHB3	--->	PSEN2	-0.025348	-0.042014	-
[5909]	FYN	--->	GRIN2B	0.56795	-0.41064	+
[5910]	CCNE1	--->	NTRK1	-0.064542	-0.074428	+
[5911]	AGRN	<---	HS3ST6	-0.072715	0.04628	+
[5912]	FASLG	<---	FOXO1	-1.4944	1.4539	+
[5913]	HDAC2	<---	RIF1	-0.28027	0.33535	-
[5914]	CSF3R	<---	JAK2	-0.14471	0.060656	-
[5915]	CCM2	<---	MAPK14	-0.25084	0.44817	-
[5916]	NTRK1	<---	SPRY3	-0.37241	0.11479	+
[5917]	FGFR2	<---	VEGFC	-0.38721	0.2003	+
[5918]	GNAI1	<---	RGS7	-0.736	0.71839	+
[5919]	RELA	--->	STAT4	0.49507	-0.41746	-
[5920]	BIRC2	<---	TCF7L2	-0.61109	0.8289	+
[5921]	ITGA1	<---	MAPK8	-0.13121	0.054343	+
[5922]	ITGA4	--->	THBS4	0.046345	-0.039839	-
[5923]	E2F4	--->	PLK4	0.2562	-0.027732	+
[5924]	KRAS	--->	MAP3K1	0.85242	-0.72759	+
[5925]	SFN	--->	TBC1D4	0.21759	0.06516	-
[5926]	MYC	<---	TEAD4	-0.41015	0.20353	+

[5927]	PRKX	--->	TRPV5	0.54889	-0.41958	+
[5928]	CCNA1	<---	E2F1	-0.39339	0.45542	-
[5929]	KIDINS220	--->	NTRK1	0.044054	-0.23575	-
[5930]	CCL4	<---	NFKB1	-1.2272	1.0745	+
[5931]	CCNG1	<---	MDM2	-0.32193	0.22764	-
[5932]	ACTN2	<---	CAMK2A	-0.14999	0.15958	-
[5933]	NFATC2	--->	TRPC6	0.21608	-0.35616	+
[5934]	ERN2	<---	TRIM2	0.0068313	0.044647	-
[5935]	GNAO1	--->	PLA2G4F	-0.10668	-0.13723	+
[5936]	CREB1	<---	REN	-0.060871	0.092699	-
[5937]	SP3	--->	TNC	0.013217	-0.012664	+
[5938]	FCER1G	<---	PIK3R2	-0.041485	-0.036893	-
[5939]	CTNNB1	<---	DCHS1	-0.19204	0.11503	-
[5940]	BCR	<---	SRF	-0.12751	0.064166	+
[5941]	PLEKHG5	--->	RHOT1	0.33144	-0.31781	+
[5942]	ARHGAP15	<---	RHOT1	-0.52838	0.35211	-
[5943]	GNB1	<---	PRKAR1A	-0.29212	0.25254	+
[5944]	FZD2	<---	TCF7L2	-0.12979	0.22285	+
[5945]	CEBPA	--->	FGA	-0.021717	-0.10041	+
[5946]	HIRA	--->	MXI1	0.38477	-0.38125	-
[5947]	COL4A6	--->	ITGA8	-0.013036	-0.10803	+
[5948]	RBL1	<---	SMAD3	-0.16383	0.070647	+
[5949]	CHUK	<---	ZBP1	-0.15021	0.19508	+
[5950]	ACTA2	<---	HTT	-0.72444	0.64593	+
[5951]	FZD2	<---	GNB2	-0.19257	0.19185	-
[5952]	ITPR1	<---	STAT3	0.0052535	0.053883	+
[5953]	HBEGF	<---	MMP9	-0.20414	0.010381	+
[5954]	CTGF	--->	JUN	0.034809	-0.056716	-
[5955]	GRK7	--->	SSTR1	0.33635	-0.28786	+
[5956]	EGF	<---	FGFR2	-0.034049	0.014755	-
[5957]	ADRBK1	--->	HTR1D	0.25327	-0.21301	+
[5958]	EPHA2	--->	FGF11	-0.0069714	-0.050497	-
[5959]	CRKL	<---	NGFR	-0.061648	-0.042731	+
[5960]	LIMK1	--->	PAK6	0.52276	-0.38894	-
[5961]	ERF	<---	NR3C1	-0.14879	-0.015388	+
[5962]	ADCY1	<---	PRKACA	-0.63235	0.8673	-
[5963]	PIK3R2	<---	RHOG	-0.33729	0.22452	-
[5964]	CAMK2B	<---	NRAS	-0.038527	0.42208	-
[5965]	CHRM1	<---	PRKCB	-0.37622	0.27696	-
[5966]	FGFR3	<---	PDGFD	-0.076002	0.030896	+
[5967]	FER	<---	PTPN1	-1.0089	0.61386	-
[5968]	CDKN1A	--->	TP53	0.14326	-0.065967	-
[5969]	GH1	--->	GHR	0.073187	-0.17721	+
[5970]	IKBKB	<---	PRKCQ	-0.6951	0.07681	+
[5971]	IKBKB	--->	IRS2	0.33127	-0.0041474	+
[5972]	BMP4	--->	BMPR2	1.3161	-0.85941	+
[5973]	KCNJ6	<---	OPRK1	-0.2522	0.11779	-
[5974]	INSR	<---	PRKCZ	-0.082736	0.15778	-
[5975]	AKT2	--->	PIK3CG	0.028477	-0.051515	-
[5976]	CDH2	<---	ROBO1	-0.14331	-0.088364	+
[5977]	FOXF1	--->	GATA2	0.32839	-0.37846	-
[5978]	ACTB	<---	PRKCD	-0.37374	0.26975	+
[5979]	CD14	<---	IRAK3	-0.43727	0.56911	+
[5980]	DUSP2	--->	MAP3K4	1.353	-1.036	+
[5981]	MAPK11	<---	RALA	-0.20304	1.2491	+
[5982]	AKT1	--->	BCL2	0.30677	-0.44298	+
[5983]	BHLHE40	<---	ZBTB7A	-0.23178	0.2196	+
[5984]	PDPK1	--->	PKN1	0.6105	-0.69991	+
[5985]	EGF	<---	UBE2D3	-0.1934	0.19772	-
[5986]	PRKD3	<---	RASA1	0.047727	0.12381	+
[5987]	GCK	--->	NUP37	0.059668	-0.060578	-
[5988]	CXCR1	--->	PF4V1	0.21658	-0.35895	-
[5989]	MYL7	--->	PPP1R12B	0.48495	-0.4442	-
[5990]	GNA15	<---	LTB4R2	-1.1303	1.1631	+
[5991]	EDNRA	<---	GHRL	-0.75338	0.61472	+
[5992]	FN1	<---	RELA	-0.71218	0.35097	+
[5993]	PIK3R1	<---	TRAF2	-0.1563	0.11988	+
[5994]	GNA11	<---	RGS19	-1.2531	1.2063	+
[5995]	DLC1	<---	RHOBTB2	-0.30675	0.061607	-
[5996]	FOS	<---	FOXA2	-0.49625	0.62806	-
[5997]	ARHGEF1	<---	RAC2	-0.0089688	0.02783	-
[5998]	CD80	<---	FYN	-0.2937	0.39084	+
[5999]	NGF	<---	PCSK5	-0.20029	0.16968	+
[6000]	CTCF	<---	NR2C1	-0.051394	0.053678	-
[6001]	ACAN	<---	MMP1	-0.070078	0.0083701	+
[6002]	BIRC2	--->	CASP1	0.072187	-0.29878	-

[6003]	GAB1	<---	LPAR3	-0.45847	0.44741	+
[6004]	COL4A3	--->	ITGAV	-0.013036	-0.10803	+
[6005]	CBLC	--->	IL2RB	0.4878	-0.21009	+
[6006]	BCAR1	--->	ITGB5	0.069768	-0.069539	-
[6007]	PAK4	<---	RAC2	-1.1901	0.96651	+
[6008]	CX3CR1	<---	GRK5	-0.28786	0.33635	+
[6009]	COL1A2	--->	ITGA9	-0.013036	-0.10803	+
[6010]	ACTB	<---	WASL	-0.22029	0.18415	+
[6011]	LPAR1	<---	NFKB1	-0.43941	0.47827	-
[6012]	PIK3CD	<---	PLCG1	-0.0055153	0.0039515	-
[6013]	GRM1	<---	KCNJ5	-0.25173	0.20895	+
[6014]	GNAQ	<---	GNRHR	-1.1303	1.1631	+
[6015]	DLG1	<---	NCALD	-0.053695	0.15323	+
[6016]	IL10RB	--->	TYK2	0.48376	-0.37166	+
[6017]	ATP2B4	<---	PRKG1	-0.1935	0.11836	+
[6018]	PRICKLE1	--->	PSMD14	0.31126	-0.25332	-
[6019]	APP	<---	MAPK9	-0.023982	0.030823	-
[6020]	PLCG1	--->	RGS4	0.026624	-0.0038693	-
[6021]	CCL11	<---	CXCR6	-0.35895	0.21658	-
[6022]	GAB1	<---	HRAS	-0.3085	0.35789	-
[6023]	ARAP1	<---	RHOH	-0.48615	0.36733	-
[6024]	IKBKG	<---	MAP2K6	-0.45312	0.62218	-
[6025]	CCR7	<---	GRK7	-0.28786	0.33635	+
[6026]	CTNNB1	<---	MLLT4	-0.12776	0.52662	+
[6027]	IL22RA1	<---	IL26	-0.087402	0.20113	+
[6028]	SRC	--->	TNC	0.25438	-0.21474	-
[6029]	HRAS	<---	RASA4	-0.66794	0.71076	+
[6030]	KALRN	<---	RHOD	-0.021179	0.042607	-
[6031]	LYN	--->	MAPK1	0.92013	-1.0925	+
[6032]	BMPR1A	--->	SMAD4	0.7946	-0.60139	+
[6033]	FCGR1A	<---	FYN	-0.94853	1.1589	+
[6034]	MAP2K6	<---	MAP3K2	-1.347	1.3429	+
[6035]	CAV1	<---	TRADD	-0.30009	0.14194	-
[6036]	ESR1	--->	JUNB	0.10351	-0.20904	+
[6037]	EP300	--->	HIST2H2BE	0.01343	-0.01897	+
[6038]	MAPK12	--->	TP73	0.096521	-0.076704	+
[6039]	AKAP13	--->	CDKN1B	0.030495	-0.028881	-
[6040]	RAC3	<--->	RHOA	0.1628	0.1628	-
[6041]	ITGAV	<---	PTK2	-0.076638	-0.038051	-
[6042]	GRK6	--->	HTR1B	0.33635	-0.28786	+
[6043]	BIRC3	<---	TICAM2	-0.12724	0.095174	-
[6044]	ITGB5	--->	MYL6B	0.063371	-0.085492	-
[6045]	FZD2	<---	WNT2	-0.78209	0.63734	+
[6046]	SEMA3A	<---	STAT1	-0.31741	0.25816	+
[6047]	PRKCG	--->	RRAS2	0.85615	-0.68511	+
[6048]	BRD2	<---	SMARCA4	-0.033126	-0.00089051	+
[6049]	CCL3L1	<---	CCR8	-0.35895	0.21658	-
[6050]	KRAS	--->	MAPK3	1.2491	-0.20304	-
[6051]	ILK	<---	RXRA	-0.070039	-0.056828	+
[6052]	CALM1	--->	IFNGR1	0.15757	-0.50549	-
[6053]	EP300	<---	FOXO1	-0.016032	0.013464	-
[6054]	ESR2	--->	PPARG	-0.0075988	-0.021251	+
[6055]	CSNK1A1	<---	PPP6C	-0.12622	0.14153	-
[6056]	IL10RA	<---	SOCS5	-1.0456	0.57217	+
[6057]	JAK1	--->	MAPK8	0.43202	-1.1344	+
[6058]	CYSLTR2	--->	GNA11	1.1631	-1.1303	+
[6059]	SHC1	<---	TEK	-0.16364	0.077594	+
[6060]	MAPK3	--->	RPS6KA1	0.9519	-1.129	+
[6061]	ITGB7	--->	TNC	0.031276	-0.080829	-
[6062]	CACNA1S	<---	PRKG1	-0.68145	0.50273	+
[6063]	CXCR1	<---	GRK4	-0.28786	0.33635	+
[6064]	ABL1	--->	STAT5A	0.17147	-0.39405	+
[6065]	RAB5A	<--->	RAB7A	0.1628	0.1628	-
[6066]	NKD1	<---	TEAD2	-0.94584	0.87227	+
[6067]	GNAT3	<---	RGS4	-1.2531	1.2063	+
[6068]	SLC22A4	<---	SP1	-0.2032	0.35054	+
[6069]	ACTG2	<---	MYL6B	-0.10988	0.043745	+
[6070]	LCK	--->	MAPK11	0.92013	-1.0925	+
[6071]	CCL27	<---	CXCR1	-0.35895	0.21658	-
[6072]	MAP3K1	--->	SMAD2	0.78509	-0.82284	+
[6073]	BAD	<---	PRKX	-0.56837	0.82969	+
[6074]	FCER1A	--->	FER	0.66819	-0.86776	+
[6075]	FOXA2	--->	GLI1	0.45758	-0.30022	-
[6076]	OCRL	<---	RHOD	-0.92575	0.61511	-
[6077]	EP300	--->	ZMYND8	-0.0015178	-0.0036136	+
[6078]	CD79B	--->	INPP5D	0.047691	-0.118	+

[6079]	ITGA2	--->	THBS1	0.092882	-0.042129	-
[6080]	JUN	<---	TCF7L2	-0.51678	0.699	+
[6081]	FOSL1	--->	KLF1	0.16224	0.14761	+
[6082]	DNM2	<---	PIK3CG	-0.010232	0.0072856	+
[6083]	CDK8	<---	TNF	0.048942	0.10494	+
[6084]	ACP1	--->	CTNNB1	0.55429	-0.67589	+
[6085]	CCL14	<---	CCR1	-0.35895	0.21658	-
[6086]	IL12RB1	<---	LEP	-0.29036	0.12248	+
[6087]	BTK	<---	IRAK3	-0.067152	0.12443	+
[6088]	EP300	--->	MYOG	0.024715	-0.02899	+
[6089]	PPP2CB	<---	PTPN11	-0.13872	0.28656	+
[6090]	JAK2	--->	PRKACB	0.43202	-1.1344	-
[6091]	CCL26	<---	CXCR6	-0.35895	0.21658	-
[6092]	NCK1	--->	TRIO	0.060041	0.010238	+
[6093]	IL1B	<---	MAPK3	-0.078488	0.15109	+
[6094]	MDM2	--->	RASSF1	0.060668	0.010864	-
[6095]	CTTN	<---	SRC	-0.21339	0.32492	+
[6096]	CCL2	<---	SMAD4	-0.11489	-0.043113	+
[6097]	CRKL	--->	ITGB6	0.030916	-0.04526	-
[6098]	ARHGAP25	<---	RHOU	-1.2097	0.92798	-
[6099]	CCND1	<---	TCF7L2	-0.21775	0.37715	+
[6100]	JUND	<---	SFRP1	-0.33818	0.36068	-
[6101]	SIX5	--->	ZNF605	0.11044	0.025818	+
[6102]	C1S	<---	C4A	-0.28897	0.23739	-
[6103]	PTEN	--->	TP53	-0.0077509	-0.0094231	-
[6104]	EP300	--->	TNFRSF9	0.012375	-0.020768	+
[6105]	OSMR	<---	TYK2	-0.37792	0.17946	-
[6106]	GRK1	--->	HTR1E	0.33635	-0.28786	+
[6107]	ROCK1	--->	SH3GL2	0.068327	0.046815	+
[6108]	FCGR1A	<---	FGR	-0.94853	1.1589	+
[6109]	CRK	--->	ITGAL	0.018691	-0.085251	-
[6110]	LPAR4	--->	PIK3CG	0.029282	-0.062766	+
[6111]	IGFBP3	--->	MMP1	-0.0056809	-0.18543	-
[6112]	BAD	<---	MAPK1	-0.56837	0.82969	+
[6113]	ELMO1	<---	RAC3	-0.038582	0.076038	-
[6114]	BCL3	<---	DDIT3	-0.18789	0.205	-
[6115]	ARNT	<---	PFKL	0.0023816	0.16054	-
[6116]	NFATC2	--->	RAN	0.18991	-0.34579	-
[6117]	CHEK2	--->	MDC1	0.075161	-0.073814	-
[6118]	CTNNB1	<---	PRKACB	-1.3965	1.0802	+
[6119]	HDAC1	<---	UBTF	-0.31093	0.47627	-
[6120]	ILK	--->	MYL9	0.16146	-0.1945	+
[6121]	FGF17	--->	IGF1R	0.25346	-0.0044024	+
[6122]	PPFIA4	--->	RAB3A	0.39732	-0.4667	-
[6123]	COL3A1	--->	ITGA1	0.00018599	-0.07158	+
[6124]	CHUK	--->	REL	0.51654	-0.39935	+
[6125]	FZD10	<---	GNB2	-0.19257	0.19185	-
[6126]	CSNK1A1	--->	YWHAZ	0.079981	-0.071802	+
[6127]	PTK2	<---	RAC1	-0.14524	-0.084837	-
[6128]	C4BPB	--->	NFIC	0.095472	-0.0055258	-
[6129]	CXCL13	<---	XCR1	-0.35895	0.21658	-
[6130]	CTNNB1	<---	SNX1	-0.44924	0.34206	+
[6131]	GLI3	--->	PSMD10	0.37436	-0.001863	-
[6132]	FGF4	--->	FGFR2	0.40218	-0.67149	+
[6133]	PRKCZ	--->	STAT3	0.53838	-0.49624	-
[6134]	KRAS	<---	RASGRP1	-0.17492	0.5048	+
[6135]	AKT1	--->	CSF2RA	0.17677	-0.26763	-
[6136]	MYL12A	--->	RAC1	0.20073	-0.14858	-
[6137]	FRS2	--->	SPRY2	0.23114	-0.13172	+
[6138]	CREBBP	<---	PIAS4	-0.0030573	7.1807e-005	+
[6139]	MAPK13	<--->	PRKACB	0.31014	0.31014	-
[6140]	FN1	<---	TLN1	-0.018909	0.0087354	-
[6141]	DKK2	--->	WNT10A	0.54164	-0.69755	+
[6142]	CTBP2	--->	HAND2	0.21332	-0.33732	+
[6143]	NTF4	--->	NTRK2	0.25731	-0.6758	+
[6144]	PLA2G6	<---	PRKCE	-0.21133	0.19522	+
[6145]	STAT1	--->	TNFSF11	0.25506	-0.31754	+
[6146]	ATP1A3	<---	PRKACG	-0.09775	0.14787	+
[6147]	ITGA9	<---	LAMC3	-0.041507	-0.0065623	+
[6148]	PIK3CG	<---	PRKCB	-0.10023	0.083943	-
[6149]	BIRC2	<---	TICAM1	-0.10671	0.09116	-
[6150]	CBX5	<---	TCF7L2	0.059308	0.086299	+
[6151]	RDX	--->	ROCK2	0.036127	-0.038345	-
[6152]	FYN	--->	GRAP2	0.41943	0.028419	+
[6153]	DLK1	<---	ZBTB7A	-0.022471	0.066314	+
[6154]	PPP2CB	<---	PRKCB	-0.12382	0.10585	-

[6155]	ATF2	<---	RPS6KA4	-0.7127	0.90464	+
[6156]	CTCFL	<---	ZNF280A	-0.039841	-0.037992	-
[6157]	BATF	--->	IKZF2	0.16224	0.14761	+
[6158]	SNAI2	<---	SP1	-0.039841	-0.037992	-
[6159]	IL2RG	<---	STAT1	-0.12983	0.10573	-
[6160]	GNB2	--->	WNT2	0.3343	-0.42584	-
[6161]	ADRA2A	--->	GNAQ	1.1631	-1.1303	+
[6162]	SP110	--->	STAT2	0.44408	-0.32334	-
[6163]	H3F3A	<---	SIRT1	-0.32305	0.21329	+
[6164]	FOXA2	--->	TTC3	0.32734	-0.57793	+
[6165]	COL5A2	--->	ITGA6	0.0052169	-0.090529	+
[6166]	DRD4	<---	GRK7	-0.28786	0.33635	+
[6167]	CTNNB1	<---	PCDH8	-0.19204	0.11503	-
[6168]	GH1	--->	IL7R	0.12248	-0.29036	+
[6169]	SOST	--->	WNT3A	0.41775	-0.59125	+
[6170]	CCND2	<---	CDKN2C	-0.066649	0.24757	+
[6171]	IL6	<---	MYD88	-0.50188	0.41626	+
[6172]	PRKCA	--->	RAC1	0.85615	-0.68511	+
[6173]	CSF3R	<---	IL23A	-0.10858	0.043291	+
[6174]	ITGA2B	--->	THBS3	-0.014728	-0.021497	-
[6175]	CDK8	--->	SMAD2	0.88243	-0.89987	+
[6176]	CD3D	<---	LCK	-0.45391	0.63648	-
[6177]	FZD8	<---	WNT7A	-0.78209	0.63734	+
[6178]	EHF	--->	TCF12	0.26575	-0.31912	-
[6179]	RAC3	<---	WASF2	-0.076889	0.12856	-
[6180]	ELK4	<---	MAPK3	-0.26091	0.40675	+
[6181]	COL11A1	--->	ITGA6	-0.013036	-0.10803	+
[6182]	GNAO1	--->	SCN1A	0.16571	-0.1017	+
[6183]	ACTA1	<---	MMP19	-0.29263	0.085904	+
[6184]	COL6A3	--->	ITGA2B	-0.0067115	-0.019363	+
[6185]	PLD2	<---	PRKCG	-0.11393	0.14533	+
[6186]	FCGR1A	<---	YES1	-0.94853	1.1589	+
[6187]	GNAI2	<--->	GNAT1	0.064764	0.064764	-
[6188]	ACTB	<---	PRKCQ	-0.37374	0.26975	+
[6189]	CREB5	<---	INS	-0.24765	0.36121	-
[6190]	MAPK14	--->	S1PR2	0.19743	-0.10024	-
[6191]	FGF4	--->	FGFR4	0.40218	-0.67149	+
[6192]	MAPK8	<---	TRAF2	-0.60861	1.3226	+
[6193]	ACAN	<---	MMP7	-0.070078	0.0083701	+
[6194]	IKBKG	<---	PRKCZ	-0.10817	0.035853	+
[6195]	EGFR	--->	SPRY1	0.27575	-0.22962	+
[6196]	ADRBK1	--->	PRKCG	0.18696	-0.025631	-
[6197]	NTRK1	<---	THPO	-0.42094	0.11634	+
[6198]	GRK5	--->	HTR1A	0.33635	-0.28786	+
[6199]	ARHGAP22	<---	CDC42	-0.82056	0.64419	-
[6200]	CD83	<---	DAND5	-0.097561	0.04605	+
[6201]	ARHGAP29	<---	RHOJ	-1.011	0.68428	-
[6202]	CBLC	--->	PRLR	0.30049	-0.14845	+
[6203]	GATA2	<---	TLR9	-0.60012	0.32045	-
[6204]	IRAK3	<---	IRAK4	-1.1581	0.35663	-
[6205]	BUB1B	--->	PSMD14	0.2441	-0.083683	-
[6206]	NRK	--->	TRADD	0.24439	-0.21179	-
[6207]	IRS1	<---	PIK3R2	-0.096507	-0.04328	-
[6208]	MLLT4	<---	RRAS	-1.0278	0.068833	+
[6209]	GFRA1	<---	PRKACA	-0.030888	0.17118	+
[6210]	CCL17	<---	CCR6	-0.35895	0.21658	-
[6211]	LRP6	<---	WNT11	-0.28769	0.39899	+
[6212]	MAPK12	<--->	MAPKAPK3	0.31014	0.31014	-
[6213]	CREB1	<---	PCSK1	-0.060871	0.092699	-
[6214]	BAX	<---	CAPN2	-0.22344	0.11805	-
[6215]	GRIN2D	<---	PRKACB	-1.006	1.5014	+
[6216]	GLI1	--->	WNT10A	0.86373	-1.47	+
[6217]	COL6A1	--->	ITGA4	-0.0092525	-0.030111	+
[6218]	GNB5	<---	PRKACA	-0.31609	0.54982	+
[6219]	GRB2	--->	IGHD	0.17309	-0.31712	-
[6220]	GLI2	<---	STK36	-0.57298	0.8559	+
[6221]	CAMK2G	--->	FLNB	0.30815	-0.21817	+
[6222]	MAP2K3	--->	TAOK3	1.1648	-0.18663	-
[6223]	FOXO3	<---	SGK2	-0.40959	0.15123	+
[6224]	MYC	<---	SRSF7	-0.13606	-0.02655	-
[6225]	MAX	<---	SFRP1	-0.3976	0.42436	-
[6226]	PTK2	<---	PTPN13	-0.13482	0.011372	-
[6227]	BTK	--->	RAC1	0.42475	0.11361	+
[6228]	IL12B	<---	MAPK8	-0.50839	0.57211	+
[6229]	LPAR2	--->	PIK3R5	0.56062	-0.77034	+
[6230]	C2CD5	--->	MYO5A	0.023947	-0.070792	+

[6231] AKT1 ---> RELN 0.16613 -0.26458 -
[6232] AURKA ---> PSMD1 0.57437 -0.5963 -
[6233] FN1 <--- NFIC -0.29227 0.092312 +
[6234] COL18A1 ---> MMP9 0.003315 -0.13237 +
[6235] CSF2 ---> IL11RA 0.17123 -0.17539 +
[6236] INPP5B <--- RHOD -0.92575 0.61511 -
[6237] EHF ---> MEF2A 0.072716 -0.18251 -
[6238] AKT1 ---> MST1 0.11974 -0.20473 +
[6239] ATP2B4 <--- PRKG2 -0.1361 0.12011 +
[6240] GNB1 <--- PTK2 -0.085695 -0.034953 -
[6241] GMIP <--- RHOU -1.011 0.68428 -
[6242] LIMK1 ---> RHOC 0.41565 -0.24298 -
[6243] GNB1 <--- PRKCG -0.27923 0.22728 +
[6244] CTCF ---> ZNF746 -0.0037425 -0.012548 +
[6245] CCR2 ---> CXCL12 0.21658 -0.35895 -
[6246] MEF2C <--- TLR1 -0.3634 0.073374 -
[6247] HNRNPD <--- YY1 0.008509 0.25992 +
[6248] ITGA2 <--- PIK3CB -0.024164 -0.004208 -
[6249] CASP3 <--- HTT -0.30768 0.080675 +
[6250] EP300 ---> ZNF483 0.016752 -0.010456 +
[6251] WIF1 ---> WNT8B 0.49247 -0.8346 +
[6252] IL2RG <--- TAF1 -0.11334 0.056231 +
[6253] BLK ---> IGHD 0.83863 -0.55361 +
[6254] FGF10 ---> KDR 0.21774 -0.0015767 +
[6255] DKK4 ---> FZD3 0.3176 -0.35737 +
[6256] COL9A1 <--- LEPRE1 -0.18291 0.065305 +
[6257] PSMD14 ---> SUFU 0.416 -0.45428 +
[6258] F2R ---> PRSS3 0.11779 -0.2522 -
[6259] ITPR1 <--- STIM1 -0.28344 0.46398 -
[6260] GNAQ <--- LPAR4 -1.1303 1.1631 +
[6261] IL1A <--- MAPK1 -0.078488 0.15109 +
[6262] SPTA1 <--- SRC 0.01761 0.30667 +
[6263] ARR3 <--- CSNK1G2 -0.09775 0.14787 -
[6264] ARHGAP11A <--- RHOU -1.5245 1.016 -
[6265] HDAC3 <--- TNFRSF1A -0.50088 0.30959 +
[6266] GRIN2C <--- RAC2 -0.38938 0.54247 -
[6267] MYH3 ---> MYL1 0.063371 -0.085492 -
[6268] ADORA2B <--- GRK7 -0.28786 0.33635 +
[6269] FGF10 <--- MAPK3 -0.47959 0.71493 +
[6270] ARHGAP11B <--- RAC2 -1.5245 1.016 -
[6271] RRAS <--- SRC 0.16613 1.0122 +
[6272] KRAS <--- LCK 0.16613 1.0122 +
[6273] KCNJ3 <--- OPRL1 -0.2522 0.11779 -
[6274] BAZ2A ---> HIST2H3A 0.12827 -0.19704 +
[6275] EGR1 ---> TP53 0.1064 -0.015277 -
[6276] JAK2 ---> NFKBIA 0.13473 -0.30187 +
[6277] GNB5 <--- HTR1E -0.92616 1.1575 +
[6278] MTOR ---> RPS6KB2 1.3242 -0.84867 +
[6279] DUSP4 ---> EGR1 0.71798 -0.52013 -
[6280] AMH ---> BMPR1A 0.068408 -0.12754 +
[6281] ATF2 <--- PCK1 0.029124 0.05455 -
[6282] HS3ST3B1 ---> HSPG2 0.011685 -0.0011296 +
[6283] GNB5 <--- MAPK14 -0.31609 0.54982 -
[6284] CD274 ---> STAT3 0.26367 -0.19284 -
[6285] IRS1 <--- MET -0.23744 0.29011 +
[6286] GATA1 ---> RXRA -0.056454 -0.16004 -
[6287] PIK3R1 <--- RAF1 -0.17832 0.099119 +
[6288] IL12RB2 <--- IL23A -0.11334 0.056231 +
[6289] GNAI3 <--- PLCG1 -0.0052535 0.030885 -
[6290] EPO ---> IL12RB1 0.068084 -0.27035 +
[6291] NCOA1 <--- PPARA -0.054726 0.035483 -
[6292] IL18 <--- NFKB1 -0.22506 0.22491 -
[6293] EPHA1 <--- YES1 -0.26855 0.35424 +
[6294] CSF2RA ---> SOCS7 -0.014037 -0.24629 -
[6295] MAPK14 ---> STAT3 1.4162 -0.966 +
[6296] GRIN1 <--- RHOG -0.38938 0.54247 -
[6297] AKT2 <--- PPP2CB -0.049067 0.049194 -
[6298] PIK3CG <--- RAF1 -0.058386 0.011774 +
[6299] GPC6 ---> LRP1 0.24324 -0.20221 -
[6300] ADH6 <--- DAND5 -0.044865 0.021163 +
[6301] EPHB4 ---> GIT1 -0.0039653 -0.019814 +
[6302] NTRK2 ---> SHC2 -0.057473 -0.1181 +
[6303] GNAQ <--- LPAR5 -1.1303 1.1631 +
[6304] CSF2RA <--- STAT1 -0.31461 0.26984 -
[6305] ACTB <--- ARPC1A -0.37291 0.34929 +
[6306] PIK3CA <--- PLCG2 -0.0055153 0.0039515 -

[6307]	JUN	<---	TWF2	-0.22115	0.16075	-
[6308]	EPHB2	<---	LCK	-0.26855	0.35424	-
[6309]	RAC1	--->	STAT3	0.3922	-0.18366	+
[6310]	IL20	--->	IL22RA1	0.20113	-0.087402	+
[6311]	EXOC8	<---	RAB8A	-0.50681	0.59188	+
[6312]	DKK4	--->	WNT1	0.54164	-0.69755	+
[6313]	ATF4	<---	CAMK2G	-0.41113	0.72002	+
[6314]	CXCL9	<---	XCR1	-0.35895	0.21658	-
[6315]	EPHB1	--->	MMP9	-0.015392	-0.077231	-
[6316]	NTRK3	--->	SH2B1	0.0062143	-0.011688	+
[6317]	INSR	--->	IRS4	0.49489	0.047363	+
[6318]	CSNK2A2	--->	DVL2	0.52194	-0.62577	+
[6319]	ADCY9	<---	GNAI2	-0.63317	1.0384	-
[6320]	CAMK2B	--->	GRIA2	0.94748	-0.64695	-
[6321]	EGR1	<---	RANBP2	-0.22007	0.29364	-
[6322]	CDH15	<---	MAP2K6	-0.37971	0.32067	+
[6323]	CBL	--->	ERBB4	1.4559	-0.015521	+
[6324]	GAB2	<---	PRKD1	-0.1056	0.2913	-
[6325]	PIAS1	--->	STAT3	1.2392	-0.72187	+
[6326]	FGB	<---	TLN1	-0.018067	0.0095716	-
[6327]	CCND1	<---	CDK4	-0.55661	0.75243	+
[6328]	SOAT2	--->	SREBF1	0.43996	-0.52432	-
[6329]	CD8B	--->	IL2RB	-0.033108	-0.046475	+
[6330]	EDN1	<---	NFKB1	-0.23502	0.23585	+
[6331]	GPR183	--->	TCF12	0.60063	-0.73364	-
[6332]	ACTB	<---	PRKCE	-0.43362	0.33453	+
[6333]	PDCD1LG2	--->	TCRA	0.16771	-0.13441	+
[6334]	CDC42	--->	PARD6G	0.089672	0.018694	+
[6335]	PIK3CA	<---	RAP1B	-0.01026	0.011011	+
[6336]	CSF1R	<---	PTPN1	-0.55175	0.66899	+
[6337]	GNAL	<---	HTR6	-1.1303	1.1631	+
[6338]	GATA2	--->	SERPIND1	0.33364	-0.53505	+
[6339]	CNTF	--->	IL10RA	0.20113	-0.087402	+
[6340]	MAP2K4	<--->	MAPK14	0.31014	0.31014	-
[6341]	INPP5B	<---	RHOC	-0.92575	0.61511	-
[6342]	PORCN	--->	WNT6	0.38949	-0.56891	+
[6343]	FGFR3	--->	SOS1	0.060222	-0.017704	+
[6344]	PTPN1	--->	SPRY2	1.0769	-1.2039	-
[6345]	LCK	--->	PIK3CD	0.084876	-0.06481	+
[6346]	CD86	<---	PIK3CA	-0.013666	0.0055155	-
[6347]	CCNT1	<---	SMAD3	-0.18584	0.054214	-
[6348]	ARHGAP10	<---	RHOU	-0.65972	0.48549	-
[6349]	MAX	<---	SPI1	-0.31912	0.26575	-
[6350]	IL12RB1	<---	LCK	-0.55606	0.6862	-
[6351]	FGF16	<---	KRAS	-0.25726	0.38422	-
[6352]	ADAM17	--->	PSEN1	0.10001	-0.18529	+
[6353]	DUSP4	--->	MAPK8	1.353	-1.036	+
[6354]	ABL1	--->	EPHA1	0.06347	-0.16395	-
[6355]	ANK3	--->	HOXA9	0.1534	-0.22288	-
[6356]	EP300	<---	TRIM69	-0.0022202	0.010495	-
[6357]	PRKAG1	--->	TSC2	-0.061017	-0.063939	+
[6358]	RASGRP1	--->	SOS1	0.022248	0.020347	-
[6359]	GRIN3B	<---	PPP1CC	-0.71165	0.54635	+
[6360]	SFRP5	--->	WNT9A	1.0317	-1.1063	+
[6361]	PFKFB1	<---	PRKAA1	-0.15935	0.24561	+
[6362]	EGR3	<---	FOS	0.14761	0.16224	+
[6363]	CXCR5	--->	GNAI2	1.1631	-1.1303	+
[6364]	ACTR2	<---	RAC1	-0.51168	0.52339	+
[6365]	CD247	--->	VAV2	0.039241	-0.012505	+
[6366]	GNAO1	<---	RGS13	-1.2531	1.2063	+
[6367]	CNOT1	<---	EIF4A2	-0.16976	0.10767	-
[6368]	MYL6	<---	MYLK3	-0.29873	0.36004	+
[6369]	FN1	<---	PRKAR1A	-0.65543	0.45492	-
[6370]	IRS4	--->	PIK3CD	0.018176	-0.01402	+
[6371]	G6PC	<---	RXRA	-0.47696	0.062675	+
[6372]	EP300	--->	ZNF710	0.016752	-0.010456	+
[6373]	CCR8	--->	CXCL12	0.21658	-0.35895	-
[6374]	FOXA2	<---	STAT5B	0.11967	0.28002	-
[6375]	ITGA2B	<---	LAMA5	-0.041507	-0.0065623	+
[6376]	DLG4	--->	GRIA2	0.19945	-0.095793	-
[6377]	CARD9	<---	NFKB1	-0.32809	0.33175	-
[6378]	GNAI1	<---	RGS13	-1.2531	1.2063	+
[6379]	IRAK1	<---	MAP3K1	-0.42279	0.68564	-
[6380]	ARHGAP6	<---	RHOBTB1	-0.92575	0.61511	-
[6381]	CCL27	<---	CCR6	-0.35895	0.21658	-
[6382]	CD3G	--->	VAV3	0.06651	-0.018805	+

[6383]	DTX1	--->	PSEN2	-0.031869	-0.044399	-
[6384]	PIK3CA	--->	PREX1	0.012929	-0.015256	+
[6385]	ADIPOR1	--->	PRKAG1	-0.018145	-0.15746	+
[6386]	DUSP2	--->	MAPK14	1.353	-1.036	+
[6387]	CCR5	--->	CXCL14	0.21658	-0.35895	-
[6388]	FSHB	--->	INHBC	0.17799	-0.37709	-
[6389]	COL23A1	--->	PPIB	-0.024575	-0.068802	-
[6390]	MAPK8	<---	RASA1	-0.09092	0.19813	+
[6391]	RAN	<---	YWHAQ	-0.25073	0.15053	-
[6392]	CXCR4	<---	GRK1	-0.28786	0.33635	+
[6393]	COL10A1	<---	FYN	-0.27641	0.38494	-
[6394]	DRD2	--->	GNAI3	1.1631	-1.1303	+
[6395]	ITSN1	--->	RHOD	0.16745	-0.11028	+
[6396]	CDH15	<---	DAND5	-0.26522	0.096228	+
[6397]	RANBP2	--->	SRSF11	0.34767	-0.31854	+
[6398]	GNGT2	<---	HTR7	-0.26888	0.10335	+
[6399]	IRS1	<---	MAPK3	-0.20734	0.74413	-
[6400]	FCGR1A	--->	VAV3	0.18158	-0.03415	+
[6401]	HNRNPH3	<---	TAF1	-0.31439	0.36909	+
[6402]	COL11A1	--->	ITGA10	-0.013036	-0.10803	+
[6403]	GRK4	--->	HTR1A	0.33635	-0.28786	+
[6404]	MACC1	--->	TAL1	0.53211	-0.64108	-
[6405]	HDAC8	<---	TCF7L1	-0.12264	0.37175	-
[6406]	ATF4	<---	NPY	-0.81635	0.84205	-
[6407]	ETS1	--->	ITGA4	-0.0090065	-0.066621	+
[6408]	ARHGAP40	<---	RAC2	-1.5245	1.016	-
[6409]	KCNMB4	<---	PRKG2	-0.78834	0.62769	+
[6410]	EP300	<---	RHOH	0.00089005	0.00089974	-
[6411]	JUN	<---	TCF7	-0.51678	0.699	+
[6412]	DUSP21	--->	MAPK11	1.4184	-1.4202	+
[6413]	CACNA1F	<---	HTR2A	-0.36046	0.31836	+
[6414]	MAPK3	--->	RXRB	0.17534	-0.43654	+
[6415]	CREM	--->	PCK1	0.037806	0.031112	+
[6416]	MAPKAPK3	--->	SCMH1	0.40675	-0.26091	+
[6417]	ADRBK1	--->	HTR1E	0.25327	-0.21301	+
[6418]	ACTA2	<---	MMP13	-0.29263	0.085904	+
[6419]	MBTPS1	--->	SREBF2	0.43996	-0.52432	+
[6420]	ADCY8	<---	CAMK2G	-0.44346	0.58749	+
[6421]	MAP2K3	<---	TRAF6	-0.32382	0.68697	+
[6422]	AR	<---	ECM1	-0.13385	0.0074305	-
[6423]	STAT2	<---	UBE2L6	-0.35949	0.48292	-
[6424]	IRS2	<---	MAPK8	-0.20734	0.74413	+
[6425]	FZD10	<---	GNB4	-0.19257	0.19185	-
[6426]	HLA-DQA2	<---	PTPRC	-0.17668	0.081122	+
[6427]	PRKCB	--->	PRKD2	0.22455	-0.66161	+
[6428]	HOXC10	--->	TCF12	0.23888	-0.32236	-
[6429]	CD19	--->	CD79B	0.1696	-0.17227	-
[6430]	CDH5	<---	PAK3	-0.091123	0.25245	+
[6431]	CAPN3	<---	HTT	-0.25198	0.22116	-
[6432]	ATP1B4	<---	PRKACA	-0.26091	0.40675	+
[6433]	SLC9A3	--->	SP3	0.28002	-0.15205	-
[6434]	ADCY8	<---	CALML3	-0.63894	0.65698	+
[6435]	AR	<---	SMAD3	-0.10889	0.044828	+
[6436]	GNAI2	--->	GPSM1	-0.0080287	-0.042052	-
[6437]	GNAQ	<---	TBXA2R	-1.1303	1.1631	-
[6438]	BMP1	<---	PCOLCE	-0.47461	0.3932	+
[6439]	RHOD	<---	SOS1	-0.33772	0.48674	+
[6440]	EP300	--->	PFKL	0.0036818	-0.0061266	+
[6441]	ELF1	--->	TBP	0.24841	-0.29244	+
[6442]	CCKBR	--->	GNA15	1.1631	-1.1303	+
[6443]	CCL3	<---	EP300	-0.010336	0.0055944	+
[6444]	MAP4K3	--->	TRADD	0.24439	-0.21179	-
[6445]	BATF	<---	CIITA	-0.1955	0.3362	-
[6446]	ITGB4	<---	LAMA4	-0.041712	0.014159	+
[6447]	EP300	<---	PRKCB	-0.021229	0.012596	-
[6448]	CAMK2B	--->	GRM1	0.65286	-0.45637	+
[6449]	IL6R	<---	MAP2K1	-0.26408	0.28918	-
[6450]	HRAS	--->	RPS6KA2	0.39663	-0.23906	-
[6451]	FADD	--->	MAP3K1	0.054865	-0.31501	+
[6452]	PPARG	<---	PTGER2	-0.10397	-0.019436	-
[6453]	GNAO1	<--->	GNAT2	0.064764	0.064764	-
[6454]	ARHGEF11	--->	RHOF	0.092701	0.0073174	+
[6455]	HSPA9	--->	TLR6	-0.17832	-0.20601	+
[6456]	CISH	--->	STAT6	0.16522	-0.45416	+
[6457]	ADAM10	<---	CTNNA1	-0.19902	0.16595	-
[6458]	FGF19	--->	FGFR2	0.40218	-0.67149	+

[6459]	ITGAV	--->	SP3	0.010762	-0.053198	-
[6460]	IL13RA2	--->	STAT3	0.23411	-0.16189	-
[6461]	EGF	--->	INSR	0.1552	-0.058377	+
[6462]	CDK1	--->	ETS2	0.24561	-0.15935	-
[6463]	PDGFRB	--->	PIK3R5	0.078826	-0.053293	+
[6464]	AKT3	--->	NFKBIE	0.28123	-0.28841	+
[6465]	NFATC4	<---	PPP3CC	-0.50133	-0.28042	+
[6466]	ARRB2	--->	GNA14	0.030816	-0.0080738	-
[6467]	FLT4	--->	PIK3R1	0.27448	-0.14066	+
[6468]	IFNAR2	<---	IFNB1	-0.29094	0.42632	+
[6469]	CREBBP	--->	HK3	-0.00068954	-0.0041449	+
[6470]	CDC42	--->	EGR1	0.39894	-0.14775	-
[6471]	FYN	--->	PAK1	0.81303	-0.81947	+
[6472]	RAC2	<---	TRIP10	0.074898	0.19853	+
[6473]	RAP1A	<---	STAT1	-0.34544	0.34162	+
[6474]	NUP153	--->	SRSF9	0.60094	-0.54167	+
[6475]	IRAK4	<---	PPP2R1B	-0.069464	-0.068858	+
[6476]	CHD9	--->	NR1H4	0.014854	-0.17168	+
[6477]	MMP9	<---	NCF4	-0.124	0.06344	+
[6478]	IKBKB	<---	TIRAP	-0.42104	0.7066	+
[6479]	FOXO4	<---	MAPK14	-0.22085	0.2499	+
[6480]	ADAM17	--->	ZNF274	0.20758	-0.21293	+
[6481]	HSP90B1	<---	NFKB1	-0.057988	0.052963	+
[6482]	GH1	--->	IL20RB	0.20113	-0.087402	+
[6483]	FMR1	--->	USF1	0.43996	-0.52432	-
[6484]	GRIN1	<---	PRKACB	-1.006	1.5014	+
[6485]	FCGR2B	--->	TNFRSF11A	0.29671	-0.35844	-
[6486]	NR1D1	--->	PPARG	-0.0075988	-0.021251	-
[6487]	GRIN2B	<---	RHOQ	-0.38938	0.54247	-
[6488]	E2F1	<---	SMAD2	-0.30541	0.36653	-
[6489]	GRIN2C	<---	RHOG	-0.38938	0.54247	-
[6490]	CD22	<---	PTPN6	-0.097349	0.04352	-
[6491]	ADORA2B	--->	KCNJ5	0.11779	-0.2522	-
[6492]	CCND3	<---	CDKN1A	-1.0232	1.4682	+
[6493]	GRIN2B	<---	PPP1CA	-0.67094	0.67498	+
[6494]	FOXO4	--->	PLK4	1.0275	-0.62691	+
[6495]	F9	<---	HLF	-0.19345	0.1676	+
[6496]	ARF6	--->	CYTH3	0.19683	-0.33222	-
[6497]	E2F6	<---	FGFR3	-0.11324	0.074337	-
[6498]	BIRC3	<---	CFLAR	-0.20025	0.019553	-
[6499]	ASCL1	<---	SPI1	-0.52432	0.43996	+
[6500]	JUN	<---	LMO2	-0.45628	0.47419	-
[6501]	PPP4C	--->	YWHAB	-0.03225	-0.31369	-
[6502]	ADCY8	<---	GNAT3	-0.63317	1.0384	-
[6503]	PRKAG2	--->	SLC2A4	0.34513	-0.50137	+
[6504]	EP300	<---	RELA	-0.005516	0.0083594	-
[6505]	EGR1	<---	EHF	-0.093323	0.16874	-
[6506]	PLCE1	<---	RGS4	-0.044025	0.025988	+
[6507]	ARR3	--->	FZD8	0.13556	-0.17053	+
[6508]	IL12RB1	<---	PRL	-0.29036	0.12248	+
[6509]	RGMB	<---	TAB1	-0.45188	0.71032	-
[6510]	CRK	--->	GDNF	0.13475	-0.18141	-
[6511]	LYN	<---	PRKCD	0.097057	0.26845	-
[6512]	AREG	<---	EGR1	-0.047182	0.17079	+
[6513]	ADAM10	--->	DLL4	0.030486	-0.045674	+
[6514]	ADRBK1	--->	PRKCA	0.18696	-0.025631	-
[6515]	LTB	--->	LTBR	1.0868	-1.3016	+
[6516]	NFKB1	<---	RAF1	-0.025613	-0.012124	+
[6517]	GNB2	--->	RASGRF2	1.2179	-1.2056	+
[6518]	CACNA1F	<---	PPP2R1A	-0.39164	0.37653	+
[6519]	GAB2	<---	IL6ST	-0.029955	0.032714	+
[6520]	CTCF	<---	SOST	-0.017333	0.093603	-
[6521]	CNR1	--->	GNB4	1.1575	-0.92616	+
[6522]	MAP3K4	<---	RAC2	-0.20304	1.2491	+
[6523]	EGFR	--->	ITGB4	0.036496	-0.051888	+
[6524]	CD180	--->	TLR4	0.65283	-1.4467	+
[6525]	H2AFX	<---	UBE2N	-0.48509	0.38736	+
[6526]	ELK4	--->	TLE4	0.10019	-0.098063	+
[6527]	GNAO1	<---	PXN	-0.057204	0.12598	-
[6528]	BIRC5	--->	CASP9	0.2018	-0.4731	+
[6529]	CD79A	--->	SHC1	-0.003509	-0.082483	+
[6530]	BUB1B	--->	PLK1	0.29896	-0.098976	-
[6531]	MAPK13	--->	MEF2A	1.0903	-1.4506	+
[6532]	PGR	--->	VEGFA	-0.038004	-0.064123	+
[6533]	ARHGAP6	<---	RAC2	-1.5245	1.016	-
[6534]	ARHGEF16	<---	RHOBTB1	-0.041777	0.050621	-

```
[6535]  PTGS2    <---    RELA    -0.48141        0.30729 +
[6536]  MYC      <---    PIN1    -0.24167        0.078655        +
[6537]  CDC42    <---    PARD3   0.053034        0.23118 -
[6538]  FOXN2    --->    MAFK    0.5525  -0.61146        -
[6539]  CAMK2B   --->    CREB1   0.43562 -0.25046        +
[6540]  HMHA1    <---    RHOV    -0.62084        0.42373 -
[6541]  ATP1A4   --->    PIK3CD  -0.0036365      -0.0057548      -
[6542]  PRKCA    --->    SOS2    0.13325 -0.18146        -
[6543]  ARHGAP44         <---    RHOJ    -0.92575        0.61511 -
[6544]  CASP7    --->    GAS2    0.17116 -0.52748        +
[6545]  FN1      <---    ITGA4   -0.10408        -0.047194       -
[6546]  GHR      <---    IL6     -0.17721        0.073187        +
[6547]  CX3CR1   --->    GNAI2   1.1631  -1.1303 +
[6548]  CRADD    <---    MAP3K14 -0.56263        0.35307 -
[6549]  ARF6     --->    SLC2A4  0.3698  -0.60917        +
[6550]  IRS1     --->    SHC1    -0.012631       -0.14486        -
[6551]  GCKR     --->    NUP43   0.37289 -0.35015        -
[6552]  GP1BB    --->    SERPINE2        0.39784 -0.66389        -
[6553]  BMP2     <---    SMAD5   -0.24379        0.060804        -
[6554]  DCC      <---    MAP2K1  -0.10556        0.15829 -
[6555]  CACNA2D2         <---    CAMK2D  -0.2316 0.31309 +
[6556]  MAPK10   <---    TRAF6   -0.47605        1.1617  +
[6557]  FGR      --->    MAPK14  0.92013 -1.0925 +
[6558]  ARRB2    --->    HDAC1   0.22783 -0.18981        +
[6559]  LRP5     <---    WNT10B  -0.28769        0.39899 +
[6560]  GNAO1    <---    JAK2    -0.20109        0.14563 +
[6561]  HDAC2    <---    PPM1F   -0.82994        1.1353  -
[6562]  FST      --->    INHBB   0.51875 -0.80349        +
[6563]  CD36     <---    PRKAA1  -0.49798        0.49396 +
[6564]  NRAS     <---    RASGRP2 -0.1488 0.16462 +
[6565]  BID      <---    PLEKHG5 -0.45928        0.29273 +
[6566]  TP73     <---    WWOX    -0.10552        -0.010727       +
[6567]  ADCY9    <---    CAMK4   -0.63235        0.8673  +
[6568]  EIF2AK3  <--->   MAPK13  0.31014 0.31014 -
[6569]  CD247    --->    GNB1    0.029779        -0.043979       -
[6570]  CSNK1A1L         --->    WNT6    0.83912 -0.78694        -
[6571]  COL6A3   --->    ITGA8   -0.0067115      -0.019363       +
[6572]  ARF4     <---    EGFR    -0.2366 0.22331 +
[6573]  CSF2     --->    IL21R   0.059874        -0.059106       +
[6574]  CAMK2D   --->    GRM1    0.65286 -0.45637        +
[6575]  EP300    --->    FZR1    0.007799        -0.0077682      +
[6576]  RAP1B    --->    TIAM1   0.20928 -0.13672        +
[6577]  LAMC2    --->    MMP20   -0.0045452      -0.11357        -
[6578]  CTCF     <---    NR0B2   -0.028865       0.089984        -
[6579]  COL4A5   --->    PPIB    -0.024575       -0.068802       -
[6580]  AKT1     --->    BIRC5   0.13327 -0.28214        +
[6581]  RELA     <---    TRAF6   -0.37539        0.41785 -
[6582]  ABL1     --->    NR2C2   0.11209 -0.16505        -
[6583]  MOS      <---    NRAS    -0.12425        0.75653 +
[6584]  ITGB7    --->    TNXB    0.051076        -0.12975        -
[6585]  PPP2CA   <---    PRKCA   -0.12382        0.10585 -
[6586]  FSHB     --->    SMAD4   -0.050142       -0.10303        -
[6587]  KRAS     <---    RASGRP4 -0.17952        0.14935 +
[6588]  CLOCK    <---    CSNK1D  -0.29216        0.3669  +
[6589]  RAC3     <---    RASGRF2 -0.52428        0.44606 +
[6590]  FZD2     <---    SOST    -0.2929 0.24246 +
[6591]  ARHGAP10         <---    RHOT2   -0.27601        0.16621 -
[6592]  EIF2AK4  <--->   MAP2K7  0.31014 0.31014 -
[6593]  IL20     --->    IL6ST   0.056231        -0.11334        +
[6594]  FCER1A   --->    RAF1    0.032151        -0.14669        +
[6595]  BMPR1A   <---    E2F1    -0.10278        0.002004        +
[6596]  CALML5   --->    RASGRF1 0.29154 -0.36654        +
[6597]  FGF20    <---    NGFR    -0.47081        0.24157 -
[6598]  FOS      <---    HOXA7   0.041297        0.055709        +
[6599]  EDN1     <---    EP300   -0.0050208      0.00067605      +
[6600]  ITGAL    <---    SRF     -0.2365 0.062224        +
[6601]  GH1      --->    STAT5B  0.44408 -0.32334        +
[6602]  CRK      --->    ITGA2B  0.032574        -0.078547       -
[6603]  RAC2     --->    WASF1   0.076038        -0.038582       +
[6604]  HRAS     <---    LCP2    -0.79256        0.80926 +
[6605]  ADCY4    <---    CAMK2D  -0.44346        0.58749 +
[6606]  PSME4    --->    TNKS2   0.08803 -0.053465       +
[6607]  ADRB1    <---    GRK4    -0.28786        0.33635 +
[6608]  SOS2     --->    SPRED1  0.80058 -0.34155        +
[6609]  PDE4C    <---    PRKACG  -0.95055        1.4803  +
[6610]  GNA15    <---    GRPR    -1.1303 1.1631  +
```

[6611]	COL6A6	--->	ITGA10	-0.0047903	-0.03918	+
[6612]	FGF4	--->	IGF1R	0.25346	-0.0044024	+
[6613]	BARD1	--->	CCNE1	0.39798	-0.37735	-
[6614]	CCL23	<---	CCR3	-0.35895	0.21658	-
[6615]	ADCY1	<---	PRKX	-0.63235	0.8673	-
[6616]	CCL13	<---	CX3CR1	-0.35895	0.21658	-
[6617]	IL12RB2	<---	LEP	-0.11334	0.056231	+
[6618]	SP1	<---	TFDP2	0.008939	0.10042	+
[6619]	PSMD7	--->	RBX1	-0.026181	-0.051012	+
[6620]	BLK	--->	PDGFB	0.37363	-0.26526	-
[6621]	MAPK3	--->	RARA	0.29076	-0.30308	+
[6622]	COL6A1	<---	PTK2B	-0.074601	-0.061837	+
[6623]	MMP12	<---	PLG	-0.1166	-0.013996	+
[6624]	FOS	--->	PDHA1	0.083245	-0.0046992	+
[6625]	NUP37	--->	U2AF2	0.31391	-0.17132	+
[6626]	MAPK11	<---	PPP2R2A	-0.068084	0.2712	-
[6627]	EPOR	<---	NFKB1	-0.17714	0.16433	-
[6628]	NFATC2	<---	PRKCQ	-0.60816	0.44346	+
[6629]	CREB1	<---	INS	-0.15131	0.21799	-
[6630]	ADORA1	<---	GRK1	-0.28786	0.33635	+
[6631]	IKBKB	--->	NOD2	0.12131	-0.17609	-
[6632]	CAMK2D	--->	RASGRF2	0.24914	-0.57965	+
[6633]	CBL	--->	ITSN1	0.023503	-0.010361	-
[6634]	FGD3	<---	RAC2	-0.14219	0.16153	-
[6635]	CSNK1D	--->	WNT7A	0.83912	-0.78694	-
[6636]	ACTA2	<---	MMP15	-0.29263	0.085904	+
[6637]	PFKFB1	--->	PRKAG3	-0.012105	-0.096604	-
[6638]	ARHGAP39	<---	RHOJ	-0.82986	0.43536	-
[6639]	MYLK	<---	PRKACG	0.025869	0.19898	+
[6640]	ADCY3	<---	PRKCA	-0.49495	0.42642	+
[6641]	EGR1	<---	ZMIZ2	-0.18136	0.32136	-
[6642]	ELF5	--->	STAT5B	0.26825	-0.19721	-
[6643]	SGMS1	<---	SMPD1	-0.5028	0.29452	+
[6644]	ITGA1	<---	VWF	-0.064609	-0.0018341	+
[6645]	MEF2A	<---	TNS4	-0.10793	-0.031176	-
[6646]	LEP	--->	STAT3	0.44408	-0.32334	+
[6647]	CDK6	--->	MAX	0.60415	-0.53416	-
[6648]	POTEKP	<---	RAC1	-0.51168	0.52339	+
[6649]	SMAD4	--->	TLE4	-0.016256	-0.083613	-
[6650]	GRIP1	--->	MYOD1	0.48274	-0.46191	+
[6651]	FADD	--->	TNFRSF10C	0.2446	-0.54302	-
[6652]	IFNGR2	<---	PRKCD	-0.22841	0.2356	+
[6653]	PDGFRB	--->	SHC2	0.19746	-0.1352	+
[6654]	EBF1	--->	HOMEZ	0.23604	-0.26388	+
[6655]	PTPN11	--->	SRC	-0.082668	-0.41996	-
[6656]	MAPK13	<---	NCF4	-0.26379	0.013605	+
[6657]	COL1A2	<---	LEPREL2	-0.18291	0.065305	+
[6658]	FOXO1	--->	IL6R	0.24623	-0.4117	-
[6659]	GNAI3	<---	KCNJ9	-0.011498	0.05262	-
[6660]	APOA1	<---	ESR1	-0.091347	-0.010433	+
[6661]	HLA-DQA2	--->	RFXANK	0.70499	-0.72198	-
[6662]	DRD3	<---	GRK6	-0.28786	0.33635	+
[6663]	CCR1	--->	CXCL6	0.21658	-0.35895	-
[6664]	ASCL1	<---	EP300	-0.02899	0.024715	+
[6665]	ANGPT2	--->	ARNT	0.0052685	-0.16068	-
[6666]	ESR2	--->	JUN	0.10351	-0.20904	+
[6667]	LRP5	<---	WNT8A	-0.28769	0.39899	+
[6668]	PRKCA	--->	TRPV4	0.31032	-0.3562	+
[6669]	PPP2CB	--->	STK3	0.77647	-0.59079	+
[6670]	RAP1GAP2	<---	YWHAB	-0.18529	-0.030101	-
[6671]	SEH1L	--->	SRSF1	0.31391	-0.17132	+
[6672]	FIGF	--->	KDR	0.31113	-0.023732	+
[6673]	ARR3	--->	WNT11	0.23514	-0.34616	-
[6674]	NGFR	--->	PLCB3	0.0056177	-0.057994	+
[6675]	CUL2	--->	UBE2D1	0.065927	-0.14391	+
[6676]	MAP2K4	<---	TRAF6	-0.47605	1.1617	+
[6677]	PBX3	--->	ZNF687	0.11044	0.025818	+
[6678]	AKT2	<---	PHLPP2	-0.79489	1.3907	+
[6679]	DLL1	<---	DNM1	-0.01106	0.0095349	+
[6680]	CCR4	--->	CXCL12	0.21658	-0.35895	-
[6681]	PPARGC1A	--->	STAT3	0.3835	-0.23142	-
[6682]	IL2RB	--->	PIK3R5	0.38701	-0.52584	+
[6683]	JAK2	--->	TEC	1.3974	-0.78729	+
[6684]	CD79A	<---	DOK1	0.064355	0.14963	-
[6685]	AKT1	--->	GRB10	0.087326	0.081418	-
[6686]	FZD1	<---	GNB4	-0.19257	0.19185	-

```
[6687]  RAP1B    <---    YWHAB   -0.25073        0.15053 +
[6688]  ACTG1    <---    MYL10   -0.74059        0.64379 +
[6689]  PLCG1    <---    PRKCZ   -0.027756       0.040945        -
[6690]  EGFR     --->    HSP90AA1        0.093026        -0.078504       +
[6691]  NFATC4   --->    PTGS2   0.30729 -0.48141        +
[6692]  PPP2CB   <---    PRKCG   -0.12382        0.10585 -
[6693]  CX3CR1   <---    GRK1    -0.28786        0.33635 +
[6694]  FGFR3    --->    SHC3    0.23069 -0.27006        +
[6695]  MYC      <---    ZNF280B -0.1035 0.01902 -
[6696]  IL10     --->    MAP3K5  0.56266 -0.019907       +
[6697]  FGF13    --->    FGFR4   0.26645 -0.41462        +
[6698]  FGFR3    --->    MAPK1   0.37337 -0.44107        -
[6699]  F2R      --->    SNX2    0.11779 -0.2522 -
[6700]  CRKL     --->    RHOQ    0.43868 -0.057401       +
[6701]  GNAI2    <---    MAPK9   -0.22953        0.50225 -
[6702]  CRKL     <---    INSR    0.024543        0.059416        +
[6703]  CTNNB1   <---    FZD9    -0.12047        0.05338 +
[6704]  BRAF     <---    RAP1B   -0.86318        0.78219 +
[6705]  ITGB7    --->    LAMA2   -0.0073727      -0.008847       -
[6706]  BMP8A    <---    NOG     -0.37709        0.17799 +
[6707]  CDH13    <---    CTNNB1  -0.31672        0.31045 +
[6708]  CEBPB    <---    DCN     -0.36538        0.16947 -
[6709]  MAP3K1   <---    TLR4    -0.15867        0.0069824       +
[6710]  EPOR     <---    THPO    -0.16507        0.040195        +
[6711]  MAPK8    <---    TNFSF12 0.048942        0.10494 -
[6712]  PTK2B    <---    RAC2    -0.14524        -0.084837       -
[6713]  FGFR1    <---    SOS1    0.019724        0.054101        -
[6714]  DEPDC1B <---    RHOT2   -0.38519        0.18509 -
[6715]  EP300    --->    KLF1    0.016752        -0.010456       +
[6716]  PRKCB    --->    USF1    0.45556 -0.54995        -
[6717]  KLF1     <---    POU2F2  0.049909        0.078944        +
[6718]  CCR4     <---    GRK1    -0.28786        0.33635 +
[6719]  FRZB     --->    WNT9A   1.0317  -1.1063 +
[6720]  CSF1R    <---    EGF     0.0086367       0.054796        +
[6721]  ARHGEF7 <---    RAC3    -0.044321       0.055318        -
[6722]  EPHB2    --->    GIT1    -0.0039653      -0.019814       +
[6723]  ACTB     <---    MMP24   -0.29263        0.085904        +
[6724]  CACNA1S <---    GNB1    -0.095409       0.10645 +
[6725]  IL16     --->    TCF12   0.48274 -0.46191        -
[6726]  CAV1     <---    SRC     -0.45391        0.63648 +
[6727]  CCNC     <---    SMAD3   -0.18584        0.054214        -
[6728]  EPHB1    --->    GIT1    -0.0039653      -0.019814       +
[6729]  ARHGAP25        <---    CDC42   -1.2097 0.92798 -
[6730]  E2F4     <---    UBE2C   -0.24498        0.18226 -
[6731]  IL6R     <---    PRKCD   -0.18653        0.14297 -
[6732]  GNAI1    <---    HTR1D   -1.1303 1.1631  +
[6733]  GRK7     --->    HTR1A   0.33635 -0.28786        +
[6734]  GNB1     <---    KCNJ9   -0.070696       0.05091 -
[6735]  CTNNA1   --->    MMP8    0.38719 -0.55042        -
[6736]  TRAF6    --->    UBE2N   0.57542 -0.40839        -
[6737]  PIK3CD   <---    THRB    -0.021852       -0.0056486      +
[6738]  CXCR4    --->    FOS     0.41745 -0.36998        -
[6739]  ADORA1   <---    GRK7    -0.28786        0.33635 +
[6740]  CCL15    <---    CCR6    -0.35895        0.21658 -
[6741]  FZD7     <---    WNT3A   -0.78209        0.63734 +
[6742]  CXCR3    --->    GNAZ    1.1631  -1.1303 +
[6743]  EGR1     <---    MBD1    -0.10808        0.18562 -
[6744]  CDC42    --->    MAPK13  1.2491  -0.20304        +
[6745]  COL11A2 --->    ITGA11  -0.013036       -0.10803        +
[6746]  LIFR     <---    PIK3CB  -0.016602       0.0042292       -
[6747]  FGD3     <---    RAC1    -0.14219        0.16153 -
[6748]  CSF2RB   <---    SOCS1   -0.095304       0.022627        +
[6749]  FLT1     --->    IRS1    1.0665  -0.046952       +
[6750]  AKT3     <---    PPP2CA  -0.049067       0.049194        -
[6751]  PDGFRA   <---    POU5F1  -0.23272        0.21112 +
[6752]  CSF2     --->    IFNLR1  0.48366 -0.22056        +
[6753]  CDH3     <---    MMP13   -0.24168        -0.0076207      +
[6754]  INSR     <---    PDGFD   -0.033666       0.27735 +
[6755]  PLD1     <---    PRKCG   -0.11393        0.14533 +
[6756]  IGSF5    <---    PRKCE   -0.26358        0.22932 +
[6757]  GNAO1    <---    HTR1F   -1.1303 1.1631  +
[6758]  MPL      <---    SOCS4   -0.10625        0.079572        +
[6759]  HAP1     <---    RAC3    -0.11932        0.38072 -
[6760]  GNB2     <---    KCNJ6   -0.098063       0.10019 -
[6761]  EP300    --->    ID1     0.024715        -0.02899        +
[6762]  CREB1    <---    FAS     -0.24954        0.14229 -
```

[6763]	FLT4	<---	VEGFC	-0.076002	0.030896	+
[6764]	EP300	<---	PRKACA	-0.006941	0.013728	-
[6765]	IFNAR1	<---	IL12B	0.012893	0.051025	+
[6766]	ADORA3	<---	KCNJ6	-0.068284	-0.033535	+
[6767]	CREBBP	--->	ZFHX3	0.002889	-0.0075585	+
[6768]	PIK3CA	<---	PRKCG	-0.06189	0.049814	-
[6769]	COPA	<---	GPLD1	-0.089917	0.078044	+
[6770]	FCGR2A	--->	SYK	1.098	-0.94713	+
[6771]	MYL3	<---	TPM4	-0.091663	0.035745	-
[6772]	MYH8	--->	TPM4	0.064903	-0.10325	+
[6773]	KCNMB3	<---	PRKX	-0.31173	0.40189	+
[6774]	IL2RB	<---	PRKCB	-0.41496	0.33151	-
[6775]	HBEGF	<---	HSP90AA1	-0.46112	0.41042	+
[6776]	RAC3	<---	TRIP10	0.074898	0.19853	+
[6777]	HRAS	--->	PLD2	0.077686	-0.071006	+
[6778]	TBK1	--->	USF1	0.99789	-0.87887	-
[6779]	LIPE	<---	PRKX	-0.43325	0.64376	+
[6780]	IKBKG	<---	UBE2V1	-0.39712	0.34662	+
[6781]	CSF2	<---	PRKACG	-0.1175	0.35399	-
[6782]	MAPK11	<---	TNF	0.048942	0.10494	-
[6783]	PPARG	<---	SORBS1	-0.084147	0.050641	-
[6784]	PLK1	<---	PSME4	0.042964	0.07708	+
[6785]	BAD	<---	PPP3R1	-0.43309	0.33355	+
[6786]	IL11RA	<---	IRS1	0.064355	0.14963	-
[6787]	FOXA1	--->	HNF1A	0.53035	-0.57954	+
[6788]	PSMD4	--->	TNKS2	0.095602	-0.12329	+
[6789]	PIAS2	--->	STAT3	1.2392	-0.72187	+
[6790]	ANGPT4	--->	FGFR1	0.41032	-0.47379	+
[6791]	FGF5	--->	FLT4	0.071829	-0.16555	+
[6792]	PTK2B	<---	VCL	-0.31846	0.12607	-
[6793]	E2F4	--->	SP1	0.23995	-0.10098	-
[6794]	GNAI1	--->	SCN1A	0.16571	-0.1017	+
[6795]	NOTCH4	<---	PCSK7	-0.05629	0.069806	+
[6796]	FAM13A	<---	RAC2	-1.5245	1.016	-
[6797]	CTCF	<---	MAP2K1	-0.50997	0.66514	-
[6798]	RXRG	<---	SORBS1	-0.17063	0.045962	-
[6799]	HRAS	--->	PTK2	-0.052556	-0.089194	-
[6800]	IFNAR1	--->	JAK3	1.1771	-1.1167	+
[6801]	NCF4	--->	VCAM1	0.047838	-0.072954	-
[6802]	ANGPT1	<---	PLG	-0.067004	-0.029854	-
[6803]	PLXNA1	<---	PLXNA4	0.061725	0.091133	+
[6804]	ANGPT2	<---	KDR	-0.048064	0.33642	-
[6805]	IGF1	--->	KLK3	0.06155	-0.12987	-
[6806]	MAP3K2	<---	RAC3	-0.17545	1.1267	+
[6807]	RALBP1	<---	RHOBTB1	-0.92575	0.61511	-
[6808]	CREB3L1	<---	PRKG1	-1.2233	1.1994	+
[6809]	CDK6	--->	CDKN1A	0.71039	-0.49354	+
[6810]	IL2RB	<---	IRS1	0.015197	0.14815	-
[6811]	IL6ST	<---	PTPRE	-0.51349	0.43351	+
[6812]	EPOR	<---	IFNB1	-0.55283	0.27509	+
[6813]	SPTBN1	<---	TGFBR1	-0.036463	0.11863	+
[6814]	GRB2	--->	SPRY2	0.18885	-0.20205	-
[6815]	CTNNA3	--->	YWHAQ	1.0272	-1.0665	+
[6816]	ARHGAP18	<---	RHOD	-1.5245	1.016	-
[6817]	CRADD	<---	SMPD3	-0.081649	-0.042689	-
[6818]	BMPR2	--->	SMAD2	0.6082	-0.49302	-
[6819]	DAND5	--->	GJA1	0.021163	-0.044865	+
[6820]	AKAP13	<---	RHOB	-0.22586	0.072537	-
[6821]	ITGA9	<---	PTK2	-0.076638	-0.038051	-
[6822]	SIX5	--->	ZNF569	0.16974	-0.14212	+
[6823]	ARHGEF6	<---	HRAS	-0.026438	0.029604	+
[6824]	PRKCA	--->	TBXA2R	0.27696	-0.37622	+
[6825]	ITGB1	<---	PRKACG	-0.09775	0.14787	-
[6826]	ITGA5	<---	RAC1	-0.10645	0.030711	-
[6827]	CAMK2D	<---	NRAS	-0.038527	0.42208	-
[6828]	FZD6	<---	SAG	-0.073345	0.063196	-
[6829]	NGEF	<---	RHOF	0.091479	0.13323	-
[6830]	CSF2RB	<---	IFNG	-0.21521	0.082568	+
[6831]	ATF3	<---	HOXC13	0.041297	0.055709	-
[6832]	SRF	<---	VCL	-0.32582	0.28827	-
[6833]	CD3D	<---	PTPN6	-0.29307	0.36484	-
[6834]	CTBP2	<---	FOXC1	-0.28308	0.17988	-
[6835]	PDGFD	--->	SHC1	0.14513	-0.23434	+
[6836]	LAMB3	--->	MMP20	-0.0045452	-0.11357	-
[6837]	AKAP13	<---	RHOBTB1	-0.13809	0.042896	-
[6838]	PRKCA	--->	PTK2B	0.09356	-0.1078	+

[6839]	DEPDC7	<---	RHOA	-0.058594	0.11751	-
[6840]	PRKACB	--->	TRPV5	0.54889	-0.41958	+
[6841]	MAPK13	--->	MEF2C	1.0903	-1.4506	+
[6842]	ADORA3	--->	GNAI1	0.027216	-0.0021531	+
[6843]	EPHB2	--->	PIK3CA	-0.0062104	-0.007372	+
[6844]	ATP1B2	<---	NR3C2	-0.14879	-0.015388	+
[6845]	PRKCI	--->	STAT5A	0.53838	-0.49624	-
[6846]	CASP7	--->	PTK2	0.10272	-0.39495	+
[6847]	PLEKHG5	--->	RHOT2	0.33144	-0.31781	+
[6848]	CBLC	--->	SPRY2	0.48869	-0.21829	-
[6849]	PAK2	<---	RAC1	-1.1901	0.96651	-
[6850]	CAMK2B	<---	STAT1	-0.75243	0.87452	-
[6851]	DVL2	<---	FZD6	-0.12172	0.085851	+
[6852]	CTNNA1	--->	GRIA2	0.86321	-0.75697	+
[6853]	LIPE	<---	PRKAR2A	-0.52243	0.57268	+
[6854]	IGF1R	<---	MAPK3	0.083131	0.1759	-
[6855]	DUSP1	--->	MAPK10	1.353	-1.036	+
[6856]	GRIA1	<---	PPP1CA	-0.67094	0.67498	+
[6857]	FOXO1	<---	PRMT1	-0.62598	0.62127	+
[6858]	BCL2L1	<---	FOXA2	-0.9984	0.76486	+
[6859]	CHD2	--->	SPIB	0.050188	-0.12153	+
[6860]	GCGR	--->	GNB4	0.059668	-0.060578	+
[6861]	MYC	<---	NR0B2	-0.30736	0.080113	-
[6862]	ETV7	--->	ZEB1	0.17554	-0.13167	-
[6863]	ARHGAP26	<---	RHOBTB2	-0.40124	0.29337	-
[6864]	RNF10	--->	TAL1	0.24049	-0.52538	-
[6865]	MAP3K14	--->	RELB	0.92767	-0.99472	+
[6866]	RELA	--->	TP73	0.01517	-0.15168	-
[6867]	TP53BP2	--->	YAP1	-0.055663	-0.057025	+
[6868]	LMO4	--->	SOS1	0.16687	-0.0085893	+
[6869]	CRKL	--->	PIK3R5	0.25175	-0.30884	+
[6870]	ACVR1C	<---	INHBE	-0.20846	0.1146	+
[6871]	ITGA4	<---	LAMB3	-0.066621	-0.0090065	+
[6872]	DKK2	--->	WNT7B	0.54164	-0.69755	+
[6873]	MYB	<---	SMARCA2	-0.015539	-0.01424	-
[6874]	SOX2	<---	TEAD2	-0.42834	0.19226	+
[6875]	ITGB5	<---	PTK2B	-0.04335	-0.039708	+
[6876]	MAP3K2	<---	SRC	-0.36926	0.40451	+
[6877]	CCNB1	<---	SFN	-0.62256	0.59442	+
[6878]	HMOX1	--->	USF2	0.43996	-0.52432	-
[6879]	GZMA	<---	MAPK8	-0.26091	0.40675	-
[6880]	FCGR2A	--->	VAV2	0.17916	-0.063802	+
[6881]	MEF2A	<---	TLR10	-0.53006	0.28352	-
[6882]	GJD2	<---	MAPK3	-0.26091	0.40675	+
[6883]	GRAP	<---	PRKCB	-0.20419	0.22188	-
[6884]	CSNK1G2	--->	DVL1	0.31547	-0.38065	+
[6885]	ATP1A3	--->	PIK3R1	0.016367	-0.047791	-
[6886]	MYD88	<---	TLR4	0.05829	0.13137	+
[6887]	CD86	--->	PIK3R3	0.0075342	-0.12681	+
[6888]	AKT1	--->	GRB14	0.087326	0.081418	-
[6889]	BHLHE40	<---	ZNF805	-0.38032	0.36387	-
[6890]	ARHGEF6	<---	PAK2	0.02191	0.098798	+
[6891]	ADCY7	<---	GNAI3	-0.63317	1.0384	-
[6892]	CHRM4	--->	GNB5	1.1575	-0.92616	+
[6893]	FGF14	--->	FGFR4	0.40218	-0.67149	+
[6894]	BCAR1	--->	MAPK8	0.16026	-0.10066	+
[6895]	BCL6B	<---	EP300	-0.0074415	0.0090612	+
[6896]	LPAR5	--->	PIK3R5	0.56062	-0.77034	+
[6897]	CASP3	--->	NGF	0.635	-0.97212	-
[6898]	CSF2RB	<---	IL3RA	-0.039925	0.014761	+
[6899]	CCR7	--->	CXCL9	0.21658	-0.35895	-
[6900]	CREB3	<---	CREBBP	-0.010885	0.015595	-
[6901]	RHOC	<---	TRIP10	0.074898	0.19853	+
[6902]	PARD6B	<---	RAC1	0.018694	0.089672	+
[6903]	NFIC	<---	PGK1	-0.073047	-0.044792	-
[6904]	CDH1	<---	RAB5A	-0.40807	0.12902	+
[6905]	OCRL	<---	RHOV	-0.92575	0.61511	-
[6906]	IL3RA	<---	PIK3CA	-0.0068344	-0.0060807	-
[6907]	ITGB2	<---	RHOA	-0.01596	0.026206	-
[6908]	CNTF	--->	IFNAR2	0.20113	-0.087402	+
[6909]	NFATC2	--->	PPARG	0.0055153	-0.18215	+
[6910]	GDF5	<---	NOG	-0.37709	0.17799	+
[6911]	EDN1	--->	HDAC2	0.37744	-0.31114	-
[6912]	ITGAM	<---	RGS14	-1.0937	-0.060891	+
[6913]	KRAS	--->	NTF3	0.53989	-0.48322	-
[6914]	ABCB1	<---	HIF1A	0.033537	0.087688	+

[6915]	MAP3K14	<---	TAB3	-0.13728	0.23085	-
[6916]	IL20RA	<---	PTPN6	-0.75018	0.80704	+
[6917]	CCL22	<---	CX3CR1	-0.35895	0.21658	-
[6918]	GNB4	<---	PIK3CD	-0.012551	0.008521	-
[6919]	HOMER3	--->	PIK3CG	0.0081607	-0.01428	+
[6920]	CDC25B	<---	MAPK13	-0.97911	0.26855	+
[6921]	ELK1	<---	NFKB1	-0.23502	0.23585	+
[6922]	MAPK13	<--->	MAPKAPK5	0.31014	0.31014	-
[6923]	CCL25	<---	CXCR6	-0.35895	0.21658	-
[6924]	MYC	--->	STAT5A	0.048778	-0.065592	-
[6925]	TRIM11	--->	ZNF263	0.2556	-0.045889	-
[6926]	DTX1	<---	NOTCH2	-0.22258	0.25014	+
[6927]	CCL27	<---	CX3CR1	-0.35895	0.21658	-
[6928]	FOXO4	<---	STAT3	0.11967	0.28002	+
[6929]	PLA2G6	<---	SREBF1	-0.051309	0.10531	+
[6930]	CTCF	--->	ZNF354A	-0.0066414	-0.044983	+
[6931]	BTK	--->	FOS	0.24363	-0.11138	+
[6932]	BHLHE40	<---	MEF2C	-0.26031	0.094795	+
[6933]	IFNGR1	<---	SOCS2	-0.31187	-0.11646	+
[6934]	STAT4	<---	TGFB1	-0.15145	0.19016	+
[6935]	BCAR1	--->	FGB	0.15003	-0.15216	-
[6936]	MAP4K4	--->	MYD88	0.12293	-0.044474	-
[6937]	HLA-DQA2	--->	LAT	0.059049	-0.30562	+
[6938]	ERBB2	--->	NRAS	0.17363	-0.11617	+
[6939]	APC	<---	AXIN1	-0.0071738	-0.0025842	+
[6940]	GNA13	--->	RHOB	1.2694	-0.93465	+
[6941]	ITGB4	--->	THBS4	0.015237	-0.011799	-
[6942]	CFTR	<---	PRKAA1	-0.37336	0.33589	+
[6943]	MYLK4	<--->	PRKACG	0.31014	0.31014	-
[6944]	ATF4	<---	PRKCA	-0.63701	0.72046	+
[6945]	RBX1	<---	UBE2D2	-0.050403	-0.029332	-
[6946]	GNAI2	<---	HTR1A	-1.1303	1.1631	+
[6947]	GLI1	<---	STK36	-0.57298	0.8559	+
[6948]	GAB2	<---	PIK3R2	-0.32654	0.0213	-
[6949]	PRKAG1	--->	STAT3	0.41852	-0.31782	-
[6950]	DDX20	--->	EGR2	0.18149	-0.10437	-
[6951]	CBX5	--->	JUN	0.17999	-0.25166	-
[6952]	HDAC8	<---	SMAD1	-0.21285	0.15191	-
[6953]	CRKL	<---	SRF	-0.027624	-0.000937	+
[6954]	ADAMTS3	--->	COL5A1	-0.02131	-0.035774	+
[6955]	CAMK2B	--->	ITPR1	0.31376	-0.17692	-
[6956]	GNA15	<---	PLCL2	-0.023623	0.0010472	-
[6957]	CHRM3	--->	KCNJ3	0.11779	-0.2522	-
[6958]	CAMK2B	--->	PIK3C3	0.12615	-0.096379	+
[6959]	GMIP	<---	RHOB	-1.011	0.68428	-
[6960]	FFAR2	--->	GNAI1	1.1631	-1.1303	+
[6961]	ARHGAP44	<---	RAC3	-0.92575	0.61511	-
[6962]	BCAR1	--->	VAV3	0.026378	-0.017247	+
[6963]	CCR2	--->	CXCL6	0.21658	-0.35895	-
[6964]	BCR	<---	CBLC	-0.07596	0.10324	-
[6965]	ITCH	--->	TP63	0.051699	-0.11799	+
[6966]	CDKN1A	<---	CUL1	-0.25493	0.27648	-
[6967]	PCK1	--->	RXRA	-0.16619	-0.17934	-
[6968]	CCNB1	<---	CDC25B	-0.245	0.10994	+
[6969]	RIF1	--->	YY1	0.23793	-0.12118	-
[6970]	PLEKHG2	<---	RHOV	-0.23262	0.26814	-
[6971]	BRCA1	--->	CCNC	0.56829	-0.54919	+
[6972]	NRAS	--->	PTK2	-0.052556	-0.089194	-
[6973]	GAB1	<---	SRC	-1.1861	0.83596	-
[6974]	CTBP2	--->	HLX	0.04296	-0.16727	+
[6975]	HSPA1A	--->	MAPK9	1.2218	-0.90384	+
[6976]	MOS	<---	MRAS	-0.20304	1.2491	+
[6977]	BCL10	<---	RELA	-0.88183	0.6154	-
[6978]	AR	<---	MAPK14	-0.27508	0.26535	+
[6979]	DUSP4	--->	MAPK10	1.353	-1.036	+
[6980]	GRK6	--->	HTR5A	0.33635	-0.28786	+
[6981]	AKT3	--->	FGFR2	0.053523	-0.092062	-
[6982]	MAP2K7	<--->	MAPK10	0.31014	0.31014	-
[6983]	IGF1	--->	PIK3CA	0.00905	-0.022614	+
[6984]	CRKL	--->	ITGA2	0.018691	-0.085251	-
[6985]	FOXA2	--->	SMARCA1	0.059755	-0.081264	+
[6986]	BNC1	<---	SPI1	-0.15205	0.28002	+
[6987]	CNTF	--->	IL6R	0.12377	-0.18387	+
[6988]	NANOG	--->	UNC45B	-0.024885	-0.13225	+
[6989]	ARNT	<---	EDN1	-0.16173	0.16165	-
[6990]	G6PC2	--->	USF2	0.25527	-0.35391	-

[6991]	ARHGEF6	<---	NRAS	-0.026438	0.029604	+
[6992]	MAPK13	<--->	PRKACA	0.31014	0.31014	-
[6993]	MAPK1	<---	RAC1	-0.20304	1.2491	-
[6994]	CCL8	<---	XCR1	-0.35895	0.21658	-
[6995]	ATF6	<---	DDIT3	-0.098546	0.15465	-
[6996]	GNAO1	<---	PLCG1	-0.0052535	0.030885	-
[6997]	CD40	<---	MAPK14	-0.57721	0.64173	-
[6998]	ATF2	<---	NT5E	-0.66816	0.44089	-
[6999]	ARHGEF12	<---	PLXNB3	-0.033014	0.061067	+
[7000]	CDK7	--->	USF2	0.99789	-0.87887	-
[7001]	FOS	<---	ZNF76	-0.0074278	0.17997	-
[7002]	GRK4	--->	OPRM1	0.33635	-0.28786	+
[7003]	FZD5	<---	WNT11	-0.78209	0.63734	+
[7004]	AR	<---	KLKB1	-0.16924	0.10365	-
[7005]	EGR2	<---	NFKB1	-0.13106	0.32527	+
[7006]	GNB1	<---	SRC	-0.29244	0.41365	-
[7007]	FOS	<---	PGF	-0.18489	0.28082	-
[7008]	DAND5	--->	TIMP1	0.036704	-0.072158	+
[7009]	CUL1	--->	GLI2	0.25589	-0.19462	+
[7010]	MMP19	--->	POTEKP	0.085904	-0.29263	+
[7011]	MAPK12	<---	TAB2	-0.13728	0.23085	+
[7012]	AREG	<---	WWTR1	-1.4107	1.1931	+
[7013]	RACGAP1	<---	RHOA	-1.011	0.68428	-
[7014]	ITGA2B	<---	LAMB3	-0.066621	-0.0090065	+
[7015]	FOS	<---	MAPK12	-0.57905	1.0858	+
[7016]	CDH1	<---	RAB7A	-0.40807	0.12902	+
[7017]	BID	<---	CTSD	-0.29746	0.19787	+
[7018]	CCL26	<---	CCR2	-0.35895	0.21658	-
[7019]	APP	--->	RXRA	-0.010057	-0.024075	-
[7020]	GNAO1	<---	HTR1B	-1.1303	1.1631	+
[7021]	FZD5	<---	GNB4	-0.19257	0.19185	-
[7022]	CSF2RB	<---	PIK3CA	-0.015702	0.0074192	-
[7023]	CCR10	--->	CXCL1	0.21658	-0.35895	-
[7024]	IL23A	<---	MAPK12	-0.26091	0.40675	+
[7025]	RHOT1	<---	VAV3	0.0058267	0.038116	+
[7026]	GNA11	<---	GNRHR	-1.1303	1.1631	+
[7027]	PRKCA	--->	PRKD1	0.11731	-1.1868	+
[7028]	CSF2	--->	ETS2	0.059874	-0.059106	-
[7029]	RGMB	--->	SMAD5	0.016452	-0.10584	+
[7030]	CCL3	<---	CCR5	-0.35895	0.21658	-
[7031]	BRAF	<---	RIT1	-0.86318	0.78219	+
[7032]	MYO16	--->	STAT3	0.25001	-0.18672	-
[7033]	IL1A	<---	MAPK11	-0.078488	0.15109	+
[7034]	AR	<---	CEBPA	-0.16924	0.10365	-
[7035]	PIK3R1	<---	RET	-0.21657	0.14268	+
[7036]	EP300	--->	MYH14	-0.0015178	-0.0036136	+
[7037]	EPO	<---	JAK1	-0.25312	0.099846	-
[7038]	CD3E	<---	PLCG1	-0.021591	0.04532	-
[7039]	ACTA2	<---	RAC1	-0.51168	0.52339	+
[7040]	PRKCQ	--->	RASAL2	0.20982	-0.11373	-
[7041]	JUP	<---	MMP13	-0.26058	0.11034	+
[7042]	KIT	--->	PLCB4	0.013976	-0.014907	+
[7043]	CHRM1	--->	GNAQ	1.1631	-1.1303	+
[7044]	MYLK2	<--->	PRKX	0.31014	0.31014	-
[7045]	CRK	--->	ITGB4	0.01784	-0.030738	-
[7046]	CTNNB1	<---	SRC	-0.4317	0.52076	-
[7047]	NGFR	--->	PIK3CD	0.006882	-0.049773	+
[7048]	GRB2	--->	IL6ST	0.13981	-0.11713	-
[7049]	AKT2	<---	PDPK1	-1.2643	0.99099	+
[7050]	BMP7	<---	SMAD5	-0.24379	0.060804	-
[7051]	CACNA1S	<---	PPP2R2A	-0.37563	0.37154	+
[7052]	MAPK11	<--->	MKNK1	0.31014	0.31014	-
[7053]	CTBP2	--->	HOXD8	0.04296	-0.16727	+
[7054]	ADORA3	--->	GNAI3	0.027216	-0.0021531	+
[7055]	HDAC1	<---	SMAD1	-0.21285	0.15191	-
[7056]	EIF4EBP1	<---	RHEB	-0.36494	0.41934	+
[7057]	CCL15	<---	CXCR3	-0.35895	0.21658	-
[7058]	H1F0	--->	RB1	0.22906	-0.33471	-
[7059]	PHF19	--->	ZBTB7A	0.18798	-0.12383	-
[7060]	PRKCI	--->	SHC3	0.46896	-0.43625	-
[7061]	CASP1	<---	NLRC4	-0.47905	-0.15785	+
[7062]	RHOV	--->	VAV2	0.058196	0.018587	-
[7063]	NR2F2	--->	TCF12	0.27747	-0.4408	-
[7064]	FGFR1	<---	PDGFD	-0.61313	0.56088	+
[7065]	CALML6	--->	PPP3R1	0.26507	0.098466	+
[7066]	E2F1	<---	RPS6KB1	-0.40203	0.18988	-

[7067]	DVL1	<---	PSMD14	-0.62919	0.4836	+
[7068]	ADRBK1	--->	EDN1	0.33027	-0.27212	+
[7069]	SDF2L1	<---	STAT1	-0.31832	0.26282	+
[7070]	L1CAM	<---	RPS6KA2	-0.12605	0.076189	+
[7071]	IL3RA	<---	PTPN6	-0.29595	0.42284	+
[7072]	CHD9	--->	RXRA	0.0079285	-0.16137	-
[7073]	CACNA1D	<---	HTR2A	-0.36046	0.31836	+
[7074]	RGL2	<---	RRAS	-1.2366	1.4276	+
[7075]	CBL	--->	PRLR	0.18092	-0.10684	+
[7076]	LEPR	--->	SOCS2	-0.098904	-0.12483	-
[7077]	HSPA8	--->	RIMS1	0.27786	-0.29689	+
[7078]	OSMR	<---	SOCS4	-0.1723	0.11167	+
[7079]	IL10RA	<---	SOCS7	-0.40298	0.060066	+
[7080]	FOXO3	--->	JUND	0.38594	-0.42469	-
[7081]	APC	<---	POU2F1	-0.0092396	-0.0038944	+
[7082]	GRK4	--->	HTR1D	0.33635	-0.28786	+
[7083]	GFRA1	<---	SRC	-0.36929	0.54982	+
[7084]	GNAI2	<---	RGS5	-1.2531	1.2063	+
[7085]	NOS1	<---	PRKAR2B	-0.04911	0.047333	+
[7086]	PLXNA2	<---	RAC3	-0.31182	0.50205	-
[7087]	GSK3B	--->	PIK3R2	0.14156	-0.28902	-
[7088]	CCL18	<---	CXCR3	-0.35895	0.21658	-
[7089]	SFRP4	--->	WNT2	1.0317	-1.1063	+
[7090]	GNAI1	--->	KRAS	1.2694	-0.93465	+
[7091]	MAP2K1	--->	SOS1	0.25162	-0.40162	-
[7092]	JUN	<---	TNF	-0.22939	0.14476	-
[7093]	CTNNB1	<---	WNT9A	-0.187	0.023012	+
[7094]	GRAP2	--->	PLCG2	0.031512	0.020726	+
[7095]	CXCL14	<---	CXCR5	-0.35895	0.21658	-
[7096]	CTNNB1	<---	ENAH	-0.26627	0.30998	-
[7097]	CCND1	<---	CREM	-0.14825	0.24653	+
[7098]	GNA14	<---	MAPK8	-0.22953	0.50225	-
[7099]	COL11A2	--->	ITGA7	-0.013036	-0.10803	+
[7100]	ITGA9	--->	THBS4	0.046345	-0.039839	-
[7101]	CTNNB1	<---	PCDHAC2	-0.19204	0.11503	-
[7102]	E2F6	<---	ZNF644	-0.044303	-0.0061265	-
[7103]	IKBKG	<---	MAP2K7	-0.45312	0.62218	-
[7104]	EPHA6	--->	NGEF	0.030553	-0.091172	+
[7105]	GNB1	<---	PLCB3	-0.011346	-0.0053379	-
[7106]	ARHGAP26	<---	RHOJ	-0.65972	0.48549	-
[7107]	HSP90AA1	--->	ITGB3	0.077183	-0.098395	-
[7108]	EZR	<---	FASLG	-0.069873	-0.0098245	-
[7109]	PPP1R1B	<---	PRKACB	-0.92753	1.2659	-
[7110]	MAPK11	<---	NCK2	-0.31116	0.22669	+
[7111]	AKT3	--->	ATF6B	0.82132	-1.3257	+
[7112]	APP	<---	MAPK7	-0.023982	0.030823	-
[7113]	MYB	--->	MYOD1	0.2389	-0.25448	-
[7114]	CXCR6	--->	XCL1	0.21658	-0.35895	-
[7115]	SP5	<---	TCF7L2	-0.026148	0.31266	+
[7116]	AKT2	--->	STAT4	1.212	-1.5407	-
[7117]	EPHA4	--->	PLCG1	-0.0086575	-0.025441	+
[7118]	GLI1	<---	PSMD1	-0.077956	0.20686	+
[7119]	CD3D	--->	GNAI2	0.05262	-0.011498	-
[7120]	MAPK14	--->	PLA2G4A	0.18871	-0.84715	+
[7121]	GNAO1	<---	MAPK1	-0.22953	0.50225	-
[7122]	FOXA1	--->	IRS1	0.23139	-0.047044	+
[7123]	CRKL	--->	PXN	0.098826	-0.1839	+
[7124]	MAPK1	--->	TAL1	0.99789	-0.87887	-
[7125]	HNF4A	--->	NELFE	0.23448	-0.31347	+
[7126]	ESR1	--->	HMBOX1	0.10711	-0.27075	+
[7127]	CEBPB	<---	TRIB3	-0.26091	0.40675	-
[7128]	ADRBK1	--->	GRM6	0.53935	-0.4166	+
[7129]	ETV1	<---	MAPKAPK2	-0.26091	0.40675	+
[7130]	APBB1IP	--->	FGB	0.097072	-0.12087	-
[7131]	ESRRA	--->	SLC2A4RG	0.0080497	-0.15747	+
[7132]	CCL18	<---	CXCR5	-0.35895	0.21658	-
[7133]	IKBKG	<---	MAP4K4	-0.56769	0.96826	+
[7134]	CCND1	--->	STAT3	0.17961	-0.084126	-
[7135]	SHH	<---	SPI1	-0.19988	0.16653	+
[7136]	EGR2	<---	EP300	-0.010456	0.016752	+
[7137]	CEACAM6	--->	FN1	0.013588	-0.14346	+
[7138]	MYL5	<---	PPP1CC	-0.50655	0.3817	+
[7139]	GRB2	--->	RASA1	0.087688	0.0087888	-
[7140]	CDK6	--->	EP300	0.013728	-0.006941	-
[7141]	CDC42	<---	EPHB2	0.014723	0.039661	+
[7142]	JAK3	--->	STAT4	0.56357	-1.1931	+

[7143]	CCL14	<---	CCR3	-0.35895	0.21658	-
[7144]	HGS	--->	SOS1	0.019457	0.0076189	+
[7145]	GNB5	--->	PIK3R1	0.028992	-0.072476	+
[7146]	ADRBK1	--->	HRH2	0.25327	-0.21301	+
[7147]	FYN	--->	IGHD	0.83863	-0.55361	+
[7148]	FOXO1	--->	G6PC3	0.4988	-0.56315	+
[7149]	GNAI2	--->	PLA2G4F	-0.10668	-0.13723	+
[7150]	RAE1	--->	SRSF2	0.31391	-0.17132	+
[7151]	CDKN1C	<---	SKP1	-0.032551	0.085167	+
[7152]	ACTA2	<---	MMP14	-0.29263	0.085904	+
[7153]	GYS1	<---	PPP1CA	-0.9036	0.53904	+
[7154]	RASAL3	--->	RRAS2	0.87061	-0.68738	+
[7155]	COL6A5	<---	PTK2	-0.046347	-0.038605	-
[7156]	SAG	--->	WNT4	0.23514	-0.34616	+
[7157]	CACNA2D1	<---	PRKX	-0.13162	0.13867	+
[7158]	CD274	--->	CD4	0.025256	-0.043726	+
[7159]	SFRP2	--->	WNT8B	1.0317	-1.1063	+
[7160]	COL5A2	--->	ITGB3	0.0024845	-0.035647	+
[7161]	GNB3	--->	SCN1A	0.036789	-0.042688	+
[7162]	KLRK1	<---	LCK	-0.45391	0.63648	+
[7163]	CD14	--->	VCAN	0.10978	-0.18833	+
[7164]	CCND1	<---	HDAC1	-0.16943	0.31701	-
[7165]	ITGA9	<---	LAMB1	-0.066621	-0.0090065	+
[7166]	DUSP21	--->	MAPK12	1.4184	-1.4202	+
[7167]	CD79B	<---	SH3KBP1	-0.16825	0.10242	-
[7168]	IQGAP1	<---	MAP2K1	-0.17128	0.26162	-
[7169]	MYL10	--->	PPP1R12C	0.27396	-0.40226	-
[7170]	MTOR	<---	PIK3R1	-0.088245	0.11889	+
[7171]	GRB2	--->	RRAS2	0.63892	0.0030067	+
[7172]	CACNB4	<---	PPP2R3B	-0.060248	0.10014	+
[7173]	CSNK1D	--->	PLK1	0.29896	-0.098976	-
[7174]	GNB4	--->	WNT8B	0.3343	-0.42584	-
[7175]	IL13RA2	--->	SOS2	0.041847	0.039674	+
[7176]	ARHGEF17	<---	RHOBTB2	-0.0089688	0.02783	-
[7177]	PTK2	<---	VCAM1	-0.075495	-0.0062259	+
[7178]	ESR1	<---	RAPGEF3	-0.12168	0.23411	-
[7179]	CD79A	<---	RPS6KB1	-0.30073	0.16282	-
[7180]	HSPA8	--->	STX1A	0.64767	-0.69131	+
[7181]	PDGFRA	--->	SHC2	0.00054908	-0.0812	+
[7182]	BLK	--->	EPHB2	0.35424	-0.26855	-
[7183]	CACNB1	<---	PPP2R3A	-0.060248	0.10014	+
[7184]	IL2RG	<---	JAK1	-0.1451	0.11865	-
[7185]	HCN3	--->	REST	0.27672	-0.20732	-
[7186]	ITCH	--->	RIPK2	0.38725	-0.39006	+
[7187]	ATF3	<---	RCBTB1	-0.16066	0.25678	-
[7188]	NCK1	--->	PAK2	0.73462	-0.72841	+
[7189]	FOXO1	<---	LMO4	-0.059274	0.13832	+
[7190]	COL1A1	--->	ITGA8	0.0052169	-0.090529	+
[7191]	KRAS	--->	MAPK10	1.2491	-0.20304	+
[7192]	CBL	--->	SPRY4	0.29155	-0.14969	-
[7193]	GRK7	--->	GRM2	0.80802	-0.62351	+
[7194]	IL6	<---	MAPK8	-0.26091	0.40675	+
[7195]	CCND2	<---	TCF7L2	-0.21775	0.37715	+
[7196]	IFNG	<---	RELA	-0.39685	0.0011643	+
[7197]	DDX5	--->	EGR1	0.18149	-0.10437	-
[7198]	CTNNA1	<---	PTPN1	-0.49082	0.37294	+
[7199]	DTX2	<---	NOTCH3	-0.36083	0.42037	+
[7200]	FRZB	--->	FZD4	0.73901	-1.3633	+
[7201]	CCR2	<---	JAK2	-0.12268	-0.039248	-
[7202]	TBC1D4	<---	YWHAH	0.06516	0.21759	-
[7203]	PIAS2	--->	STAT2	1.2392	-0.72187	+
[7204]	PRKD2	<---	RAP1B	-0.19045	0.8444	-
[7205]	ARHGAP28	<---	RAC2	-1.5245	1.016	-
[7206]	CDH3	<---	MMP9	-0.24168	-0.0076207	+
[7207]	LPAR2	--->	PTK2	-0.05393	-0.18014	+
[7208]	CHD9	--->	PPARGC1B	0.16299	-0.34613	+
[7209]	EPOR	<---	IL6	-0.17721	0.073187	+
[7210]	PRKCD	--->	SOCS3	0.5511	-0.54877	-
[7211]	RHOF	--->	SYDE1	1.016	-1.5245	-
[7212]	FCGR3A	<---	FYN	-1.4127	1.4215	+
[7213]	CREBBP	--->	POU4F1	0.010731	-0.015032	-
[7214]	ICAM1	<---	MST1	-0.20985	0.22401	+
[7215]	LAMC2	--->	MMP19	-0.0045452	-0.11357	-
[7216]	CACNA1S	<---	GNB3	-0.095409	0.10645	+
[7217]	ACTG1	<---	MMP12	-0.29263	0.085904	+
[7218]	MMP3	<---	NFKB1	-0.17897	0.14673	+

[7219]	PKN1	--->	RELA	0.27883	-0.39888	+
[7220]	ANK2	--->	ITPR2	0.17325	-0.10902	+
[7221]	ATF3	<---	PRKACB	-0.57905	1.0858	+
[7222]	ADRBK1	--->	HRH1	0.25327	-0.21301	+
[7223]	IL2RB	<---	MAPK14	-0.27385	0.44193	-
[7224]	H3F3A	<---	RELA	-0.74487	0.44178	-
[7225]	BTK	--->	PIK3R3	0.14102	-0.25088	-
[7226]	ITGB1	--->	VLDLR	0.070736	-0.055631	-
[7227]	ETV4	--->	HDAC2	0.37744	-0.31114	-
[7228]	ADCY6	<---	EDN1	-0.41432	0.41767	+
[7229]	PTPN1	<---	PTPN2	0.68663	0.70286	+
[7230]	MEF2C	--->	SERPINB3	-0.070722	-0.17681	+
[7231]	ACACA	<---	SP3	-0.32959	0.36494	+
[7232]	FOXA2	--->	JUN	0.38594	-0.42469	-
[7233]	CCNB2	<---	NCAPG	-0.40564	0.45419	-
[7234]	NR1H2	<---	SRF	-0.20741	0.044585	+
[7235]	BTK	--->	LCP2	0.071838	-0.13386	+
[7236]	IQGAP1	<---	KDR	-0.0055503	0.069031	+
[7237]	CSNK1E	--->	WNT8A	0.83912	-0.78694	-
[7238]	AURKA	--->	CPEB3	1.2133	-0.47902	+
[7239]	MAPK1	<--->	PRKACA	0.31014	0.31014	-
[7240]	MYO9A	<---	RHOC	-0.54674	0.38058	-
[7241]	GNAO1	--->	RHOA	1.2694	-0.93465	+
[7242]	MAPK7	--->	SPIC	0.40675	-0.26091	+
[7243]	CD44	<---	COL5A1	-0.18268	0.0085485	+
[7244]	NR3C1	--->	SMAD4	-0.024404	-0.1374	-
[7245]	FOXA1	--->	LEF1	0.26526	-0.086733	+
[7246]	CCL27	<---	CCR5	-0.35895	0.21658	-
[7247]	MAP2K1	--->	YWHAQ	0.079981	-0.071802	-
[7248]	GNA15	--->	WNT9B	0.42087	-0.47341	-
[7249]	FOXO1	<---	PTPN11	-0.095738	0.21579	+
[7250]	CSF2RB	<---	IL19	-0.10334	0.076998	+
[7251]	ITGA11	<---	LAMB4	-0.066621	-0.0090065	+
[7252]	CD3D	<---	MAP4K1	-0.45111	0.8376	-
[7253]	HNRNPUL1	--->	YY1	0.34937	-0.17756	-
[7254]	MYL6	<---	MYLK2	-0.29873	0.36004	+
[7255]	IL2RB	--->	SOCS3	-0.014037	-0.24629	-
[7256]	JUNB	<---	SMAD4	-0.1492	0.050351	-
[7257]	GP5	--->	PIK3CA	0.016708	-0.05131	+
[7258]	ADRB1	<---	GRK1	-0.28786	0.33635	+
[7259]	DAB2	--->	FOS	0.26685	-0.11924	-
[7260]	CDH1	<---	NCSTN	-0.40965	0.085277	+
[7261]	CD3G	--->	VAV2	0.06651	-0.018805	+
[7262]	PTPN2	--->	STAT6	0.27865	-0.24822	+
[7263]	CHRM4	--->	GNB3	0.69217	-0.57296	+
[7264]	IKBKG	<---	TRAF6	-0.63055	0.80437	+
[7265]	GNAI3	--->	GRM7	0.24033	-0.15623	-
[7266]	EGF	<---	PRKCB	-0.12658	0.10812	+
[7267]	LHX5	--->	TCF12	0.45723	-0.43175	-
[7268]	CPEB3	<---	PLK1	0.14821	0.4305	+
[7269]	EP300	--->	IGSF1	0.0013519	0.00070187	+
[7270]	FGR	--->	PIK3R1	0.28568	-0.27345	+
[7271]	MYL12B	--->	MYLK3	0.41687	-0.23601	-
[7272]	TCF7L2	--->	ZNF217	0.22714	-0.018993	+
[7273]	NCK2	--->	WAS	0.22109	-0.26051	+
[7274]	GADD45A	--->	PPARG	0.027313	-0.17877	-
[7275]	MAPK13	--->	RELA	0.92767	-0.99472	+
[7276]	CAV1	--->	TGFB1	0.17799	-0.37709	-
[7277]	FCER1G	--->	JUN	0.096401	-0.13523	+
[7278]	PTPN11	--->	TRBV12-3	0.37051	-0.30848	+
[7279]	PRKCB	--->	PRKD3	0.11731	-1.1868	+
[7280]	COMP	<---	ITGA2B	-0.021497	-0.014728	-
[7281]	PDGFB	--->	SPHK1	-0.012527	-0.0418	+
[7282]	EBF1	<---	NFKB1	-0.21206	0.22991	+
[7283]	BIRC7	<---	CASP6	-0.35112	0.045424	-
[7284]	MAPK11	<---	YES1	-1.0925	0.92013	+
[7285]	BIRC2	<---	TNFRSF1A	-0.076134	-0.053921	+
[7286]	CACNA2D2	<---	CAMK2G	-0.2316	0.31309	+
[7287]	CTCFL	<---	EP300	-0.0076085	0.01223	+
[7288]	CTGF	--->	TGFBR3	0.011097	-0.029113	+
[7289]	CAMK2B	--->	RASGRF2	0.24914	-0.57965	+
[7290]	FOS	<---	LPAR1	-0.36998	0.41745	+
[7291]	ACTB	<---	RAC1	-0.51168	0.52339	+
[7292]	ITGA7	<---	LAMC1	-0.041507	-0.0065623	+
[7293]	ATF3	<---	KAT5	-0.098546	0.15465	-
[7294]	GSK3B	--->	PSMD10	0.68643	-0.45084	-

[7295] ARHGEF12 ---> RHOT1 0.16719 -0.11927 +
[7296] IL6ST ---> SHC1 -0.010021 -0.02434 +
[7297] CTCF ---> ZNF587 -0.0037425 -0.012548 +
[7298] COL1A2 <--- PTK2 -0.11304 -0.10314 -
[7299] FOS <--- HSP90B1 -0.20445 0.22821 -
[7300] FOXH1 ---> SMAD2 0.17505 -0.30657 +
[7301] AURKA ---> CPEB2 0.73481 -0.29164 +
[7302] ARHGAP22 <--- RHOF -0.82056 0.64419 -
[7303] PAK1 <--- SRC -0.81947 0.81303 +
[7304] RDX <--- ROCK1 0.020811 0.044153 +
[7305] CALML5 ---> CAMK1 0.41687 -0.23601 +
[7306] GPR50 <--- GRK4 -0.28786 0.33635 +
[7307] PVR <--- TIGIT -0.070058 0.015031 -
[7308] ECT2 <--- RHOG -0.012974 0.047696 -
[7309] GRAP2 <---> GRB2 0.27757 0.27757 -
[7310] GNA15 <--- SRF -0.32808 0.37145 +
[7311] CAMK2D ---> GRIA3 0.94748 -0.64695 -
[7312] EPAS1 ---> KDR 0.0015197 -0.58026 +
[7313] DLC1 <--- RHOQ -0.50393 0.10338 -
[7314] SOCS1 ---> STAT2 0.27422 -0.74698 -
[7315] PIN1 <--- SFN -0.12941 -0.092158 -
[7316] CXCR6 <--- GRK5 -0.28786 0.33635 +
[7317] AKT1 ---> CREBBP 0.0040783 -0.012541 +
[7318] CHRM1 ---> KCNJ5 0.11779 -0.2522 -
[7319] IL10 ---> IL3RA -0.0077042 -0.055821 +
[7320] CTBP2 ---> OSR2 0.064439 -0.044029 +
[7321] IL10RA <--- SLA2 -0.077015 0.17317 -
[7322] CCNB1 <--- FZR1 -0.33759 0.44625 -
[7323] EP300 <--- EVL -8.9738e-005 0.0043594 -
[7324] EIF3B ---> MYC -0.02655 -0.13606 -
[7325] GARNL3 ---> RAP1A 0.57378 -0.48764 +
[7326] BDNF ---> CDH2 -0.078298 -0.23901 +
[7327] PRKAG1 ---> TSC1 0.26632 -0.42155 +
[7328] FGF6 ---> FLT4 0.071829 -0.16555 +
[7329] FES <--- KDR -1.0931 1.2161 +
[7330] CTBP2 ---> HOXD12 0.04296 -0.16727 +
[7331] CHRM4 <--- PRKCQ -0.2791 0.17482 -
[7332] CACNA1F <--- GNAO1 -0.17558 0.23994 +
[7333] NFKBIA <--- NGFR -0.43782 0.083755 +
[7334] FOXL1 ---> JUN 0.38594 -0.42469 -
[7335] JUN ---> NGF 0.50337 -0.45556 +
[7336] GNA11 ---> PLA2G4C 0.034723 -0.073024 +
[7337] CCNB3 <--- FZR1 -0.33759 0.44625 +
[7338] KRAS <--- LCP2 -1.3049 1.3361 +
[7339] MET <--- NRAS 0.033728 0.072771 -
[7340] PIAS3 ---> STAT5B 1.2392 -0.72187 +
[7341] ETS1 ---> LTF -0.016705 -0.055385 +
[7342] CCNB2 <--- CLASP2 -0.38312 0.41421 -
[7343] NFKBIB <--- PSMD1 -0.19284 0.32731 +
[7344] CAMK2D ---> GRIN2C 0.94748 -0.64695 -
[7345] PPARGC1B <--- SMARCD3 -0.34334 0.38529 +
[7346] MAPK3 <---> PRKACA 0.31014 0.31014 -
[7347] SREBF1 <--- ZNF460 -0.3584 0.51298 -
[7348] PIK3R3 <--- TXK -0.74223 0.79696 -
[7349] COL6A2 ---> ITGA11 -0.006085 -0.062785 +
[7350] KSR1 ---> YWHAB 0.11407 -0.07813 -
[7351] CNR1 ---> GNB5 1.1575 -0.92616 +
[7352] MYO9A <--- RAC2 -0.54674 0.38058 -
[7353] BIRC3 <--- TLR4 -0.14666 -0.014089 -
[7354] MYH6 <--- TNNT1 -0.020839 -0.0058045 -
[7355] CDK2 ---> SMAD3 0.88243 -0.89987 +
[7356] INPP5B <--- RHOV -0.92575 0.61511 -
[7357] FCGR1A ---> TNFRSF11A 0.10826 -0.16924 -
[7358] PPP1R1A ---> PPP3R1 0.068088 -0.035483 -
[7359] BUB3 <--- PSMD7 -0.26071 0.27742 +
[7360] LCK <--- NFKB1 -0.0047992 0.053603 -
[7361] INPP5B <--- RHOJ -0.92575 0.61511 -
[7362] EP300 ---> MEF2C 0.0067193 -0.017686 +
[7363] ARHGAP40 <--- RHOD -1.5245 1.016 -
[7364] MAPK14 <--- RAPGEF4 -0.093777 0.86463 +
[7365] FOXA2 ---> MYB 0.059212 -0.062176 +
[7366] ICAM3 <--- ITGAL -0.04811 0.017809 -
[7367] ARHGAP22 <--- RHOC -0.82056 0.64419 -
[7368] COPS5 ---> POU2F1 0.33631 -0.4066 -
[7369] ARHGAP20 <--- RHOC -1.4209 0.96682 -
[7370] PRKCZ <--- ROCK2 -0.027802 0.02441 +

[7371]	HNRNPR	--->	TBP	0.083732	-0.042563	-
[7372]	DAND5	--->	TNF	0.023118	-0.098515	+
[7373]	CCR7	--->	CXCL2	0.21658	-0.35895	-
[7374]	FIZ1	--->	YY1	-0.0051278	-0.028383	-
[7375]	CCNK	<---	POU2F2	-0.30187	0.35776	+
[7376]	IL4R	<---	IL6	-0.17721	0.073187	+
[7377]	E2F2	--->	MEF2C	0.097401	-0.26812	+
[7378]	ARHGAP25	<---	RHOBTB2	-0.73485	0.56175	-
[7379]	ESR1	<---	NT5E	-0.29434	0.055849	-
[7380]	CEBPA	--->	RXRA	-0.091263	-0.26204	-
[7381]	CDC25C	<---	PLK3	-0.32818	-0.022793	+
[7382]	CCL2	<---	STAT1	-0.32842	0.27027	+
[7383]	KHDRBS2	--->	PBX3	0.15908	-0.24396	-
[7384]	FGF9	<---	SOS1	0.067334	0.085583	-
[7385]	EXOC8	<---	MYH2	-0.093425	0.10372	+
[7386]	ADAM17	--->	NRG3	0.12479	-0.1789	+
[7387]	KCNMB3	<---	PRKACG	-0.31173	0.40189	+
[7388]	PLCG2	--->	PPP3CB	0.02904	-0.011667	+
[7389]	FGF20	--->	KDR	0.21774	-0.0015767	+
[7390]	E2F1	--->	SRSF3	0.32902	-0.26332	+
[7391]	GRIN2C	<---	GRM1	-0.36981	-0.05834	+
[7392]	ARHGAP17	<---	RHOH	-0.92575	0.61511	-
[7393]	AKT2	--->	STAT5B	1.212	-1.5407	-
[7394]	FOS	<---	PTGER4	-0.36998	0.41745	-
[7395]	CASP6	--->	CASP9	-0.039589	-0.88853	-
[7396]	ITGA11	<---	PTK2	-0.076638	-0.038051	-
[7397]	ANGPT1	--->	INSR	0.25337	-0.017626	+
[7398]	HOXD13	--->	TCF12	0.23888	-0.32236	-
[7399]	CACNA1S	<---	KCNMA1	-0.21987	-0.071398	+
[7400]	DLK1	<---	EP300	-0.0143	0.0094904	+
[7401]	COL22A1	<---	LEPRE1	-0.12547	0.043451	+
[7402]	GATA4	<---	THRB	-0.091347	-0.010433	+
[7403]	ATF4	<---	PPP1CA	-1.0855	0.69008	+
[7404]	LRP5	<---	WNT4	-0.28769	0.39899	+
[7405]	BDKRB2	--->	DAND5	0.08851	-0.17261	-
[7406]	EPS15	--->	JAG1	0.0037828	-0.043203	+
[7407]	CRKL	--->	PIK3CB	0.010761	-0.019381	+
[7408]	AXIN2	<---	YAP1	-0.18818	0.074581	+
[7409]	GADD45A	--->	PPARA	0.025734	-0.26567	-
[7410]	CAMK2D	--->	CREBBP	0.010341	-0.008238	+
[7411]	IL10RA	--->	JAK3	1.1771	-1.1167	+
[7412]	COL6A3	<---	ITGB5	-0.011216	-0.0071171	-
[7413]	IL6ST	<---	JAK1	-0.1451	0.11865	+
[7414]	CREBBP	--->	PFKFB2	-0.00068954	-0.0041449	+
[7415]	RAB5A	<---	SRC	0.16613	1.0122	+
[7416]	DUSP22	--->	EGR1	0.88546	-0.78754	-
[7417]	CD22	--->	POU2F2	0.053036	-0.026827	-
[7418]	KALRN	<---	RAC2	-0.021179	0.042607	-
[7419]	CCNG1	--->	TP53	0.064257	-0.038691	-
[7420]	CACNB2	<---	PRKX	-0.016941	0.18215	+
[7421]	MOB1A	<---	SAV1	-0.77343	0.57923	+
[7422]	MAPK12	--->	NFKB1	0.20986	-0.059594	+
[7423]	GRB2	<---	SHC4	0.018816	0.29675	+
[7424]	CSNK1A1L	--->	WNT4	0.83912	-0.78694	-
[7425]	ADRBK1	--->	GNB1	0.22583	-0.18333	+
[7426]	COL13A1	<---	PTK2B	-0.11304	-0.10314	+
[7427]	NR1H3	--->	SREBF1	0.27747	-0.4408	+
[7428]	GABBR1	<---	GHRL	-0.48927	0.44332	+
[7429]	AKT3	--->	CREB3L1	0.82132	-1.3257	+
[7430]	BCL2L11	<---	E2F1	-0.080085	0.021878	+
[7431]	PRKG2	--->	SLC8A3	0.33129	-0.36515	+
[7432]	ARHGAP23	<---	RHOJ	-0.46335	0.36352	-
[7433]	MAP2K3	<---	TAB3	-0.11835	0.1224	+
[7434]	CSF2RB	<---	PRKACA	-0.15198	0.17732	+
[7435]	GNAI3	<---	PIK3CA	-0.00083608	0.0020832	-
[7436]	PICK1	<---	PRKCB	-0.40252	0.33758	+
[7437]	CDC25A	<---	CDK2	-0.97911	0.26855	-
[7438]	GNA13	--->	RAC2	1.2694	-0.93465	+
[7439]	MAPK1	--->	YWHAG	0.079981	-0.071802	-
[7440]	CALML6	--->	NOS1	0.024644	-0.024577	+
[7441]	PPP2CA	<---	RAF1	-0.12371	0.0693	-
[7442]	PORCN	--->	WNT3	0.38949	-0.56891	+
[7443]	ITGAV	<---	PLCG1	-0.014214	0.01513	-
[7444]	CACNB3	<---	PPP1CC	-0.20297	0.15963	+
[7445]	FGR	--->	MEF2A	0.43969	-0.37646	-
[7446]	ESR2	<---	MAPK1	-0.30308	0.29076	+

[7447]	CTNND1	<---	MGAT3	-0.32795	0.17155	-
[7448]	ADRBK2	-->	CHRM1	0.25327	-0.21301	+
[7449]	ARRB2	-->	SLC9A3R1	0.010744	-0.001156	-
[7450]	COL6A2	<---	FYN	-0.28135	0.38676	-
[7451]	CSF2	-->	GHR	0.16017	-0.20921	+
[7452]	LRP1B	<---	PSEN1	-0.19592	0.23551	+
[7453]	CACNA1S	<---	CAMK2A	-0.26963	0.34735	+
[7454]	ABCC2	<---	EP300	-0.012129	0.0051045	+
[7455]	NRAS	-->	RALGDS	0.86481	-0.75112	+
[7456]	DUSP1	-->	JUN	0.42953	-0.3941	-
[7457]	PRKCB	-->	STAT3	0.53803	-0.49743	-
[7458]	CRK	<---	PRKCG	-0.20419	0.22188	+
[7459]	ARHGEF9	<---	RHOQ	0.091479	0.13323	-
[7460]	CTNNB1	<---	PCDHB14	-0.19204	0.11503	-
[7461]	AKT3	<---	TCL1A	-1.4472	1.0764	+
[7462]	ACVR2B	<---	INHBA	-0.85941	1.3161	+
[7463]	ARAP2	<---	RHOB	-0.48615	0.36733	-
[7464]	ATF3	<---	PRKACG	-0.57905	1.0858	+
[7465]	ADRBK1	-->	DRD2	0.25327	-0.21301	+
[7466]	ATP1A1	-->	PIK3CA	-0.0036365	-0.0057548	-
[7467]	CACNA1A	<---	GNAI1	-0.17558	0.23994	+
[7468]	CCR1	<---	GRK5	-0.28786	0.33635	+
[7469]	H2AFX	<---	MDC1	-0.32397	0.19879	-
[7470]	SMAD3	<---	VEGFA	-0.091161	-0.0042879	-
[7471]	PRKCA	-->	SLC9A1	0.32366	-0.41207	+
[7472]	BCAR1	-->	TLN1	0.013833	-0.016896	-
[7473]	CCND1	<---	CTCF	-0.096776	0.17982	+
[7474]	EPHB2	-->	PAK1	0.2976	-0.21305	+
[7475]	ITGB7	-->	MEF2C	0.072716	-0.18251	-
[7476]	SOS2	-->	SPRY4	1.2804	-0.54968	-
[7477]	RAC1	<---	SOS2	-0.33772	0.48674	+
[7478]	CNOT1	<---	EIF4A3	-0.16976	0.10767	-
[7479]	CBX5	<---	EP300	-0.0034415	-0.0018284	+
[7480]	EP300	<---	TRAF3	-0.024929	0.030746	-
[7481]	CAMK4	-->	CREB3L1	1.0858	-0.57905	+
[7482]	CCL21	<---	CXCR1	-0.35895	0.21658	-
[7483]	FCER1G	<---	PLA2G4A	-0.10331	-0.084011	-
[7484]	NANOG	<---	ZMYND8	-0.24396	0.15908	-
[7485]	DDR2	-->	EP300	0.011824	-0.021169	-
[7486]	CALML6	-->	HRAS	0.10721	-0.083432	+
[7487]	ERBB4	<---	PRKCQ	-0.059824	0.12383	-
[7488]	CRKL	-->	HDAC2	0.24347	-0.21691	-
[7489]	EGFR	-->	HRAS	0.17363	-0.11617	+
[7490]	ATF3	-->	SELE	0.097266	-0.04381	+
[7491]	PPARA	-->	VEGFA	-0.038004	-0.064123	+
[7492]	ARHGAP1	<---	RHOD	-0.92575	0.61511	-
[7493]	LRP5	<---	WNT1	-0.28769	0.39899	+
[7494]	HRAS	-->	MAP3K1	0.51592	-0.44241	+
[7495]	EP300	-->	SNIP1	0.00063033	-0.0057428	+
[7496]	BLK	-->	DOK1	0.54748	-0.63899	+
[7497]	HLA-DQA2	-->	PDCD1	0.12456	-0.36464	-
[7498]	CCNB2	<---	CPEB4	-0.17449	0.31504	-
[7499]	ANK1	-->	ITPR1	0.099591	-0.057766	+
[7500]	CASR	<---	FYN	-0.53074	0.72912	-
[7501]	BIRC3	<---	FAS	-0.076134	-0.053921	-
[7502]	CCL16	<---	CCR6	-0.35895	0.21658	-
[7503]	FGFR1	<---	PDGFC	-0.61313	0.56088	+
[7504]	EP300	-->	FGFR1	0.018309	-0.024282	+
[7505]	MAPK12	<---	PPP2R1B	-0.25679	0.26693	-
[7506]	CD247	-->	TCRB	-0.021439	-0.042516	-
[7507]	CAMK2D	-->	PDE4D	0.95928	-0.63646	+
[7508]	JUP	<---	TCF12	-0.15362	0.05043	+
[7509]	FRZB	-->	WNT6	1.0317	-1.1063	+
[7510]	FRZB	-->	WNT2	1.0317	-1.1063	+
[7511]	JUNB	<---	SRF	-0.78321	0.82243	+
[7512]	FCER1A	-->	USF1	0.72594	-0.75605	-
[7513]	HOXB9	-->	NCAM1	0.13977	-0.28242	+
[7514]	EP300	<---	NEK6	-0.006941	0.013728	-
[7515]	DVL1	<---	WNT7A	-0.54867	0.38842	+
[7516]	MAPK3	<--->	MAPK8	0.31014	0.31014	-
[7517]	G6PC3	<---	PRKAG1	-0.20283	0.012951	+
[7518]	ESR1	-->	STAT5B	0.27087	-0.22437	-
[7519]	COLEC12	<---	EP300	-0.0041449	-0.00068954	+
[7520]	HIPK2	<---	TAB2	-0.13728	0.23085	+
[7521]	CHD3	-->	TBP	0.10374	-0.18285	+
[7522]	CALM1	-->	CSNK1A1	0.41687	-0.23601	+

[7523]	CCL23	<---	CCR6	-0.35895	0.21658	-
[7524]	PIK3R5	<---	PRKCI	-0.1809	0.086594	-
[7525]	CSF1R	<---	IGF1	-0.12899	0.40501	+
[7526]	CCND1	<---	HIF1A	-0.01932	0.17481	+
[7527]	FGG	<---	PTK2	-0.1342	-0.09535	-
[7528]	CREB1	<---	SMAD6	-0.074842	0.046649	-
[7529]	CAMK2A	--->	SCN4B	0.31382	-0.23358	+
[7530]	MAPKAPK2	<---	TNF	0.048942	0.10494	+
[7531]	ACVR2B	--->	ZFYVE16	0.09539	0.036963	+
[7532]	GNB4	<---	PRKACA	-0.31609	0.54982	+
[7533]	GNAI2	--->	RAC1	1.2694	-0.93465	+
[7534]	CSNK1G3	--->	WNT10B	0.50785	-0.4784	-
[7535]	BAD	<---	PRKG2	-0.61323	0.68556	+
[7536]	AKT2	<---	PPP2R3B	-0.7767	0.80883	+
[7537]	CREB1	<---	RPS6KA2	-0.59606	0.7545	+
[7538]	RICTOR	--->	TRIB3	0.18178	-0.10456	-
[7539]	FES	--->	RRAS	0.17198	-0.078705	+
[7540]	CDC42	--->	PTK2	-0.084837	-0.14524	-
[7541]	ARPC1B	<---	WASF3	-0.098063	0.10019	+
[7542]	EPOR	<---	IRS2	0.018455	0.089853	-
[7543]	SIRT1	--->	SP1	0.28002	-0.15205	-
[7544]	PDPK1	--->	PIK3R1	0.36308	-0.29865	-
[7545]	MYO9A	<---	RHOT1	-0.25676	0.15261	-
[7546]	EGFR	--->	FGF11	0.25979	-0.22928	-
[7547]	GNB1	--->	NTRK1	0.065565	-0.23218	+
[7548]	CCR5	--->	JUN	0.2582	-0.3481	-
[7549]	IRAK4	<---	TAB3	-0.67287	0.46157	-
[7550]	GSK3B	<---	PARD6A	-0.24522	-0.16111	+
[7551]	HOXC11	--->	TCF12	0.23888	-0.32236	-
[7552]	GRK6	--->	HTR6	0.33635	-0.28786	+
[7553]	ATP2B1	<---	PRKX	-0.060388	0.088587	+
[7554]	CDC42	--->	MAPK10	1.2491	-0.20304	+
[7555]	GNAI3	<---	SSTR5	-1.1303	1.1631	+
[7556]	BCL2L1	<---	MAPK1	-0.9271	0.81238	+
[7557]	AGTR1	--->	GNA14	1.1631	-1.1303	+
[7558]	MAPK10	<---	RAPGEF4	-0.093777	0.86463	+
[7559]	FOXO1	<---	SGK1	-0.24953	0.090624	+
[7560]	LCP2	--->	PLCG1	0.036383	-0.0042314	-
[7561]	GNB3	<---	MAPK13	-0.28574	0.38931	-
[7562]	JUNB	<---	SMARCA4	-0.096099	0.042262	+
[7563]	PRKCQ	--->	RASGRP1	0.099115	-0.34557	+
[7564]	MAPK9	<---	RAC3	-0.20304	1.2491	+
[7565]	MAP4K2	--->	TNF	0.62058	-0.22921	-
[7566]	GNAO1	--->	PLA2G4D	-0.10668	-0.13723	+
[7567]	FLNA	--->	IKBKG	0.17255	-0.23509	+
[7568]	NOTCH4	<---	PCSK6	-0.033415	0.040257	+
[7569]	MBD3	--->	UBTF	0.013769	-0.0063504	+
[7570]	C1GALT1	--->	MUC3A	-0.023312	-0.02992	+
[7571]	RAPGEF5	<---	RRAS	-1.2366	1.4276	+
[7572]	LCK	<--->	SRC	0.15012	0.15012	-
[7573]	ADAMTS14	<---	COL3A1	-0.054632	-0.00053524	-
[7574]	ATP1A4	--->	PIK3R3	0.057037	-0.12143	-
[7575]	CALML3	--->	MYLK2	0.41687	-0.23601	+
[7576]	MAPK3	--->	PXN	0.091435	0.065619	+
[7577]	CTNNA2	--->	MMP15	0.38719	-0.55042	-
[7578]	ITGA5	<---	STAT1	-0.12952	0.09335	+
[7579]	MYO5A	<---	RAB10	-0.046655	0.010173	+
[7580]	GATA2	--->	SERPINE1	0.33364	-0.53505	+
[7581]	IL2RB	<---	SOCS5	-0.19297	0.16027	+
[7582]	ADCY7	<---	CAMK2G	-0.44346	0.58749	+
[7583]	MSC	<---	TAF1	-0.52432	0.43996	+
[7584]	NGFR	--->	PDGFD	0.039957	-0.32359	-
[7585]	GRK7	--->	GRM7	0.80802	-0.62351	+
[7586]	GRAP2	--->	HLA-DQA2	-0.052856	-0.066521	-
[7587]	CD3E	<---	ITK	-0.060744	0.20777	-
[7588]	CBX8	<---	MAPK11	-0.22919	0.35645	+
[7589]	GNA11	--->	PLA2G4F	-0.10668	-0.13723	+
[7590]	EGFR	--->	LPAR2	0.19514	-0.18069	-
[7591]	GATA1	<---	NR0B2	-0.059669	-0.015929	-
[7592]	MLYCD	<---	PRKAG3	-0.050797	0.49837	+
[7593]	NFIC	--->	PCK2	0.15176	-0.24847	+
[7594]	ARNT	<---	TIMP1	-0.16173	0.16165	-
[7595]	DIAPH2	<---	PFN1	-0.1109	0.062206	-
[7596]	MYC	<---	TCF7	-0.15083	0.096323	+
[7597]	IL23R	<---	TYK2	-0.21238	0.15548	-
[7598]	GABBR1	<---	NPY	-0.90742	0.79421	+

[7599] COL5A1 <--- PCOLCE2 -0.32363 0.21278 +
[7600] BHLHE40 <--- PAX5 -0.16866 0.11159 +
[7601] GRB2 ---> SOS2 1.1653 -1.204 +
[7602] ETS1 <--- MAPK14 -0.15935 0.24561 -
[7603] PRKCB ---> RRAS2 0.85615 -0.68511 +
[7604] PKN2 ---> RHOA 0.33774 -0.17533 -
[7605] E2F6 <--- ZNF292 -0.027971 -0.0048155 -
[7606] EP300 ---> HEY1 0.022073 -0.031481 +
[7607] BCL3 <--- F2R -0.43492 0.43724 -
[7608] CCL3 <--- NFKB1 -1.2272 1.0745 +
[7609] HSPA8 ---> SMAD3 0.3687 -0.49632 +
[7610] CSNK1A1L <---> GSK3B 0.31014 0.31014 -
[7611] ADRB2 <--- GRK4 -0.28786 0.33635 +
[7612] CCL20 <--- XCR1 -0.35895 0.21658 -
[7613] HSPA1L ---> MAPK10 1.2218 -0.90384 +
[7614] SMAD6 ---> TCF7L2 1.1041 -1.2424 -
[7615] GNB2 ---> GRM3 0.15376 -0.10866 -
[7616] PRKACA ---> SUFU 0.58123 -0.48787 +
[7617] NFKB1 ---> SREBF1 0.47748 -0.29534 +
[7618] IRS2 ---> PIK3R1 -0.026224 -0.083368 +
[7619] ERBB4 <--- PRKCZ -0.081967 0.11234 -
[7620] E2F1 <--- POLQ -0.062456 0.016613 -
[7621] IFNG <--- JAK2 -0.17897 -0.024165 +
[7622] MAP3K14 <---> MAPK3 0.31014 0.31014 -
[7623] IL10RA <--- LEP -0.087402 0.20113 +
[7624] BATF <--- IRF2 -0.16066 0.25678 -
[7625] ITGA3 ---> LRP8 0.19632 -0.1571 -
[7626] IGFBP5 ---> PAPPA2 -0.036622 -0.047516 -
[7627] GTF3A ---> MYC 0.017786 -0.08008 -
[7628] BCL3 <--- ZBTB37 -0.04047 0.37639 -
[7629] AKT2 <--- ERBB2 0.022566 0.027633 +
[7630] MAPK3 <--- TRAF6 -0.47605 1.1617 +
[7631] GNB1 <--- PXN -0.073778 0.087501 -
[7632] EP300 <--- LIMK1 -0.015907 0.012437 -
[7633] GAB1 <--- LPAR2 -0.45847 0.44741 +
[7634] CRK ---> RHOA 0.43868 -0.057401 +
[7635] CBL ---> GRB2 0.10181 -0.16173 +
[7636] DZIP1 <--- RBX1 -0.21772 0.15028 -
[7637] CD247 <--- CSK -0.27641 0.38494 -
[7638] DOK1 <--- PRKCG -0.43174 0.52481 -
[7639] PROC <--- THBD -0.045574 -0.0072359 +
[7640] FGFR2 ---> SOS2 0.041735 0.010746 +
[7641] MAX <--- NR1D2 -0.26846 0.16719 -
[7642] ADRBK1 ---> TBXA2R 0.25327 -0.21301 +
[7643] JUN <--- SPI1 -0.22115 0.16075 -
[7644] POU5F1 <--- SOX2 -0.14436 -0.10381 -
[7645] CBL ---> IL23R 0.17024 -0.087588 +
[7646] MAPK14 <--- TAB2 -0.13728 0.23085 -
[7647] GHR <--- JAK2 -0.23677 0.10182 +
[7648] GNAO1 <--- RGS11 -0.736 0.71839 +
[7649] CACNA1F <--- KCNMB2 -0.74334 0.88134 +
[7650] PAK1 <--- PTK2 -0.26373 0.25722 +
[7651] ELK4 <--- MAPK11 -0.26091 0.40675 +
[7652] FZD1 <--- WNT8A -0.78209 0.63734 +
[7653] FGD1 <--- RHOH -0.14219 0.16153 -
[7654] SOX2 <--- YAP1 -0.40605 0.073493 +
[7655] JUN <--- PTPRB -0.46533 0.35853 -
[7656] MYL6 <--- TPM2 -0.22759 0.17706 -
[7657] F2RL3 ---> PRSS3P2 0.11779 -0.2522 -
[7658] CD244 ---> CD48 0.22488 0.16759 -
[7659] IL13RA2 <--- SOCS2 -0.10066 -0.08965 +
[7660] ACTR1A <--- RAB8A -0.51168 0.52339 -
[7661] ERBB2 ---> PTPN11 0.78373 -0.22985 -
[7662] GNAI1 <--- RGS6 -0.736 0.71839 +
[7663] RXRA <--- ZMYND8 -0.26204 -0.091263 -
[7664] COL7A1 <--- SMAD3 -0.049849 -0.001679 +
[7665] LCK ---> PIK3CA 0.084876 -0.06481 +
[7666] ELK1 ---> PSEN1 -0.012403 -0.076922 +
[7667] ARNT ---> BCL2 0.10931 -0.1855 +
[7668] HRAS ---> IL2RG 0.059541 -0.068887 -
[7669] CRK ---> ITGA7 0.032574 -0.078547 -
[7670] MET ---> PGF 0.12914 -0.19534 -
[7671] ADIPOR1 <--- PRKAA1 -0.26091 0.40675 -
[7672] FZD10 <--- WNT9A -0.78209 0.63734 +
[7673] FGF23 <--- HRAS -0.15714 0.23194 -
[7674] NFKBIA <--- PRKACG -0.41321 0.60616 +

[7675]	BCR	<---	RHOBTB1	-0.1232	0.062954	-
[7676]	MYLK2	<--->	PRKACA	0.31014	0.31014	-
[7677]	ANKLE2	<---	PPP2R2A	-0.41198	0.28299	-
[7678]	COL1A1	--->	PPIB	0.022658	-0.10782	-
[7679]	ATF4	<---	TAF1	-0.16066	0.25678	+
[7680]	IL22RA2	--->	TYK2	0.48376	-0.37166	+
[7681]	CEBPB	--->	TNFAIP6	0.0085485	-0.18268	+
[7682]	BCL2	<---	HSPB1	-0.79485	0.53967	-
[7683]	CREB5	<---	PRKG1	-1.2233	1.1994	+
[7684]	IRS2	--->	PIK3R5	1.4647	-1.4621	+
[7685]	CRLF2	<---	CSF2	-0.21706	0.1815	+
[7686]	PDE4C	<---	PRKACB	-0.95055	1.4803	+
[7687]	SFRP4	--->	WNT1	1.0317	-1.1063	+
[7688]	DUSP22	--->	STAT3	0.4796	-0.36001	-
[7689]	CD79A	--->	POU2F1	-0.020898	-0.037712	-
[7690]	CDKN2D	<---	FOXO4	-0.51151	0.56498	+
[7691]	BLK	--->	S1PR1	0.55948	-0.3948	-
[7692]	DNM2	<---	KDR	-0.027912	0.16602	-
[7693]	ARHGEF3	<---	RHOB	-0.012974	0.047696	-
[7694]	PDHX	<---	PDK1	-0.5004	0.41454	+
[7695]	FLI1	--->	MMP9	-0.0045452	-0.11357	+
[7696]	COL8A2	<---	PTK2B	-0.06966	-0.063654	+
[7697]	GATA6	--->	TCF12	0.43996	-0.52432	-
[7698]	MAPK12	<---	NCF4	-0.26379	0.013605	+
[7699]	ARHGAP26	<---	RAC2	-0.65972	0.48549	-
[7700]	GNA11	--->	LRP5	0.60405	-0.41162	-
[7701]	FOXA1	--->	RXRA	0.04532	-0.3855	-
[7702]	F2	<---	IGF2	-0.09545	0.031228	-
[7703]	BTK	--->	CD19	0.49661	-0.41118	+
[7704]	SREBF1	<---	ZNF595	-0.20142	0.33079	-
[7705]	IGF1	<---	KIT	-0.035009	0.08054	-
[7706]	GNAI3	<---	MAPK14	-0.22953	0.50225	-
[7707]	CD86	--->	GAB2	0.15962	-0.047056	+
[7708]	IGKV1-5	<---	SH3KBP1	-0.27558	0.17068	-
[7709]	MAX	<---	RBBP4	-0.23234	0.23228	-
[7710]	IFNLR1	<---	IL20	-0.20115	0.093836	+
[7711]	GNRHR	<---	GRK7	-0.28786	0.33635	+
[7712]	CCKBR	--->	EGR1	0.44069	-0.36137	-
[7713]	MED16	--->	TGFB1	0.14451	-0.29031	-
[7714]	ATP1A2	<---	PRKX	-0.09775	0.14787	+
[7715]	ACTR2	<---	NCK2	-0.46159	0.41422	+
[7716]	E2F7	--->	SP1	0.23995	-0.10098	-
[7717]	DUSP22	<---	NFKB1	-0.099925	0.094057	+
[7718]	ADRA2A	<---	GRK6	-0.28786	0.33635	+
[7719]	CHRM5	<---	GRK7	-0.28786	0.33635	+
[7720]	GRK4	--->	SSTR5	0.33635	-0.28786	+
[7721]	CEBPB	<---	FOXF1	-0.37846	0.32839	-
[7722]	BCL3	<---	TRAF4	-0.29396	0.2939	-
[7723]	PRKAG1	--->	SLC2A4	0.34513	-0.50137	+
[7724]	ENG	--->	TGFB1	0.10765	-0.2955	-
[7725]	BCL3	<---	CBX3	-0.33847	0.35903	-
[7726]	CSNK1D	--->	DVL1	0.52194	-0.62577	-
[7727]	HDAC3	<---	SMAD1	-0.21285	0.15191	-
[7728]	GNAI1	<---	SUCNR1	-1.1303	1.1631	+
[7729]	FGF17	<---	MET	-0.26028	0.20868	-
[7730]	MYLK3	<---	PAK6	-1.2674	1.5523	+
[7731]	PDHA1	<---	PDK4	-1.3804	1.1701	+
[7732]	KCNB1	<---	PRKX	-0.44517	0.61419	+
[7733]	CCND3	--->	STAT6	0.10784	-0.052125	-
[7734]	RHOG	<---	SOS2	-0.33772	0.48674	+
[7735]	CXCR4	--->	GNAI2	1.1631	-1.1303	+
[7736]	CREBBP	--->	NOS3	-0.0020202	-0.0032913	+
[7737]	GNAZ	<---	PLCG1	-0.0052535	0.030885	-
[7738]	AP2S1	<---	RPS6KA6	-0.40283	0.20087	+
[7739]	IRS1	<---	MAPK9	-0.20734	0.74413	+
[7740]	GAB3	<---	PRKCZ	-0.15343	0.32961	-
[7741]	CHRM3	<---	GRK1	-0.28786	0.33635	+
[7742]	PORCN	--->	WNT8A	0.38949	-0.56891	+
[7743]	MAPK14	<---	SRC	-1.0925	0.92013	+
[7744]	IGF1R	<---	PLCB2	-0.0065784	0.032363	-
[7745]	GNAO1	--->	PLA2G4A	-0.10668	-0.13723	+
[7746]	HNRNPR	--->	MYC	-0.02655	-0.13606	-
[7747]	EP300	--->	HNRNPR	0.021187	-0.018539	+
[7748]	AKT3	--->	SHC3	0.32159	-0.42272	-
[7749]	GHR	<---	LEP	-0.17721	0.073187	+
[7750]	RHOH	--->	TIAM2	0.20928	-0.13672	-

[7751]	GATA3	<---	IFNG	-0.22733	0.024975	-
[7752]	CCR5	<---	STAT1	-0.29222	0.22857	+
[7753]	APOA1	--->	LRP2	0.20747	-0.15861	-
[7754]	MAP2K1	<---	MAP3K2	-1.347	1.3429	-
[7755]	MAPK8	<---	TRAF5	-0.47605	1.1617	+
[7756]	FGFR1	<---	PRKCB	-0.22639	0.23769	-
[7757]	ATF1	<---	COPA	-0.011771	0.12118	-
[7758]	LEPR	<---	PTPN1	-0.83695	0.69521	+
[7759]	CTNNB1	<---	MMP14	-0.26058	0.11034	+
[7760]	ARR3	<---	CSNK1E	-0.15935	0.24561	-
[7761]	PPP2R1A	<---	SRC	-0.12863	0.23397	+
[7762]	LYN	--->	TRPV6	0.44853	-0.34759	+
[7763]	FOXA1	--->	HDAC9	0.74871	-0.64672	+
[7764]	HSPA8	--->	MAPK8	1.2218	-0.90384	+
[7765]	AKT2	--->	STAT3	1.212	-1.5407	-
[7766]	CCR5	<---	GRK7	-0.28786	0.33635	+
[7767]	FCGR1A	<---	SRC	-0.94853	1.1589	+
[7768]	CDK4	--->	E2F2	0.45782	-0.30098	+
[7769]	FXYD2	--->	PIK3R1	0.049293	-0.12511	-
[7770]	CREBBP	--->	ENO2	-0.00068954	-0.0041449	+
[7771]	IL6	<---	MAPK9	-0.26091	0.40675	+
[7772]	PRKG1	--->	SLC8A1	0.32653	-0.52118	+
[7773]	RHOA	<---	SOS2	-0.33772	0.48674	+
[7774]	MAP2K1	--->	PIK3R1	0.39782	-0.48655	-
[7775]	GNAI2	<---	RGS8	-1.2531	1.2063	+
[7776]	APOA1	--->	SAR1B	-0.077489	-0.081642	-
[7777]	EPHB2	--->	PIK3R1	0.06451	-0.10365	+
[7778]	ACTB	<---	EZR	-0.17417	0.099698	+
[7779]	EGF	<---	SPRY2	-0.13445	0.1443	-
[7780]	JUN	--->	NOTCH1	0.26934	-0.19565	-
[7781]	ARHGAP20	<---	RHOT2	-0.65643	0.37566	-
[7782]	CSNK2A2	<---	PPP2CA	-0.15489	0.41077	-
[7783]	VAV2	<---	YES1	-0.065501	0.072747	+
[7784]	DLG4	--->	GRIA1	0.19945	-0.095793	-
[7785]	ITGAM	<---	PTK2B	-0.084093	-0.024145	+
[7786]	RAC2	--->	SRGAP1	0.39011	-0.42732	-
[7787]	CREBBP	--->	SNIP1	0.00063033	-0.0057428	+
[7788]	GHRL	--->	SSTR2	0.61472	-0.75338	+
[7789]	IL23R	--->	SOCS7	-0.0096134	-0.15048	-
[7790]	CCR4	--->	GNAI3	1.1631	-1.1303	+
[7791]	CSNK1G2	--->	FZD3	0.33941	-0.31414	-
[7792]	EPHB2	<---	HCK	-0.26855	0.35424	-
[7793]	BCR	<---	SHC3	-0.093275	-0.046077	-
[7794]	PRKAR1B	--->	PXN	0.1254	-0.15337	+
[7795]	ARRB1	--->	WNT9B	0.23514	-0.34616	-
[7796]	CCL3	<---	CCR1	-0.35895	0.21658	-
[7797]	RPS6KA6	--->	SH3GL2	0.066268	-0.19855	+
[7798]	CCL5	<---	CX3CR1	-0.35895	0.21658	-
[7799]	DAND5	--->	E2F1	0.087771	-0.11222	+
[7800]	CTCF	<---	ZNF511	-0.0054925	0.007473	-
[7801]	ITGB2	--->	PIK3CD	-0.0036365	-0.0057548	+
[7802]	ITGB6	--->	THBS2	0.093347	-0.066778	-
[7803]	CSF1R	<---	VEGFC	-0.30912	0.56567	+
[7804]	ACTA1	<---	MMP27	-0.29263	0.085904	+
[7805]	ARRB2	<---	CXCR4	-0.15406	0.070342	-
[7806]	ANGPT2	--->	CSF1R	0.33967	-0.087672	+
[7807]	PPP1R1A	--->	PPP3CA	1.3031	-1.1767	-
[7808]	E2F1	<---	NFATC2	-0.50477	0.23668	-
[7809]	INSM2	--->	REST	0.24138	-0.13774	-
[7810]	IL20RB	<---	SOCS1	-0.40298	0.060066	+
[7811]	HDAC2	<---	RELA	-0.73296	0.60593	+
[7812]	GNB3	--->	WNT11	0.19155	-0.26814	-
[7813]	PRKD1	--->	STAT3	0.84558	-0.79252	-
[7814]	ARHGEF12	--->	CDC42	0.092701	0.0073174	+
[7815]	CACNA1S	<---	GNAI2	-0.17558	0.23994	+
[7816]	CTNNB1	<---	PVRL2	-0.12018	0.064	+
[7817]	IL5RA	<---	IL6	-0.12599	0.11381	+
[7818]	ADCY8	<---	CALML6	-0.31614	0.34641	+
[7819]	COL6A1	<---	ITGB7	-0.025688	-0.014544	-
[7820]	JUN	<---	STAT1	-0.21779	0.18014	+
[7821]	CSNK1E	<--->	CSNK2A1	0.31014	0.31014	-
[7822]	ICAM1	<---	ITGAM	-0.077252	0.026053	+
[7823]	MAPK10	--->	PLA2G4B	0.18871	-0.84715	+
[7824]	HDAC2	<---	NOD2	-0.1019	-0.027831	-
[7825]	CNOT4	<---	EIF4A3	-0.20283	0.23551	-
[7826]	ARRB1	--->	CTNNB1	0.20637	-0.27358	+

[7827]	SHB	<---	SRC	-0.74223	0.79696	+
[7828]	LRP5L	<---	PSEN2	-0.16338	0.1035	+
[7829]	JAK1	--->	SGMS1	0.087709	-0.22013	+
[7830]	ITGB4	--->	THBS2	0.028041	-0.023304	-
[7831]	FZD10	<---	WNT2	-0.78209	0.63734	+
[7832]	CTNNB1	<---	PTPN11	-0.43489	0.4441	-
[7833]	FGF23	--->	INSR	0.25346	-0.0044024	+
[7834]	CD8B	--->	JUN	0.14534	-0.21548	+
[7835]	E2F1	--->	HNRNPA3	0.32902	-0.26332	+
[7836]	BAIAP2	--->	WASF2	0.047829	-0.0078627	+
[7837]	IQGAP3	--->	RAC2	0.12856	-0.076889	-
[7838]	IGKV1-5	--->	SOS1	0.093491	0.04514	+
[7839]	GAB2	<---	PRKCG	-0.50087	0.66104	-
[7840]	JUN	<---	NR0B2	-0.25371	0.17193	-
[7841]	JUND	<---	STAT1	-0.21779	0.18014	-
[7842]	PRKCG	--->	TBXA2R	0.27696	-0.37622	+
[7843]	TXK	--->	VAV2	0.072747	-0.065501	+
[7844]	GNA11	<---	RGS4	-1.2531	1.2063	+
[7845]	CD40	<---	MAP3K14	-0.57721	0.64173	-
[7846]	HEY1	<---	NR5A1	-0.42437	0.26687	-
[7847]	FRS2	<---	PRKCB	-0.48425	0.40952	-
[7848]	ARHGAP39	<---	RHOB	-0.82986	0.43536	-
[7849]	AR	<---	HNRNPA1	-0.07732	0.04307	+
[7850]	CTNNB1	<---	PCDHB4	-0.19204	0.11503	-
[7851]	EP300	<---	FOXO4	-0.016032	0.013464	-
[7852]	FGF6	<---	MAPK1	-0.47959	0.71493	+
[7853]	GRB2	<---	INSRR	0.015827	0.095474	+
[7854]	IGF1R	<---	MAP2K2	0.083131	0.1759	-
[7855]	CCNA2	<---	PTP4A1	-0.80981	0.84694	+
[7856]	GH2	--->	IL13RA1	0.094523	-0.18506	+
[7857]	CDK1	--->	SEH1L	0.54982	-0.31609	+
[7858]	C3	--->	C3AR1	0.0034647	-0.16296	+
[7859]	CCL11	<---	CCR4	-0.35895	0.21658	-
[7860]	RAC1	--->	SPATA13	0.13323	0.091479	-
[7861]	IL2RG	<---	PRKCE	-0.17474	0.13376	-
[7862]	EXOC2	--->	MYO1C	0.21178	-0.030695	-
[7863]	CTNNB1	<---	RAB5A	-0.60792	0.46891	+
[7864]	ARHGEF12	--->	RHOJ	0.092701	0.0073174	+
[7865]	ETS1	--->	ITGA5	-0.0090065	-0.066621	+
[7866]	DLG4	--->	RHOQ	0.15149	0.1492	+
[7867]	CX3CR1	--->	PF4V1	0.21658	-0.35895	-
[7868]	RAP1A	<---	RAPGEF2	-1.3789	0.84353	+
[7869]	ERBB3	<---	HBEGF	-0.82917	1.3656	+
[7870]	CXCR6	--->	PF4	0.21658	-0.35895	-
[7871]	EPHA4	--->	NGEF	0.054317	-0.13142	+
[7872]	MCF2	<---	RHOF	-0.14219	0.16153	-
[7873]	CDC42	<---	NTN1	-0.077076	0.078093	+
[7874]	SETDB1	<---	ZNF350	0.012459	0.049815	-
[7875]	EDN1	--->	YY1	0.28002	-0.15205	-
[7876]	CNTFR	--->	SOCS2	0.17881	-0.34174	-
[7877]	GNAI1	--->	GRM2	0.24033	-0.15623	-
[7878]	IRF2	--->	PAX5	0.056756	-0.091513	-
[7879]	CCL22	<---	CCR9	-0.35895	0.21658	-
[7880]	HCK	--->	RGS1	0.53308	-0.35367	+
[7881]	JUN	<---	TIMP1	-0.22115	0.16075	-
[7882]	MLST8	<---	RPS6KB1	-0.4112	0.24568	-
[7883]	GPC5	--->	LRP2	0.34864	-0.2711	-
[7884]	ITGA8	--->	THBS1	0.16838	-0.067658	-
[7885]	EPHA2	<---	PLCB3	-0.0092396	-0.0084643	-
[7886]	CASP3	<---	CFLAR	-1.4016	0.0509	+
[7887]	ACTR2	<---	NCK1	-0.46159	0.41422	+
[7888]	ERBB2IP	--->	NOD2	-0.21381	-0.22896	+
[7889]	GNAL	<---	OR1J1	-0.011498	0.05262	+
[7890]	MAPK14	--->	SHC1	0.70518	-0.81557	-
[7891]	ARHGAP11B	<---	RHOQ	-1.5245	1.016	-
[7892]	MAPK3	<---	RAC1	-0.20304	1.2491	-
[7893]	CTCF	--->	TCF12	0.16851	-0.093907	+
[7894]	JUP	<---	PAX5	-0.12197	0.079206	+
[7895]	IL1B	--->	MEF2A	0.12176	-0.23984	-
[7896]	HIST1H3A	<---	KAT7	-0.32305	0.21329	+
[7897]	SRSF2	<---	TAF1	-0.31439	0.36909	+
[7898]	ARR3	--->	LRP5L	0.082322	-0.11868	-
[7899]	ACVR1B	<---	INHBC	-0.38171	0.71751	+
[7900]	LAMA3	--->	PLG	-0.01358	-0.017471	-
[7901]	GNA11	--->	PLCB3	0.0066317	-0.024514	+
[7902]	SFRP4	--->	WNT5A	1.0317	-1.1063	+

[7903]	BLK	--->	TDGF1	0.70613	-0.49464	-
[7904]	PRAME	<---	SMARCA4	-0.067524	-0.005393	+
[7905]	CAMK2B	--->	PDE4C	0.95928	-0.63646	+
[7906]	CASP2	<---	NGF	-0.22167	0.036398	+
[7907]	CTNND1	<---	FMN1	-0.33421	0.17619	-
[7908]	DVL3	<---	GSK3B	-0.62577	0.52194	-
[7909]	MYL12B	--->	MYLK2	0.41687	-0.23601	-
[7910]	IL12RB1	--->	SOCS2	-0.16125	-0.20399	-
[7911]	CTCF	<---	LRRC32	-0.13107	0.12168	-
[7912]	ATM	<---	TAOK2	-0.21939	0.2553	-
[7913]	IL18R1	--->	STAT4	0.21956	-0.11052	+
[7914]	DUSP4	--->	MAPK7	1.353	-1.036	+
[7915]	ARRB1	--->	WNT7B	0.23514	-0.34616	+
[7916]	MYL3	<---	TPM2	-0.22759	0.17706	-
[7917]	CHRM2	<---	PRKCH	-0.37622	0.27696	-
[7918]	CDK1	--->	HCFC1	0.57211	-0.50839	+
[7919]	CDC42	--->	FGD3	0.16153	-0.14219	-
[7920]	CNTFR	--->	SOCS4	-0.0038582	-0.019894	-
[7921]	CEBPE	--->	LTF	-0.025729	-0.089501	+
[7922]	IFNG	<---	JAK1	-0.17897	-0.024165	-
[7923]	GNAI3	<---	SSTR1	-1.1303	1.1631	+
[7924]	ALYREF	<---	SEH1L	-0.17132	0.31391	+
[7925]	EGFL7	--->	GATA2	0.012572	-0.10742	-
[7926]	IGF2	--->	PAPPA	0.067808	-0.1345	-
[7927]	AAAS	--->	SRSF3	0.31391	-0.17132	+
[7928]	ARHGEF6	<---	GNB1	-0.07454	0.082981	+
[7929]	EBI3	<---	ETS1	-0.17721	0.073187	+
[7930]	DAPP1	<---	RAC1	0.14154	0.33406	-
[7931]	MYL5	--->	MYLK	-0.0010631	-0.0039627	-
[7932]	GRIA2	<---	PRKACB	-1.006	1.5014	+
[7933]	AGT	<---	CEBPB	-0.32562	0.20126	+
[7934]	PSMD14	<---	RELA	-0.28748	0.022968	-
[7935]	CSF1R	<---	FGF2	-0.64493	0.77791	+
[7936]	INSR	<---	PGF	-0.033666	0.27735	+
[7937]	JAK1	--->	PIK3CG	0.020536	-0.05391	+
[7938]	MAPK3	--->	S1PR1	0.19743	-0.10024	-
[7939]	CTCF	--->	WNT7A	0.5734	-0.94937	+
[7940]	HDAC2	<---	ITGA5	-0.11172	0.046601	-
[7941]	F2RL2	--->	GNA14	1.1631	-1.1303	+
[7942]	GNB5	<---	PRKX	-0.31609	0.54982	+
[7943]	ANTXR1	--->	TP53	0.13842	-0.13107	-
[7944]	IL6ST	<---	MAPK1	-0.19355	0.22164	-
[7945]	IL2RG	<---	MAP2K2	-0.19355	0.22164	-
[7946]	CDK1	--->	ERCC6L	0.2465	-0.19082	+
[7947]	CCL18	<---	CCR8	-0.35895	0.21658	-
[7948]	GRK6	--->	SSTR1	0.33635	-0.28786	+
[7949]	SFRP2	--->	WNT16	1.0317	-1.1063	+
[7950]	GSK3B	--->	JUN	0.66358	-0.47491	+
[7951]	EPO	--->	IL10RA	0.16814	-0.075265	+
[7952]	CTGF	<---	TEAD3	-0.084232	0.058657	+
[7953]	CCND3	<---	SOS1	0.0039278	0.094104	+
[7954]	CBL	--->	EPOR	0.18092	-0.10684	-
[7955]	EML4	<---	PIK3CD	-0.012551	0.008521	-
[7956]	HCFC1	<---	POU2F1	-0.088399	-0.078596	+
[7957]	CXCR1	--->	PF4	0.21658	-0.35895	-
[7958]	NCK1	--->	TEK	0.0070645	-0.13697	-
[7959]	ATF1	<---	ATM	0.00468	0.01209	-
[7960]	TICAM2	--->	UBE2D1	0.28095	-0.27162	-
[7961]	SHC1	<---	SPRY2	-0.15769	0.090914	-
[7962]	CASP2	<---	TNF	-0.51125	0.2901	+
[7963]	GNA14	--->	WNT10B	0.42087	-0.47341	-
[7964]	ATAD2	--->	CTCF	0.083205	-0.0091154	-
[7965]	CACNA1C	<---	PRKACB	-0.38391	0.51195	+
[7966]	CBLB	--->	CSF2RB	0.11324	-0.075178	+
[7967]	PRKCH	--->	SRC	0.23398	-0.027179	+
[7968]	AKT1	--->	PDE3B	0.55876	-0.51658	+
[7969]	NKX2-5	<---	SMAD6	-0.71172	0.56478	+
[7970]	IL3RA	<---	PRKACA	-0.27385	0.44193	+
[7971]	FOS	--->	JUND	1.1275	-0.11171	-
[7972]	FYN	--->	ITGA1	0.084352	-0.098236	-
[7973]	CTBP1	--->	SMAD1	-0.063311	-0.074632	+
[7974]	FRZB	--->	WNT10B	1.0317	-1.1063	+
[7975]	FGR	--->	SHC3	0.48646	-0.49397	+
[7976]	MAPK3	--->	PLA2G6	0.41524	-0.27455	+
[7977]	GHR	<---	PTPN11	-0.26697	0.28395	+
[7978]	FZD10	<---	GNA11	-0.22143	0.24435	-

[7979]	CREB1	<---	FN1	0.0017488	0.0045442	-
[7980]	GNB3	--->	WNT9A	0.19155	-0.26814	-
[7981]	COL26A1	<---	LEPRE1	-0.18291	0.065305	+
[7982]	ARRB2	<---	CCR5	-0.15406	0.070342	-
[7983]	ADCY4	<---	GNAZ	-0.63317	1.0384	-
[7984]	FYN	--->	NCK2	0.23434	0.063181	+
[7985]	RPS12	--->	RPS13	0.24487	-0.45553	+
[7986]	ARL8B	--->	CTCF	0.050128	-0.039673	-
[7987]	ANGPT2	--->	FGFR2	0.086464	-0.29412	+
[7988]	E2F4	<---	MAPK1	-0.30098	0.45782	-
[7989]	NFATC1	<---	PPP3CC	-0.50133	-0.28042	+
[7990]	CD79B	<---	FYN	-0.29182	0.38571	+
[7991]	EP300	<---	FYN	-0.033546	0.038927	-
[7992]	POU5F1	--->	SHOX	-0.056605	-0.39339	+
[7993]	COL5A3	--->	TLL1	0.13754	-0.19386	-
[7994]	RAB14	<---	TBC1D4	-0.77234	1.3656	+
[7995]	JUN	<---	TNFRSF1A	-0.3239	0.14599	-
[7996]	ARHGAP11A	<---	RHOT1	-0.58534	0.26947	-
[7997]	RAB3A	<---	RIMS2	-0.29356	0.45861	+
[7998]	DAXX	<---	TGFBR2	-0.40607	0.17166	+
[7999]	NFKB1	--->	VIPR1	0.14195	-0.14364	+
[8000]	ACTB	<---	THRA	-0.43574	0.25595	+
[8001]	MAPK3	--->	NCOR2	0.24411	-0.046655	+
[8002]	DAPP1	<---	PPP3CB	-0.35962	0.52503	+
[8003]	RHOD	<---	VAV1	0.1025	0.10902	+
[8004]	ACTA1	<---	CASP3	-1.0006	0.61851	+
[8005]	MYC	<---	PPRC1	-0.13606	-0.02655	-
[8006]	CCR10	--->	CXCL10	0.21658	-0.35895	-
[8007]	EIF2AK2	<---	MAP4K4	-0.9701	1.3976	-
[8008]	COMP	<---	ITGA5	-0.021497	-0.014728	-
[8009]	CALM1	--->	HRAS	0.12065	-0.091216	+
[8010]	MEPCE	--->	ZBTB7A	0.45618	-0.37402	-
[8011]	ATF4	--->	TBP	0.17697	-0.11068	-
[8012]	BTK	--->	JUN	0.1475	-0.08525	+
[8013]	DVL2	<---	PRICKLE1	-0.65965	0.52955	-
[8014]	ABLIM1	--->	RAC2	0.4782	-0.31163	-
[8015]	MAP2K3	--->	PKN1	0.42367	-0.30613	-
[8016]	CD8A	--->	IL12B	0.097075	-0.28101	+
[8017]	CD3E	--->	TCRB	-0.033535	-0.068284	-
[8018]	ABI2	--->	ARPC1B	0.3044	-0.30536	+
[8019]	ITGB4	<---	LAMC1	-0.017112	0.0034424	+
[8020]	CDKN2D	<---	DAND5	-0.32572	0.38806	+
[8021]	RAC3	--->	RALGDS	1.4276	-1.2366	-
[8022]	IRAK2	--->	MAP2K7	0.73444	-0.20293	+
[8023]	CCL13	<---	CCR7	-0.35895	0.21658	-
[8024]	EPHA2	--->	FGF12	-0.0069714	-0.050497	-
[8025]	SOS1	<---	TREM2	0.04514	0.093491	+
[8026]	BMP4	<---	SMAD9	-0.24379	0.060804	-
[8027]	CCL2	<---	CCR3	-0.35895	0.21658	-
[8028]	IL16	<---	IRS1	0.048997	0.16466	-
[8029]	CHRM3	--->	GNA14	1.1631	-1.1303	+
[8030]	LTA	<---	NFKB1	-0.39904	0.30264	+
[8031]	GRM5	--->	TRPC1	0.066059	-0.097475	+
[8032]	ADRB3	<---	GRK1	-0.28786	0.33635	+
[8033]	ITGA9	<---	VWF	-0.05402	0.0049709	+
[8034]	RELA	--->	TNFRSF1A	-0.2175	-0.21911	-
[8035]	SMAD5	<---	SMURF1	-0.50288	0.48017	+
[8036]	RPS6KA3	--->	TSC2	0.54	-0.69281	+
[8037]	POU4F1	--->	TCF12	0.23888	-0.32236	-
[8038]	NRAS	--->	RASSF1	0.26182	-0.1115	+
[8039]	C2	--->	FCN2	-0.011958	-0.022757	-
[8040]	ITGB3	<---	RAP1A	-0.01596	0.026206	-
[8041]	CHRM2	<---	GHRL	-0.75338	0.61472	+
[8042]	IGF1R	--->	NTRK1	-0.0020789	-0.10876	+
[8043]	ERCC2	--->	HIST1H3A	0.69916	-0.72586	-
[8044]	CCNB2	<---	GADD45A	-0.67465	0.65424	+
[8045]	JAM3	--->	PARD3	0.15016	-0.13856	-
[8046]	BIRC2	<---	CASP3	-0.16002	-0.0075246	-
[8047]	COL18A1	--->	TAL1	0.27871	-0.35012	-
[8048]	PLA2G4E	<---	PLCG2	-0.0045292	0.028429	+
[8049]	IL1A	<---	NFKB1	-0.1256	0.12086	+
[8050]	CSF2	--->	LEPR	0.16017	-0.20921	+
[8051]	MTF2	<---	TCF7L2	0.059308	0.086299	+
[8052]	MEF2A	<---	SPIB	-0.29909	0.1217	-
[8053]	CEBPB	--->	IL16	0.019526	-9.2943e-005	+
[8054]	ATF1	--->	INHBA	0.063524	-0.11958	+

[8055]	AKT3	--->	STAT3	1.212	-1.5407	-
[8056]	AGAP2	<---	NTRK1	-0.053506	0.02613	+
[8057]	IGF1R	<---	PDGFB	-0.021519	0.16712	+
[8058]	CREBBP	--->	MYC	0.0030066	-0.01706	-
[8059]	GATA2	--->	SERPINB2	0.33364	-0.53505	+
[8060]	SNX17	<---	TAF1	-0.10515	0.0016017	+
[8061]	DGCR8	--->	TAF1	0.11522	-0.33924	-
[8062]	CREB3L1	<---	NPY	-0.81635	0.84205	-
[8063]	SMAD3	--->	ZNF263	0.73379	-0.80018	-
[8064]	MAFK	<---	ZNF280B	0.027424	0.042605	-
[8065]	EGFR	--->	FGF2	0.25979	-0.22928	-
[8066]	EGR1	<---	VEGFB	-0.17627	0.30406	-
[8067]	JAK2	--->	PIK3R3	0.59646	-1.0692	+
[8068]	FOSL1	--->	JUNB	1.1275	-0.11171	+
[8069]	EDN1	--->	JUN	0.16075	-0.22115	-
[8070]	RAF1	--->	WNK2	0.60653	-0.96022	-
[8071]	DVL3	<---	WNT8B	-0.54867	0.38842	+
[8072]	FCER1A	--->	LAT	0.19986	-0.23925	+
[8073]	CACNA1C	<---	KCNMA1	-0.21987	-0.071398	+
[8074]	FUS	<---	TAF1	-0.051163	0.08491	+
[8075]	PLD2	<---	PPAP2A	-0.019741	0.020601	-
[8076]	NFATC1	--->	TNF	0.93112	-1.1394	+
[8077]	CCR4	--->	PF4V1	0.21658	-0.35895	-
[8078]	CASP3	--->	MAPT	0.17116	-0.52748	+
[8079]	CREM	<---	PRKACG	-0.35231	0.65748	+
[8080]	DLG1	--->	GRIK1	0.19945	-0.095793	-
[8081]	DAND5	--->	TERT	0.021163	-0.044865	+
[8082]	SFN	--->	SLC2A4	0.64882	-0.85287	+
[8083]	LCP2	--->	SOS1	0.34189	-0.34402	-
[8084]	C3	--->	CEBPD	0.21278	-0.32363	-
[8085]	CAMK2D	--->	SCN5A	0.43397	-0.32993	+
[8086]	BRCA1	--->	DDB2	0.45211	-0.38063	+
[8087]	PROC	<---	SERPINA5	-0.054939	-0.01346	+
[8088]	CTNNB1	<---	MAGI1	0.024596	0.054591	-
[8089]	CDK5	<---	EPHA6	-0.4085	0.3522	+
[8090]	CREB3	<---	RPS6KA5	-0.98092	1.2458	+
[8091]	ITGB1	<---	LAMB2	-0.011824	0.005707	+
[8092]	FGF1	--->	KIT	0.015796	-0.016964	+
[8093]	ADCY2	<---	GNAZ	-0.63317	1.0384	-
[8094]	EGR1	<---	IGF2	-0.23904	0.38445	-
[8095]	AAAS	<---	SNRPD3	-0.10631	0.074498	-
[8096]	CD3G	<---	MAP4K1	-0.45111	0.8376	-
[8097]	PTPRC	--->	SPRY2	0.17004	-0.22654	-
[8098]	PLCG2	<---	PRKCI	-0.027756	0.040945	-
[8099]	CREBBP	--->	SREBF1	0.024715	-0.02899	+
[8100]	ARHGEF7	<---	PTK2	-0.082679	0.0077858	+
[8101]	PRKAA2	--->	TSC1	1.3112	-1.0821	+
[8102]	CTNNA1	--->	PVRL1	0.16037	-0.1555	-
[8103]	CCR9	--->	CXCL6	0.21658	-0.35895	-
[8104]	ADCY4	<---	EDNRA	-0.62364	0.57833	+
[8105]	MYL6B	--->	MYLK	-0.0010631	-0.0039627	-
[8106]	GNAI1	<---	LYN	-0.44236	0.67161	-
[8107]	FZD4	<---	GNB3	-0.12321	0.10912	-
[8108]	FZD1	<---	GNA15	-0.22143	0.24435	-
[8109]	IL20	--->	IL23R	-0.0057724	-0.034956	+
[8110]	ADRBK2	--->	HTR2A	0.25327	-0.21301	+
[8111]	NRAS	<---	RASGRP3	-0.17952	0.14935	+
[8112]	MMP9	<---	VEGFA	-0.14114	0.022679	-
[8113]	BTRC	--->	FZR1	0.43842	-0.060711	+
[8114]	CCR10	<---	GRK7	-0.28786	0.33635	+
[8115]	ADRBK2	--->	SSTR3	0.25327	-0.21301	+
[8116]	ARHGAP11B	<---	RHOT2	-0.58534	0.26947	-
[8117]	TIRAP	--->	VDR	0.1459	-0.25971	+
[8118]	CHUK	<---	NTRK1	-0.15785	0.11688	+
[8119]	ESR2	--->	TERT	-0.010433	-0.091347	+
[8120]	CHD6	--->	TCF12	0.18336	-0.31584	-
[8121]	CCL24	<---	CCR1	-0.35895	0.21658	-
[8122]	CCL8	<---	CCR3	-0.35895	0.21658	-
[8123]	CDC42	--->	MAPK1	1.2491	-0.20304	+
[8124]	CD22	--->	CD79A	0.02681	-0.011184	-
[8125]	PXN	<---	SRC	-0.41502	0.50966	-
[8126]	PIK3R3	<---	SRC	-0.74223	0.79696	+
[8127]	MAPK3	--->	YWHAG	0.079981	-0.071802	-
[8128]	GNAO1	<---	PRKACB	-0.22953	0.50225	+
[8129]	FOS	<---	SCN8A	0.016551	0.10247	-
[8130]	GDF1	--->	LEFTY1	-0.050062	-0.38124	-

[8131]	CTCF	--->	HDAC7	0.14551	-0.015482	+
[8132]	BAK1	<---	CAPN1	-0.20179	0.14315	-
[8133]	CSF2	--->	PRLR	0.16017	-0.20921	+
[8134]	MAPK14	<---	PTPN7	-1.4016	1.2548	+
[8135]	JAM3	<---	PRKCH	-0.20759	0.21462	+
[8136]	BATF3	<---	NFATC1	-0.58249	0.48528	+
[8137]	CDH2	<---	FER	-0.19504	-0.010297	-
[8138]	GNAI3	--->	PLCB1	0.052816	-0.052991	+
[8139]	GATA2	--->	ZIM2	0.28002	-0.15205	+
[8140]	EIF4E	<---	EIF4H	-0.10366	0.16575	+
[8141]	ARRB2	--->	DNM1	0.027228	-0.026147	-
[8142]	ATF3	<---	PRKX	-0.57905	1.0858	+
[8143]	ATM	<---	CHEK2	-0.13429	0.15179	-
[8144]	CD8A	<---	LAT	-0.12445	7.9204e-005	-
[8145]	AGTR1	--->	GNA13	1.1631	-1.1303	+
[8146]	CEBPA	--->	MED26	0.21329	-0.32305	-
[8147]	MAX	<---	PIN1	-0.30368	0.21825	+
[8148]	ANGPT1	--->	PIK3R1	0.02813	-0.11732	+
[8149]	CSH1	--->	IL2RG	0.056231	-0.11334	+
[8150]	TICAM2	--->	UBE2D2	0.28095	-0.27162	-
[8151]	MAPK8	--->	PLA2G4F	0.18871	-0.84715	+
[8152]	LAT	<---	LCK	-0.91192	0.96251	+
[8153]	FGFR2	<---	PGF	-0.38721	0.2003	+
[8154]	CCND1	<---	YAP1	-0.60411	0.57561	+
[8155]	CXXC4	--->	DVL2	0.89837	-0.93441	+
[8156]	FGF10	<---	HRAS	-0.15714	0.23194	-
[8157]	CDK4	--->	CDKN1B	0.71039	-0.49354	-
[8158]	ITGB2	<---	MST1R	-0.084778	0.057057	+
[8159]	AKT1	--->	FOXA2	0.30488	-0.37709	+
[8160]	EPO	--->	IL22RA1	0.16814	-0.075265	+
[8161]	PIK3AP1	--->	PIK3CD	-0.0050831	-0.010841	-
[8162]	LRP6	<---	PI4K2A	-0.43822	0.58537	-
[8163]	EPHA2	<---	VEGFB	-0.055219	-0.01111	+
[8164]	CALML3	<---	PPP1CA	-0.26348	0.069904	+
[8165]	EP300	<---	SFRP1	-0.017349	0.017548	-
[8166]	CHRM1	<---	PRKCE	-0.37622	0.27696	-
[8167]	EGFR	--->	ZFYVE28	0.24184	-0.21654	-
[8168]	CD40	<---	MAP3K1	-0.33577	0.46329	-
[8169]	AKT3	--->	RORC	0.7641	-0.82222	+
[8170]	CTNNB1	<---	WNT1	-0.187	0.023012	+
[8171]	FOXO3	<---	MAPK14	-0.22085	0.2499	+
[8172]	ATF3	--->	ZNF280A	0.16224	0.14761	+
[8173]	ROBO1	<---	SLIT2	-0.13235	0.034182	+
[8174]	FOXF1	--->	JUNB	0.38594	-0.42469	-
[8175]	CAMK2D	--->	MEF2B	0.27897	-0.28169	+
[8176]	ACTR2	<---	HTT	-0.72444	0.64593	+
[8177]	CACNB2	<---	PRKACB	-0.016941	0.18215	+
[8178]	ACAP2	<---	ARF6	-0.17356	0.099163	-
[8179]	GLI3	--->	WNT8A	0.86373	-1.47	+
[8180]	STAT3	<---	ZNF595	-0.25129	0.41972	-
[8181]	CSF2RB	<---	IL26	-0.10334	0.076998	+
[8182]	CHUK	<---	TNFRSF1A	-0.16508	0.036616	+
[8183]	ARHGAP1	<---	RHOJ	-0.92575	0.61511	-
[8184]	CBL	--->	EGF	0.082889	-0.053289	-
[8185]	ARHGEF7	<---	RHOBTB1	-0.027982	0.032452	-
[8186]	IL13RA2	<---	JAK2	-0.23741	0.19744	-
[8187]	NUP43	--->	SRSF9	0.31391	-0.17132	+
[8188]	NTRK3	<---	SHC2	-0.080387	-0.019524	-
[8189]	NR2C2	--->	SNRPB2	0.23448	-0.31347	+
[8190]	CD247	--->	IL12RB1	0.073187	-0.17721	+
[8191]	ARHGAP19	<---	RHOC	-1.5245	1.016	-
[8192]	MAPK11	<---	RNF2	0.044099	0.35592	-
[8193]	CBL	--->	PIK3CB	0.035594	-0.021832	+
[8194]	BCAR1	--->	RAC2	0.21814	-0.11421	+
[8195]	PML	<---	TGFBR1	-0.056757	0.24476	+
[8196]	MAPK1	--->	SRF	0.35865	-0.36564	-
[8197]	AKT1	--->	YWHAQ	0.043615	-0.1933	+
[8198]	CDKN1B	<---	RHOA	-0.047556	0.26115	-
[8199]	MYO9A	<---	RHOD	-0.54674	0.38058	-
[8200]	ID1	<---	JUN	-0.13126	0.071708	+
[8201]	LIMK2	--->	PAX5	0.11972	-0.12307	-
[8202]	DAND5	<---	EGFR	-0.19878	0.23531	-
[8203]	PPP4C	--->	YWHAQ	-0.03225	-0.31369	-
[8204]	NF1	<---	PRKCI	-0.031603	-0.017252	-
[8205]	RAF1	--->	YWHAZ	-0.029863	-0.22693	+
[8206]	COL4A1	<---	GP6	-0.27286	0.25603	-

[8207]	AP2S1	<---	RPS6KA1	-0.40283	0.20087	+
[8208]	GNB2	<---	PRKCG	-0.42438	0.35558	+
[8209]	GNAI2	<---	PRKACG	-0.22953	0.50225	+
[8210]	PLCG1	<---	PRKCA	-0.0064763	0.024762	-
[8211]	PIK3CA	<---	TRAF3	-0.073566	0.090661	+
[8212]	ITGA11	--->	THBS3	-0.014728	-0.021497	-
[8213]	CEBPB	<---	IGSF1	0.013897	0.070242	-
[8214]	BRCA1	--->	PFN1	0.055278	-0.10867	+
[8215]	LAMA2	--->	SV2A	0.099369	-0.11816	+
[8216]	MYBPC1	<---	MYL3	-0.12286	0.036048	+
[8217]	RBL1	<---	SMAD4	-0.16383	0.070647	+
[8218]	DKK4	--->	WNT6	0.54164	-0.69755	+
[8219]	SETDB1	--->	TLE4	0.098812	-0.086309	+
[8220]	APC	<---	ARR3	-0.012802	0.0026103	+
[8221]	PRKACA	--->	RARG	0.29076	-0.30308	+
[8222]	BLK	--->	MLLT4	0.12276	-0.082867	+
[8223]	NUP37	--->	SRSF3	0.31391	-0.17132	+
[8224]	IL2RB	--->	SHC1	0.12767	-0.16166	+
[8225]	HOXC13	--->	USF2	0.23888	-0.32236	-
[8226]	ATF3	<---	PKN2	-0.45705	0.56244	-
[8227]	CAMK2G	--->	CREB3	0.72002	-0.41113	+
[8228]	BRCA1	--->	HEY1	0.64354	-0.74756	-
[8229]	GNGT2	<---	KCNJ5	-0.059562	-0.05732	-
[8230]	CNOT6L	--->	EIF4A1	0.034994	-0.11982	+
[8231]	JUNB	<---	ZBTB7A	-0.016444	0.05624	+
[8232]	ATP1A4	<---	PRKG1	-0.31721	0.19695	+
[8233]	LDLR	<---	SREBF2	-0.15862	0.2452	+
[8234]	FZD6	<---	WNT10A	-0.30406	0.25758	+
[8235]	NFKB1	<---	TLR7	-1.1204	1.0762	-
[8236]	ATP1B1	<---	PRKACG	-0.26091	0.40675	+
[8237]	CD44	<---	LAMB3	-0.1119	0.0040853	+
[8238]	ACTG1	<---	ARPC1A	-0.37291	0.34929	+
[8239]	AMICA1	--->	ITGA4	-0.022382	-0.10459	-
[8240]	CD3G	--->	IL12RB2	0.056231	-0.11334	+
[8241]	FZD6	<---	GNB2	-0.08719	0.093693	-
[8242]	GLI2	<---	SKP1	-0.059737	0.13985	+
[8243]	PPP2R3B	--->	RB1	0.19962	-0.30995	+
[8244]	PLCG1	--->	RAC1	0.2022	-0.071607	+
[8245]	LEPR	<---	TYK2	-0.37792	0.17946	-
[8246]	ACTB	<---	MYL5	-0.10988	0.043745	+
[8247]	CCND2	<---	CTNNB1	-0.034577	0.033828	+
[8248]	ADORA3	<---	GRK7	-0.40072	0.46898	+
[8249]	MAPK13	<---	RAPGEF3	-0.093777	0.86463	+
[8250]	CSF2RB	<---	THPO	-0.098871	0.064861	+
[8251]	GRIN2D	<---	PPP1CA	-0.67094	0.67498	+
[8252]	ATF6	<---	PSEN1	-0.13019	0.22274	+
[8253]	MAPK1	--->	RXRB	0.17534	-0.43654	+
[8254]	HDAC2	<---	ZNF280A	0.01176	0.26827	-
[8255]	FRZB	--->	FZD5	0.73901	-1.3633	+
[8256]	PLCG1	<---	PRKCI	-0.027756	0.040945	-
[8257]	NR1D1	--->	TBL1XR1	0.042199	-0.11165	+
[8258]	GPC6	--->	HS3ST6	-0.029045	-0.085212	-
[8259]	DLL4	<---	RBPJ	-0.0051693	0.0086145	-
[8260]	CD14	<---	SIGIRR	-0.13061	0.28424	+
[8261]	PDPK1	--->	RXRG	0.22813	-0.26832	-
[8262]	C1QB	--->	C1R	0.048831	-0.073083	-
[8263]	BRCA1	--->	HNF4A	0.064658	-0.18678	-
[8264]	FGD1	<---	RHOBTB2	-0.087343	0.096875	-
[8265]	FGFR2	<---	PIK3CG	-0.067527	0.022286	-
[8266]	AKT1	--->	USP8	0.23053	-0.26534	+
[8267]	AKT2	--->	MAP3K8	1.3215	-1.2112	+
[8268]	PIK3R5	<---	SYK	-0.53439	0.3563	+
[8269]	FRS2	<---	PRKCQ	-0.45721	0.39339	-
[8270]	CCNB1	<---	NUP153	-0.56222	0.62034	-
[8271]	RHOT1	<---	VAV1	0.032698	0.092967	+
[8272]	MAPK11	--->	PPARGC1A	1.2133	-0.47902	+
[8273]	FURIN	--->	TGFB1	0.10685	-0.22982	+
[8274]	BDNF	<---	CTNNA1	-1.0893	1.0687	-
[8275]	TGFB3	--->	TGFBR1	0.71751	-0.38171	+
[8276]	PRKAR2B	<---	PTPN11	-0.091434	0.14342	-
[8277]	KPNA7	<---	NUP37	-0.19114	0.12101	+
[8278]	ERBB4	--->	PIK3R2	0.54799	-0.28951	+
[8279]	FOS	--->	NOTCH1	0.44959	-0.32095	-
[8280]	ATP1A2	--->	PIK3R3	0.057037	-0.12143	-
[8281]	NOD2	<---	TRAF6	-0.21418	0.19175	+
[8282]	CD3G	<---	ITK	-0.060744	0.20777	-

[8283]	CARD6	<---	EP300	-0.025788	0.022074	+
[8284]	CFLAR	--->	DAXX	0.12709	-0.6543	-
[8285]	EP300	--->	SIX1	0.010731	-0.015032	+
[8286]	RAC3	--->	SYDE1	1.016	-1.5245	-
[8287]	GNB1	<---	HTR5A	-0.57296	0.69217	+
[8288]	ARRB1	--->	NKD1	0.3133	-0.37274	-
[8289]	ARHGEF2	<---	RHOH	-0.093742	0.10079	-
[8290]	AKT1	<---	HSP90B1	-0.32359	0.3448	+
[8291]	JUN	<---	PPP1R15B	-0.3439	0.30421	-
[8292]	EDNRB	--->	GNAQ	1.1631	-1.1303	+
[8293]	MYL2	<---	PPP1CB	-1.0727	0.85575	+
[8294]	IKBKB	<---	PRKCZ	-0.7724	0.12674	+
[8295]	EGF	<---	ITGA1	-0.023315	-0.0017728	+
[8296]	KAT2B	--->	RBPJL	0.055633	-0.056694	+
[8297]	ATP2B2	<---	PRKG1	-0.1935	0.11836	+
[8298]	EIF2AK1	--->	NFKBIA	0.60616	-0.41321	+
[8299]	GLI1	<---	RBBP4	-0.13732	0.49296	+
[8300]	MYO5A	<---	RAB14	-0.046655	0.010173	+
[8301]	JUND	<---	ZNF131	0.012343	0.031777	-
[8302]	CD3E	<---	PTPRC	-0.21196	0.14725	+
[8303]	BATF	--->	GFI1	0.096524	0.050503	+
[8304]	MARK2	--->	MIB1	0.03018	-0.024979	+
[8305]	CDKN1C	<---	SKP2	-0.12595	-0.019483	+
[8306]	ARF6	<---	NCK1	-0.32063	0.21909	+
[8307]	FGD4	<---	RHOU	-0.12937	0.14242	-
[8308]	AR	<---	ZMIZ1	-0.17578	0.11288	+
[8309]	GRIA3	<---	PRKACG	-1.006	1.5014	+
[8310]	DTX3L	--->	NOTCH4	0.024069	-0.0013567	-
[8311]	PSMD1	<---	RNF146	-0.13767	0.077085	-
[8312]	ARHGAP39	<---	RAC3	-0.82986	0.43536	-
[8313]	AKT3	--->	STAT6	0.73399	-0.93559	-
[8314]	IL1B	<---	MAPK12	-0.078488	0.15109	+
[8315]	GNA11	<---	PTAFR	-1.1303	1.1631	+
[8316]	ADCY8	<---	CAMK2B	-0.44346	0.58749	+
[8317]	FOXO1	<---	MAPK3	-0.22085	0.2499	+
[8318]	PLXNA2	<---	PLXNA4	0.061725	0.091133	-
[8319]	PHKG1	<--->	PRKACB	0.31014	0.31014	-
[8320]	MAPK13	<---	RAPGEF4	-0.093777	0.86463	+
[8321]	PDGFRA	--->	PLCG2	0.0095253	-0.021627	+
[8322]	RBL2	<---	TFDP2	-0.064179	0.022122	+
[8323]	PIK3CA	<---	TRAF2	-0.045672	0.034445	+
[8324]	EPHB2	--->	RHOA	0.039661	0.014723	+
[8325]	SERPINE1	<---	TP53	-0.034493	0.0044493	+
[8326]	CDK7	--->	CDKN1A	0.71039	-0.49354	+
[8327]	CDK1	--->	RB1	0.24655	-0.30848	+
[8328]	HDAC2	<---	ZNF175	0.01176	0.26827	-
[8329]	DEPDC1B	<---	RHOBTB1	-0.56996	0.38724	-
[8330]	CUL5	<---	HSP90AA1	-0.044374	-0.0058158	-
[8331]	PIK3R1	--->	STAT3	0.12624	-0.31035	+
[8332]	MAPK10	--->	PRKCQ	-0.20738	-0.21837	-
[8333]	PTK2	<---	SHC3	-0.31988	-0.26189	-
[8334]	AKT1	--->	SOS1	0.052556	-0.15964	-
[8335]	RHOT1	<---	STARD13	-0.14803	-0.10028	+
[8336]	ADCY6	<---	CAMK4	-0.63235	0.8673	+
[8337]	ARHGAP20	<---	RHOBTB1	-0.8629	0.5853	-
[8338]	LYN	--->	PLA2G4A	0.30785	-0.29234	+
[8339]	LAMA5	<---	PTK2	-0.04335	-0.039708	-
[8340]	BTK	--->	SOS1	0.088396	-0.30914	+
[8341]	BMPR2	--->	SMAD9	0.6082	-0.49302	+
[8342]	SLC25A13	<---	USF1	-0.13384	0.077786	+
[8343]	CCR7	--->	GNAI2	1.1631	-1.1303	+
[8344]	DUSP1	--->	MAP3K4	1.353	-1.036	+
[8345]	PBX3	<---	SRCAP	-0.14817	0.063916	-
[8346]	ARHGAP5	<---	RHOH	-0.078715	0.62868	-
[8347]	AMH	<---	SMAD1	-0.094696	-0.017685	-
[8348]	MMP16	--->	POTEKP	0.085904	-0.29263	+
[8349]	NEK9	--->	NUP153	0.17193	-0.084754	+
[8350]	ELF3	<---	EP300	-0.0036136	-0.0015178	+
[8351]	FCGR2C	--->	TNFRSF11A	0.29671	-0.35844	-
[8352]	CCR2	--->	POU2F1	0.31975	-0.45327	-
[8353]	EBF1	<---	PPP1R13L	-0.043406	-0.0011381	-
[8354]	PLK1	--->	TP53	0.076172	0.030749	-
[8355]	GNAI1	<--->	GNAI2	0.064764	0.064764	-
[8356]	PTEN	<---	ROCK1	-0.0084311	0.10581	+
[8357]	BTK	--->	MAPK8	0.36334	-0.30428	+
[8358]	IL1B	--->	SQSTM1	0.0058145	-0.068238	+

[8359]	CD86	<---	PIK3CG	-0.020719	0.010906	-
[8360]	CAMK2D	--->	GRIN1	0.94748	-0.64695	-
[8361]	FYN	--->	S1PR1	0.55948	-0.3948	-
[8362]	EGR1	<---	GSC	0.049909	0.078944	-
[8363]	BIRC8	<---	CASP7	-0.35112	0.045424	-
[8364]	CRKL	--->	RAPGEF1	0.14908	-0.16086	+
[8365]	JUND	<---	MEF2C	-0.26942	0.21547	-
[8366]	FZD8	<---	WNT7B	-0.78209	0.63734	+
[8367]	FOS	<---	TGFBR2	-0.42175	0.39759	-
[8368]	AJUBA	--->	LATS1	0.8544	-0.694	+
[8369]	E2F6	<---	MEPCE	-0.54574	0.48209	-
[8370]	AVPR1A	--->	GNA13	1.1631	-1.1303	-
[8371]	CCR7	<---	GRK5	-0.28786	0.33635	+
[8372]	GNAI1	--->	PIK3C2G	-0.0031928	-0.019337	+
[8373]	ACTN3	--->	MYL4	0.10182	0.0089705	-
[8374]	ESR2	--->	GNAQ	0.042535	-0.12976	+
[8375]	EP300	--->	F2	-0.00093868	-0.0049207	+
[8376]	GNAI1	<---	PLCB4	-0.023623	0.0010472	-
[8377]	FGF7	<---	MET	-0.26028	0.20868	-
[8378]	MEF2C	<---	NCOR2	-0.39774	0.25165	+
[8379]	F2R	--->	PIK3CG	0.029282	-0.062766	+
[8380]	SETDB1	<---	SUV39H2	0.0061002	0.0076026	-
[8381]	CTBP2	--->	PRDM6	0.035043	-0.0053799	+
[8382]	FGF10	<---	NGFR	-0.47081	0.24157	-
[8383]	F11R	<---	PRKCQ	-0.2345	0.21916	+
[8384]	BCL2	<---	CASP10	-0.82979	0.44654	-
[8385]	SFRP5	--->	WNT3	1.0317	-1.1063	+
[8386]	MAPK3	--->	RB1	0.24655	-0.30848	+
[8387]	CREB1	<---	GH2	-0.098546	0.15465	-
[8388]	PTPN6	--->	USF2	0.66434	-0.59645	-
[8389]	CHRM2	<---	PRKCQ	-0.2791	0.17482	-
[8390]	PLCG1	--->	PPP3CB	0.02904	-0.011667	+
[8391]	ATP1A1	<---	PRKACA	-0.09775	0.14787	+
[8392]	GNAZ	<---	PXN	-0.057204	0.12598	-
[8393]	RAP1GAP2	<---	SIPA1	0.043612	0.33502	-
[8394]	CDK4	--->	MYC	0.75491	-0.9874	-
[8395]	MAP2K4	<---	TRAF2	-0.60861	1.3226	+
[8396]	FGD2	<---	RHOC	-0.12937	0.14242	-
[8397]	IL2RG	<---	RHOA	-0.11176	0.099979	-
[8398]	PSMD7	--->	SPOP	0.21764	-0.25607	+
[8399]	INHA	--->	INHBB	-0.050062	-0.38124	-
[8400]	DEPTOR	<---	RRAGD	-0.18677	0.15952	+
[8401]	ADCY1	<---	GNAI3	-0.63317	1.0384	-
[8402]	GNAI3	--->	NRAS	0.76886	-0.56799	+
[8403]	TCF7L2	--->	ZNRF3	0.069361	-0.050176	+
[8404]	CREB3L2	--->	JUN	1.1275	-0.11171	+
[8405]	CACNA1C	--->	GRIN2C	0.15899	-0.088421	-
[8406]	AGRN	--->	LRP1	0.0034141	-0.024585	-
[8407]	BCL6	<---	FOS	0.088432	0.097302	-
[8408]	FHL2	--->	FOS	0.77356	-0.54833	-
[8409]	COL2A1	--->	ITGB8	0.011556	-0.092098	+
[8410]	APEX1	--->	MYC	0.073333	-0.28761	-
[8411]	ARHGAP4	<---	RHOA	-0.42732	0.39011	-
[8412]	PPP1R1A	--->	PPP2R3C	0.068088	-0.035483	-
[8413]	CTSG	<---	IGF2	-0.12987	0.06155	-
[8414]	PRKCQ	--->	STAT5B	0.82627	-0.74285	-
[8415]	FOXA1	<---	MDM2	-0.10304	0.008815	-
[8416]	COL6A1	<---	GP6	-0.18003	0.15913	-
[8417]	ATF4	<--->	FOS	0.36709	0.36709	-
[8418]	CSF1	--->	FGFR2	0.44353	-0.53511	+
[8419]	CDK1	--->	HCFC2	0.57211	-0.50839	+
[8420]	ATP1A3	<---	PRKG2	-0.22258	0.19984	+
[8421]	MMP1	--->	RXRA	-0.1147	-0.15215	-
[8422]	CASP7	--->	ITGA6	0.065703	-0.2863	+
[8423]	MAX	<---	PRMT1	-0.59982	0.55318	-
[8424]	KCNJ11	<---	PRKACA	-0.26091	0.40675	+
[8425]	FERMT3	<---	ITGB3	-0.053549	0.042797	-
[8426]	CACNA1C	<---	HTR2C	-0.36046	0.31836	+
[8427]	MYH4	<---	RHOQ	-0.01596	0.026206	+
[8428]	ADCY6	<---	CAMK2G	-0.44346	0.58749	+
[8429]	CHN2	<---	RHOF	-0.4978	0.36255	-
[8430]	CCNB1	<---	USF1	-0.13151	0.18771	+
[8431]	STAT5A	<---	TNFSF11	-0.32637	0.4382	-
[8432]	ABCB1	--->	JUN	-0.00086664	-0.064647	-
[8433]	JAK2	--->	SHC2	0.36756	-0.71996	+
[8434]	NR5A1	--->	USF1	0.27747	-0.4408	-

[8435]	HNRNPA2B1	<---	SMARCA4	-0.14583	0.094304	+
[8436]	COL1A1	--->	ITGA2	0.0021195	-0.1162	+
[8437]	DLL4	--->	NOTCH1	-0.002934	-0.4558	+
[8438]	ABL1	--->	GATA2	0.23139	-0.27462	-
[8439]	CCNE1	<---	SKP1	-0.25849	0.28986	+
[8440]	PEBP1	<---	PPP2R1A	-0.46148	0.38342	-
[8441]	GJA1	--->	JUN	0.096401	-0.13523	-
[8442]	CEBPE	--->	CTCF	0.083205	-0.0091154	-
[8443]	CCL3	<---	CCR2	-0.35895	0.21658	-
[8444]	MAPK6	--->	STAT5B	1.4162	-0.966	+
[8445]	DUSP3	--->	MAPK12	1.4184	-1.4202	+
[8446]	RHOC	--->	SRGAP2	0.39011	-0.42732	-
[8447]	OPHN1	<---	RHOBTB2	-0.58391	0.47169	-
[8448]	F2RL2	--->	ROCK2	0.14216	-0.13417	-
[8449]	GNAO1	<---	PIK3CB	-0.00083608	0.0020832	-
[8450]	NCOA2	--->	WNT1	-0.0027487	-0.013491	-
[8451]	FGB	<---	VWF	-0.16084	0.087993	-
[8452]	CASP1	<---	CASP7	-0.88853	-0.039589	-
[8453]	PRKCE	--->	TRPV4	0.31032	-0.3562	+
[8454]	ARHGAP20	<---	RHOD	-1.4209	0.96682	-
[8455]	E2F6	--->	SP5	0.23995	-0.10098	+
[8456]	CNTFR	--->	SOCS5	-0.0038582	-0.019894	-
[8457]	ELK1	--->	PLAT	0.09819	-0.14736	+
[8458]	IL12B	--->	IL13RA2	-0.19263	-0.20337	+
[8459]	ERBB3	--->	PIK3CG	0.052945	-0.051452	+
[8460]	CD70	--->	STAT3	0.4382	-0.32637	-
[8461]	IL1B	<---	MAPK8	-0.078488	0.15109	+
[8462]	FGFR2	<---	FOXA2	-0.5105	0.34654	+
[8463]	NTF4	--->	NTRK1	0.52121	-1.1724	+
[8464]	IL2RG	<---	PRKCB	-0.17474	0.13376	-
[8465]	SOCS4	<---	STAT4	-0.73574	0.49958	+
[8466]	COL4A1	--->	ITGA4	-0.013036	-0.10803	+
[8467]	CAMK2D	--->	ITPR1	0.31376	-0.17692	-
[8468]	BUB1	--->	EP300	0.013728	-0.006941	-
[8469]	ATP2B4	<---	PRKACA	-0.060388	0.088587	+
[8470]	CD3E	--->	IL12RB1	0.12248	-0.29036	+
[8471]	MAP2K1	<--->	MAPK6	0.31014	0.31014	-
[8472]	PIK3C3	<---	PRKD2	-0.13755	0.19262	-
[8473]	PIK3CA	<---	PXN	-0.014324	0.0086969	-
[8474]	MYO9A	<---	RHOA	-0.54674	0.38058	-
[8475]	EGFR	--->	FGF1	0.25979	-0.22928	-
[8476]	PLCG2	--->	SPRY2	0.055284	-0.026882	+
[8477]	KIT	--->	PLCB3	0.012299	-0.015507	+
[8478]	ARNT	--->	EGF	0.037422	-0.029892	+
[8479]	ADCY4	<---	PRKCD	-0.47485	0.42125	+
[8480]	GRK4	--->	HTR2C	0.33635	-0.28786	+
[8481]	AKT3	--->	IKBKG	0.24308	-0.33358	+
[8482]	JAK3	--->	PRL	0.087709	-0.22013	-
[8483]	RELA	--->	WT1	0.50851	-0.57388	+
[8484]	CCL3	--->	MEF2A	0.056951	-0.23918	-
[8485]	CCL5	--->	FOS	0.15003	-0.061875	-
[8486]	ANGPT4	--->	IGF1R	0.25337	-0.017626	+
[8487]	FXYD2	--->	PIK3CA	-0.0050831	-0.010841	-
[8488]	BCL3	<---	IL12RB1	-0.20767	0.18969	-
[8489]	CCR5	--->	GNAI1	1.1631	-1.1303	+
[8490]	BCAR1	--->	ITGA8	0.070773	-0.089729	-
[8491]	IL23A	--->	IL2RB	-0.0077042	-0.055821	+
[8492]	SNAI2	<---	TEAD2	-1.1565	1.4387	+
[8493]	MYL12A	--->	MYLK2	0.41687	-0.23601	-
[8494]	MAP2K7	<---	TLR3	-0.24404	0.037699	+
[8495]	PRKCQ	--->	RASA1	0.054873	0.035692	-
[8496]	NGF	<---	PCSK6	-0.20029	0.16968	+
[8497]	DUSP10	--->	MAPK3	1.353	-1.036	+
[8498]	FGA	--->	FN1	0.064951	-0.2692	+
[8499]	CACNB2	<---	PPP2CA	-0.089071	0.21867	+
[8500]	MAPK8	<---	RHOG	-0.20304	1.2491	+
[8501]	JUN	<---	PPP1R15A	-0.3439	0.30421	-
[8502]	MAFK	<---	TRIB3	-0.75585	0.86539	-
[8503]	CCL16	<---	CCR5	-0.35895	0.21658	-
[8504]	CAMKK1	--->	PRKAG1	1.1791	-1.2499	+
[8505]	CRKL	--->	ITGB5	0.030916	-0.04526	-
[8506]	FAM13A	<---	RHOBTB1	-0.92575	0.61511	-
[8507]	INS	<---	KDR	-0.0084341	0.31135	-
[8508]	CDC42	<---	LCP2	-1.3049	1.3361	+
[8509]	PDGFRA	--->	SHC4	0.00054908	-0.0812	+
[8510]	AKT2	--->	CTNNB1	0.31031	-0.50152	+

[8511]	DUSP3	--->	MAP2K4	1.4184	-1.4202	+
[8512]	ACTG1	<---	WASL	-0.22029	0.18415	+
[8513]	CREB1	--->	TGFB3	0.063524	-0.11958	+
[8514]	CAMK2B	<---	PPP1CC	-0.015111	0.02592	+
[8515]	RIPK1	<---	UBE2D2	0.00032664	0.048994	+
[8516]	SFRP1	--->	WNT6	1.0317	-1.1063	+
[8517]	AKT1	--->	FRS2	0.18047	-0.25572	-
[8518]	DLG3	<---	YAP1	-0.05901	0.042003	-
[8519]	ACOX1	--->	RXRB	-0.03534	-0.098167	-
[8520]	GPR50	<---	GRK6	-0.28786	0.33635	+
[8521]	FYN	--->	GNAI1	0.67161	-0.44236	+
[8522]	PRKCD	--->	SHC1	0.60323	-0.51764	-
[8523]	ARHGAP36	<---	CDC42	-1.5245	1.016	-
[8524]	ACTG1	<---	WASF3	-0.51597	0.40448	+
[8525]	PRLR	<---	TYK2	-0.37792	0.17946	-
[8526]	ANGPT4	<---	KDR	-0.048064	0.33642	-
[8527]	AGTR1	--->	GNA15	1.1631	-1.1303	+
[8528]	BRCA1	--->	PPP1R15A	0.10665	-0.088921	+
[8529]	PRKACA	--->	TBXA2R	0.19743	-0.10024	+
[8530]	CSNK1G2	--->	FZD4	0.33941	-0.31414	-
[8531]	CCL2	--->	JUN	0.096001	-0.16123	-
[8532]	KIF2C	--->	PLK1	0.25675	-0.035893	-
[8533]	CNTN1	<---	JUND	-0.025751	-0.0040576	+
[8534]	FGFR3	--->	SPI1	0.055551	-0.098498	-
[8535]	CREB1	<---	MYH10	-0.03802	0.055125	-
[8536]	ESR1	<---	MAPK14	-0.30308	0.29076	+
[8537]	FN1	<---	ITGB7	-0.17721	0.073187	-
[8538]	FOSL2	<---	SRF	-1.0874	1.3477	+
[8539]	IL13RA2	<---	SOCS3	-0.10066	-0.08965	+
[8540]	FADS2	<---	PPARA	-0.19474	0.024476	+
[8541]	DKK2	--->	WNT1	0.54164	-0.69755	+
[8542]	CALM1	--->	KRAS	0.20073	-0.14858	+
[8543]	FAM13B	<---	RHOD	-1.5245	1.016	-
[8544]	COMP	<---	ITGAV	-0.021497	-0.014728	-
[8545]	ATP1B4	--->	PIK3R1	0.049293	-0.12511	-
[8546]	CSF1	--->	FGFR1	0.96048	-0.85465	+
[8547]	HDAC3	<---	SMARCD3	-0.34009	0.39365	-
[8548]	RXRA	<---	SMARCD3	-0.27269	-0.085302	-
[8549]	FASN	--->	SREBF2	0.0053147	-0.012347	-
[8550]	GNA11	--->	WNT2	0.42087	-0.47341	-
[8551]	MAFK	<---	NKX2-4	-0.073912	-0.019848	-
[8552]	CACNA1C	<---	PPP2R3A	-0.36974	0.36606	+
[8553]	IL2RG	<---	PTPN6	-0.16409	0.19246	-
[8554]	ETS1	<---	NFKB1	-0.14364	0.14195	+
[8555]	BCL10	<---	UBE2N	-0.62204	0.56098	-
[8556]	CCL27	<---	CXCR2	-0.35895	0.21658	-
[8557]	ACVR1B	--->	SMAD3	0.72329	-0.52231	+
[8558]	BATF	<---	EP300	-0.010885	0.015595	+
[8559]	MTOR	<---	RRAGA	-0.26816	0.38257	+
[8560]	LCK	<--->	LYN	0.15012	0.15012	-
[8561]	PPP2R1A	--->	YWHAB	0.1764	-0.3228	-
[8562]	ARNT	<---	PFKFB3	-0.099193	0.096943	-
[8563]	PTGFR	<---	STAT1	-0.29222	0.22857	+
[8564]	GNB3	--->	WNT6	0.19155	-0.26814	-
[8565]	CACNA1S	<---	GNAI3	-0.17558	0.23994	+
[8566]	KCNJ9	<---	PRKACA	-0.26091	0.40675	+
[8567]	CBX3	<---	UBTF	-0.010951	0.0056471	-
[8568]	HGS	--->	VPS4A	-0.0027478	-0.021506	-
[8569]	GNB3	--->	WNT5A	0.19155	-0.26814	-
[8570]	MAP2K4	<---	RAF1	-1.3266	0.99795	+
[8571]	FRS3	--->	SPRY4	0.23114	-0.13172	+
[8572]	ATP1A2	--->	PIK3CB	-0.0036365	-0.0057548	-
[8573]	ITGA2	<---	THBS3	-0.046542	-0.014105	+
[8574]	FGFR4	<---	PDGFC	-0.38721	0.2003	+
[8575]	FGF7	--->	FLT4	0.071829	-0.16555	+
[8576]	E2F4	<---	HNRNPUL1	-0.10846	0.077535	-
[8577]	ELK1	--->	PBX1	0.15908	-0.24396	+
[8578]	DIAPH2	<---	PFN3	-0.1109	0.062206	-
[8579]	FGF8	--->	KIT	0.015796	-0.016964	+
[8580]	SMAD3	--->	ZNF823	1.148	-1.3141	+
[8581]	PIK3R3	<---	TYK2	-1.0298	0.46635	+
[8582]	IL1B	--->	NOD1	0.17679	-0.19911	-
[8583]	GRIN2D	<---	PPP1CC	-0.67094	0.67498	+
[8584]	GRIN2B	<---	RHOG	-0.38938	0.54247	-
[8585]	ESR1	<---	WWC1	-0.28508	0.094622	-
[8586]	ARRB2	--->	LRP5	0.35827	-0.27207	-

```
[8587]  CSNK1G3 --->    SAG     0.14787 -0.09775        -
[8588]  ADRB1   <---    GRK7    -0.28786        0.33635 +
[8589]  CNTN1   <---    NRCAM   -0.025365       -0.021378       -
[8590]  CACNA1C <---    GNB1    -0.095409       0.10645 +
[8591]  ERBB4   --->    HRAS    0.17363 -0.11617        +
[8592]  CCL18   <---    CCR1    -0.35895        0.21658 -
[8593]  PLG     --->    SERPINA1        0.18568 -0.33063        -
[8594]  CHUK    <---    RIPK2   -0.29159        0.50882 +
[8595]  PPP2R2A <---    YWHAE   -0.1213 -0.088103       -
[8596]  KIT     --->    PLCB2   0.012299        -0.015507       +
[8597]  MLLT4   --->    PIK3R1  0.02885 -0.048701       +
[8598]  PIK3CG  <---    PRKCD   -0.073525       0.055854        -
[8599]  RPL30   <--->   RPS12   0.2632  0.2632  -
[8600]  MAPK12  <--->   MAPK8   0.31014 0.31014 -
[8601]  ATP1A2  <---    NR3C2   -0.056504       -0.0074276      +
[8602]  BAMBI   --->    SMAD1   0.16225 -0.34174        -
[8603]  RHOU    --->    SRGAP1  0.39011 -0.42732        -
[8604]  MAP2K6  <--->   MAP3K4  0.31014 0.31014 -
[8605]  LRP6    <---    WNT9B   -0.28769        0.39899 +
[8606]  ADRBK1  --->    CXCR5   0.25327 -0.21301        +
[8607]  ATF3    --->    TGFB2   0.10655 -0.19533        +
[8608]  CD80    <---    RAC1    -0.045032       0.064614        -
[8609]  AKT1    --->    RXRA    0.46238 -0.59236        +
[8610]  NCOA3   --->    WNT10B  -0.0027487      -0.013491       -
[8611]  CSNK2A1 --->    L1CAM   0.1354  -0.12611        +
[8612]  PTEN    --->    ZEB1    0.018013        -0.011332       -
[8613]  ATM     <---    FOXO3   -0.12   0.11865 +
[8614]  DVL1    <---    WNT11   -0.54867        0.38842 +
[8615]  CD274   <---    CD3E    -0.047639       -0.0075134      -
[8616]  DDX17   <---    EBF1    -0.030514       0.02426 +
[8617]  CBLC    --->    SPRY4   0.48869 -0.21829        -
[8618]  DEPDC7  <---    RHOBTB2 -0.036638       0.070172        -
[8619]  PPP1R1A --->    PPP3CC  1.0742  -1.0103 -
[8620]  BCL3    <---    SDF2L1  -0.28142        0.32029 -
[8621]  PLCB2   <---    RGS4    -0.075217       0.041005        +
[8622]  CDC42   <---    RASGRF2 -0.52428        0.44606 +
[8623]  MAP2K2  --->    SFN     0.079981        -0.071802       -
[8624]  ITGA2   <---    PIK3CA  -0.024164       -0.004208       -
[8625]  APOA1   --->    HSPG2   0.0058271       -0.0016468      -
[8626]  NANOG   --->    REST    0.078944        0.049909        +
[8627]  ACTG1   <---    FLNB    -0.51597        0.40448 +
[8628]  CTCF    --->    NCOA3   0.0206  -0.01067        +
[8629]  CHD6    --->    EBF1    -0.0040582      -0.10088        -
[8630]  BRCA1   --->    SYCP3   0.21008 -0.22984        +
[8631]  ARHGEF6 <---    PAK4    0.02191 0.098798        +
[8632]  RHOC    --->    VAV3    0.058196        0.018587        -
[8633]  PREX1   <---    RAC1    -0.049683       0.1134  -
[8634]  MAPK3   --->    S1PR4   0.19743 -0.10024        -
[8635]  PRKCE   --->    STAT6   0.32523 -0.30281        -
[8636]  ADAMTS2 <---    COL5A2  -0.054632       -0.00053524     -
[8637]  BCL11A  <---    KLF6    -0.039841       -0.037992       -
[8638]  CD3E    --->    IL2RG   0.056231        -0.11334        +
[8639]  E2F6    --->    GSC     0.11901 -0.19289        +
[8640]  EP300   --->    IL36G   -0.0017666      -0.0027987      +
[8641]  ATF2    --->    TGFB2   0.32646 -0.28355        +
[8642]  ARHGAP12        <---    RHOH    -0.36596        0.22724 -
[8643]  IGF1R   --->    IRS4    0.49489 0.047363        +
[8644]  GRIA2   <---    PRKCB   -0.43995        0.47541 +
[8645]  MYC     <---    SMARCA4 -0.093387       0.019386        +
[8646]  CEBPG   <---    NFKB1   -0.23502        0.23585 +
[8647]  CBL     --->    HIF1A   0.077099        -0.017992       +
[8648]  AJUBA   --->    CDH1    0.27539 -0.55227        -
[8649]  PRKCB   --->    TTF2    0.19541 -0.25805        +
[8650]  CSF2RB  <---    IL6     -0.10334        0.076998        +
[8651]  CDC42   --->    MYO10   0.39857 -0.56864        -
[8652]  ANGPT1  <---    DOK1    -0.011847       0.13965 -
[8653]  EP300   --->    FCER2   0.00067605      -0.0050208      +
[8654]  BRSK1   --->    MAP2    0.40675 -0.26091        +
[8655]  MAP2K1  --->    NRG1    0.38187 -0.37016        -
[8656]  AKT2    <---    TCL1B   -1.4472 1.0764  +
[8657]  F2      <---    GP1BA   -0.23217        0.098653        -
[8658]  CSNK1E  --->    WNT9B   0.83912 -0.78694        -
[8659]  BLNK    --->    PLCG1   0.050029        0.0024742       +
[8660]  CCL20   <---    CXCR6   -0.35895        0.21658 -
[8661]  CDKN1A  --->    JUN     0.15531 -0.10887        -
[8662]  PSME4   <---    RELA    -0.25054        -0.060722       -
```

[8663]	BID	<---	DAXX	-0.53278	0.27228	+
[8664]	PAK3	<---	RAC3	-1.1901	0.96651	+
[8665]	CHN1	<---	RHOC	-0.4978	0.36255	-
[8666]	ZNF263	<---	ZNF302	-0.025264	-0.024143	-
[8667]	ARHGAP44	<---	RHOA	-0.92575	0.61511	-
[8668]	LCK	<---	PRKCZ	-0.35659	0.56064	-
[8669]	IL16	<---	SLA2	-0.13646	0.16474	-
[8670]	RAB8A	--->	TTBK2	1.2491	-0.20304	-
[8671]	AKAP13	<---	RHOJ	-0.22586	0.072537	-
[8672]	BCL6	<---	CTCF	-0.028383	-0.0051278	+
[8673]	FOSL1	<---	MAPK8	-0.57905	1.0858	+
[8674]	IKBKE	--->	ZBP1	0.077555	-0.010113	-
[8675]	CREB3L4	<---	PRKX	-0.57905	1.0858	+
[8676]	FGF5	<---	FRS2	-0.28504	0.43716	-
[8677]	ATF1	<---	TFRC	-0.03802	0.055125	-
[8678]	MAX	<---	ZNF84	-0.037258	0.11789	-
[8679]	EPHA1	<---	FYN	-0.26855	0.35424	+
[8680]	CD19	--->	PIK3CB	0.035369	-0.041966	+
[8681]	HIF1A	--->	PFKP	0.83907	0.11601	+
[8682]	COL4A6	--->	ITGA7	-0.013036	-0.10803	+
[8683]	IRS1	<---	PDGFRB	0.0012647	0.51189	+
[8684]	PAX8	<---	PRKX	-0.15935	0.24561	+
[8685]	CTNNB1	<---	PCDHB13	-0.19204	0.11503	-
[8686]	CSNK2A1	--->	SRF	0.35865	-0.36564	+
[8687]	C4A	--->	FCN1	0.1408	-0.23228	-
[8688]	FGFR3	--->	NGF	-0.074067	-0.088225	-
[8689]	POU2F2	--->	ZBTB32	0.046782	0.029172	+
[8690]	NCK2	--->	PAK1	0.73462	-0.72841	+
[8691]	EPHB2	<---	L1CAM	-0.041976	-0.038328	-
[8692]	MAP2K1	<--->	MAP2K2	0.31014	0.31014	-
[8693]	CSNK1A1	--->	YWHAG	0.079981	-0.071802	+
[8694]	FOXO1	--->	SOD2	0.19808	-0.23065	+
[8695]	CRHR1	<---	GNAI3	-0.040633	0.044298	-
[8696]	CTCFL	<---	TCF7L2	-0.018993	0.22714	+
[8697]	C4BPB	<---	EP300	0.0019625	0.0040377	+
[8698]	CALML3	--->	CAMK1	0.41687	-0.23601	+
[8699]	AKT1	--->	MAP3K8	1.3215	-1.2112	+
[8700]	MYC	<---	PWP1	-0.23498	0.053032	-
[8701]	FCGR3B	<---	MAPK12	0.12024	0.14714	-
[8702]	BAMBI	--->	DVL3	0.50701	-0.66044	+
[8703]	CNTN1	<---	JUN	-0.025751	-0.0040576	+
[8704]	SMAD4	--->	ZBTB17	0.44396	-0.48644	+
[8705]	GSG2	--->	ZBTB33	0.66556	-0.40919	-
[8706]	E2F7	<---	RYBP	-0.31023	0.24004	+
[8707]	ATP1A1	<---	MAPK1	-0.09775	0.14787	+
[8708]	GLI2	--->	WNT3	0.86373	-1.47	+
[8709]	RHOT1	--->	SRGAP3	0.10836	-0.13505	-
[8710]	ESM1	--->	JUN	0.16075	-0.22115	-
[8711]	C1QB	<---	C4B	-0.19739	0.12796	-
[8712]	MCL1	<---	SRF	-1.1204	0.86149	+
[8713]	F2RL1	--->	GNAQ	1.1631	-1.1303	+
[8714]	CEBPB	--->	POU5F1	0.15908	-0.24396	+
[8715]	BCR	<---	RHOH	-0.20131	0.10561	-
[8716]	DAND5	--->	SMAD4	-0.0018717	-0.12079	+
[8717]	AXIN1	--->	NOTCH1	0.18717	-0.14894	-
[8718]	NPAS2	<---	RORC	-0.14396	0.10125	-
[8719]	JAK2	<---	SOCS5	-0.73315	0.53539	+
[8720]	GMIP	<---	RAC2	-1.011	0.68428	-
[8721]	CAMK2D	<---	KRAS	-0.061708	0.69771	-
[8722]	PIAS1	--->	STAT1	1.4095	-0.71172	+
[8723]	FOXO1	--->	KLF2	0.65129	-0.48763	+
[8724]	DHX38	--->	RAE1	0.072414	-0.071625	-
[8725]	ESR1	<---	GADD45A	-0.26567	0.025734	-
[8726]	IL12RB2	<---	NFKB2	-0.14133	0.12027	-
[8727]	CDC42	<---	GNAZ	-0.93465	1.2694	+
[8728]	CCL22	<---	CCR5	-0.35895	0.21658	-
[8729]	PORCN	--->	WNT3A	0.38949	-0.56891	+
[8730]	DKK4	--->	FZD9	0.3176	-0.35737	+
[8731]	FZD1	<---	WNT5A	-0.78209	0.63734	+
[8732]	GRK6	--->	HTR2C	0.33635	-0.28786	+
[8733]	FZD10	<---	WNT3A	-0.78209	0.63734	+
[8734]	ANK1	--->	ITPR2	0.099591	-0.057766	+
[8735]	IL26	--->	IL2RB	-0.0077042	-0.055821	+
[8736]	CCL27	<---	CCR4	-0.35895	0.21658	-
[8737]	CAMK2B	--->	GRIN1	0.94748	-0.64695	-
[8738]	GRIN2C	<---	RAC1	-0.38938	0.54247	-

[8739]	ARRB1	--->	WNT2B	0.23514	-0.34616	+
[8740]	E2F1	<---	HBP1	-0.082739	0.10702	+
[8741]	DAND5	--->	MTMR4	0.036704	-0.072158	+
[8742]	RGMB	--->	SMAD9	0.016452	-0.10584	+
[8743]	FLI1	--->	TNC	0.031276	-0.080829	+
[8744]	LRP5L	<---	PSEN1	-0.16338	0.1035	+
[8745]	ARHGAP32	<---	RHOBTB1	-0.29676	0.17827	-
[8746]	CEBPB	<---	CXCR4	-0.2522	0.11779	-
[8747]	BDKRB2	--->	GNA14	1.1631	-1.1303	+
[8748]	GRIN2C	<---	RHOJ	-0.38938	0.54247	-
[8749]	PRKAG1	--->	SLC2A1	0.34513	-0.50137	+
[8750]	MYC	<---	TCF7L2	-0.15083	0.096323	+
[8751]	DAND5	--->	PCK1	0.26151	-0.27583	+
[8752]	PIM1	--->	STAT3	1.4162	-0.966	-
[8753]	GNB4	<---	PIK3CA	-0.012551	0.008521	-
[8754]	COL4A1	--->	ITGA5	-0.013036	-0.10803	+
[8755]	ITGB1	--->	PIK3R3	0.057037	-0.12143	+
[8756]	IL22RA1	<---	SOCS4	-1.0456	0.57217	+
[8757]	LCK	--->	RAC1	1.0122	0.16613	+
[8758]	DVL3	<---	WNT5A	-0.54867	0.38842	+
[8759]	BRD2	--->	MYC	0.073333	-0.28761	-
[8760]	GRB2	--->	IFNAR1	0.17317	-0.077015	-
[8761]	CCR7	--->	GNAI3	1.1631	-1.1303	+
[8762]	ERBB3	--->	PIK3R1	0.59225	-0.30243	+
[8763]	CTNND1	<---	MET	-0.40235	0.29277	-
[8764]	RHOBTB1	--->	SRGAP1	0.23551	-0.26028	-
[8765]	FGF7	--->	INSR	0.25346	-0.0044024	+
[8766]	ITGA3	--->	THBS3	0.01404	-0.033876	-
[8767]	IKZF3	<---	SOCS3	-0.050058	-0.028263	+
[8768]	PPIA	--->	VPS4B	-0.028507	-0.068349	-
[8769]	PIK3R1	<---	THRA	-0.12175	0.033109	-
[8770]	GHRL	--->	LEP	0.29058	-0.3626	-
[8771]	FGF11	--->	PDGFRB	0.48772	-0.57252	+
[8772]	ADRBK1	--->	DRD5	0.25327	-0.21301	+
[8773]	ITGA1	<---	KDR	-0.020519	0.042957	-
[8774]	MEF2C	<---	MYH14	-0.1118	0.043005	-
[8775]	CTNND1	<---	ENAH	-0.19176	0.27658	-
[8776]	CREB3L3	<---	RPS6KA5	-0.98092	1.2458	+
[8777]	CREB3L2	<---	MAPK3	-0.57905	1.0858	+
[8778]	KEAP1	<---	PRKCA	-0.41207	0.32366	+
[8779]	RHOG	--->	SYDE1	1.016	-1.5245	-
[8780]	CREB5	<--->	CREM	0.22155	0.22155	-
[8781]	PRKCG	--->	SPHK2	0.32366	-0.41207	+
[8782]	ARHGAP31	<---	RHOF	-1.5245	1.016	-
[8783]	GNA14	<---	LHCGR	-1.1303	1.1631	+
[8784]	COL27A1	--->	ITGA5	-0.013036	-0.10803	+
[8785]	HMHA1	<---	RHOC	-0.62084	0.42373	-
[8786]	GNAQ	--->	WNT3	0.42087	-0.47341	-
[8787]	FSHB	<---	GPR119	-0.2522	0.11779	-
[8788]	ATF4	<---	DDIT3	-0.098546	0.15465	-
[8789]	GNAI2	<---	PXN	-0.057204	0.12598	-
[8790]	GNB3	--->	LRP6	0.34224	-0.24336	-
[8791]	CREBBP	--->	WNT1	0.022202	-0.03121	-
[8792]	PSMD10	<---	TNKS	-0.019588	-0.010926	-
[8793]	BMP8B	--->	BMPR1A	0.27387	-0.33406	+
[8794]	CSNK1A1L	--->	WNT3A	0.83912	-0.78694	-
[8795]	CCL19	<---	CCR2	-0.35895	0.21658	-
[8796]	HRAS	<---	HSP90AA1	-0.11666	0.13065	+
[8797]	PAK1	<---	RHOA	-1.1901	0.96651	-
[8798]	COL5A3	--->	ITGA1	-0.0065074	-0.1017	+
[8799]	MYC	<---	TBRG4	-0.27886	0.067885	-
[8800]	CRADD	<---	MAP2K4	-0.56263	0.35307	-
[8801]	DUSP19	--->	MAPK10	1.4184	-1.4202	+
[8802]	PRKCH	--->	PTPN11	0.55493	-0.48581	-
[8803]	GRK7	--->	HTR7	0.33635	-0.28786	+
[8804]	EXO1	--->	SP3	0.28002	-0.15205	-
[8805]	BIRC5	<---	TEAD2	-0.5426	0.36567	+
[8806]	BAMBI	--->	BMP3	0.39038	-0.6158	+
[8807]	EP300	--->	RHOXF2B	0.010731	-0.015032	+
[8808]	PIK3C3	<---	PRKCA	-0.16343	0.14021	-
[8809]	CAV1	--->	SMAD1	-0.050142	-0.10303	+
[8810]	GLI1	<---	NUMB	-0.046872	0.14661	+
[8811]	PAX3	--->	RB1	0.060375	-0.13136	-
[8812]	CEACAM5	<---	EGR1	-0.01152	-0.0018337	+
[8813]	RAP1B	<---	RAP1GAP2	-0.50061	0.67654	+
[8814]	CSK	--->	PTK2B	0.34449	-0.5161	+

[8815]	MAPK11	--->	SCMH1	0.40675	-0.26091	+
[8816]	DCLK1	--->	EP300	0.013728	-0.006941	-
[8817]	ENG	<---	NFKB1	-0.26089	0.26587	+
[8818]	CAMK2G	--->	SCN5A	0.43397	-0.32993	+
[8819]	CRLF1	--->	MYC	-0.0082814	-0.085194	-
[8820]	JUND	<---	PTGIR	-0.3481	0.2582	-
[8821]	EPO	--->	VAV2	0.062045	-0.017162	+
[8822]	EP300	--->	PFKFB4	-0.00068954	-0.0041449	+
[8823]	PAX8	--->	TP53	0.087796	-0.082414	+
[8824]	ARF4	--->	PLD2	0.041749	-0.076299	+
[8825]	CTBP2	--->	PAX6	0.024957	-0.10255	+
[8826]	AKT2	<---	FGFR3	-0.040847	0.067461	+
[8827]	INSR	<---	PRKCI	-0.082736	0.15778	-
[8828]	GDF15	<---	HNF4A	-0.25297	0.060298	+
[8829]	KMT2C	--->	ZBTB7A	0.20133	-0.10609	-
[8830]	EP300	<---	UBE2B	-0.0073912	0.0036459	-
[8831]	CBLC	--->	THBS1	0.10256	-0.04331	+
[8832]	CBL	--->	IL6R	0.11953	-0.076091	+
[8833]	HCK	--->	NGEF	0.49172	-0.08758	+
[8834]	IL10RA	<---	SOCS4	-1.0456	0.57217	+
[8835]	PDGFC	--->	SMAD1	-0.035562	-0.11772	-
[8836]	CALML6	--->	MYLK	0.50863	-0.51028	+
[8837]	ERG	<---	TGFBR2	-0.21357	0.12981	-
[8838]	PAX8	<---	PRKCA	-0.25103	0.19521	+
[8839]	ACVRL1	--->	EP300	0.0039961	-0.013125	-
[8840]	INSR	<---	VEGFC	-0.033666	0.27735	+
[8841]	IGHD	<---	PIK3CD	-0.03103	0.014005	+
[8842]	MYBPC1	<---	MYH3	-0.15073	0.094518	+
[8843]	CHEK2	--->	PLK3	0.17826	-0.061256	-
[8844]	ACP1	--->	ARHGAP35	0.2073	-0.1739	+
[8845]	RHOV	--->	STARD8	1.016	-1.5245	-
[8846]	CEBPB	<---	RGS1	-0.4184	0.31307	-
[8847]	CBL	--->	DAPP1	0.17391	-0.20135	+
[8848]	CTNNB1	<---	PTPRF	-0.27338	0.16142	+
[8849]	MAP3K2	<---	RAF1	-0.63747	0.59893	+
[8850]	BCL3	<---	FAIM3	-0.3023	0.32438	-
[8851]	BUB1B	--->	UBE2C	0.24471	-0.086834	-
[8852]	RIPK1	--->	TRADD	0.054904	-0.27429	-
[8853]	FGF21	--->	FLT4	0.071829	-0.16555	+
[8854]	IL10	--->	IL10RA	0.20113	-0.087402	+
[8855]	CCL2	<---	CCR5	-0.35895	0.21658	-
[8856]	GRAP	--->	SPRY4	0.12642	-0.14227	-
[8857]	VAV1	--->	VAV2	-0.0022972	-0.0026907	+
[8858]	RAP1B	<---	RAPGEF1	-0.72612	0.70033	+
[8859]	RAN	<---	RELA	-0.34579	0.18991	-
[8860]	CCL2	--->	STAT3	0.42026	-0.3013	-
[8861]	MYL4	<---	TNNC2	0.034293	0.19015	-
[8862]	CDC25B	<---	CHEK1	-0.97911	0.26855	+
[8863]	PIK3R3	<---	SYK	-1.0692	0.59646	+
[8864]	NKX2-1	--->	TCF12	0.23888	-0.32236	-
[8865]	DDX17	<---	NFKB1	-0.1408	0.13949	+
[8866]	ITGA6	<---	PTK2	-0.076638	-0.038051	-
[8867]	LCP2	<---	PTPN11	-0.74716	0.41039	+
[8868]	CDH1	<---	GNA12	-0.37452	0.096821	+
[8869]	AXIN2	<---	TEAD4	-0.2017	0.14662	+
[8870]	KALRN	<---	RHOV	-0.021179	0.042607	-
[8871]	RELA	--->	RIPK2	0.18977	-0.41136	-
[8872]	ADCY6	<---	PRKX	-0.63235	0.8673	-
[8873]	ADRA2A	<---	GRK4	-0.28786	0.33635	+
[8874]	DUSP1	--->	FOXA1	0.31859	-0.22873	-
[8875]	GHR	<---	TYK2	-0.37792	0.17946	-
[8876]	PDK4	--->	RXRA	0.10013	-0.32848	-
[8877]	IFNG	--->	IL20RB	0.21627	-0.39151	+
[8878]	FGB	<---	FGG	-0.11145	-0.0094624	-
[8879]	ADCY2	<---	PRKX	-0.63235	0.8673	-
[8880]	DUSP19	--->	MAP2K4	1.4184	-1.4202	+
[8881]	ATR	<---	CDC7	-0.35989	0.42273	-
[8882]	NR4A2	<---	TCF7L2	-0.983	0.9955	+
[8883]	MCF2L	<---	RHOBTB1	-0.017135	0.016856	-
[8884]	ADRA1B	<---	ADRBK2	-0.21301	0.25327	+
[8885]	CSNK1A1	--->	FOXO1	0.2499	-0.22085	+
[8886]	AMPH	--->	EGF	0.1229	-0.12453	+
[8887]	NFKBIB	<---	PSME4	-0.19284	0.32731	+
[8888]	BZRAP1	<---	SYT1	-0.093827	-0.0028077	+
[8889]	MYL9	<---	MYLK3	-1.5134	1.3994	+
[8890]	AKT3	--->	MAP3K8	1.3215	-1.2112	+

[8891]	CCL4L1	<---	CXCR6	-0.35895	0.21658	-
[8892]	ACACB	<---	PRKAG3	-0.95328	1.0154	+
[8893]	DKK4	--->	WNT3A	0.54164	-0.69755	+
[8894]	ARHGEF17	<---	RAC2	-0.012974	0.047696	-
[8895]	GNAI3	<--->	GNAT1	0.064764	0.064764	-
[8896]	ARHGAP11B	<---	RHOV	-1.5245	1.016	-
[8897]	ACTG1	<---	ARHGAP10	-0.17746	0.10822	+
[8898]	CCL7	<---	XCR1	-0.35895	0.21658	-
[8899]	PLCG2	<---	SHC1	0.0014679	0.027414	-
[8900]	CACNA1D	--->	GRIN2A	0.15899	-0.088421	-
[8901]	ITGA6	<---	VWF	-0.05402	0.0049709	+
[8902]	CAMK2G	--->	PDE4D	0.95928	-0.63646	+
[8903]	GNB1	<---	KCNJ5	-0.070696	0.05091	-
[8904]	CLOCK	<---	RORB	-0.14396	0.10125	-
[8905]	CREB3L3	<---	GSK3B	-0.57905	1.0858	+
[8906]	PGR	--->	PIK3R5	0.2532	-0.46306	+
[8907]	ITGB1	--->	THBS1	0.17581	-0.068027	-
[8908]	IGF1R	<---	PDGFD	-0.033666	0.27735	+
[8909]	JAM2	<---	PPP2CA	-0.1941	0.28865	+
[8910]	EGF	<---	PPP2CA	-0.14579	0.18616	-
[8911]	MYBL1	--->	STAT6	0.10758	-0.082415	+
[8912]	JUNB	--->	STAT3	0.22495	-0.1553	-
[8913]	FGF19	<---	KRAS	-0.25726	0.38422	-
[8914]	DUSP12	--->	MAPK8	1.4184	-1.4202	+
[8915]	EGFR	--->	VEGFB	0.23053	-0.20539	-
[8916]	FZD7	<---	SFRP5	-1.3633	0.73901	+
[8917]	DKK4	--->	WNT4	0.54164	-0.69755	+
[8918]	MEF2A	<---	POU2F2	-0.1766	-0.00025604	-
[8919]	AKAP13	--->	RHOT1	-0.012865	-0.084256	+
[8920]	MAPK1	<---	PRKCA	-0.092669	0.0037501	-
[8921]	CREB5	<---	PRKCG	-0.63701	0.72046	+
[8922]	CIT	--->	MYL2	0.33713	-0.28387	+
[8923]	SREBF2	<---	STRN3	-0.38125	0.38477	-
[8924]	ITGA9	--->	THBS1	0.16838	-0.067658	-
[8925]	LPAR2	<---	TRIP6	-0.26988	0.26458	-
[8926]	FZD10	<---	WNT10A	-0.78209	0.63734	+
[8927]	GRB2	--->	IL2RB	0.28454	-0.16347	-
[8928]	FGF8	<---	NRAS	-0.15714	0.23194	-
[8929]	COL14A1	<---	PTK2B	-0.083928	-0.020315	+
[8930]	ACTR3	<---	MYO10	-0.064534	0.043881	+
[8931]	PABPC1L	<---	PAN3	-0.47902	1.2133	-
[8932]	RRAS	<---	SOS2	-0.33772	0.48674	+
[8933]	EGR1	<---	PRKCQ	-0.48974	0.50654	-
[8934]	FZD8	<---	WNT10B	-0.78209	0.63734	+
[8935]	CSF2	--->	TCF12	0.58337	-0.57708	-
[8936]	JAK3	--->	SMPD1	0.13921	-0.35667	+
[8937]	IL12B	<---	REL	-0.71218	0.35097	+
[8938]	GH2	--->	IL11RA	0.084246	-0.14339	+
[8939]	CHUK	<---	TRAF6	-0.40881	0.8262	+
[8940]	CDKN1B	<---	E2F4	-0.00078709	0.10216	-
[8941]	CDK11A	--->	CREB1	0.65748	-0.35231	-
[8942]	STAT5B	<---	TCF7L2	-0.18223	0.30036	+
[8943]	PRLR	<---	THPO	-0.16507	0.040195	+
[8944]	GLI1	--->	WNT8B	0.86373	-1.47	+
[8945]	ATP1B4	--->	PIK3CG	-0.0065677	-0.016061	-
[8946]	EP300	--->	TSC2	-0.0015837	-0.0063766	+
[8947]	ARHGEF18	<---	RHOB	-0.012974	0.047696	-
[8948]	ARHGAP12	<---	RAC2	-0.36596	0.22724	-
[8949]	AKT3	--->	RELA	0.35546	-0.53283	+
[8950]	PBX2	<---	SPI1	-0.24396	0.15908	+
[8951]	KALRN	<---	RHOB	-0.021179	0.042607	-
[8952]	IGF1	<---	MET	-0.08293	0.060414	-
[8953]	CBX3	--->	E2F1	0.039909	-0.13325	-
[8954]	COL6A3	--->	ITGA4	-0.0067115	-0.019363	+
[8955]	FOS	--->	TGFB1	0.10655	-0.19533	+
[8956]	CEBPD	<---	IL1B	-0.12163	0.053753	-
[8957]	HDAC2	<---	ZNF718	0.01176	0.26827	-
[8958]	AKT2	--->	CREB3L4	0.82132	-1.3257	+
[8959]	IFNAR1	<---	PTPN1	-1.0971	1.1363	+
[8960]	GRIN2D	<---	HRAS	-0.23727	0.32792	-
[8961]	GP5	--->	SERPIND1	0.49466	-0.83407	-
[8962]	MAPK1	<---	RAP1A	-0.20304	1.2491	+
[8963]	GNRHR	--->	KCNJ5	0.11779	-0.2522	-
[8964]	C4A	--->	MBL2	0.12796	-0.19739	-
[8965]	CD44	<---	EP300	-0.011981	0.0032365	+
[8966]	ADORA1	--->	GNAI2	1.1631	-1.1303	+

[8967]	MAPK14	<---	TRAF6	-0.47605	1.1617	-
[8968]	ADRA1D	--->	GNA15	1.1631	-1.1303	+
[8969]	CSF2RB	--->	STAT5B	0.078645	-0.047643	+
[8970]	CLASP1	--->	EP300	0.0060622	-0.010919	-
[8971]	TCF7L2	--->	ZNF703	0.14025	0.031386	+
[8972]	CSF1R	<---	FGF5	-0.64493	0.77791	+
[8973]	GNA12	--->	RHOA	1.2694	-0.93465	+
[8974]	CREB3L1	<---	PRKACA	-0.57905	1.0858	+
[8975]	BMP1	<---	COL27A1	-0.19386	0.13754	-
[8976]	HIF1A	--->	TP53	0.072953	0.014945	+
[8977]	PVRL2	<---	TIGIT	-0.070058	0.015031	-
[8978]	PPP6C	--->	YWHAH	-0.03225	-0.31369	-
[8979]	MYLK2	<---	PAK4	-1.2674	1.5523	+
[8980]	H3F3A	<---	UBE2D1	-0.48509	0.38736	-
[8981]	GYS2	<---	PPP1CC	-0.9036	0.53904	+
[8982]	COL4A5	--->	ITGA11	-0.013036	-0.10803	+
[8983]	FGF21	--->	INSR	0.25346	-0.0044024	+
[8984]	FADD	<---	MAP4K4	-0.12886	0.0010581	-
[8985]	CCL8	<---	CXCR1	-0.35895	0.21658	-
[8986]	KPNA4	<---	NUP43	-0.19114	0.12101	+
[8987]	SERPINF2	--->	TAL1	-0.052208	-0.1478	-
[8988]	ITGAV	--->	LRP1	0.074407	-0.073001	-
[8989]	FOSL1	<---	MAPK11	-0.57905	1.0858	+
[8990]	CRADD	<---	SMPD1	-0.3606	0.16195	-
[8991]	STXBP1	<---	SYT1	-0.31929	0.15277	+
[8992]	CRK	--->	ITGAV	0.032574	-0.078547	-
[8993]	PRF1	<---	STAT1	-0.29331	0.22348	+
[8994]	IL19	--->	IL4R	0.073187	-0.17721	+
[8995]	IL26	--->	IL3RA	-0.0077042	-0.055821	+
[8996]	CCL17	<---	CCR4	-0.35895	0.21658	-
[8997]	MYBPC2	--->	MYL2	-0.064509	-0.083906	-
[8998]	ARHGAP24	<---	RHOJ	-0.49576	0.47865	-
[8999]	CBL	--->	IL22RA2	0.61825	-0.39145	+
[9000]	CORO1A	<---	EP300	-0.0077682	0.007799	+
[9001]	PIK3CB	<---	THRA	-0.021852	-0.0056486	-
[9002]	COL6A1	<---	ITGB8	-0.040539	-0.022167	-
[9003]	CNTF	--->	IL2RG	0.056231	-0.11334	+
[9004]	MMP2	<---	SRC	-0.33465	0.39283	+
[9005]	CREBBP	--->	PGK1	0.00067605	-0.0050208	+
[9006]	CCL20	<---	CCR7	-0.35895	0.21658	-
[9007]	APP	<---	MAPK15	-0.023982	0.030823	-
[9008]	ABL1	--->	BAIAP2	0.084793	-0.11794	+
[9009]	HES3	<---	REST	-0.20142	0.33079	+
[9010]	DUSP3	--->	MAPK13	1.4184	-1.4202	+
[9011]	BAK1	<---	STAT1	-0.41695	0.33614	+
[9012]	PIAS2	--->	STAT4	1.2392	-0.72187	+
[9013]	EPHA2	--->	FGF22	-0.0069714	-0.050497	-
[9014]	EP300	--->	TNPO1	-0.0033278	-0.0034595	+
[9015]	GAB1	<---	IL6ST	-0.029955	0.032714	+
[9016]	RXRA	--->	WNT10B	-0.10298	-0.28478	-
[9017]	DTX3	--->	NOTCH4	0.024069	-0.0013567	-
[9018]	GNAO1	<---	PRKACG	-0.22953	0.50225	+
[9019]	COL2A1	<---	FYN	-0.26218	0.36104	-
[9020]	MAP2K2	--->	YWHAH	0.079981	-0.071802	-
[9021]	FES	--->	SEMA3A	-0.12474	-0.15867	+
[9022]	ABL1	--->	LRP8	0.12881	-0.11877	-
[9023]	EP300	--->	ZBTB37	0.0090612	-0.0074415	+
[9024]	SETDB1	--->	ZNF32	0.038082	-0.022201	+
[9025]	MAPK11	<---	PPP2CA	-0.15489	0.41077	-
[9026]	FGF21	--->	HDAC2	0.15876	-0.0029576	-
[9027]	MAGI1	--->	RAPGEF2	-0.013418	-0.51575	+
[9028]	AKT3	--->	NF1	0.083275	-0.14612	-
[9029]	BIRC5	--->	TP53	0.085273	-0.11396	-
[9030]	HTRA1	--->	NANOG	0.095104	-0.094894	-
[9031]	HNF1A	<---	HNF4A	-0.27075	0.10711	-
[9032]	FZD2	<---	SFRP4	-1.3633	0.73901	+
[9033]	CAMK2G	--->	CREB5	0.72002	-0.41113	+
[9034]	GLI3	--->	WNT10A	0.86373	-1.47	+
[9035]	CHEK1	--->	HES1	0.76849	-0.79121	+
[9036]	BAD	<---	YWHAE	-0.92512	0.50537	+
[9037]	ATF2	<---	PPP1CB	-0.66816	0.44089	+
[9038]	JAG2	--->	MIB1	0.0057516	-0.0066536	-
[9039]	CAMTA2	--->	CTCFL	0.12813	-0.041016	-
[9040]	LCK	<---	PRKCQ	0.097057	0.26845	-
[9041]	FZD9	<---	GNA15	-0.22143	0.24435	-
[9042]	EP300	--->	ITGA7	-0.0012935	-0.0081186	+

[9043]	ITGAV	<---	PLCD4	-0.011601	-0.0076751	-
[9044]	GNB1	<---	MTNR1B	-0.57296	0.69217	+
[9045]	ARRB2	<---	CXCR2	-0.15406	0.070342	-
[9046]	ATP1B4	<---	MAPK1	-0.26091	0.40675	+
[9047]	GLI2	<---	MAP2K1	-0.99235	1.4077	+
[9048]	JUND	<---	STRN3	-0.13437	0.12728	-
[9049]	JUND	<---	NFKBIA	-0.17413	0.079011	-
[9050]	IL4R	<---	THPO	-0.16507	0.040195	+
[9051]	NR3C1	--->	RBM39	0.23448	-0.31347	+
[9052]	HDAC2	--->	ITGAL	-0.068773	-0.072078	+
[9053]	STAT1	--->	UBE2L6	0.23497	-0.29041	-
[9054]	ITGA9	<---	LAMB3	-0.066621	-0.0090065	+
[9055]	ADRBK1	--->	OPRL1	0.25327	-0.21301	+
[9056]	APP	<---	MAPK4	-0.023982	0.030823	-
[9057]	RHOB	--->	TRIO	0.11717	0.0097792	-
[9058]	PIK3R5	<---	TXK	-0.64452	0.79939	-
[9059]	KDR	--->	MAPK11	0.53814	-0.24706	+
[9060]	ID2	<---	TBP	-0.043597	0.00042063	+
[9061]	CAPN5	<---	HTT	-0.12423	0.078688	-
[9062]	CDC25B	<---	CDK1	-0.97911	0.26855	-
[9063]	C1GALT1	<---	MUC2	-0.15003	0.020865	-
[9064]	E2F1	<---	STK24	-0.30098	0.45782	-
[9065]	FZD8	<---	WNT2B	-0.78209	0.63734	+
[9066]	GNB1	<---	HRAS	-0.14023	0.10671	-
[9067]	PLXNA3	<---	RND1	-0.31182	0.50205	-
[9068]	FOS	<---	PPP1R15A	-0.36305	0.49331	-
[9069]	KCNQ3	--->	TRPC4	-0.051301	-0.15927	+
[9070]	ARHGAP22	<---	RHOBTB1	-0.49879	0.38962	-
[9071]	IL5RA	<---	PRKACA	-0.25823	0.38652	+
[9072]	BTRC	--->	RELB	0.19423	-0.10803	+
[9073]	CREB1	<---	NRAS	-0.04566	0.13829	-
[9074]	RAB5B	--->	RIN1	0.18569	-0.1143	-
[9075]	MAP4K4	--->	TLR4	0.03146	-0.10602	-
[9076]	CD8A	--->	IL2RB	-0.033108	-0.046475	+
[9077]	SFRP5	--->	WNT3A	1.0317	-1.1063	+
[9078]	ACVR1C	<---	BAMBI	-1.5601	1.0535	+
[9079]	ACTG1	<---	FLNA	-0.51597	0.40448	+
[9080]	LCP2	<---	SYK	-0.56262	0.30424	+
[9081]	CCKAR	--->	GNAQ	1.1631	-1.1303	+
[9082]	HNF1A	<---	MAPK8	-0.46198	0.60871	+
[9083]	MAPK12	<---	PTPN7	-1.4016	1.2548	+
[9084]	PDGFRB	<---	PLCB2	-0.0062583	0.0047701	-
[9085]	ITGA10	<---	LAMA4	-0.10803	-0.013036	+
[9086]	PPARG	<---	VEGFA	-0.03418	-0.032226	-
[9087]	MAPK11	<---	TAB3	-0.13728	0.23085	+
[9088]	ETV7	<---	MAPK3	-0.15935	0.24561	+
[9089]	FOS	--->	ZKSCAN2	0.16224	0.14761	+
[9090]	ADCY3	<---	CAMK2G	-0.44346	0.58749	+
[9091]	ITGA4	<---	RAP1A	-0.10645	0.030711	+
[9092]	RASA1	--->	TEK	-0.041329	-0.076216	-
[9093]	CREB1	<---	GRB2	-0.14294	0.18611	+
[9094]	ADCY7	<---	RAPGEF3	-0.28599	0.53533	-
[9095]	ADCY7	<---	PRKCE	-0.49495	0.42642	+
[9096]	IL1A	<---	MAPKAPK2	-0.078488	0.15109	-
[9097]	FYN	--->	ITGB6	0.23238	-0.16875	-
[9098]	C3	--->	ESR1	0.0062736	-0.18567	-
[9099]	PRKCH	--->	TJP1	0.14343	-0.1592	+
[9100]	CHUK	<---	TNFRSF10B	-0.16508	0.036616	+
[9101]	SFRP5	--->	WNT10A	1.0317	-1.1063	+
[9102]	FZD2	<---	GNB1	-0.12321	0.10912	-
[9103]	CAMK2A	--->	IFNG	0.19628	-0.17701	-
[9104]	HRAS	<---	RASAL3	-0.41801	0.52695	+
[9105]	EGFR	--->	ITGA6	0.088208	-0.10162	+
[9106]	COMP	<---	ITGA8	-0.021497	-0.014728	-
[9107]	ADRBK2	--->	MTNR1A	0.25327	-0.21301	+
[9108]	CTBP2	--->	HOXD9	0.04296	-0.16727	+
[9109]	SOCS1	--->	STAT5B	0.27422	-0.74698	-
[9110]	EPHA2	--->	TP53	0.045155	-0.055482	-
[9111]	CD40	--->	TRAF2	0.64461	-1.0963	+
[9112]	PRKAR1A	<---	PRKG2	0.19685	0.47393	+
[9113]	EPHA2	--->	FGF7	-0.0069714	-0.050497	-
[9114]	BRCA1	--->	FOSL2	0.60871	-0.44323	-
[9115]	MAP2K6	<---	TLR3	-0.24404	0.037699	+
[9116]	ARHGAP18	<---	RHOB	-1.5245	1.016	-
[9117]	CX3CR1	--->	CXCL13	0.21658	-0.35895	-
[9118]	ANK3	--->	ITPR1	0.17325	-0.10902	+

[9119]	GHR	--->	SH2B1	0.063632	-0.019571	-
[9120]	JAK2	--->	KIT	-0.042947	-0.13382	-
[9121]	DUSP2	--->	MAPK3	1.353	-1.036	+
[9122]	GABBR2	<---	GHRL	-0.29786	0.26779	+
[9123]	ITGA6	--->	TNN	-0.025002	-0.077063	-
[9124]	HIST1H3A	<---	JADE1	-0.47704	0.35089	+
[9125]	COL6A2	<---	ITGB3	-0.01668	-0.0099212	-
[9126]	EDNRA	--->	GNA12	1.1631	-1.1303	+
[9127]	ID3	<---	SMAD5	-0.34215	0.18982	+
[9128]	ADRBK2	--->	DRD4	0.25327	-0.21301	+
[9129]	CSNK1D	--->	YWHAB	0.079981	-0.071802	-
[9130]	PRKCA	--->	TTF1	0.32366	-0.41207	+
[9131]	IFNGR2	<---	RELA	-0.31315	0.30917	-
[9132]	FGR	--->	MLLT4	0.12276	-0.082867	+
[9133]	MAPK8	--->	PLA2G4D	0.18871	-0.84715	+
[9134]	NET1	<---	RHOG	-0.23262	0.26814	-
[9135]	GH2	--->	IL12RB1	0.12248	-0.29036	+
[9136]	CCL15	<---	CCR8	-0.35895	0.21658	-
[9137]	GAB2	<---	PRKD2	-0.40379	0.90301	-
[9138]	PRKCA	--->	SOS1	0.13325	-0.18146	-
[9139]	PLXNA3	<---	RRAS	-0.31182	0.50205	-
[9140]	APOA1	--->	SP3	0.16874	-0.093323	-
[9141]	ARHGAP39	<---	RAC1	-0.82986	0.43536	-
[9142]	ILK	<---	ITGA5	-0.041261	-0.00016819	+
[9143]	GRK6	--->	MTNR1A	0.33635	-0.28786	+
[9144]	ADRA1D	--->	GNA14	1.1631	-1.1303	+
[9145]	SNAI2	<---	TEAD4	-1.1565	1.4387	+
[9146]	NTF3	--->	NTRK3	0.28703	-0.51926	+
[9147]	OPHN1	<---	RHOF	-0.9609	0.7795	-
[9148]	DLG4	--->	RHOG	0.15149	0.1492	+
[9149]	MAPK8	<--->	PRKACA	0.31014	0.31014	-
[9150]	IL3	<---	PDPK1	-0.25757	0.68907	-
[9151]	ARHGEF12	--->	ARHGEF6	-0.0022375	-0.016625	-
[9152]	FGF21	--->	FLT1	0.21774	-0.0015767	+
[9153]	CHN1	<---	RHOV	-0.4978	0.36255	-
[9154]	CTCF	--->	RBPJL	0.060743	0.0044113	+
[9155]	LY9	--->	MEF2C	0.030214	-0.12339	-
[9156]	AMHR2	<---	BMP2	-0.85941	1.3161	+
[9157]	CD3G	<---	RGS1	-0.4184	0.31307	+
[9158]	CRTC2	--->	YWHAB	-0.0021827	-0.10486	-
[9159]	RELA	<---	TRIB3	-0.99472	0.92767	-
[9160]	CD40	--->	STAT5A	0.37351	-0.27091	+
[9161]	CDK5	--->	PPP1R1B	1.2659	-0.92753	+
[9162]	ITGA9	<---	LAMA1	-0.041507	-0.0065623	+
[9163]	HOXB9	<---	TCF7L2	-0.08189	0.2393	+
[9164]	COL5A3	<---	PTK2	-0.11304	-0.10314	-
[9165]	CDK4	<--->	MAPK1	0.31014	0.31014	-
[9166]	FOXO1	--->	GABARAP	1.0193	-0.41286	+
[9167]	RASSF1	<---	RRAS	-0.18202	0.43347	+
[9168]	CSNK1G3	--->	YWHAH	0.047412	-0.04465	+
[9169]	PIK3R3	--->	RAP1A	0.30072	-0.072693	-
[9170]	JUND	--->	PLAT	-0.012928	-0.02137	+
[9171]	FYN	<---	PTPRA	-0.25253	0.28716	-
[9172]	EPHB1	<---	PTPN13	-0.06118	0.062279	+
[9173]	ARHGEF3	<---	RHOF	-0.012974	0.047696	-
[9174]	MYL6B	<---	MYLK2	-0.29873	0.36004	+
[9175]	FZD2	<---	WNT10A	-0.78209	0.63734	+
[9176]	CRK	--->	ITGA10	0.032574	-0.078547	-
[9177]	E2F6	<---	WHSC1	-0.13119	0.16887	-
[9178]	PSMD4	<---	REL	-0.46471	0.18561	-
[9179]	NTRK1	<---	PRKCZ	-0.20474	0.16239	-
[9180]	ARHGEF12	--->	RHOV	0.092701	0.0073174	+
[9181]	ATP1B4	<---	PRKACG	-0.26091	0.40675	+
[9182]	EIF4E	<---	RPS6KB1	-0.66447	0.45012	+
[9183]	AKT3	--->	PIK3CA	0.016173	-0.032345	-
[9184]	DKK1	--->	FZD9	0.3176	-0.35737	+
[9185]	BCAR1	--->	FGG	0.19917	-0.19706	-
[9186]	STAT1	<---	STAT2	-0.13561	-0.1014	-
[9187]	ADCY5	<---	CAMK2G	-0.44346	0.58749	+
[9188]	EXOC2	<---	RALB	-0.73289	1.1482	+
[9189]	GNAO1	<---	KCNJ5	-0.011498	0.05262	-
[9190]	IGSF5	<---	PARD3	-0.13382	0.26059	+
[9191]	CREBBP	<---	PRKACA	-0.006941	0.013728	+
[9192]	CTNNA1	--->	HGF	0.36076	-0.40768	+
[9193]	NCOR1	--->	RBPJ	0.57098	-0.32057	-
[9194]	FOSL1	<---	SRF	-1.0874	1.3477	+

[9195]	ITGA1	<---	LAMC3	-0.03918	-0.0041604	+
[9196]	ARHGAP36	<---	RHOJ	-1.5245	1.016	-
[9197]	ATF3	<---	NFKBIA	-0.083142	0.12202	-
[9198]	RAC1	<---	VAV1	0.1025	0.10902	+
[9199]	CIITA	--->	MEF2A	0.16987	-0.32023	-
[9200]	CRK	<---	MAPK3	-0.19779	0.14625	+
[9201]	KDR	--->	SHC1	0.47733	-0.20389	+
[9202]	IL2RB	<---	JAK2	-0.22119	0.24536	-
[9203]	ARRB2	--->	WNT2B	0.23514	-0.34616	+
[9204]	DLL3	--->	NOTCH2	-0.01015	-0.20955	+
[9205]	STAT2	<---	TYK2	-0.77344	0.58294	+
[9206]	IFNLR1	<---	IL19	-0.20115	0.093836	+
[9207]	FZD7	<---	WNT8B	-0.78209	0.63734	+
[9208]	CEACAM6	--->	ITGA5	-0.017489	-0.054337	+
[9209]	DVL3	--->	NOTCH3	0.47526	-0.54738	+
[9210]	IGKV4-1	<---	PLCG1	-0.022856	0.045786	-
[9211]	CRLF2	<---	IFNG	-0.29693	0.13568	+
[9212]	ATF4	<---	PRKACA	-0.57905	1.0858	+
[9213]	NR3C1	--->	RXRB	1.1084	-1.1389	+
[9214]	EP300	--->	HIST1H2BK	0.01343	-0.01897	+
[9215]	FHL2	<---	TRAF2	-0.47957	0.68404	-
[9216]	KPNA5	<---	RANBP2	-0.19836	0.13604	+
[9217]	JUN	<---	MAPK1	-0.47491	0.66358	-
[9218]	TCF12	<---	ZIC1	-0.059615	0.19618	-
[9219]	GRIK1	<---	RHOJ	-0.38938	0.54247	-
[9220]	COL1A2	--->	PPIB	-0.024575	-0.068802	-
[9221]	CTNNB1	<---	EP300	-0.025162	0.01769	-
[9222]	MAPK13	<---	PPP2R2A	-0.068084	0.2712	-
[9223]	MYO9A	<---	RHOU	-0.54674	0.38058	-
[9224]	PIK3R2	<---	TYK2	-0.31772	0.076383	+
[9225]	RAE1	--->	SRSF5	0.31391	-0.17132	+
[9226]	ARHGAP40	<---	RHOF	-1.5245	1.016	-
[9227]	DVL2	<---	USF2	-0.54184	0.39446	+
[9228]	FZD3	<---	SFRP2	-1.3633	0.73901	+
[9229]	RELA	<---	TRAF5	-0.37539	0.41785	-
[9230]	CREBBP	<---	NR3C1	-0.0067479	-0.0035614	+
[9231]	TCF7L2	--->	ZMIZ1	0.099034	0.065145	+
[9232]	NCSTN	<---	TRAF6	-0.51398	0.6737	-
[9233]	EHD4	--->	NTRK2	-0.016671	-0.14443	+
[9234]	CRKL	--->	RAP1A	0.43868	-0.057401	+
[9235]	KCNJ5	<---	PRKACA	-0.26091	0.40675	+
[9236]	SAG	--->	WNT5B	0.23514	-0.34616	+
[9237]	GAB1	<---	PRKCI	-0.15343	0.32961	-
[9238]	IFNB1	--->	IL2RG	0.17869	-0.34117	+
[9239]	PRKG2	--->	RGS2	0.60012	-0.68337	+
[9240]	BRCA1	--->	MAPK8	0.35592	0.044099	-
[9241]	ARNT	<---	HK1	-0.099193	0.096943	-
[9242]	KDR	<---	PTPN11	-0.086951	-0.026835	-
[9243]	AR	<---	GNAZ	-0.14793	0.11065	-
[9244]	GNA11	--->	PLA2G6	0.21654	-0.15276	+
[9245]	IL6	--->	JUND	0.16075	-0.22115	-
[9246]	IL6R	<---	JAK3	-0.2042	0.16233	-
[9247]	CDC25A	<---	PSME4	-0.30503	-0.030403	+
[9248]	ARHGEF2	<---	RHOG	-0.093742	0.10079	-
[9249]	CD247	--->	CD86	0.041575	-0.060044	-
[9250]	JUN	<---	NOS2	-0.051516	0.033697	-
[9251]	CABLES1	<---	CTNNA1	-0.86838	0.86411	-
[9252]	PRKACB	--->	XBP1	0.40675	-0.26091	+
[9253]	PRKAA1	--->	TSC1	1.3112	-1.0821	+
[9254]	IGHD	<---	PLCG2	-0.027635	0.049708	-
[9255]	PIK3R5	<---	THRA	-0.46306	0.2532	-
[9256]	LAT	<---	PTPN1	-1.1049	0.91854	+
[9257]	CTNNB1	<---	PCDHB11	-0.19204	0.11503	-
[9258]	ADRBK1	--->	HTR5A	0.25327	-0.21301	+
[9259]	ABL1	--->	SHC1	0.040787	-0.1399	+
[9260]	FGG	<---	RAP1A	-0.096109	0.097201	-
[9261]	BCR	--->	JUND	0.0016282	-0.083326	-
[9262]	MAP2K4	<--->	MAP3K8	0.31014	0.31014	-
[9263]	IKBKG	<---	USP7	-0.21587	0.11978	+
[9264]	ATP1B1	<---	PRKX	-0.26091	0.40675	+
[9265]	ACTG2	<---	RHOJ	-0.51168	0.52339	+
[9266]	KIT	--->	PIK3R2	0.17877	-0.10885	+
[9267]	FYN	--->	UNC5B	-0.026267	-0.028765	-
[9268]	HLF	--->	SNAI2	0.23239	-0.096677	+
[9269]	CCL14	<---	CCR7	-0.35895	0.21658	-
[9270]	PIK3CD	<---	PRKCE	-0.06189	0.049814	-

[9271]	ABCB4	--->	JUNB	-0.00086664	-0.064647	-
[9272]	ADRBK1	--->	F2R	0.25327	-0.21301	+
[9273]	POU4F1	<---	PPP1R13B	-0.20652	0.18564	-
[9274]	BATF	--->	ZBTB17	0.096524	0.050503	+
[9275]	NFATC3	--->	PPP3R1	-0.039001	-0.22539	-
[9276]	ERBB2	--->	TP53	0.12382	-0.10743	-
[9277]	CTRB1	--->	MMP3	-0.0056809	-0.18543	+
[9278]	GNA11	<---	TRHR	-1.1303	1.1631	+
[9279]	MAPK8	<---	TGFB3	-0.0067309	0.13651	+
[9280]	IL6	<---	MAPK14	-0.26091	0.40675	+
[9281]	ARHGAP18	<---	RAC2	-1.5245	1.016	-
[9282]	BHLHE40	<---	NOSTRIN	-0.25529	0.20148	-
[9283]	JUP	<---	MMP23A	-0.34868	0.29051	+
[9284]	ATF4	<---	CAMK2D	-0.41113	0.72002	+
[9285]	EIF2AK3	<--->	MAP2K4	0.31014	0.31014	-
[9286]	ITGA6	<---	LAMB2	-0.045111	0.0073643	+
[9287]	AKT2	--->	NF1	0.083275	-0.14612	-
[9288]	PRKCA	--->	RAB4A	0.85615	-0.68511	+
[9289]	LMO2	--->	RXRA	0.09885	-0.40466	-
[9290]	NOS3	<---	PLCG2	-0.0069887	0.0079413	+
[9291]	ARNT	--->	SLC2A1	0.023163	-0.016123	+
[9292]	ARHGAP31	<---	RHOT1	-0.58534	0.26947	-
[9293]	DHX58	<---	STAT1	-0.18492	0.15506	+
[9294]	PPP2R1A	--->	PRKCZ	0.2396	-0.27354	+
[9295]	BIRC2	<---	CASP10	-0.20025	0.019553	-
[9296]	APC2	<---	CSNK1D	-0.10343	0.11983	+
[9297]	PRKACG	--->	SLC8A1	0.24561	-0.15935	+
[9298]	MAP3K14	<---	TNFRSF1A	-0.22533	-0.1429	+
[9299]	NCSTN	--->	NOTCH2	0.16905	-0.13586	+
[9300]	CALML5	--->	CAMKK2	0.41687	-0.23601	+
[9301]	JUND	<---	NOD2	-0.074433	-0.042657	-
[9302]	FZR1	<---	PSMD14	-0.26071	0.27742	+
[9303]	ARHGAP36	<---	RHOG	-1.5245	1.016	-
[9304]	ADH6	<---	EP300	-0.0041449	-0.00068954	+
[9305]	IL13RA2	<---	IL6	-0.18506	0.094523	+
[9306]	FOXA2	--->	FZD2	0.4505	-0.48289	+
[9307]	CASP10	--->	MAPK8	0.62794	-1.3579	-
[9308]	DLG4	<---	GRB2	0.043354	0.21122	-
[9309]	HDAC3	--->	NCOA2	0.021395	-0.0043791	+
[9310]	CCL19	<---	CCR3	-0.35895	0.21658	-
[9311]	F12	<---	GP1BB	-0.21492	0.13077	-
[9312]	TP53	<---	USP7	-0.127	0.12715	+
[9313]	FASLG	--->	IKBKB	0.98959	-0.82132	+
[9314]	FOSB	<---	MAPK11	-0.57905	1.0858	+
[9315]	FLI1	<---	PDGFC	-0.0418	-0.012527	-
[9316]	CTSG	--->	SERPINB13	0.33364	-0.53505	-
[9317]	HDAC1	<---	UBE2I	-0.47317	0.55152	+
[9318]	CBLB	--->	CSF2RA	0.28248	-0.14259	+
[9319]	GRB2	--->	IL10RA	0.17317	-0.077015	-
[9320]	CSK	--->	MEF2C	0.43969	-0.37646	-
[9321]	MYH7	<---	NFATC2	-0.17272	0.066517	+
[9322]	RANBP2	--->	SRSF5	0.34767	-0.31854	+
[9323]	CREB1	--->	TGFB2	0.063524	-0.11958	+
[9324]	MYLK4	<---	PAK6	-1.2674	1.5523	+
[9325]	GH1	--->	IL12RB1	0.12248	-0.29036	+
[9326]	CRK	--->	ITGA2	0.018691	-0.085251	-
[9327]	KCNQ4	--->	TRPC3	0.011076	-0.12465	-
[9328]	HMGB1	<---	NFKB2	-0.24723	0.25802	-
[9329]	KLRC2	--->	VAV2	0.06651	-0.018805	+
[9330]	GNB3	<---	PIK3CD	-0.010082	0.0028834	-
[9331]	EML4	<---	HRAS	-0.21926	0.18651	-
[9332]	GYS2	<---	PRKAA2	-1.2533	0.76059	+
[9333]	GRIN1	<---	RHOJ	-0.38938	0.54247	-
[9334]	BMP4	<---	SMAD5	-0.24379	0.060804	-
[9335]	JUP	<---	MMP19	-0.26058	0.11034	+
[9336]	CD70	--->	ZEB1	0.27525	-0.22352	-
[9337]	GNAO1	<---	PIK3CG	0.00043444	0.0052476	-
[9338]	MAP3K1	--->	PAK3	0.032393	0.0052037	-
[9339]	CSNK1E	--->	FOXO4	0.2499	-0.22085	+
[9340]	JAK2	<---	MAP3K11	-0.13936	0.19201	-
[9341]	DAND5	--->	MYC	0.10261	-0.29838	+
[9342]	GNGT2	<---	MAPK14	-0.41411	0.50061	-
[9343]	BAMBI	--->	FZD9	0.43771	-0.51806	+
[9344]	CTNND1	<---	RAC3	-0.32991	0.29511	-
[9345]	HTT	--->	RAC3	0.21591	-0.31065	-
[9346]	GRB2	--->	TREM2	0.13256	-0.17782	-

[9347]	KIR2DL1	<---	PTPN6	-0.32886	0.27078	-
[9348]	ATF6B	<---	CAMK4	-0.57905	1.0858	+
[9349]	RBBP7	<---	UBTF	-0.097857	0.19902	-
[9350]	RHOG	--->	STARD13	0.10338	-0.50393	-
[9351]	PRL	--->	PRLR	0.073187	-0.17721	+
[9352]	ARHGAP5	<---	RHOT1	-0.0027736	0.33344	-
[9353]	PDX1	<---	PRKACG	-0.46198	0.60871	+
[9354]	HSP90AA1	--->	PLK1	0.41693	-0.26442	-
[9355]	NOTCH3	<---	RXRA	-0.082121	0.096162	+
[9356]	ACTR2	<---	MMP16	-0.29263	0.085904	+
[9357]	DVL2	<---	WNT9A	-0.54867	0.38842	+
[9358]	NCK1	<---	PDGFRB	-0.07638	0.17777	+
[9359]	GNB4	--->	GRM3	0.15376	-0.10866	-
[9360]	CRTC2	<---	PRKAG1	-1.0463	1.4287	+
[9361]	GUCY1B3	<---	NFIC	-0.41624	0.3875	+
[9362]	EGR1	<---	TIMP2	-0.15205	0.28002	-
[9363]	CRK	--->	GFRA1	0.0021814	-0.033607	-
[9364]	IGFALS	--->	MMP2	0.11468	-0.31874	-
[9365]	APP	<---	MAPKAPK5	-0.023982	0.030823	-
[9366]	CCL1	<---	CCR3	-0.35895	0.21658	-
[9367]	CCR6	<---	GRK7	-0.28786	0.33635	+
[9368]	BCAP31	<---	CASP8	-0.49317	0.29546	+
[9369]	BCAR1	--->	FN1	0.23135	-0.23944	-
[9370]	CSNK1A1	--->	SUFU	0.58123	-0.48787	+
[9371]	AKT2	--->	BCL2L1	0.30677	-0.44298	+
[9372]	PTEN	<---	VAV1	0.00066794	0.058606	-
[9373]	AKT1	--->	NF1	0.083275	-0.14612	-
[9374]	MMP9	<---	SRC	-0.33465	0.39283	+
[9375]	IFNG	<---	REL	-0.39685	0.0011643	+
[9376]	EPO	<---	STAT1	-0.32133	0.25961	-
[9377]	CBLB	--->	PLCG2	0.0046457	-0.010821	+
[9378]	CD3D	<---	ZAP70	-0.22013	0.087709	-
[9379]	MAPK13	--->	NFATC3	0.92767	-0.99472	+
[9380]	BIRC3	--->	TRAF3	0.71646	-0.53216	+
[9381]	EXOC2	<---	RAB13	-0.73289	1.1482	+
[9382]	CCNT2	<---	FOXA2	-0.6022	0.58764	-
[9383]	ETS1	<---	KDR	-0.02553	0.20121	-
[9384]	CASP1	<---	NLRP1	-0.2888	-0.10336	+
[9385]	AGTR2	--->	GNAQ	0.65842	-0.64681	+
[9386]	PLAU	--->	VLDLR	0.11633	-0.090584	-
[9387]	JAK2	--->	KRAS	0.579	-0.13016	+
[9388]	RICTOR	--->	TSC2	0.024861	-0.033748	-
[9389]	CDH1	<---	MMP1	-0.24168	-0.0076207	+
[9390]	FGF3	--->	PDGFRB	0.48772	-0.57252	+
[9391]	PPARGC1A	--->	WNT10B	0.11798	-0.15694	-
[9392]	PPARA	<---	PRKACG	-0.30308	0.29076	+
[9393]	PTK2	<---	RAP1A	-0.14524	-0.084837	+
[9394]	ADCY9	<---	PRKCG	-0.49495	0.42642	+
[9395]	CCL5	<---	CEBPA	-0.14964	0.055907	+
[9396]	MAP3K1	<---	TRAF6	-0.24426	0.71285	+
[9397]	ACVR2A	<---	GDF5	-0.85941	1.3161	+
[9398]	PDGFD	--->	PLG	-0.017531	-0.057382	-
[9399]	CUL1	--->	PSMD7	0.065557	-0.142	-
[9400]	ERBB4	--->	PLCG1	0.05141	-0.048506	+
[9401]	GNAI2	<--->	GNAT2	0.064764	0.064764	-
[9402]	AKT1	--->	NFKB1	0.086648	-0.068126	-
[9403]	GNAI2	<---	PRKCA	-0.38932	0.32346	+
[9404]	CSNK1G2	--->	FZD6	0.13819	-0.12618	-
[9405]	CREB1	<---	PRKG1	-0.74309	0.72637	+
[9406]	CBLC	--->	IFNLR1	0.99663	-0.58388	+
[9407]	MAPK3	--->	MITF	0.60415	-0.53416	+
[9408]	CREB3L4	<---	CREBBP	-0.010885	0.015595	-
[9409]	SNAI2	<---	TCF7	-0.018993	0.22714	+
[9410]	PLCG1	--->	RGS17	0.026624	-0.0038693	-
[9411]	JUND	--->	TAL1	0.071708	-0.13126	+
[9412]	CAMK2B	--->	CREB3	0.72002	-0.41113	+
[9413]	GHSR	--->	GNAI3	1.1631	-1.1303	+
[9414]	EPHB2	--->	HRAS	0.022956	0.0078303	+
[9415]	ACTN3	--->	MYL1	0.10182	0.0089705	-
[9416]	MYC	<---	TP53	-0.047202	-0.017656	-
[9417]	PIK3CB	--->	TPTE	0.019355	-0.032639	-
[9418]	OBSCN	<---	RHOG	-0.11324	0.22832	-
[9419]	LCK	--->	MAP4K1	0.21894	-0.21273	+
[9420]	GFI1	<---	SPI1	-0.059737	0.13985	+
[9421]	FIGF	--->	PIK3R1	0.058137	-0.13402	+
[9422]	AKT2	--->	RASAL2	0.077205	0.029269	-

[9423]	ITGA3	--->	THBS4	0.10037	-0.06402	-
[9424]	AAAS	--->	SRSF4	0.31391	-0.17132	+
[9425]	ATF6B	<---	PPP1CB	-1.0855	0.69008	+
[9426]	ERBB2	<---	PRKD1	-0.031152	0.11487	-
[9427]	CBX5	--->	SMARCA4	-0.011409	-0.023317	-
[9428]	KIT	--->	NRAS	0.37837	-0.22929	+
[9429]	AMHR2	<---	BMP6	-0.85941	1.3161	+
[9430]	MYC	<---	RBPJ	-0.19719	0.08503	-
[9431]	ATF2	<---	PRKG2	-0.59883	0.73125	+
[9432]	RAC3	--->	VAV2	0.058196	0.018587	-
[9433]	PRKCA	<---	RPS6KB1	-0.1519	0.061013	-
[9434]	GNAO1	<---	HTR5A	-1.1303	1.1631	+
[9435]	MET	<---	PTPN2	-0.28781	0.44739	-
[9436]	EIF4A1	<---	PAN3	-0.1466	0.23456	+
[9437]	MET	--->	PDGFD	0.12914	-0.19534	-
[9438]	JUN	<---	NR2F1	-0.20904	0.10351	-
[9439]	ADCY3	<---	KCNQ1	-0.23711	0.26335	+
[9440]	ACAP1	<---	PLD1	-0.027236	0.0021311	+
[9441]	IGF1R	<---	VEGFA	-0.039425	0.18474	+
[9442]	GRM5	--->	POU3F2	-0.012803	-0.016973	-
[9443]	COL2A1	--->	ITGA9	0.0052169	-0.090529	+
[9444]	PRKX	--->	SCN1A	0.61419	-0.44517	+
[9445]	FOXO1	<---	STK11	-0.22085	0.2499	+
[9446]	CCL28	<---	CCR2	-0.35895	0.21658	-
[9447]	FOS	<---	NFIA	-0.16257	0.22662	-
[9448]	EDNRB	--->	PLCB1	0.081663	-0.121	+
[9449]	IL19	--->	IL2RB	-0.0077042	-0.055821	+
[9450]	PIK3CD	<---	PRKCB	-0.06189	0.049814	-
[9451]	IGF1R	--->	SHC2	0.37655	-0.22424	+
[9452]	COL4A3	<---	PTK2B	-0.11304	-0.10314	+
[9453]	KCNC2	<---	PRKACG	-0.44517	0.61419	+
[9454]	BIRC2	<---	TEAD3	-0.1016	0.046631	+
[9455]	PRKCB	--->	TTF1	0.32366	-0.41207	+
[9456]	MYC	<---	ZNF587	-0.08008	0.017786	-
[9457]	ALS2	<---	RAB5A	-0.046927	0.066828	-
[9458]	MEF2C	<---	ZNF263	-0.025114	0.03052	+
[9459]	ACTA1	<---	MMP2	-0.29263	0.085904	+
[9460]	DUSP3	--->	MAPK11	1.4184	-1.4202	+
[9461]	AR	<---	KLK3	-0.16924	0.10365	-
[9462]	GRK5	--->	SSTR2	0.33635	-0.28786	+
[9463]	ARHGAP23	<---	RHOG	-0.46335	0.36352	-
[9464]	PRKCE	--->	SOS2	0.13325	-0.18146	-
[9465]	MAP2K3	<---	MAP3K2	-0.22507	0.58679	-
[9466]	IKBKG	<---	MAP2K4	-0.45312	0.62218	-
[9467]	IL12RB1	<---	JAK3	-0.38855	0.16968	-
[9468]	AXL	--->	FOS	0.10595	-0.11082	-
[9469]	ADIPOQ	<---	PPARA	-0.091347	-0.010433	-
[9470]	H2AFX	<---	YY1	-0.0001508	0.10412	+
[9471]	BTK	--->	PIK3CD	0.026127	-0.010992	-
[9472]	CCL4	<---	CXCR1	-0.35895	0.21658	-
[9473]	RHOQ	--->	VAV2	0.058196	0.018587	-
[9474]	CSF1R	<---	FGF16	-0.64493	0.77791	+
[9475]	LCP2	<---	MAP3K7	-0.44923	0.039497	-
[9476]	ITGB1	--->	LAMA2	-0.0055713	-0.0064655	-
[9477]	ACTN2	--->	GRIA3	0.12285	-0.10108	-
[9478]	ATP1B4	<---	MAPK3	-0.26091	0.40675	+
[9479]	CBLC	--->	CSF3R	0.18116	-0.091138	+
[9480]	CCL3	<---	CCR8	-0.35895	0.21658	-
[9481]	JUNB	--->	TCF12	0.071708	-0.13126	-
[9482]	FGFR3	--->	SHC1	0.23069	-0.27006	+
[9483]	MAPK9	--->	PLA2G4D	0.18871	-0.84715	+
[9484]	HES5	<---	RBPJL	-1.5472	0.9667	+
[9485]	FZD7	<---	WNT6	-0.78209	0.63734	+
[9486]	IL23A	--->	IL7R	0.12248	-0.29036	+
[9487]	EP300	--->	MYF5	0.024715	-0.02899	+
[9488]	CCNH	--->	TP53	0.064257	-0.038691	-
[9489]	ARHGAP32	<---	RHOV	-0.48747	0.29572	-
[9490]	ITGB2	<---	PRKD1	-0.090796	0.12979	+
[9491]	GRM4	<---	KCNJ5	-0.25173	0.20895	+
[9492]	MYC	<---	TBL1X	-0.14362	0.031066	-
[9493]	IGFBP3	--->	PLG	-0.032112	-0.04269	-
[9494]	CX3CR1	<---	JAK2	-0.12268	-0.039248	-
[9495]	PHKG1	<---	PPP1CB	-0.12622	0.14153	+
[9496]	ECT2	<---	RAC3	-0.012974	0.047696	-
[9497]	IL10RA	<---	SOS2	0.049531	0.056401	-
[9498]	CD8A	<---	LCP2	-0.16182	-0.016269	-

[9499]	NRAS	--->	RPS6KA2	0.39663	-0.23906	-
[9500]	FOS	<---	KAT5	-0.098546	0.15465	-
[9501]	RAC1	--->	RAP1GDS1	0.29511	-0.32991	-
[9502]	GNB3	--->	PLCB1	0.016962	-0.028756	+
[9503]	CHRM2	<---	PKN2	-0.1993	0.11589	-
[9504]	NGEF	<---	RHOQ	0.091479	0.13323	-
[9505]	HNF1B	<---	SPI1	-0.24396	0.15908	+
[9506]	BTK	--->	SIGIRR	0.27253	-0.20751	-
[9507]	NGF	<---	RAC1	-0.48322	0.53989	-
[9508]	ITGA2	<---	LAMB1	-0.1017	-0.0065074	+
[9509]	CD3E	<---	MAP3K3	-0.11065	0.58996	-
[9510]	CD3E	<---	FYB	-0.07494	0.025087	-
[9511]	ARHGAP36	<---	RHOD	-1.5245	1.016	-
[9512]	ARHGAP24	<---	RHOB	-0.49576	0.47865	-
[9513]	CCNH	<---	ERCC2	-0.66943	0.70224	+
[9514]	MEF2C	<---	MSX2	-0.1766	-0.00025604	-
[9515]	ARHGEF17	--->	RHOT2	0.33144	-0.31781	+
[9516]	C1R	<---	C2	-0.017809	0.006907	-
[9517]	MYO9A	<---	RHOG	-0.54674	0.38058	-
[9518]	EBI3	<---	TAF1	-0.29036	0.12248	+
[9519]	PLCG1	<---	PRKD3	0.016382	0.01803	-
[9520]	PORCN	--->	WNT10A	0.38949	-0.56891	+
[9521]	COL8A2	--->	PPIB	-0.016005	-0.04283	-
[9522]	DKK1	--->	WNT7A	0.54164	-0.69755	+
[9523]	MAPK9	--->	RALGDS	0.45932	-0.42161	-
[9524]	FGFR1	<---	VEGFA	-0.58971	0.55225	+
[9525]	CRK	--->	EPHB1	-0.040532	-0.065642	-
[9526]	GRB10	--->	GRB2	0.30731	0.025146	-
[9527]	BCL3	<---	KLF6	-0.064911	0.62238	-
[9528]	EIF4A2	--->	EIF4E	0.27686	-0.32489	+
[9529]	ESR1	--->	HDAC7	0.23955	-0.3115	+
[9530]	CSNK1G2	--->	WNT6	0.50785	-0.4784	-
[9531]	FAS	--->	FOS	0.2364	-0.40961	-
[9532]	BTK	--->	IKBKB	0.30572	-0.21112	+
[9533]	FGF10	<---	KRAS	-0.25726	0.38422	-
[9534]	NFKB1	--->	PTAFR	0.47827	-0.43941	+
[9535]	ARAF	<---	PRKCA	-0.23412	0.18571	+
[9536]	EGR1	<---	MAPK1	-0.67282	1.0991	-
[9537]	SETDB1	--->	ZNF551	0.16497	-0.10617	+
[9538]	ADRBK1	--->	XCR1	0.25327	-0.21301	+
[9539]	ERBB2	--->	RNF41	0.1225	-0.07312	-
[9540]	TAB1	--->	TRADD	0.15955	-0.17273	-
[9541]	MYH2	<---	RAB11A	-0.01596	0.026206	+
[9542]	ADCY6	<---	CAMK2A	-0.44346	0.58749	+
[9543]	RHOB	--->	SEMA4D	0.22192	-0.16793	-
[9544]	CCL15	<---	CXCR1	-0.35895	0.21658	-
[9545]	EPHA2	--->	NGEF	0.030553	-0.091172	+
[9546]	MYL5	--->	ROCK1	0.14447	-0.013535	-
[9547]	PRKCZ	--->	RAF1	0.1479	-0.22585	+
[9548]	PFKFB4	--->	PRKAG3	-0.012105	-0.096604	-
[9549]	GATA1	--->	STK4	0.14898	-0.039777	+
[9550]	AKT1	--->	PIK3CB	0.016173	-0.032345	-
[9551]	ARF4	<---	HTT	-0.49203	0.32422	-
[9552]	DAB1	--->	RAP1A	0.2317	-0.084216	+
[9553]	ABI1	--->	EGF	0.22216	-0.19567	-
[9554]	FGR	--->	PIK3CB	0.084876	-0.06481	+
[9555]	CXCR4	--->	RALB	0.12209	-0.13328	+
[9556]	APBB1IP	<---	BCAR1	-0.11016	0.12155	-
[9557]	BMP1	<---	COL5A1	-0.19386	0.13754	-
[9558]	OCRL	<---	RHOU	-0.92575	0.61511	-
[9559]	ARHGAP12	<---	RHOQ	-0.36596	0.22724	-
[9560]	RPS6	<---	TAF1	-0.23161	0.25527	+
[9561]	CALM1	--->	DLG1	0.15323	-0.053695	+
[9562]	GRK6	--->	HTR2B	0.33635	-0.28786	+
[9563]	GNB4	<---	HTR5A	-0.92616	1.1575	+
[9564]	CDC20	<---	PPP3CA	-0.55798	0.43759	+
[9565]	ARIH1	<---	UBE2E1	-0.20492	0.13119	-
[9566]	CSNK1A1	--->	WNT8A	0.83912	-0.78694	-
[9567]	MEF2C	--->	RB1	0.0269	-0.12549	-
[9568]	EGR1	<---	SIRT1	-0.15205	0.28002	-
[9569]	EP300	--->	EYA2	0.00067605	-0.0050208	+
[9570]	PIK3CG	<---	PLCG1	-0.0072803	0.0083279	-
[9571]	HNF4A	--->	PCK2	0.12096	-0.27233	+
[9572]	LIPE	<---	PRKAR2B	-0.52243	0.57268	+
[9573]	HMOX1	--->	NFIC	-0.044792	-0.073047	-
[9574]	FZD4	<---	GNB1	-0.12321	0.10912	-

[9575]	RBBP4	<---	SMAD3	-0.083613	-0.016256	-
[9576]	RHOA	<---	S1PR3	-0.13328	0.12209	+
[9577]	GNA14	<---	NTSR1	-1.1303	1.1631	+
[9578]	FGR	--->	SHC4	0.48646	-0.49397	+
[9579]	DRD2	<---	GRK5	-0.28786	0.33635	+
[9580]	CNOT1	--->	EIF4E	0.22028	-0.31444	+
[9581]	ITK	--->	VAV2	0.11535	-0.012489	-
[9582]	GNA13	--->	RAC1	1.2694	-0.93465	+
[9583]	FOXA1	--->	ID2	0.81123	-0.8599	+
[9584]	EGR1	<---	TNF	-0.16564	0.25366	-
[9585]	ARHGAP32	<---	RHOBTB2	-0.29676	0.17827	-
[9586]	IGKV4-1	--->	VAV3	0.064425	-0.018037	+
[9587]	DVL2	<---	RAC1	-0.57588	0.46415	-
[9588]	GLI3	--->	WNT16	0.86373	-1.47	+
[9589]	ITGB4	--->	TNN	-0.028909	-0.031286	-
[9590]	ATF4	--->	BDNF	1.0339	-0.75934	+
[9591]	BZRAP1	--->	DAND5	-0.01257	-0.032704	-
[9592]	JUND	<---	PPARG	-0.12012	0.051652	-
[9593]	PLCB1	<---	PRKCG	-0.19559	0.16311	-
[9594]	IL2RG	<---	KRAS	-0.11176	0.099979	-
[9595]	PTP4A1	<---	SRC	-0.90499	1.108	+
[9596]	STYXL1	--->	ZBTB33	0.43438	-0.31658	-
[9597]	THRB	--->	WNT4	0.24686	-0.46784	+
[9598]	ITGB3	--->	PI4KA	-0.0065677	-0.016061	+
[9599]	NUP37	--->	SRSF9	0.31391	-0.17132	+
[9600]	EGFR	--->	RAC1	0.28807	-0.18972	+
[9601]	PDPK1	--->	RPS6KA2	1.6772	-1.9829	+
[9602]	CDH1	<---	MMP28	-0.24168	-0.0076207	+
[9603]	ITGB4	<---	LAMC3	-0.017112	0.0034424	+
[9604]	IKZF3	<---	JAK3	-0.17681	0.20949	+
[9605]	KRAS	<---	NR3C2	-0.076666	-0.012781	+
[9606]	ARHGAP12	<---	RHOC	-0.36596	0.22724	-
[9607]	EPOR	<---	PLCG1	-0.019633	0.030536	-
[9608]	HSPG2	<---	PLG	-0.0011762	0.0059783	+
[9609]	PAK2	<---	SRC	-0.81947	0.81303	+
[9610]	LCP2	<---	TEC	-0.13386	0.071838	+
[9611]	FADD	<---	TICAM2	-0.38805	0.18343	+
[9612]	FGR	--->	GNAZ	0.67161	-0.44236	+
[9613]	PRKACA	<---	RASD1	-0.20304	1.2491	+
[9614]	APC	--->	TCF7L2	0.023904	-0.0096697	+
[9615]	CD79B	--->	DAPP1	0.23467	-0.09549	+
[9616]	DTX3	--->	NOTCH2	0.015993	0.011922	-
[9617]	CCR4	<---	GRK6	-0.28786	0.33635	+
[9618]	SHC3	<---	ZAP70	-0.71996	0.36756	+
[9619]	IFNGR2	<---	NOS2	-0.021673	0.042707	-
[9620]	NEDD4L	<---	WNK1	-0.80961	0.71536	+
[9621]	CREBBP	--->	TFRC	-0.0015178	-0.0036136	+
[9622]	MYBPC2	<---	MYL4	-0.12286	0.036048	+
[9623]	GNB1	<---	OPN1LW	-0.57296	0.69217	+
[9624]	ARHGAP17	<---	RHOC	-0.92575	0.61511	-
[9625]	ACTR2	<---	MMP23A	-0.43788	0.38294	+
[9626]	COL2A1	--->	ITGA2	0.0021195	-0.1162	+
[9627]	AR	<---	NR2C2	-0.10608	0.0052826	-
[9628]	AKT1	--->	CREB5	0.82132	-1.3257	+
[9629]	PPP2CB	--->	YWHAQ	0.18205	-0.13344	-
[9630]	CASP8	--->	EZR	0.071797	-0.25067	-
[9631]	GNB1	--->	WNT8B	0.19155	-0.26814	-
[9632]	CDKN1A	--->	FOS	0.24781	0.024447	-
[9633]	IGF1	--->	PDGFRA	0.30848	-0.31438	+
[9634]	CXCL1	<---	MYD88	-0.64179	0.53642	+
[9635]	CSF2RA	<---	IFNG	-0.24027	0.060151	+
[9636]	DLG4	--->	GRIK2	0.19945	-0.095793	+
[9637]	DAND5	<---	DDIT4	-0.090335	0.038246	-
[9638]	HDAC2	<---	ZNF587	0.069686	0.15826	-
[9639]	MYO1C	<---	RHOQ	-0.038582	0.076038	+
[9640]	MAPK1	--->	RELA	0.92767	-0.99472	+
[9641]	ARF1	<---	ARFGAP1	-0.13279	-0.12595	+
[9642]	DSN1	--->	PLK1	0.26347	-0.049579	-
[9643]	PIK3CB	<---	PRKCE	-0.06189	0.049814	-
[9644]	GRB2	<---	SMPD1	-0.071438	-0.041513	-
[9645]	PIK3CG	<---	RAP1A	-0.015102	0.019966	+
[9646]	CDC42	<---	VEGFA	-0.00043626	0.020385	+
[9647]	IL10	--->	IL12RB1	0.12248	-0.29036	+
[9648]	GNAI1	<---	PXN	-0.057204	0.12598	-
[9649]	CCND1	<---	CDKN2C	-0.066649	0.24757	+
[9650]	IL1A	<---	MAPK12	-0.078488	0.15109	+

[9651]	CXCL1	<---	MAPK14	-0.36767	0.50554	+
[9652]	SHC1	<---	TEC	-0.16897	0.085968	-
[9653]	CDH2	<---	PIK3CA	-0.056703	0.0081601	-
[9654]	CDK5	--->	CREB1	0.65748	-0.35231	-
[9655]	ADCY2	<---	CAMK2A	-0.44346	0.58749	+
[9656]	PSMD7	--->	SKP2	-0.057449	-0.14741	+
[9657]	RB1	<---	SKP2	-0.1208	0.036397	+
[9658]	AR	<---	DAND5	-0.17578	0.12141	+
[9659]	IL11RA	--->	SOCS1	-0.089456	-0.13382	-
[9660]	CXCL12	<---	RALB	-0.14534	0.17483	-
[9661]	CALM1	--->	CNGA1	0.0065234	-0.029729	+
[9662]	DUSP12	--->	MAPK13	1.4184	-1.4202	+
[9663]	PAK3	<---	RAC1	-1.1901	0.96651	-
[9664]	CDK7	--->	RARG	0.29076	-0.30308	+
[9665]	CSF3R	<---	PTPN6	-0.16302	0.17112	+
[9666]	HCK	--->	S1PR1	0.55948	-0.3948	-
[9667]	GH2	<---	MAPK1	-0.26091	0.40675	-
[9668]	CXCR1	<---	GRK6	-0.28786	0.33635	+
[9669]	GNGT2	--->	PIK3CG	-0.01029	-0.019283	+
[9670]	FGD4	<---	RHOA	-0.12937	0.14242	-
[9671]	EPHA3	<---	RASA1	-0.090332	-0.087435	-
[9672]	PDE3B	<---	PRKACA	-0.95055	1.4803	+
[9673]	IL1A	<---	MAPK10	-0.078488	0.15109	+
[9674]	PLA2G6	<---	PRKCG	-0.21133	0.19522	+
[9675]	CD14	--->	DAND5	0.088861	-0.26232	-
[9676]	ARHGEF6	<---	RHOJ	-0.041777	0.050621	-
[9677]	CHRM2	--->	KCNJ3	0.11779	-0.2522	-
[9678]	CBLB	--->	FCER1G	0.16735	-0.086525	-
[9679]	CDC40	<---	RANBP2	-0.18733	0.18457	+
[9680]	MAPK11	<---	NCK1	-0.31116	0.22669	+
[9681]	ADAM10	--->	DLL3	0.084492	-0.11765	+
[9682]	NFKBIA	--->	TCF12	0.33109	-0.44329	-
[9683]	KCNQ2	--->	TRPC6	-0.051301	-0.15927	+
[9684]	BLK	--->	GNAZ	0.67161	-0.44236	-
[9685]	MYOG	<---	POU5F1	-0.32236	0.23888	+
[9686]	GLI3	--->	HDAC1	0.15826	0.069686	-
[9687]	JAK2	--->	SH2B3	0.40523	-0.44409	-
[9688]	CRKL	--->	GAB1	0.19567	-0.41522	-
[9689]	CRK	--->	RAP2A	0.43868	-0.057401	+
[9690]	FHL2	--->	SRF	0.15731	-0.11172	-
[9691]	CREB3L3	<---	PRKX	-0.57905	1.0858	+
[9692]	EHF	--->	POU2F2	0.095387	-0.14907	-
[9693]	HTR1E	<---	NPY	-1.3205	0.89835	+
[9694]	CACNB3	<---	CAMK2D	-0.047052	0.14731	+
[9695]	FN1	<---	RAP1A	-0.28606	0.2414	-
[9696]	CAMK4	--->	CREB3L4	1.0858	-0.57905	+
[9697]	NOS1	--->	PPP3CC	-0.0017651	-0.013255	-
[9698]	SIX5	--->	ZNF189	0.078944	0.049909	+
[9699]	HEY1	<---	WDTC1	-0.4894	0.39662	-
[9700]	CTNND1	<---	YES1	-0.56108	0.71707	+
[9701]	NTRK2	--->	SHC1	-0.057473	-0.1181	+
[9702]	CTNND1	<---	HGF	-0.20881	0.090183	-
[9703]	MYH4	<---	RALA	-0.01596	0.026206	-
[9704]	ACTG1	<---	MMP2	-0.29263	0.085904	+
[9705]	PRKD2	--->	STAT5B	0.99262	-0.67977	-
[9706]	ITCH	--->	NOTCH1	0.17343	-0.13272	+
[9707]	FBXW7	--->	PBX3	0.31006	-0.27909	-
[9708]	ANAPC2	--->	CCNB1	0.54369	-0.49444	-
[9709]	DAPP1	--->	VAV2	0.025512	0.025193	-
[9710]	PPP2R1B	<---	STAT1	-0.13717	0.099176	-
[9711]	JAK2	--->	MAP3K1	0.25727	-0.62209	+
[9712]	CTNNA1	--->	MMP16	0.38719	-0.55042	-
[9713]	CCR2	--->	CXCL1	0.21658	-0.35895	-
[9714]	OPRM1	--->	REST	0.44069	-0.36137	-
[9715]	ACTG1	<---	FLNC	-0.51597	0.40448	+
[9716]	ITGAV	<---	LAMB3	-0.066621	-0.0090065	+
[9717]	ARHGAP9	<---	RHOJ	-0.36596	0.22724	-
[9718]	CD3G	--->	GNAZ	0.05262	-0.011498	-
[9719]	CACNA1D	<---	PPP2CA	-0.27679	0.37996	+
[9720]	IL20RB	<---	PRL	-0.087402	0.20113	+
[9721]	GNA11	--->	LRP5L	0.16892	-0.098359	-
[9722]	KAT6B	--->	PBX3	0.15908	-0.24396	-
[9723]	PPP2R3B	--->	RYR2	0.064649	-0.042259	+
[9724]	BUB1	--->	HDAC2	0.82707	-0.52917	-
[9725]	IKBKB	--->	PEBP1	0.052703	-0.022222	-
[9726]	PRKAG3	<---	STRADA	-1.2499	1.1791	+

[9727]	CTNNA1	--->	DNM2	0.19123	-0.17557	-
[9728]	CAMK2B	--->	CDC25C	0.19972	-0.63064	+
[9729]	GRK7	--->	SSTR3	0.33635	-0.28786	+
[9730]	CDC20	<---	UBE2E1	-0.2601	0.27426	+
[9731]	CSNK1G2	--->	FZD10	0.33941	-0.31414	-
[9732]	CDC42	--->	ITGA1	0.0095864	-0.10112	-
[9733]	BHLHE41	<---	NPAS2	-0.22744	-0.07309	+
[9734]	GNB1	--->	LRP6	0.34224	-0.24336	-
[9735]	AURKB	--->	PSMD1	0.57437	-0.5963	-
[9736]	PAK6	<---	RAF1	-0.76991	0.68927	-
[9737]	CBX5	--->	E2F1	0.039909	-0.13325	-
[9738]	ABR	<---	RAC2	-0.20131	0.10561	-
[9739]	HAP1	<---	RHOJ	-0.11932	0.38072	-
[9740]	BTRC	--->	RBX1	0.29036	-0.13525	-
[9741]	MAPK11	--->	PHC3	0.40675	-0.26091	+
[9742]	FOXA1	--->	SSTR5	0.55395	-0.58151	+
[9743]	MYH3	--->	MYL2	0.18892	-0.22718	-
[9744]	HNF4G	--->	STAT5A	0.27087	-0.22437	+
[9745]	ATF2	<---	CAMK2B	-0.3671	0.58576	+
[9746]	COL4A3	--->	ITGA9	-0.013036	-0.10803	+
[9747]	ADRA1A	<---	ADRBK1	-0.21301	0.25327	+
[9748]	NGFR	--->	PRDM4	0.2505	-0.40439	+
[9749]	PRKCB	--->	TYR	0.32366	-0.41207	+
[9750]	CSF1	--->	PDGFRB	0.38317	-0.24627	+
[9751]	AKT1	--->	ITGA6	0.060159	-0.13176	-
[9752]	PIK3CG	<---	SRC	-0.10504	0.14175	+
[9753]	CCND2	<---	TEAD4	-0.6264	0.69438	+
[9754]	CACNA1C	<---	PPP3CA	-0.51246	0.42555	+
[9755]	ITSN1	--->	RAC3	0.16745	-0.11028	+
[9756]	ACTG1	<---	MYL5	-0.10988	0.043745	+
[9757]	ADRA2C	<---	GRK6	-0.28786	0.33635	+
[9758]	EIF4A2	--->	EIF4B	0.23551	-0.20283	-
[9759]	CREB1	<---	HLA-DQA2	-0.2783	0.25054	-
[9760]	BAMBI	--->	SMAD7	0.16225	-0.34174	+
[9761]	GLI1	<---	PSME4	-0.077956	0.20686	+
[9762]	GNAL	<---	HTR4	-1.1303	1.1631	+
[9763]	CD19	--->	IGHD	0.45578	-0.44842	-
[9764]	NFKB1	--->	TNC	0.10097	-0.11447	+
[9765]	CTBP2	--->	VAX1	0.04296	-0.16727	+
[9766]	CDC20	<---	SKP1	-0.060578	0.059668	+
[9767]	ITGA11	<---	RELN	-0.10572	-0.014742	+
[9768]	CXCL6	<---	CXCR3	-0.35895	0.21658	-
[9769]	MAPK3	<---	PPP2R1B	-0.25679	0.26693	+
[9770]	ABL1	--->	WASF2	0.1212	-0.15616	+
[9771]	BCL3	<---	POLDIP3	-0.058092	0.17511	-
[9772]	RHOC	--->	TAGAP	1.016	-1.5245	-
[9773]	HSPA8	--->	SMAD2	0.3687	-0.49632	+
[9774]	FGF8	--->	FGFR2	0.40218	-0.67149	-
[9775]	FOXF1	--->	GABPA	0.19808	-0.23065	-
[9776]	CCR3	--->	CXCL6	0.21658	-0.35895	-
[9777]	GHRL	--->	OXTR	0.61472	-0.75338	+
[9778]	KAT2B	<---	MEF2B	-0.13401	0.099749	-
[9779]	PRKCZ	<---	RAC3	-1.529	1.3756	+
[9780]	BCR	<---	RHOBTB2	-0.1232	0.062954	-
[9781]	GRIP1	<---	MEF2B	-0.31668	0.33835	-
[9782]	PRAME	--->	ZBTB33	0.21691	-0.11446	-
[9783]	PIK3R2	<---	PRKCI	-0.21957	0.21974	-
[9784]	EZH2	--->	HEY1	0.53515	-0.65608	-
[9785]	CSF1	--->	FLT4	0.096907	-0.082829	+
[9786]	EIF2AK4	--->	NFKBIB	0.68643	-0.45084	+
[9787]	NFATC1	<---	PIM1	-0.99472	0.92767	+
[9788]	APBB3	--->	NCSTN	0.074889	-0.18174	-
[9789]	MCF2	<---	RHOC	-0.14219	0.16153	-
[9790]	COL10A1	--->	PPIB	-0.016005	-0.04283	-
[9791]	CTBP2	--->	SIX6	0.04296	-0.16727	+
[9792]	PTK2	<---	RHOA	-0.14524	-0.084837	-
[9793]	ADCY10	<---	GNAI1	-0.63317	1.0384	+
[9794]	C2	<---	MASP2	-0.013201	-0.00086449	+
[9795]	GNB3	--->	WNT3	0.19155	-0.26814	-
[9796]	PTPN11	--->	VAV3	0.050213	-0.1064	+
[9797]	CCL5	<---	CXCR5	-0.35895	0.21658	-
[9798]	EP300	--->	LSR	0.0011413	-0.0062856	+
[9799]	ANGPT4	<---	CREBBP	-0.007885	0.0017297	+
[9800]	PTP4A1	--->	RHOA	0.56686	-0.55945	+
[9801]	ITGA4	--->	THBS2	0.081101	-0.066168	-
[9802]	ARHGAP39	<---	RHOG	-0.82986	0.43536	-

[9803]	ITGB5	--->	RELN	-0.015239	-0.019247	-
[9804]	PIK3R2	--->	SPI1	-0.059014	-0.066584	-
[9805]	DAND5	--->	IL12B	0.20206	-0.31964	+
[9806]	ADCY3	<---	CALML6	-0.31614	0.34641	+
[9807]	PIK3R1	<---	ZAP70	-0.37903	0.20388	+
[9808]	ILK	<---	ITGB1	-0.042918	0.033119	+
[9809]	CACNB3	<---	PPP2R1A	-0.20392	0.21525	+
[9810]	RHOF	--->	TRIO	0.11717	0.0097792	-
[9811]	FGF1	--->	IGF1R	0.25346	-0.0044024	+
[9812]	HCK	--->	PIK3CD	0.084876	-0.06481	+
[9813]	DUSP16	--->	MAPK10	1.353	-1.036	+
[9814]	DBP	<---	EP300	-0.0050208	0.00067605	+
[9815]	CCND1	<---	TEAD1	-0.6264	0.69438	+
[9816]	LAMA4	--->	USF1	0.43996	-0.52432	-
[9817]	DAND5	<---	HSPA5	-0.61172	0.6184	-
[9818]	CD8B	--->	IL12B	0.097075	-0.28101	+
[9819]	IL12RB1	--->	STAT5A	0.38886	-0.28644	+
[9820]	CCL15	<---	CCR1	-0.35895	0.21658	-
[9821]	CD3E	--->	PDCD1	-0.033535	-0.068284	-
[9822]	DBP	--->	E2F6	0.0081868	-0.082946	-
[9823]	PIK3CG	--->	SPRY4	0.0067327	-0.02115	+
[9824]	CCL21	<---	CCR3	-0.35895	0.21658	-
[9825]	ADRA1A	--->	PRKAG1	0.84337	-1.591	+
[9826]	GRK4	--->	OPRK1	0.33635	-0.28786	+
[9827]	ADRBK2	--->	CCR7	0.25327	-0.21301	+
[9828]	ARHGAP11A	<---	RHOJ	-1.5245	1.016	-
[9829]	CXCL3	<---	CXCR1	-0.35895	0.21658	-
[9830]	EPS15	<---	GRB2	-0.22843	0.15118	-
[9831]	COL1A1	--->	ITGA5	0.0052169	-0.090529	+
[9832]	RHOT2	<---	SOS2	-0.25271	0.34019	+
[9833]	GNB3	--->	PIK3R5	0.19789	-0.26336	+
[9834]	CSNK1E	--->	FZD5	0.5614	-0.51612	-
[9835]	ITGB1	--->	PAX6	0.033324	-0.056605	-
[9836]	GNB4	--->	WNT9A	0.3343	-0.42584	-
[9837]	IL22RA2	<---	LEP	-0.087402	0.20113	+
[9838]	CSF2	--->	LIFR	0.10899	-0.13275	+
[9839]	PIM2	--->	STAT3	1.4162	-0.966	-
[9840]	OBSCN	<---	RHOT1	-0.0015372	0.062885	-
[9841]	IL23R	--->	NFKBIA	0.012062	-0.087513	+
[9842]	CDC42	<---	DNMBP	-0.055598	0.10561	+
[9843]	MAPK1	--->	SOS1	0.25162	-0.40162	-
[9844]	GNA11	--->	PLCD3	0.050964	-0.056958	+
[9845]	CSNK1D	--->	FZD4	0.5614	-0.51612	-
[9846]	NFKBIA	--->	PSMD10	0.0027011	-0.15249	-
[9847]	HRAS	--->	SPTBN1	0.014795	-0.01078	-
[9848]	ARHGAP30	<---	RHOF	-1.5245	1.016	-
[9849]	GRIN2A	<---	PRKACB	-1.006	1.5014	+
[9850]	AGTR1	--->	GNAQ	1.1631	-1.1303	+
[9851]	PFKFB4	--->	PRKAG2	-0.012105	-0.096604	-
[9852]	APC	--->	USF2	0.0047445	-0.017853	-
[9853]	LAT	<---	NRAS	-0.0041127	0.010952	-
[9854]	ADRB1	<---	ADRBK1	-0.21301	0.25327	+
[9855]	DNM1	--->	EGF	0.026685	-0.024216	+
[9856]	IL18RAP	--->	NFKB1	0.074694	-0.056359	+
[9857]	DMD	--->	MYL3	0.058733	-0.093061	-
[9858]	CCL7	<---	CCR5	-0.35895	0.21658	-
[9859]	KRAS	<---	SFN	-0.25073	0.15053	-
[9860]	TLR4	--->	UBE2D1	-0.027081	-0.36832	-
[9861]	GRB2	--->	SPRY4	0.18885	-0.20205	-
[9862]	KIT	<---	PDGFA	-0.061502	0.075112	+
[9863]	IL11RA	--->	SHC1	-0.003509	-0.082483	+
[9864]	CDK2	--->	HCFC2	0.57211	-0.50839	+
[9865]	LEPR	<---	STK11	-0.30945	0.3459	-
[9866]	CACNA1D	<---	KCNMB2	-0.74334	0.88134	+
[9867]	FOXO3	--->	GABARAPL1	1.0193	-0.41286	+
[9868]	ADCY2	<---	PRKACB	-0.63235	0.8673	-
[9869]	ANGPT1	<---	CRK	-0.13939	0.096627	-
[9870]	IRAK4	--->	PELI3	0.14521	-0.33888	+
[9871]	CACNA1F	<---	HTR2C	-0.36046	0.31836	+
[9872]	CD3G	<---	PDCD1LG2	-0.12874	0.1523	+
[9873]	DVL2	<---	FZD1	-0.33521	0.24353	+
[9874]	PRKAR2A	--->	RHOA	0.36076	-0.3961	+
[9875]	NCSTN	--->	NGF	0.42097	-0.48752	+
[9876]	RXRA	--->	STAT5A	0.10705	-0.13717	+
[9877]	PIK3CB	<---	RAP1A	-0.01026	0.011011	+
[9878]	LYN	--->	PTK2B	0.34449	-0.5161	-

[9879]	CC2D2A	--->	RAB8A	0.48817	-0.45918	+
[9880]	PRKCB	--->	RASAL2	0.14565	-0.025686	-
[9881]	MEF2C	<---	POU5F1	-0.1766	-0.00025604	+
[9882]	CCNT2	<---	HIRA	-0.12355	0.16119	-
[9883]	HES1	--->	NOTCH1	0.26693	-0.19335	-
[9884]	LCK	<---	PTPN13	-0.03006	0.033239	+
[9885]	GLI1	--->	SMO	0.093366	-0.013138	-
[9886]	NRG2	<---	PRKCA	-0.21414	0.16951	-
[9887]	CSF1R	<---	VEGFB	-0.30912	0.56567	+
[9888]	GDNF	<---	NCK1	-0.35073	0.29556	-
[9889]	CBLB	--->	LCK	0.10935	-0.17509	+
[9890]	BUB1	--->	RPS6KA1	0.9519	-1.129	-
[9891]	CD79B	<---	GRB2	-0.10895	0.079303	-
[9892]	CNOT1	--->	EIF4B	0.15211	-0.11319	+
[9893]	ARHGAP18	<---	CDC42	-1.5245	1.016	-
[9894]	CASP2	<---	NGFR	-0.47569	0.24446	+
[9895]	GNAI3	<---	HCAR2	-1.1303	1.1631	+
[9896]	ARHGEF6	--->	RHOT2	0.049516	-0.047518	+
[9897]	CCNE2	<---	TFDP1	-0.19646	0.24842	+
[9898]	HRAS	--->	RALBP1	0.61511	-0.92575	+
[9899]	BTRC	--->	EP300	0.014656	-0.010208	-
[9900]	CASP8	--->	CD3E	0.17126	-0.59854	-
[9901]	IL23R	--->	STAT5A	0.26761	-0.18427	+
[9902]	MAPK9	<---	RALA	-0.20304	1.2491	+
[9903]	GLI1	--->	HHIP	1.4952	-0.74759	+
[9904]	CCL22	<---	CCR4	-0.35895	0.21658	-
[9905]	CDH1	<---	SMAD4	-0.25804	0.022693	-
[9906]	PIK3CD	<---	RAP1B	-0.01026	0.011011	+
[9907]	BDNF	<---	NGFR	-0.60786	0.33599	-
[9908]	ACTN2	<---	CAMK2G	-0.14999	0.15958	-
[9909]	CTNNA2	--->	MMP25	0.38719	-0.55042	-
[9910]	DDX58	<---	MAP2K4	-0.074093	0.20127	-
[9911]	MAP2	<---	MARK2	-0.13052	0.21734	+
[9912]	RAB5A	--->	RIN2	0.39608	-0.50656	-
[9913]	KCNMA1	<---	PRKG2	-0.76808	0.73036	+
[9914]	IL1A	--->	MEF2A	0.12176	-0.23984	-
[9915]	CCND1	<---	FOXO3	-0.62791	0.70491	+
[9916]	GPR37L1	<---	HSPA6	-0.53064	0.43836	+
[9917]	ARHGEF9	<---	RAC2	0.091479	0.13323	-
[9918]	GNAI1	<---	RGS5	-1.2531	1.2063	+
[9919]	ANGPTL4	<---	EP300	-0.007885	0.0017297	+
[9920]	CTNNB1	<---	PCDHGA2	-0.19204	0.11503	-
[9921]	CHUK	<---	RNF135	-0.065336	0.14588	+
[9922]	ACACB	<---	SREBF1	-0.31646	0.33438	+
[9923]	ARHGAP28	<---	RHOB	-1.5245	1.016	-
[9924]	NFKB1	<---	TLR10	-0.60532	0.5764	-
[9925]	RAC1	--->	ROR2	0.01242	-0.26951	-
[9926]	FGF17	<---	NGFR	-0.47081	0.24157	-
[9927]	PIK3CA	<---	PTPN6	-0.043669	0.048862	+
[9928]	ADAM10	--->	CTNND1	0.10295	-0.17951	+
[9929]	GRIN2B	--->	RASGRF1	0.034137	-0.062418	+
[9930]	CAV1	<---	TGFBR1	-0.094314	0.32727	+
[9931]	LAMB1	--->	SV2B	0.27773	-0.3202	+
[9932]	INS	--->	PIK3R1	0.087711	-0.15711	+
[9933]	ADCY1	<---	CALML3	-0.63894	0.65698	+
[9934]	EBF1	--->	SRCAP	0.036203	-0.074732	+
[9935]	LIFR	<---	SOCS1	-0.06215	-0.055475	+
[9936]	COL1A2	--->	FOS	0.25678	-0.16066	-
[9937]	DDX58	<---	ITCH	-0.17412	0.10188	+
[9938]	HRAS	--->	PIK3CB	0.0055787	-0.0073223	+
[9939]	IL3RA	<---	TYK2	-0.34835	0.25816	-
[9940]	FOS	<---	SIRT1	-0.16066	0.25678	+
[9941]	CCNA2	<---	FOXM1	-0.62791	0.70491	-
[9942]	DLG1	<---	YAP1	-0.05901	0.042003	-
[9943]	CTNND1	<---	DLG1	-0.037051	0.146	-
[9944]	RAPGEF1	--->	RELN	0.10738	-0.1148	-
[9945]	KLRG1	<---	SYK	-0.13461	0.052099	-
[9946]	CEBPB	<---	ELMSAN1	-0.20552	0.17137	-
[9947]	EGF	--->	IGF1R	0.1552	-0.058377	+
[9948]	GDNF	<---	PIK3CA	-0.028327	0.0060466	-
[9949]	RB1	<---	TFDP2	-0.13587	0.072342	-
[9950]	ATP1B2	<---	PRKACG	-0.26091	0.40675	+
[9951]	PTK2	--->	SPTBN1	-0.039708	-0.04335	+
[9952]	CDH1	<---	RAP1B	-0.40807	0.12902	+
[9953]	CALM1	--->	MYO5A	0.041455	-0.065093	-
[9954]	CACNA2D2	<---	OXTR	-0.27434	0.12593	+

[9955]	DCC	<---	SRC	-0.58616	0.381	+
[9956]	GATAD2A	--->	SP2	0.28002	-0.15205	-
[9957]	FOXA1	--->	TBL1XR1	0.28486	-0.26412	+
[9958]	ARRB1	<---	RAB5A	-0.024501	0.04502	+
[9959]	CD7	<---	ETS1	-0.042516	-0.021439	+
[9960]	COL4A2	<---	PTK2B	-0.11304	-0.10314	+
[9961]	PCK2	--->	RXRG	-0.16619	-0.17934	-
[9962]	GRIN2A	<---	RHOG	-0.38938	0.54247	-
[9963]	PRKX	<---	RAP1B	-0.20304	1.2491	-
[9964]	EP300	--->	SEMA3C	0.014509	-0.020845	+
[9965]	IL2RB	<---	MAPK9	-0.27385	0.44193	-
[9966]	CCL11	<---	XCR1	-0.35895	0.21658	-
[9967]	ADAM10	--->	NOTCH4	0.042445	-0.036896	+
[9968]	DNM1	<---	TBXA2R	-0.088002	0.082554	-
[9969]	CEBPB	--->	TAL1	0.43996	-0.52432	+
[9970]	ADCY4	<---	PRKACB	-0.63235	0.8673	-
[9971]	NFATC3	--->	PPP3R2	-0.039001	-0.22539	-
[9972]	IFNB1	--->	PRLR	0.27509	-0.55283	+
[9973]	GRIA4	<---	PRKCA	-0.43995	0.47541	+
[9974]	EP300	--->	ITGAX	-0.0018796	-0.010965	+
[9975]	GATA1	--->	TAL1	0.26575	-0.31912	-
[9976]	GSK3B	--->	PIK3R3	1.1072	-1.3078	-
[9977]	ACACA	<---	PRKAG3	-0.95328	1.0154	+
[9978]	CSNK1A1L	<---	PPP2CA	-0.15489	0.41077	-
[9979]	CCL3	<---	CXCR5	-0.35895	0.21658	-
[9980]	CEBPA	<---	GADD45A	-0.45553	0.24487	-
[9981]	CEBPD	--->	TGFB1	0.17799	-0.37709	-
[9982]	CSNK1A1	--->	LRP5	0.75791	-0.52696	-
[9983]	FBXO32	<---	FOXO3	-0.97709	0.60331	+
[9984]	ACTN3	--->	PTK2B	-0.010962	-0.070106	-
[9985]	CTNNA1	--->	MMP28	0.38719	-0.55042	-
[9986]	PPIA	<---	PPP3CA	-0.52872	0.31282	-
[9987]	CREB1	<---	KAT7	-0.098546	0.15465	-
[9988]	NRAS	--->	TRPV1	0.11502	-0.25165	+
[9989]	CD86	<---	PIK3C3	-0.032347	0.019795	-
[9990]	MYH6	<---	THRB	-0.056504	-0.0074276	+
[9991]	ADCYAP1R1	--->	GNB4	0.059668	-0.060578	+
[9992]	PIK3CD	<---	PRKCZ	-0.0097396	-0.0033944	-
[9993]	IL11RA	--->	IL12B	-0.043098	-0.065858	-
[9994]	GRB2	<---	SHC1	0.018816	0.29675	-
[9995]	CCR1	<---	GRK6	-0.28786	0.33635	+
[9996]	CDC42	--->	PIK3R2	0.22452	-0.33729	-
[9997]	MYC	<---	ZNF718	-0.16882	0.033171	-
[9998]	NOTCH2	<---	PCSK7	-0.083505	0.10143	+
[9999]	ETV4	<---	HNF4G	-0.14879	-0.015388	+
[10000]	RCHY1	--->	TP53	0.073186	-0.13445	-
[10001]	APP	<---	NFKB1	-0.021857	0.016795	-
[10002]	COL9A1	--->	MMP3	-0.0056809	-0.18543	-
[10003]	GLI2	<---	INTU	-0.058826	0.25834	+
[10004]	HSPA1A	--->	TAF1	0.64767	-0.69131	-
[10005]	MYH6	--->	MYL3	0.063371	-0.085492	-
[10006]	CCNB1	<---	CENPA	-0.26899	0.30979	-
[10007]	GNB5	<---	PRKCG	-0.42438	0.35558	+
[10008]	CDKN1B	<---	FOXO4	-0.33788	0.8797	+
[10009]	EED	<---	EP300	-0.0077682	0.007799	+
[10010]	IFNAR2	--->	JAK3	1.1771	-1.1167	+
[10011]	PRKACB	--->	TRPV4	0.54889	-0.41958	+
[10012]	CTCF	<---	IRF2BP1	-0.098484	0.17873	-
[10013]	CEBPB	--->	ID1	0.43996	-0.52432	+
[10014]	ARF1	<---	HTT	-0.49203	0.32422	-
[10015]	BCL2	<---	MAPK9	-0.9271	0.81238	+
[10016]	ADCY1	<---	PRKACB	-0.63235	0.8673	-
[10017]	BID	<---	CFLAR	-0.64405	0.31818	+
[10018]	PRKCH	--->	RASA1	0.06376	0.037455	-
[10019]	IL2RG	--->	NTRK1	0.011246	-0.12702	+
[10020]	CAMK2G	--->	ETS1	0.1858	-0.13343	+
[10021]	ITGAV	<---	KRAS	-0.10645	0.030711	-
[10022]	GRAP2	--->	TRBV12-3	0.13256	-0.17782	-
[10023]	MAP3K1	<---	MAP3K7	-0.7238	0.73239	+
[10024]	HTT	--->	TP73	0.1049	-0.14995	+
[10025]	IL6R	<---	JAK2	-0.2042	0.16233	+
[10026]	MAPK1	--->	PLA2G6	0.41524	-0.27455	+
[10027]	CTBP2	<---	ZNF503	-0.056097	-0.053993	-
[10028]	MAPK8	<---	TEK	-0.32312	0.2989	+
[10029]	ROCK2	<---	SLC9A3	-0.10584	0.12042	-
[10030]	PRKACA	--->	XBP1	0.40675	-0.26091	+

[10031]	ADAM10	--->	JAG2	0.017463	-0.033173	+
[10032]	LHCGR	--->	STAT5B	0.47993	-0.37004	-
[10033]	GNGT2	--->	PLCB2	-0.0094253	-0.02635	+
[10034]	STAT1	--->	TP53	0.11495	-0.13304	+
[10035]	F2RL3	--->	GZMA	0.11779	-0.2522	-
[10036]	GNAO1	--->	RASD1	1.2694	-0.93465	-
[10037]	GNA11	--->	WNT10B	0.42087	-0.47341	-
[10038]	SGK3	--->	WNK1	-0.0693	-0.071546	-
[10039]	GAB1	<---	PTPN2	-0.77583	0.87509	+
[10040]	PRKAR1A	--->	STMN1	0.28956	-0.40795	+
[10041]	PPP2CB	--->	YWHAZ	0.18205	-0.13344	-
[10042]	EBF3	--->	NANOG	0.26198	-0.27059	-
[10043]	IFNAR2	<---	IFNG	-0.39151	0.21627	+
[10044]	CACNA1S	<---	PPP1CC	-0.51246	0.42555	+
[10045]	COL1A2	--->	ITGA6	-0.013036	-0.10803	+
[10046]	CACNA2D2	<---	PRKACG	-0.28305	0.4149	+
[10047]	CCL17	<---	CXCR5	-0.35895	0.21658	-
[10048]	GNAI1	<---	PRKCA	-0.38932	0.32346	+
[10049]	PRKCI	--->	PTPN11	0.55589	-0.48259	-
[10050]	AKT1	--->	ATF6B	0.82132	-1.3257	+
[10051]	CSNK1G3	--->	FZD4	0.33941	-0.31414	-
[10052]	SGK3	--->	WNK4	-0.0693	-0.071546	-
[10053]	APBB1	--->	BACE2	0.074889	-0.18174	-
[10054]	PPP4C	--->	YWHAG	-0.03225	-0.31369	-
[10055]	NOTCH4	<---	POFUT1	-0.090993	0.34589	+
[10056]	ABI1	--->	WASF2	0.099632	-0.096896	+
[10057]	EGF	<---	UBE2D2	-0.1934	0.19772	-
[10058]	HDAC1	<---	SMURF1	-0.43565	0.35131	+
[10059]	FZD1	<---	WNT5B	-0.78209	0.63734	+
[10060]	DLC1	--->	RHOT1	-0.10028	-0.14803	+
[10061]	IL19	--->	IL23R	-0.0057724	-0.034956	+
[10062]	CBL	--->	THBS1	0.086381	-0.037679	+
[10063]	PRKACA	--->	SCN1A	0.61419	-0.44517	+
[10064]	KIT	--->	PLCB1	0.036808	-0.035657	+
[10065]	GNAI3	--->	GRM3	0.24033	-0.15623	-
[10066]	SFRP2	--->	WNT4	1.0317	-1.1063	+
[10067]	ITSN1	--->	RHOH	0.16745	-0.11028	+
[10068]	AMHR2	<---	BMP4	-0.85941	1.3161	+
[10069]	GRK5	--->	SSTR4	0.33635	-0.28786	+
[10070]	CDK4	--->	FOS	1.0858	-0.57905	-
[10071]	PIK3R2	<---	RAP1A	-0.33729	0.22452	+
[10072]	RHOBTB1	--->	SYDE2	0.61511	-0.92575	-
[10073]	MMP12	--->	NID1	0.046424	-0.088881	+
[10074]	MEFV	--->	PYCARD	0.38997	-0.30127	+
[10075]	FYN	--->	ITGB1	0.23238	-0.16875	-
[10076]	CD79B	<---	RASA1	-0.15392	0.20051	-
[10077]	ACVRL1	--->	GATA2	0.055783	-0.19314	-
[10078]	IL3	--->	PRLR	-0.018386	-0.067781	+
[10079]	FGF13	--->	FLT4	0.056788	-0.1053	+
[10080]	GAB1	<---	PIK3CG	-0.052593	0.057488	-
[10081]	PRKCG	--->	TTF1	0.32366	-0.41207	+
[10082]	KLRD1	<---	LCK	-0.45391	0.63648	+
[10083]	CSF1	<---	EGFR	-0.17162	0.2091	-
[10084]	LCK	--->	NGEF	0.49172	-0.08758	+
[10085]	NCBP2	<---	RAE1	-0.17132	0.31391	+
[10086]	ACTB	<---	FLNC	-0.51597	0.40448	+
[10087]	OPHN1	<---	RHOBTB1	-0.58391	0.47169	-
[10088]	CASP1	<---	PYCARD	-0.78024	-0.27616	+
[10089]	HIF3A	<---	UBE2D2	-0.080839	0.14071	-
[10090]	FCER1A	--->	SHC1	0.58843	-0.57728	+
[10091]	COL4A4	<---	FYN	-0.45391	0.63648	-
[10092]	PROS1	--->	SERPINA5	-0.013883	-0.066588	-
[10093]	EPOR	<---	IFNG	-0.28909	0.11434	+
[10094]	INSRR	<---	PIK3CD	-0.0047411	0.033025	-
[10095]	CEBPB	<---	MTF2	-0.093184	-0.011158	-
[10096]	MEF2A	<---	MSX2	-0.1766	-0.00025604	-
[10097]	BLNK	--->	RAC3	0.30072	-0.072693	+
[10098]	ATF1	--->	ID2	0.19431	-0.13736	+
[10099]	CCL13	<---	CCR4	-0.35895	0.21658	-
[10100]	ADCY6	<---	GNAL	-0.63317	1.0384	-
[10101]	FOXA2	--->	GLI2	0.45758	-0.30022	-
[10102]	IL18	--->	SOS1	0.10297	0.037236	+
[10103]	GNA15	<---	LPAR2	-1.1303	1.1631	+
[10104]	ERBB3	--->	FOS	0.30871	-0.24282	-
[10105]	NEDD4L	<---	WNK2	-0.80961	0.71536	+
[10106]	ACTR2	<---	GAB1	-0.18635	0.1982	+

[10107] CCL2 <--- CCR7 -0.35895 0.21658 -
[10108] ARHGAP23 <--- RAC2 -0.46335 0.36352 -
[10109] GNB4 ---> WNT9B 0.3343 -0.42584 -
[10110] CRLF2 <--- SOCS7 -0.10066 -0.08965 +
[10111] BATF ---> ZNF267 0.16224 0.14761 +
[10112] HNF4A ---> HOXB9 0.10711 -0.27075 +
[10113] EPHA10 ---> RHOA 0.039661 0.014723 +
[10114] IL12RB1 ---> SOCS1 -0.16125 -0.20399 -
[10115] LHX3 ---> REST 0.36023 -0.20596 -
[10116] AKT1 <--- PPP2R1A -0.35974 0.31573 -
[10117] FZD3 <--- WNT10A -0.78209 0.63734 +
[10118] CHRM1 <--- PRKCD -0.2791 0.17482 -
[10119] CCL28 <--- CCR10 -0.35895 0.21658 -
[10120] KCNMB1 <--- PRKG2 -0.78834 0.62769 +
[10121] LYN <--- PTPN11 -0.41996 -0.082668 +
[10122] LRP6 <--- WNT1 -0.28769 0.39899 -
[10123] ITGAV <--- PLCD3 -0.011601 -0.0076751 -
[10124] DKK2 ---> FZD2 0.3176 -0.35737 +
[10125] BATF <--- TP63 -0.11222 0.11374 -
[10126] GRK7 ---> GRM8 0.80802 -0.62351 +
[10127] GNB2 <--- KCNJ9 -0.098063 0.10019 -
[10128] CDC25A <--- PLK1 -0.32818 -0.022793 +
[10129] INSR ---> SHC3 0.37655 -0.22424 +
[10130] INS ---> STAT5A 0.46738 -0.34275 -
[10131] ARNT <--- PFKFB4 -0.099193 0.096943 -
[10132] JUNB <--- SGK1 -0.40773 0.45877 -
[10133] EIF4B <--- ESR1 -0.31347 0.23448 +
[10134] CASP10 ---> IKBKB 0.89389 -1.332 +
[10135] SIK1 <---> STK11 0.31014 0.31014 -
[10136] CRK ---> ITGAE 0.018691 -0.085251 -
[10137] DEPTOR <--- RRAGB -0.18677 0.15952 +
[10138] CTNNB1 <--- PCDHA3 -0.19204 0.11503 -
[10139] E2F6 ---> KLF1 0.23995 -0.10098 +
[10140] IL1A ---> SPI1 0.053753 -0.12163 -
[10141] FADS2 ---> RXRG -0.11913 -0.23787 -
[10142] HDAC1 <--- SP1 0.01176 0.26827 +
[10143] GRK5 ---> GRM7 0.80802 -0.62351 +
[10144] CCNE1 <--- CDK2 -0.55661 0.75243 +
[10145] MUSK ---> RAC1 0.13971 -0.076906 +
[10146] SOCS7 ---> STAT5A 0.27422 -0.74698 -
[10147] GATA2 <--- UBE2L3 -0.20492 0.13119 -
[10148] ACTG1 <--- RAC2 -0.51168 0.52339 +
[10149] FOXO3 ---> MAX 0.49094 -0.52266 +
[10150] CD80 ---> GAB2 0.15229 -0.13262 +
[10151] CAPN7 <--- HTT -0.18387 0.15402 -
[10152] CACNA1G ---> GRIN2B 0.37674 -0.2845 -
[10153] AR <--- GNAO1 -0.14793 0.11065 -
[10154] PIK3CD <--- RAP1A -0.01026 0.011011 +
[10155] ACTN2 ---> MYL2 0.11715 -0.14512 -
[10156] ADRBK1 ---> CXCR4 0.25327 -0.21301 +
[10157] CDK1 ---> E2F1 0.45782 -0.30098 -
[10158] ARHGEF3 <--- RHOU -0.012974 0.047696 -
[10159] EP300 <--- GPR65 -0.0097452 0.0029784 -
[10160] AKT1 ---> PTEN 0.24868 -0.38894 -
[10161] PRKCG ---> TJP1 0.14343 -0.1592 +
[10162] CFTR <--- PRKACB -0.37336 0.33589 +
[10163] MAP2K1 <---> PRKACB 0.31014 0.31014 -
[10164] JAK1 ---> MAP3K1 0.25727 -0.62209 +
[10165] GNA14 <--- RGS19 -1.2531 1.2063 +
[10166] ELK1 <--- XIAP -0.084395 -0.057201 -
[10167] CTCF ---> ZNF425 -0.0037425 -0.012548 +
[10168] CALML6 ---> CAMK4 1.2051 -1.1603 +
[10169] AURKA <---> PRKACA 0.31014 0.31014 -
[10170] CHD6 ---> EP300 -0.0024545 -0.0075515 -
[10171] CREB1 <---> FOS 0.22155 0.22155 -
[10172] ARAF <--- PRKCD -0.36938 0.20778 +
[10173] SOCS1 ---> STAT1 0.56704 -0.92188 -
[10174] FURIN ---> NOTCH2 0.10143 -0.083505 +
[10175] MAPK10 ---> PLA2G4E 0.18871 -0.84715 +
[10176] ARHGEF28 ---> PTK2 -0.014955 -0.050201 -
[10177] GRK1 ---> SSTR3 0.33635 -0.28786 +
[10178] F2RL2 ---> GNA11 1.1631 -1.1303 +
[10179] JAK2 ---> MAPK3 0.43202 -1.1344 +
[10180] GNAI2 <--- PIK3CG 0.00043444 0.0052476 -
[10181] EPO <--- RAP1A -0.092976 0.096048 -
[10182] ELAVL1 <--- PRKAA1 -0.47902 1.2133 +

[10183] FLNA <--- MAPK9 -0.26091 0.40675 -
[10184] LMOD1 ---> MYL9 0.59921 -0.70918 -
[10185] GNAI3 <--- KCNJ6 -0.011498 0.05262 -
[10186] E2F6 ---> TFDP2 -0.020679 -0.077916 -
[10187] MC1R ---> USF1 0.60063 -0.73364 -
[10188] IL23R <--- SOCS5 -0.11814 0.096109 +
[10189] CSNK1G1 ---> SFN 0.047412 -0.04465 +
[10190] ARAP1 <--- CDC42 -0.48615 0.36733 -
[10191] F2 ---> PROS1 0.062597 -0.095374 -
[10192] ITGA10 <--- LAMB3 -0.066621 -0.0090065 +
[10193] IRS1 <--- PXN 0.046016 0.0706 -
[10194] DRD3 ---> GNB1 0.69217 -0.57296 +
[10195] IL22RA2 <--- PRL -0.087402 0.20113 +
[10196] COL4A3 ---> ITGB4 0.014159 -0.041712 +
[10197] MAPK3 <---> PRKX 0.31014 0.31014 -
[10198] GNA13 ---> PLA2G4C 0.034723 -0.073024 +
[10199] BMPR1B ---> SMAD6 0.7946 -0.60139 +
[10200] GNAZ <--- LPAR1 -1.1303 1.1631 +
[10201] EGFR ---> PLCB1 0.064633 -0.064447 -
[10202] KDR <--- VEGFC -0.023732 0.31113 +
[10203] MYC ---> STAT4 0.048778 -0.065592 -
[10204] BRCA1 ---> CHEK2 0.21422 0.016853 +
[10205] CCR4 ---> XCL1 0.21658 -0.35895 -
[10206] HDAC2 <--- PIM1 -0.52917 0.82707 -
[10207] PLCE1 <--- RHOA -0.093653 0.086381 +
[10208] GATA2 ---> ITPR1 0.44021 -0.2432 +
[10209] FGF2 ---> KDR 0.21774 -0.0015767 +
[10210] EPAS1 <--- VEGFC 0.058443 0.072574 -
[10211] ARHGAP44 <--- RHOBTB1 -0.56259 0.37198 -
[10212] ARHGEF4 <--- RHOH 0.091479 0.13323 -
[10213] BCR <--- RHOG -0.20131 0.10561 -
[10214] BCAR1 ---> RAP2B 0.21814 -0.11421 +
[10215] CCNB2 <--- FZR1 -0.33759 0.44625 +
[10216] NEUROG3 <--- ONECUT1 -0.32236 0.23888 +
[10217] IKBKG <--- NGFR -0.56729 0.34132 +
[10218] ACTBL2 <--- MMP19 -0.29263 0.085904 +
[10219] KPNA5 <--- SEH1L -0.11171 0.067727 +
[10220] TREM2 ---> VAV2 0.064425 -0.018037 +
[10221] NKX2-1 <--- RARG -0.27075 0.10711 +
[10222] DKK2 ---> LRP5 1.4889 -1.0685 +
[10223] KCNQ1 <--- PRKACB -0.44517 0.61419 -
[10224] JUP <--- MMP8 -0.26058 0.11034 +
[10225] ITK ---> PIK3R3 0.14102 -0.25088 -
[10226] ARAF ---> MAPK9 0.99795 -1.3266 +
[10227] TP53 <--- TRAF6 -0.26152 0.34561 +
[10228] NTRK1 <--- PTK2 -0.16925 -0.068782 +
[10229] GYS1 <--- PPP1CC -0.9036 0.53904 +
[10230] CSH1 ---> OSMR 0.073187 -0.17721 +
[10231] PTK2 ---> VAV3 -0.013278 -0.033868 +
[10232] MAP2K3 ---> MAPK11 1.1648 -0.18663 +
[10233] CNTF ---> IL10RB 0.20113 -0.087402 +
[10234] ITSN1 ---> RHOU 0.16745 -0.11028 +
[10235] ERN1 <--- TRIM3 0.0068313 0.044647 -
[10236] FYN ---> RHOA 1.0122 0.16613 -
[10237] IL23R <--- PTPN6 -0.18061 0.25536 +
[10238] ARRB1 ---> DNM1 0.027228 -0.026147 -
[10239] IL6R <--- SOCS5 -0.13627 0.098644 +
[10240] HIVEP1 <--- NFKB1 -0.13106 0.32527 +
[10241] BCL2L11 <--- MAPK9 -0.27209 0.41438 +
[10242] TNFRSF14 <--- TNFSF14 -1.3016 1.0868 +
[10243] IL12RB1 <--- IL26 -0.29036 0.12248 +
[10244] CD3G ---> PDCD1 -0.033535 -0.068284 -
[10245] FGFR2 <--- PLCB4 -0.09782 0.043366 -
[10246] LEPR <--- PRKAG3 -0.16221 0.0036906 -
[10247] PPP1CC <--- PRKCE -0.32227 0.20474 +
[10248] ARRB2 <--- MAPK3 -0.15935 0.24561 -
[10249] CSK ---> GAB1 0.83596 -1.1861 -
[10250] MYLK2 <--- PAK6 -1.2674 1.5523 +
[10251] RAC2 <--- VAV1 0.1025 0.10902 +
[10252] ITGAX <--- POU2F1 -0.1208 -0.053782 +
[10253] ETS1 ---> IL4R 0.043291 -0.10858 -
[10254] MAPK1 ---> NFKBIA 0.60616 -0.41321 +
[10255] CCND1 <--- TFDP2 -0.19646 0.24842 -
[10256] EIF2AK4 <---> MAP2K6 0.31014 0.31014 -
[10257] PPARG <--- TNFRSF1A -0.13189 -0.018661 +
[10258] PLK1 <--- TAOK1 -0.098976 0.29896 -

[10259] ADIPOQ <--- FOXA1 -0.23065 0.19808 +
[10260] CRK ---> ITGAX 0.018691 -0.085251 -
[10261] CRHR1 <--- GNAI1 -0.040633 0.044298 -
[10262] MAPK9 <--- RHOA -0.20304 1.2491 +
[10263] FGFR3 <--- VEGFB -0.076002 0.030896 +
[10264] CASP1 <--- XIAP -0.29703 0.11242 -
[10265] CBLB ---> IL13RA2 0.18381 -0.1079 +
[10266] C1QC ---> IGKV1-5 -0.021439 -0.042516 -
[10267] DKK1 ---> WNT16 0.54164 -0.69755 +
[10268] NOTCH3 ---> RBPJL 0.47621 -0.34967 +
[10269] CTBP2 <--- FOXJ1 -0.28308 0.17988 -
[10270] ETS1 <--- GRPR -0.15406 0.070342 -
[10271] KCNS3 <--- PRKACA -0.44517 0.61419 +
[10272] GNA11 <--- GRPR -1.1303 1.1631 +
[10273] CSF2RB <--- JAK1 0.021591 0.044719 -
[10274] CARD9 <--- MAPK11 -1.1453 1.303 -
[10275] PIK3R3 <--- TRAF2 -0.41639 0.33842 +
[10276] PDGFRB ---> STAT1 0.25332 -0.071571 +
[10277] MAPK11 <--- PPP2R1B -0.25679 0.26693 -
[10278] GNB1 <--- MAPK11 -0.28574 0.38931 -
[10279] ADCY5 <--- PRKACA -0.63235 0.8673 -
[10280] ASAP1 <--- GGA3 -0.0078218 0.038165 +
[10281] CD3G <--- PLCG1 -0.021591 0.04532 -
[10282] ITGA11 <--- LAMA3 -0.041507 -0.0065623 +
[10283] MOS <---> PKMYT1 0.31014 0.31014 -
[10284] IL6R ---> MITF -0.0077733 -0.033843 +
[10285] IL20 ---> IL2RG 0.056231 -0.11334 +
[10286] ESR1 ---> GNA11 0.042535 -0.12976 +
[10287] CTRB2 ---> MMP10 -0.0056809 -0.18543 +
[10288] NFKB1 <--- PRKX -0.059594 0.20986 +
[10289] FZD5 <--- WNT10A -0.78209 0.63734 +
[10290] EGR1 <--- SPHK2 -0.15205 0.28002 -
[10291] IRAK4 ---> PELI1 0.36734 -0.60055 +
[10292] SOCS4 <--- STAT3 -0.73574 0.49958 -
[10293] CCL2 <--- CXCR5 -0.35895 0.21658 -
[10294] CTCFL ---> JUND 0.054205 0.022163 -
[10295] ADCY8 <--- PRKCG -0.49495 0.42642 +
[10296] CDC42 ---> KALRN 0.042607 -0.021179 -
[10297] ITGA6 <--- PRKCA -0.17355 0.11831 +
[10298] GNAI2 <--- PIK3CB -0.00083608 0.0020832 -
[10299] NFKBIB <--- ZNF263 -0.001863 0.37436 +
[10300] FZD10 <--- WNT6 -0.78209 0.63734 +
[10301] HIF1A <--- HK2 0.061721 0.06932 -
[10302] CALM1 ---> GRIN2C 0.61549 -0.52822 -
[10303] CCL16 <--- CCR7 -0.35895 0.21658 -
[10304] MYC <--- RNPS1 -0.13606 -0.02655 -
[10305] GNAI1 <--- PTGER3 -1.1303 1.1631 +
[10306] SFRP4 ---> WNT3A 1.0317 -1.1063 +
[10307] GNAO1 <--- RGS14 -0.094828 0.065188 +
[10308] FRS2 ---> RAP1B 0.13328 0.052553 +
[10309] DAND5 <--- EFEMP1 -0.027839 -0.016705 -
[10310] LPAR3 ---> NFKBIA 0.19531 -0.38696 +
[10311] CEBPB ---> STAT5A 0.44408 -0.32334 +
[10312] CDC42 ---> MAPK9 1.2491 -0.20304 +
[10313] SMAD4 <--- UBE2D3 -0.20131 0.05544 +
[10314] JAK3 ---> XCR1 -0.039248 -0.12268 -
[10315] TAL1 <--- WHSC1 -0.3989 0.43075 -
[10316] BIRC3 ---> MAP3K14 0.21894 -0.30528 +
[10317] OPHN1 <--- RHOT2 -0.45869 0.34454 -
[10318] CD8A ---> SHC1 0.11515 -0.21398 +
[10319] CHRM3 ---> PRKCI -0.029433 -0.05892 +
[10320] FXYD2 <--- MAPK1 -0.26091 0.40675 +
[10321] ESR1 ---> PPRC1 0.23448 -0.31347 +
[10322] CNGB1 ---> PDE6A 0.36568 -0.36893 -
[10323] LDLR ---> STAT3 0.041591 0.0050292 -
[10324] EGR1 <--- TIMP1 -0.15205 0.28002 -
[10325] IL6ST <--- SOCS7 -0.06215 -0.055475 +
[10326] CXCL1 <--- RELA -0.69538 0.38373 +
[10327] CRK ---> PIK3CB 0.010761 -0.019381 +
[10328] KLF11 ---> MAX 0.19953 -0.12327 -
[10329] MEF2C <--- ZBTB32 -0.063721 0.032555 -
[10330] BAD <--- MAPK10 -0.56837 0.82969 +
[10331] GRIN2B <--- PRKX -1.006 1.5014 +
[10332] ARHGAP40 <--- CDC42 -1.5245 1.016 -
[10333] NFATC2 ---> PPIA 0.20392 -0.49063 -
[10334] EBF1 ---> HOXB9 0.26198 -0.27059 +

[10335]	PCK1	<---	PRKAA1	-0.46459	0.63156	+
[10336]	CAMK2D	--->	SCN1B	0.31382	-0.23358	+
[10337]	GNAT3	<---	RGS14	-0.094828	0.065188	+
[10338]	AKT2	<---	HSP90B1	-0.32359	0.3448	+
[10339]	ADAMTSL4	<---	POFUT2	-0.07359	-0.004569	+
[10340]	CAMK2D	--->	PDE3A	0.95928	-0.63646	+
[10341]	CSNK1A1	--->	WNT4	0.83912	-0.78694	-
[10342]	CCR7	--->	CXCL1	0.21658	-0.35895	-
[10343]	YY1	--->	ZBTB17	-0.024143	-0.025264	+
[10344]	DDX20	--->	WDR77	0.072414	-0.071625	-
[10345]	ITGA2B	<---	LAMB4	-0.066621	-0.0090065	+
[10346]	ABL1	<---	PDGFRB	-0.18627	0.45809	-
[10347]	CD14	<---	RELA	-0.2686	-0.14692	-
[10348]	ARF1	--->	COPA	0.010277	-0.18113	+
[10349]	CNTN1	<---	EGR1	-0.037704	0.042147	+
[10350]	ARHGEF17	<---	RHOA	-0.012974	0.047696	-
[10351]	FGFR1	<---	SHC3	-0.10391	-0.029269	-
[10352]	PLA2G4C	<---	PLCG1	-0.023892	0.051576	+
[10353]	ACTN2	<---	PTK2	-0.072715	-0.012479	+
[10354]	LMO4	--->	PIAS3	0.23878	-0.17754	+
[10355]	IL20RA	--->	JAK3	1.1771	-1.1167	+
[10356]	IL12B	--->	STAT3	0.38886	-0.28644	+
[10357]	ADRBK2	--->	CHRM4	0.25327	-0.21301	+
[10358]	PPP1CB	<---	PRKG2	-0.35613	0.22476	+
[10359]	PRKD2	<---	RASA1	-0.012166	0.082785	+
[10360]	CXCR1	--->	PIK3CA	0.016661	-0.039169	+
[10361]	EGR1	--->	F3	0.13491	0.10845	+
[10362]	MAP2K7	<---	TRAF6	-0.47605	1.1617	+
[10363]	ITGA4	--->	TNR	-0.015417	-0.048468	-
[10364]	CACNA1F	<---	GNB3	-0.095409	0.10645	+
[10365]	IFNGR1	<---	TYK2	-0.36315	-0.005631	-
[10366]	HNF4A	--->	MTF2	-0.045899	-0.12955	+
[10367]	LTBP1	--->	TGFB3	1.0128	-1.5309	+
[10368]	ADCY7	<---	EDN1	-0.41432	0.41767	+
[10369]	GSK3A	<--->	PRKACG	0.31014	0.31014	-
[10370]	GNAQ	<---	TSHR	-1.1303	1.1631	+
[10371]	PEBP1	<---	PRKD2	-0.49347	0.4692	+
[10372]	MAP2K4	<---	MAP3K5	-0.99422	1.146	-
[10373]	IL23A	--->	IL6R	0.12377	-0.18387	+
[10374]	ADRBK1	--->	HTR1F	0.25327	-0.21301	+
[10375]	SETDB1	--->	ZNF426	0.12238	-0.066729	+
[10376]	RHOD	--->	STARD8	1.016	-1.5245	-
[10377]	IL2RG	<---	PIK3CD	-0.016602	0.0042292	-
[10378]	GAB2	<---	JAK1	-1.3697	0.33827	+
[10379]	CCR9	--->	CX3CL1	0.21658	-0.35895	-
[10380]	CTNNB1	<---	WNT9B	-0.187	0.023012	+
[10381]	CCR4	<---	GRK4	-0.28786	0.33635	+
[10382]	ITGA2	<---	LAMA4	-0.16586	-0.008916	+
[10383]	GFRA1	--->	RAP1A	0.19144	-0.15953	+
[10384]	IGFALS	--->	IGFBP5	0.16947	-0.36538	-
[10385]	FYN	<--->	SRC	0.15012	0.15012	-
[10386]	CACNA1C	<---	OXTR	-0.36046	0.31836	+
[10387]	OBSCN	<---	RAC1	-0.11324	0.22832	-
[10388]	NFKBIE	<---	PSME4	-0.19284	0.32731	+
[10389]	HOXA5	--->	NCAM1	0.13977	-0.28242	+
[10390]	BRD9	--->	E2F4	0.0081868	-0.082946	-
[10391]	COLEC12	--->	FOS	0.15465	-0.098546	-
[10392]	NCBP2	<---	NUP43	-0.17132	0.31391	+
[10393]	LAT	--->	RXRA	-0.078897	-0.28271	-
[10394]	CDKN1B	--->	RBL1	0.14422	-0.21852	+
[10395]	PLAU	<---	UBE2D1	-0.14097	0.07347	+
[10396]	CDC42	--->	FGD1	0.16153	-0.14219	-
[10397]	IL1B	--->	PAX5	0.063072	-0.091377	-
[10398]	GRK4	--->	HRH4	0.33635	-0.28786	+
[10399]	CDH1	<---	DLG1	-0.12204	0.046703	-
[10400]	ATAD2	<---	FOXA2	-0.37846	0.32839	+
[10401]	MAPK14	--->	PHC3	0.40675	-0.26091	+
[10402]	MRAS	--->	PIK3CG	0.019966	-0.015102	+
[10403]	FZD9	<---	WNT5A	-0.78209	0.63734	+
[10404]	ARHGAP24	<---	RHOQ	-0.49576	0.47865	-
[10405]	POU2F1	<---	VCAM1	-0.022623	0.0033782	-
[10406]	MYL6	--->	MYL9	-0.13994	-0.49178	-
[10407]	NR1H4	--->	RXRA	1.1084	-1.1389	-
[10408]	GLI1	<---	PVR	0.0095228	0.023655	-
[10409]	RAB3A	<---	UNC13B	-0.43795	0.46167	-
[10410]	PPP1CC	--->	RYR2	0.066557	-0.046457	+

[10411] KAT2B ---> MEF2C 0.043005 -0.1118 +
[10412] SOS1 <--- VAV1 -0.16187 0.0010604 -
[10413] GNB2 <--- MAPK14 -0.31609 0.54982 -
[10414] HCK <--- PTPRC -0.044714 0.034382 +
[10415] DTX3 ---> NOTCH3 0.04408 0.031528 -
[10416] PSMD14 ---> SKP2 -0.057449 -0.14741 +
[10417] CER1 ---> FZD10 0.27359 -0.29711 +
[10418] CCR9 ---> CXCL1 0.21658 -0.35895 -
[10419] TGFB2 ---> TGFBR1 0.71751 -0.38171 +
[10420] CSNK1A1L <---> CSNK2A1 0.31014 0.31014 -
[10421] GNB4 ---> WNT16 0.3343 -0.42584 -
[10422] LAMC3 ---> SV2C 0.16735 -0.19531 +
[10423] AKT1 ---> EGR1 0.41854 -0.41441 +
[10424] CCL4 <--- CCR9 -0.35895 0.21658 -
[10425] ATF3 <--- INSL3 -0.24765 0.36121 -
[10426] CCNT2 <--- PHF19 -0.31692 0.2481 -
[10427] IL4R <--- SLA2 -0.19065 0.1367 -
[10428] CDC42 <--- GNAI2 -0.93465 1.2694 +
[10429] CTNNA1 ---> RAP1A 0.60916 -0.52573 -
[10430] MAP2K1 <--- PAK7 -1.2674 1.5523 +
[10431] AGTR1 ---> ARF6 0.034722 -0.34211 +
[10432] CXCL14 <--- CXCR4 -0.35895 0.21658 -
[10433] DAPP1 <--- SRC -0.46872 0.73349 +
[10434] LYN ---> PXN 0.50966 -0.41502 +
[10435] CTSD <--- RAB7A -0.024501 0.04502 +
[10436] CASP6 ---> NGFR -0.17675 -0.22226 -
[10437] IL5RA ---> STAT5A 0.25207 -0.14709 +
[10438] DTX3L <--- STAT1 -0.34096 0.25675 +
[10439] PTPN6 ---> VAV2 0.050213 -0.1064 +
[10440] PLD2 <--- TEK -0.058067 0.035396 +
[10441] LAMA1 ---> SV2C 0.16735 -0.19531 +
[10442] PRKD1 ---> STAT4 0.84558 -0.79252 -
[10443] SCN8A <--- SMARCC1 -0.24986 0.22957 +
[10444] MMP7 <--- TCF7L1 -0.68636 0.071508 +
[10445] HIF1A ---> TUFT1 0.052783 0.00040878 +
[10446] FGF3 <--- NRAS -0.15714 0.23194 -
[10447] APBB3 ---> MAX 0.22195 -0.28844 -
[10448] GNB5 <--- MAPK3 -0.31609 0.54982 -
[10449] AKT3 ---> STAT4 1.212 -1.5407 -
[10450] LYN ---> PLCG2 0.066589 -0.05234 +
[10451] CDH3 <--- CTNNB1 -0.83107 0.8938 +
[10452] CSF2 ---> STAT5B 0.47608 -0.33511 +
[10453] DLG4 ---> GRIA3 0.19945 -0.095793 -
[10454] GNAO1 ---> PLCB2 0.0066317 -0.024514 +
[10455] ATF4 ---> USF1 0.32218 -0.22466 -
[10456] JUN <--- SMAD4 -0.1492 0.050351 -
[10457] HOXD3 <--- ITGB1 -0.091513 0.056756 -
[10458] HDAC3 <--- SMAD4 -0.21285 0.15191 -
[10459] UBE2L6 <---> UBE2N 0.18452 0.18452 -
[10460] CCNL2 <--- PBX3 -0.19893 0.23468 +
[10461] ARHGAP9 <--- RHOB -0.36596 0.22724 -
[10462] PML ---> TP63 0.064063 -0.10427 +
[10463] DVL3 <--- FZD5 -0.33521 0.24353 +
[10464] CD86 <--- CD8A -0.052926 0.00095551 -
[10465] RIPK1 <--- TAB1 -0.16481 0.54011 -
[10466] AKAP13 <--- CDC42 -0.22586 0.072537 -
[10467] PIK3CB <--- PRKCG -0.06189 0.049814 -
[10468] CSNK2A1 <---> GSK3B 0.31014 0.31014 -
[10469] GNAZ ---> PF4 0.087289 -0.054136 -
[10470] F2R <--- GRK1 -0.28786 0.33635 +
[10471] IL20 ---> MPL 0.056231 -0.11334 +
[10472] CSF1R <--- FGF20 -0.64493 0.77791 +
[10473] CHN1 <--- RHOT1 -0.1778 0.091646 -
[10474] SLC2A4 <--- TBC1D4 -0.029745 0.37026 +
[10475] CD40LG ---> STAT5A 0.4382 -0.32637 +
[10476] IGF1R ---> PLCG1 0.027447 -0.017905 +
[10477] PTPN1 ---> RAP1A 0.97901 -1.0196 +
[10478] MAPK1 ---> ZBTB33 0.66556 -0.40919 -
[10479] OSMR ---> SOCS2 -0.098904 -0.12483 -
[10480] PRKD1 ---> SOS1 0.043061 -0.1405 -
[10481] LMO4 ---> MAP2K6 0.46988 -0.1541 +
[10482] JAK3 ---> PIK3R1 0.20388 -0.37903 +
[10483] GNAO1 ---> HRAS 0.76886 -0.56799 +
[10484] GNAI2 <--- LPAR1 -1.1303 1.1631 +
[10485] FURIN ---> PROS1 0.06833 -0.093533 +
[10486] DUSP9 ---> MAP2K4 1.353 -1.036 +

[10487]	DAND5	--->	NOL3	0.46649	-0.48928	+
[10488]	HCFC1	--->	JUND	0.010648	-0.12085	-
[10489]	FOXA1	<--->	FOXA2	0.41711	0.41711	-
[10490]	PAK2	<---	YES1	-0.81947	0.81303	+
[10491]	HRAS	<---	RASAL1	-0.66794	0.71076	+
[10492]	NUP153	--->	SRSF5	0.60094	-0.54167	+
[10493]	CSNK1A1L	--->	WNT9A	0.83912	-0.78694	-
[10494]	ARHGEF11	--->	RHOBTB1	0.055126	0.0033386	+
[10495]	MAPK11	--->	MYC	0.75491	-0.9874	+
[10496]	ACTG1	<---	MMP8	-0.29263	0.085904	+
[10497]	CACNA1F	<---	PPP2CB	-0.27679	0.37996	+
[10498]	ENO3	--->	HIF1A	0.06932	0.061721	-
[10499]	ITGB3	<---	RAP1B	-0.01596	0.026206	-
[10500]	COL1A1	--->	DAND5	-0.0062022	-0.043827	-
[10501]	GLI3	<---	PSMD1	-0.077956	0.20686	+
[10502]	GRK6	--->	GRM1	0.80802	-0.62351	+
[10503]	PPP2CA	--->	YWHAE	0.18205	-0.13344	-
[10504]	CCL27	<---	CXCR3	-0.35895	0.21658	-
[10505]	NR3C1	<---	RELA	-0.27124	-0.010205	-
[10506]	ASH2L	<---	FOXA2	-0.30912	0.30288	+
[10507]	NUP37	<---	NXF1	-0.39709	0.26121	-
[10508]	CBLC	--->	PIK3CG	0.10448	-0.04949	+
[10509]	MAP2K1	<---	PRKCE	-0.092669	0.0037501	+
[10510]	CREB3	<---	PRKCB	-0.63701	0.72046	+
[10511]	EPHA2	<---	FIGF	-0.055219	-0.01111	+
[10512]	CAMK2A	--->	MDM2	-0.043766	-0.12114	+
[10513]	CASP6	--->	NGF	0.635	-0.97212	-
[10514]	BCAR1	--->	ITGAD	0.041847	-0.077667	-
[10515]	CACNB4	<---	PRKACA	-0.016941	0.18215	+
[10516]	GLI1	<---	INTU	-0.058826	0.25834	+
[10517]	FYN	--->	ITGAL	0.14089	-0.16015	-
[10518]	PIM1	--->	STAT2	1.4162	-0.966	-
[10519]	DUSP6	--->	TCF7L2	0.35634	-0.18912	-
[10520]	ARRB1	<---	CXCR2	-0.15406	0.070342	-
[10521]	ACTR2	<---	CDC42	-0.51168	0.52339	+
[10522]	ITGB5	--->	THBS2	0.093347	-0.066778	-
[10523]	GPER1	--->	TCF12	0.60063	-0.73364	-
[10524]	CBLC	--->	PIK3CB	0.062274	-0.031117	+
[10525]	EP300	--->	WNT7A	0.022202	-0.03121	+
[10526]	SIRT2	--->	YY1	0.28002	-0.15205	-
[10527]	ATF2	<---	BRCA1	-0.27862	0.39154	+
[10528]	CXXC1	--->	ZEB1	0.30812	-0.23004	-
[10529]	MAP3K14	<---	RIPK1	-1.2598	0.76413	+
[10530]	FGF17	<---	NRAS	-0.15714	0.23194	-
[10531]	GRIA3	<---	PRKCB	-0.43995	0.47541	+
[10532]	ARHGAP18	<---	RHOF	-1.5245	1.016	-
[10533]	NGEF	<---	RHOA	0.091479	0.13323	-
[10534]	MYC	<---	PBRM1	-0.1755	0.065431	-
[10535]	HECW1	--->	PSMD1	0.21231	-0.35498	-
[10536]	EIF2AK3	--->	PSEN2	0.43723	-0.29495	-
[10537]	COL5A1	--->	ITGA2	-0.008916	-0.16586	+
[10538]	JUN	<---	RXRA	-0.12784	-0.090928	-
[10539]	EP300	--->	NOS3	-0.0020202	-0.0032913	+
[10540]	DIAPH2	<---	PFN2	-0.1109	0.062206	-
[10541]	MYBPC2	<---	MYL3	-0.12286	0.036048	+
[10542]	EBF1	--->	NCOA3	0.071626	-0.064867	+
[10543]	ESR1	<---	TRIM55	-0.070696	-0.032513	-
[10544]	MYLK3	<---	ROCK1	-0.4474	0.43952	+
[10545]	MAPK8	--->	PRKG1	0.5348	-0.19389	+
[10546]	PPP2R1A	<---	STAT1	-0.13717	0.099176	-
[10547]	CSF1	<---	MET	-0.10355	0.07089	-
[10548]	PRF1	--->	STAT3	0.47814	-0.37844	-
[10549]	ITGB2	<---	PTK2	-0.04335	-0.039708	-
[10550]	AKT3	--->	PIK3R1	0.18336	-0.2541	-
[10551]	MAPK1	<--->	PRKACB	0.31014	0.31014	-
[10552]	EIF2AK4	--->	NFKBIA	0.60616	-0.41321	+
[10553]	PRKD3	--->	STAT5B	0.84558	-0.79252	-
[10554]	COL4A4	--->	ITGA3	-0.020349	-0.077049	+
[10555]	EPHA2	--->	FGF21	-0.0069714	-0.050497	-
[10556]	FGF2	--->	IGF1R	0.25346	-0.0044024	+
[10557]	AKT1	--->	PPP6C	-0.032241	-0.11483	+
[10558]	BUB3	--->	CCNB1	0.44625	-0.33759	-
[10559]	EZH2	--->	ZBTB33	0.2738	-0.17323	-
[10560]	KMT2E	--->	YY1	0.45323	-0.2838	-
[10561]	EP300	--->	SLC2A1	0.025698	-0.029079	+
[10562]	CTCF	<---	KAT5	-0.0066284	0.049367	-

[10563] CDK1 ---> KIF2C 0.44436 -0.30006 +
[10564] CREB1 <--- PPP1CA -0.65948 0.41745 +
[10565] CCR8 <--- JAK3 -0.12268 -0.039248 -
[10566] IL10RB <--- IL12B 0.012893 0.051025 +
[10567] FLI1 <--- TGFBR2 -0.21357 0.12981 -
[10568] FGA ---> SERPINE2 0.27611 -0.51388 +
[10569] JAK1 <--- PTPRE -1.368 1.0117 +
[10570] ARHGEF12 <--- GNA13 0.011916 0.034755 +
[10571] PTK2 <--- TLN1 -0.014805 -0.008901 -
[10572] PLD2 ---> RALGDS 0.081409 -0.052912 -
[10573] E2F1 <--- TCF19 -0.083864 -0.0063138 -
[10574] DKK2 ---> WNT11 0.54164 -0.69755 +
[10575] HGF ---> PDGFRA 0.16108 -0.15774 +
[10576] CSF2 ---> IL23R 0.081209 -0.066955 +
[10577] PCGF2 ---> SPI1 0.30904 -0.32545 -
[10578] ARHGEF6 <--- RHOV -0.041777 0.050621 -
[10579] SIX5 <--- THAP11 -0.13058 0.074739 -
[10580] FYN <---> YES1 0.15012 0.15012 -
[10581] PSMD1 <--- UBE2D1 -0.24701 0.16206 -
[10582] CCL2 <--- CCR10 -0.35895 0.21658 -
[10583] PRKD1 <--- RAP1B -0.31045 0.65185 -
[10584] ABR <--- RHOB -0.20131 0.10561 -
[10585] CALM1 ---> CAMK1G 0.41687 -0.23601 +
[10586] OSMR <--- SOCS5 -0.1723 0.11167 +
[10587] CRKL ---> RAC3 0.43868 -0.057401 +
[10588] ARF6 ---> PLD1 0.041749 -0.076299 +
[10589] E2F3 <--- TAF1 -0.082946 0.0081868 +
[10590] NRAS ---> SPTBN1 0.014795 -0.01078 -
[10591] CACNA1C <--- PPP2R1B -0.39164 0.37653 +
[10592] APC <--- CSNK1A1L -0.047997 0.037774 +
[10593] APBB3 ---> PSEN2 0.093374 -0.20238 -
[10594] NR1H3 ---> RXRG 1.1084 -1.1389 -
[10595] GADD45A ---> MAP3K4 0.89119 -1.126 +
[10596] CCNT2 <--- PRAME -0.30146 0.29579 -
[10597] DAPP1 <--- SH3KBP1 -0.285 0.53002 +
[10598] IRAK1 ---> PELI1 0.95521 -0.79521 +
[10599] IKBKB <--- SQSTM1 -0.29176 0.0081481 +
[10600] AMHR2 <--- GDF6 -0.85941 1.3161 +
[10601] NRAS ---> TIAM1 0.12584 -0.084027 +
[10602] EP300 ---> OPTN 0.011402 -0.01459 +
[10603] CTBP2 <--- NR2F2 -0.12315 -0.021474 -
[10604] CALML6 ---> KRAS 0.17857 -0.13574 +
[10605] IFNGR1 <--- IL20 -0.36925 0.012421 +
[10606] NRAS ---> SPTBN5 0.1299 -0.11526 -
[10607] HOXC6 ---> MEF2A -0.00025604 -0.1766 -
[10608] PXN ---> RAP1B 0.070667 -0.0041104 -
[10609] NCOA1 <--- NR1H4 -0.054726 0.035483 -
[10610] GNB5 <--- OPRM1 -0.92616 1.1575 +
[10611] CD44 <--- COL4A1 -0.18268 0.0085485 +
[10612] E2F1 ---> SNRPA 0.32902 -0.26332 +
[10613] GNAT3 <--- RHO -1.1303 1.1631 +
[10614] FGFR2 ---> PIK3R2 -0.065566 -0.099 +
[10615] BRCA1 ---> HERC1 0.14774 -0.11028 +
[10616] CXCL12 <--- GNAO1 -0.054136 0.087289 +
[10617] NUP43 ---> SRSF3 0.31391 -0.17132 +
[10618] BCL2L1 <--- PRKACA -0.9271 0.81238 +
[10619] GNGT2 ---> PLCB3 -0.0094253 -0.02635 +
[10620] MAP3K3 <--- RAC2 -0.17545 1.1267 +
[10621] GNB4 ---> LRP5L 0.082356 -0.05079 -
[10622] FGF22 <--- SOS1 0.067334 0.085583 -
[10623] NOTCH1 <--- PCSK1 -0.19909 0.2729 +
[10624] KCNJ6 <--- OPRD1 -0.2522 0.11779 -
[10625] ERBB2 ---> PIK3CD 0.031013 -0.032307 +
[10626] ATF1 <--- VIPR1 -0.060871 0.092699 -
[10627] ITGA3 <--- SRC -0.29478 0.38743 -
[10628] CRKL ---> LRP8 0.14847 -0.11977 -
[10629] PIAS3 ---> STAT4 1.2392 -0.72187 +
[10630] ACVRL1 ---> SMAD5 0.49955 -0.48017 +
[10631] CBLC ---> IL6R 0.19293 -0.10242 +
[10632] CDKN1B ---> RB1 0.14422 -0.21852 +
[10633] CALM1 ---> RASGRF2 0.29154 -0.36654 +
[10634] ARHGAP28 <--- RHOT2 -0.58534 0.26947 -
[10635] ERG <--- ESR1 -0.091347 -0.010433 -
[10636] CAV1 <--- IRS1 0.023045 0.12681 +
[10637] EPHA2 <--- INS -0.19078 0.19924 +
[10638] ADRBK1 ---> CXCR6 0.25327 -0.21301 +

[10639] CSNK1D ---> LRP5L 0.58717 -0.41189 -
[10640] RAC1 <--- TLR4 -0.80069 0.47206 +
[10641] ATF2 ---> HIST1H2BC 0.048146 0.0081543 +
[10642] IL6ST ---> SOS2 0.024282 0.022964 +
[10643] EBF1 <--- RARA -0.086379 -0.031541 -
[10644] NR1D1 ---> RXRA 1.1084 -1.1389 -
[10645] HIRA ---> TP53 0.12626 -0.081433 -
[10646] ACVR2A <--- INHBE -0.22266 0.60442 +
[10647] ARHGAP18 <--- RHOC -1.5245 1.016 -
[10648] PRKCB ---> SOS2 0.13325 -0.18146 -
[10649] CD80 <--- STK39 -0.10629 0.057301 -
[10650] ARRB1 ---> WNT3 0.23514 -0.34616 +
[10651] RHOBTB2 <--- TRIP10 0.044329 0.11932 +
[10652] CSF3R <--- LEP -0.10858 0.043291 +
[10653] SOD2 ---> STAT3 0.26825 -0.19721 -
[10654] EPO ---> EPOR 0.040195 -0.16507 +
[10655] CCNG2 <--- FOXO4 -0.6022 0.58764 +
[10656] CD19 ---> CD79A 0.27535 -0.27308 -
[10657] EGFR ---> PLCB3 0.029176 -0.032969 -
[10658] SOCS5 <--- STAT5B -0.73574 0.49958 -
[10659] CHRM2 ---> GNAI1 1.1631 -1.1303 +
[10660] CACNA1H ---> GRIN2D 0.37674 -0.2845 -
[10661] DKK1 ---> WNT7B 0.54164 -0.69755 +
[10662] DRD4 ---> GNB2 1.1575 -0.92616 +
[10663] EXOC2 <--- RAB14 -0.73289 1.1482 +
[10664] FASLG <--- LUM -0.38137 0.16123 +
[10665] SMAD3 ---> TGIF2 -0.024788 -0.1488 +
[10666] SRPK1 ---> SRSF1 1.2133 -0.47902 +
[10667] GNB3 ---> RASGRF2 0.73531 -0.73429 +
[10668] ADAMTS3 ---> COL1A2 -0.02131 -0.035774 +
[10669] SFRP4 ---> WNT16 1.0317 -1.1063 +
[10670] ADRA1B ---> GNA13 1.1631 -1.1303 +
[10671] CTCFL <--- ZNF280B -0.025264 -0.024143 -
[10672] CTCF ---> MYC 0.028311 -0.070511 +
[10673] JUNB ---> STAT5A 0.22495 -0.1553 +
[10674] DUSP18 ---> MAP3K4 1.4184 -1.4202 +
[10675] MAPK8 ---> NGF 0.8706 -0.70555 -
[10676] STRAP <--- TAB1 -0.61686 0.85802 -
[10677] ESR2 ---> GNAL 0.042535 -0.12976 +
[10678] KIT <--- PDGFC -0.099588 0.12565 +
[10679] CRIP1 ---> EP300 0.020708 -0.018625 -
[10680] LRP5L <--- NCSTN -0.19386 0.13754 +
[10681] IL10RA <--- SOCS2 -0.40298 0.060066 +
[10682] PAX1 ---> TCF12 0.43996 -0.52432 -
[10683] EIF2AK3 <---> MAPK14 0.31014 0.31014 -
[10684] HIST1H2BL <--- KAT2A -0.12061 0.076698 +
[10685] CACNB1 <--- PRKACG -0.016941 0.18215 +
[10686] CSNK1A1 ---> FZD3 0.5614 -0.51612 -
[10687] PAK3 ---> VAV2 0.070232 -0.022692 +
[10688] ITGB6 ---> LAMA2 -0.0055713 -0.0064655 -
[10689] CD3D <--- LCP2 -0.14641 -0.021938 -
[10690] ENO1 ---> MYC 0.043379 -0.17554 -
[10691] PTCRA <--- SPI1 -0.33485 0.27234 +
[10692] IKZF3 <--- PRDM1 -0.043094 -0.00070136 +
[10693] GAB2 <--- RASA1 -0.1716 0.39661 +
[10694] RPL30 ---> RPS13 0.24487 -0.45553 -
[10695] RALBP1 <--- RHOC -1.5245 1.016 -
[10696] CTNNB1 ---> DVL2 0.22164 -0.31127 -
[10697] AGRN ---> LRP8 0.061778 -0.047155 -
[10698] F2 <--- IGF1 -0.09545 0.031228 -
[10699] PLEKHG5 <--- RAC3 -0.012974 0.047696 -
[10700] DEPDC1B <--- RHOT1 -0.38519 0.18509 -
[10701] RASGRP3 <--- SOS1 0.001776 0.018372 +
[10702] GAB2 <--- PRKD3 -0.1056 0.2913 -
[10703] CER1 ---> WNT1 0.60582 -0.57968 +
[10704] EHF ---> RXRA -0.056454 -0.16004 -
[10705] EGR3 <--- FASLG -0.16564 0.25366 -
[10706] LEF1 <--- NT5E -0.16215 0.24471 -
[10707] IL20RB ---> JAK2 1.1771 -1.1167 +
[10708] CDK4 ---> ETS1 0.24561 -0.15935 -
[10709] COPA <--- GGA3 -0.098063 0.10019 +
[10710] GNA11 ---> WNT5A 0.42087 -0.47341 -
[10711] LIMK1 ---> PAK7 0.52276 -0.38894 -
[10712] FGF18 <--- TEK -0.099363 0.062698 -
[10713] PRKACG <--- RAP1B -0.20304 1.2491 -
[10714] HIF3A <--- UBE2D1 -0.080839 0.14071 -

[10715]	CHN1	<---	COPA	-0.064538	-0.047462	-
[10716]	FZD10	<---	WNT3	-0.78209	0.63734	+
[10717]	GRK5	--->	GRM5	0.80802	-0.62351	+
[10718]	SHC3	<---	TEK	-0.16364	0.077594	+
[10719]	E2F4	<---	SMAD4	-0.30541	0.36653	+
[10720]	PRKCZ	<---	TBXA2R	-0.05892	-0.029433	+
[10721]	ESR1	--->	JUN	0.10351	-0.20904	+
[10722]	EPHB2	<---	FGR	-0.26855	0.35424	-
[10723]	EGF	<---	PTPN6	-0.15582	0.18272	-
[10724]	CBL	--->	CD3D	0.27772	-0.14084	-
[10725]	CSNK1D	--->	GJA1	0.24561	-0.15935	+
[10726]	GH2	--->	IFNGR1	0.012421	-0.36925	+
[10727]	ELK1	<---	MAPK7	-0.26091	0.40675	+
[10728]	KCNJ3	<---	OPRM1	-0.2522	0.11779	-
[10729]	CALML5	--->	HRAS	0.12065	-0.091216	+
[10730]	GHR	<---	PRL	-0.17721	0.073187	+
[10731]	CD4	<---	PTPN11	-0.20056	0.16314	-
[10732]	KRAS	<--->	RALB	0.1628	0.1628	-
[10733]	CD8B	<---	PTPN6	-0.30848	0.37051	-
[10734]	ITGB1	--->	PIK3R5	0.14537	-0.20815	+
[10735]	IL4R	<---	TYK2	-0.37792	0.17946	-
[10736]	MAPK1	<---	MRAS	-0.20304	1.2491	+
[10737]	ESR1	<---	MAPK11	-0.30308	0.29076	+
[10738]	KCNMA1	<---	PRKACG	-0.44517	0.61419	+
[10739]	CD19	--->	DAND5	0.22675	-0.19328	-
[10740]	ECT2	--->	PLK1	0.18525	-0.037481	-
[10741]	CACNB3	<---	CAMK2A	-0.047052	0.14731	+
[10742]	ATF1	--->	MYC	0.017098	-0.10868	-
[10743]	DVL2	<---	PSMD7	-0.62919	0.4836	+
[10744]	ELF3	<---	NFKB1	-0.088224	0.084999	+
[10745]	CAMK2A	<---	MAP3K7	-0.6709	0.58554	-
[10746]	BIRC2	<---	CASP7	-0.16002	-0.0075246	-
[10747]	NBN	<---	UBE2N	-0.21942	0.13028	+
[10748]	NUP153	--->	RBM8A	0.60094	-0.54167	+
[10749]	CAMK2B	--->	MYO6	0.1858	-0.13343	+
[10750]	MAPK3	<---	PPP2R1A	-0.25679	0.26693	+
[10751]	NTRK1	<---	PIK3R3	-0.2539	-0.16045	-
[10752]	KCNJ5	<---	SSTR5	-0.2522	0.11779	-
[10753]	CSNK1A1L	--->	WNT5A	0.83912	-0.78694	-
[10754]	NR0B2	--->	TAF1	-0.02445	-0.096565	-
[10755]	GNA15	<---	HTR2A	-1.1303	1.1631	+
[10756]	FZD2	<---	KREMEN2	-0.26377	0.23032	+
[10757]	CCNK	<---	MEF2A	-0.16823	0.1249	+
[10758]	CXCL2	<---	CXCR4	-0.35895	0.21658	-
[10759]	ITGAV	<---	SYK	-0.13763	0.033232	-
[10760]	BTRC	--->	PSME4	0.36556	-0.20278	-
[10761]	GNA11	<---	PLCD1	-0.023623	0.0010472	-
[10762]	CD4	<---	DBNL	-0.10077	0.074792	-
[10763]	GNAI2	--->	GRM4	0.24033	-0.15623	-
[10764]	FOXO1	<---	MAPK14	-0.22085	0.2499	+
[10765]	EPHA3	<---	YES1	-0.26002	0.33922	+
[10766]	CTNNA1	--->	YWHAB	1.0272	-1.0665	+
[10767]	IL12B	--->	IL6ST	-0.11794	-0.12445	+
[10768]	ARHGAP23	<---	RHOBTB1	-0.28214	0.21939	-
[10769]	CCL15	<---	CCR3	-0.35895	0.21658	-
[10770]	FYN	--->	SPTBN2	0.23238	-0.16875	+
[10771]	REST	--->	SCRT1	-0.024143	-0.025264	+
[10772]	ADCY2	<---	CALML3	-0.63894	0.65698	+
[10773]	BCAR1	--->	JAK2	0.084064	-0.061487	-
[10774]	CRKL	<---	JAK2	-0.20876	0.46073	+
[10775]	ADIPOR2	<---	PRKAA1	-0.26091	0.40675	-
[10776]	SOCS6	--->	STAT5B	0.27422	-0.74698	+
[10777]	PLCL1	<---	RGS4	-0.082548	0.03305	+
[10778]	MAP2K1	<--->	MAPK9	0.31014	0.31014	-
[10779]	PICK1	<---	PRKCG	-0.40252	0.33758	+
[10780]	RHOBTB1	<---	TRIP10	0.044329	0.11932	+
[10781]	IL6	--->	IL7R	0.12248	-0.29036	+
[10782]	STAT1	--->	TAF1	0.2569	-0.31397	-
[10783]	MAP2K4	<---	RAC1	-0.20304	1.2491	+
[10784]	KCNJ9	<---	OPRD1	-0.2522	0.11779	-
[10785]	HOXB7	--->	SP3	0.078944	0.049909	-
[10786]	SRPK2	--->	ZBTB7A	0.66556	-0.40919	-
[10787]	PAK6	<---	RAC1	-1.1901	0.96651	-
[10788]	CTNNA1	--->	MEP1B	0.9277	-0.97185	-
[10789]	JAK3	--->	SOCS1	1.1559	-1.1639	-
[10790]	CFTR	<---	PRKAG1	-0.73719	0.42017	+

[10791]	CD3E	<---	CSK	-0.45391	0.63648	-
[10792]	CREB3L1	--->	JUN	1.1275	-0.11171	+
[10793]	LYN	<---	PRKCG	-0.027179	0.23398	-
[10794]	FYN	--->	ITGA11	0.23404	-0.20204	-
[10795]	SHC1	<---	SMPD1	-0.41798	0.25145	-
[10796]	AKT1	--->	KHSRP	0.16383	-0.26287	+
[10797]	APP	<---	MAPK11	-0.023982	0.030823	-
[10798]	NCK1	--->	WAS	0.22109	-0.26051	+
[10799]	CHRM4	<---	PRKCA	-0.37622	0.27696	-
[10800]	GRB2	<---	RPS6KB1	-0.17009	0.21706	+
[10801]	SLC2A1	<---	SMAD2	-0.34323	0.2018	+
[10802]	GCKR	--->	NUP37	0.37289	-0.35015	-
[10803]	ADAMTS3	<---	COL5A2	-0.054632	-0.00053524	-
[10804]	CUL5	--->	ERBB2IP	-0.014643	-0.090956	+
[10805]	FOSL2	<---	MAPK11	-0.57905	1.0858	+
[10806]	MYC	<---	TCF12	-0.30347	-0.19793	-
[10807]	GNAI2	<---	PRKACB	-0.22953	0.50225	+
[10808]	ITGB6	--->	THBS1	0.17581	-0.068027	-
[10809]	GRB14	<---	TEK	-0.0765	0.06631	+
[10810]	BDKRB1	--->	GNA14	1.1631	-1.1303	+
[10811]	IRF2	--->	SIX5	0.15908	-0.24396	-
[10812]	FCGR2A	<---	MAPK12	0.12024	0.14714	-
[10813]	CTTN	--->	FER	-0.035678	-0.12309	-
[10814]	ARHGEF3	<---	RHOA	-0.012974	0.047696	-
[10815]	DOK1	<---	HCK	-0.63899	0.54748	+
[10816]	DOK1	<---	HRAS	-0.018972	0.12513	+
[10817]	CASP3	--->	CASP9	-0.039589	-0.88853	-
[10818]	CCNB3	<---	FOXO4	-0.62791	0.70491	+
[10819]	CNTFR	--->	THPO	0.016221	-0.099296	-
[10820]	ARHGEF2	<---	RHOBTB2	-0.057957	0.060033	-
[10821]	SREBF1	<---	ZNF805	-0.20142	0.33079	-
[10822]	PIK3R2	<---	RAC3	-0.33729	0.22452	-
[10823]	PARD6B	--->	TAF1	0.0097797	-0.03161	-
[10824]	PPP3CC	<---	YWHAG	-0.31613	0.09356	-
[10825]	FGFR2	<---	PRKCQ	-0.2199	0.24363	-
[10826]	STAT3	<---	VEGFC	-0.32874	0.44945	-
[10827]	CDK2	--->	SMAD4	0.88243	-0.89987	+
[10828]	MPL	<---	THPO	-0.10598	0.036221	+
[10829]	ATF1	<---	PGK1	-0.098546	0.15465	-
[10830]	CCL8	<---	CCR2	-0.35895	0.21658	-
[10831]	ADORA2A	--->	NTRK1	0.19546	-0.50531	+
[10832]	EPS8	--->	NANOG	0.16108	-0.15488	-
[10833]	CTNNB1	<---	MMP24	-0.26058	0.11034	+
[10834]	IGKV4-1	<---	LAT	-0.12445	7.9204e-005	-
[10835]	EP300	--->	HDAC2	0.021603	-0.018377	+
[10836]	CSH1	--->	IL20RB	0.20113	-0.087402	+
[10837]	MYH11	<---	RHOA	-0.01596	0.026206	+
[10838]	ANGPT4	<---	ETS1	-0.062	-0.014272	+
[10839]	HDAC1	<---	HNF4G	-0.3115	0.23955	+
[10840]	GNB1	--->	LRP5	0.34224	-0.24336	-
[10841]	ANAPC2	<---	CDK1	-0.15544	0.29121	+
[10842]	FGF23	--->	FLT4	0.071829	-0.16555	+
[10843]	ITGA6	<---	SPI1	-0.10803	-0.013036	+
[10844]	EXOC8	<---	MYH4	-0.093425	0.10372	+
[10845]	ERCC2	--->	TBP	0.5431	-0.53675	+
[10846]	DUSP16	--->	MAPK12	1.353	-1.036	+
[10847]	HMHA1	<---	RHOD	-0.62084	0.42373	-
[10848]	MYBL2	--->	MYC	0.054207	-0.12958	+
[10849]	GNAI2	--->	PLCB1	0.052816	-0.052991	+
[10850]	DVL2	<---	WNT6	-0.54867	0.38842	+
[10851]	AFAP1L2	<---	HNF4A	-0.10707	0.029088	+
[10852]	ARHGAP15	<---	RHOC	-1.2097	0.92798	-
[10853]	CADM1	<---	RAC1	-0.12239	0.12643	-
[10854]	CFLAR	--->	MEF2A	0.19303	-0.5291	-
[10855]	RASGRP2	--->	RRAS	0.27322	-0.24352	+
[10856]	MYL7	<---	PPP1CB	-0.26348	0.069904	+
[10857]	CCL24	<---	CCR10	-0.35895	0.21658	-
[10858]	FOSL1	<---	PRAME	-0.1955	0.3362	-
[10859]	CCL1	<---	CXCR6	-0.35895	0.21658	-
[10860]	CFL1	<---	SRF	-0.35947	0.27595	+
[10861]	RBL1	<---	SMAD2	-0.16383	0.070647	+
[10862]	PRKCQ	--->	ZAP70	0.68957	-0.23748	-
[10863]	CTNNB1	<---	WNT16	-0.187	0.023012	+
[10864]	DLG4	<---	EP300	-0.0012996	0.0026009	+
[10865]	EPOR	<---	PRL	-0.17721	0.073187	+
[10866]	CDC42	--->	MET	0.12179	0.057422	-

10869 [10867] E2F7 ---> TFDP2 -0.020679 -0.077916 -
10870 [10868] IL12B <--- IL21R -0.17721 0.073187 -
10871 [10869] CEBPB ---> CXCL2 0.055907 -0.14964 +
10872 [10870] PORCN ---> WNT9A 0.38949 -0.56891 +
10873 [10871] ATF4 <--- RPS6KA5 -0.98092 1.2458 +
10874 [10872] ACTR2 <--- MMP19 -0.29263 0.085904 +
10875 [10873] ARPC1A <--- EP300 -0.0077682 0.007799 +
10876 [10874] ATF1 <--- ERBB2 -0.14838 0.18614 -
10877 [10875] GLI2 ---> WNT5B 0.86373 -1.47 +
10878 [10876] F3 <--- HIF1A 0.055867 0.11265 +
10879 [10877] ARHGAP20 <--- RHOV -1.4209 0.96682 -
10880 [10878] CCR4 ---> CX3CL1 0.21658 -0.35895 -
10881 [10879] CSNK1A1 ---> YWHAQ 0.079981 -0.071802 +
10882 [10880] MAP3K6 <--- RAC1 -0.24954 0.91133 +
10883 [10881] AKT1 ---> RASAL2 0.077205 0.029269 -
10884 [10882] SAFB2 <--- YY1 0.008509 0.25992 +
10885 [10883] ATF3 <--- PRAME -0.1955 0.3362 -
10886 [10884] CREBBP ---> ENO1 -0.00068954 -0.0041449 +
10887 [10885] ATP1A1 ---> PIK3R5 0.14537 -0.20815 -
10888 [10886] CD3D ---> VAV1 0.074697 0.032911 +
10889 [10887] ACOX1 ---> USF1 0.16008 -0.19465 -
10890 [10888] CSNK1D ---> FZD1 0.5614 -0.51612 -
10891 [10889] AURKC ---> H3F3A 0.922 -0.92817 +
10892 [10890] CAMK2G ---> RASGRF2 0.24914 -0.57965 +
10893 [10891] CDC20 <--- PPP3CC -0.05461 -0.03116 +
10894 [10892] HRAS ---> PAK2 0.58512 -0.72293 +
10895 [10893] CDK4 ---> TFDP2 0.34787 -0.22767 +
10896 [10894] LRP6 <--- PSEN1 -0.43554 0.57998 +
10897 [10895] CXCR6 <--- JAK2 -0.12268 -0.039248 -
10898 [10896] BCAR1 ---> TNK2 -0.0066719 -0.010212 -
10899 [10897] RHOBTB1 ---> TAGAP 0.61511 -0.92575 -
10900 [10898] ERBB2 <--- HBEGF -0.82917 1.3656 +
10901 [10899] HRAS <--- IGF1 -0.0077402 0.03884 +
10902 [10900] BRAF <--- PRKCA -0.23412 0.18571 +
10903 [10901] GNAO1 ---> GPSM2 -0.0080287 -0.042052 -
10904 [10902] CCL14 <--- CCR8 -0.35895 0.21658 -
10905 [10903] COL6A6 ---> ITGA2 -0.0037591 -0.061373 +
10906 [10904] PRKCG ---> PTPN11 0.55493 -0.48581 -
10907 [10905] CTNND1 ---> FGF6 -0.026025 -0.04249 +
10908 [10906] NCK2 ---> PAK3 0.73462 -0.72841 +
10909 [10907] IL2RG <--- IL6 -0.11334 0.056231 +
10910 [10908] LYN ---> STAT5B 0.43807 -0.74539 +
10911 [10909] ATP1A4 ---> PIK3R1 0.016367 -0.047791 -
10912 [10910] RAB11A <---> RALA 0.1628 0.1628 -
10913 [10911] MYLK2 <--- PAK1 -1.2674 1.5523 +
10914 [10912] GNAO1 ---> PLCB1 0.052816 -0.052991 +
10915 [10913] IKBKB <--- TLR3 -0.19289 0.066908 +
10916 [10914] CSNK1D <---> GSK3A 0.31014 0.31014 -
10917 [10915] ANGPT1 <--- DOK2 -0.011847 0.13965 -
10918 [10916] CDH1 <--- PIK3CA -0.056703 0.0081601 -
10919 [10917] CCL18 <--- CCR6 -0.35895 0.21658 -
10920 [10918] PRKCG ---> SHC3 0.468 -0.43947 -
10921 [10919] ATF4 <--- POU2F2 0.041297 0.055709 +
10922 [10920] DRD1 <--- GRK5 -0.28786 0.33635 +
10923 [10921] NCOA3 <--- RARG -0.054726 0.035483 +
10924 [10922] PARD3 <--- PRSS1 -9.2943e-005 0.019526 +
10925 [10923] FGF2 ---> FLT4 0.071829 -0.16555 +
10926 [10924] GHR <--- IL10 -0.17721 0.073187 +
10927 [10925] JAK2 <--- PTPN6 -1.1104 0.85941 -
10928 [10926] HRAS ---> NRG2 0.14041 -0.14079 -
10929 [10927] CACNA1C <--- CAMK2G -0.26963 0.34735 +
10930 [10928] LAT ---> TAF1 -0.0092662 -0.099047 -
10931 [10929] CBL ---> PIK3CD 0.035594 -0.021832 -
10932 [10930] CRK ---> EPHA2 -0.040532 -0.065642 -
10933 [10931] CSNK1G1 ---> WNT3A 0.50785 -0.4784 +
10934 [10932] CCKBR ---> GNA11 1.1631 -1.1303 +
10935 [10933] PRKCI ---> SOS2 0.088396 -0.13444 -
10936 [10934] EPHB1 <--- FGR -0.26855 0.35424 -
10937 [10935] GATA1 <--- IL1RN -0.056865 -3.6283e-005 -
10938 [10936] RHOV ---> TIAM1 0.20928 -0.13672 -
10939 [10937] GPC5 ---> HS3ST3B1 -0.029045 -0.085212 -
10940 [10938] MYL7 ---> PPP1R12C 0.27396 -0.40226 -
10941 [10939] ITSN1 ---> RHOQ 0.16745 -0.11028 +
10942 [10940] PIK3CB <--- TRAF5 -0.073566 0.090661 +
10943 [10941] APEX1 ---> ATF1 0.15465 -0.098546 -
10944 [10942] FZD10 <--- WNT5B -0.78209 0.63734 +

[10943]	CAMK1G	--->	NOS3	0.088587	-0.060388	+
[10944]	CCL21	<---	CCR1	-0.35895	0.21658	-
[10945]	IFNGR1	<---	PTPN1	-1.1189	0.70682	+
[10946]	AKT1	--->	IKBKG	0.24308	-0.33358	+
[10947]	GNB1	<---	MTNR1A	-0.57296	0.69217	+
[10948]	ACTB	<---	MMP14	-0.29263	0.085904	+
[10949]	SMAD3	<---	ZFYVE16	-0.063591	-0.031512	+
[10950]	CBLC	--->	PIK3CA	0.062274	-0.031117	+
[10951]	PRKCB	--->	TRPV4	0.31032	-0.3562	+
[10952]	BCL2L1	<---	EP300	-0.042369	0.027529	+
[10953]	PDGFRA	--->	RASA1	-0.012733	-0.067986	+
[10954]	CCND3	--->	STAT5B	0.17961	-0.084126	-
[10955]	CD3D	--->	PIK3R3	0.15984	-0.32529	+
[10956]	DEPDC1B	<---	RHOH	-0.93788	0.64026	-
[10957]	ANAPC2	<---	UBE2C	-0.099447	0.015655	+
[10958]	MYO3B	--->	TAF1	0.24561	-0.15935	-
[10959]	IL20RB	--->	JAK1	1.1771	-1.1167	+
[10960]	GNB2	--->	WNT10B	0.3343	-0.42584	-
[10961]	NRAS	--->	PIK3R2	0.13508	-0.20568	-
[10962]	ANKLE2	<---	PPP2CA	-0.32988	0.42727	-
[10963]	HBEGF	<---	MMP2	-0.20414	0.010381	+
[10964]	EBF1	--->	SHH	0.20439	-0.20967	+
[10965]	CCL21	<---	NFKB2	-0.88578	0.76708	+
[10966]	CREBBP	--->	LDHA	-0.00068954	-0.0041449	+
[10967]	CEACAM8	--->	ITGA5	-0.017489	-0.054337	+
[10968]	PPP1CB	--->	WWTR1	0.95833	-1.0588	+
[10969]	GRM1	<---	KCNJ6	-0.25173	0.20895	+
[10970]	GRK4	--->	XCR1	0.33635	-0.28786	+
[10971]	BATF	<---	SP140	-0.16066	0.25678	-
[10972]	PIK3R1	<---	RRAS	-0.36411	0.30089	+
[10973]	GRB2	--->	MYO10	0.025027	-0.14986	-
[10974]	PLEKHG2	--->	RHOT1	0.064274	-0.048106	+
[10975]	ITGA7	--->	TNC	-0.015417	-0.048468	-
[10976]	DAND5	<---	VEGFA	-0.024293	2.0089e-005	-
[10977]	IL2RG	<---	IRS1	0.0072067	0.061248	-
[10978]	CTNNB1	<---	PCDHB18	-0.19204	0.11503	-
[10979]	GRIN2B	<---	RAC1	-0.38938	0.54247	-
[10980]	ADCY6	<---	GNAI1	-0.63317	1.0384	-
[10981]	E2F1	<---	ZBTB25	-0.027971	-0.0048155	-
[10982]	CSNK1D	<--->	CSNK2A1	0.31014	0.31014	-
[10983]	GRIK4	<---	RHOQ	-0.38938	0.54247	-
[10984]	FGFR4	<---	FIGF	-0.38721	0.2003	+
[10985]	ADORA1	--->	EGR1	0.44069	-0.36137	-
[10986]	HNRNPF	--->	MYC	-0.02655	-0.13606	-
[10987]	GNAI3	--->	PLA2G4D	-0.10668	-0.13723	+
[10988]	ARAF	--->	MAPK13	0.99795	-1.3266	+
[10989]	CD14	--->	UBE2D3	-0.043898	-0.16783	-
[10990]	MAP4K4	--->	TNF	0.62058	-0.22921	-
[10991]	CSNK1G2	--->	WNT8A	0.50785	-0.4784	-
[10992]	GFRA1	<---	NCK1	-0.20293	0.16299	-
[10993]	FOXA2	--->	INHA	0.45318	-0.55022	+
[10994]	FOXA2	--->	GATA1	0.19808	-0.23065	+
[10995]	ADRBK2	--->	DRD2	0.25327	-0.21301	+
[10996]	CDKN1A	--->	JUNB	0.15531	-0.10887	-
[10997]	CCNT1	--->	NCBP2	0.14535	-0.055137	+
[10998]	SPI1	--->	ZEB1	0.29124	-0.21528	+
[10999]	GNAQ	--->	WNT2B	0.42087	-0.47341	-
[11000]	EPOR	<---	IL3	-0.067781	-0.018386	+
[11001]	CDK6	--->	SMAD6	0.88243	-0.89987	-
[11002]	PIAS1	--->	STAT5B	1.2392	-0.72187	+
[11003]	FADD	<---	IL1R1	-0.3744	0.56723	+
[11004]	GRIA1	<---	PPP1CB	-0.67094	0.67498	+
[11005]	HMHA1	<---	RHOA	-0.62084	0.42373	-
[11006]	FGF6	--->	FGFR4	0.40218	-0.67149	+
[11007]	ARR3	<---	RARG	-0.091347	-0.010433	+
[11008]	LRP6	<---	WNT7A	-0.28769	0.39899	+
[11009]	CSNK2A1	--->	DVL3	0.52194	-0.62577	+
[11010]	HLA-DQA2	<---	PDCD1LG2	-0.032853	-0.027446	+
[11011]	PLCG2	<---	PRKCG	-0.0064763	0.024762	-
[11012]	RELA	--->	TYK2	-0.11509	-0.25929	-
[11013]	RHOA	--->	SYDE1	1.016	-1.5245	-
[11014]	EP300	--->	SALL4	0.01223	-0.0076085	+
[11015]	PPFIA2	--->	RAB3A	0.39732	-0.4667	-
[11016]	GRIN2B	<---	PRKACA	-1.006	1.5014	+
[11017]	PAK2	--->	VAV3	0.070232	-0.022692	+
[11018]	CCND1	<---	WDR77	-0.33759	0.44625	+

[11019]	LRP8	<---	NCK2	-0.15755	0.18435	-
[11020]	NCK2	--->	PAK6	0.73462	-0.72841	+
[11021]	GNA11	<---	OXTR	-1.1303	1.1631	+
[11022]	RHOQ	--->	TRIO	0.11717	0.0097792	-
[11023]	BIRC7	--->	CASP9	0.11242	-0.29703	+
[11024]	IQGAP2	--->	RAC1	0.12856	-0.076889	-
[11025]	CTBP1	--->	CTNNB1	0.17457	-0.28462	+
[11026]	PML	--->	STAT2	0.45606	-0.35168	-
[11027]	ARHGAP5	<---	SRC	0.061074	0.37496	+
[11028]	GNAO1	<--->	GNAT3	0.064764	0.064764	-
[11029]	EP300	<---	MACF1	-0.0056318	0.00076966	-
[11030]	MAPK11	--->	MEF2B	0.35865	-0.36564	+
[11031]	ARAP1	<---	COPA	-0.0098161	-0.0079611	-
[11032]	CDC42	--->	GRIN2A	0.54247	-0.38938	-
[11033]	CHUK	--->	IRS1	0.33127	-0.0041474	+
[11034]	GAB1	<---	PRKCH	-0.50087	0.66104	-
[11035]	MAPK10	<---	RIPK2	-0.29078	0.20201	+
[11036]	IGKV4-1	<---	SH3KBP1	-0.27558	0.17068	-
[11037]	FOXO4	--->	PLK1	1.0275	-0.62691	+
[11038]	CREBBP	--->	CTBP1	-0.0015958	-0.0067551	-
[11039]	PLCD4	<---	RGS13	-0.06854	0.054727	+
[11040]	IL2RG	<---	SOCS3	-0.06215	-0.055475	-
[11041]	DUSP21	--->	MAPK9	1.4184	-1.4202	+
[11042]	OBSCN	<---	RAC2	-0.11324	0.22832	-
[11043]	HIST2H3A	<---	PRDM9	-0.081099	0.16711	+
[11044]	DLG4	--->	SHC1	0.064124	0.048254	+
[11045]	IL3RA	<---	JAK2	-0.22119	0.24536	-
[11046]	ACTC1	<---	MMP20	-0.29263	0.085904	+
[11047]	IFNG	--->	TERT	0.014048	-0.13898	+
[11048]	ITGA2B	--->	THBS1	0.16838	-0.067658	-
[11049]	HES1	<---	NEUROG3	-0.59347	-0.24839	-
[11050]	LYN	--->	S1PR1	0.55948	-0.3948	-
[11051]	PARD3	<---	PRSS2	-9.2943e-005	0.019526	+
[11052]	GNAI3	<---	PXN	-0.057204	0.12598	-
[11053]	CHRM5	<---	PRKCH	-0.37622	0.27696	-
[11054]	ABL1	--->	RELN	0.23279	-0.27566	-
[11055]	ERBB2	--->	HGF	0.14906	-0.13132	-
[11056]	CAMK2B	--->	CREB3L3	0.72002	-0.41113	+
[11057]	CCL3L1	<---	CCR4	-0.35895	0.21658	-
[11058]	PPM1A	--->	SMAD9	0.32113	-0.52898	+
[11059]	CACNA1C	<---	PPP1CC	-0.51246	0.42555	+
[11060]	PDK1	--->	PPARD	0.16681	-0.28815	-
[11061]	ARNT	<---	HKDC1	-0.099193	0.096943	-
[11062]	IL22RA2	<---	SOCS7	-0.40298	0.060066	+
[11063]	PDGFRA	<---	TGFA	-0.23554	0.23128	+
[11064]	LEF1	<---	SMAD7	-1.2424	1.1041	+
[11065]	CAMK2G	--->	PIK3C3	0.12615	-0.096379	+
[11066]	RELA	--->	TWF2	0.18561	-0.46471	+
[11067]	LEF1	--->	PAX5	0.086269	-0.031892	-
[11068]	RASA1	--->	SOCS3	0.02657	0.020625	+
[11069]	MED16	--->	TBP	0.21494	-0.20566	-
[11070]	ANGPT1	<---	MAPK8	-0.31844	0.42791	-
[11071]	ERN1	<---	TRAF2	-0.60861	1.3226	-
[11072]	BMP6	--->	BMPR1B	0.27387	-0.33406	+
[11073]	ITGA11	--->	THBS4	0.046345	-0.039839	-
[11074]	IKZF3	--->	TCF12	0.19618	-0.059615	-
[11075]	CBLC	--->	IL20RB	1.4386	-0.93248	+
[11076]	ITGA5	<---	LAMC1	-0.041507	-0.0065623	+
[11077]	CCNA2	<---	CDKN1B	-1.0232	1.4682	+
[11078]	LRP5	<---	MMP15	-0.26418	0.30003	+
[11079]	ARHGAP26	<---	RND3	-0.65972	0.48549	-
[11080]	CSNK1A1	--->	LRP5L	0.58717	-0.41189	-
[11081]	DAND5	<---	UBE2L3	-0.12533	0.10192	-
[11082]	ATP1B3	<---	PRKX	-0.26091	0.40675	+
[11083]	PRKCG	--->	RXRG	0.18741	-0.28891	+
[11084]	AFAP1L2	<---	EBF1	-0.27578	0.38965	+
[11085]	MTOR	<---	PIN1	-0.16748	0.22025	+
[11086]	DKK1	--->	WNT8B	0.54164	-0.69755	+
[11087]	NOD2	<---	RIPK2	-0.1353	-0.12328	-
[11088]	AAAS	<---	DHX38	-0.071625	0.072414	-
[11089]	DRD1	<---	GRK6	-0.28786	0.33635	+
[11090]	GNGT2	<---	KCNJ3	-0.059562	-0.05732	-
[11091]	ADRBK1	--->	HTR2B	0.25327	-0.21301	+
[11092]	DOK2	<---	HRAS	-0.018972	0.12513	-
[11093]	CMKLR1	--->	ZEB1	0.38869	-0.34224	-
[11094]	EPHA2	--->	FGF18	-0.0069714	-0.050497	-

[11095]	ABCC2	<---	NR1H4	-0.18861	0.060153	+
[11096]	CDH5	<---	RHOA	-0.24486	0.12008	+
[11097]	IGF1R	--->	PLCG2	0.027447	-0.017905	+
[11098]	ADCY9	<---	CAMK2A	-0.44346	0.58749	+
[11099]	PLD1	<---	SPHK2	-0.057765	0.061811	-
[11100]	GNA11	--->	PLCB2	0.0066317	-0.024514	+
[11101]	GNAI1	<---	SSTR1	-1.1303	1.1631	+
[11102]	PIK3R2	--->	PLCG1	0.017792	-0.0047058	+
[11103]	WIF1	--->	WNT9A	0.49247	-0.8346	+
[11104]	FMN1	--->	JUP	0.34206	-0.44924	-
[11105]	KREMEN2	--->	WNT3A	0.3638	-0.57716	+
[11106]	ATP1B3	<---	MAPK1	-0.26091	0.40675	+
[11107]	CEBPB	<---	RRAGD	-0.16676	0.11805	-
[11108]	DKK2	--->	WNT5A	0.54164	-0.69755	+
[11109]	LAMA3	--->	MMP12	-0.0038564	-0.069983	-
[11110]	PIK3R3	--->	RAC2	0.30072	-0.072693	+
[11111]	ADCY1	<---	RAPGEF4	-0.28599	0.53533	-
[11112]	CCL3	<---	ETS1	-0.091858	0.03281	+
[11113]	CTNNB1	<---	PTPN1	-1.1138	0.95926	+
[11114]	ACTG1	<---	PRKCA	-0.43362	0.33453	+
[11115]	CACNA1C	<---	GNAO1	-0.17558	0.23994	+
[11116]	FGFR3	--->	PIK3R3	0.33968	-0.38283	+
[11117]	ACTR2	<---	WASL	-0.22029	0.18415	+
[11118]	ARPC1B	--->	WAS	0.012602	-0.037247	-
[11119]	ATF6B	<---	PRKACG	-0.57905	1.0858	+
[11120]	EP300	<---	PSTPIP1	-0.0061452	0.0014873	-
[11121]	FOXA2	--->	HNF1A	0.53035	-0.57954	+
[11122]	ADRBK2	--->	CCR8	0.25327	-0.21301	+
[11123]	ITGAV	--->	SHB	-0.033449	-0.11693	-
[11124]	CTBP2	--->	PAX7	0.024957	-0.10255	+
[11125]	MET	--->	PIK3R3	0.34417	-0.64184	+
[11126]	ACTB	<---	WASF2	-0.35324	0.28498	+
[11127]	ARHGAP26	<---	RHOH	-0.65972	0.48549	-
[11128]	IL12RB1	<---	JAK2	-0.38855	0.16968	-
[11129]	EP300	--->	HK3	-0.00068954	-0.0041449	+
[11130]	AKT1	--->	CREB3	0.82132	-1.3257	+
[11131]	JUN	<---	TNFRSF10A	-0.3239	0.14599	-
[11132]	PIK3R2	--->	PREX1	0.031856	-0.1005	+
[11133]	MAPK14	--->	PRKG1	0.5348	-0.19389	-
[11134]	EP300	--->	ZNF805	0.016752	-0.010456	+
[11135]	CASP1	<---	IL1B	-0.36352	0.2336	-
[11136]	KPNA5	<---	RAE1	-0.11171	0.067727	+
[11137]	JUP	<---	MMP28	-0.26058	0.11034	+
[11138]	GNB1	<---	PRKACA	-0.28574	0.38931	+
[11139]	CACNB3	<---	PPP2R2A	-0.06614	0.10562	+
[11140]	NR3C1	--->	ZNF624	0.18046	-0.21501	+
[11141]	BDNF	<---	MAPK8	-0.70555	0.8706	-
[11142]	ADRBK2	--->	CXCR4	0.25327	-0.21301	+
[11143]	ARRB1	<---	CXCR1	-0.15406	0.070342	-
[11144]	LPAR3	--->	PTK2	-0.05393	-0.18014	+
[11145]	GNB2	<---	KCNJ3	-0.098063	0.10019	-
[11146]	JAK1	--->	LEPR	0.10182	-0.23677	-
[11147]	CDK6	--->	CDKN2D	0.68643	-0.45084	-
[11148]	RAPGEF3	--->	RASSF5	0.2255	-0.11125	+
[11149]	IL13RA1	<---	IL3	-0.07563	0.0029493	+
[11150]	HSPA1A	--->	STAT3	0.59817	-0.46802	-
[11151]	CCR5	--->	PF4V1	0.21658	-0.35895	-
[11152]	GNAQ	--->	GNB1	0.069944	-0.012773	+
[11153]	FZD3	<---	SFRP4	-1.3633	0.73901	+
[11154]	PRKCE	--->	SHC1	0.468	-0.43947	-
[11155]	GRAP	<---	JAK2	-0.20876	0.46073	+
[11156]	ARHGAP12	<---	RHOF	-0.36596	0.22724	-
[11157]	SYK	--->	VAV1	0.14791	-0.11071	+
[11158]	IL20	--->	IL2RB	-0.0077042	-0.055821	+
[11159]	ATF1	<---	PRKACG	-0.35231	0.65748	+
[11160]	GFI1	<---	NFKB1	-0.050435	0.12651	+
[11161]	CCR9	--->	CXCL10	0.21658	-0.35895	-
[11162]	ADCY2	<---	PRKCE	-0.49495	0.42642	+
[11163]	MAP3K2	<---	RAP1A	-0.17545	1.1267	+
[11164]	ARHGAP11A	<---	RHOC	-1.5245	1.016	-
[11165]	GNGT2	<---	MAPK3	-0.41411	0.50061	-
[11166]	FZD3	<---	GNB4	-0.19257	0.19185	-
[11167]	MAP2K4	<---	TAB3	-0.13728	0.23085	+
[11168]	EBF1	--->	HOXC6	0.26198	-0.27059	+
[11169]	ACTB	<---	MMP16	-0.29263	0.085904	+
[11170]	FCGR2A	<---	PRKCB	-0.32503	0.3549	-

```
[11171] GRIK3   <---    NCALD   -0.52822        0.61549 +
[11172] ARAP2   <---    RHOBTB2 -0.29597        0.2217  -
[11173] E2F1    --->    SPEN    0.32902 -0.26332        +
[11174] CCL8    <---    CXCR5   -0.35895        0.21658 -
[11175] ARRB1   --->    AXIN1   0.0088597       -0.049113       +
[11176] AKT1    --->    RPTOR   0.033101        -0.096316       +
[11177] FOXO3   --->    MST1    0.19308 -0.24623        -
[11178] ADCY5   <---    PRKCE   -0.49495        0.42642 +
[11179] CDC42   <---    IQGAP3  -0.076889       0.12856 -
[11180] BCAR1   <---    YES1    0.023313        1.0891  +
[11181] MTOR    <---    PPP3CC  -0.15223        0.20492 -
[11182] AKT1    --->    MEF2D   0.40901 -0.63556        +
[11183] CCL25   <---    CX3CR1  -0.35895        0.21658 -
[11184] CTNNB1  <---    MMP12   -0.26058        0.11034 +
[11185] EGFR    --->    UBE2D1  0.20569 -0.1777 -
[11186] PIK3CB  --->    PLD1    0.0053638       -0.0095341      +
[11187] ARRB2   --->    FZD8    0.13556 -0.17053        +
[11188] ADRBK2  --->    CCR3    0.25327 -0.21301        +
[11189] ITGA11  --->    TNXB    -0.025002       -0.077063       -
[11190] ABL1    --->    MAP2K6  0.40343 -0.66195        -
[11191] INSRR   <---    PDGFD   -0.033666       0.27735 +
[11192] SREBF1  <---    ZNF584  -0.27513        0.36066 -
[11193] ACVR2B  --->    SMAD9   0.6082  -0.49302        +
[11194] GAB1    <---    PRKD1   -0.1056 0.2913  -
[11195] DOK1    <---    GRB2    -0.22736        0.09185 +
[11196] CASP9   <---    PIK3CA  -0.068588       0.037418        +
[11197] G6PC2   <---    PRKAG2  -0.20283        0.012951        +
[11198] CD3D    --->    TRBV12-3        -0.033535       -0.068284       -
[11199] LAMC3   --->    SV2B    0.16735 -0.19531        +
[11200] CDK8    --->    SMAD3   0.88243 -0.89987        +
[11201] RAF1    --->    SFN     -0.029863       -0.22693        +
[11202] ESR2    --->    STAT5B  0.27087 -0.22437        -
[11203] ADRA1A  <---    PRKAA2  -0.10024        0.19743 -
[11204] ICAM1   <---    RELA    -1.4607 1.4042  +
[11205] FGF1    --->    FGFR4   0.40218 -0.67149        +
[11206] E2F4    --->    STAT5A  0.43514 -0.31194        +
[11207] EXOC2   <---    RAB11A  -0.73289        1.1482  +
[11208] CTNND1  <---    RHOA    -0.32991        0.29511 -
[11209] ITSN1   --->    RHOB    0.16745 -0.11028        +
[11210] CXCL3   <---    CXCR4   -0.35895        0.21658 -
[11211] CCNT1   --->    USF2    0.21622 -0.26507        -
[11212] NOTCH1  <---    PCSK7   -0.19909        0.2729  +
[11213] FOXO1   <---    MAPK13  -0.22085        0.2499  +
[11214] GHRL    --->    HTR1F   0.61472 -0.75338        +
[11215] SOS1    <---    TEC     -0.30914        0.088396        +
[11216] GNAI3   <---    HTR1D   -1.1303 1.1631  +
[11217] GNA13   --->    PLA2G4F -0.10668        -0.13723        +
[11218] ITGB4   <---    LAMB3   -0.026399       0.0074885       +
[11219] ARNTL   <---    GSK3A   -0.29216        0.3669  +
[11220] CHRM5   <---    PRKCB   -0.37622        0.27696 -
[11221] JAK1    --->    PDPK1   0.2569  -0.48656        +
[11222] COMP    <---    ITGB1   -0.023154       0.01856 -
[11223] IL13RA2 <---    PIK3CA  -0.025559       0.0087857       -
[11224] EPO     --->    IFNGR1  -0.020571       -0.35712        +
[11225] PIK3R5  <---    PLD1    -0.16007        0.13645 -
[11226] PRKCG   --->    TRPV5   0.31032 -0.3562 +
[11227] IL12B   --->    IL12RB2 -0.11794        -0.12445        +
[11228] LMO4    --->    PRKCD   0.16874 -0.15599        +
[11229] CALCOCO1        <---    ESR1    -0.29011        0.12054 +
[11230] F2      --->    SERPINA5        0.18568 -0.33063        -
[11231] RAN     --->    RANGAP1 0.071474        -0.10113        -
[11232] FGFR4   <---    VEGFB   -0.38721        0.2003  +
[11233] FGFR4   --->    PIK3R3  -0.019415       -0.36798        +
[11234] EP300   --->    OLFM1   0.00067605      -0.0050208      +
[11235] IRAK3   <---    TLR4    -0.13421        0.0023645       -
[11236] CSNK1G2 --->    YWHAG   0.047412        -0.04465        +
[11237] EGF     <---    HCK     -0.13921        0.19166 +
[11238] ANKRD1  <---    NR3C1   -0.2617 0.2121  +
[11239] PLK1    --->    PPP1R12B        0.23067 -0.0077656      -
[11240] BLK     <---    CBL     -0.17509        0.10935 -
[11241] ACTN2   <---    CAMK2D  -0.14999        0.15958 -
[11242] IL10    --->    PRLR    0.073187        -0.17721        +
[11243] GNAI2   <---    KCNJ3   -0.011498       0.05262 -
[11244] E2F1    --->    SRSF10  0.32902 -0.26332        +
[11245] EP300   --->    HIF3A   0.0036358       -0.0049315      +
[11246] ID2     <---    SMAD4   -0.34215        0.18982 +
```

```
[11247] CCL3L1  <---    NFKB1   -1.2272 1.0745  +
[11248] MAPK10  --->    PLA2G4A 0.18871 -0.84715        +
[11249] CDC42   <---    CTNNA1  -0.52573        0.60916 -
[11250] DLC1    <---    RAC3    -0.50393        0.10338 -
[11251] FOXO1   --->    IL7R    0.45535 -0.90965        +
[11252] ITGB7   <---    LAMB2   -0.017681       0.011222        +
[11253] ADRBK1  --->    GRM2    0.53935 -0.4166 -
[11254] IFNGR1  <---    SOCS4   -0.4866 0.21014 +
[11255] MYL12A  --->    MYLK    0.33394 -0.31058        -
[11256] GRAP    <---    PRKCG   -0.20419        0.22188 -
[11257] PTEN    <---    ROCK2   -0.012085       -0.0099612      +
[11258] RAC3    --->    TIAM1   0.20928 -0.13672        -
[11259] FOS     <--->   FOSB    0.36709 0.36709 -
[11260] ARNT    <---    TEK     -0.066074       0.040011        -
[11261] CSF2RB  <---    GH1     -0.10334        0.076998        +
[11262] PIK3R2  --->    TPTE    0.10854 -0.1655 -
[11263] ETS1    --->    MMP9    -0.0045452      -0.11357        +
[11264] ACTC1   <---    MMP27   -0.29263        0.085904        +
[11265] FIGF    <---    GRB2    -0.14783        0.1122  -
[11266] CSF1R   <---    FGF13   -0.39456        0.47941 +
[11267] CD70    --->    TCF12   0.4136  -0.53791        -
[11268] GNAI3   <---    MAPK1   -0.22953        0.50225 -
[11269] EGFR    --->    SHC3    1.2029  -1.0554 +
[11270] IL3RA   <---    SOCS4   -0.19297        0.16027 +
[11271] HDAC9   <---    NUP153  -0.53842        0.60929 +
[11272] CSRP1   <---    RELA    -0.16136        0.018875        +
[11273] ARHGAP19        <---    RHOBTB2 -0.92575        0.61511 -
[11274] ARHGAP12        <---    RHOJ    -0.36596        0.22724 -
[11275] SFRP5   --->    WNT6    1.0317  -1.1063 +
[11276] MAP2K3  --->    MAPKAPK5        1.1648  -0.18663        +
[11277] MAP2K1  <---    PAK4    -1.2674 1.5523  +
[11278] FOXO4   --->    S1PR4   0.55395 -0.58151        +
[11279] TCF12   <---    TP63    -0.246  0.18113 -
[11280] GNA13   <---    LPAR1   -1.1303 1.1631  +
[11281] CSNK1A1L        <---    PPP2CB  -0.15489        0.41077 -
[11282] BCAR1   --->    EPHA2   0.062403        -0.088148       -
[11283] PLA2G6  <---    PRKCD   -0.22176        0.21934 +
[11284] CSNK2A2 --->    L1CAM   0.1354  -0.12611        +
[11285] ADCY4   <---    PRKX    -0.63235        0.8673  -
[11286] ATP1A2  <---    MAPK1   -0.09775        0.14787 +
[11287] ERBB3   --->    MAX     0.20918 -0.19783        -
[11288] MAPK7   --->    MEF2A   1.0903  -1.4506 +
[11289] ARHGAP17        <---    RAC1    -0.92575        0.61511 -
[11290] AGT     <---    JUND    -0.084657       0.0032819       +
[11291] BIRC5   <---    TCF7L2  -0.21066        0.34564 +
[11292] CDC25C  <---    PRKX    -0.97911        0.26855 +
[11293] SFRP5   --->    WNT8A   1.0317  -1.1063 +
[11294] DLG3    --->    GRIK3   0.19945 -0.095793       -
[11295] RPS6KA2 --->    TSC2    0.54    -0.69281        +
[11296] CREBBP  --->    HDAC3   0.021603        -0.018377       -
[11297] BCL6    --->    STAT3   0.25348 -0.15351        -
[11298] SLTM    <---    STAT1   -0.35071        0.31265 +
[11299] FGF21   --->    PDGFRA  0.68139 -0.83032        +
[11300] NRG1    --->    STAT5B  0.11008 -0.076118       +
[11301] ADAM17  <---    PRKCH   -0.12963        0.082665        +
[11302] CSF2RB  <---    EPO     -0.098871       0.064861        +
[11303] FGF14   --->    FGFR3   0.071829        -0.16555        +
[11304] BHLHE40 <---    NPAS2   -0.22744        -0.07309        +
[11305] EBF1    --->    GATA3   0.060024        -0.069511       +
[11306] PBX3    <---    SUV420H2        -0.3757 0.33229 -
[11307] CBLB    --->    EPOR    0.18092 -0.10684        +
[11308] ARHGAP20        <---    RHOH    -1.4209 0.96682 -
[11309] G6PC    <---    PRKAA2  -0.4456 0.57716 +
[11310] ABCC8   <---    KCNJ11  -0.18748        0.089268        -
[11311] CCNB2   <---    PAFAH1B1        -0.20586        0.26956 -
[11312] ARHGAP20        <---    RHOT1   -0.65643        0.37566 -
[11313] ALDOA   <---    CREBBP  -0.0050208      0.00067605      +
[11314] ACVR2A  --->    SMAD5   0.6082  -0.49302        +
[11315] AKT1    --->    NCOR2   0.104   -0.18405        +
[11316] EPHB1   <---    MAP4K4  -0.12553        0.20213 -
[11317] NFKB1   --->    TNFSF10 0.30264 -0.39904        +
[11318] FGF5    <---    TEK     -0.099363       0.062698        -
[11319] MYH14   <---    ROCK1   0.024697        0.042724        +
[11320] ITGA1   <---    RHOA    -0.10112        0.0095864       -
[11321] CEP41   <---    RAB8A   -0.031778       0.046557        -
[11322] ADRA1A  --->    GNA12   1.1631  -1.1303 +
```

```
[11323] IL7R    <---    PTPN6   -0.43834        0.46997 +
[11324] IL1R1   --->    MAP4K4  0.54302 -0.20664        +
[11325] ARR3    --->    WNT2    0.23514 -0.34616        -
[11326] IFNAR1  <---    IFNG    -0.39151        0.21627 +
[11327] EPOR    <---    GH2     -0.17721        0.073187        +
[11328] ARHGAP6 <---    RHOQ    -1.5245 1.016   -
[11329] GAB2    <---    PRKCE   -0.50087        0.66104 -
[11330] ATM     --->    H2AFX   0.012042        -0.026705       +
[11331] CEBPB   --->    ZKSCAN2 0.28002 -0.15205        +
[11332] LMO4    <---    PTPN11  -0.37676        0.30399 -
[11333] CREB1   <---    MAPK1   -0.35231        0.65748 +
[11334] CTNNB1  <---    MMP1    -0.26058        0.11034 +
[11335] ETS2    --->    THBD    0.014117        -0.033054       +
[11336] MAPK13  --->    MAPT    0.40675 -0.26091        +
[11337] LCK     <---    PDGFRB  -0.15501        0.75705 +
[11338] CREB5   <---    RPS6KA4 -0.98092        1.2458  +
[11339] HOMER1  <---    PIK3CD  -0.01789        0.011376        -
[11340] FGFR4   --->    SHC4    0.029603        -0.2849 +
[11341] ITGA2   --->    PIK3R5  -0.067056       -0.12453        +
[11342] GNB4    <---    KCNJ6   -0.098063       0.10019 -
[11343] CHD2    --->    CHD6    0.0078972       0.006357        +
[11344] GMIP    <---    RHOH    -1.011  0.68428 -
[11345] FOSL2   <---    NR3C1   -0.22023        0.16637 +
[11346] FOS     --->    THBS1   0.47303 -0.15552        +
[11347] DLG1    --->    GRIK5   0.19945 -0.095793       +
[11348] PIK3CB  <---    PLCG1   -0.0055153      0.0039515       -
[11349] PPFIA3  --->    RAB3A   0.39732 -0.4667 -
[11350] ERBB2   --->    VEGFA   0.12082 -0.10806        -
[11351] ACTC1   <---    ADAM17  -0.34681        0.25337 +
[11352] CBLC    --->    LCK     0.66741 -0.55096        +
[11353] COL5A1  --->    ITGA4   -0.013036       -0.10803        +
[11354] CCNB2   <---    CENPK   -0.15303        0.19277 -
[11355] CDC25A  <---    PSMD4   -0.32375        -0.0048732      +
[11356] E2F1    <---    STRADA  -0.30098        0.45782 -
[11357] CREB3   <---    INS     -0.24765        0.36121 -
[11358] CREB3L3 --->    JUN     1.1275  -0.11171        +
[11359] DAG1    <---    RAC1    -0.038582       0.076038        -
[11360] PRKCQ   --->    TCRB    0.25526 -0.34755        -
[11361] ITGA1   <---    LAMB3   -0.062785       -0.0050465      +
[11362] AKT1    --->    GNB1    0.12487 -0.17772        +
[11363] HSP90AA1        --->    PTK2B   0.019262        -0.15031        -
[11364] COL1A1  --->    JUN     0.11985 -0.17465        -
[11365] CSF2RA  <---    PTPN6   -0.29595        0.42284 +
[11366] PIK3CA  <---    TRAF5   -0.073566       0.090661        +
[11367] MAPK10  <--->   MAPK12  0.31014 0.31014 -
[11368] RELA    <---    TLR5    -0.54488        0.15126 +
[11369] ILK     <---    RXRB    -0.070039       -0.056828       +
[11370] GADD45A <---    NFKB1   -1.2754 0.97443 +
[11371] ARHGEF18        <---    RHOU    -0.012974       0.047696        -
[11372] MAPK8   --->    PGR     0.29076 -0.30308        -
[11373] FGF3    <---    SOS1    0.067334        0.085583        -
[11374] NUP43   --->    SRSF11  0.31391 -0.17132        +
[11375] CTNND1  <---    VASP    -0.19176        0.27658 -
[11376] CXCR4   <---    JAK2    -0.12268        -0.039248       -
[11377] EP300   <---    NFAT5   -0.005516       0.0083594       -
[11378] ITGA7   <---    LAMA2   -0.025761       -0.0054603      +
[11379] ITGA11  <---    LAMC2   -0.066621       -0.0090065      +
[11380] CAV1    <---    DAND5   -0.072158       0.036704        +
[11381] CBLB    --->    SHC1    0.14145 -0.20863        +
[11382] GNAO1   <---    HTR1A   -1.1303 1.1631  +
[11383] POTEKP  <---    RHOQ    -0.51168        0.52339 +
[11384] ARAP1   <---    RHOB    -0.48615        0.36733 -
[11385] PDPK1   --->    PIK3R2  0.20765 -0.17885        -
[11386] CACNB2  <---    OXTR    -0.050969       0.05244 +
[11387] MAP3K4  <--->   MAPK14  0.31014 0.31014 -
[11388] CAMK2A  <---    HRAS    -0.038527       0.42208 -
[11389] IL6R    <---    TYK2    -0.32054        0.21304 -
[11390] PRKCI   --->    STAT3   0.53838 -0.49624        -
[11391] ATF2    --->    HIST1H2BD       0.048146        0.0081543       +
[11392] CHUK    --->    IRS2    0.33127 -0.0041474      +
[11393] BLNK    <---    IGKV4-1 -0.31594        0.13444 +
[11394] GAB2    <---    RAP1A   -0.50681        0.59188 -
[11395] CD3E    --->    SHC1    0.13055 -0.21965        +
[11396] MAPK13  <--->   MAPKAPK3        0.31014 0.31014 -
[11397] MMP12   --->    POTEKP  0.085904        -0.29263        +
[11398] FOXO1   --->    RBL2    0.21631 -0.28948        +
```

[11399]	SREBF1	<---	ZNF775	-0.023698	0.070404	-
[11400]	GLI1	<---	GNAZ	-0.038754	0.25143	+
[11401]	BAMBI	--->	FZD1	0.43771	-0.51806	+
[11402]	CALM1	--->	CAMK2D	0.19292	-0.081704	-
[11403]	EIF4E2	<---	GABPA	-0.31385	0.26411	+
[11404]	IL11RA	<---	IL19	-0.14339	0.084246	+
[11405]	PPARG	<---	RARB	-0.021251	-0.0075988	-
[11406]	GNA15	<---	TACR1	-1.1303	1.1631	+
[11407]	CTNNB1	<---	ILK	-0.092311	0.19873	+
[11408]	DVL1	<---	LRP6	-0.14258	0.10923	+
[11409]	ACVR2B	<---	LEFTY1	-0.85941	1.3161	+
[11410]	DKK1	--->	FZD1	0.3176	-0.35737	+
[11411]	CCND1	--->	STAT4	0.17961	-0.084126	-
[11412]	CALML5	--->	MYLK4	0.41687	-0.23601	+
[11413]	COL11A1	--->	ITGA4	-0.013036	-0.10803	+
[11414]	FAM13B	<---	RHOBTB1	-0.92575	0.61511	-
[11415]	DEF6	<---	RHOA	-0.65413	0.6983	-
[11416]	KIDINS220	<---	RAP1A	-0.30133	0.065636	-
[11417]	PCK2	--->	RXRA	-0.16619	-0.17934	-
[11418]	RANBP2	--->	SRSF3	0.34767	-0.31854	+
[11419]	AKT1	<---	PPP2R3A	-0.7767	0.80883	+
[11420]	PDHX	<---	PDK4	-0.5004	0.41454	+
[11421]	FXYD1	<---	PRKACB	-0.26091	0.40675	+
[11422]	FN1	<---	ITGA2B	-0.10408	-0.047194	-
[11423]	CSF2RB	<---	SOCS5	-0.16792	0.11632	+
[11424]	CHUK	--->	PEBP1	0.052703	-0.022222	-
[11425]	ORAI1	<---	PRKX	-0.46035	0.48012	+
[11426]	PLCL1	<---	RGS17	-0.082548	0.03305	+
[11427]	E2F1	<---	MYBL2	-0.092111	0.080065	-
[11428]	SOCS4	<---	STAT2	-0.73574	0.49958	+
[11429]	CD3G	--->	IGKV4-1	-0.033535	-0.068284	-
[11430]	NGEF	<---	RHOD	0.091479	0.13323	-
[11431]	MMP9	<---	PLG	-0.1166	-0.013996	+
[11432]	CBX6	<---	MAPK11	-0.22919	0.35645	+
[11433]	PIK3CB	<---	PLCG2	-0.0055153	0.0039515	-
[11434]	KPNA3	<---	RAE1	-0.19114	0.12101	+
[11435]	IL1B	<---	IRAK4	-0.26346	0.12212	-
[11436]	GADD45G	--->	TP53	0.25242	-0.28587	-
[11437]	EPO	--->	OSMR	0.040195	-0.16507	+
[11438]	SETDB1	--->	ZNF117	0.12238	-0.066729	+
[11439]	CHD2	--->	ETV4	0.050188	-0.12153	+
[11440]	BPTF	--->	CTCF	0.083205	-0.0091154	-
[11441]	CD22	--->	MEF2C	0.03694	-0.050847	-
[11442]	ACVR2A	--->	SMAD2	0.6082	-0.49302	+
[11443]	BCL2	<---	GLI2	-0.32581	0.28403	+
[11444]	CFB	<---	CFD	-0.01668	-0.0099212	+
[11445]	AKT3	<---	HSP90AB1	-0.59063	0.6369	+
[11446]	PDGFD	--->	PDGFRB	0.27548	-0.23671	+
[11447]	TNIK	--->	TRADD	0.24439	-0.21179	-
[11448]	APP	--->	JUND	0.01063	-0.020718	-
[11449]	BMP8A	<---	CHRD	-1.4437	0.57812	+
[11450]	BARHL2	<---	CTBP2	-0.16727	0.04296	+
[11451]	IL5RA	<---	THPO	-0.11385	0.080818	+
[11452]	CSH1	--->	IL13RA2	0.094523	-0.18506	+
[11453]	PRKCI	<---	RAP1A	-1.529	1.3756	-
[11454]	HOXD1	--->	MAFK	-0.019848	-0.073912	-
[11455]	PPP2CB	--->	YWHAE	0.18205	-0.13344	-
[11456]	AKT1	--->	S1PR1	0.086823	-0.20377	+
[11457]	IL4R	<---	IRS1	0.018455	0.089853	-
[11458]	SMYD3	--->	TCF7L2	0.14513	0.036901	-
[11459]	CAPN1	<---	PTPN1	-0.23199	0.19504	-
[11460]	FOS	<---	NFIC	-0.16257	0.22662	-
[11461]	RAN	<---	SFN	-0.25073	0.15053	-
[11462]	MAP2K2	<---	MAP3K3	-1.347	1.3429	-
[11463]	MYL12B	<---	SORBS3	-0.15772	0.17183	-
[11464]	MAP3K2	<---	PAK2	-0.99683	1.5058	+
[11465]	ADRBK2	--->	HTR2B	0.25327	-0.21301	+
[11466]	ATP1A2	<---	PRKG2	-0.22258	0.19984	+
[11467]	CCL24	<---	CCR6	-0.35895	0.21658	-
[11468]	PIAS1	--->	STAT2	1.2392	-0.72187	+
[11469]	AKT1	--->	SHC3	0.32159	-0.42272	-
[11470]	CCR2	--->	GNAI1	1.1631	-1.1303	+
[11471]	MMP8	--->	POTEKP	0.085904	-0.29263	+
[11472]	KIT	<---	LYN	-1.1084	1.4836	-
[11473]	ARHGEF16	<---	RHOU	-0.067065	0.085273	-
[11474]	CREB3L4	<---	PRKCG	-0.63701	0.72046	+

[11475]	AXIN2	--->	PSMD10	0.08645	-0.095154	-
[11476]	CALML3	--->	PPP3CC	0.12401	-0.25417	+
[11477]	FZD4	<---	GNA15	-0.22143	0.24435	-
[11478]	SERPINB1	<---	SPI1	-0.53505	0.33364	+
[11479]	PIK3R1	<---	VEGFC	-0.13402	0.058137	+
[11480]	EPHA2	--->	FGF6	-0.0069714	-0.050497	-
[11481]	ARRB2	<---	LHCGR	-0.15406	0.070342	+
[11482]	CXCL1	<---	CXCR6	-0.35895	0.21658	-
[11483]	C1QA	--->	C1S	0.048831	-0.073083	+
[11484]	EBI3	--->	STAT2	0.38886	-0.28644	+
[11485]	LPAR1	--->	PIK3CB	0.016661	-0.039169	+
[11486]	DVL3	<---	FZD7	-0.33521	0.24353	+
[11487]	NRAS	<---	SHC2	-0.081651	0.1917	+
[11488]	AAAS	--->	ALYREF	0.31391	-0.17132	+
[11489]	ITGAV	<---	MAPK1	-0.18823	0.15237	-
[11490]	GATA2	--->	ITGA2	-0.008916	-0.16586	+
[11491]	STAT3	<---	YES1	-0.74539	0.43807	+
[11492]	ECT2	<---	RHOBTB2	-0.0089688	0.02783	-
[11493]	HDAC8	<---	TCF7L2	-0.12264	0.37175	-
[11494]	CRK	--->	PIK3CG	0.019554	-0.030141	+
[11495]	PTCRA	<---	RBPJ	-0.47399	0.60322	+
[11496]	HNF1B	<---	RARA	-0.27075	0.10711	+
[11497]	COL6A1	<---	TAF1	-0.040539	-0.022167	+
[11498]	AFAP1L2	<---	CTCF	0.021629	0.085685	+
[11499]	NFKBIA	<---	PRKCZ	-0.1199	0.05307	+
[11500]	EP300	<---	RBCK1	-0.0075848	-0.0031669	-
[11501]	DVL3	<---	PSMD7	-0.62919	0.4836	+
[11502]	MUSK	--->	NTRK1	0.025286	-0.16391	+
[11503]	SREBF2	<---	ZNF595	-0.20142	0.33079	-
[11504]	NKD1	<---	TCF7L2	-1.0901	1.4076	+
[11505]	CDKN1B	<---	SKP2	-0.12595	-0.019483	-
[11506]	EP300	--->	SIRT1	0.00067605	-0.0050208	+
[11507]	RASA1	--->	SPHK1	0.33114	-0.24852	-
[11508]	CDC42	<---	EPHB6	-0.29565	0.29551	+
[11509]	BAX	<---	BCL2L11	-0.48613	0.28154	+
[11510]	MAP2K2	--->	YWHAB	0.079981	-0.071802	-
[11511]	MAPK3	--->	PTPN6	1.0948	-1.0232	-
[11512]	BCL2	<---	CASP9	-0.28111	0.03758	-
[11513]	OCRL	<---	RHOF	-0.92575	0.61511	-
[11514]	CD3E	--->	IL12B	0.12248	-0.29036	+
[11515]	ELK1	<---	SRF	-0.35947	0.27595	+
[11516]	HBEGF	<---	KRAS	-0.36578	0.39888	-
[11517]	ERN2	--->	PSEN2	0.43723	-0.29495	-
[11518]	PIK3CA	<---	PRKCQ	-0.045695	0.032777	-
[11519]	AMHR2	--->	SMAD5	0.6082	-0.49302	+
[11520]	PRKACA	--->	PTGDR	0.19743	-0.10024	-
[11521]	MYLK2	<--->	PRKACB	0.31014	0.31014	-
[11522]	COL4A2	--->	PPIB	-0.024575	-0.068802	-
[11523]	AKT1	--->	RARA	0.7641	-0.82222	-
[11524]	GATA2	--->	JUP	0.34206	-0.44924	+
[11525]	CNTFR	--->	SOCS3	0.17881	-0.34174	-
[11526]	ARHGEF18	<---	RAC1	-0.012974	0.047696	-
[11527]	LAMA3	--->	MMP19	-0.0038564	-0.069983	-
[11528]	ERBB4	--->	STAT5B	0.45125	-0.30603	+
[11529]	GNAT1	<---	OPN1LW	-1.1303	1.1631	+
[11530]	ARHGEF7	<---	RHOG	-0.044321	0.055318	-
[11531]	CCND2	<---	HDAC1	-0.16943	0.31701	-
[11532]	ATF6B	<---	DDIT3	-0.098546	0.15465	-
[11533]	MCF2	--->	RHOT2	0.037885	-0.030277	+
[11534]	GABBR2	<---	GNB3	-0.047054	0.048663	-
[11535]	DRD1	--->	KCNJ3	0.11779	-0.2522	-
[11536]	ADCY6	<---	GNAT3	-0.63317	1.0384	-
[11537]	NANOG	<---	NR6A1	-0.27075	0.10711	+
[11538]	CDH1	<---	PVRL2	-0.12405	-0.0068302	+
[11539]	DRD2	<---	NPY	-1.3205	0.89835	+
[11540]	ADCY3	<---	CAMK2D	-0.44346	0.58749	+
[11541]	EPHB2	<---	SYNJ1	-0.03656	-0.029491	-
[11542]	MAPK9	<---	TRAF3	-0.47605	1.1617	+
[11543]	BCL2	<---	CREB1	-0.63807	0.39226	+
[11544]	NFKB1	<---	NOD2	-0.28183	0.25836	-
[11545]	ITCH	--->	VPS4B	0.21151	-0.35307	-
[11546]	COL3A1	--->	ITGA2B	0.0052169	-0.090529	+
[11547]	PPP2R2A	--->	PRKCI	0.38699	-0.50418	+
[11548]	CAMK2G	<---	KRAS	-0.061708	0.69771	-
[11549]	LRP1	<---	PLAU	-0.12023	0.14257	-
[11550]	IRS1	--->	PIK3R3	-0.053716	-0.21576	+

[11551]	CSF2RB	<---	PIK3R1	-0.021298	0.013892	-
[11552]	LCK	--->	NFKBIA	0.665	-0.50348	+
[11553]	CACNA1D	<---	PRKACA	-0.38391	0.51195	+
[11554]	IFNGR2	<---	STAT1	-0.18674	0.16294	-
[11555]	IL3RA	--->	SOS1	0.095178	0.048636	+
[11556]	CD3G	--->	TCRB	-0.033535	-0.068284	-
[11557]	PRLR	--->	SOCS1	-0.098904	-0.12483	-
[11558]	CTCF	<---	TFAM	-0.0090394	0.11956	-
[11559]	AREG	<---	RELA	-0.79191	0.50845	+
[11560]	DRD2	--->	KCNJ6	0.11779	-0.2522	-
[11561]	EP300	--->	SHOX	0.018159	-0.022857	+
[11562]	ITGAV	<---	LAMC3	-0.041507	-0.0065623	+
[11563]	GDNF	<---	RASA1	-0.27176	0.34803	-
[11564]	CD3E	<---	RGS1	-0.4184	0.31307	+
[11565]	AKT2	<---	HSP90AB1	-0.59063	0.6369	+
[11566]	CD44	--->	TIAM1	0.089365	-0.09365	+
[11567]	GRIN2A	<---	PPP1CC	-0.67094	0.67498	+
[11568]	CCL26	<---	CXCR2	-0.35895	0.21658	-
[11569]	CCL16	<---	CCR1	-0.35895	0.21658	-
[11570]	STAT3	<---	ZNF460	-0.19761	0.30058	-
[11571]	CD79B	--->	PIK3R1	0.02313	-0.074896	-
[11572]	ELF3	<---	RELA	-0.17272	0.066517	+
[11573]	ATP2A2	<---	THRA	-0.056504	-0.0074276	+
[11574]	IL1R1	--->	TRAF6	0.18296	0.076641	+
[11575]	AKT2	--->	ATF6B	0.82132	-1.3257	+
[11576]	INS	<---	PPP1CC	-0.39837	0.20753	-
[11577]	CTCF	--->	ZNF594	-0.0037425	-0.012548	+
[11578]	ERBB4	--->	KRAS	0.28807	-0.18972	+
[11579]	FGF18	--->	INSR	0.25346	-0.0044024	+
[11580]	EGFR	--->	VEGFA	0.12082	-0.10806	-
[11581]	MYL9	<---	RHOA	-0.70657	0.67133	+
[11582]	RAP1B	<--->	RHOA	0.1628	0.1628	-
[11583]	POU2F2	<---	SPI1	-0.24396	0.15908	-
[11584]	KCNJ6	<---	OPRM1	-0.2522	0.11779	-
[11585]	IL13RA2	<---	THPO	-0.17292	0.061531	+
[11586]	ATR	<---	BRCA1	-0.17653	0.19032	-
[11587]	PLCB4	<---	RGS4	-0.082548	0.03305	+
[11588]	PRKCA	--->	YWHAE	0.25075	-0.36987	+
[11589]	PIK3R2	<---	SRC	-0.26628	0.26317	+
[11590]	ARHGAP5	<---	RHOBTB2	-0.048842	0.38021	-
[11591]	IFNAR1	--->	STAT2	0.27615	-0.18365	+
[11592]	RAP1B	<---	RAPGEF6	-1.3789	0.84353	+
[11593]	GRIK2	<---	RAC2	-0.38938	0.54247	-
[11594]	GRIN2B	<---	RAC2	-0.38938	0.54247	-
[11595]	KDR	--->	SHB	0.77318	-0.31205	-
[11596]	CACNA2D1	<---	PPP2R1A	-0.24964	0.16125	+
[11597]	JAK3	--->	RASA1	0.32505	-0.84134	-
[11598]	COL5A2	--->	ITGA11	0.0052169	-0.090529	+
[11599]	EGF	<---	SLC9A1	-0.15724	0.15888	-
[11600]	MAP4K4	--->	MAPK8	1.3976	-0.9701	-
[11601]	CTNNA3	--->	YWHAB	1.0272	-1.0665	+
[11602]	ATF6B	--->	BCL2	0.64854	-1.0502	+
[11603]	E2F1	--->	TFDP1	-0.020679	-0.077916	-
[11604]	KRAS	--->	MAPK11	1.2491	-0.20304	+
[11605]	IFNB1	--->	IL4R	0.27509	-0.55283	+
[11606]	GRIA4	<---	PRKACB	-1.006	1.5014	+
[11607]	SFRP1	--->	WNT3	1.0317	-1.1063	+
[11608]	FZD1	<---	SFRP5	-1.3633	0.73901	+
[11609]	CRKL	<---	MAPK8	-0.19779	0.14625	-
[11610]	PPP1R1A	<---	PPP2R1A	-0.23396	0.23393	+
[11611]	CTNND1	--->	IGF1R	0.2012	0.026532	+
[11612]	CDC25C	<---	PRKACG	-0.97911	0.26855	+
[11613]	CREB3L1	<---	MAPK3	-0.57905	1.0858	+
[11614]	DEPDC7	--->	RHOT1	0.26799	-0.26312	+
[11615]	GRK5	--->	HRH2	0.33635	-0.28786	+
[11616]	HNRNPA1	--->	MYC	-0.02655	-0.13606	-
[11617]	EP300	--->	KLF7	0.016752	-0.010456	+
[11618]	IL2RG	<---	PTPN11	-0.16409	0.19246	+
[11619]	CD7	<---	GATA2	-0.068284	-0.033535	+
[11620]	CTCFL	<---	MAPK1	-0.99235	1.4077	-
[11621]	FRZB	--->	FZD8	0.73901	-1.3633	+
[11622]	CACNA1D	<---	GNAI2	-0.17558	0.23994	+
[11623]	E2F4	<---	GABPA	-0.051409	0.003866	+
[11624]	EPO	--->	IFNAR2	0.16814	-0.075265	+
[11625]	CCND1	<---	MAPK3	-0.55661	0.75243	+
[11626]	MAP2K1	--->	YWHAZ	0.079981	-0.071802	-

[11627]	MSC	<---	REST	-0.20142	0.33079	+
[11628]	CCNB2	<---	FOXO4	-0.62791	0.70491	+
[11629]	KALRN	<---	RHOA	-0.021179	0.042607	-
[11630]	ADORA3	<---	GRK4	-0.40072	0.46898	+
[11631]	CDK5	<---	EPHA4	-0.5179	0.41679	+
[11632]	IKBKB	--->	IKBKG	0.18012	-0.073616	-
[11633]	MYH6	--->	MYL2	0.18892	-0.22718	-
[11634]	CSF2RB	<---	IL12B	-0.088901	-0.056448	+
[11635]	NFATC1	<---	PRKCZ	-0.22902	0.10402	+
[11636]	FYN	--->	PLXNA3	0.62668	-0.41605	+
[11637]	FGD3	<---	RHOV	-0.14219	0.16153	-
[11638]	CSF2RA	<---	JAK2	-0.22119	0.24536	-
[11639]	EIF4A2	--->	EIF4H	0.23551	-0.20283	+
[11640]	MYL2	<---	MYL3	-0.45619	-0.35268	+
[11641]	CSF2	--->	CSF2RA	0.1357	-0.10858	+
[11642]	ARHGEF11	--->	RAC3	0.092701	0.0073174	+
[11643]	HDAC2	--->	TBP	0.085704	-0.037498	+
[11644]	MYL12A	--->	PAK1	0.48303	-0.046893	-
[11645]	RALBP1	<---	RHOBTB2	-0.92575	0.61511	-
[11646]	CBLC	--->	IFNAR2	1.4386	-0.93248	+
[11647]	GRIN2B	<---	PRKACB	-1.006	1.5014	+
[11648]	RAB11B	--->	SLC2A4	0.57863	-0.5218	+
[11649]	GNAQ	--->	WNT8A	0.42087	-0.47341	-
[11650]	AKT3	--->	PIK3R3	0.51097	-0.68225	-
[11651]	BCL2L1	<---	SOS2	-0.046213	0.10222	+
[11652]	DDX20	--->	NUP37	0.072414	-0.071625	-
[11653]	MCF2L	<---	RHOU	-0.026438	0.029604	-
[11654]	ASAP1	<---	COPA	-0.019529	0.052813	-
[11655]	CD8A	<---	JAK2	-0.26201	0.089794	+
[11656]	CSNK1D	--->	WNT2	0.83912	-0.78694	-
[11657]	BMPR1B	--->	SMAD9	0.7946	-0.60139	+
[11658]	SGMS1	--->	SOS1	0.096929	0.043876	-
[11659]	C1S	<---	EP300	-0.008704	0.0047587	+
[11660]	GRK4	--->	GRM7	0.80802	-0.62351	+
[11661]	GH2	--->	IFNLR1	0.093836	-0.20115	+
[11662]	AKT1	--->	FGFR2	0.053523	-0.092062	-
[11663]	AAAS	<---	RAN	-0.35969	0.30932	-
[11664]	IRS1	<--->	IRS2	0.22966	0.22966	-
[11665]	ARHGEF7	<---	RHOBTB2	-0.027982	0.032452	-
[11666]	NOTCH2	<---	PSEN2	-0.43417	0.16726	+
[11667]	ALYREF	<---	RAE1	-0.17132	0.31391	+
[11668]	ARRB2	--->	HDAC2	0.22783	-0.18981	+
[11669]	PPEF1	--->	RHO	0.18988	-0.29727	+
[11670]	FCER1G	--->	NTRK1	0.081601	-0.26088	+
[11671]	LIMK1	--->	PLXNA2	0.24614	-0.21791	-
[11672]	FOXO1	--->	PAX6	0.32057	-0.35261	-
[11673]	EPHA1	<---	FGR	-0.26855	0.35424	+
[11674]	FYN	--->	ITGA8	0.23404	-0.20204	-
[11675]	REST	<---	STXBP5L	-0.51899	0.6297	-
[11676]	MAPK1	--->	YWHAZ	0.079981	-0.071802	-
[11677]	COMP	<---	ITGA3	-0.033876	0.01404	-
[11678]	KLF5	<---	RELA	-0.57388	0.50851	+
[11679]	IL6R	<---	PIAS1	-0.13012	0.10513	-
[11680]	HIF3A	<---	NR3C1	-0.055353	0.063299	+
[11681]	KIR3DL1	<---	PTPN6	-0.32886	0.27078	-
[11682]	CASP7	--->	CASP9	-0.039589	-0.88853	-
[11683]	LPAR1	--->	PTK2	-0.05393	-0.18014	+
[11684]	MAPK3	<---	TLR4	-0.30538	0.0039737	+
[11685]	IL6ST	<---	PIK3R2	-0.060398	0.011843	-
[11686]	ERBB2	<---	PRKCB	-0.085576	0.11629	-
[11687]	CSNK1A1L	--->	FZD1	0.5614	-0.51612	-
[11688]	CCNB1	<---	DAND5	-0.44212	0.52799	+
[11689]	MTOR	<---	TSC1	-0.2152	0.31885	+
[11690]	FLT1	--->	IGF1	0.31135	-0.0084341	-
[11691]	PRKCE	<---	PRKG2	-0.055629	-0.028296	+
[11692]	CAMK2D	--->	RASGRF1	0.24914	-0.57965	+
[11693]	FGFR1	<---	SPRY4	-0.37589	0.41083	-
[11694]	GLI2	--->	WNT5A	0.86373	-1.47	+
[11695]	IGKV4-1	<---	PIK3AP1	-0.068284	-0.033535	-
[11696]	ICAM1	<---	ITGB2	-0.12727	0.13825	-
[11697]	GRK6	--->	HTR1A	0.33635	-0.28786	+
[11698]	ACTG2	<---	MYL6	-0.10988	0.043745	+
[11699]	OPHN1	<---	RHOT1	-0.45869	0.34454	-
[11700]	GRIN2A	<---	HRAS	-0.23727	0.32792	-
[11701]	BUB3	--->	CCNB2	0.44625	-0.33759	-
[11702]	LPAR3	--->	PIK3CB	0.016661	-0.039169	+

[11703] KCNJ6 <--- SSTR1 -0.2522 0.11779 -
[11704] DKK2 ---> WNT8B 0.54164 -0.69755 +
[11705] EBF1 ---> EHF 0.035307 -0.04326 +
[11706] CDC42 ---> FGD4 0.14242 -0.12937 -
[11707] PSEN1 <--- PVRL1 -0.090446 0.077085 -
[11708] CCL8 <--- CCR5 -0.35895 0.21658 -
[11709] SCRIB <--- YAP1 -0.11382 -0.094149 -
[11710] NFKBIA <--- TRADD -0.39975 0.17105 +
[11711] BAMBI ---> INHBA 0.39038 -0.6158 +
[11712] C4A ---> MASP2 0.088812 -0.16376 -
[11713] MCF2L <--- RHOG -0.026438 0.029604 -
[11714] EIF2AK3 <--- PPP1CA -0.12622 0.14153 +
[11715] ADRBK1 ---> OPRK1 0.25327 -0.21301 +
[11716] ABL1 <--- HCK -1.0203 0.50083 +
[11717] PTK2B <--- RRAS -0.14524 -0.084837 -
[11718] CCR7 ---> CXCL11 0.21658 -0.35895 -
[11719] DNM1 ---> NOTCH1 0.098085 -0.073939 +
[11720] PPP1CA <--- TGFBR2 -0.22941 0.017499 +
[11721] MYL6 ---> MYLK -0.0010631 -0.0039627 -
[11722] CACNA1S <--- PRKX -0.38391 0.51195 +
[11723] LRP8 <--- VLDLR -0.015488 -0.015396 -
[11724] ADCY2 <--- RAPGEF4 -0.28599 0.53533 -
[11725] BZRAP1 ---> RAB3A 0.039056 -0.057739 -
[11726] RHOH ---> TIAM1 0.20928 -0.13672 -
[11727] CD247 ---> TCRA -0.021439 -0.042516 -
[11728] AXIN2 <--- CSNK1D -0.20403 0.2231 +
[11729] CDK10 ---> ZEB1 0.78805 -0.53115 -
[11730] ARHGAP39 <--- CDC42 -0.82986 0.43536 -
[11731] PBX3 ---> RBM39 0.16801 -0.11243 +
[11732] CDK2 ---> TP73 0.096521 -0.076704 +
[11733] PLN <--- THRB -0.14879 -0.015388 +
[11734] KPNA2 <--- PTPN2 -0.82742 0.80804 -
[11735] JUN <--- LPAR1 -0.3481 0.2582 +
[11736] CALM1 <---> NCALD 0.25957 0.25957 -
[11737] ARR3 ---> WNT7A 0.23514 -0.34616 -
[11738] CAV1 ---> TNF -0.056467 -0.069238 +
[11739] CTSG ---> SERPINB6 0.33364 -0.53505 -
[11740] BUB1 ---> RPS6KA6 0.9519 -1.129 -
[11741] FGFR1 <--- TAL1 -0.35676 0.2534 +
[11742] EPHB6 ---> NCSTN 0.028038 -0.13888 -
[11743] GATA3 ---> PAX5 0.056756 -0.091513 -
[11744] JAK3 ---> LIFR 0.11865 -0.1451 -
[11745] HSP90AA1 ---> SGK1 0.18303 -0.22881 +
[11746] COL16A1 <--- PTK2B -0.11304 -0.10314 +
[11747] ATF2 <--- PRKCA -0.43038 0.49111 +
[11748] GRK7 ---> HTR1D 0.33635 -0.28786 +
[11749] GAB2 ---> STAT3 0.46567 -0.58055 -
[11750] RASGRF2 ---> RHOA 0.44606 -0.52428 +
[11751] CRK ---> ITGAM 0.018691 -0.085251 -
[11752] PSMD10 <--- UBE2C -0.8199 0.93651 -
[11753] COL5A3 ---> ITGA4 -0.013036 -0.10803 +
[11754] FBXW11 ---> GLI2 1.5328 -0.71684 +
[11755] FN1 <--- GATA2 -0.29036 0.12248 +
[11756] EP300 <--- PRKAR2B -0.0175 0.01153 -
[11757] POU2F2 ---> USF2 0.23888 -0.32236 -
[11758] CCNB2 <--- FOXO3 -0.62791 0.70491 +
[11759] CREBBP ---> SERPINE1 0.019422 -0.029524 +
[11760] PF4V1 <--- XCR1 -0.35895 0.21658 -
[11761] C1QB ---> SERPING1 0.20126 -0.32562 -
[11762] IKBKG <--- ZBP1 -0.81315 0.71805 +
[11763] FGF20 <--- TEK -0.099363 0.062698 -
[11764] IFNAR1 <--- IL19 -0.087402 0.20113 +
[11765] CCL4 <--- CXCR5 -0.35895 0.21658 -
[11766] CTNNB1 <--- MET -0.394 0.3198 -
[11767] RAC3 <---> RHOC 0.1628 0.1628 -
[11768] IKBKG <--- IRAK4 -1.1039 1.0517 +
[11769] HIF1A <--- PGF 0.098168 0.12147 -
[11770] ERCC2 ---> FOS 0.74265 -0.56347 -
[11771] CABIN1 <--- PRKCD -0.31979 0.26005 +
[11772] AKT3 <--- PHLPP1 -0.79489 1.3907 +
[11773] APEX1 ---> ATF3 0.25678 -0.16066 -
[11774] COL7A1 <--- PTK2 -0.052005 -0.013421 +
[11775] CBLB ---> TYK2 0.52229 -0.18592 +
[11776] EED ---> H3F3A 0.15811 -0.17998 +
[11777] IKBKB <--- TLR6 -0.25848 0.075737 +
[11778] PIK3CD <--- PRKCQ -0.045695 0.032777 -

[11779]	PAK1	<---	RAC3	-1.1901	0.96651	+
[11780]	IRS1	--->	SOCS2	-0.098579	-0.19619	-
[11781]	ECT2	<---	RHOJ	-0.012974	0.047696	-
[11782]	GNA11	<---	TBXA2R	-1.1303	1.1631	-
[11783]	BRAF	--->	MAP2K4	0.99795	-1.3266	+
[11784]	PREX2	<---	RAC1	-0.049683	0.1134	-
[11785]	MED26	--->	WNT10B	0.10932	-0.31274	-
[11786]	CAV1	<---	TNFRSF1A	-0.33817	0.05465	-
[11787]	HIF1A	<---	PIK3CB	0.01233	0.01701	+
[11788]	CACNA1S	<---	PPP2CA	-0.27679	0.37996	+
[11789]	COL24A1	--->	ITGA9	-0.013036	-0.10803	+
[11790]	ERBB2	--->	PLCG2	0.05141	-0.048506	+
[11791]	DUSP21	--->	MAPK13	1.4184	-1.4202	+
[11792]	CBLC	--->	LEPR	0.30049	-0.14845	+
[11793]	CALML6	--->	NOS3	0.037321	-0.042221	+
[11794]	CDK6	--->	MYC	0.75491	-0.9874	-
[11795]	IKBKG	--->	NTRK1	-0.0026159	-0.25443	-
[11796]	ARF3	<---	HTT	-0.49203	0.32422	-
[11797]	CSNK1G3	--->	YWHAZ	0.047412	-0.04465	+
[11798]	CTNND1	<---	PIK3R1	-0.05788	-0.044517	-
[11799]	CSNK1D	--->	WNT5A	0.83912	-0.78694	-
[11800]	CSF1R	<---	PLCB3	-0.016058	0.044043	-
[11801]	FOXA1	--->	NFIC	0.32648	-0.40863	-
[11802]	HOXB9	--->	TCF12	0.23888	-0.32236	-
[11803]	HNRNPD	<---	PSMD14	-0.47703	0.54632	+
[11804]	PRKCQ	--->	VAV1	0.067765	-0.017473	-
[11805]	MYC	<---	NCL	-0.13606	-0.02655	-
[11806]	PLCB1	<---	VEGFA	-0.056307	0.036703	+
[11807]	CD274	<---	TCRB	-0.0347	-0.027692	-
[11808]	HDAC3	<---	PPARA	-0.3115	0.23955	-
[11809]	INS	--->	MYO5A	0.011358	-0.034384	-
[11810]	CFLAR	<---	TRADD	-0.18102	0.0084439	-
[11811]	RALGDS	<---	RHOA	-1.2366	1.4276	-
[11812]	GRM7	<---	KCNJ3	-0.25173	0.20895	+
[11813]	GNAT3	<---	RGS1	-1.2531	1.2063	+
[11814]	MAPK8	--->	ROR2	0.5369	-1.3322	-
[11815]	ADRB2	--->	EP300	0.0029784	-0.0097452	-
[11816]	CTCF	<---	QKI	-0.0091154	0.083205	-
[11817]	MMP14	<---	RELA	-0.44128	0.13291	+
[11818]	FN1	<---	IRS1	0.032241	0.14995	-
[11819]	E2F4	<---	FOXM1	-0.41853	0.37946	-
[11820]	PLCD3	<---	RGS13	-0.06854	0.054727	+
[11821]	HSPA1A	--->	PPARGC1A	1.0596	-0.96282	-
[11822]	GNAI3	<---	PTGER3	-1.1303	1.1631	+
[11823]	RASA1	--->	SOS1	0.056544	-0.27498	+
[11824]	FZD2	<---	WNT5A	-0.78209	0.63734	+
[11825]	ERBB2	--->	HRAS	0.17363	-0.11617	+
[11826]	E2F2	--->	RBL1	0.13728	-0.24843	-
[11827]	ADCY6	<---	EDNRA	-0.62364	0.57833	+
[11828]	CEBPD	<---	IL1A	-0.12163	0.053753	-
[11829]	ACTG1	<---	RAC3	-0.51168	0.52339	+
[11830]	COL1A2	--->	ITGA10	-0.013036	-0.10803	+
[11831]	NR2F1	--->	TCF12	0.27747	-0.4408	-
[11832]	RHOG	<---	TJP1	0.07236	0.080628	-
[11833]	GNAT3	--->	TAS1R3	0.24033	-0.15623	-
[11834]	IL20RA	<---	SOCS2	-0.40298	0.060066	+
[11835]	COL4A1	--->	ITGA6	-0.013036	-0.10803	+
[11836]	IL22RA1	--->	JAK3	1.1771	-1.1167	+
[11837]	EGFR	--->	ESR1	0.11843	-0.12982	-
[11838]	PRKCI	--->	STAT4	0.53838	-0.49624	-
[11839]	AKT1	--->	GRAP	0.077189	-0.13812	-
[11840]	CTNNA1	--->	MMP21	0.38719	-0.55042	-
[11841]	GNB4	--->	PLCB1	0.0335	-0.042377	+
[11842]	RAC1	<---	SOS1	-0.33772	0.48674	+
[11843]	ARHGAP40	<---	RHOBTB1	-0.92575	0.61511	-
[11844]	FGF5	<---	KRAS	-0.25726	0.38422	-
[11845]	MAPK3	--->	MEF2C	1.0903	-1.4506	+
[11846]	MYL2	<---	TNNC2	-0.41458	0.39244	-
[11847]	PPP2R1B	--->	TGFBR2	0.030073	-0.11911	-
[11848]	IL1A	--->	IL1R2	0.064011	-0.12065	+
[11849]	ZNF274	<---	ZNF502	-0.073586	-0.0085226	-
[11850]	MYBPC1	--->	MYL2	-0.064509	-0.083906	-
[11851]	JAK1	--->	LIFR	0.11865	-0.1451	-
[11852]	CD44	<---	COL6A6	-0.065973	-0.0067681	+
[11853]	CAV1	<---	EGFR	-0.21654	0.24184	+
[11854]	BAD	<---	PPP3CC	-0.17753	0.058082	+
[truncated: 541,366 more chars]
